# Evaluating the effects of population mobility on transmission during the COVID-19 pandemic

**DOI:** 10.1038/s41467-026-78247-9

**Published:** 2026-10-14

**Authors:** James M. Trauer, David S. Shipman, Matthew Ryan, Alec S. Henderson, Emma S. McBryde, Michael T. Meehan, Romain Ragonnet

**Affiliations:** 1 School of Public Health and Preventive Medicine, Monash University, Melbourne, VIC Australia; 2 Commonwealth Scientific and Industrial Research Organisation, Adelaide, SA Australia; 3 The Frazer Institute, University of Queensland, Brisbane, QLD Australia; 4 The Burnet Institute, Melbourne, VIC Australia; 5 Australian Institute of Tropical Health and Medicine, James Cook University, Townsville, QLD Australia

**Keywords:** Computational models, Viral infection

## Abstract

Changes to community mobility during the COVID-19 pandemic, together with unprecedented availability of surveillance and mobility data, provide a unique opportunity to quantify the effect of mobility on respiratory infection transmission. We present a renewal model that incorporates epidemiological mechanisms influencing SARS-CoV-2 transmission along with a non-mechanistic scaling process. We compare models with and without mobility using epidemic data from 122 countries and quantify the influence of mobility through reductions in non-mechanistic scaling. In several countries, this non-mechanistic process closely tracks mobility trends and varies substantially less when mobility is incorporated. This is especially evident in Western Europe and North America, but not in several countries of West Africa and the Arabian Peninsula. Google and Facebook data produce similar findings, and parks is frequently assigned the lowest weight in the multi-location Google analysis. Mobility trends are strongly aligned with short-term transmission dynamics in many high-income settings, although between-country heterogeneity remains.

## Abstract (web summary)

Here the authors analyse mobility and COVID-19 data from 122 countries to examine how changes in movement affected virus transmission. They find that mobility data improved estimates of SARS-CoV-2 spread, particularly in Western Europe and North America.

## Introduction

The COVID-19 pandemic was a generational public health crisis^1,2^, but also provides a unique opportunity to evaluate the population-level effects of non-pharmaceutical interventions implemented before targeted biomedical technologies became available. Following early predictions that major morbidity and mortality were likely in the absence of control measures^3^, many countries instituted public health and social measures (PHSMs) to reduce transmission and mitigate the anticipated health system load^4^. With more behavioural and surveillance data available to understand the COVID-19 pandemic than for any comparable emerging infection^5,6^, we now have an unprecedented opportunity to quantify and compare the effects of mobility on the transmission of SARS-CoV-2 across a wide range of international contexts^7^. Moreover, an enhanced understanding of how mobility shaped transmission during COVID‑19 can help inform responses to future emerging respiratory-transmitted pathogens^8^.

As the pandemic progressed, it became clear that SARS-CoV-2 could be transmitted by aerosol^9,10^, implying that simpler droplet precautions could not be relied upon to fully mitigate transmission. Analyses from several settings have shown that behavioural interventions can influence transmission^11–14^, although many relied on timelines of changes in government mandates and recommendations rather than quantitative estimates of population movement^15^ and were often limited to specific settings or countries. Although government policy has been a key mechanism for implementing PHSMs, such policies may not map directly to changes in population behaviour, even when mandated^16^. More generally, population behaviour change requires public engagement and is ultimately driven by individuals and their communities^17^. For this reason, the timeline of government recommendations sheds light on only one aspect of the process of using behaviour change to mitigate transmission of respiratory infections^18^. By contrast, several time series are publicly available that provide empirical metrics of population mobility over time and across international contexts throughout the pandemic period, derived from subsets of mobile-device users who have opted into location services^19,20^.

In addition to publicly accessible mobility and surveillance data, the extensive epidemiological evidence now available, including the recent completion of comprehensive systematic reviews of key aspects of COVID-19 epidemiology^21,22^, strengthens our understanding of the pandemic. To estimate the influence of mobility time series on transmission, we propose an approach that quantifies the extent to which short‑term temporal variation in transmission must be absorbed by a non-mechanistic process with and without mobility included. We implemented this by estimating the reduction in the variability of a regularised non-mechanistic transmission process when mobility covariates were introduced. We integrated this technique with epidemiological data sources and tools for high-performance numerical computing, and used this platform to estimate country-level effects of changes in mobility on SARS-CoV-2 transmission in 122 countries.

## Results

### Calibration fit

**Fig. 1:**
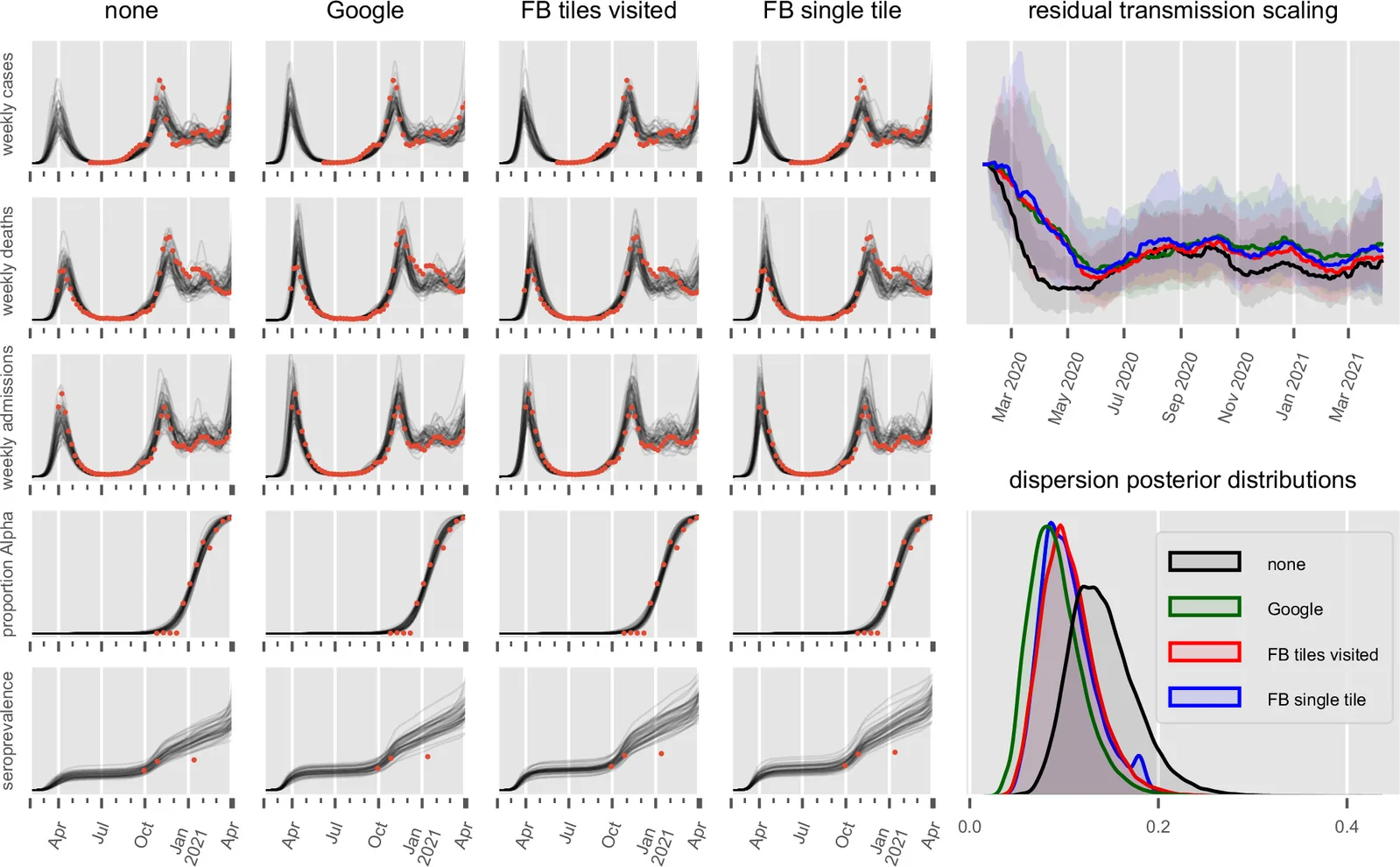
Example fit of renewal model to calibration targets for arbitrarily selected country, France. Left sided 20 panels show the fit of individually accepted model runs to calibration targets (red circles) for each approach to including mobility in the analysis (columns) and for each calibration target (rows). Upper right panel shows the residual transmission scaling under each approach to incorporating mobility with associated 95% credible interval ranges shaded; we interpret greater changes in residual scaling as indicating that more of the changes in transmission are explained when mobility variation is included. The lower right panel shows the posterior distribution of the residual transmission dispersion parameter under each of the four approaches; we interpret lower dispersion values as indicating that the model can accommodate less variation in residual scaling when mobility variation is included. See Supplementary Information for vertical axis scale.

Using a renewal modelling framework incorporating susceptible depletion, variant-specific transmissibility and incomplete immunity to emerging variants, we closely replicated epidemiological targets for each country analysed (Supplementary Fig. 1 shows country inclusion, calibration fits for the base case model are presented through Figshare, Fig. 1 shows results for France as an example country). These targets included time series profiles for daily new deaths, case notifications and up to one hospitalisation indicator, and the proportional prevalence of key variants of concern. Where seroprevalence targets could be sourced, these were included to give a broad guide to epidemic size and the effect of susceptible depletion, and were matched in many cases, including most high-income settings. We conducted analyses with and without adjustment of transmission according to changes in mobility, and focused on epidemiological contexts with low pre-existing natural immunity and for which changes in vaccine-induced immunity were likely to be negligible – either because the analysis period preceded reaching 5% vaccination coverage (most countries) or because coverage had recently reached 90% of the final coverage achieved (Singapore and countries of Oceania).

### Mobility metrics compared to non-mechanistic transmission scaling

**Fig. 2:**
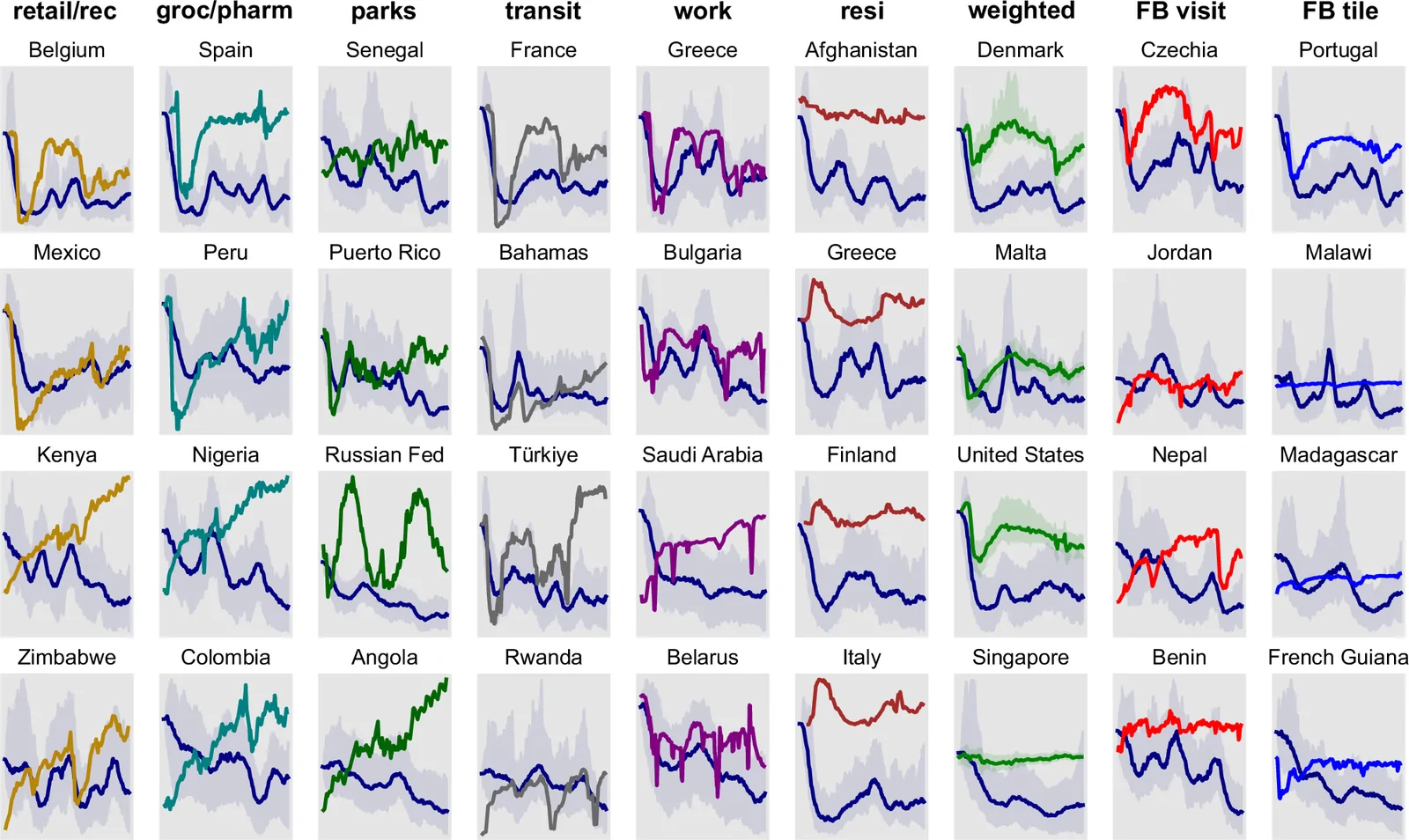
Comparison of mobility metrics against the non-mechanistic transmission scaling estimates (without mobility scaling) for selected illustrative countries. Dark blue line, residual transmission median estimate; shaded blue area, 95% credible interval; other coloured lines, raw mobility data available from Google (first six columns) or Facebook (last two columns), or weighted Google mobility series from calibrated weight parameters (seventh column). retail/rec, retail and recreation; groc/pharm, grocery and pharmacy; work, workplaces; transit, transit stations; resi, residential; FB visit, Facebook all day Bing tiles visited relative change metric; FB single tile, Facebook all day ratio single tile users metric; Russian Fed, Russian Federation. See Supplementary Information for results for all country and mobility combinations with scale on axes.

To explore how mobility patterns aligned with short‑term fluctuations in transmission, we first examined the non‑mechanistic transmission‑scaling process under the model configuration in which mobility was not included. Figure 2 shows a representative set of country-metric combinations comparisons between mobility metrics from Google and Facebook and the non‑mechanistic transmission estimates, with full results provided in the Supplementary Information. Both mobility datasets are relative measures of population mobility collected from mobile device users who have opted into location tracking via specific apps or services^23,24^. In several instances, the temporal alignment between mobility and the non‑mechanistic process was visually striking, with the two series tracking each other closely despite being derived independently. This exploratory analysis was intended to illustrate the diversity of correspondence patterns observed across settings and mobility domains, and to motivate the formal, weighted analyses that follow.

Across many high‑income settings, strong correspondence was observed between our estimated non‑mechanistic transmission scaling function and the empiric estimates of mobility. Examples include the retail and recreation Google location in Belgium, United Kingdom and Mexico, as well as country-metric combinations including Spain-grocery and pharmacy and Czechia-Facebook tiles visited. For these country-metric combinations, changes in mobility appeared to mirror the short-term fluctuations in the inferred scaling required under the no mobility configuration. In other settings, a lesser extent of correspondence appeared to be present, or was only observed for specific sub-periods within our analysis window, e.g. France-transit stations, Peru-grocery and pharmacy and Türkiye-transit stations.

In contrast, weaker, inconsistent, or absent correspondence was commonly seen in some settings and in certain mobility domains. This was often, but not exclusively, seen in lower income settings. Several African countries exhibited strong secular upward trends in specific Google mobility categories – particularly retail and recreation or grocery and pharmacy – without clear correspondence to non‑mechanistic scaling (e.g. Kenya, Nigeria). Other combinations showed partial or period‑specific alignment (e.g. Colombia-grocery and pharmacy early in the pandemic), while some exhibited inverse or unclear patterns depending on domain and setting (e.g. Russian Federation-parks, Saudi Arabia-workplaces).

Additional domain‑specific features were also evident. The residential Google metric is derived differently from the other Google location categories and exhibited less variation for many countries (e.g. Afghanistan), but in some settings showed an inverse relationship to non‑mechanistic scaling (e.g. Zimbabwe). Many European settings showed a characteristic early‑pandemic pattern in which the non-mechanistic scaling term dropped sharply as residential mobility rose (e.g. Finland and Italy), after which residential mobility remained relatively stable even as scaling for transmission continued to fluctuate.

The calibrated composite Google mobility series (described further below) exhibited varying degrees of correspondence: close alignment in some settings (e.g. Denmark), more modest correspondence in others (e.g. Malta), period-specific alignment in some (e.g. United States) and limited variation in some contexts (e.g. Singapore).

Finally, Facebook metrics showed both similarities and contrasts with Google. In some settings, the Facebook tiles visited series exhibited strong correspondence with non‑mechanistic scaling (e.g. Czechia), while other settings showed no consistent relationship (e.g. Jordan and Nepal). Notably, in many African contexts Facebook mobility series displayed little temporal variation (e.g. Benin-Facebook tiles visited) and did not exhibit the strong secular trends observed in some Google categories. The Facebook single‑tile metric generally showed similar patterns to the tiles‑visited series but often with reduced variability.

### Evidence for an effect of mobility

Because there appeared to be correspondence between the non-mechanistic transmission scaling required in analyses without mobility effects and reported mobility metrics in several settings (see Fig. 2), we sought to quantify this formally. All fits to data, with and without mobility included, matched our calibration targets closely, such that we compared these alternative configurations by considering the effect of mobility on the inferred need for non-mechanistic scaling of transmission. That is, if a mobility trend was able to explain variation in transmission, we expected the transmission scaling function to show less variation when the mobility trend was included.

The upper-right panel of Fig. 1 shows how residual transmission scaling changed under each mobility assumption for an example country, suggesting that the changes were less abrupt when mobility was included in this case, with results for all countries presented in the Supplementary Information. We quantified this reduction by considering whether the posterior distribution of the dispersion parameter governing the Wiener process updates was greater under the comparator analysis that did not incorporate the effect of mobility on transmission than it was under the corresponding analysis including mobility. The posterior distributions of this parameter for the example country and for each country-mobility series combination are presented in the lower-right panel of Fig. 1 and Supplementary Information, respectively.

**Fig. 3:**
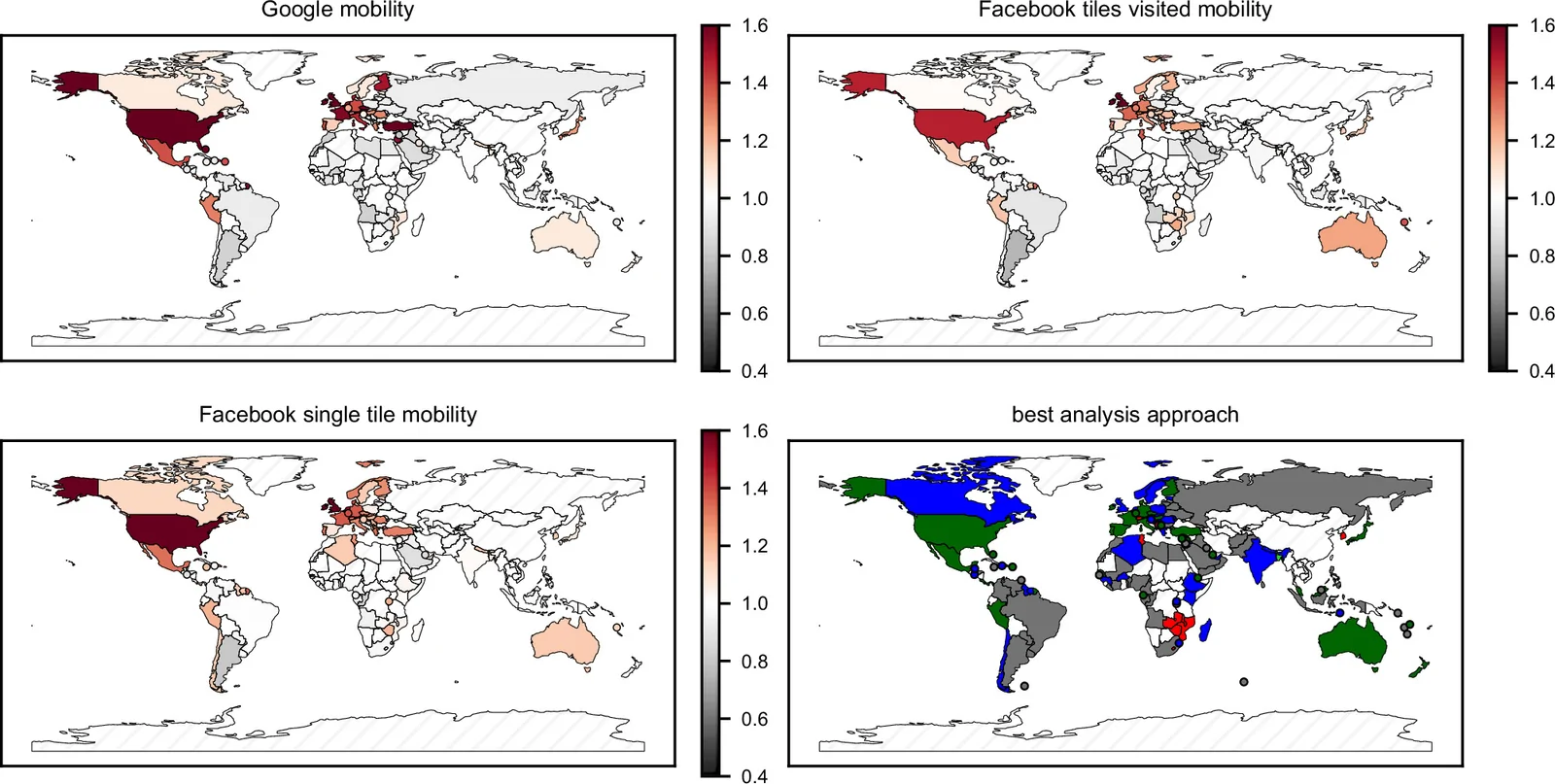
Strength of evidence that mobility influenced transmission as measured by the median of the relative value of the residual transmission dispersion posterior when comparing the no mobility baseline simulation against the corresponding analysis with scaling of transmission for each mobility metric. Upper left, upper right and lower left panels, median of the relative values of the dispersion parameters for each mobility time series. Lower right panel, approach to mobility inclusion resulting in the lowest median dispersion parameter. Lower right panel colours: Green, weighted Google metric; blue, Facebook single tile metric; red, Facebook tiles visited metric; grey, mobility not included; diagonal hatching, country not included in analysis. Country boundary shapefiles were obtained from Natural Earth (https://www.naturalearthdata.com/).

The first three panels of Fig. 3 show the median reduction in the dispersion parameter obtained by comparing the posterior distributions with and without mobility for each country analysed, summarising the full country‑specific distributions presented in the Supplementary Information. Specifically, for each country-metric combination, accepted iterations from the calibration algorithm with and without mobility were randomly paired, and the ratio of the dispersion parameter value without mobility to the value with mobility was calculated for each pair. The median of this distribution of ratios was then interpreted as the strength of evidence that inclusion of mobility helped to reduce the variation needed in the non-mechanistic component of the model, with values greater than one indicating that inclusion of mobility was helpful in explaining transmission variation.

For most countries of Western Europe, Scandinavia and North America, inclusion of weighted Google mobility data or either of the two Facebook metrics resulted in a markedly lower dispersion parameter posterior. This was also the case for some Pacific-coast countries of South America and the Guianas, some countries of south-eastern Africa, Türkiye, Israel, South Korea, Japan, Nepal and Bangladesh. For Eastern Europe, Russia and many of the remaining countries across South America and Africa, the dispersion parameter was similar when scaling for mobility data was included, providing no evidence of improved explanatory value in these settings. For countries in which the Google mobility metrics exhibited strong secular increases over time, detrending these mobility metrics prior to inclusion in our analysis had little effect on the dispersion parameter distributions and so did not substantially alter our findings.

The lower right panel of Fig. 3 shows the approach to analysis that was associated with the lowest dispersion parameter, with the weighted Google analysis being the optimal approach identified for 31 countries (including many countries of Europe, the Middle East, Central America, Oceania and Japan). Inclusion of the Facebook single tile mobility data was the optimal configuration in 34 countries, including India and several countries of Eastern Europe, West Africa and North America. Facebook tiles visited mobility was optimal in only 10 countries, including several neighbouring countries in south-eastern Africa. The no mobility analysis was preferred in 47 countries, including the Russian Federation, Indonesia and some countries of Africa and Eastern Europe.

### Scale of the mobility effect

Next, for the analyses that included mobility, we considered the mobility‑exponent parameter, which governs how strongly the mobility time series must be transformed to reduce the need for non-mechanistic scaling of transmission. We quantified this through the exponent parameter that was applied to the mobility time series. This was assigned a uniform prior distribution on [0,2] to span epidemiologically plausible assumptions that ranged from no effect, through a linear mapping, to a quadratic effect of mobility on the transmission potential of an infectious individual. Intuitively, this exponent describes how strongly the raw mobility series must be compressed or expanded for changes in mobility to align with the short‑term fluctuations in transmission inferred by the model.

**Fig. 4:**
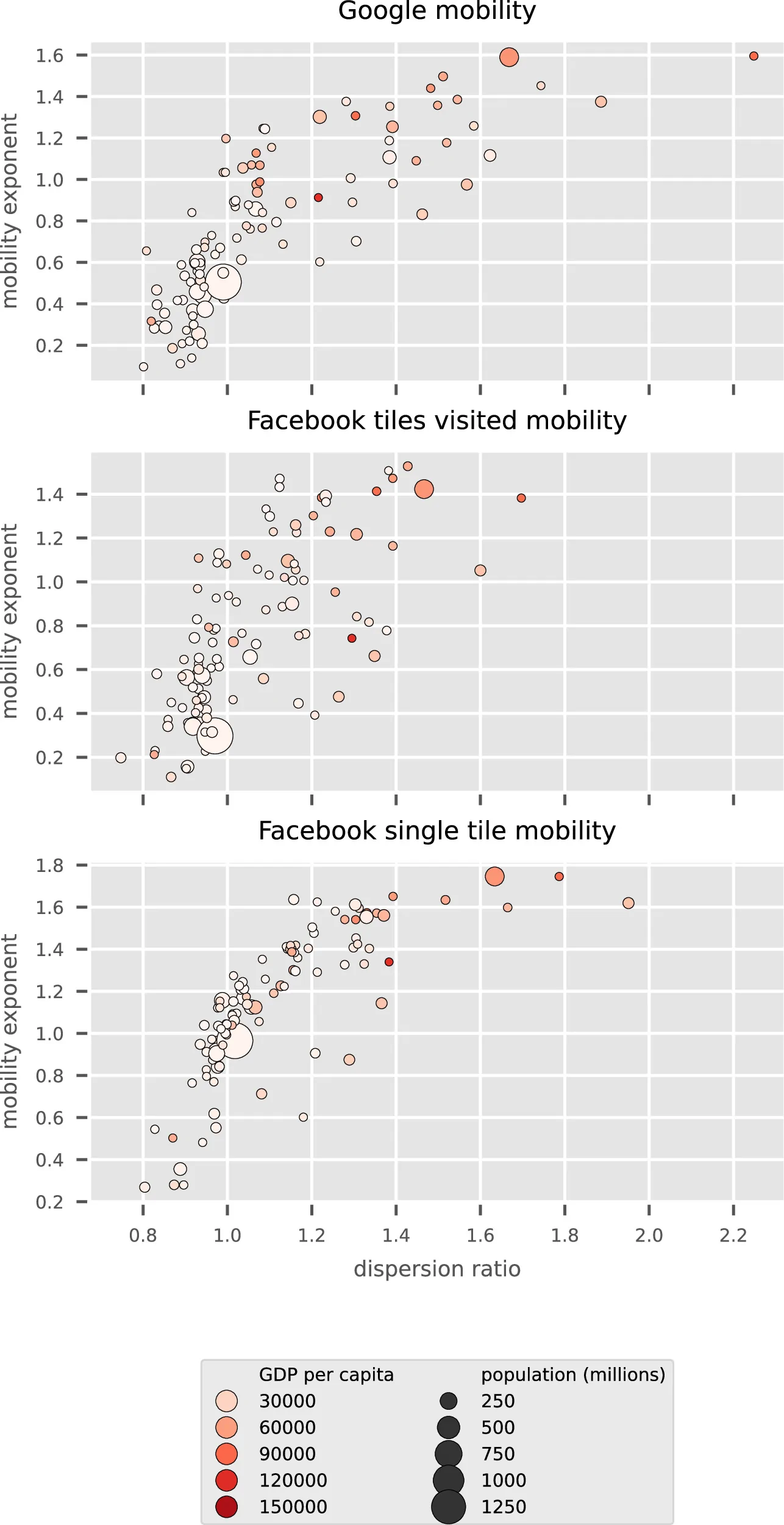
Median mobility exponent plotted against dispersion ratio values by country. The mobility exponent parameter values show the value of the parameter needed to transform the mobility metric to optimise its effect on transmission. As in Fig. 3, dispersion ratio is intended to represent the strength of evidence for an effect of mobility on transmission. Markers coloured by gross domestic product per capita in US dollars in 2020. Marker size shows population in millions. GDP, gross domestic product. Interactive versions of these plots are available at https://monash-emu.github.io/outputs/renewal-covid/.

Full results of this analysis are presented in the Supplementary Information. Many lower-income countries (as defined by the World Bank^25^) had dispersion ratios of close to one, several of which also had mobility exponent parameters approaching zero, consistent with no evidence of an effect of mobility (Fig. 4). However, most higher-income countries showed reduced dispersion when mobility was included, with values of the mobility exponent parameter in the range 0.8 to 2. This implies a linear to quadratic mapping between the mobility time series and the optimal scaling of the transmission rate due to mobility, indicating that while a direct mapping of mobility to transmission was optimal in some settings, others required close to quadratic scaling.

Patterns often appeared to be grouped by region. Many countries of Western Europe showed posterior distributions of the mobility exponent above one under the Google analysis, along with high-income countries of North America, Western Asia and some countries of Northern and Eastern Europe, while several countries of Southern Europe had distributions centred around one. By contrast, many countries of Africa and Southern Asia had posterior distributions that were skewed towards zero, typically in the same settings where dispersion ratios remained close to one, indicating little evidence for an effect of mobility.

For the Facebook tiles visited metric, several countries of south-eastern Africa showed posterior distributions centred above one, while most other African countries had mobility exponents close to zero. By contrast, for the Facebook within tile metric, many countries had uninformative posterior distributions, although exponent values approaching two were suggested for the United States, Türkiye and several countries of Western Europe.

### Google mobility locations

The Google mobility analysis involved estimating a weight parameter for each of the six location types during calibration, such that a higher weight suggested a greater contribution of that location’s metric to the composite mobility function and reflected the domains that were most closely aligned with transmission variation in each setting. Distinct patterns by country were observed, with certain locations being weighted towards the maximum pre-normalisation value of one and others being weighted towards zero (Supplementary Information).

**Fig. 5:**
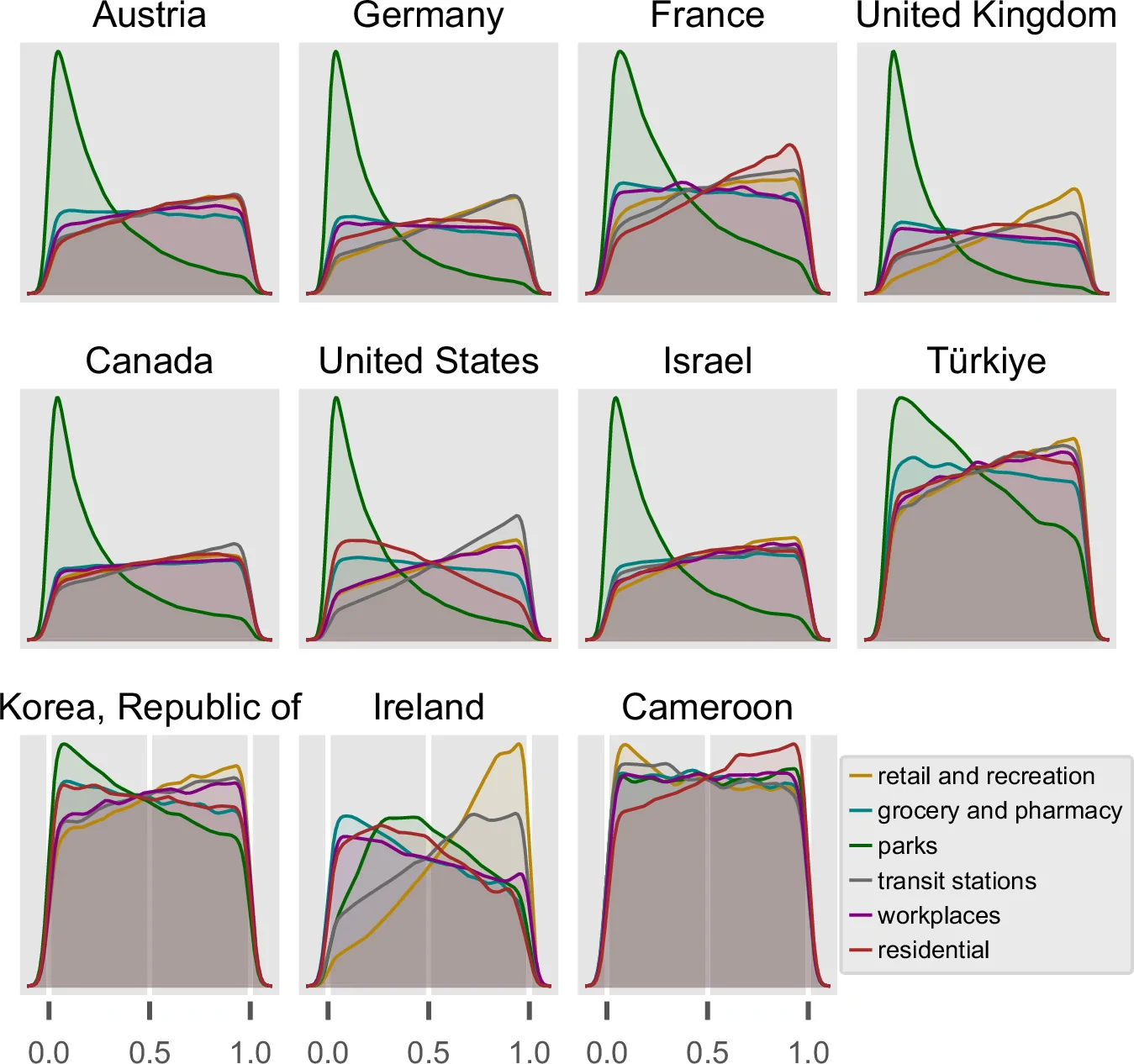
Google mobility location weight parameter posterior distributions (before normalisation) for selected illustrative countries. See Supplementary Information for results for all countries for which Google mobility data was available.

Across many high-income countries, there was a consistent pattern of the posterior distribution of the weight associated with the parks mobility metric having a mode near zero (Fig. 5), indicating that mobility changes in parks contributed little to explaining transmission variation. This included many countries of Europe (e.g. Austria, Germany, France, the United Kingdom), high-income countries of North America (e.g. Canada, the United States) and several Asian countries (Israel, Türkiye, the Republic of Korea). The weight for the retail and recreation location approached the upper bound of one in the analysis of Ireland.

A trend towards higher weights for the residential location was suggested in some countries, which appeared to be attributable to particularly sharp changes in non-residential mobility that were greater than the decline in transmission needing to be offset by an increase in residential mobility. Higher weights for the residential mobility location in some countries in Asia and Africa (e.g. Cameroon) may also reflect relatively smoother changes in this metric at times when transmission was changing only gradually.

### Robustness checks

To assess the robustness of our modelling framework, we conducted a sensitivity analysis in which we explicitly incorporated host-related waning immunity with time from infection. This produced non‑mechanistic transmission scaling functions and dispersion parameter posteriors that were essentially unchanged from the base case configuration (Supplementary Information). We also conducted an identifiability analysis in which synthetic data were generated from parameter sets sampled from the calibrated posteriors, after which the model was refit to these data with all other parameters fixed to their generating values and only the non‑mechanistic transmission updates and mobility‑mapping exponent free to vary; full details are provided in the Supplementary Information.

## Discussion

We present harmonised analyses of the effect of changes in mobility metrics on transmission during the COVID-19 pandemic, with clear patterns emerging across groups of countries that shared characteristics such as income status and geographical location. In many settings, there was a notable correspondence between mobility estimates and the degree of non‑mechanistic transmission scaling needed to reproduce observed epidemiological trends (Fig. 2). This correspondence was reflected in reduced variability in the non-mechanistic component of the model when mobility was introduced, implying that mobility metrics provided meaningful behavioural information in these settings. The consistency of these findings across numerous high-income countries provides evidence that changes in population mobility were strongly associated with short-term transmission dynamics in multiple settings. By contrast, a clear correspondence was not observed in many countries of Africa, Asia, Eastern Europe and South America, highlighting important heterogeneity in the informativeness of digital mobility data across global settings.

The strongest evidence for an effect of mobility on transmission was seen in several high-income countries, including those of Western Europe and North America (Fig. 3). In these settings, the close correspondence between the non-mechanistic transmission scaling function in the base case analysis and the observed mobility metric, together with the marked reduction in the need for transmission scaling when mobility was included, indicates that mobility provided substantial explanatory value for short-term transmission dynamics. This pattern was particularly evident for countries such as Ireland, the United Kingdom and Czechia, where inclusion of mobility substantially reduced the model’s reliance on its non-mechanistic component. Given the structure of our framework, this would be consistent with mobility accounting for a major share of the short-term variation in transmission beyond that attributable to the emergence of new variants during the pre-vaccination period in these countries.

By contrast, there was limited evidence for an effect of mobility on transmission for many countries in Africa and the Arabian Peninsula, as well as the Russian Federation and several countries of Eastern Europe and South America. In these settings, a genuinely weaker epidemiological relationship between mobility and transmission is plausible: in many low‑income settings, core subsistence, caregiving and informal work-related contacts may have been important for transmission but less amenable to behavioural change, even when aggregate mobility fluctuated. Data‑related factors are also likely to have contributed. Mobility datasets in some countries are likely to have lower or more variable representativeness, with changes in the underlying smartphone‑using population and platform uptake potentially obscuring behavioural signals. The strong secular upward trends observed in several Google mobility categories typically affected only specific location types, did not align with known changes in population movement and were absent from the Facebook data. This suggests that these trends reflect shifts in data capture or location categorisation rather than true behavioural change. Model misspecification is also a possible contributor in principle. However, we applied our framework only to countries with surveillance data of sufficient quality to support calibration across multiple indicators, and the same model structure produced consistent results in many high-income settings. Although pre‑Delta variants were rarely included in the analyses of African countries due to data limitations, the marked regional differences observed appear most plausibly explained by epidemiological or data‑related factors.

In many of the countries for which a correspondence between transmission and mobility metrics was observed, the value of the exponential parameter needed to optimally scale the mobility time series was one or greater (e.g. Ireland, the Netherlands, Denmark and the United States, see Fig. 4). This is epidemiologically plausible because a reduction in mobility could affect both the infector and infectee, resulting in a quadratic effect. Alternatively, if infectious individuals are socially immobile and the mobility time series reflects the rate of interactions of the remainder of the general population, linear effects may be observed. Data-related factors may also influence the estimated exponent; for example, a decrease in the variation of the mobility metric could artefactually increase the degree of scaling of the exponent parameter required to align it with transmission. Nevertheless, our results indicate that the public health impact of behavioural change cannot be assumed to follow a simple proportional relationship with mobility metrics.

The epidemiological messages were broadly consistent between the Google and Facebook analyses, with various measures of population mobility (e.g. Facebook tiles visited, Google retail and recreation, Google grocery and pharmacy) typically changing in concert^20^. However, the Google analyses had greater flexibility, because of the greater number of parameters used in assigning weights to each of the available locations when constructing the composite mobility estimate. The metrics also differ conceptually, with Google relating to visits to locations with specific purposes, whereas Facebook captures spatial movements. For some countries, results may have differed if negative location weights had been permitted under the Google analysis (particularly for the parks and residential mobility estimates). During the pandemic period, Apple also provided a mobility time series metric and analyses of these data were particularly illuminating because metrics by transportation type were reported. Unfortunately, the terms of use of these data precluded their inclusion in this manuscript and although we contacted Apple to request its inclusion in this analysis, Apple denied our request.

Our analyses provide a more comprehensive international view of the effects of mobility changes over time than other studies of which we are aware, many of which have focused on one or several high-income settings or considered policy indicators rather than empirical mobility metrics^14,26–33^. By contrast, our harmonised framework combined with the deep epidemiological understanding of the COVID-19 pandemic now available allowed direct comparison of mobility-transmission relationships within and between geographically or culturally connected regions. Through this approach, distinct and coherent patterns emerged naturally, including the striking similarity between mobility metrics and transmission in some settings and the consistent effects of mobility changes in high-income countries. Although our findings are broadly consistent with earlier studies showing that individual PHSMs can substantially influence the effective reproduction number in high-income countries, our results extend this understanding by demonstrating that these relationships are highly heterogeneous globally and by identifying the relative importance of specific location types to transmission.

For policymakers to influence the course of a pandemic, two components are necessary: policies must be implemented that result in behaviour changes, and these behaviour changes must translate to a reduction in transmission. Our analysis aims to address only the second of these two components. Our model also did not incorporate variation in testing rates, the effect of contact tracing or improvements in clinical care. We also did not simulate the effects of vaccination prior to population vaccination coverage reaching 5%, deliberately excluding time periods of rapid vaccination roll-out. We aimed to incorporate the main immunity‑related processes relevant to short‑term transmission dynamics, including variant‑specific susceptibility profiles, directional cross‑variant immunity and a sensitivity analysis addressing waning immunity. We did not, however, represent the full range of possible cross‑immunity strengths between specific variant pairs, but rather adopted the simplifying assumption of fixed levels of protection from earlier to later variants. With regards to data-related considerations, strong secular trends in some metrics may reflect data limitations; Google mobility locations with lesser variation may have received higher weights if no location mapped well to transmission; and our findings regarding the scale of the mobility effect could be over-estimated if variation in the mobility time series is artefactually low. This latter consideration may be particularly important for the Facebook within-tile metric which considers the time spent within a 600m-sided Bing tile, and so may be less likely to represent opportunities for transmission.

Our results strongly support the conclusion that population mobility metrics varied in concert with transmission in many high-income countries. Although consistent patterns emerged within geographical and cultural groups of countries, the optimal choice of mobility time series and the degree of scaling required differed markedly between regions. This indicates that the various mobility metrics capture different aspects of human behaviour, which influence transmission in ways that depend on the local epidemiology and social contact patterns. In the countries in which mobility was most informative, attendance at locations including retail and recreation, grocery and pharmacy, and transit stations were most often associated with transmission, whereas visits to parks were not. This is consistent with public health guidance that restricting visits to enclosed indoor locations can mitigate transmission of respiratory pathogens^34,35^, whereas restricting visits to outdoor locations may not. Our inferred scaling relationships show that behavioural interventions do not necessarily translate to proportional changes in the rate of transmission. In several settings, exponent values greater than one suggested that reductions in mobility were associated with disproportionately larger decreases in transmission – potentially reflecting simultaneous changes for both infectors and infectees or particular reductions in opportunities for high‑risk contact. Looking towards the next pandemic, our findings highlight the importance of using context‑specific behavioural indicators when designing PHSMs, as the informativeness of mobility metrics varies markedly across global settings.

## Methods

Because this study used only publicly available aggregate data and did not involve human participants, ethics approval and informed consent were not required. Full methods are described in the Supplementary Information, which was generated directly from the code used to run the analysis to ensure consistency between implementation and documentation^36^. Here, we summarise the key elements of the modelling framework, which combines mechanistic components expected to influence short‑term transmission with a regularised non‑mechanistic process designed to capture residual temporal variation. This structure, together with a nonlinear mapping between mobility metrics and transmission, underpins the inferential approach used to assess the contribution of mobility to short‑term changes in transmission.

### Epidemiological model

We created a software platform for renewal modelling that incorporated the key mechanistic features expected to influence short-term transmission, including susceptible depletion and multiple competing SARS-CoV-2 variants. In this framework, we implemented a gamma-distributed renewal process with daily innovations^37^. New variants with increased transmissibility, partial immune escape and variable severity were seeded into the system with triangular pulses^38–40^. In our base case analysis, individuals previously infected with a particular variant were assumed to derive complete immunity to reinfection with that variant or earlier emerging variants.

### Residual scaling

We also included a regularised non-mechanistic scaling process to capture short-term fluctuations in transmission that were not explained by the mechanistic components of the model. This process evolved through weekly updates to the log-transformed scaling term, with each update assigned a normal distribution centred at zero and with its standard deviation governed by a single calibrated dispersion parameter. The dispersion parameter was assigned a half-normal prior, which penalises larger changes and therefore constrained the non-mechanistic process to introducing additional variation only when the mechanistic structure alone could not reproduce the transmission dynamics. The dispersion parameter therefore quantifies how much short‑term variation in transmission must be attributed to the non-mechanistic scaling process and remains unexplained by the mechanistic model.

### Mobility scaling

In the reference analysis without mobility included, no adjustment was made for mobility. However, for the mobility analyses, the instantaneous reproduction number was scaled according to mobility changes using data from Google and Facebook^23,24^. Under the Facebook analyses, transmission at time t was scaled by ${{{M}_{t}=f}_{t}}^{m}$ where ${f}_{t}$ is the Facebook mobility metric at time t and m represents the mobility exponent parameter that governs the strength and nonlinearity of the mapping from mobility to transmission. Under the Google analyses, we scaled transmission by ${M}_{t}={\left(\frac{{\sum}_{l}{w}_{l}{g}_{l,t}}{{\sum}_{l}{w}_{l}}\right)}^{m}$, where ${g}_{l,t}$ is the Google mobility metric for location l at time t and ${w}_{l}$ is the weight assigned to each Google mobility location. For countries in which the Google mobility metrics exhibited strong secular increases and reached values beyond two by the end of the reporting period, we ran an additional analysis in which each location-specific metric was linearly detrended before inclusion.

### Model summary

Full model equations are provided in the Supplementary Information. Briefly, incidence is generated through the following renewal process:

1 $${i}_{p,v,t}={n}_{p,t}{x}_{p,v}\left(1-{e}^{{-\lambda }_{v,t}}\right),$$

and the variant-specific force of infection at time t is given by:

2 $${\lambda }_{v,t}=\beta {\sigma }_{v}{e}^{{W}_{t}}{M}_{t}{\sum}_{\tau=t-50}^{t-1}({s}_{v,t}+{\sum}_{{p}^{{\prime} }}{i}_{{p}^{{\prime} },v,t}){g}_{t-\tau }$$

Here, p indexes prior variant exposure status, v indexes the modelled variants, and t indexes model time. The population with variant exposure status p at time t is denoted ${n}_{p,t}$, while ${x}_{p,v}$ denotes susceptibility to infection with variant v for individuals with variant exposure status p. Infectiousness is scaled by β and adjusted through a variant-specific factor ${\sigma }_{v}$. The non-mechanistic transmission scaling process is denoted ${W}_{t}$, while ${s}_{v,t}$ denotes the seeding rate with variant v at time t, and g is the generation interval distribution.

### Calibration

We calibrated all models to national time series of deaths and case notifications, as well as one hospitalisation indicator where available. In addition to the starting strain, we explicitly represented one or both of the Alpha and Delta variants of concern, if sufficient data were available to inform the profile of their emergence and where the analysis period spanned their emergence. We then ran the analysis from when the per capita death rate first reached two per million population until population vaccination coverage first reached 5%. We also calibrated to seroprevalence data where available to broadly capture the size of the epidemic and extent of population immunity, although we assigned a lower contribution of this indicator to the overall likelihood. The modelled outputs used to compare against the time series of deaths, cases and hospital-related indicators were calculated by applying a series of gamma-distributed convolutions to the modelled incidence. These methods were followed for all countries except Oceania and Singapore, for which a sequence of Omicron sub-variants was considered (BA.1, BA.2 and BA.5), and we focused on a time during which high and stable vaccination coverage had been achieved but with low levels of pre-existing natural immunity.

### Implementation

We implemented the above-described model and its associated analysis using the JAX library in Python, which allows for autodifferentiable just-in-time compiled model code, and convenient use of no U-turn sampling in NumPyro for calibration^41–43^. We chose Pixi for environment and project management to ensure model outputs are reproducible across users and architectures^44^. 

### Additional analyses

We conducted a sensitivity analysis in which explicit waning of post‑infection immunity was incorporated into the model. We also performed an identifiability analysis by refitting the model to synthetic data generated using selected parameters fixed to values sampled from the base case fits, allowing only the non‑mechanistic transmission updates and the mobility‑mapping exponent to vary.

### Reporting summary

Further information on research design is available in the Nature Portfolio Reporting Summary linked to this article.

## Supplementary information

Supplementary Information

Reporting Summary

Transparent Peer Review file

## Data Availability

The epidemiological, mobility and other input data used in generating these analyses are available at https://github.com/monash-emu/renewal/tree/main/data, with the scripts used to download and process these inputs prior to storage provided at https://github.com/monash-emu/renewal/tree/main/notebooks/inputs. Selected outputs from model runs are available at https://github.com/monash-emu/renewal_data, together with instructions for recreating the figures from these outputs. Full simulation outputs for all countries have not been deposited due to their size; however, all analyses can be reproduced from the publicly available code, input data and commit identifiers provided in the Supplementary Information. The complete simulation outputs are also available from the authors upon request.

## Code availability

The code used for the simulations presented in this study is available at https://github.com/monash-emu/renewal and has been archived in Zenodo at https://doi.org/10.5281/zenodo.21349171^45^. Instructions for reproducing the analyses are provided in the repository documentation.
