# Supplementary material for: Evaluating the effects of population mobility on transmission during the COVID-19 pandemic
Source: Nat Commun. 2026 Oct 14;17:11634. doi: 10.1038/s41467-026-78247-9 (PMC13755426; doi:10.1038/s41467-026-78247-9)
Supplement: Supplementary file 1 [file 41467_2026_78247_MOESM1_ESM.pdf]

Full Supplemental Appendix

Table of contents

Documentation 2

Analysis methods 2

Summary equation . . . 2
Renewal model . . . 3
Generation interval . . . 4
Renewal process . . . 4
Variant seeding . . . 4
Immunity . . . 4
Waning immunity . . . 5
Outputs . . . 5
Modelled population size . . . 6
Analysis period . . . 6
Mobility . . . 7
No mobility . . . 8
Google mobility . . . 8
Facebook mobility . . . 8
Residual transmission process . . . 8
Calibration targets . . . 9
WHO indicators . . . 9
Hospitalisations . . . 10
Seroprevalence . . . 10
Variants . . . 11
Calibration algorithm . . . 13
Initialisation . . . 13
Parameter processing . . . 13
Algorithm . . . 14

Selection of countries for inclusion 14

Parameter choices and supporting evidence 22
Time period parameters . . . 22
Proportion parameters . . . 32
Fixed parameters . . . 35
Between-variant relative parameters . . . 36

Calibration results 37
Country grouping . . . 37
Prior posterior comparisons by continent and country, base case . . . 38
Africa . . . 39
Asia . . . 71
Europe . . . 95
North America . . . 132
Oceania . . . 146
South America . . . 150
Prior posterior comparisons by continent and country, waning immunity included . . . 161
Africa . . . 162
Asia . . . 194
Europe . . . 218
North America . . . 255
Oceania . . . 269
South America . . . 273

Comparison of the non-mechanistic scaling of transmission process with mobility domains 284
Individual mobility metric comparisons (Google and Facebook) . . . 285
Google retail and recreation mobility metric comparison . . . 285
Google grocery and pharmacy mobility metric comparison . . . 294
Google parks mobility metric comparison . . . 303
Google transit stations mobility metric comparison . . . 312
Google workplaces mobility metric comparison . . . 321
Google residential mobility metric comparison . . . 330
Facebook tiles visited mobility metric comparison . . . 339
Facebook single tile mobility metric comparison . . . 349
Weighted scaling process versus residual comparison . . . 359
Africa . . . 359
Asia . . . 361
Europe . . . 363
North America . . . 366
Oceania . . . 367
South America . . . 367

Comparisons of the non-mechanistic scaling of transmission process under each mobility configuration **367**
Results by continent . . . 369
Africa . . . 369
Asia . . . 373
Europe . . . 376
North America . . . 381
Oceania . . . 383
South America . . . 384

Comparisons of the non-mechanistic transmission scaling dispersion parameter **386**
Dispersion parameter distributions by analysis and country . . . 387
Africa . . . 387
Asia . . . 389
Europe . . . 391
North America . . . 394
Oceania . . . 395
South America . . . 396

Mobility exponent parameter posteriors **396**
Mobility exponent parameter posterior distributions by country . . . 397
Google mobility . . . 397
de-trended Google mobility . . . 398
Facebook tiles visited mobility . . . 399
Facebook single tile mobility . . . 400

Mobility weight posteriors **400**
Results by continent . . . 402
Africa . . . 402
Asia . . . 404
Europe . . . 406
North America . . . 409
Oceania . . . 410
South America . . . 411

Effect of waning immunity **411**
Example dispersion parameter posterior distributions . . . 412
Quantile-quantile posterior comparisons . . . 413

Identifiability analysis **420**
Analysis steps . . . 420
Analysis results . . . 421
Equatorial Guinea, Facebook tiles visited mobility . . . 421

Angola, Facebook single tile mobility 422
United Kingdom, no mobility 423
India, Facebook tiles visited mobility 424
Portugal, no mobility 425
Israel, Google mobility 426
Qatar, Google mobility 427
Germany, Facebook single tile mobility 428
Puerto Rico, no mobility 429
Ireland, Google mobility 430
Gabon, Facebook single tile mobility 431
Czechia, Facebook tiles visited mobility 432
United Arab Emirates, Facebook single tile mobility 433
Sri Lanka, Google mobility 434
Korea, Republic of, Facebook single tile mobility 435

Commits used for analyses **436**

References **443**

Documentation

The following document was documented algorithmically from the code used in the analysis. This version was produced from commit 2cab751.

The rationale for our methods is provided in the Methods section of the main manuscript.

Analysis methods

Summary equation

The mechanics of the renewal process can be summarised in the following equations, the detail of which is expanded in the following sections.

$$i_{p,v,t} = n_{p,t} x_{p,v} (1 - e^{-\lambda_{v,t}})$$

$$\lambda_{v,t} = \beta \sigma_v e^{W_t} M_t \sum_{\tau=t-50}^{t-1} (s_{v,\tau} + \sum_{p'} i_{p',v,\tau}) g_{t-\tau}$$

$$n_{p,t} = n_{p,t-1} - \sum_{v} i_{p,v,t} + i_{\tilde{p},\tilde{v},t} - qzn_{p,t-1} + qzr_p \sum_{p'} n_{p',t-1}$$

$$M_t = \begin{cases} 1 & \text{if no mobility} \\ (\frac{\sum_l w_l G_{l,t}}{\sum_l w_l})^m & \text{if Google mobility} \\ F_t^m & \text{if Facebook mobility} \end{cases}$$

$$o_t = a \sum_{v} (\prod_{v'=v_1}^{v} y_{v'}) \sum_{p} \sum_{\tau=1}^{50} i_{p,v,t-\tau} \times c_\tau$$

$$h_t = \sum_{\tau=1}^{50} o_{t-\tau} (1 - \sum_{u=0}^{\tau-1} d_u)$$

Where:

- $i_{p,v,t}$ represents the incidence of variant v in immunity status group p at time t, where p represents the immunity status prior to infection with variant v
- v takes sequential values from v_1 to v_V, where V is the number of variants included in the analysis
- $p \in \{0,1\}^V$ are the possible combination of past variant exposure statuses with V variants
- $n_{p,t}$ is the size of the population with immunity status p at time t
- q indicates whether waning immunity is incorporated (i.e. $q = 0$ base case analysis, $q = 1$ waning immunity sensitivity analysis)

- z is the reciprocal of the duration of natural immunity
- r_p indicates whether $p = \{0\}^V$ and represents the infection-naive population (i.e. $r = 1$ if $p = \{0\}^V$ and $r = 0$ otherwise)
- $x_{p,v}$ is the relative susceptibility of persons with immunity status p to infection with variant v
- $x_{\{0\}^V,v} = 1$, full susceptibility for those never previously infected
- $x_{p,v} = 0$ for infection with a variant to which the person has previously been infected with or a preceding variant (e.g. infection with Alpha following infection with Delta)
- $x_{p,v}$ for immunity statuses other than the two preceding cases is the complement of a single calibrated cross-immunity parameter with a beta-distributed prior
- β is the infectiousness of the starting variant
- σ_v is the infectiousness of variant v relative to the starting variant
- W_t is the non-mechanistic residual transmission process, under which a Wiener sequence of updates from a starting value of zero is linked with a cosine spline at time t
- $s_{v,t}$ is the rate of seeding with variant v at time t
- g_t is $\int_t^{t+1} g(u)\,du$, where g is the probability density function of the generation interval distribution
- $\tilde{v}$ is the variant for which infection results in transition from immunity status $\tilde{p}$ to immunity status p
- $G_{l,t}$ is the Google mobility estimate at location l and time t
- F_t is the Facebook mobility estimate (either tiles visited or single tile) at time t
- w_l is a calibrated location-specific weight parameter with support $[0, 1]$
- o_t is any one of the incident indicator quantities calculated from the incidence time series, i.e. either deaths, case notifications, hospital admission or ICU admissions
- y_v is the relative severity of the infecting variant relative to the preceding variant, with $y_1 = 1$ (i.e. the severity of the starting strain is set to one)
- c_t is $\int_t^{t+1} c(u)\,du$, where c represents the probability density function of the distribution of the delay from the onset of the infection episode to the onset of the incident indicator considered
- a is the proportion of infection episodes resulting in the indicator considered
- h_t is either one of the prevalent indicators calculated from the incident indicators, i.e. either hospital occupancy or ICU occupancy
- d_t is $\int_t^{t+1} d(u)\,du$, where d represents the probability density function of the distribution of the time from the onset of the incident indicator (either hospital or ICU admission) to the end of the prevalent indicator period (either hospital or ICU discharge)

Renewal model

We developed a discrete renewal model with daily innovations. The core aspect of our approach was the convolution of the preceding strain-specific incidence rate with the generation interval for subsequent infections, adjusted for past immunity. We then applied convolutions

to this model to estimate model outputs that could be compared against our indicator targets described below.

For all analyses, the starting population was assigned to the fully susceptible category.

Generation interval

A gamma-distributed generation interval was used for the renewal process. This is consistent with an investigation of household clusters from Germany that showed the profile of serial intervals was well matched by a gamma distribution.[1] The generation interval was truncated at 50 days, because the density reached negligible values beyond this point.

Renewal process

The strain-specific incidence array updated for strain seeding was convolved with the generation interval distribution vector to create an array of the effective number of infectious individuals for each strain. These quantities were then multiplied by scalar values representing residual transmission scaling and adjustment for mobility and divided through by the population size (to obtain the scaled per capita infectious population). This was multiplied by the strain-specific vector for the relative infectiousness of each strain to calculate a per capita rate of infection. We then determined the actual force of infection for infection-naive persons for each strain as $1 - e^{-\lambda}$ (where λ represents the calculated infection rate) to ensure that the per capita risk of infection could not exceed one in a time step.

Variant seeding

Each strain (including the starting strain) was seeded into the model using a triangular pulse of new infections for which the peak per capita seeding rate was specified as a parameter. At each calculation day, the new strain-specific seeding values were added to the most recent value for the strain-specific history of incidence. Infectiousness of each variant was specified with reference to the first modelled variant strain.

Immunity

The strain-specific force of infection was then applied to each possible immunological past history of preceding exposure to variant combinations and their associated susceptibility to infection to calculate the number of people infected from each category and transition them to their new immunological states. Persons who had never previously been infected with any strain were considered to have no immunological protection, and the rate of infection was not adjusted further. We considered partial cross immunity was provided by infection with

a preceding variant strain to infection with subsequent strains. However, previously infected persons were assumed to derive complete immunity to the infecting strain and to variant strains that had emerged prior to the infecting strain (for example, past infection with Delta conferred complete immunity against future infection with Alpha).

Waning immunity

For our sensitivity analyses in which waning host-related immunity from past infection was incorporated, individuals were transitioned from each immune category into the fully susceptible population at a constant rate represented by the reciprocal of the duration immune.

Outputs

Once an iteration of the renewal analysis had been run to calculate incidence values over time, the other epidemiological outputs were calculated using a series of convolution operations applied to this series. Cases were calculated by convolving the total incidence summed over strains with a gamma-distributed set of delays from incidence to notification which was truncated after 50 days. This was then multiplied through by the case detection rate (a proportion) and weekly cases were calculated by summing over the preceding 7 days.

By contrast, deaths were calculated from the strain-specific incidence to allow for strain-specific severity for analyses considering the pre-Omicron/pre-vaccination period. The severity of each variant was interpreted with reference to the preceding modelled variant, such that the severity of each variant relative to the starting variant was calculated as the cumulative product of the severity parameter for each modelled emerging variant. Strain-specific variant severity was not included for the Omicron era analyses that considered Omicron subvariants BA.1, BA.2 and BA.5. A separate set of parameters governed the time from incidence to death with the fraction of incident episodes resulting in death given by the infection fatality rate adjusted for strain-specific severity. For Singapore and countries of Oceania, a reduction in the risk of death was applied because this analysis was performed after wide-scale population vaccination had been achieved and Omicron sub-variants were the dominant strains, with severity assumed to be equivalent for all Omicron sub-variants. The relative reduction in the risk of death during the later Omicron/vaccination period was set according to a parameter specifying the protection of vaccination against death given infection and was not varied during calibration, because this would have been collinear with the risk of death parameter.

As for cases and deaths, hospitalisations were estimated through a convolution distribution with independently calibrated parameters, a hospital admission fraction, but the same strain-specific severity parameters as for deaths. As for the approach to deaths, strain-specific severity was included for the pre-Omicron period analyses, and a reduction in the risk of hospitalisation was applied to account for vaccination for the Omicron period analyses. The relative reduction

in the risk of hospitalisation was set according to a vaccination protection against hospitalisation given infection parameter. As for cases, deaths and hospitalisations, the convolution of time to ICU admission was parameterised independently.

Hospital and ICU occupancy were obtained by convolving the time series of hospital and ICU admissions with the complement of the cumulative distribution of the time to hospital or ICU discharge. For comparison to serosurveillance data, seropositivity was calculated as the complement of as the proportion of the population remaining in the entirely infection-naïve immunity sub-population. Finally, the proportion of incidence attributable to each variant strain was calculated from the strain-specific incidence.

The parameters to each gamma distribution used in our analysis were parameterised by analytically calculating the "a" (shape) and scale parameters from the mean and standard deviation determined by our literature review.

Modelled population size

We used the total population size estimated by the World Bank where a population size was available from this source, and used the estimate provided by the United Nations Department of Economic and Social Affairs otherwise. Population data was downloaded from the World Bank on 1 April 2025. The population size for 2020 was used for all countries except for Singapore and countries of Oceania, for which the population size in 2022 was used (because of the later analysis period for these countries). UN DESA population data was downloaded on 18 March 2025 and where UN DESA population was needed, data for 2020 was used.

Analysis period

For all countries, we aimed to place our analysis period during a time period for which the variation in transmission rate may have been substantially attributable to changes in mobility, although we developed an approach (described below) to address the consideration that the emergence of new SARS-CoV-2 variants likely contributed substantially. Therefore, we aimed to select time periods during which the roll-out of vaccination programs would have contributed less to variations in transmission rate.

For all countries other than Oceania and Singapore, we addressed this by limiting our analysis period to the time prior to vaccination reaching levels that may have had a significant population-level effect.

For most countries, a single base case no mobility analysis was run, against which the Google and Facebook analyses could be compared. However, for Oceania and Singapore, the analysis period for the Facebook analysis was shorter due to the discontinuation of Facebook data after May 2022. For these countries, an additional no mobility comparison analysis was run. For these countries, the start of the calibration period was set to be the time at which the per

capita daily rate of deaths passed 2 deaths per million population. However, if this threshold was not reached by 1 June 2020, the simulation commenced at this default time instead. For Singapore and countries of Oceania, the simulation commenced from the time that vaccination coverage reached 90% of its final value.

For all countries other than Singapore and those of Oceania, the end time for the analysis was calculated as the time that population vaccination coverage passed 5%, provided that vaccination coverage did reach this value before the default end time of 1 December 2021. Otherwise, this default end date was used instead. For Oceania and Singapore, the latest date for which Google mobility data was available was used. We substituted Germany's data for Switzerland and Ireland because these two countries had almost identical profiles of vaccine doses administered per person in the earliest phases of the roll-out and vaccination data was not available for these two countries through the period when coverage approached the analysis start threshold. Similarly, we substituted Great Britain for Qatar based on the same rationale.

Mobility

The main purpose of our analysis was to understand the effect of empirically observed changes in mobility on the observed fluctuations of the COVID-19 epidemic. We addressed this by including various mobility observation streams available from Big Tech companies within our model.

For each country, we ran one analysis with no mobility scaling to the transmission rate. We further ran one analysis in which Google mobility was used to scale the transmission rate, if mobility data was available from Google. For countries for which any of the Google mobility domains reached an average value of 2 or above during the last 30 (i.e. 2 times the starting value which is normalised to one), we additionally ran a detrended Google mobility analysis. Under this detrended analysis, each Google location was individually detrended by dividing through by a linear estimate of the increase in that mobility domain over time. We also ran two analyses in which Facebook mobility was used to scale the transmission rate, if mobility data was available from Facebook. Although Apple mobility data was available and we were able to run analyses using this data source, Apple's terms of use indicate that this source of data cannot be used for this purpose. We contacted Apple, who declined to allow their data to be used for this project. For all mobility sources, we smoothed the raw data using a 7-day centred rolling average. For all analyses incorporating mobility scaling, we used an exponential scaling parameter (described in more detail below) which was assigned a uniform prior over domain [0, 2].

No mobility

For the analysis without mobility, no empiric data was used to scale the transmission rate over time (which was implemented by setting the mobility scaling value to one throughout the simulation).

Google mobility

We obtained mobility data from the Google Community Mobility Reports on 14 January 2025 and extracted national mobility estimates by Google mobility domain. For this analysis, we used the raw value interpreted as a percentage plus one to scale the transmission rate. This mobility analysis type used a set of priors weighting each mobility domain, along with one further prior governing the overall effect of the weighted mobility estimate in scaling the transmission rate. The weights for each mobility domain were normalised to sum to one, with the resulting weighted mobility profile exponentiated to the value specified by the mobility exponential parameter.

Facebook mobility

For each geographic region included in the Facebook data, we estimated a population by intersecting polygons with the centroid of population data grids, and then weighted the resulting series by these calculated populations. For the small proportion of (low-population) time series that had missing data, we imputed population estimates by nearest neighbour interpolation. In general, these series were found to have a negligible contribution to the final outputs.

We used the `all_day_bing_tiles_visited_relative_change` for the first Facebook mobility analysis, and scaled transmission transmission according to one plus this mobility metric. For the second Facebook mobility analysis, we used the `all_day_ratio_single_tile_users` estimate and scaled transmission according to one minus this mobility metric.

Because weighting was not required, only one mobility-related parameter was needed under each of these approaches, which specifies the mobility exponent value for the effect of the mobility data in scaling the transmission rate. This mobility approach was used for each of the two analyses that incorporated Facebook data, using both the tiles visited and the within tile estimates.

Residual transmission process

A Wiener variable process was used to capture variation in transmission over time. The starting value for this process was fixed at zero and the subsequent updates to the process were explored in calibration. This exploration was performed in logarithmic space, with the calibrated values for each update exponentiated before being used to scale the transmission

rate. Each parameter pertaining to the updates to residual transmission scaling was assigned the same prior centred at zero (i.e. no change), and so can be interpreted as the change in the log-transformed residual transmission scaling relative to the previous value. The cosine function was obtained by translating and scaling a half cosine function (i.e. a cosine function with support $[0, \pi]$), such that it intersected the starting point (t_1, y_1) and finishing point (t_2, y_2) with a gradient of zero at both of these points. This choice of fitting approach ensured that the residual transmission scaling function, its derivative and its higher order derivatives are continuous. Each time point for fitting the residual transmission scaling process was set at intervals through the analysis period spaced by 7 days working backwards from the end of the analysis period. The scaling process was then fit to these points using piecewise cosine functions from a starting value of zero.

Calibration targets

We only included countries for which multiple data streams were available to estimate variations in transmission rates over time. Specifically, we required that a time series for both COVID-19-attributable deaths and case notifications were available. However, we also included one time series for a hospital-related indicator, where this could be sourced. Because we considered that the emergence of new SARS-CoV-2 variants could have substantially affected transmission, we included explicit calibration targets for the strain-specific proportional prevalence of variants where available. Although possibly less epidemiologically relevant to this analysis, we sought to broadly capture the overall size of the epidemic. This was addressed by explicitly incorporating susceptible depletion and including seroprevalence estimates as calibration targets where available. We did not include vaccination explicitly within the model because our approach to country and time period inclusion was intended to focus on time periods during which vaccination would not have substantially influenced transmission rates.

WHO indicators

The number of deaths by week reported by WHO was used as a calibration target for all included countries. Any values of zero in this series were replaced with a value of 0.5 to enable comparison of the deaths target to our modelled outputs in logarithmic space. The log of the reported target deaths value was compared against modelled deaths by calculating the density of a normal distribution centred at the log-transformed modelled value. Deaths was one of up to three time-series indicators for which a common dispersion parameter was used for this normal distribution for the target comparison. The value for weighting the deaths indicator time series was set to 20 (a quantity that can be interpreted in relation to the weights of the other indicators, but whose absolute size is arbitrary).

The number of cases by week reported by WHO was included as the second calibration target for all countries. As for deaths, any zero values were replaced with a value of 0.5. We only calibrated to cases from 1 June 2020 onward, because we considered that prior to this time

many countries were still rapidly scaling up testing capacity and this indicator may have been less reliable. Cases was the second indicator for which a common dispersion parameter was applied. A total weight was calculated for the overall time-series of cases such that the weight for each individual observation (date) of the cases time-series was equal to that for each death observation.

Hospitalisations

A maximum of one hospitalisation-related indicator for calibration was chosen using a hierarchical approach. In selecting the indicator, the number of new admissions was preferred over estimates of total bed occupancy, and total hospital indicators were preferred over ICU indicators. As such, the final hierarchy of indicators was:

1. New hospital admissions
2. Hospital occupancy
3. New ICU admissions
4. ICU occupancy
5. No hospital indicator

That is, the highest ranked indicator was used based on data availability, and no hospital indicator was incorporated if none was available. This indicator was the final calibration target for which a common dispersion parameter was applied. As for the cases target, a weight was applied to the overall hospitalisation time series such that the weight for each observation point was equal to that for each death observation.

Seroprevalence

Seroprevalence data was obtained from SeroTracker on 11 December 2024, with the date for each serosurvey estimate calculated as the mid-point between the reported start and end sampling dates for each survey. This date was then lagged earlier by 14 days for comparison to modelled outputs to account for the delay between infection and the subsequent development of detectable antibodies. We filtered the SeroTracker data to include only the estimate reported as primary from Unity-aligned national-level surveys for which the number of participants was at least 600. We also considered only a maximum of one seroprevalence value for any given date (selecting only the largest of three surveys done on the same day for Mexico). For any consecutive estimates for which a lower estimate followed an immediately preceding higher estimate, we pooled these two estimates and placed the pooled estimate at the mid-point of the dates of the two estimates. We recursively applied this process until seroprevalence estimates were monotonically increasing over time.

We compared the modelled proportion ever infected against the seroprevalence reported at least six months (183 days) after the start of the simulation. We chose to exclude seroprevalence estimates from the early months of the pandemic period because the seropositive proportions reported at this time were less likely to be comparable to our model outputs. This is because our modelled seroprevalence would remain close to zero with plausible parameter values for some time after the start of the analysis, whereas seroprevalence estimates could reach higher values due to factors that include low levels of transmission prior to the analysis period and sampling bias. In contrast, we considered that later seroprevalence should provide a broad indication of epidemic size. For countries for which seroprevalence calibration targets were used, we assigned a target weight to this indicator of 5 (which is an arbitrary quantity in absolute terms, but can be interpreted with reference to the deaths indicator weight of 20). The seroprevalence target had a dispersion parameter that was normally distributed and independent of the dispersion parameters for the other targets, including the shared dispersion parameter of the time series indicators.

We discarded seroprevalence estimates that fell less than 5% away from a value of zero or 100%. We also ignored seroprevalence estimates for Oceania and Singapore, for which the analysis was run largely through 2022, during which time seroprevalence values would not reflect the attack rate during the simulation period. We further ignored seroprevalence estimates from low and lower middle income countries of Africa, because we were unable to obtain good fits for several of these countries while also maintaining plausible detection/severity parameters (i.e. case detection rate, hospital admission rate and infection fatality rate). That is, we applied much lower values for these detection-related parameters in these countries, although the modelled attack rate still often remained below seroprevalence estimates for some countries.

Country income classifications were obtained from the World Bank.

Variants

Data extraction

Reports of the number of isolates of specific variants of SARS-CoV-2 were obtained from the covariants GitHub repository. Each variant-specific file was downloaded and used to create country-specific tables of the variant-specific counts by date. The terms used to identify variants prior to Alpha were: 20A.EU1, 20A.EU2, 20B.S.732A, 21C.Epsilon. The text "Delta" was used to identify Delta variants and the text "21L.Omicron" was used to distinguish the BA.2 variant from later variants (modelled as BA.5). Variant data was considered for dates on which at least 5 sequences were available for the country considered. Further, we required at least 5 such dates be available for that country's variant data to be used as a calibration target.

To obtain data to use as calibration targets for the strain-specific proportional prevalence of both the Alpha and the Delta variants, we used national data for the country analysed where available. If data was not available for the country considered or if all values for the

proportion of the variant were one, we used pooled data from all the other countries from the same continent where available. If insufficient data on a particular variant was available for the country or its continent, prevalence of that variant was not included as a calibration target.

Alpha

For countries other than Singapore and countries of Oceania and Africa, a target for the Alpha variant was included in our calibration algorithm. Calibration against data for Alpha was permitted from the beginning of the simulation period (from 1 January 2020) for these countries. The periods for calibration against the Alpha and the Delta variants were set so as to be mutually exclusive in time. Specifically, the date for transitioning from calibrating against available data for the Alpha to calibrating against data for Delta was set as 1 March 2021. Exceptions were made for several Asian countries for which this transition date was set one month earlier and two countries of North America for which it was set six weeks later. (Specifically, the exceptions were Afghanistan: 1 February 2021, India: 1 February 2021, Nepal: 1 December 2020, Indonesia: 1 February 2021, Saudi Arabia: 1 February 2021, Oman: 1 February 2021, Kuwait: 1 February 2021, South Korea: 1 February 2021, Malaysia: 1 February 2021, Honduras: 15 April 2021, Puerto Rico: 15 April 2021.) If this date occurred after the end of the simulation, the Alpha calibration period continued to the end of the simulation. Comparison of the target estimate against the modelled value was undertaken using a normal distribution with a single dispersion parameter that applied to all modelled variants (but was independent of the time series dispersion parameter). The target weight for the Alpha target was set to 5.

Delta

For countries other than those of Oceania and Singapore, the Delta variant was included if the end date of the calibration fell later than 1 May 2021. The target weight for calibration to Delta was set to 5 for most countries. Exceptions were made if the target time series for Delta began towards the very end of the calibration (last 25 days), in which case a higher weight (of 25) was needed to achieve a plausible fit to the profile of the emergence of this variant with fewer data points.

Omicron BA.2

A calibration target for Omicron BA.2 was only included for Oceania and Singapore. As for other variants, the target weight for BA.2 was set to 5. The BA.2 calibration target for these countries used the data available from 1 January 2022 to 15 April 2022.

Omicron BA.5

As for BA.2, a calibration target for Omicron BA.5 was only included for Oceania and Singapore. As for other variants, the target weight for BA.5 was set to 5. The BA.5 calibration target for these countries used the data available from 1 April 2022 to 1 September 2022.

Calibration algorithm

We used a gradient-based algorithm for model calibration, which efficiently explored our multidimensional parameter space. By exploring all time series in log-space with a common dispersion parameter, we were able to apply an algorithm that provided epidemiologically plausible results for all countries simulated without the need for extensive country-specific adaptations.

Initialisation

To initialise the model parameters, we started all the updates to the transmission scaling process from a value of zero (in logarithmic space) to represent no update, such that the initialisation commenced with transmission scaling being constant over time. For all other parameters, we used `numpyro`'s `init_to_uniform` method, with a radius of 0.1.

Parameter processing

The calibration process estimated parameters for each consecutive update to the transmission scaling process in logarithmic space. The prior distribution for the update for each period of was given by a normal distribution centred at a value of zero to represent no change from the previous value. The standard deviation of each normal distribution is provided by the (single) dispersion parameter of the transmission scaling process introduced above. The dispersion parameter for transmission scaling was set to a half normal distribution with standard deviation 0.5 under analyses both with and without mobility included. This approach was adopted in order to favour lesser changes in transmission scaling at each sequential update point.

For all prior distributions representing durations of time (including the generation interval, time from infection to reporting and time from infection to death), we used a truncated normal prior with the lower truncation limit set at 1 day and the upper truncation limit set at 2.5 times the mean value of the prior. For each of the beta-distributed proportions of incidence resulting in the key outputs for calibration, we analytically calculated the parameters to the beta distribution from the desired mean and standard deviation determined from the literature. These parameters comprised the infection fatality rate, the case detection proportion and, where applicable, the hospital or ICU admission proportion. For high-income countries, the mean value used was as presented in the parameter choices section, whereas this was reduced for other countries. The reductions based on income status were: Low income: 0.4, Lower

middle income: 0.6, Upper middle income: 0.8, High income: 1.0, with Venezuela (for which a World Bank class was not available) considered a low-income country for this purpose.

The starting transmissibility scaling parameter was assigned a uniform prior distribution on domain 0.5 to 3.5.

The seeding rate for each variant was calibrated from 1×10^{-7} to 5×10^{-6} cases per capita per day using a uniform distribution in logarithmic space. The seeding offset (i.e. the time from modelled seeding to the first calibration data point) for each variant except for the first one simulated was calibrated from 4.0 to 90.0 days using a uniform distribution in untransformed space.

The relative infectiousness of each variant compared to the preceding variant (other than the first variant included) was calibrated using truncated normal distributions with mean 1.25, standard deviation 0.2, lower truncation limit 1 and upper truncation limit 2.

As for our approach for infectiousness, the relative severity of each variant compared to the preceding variant (other than the first variant included) was calibrated using truncated normal distributions with mean 1.5, standard deviation 0.2, lower truncation limit 1.0 and upper truncation limit 2.0.

The prior for the shared dispersion parameter for all time series data was a half-normal distribution with standard deviation 0.5. The dispersion parameter for all variant incidence proportions was set at 0.05. The dispersion parameter for all seroprevalence targets was set at 0.2.

Algorithm

We ran the calibration algorithm with a warm-up of 10000 iterations for each of 8 chains followed by 10000 evaluated iterations for the main analysis. We used a Hamiltonian Monte Carlo inference approach with the No U-Turn Sampler (NUTS) with adaptive path length and mass matrix adaptation from `numpyro`.

Selection of countries for inclusion

To select countries for inclusion in our analysis, we first identified all countries for which either Google or Facebook mobility data was available. Next, we considered the quality of the data for our two main WHO indicators which we required for inclusion in the analysis: `New_cases` and `New_deaths` from the start of data availability through to the end time of the analysis for each country. We considered data quality from 1 November 2021 for countries of Oceania and from 1 April 2020 for the countries of all other continents.

Using this data, we excluded any countries for which no deaths or cases were reported throughout this data availability period. We also excluded any countries for which any negative values

were present within the available data. Last we excluded any countries for which multiple consecutive missing values were present in the surveillance data because it was unclear whether these should be interpreted as unavailable or as zeroes. Similarly, we excluded any countries (which affected only one country) for which repeated identical values were present (defined as changes within 1×10^{-7}). For both consecutive missing values and consecutive repeated values, we set the threshold for the number of observations for exclusion to be 15.

The final results of the selection are presented in Supplementary Table 1. The final included countries are illustrated in Supplementary Figure 1 below.

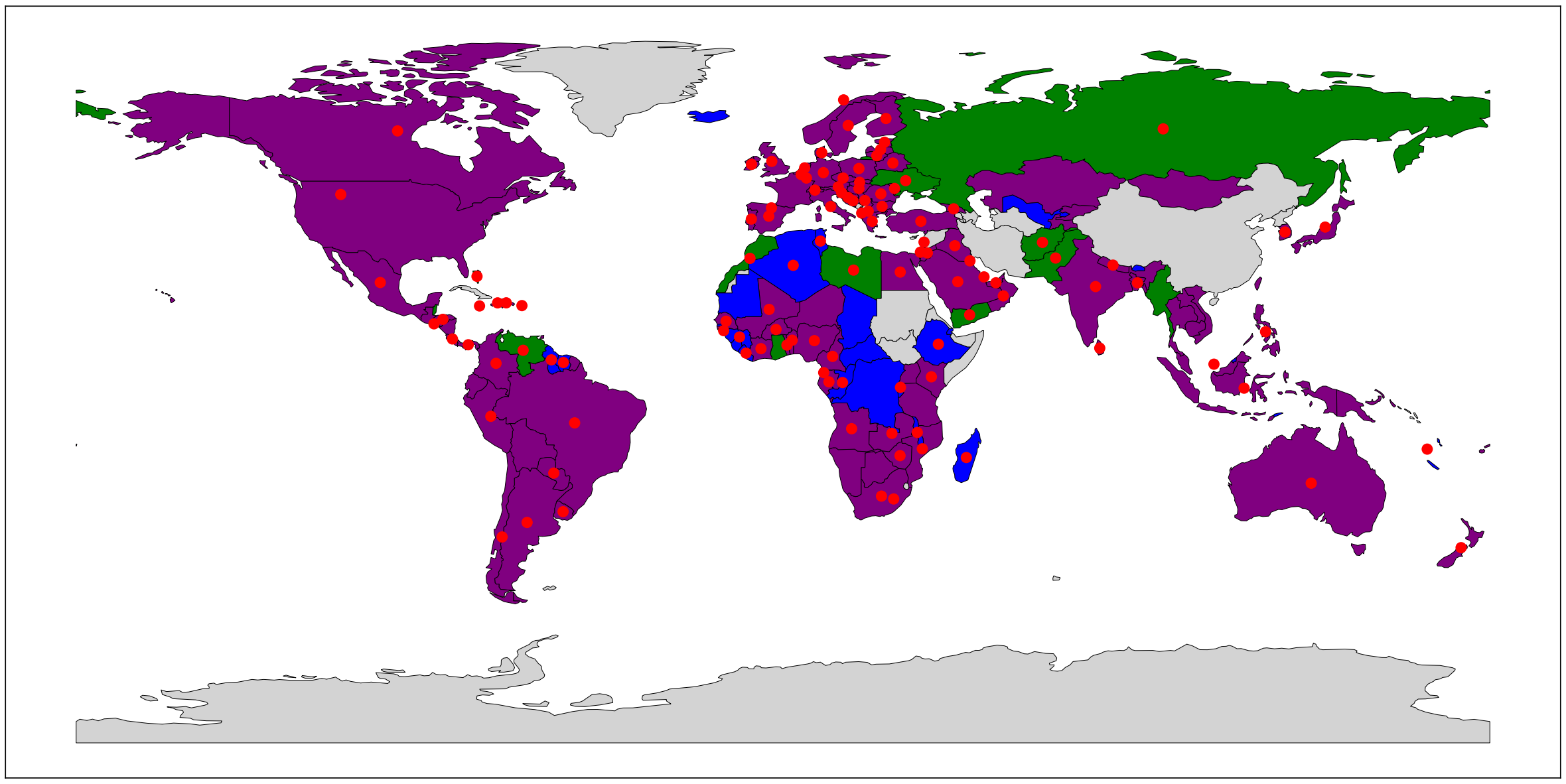

Supplementary Figure 1: Included countries and mobility availability. Countries coloured according to mobility availability. Neither source available, grey; Google mobility only available, green; Facebook mobility only available, blue; both sources available, purple. Red markers indicate included in analysis.

Supplementary Table 1: Reasons for country inclusion and exclusion.

	Google available	FB available	No death data	No case data	Negative values present	Outlier values present	Absent or repeat values	Included
Afghanistan	Yes	No	No	No	No	No	No	Yes
Albania	No	Yes	No	No	No	No	No	Yes
Algeria	No	Yes	No	No	No	No	No	Yes
American Samoa	No	Yes	Yes	No	No	Yes	No	No

Supplementary Table 1: Reasons for country inclusion and exclusion.

	Google available	FB available	No death data	No case data	Negative values present	Outlier values present	Absent or repeat values	Included
Angola	Yes	Yes	No	No	No	No	No	Yes
Antigua and Barbuda	Yes	No	No	No	No	No	Yes	No
Argentina	Yes	Yes	No	No	No	No	No	Yes
Aruba	Yes	No	No	No	No	No	No	Yes
Australia	Yes	Yes	No	No	No	No	No	Yes
Austria	Yes	Yes	No	No	No	No	No	Yes
Bahamas	Yes	No	No	No	No	No	No	Yes
Bahrain	Yes	No	No	No	No	No	No	Yes
Bangladesh	Yes	Yes	No	No	No	No	No	Yes
Barbados	Yes	No	No	No	No	No	Yes	No
Belarus	Yes	Yes	No	No	No	No	No	Yes
Belgium	Yes	Yes	No	No	No	No	No	Yes
Belize	Yes	No	No	No	No	No	Yes	No
Benin	Yes	Yes	No	No	No	No	No	Yes
Bhutan	No	Yes	No	No	No	No	Yes	No
Bolivia, Plurinational State of	Yes	Yes	No	No	No	Yes	No	No
Bosnia and Herzegovina	Yes	Yes	No	No	No	No	No	Yes
Botswana	Yes	Yes	No	No	No	No	Yes	No
Brazil	Yes	Yes	No	No	No	No	No	Yes
Brunei Darussalam	No	Yes	No	No	No	No	Yes	No
Bulgaria	Yes	Yes	No	No	No	No	No	Yes
Burkina Faso	Yes	Yes	No	No	No	No	No	Yes
Burundi	No	Yes	No	No	No	No	Yes	No
Cabo Verde	Yes	No	No	No	No	No	No	Yes
Cambodia	Yes	Yes	No	No	No	Yes	Yes	No
Cameroon	Yes	Yes	No	No	No	No	No	Yes
Canada	Yes	Yes	No	No	No	No	No	Yes
Central African Republic	No	Yes	No	No	No	No	Yes	No
Chad	No	Yes	No	No	No	No	Yes	No
Chile	Yes	Yes	No	No	No	No	No	Yes

Supplementary Table 1: Reasons for country inclusion and exclusion.

	Google available	FB available	No death data	No case data	Negative values present	Outlier values present	Absent or repeat values	Included
Colombia	Yes	Yes	No	No	No	No	No	Yes
Congo	No	Yes	No	No	No	No	No	Yes
Congo, The Democratic Republic of the	No	Yes	No	No	No	Yes	No	No
Costa Rica	Yes	Yes	No	No	No	No	No	Yes
Croatia	Yes	Yes	No	No	No	No	No	Yes
Czechia	Yes	Yes	No	No	No	No	No	Yes
Côte d'Ivoire	Yes	Yes	No	No	No	No	No	Yes
Denmark	Yes	Yes	No	No	No	No	No	Yes
Djibouti	No	Yes	No	No	No	No	Yes	No
Dominica	No	Yes	Yes	No	No	No	Yes	No
Dominican Republic	Yes	Yes	No	No	No	No	No	Yes
Ecuador	Yes	Yes	No	No	Yes	Yes	No	No
Egypt	Yes	Yes	No	No	No	No	No	Yes
El Salvador	Yes	Yes	No	No	No	No	No	Yes
Equatorial Guinea	No	Yes	No	No	No	No	No	Yes
Estonia	Yes	Yes	No	No	No	No	No	Yes
Ethiopia	No	Yes	No	No	No	No	No	Yes
Faroe Islands	No	Yes	No	No	No	No	Yes	No
Fiji	Yes	Yes	No	No	No	No	No	Yes
Finland	Yes	Yes	No	No	No	No	No	Yes
France	Yes	Yes	No	No	No	No	No	Yes
French Guiana	No	Yes	No	No	No	No	No	Yes
Gabon	Yes	Yes	No	No	No	No	No	Yes
Gambia	No	Yes	No	No	No	Yes	Yes	No
Georgia	Yes	Yes	No	No	No	No	No	Yes
Germany	Yes	Yes	No	No	No	No	No	Yes
Ghana	Yes	No	No	No	No	Yes	Yes	No
Greece	Yes	Yes	No	No	No	No	No	Yes
Guadeloupe	No	Yes	No	No	Yes	No	No	No
Guatemala	Yes	Yes	No	No	Yes	No	No	No
Guinea	No	Yes	No	No	No	No	No	Yes

Supplementary Table 1: Reasons for country inclusion and exclusion.

	Google available	FB available	No death data	No case data	Negative values present	Outlier values present	Absent or repeat values	Included
Guinea-Bissau	Yes	Yes	No	No	No	No	No	Yes
Guyana	No	Yes	No	No	No	No	No	Yes
Haiti	Yes	Yes	No	No	No	No	No	Yes
Honduras	Yes	Yes	No	No	No	No	No	Yes
Hong Kong	Yes	No	Yes	Yes	No	No	No	No
Hungary	Yes	Yes	No	No	No	No	No	Yes
Iceland	No	Yes	No	No	No	No	Yes	No
India	Yes	Yes	No	No	No	No	No	Yes
Indonesia	Yes	Yes	No	No	No	No	No	Yes
Iraq	Yes	Yes	No	No	No	No	No	Yes
Ireland	Yes	Yes	No	No	No	No	No	Yes
Israel	Yes	Yes	No	No	No	No	No	Yes
Italy	Yes	Yes	No	No	No	No	No	Yes
Jamaica	Yes	Yes	No	No	No	No	No	Yes
Japan	Yes	Yes	No	No	No	No	No	Yes
Jordan	Yes	Yes	No	No	No	No	No	Yes
Kazakhstan	Yes	Yes	No	No	No	Yes	No	No
Kenya	Yes	Yes	No	No	No	No	No	Yes
Korea, Republic of	Yes	Yes	No	No	No	No	No	Yes
Kuwait	Yes	No	No	No	No	No	No	Yes
Kyrgyzstan	Yes	Yes	No	No	No	No	Yes	No
Lao People's Democratic Republic	Yes	Yes	No	No	No	No	Yes	No
Latvia	Yes	Yes	No	No	No	No	No	Yes
Lebanon	Yes	Yes	No	No	No	No	No	Yes
Lesotho	No	Yes	No	No	No	No	No	Yes
Liberia	No	Yes	No	No	No	No	No	Yes
Libya	Yes	No	No	No	No	No	No	Yes
Liechtenstein	Yes	No	No	No	No	No	Yes	No
Lithuania	Yes	Yes	No	No	No	No	No	Yes
Luxembourg	Yes	Yes	No	No	No	No	No	Yes
Madagascar	No	Yes	No	No	No	No	No	Yes
Malawi	No	Yes	No	No	No	No	No	Yes
Malaysia	Yes	Yes	No	No	No	No	No	Yes

Supplementary Table 1: Reasons for country inclusion and exclusion.

	Google available	FB available	No death data	No case data	Negative values present	Outlier values present	Absent or repeat values	Included
Mali	Yes	Yes	No	No	No	No	No	Yes
Malta	Yes	Yes	No	No	No	No	No	Yes
Martinique	No	Yes	No	No	No	No	No	Yes
Mauritania	No	Yes	No	No	No	Yes	No	No
Mauritius	Yes	Yes	No	No	No	No	Yes	No
Mexico	Yes	Yes	No	No	No	No	No	Yes
Moldova, Republic of	Yes	Yes	No	No	No	No	No	Yes
Mongolia	Yes	Yes	No	No	No	No	Yes	No
Morocco	Yes	No	No	No	No	No	No	Yes
Mozambique	Yes	Yes	No	No	No	No	No	Yes
Myanmar	Yes	No	No	No	No	No	Yes	No
Namibia	Yes	Yes	Yes	Yes	No	No	No	No
Nepal	Yes	Yes	No	No	No	No	No	Yes
Netherlands	Yes	Yes	No	No	No	No	No	Yes
New Caledonia	No	Yes	No	No	No	Yes	No	No
New Zealand	Yes	Yes	No	No	No	No	No	Yes
Nicaragua	Yes	Yes	No	No	No	No	Yes	No
Niger	Yes	Yes	No	No	No	Yes	Yes	No
Nigeria	Yes	Yes	No	No	No	No	No	Yes
North Macedonia	Yes	Yes	No	No	No	No	No	Yes
Norway	Yes	Yes	No	No	No	No	No	Yes
Oman	Yes	Yes	No	No	No	No	No	Yes
Pakistan	Yes	No	No	No	No	No	No	Yes
Panama	Yes	Yes	No	No	No	No	No	Yes
Papua New Guinea	Yes	Yes	No	No	No	Yes	No	No
Paraguay	Yes	Yes	No	No	No	No	No	Yes
Peru	Yes	Yes	No	No	No	No	No	Yes
Philippines	Yes	Yes	No	No	No	No	No	Yes
Poland	Yes	Yes	No	No	No	No	No	Yes
Portugal	Yes	Yes	No	No	No	No	No	Yes
Puerto Rico	Yes	No	No	No	No	No	No	Yes
Qatar	Yes	Yes	No	No	No	No	No	Yes
Romania	Yes	Yes	No	No	No	No	No	Yes

Supplementary Table 1: Reasons for country inclusion and exclusion.

	Google available	FB available	No death data	No case data	Negative values present	Outlier values present	Absent or repeat values	Included
Russian Federation	Yes	No	No	No	No	No	No	Yes
Rwanda	Yes	Yes	No	No	No	No	No	Yes
Réunion	Yes	Yes	No	No	Yes	No	No	No
Samoa	No	Yes	Yes	No	No	No	No	No
Sao Tome and Principe	No	Yes	No	No	No	No	No	Yes
Saudi Arabia	Yes	Yes	No	No	No	No	No	Yes
Senegal	Yes	Yes	No	No	No	No	No	Yes
Serbia	Yes	Yes	No	No	No	No	No	Yes
Sierra Leone	No	Yes	No	No	No	No	Yes	No
Singapore	Yes	No	No	No	No	No	No	Yes
Slovakia	Yes	Yes	No	No	No	No	No	Yes
Slovenia	Yes	Yes	No	No	No	No	No	Yes
South Africa	Yes	Yes	No	No	No	No	No	Yes
Spain	Yes	Yes	No	No	No	No	No	Yes
Sri Lanka	Yes	Yes	No	No	No	No	No	Yes
Suriname	No	Yes	No	No	No	No	No	Yes
Sweden	Yes	Yes	No	No	No	No	No	Yes
Switzerland	Yes	Yes	No	No	No	No	No	Yes
Taiwan, Province of China	Yes	Yes	Yes	Yes	No	No	No	No
Tajikistan	Yes	Yes	No	No	No	No	Yes	No
Tanzania, United Republic of	Yes	Yes	No	No	No	Yes	Yes	No
Thailand	Yes	Yes	No	No	Yes	No	No	No
Timor-Leste	No	Yes	No	No	No	No	Yes	No
Togo	Yes	Yes	No	No	No	No	No	Yes
Trinidad and Tobago	Yes	Yes	No	No	No	No	Yes	No
Tunisia	No	Yes	No	No	No	No	No	Yes
Türkiye	Yes	Yes	No	No	No	No	No	Yes
Uganda	Yes	Yes	No	No	No	Yes	Yes	No
Ukraine	Yes	No	No	No	No	No	No	Yes

Supplementary Table 1: Reasons for country inclusion and exclusion.

	Google available	FB available	No death data	No case data	Negative values present	Outlier values present	Absent or repeat values	Included
United Arab Emirates	Yes	Yes	No	No	No	No	No	Yes
United Kingdom	Yes	Yes	No	No	No	No	No	Yes
United States	Yes	Yes	No	No	No	No	No	Yes
Uruguay	Yes	Yes	No	No	No	No	No	Yes
Uzbekistan	No	Yes	No	No	No	No	Yes	No
Vanuatu	No	Yes	Yes	Yes	No	No	No	No
Venezuela, Bolivarian Republic of	Yes	No	No	No	No	No	No	Yes
Viet Nam	Yes	Yes	No	No	No	No	Yes	No
Virgin Islands, U.S.	No	Yes	No	No	Yes	No	No	No
Yemen	Yes	No	No	No	No	No	No	Yes
Zambia	Yes	Yes	No	No	No	No	No	Yes
Zimbabwe	Yes	Yes	No	No	No	No	No	Yes

Parameter choices and supporting evidence

Time period parameters

Supplementary Table 2: Parameters and supporting evidence to time period priors.

	Mean	SD	Evidence
Generation time mean (days)	4.25	0.75	We used analyses of case-contact transmission pairs from multiple contexts for SARS-CoV-2 as the main evidence to quantify the generation time distribution.<br><br>Case-contact data from the origin of the pandemic in Wuhan estimated a mean generation time of around seven days prior to the scaling up of population-wide public health and social measures, such as expanded contact tracing and movement restrictions. This led to a notable contraction of the mean generation time to around four to five days after these interventions had been implemented.[2] This study also found marked differences between the generation time and serial interval, due to changes in the epidemic that were sufficiently rapid to result in differences in the incubation period between infectors and infectees. Values of around four to five days are also consistent with an analysis from early 2020 from Tianjin, China and Singapore, in which estimates of the serial interval estimates were close to that of the generation time[3].<br><br>Estimates of the serial interval from household clusters in Germany[1] also found that values of four to five days were appropriate for wild-type, Alpha and Delta SARS-CoV-2 variants, although these appeared less applicable to Omicron. Estimates from close contacts in Spain[4] similarly placed the serial interval of the Delta variant at around five days, while an early study from China similarly placed the mean serial interval for wild-type SARS-CoV-2 at around four to five days.[5]<br><br>A systematic review and meta-analysis of this topic found more studies estimating the serial interval than the generation time, with mean estimates for the serial interval of 4.82 days, 3.59 days and 3.47 days reported for wild-type, Alpha and Delta respectively.[6] Estimates for the generation time from this review were similar, but with wider confidence limits. A rapid review of serial intervals from a different research group reported similar findings.[7] There was notable variation in these findings, with results of individual studies from these systematic reviews estimating mean values for the serial interval of less than three days to more than six days.

	Mean	SD	Evidence
Generation time mean (days), Oceania and Singapore	3	0.75	An analysis from China estimated that the generation time fell from 5.2 days to 2.2 days over the course of an Omicron outbreak in Hangzhou in 2022.[8] The systematic review of generation times by variant cited above estimated a mean generation time of 2.96 days (95% confidence interval 2.54 to 3.38 days).[6] A rapid review and meta-analysis of generation times by variant found a pooled estimate of 3.3 days for Omicron.[7]<br><br>As such, we chose a generation interval mean for the Oceania and Singapore analyses of three days.
Generation time standard deviation (days)	2	0.4	For the standard deviation of the generation interval, we generally used the same sources of evidence as for the mean. However, the systematic reviews and many of the individual studies presented their results as point estimates with confidence intervals, rather than reporting the standard deviations of the data of the individual studies, making these results less applicable to this parameter.<br><br>In studies that reported standard deviations, estimates included two to four days for early Wuhan[2] and 1.5 to 2.5 days from Tianjin and Singapore.[3]

	Mean	SD	Evidence
Infection to reporting mean (days)	8	0.5	This quantity is a composite of the incubation period and the delay from symptom onset to diagnosis, although the latter of these quantities is not widely reported. The review cited above in estimating the generation time found estimates of the incubation period of 6.5, 4.92 and 4.63 days for the wild-type, Alpha and Delta variants respectively.[6] A meta-analysis of early cases reported a mean incubation period of 5.68 days[9], while an analysis of early cases in China found a mean incubation period of 5.2 days.[10] As such, although the incubation period was not an explicit model parameter, we considered five days to be a plausible central estimate when considering parameters required for the convolutions applied in our analysis, including this one.<br><br>Regarding the delay from symptom onset to case notification, an analysis from Australia found that this quantity was often in the range of one to seven days after the first wave of cases in early 2020 had declined.[11] This is consistent with a survey from the USA that reported a median of three days unwell before testing.[12] A study of case notifications in New York City found a mean delay from testing to case notification of 3.3 days.[13] WHO reported that the median time from symptom onset to laboratory confirmation had decreased to three days by early February 2020.[14] Our previous analysis of the Australian epidemic found that our prior estimate of three to five days was not markedly influenced through calibration.[15]<br><br>Studies of the time from exposure to diagnosis include a study from South Korea which found an average of 3.7 days,[16] although this very short interval likely reflects proactive testing for these quarantined close contacts. An analysis from the USA estimated that infections could peak anywhere from six to 41 days before peaks in case notification.[17]<br><br>Overall, we considered that a mean time from infection to case notification of eight days would be consistent with a five-day incubation period followed by a three-day average delay from symptom onset to case notification.

	Mean	SD	Evidence
Infection to reporting standard deviation (days)	3	0.5	Although person-to-person variation in the incubation period was not typically quantified in systematic reviews,[6] individual studies reported standard deviations of around three days[10].<br><br>The survey from the USA reported an interquartile range for the time to testing from feeling unwell that spanned five days.[12] Most delays in the study from New York fell in the range from one to five days,[13] while the WHO report cited above described an interquartile range that spanned six days.[14]<br><br>Given this background, we considered that a standard deviation for the time from infection to case notification of three days was plausible, and set the standard deviation for the prior of this parameter distribution to 0.5 days.
Infection to death mean (days)	22	5	The meta-analysis of early studies from China cited above[9] estimated the mean duration from symptom onset to death to be 15.93 days. A study leveraging individual-level data from Hubei, China in combination with early cases outside of China (not included in the meta-analysis) found a mean time from symptom onset to death of 17.8 days.[18] This was similar to a report from a single centre study from Wuhan (Hubei, China) during a time of major health system strain, which found a median time to death of 15 days,[19] and another single centre study of early cases from Wuhan which reported a median time to death of 16 days.[20] A cohort study from a north-eastern state of Brazil found a median 19 days from symptom onset to death.[21] An analysis of deaths from Victoria, Australia over the course of 2020 found a mean time to death of 18.1 days.[22]<br><br>We therefore considered 17 days to be a reasonable estimate for the time from symptom onset to death, which we increased by five days to account for the time from infection to symptom onset.

	Mean	SD	Evidence
Infection to death standard deviation (days)	3	0.5	The first single centre study from Wuhan cited above reported an interquartile range which spanned nine days,[19] while the second reported a span of eight days.[20] The interquartile range from the study in north-eastern Brazil spanned 11 days.[21]<br><br>We considered that the standard deviation for the infection to death prior would be broadly of the order of one quarter the ranges reported by these individual studies, although inflated slightly to account for the uncertainty in the incubation period. We therefore set this parameter to three days, with a standard deviation to the distribution of 0.5 days.
Infection to hospitalisation mean (days)	10	3	One of the earliest studies of cases from China found a median time from onset of symptoms to hospital admission of seven days,[23] while a single centre study from Wuhan reported a median time from symptom onset to hospital admission of ten days.[20] A cohort of adult patients admitted to an Italian hospital found a mean time from onset of symptoms to hospital admission of 3.6 days,[24] while a study from Belgium reported the mean duration from symptom onset to hospitalisation to be 5.6 days.[25] The final report of the ISARIC collaboration reported a mean of 5.4 days from symptom onset to admission.[26]<br><br>Given this evidence, we chose a mean value for the time from symptom onset to hospitalisation prior of five days. As for the time from infection to death parameter, we inflated this by five days to allow for the incubation period.
Infection to hospitalisation standard deviation (days)	2	0.5	The early study of cases from China reported an interquartile range that spanned four days,[23] while the single centre study from Wuhan reported an interquartile range spanning six days.[20] The Italian cohort of hospital admissions reported the standard deviation of the time from symptom onset to hospitalisation to be 3.2 days.[24] The study from Belgium cited above found considerable variation in the time to admission, with the interquartile range spanning six days.[25]<br><br>As with previous parameters, we selected a prior standard deviation of slightly more than one quarter of the spread in the data in the constituent studies. We therefore chose a central estimate for this standard deviation parameter of two days, with a standard deviation to this distribution of 0.5 days.

	Mean	SD	Evidence
Hospital stay duration mean (days)	10	3	An analysis of surveillance data from Belgium reported a mean length of hospital stay of 11.0 days,[25] while a multi-centre study from the USA reported a mean length of hospital stay of 14.7 days.[27] The single centre study from Wuhan cited above reported a median time from hospital admission to discharge of 16 days, with a median time from admission to death of five days.[20]<br><br>A systematic review of hospital admission durations found a median length of stay of 14 days in China, but only five days outside of China.[28], while a scoping review of infectious durations reported that the mean time from symptom onset to hospital discharge or death was 18.1 days.[29] Another systematic review undertaken later in the pandemic period found similar durations, with a mean length of stay of 15.35 days.[30]<br><br>The final report of the ISARIC collaboration reported a mean 9.7 days (median seven days) length of hospital stay.[26]
Hospital stay duration standard deviation (days)	2.5	0.5	The analysis from Belgium cited above reported an interquartile range that spanned ten days,[25] while the multi-centre study from the USA reported a standard deviation of 13.7 days.[27]<br><br>The interquartile range reported in the first systematic review above[28] was ten to 19 days, while the individual-level standard deviation identified in the later systematic review above[30] was 7.92 days.<br><br>As such, we implemented a standard deviation for hospital admission duration of 2.5 days, with a standard deviation to this parameter prior of 0.5 days.

	Mean	SD	Evidence
Infection to ICU admission mean (days)	12	3	A single centre study from Wuhan found a median duration from symptom onset to ICU admission of 9.5 days,[31] while an early study of cases from China found a median time from onset of symptoms to ICU admission of 10.5 days.[23] A study from two hospitals in China reported a median time from illness onset to ICU admission of 12.0 days.[32] The study from north-eastern Brazil reported a median six days from symptom onset to ICU admission.[21]<br><br>The final report from the ISARIC collaboration estimated a mean time from symptom onset to hospital admission of 5.4 days and a mean time from hospital admission to ICU admission of 1.8 days.[26]<br><br>Given this evidence, we implemented a mean for our prior distribution of time from infection to ICU admission of twelve days to allow for a five day incubation period.
Infection to ICU admission standard deviation (days)	2.5	0.5	The single centre study from Wuhan cited above reported an interquartile range around the time from admission to discharge that spanned five days, while the time to death spanned six days.[20] The study of early cases from China reported an interquartile range that spanned nine days,[23] while the study from two hospitals in China reported an interquartile range that spanned 7.0 days.[32] The second single centre study from Wuhan reported an interquartile range from the onset of symptoms to ICU admission that spanned 5.5 days.[31] The analysis from Belgium cited above reported an interquartile range for ICU admission that spanned 13 days.[25] The multi-centre study from the USA reported a standard deviation for the ICU admission duration of 8.3 days.[27] The study in north-eastern Brazil reported an interquartile range around the time to ICU admission that spanned 6.5 days.[21]<br><br>We therefore implemented a standard deviation for our time from infection to ICU admission parameter of 2.5 days, with a standard deviation to this distribution of 0.5 days.

	Mean	SD	Evidence
ICU stay duration mean (days)	10	3	The analysis from Belgium cited above reported a mean length of ICU stay of 12.2 days,[25] while the multi-centre study from the USA cited above reported a mean ICU admission duration of 8.3 days.[27] The study from multiple regions of Brazil indicated the median length of ICU stay was five days.[33]<br><br>The systematic review introduced above[28] reported a median ICU length of stay outside of China of seven days. The final report from ISARIC gave a mean duration of stay in ICU of 10.3 days.[26]<br><br>We therefore chose a mean for the prior distribution of the duration of ICU stay of ten days.
ICU stay duration standard deviation (days)	2.5	0.5	The study from multiple regions of Brazil reported an interquartile range that spanned eight days,[33] while the final ISARIC report provided a standard deviation for ICU duration of 10.4 days.[26]<br><br>We therefore chose a standard deviation of 2.5 days for this prior distribution and assigned a standard deviation for the spread of this parameter of 0.5 days.
Duration immune	180	30	For our sensitivity analysis considering waning of host-related natural immunity, we assumed a mean duration of immunity of around 180 days, consistent with empirical studies showing substantial decline in protection against reinfection over 3 to 9 months.

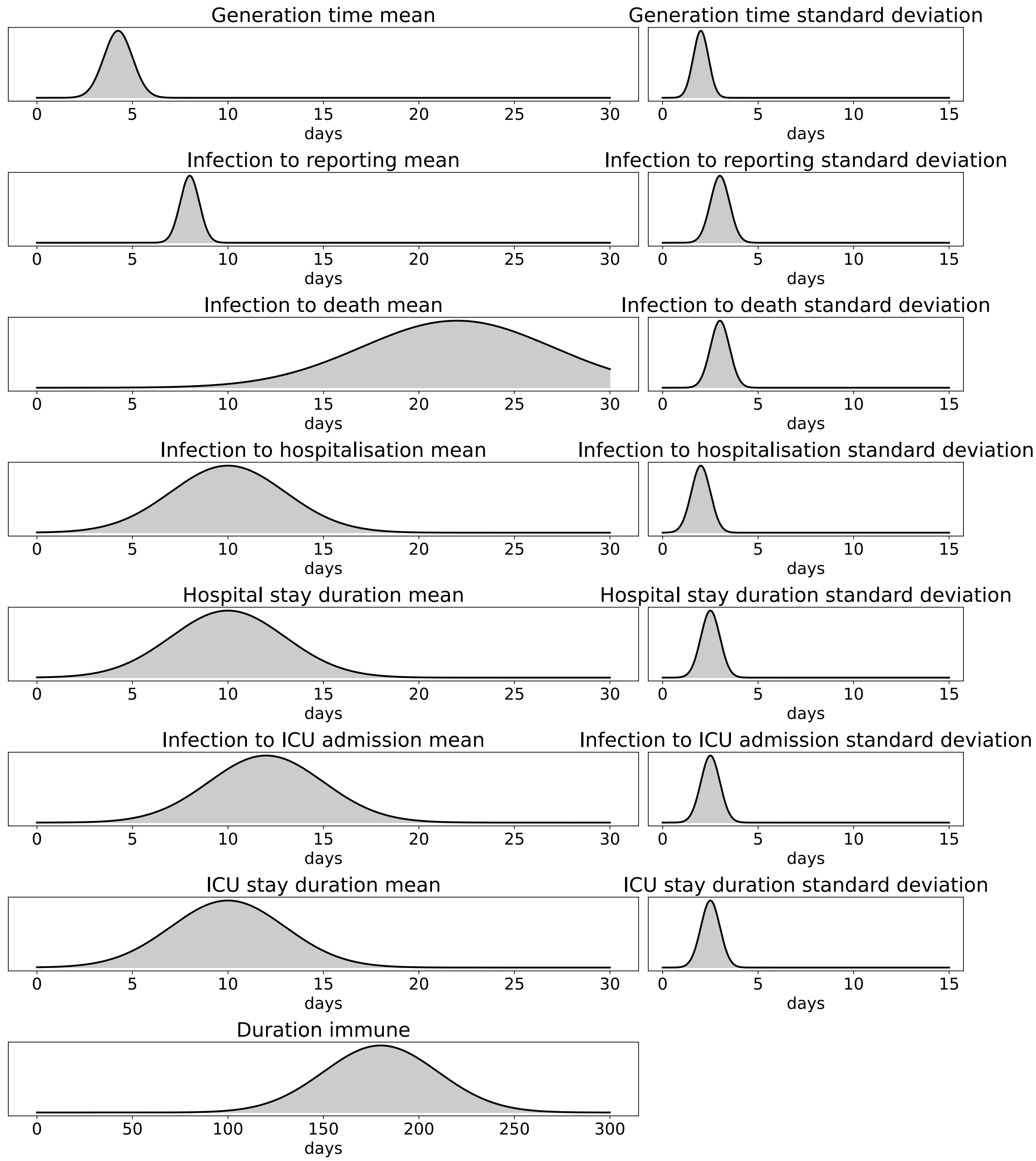

Supplementary Figure 2: Duration-related parameter prior distributions.

Proportion parameters

Supplementary Table 3: Parameters and supporting evidence to beta-distributed priors.

	Mean	SD	Evidence
Case detection proportion	0.4	0.1	An analysis from France in early to mid-2020 of the proportion of cases detected estimated that case ascertainment increased from 7% to 38% (95% confidence interval 35 to 44%) over time.[34] A study of multiple countries found that case detection was highly dependent on the country considered, but reached 20 to 60% in several European countries by the middle of 2020.[35] Estimates from the UK Health Security Agency placed the proportion of cases detected over time at around 30 to 60% from the latter half of 2020.[36] These estimates are consistent with our previous estimate that one in two to one in six cases were detected from the early stages of Australia's Omicron outbreak.[15] An analysis from Africa estimated generally lower case ascertainment proportions, which also varied significantly by country.[37]<br><br>Given the difficulty in determining an appropriate prior for this parameter and the likelihood that the quantity is highly setting-dependent, we selected a broad prior distribution for this parameter.
Cross immunity	0.5	0.1	A pre-print describing a large dataset from the USA in 2020 found an 87% lower rate of reinfection compared to primary infection over a median five months of follow-up.[38]<br><br>A systematic review and meta-analysis found that protection against re-infection declined with time and varied with the variant considered, but was estimated at 78.6% after 40 weeks from infection (95% uncertainty interval 49.8 to 93.6%).[39] Another systematic review and meta-analysis of the same topic found 65% protection (credible interval 53 to 74%) overall, with the past infecting variant and time from infection appearing to modify the extent of protection.[40] A systematic review and meta-regression of reinfections with Omicron variants found that protection from previous infections waned to lower levels (25%) by 12 months from infection.[41]

	Mean	SD	Evidence
Infection hospitalisation proportion	0.03	0.006	This quantity was estimated to be 3.39% (credible interval 2.79 to 3.97%) from officially reported data in England prior to the marked declines that occurred as population-wide vaccination was rolled out.[42] An analysis from Indiana, USA estimated a value of 2.1% overall (credible interval 1.6 to 3.0%), with substantial variation by demographic factors, particularly age.[43] A study from the USA estimated that one in three adults aged 70 years and above who were infected were hospitalised, while one in 21.6 infections in those aged 18 to 49 years resulted in hospitalisation.[44]
Infection fatality proportion	0.008	0.001	An analysis that integrated serosurveillance with age-specific death rates from 45 countries estimated an infection fatality rate of 0.79% (credible interval 0.68 to 0.92%) for the reference population distribution of France.[45] Other groups have found similar estimates using a combination of modelling and serosurvey results, including an overall IFR estimated at 0.49 to 2.53%.[46] The analysis from England of the hospitalisation risk estimated a peak infection fatality risk of 0.97% in January 2021, which fell to 0.06% by April 2022.[42]<br><br>We selected a prior distribution with a central estimate of 0.8%. Our prior estimate is relatively broadly distributed and so should be applicable to a range of higher income settings.
ICU admission proportion	0.01	0.001	The analysis from Indiana cited above found an infection ICU admission risk of 0.51% (credible interval 0.38% to 0.70%).[43] An analysis from Chile found a risk of ICU admission given infection of 0.33, 1.18, 3.59 and 7.18% for the age groups under 40, 40 to 49, 50 to 59 and 60 to 69 respectively.[47] A systematic review and meta-analysis from early in the pandemic period found that 32% of hospital admissions resulted in ICU admission.[48]<br><br>We therefore chose a central estimate for this parameter of 1%.

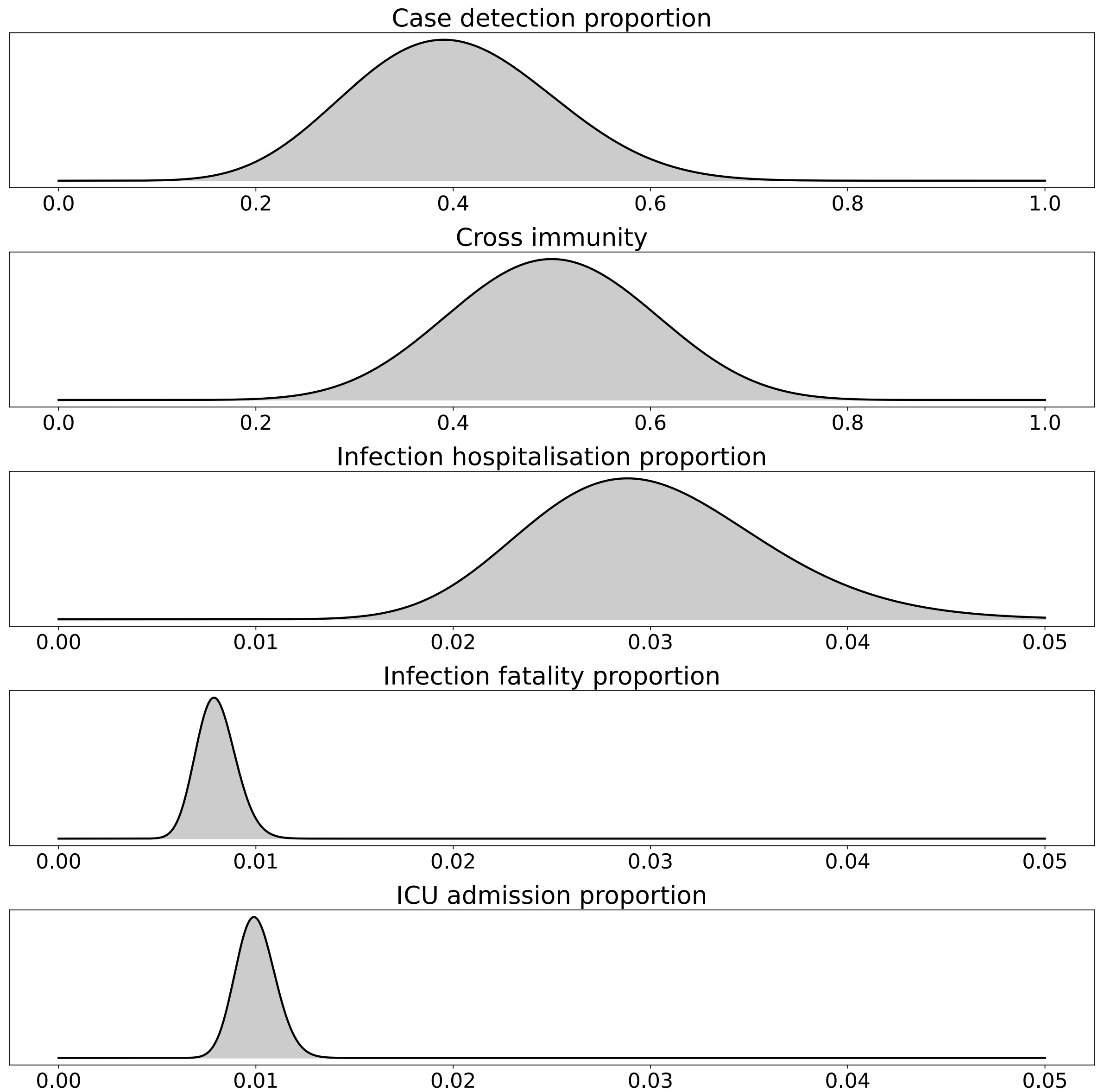

Supplementary Figure 3: Proportion parameter prior distributions. Note horizontal axis range differs between upper two panels and lower three panels.

Fixed parameters

Supplementary Table 4: Fixed parameters and supporting evidence.

	Value	Evidence
Vaccination protection against hospitalisation	0.5	The first estimate of the efficacy of BNT162b2 mRNA vaccine that was widely used in Australia, found a total efficacy of 95% protection against COVID-19.[49]<br><br>However, protection against infection will be captured by scaling the modelled transmission parameters, including the residual transmission. As such, the evidence needed for this quantity is the real-world reduction in the risk of hospitalisation and death given infection.<br><br>Estimates of the real-world protection of vaccination against SARS-CoV-2 infections found 85% protection (95% confidence interval 81 to 89%) against infection, but higher protection against severe outcomes, including hospitalisation (93%), ICU admission (98%) and death (95%).[50]<br><br>As such, we estimated the reduction in hospitalisation given infection to be 50%.
Vaccination protection against death	0.67	Using the same evidence as for the protection against hospitalisation, we estimated this parameter at 67%.

Between-variant relative parameters

Estimates of the relative severity of Alpha compared to preceding strains include a large community-based study from England, which reported a 1.73-fold adjusted hazard of death and a 1.62-fold hazard of hospital admission for Alpha versus wild-type[51]. In Scotland, a genomics-linked cohort analysis incorporating graded clinical severity found a 1.40-fold increase in overall disease severity associated with Alpha[52].

For Delta compared with Alpha, a cohort study from the UK estimated an adjusted hazard ratio of 2.26 for hospital admission and 1.45 for the composite outcome of hospital admission or emergency care[53], with similar estimates observed among unvaccinated individuals. However, an independent national analysis from Norway did not identify an increased risk of hospitalisation for Delta relative to Alpha[54].

Calibration results

Results of individual model calibrations to epidemiological targets (with and without waning immunity included) were too large for inclusion in this document and so are presented on Figshare here. This section presents the comparisons of prior and posterior distributions for each individual country analysis.

Country grouping

Throughout our extended results, we group countries according to the following continent groupings.

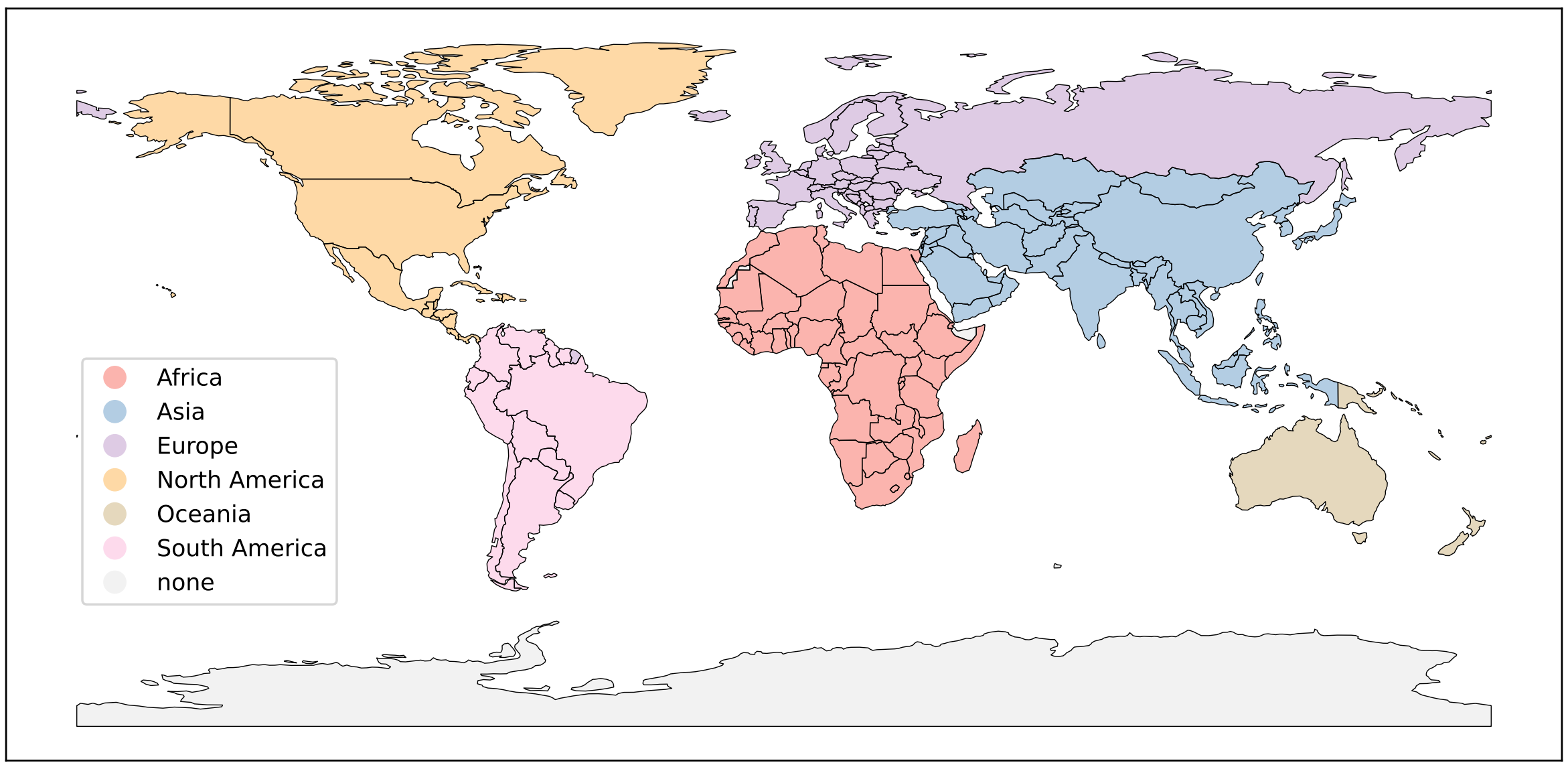

Prior posterior comparisons by continent and country, base case

This section presents comparisons of parameter prior distributions to their corresponding posterior distributions.

Africa

Algeria

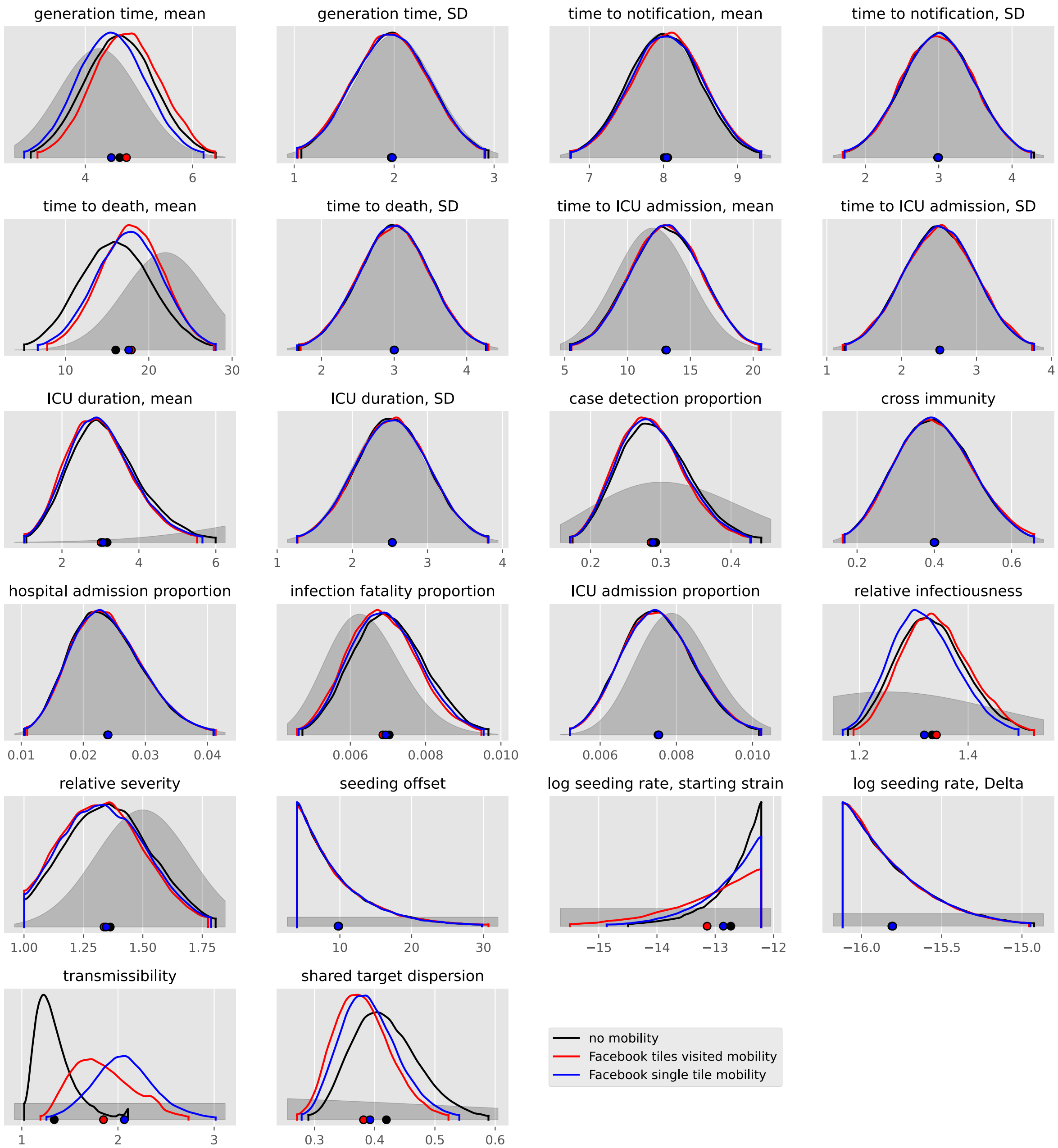

Angola

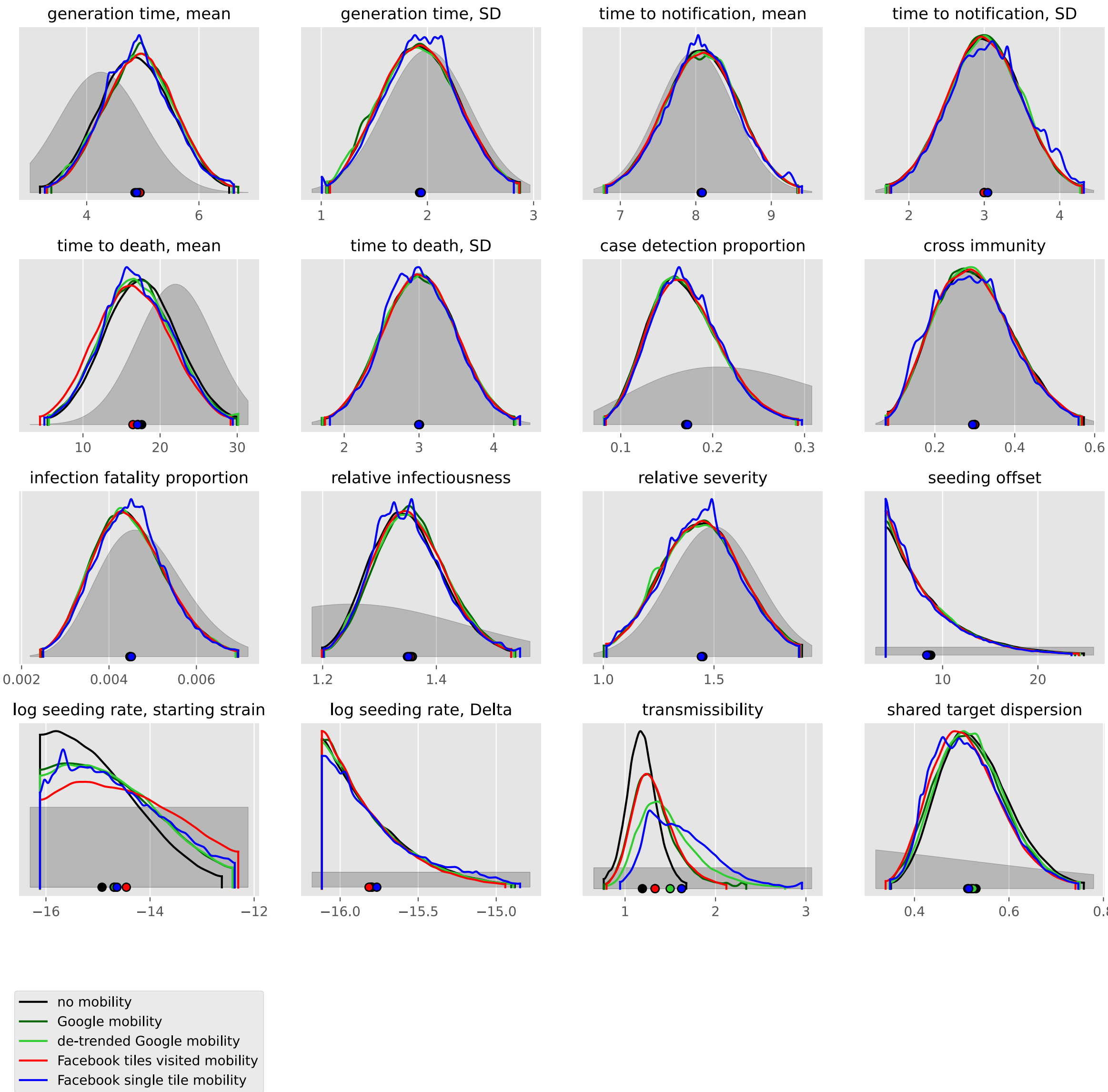
generation time, mean
generation time, SD
time to notification, mean
time to notification, SD
time to death, mean
time to death, SD
case detection proportion
cross immunity
infection fatality proportion
relative infectiousness
relative severity
seeding offset
log seeding rate, starting strain
log seeding rate, Delta
transmissibility
shared target dispersion
no mobility
Google mobility
de-trended Google mobility
Facebook tiles visited mobility
Facebook single tile mobility

Benin

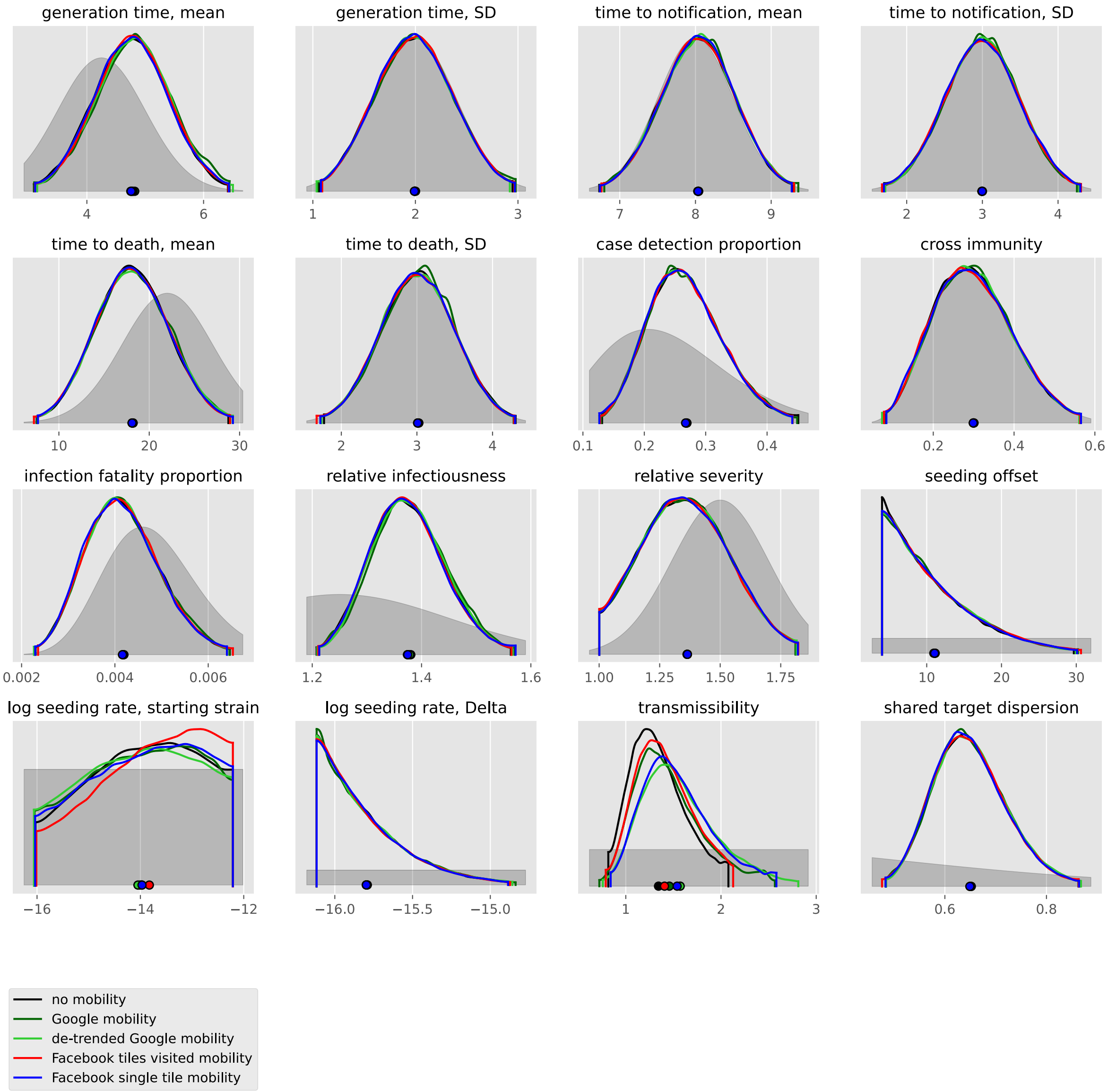
generation time, mean
generation time, SD
time to notification, mean
time to notification, SD
time to death, mean
time to death, SD
case detection proportion
cross immunity
infection fatality proportion
relative infectiousness
relative severity
seeding offset
log seeding rate, starting strain
log seeding rate, Delta
transmissibility
shared target dispersion
no mobility
Google mobility
de-trended Google mobility
Facebook tiles visited mobility
Facebook single tile mobility

Burkina Faso

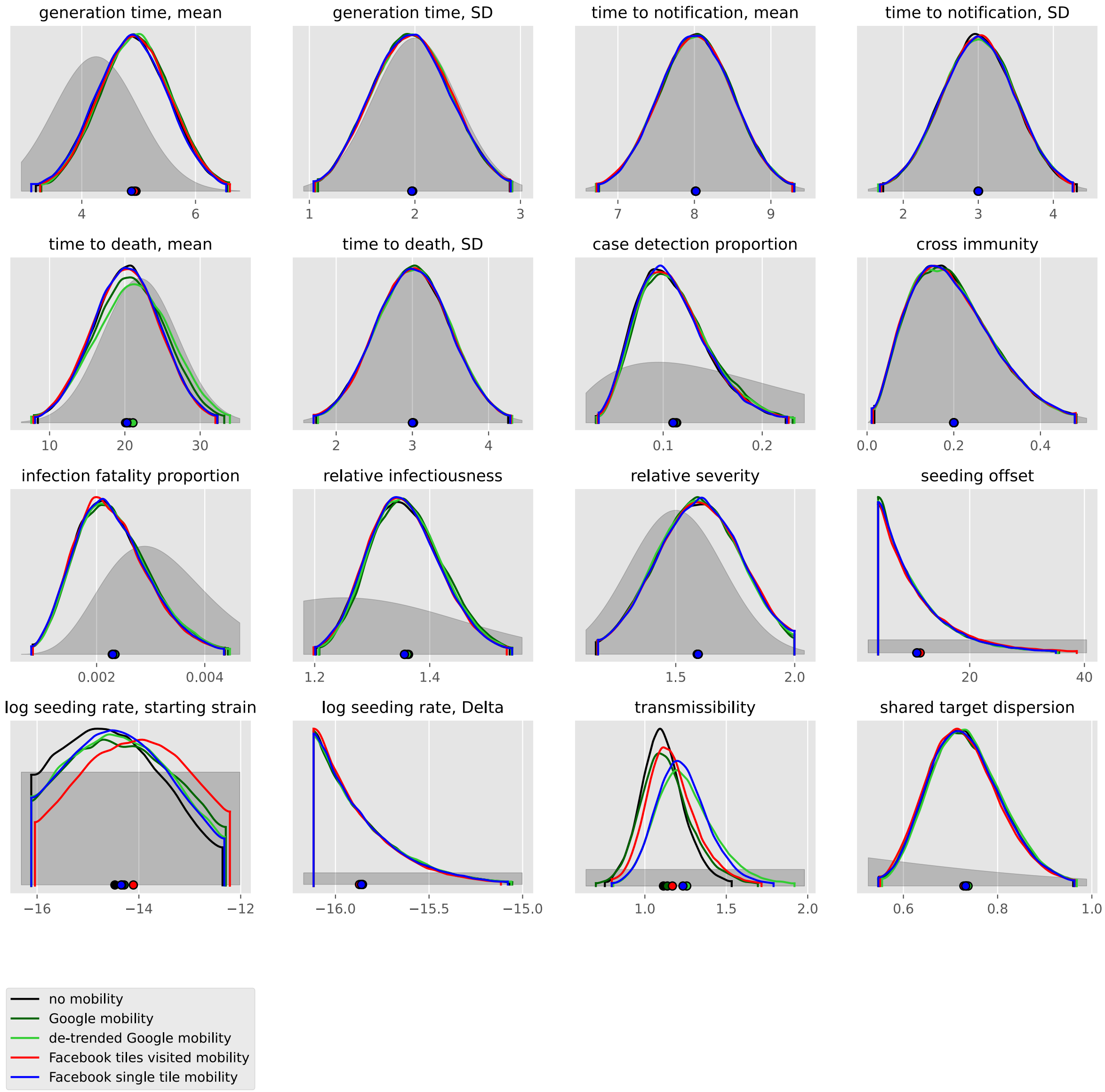

Cabo Verde

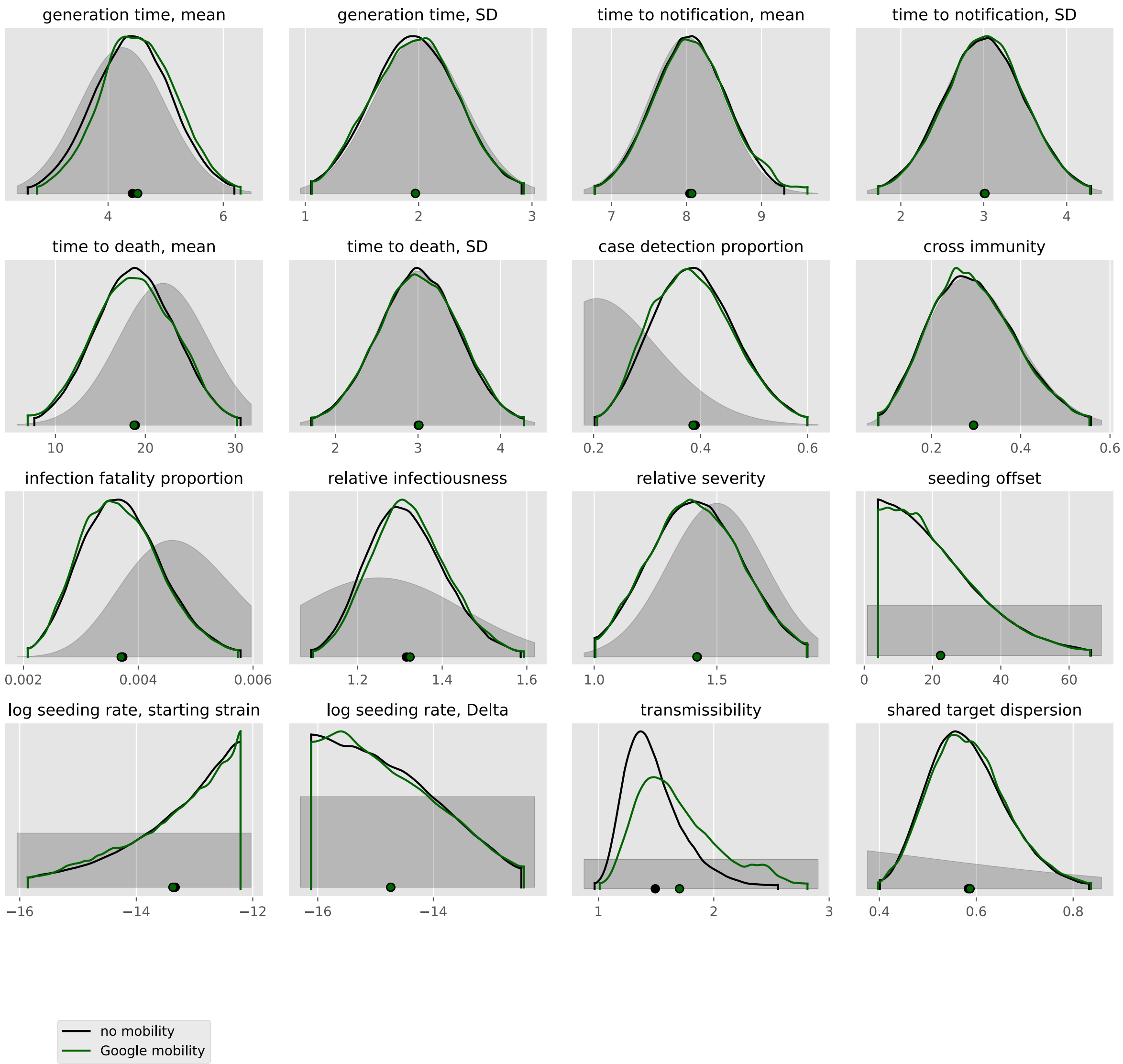
generation time, mean
generation time, SD
time to notification, mean
time to notification, SD
time to death, mean
time to death, SD
case detection proportion
cross immunity
infection fatality proportion
relative infectiousness
relative severity
seeding offset
log seeding rate, starting strain
log seeding rate, Delta
transmissibility
shared target dispersion
no mobility
Google mobility

Cameroon

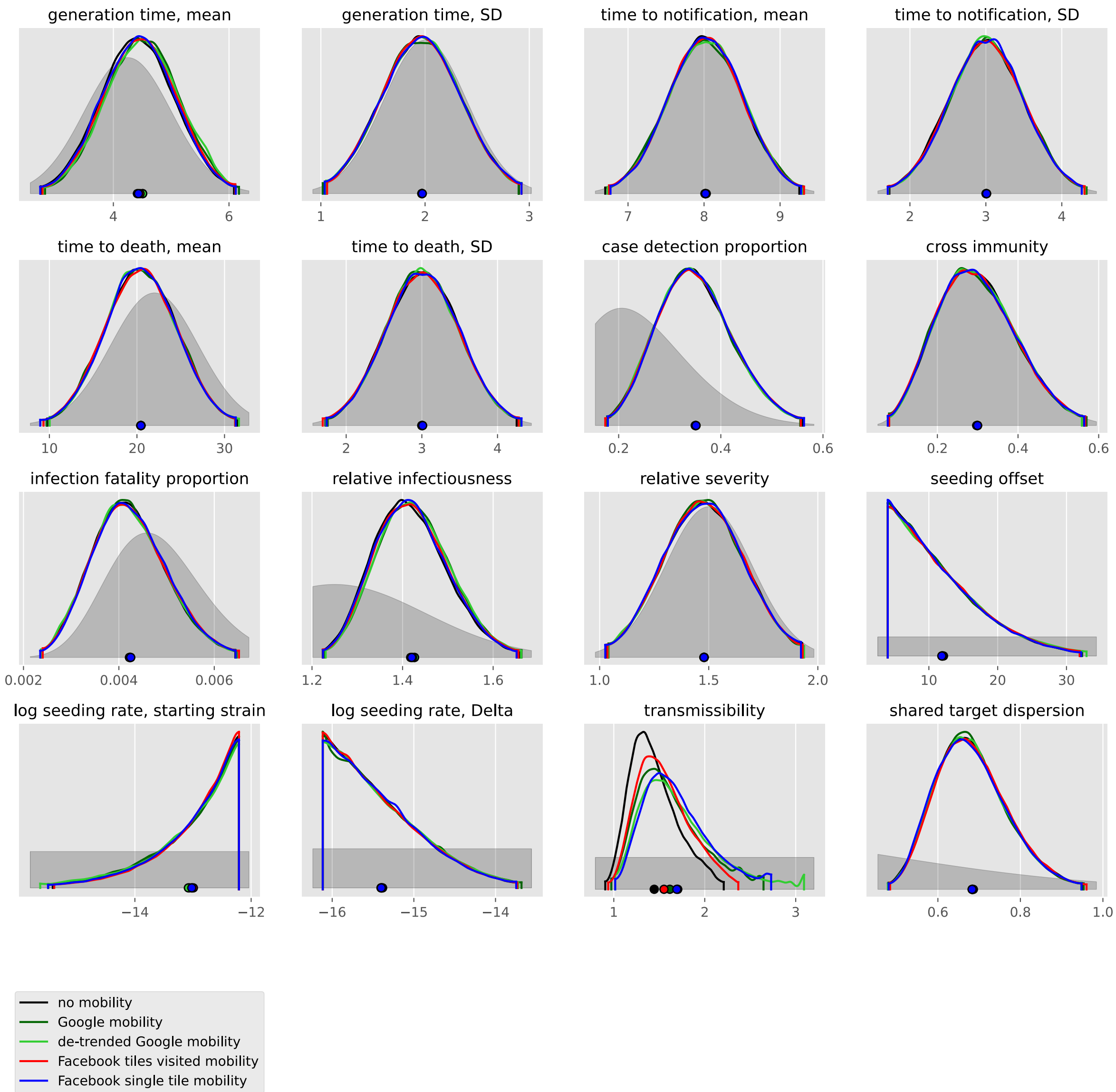

generation time, mean
generation time, SD
time to notification, mean
time to notification, SD
time to death, mean
time to death, SD
case detection proportion
cross immunity
infection fatality proportion
relative infectiousness
relative severity
seeding offset
log seeding rate, starting strain
log seeding rate, Delta
transmissibility
shared target dispersion
no mobility
Google mobility
de-trended Google mobility
Facebook tiles visited mobility
Facebook single tile mobility

Congo

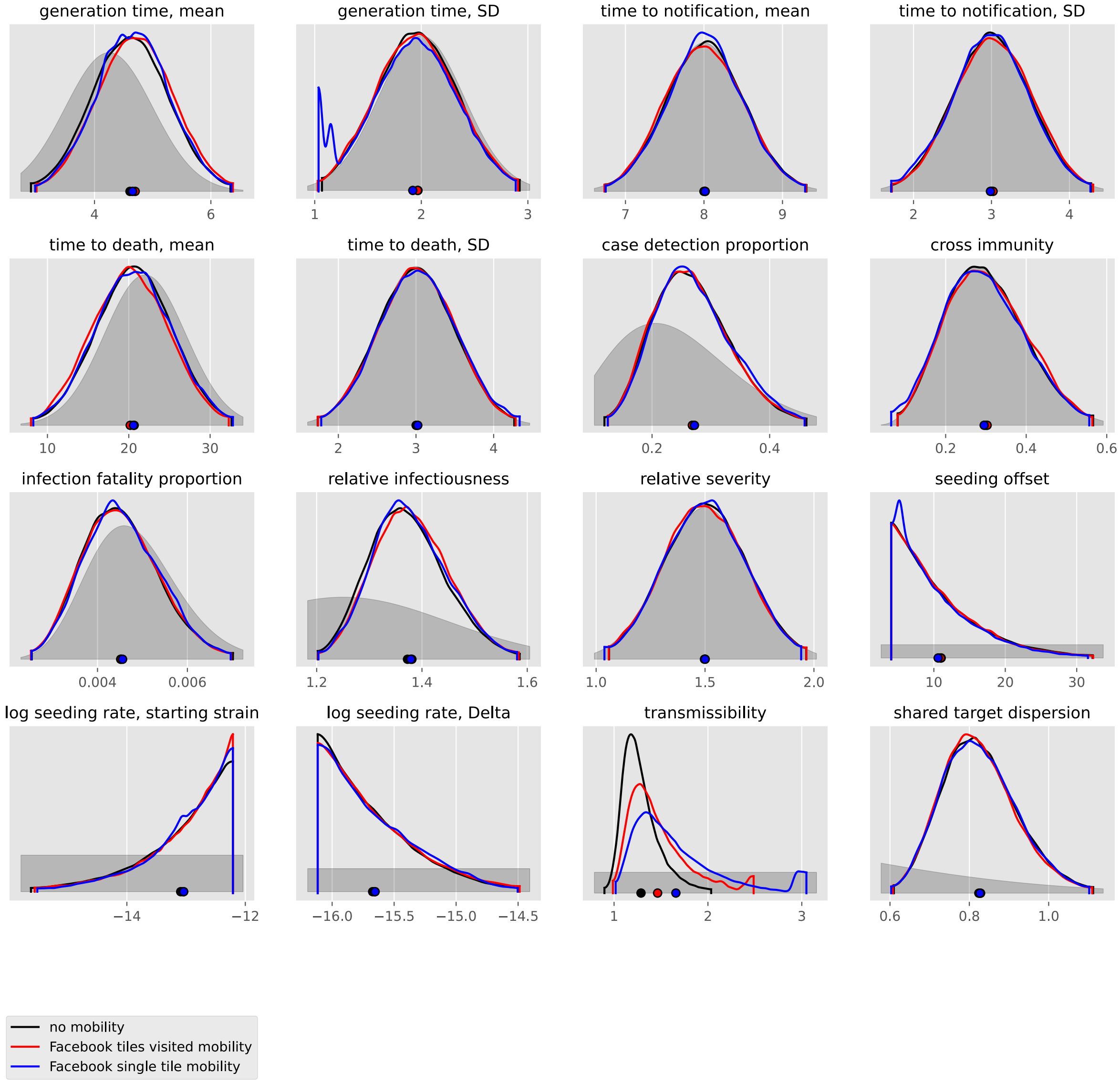

generation time, mean
generation time, SD
time to notification, mean
time to notification, SD
time to death, mean
time to death, SD
case detection proportion
cross immunity
infection fatality proportion
relative infectiousness
relative severity
seeding offset
log seeding rate, starting strain
log seeding rate, Delta
transmissibility
shared target dispersion
no mobility
Facebook tiles visited mobility
Facebook single tile mobility

Côte d'Ivoire

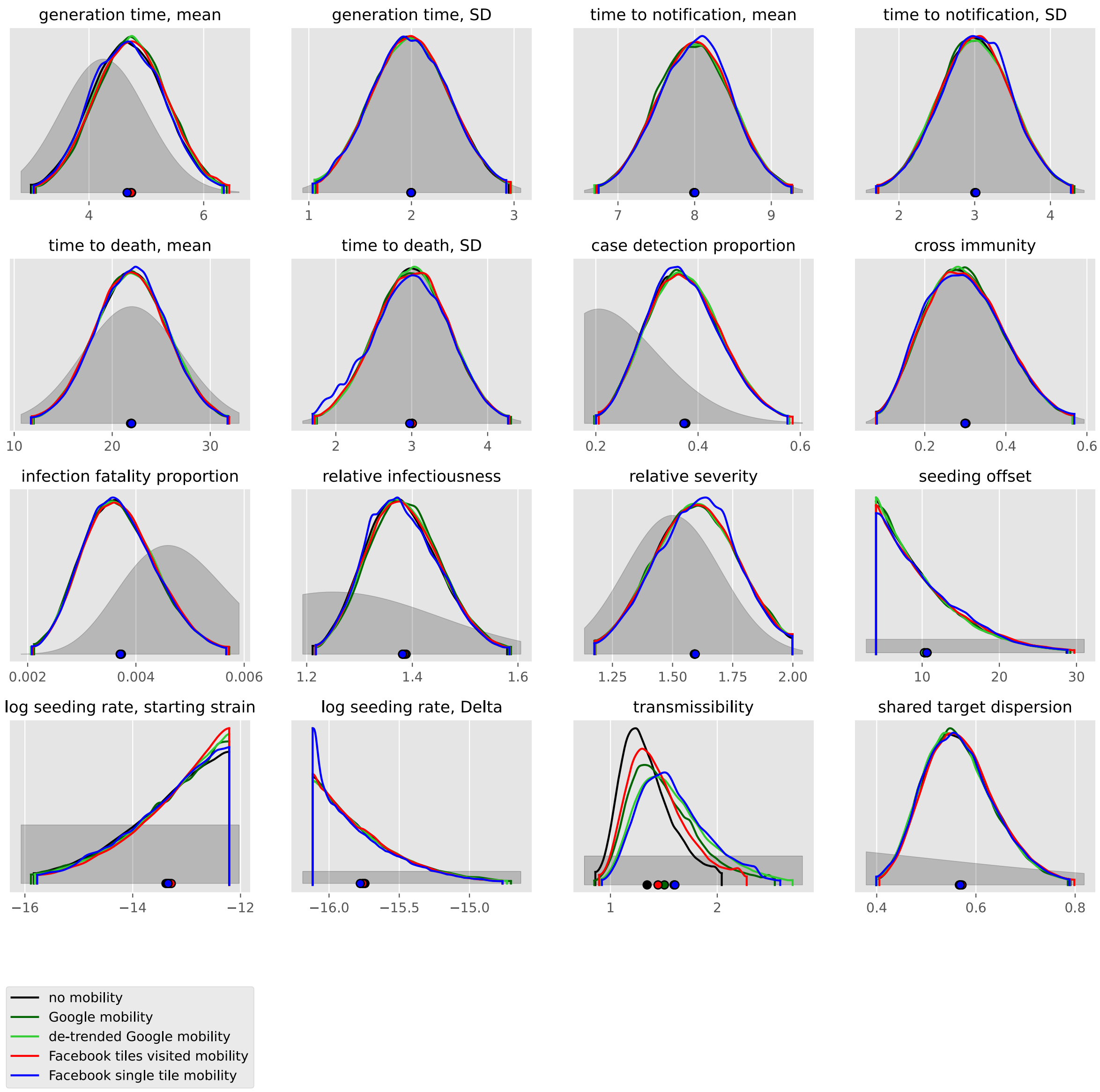

Egypt

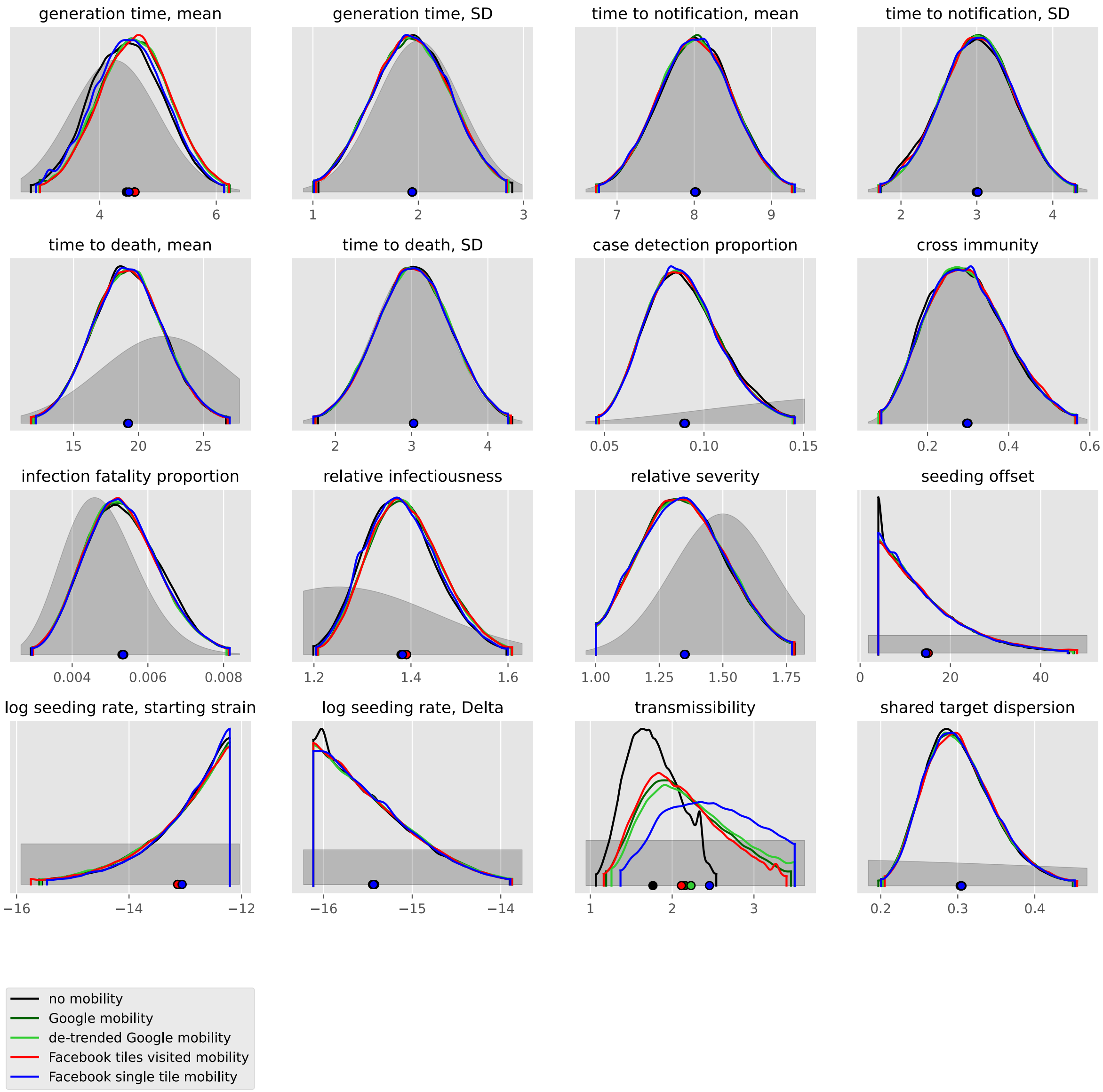

generation time, mean
generation time, SD
time to notification, mean
time to notification, SD
time to death, mean
time to death, SD
case detection proportion
cross immunity
infection fatality proportion
relative infectiousness
relative severity
seeding offset
log seeding rate, starting strain
log seeding rate, Delta
transmissibility
shared target dispersion
no mobility
Google mobility
de-trended Google mobility
Facebook tiles visited mobility
Facebook single tile mobility

Equatorial Guinea

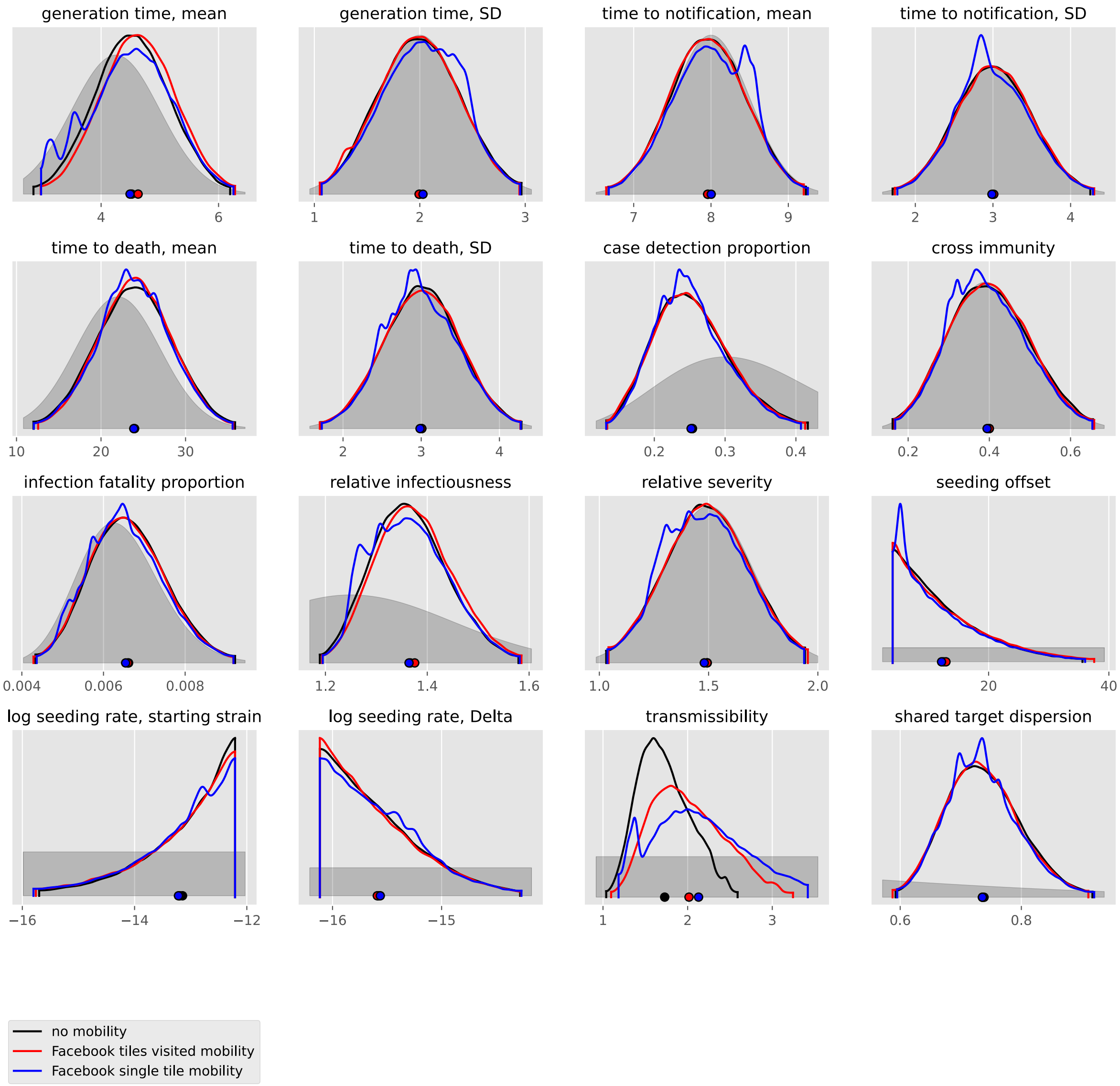

Ethiopia

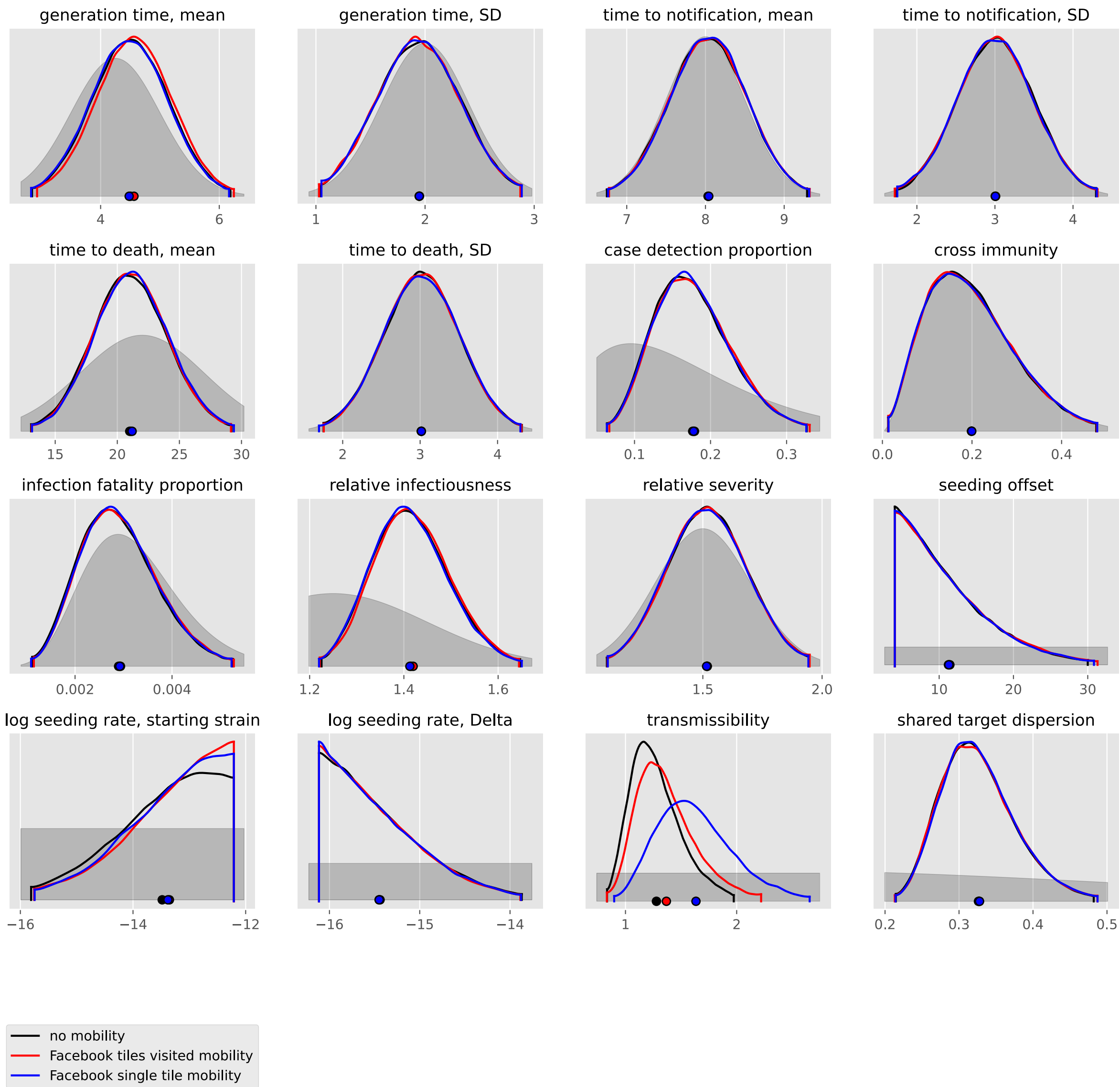

generation time, mean
4
6
generation time, SD
1
2
3
time to notification, mean
7
8
9
time to notification, SD
2
3
4
time to death, mean
15
20
25
30
time to death, SD
2
3
4
case detection proportion
0.1
0.2
0.3
cross immunity
0.0
0.2
0.4
infection fatality proportion
0.002
0.004
relative infectiousness
1.2
1.4
1.6
relative severity
1.5
2.0
seeding offset
10
20
30
log seeding rate, starting strain
−16
−14
−12
log seeding rate, Delta
−16
−15
−14
transmissibility
1
2
shared target dispersion
0.2
0.3
0.4
0.5
no mobility
Facebook tiles visited mobility
Facebook single tile mobility

Gabon

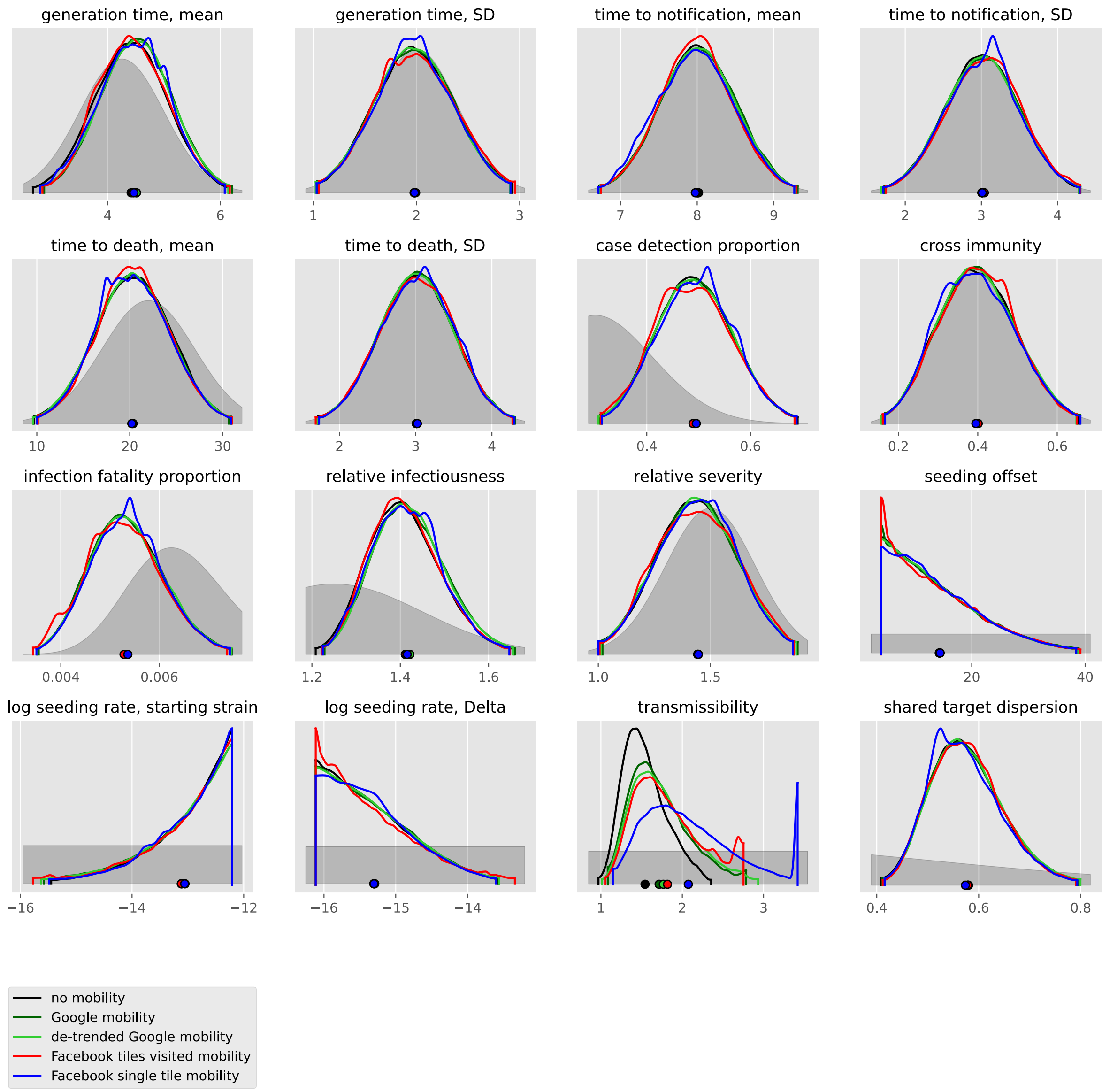

generation time, mean
generation time, SD
time to notification, mean
time to notification, SD
time to death, mean
time to death, SD
case detection proportion
cross immunity
infection fatality proportion
relative infectiousness
relative severity
seeding offset
log seeding rate, starting strain
log seeding rate, Delta
transmissibility
shared target dispersion
no mobility
Google mobility
de-trended Google mobility
Facebook tiles visited mobility
Facebook single tile mobility

Guinea

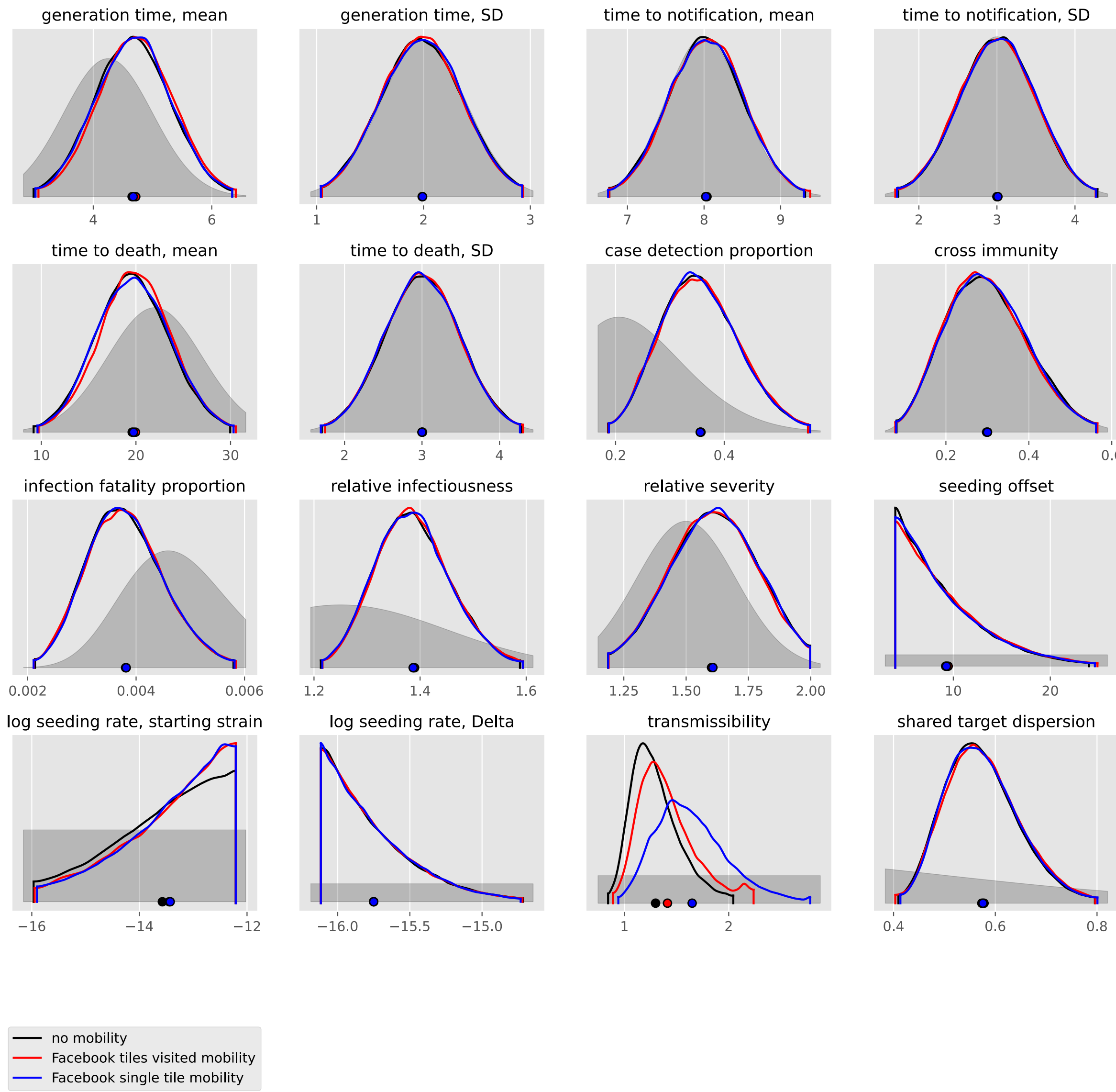

generation time, mean
4
6
generation time, SD
1
2
3
time to notification, mean
7
8
9
time to notification, SD
2
3
4
time to death, mean
10
20
30
time to death, SD
2
3
4
case detection proportion
0.2
0.4
cross immunity
0.2
0.4
0.6
infection fatality proportion
0.002
0.004
0.006
relative infectiousness
1.2
1.4
1.6
relative severity
1.25
1.50
1.75
2.00
seeding offset
10
20
log seeding rate, starting strain
−16
−14
−12
log seeding rate, Delta
−16.0
−15.5
−15.0
transmissibility
1
2
shared target dispersion
0.4
0.6
0.8
no mobility
Facebook tiles visited mobility
Facebook single tile mobility

Guinea-Bissau

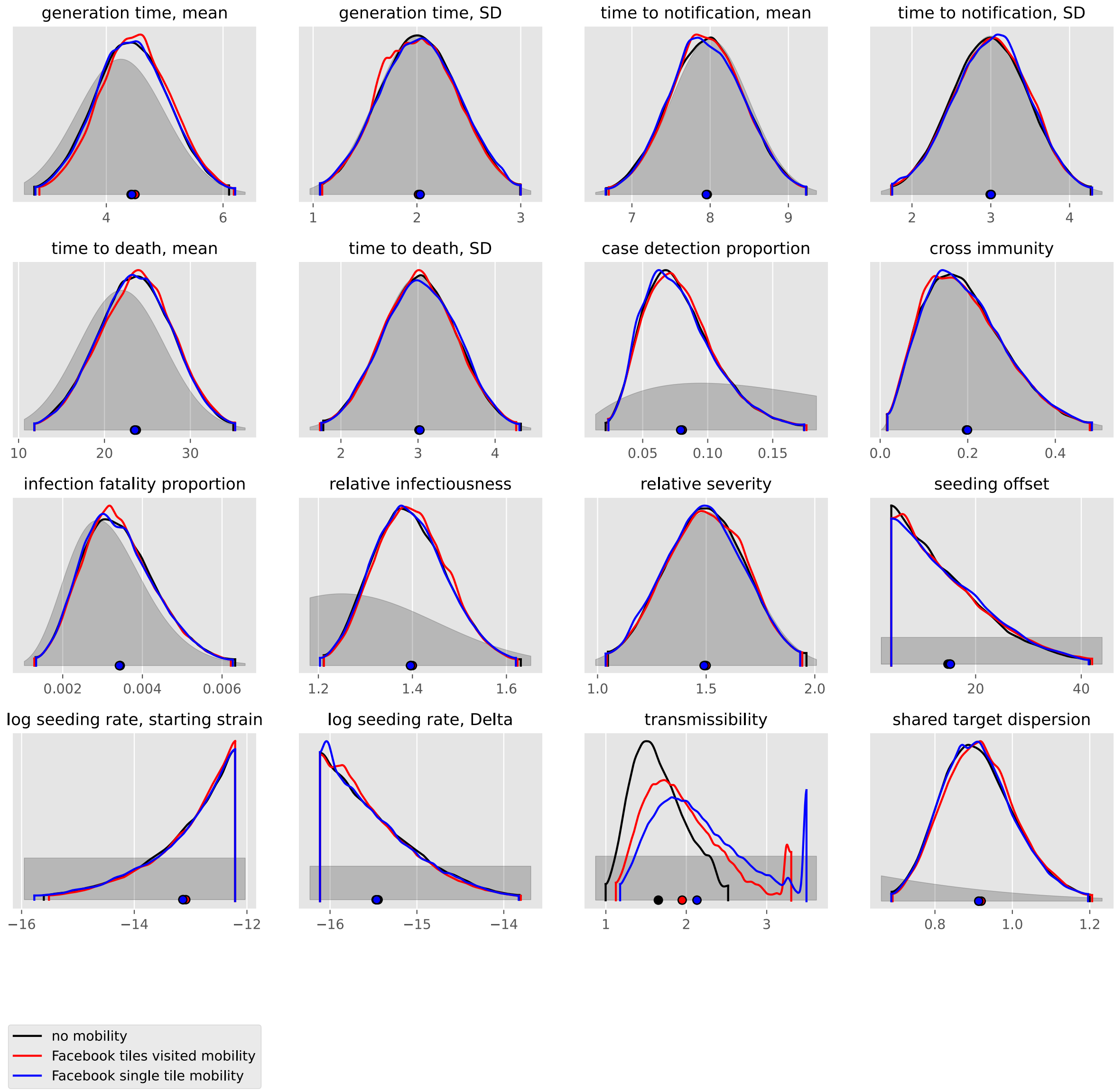

Kenya

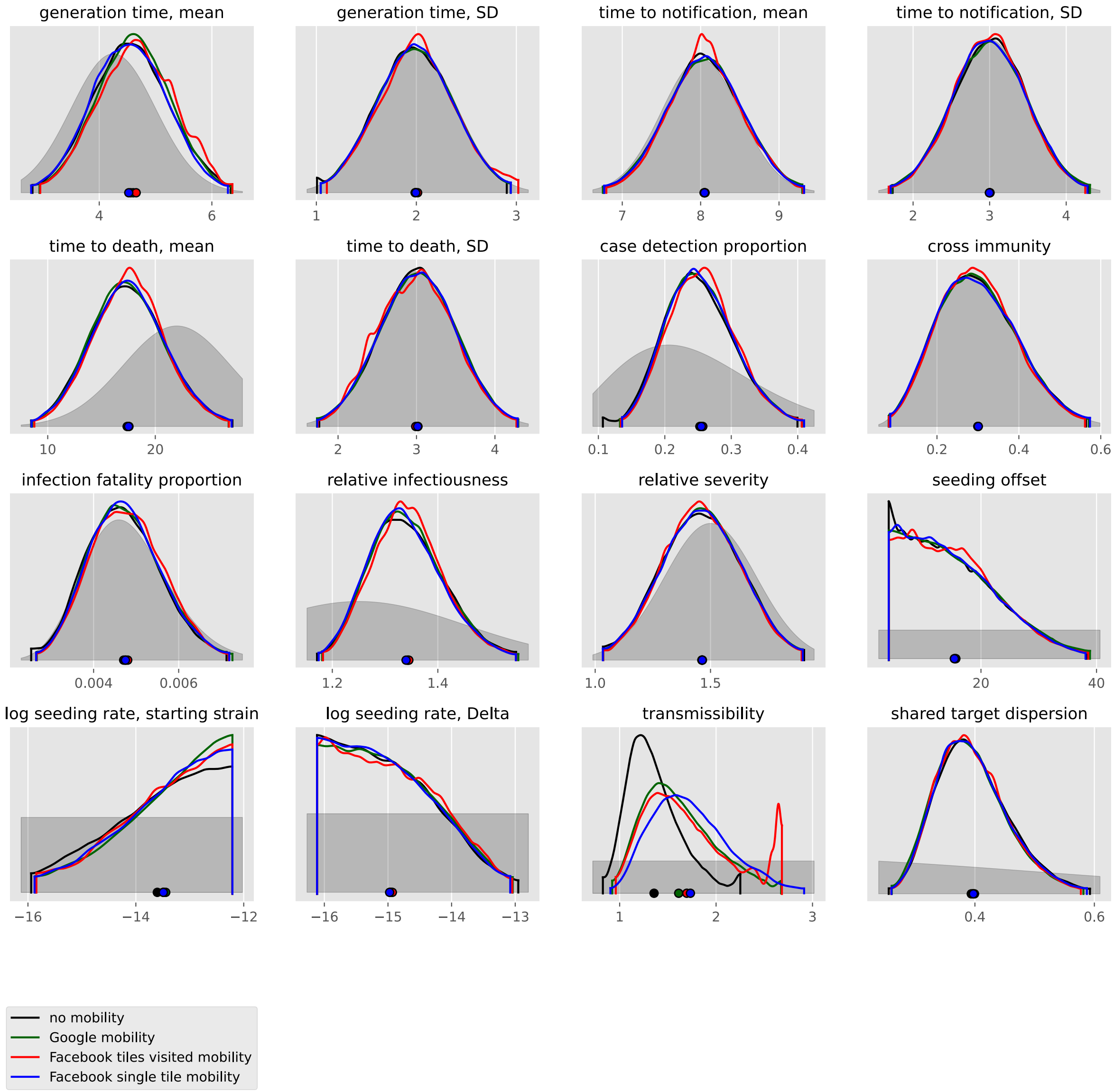

generation time, mean
generation time, SD
time to notification, mean
time to notification, SD
time to death, mean
time to death, SD
case detection proportion
cross immunity
infection fatality proportion
relative infectiousness
relative severity
seeding offset
log seeding rate, starting strain
log seeding rate, Delta
transmissibility
shared target dispersion
no mobility
Google mobility
Facebook tiles visited mobility
Facebook single tile mobility

Lesotho

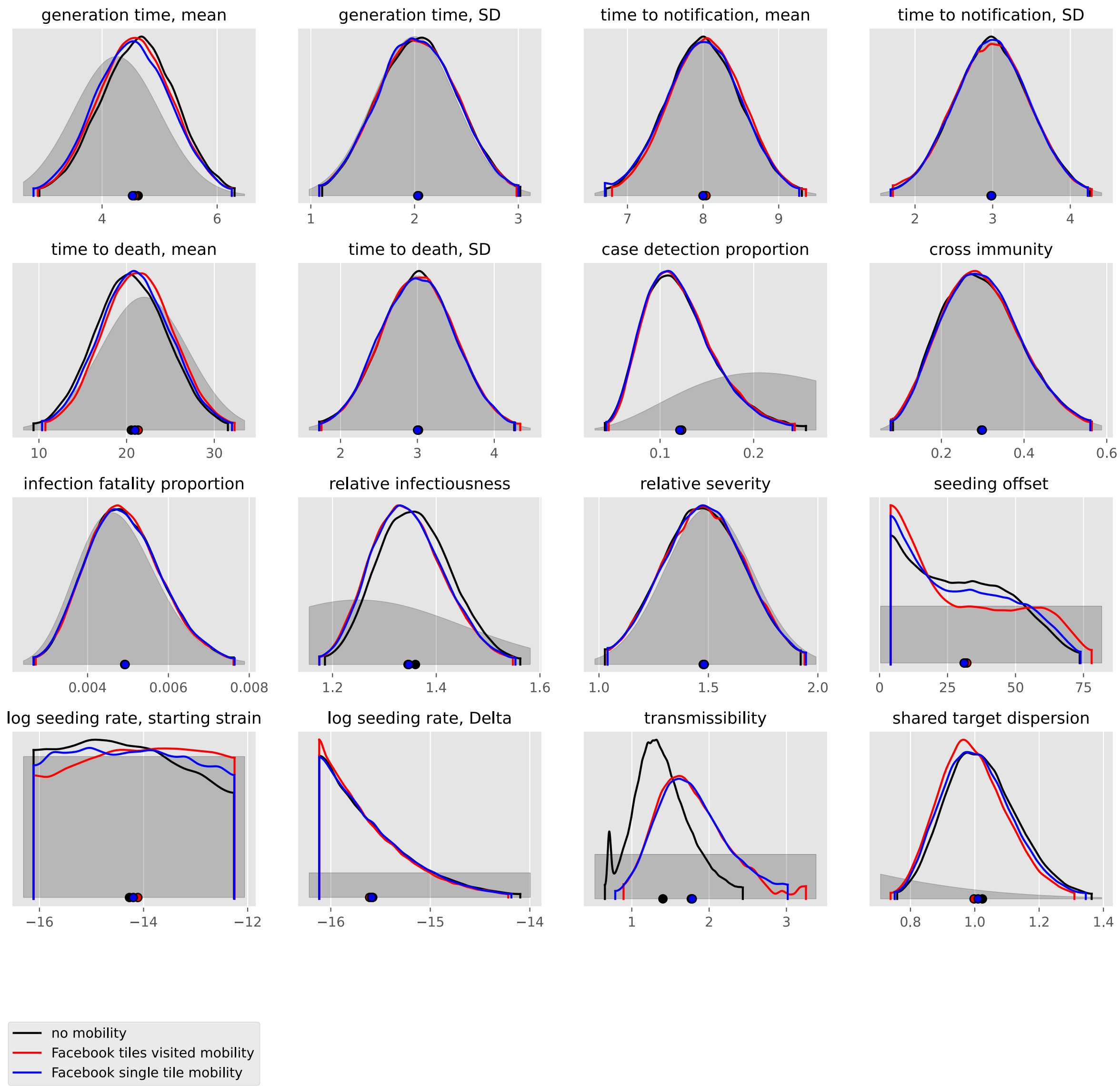
generation time, mean
generation time, SD
time to notification, mean
time to notification, SD
time to death, mean
time to death, SD
case detection proportion
cross immunity
infection fatality proportion
relative infectiousness
relative severity
seeding offset
log seeding rate, starting strain
log seeding rate, Delta
transmissibility
shared target dispersion
no mobility
Facebook tiles visited mobility
Facebook single tile mobility

Liberia

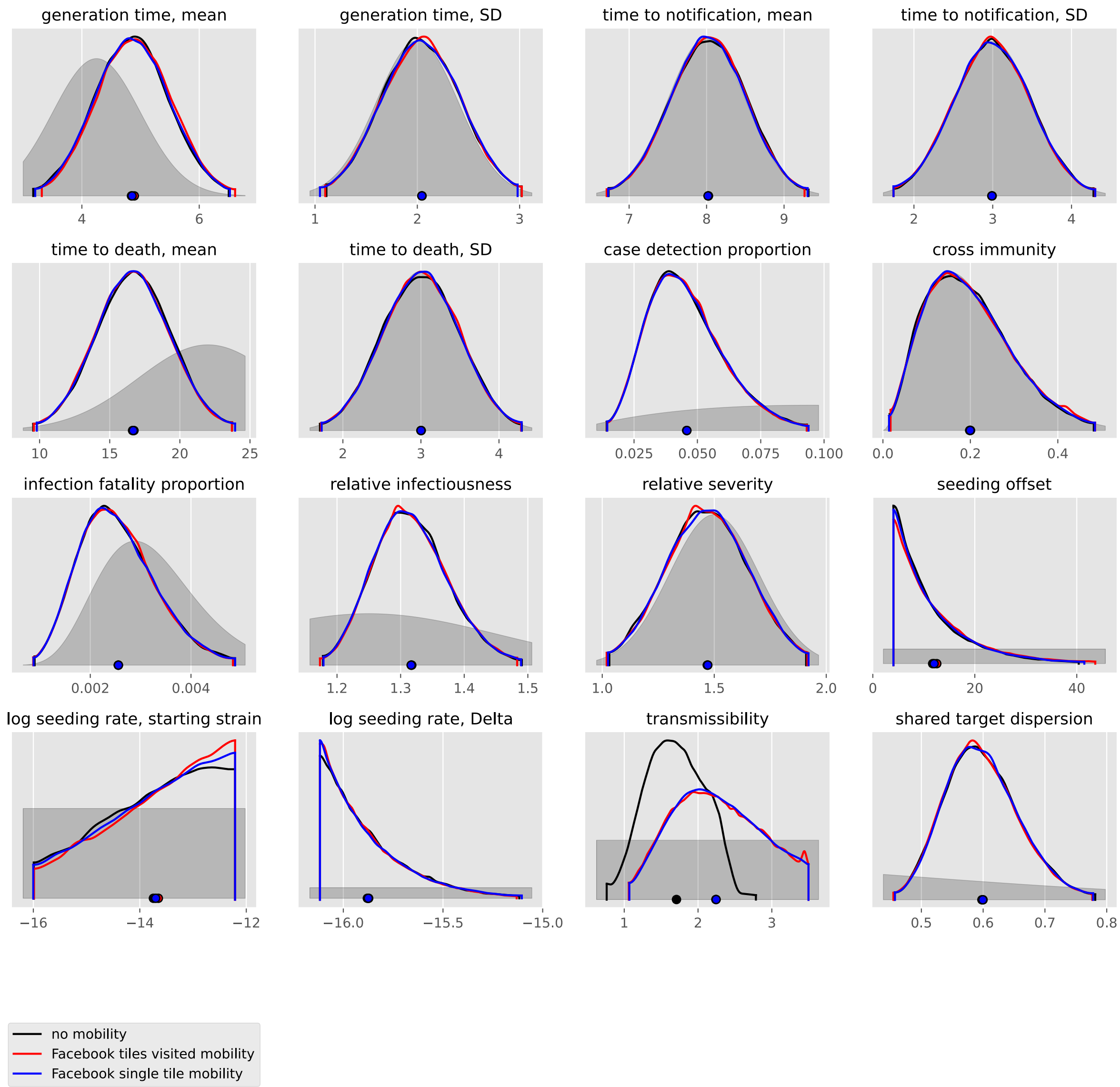
generation time, mean
generation time, SD
time to notification, mean
time to notification, SD
time to death, mean
time to death, SD
case detection proportion
cross immunity
infection fatality proportion
relative infectiousness
relative severity
seeding offset
log seeding rate, starting strain
log seeding rate, Delta
transmissibility
shared target dispersion
no mobility
Facebook tiles visited mobility
Facebook single tile mobility

Libya

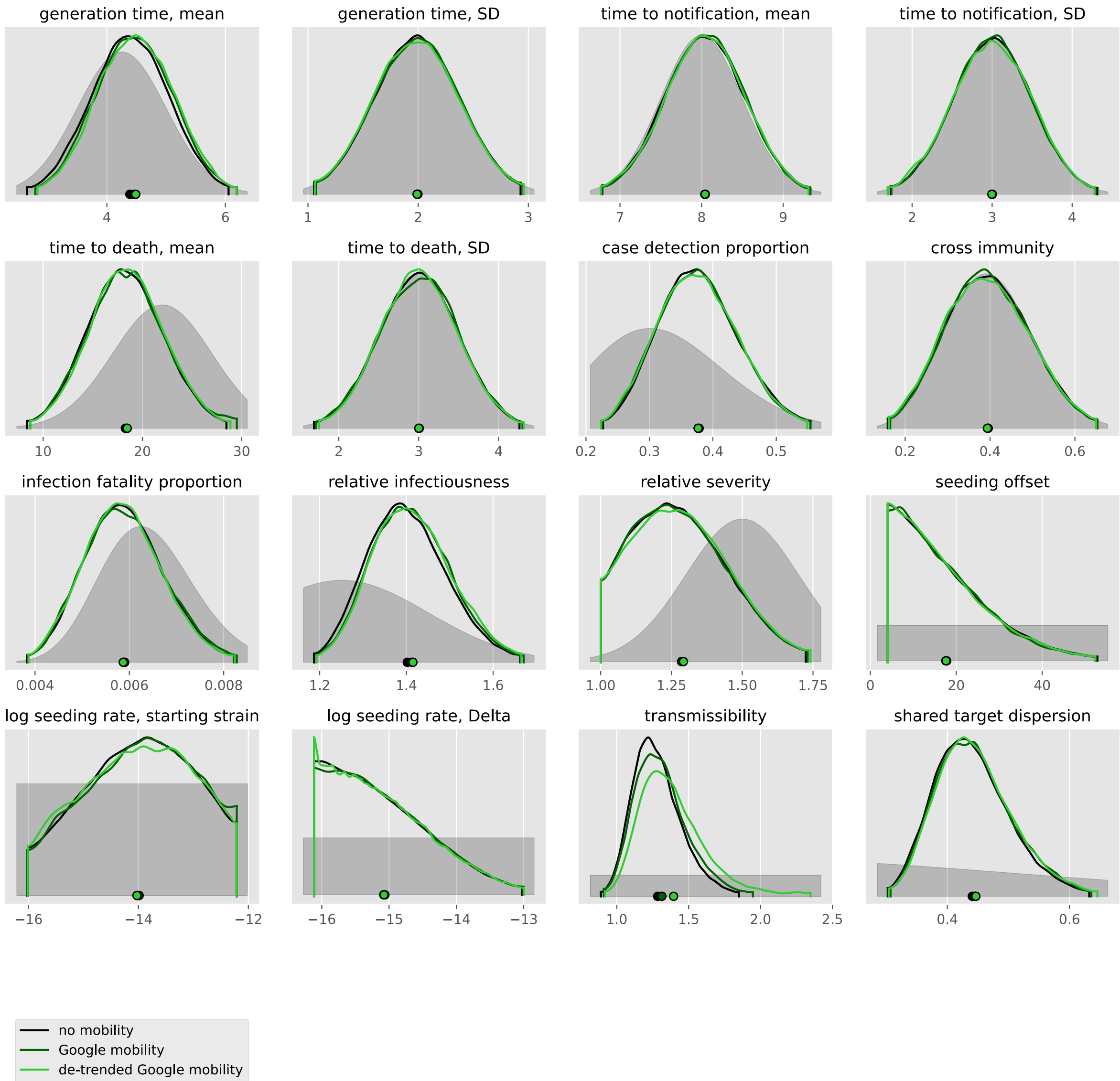
generation time, mean
generation time, SD
time to notification, mean
time to notification, SD
time to death, mean
time to death, SD
case detection proportion
cross immunity
infection fatality proportion
relative infectiousness
relative severity
seeding offset
log seeding rate, starting strain
log seeding rate, Delta
transmissibility
shared target dispersion
no mobility
Google mobility
de-trended Google mobility

Madagascar

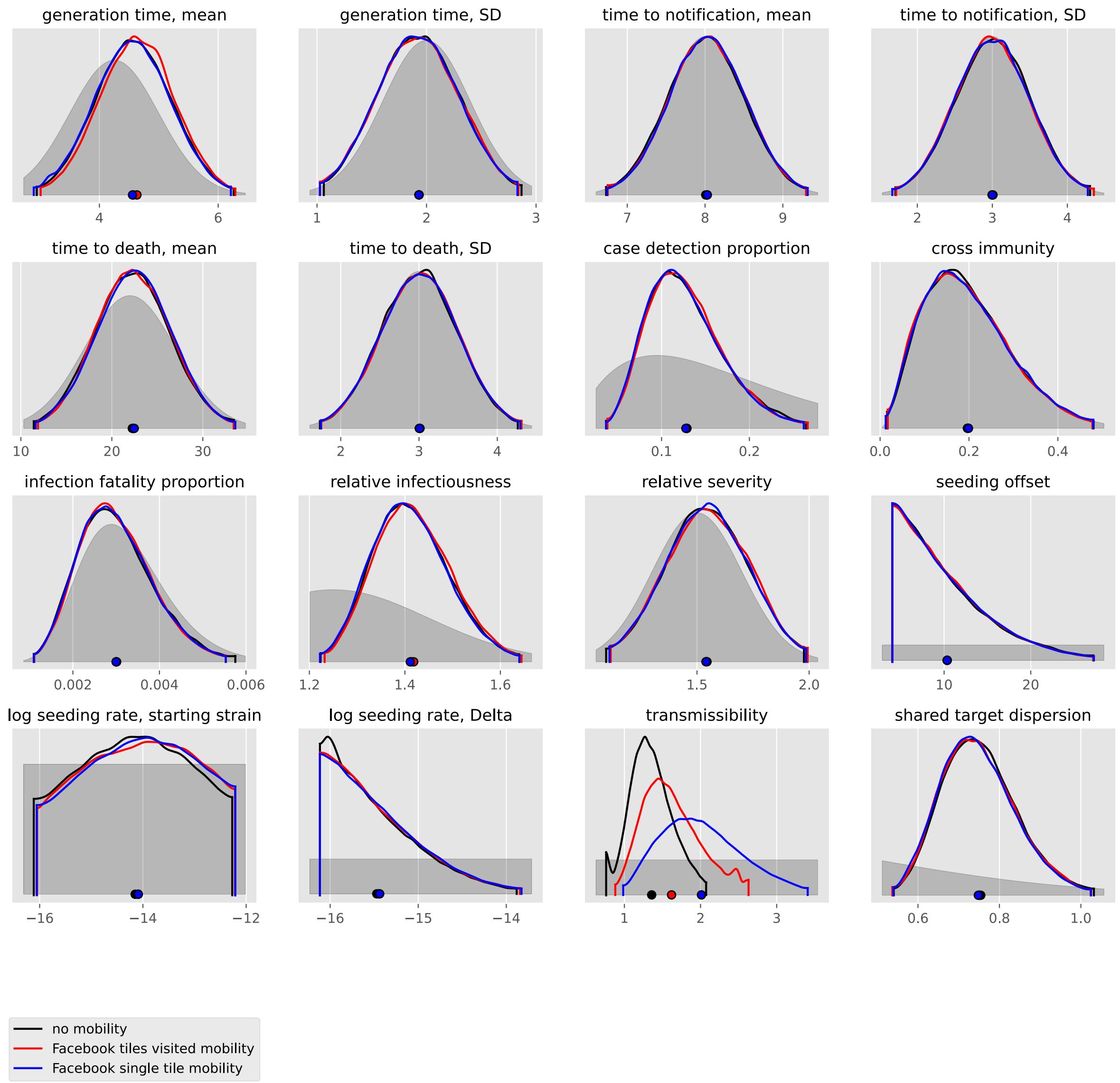
generation time, mean
generation time, SD
time to notification, mean
time to notification, SD
time to death, mean
time to death, SD
case detection proportion
cross immunity
infection fatality proportion
relative infectiousness
relative severity
seeding offset
log seeding rate, starting strain
log seeding rate, Delta
transmissibility
shared target dispersion
no mobility
Facebook tiles visited mobility
Facebook single tile mobility

Malawi

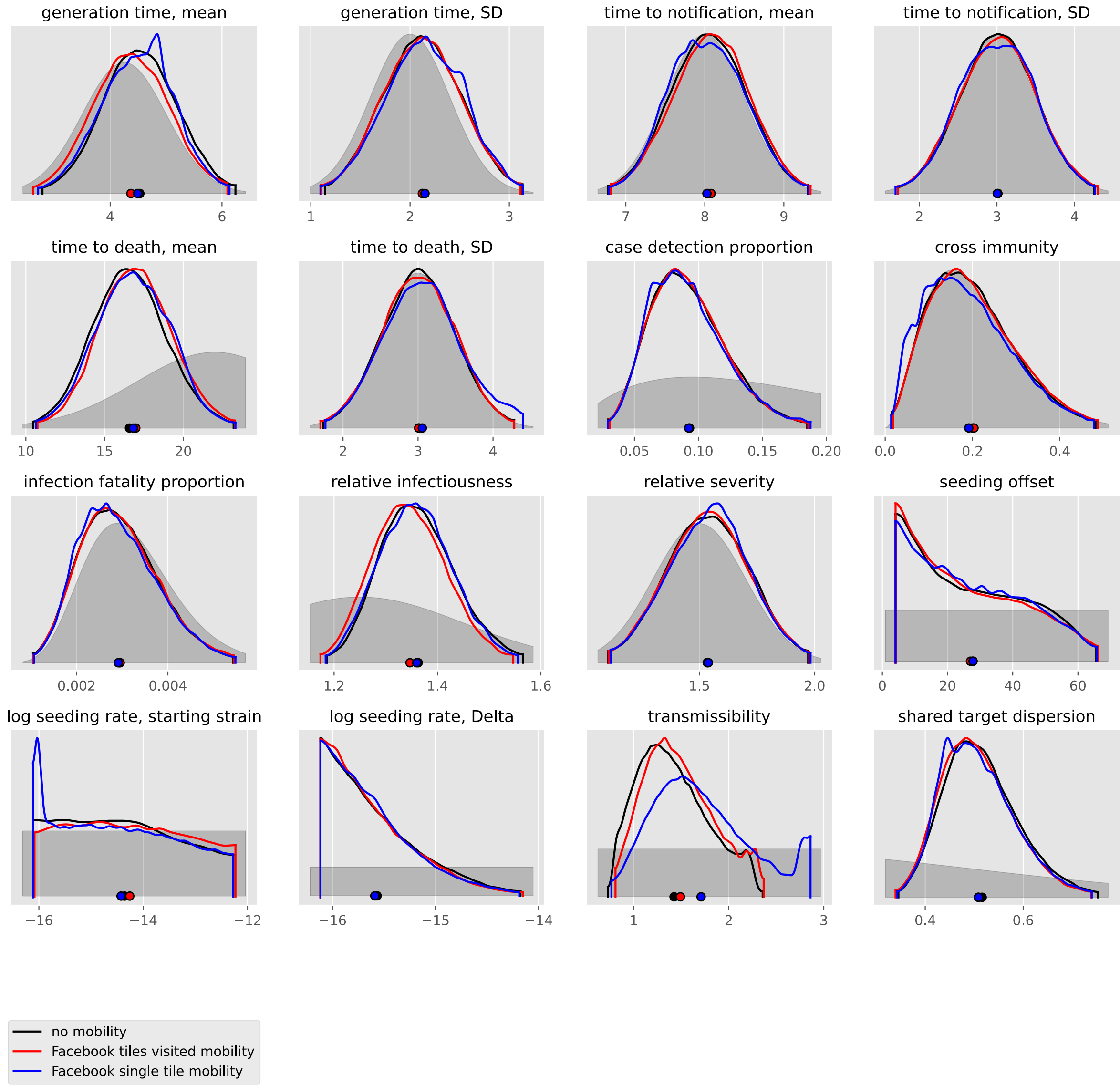
generation time, mean
4
6
generation time, SD
1
2
3
time to notification, mean
7
8
9
time to notification, SD
2
3
4
time to death, mean
10
15
20
time to death, SD
2
3
4
case detection proportion
0.05
0.10
0.15
0.20
cross immunity
0.0
0.2
0.4
infection fatality proportion
0.002
0.004
relative infectiousness
1.2
1.4
1.6
relative severity
1.5
2.0
seeding offset
0
20
40
60
log seeding rate, starting strain
−16
−14
−12
log seeding rate, Delta
−16
−15
−14
transmissibility
1
2
3
shared target dispersion
0.4
0.6
no mobility
Facebook tiles visited mobility
Facebook single tile mobility

Mali

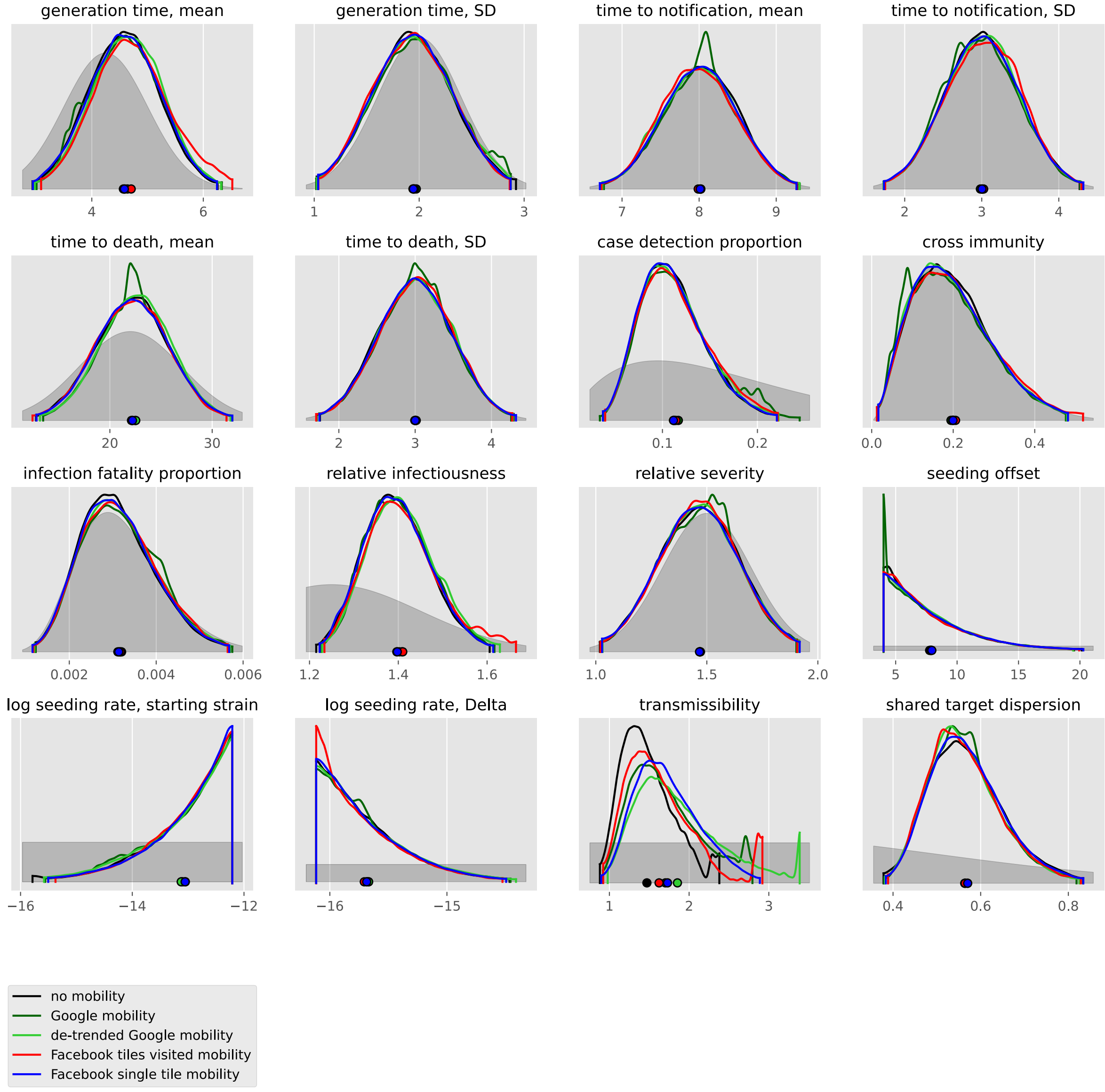
generation time, mean
4
6
generation time, SD
1
2
3
time to notification, mean
7
8
9
time to notification, SD
2
3
4
time to death, mean
20
30
time to death, SD
2
3
4
case detection proportion
0.1
0.2
cross immunity
0.0
0.2
0.4
infection fatality proportion
0.002
0.004
0.006
relative infectiousness
1.2
1.4
1.6
relative severity
1.0
1.5
2.0
seeding offset
5
10
15
20
log seeding rate, starting strain
−16
−14
−12
log seeding rate, Delta
−16
−15
transmissibility
1
2
3
shared target dispersion
0.4
0.6
0.8
no mobility
Google mobility
de-trended Google mobility
Facebook tiles visited mobility
Facebook single tile mobility

Morocco

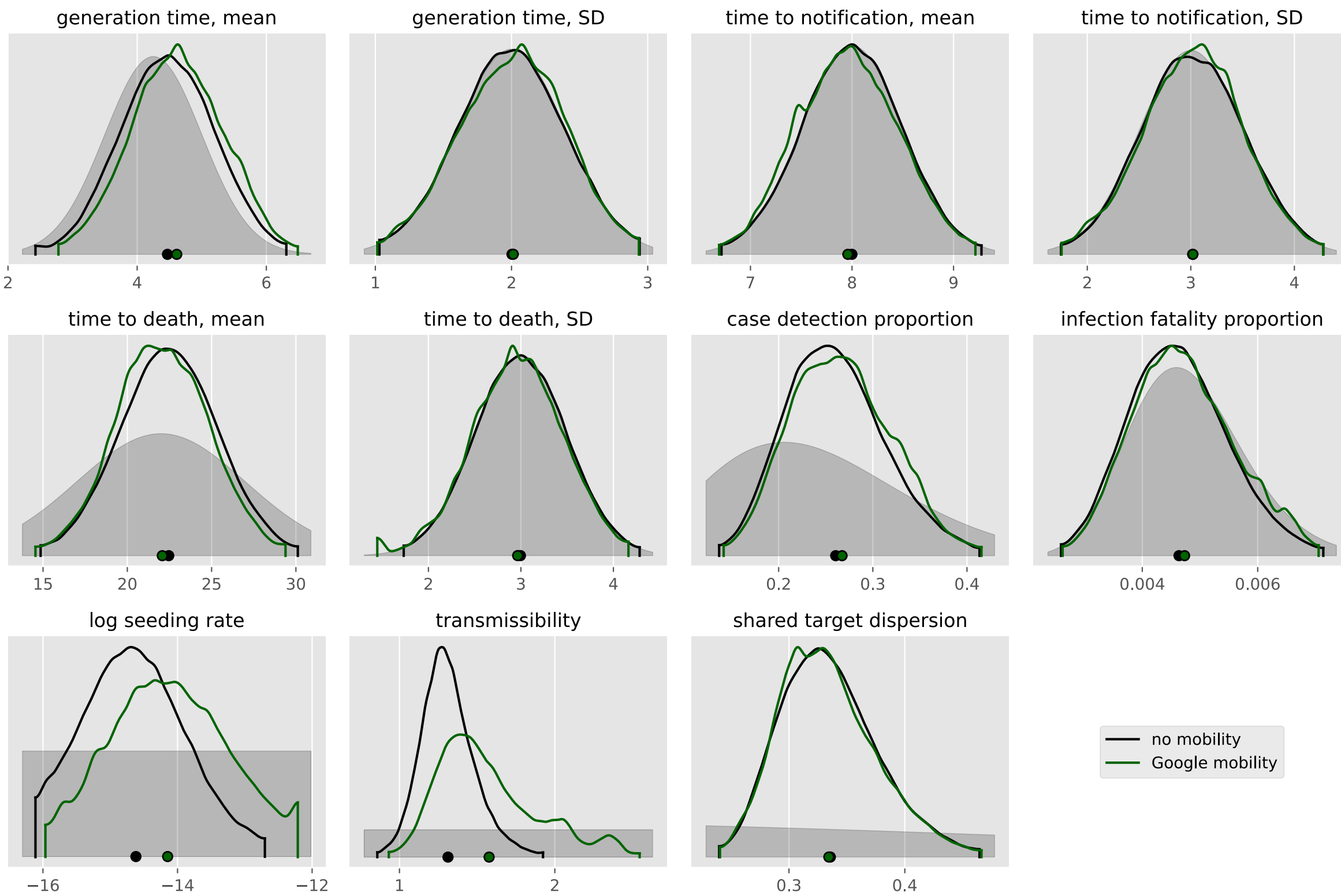

generation time, mean
2
4
6
generation time, SD
1
2
3
time to notification, mean
7
8
9
time to notification, SD
2
3
4
time to death, mean
15
20
25
30
time to death, SD
2
3
4
case detection proportion
0.2
0.3
0.4
infection fatality proportion
0.004
0.006
log seeding rate
−16
−14
−12
transmissibility
1
2
shared target dispersion
0.3
0.4
no mobility
Google mobility

Mozambique

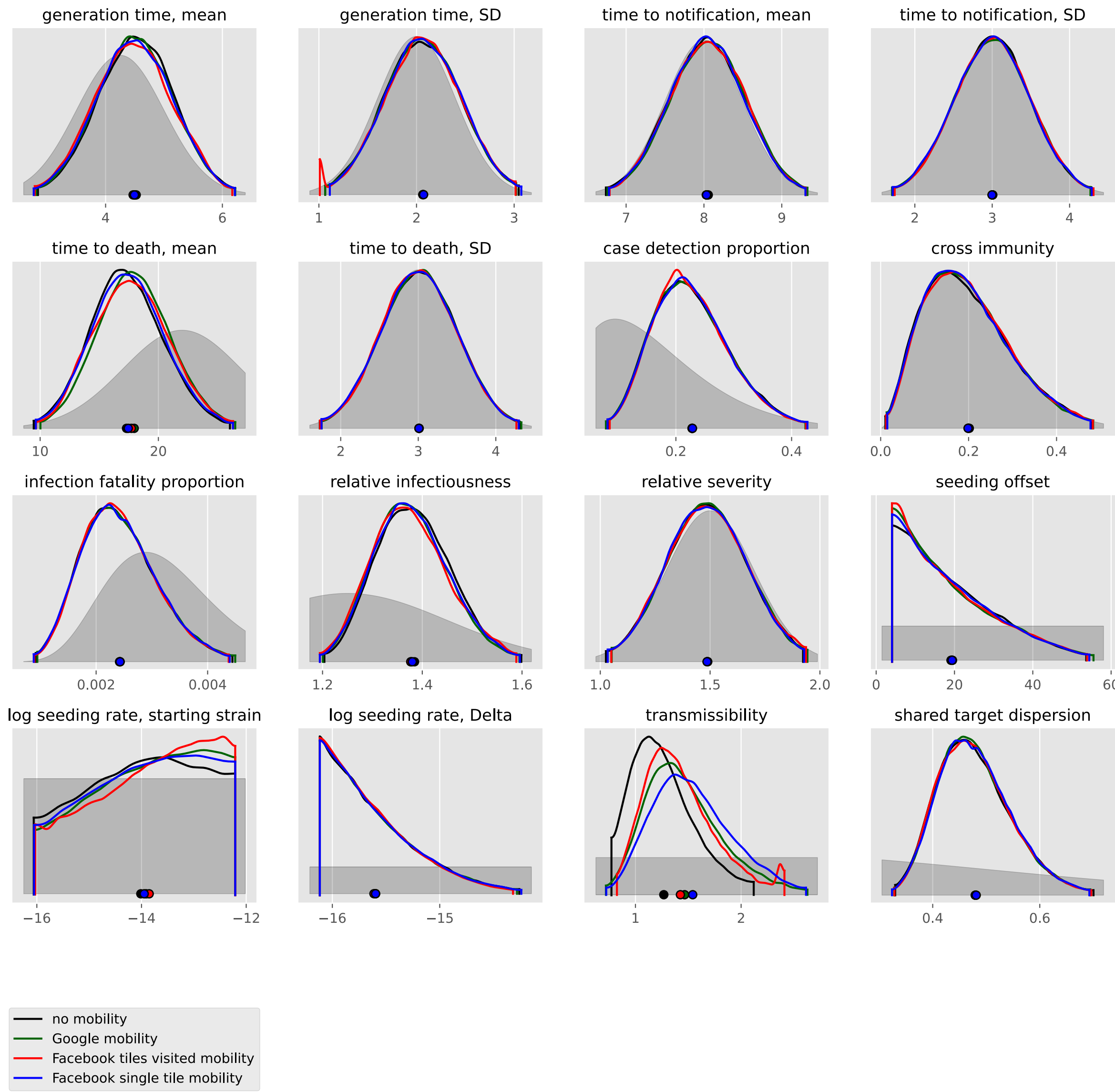

generation time, mean
generation time, SD
time to notification, mean
time to notification, SD
time to death, mean
time to death, SD
case detection proportion
cross immunity
infection fatality proportion
relative infectiousness
relative severity
seeding offset
log seeding rate, starting strain
log seeding rate, Delta
transmissibility
shared target dispersion
no mobility
Google mobility
Facebook tiles visited mobility
Facebook single tile mobility

Nigeria

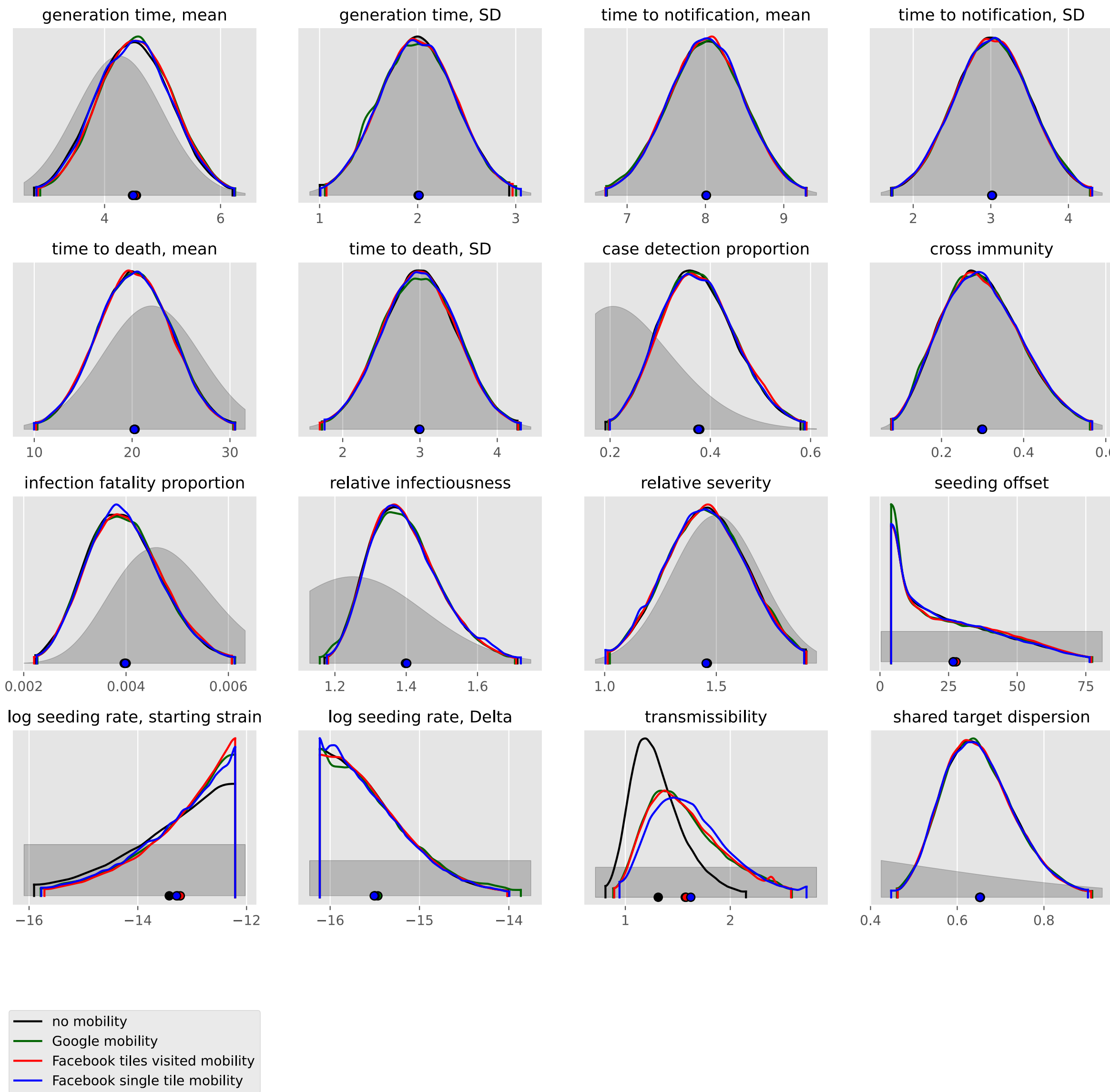

generation time, mean
generation time, SD
time to notification, mean
time to notification, SD
time to death, mean
time to death, SD
case detection proportion
cross immunity
infection fatality proportion
relative infectiousness
relative severity
seeding offset
log seeding rate, starting strain
log seeding rate, Delta
transmissibility
shared target dispersion
no mobility
Google mobility
Facebook tiles visited mobility
Facebook single tile mobility

Rwanda

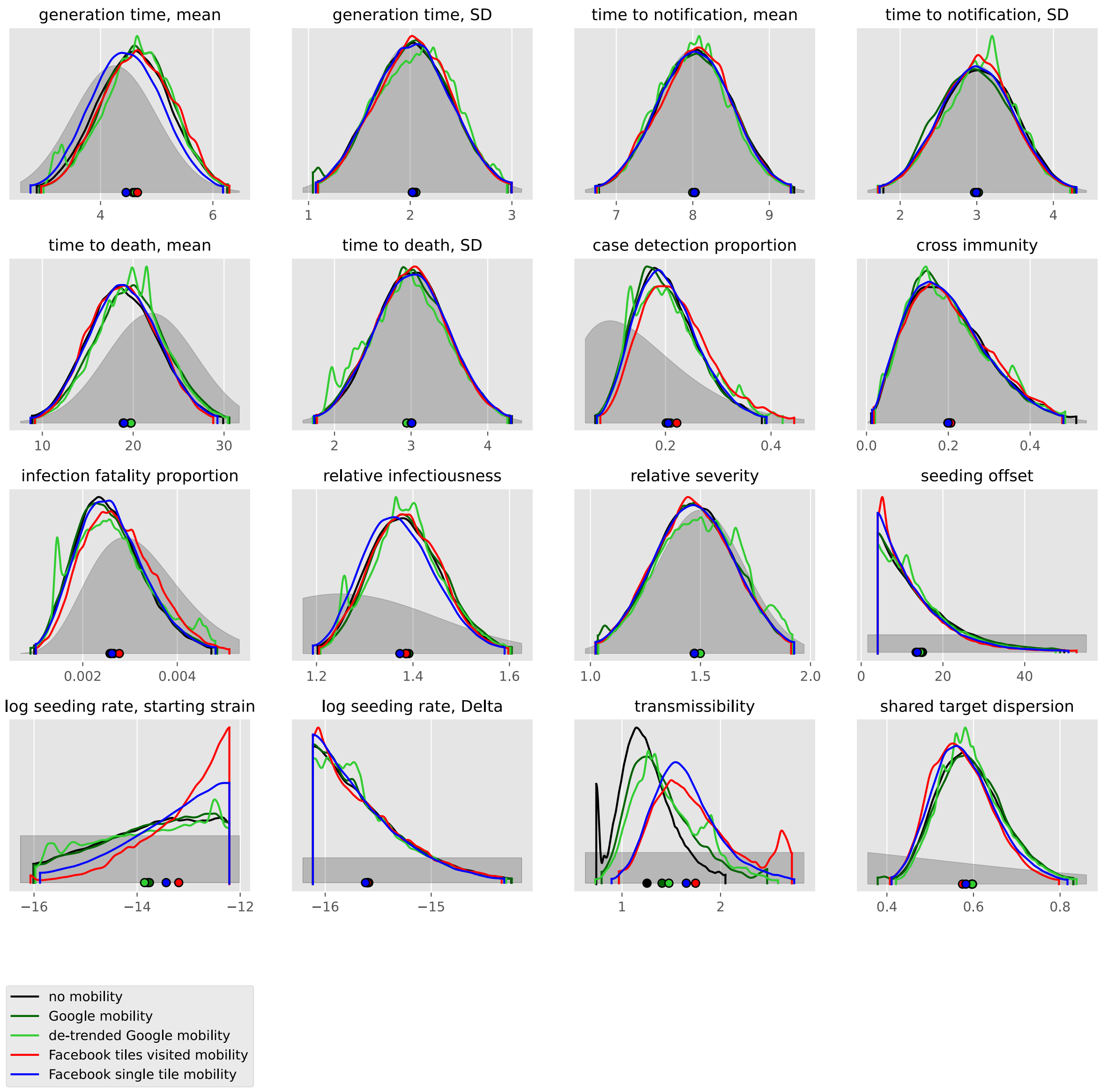
generation time, mean
generation time, SD
time to notification, mean
time to notification, SD
time to death, mean
time to death, SD
case detection proportion
cross immunity
infection fatality proportion
relative infectiousness
relative severity
seeding offset
log seeding rate, starting strain
log seeding rate, Delta
transmissibility
shared target dispersion
no mobility
Google mobility
de-trended Google mobility
Facebook tiles visited mobility
Facebook single tile mobility

Sao Tome and Principe

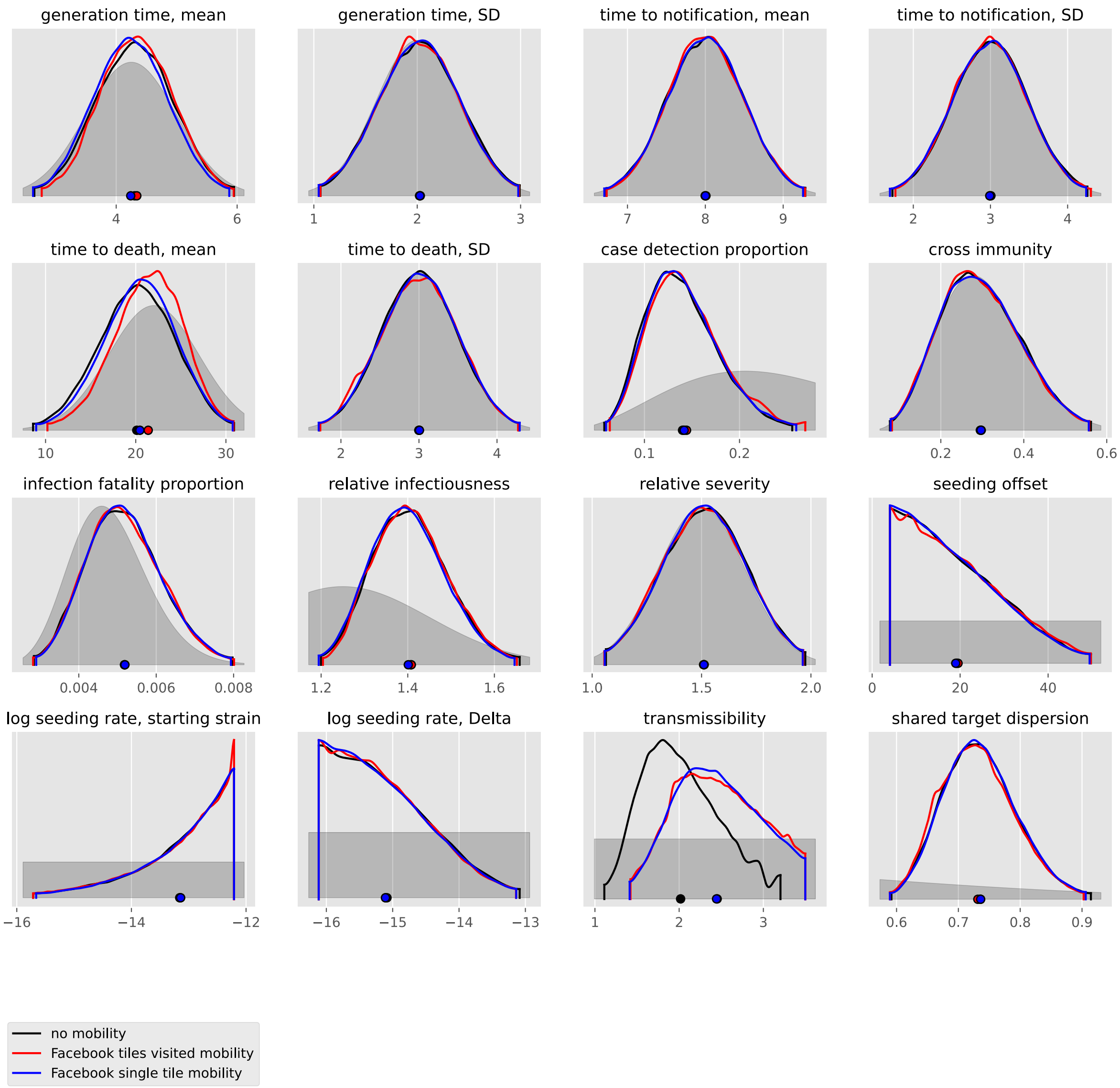

Senegal

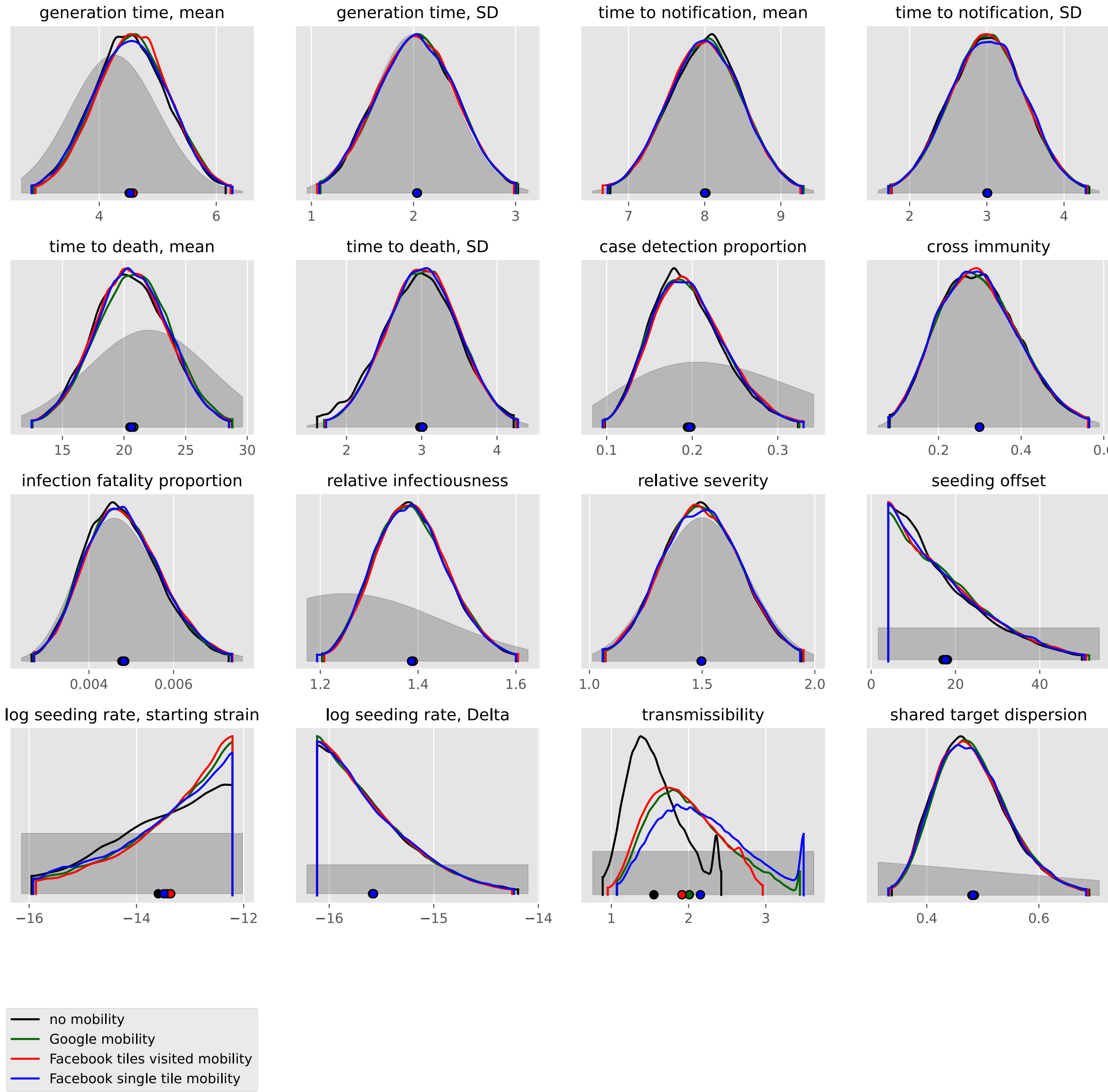
generation time, mean
4
6
generation time, SD
1
2
3
time to notification, mean
7
8
9
time to notification, SD
2
3
4
time to death, mean
15
20
25
30
time to death, SD
2
3
4
case detection proportion
0.1
0.2
0.3
cross immunity
0.2
0.4
0.6
infection fatality proportion
0.004
0.006
relative infectiousness
1.2
1.4
1.6
relative severity
1.0
1.5
2.0
seeding offset
0
20
40
log seeding rate, starting strain
−16
−14
−12
log seeding rate, Delta
−16
−15
−14
transmissibility
1
2
3
shared target dispersion
0.4
0.6
no mobility
Google mobility
Facebook tiles visited mobility
Facebook single tile mobility

South Africa

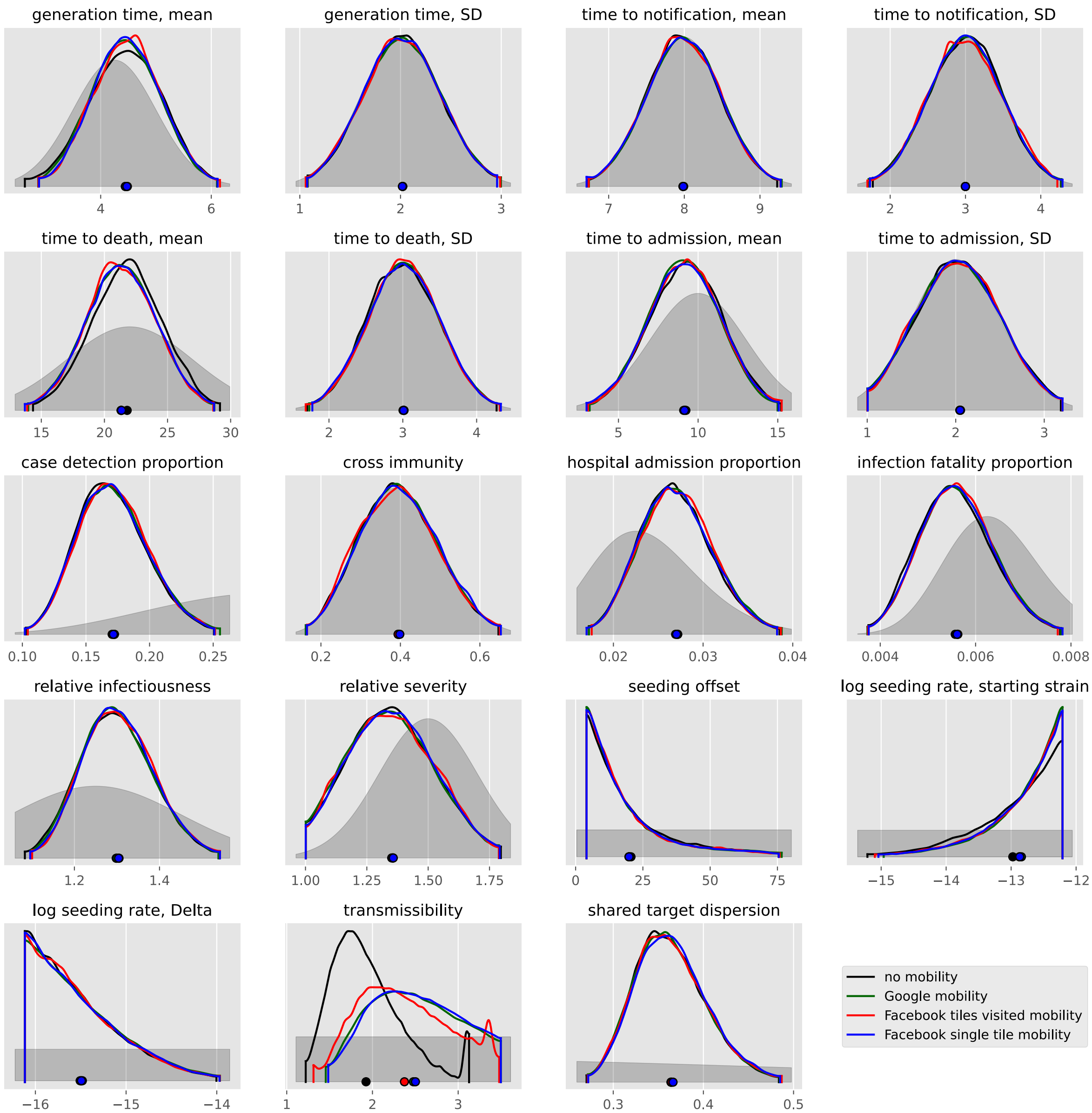

Togo

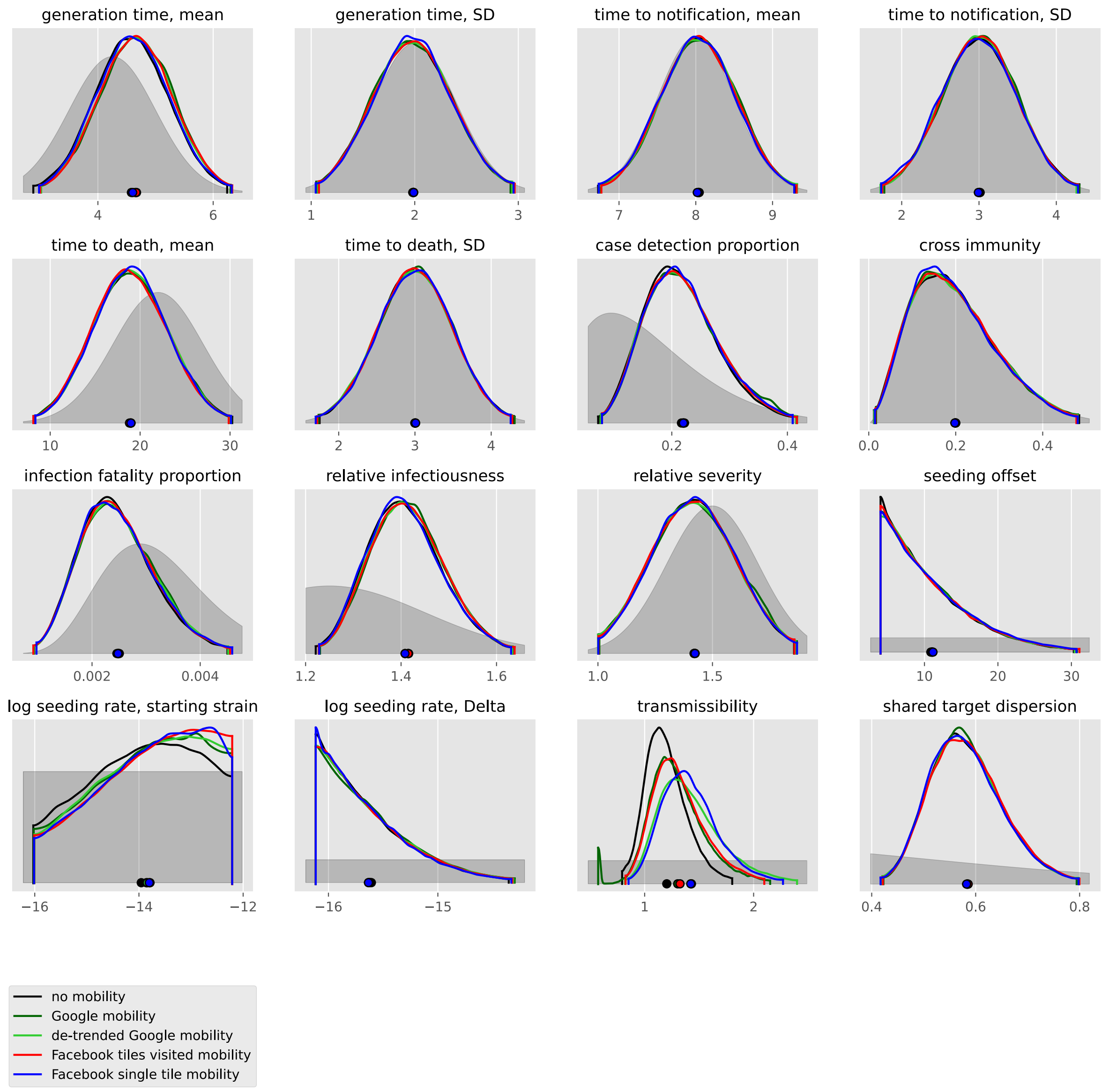

generation time, mean
generation time, SD
time to notification, mean
time to notification, SD
time to death, mean
time to death, SD
case detection proportion
cross immunity
infection fatality proportion
relative infectiousness
relative severity
seeding offset
log seeding rate, starting strain
log seeding rate, Delta
transmissibility
shared target dispersion
no mobility
Google mobility
de-trended Google mobility
Facebook tiles visited mobility
Facebook single tile mobility

Tunisia

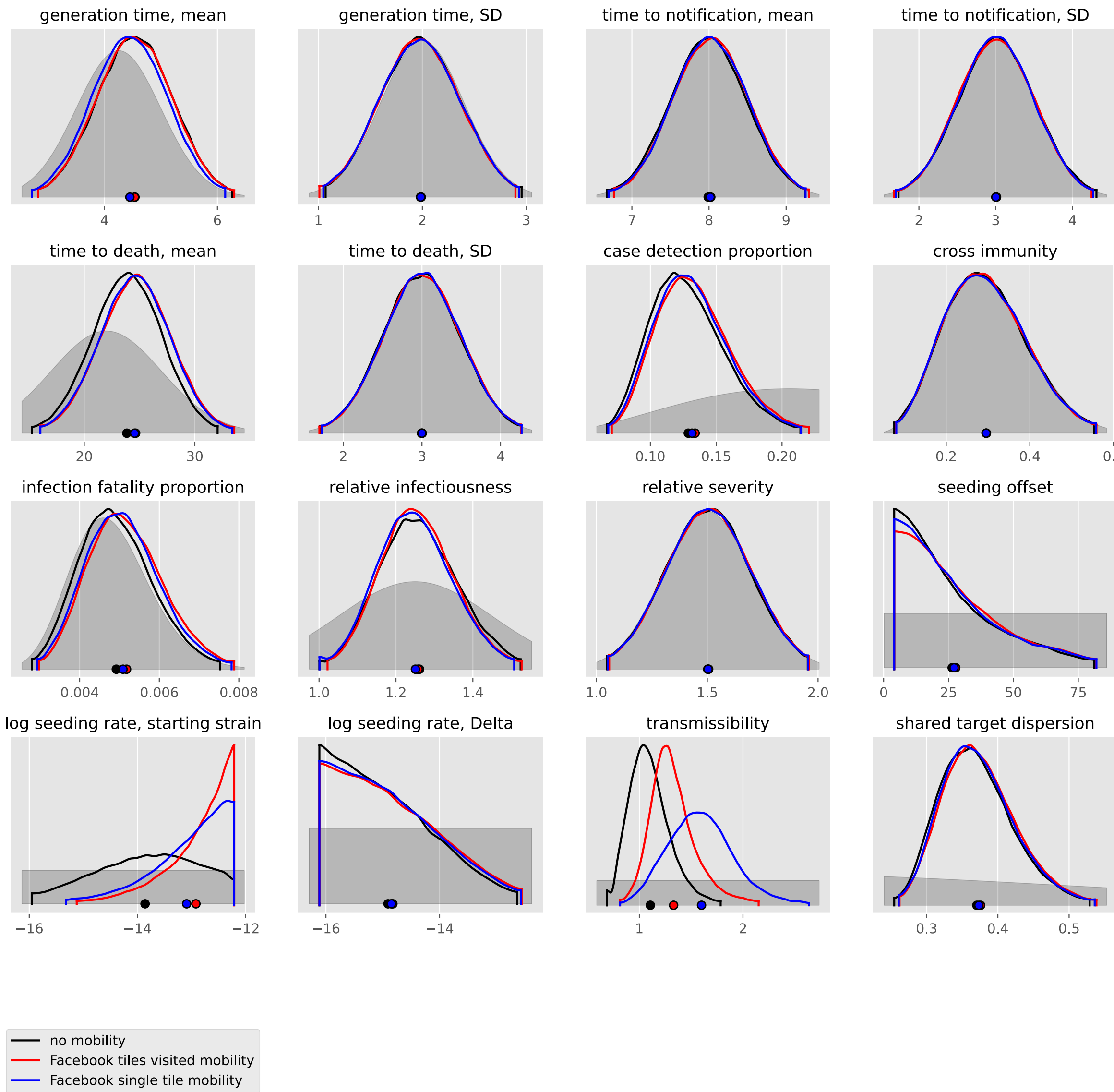

generation time, mean
generation time, SD
time to notification, mean
time to notification, SD
time to death, mean
time to death, SD
case detection proportion
cross immunity
infection fatality proportion
relative infectiousness
relative severity
seeding offset
log seeding rate, starting strain
log seeding rate, Delta
transmissibility
shared target dispersion
no mobility
Facebook tiles visited mobility
Facebook single tile mobility

Zambia

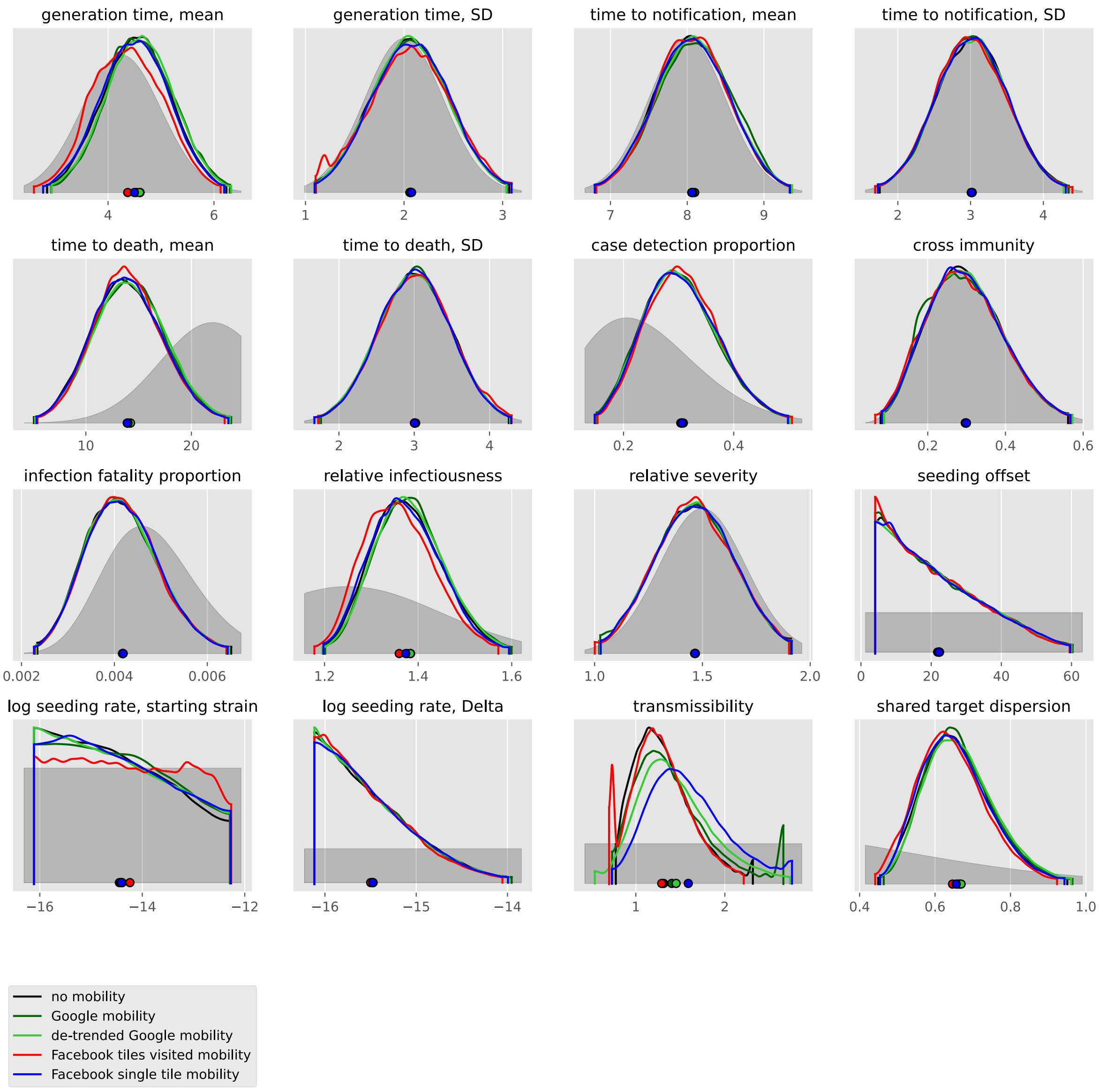

generation time, mean
4
6
generation time, SD
1
2
3
time to notification, mean
7
8
9
time to notification, SD
2
3
4
time to death, mean
10
20
time to death, SD
2
3
4
case detection proportion
0.2
0.4
cross immunity
0.2
0.4
0.6
infection fatality proportion
0.002
0.004
0.006
relative infectiousness
1.2
1.4
1.6
relative severity
1.0
1.5
2.0
seeding offset
0
20
40
60
log seeding rate, starting strain
−16
−14
−12
log seeding rate, Delta
−16
−15
−14
transmissibility
1
2
shared target dispersion
0.4
0.6
0.8
1.0
no mobility
Google mobility
de-trended Google mobility
Facebook tiles visited mobility
Facebook single tile mobility

Zimbabwe

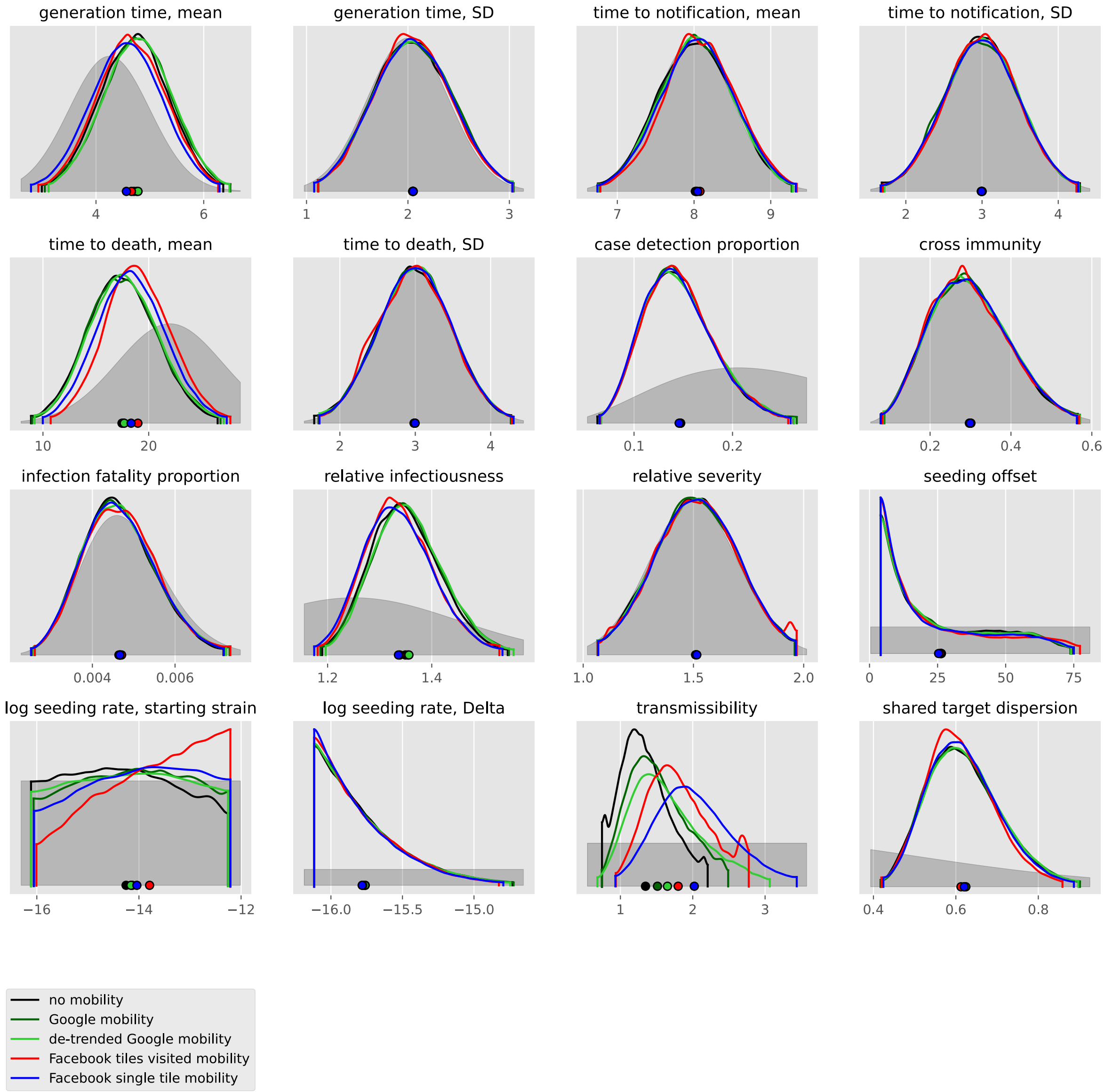

generation time, mean
generation time, SD
time to notification, mean
time to notification, SD
time to death, mean
time to death, SD
case detection proportion
cross immunity
infection fatality proportion
relative infectiousness
relative severity
seeding offset
log seeding rate, starting strain
log seeding rate, Delta
transmissibility
shared target dispersion
no mobility
Google mobility
de-trended Google mobility
Facebook tiles visited mobility
Facebook single tile mobility

Asia

Afghanistan

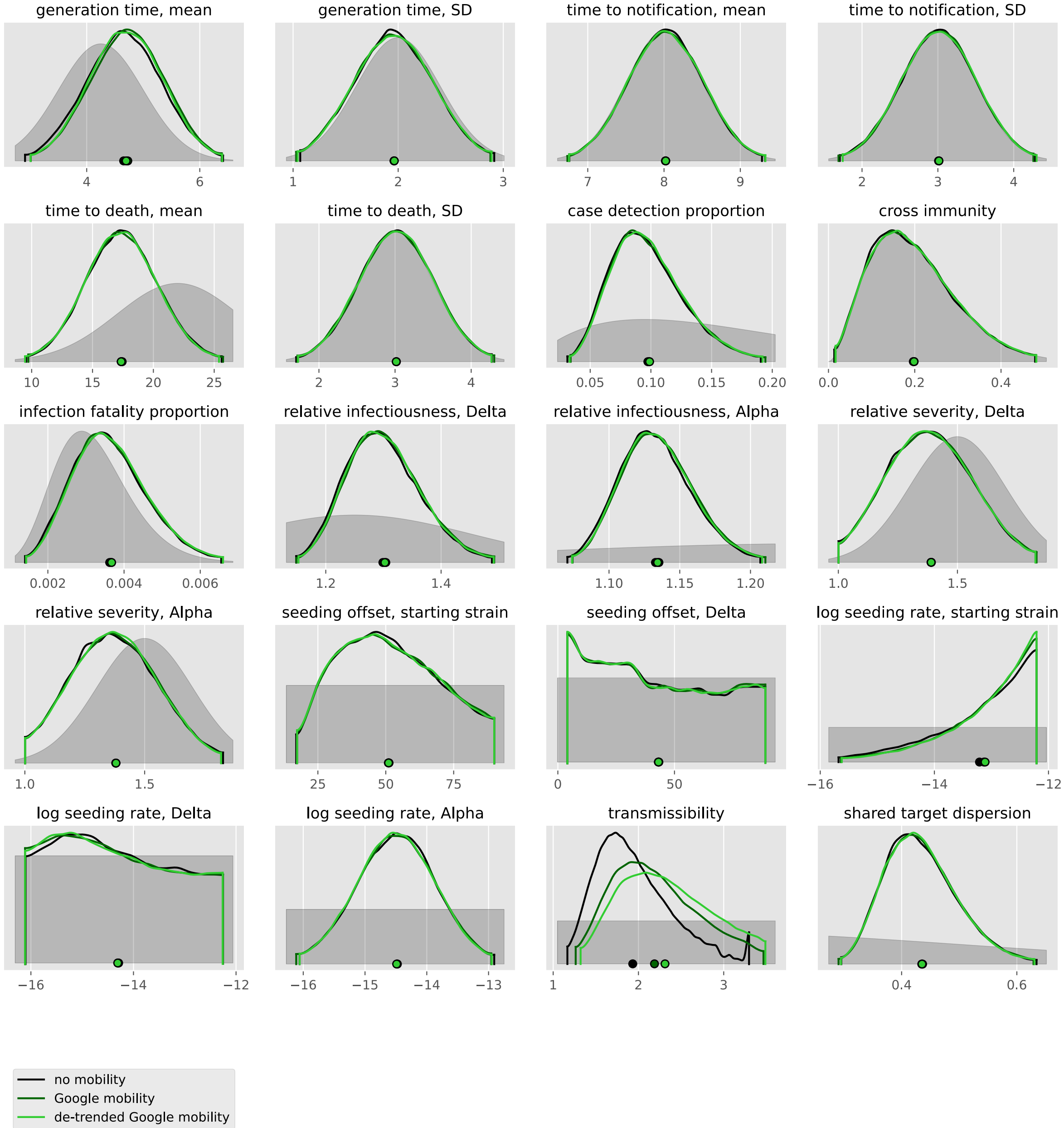

Bahrain

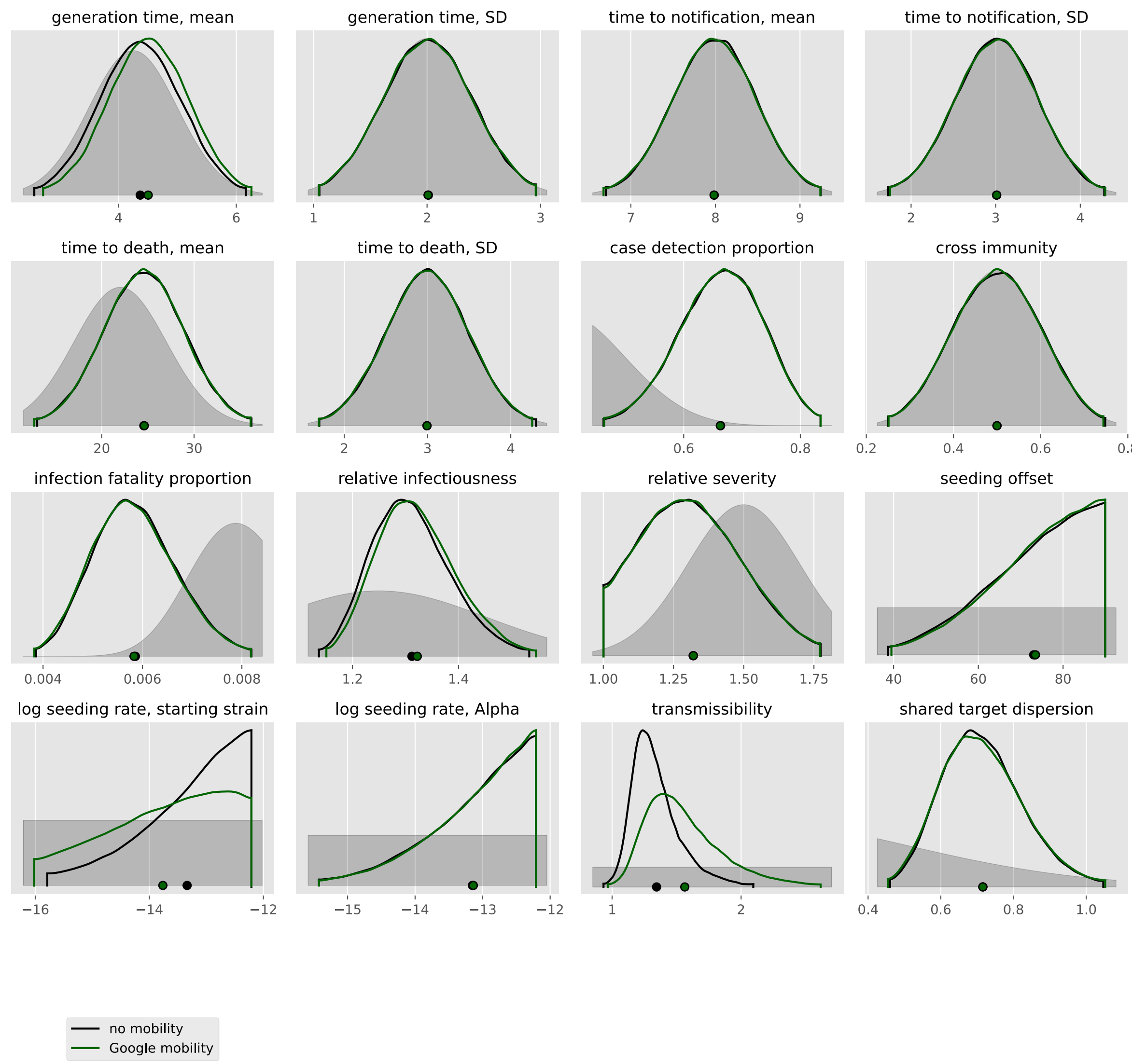

generation time, mean
4
6
generation time, SD
1
2
3
time to notification, mean
7
8
9
time to notification, SD
2
3
4
time to death, mean
20
30
time to death, SD
2
3
4
case detection proportion
0.6
0.8
cross immunity
0.2
0.4
0.6
0.8
infection fatality proportion
0.004
0.006
0.008
relative infectiousness
1.2
1.4
relative severity
1.00
1.25
1.50
1.75
seeding offset
40
60
80
log seeding rate, starting strain
−16
−14
−12
log seeding rate, Alpha
−15
−14
−13
−12
transmissibility
1
2
shared target dispersion
0.4
0.6
0.8
1.0
no mobility
Google mobility

Bangladesh

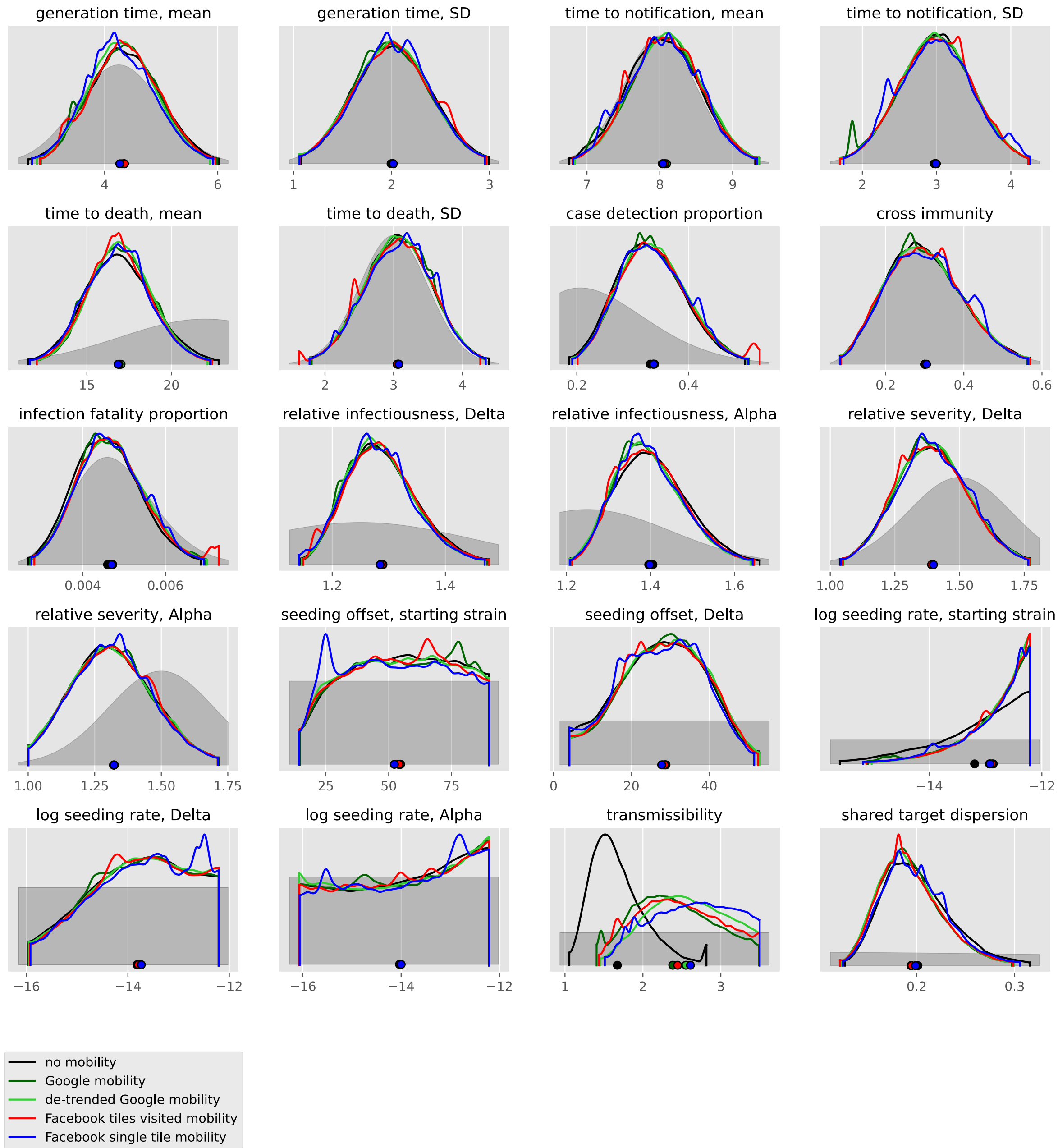
generation time, mean
4
6
generation time, SD
1
2
3
time to notification, mean
7
8
9
time to notification, SD
2
3
4
time to death, mean
15
20
time to death, SD
2
3
4
case detection proportion
0.2
0.4
cross immunity
0.2
0.4
0.6
infection fatality proportion
0.004
0.006
relative infectiousness, Delta
1.2
1.4
relative infectiousness, Alpha
1.2
1.4
1.6
relative severity, Delta
1.00
1.25
1.50
1.75
relative severity, Alpha
1.00
1.25
1.50
1.75
seeding offset, starting strain
25
50
75
seeding offset, Delta
0
20
40
log seeding rate, starting strain
−14
−12
log seeding rate, Delta
−16
−14
−12
log seeding rate, Alpha
−16
−14
−12
transmissibility
1
2
3
shared target dispersion
0.2
0.3
no mobility
Google mobility
de-trended Google mobility
Facebook tiles visited mobility
Facebook single tile mobility

Georgia

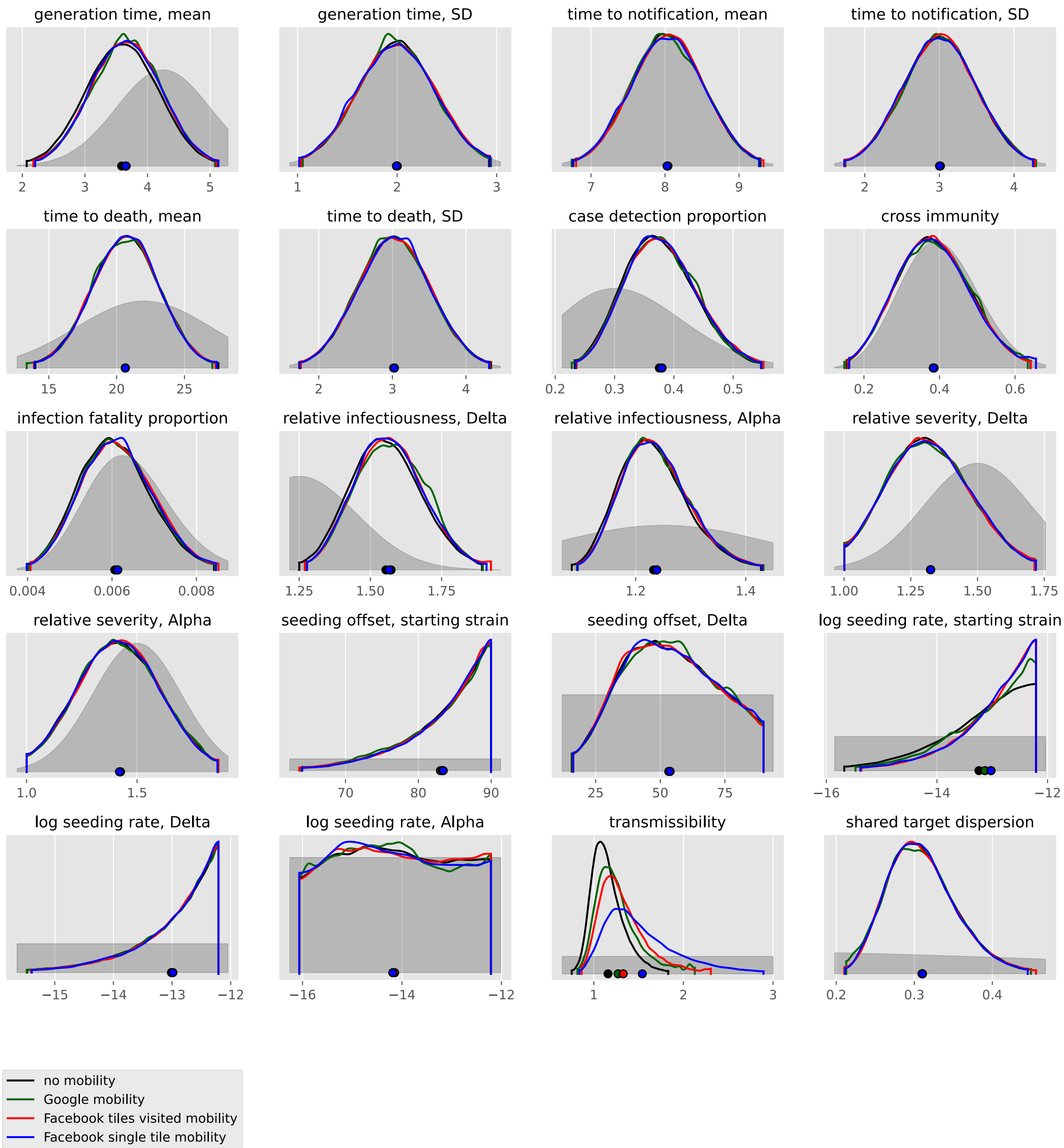

generation time, mean
generation time, SD
time to notification, mean
time to notification, SD
time to death, mean
time to death, SD
case detection proportion
cross immunity
infection fatality proportion
relative infectiousness, Delta
relative infectiousness, Alpha
relative severity, Delta
relative severity, Alpha
seeding offset, starting strain
seeding offset, Delta
log seeding rate, starting strain
log seeding rate, Delta
log seeding rate, Alpha
transmissibility
shared target dispersion
no mobility
Google mobility
Facebook tiles visited mobility
Facebook single tile mobility

India

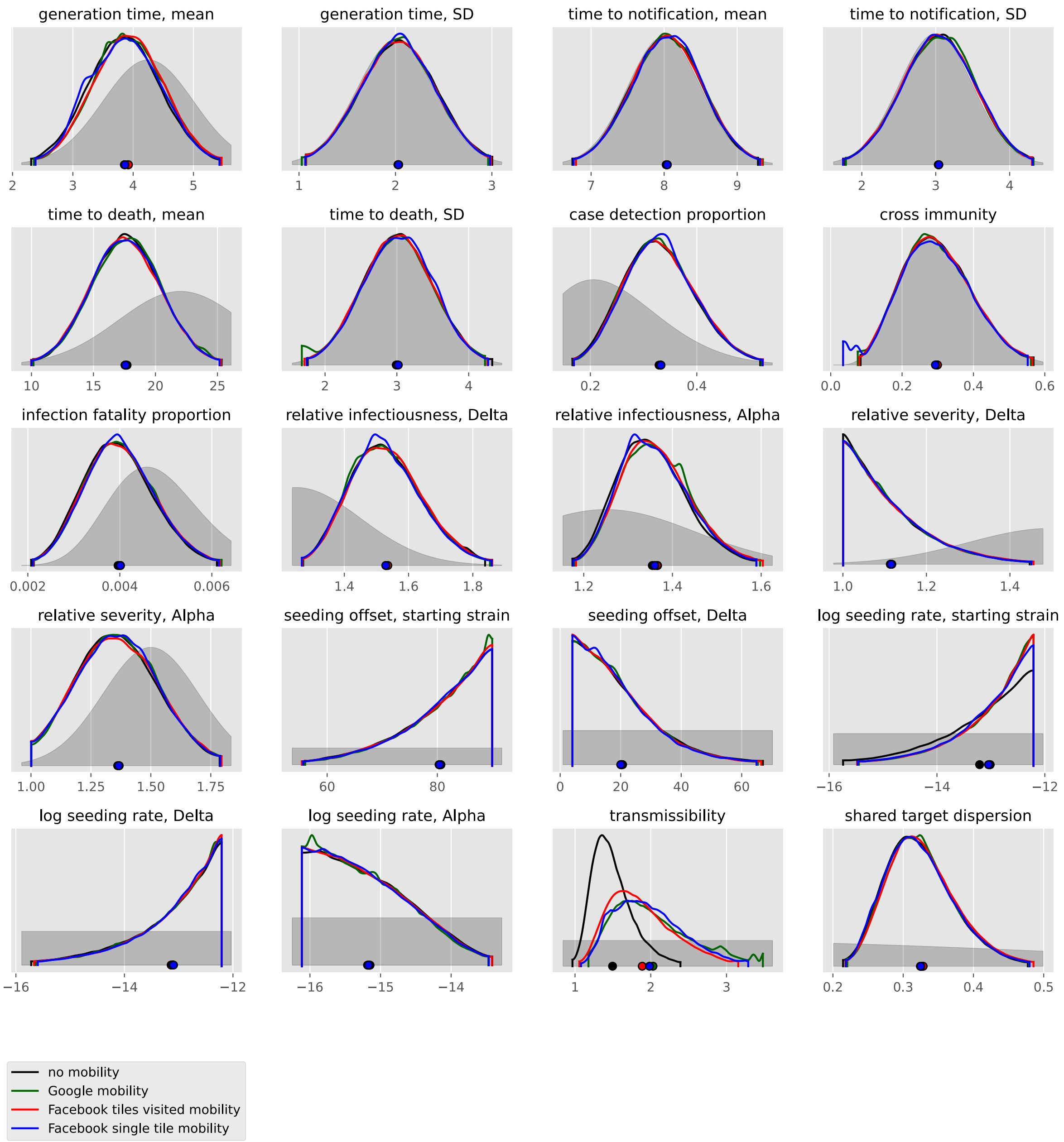
generation time, mean
2 3 4 5
generation time, SD
1 2 3
time to notification, mean
7 8 9
time to notification, SD
2 3 4
time to death, mean
10 15 20 25
time to death, SD
2 3 4
case detection proportion
0.2 0.4
cross immunity
0.0 0.2 0.4 0.6
infection fatality proportion
0.002 0.004 0.006
relative infectiousness, Delta
1.4 1.6 1.8
relative infectiousness, Alpha
1.2 1.4 1.6
relative severity, Delta
1.0 1.2 1.4
relative severity, Alpha
1.00 1.25 1.50 1.75
seeding offset, starting strain
60 80
seeding offset, Delta
0 20 40 60
log seeding rate, starting strain
−16 −14 −12
log seeding rate, Delta
−16 −14 −12
log seeding rate, Alpha
−16 −15 −14
transmissibility
1 2 3
shared target dispersion
0.2 0.3 0.4 0.5
no mobility
Google mobility
Facebook tiles visited mobility
Facebook single tile mobility

Indonesia

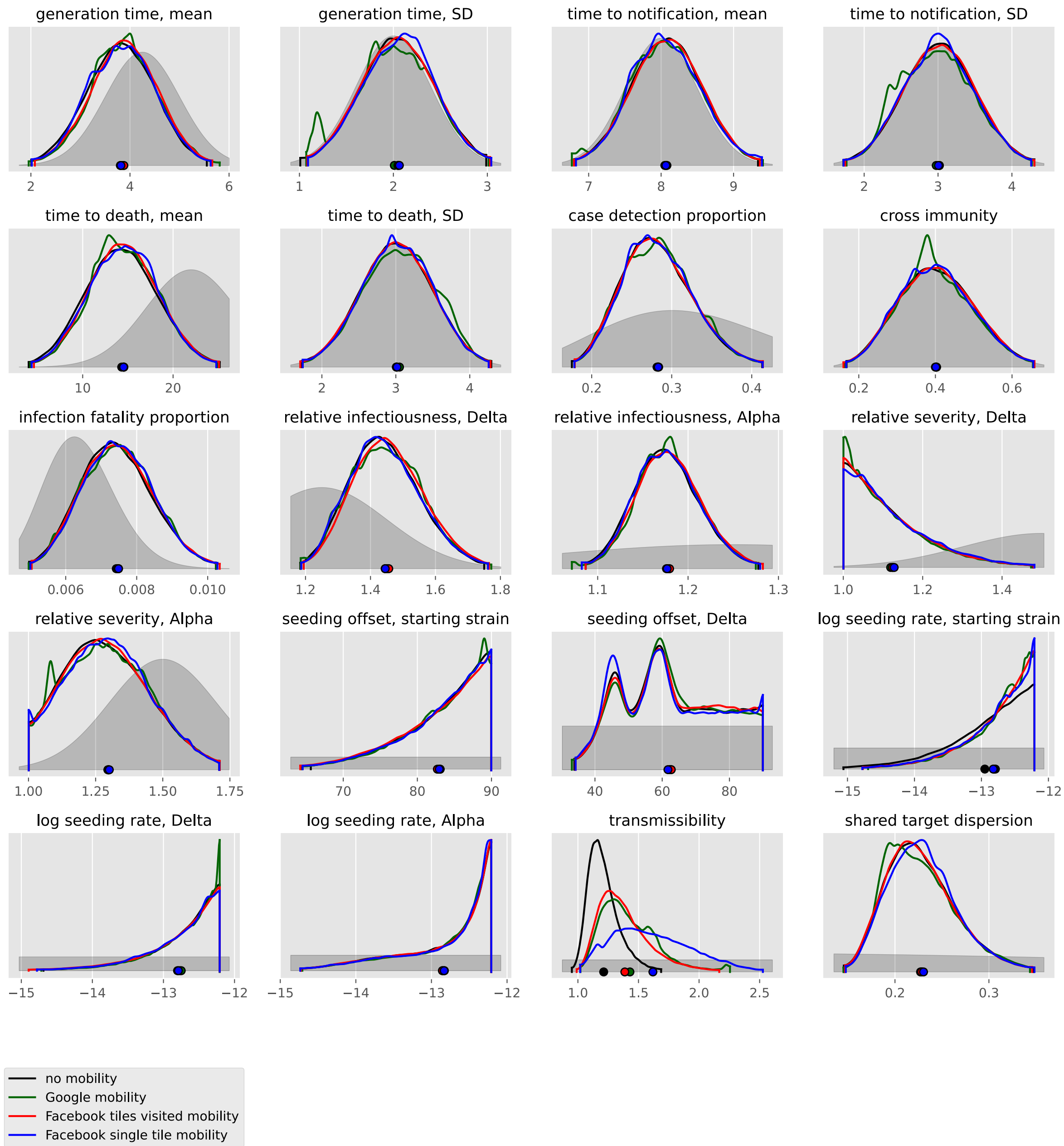
generation time, mean
generation time, SD
time to notification, mean
time to notification, SD
time to death, mean
time to death, SD
case detection proportion
cross immunity
infection fatality proportion
relative infectiousness, Delta
relative infectiousness, Alpha
relative severity, Delta
relative severity, Alpha
seeding offset, starting strain
seeding offset, Delta
log seeding rate, starting strain
log seeding rate, Delta
log seeding rate, Alpha
transmissibility
shared target dispersion
no mobility
Google mobility
Facebook tiles visited mobility
Facebook single tile mobility

Iraq

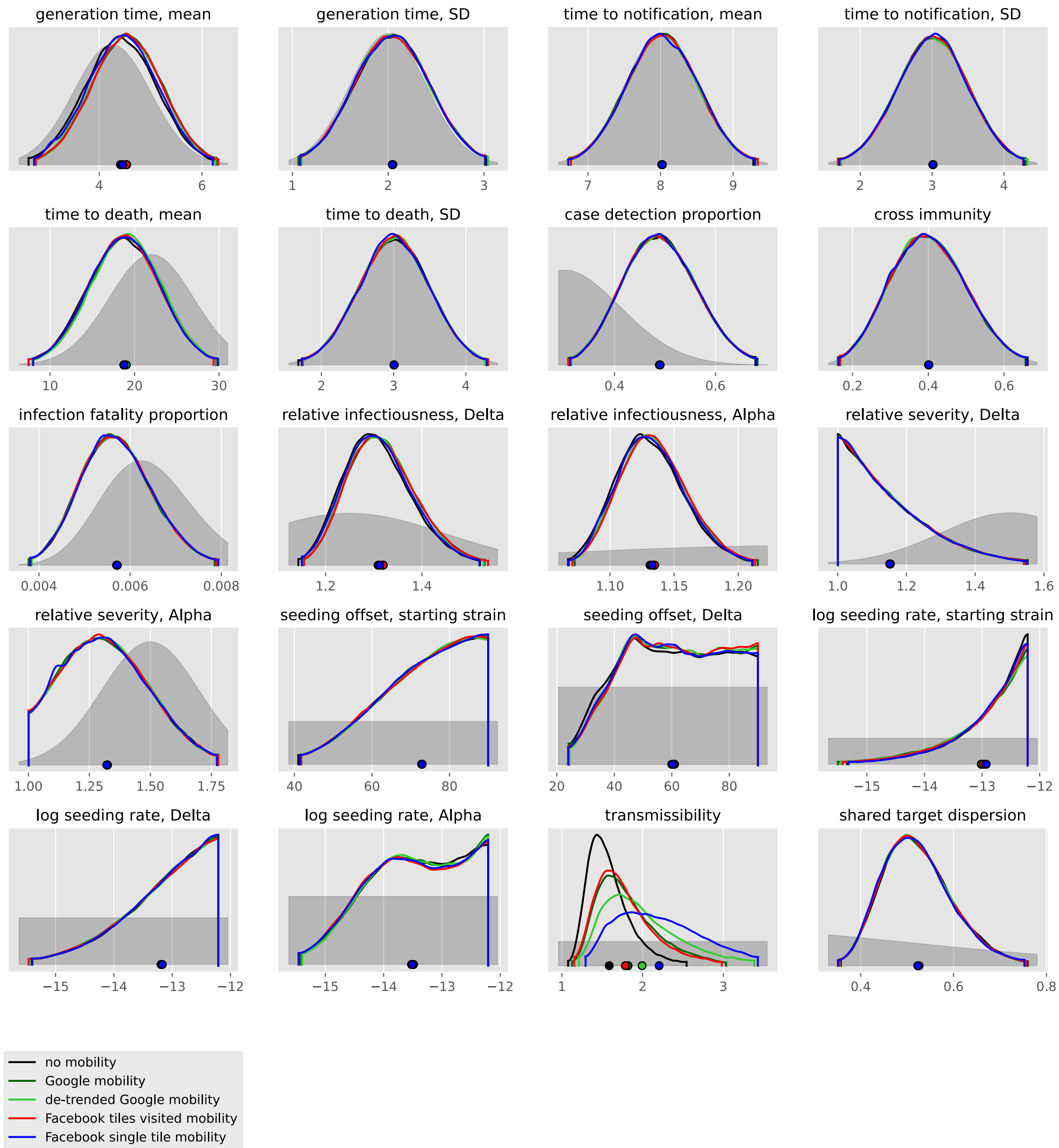

generation time, mean
generation time, SD
time to notification, mean
time to notification, SD
time to death, mean
time to death, SD
case detection proportion
cross immunity
infection fatality proportion
relative infectiousness, Delta
relative infectiousness, Alpha
relative severity, Delta
relative severity, Alpha
seeding offset, starting strain
seeding offset, Delta
log seeding rate, starting strain
log seeding rate, Delta
log seeding rate, Alpha
transmissibility
shared target dispersion
no mobility
Google mobility
de-trended Google mobility
Facebook tiles visited mobility
Facebook single tile mobility

Israel

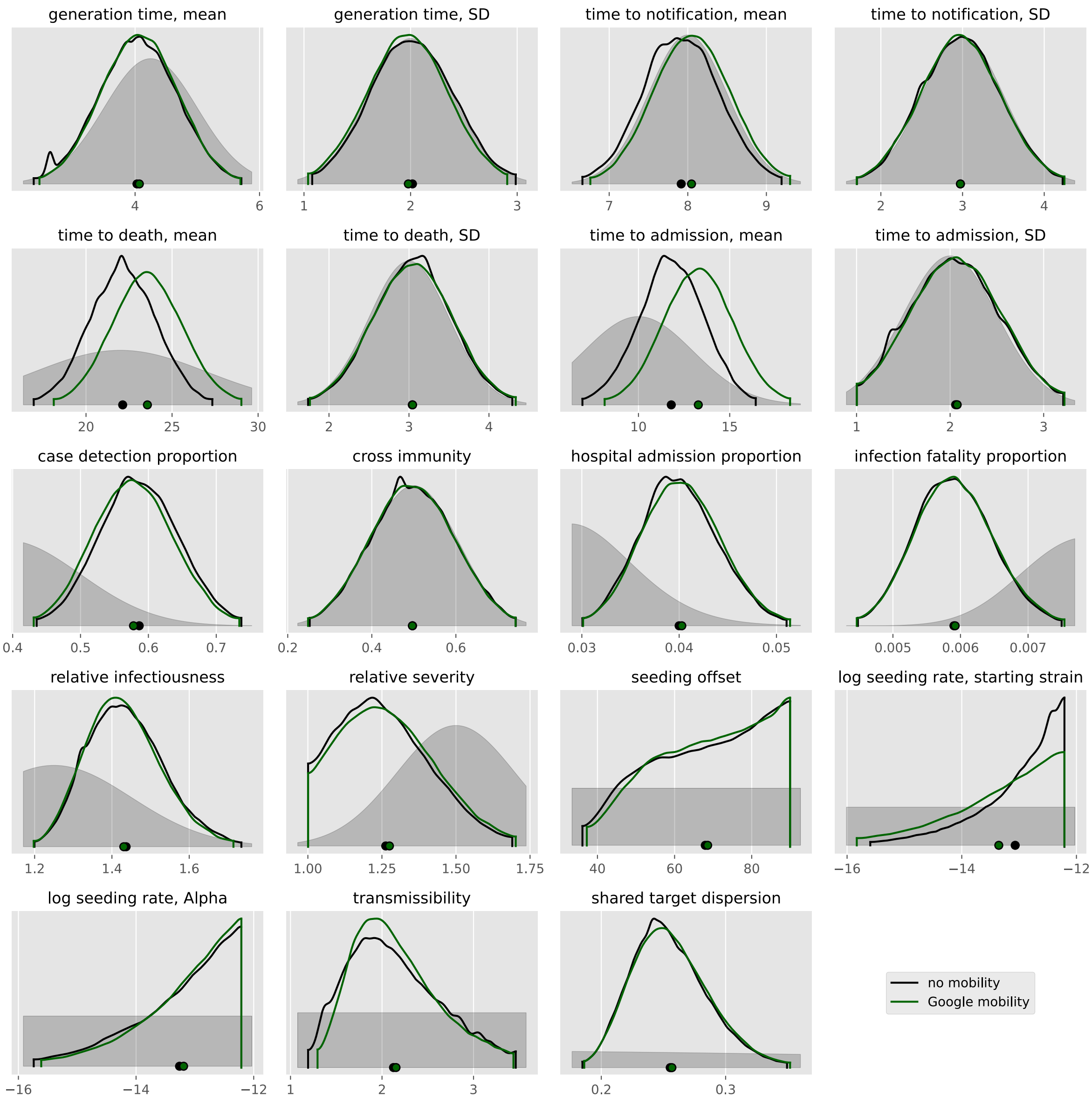
generation time, mean
generation time, SD
time to notification, mean
time to notification, SD
time to death, mean
time to death, SD
time to admission, mean
time to admission, SD
case detection proportion
cross immunity
hospital admission proportion
infection fatality proportion
relative infectiousness
relative severity
seeding offset
log seeding rate, starting strain
log seeding rate, Alpha
transmissibility
shared target dispersion
no mobility
Google mobility

Japan

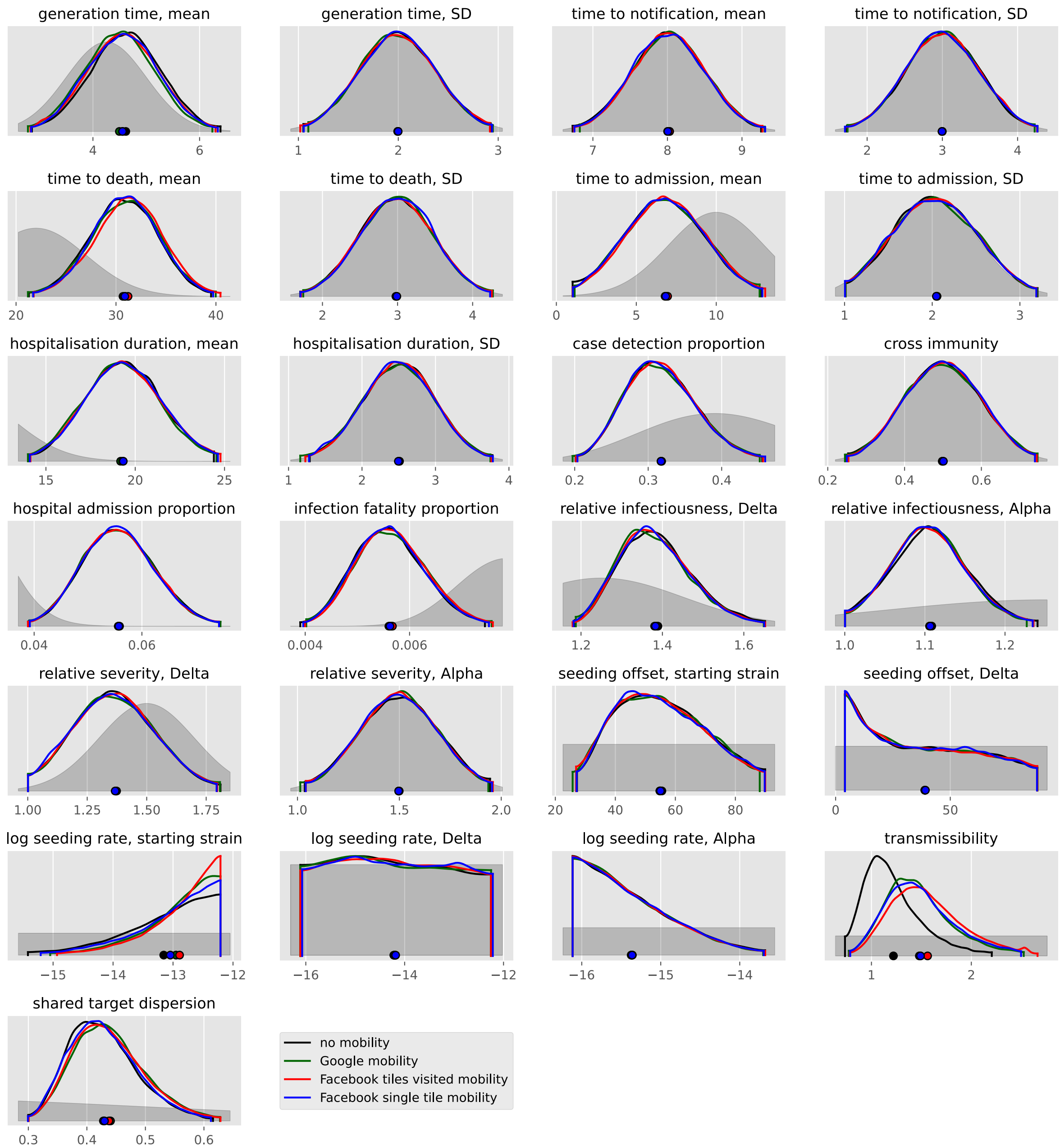
generation time, mean
generation time, SD
time to notification, mean
time to notification, SD
time to death, mean
time to death, SD
time to admission, mean
time to admission, SD
hospitalisation duration, mean
hospitalisation duration, SD
case detection proportion
cross immunity
hospital admission proportion
infection fatality proportion
relative infectiousness, Delta
relative infectiousness, Alpha
relative severity, Delta
relative severity, Alpha
seeding offset, starting strain
seeding offset, Delta
log seeding rate, starting strain
log seeding rate, Delta
log seeding rate, Alpha
transmissibility
shared target dispersion
no mobility
Google mobility
Facebook tiles visited mobility
Facebook single tile mobility

Jordan

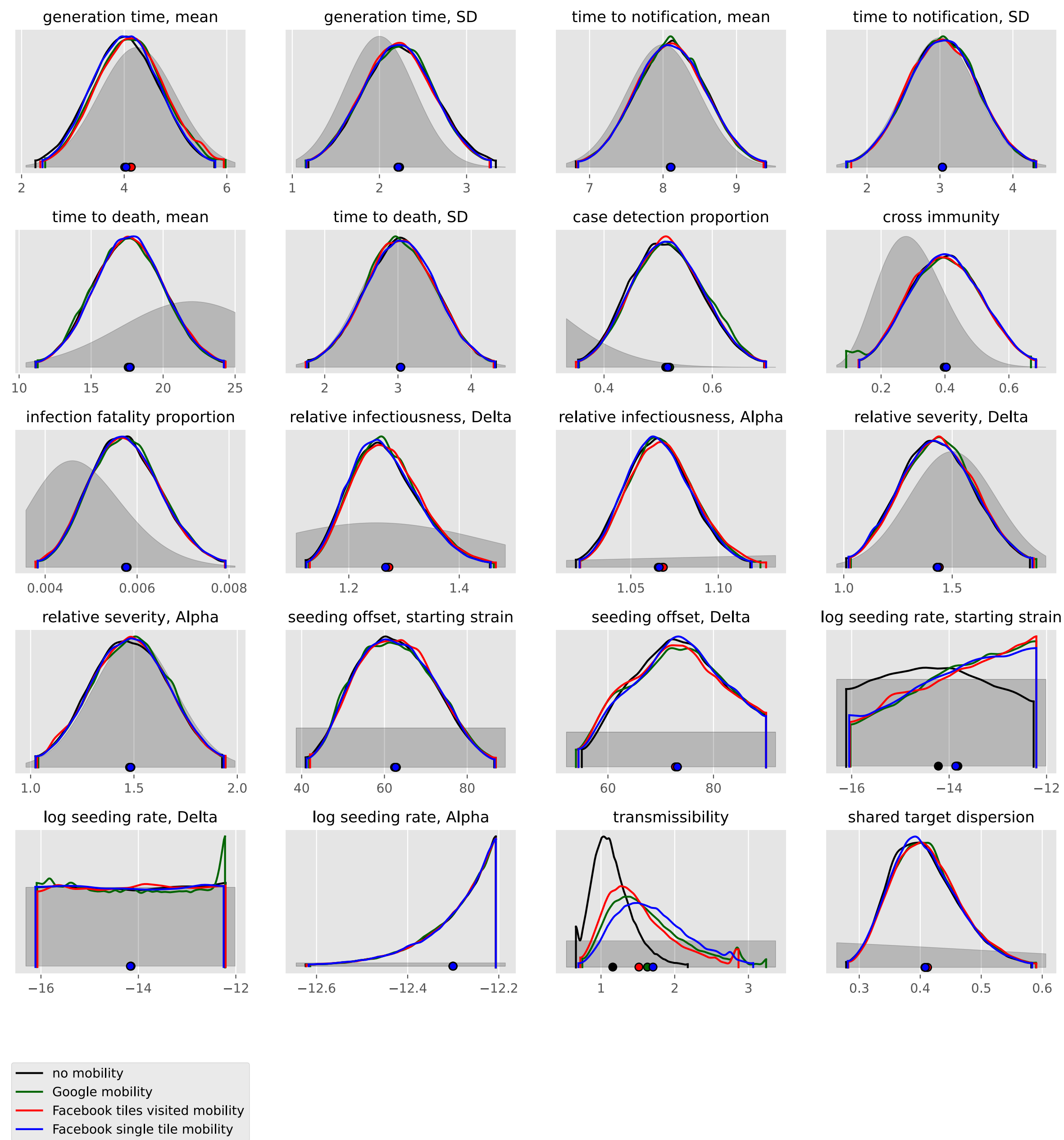
generation time, mean
generation time, SD
time to notification, mean
time to notification, SD
time to death, mean
time to death, SD
case detection proportion
cross immunity
infection fatality proportion
relative infectiousness, Delta
relative infectiousness, Alpha
relative severity, Delta
relative severity, Alpha
seeding offset, starting strain
seeding offset, Delta
log seeding rate, starting strain
log seeding rate, Delta
log seeding rate, Alpha
transmissibility
shared target dispersion
no mobility
Google mobility
Facebook tiles visited mobility
Facebook single tile mobility

Korea, Republic of

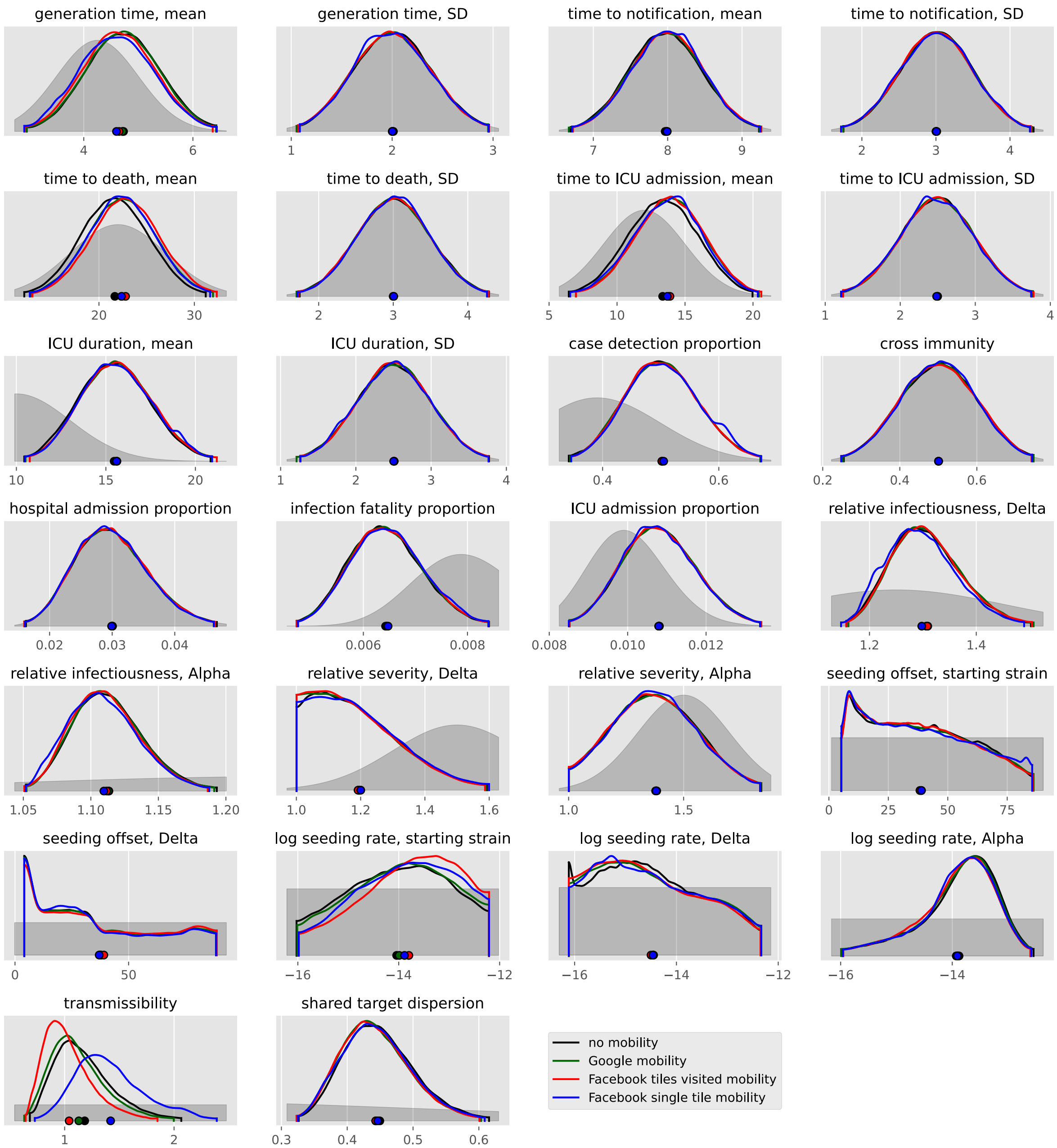

Kuwait

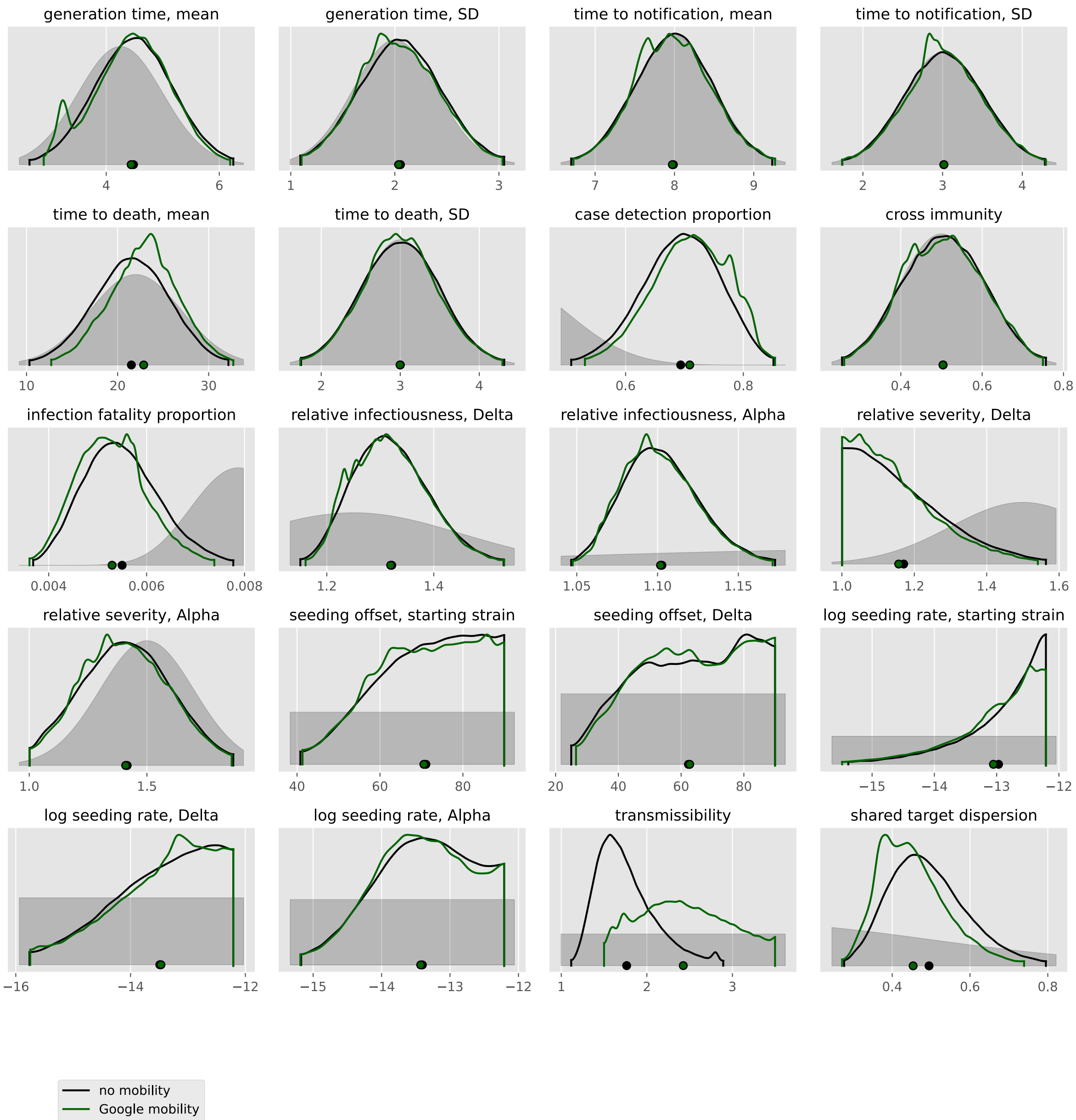

generation time, mean
generation time, SD
time to notification, mean
time to notification, SD
time to death, mean
time to death, SD
case detection proportion
cross immunity
infection fatality proportion
relative infectiousness, Delta
relative infectiousness, Alpha
relative severity, Delta
relative severity, Alpha
seeding offset, starting strain
seeding offset, Delta
log seeding rate, starting strain
log seeding rate, Delta
log seeding rate, Alpha
transmissibility
shared target dispersion
no mobility
Google mobility

Lebanon

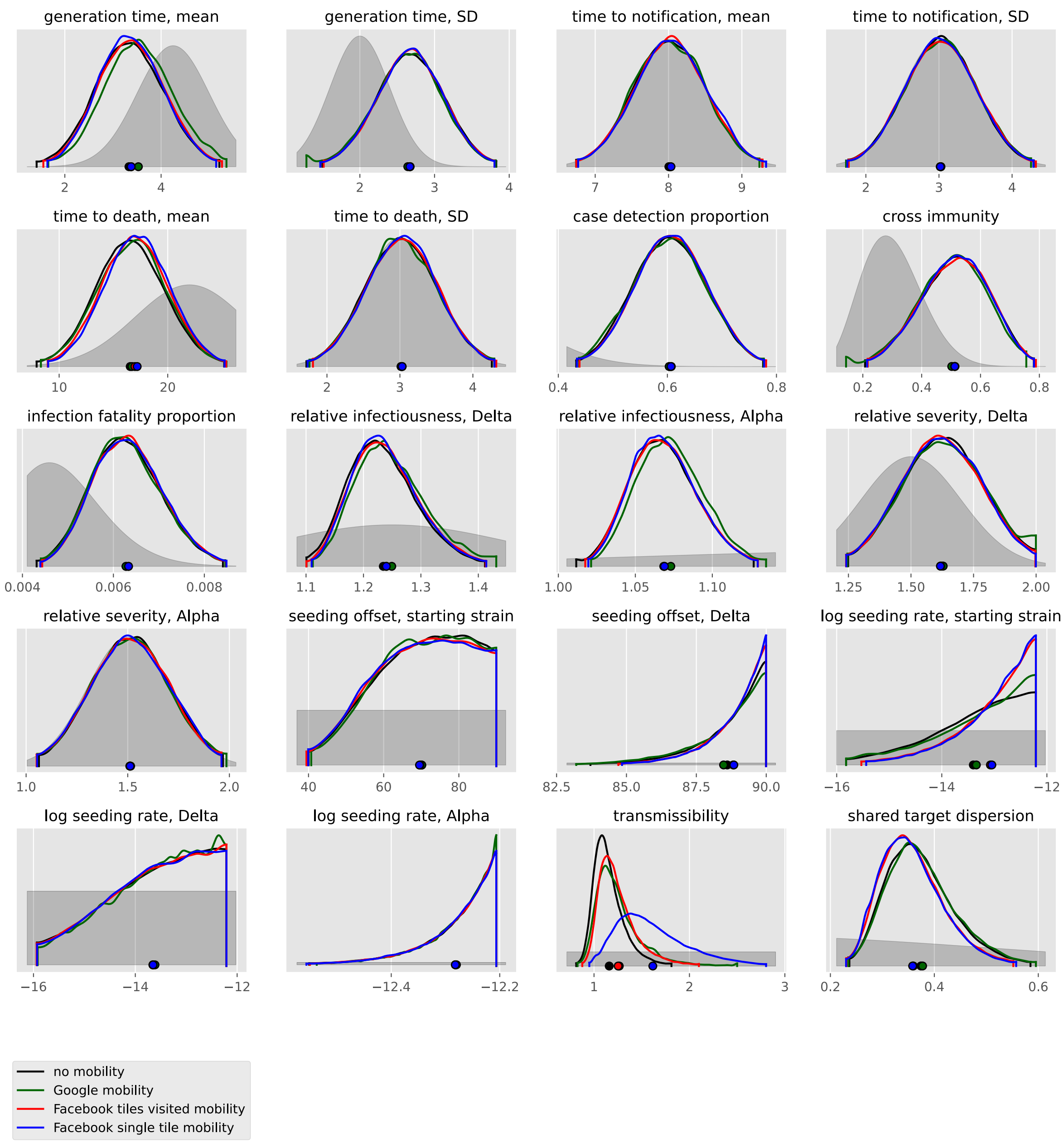

generation time, mean
generation time, SD
time to notification, mean
time to notification, SD
time to death, mean
time to death, SD
case detection proportion
cross immunity
infection fatality proportion
relative infectiousness, Delta
relative infectiousness, Alpha
relative severity, Delta
relative severity, Alpha
seeding offset, starting strain
seeding offset, Delta
log seeding rate, starting strain
log seeding rate, Delta
log seeding rate, Alpha
transmissibility
shared target dispersion
no mobility
Google mobility
Facebook tiles visited mobility
Facebook single tile mobility

Malaysia

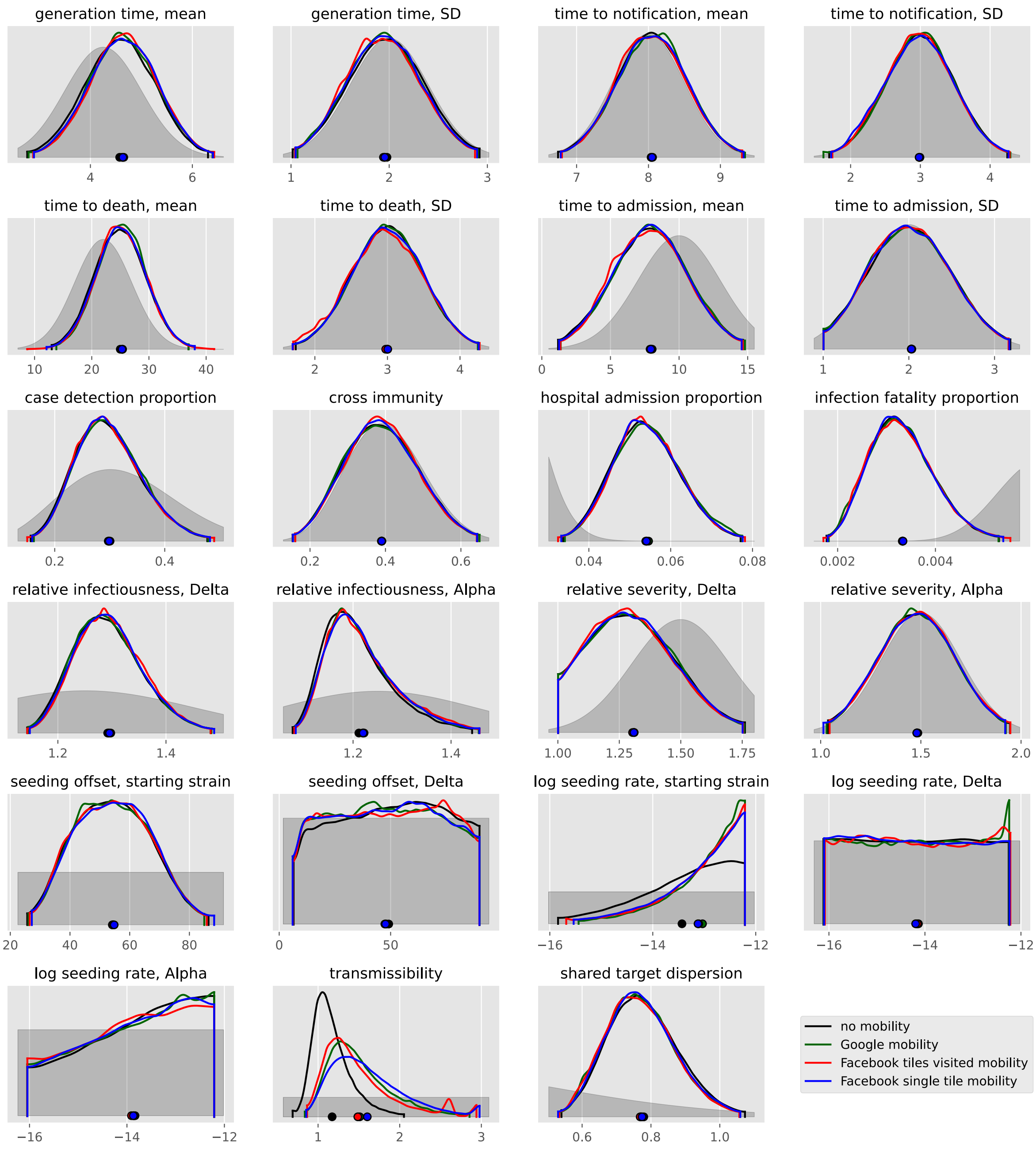
generation time, mean
generation time, SD
time to notification, mean
time to notification, SD
time to death, mean
time to death, SD
time to admission, mean
time to admission, SD
case detection proportion
cross immunity
hospital admission proportion
infection fatality proportion
relative infectiousness, Delta
relative infectiousness, Alpha
relative severity, Delta
relative severity, Alpha
seeding offset, starting strain
seeding offset, Delta
log seeding rate, starting strain
log seeding rate, Delta
log seeding rate, Alpha
transmissibility
shared target dispersion
no mobility
Google mobility
Facebook tiles visited mobility
Facebook single tile mobility

Nepal

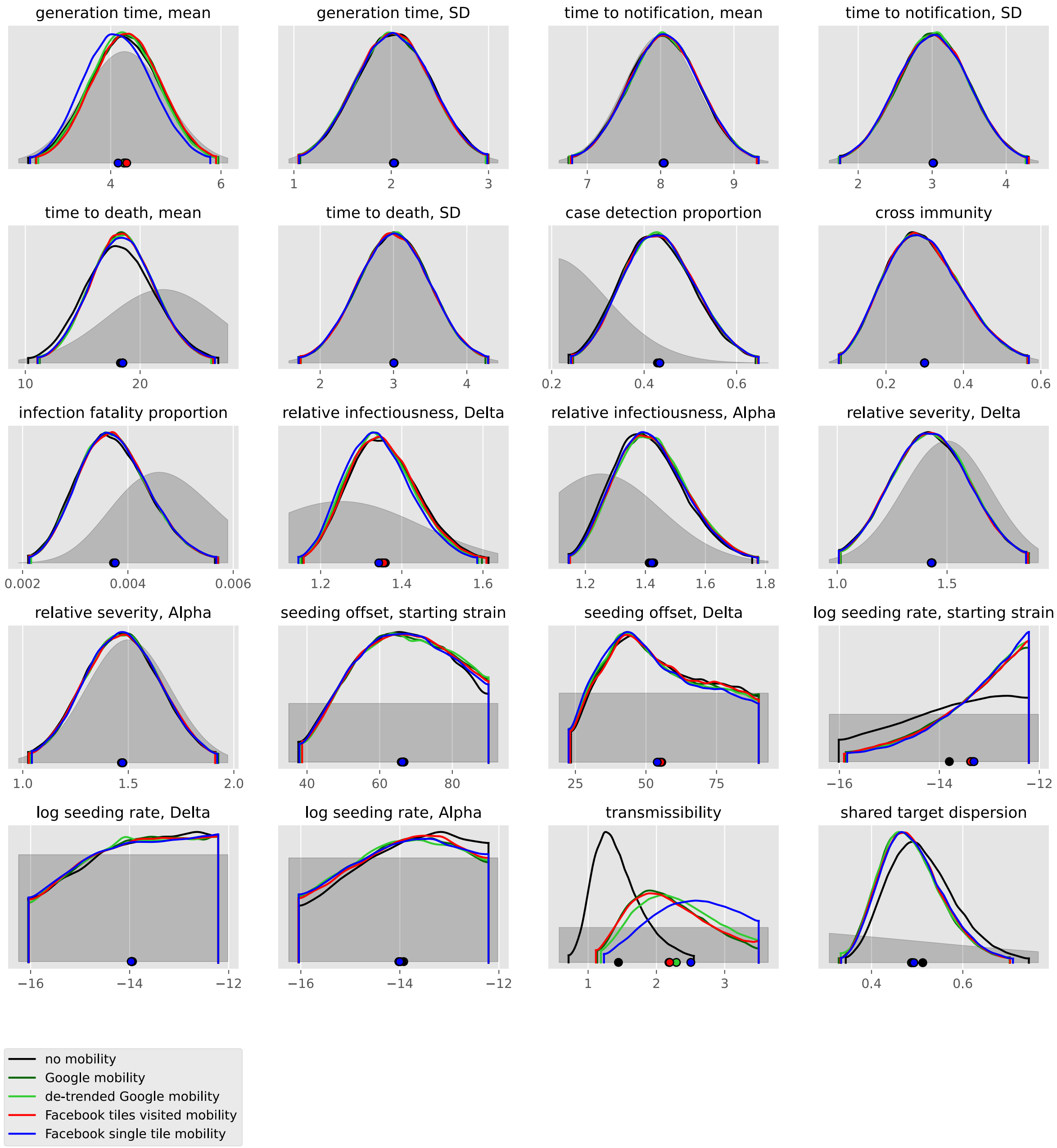

generation time, mean
generation time, SD
time to notification, mean
time to notification, SD
time to death, mean
time to death, SD
case detection proportion
cross immunity
infection fatality proportion
relative infectiousness, Delta
relative infectiousness, Alpha
relative severity, Delta
relative severity, Alpha
seeding offset, starting strain
seeding offset, Delta
log seeding rate, starting strain
log seeding rate, Delta
log seeding rate, Alpha
transmissibility
shared target dispersion
no mobility
Google mobility
de-trended Google mobility
Facebook tiles visited mobility
Facebook single tile mobility

Oman

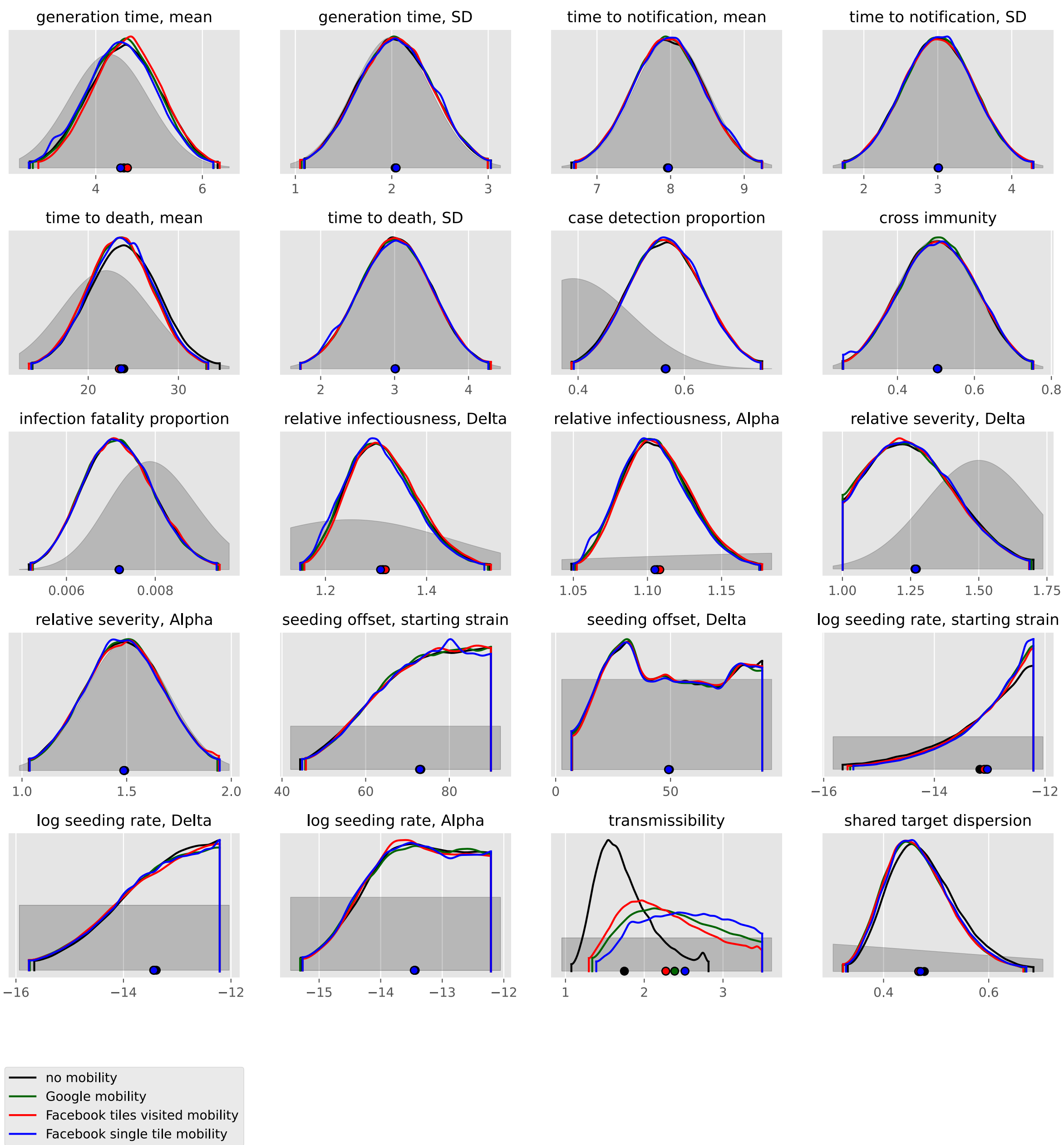
generation time, mean
generation time, SD
time to notification, mean
time to notification, SD
time to death, mean
time to death, SD
case detection proportion
cross immunity
infection fatality proportion
relative infectiousness, Delta
relative infectiousness, Alpha
relative severity, Delta
relative severity, Alpha
seeding offset, starting strain
seeding offset, Delta
log seeding rate, starting strain
log seeding rate, Delta
log seeding rate, Alpha
transmissibility
shared target dispersion
no mobility
Google mobility
Facebook tiles visited mobility
Facebook single tile mobility

Pakistan

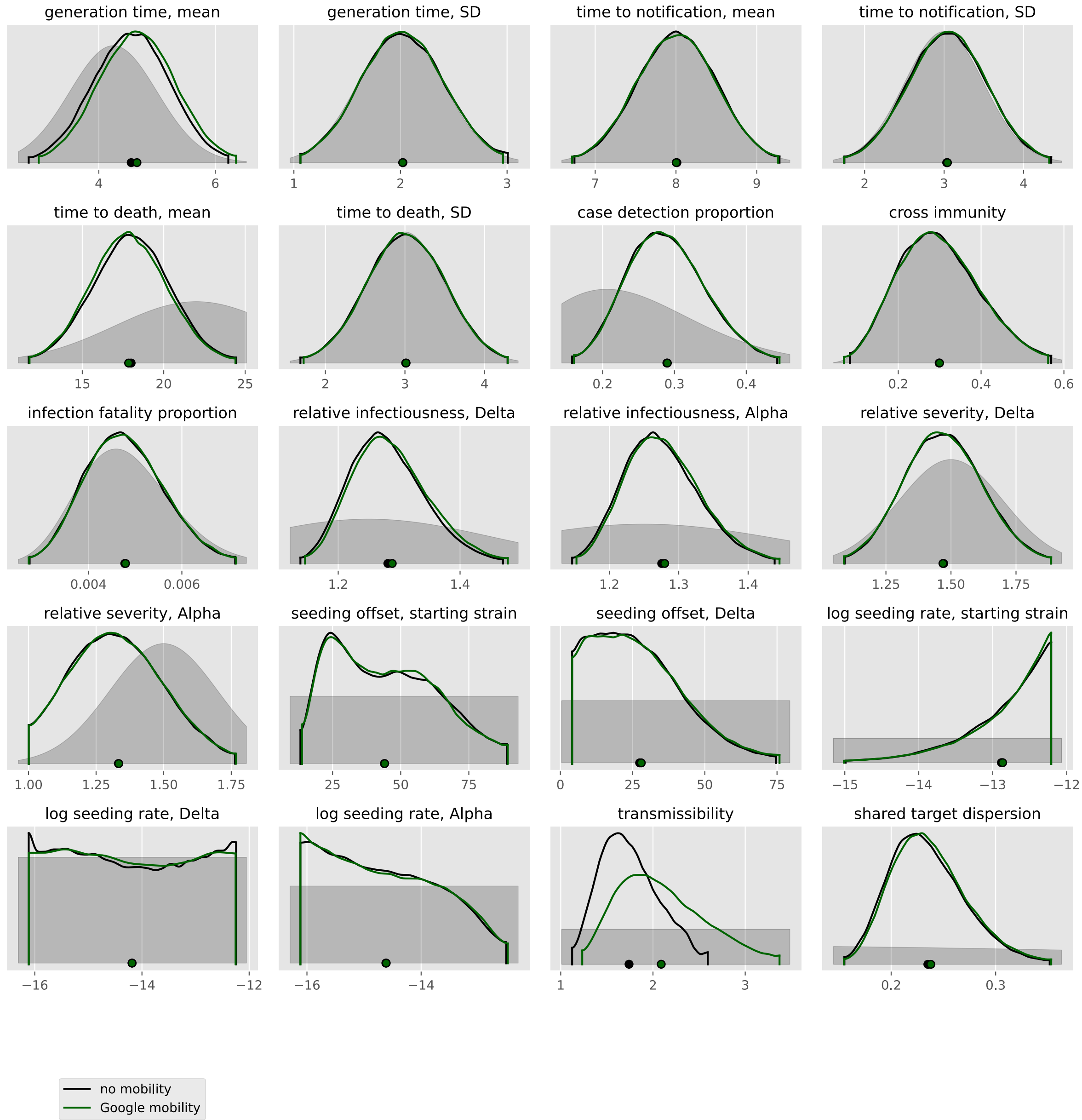

generation time, mean
generation time, SD
time to notification, mean
time to notification, SD
time to death, mean
time to death, SD
case detection proportion
cross immunity
infection fatality proportion
relative infectiousness, Delta
relative infectiousness, Alpha
relative severity, Delta
relative severity, Alpha
seeding offset, starting strain
seeding offset, Delta
log seeding rate, starting strain
log seeding rate, Delta
log seeding rate, Alpha
transmissibility
shared target dispersion
no mobility
Google mobility

Philippines

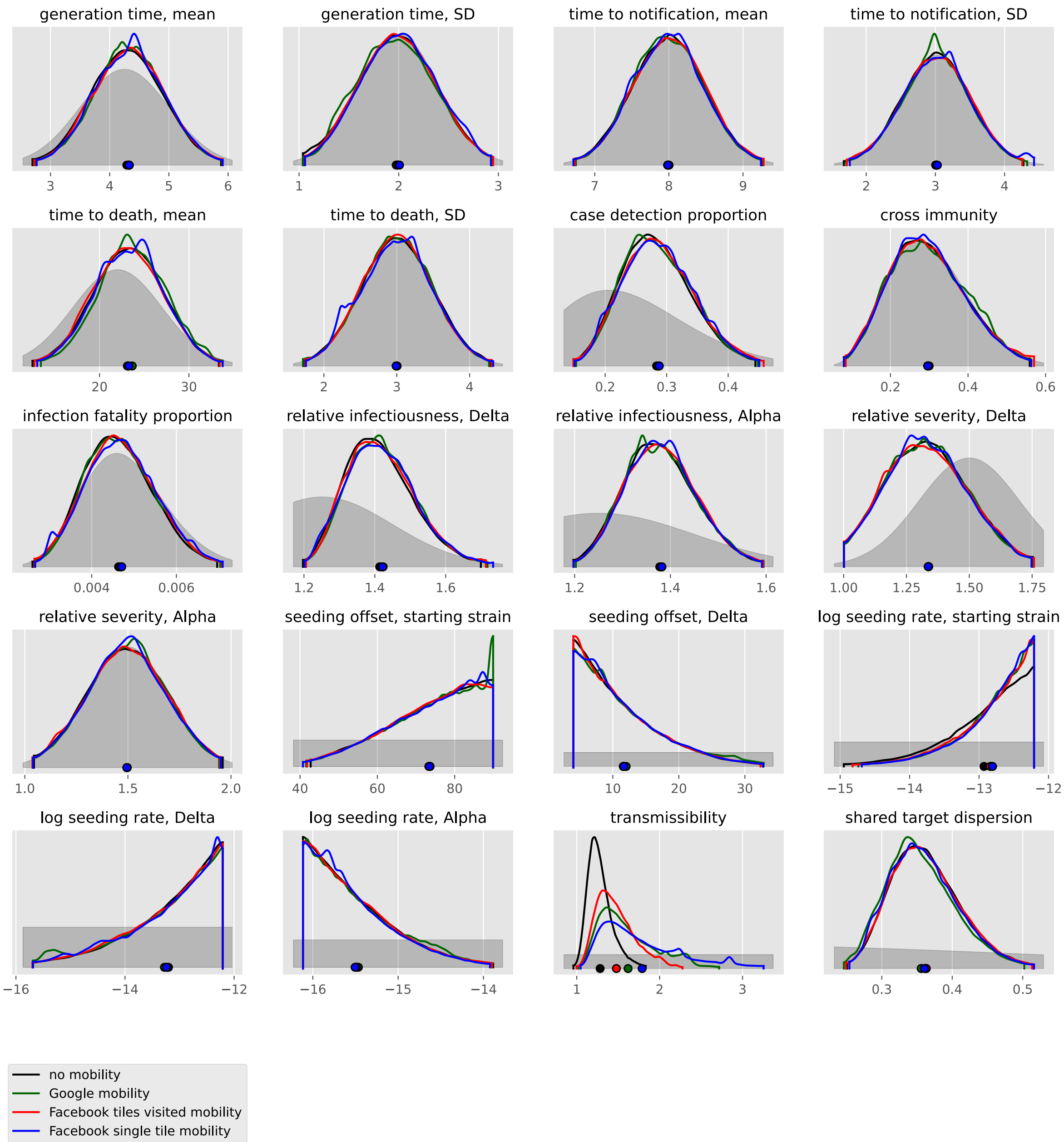

Qatar

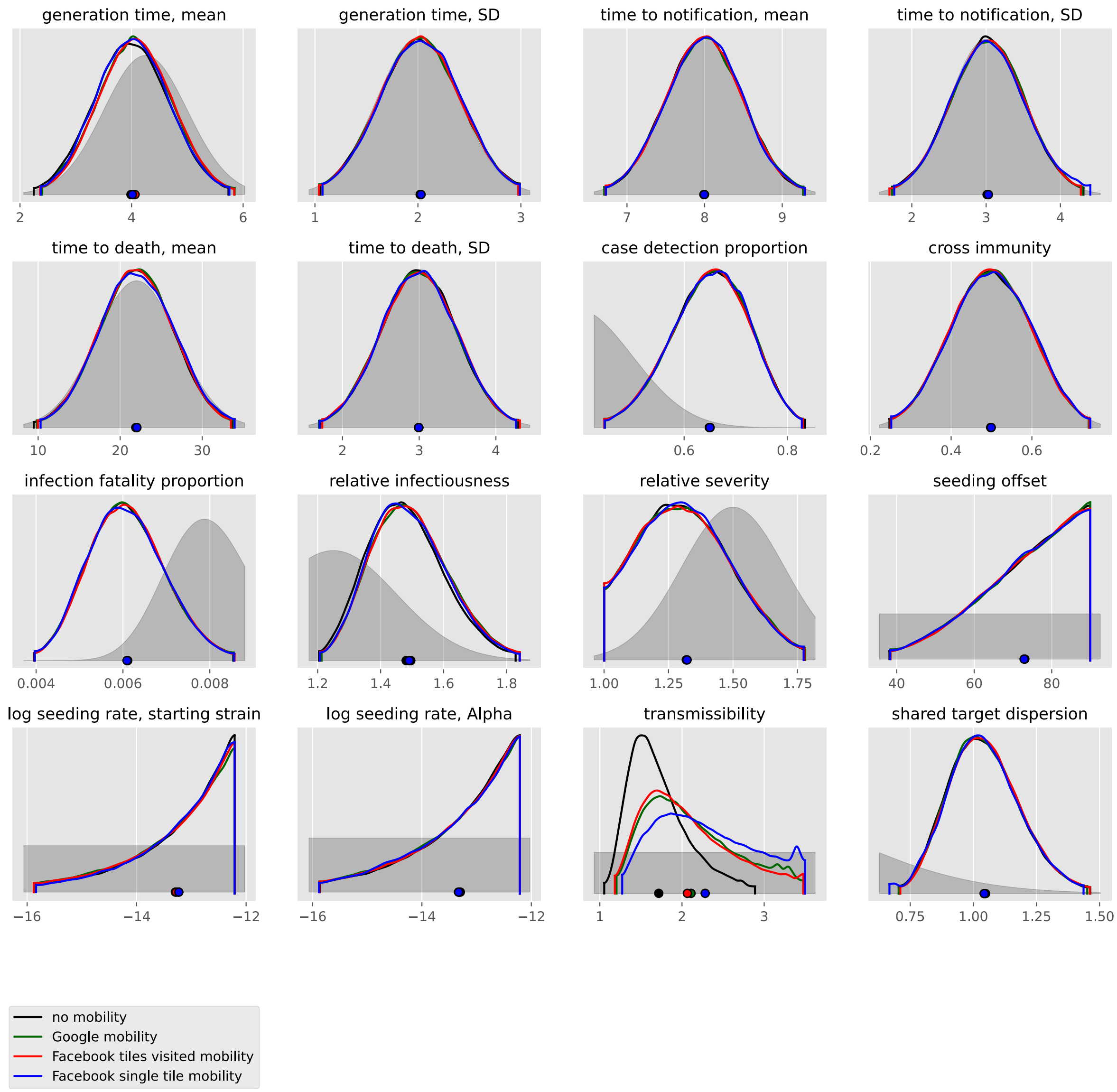
generation time, mean
generation time, SD
time to notification, mean
time to notification, SD
time to death, mean
time to death, SD
case detection proportion
cross immunity
infection fatality proportion
relative infectiousness
relative severity
seeding offset
log seeding rate, starting strain
log seeding rate, Alpha
transmissibility
shared target dispersion
no mobility
Google mobility
Facebook tiles visited mobility
Facebook single tile mobility

Saudi Arabia

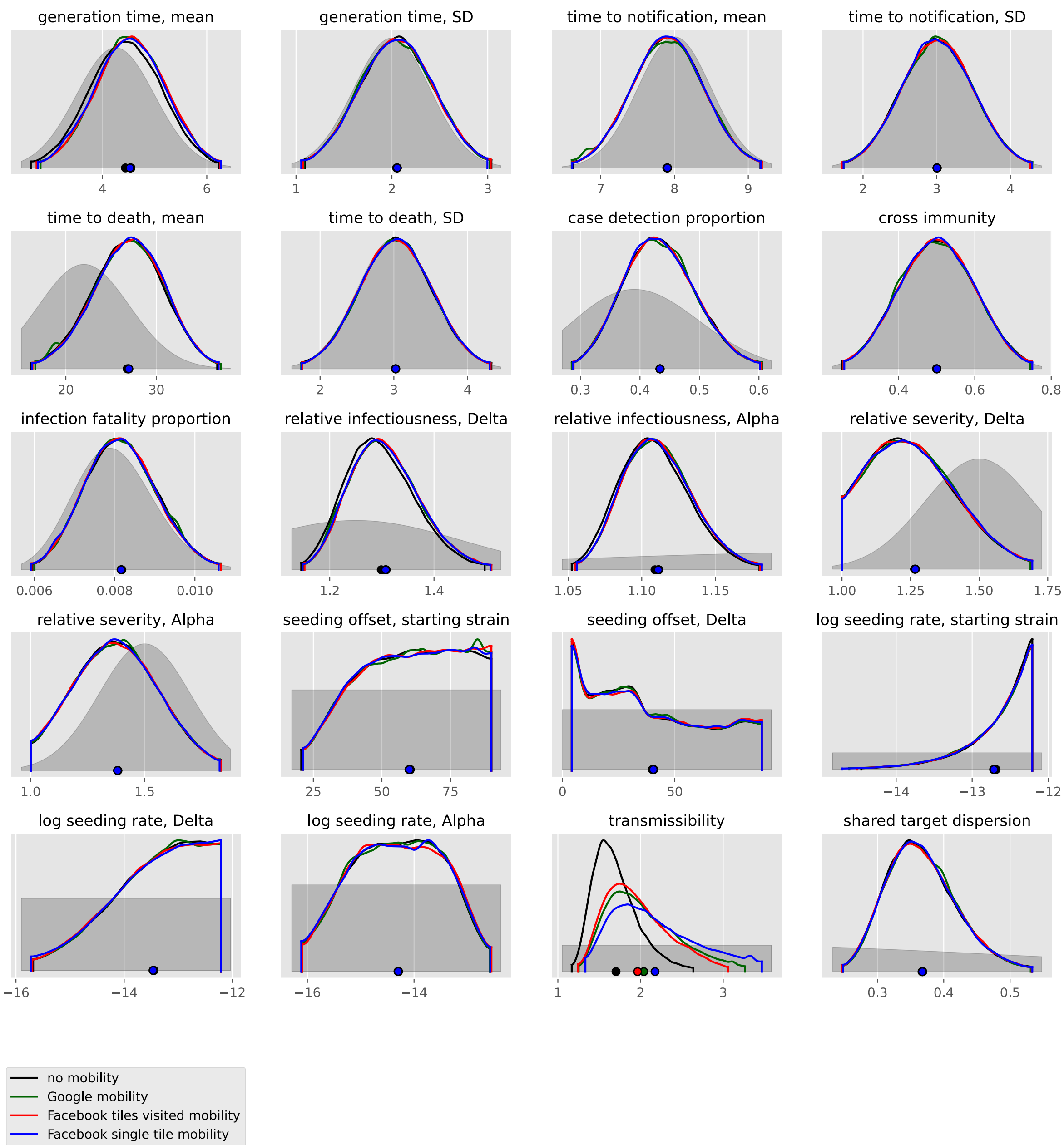

Sri Lanka

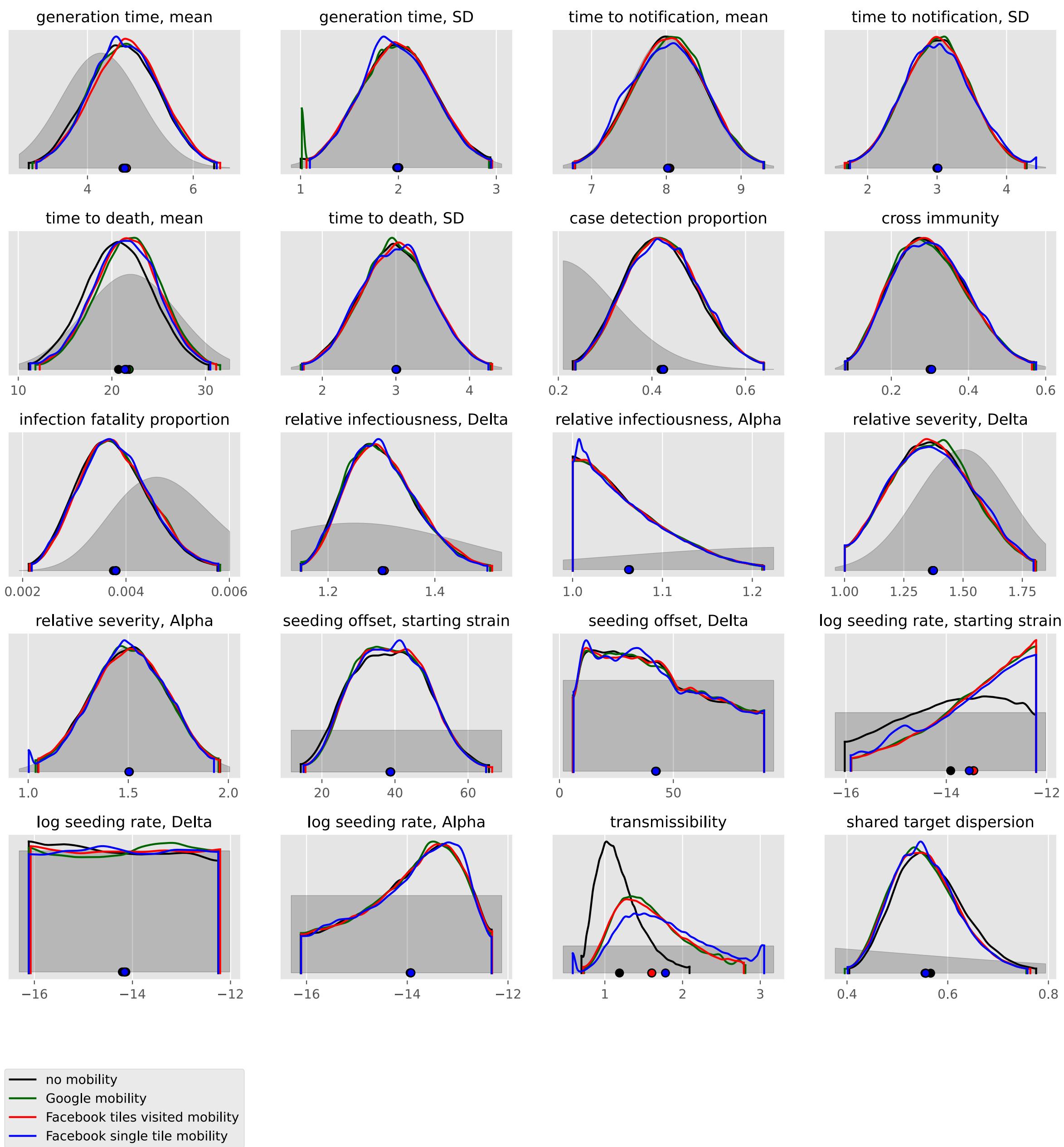
generation time, mean
generation time, SD
time to notification, mean
time to notification, SD
time to death, mean
time to death, SD
case detection proportion
cross immunity
infection fatality proportion
relative infectiousness, Delta
relative infectiousness, Alpha
relative severity, Delta
relative severity, Alpha
seeding offset, starting strain
seeding offset, Delta
log seeding rate, starting strain
log seeding rate, Delta
log seeding rate, Alpha
transmissibility
shared target dispersion
no mobility
Google mobility
Facebook tiles visited mobility
Facebook single tile mobility

Türkiye

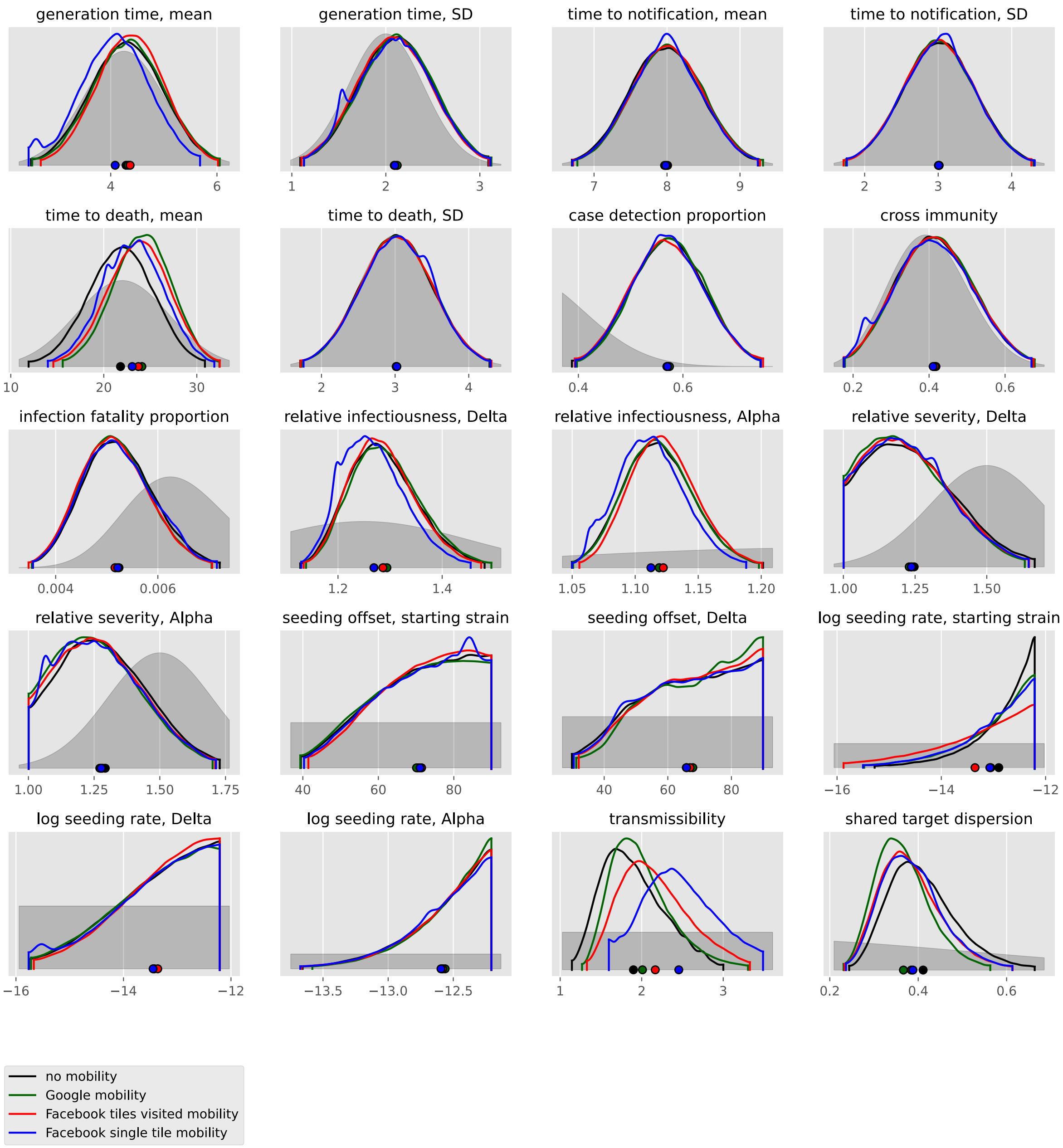

generation time, mean
generation time, SD
time to notification, mean
time to notification, SD
time to death, mean
time to death, SD
case detection proportion
cross immunity
infection fatality proportion
relative infectiousness, Delta
relative infectiousness, Alpha
relative severity, Delta
relative severity, Alpha
seeding offset, starting strain
seeding offset, Delta
log seeding rate, starting strain
log seeding rate, Delta
log seeding rate, Alpha
transmissibility
shared target dispersion
no mobility
Google mobility
Facebook tiles visited mobility
Facebook single tile mobility

United Arab Emirates

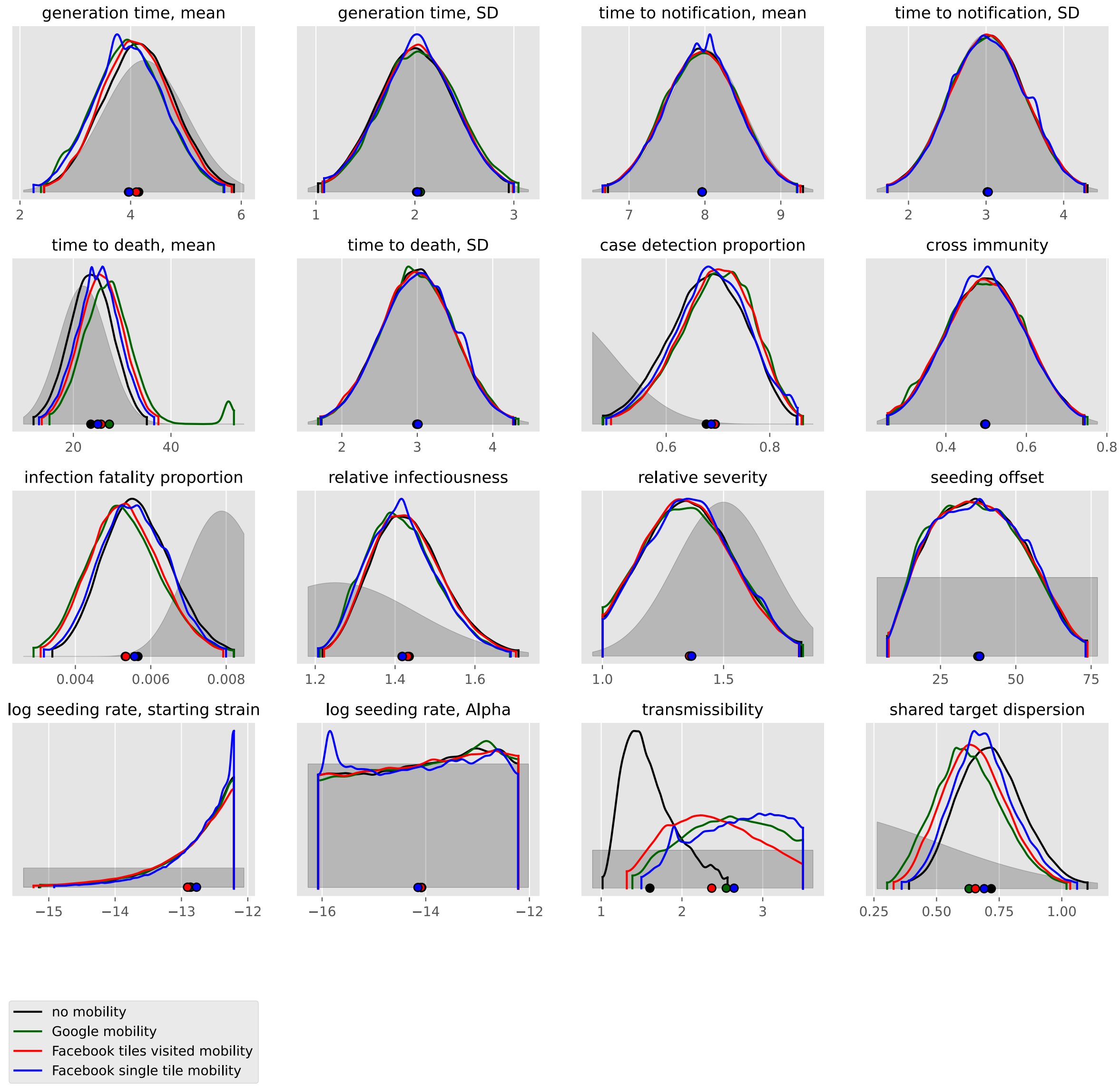

Yemen

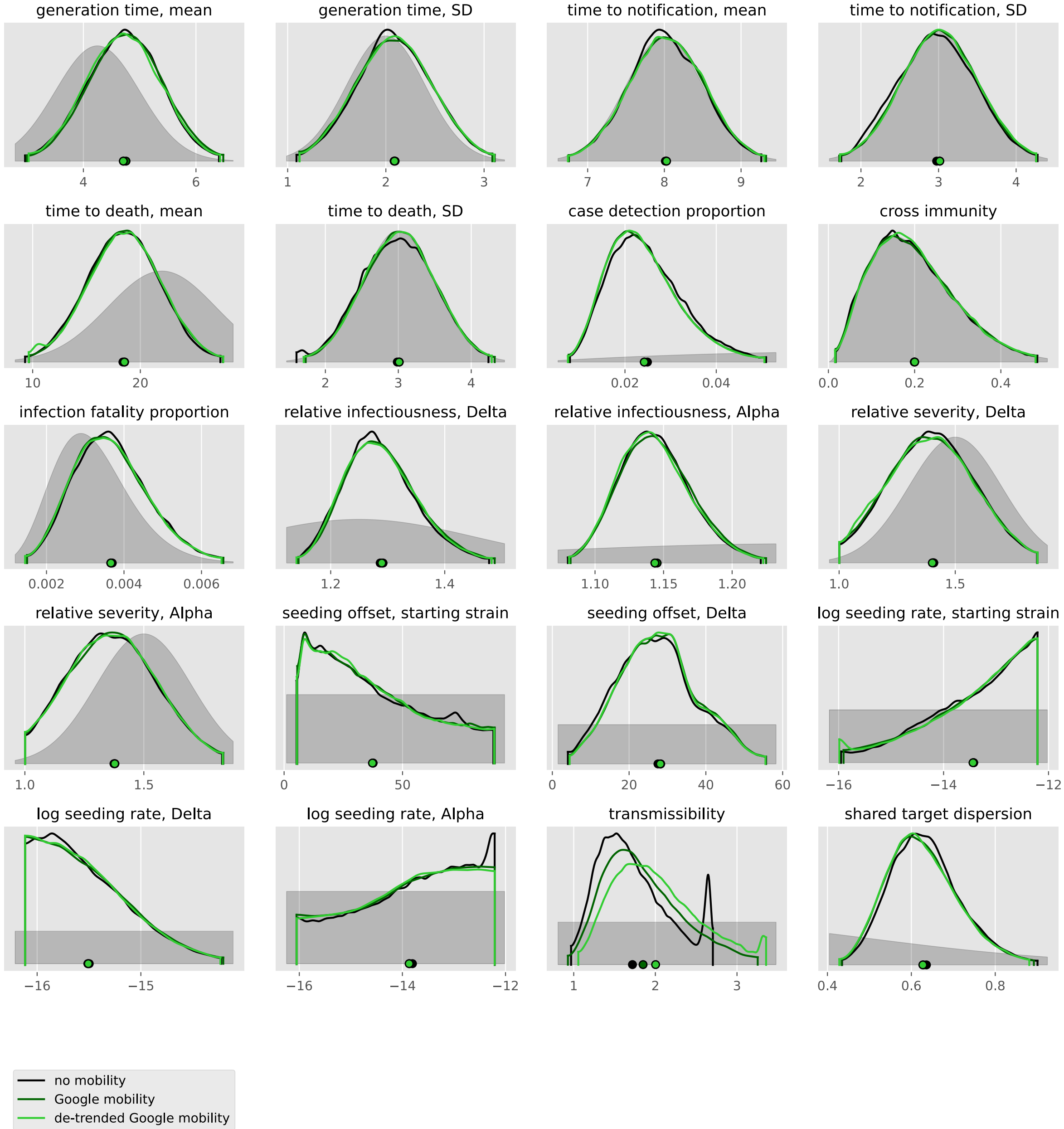

generation time, mean
generation time, SD
time to notification, mean
time to notification, SD
time to death, mean
time to death, SD
case detection proportion
cross immunity
infection fatality proportion
relative infectiousness, Delta
relative infectiousness, Alpha
relative severity, Delta
relative severity, Alpha
seeding offset, starting strain
seeding offset, Delta
log seeding rate, starting strain
log seeding rate, Delta
log seeding rate, Alpha
transmissibility
shared target dispersion
no mobility
Google mobility
de-trended Google mobility

Europe

Albania

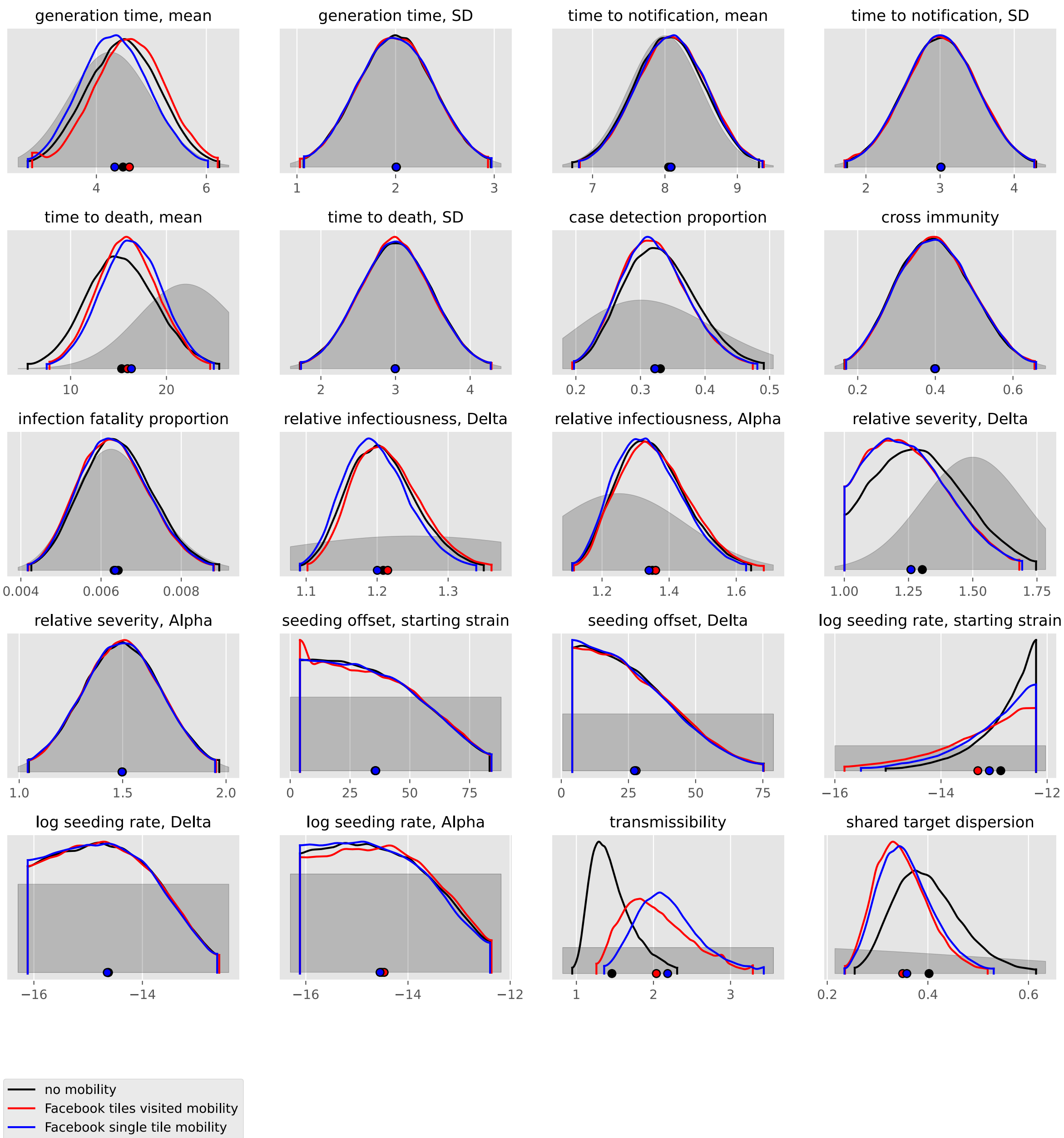

Austria

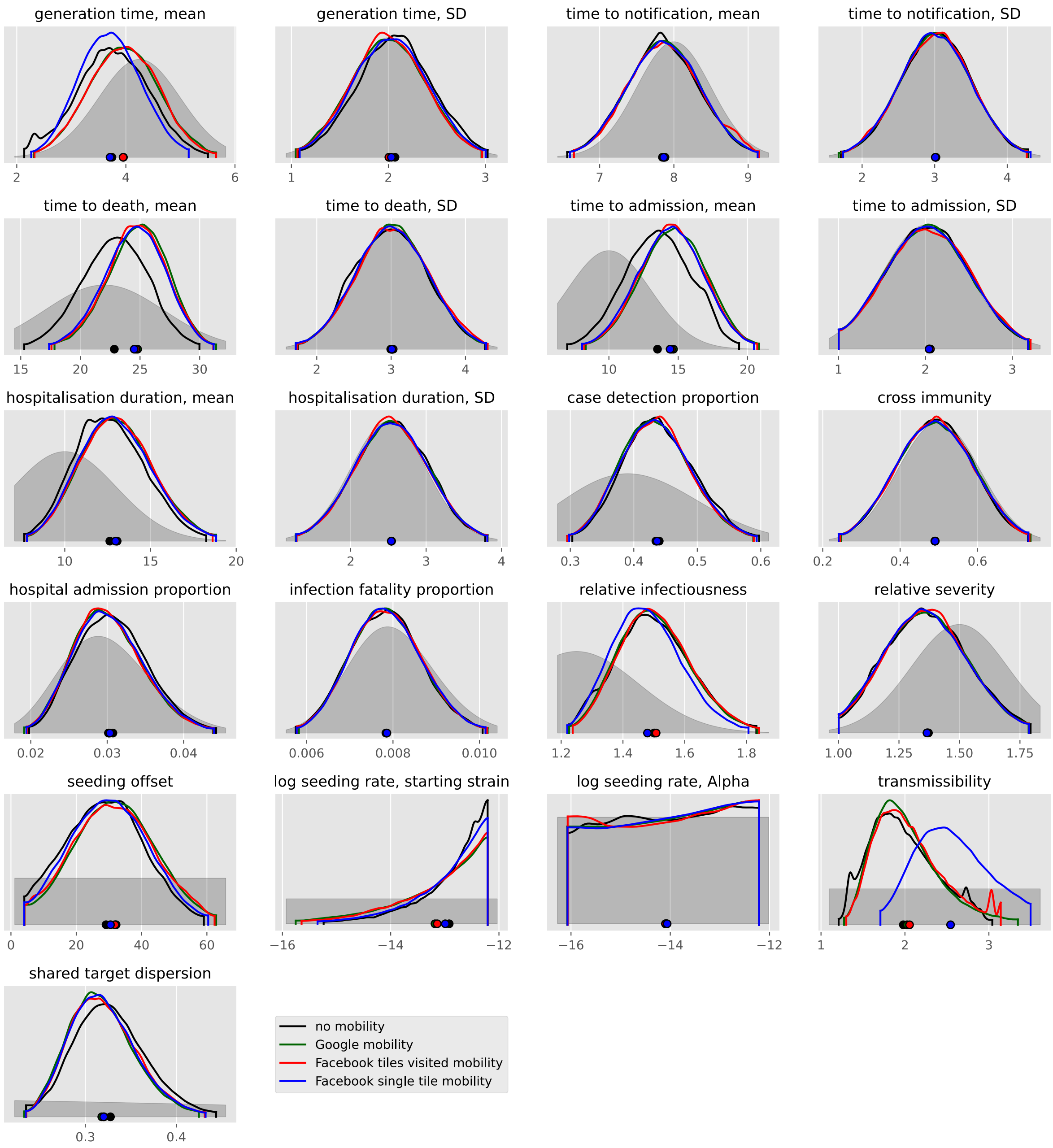

generation time, mean
generation time, SD
time to notification, mean
time to notification, SD
time to death, mean
time to death, SD
time to admission, mean
time to admission, SD
hospitalisation duration, mean
hospitalisation duration, SD
case detection proportion
cross immunity
hospital admission proportion
infection fatality proportion
relative infectiousness
relative severity
seeding offset
log seeding rate, starting strain
log seeding rate, Alpha
transmissibility
shared target dispersion
no mobility
Google mobility
Facebook tiles visited mobility
Facebook single tile mobility

Belarus

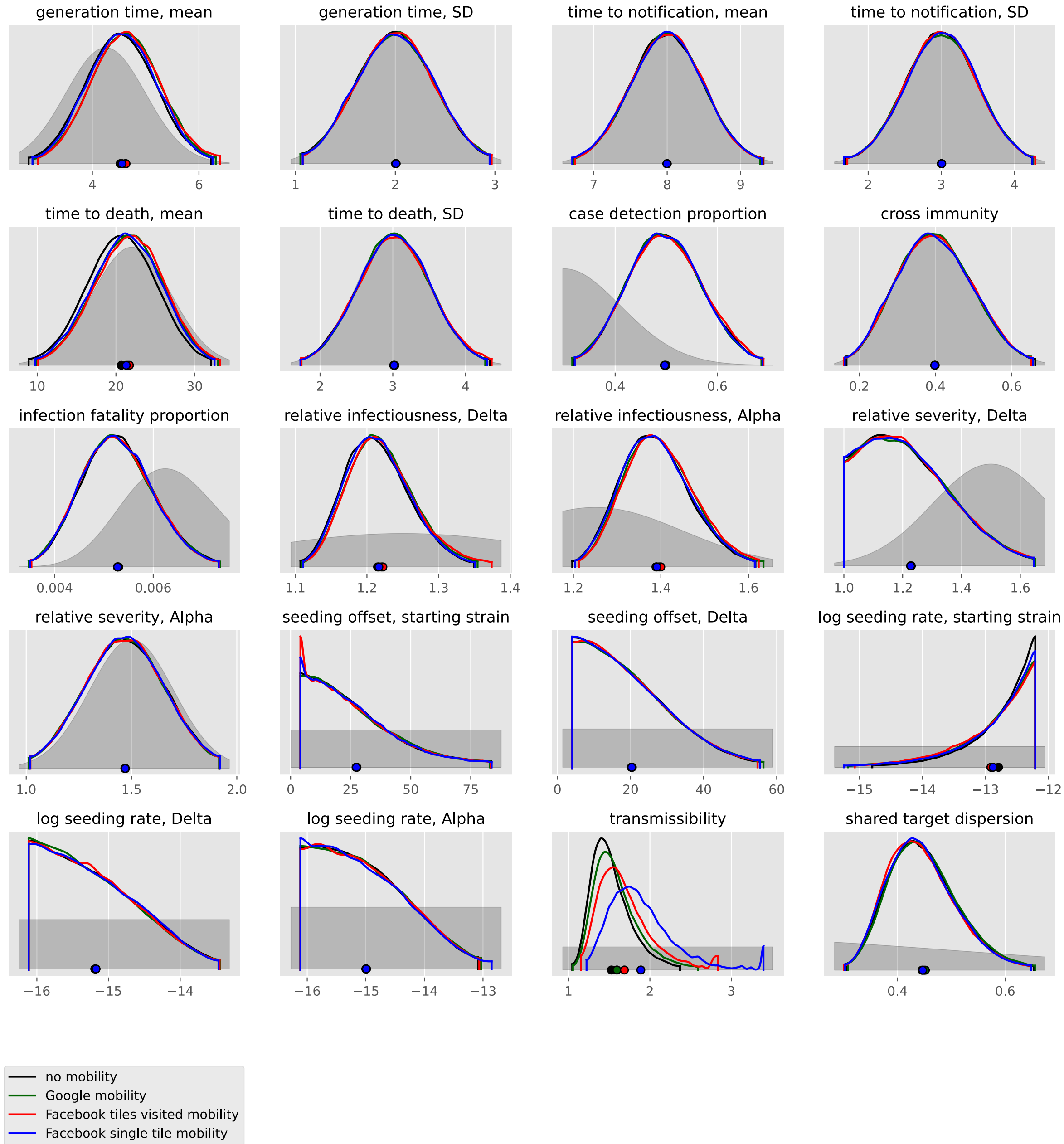
generation time, mean
generation time, SD
time to notification, mean
time to notification, SD
time to death, mean
time to death, SD
case detection proportion
cross immunity
infection fatality proportion
relative infectiousness, Delta
relative infectiousness, Alpha
relative severity, Delta
relative severity, Alpha
seeding offset, starting strain
seeding offset, Delta
log seeding rate, starting strain
log seeding rate, Delta
log seeding rate, Alpha
transmissibility
shared target dispersion
no mobility
Google mobility
Facebook tiles visited mobility
Facebook single tile mobility

Belgium

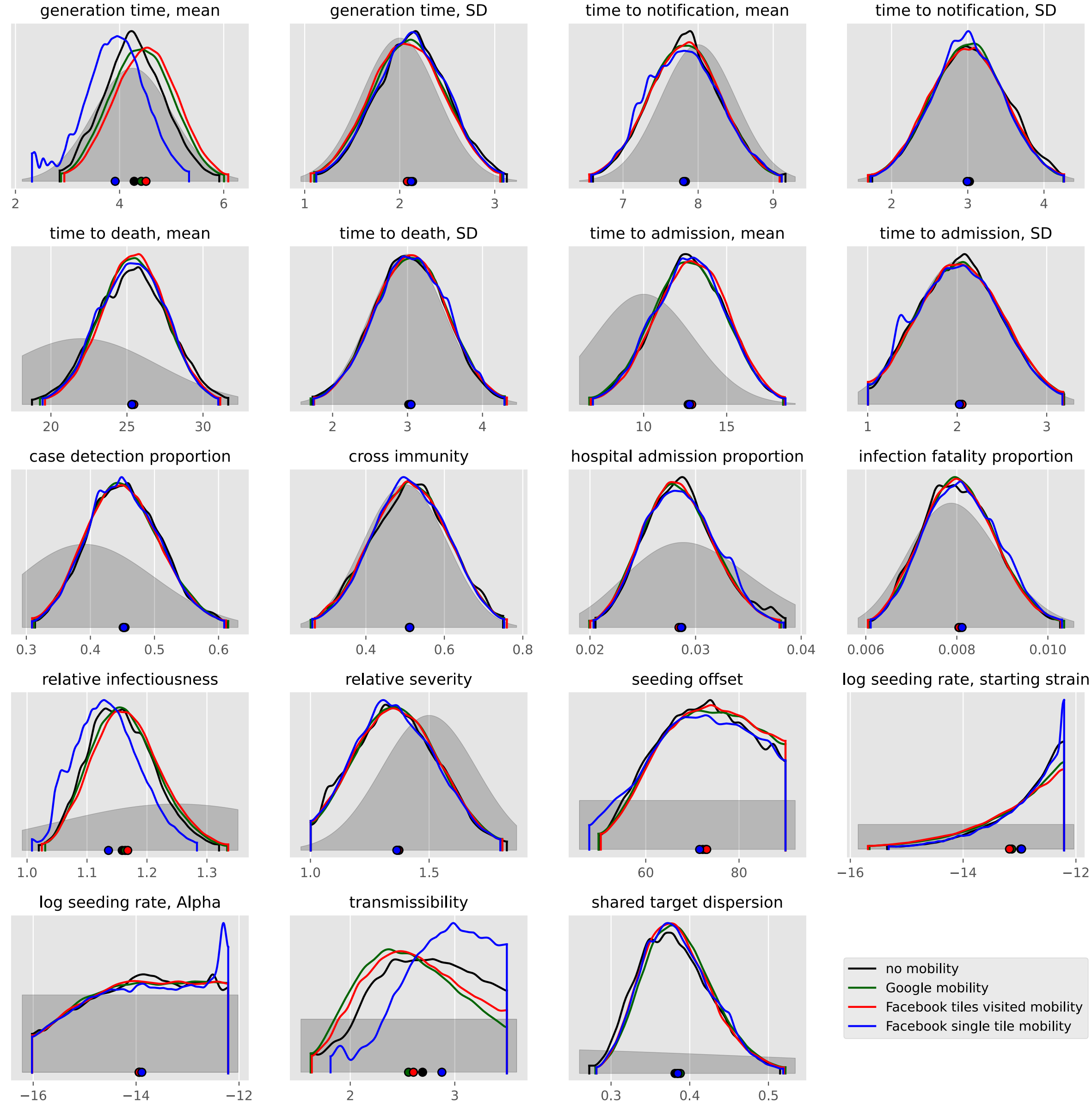

generation time, mean
generation time, SD
time to notification, mean
time to notification, SD
time to death, mean
time to death, SD
time to admission, mean
time to admission, SD
case detection proportion
cross immunity
hospital admission proportion
infection fatality proportion
relative infectiousness
relative severity
seeding offset
log seeding rate, starting strain
log seeding rate, Alpha
transmissibility
shared target dispersion
no mobility
Google mobility
Facebook tiles visited mobility
Facebook single tile mobility

Bosnia and Herzegovina

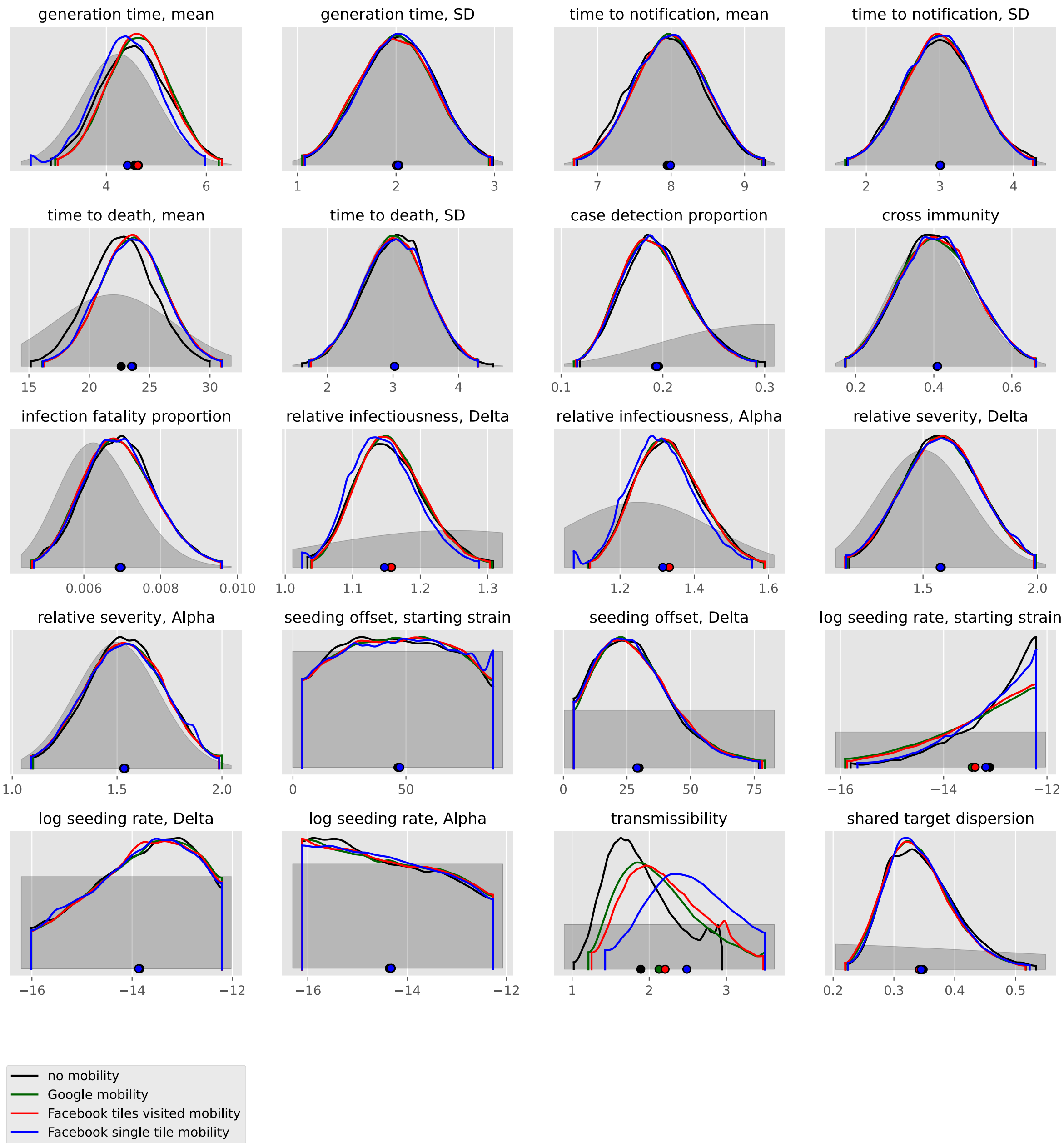

Bulgaria

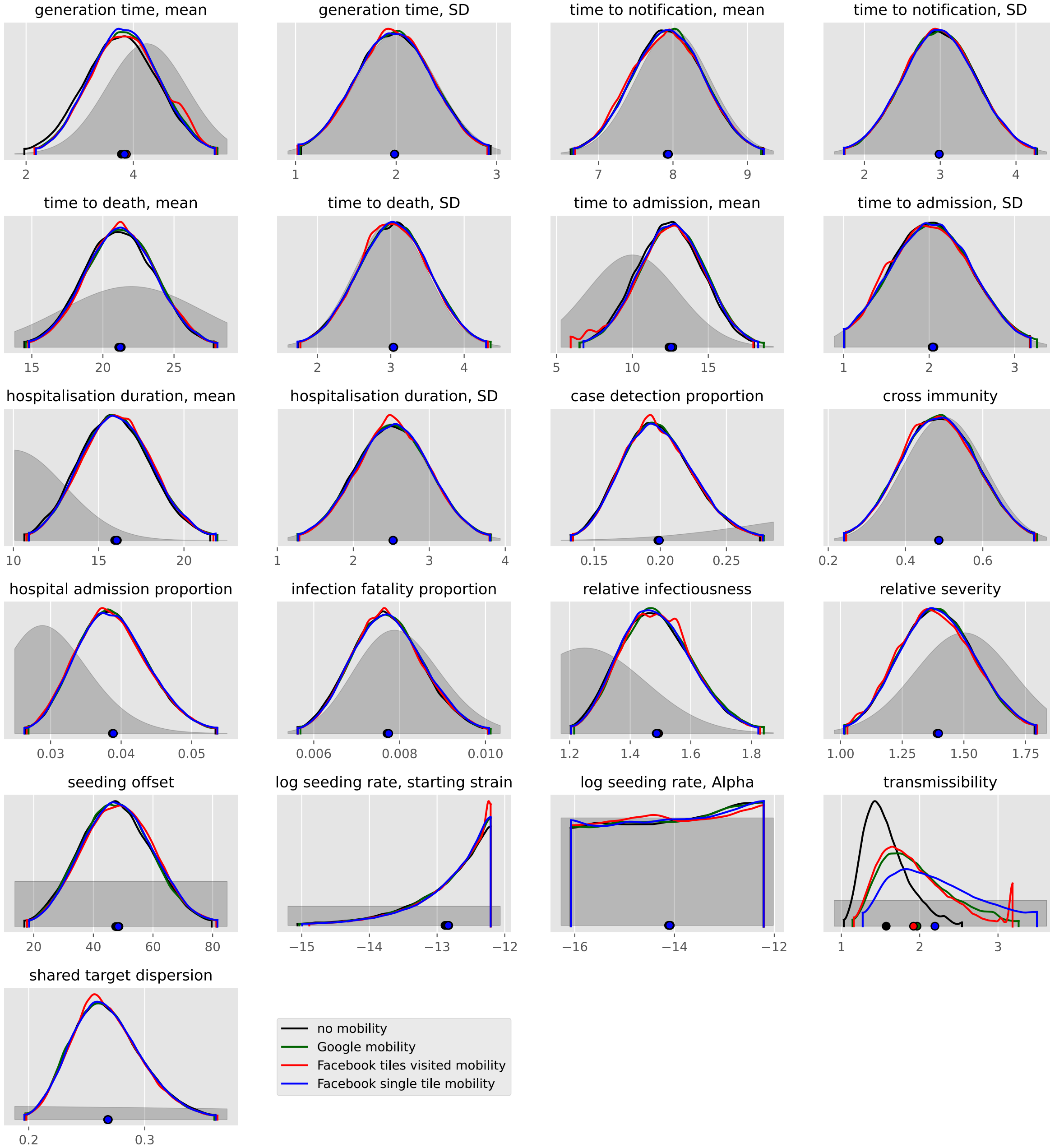
generation time, mean
2
4
generation time, SD
1
2
3
time to notification, mean
7
8
9
time to notification, SD
2
3
4
time to death, mean
15
20
25
time to death, SD
2
3
4
time to admission, mean
5
10
15
time to admission, SD
1
2
3
hospitalisation duration, mean
10
15
20
hospitalisation duration, SD
1
2
3
4
case detection proportion
0.15
0.20
0.25
cross immunity
0.2
0.4
0.6
hospital admission proportion
0.03
0.04
0.05
infection fatality proportion
0.006
0.008
0.010
relative infectiousness
1.2
1.4
1.6
1.8
relative severity
1.00
1.25
1.50
1.75
seeding offset
20
40
60
80
log seeding rate, starting strain
−15
−14
−13
−12
log seeding rate, Alpha
−16
−14
−12
transmissibility
1
2
3
shared target dispersion
0.2
0.3
no mobility
Google mobility
Facebook tiles visited mobility
Facebook single tile mobility

Croatia

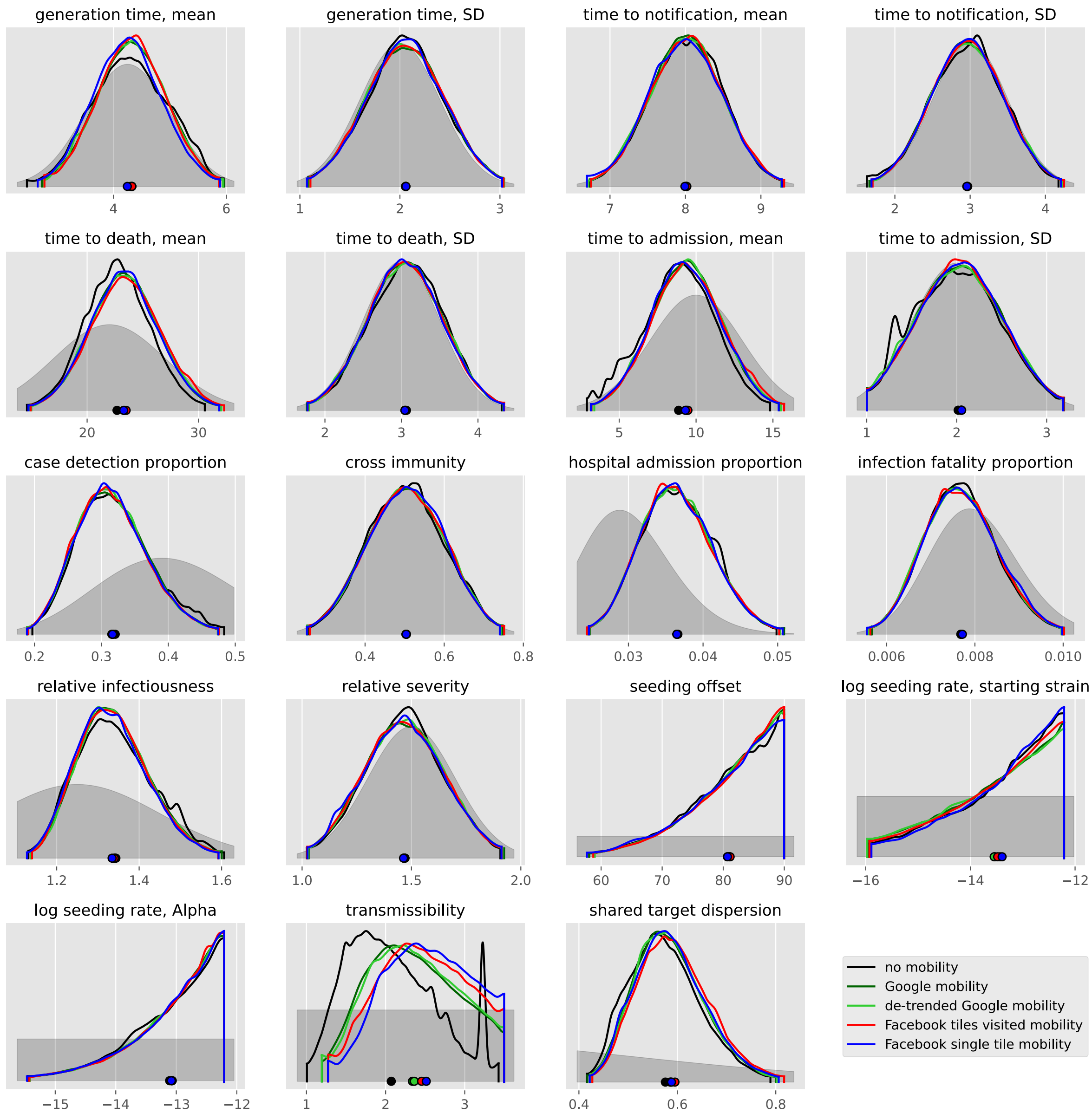
generation time, mean
generation time, SD
time to notification, mean
time to notification, SD
time to death, mean
time to death, SD
time to admission, mean
time to admission, SD
case detection proportion
cross immunity
hospital admission proportion
infection fatality proportion
relative infectiousness
relative severity
seeding offset
log seeding rate, starting strain
log seeding rate, Alpha
transmissibility
shared target dispersion
no mobility
Google mobility
de-trended Google mobility
Facebook tiles visited mobility
Facebook single tile mobility

Czechia

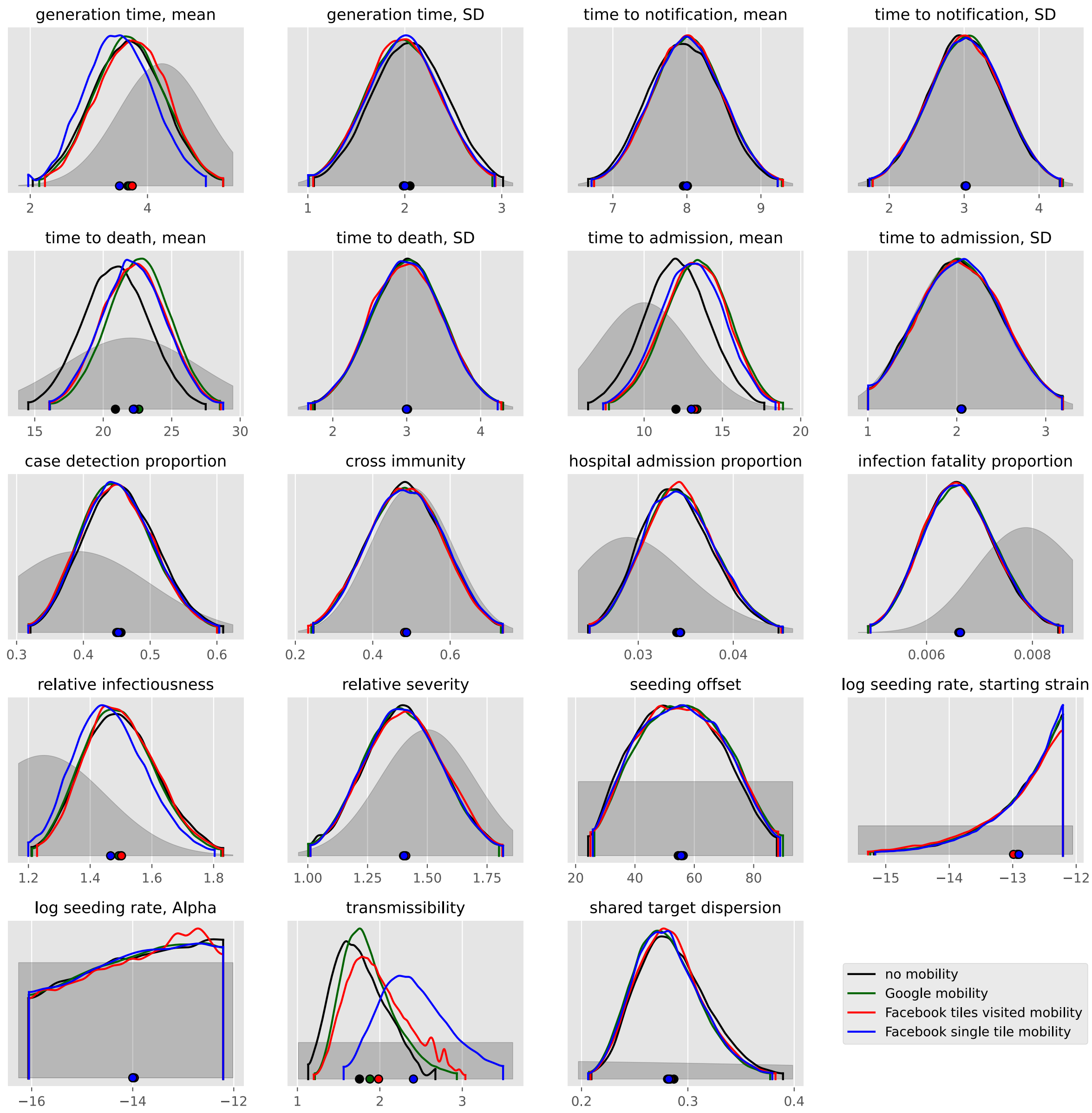

generation time, mean
generation time, SD
time to notification, mean
time to notification, SD
time to death, mean
time to death, SD
time to admission, mean
time to admission, SD
case detection proportion
cross immunity
hospital admission proportion
infection fatality proportion
relative infectiousness
relative severity
seeding offset
log seeding rate, starting strain
log seeding rate, Alpha
transmissibility
shared target dispersion
no mobility
Google mobility
Facebook tiles visited mobility
Facebook single tile mobility

Denmark

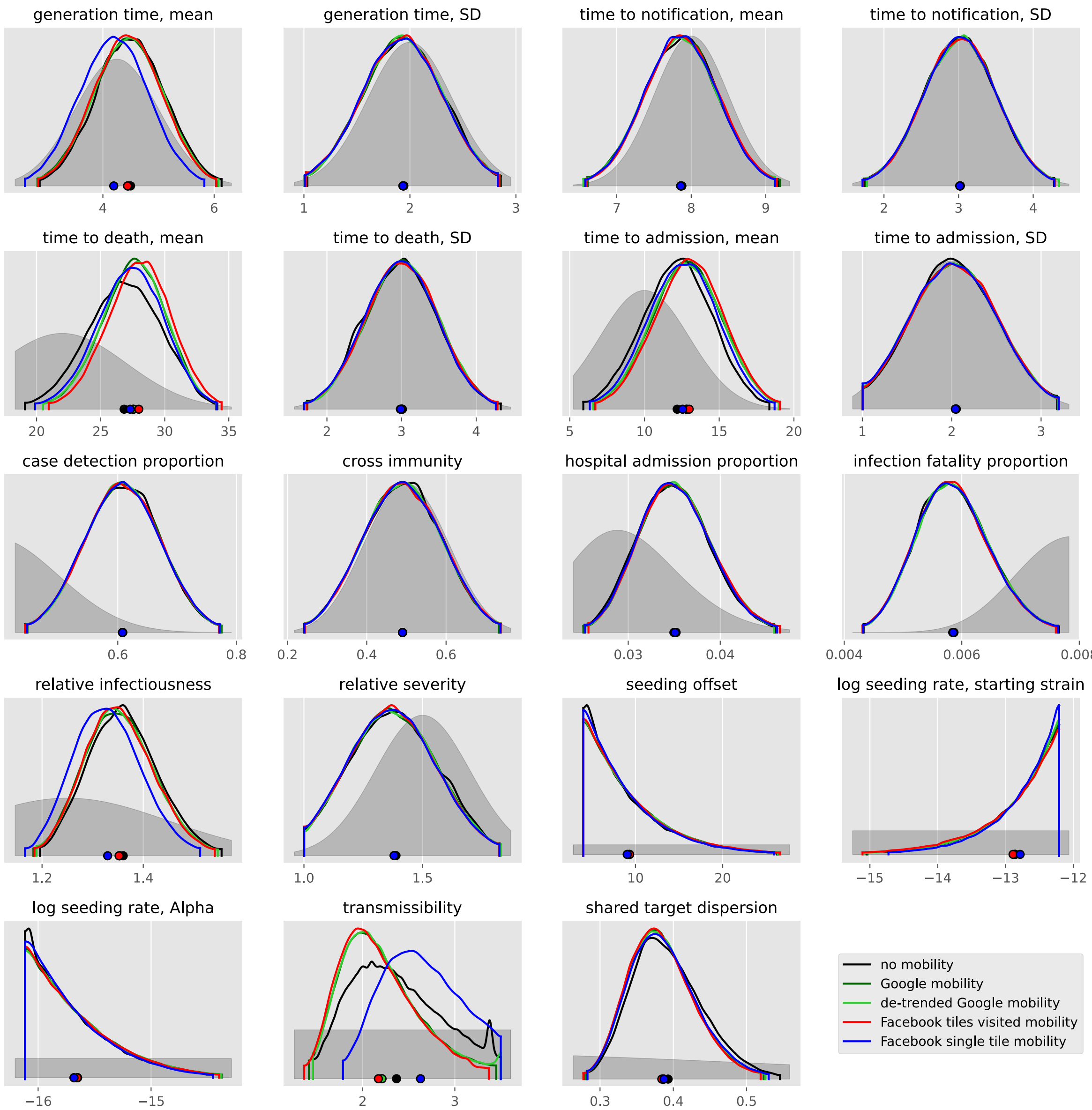
generation time, mean
4
6
generation time, SD
1
2
3
time to notification, mean
7
8
9
time to notification, SD
2
3
4
time to death, mean
20
25
30
35
time to death, SD
2
3
4
time to admission, mean
5
10
15
20
time to admission, SD
1
2
3
case detection proportion
0.6
0.8
cross immunity
0.2
0.4
0.6
hospital admission proportion
0.03
0.04
infection fatality proportion
0.004
0.006
0.008
relative infectiousness
1.2
1.4
relative severity
1.0
1.5
seeding offset
10
20
log seeding rate, starting strain
−15
−14
−13
−12
log seeding rate, Alpha
−16
−15
transmissibility
2
3
shared target dispersion
0.3
0.4
0.5
no mobility
Google mobility
de-trended Google mobility
Facebook tiles visited mobility
Facebook single tile mobility

Estonia

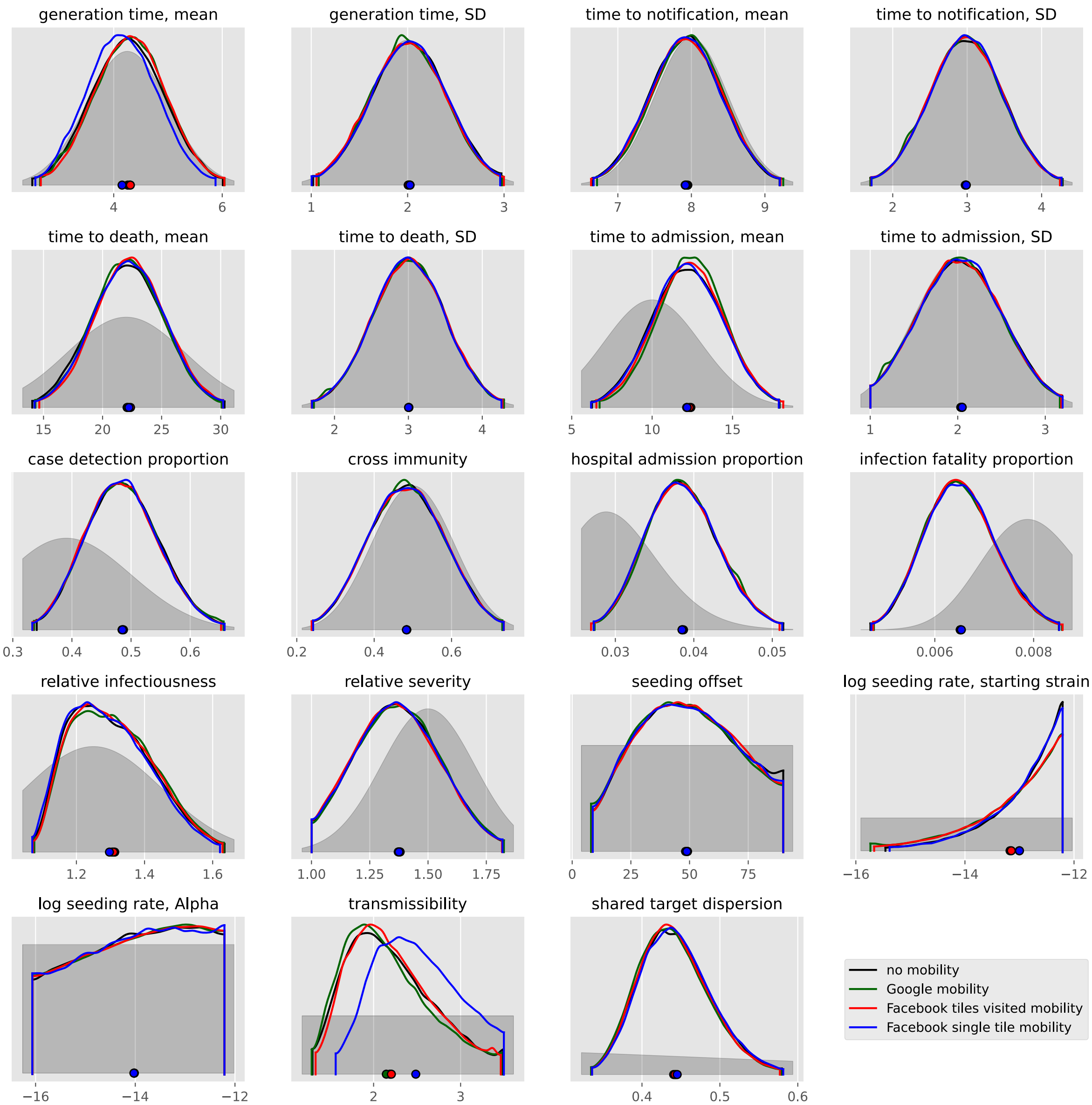

generation time, mean
generation time, SD
time to notification, mean
time to notification, SD
time to death, mean
time to death, SD
time to admission, mean
time to admission, SD
case detection proportion
cross immunity
hospital admission proportion
infection fatality proportion
relative infectiousness
relative severity
seeding offset
log seeding rate, starting strain
log seeding rate, Alpha
transmissibility
shared target dispersion
no mobility
Google mobility
Facebook tiles visited mobility
Facebook single tile mobility

Finland

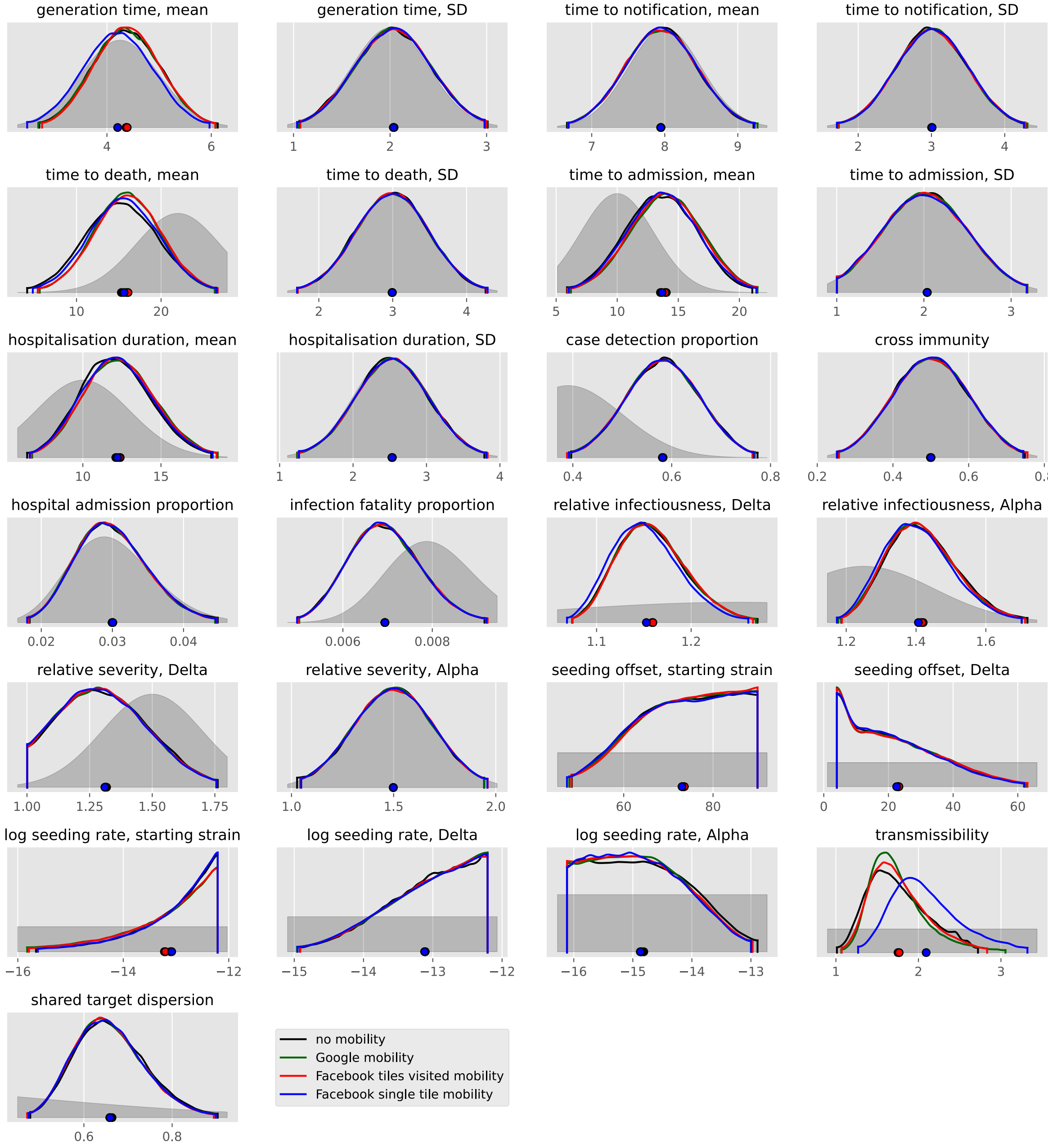
generation time, mean
generation time, SD
time to notification, mean
time to notification, SD
time to death, mean
time to death, SD
time to admission, mean
time to admission, SD
hospitalisation duration, mean
hospitalisation duration, SD
case detection proportion
cross immunity
hospital admission proportion
infection fatality proportion
relative infectiousness, Delta
relative infectiousness, Alpha
relative severity, Delta
relative severity, Alpha
seeding offset, starting strain
seeding offset, Delta
log seeding rate, starting strain
log seeding rate, Delta
log seeding rate, Alpha
transmissibility
shared target dispersion
no mobility
Google mobility
Facebook tiles visited mobility
Facebook single tile mobility

France

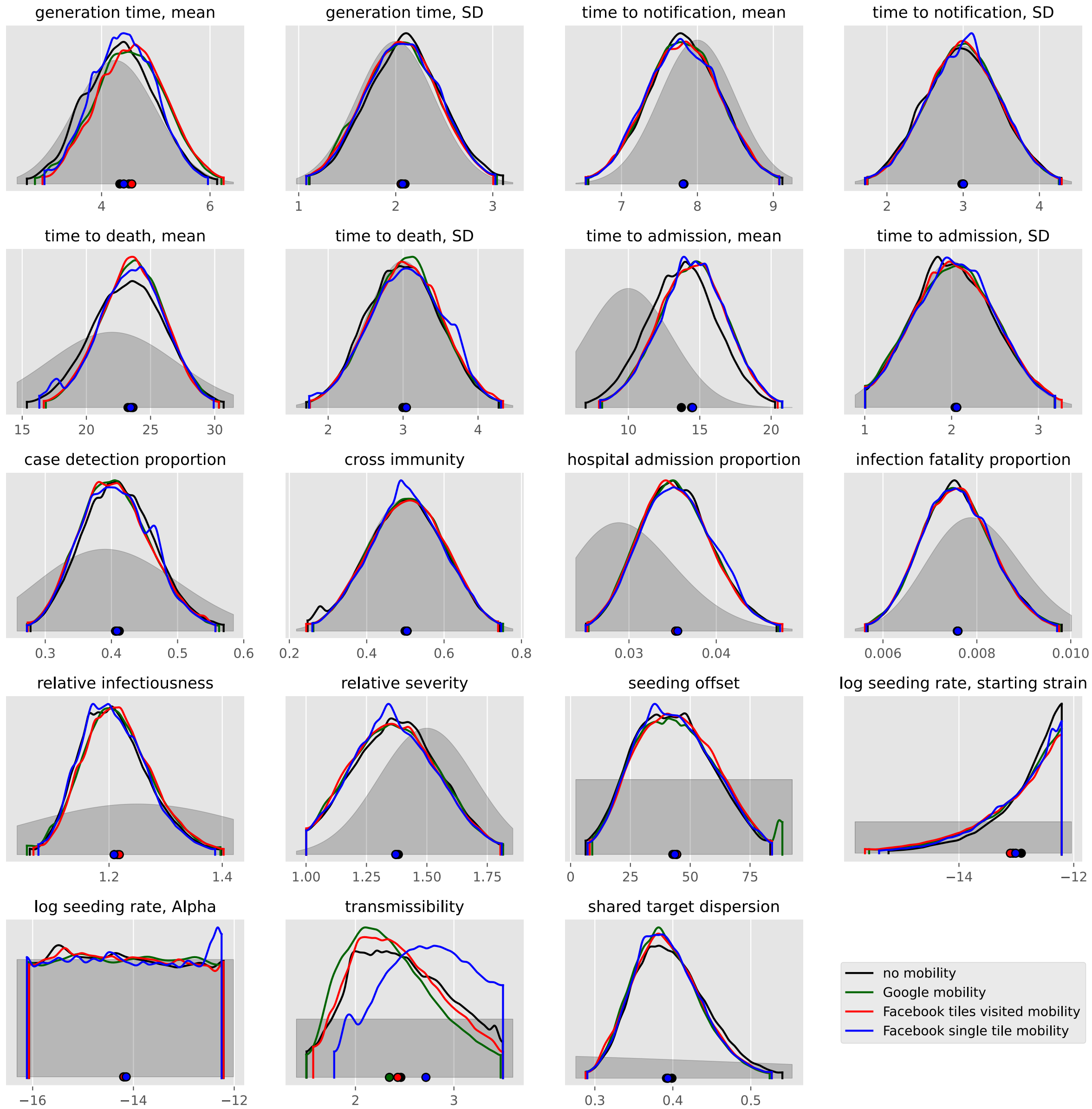
generation time, mean
generation time, SD
time to notification, mean
time to notification, SD
time to death, mean
time to death, SD
time to admission, mean
time to admission, SD
case detection proportion
cross immunity
hospital admission proportion
infection fatality proportion
relative infectiousness
relative severity
seeding offset
log seeding rate, starting strain
log seeding rate, Alpha
transmissibility
shared target dispersion
no mobility
Google mobility
Facebook tiles visited mobility
Facebook single tile mobility

Germany

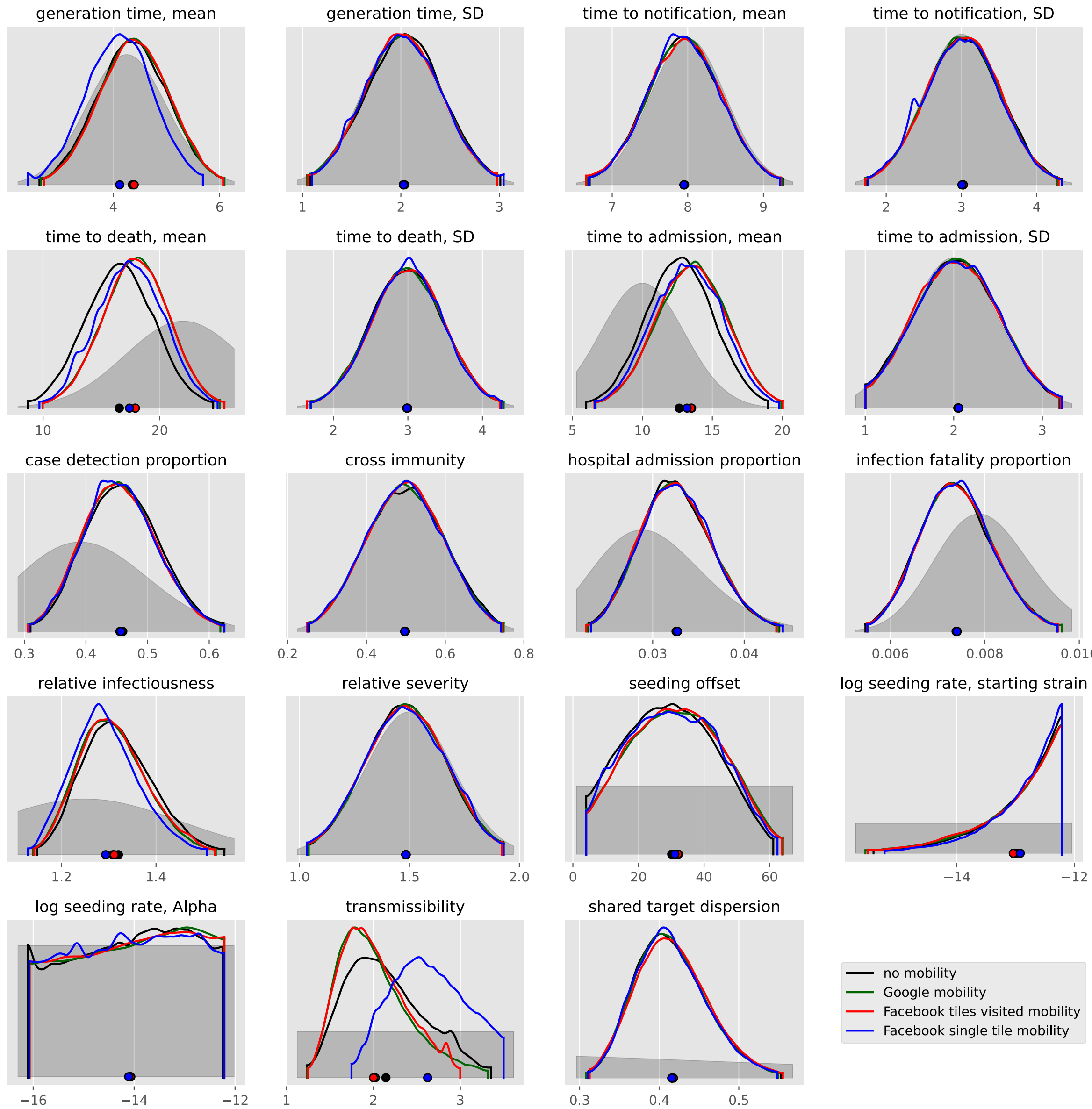

generation time, mean
generation time, SD
time to notification, mean
time to notification, SD
time to death, mean
time to death, SD
time to admission, mean
time to admission, SD
case detection proportion
cross immunity
hospital admission proportion
infection fatality proportion
relative infectiousness
relative severity
seeding offset
log seeding rate, starting strain
log seeding rate, Alpha
transmissibility
shared target dispersion
no mobility
Google mobility
Facebook tiles visited mobility
Facebook single tile mobility

Greece

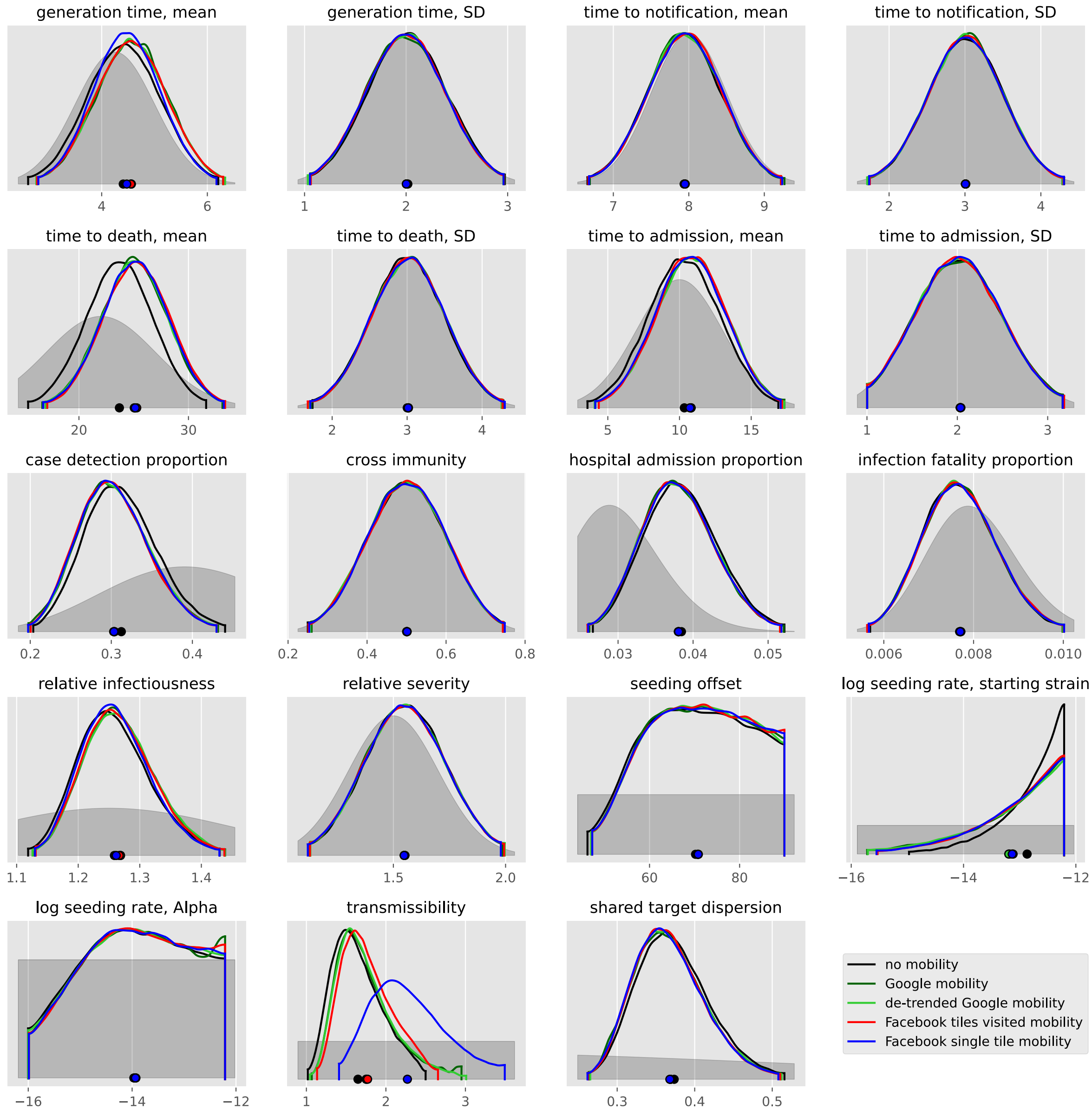
generation time, mean
generation time, SD
time to notification, mean
time to notification, SD
time to death, mean
time to death, SD
time to admission, mean
time to admission, SD
case detection proportion
cross immunity
hospital admission proportion
infection fatality proportion
relative infectiousness
relative severity
seeding offset
log seeding rate, starting strain
log seeding rate, Alpha
transmissibility
shared target dispersion
no mobility
Google mobility
de-trended Google mobility
Facebook tiles visited mobility
Facebook single tile mobility

Hungary

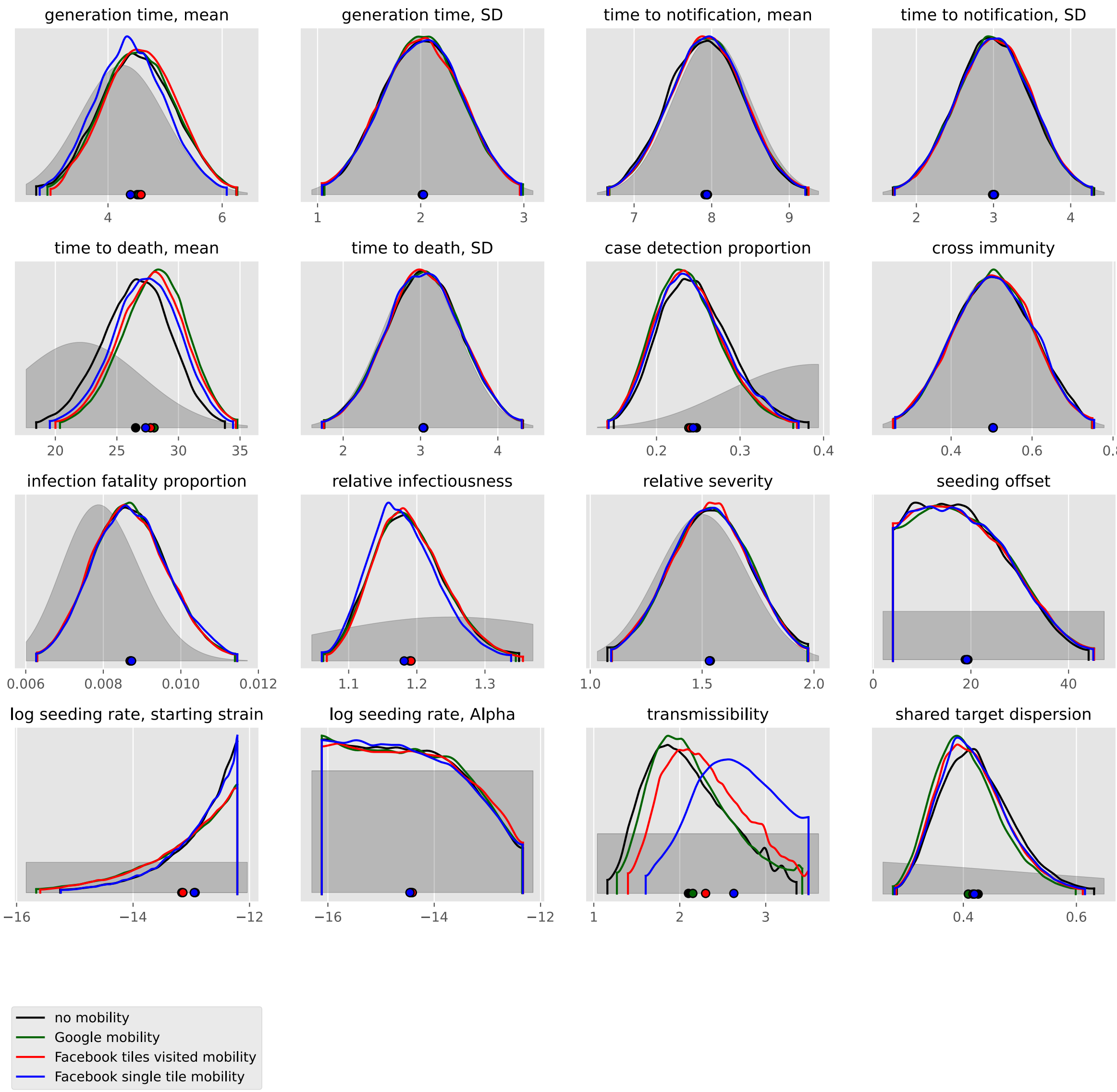

generation time, mean
generation time, SD
time to notification, mean
time to notification, SD
time to death, mean
time to death, SD
case detection proportion
cross immunity
infection fatality proportion
relative infectiousness
relative severity
seeding offset
log seeding rate, starting strain
log seeding rate, Alpha
transmissibility
shared target dispersion
no mobility
Google mobility
Facebook tiles visited mobility
Facebook single tile mobility

Ireland

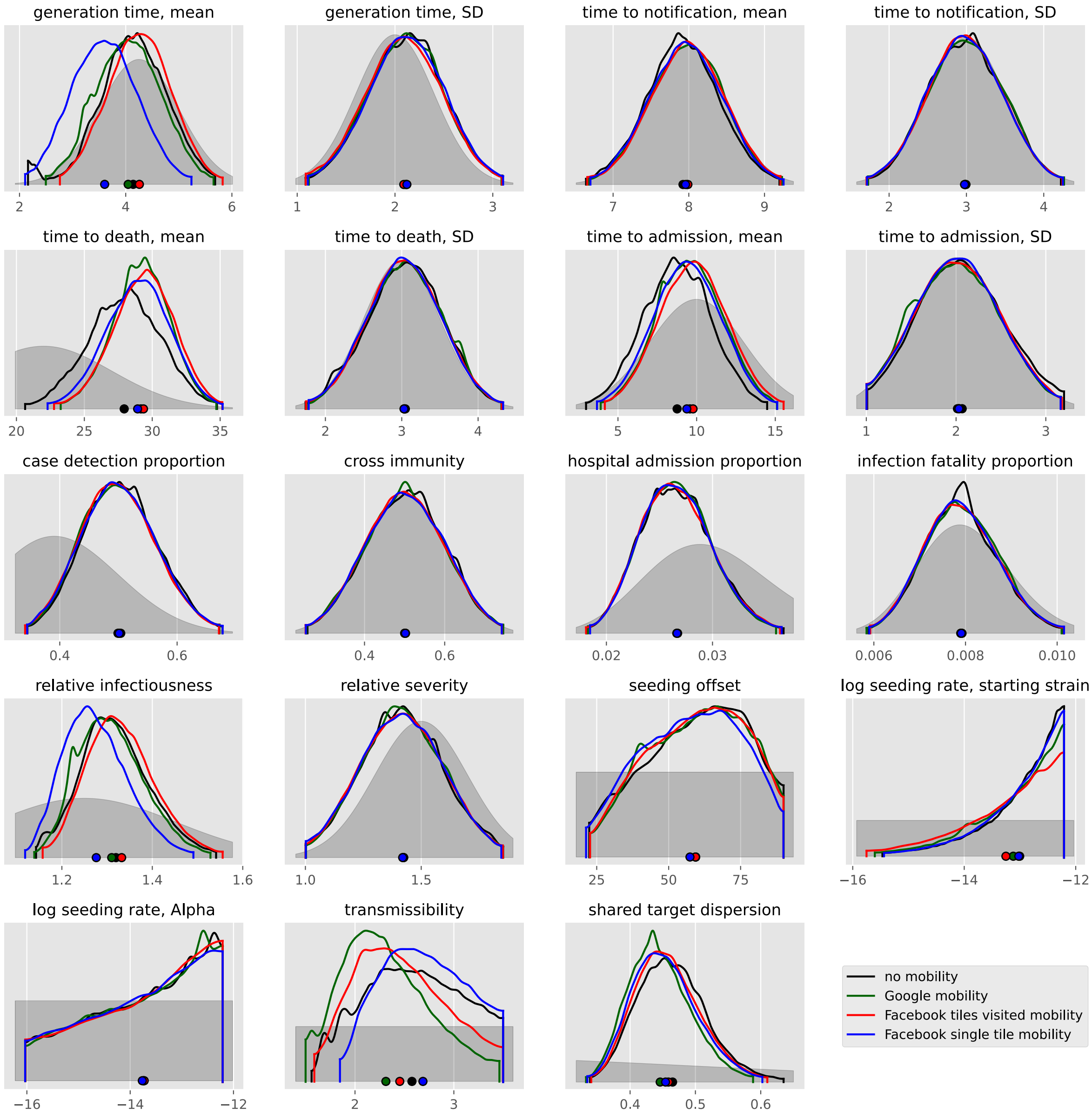

generation time, mean
generation time, SD
time to notification, mean
time to notification, SD
time to death, mean
time to death, SD
time to admission, mean
time to admission, SD
case detection proportion
cross immunity
hospital admission proportion
infection fatality proportion
relative infectiousness
relative severity
seeding offset
log seeding rate, starting strain
log seeding rate, Alpha
transmissibility
shared target dispersion
no mobility
Google mobility
Facebook tiles visited mobility
Facebook single tile mobility

Italy

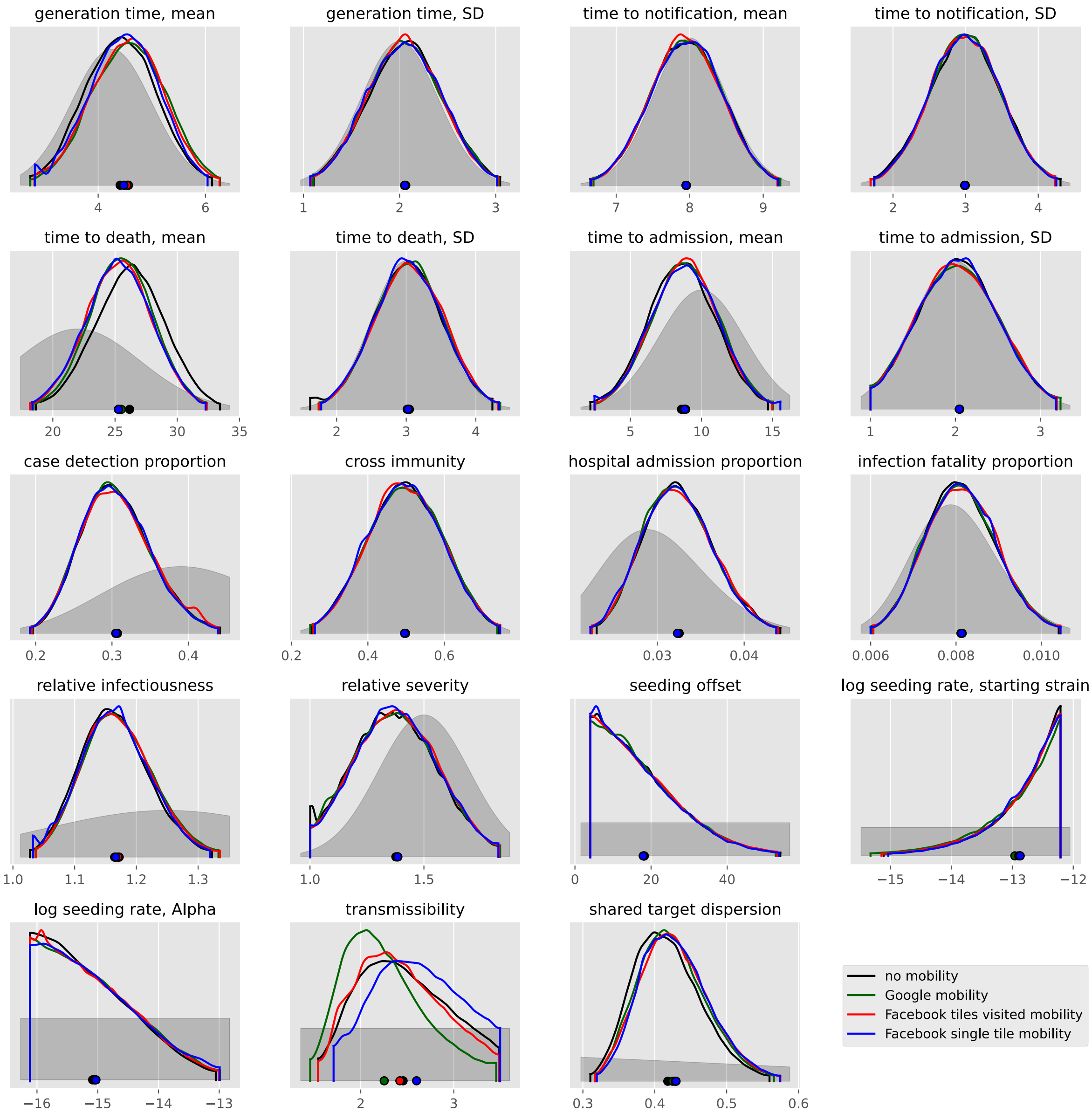

generation time, mean
generation time, SD
time to notification, mean
time to notification, SD
time to death, mean
time to death, SD
time to admission, mean
time to admission, SD
case detection proportion
cross immunity
hospital admission proportion
infection fatality proportion
relative infectiousness
relative severity
seeding offset
log seeding rate, starting strain
log seeding rate, Alpha
transmissibility
shared target dispersion
no mobility
Google mobility
Facebook tiles visited mobility
Facebook single tile mobility

Latvia

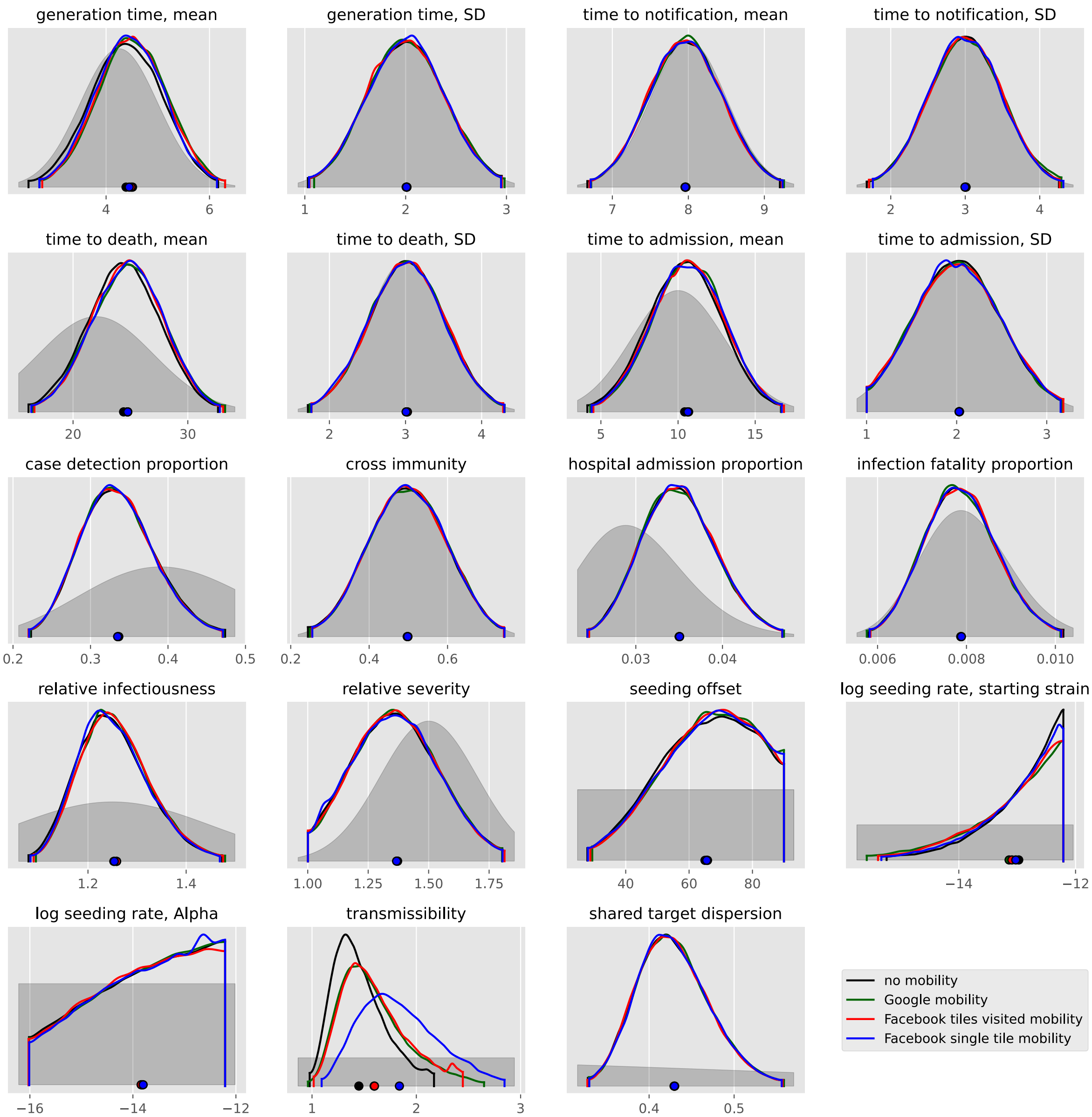

generation time, mean
generation time, SD
time to notification, mean
time to notification, SD
time to death, mean
time to death, SD
time to admission, mean
time to admission, SD
case detection proportion
cross immunity
hospital admission proportion
infection fatality proportion
relative infectiousness
relative severity
seeding offset
log seeding rate, starting strain
log seeding rate, Alpha
transmissibility
shared target dispersion
no mobility
Google mobility
Facebook tiles visited mobility
Facebook single tile mobility

Lithuania

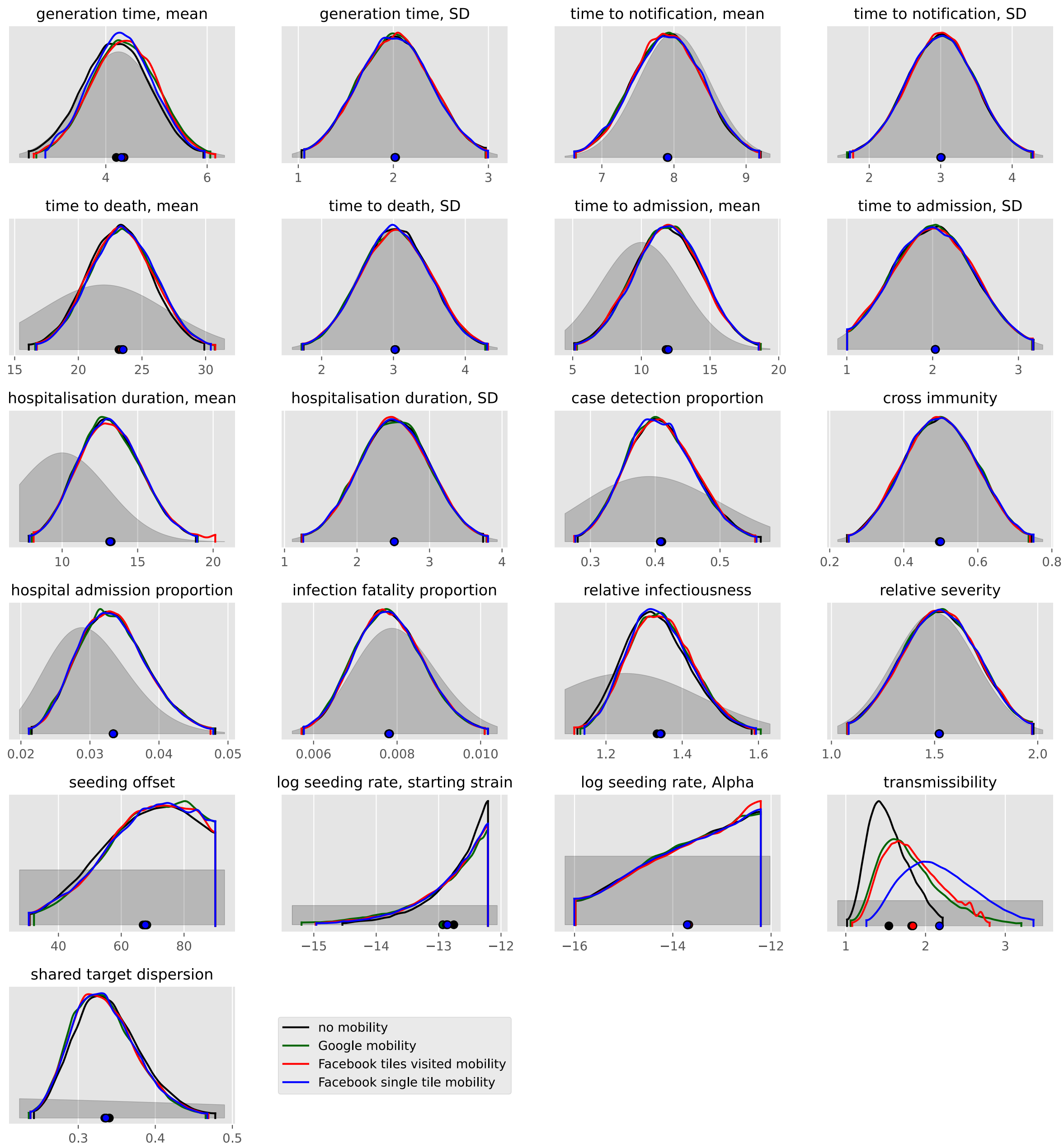

generation time, mean
generation time, SD
time to notification, mean
time to notification, SD
time to death, mean
time to death, SD
time to admission, mean
time to admission, SD
hospitalisation duration, mean
hospitalisation duration, SD
case detection proportion
cross immunity
hospital admission proportion
infection fatality proportion
relative infectiousness
relative severity
seeding offset
log seeding rate, starting strain
log seeding rate, Alpha
transmissibility
shared target dispersion
no mobility
Google mobility
Facebook tiles visited mobility
Facebook single tile mobility

Luxembourg

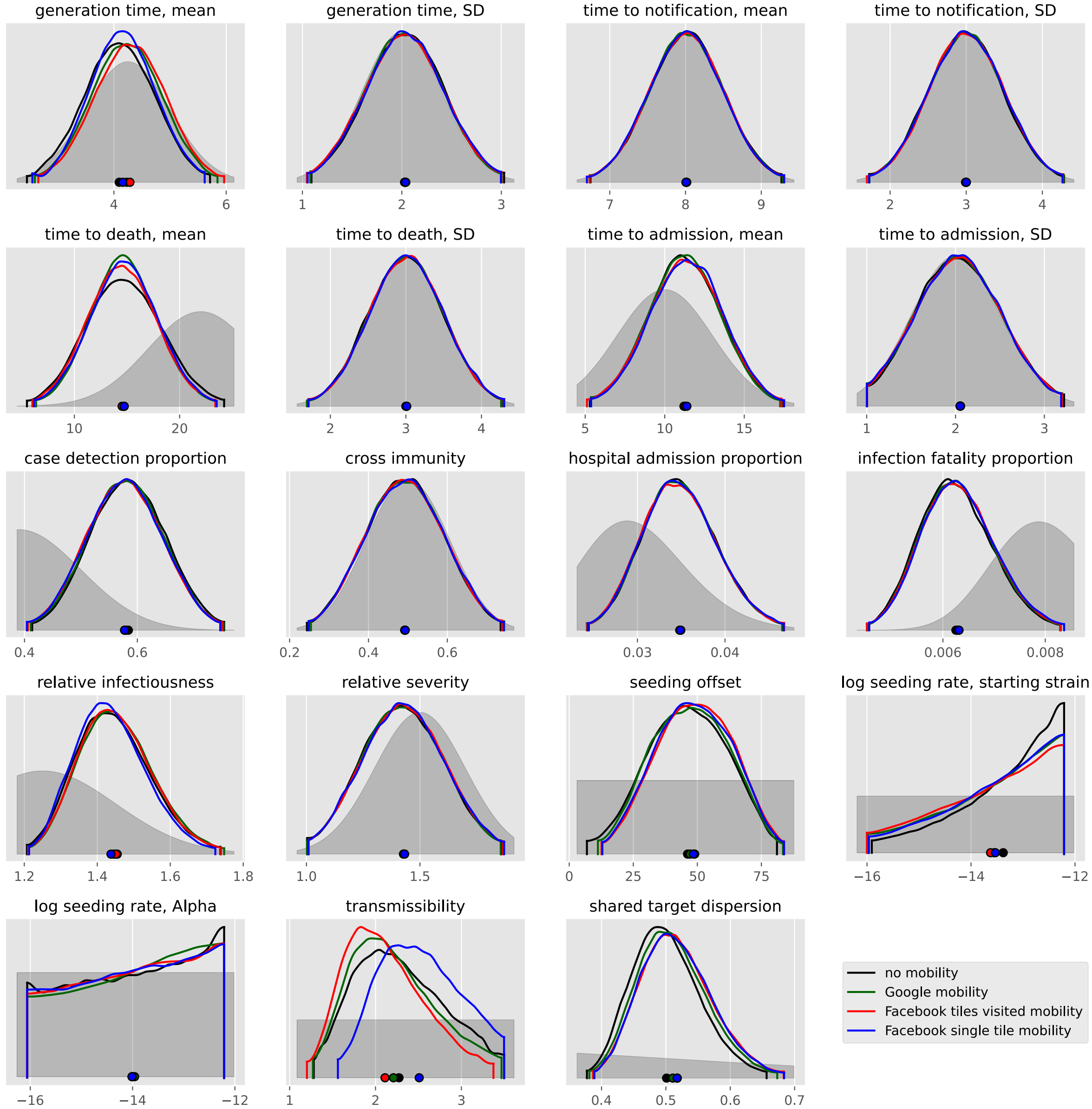
generation time, mean
generation time, SD
time to notification, mean
time to notification, SD
time to death, mean
time to death, SD
time to admission, mean
time to admission, SD
case detection proportion
cross immunity
hospital admission proportion
infection fatality proportion
relative infectiousness
relative severity
seeding offset
log seeding rate, starting strain
log seeding rate, Alpha
transmissibility
shared target dispersion
no mobility
Google mobility
Facebook tiles visited mobility
Facebook single tile mobility

Malta

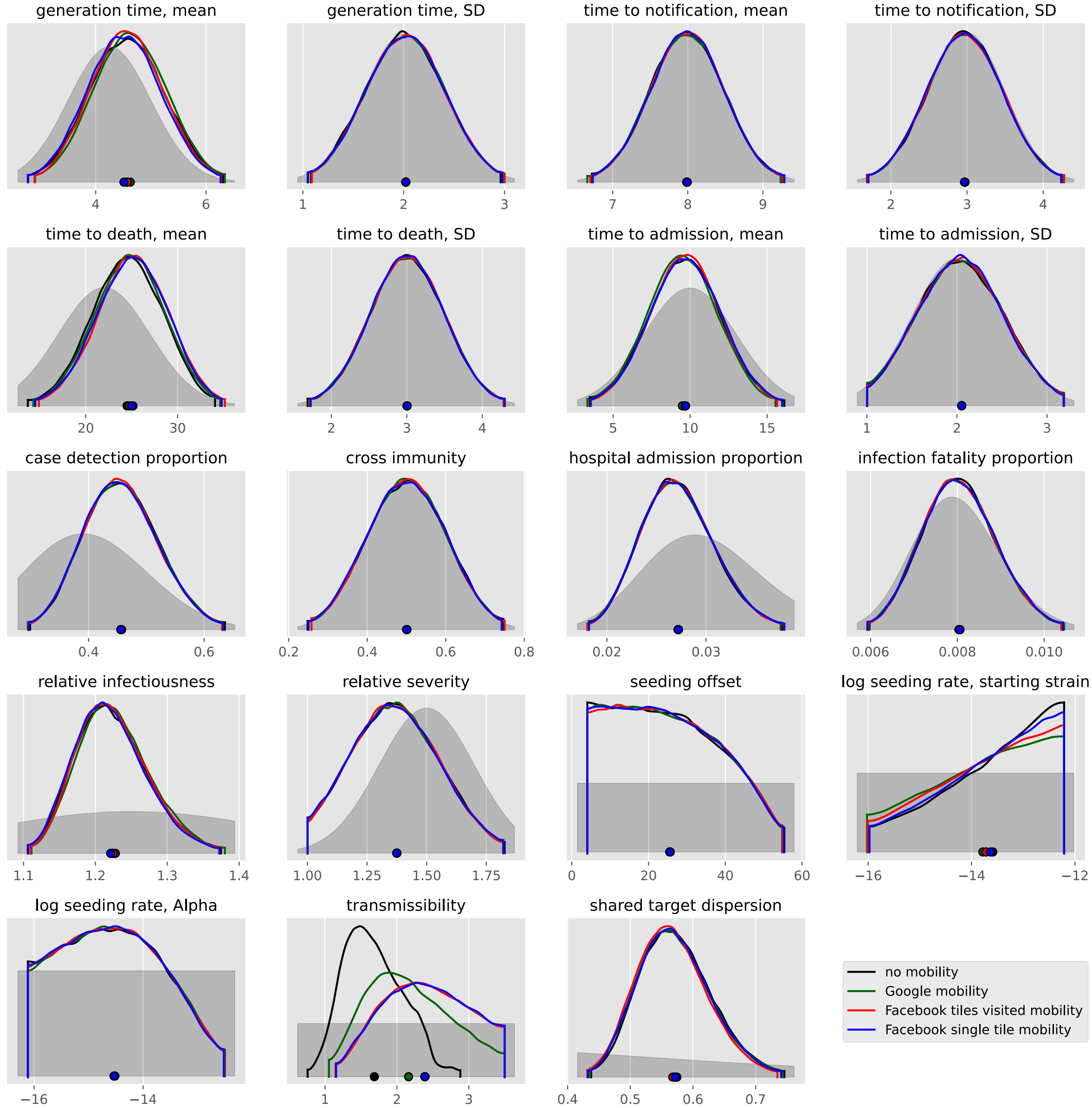

generation time, mean
generation time, SD
time to notification, mean
time to notification, SD
time to death, mean
time to death, SD
time to admission, mean
time to admission, SD
case detection proportion
cross immunity
hospital admission proportion
infection fatality proportion
relative infectiousness
relative severity
seeding offset
log seeding rate, starting strain
log seeding rate, Alpha
transmissibility
shared target dispersion
no mobility
Google mobility
Facebook tiles visited mobility
Facebook single tile mobility

Moldova, Republic of

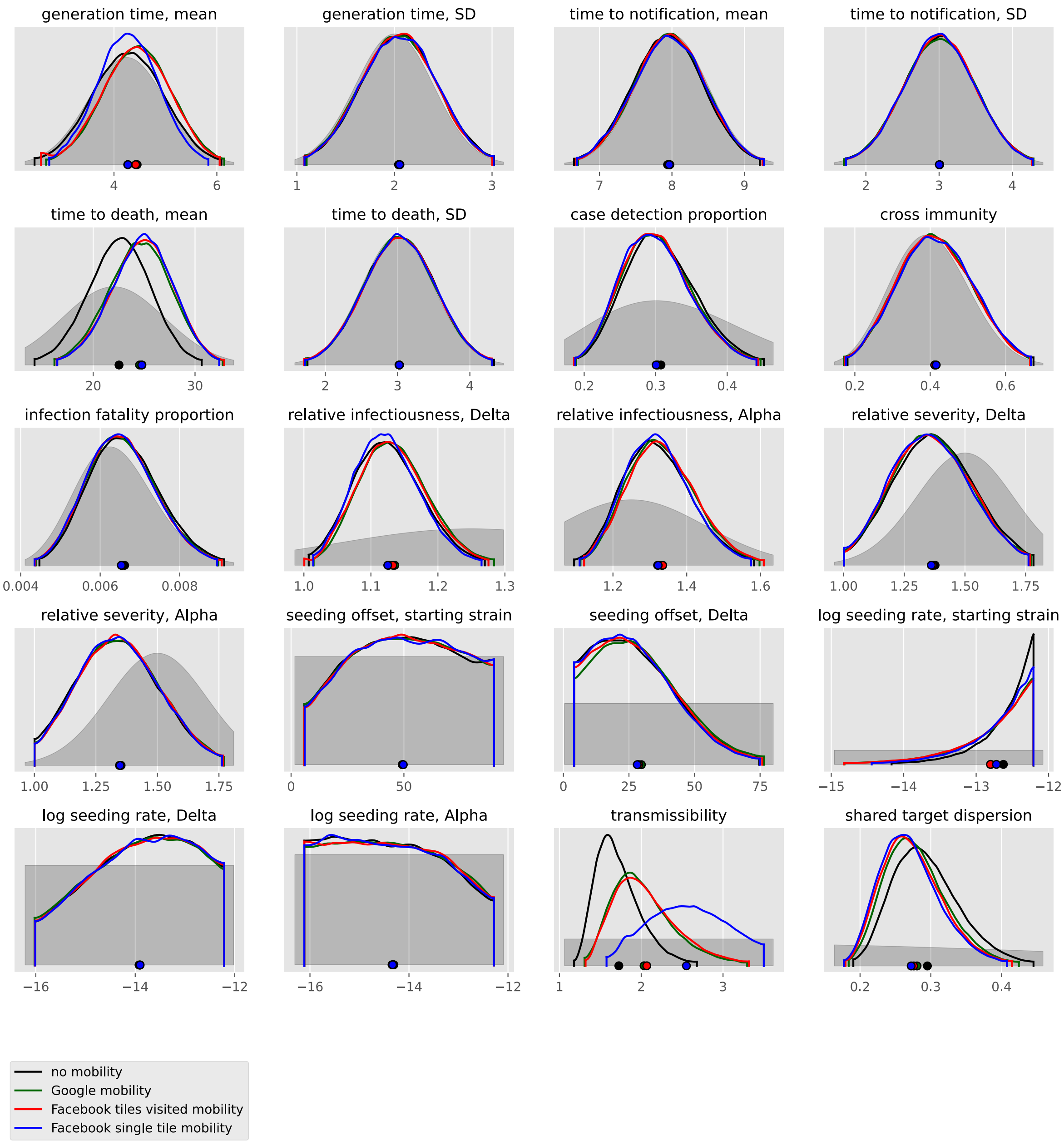

Netherlands

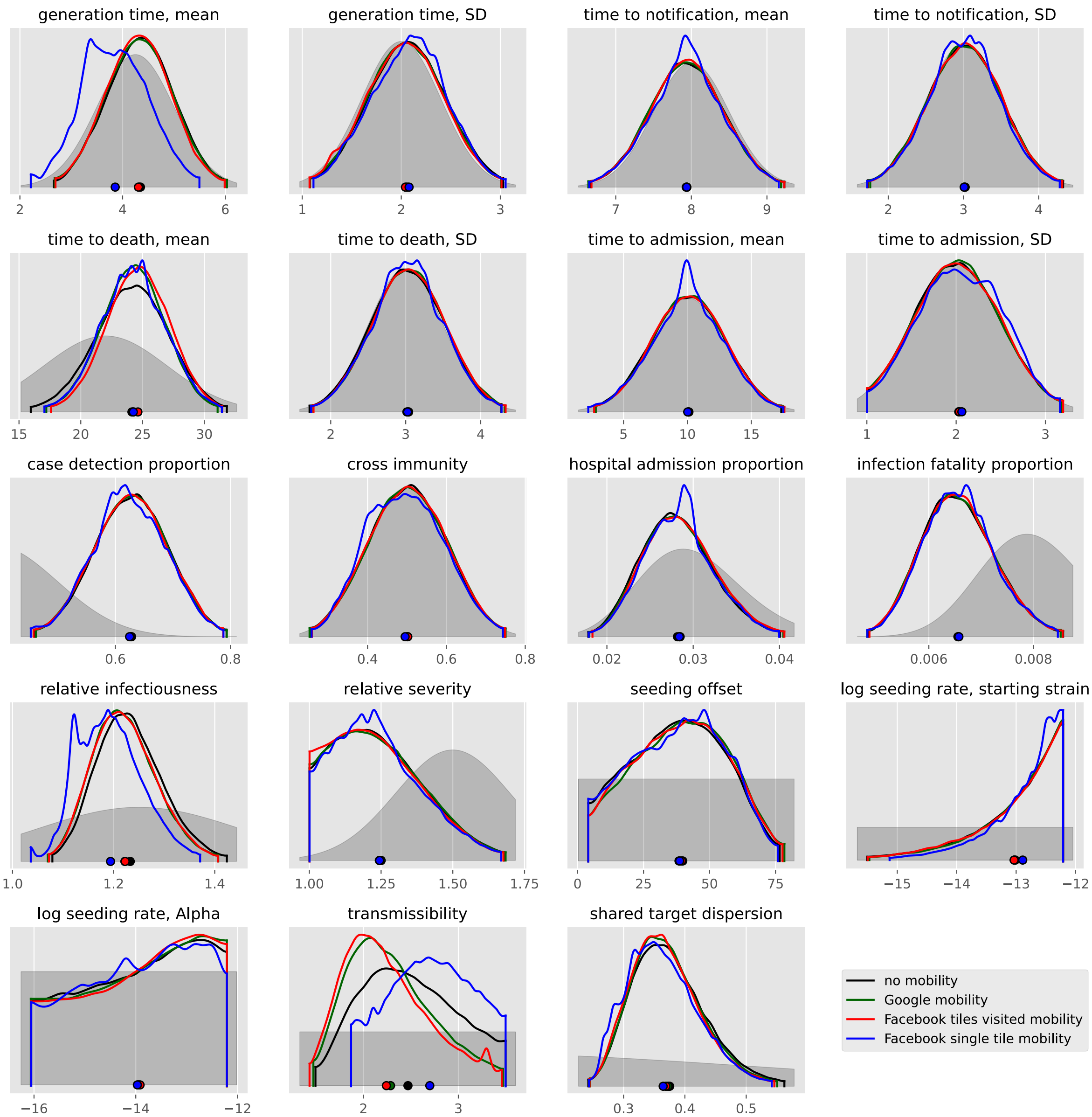

North Macedonia

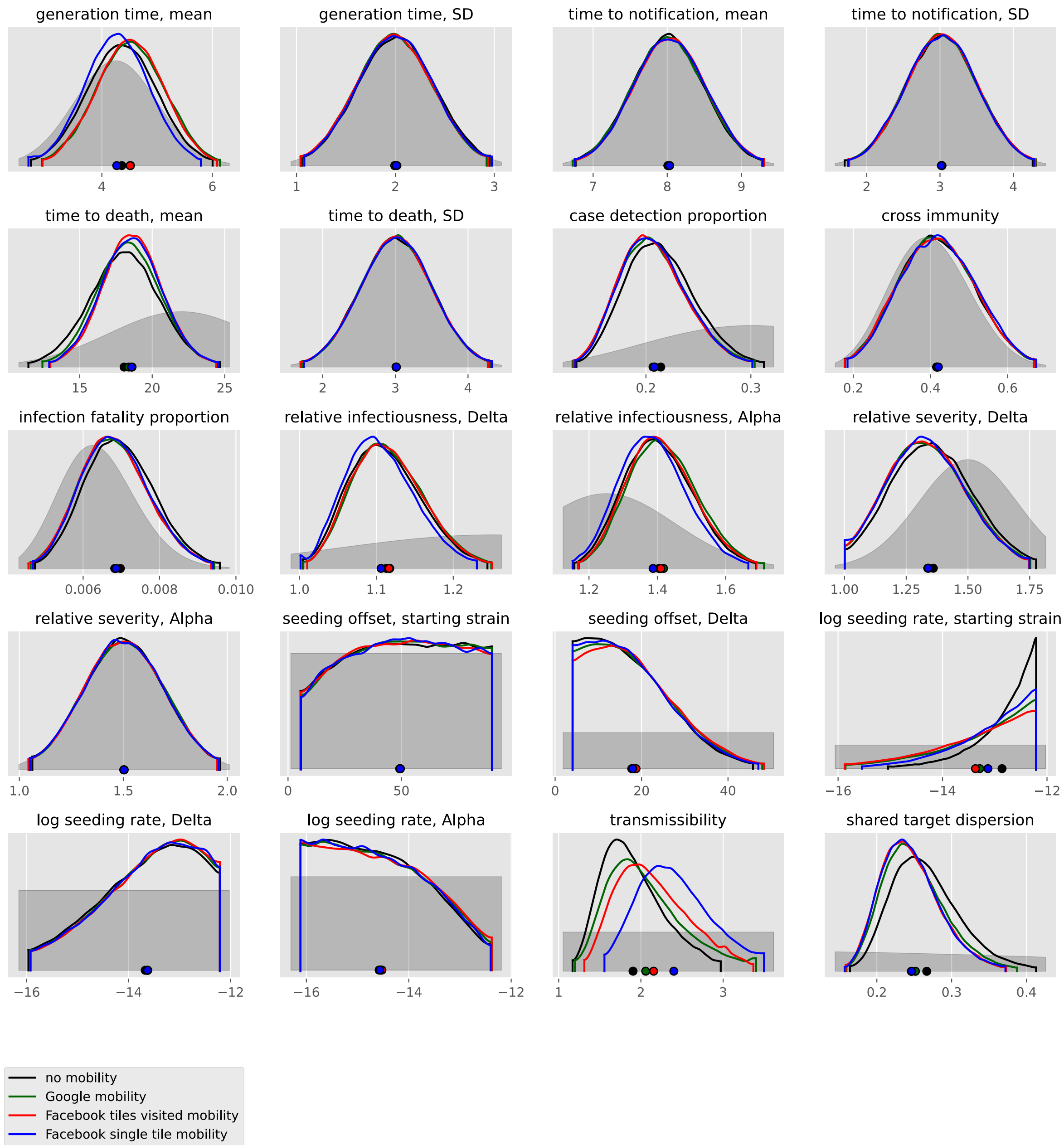

Norway

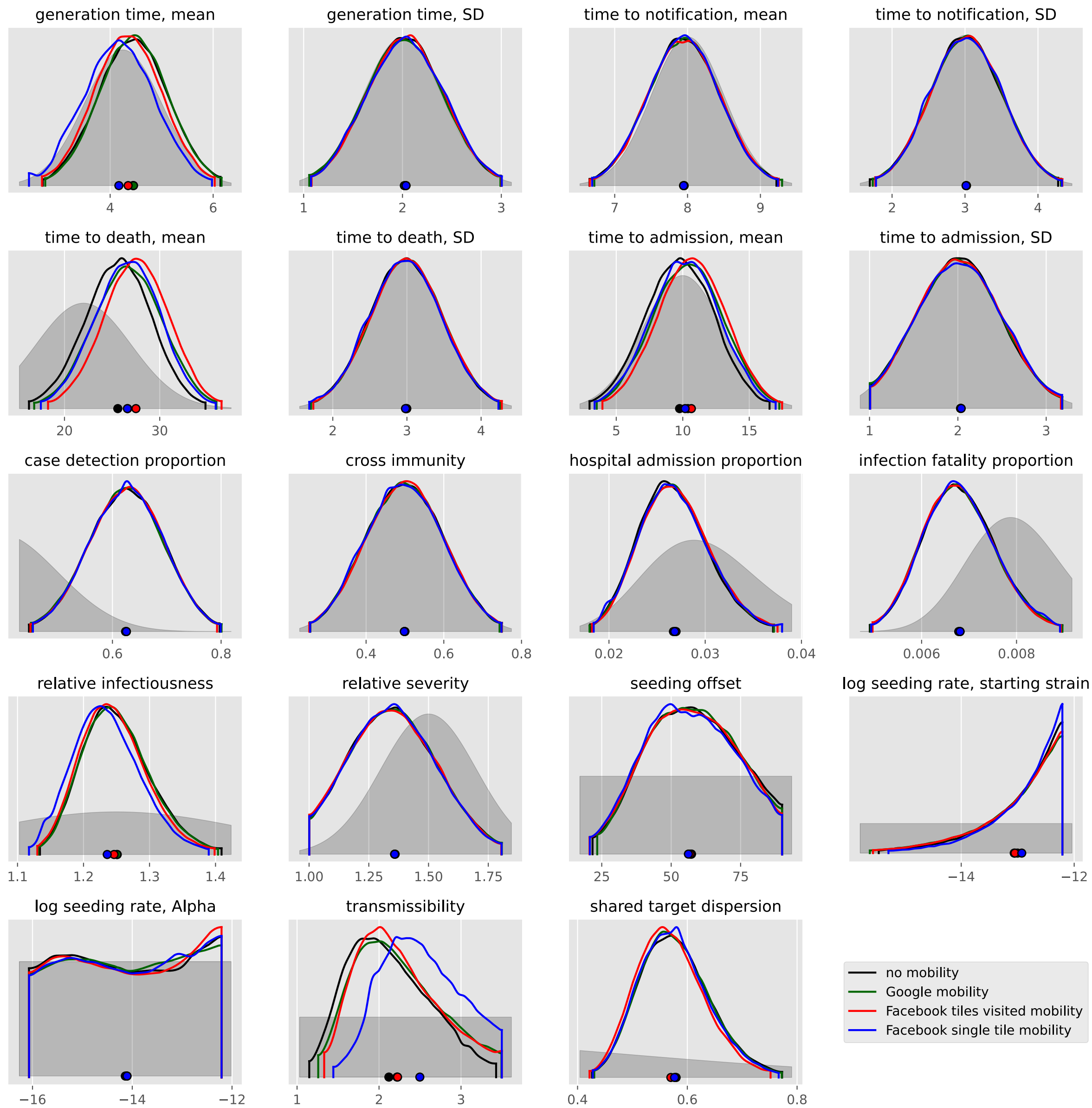

generation time, mean
generation time, SD
time to notification, mean
time to notification, SD
time to death, mean
time to death, SD
time to admission, mean
time to admission, SD
case detection proportion
cross immunity
hospital admission proportion
infection fatality proportion
relative infectiousness
relative severity
seeding offset
log seeding rate, starting strain
log seeding rate, Alpha
transmissibility
shared target dispersion
no mobility
Google mobility
Facebook tiles visited mobility
Facebook single tile mobility

Poland

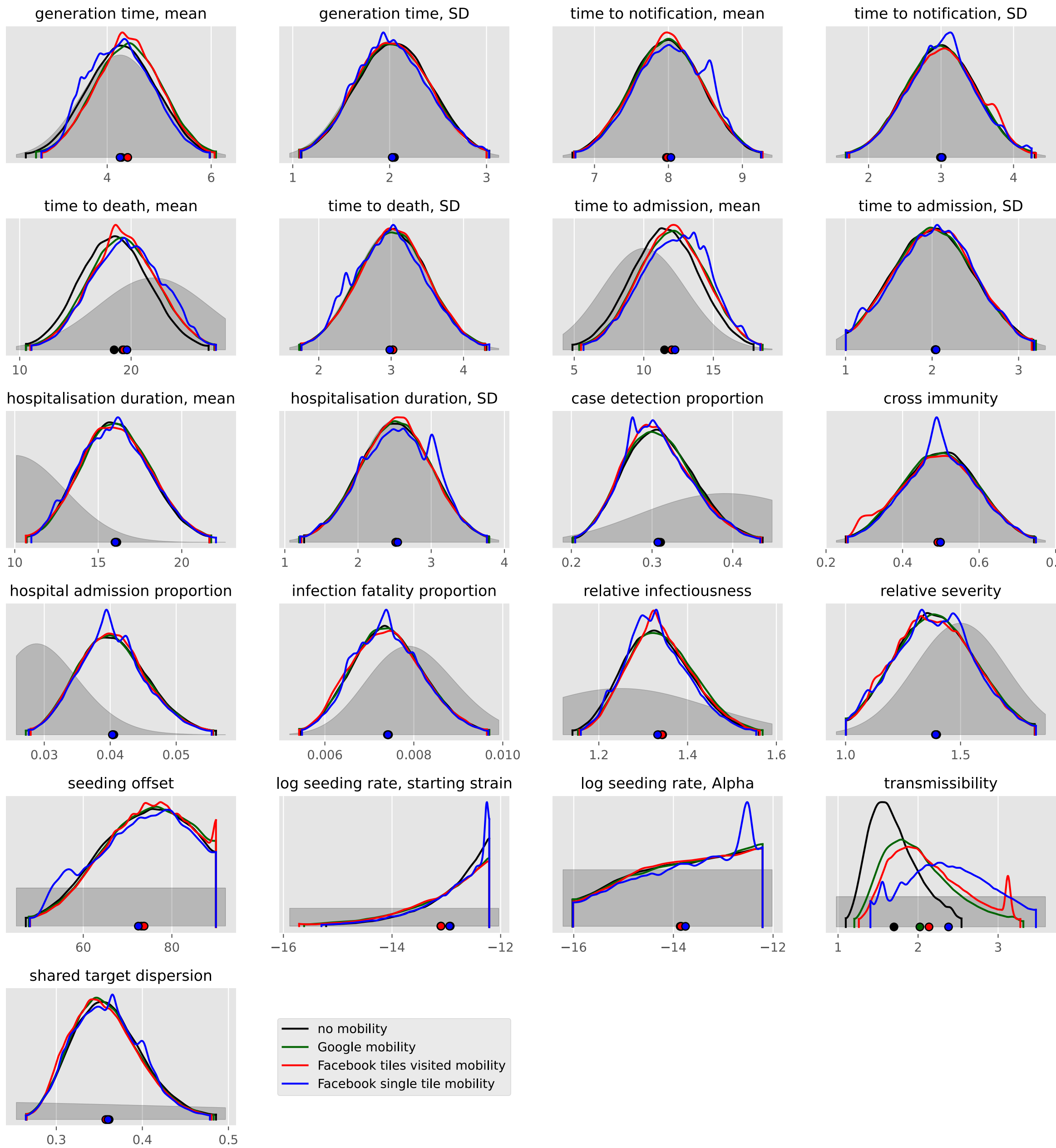
generation time, mean
generation time, SD
time to notification, mean
time to notification, SD
time to death, mean
time to death, SD
time to admission, mean
time to admission, SD
hospitalisation duration, mean
hospitalisation duration, SD
case detection proportion
cross immunity
hospital admission proportion
infection fatality proportion
relative infectiousness
relative severity
seeding offset
log seeding rate, starting strain
log seeding rate, Alpha
transmissibility
shared target dispersion
no mobility
Google mobility
Facebook tiles visited mobility
Facebook single tile mobility

Portugal

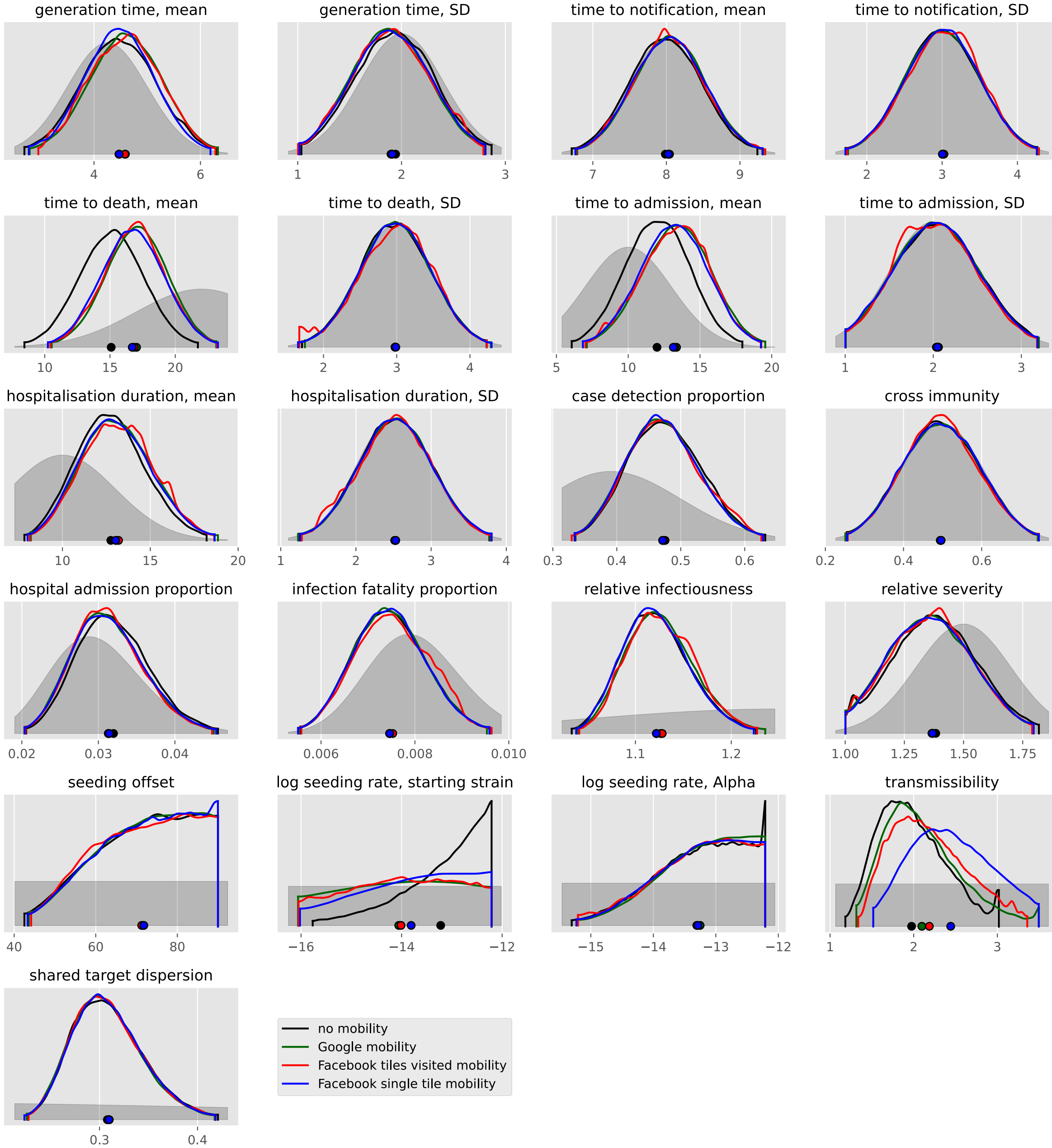

generation time, mean
generation time, SD
time to notification, mean
time to notification, SD
time to death, mean
time to death, SD
time to admission, mean
time to admission, SD
hospitalisation duration, mean
hospitalisation duration, SD
case detection proportion
cross immunity
hospital admission proportion
infection fatality proportion
relative infectiousness
relative severity
seeding offset
log seeding rate, starting strain
log seeding rate, Alpha
transmissibility
shared target dispersion
no mobility
Google mobility
Facebook tiles visited mobility
Facebook single tile mobility

Romania

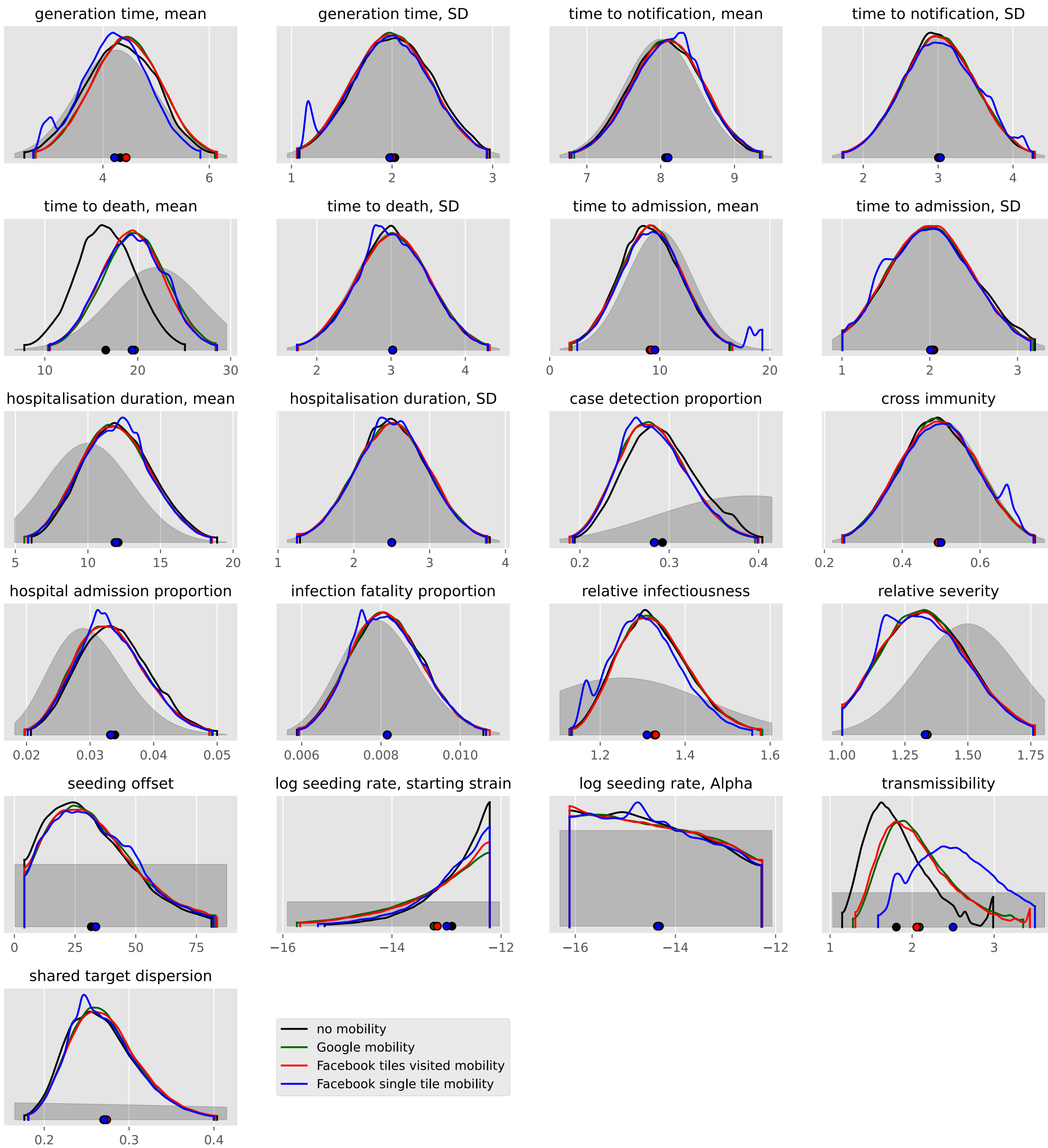
generation time, mean
4 6
generation time, SD
1 2 3
time to notification, mean
7 8 9
time to notification, SD
2 3 4
time to death, mean
10 20 30
time to death, SD
2 3 4
time to admission, mean
0 10 20
time to admission, SD
1 2 3
hospitalisation duration, mean
5 10 15 20
hospitalisation duration, SD
1 2 3 4
case detection proportion
0.2 0.3 0.4
cross immunity
0.2 0.4 0.6
hospital admission proportion
0.02 0.03 0.04 0.05
infection fatality proportion
0.006 0.008 0.010
relative infectiousness
1.2 1.4 1.6
relative severity
1.00 1.25 1.50 1.75
seeding offset
0 25 50 75
log seeding rate, starting strain
−16 −14 −12
log seeding rate, Alpha
−16 −14 −12
transmissibility
1 2 3
shared target dispersion
0.2 0.3 0.4
no mobility
Google mobility
Facebook tiles visited mobility
Facebook single tile mobility

Russian Federation

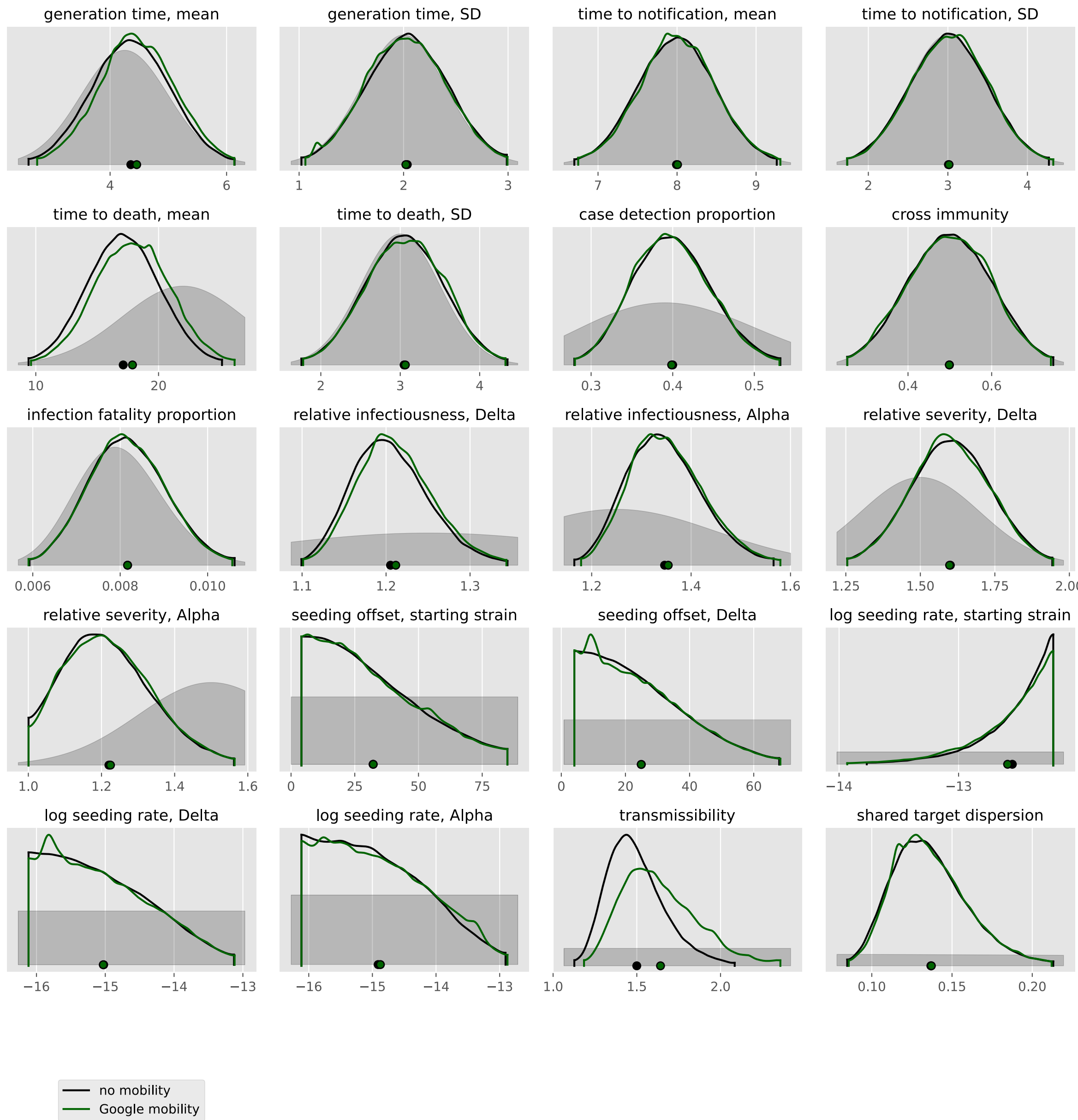

Serbia

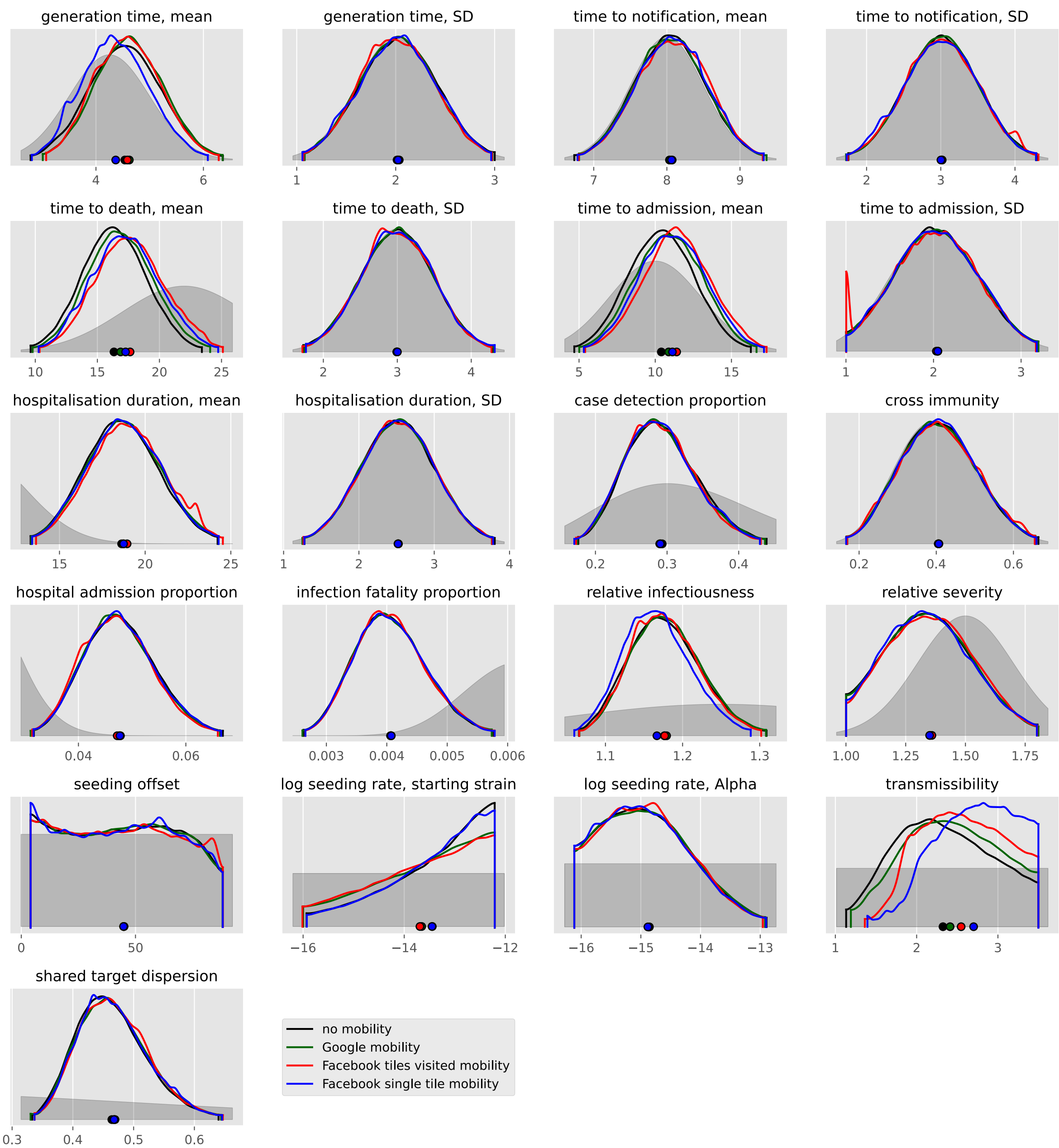

generation time, mean
generation time, SD
time to notification, mean
time to notification, SD
time to death, mean
time to death, SD
time to admission, mean
time to admission, SD
hospitalisation duration, mean
hospitalisation duration, SD
case detection proportion
cross immunity
hospital admission proportion
infection fatality proportion
relative infectiousness
relative severity
seeding offset
log seeding rate, starting strain
log seeding rate, Alpha
transmissibility
shared target dispersion
no mobility
Google mobility
Facebook tiles visited mobility
Facebook single tile mobility

Slovakia

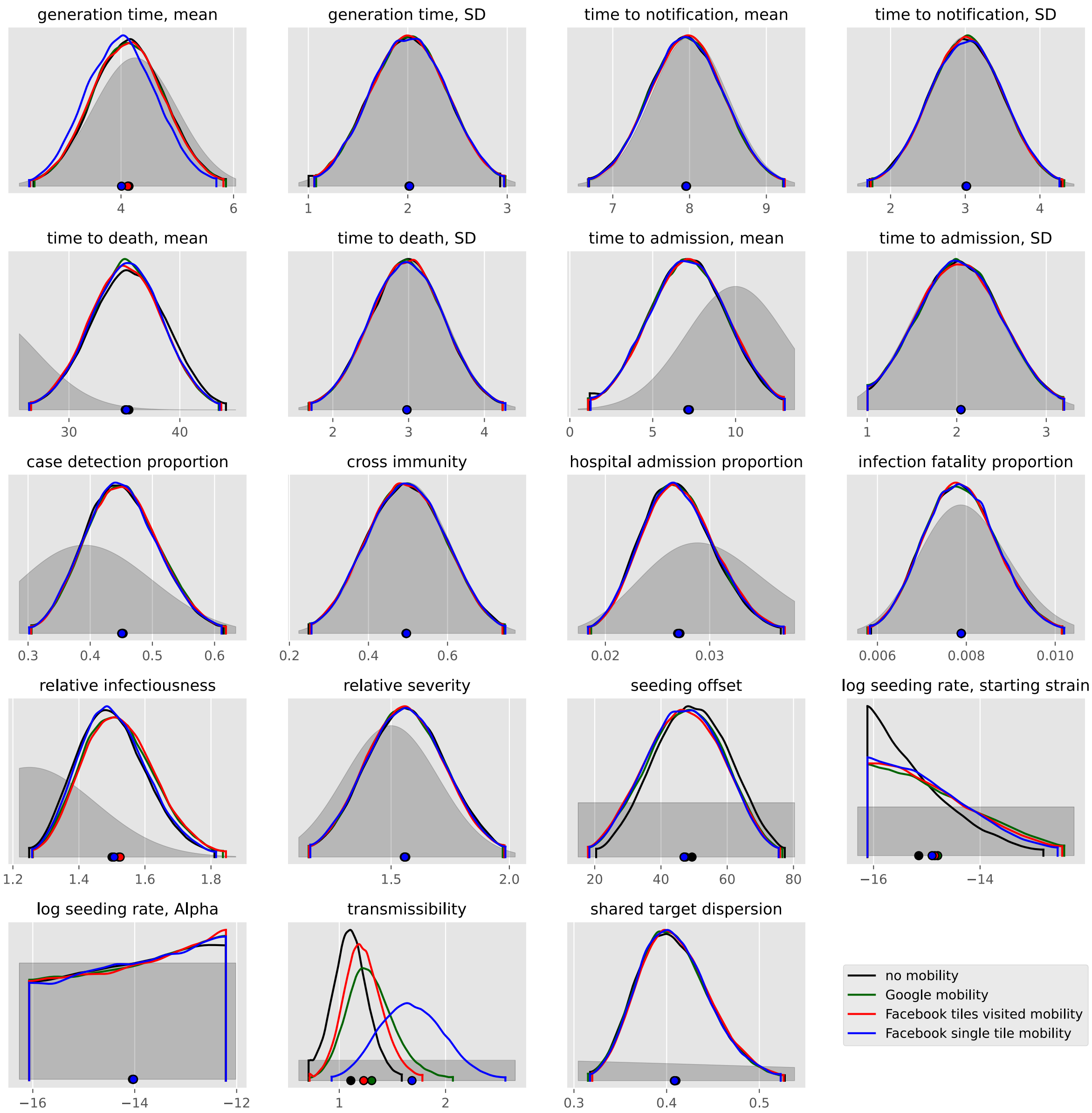
generation time, mean
generation time, SD
time to notification, mean
time to notification, SD
time to death, mean
time to death, SD
time to admission, mean
time to admission, SD
case detection proportion
cross immunity
hospital admission proportion
infection fatality proportion
relative infectiousness
relative severity
seeding offset
log seeding rate, starting strain
log seeding rate, Alpha
transmissibility
shared target dispersion
no mobility
Google mobility
Facebook tiles visited mobility
Facebook single tile mobility

Slovenia

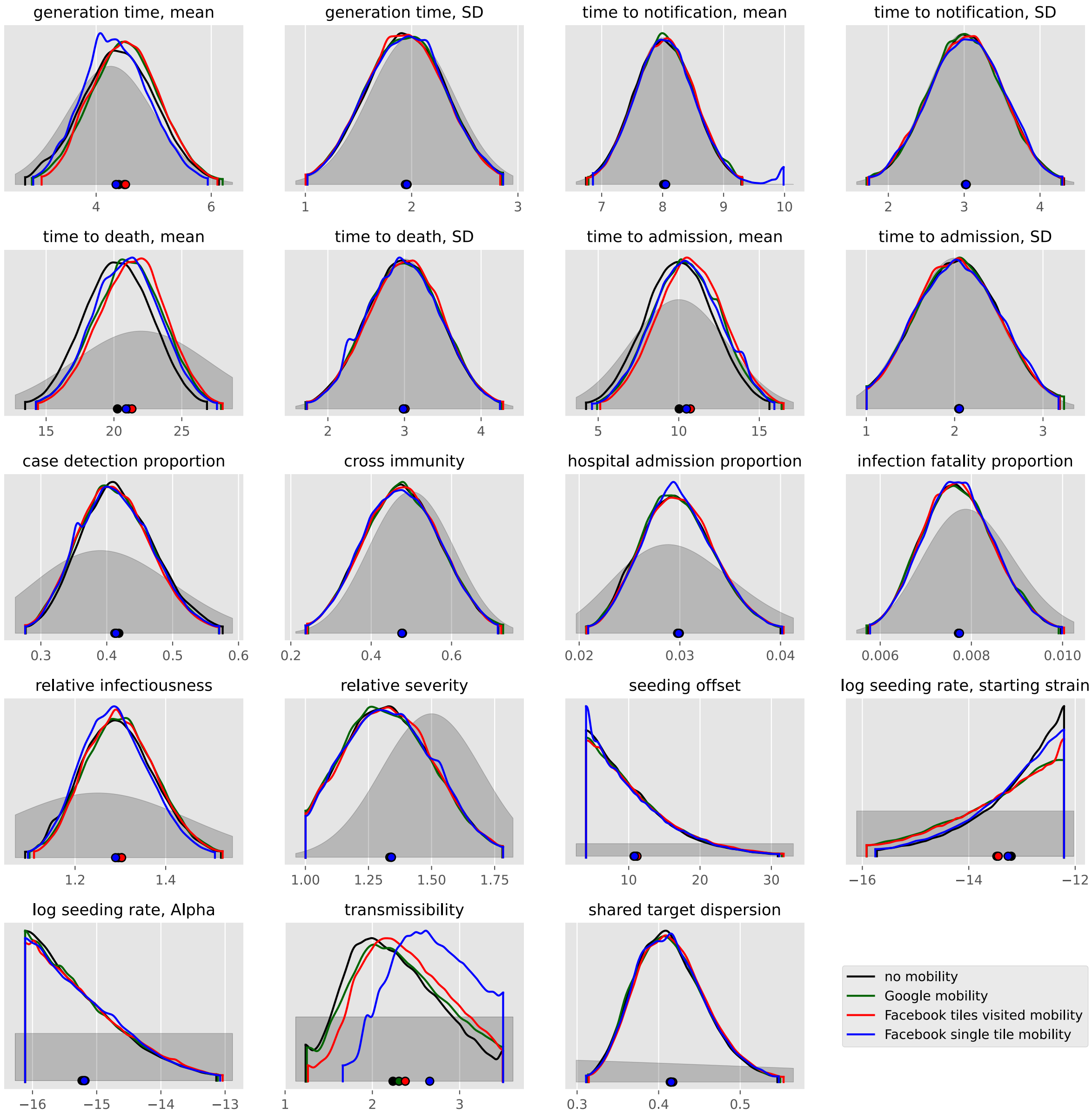
generation time, mean
generation time, SD
time to notification, mean
time to notification, SD
time to death, mean
time to death, SD
time to admission, mean
time to admission, SD
case detection proportion
cross immunity
hospital admission proportion
infection fatality proportion
relative infectiousness
relative severity
seeding offset
log seeding rate, starting strain
log seeding rate, Alpha
transmissibility
shared target dispersion
no mobility
Google mobility
Facebook tiles visited mobility
Facebook single tile mobility

Spain

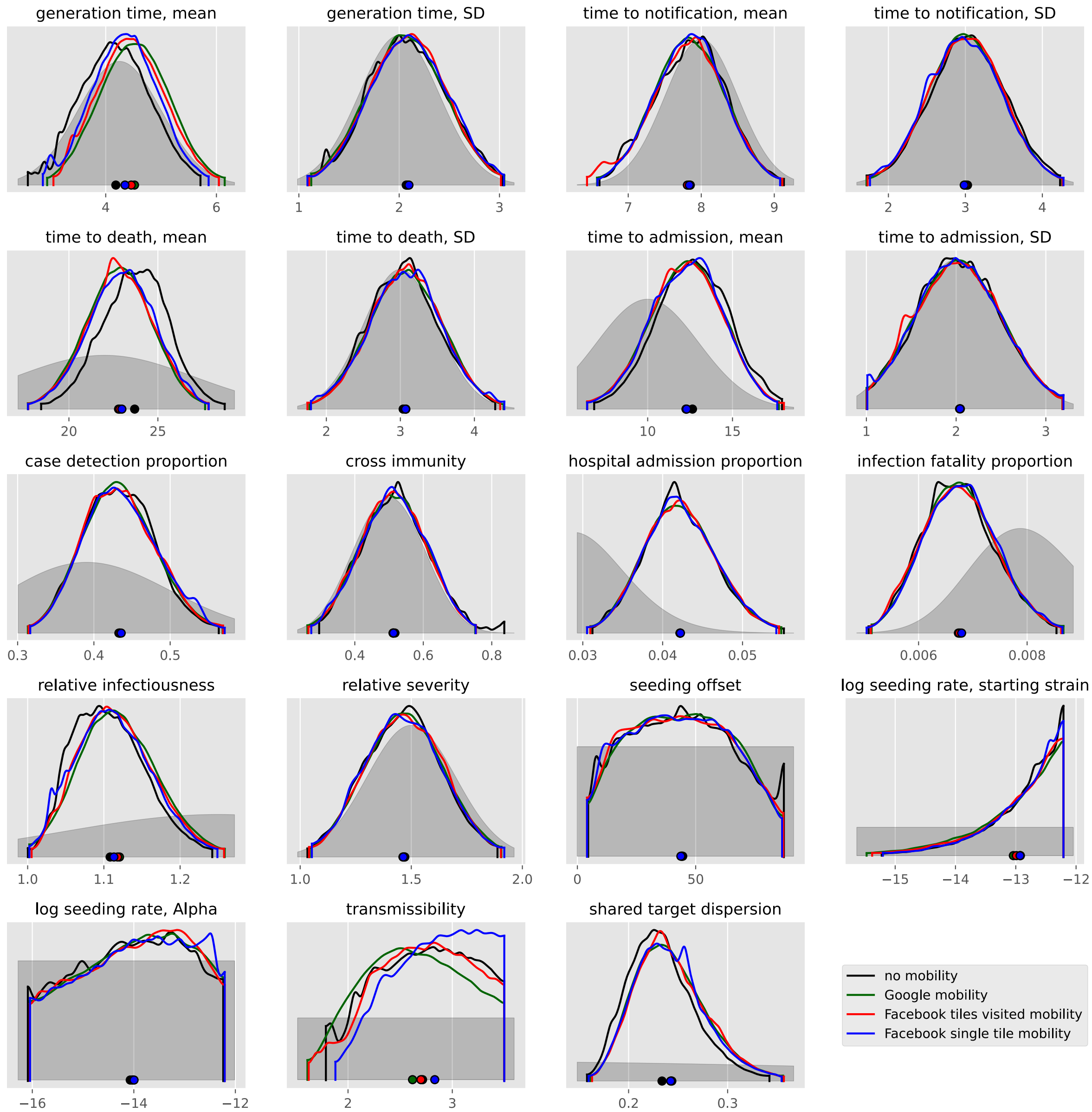

generation time, mean
4
6
generation time, SD
1
2
3
time to notification, mean
7
8
9
time to notification, SD
2
3
4
time to death, mean
20
25
time to death, SD
2
3
4
time to admission, mean
10
15
time to admission, SD
1
2
3
case detection proportion
0.3
0.4
0.5
cross immunity
0.4
0.6
0.8
hospital admission proportion
0.03
0.04
0.05
infection fatality proportion
0.006
0.008
relative infectiousness
1.0
1.1
1.2
relative severity
1.0
1.5
2.0
seeding offset
0
50
log seeding rate, starting strain
−15
−14
−13
−12
log seeding rate, Alpha
−16
−14
−12
transmissibility
2
3
shared target dispersion
0.2
0.3
no mobility
Google mobility
Facebook tiles visited mobility
Facebook single tile mobility

Sweden

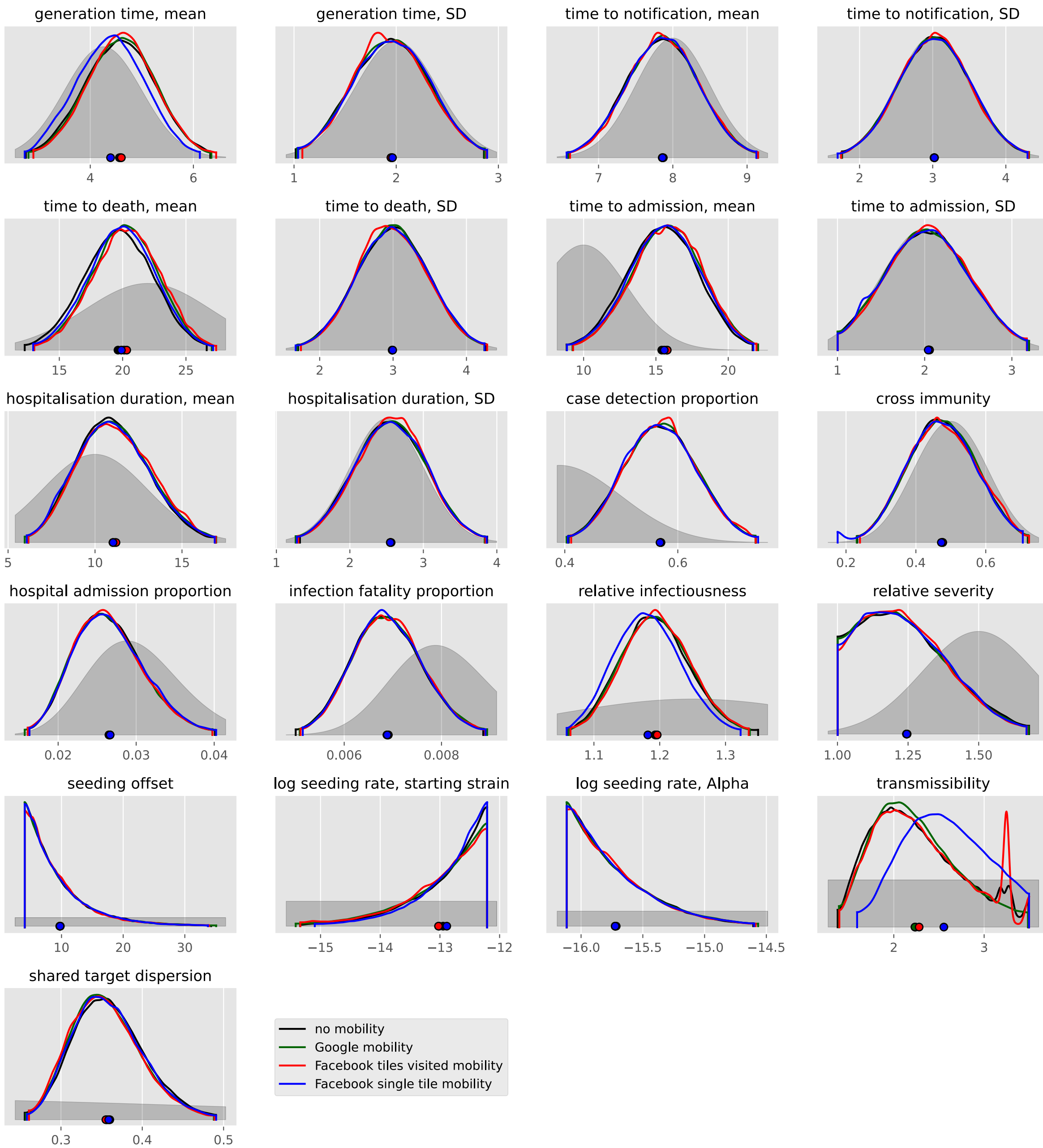
generation time, mean
generation time, SD
time to notification, mean
time to notification, SD
time to death, mean
time to death, SD
time to admission, mean
time to admission, SD
hospitalisation duration, mean
hospitalisation duration, SD
case detection proportion
cross immunity
hospital admission proportion
infection fatality proportion
relative infectiousness
relative severity
seeding offset
log seeding rate, starting strain
log seeding rate, Alpha
transmissibility
shared target dispersion
no mobility
Google mobility
Facebook tiles visited mobility
Facebook single tile mobility

Switzerland

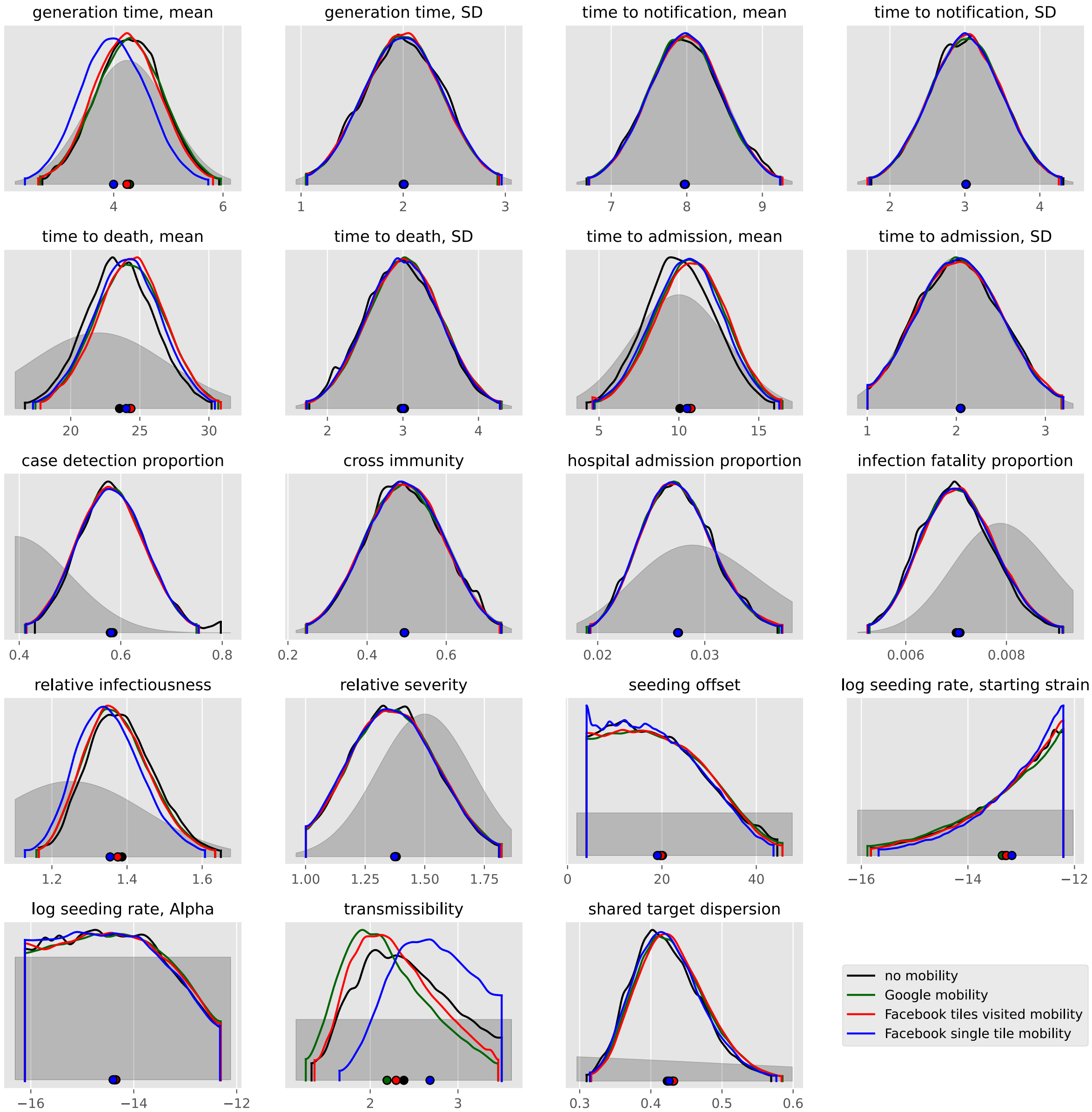

Ukraine

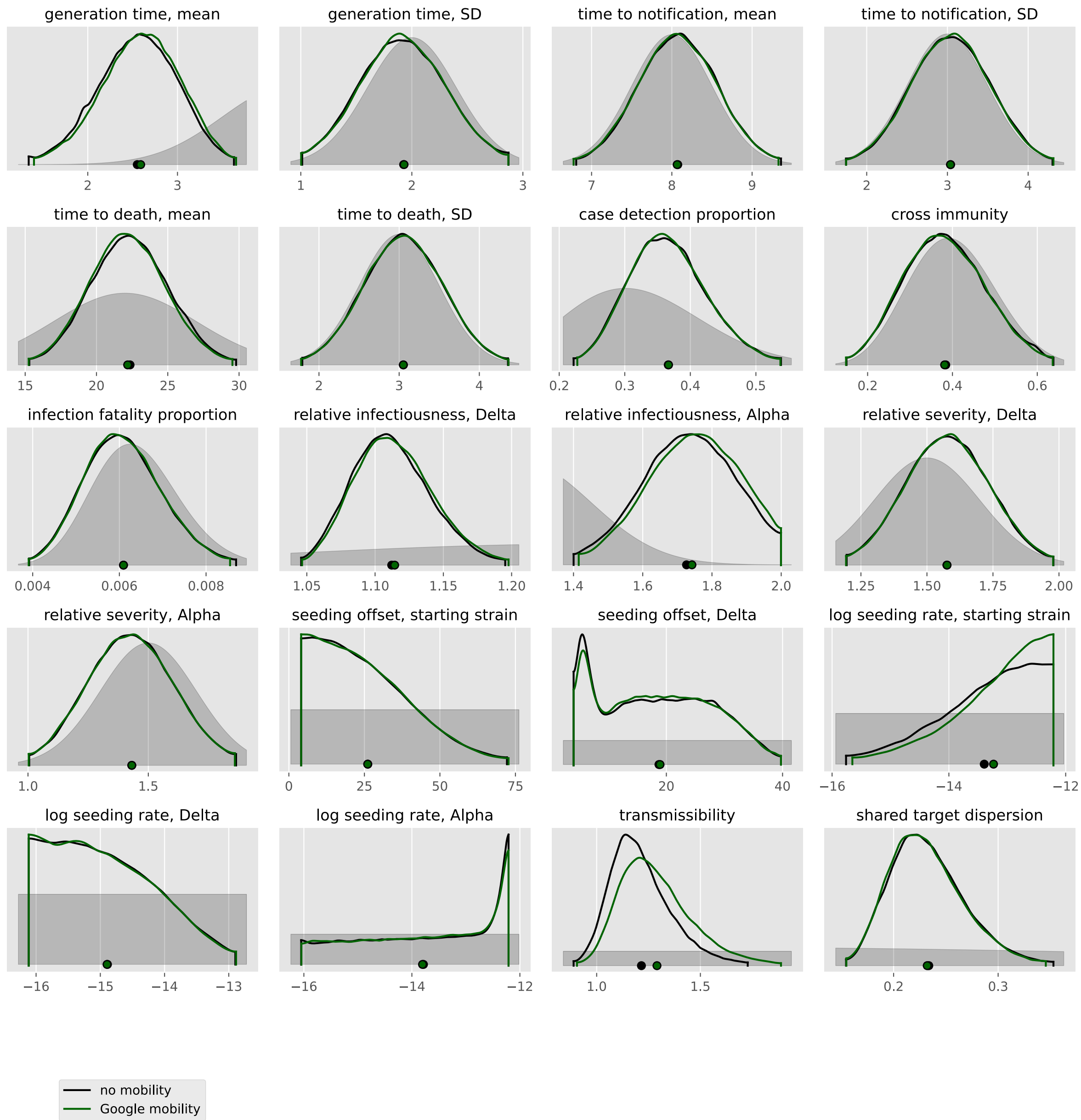
generation time, mean
generation time, SD
time to notification, mean
time to notification, SD
time to death, mean
time to death, SD
case detection proportion
cross immunity
infection fatality proportion
relative infectiousness, Delta
relative infectiousness, Alpha
relative severity, Delta
relative severity, Alpha
seeding offset, starting strain
seeding offset, Delta
log seeding rate, starting strain
log seeding rate, Delta
log seeding rate, Alpha
transmissibility
shared target dispersion
no mobility
Google mobility

United Kingdom

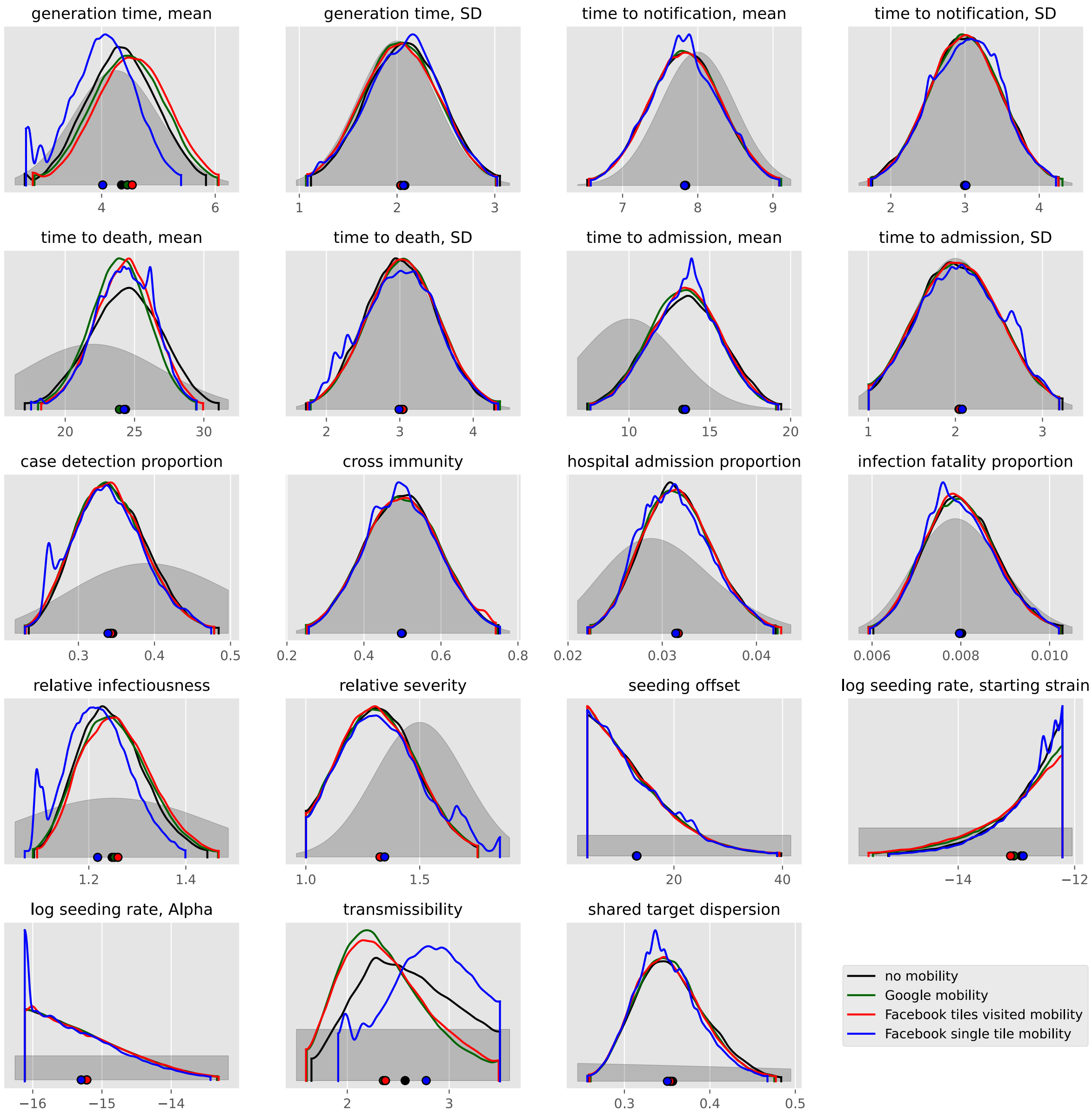

North America

Aruba

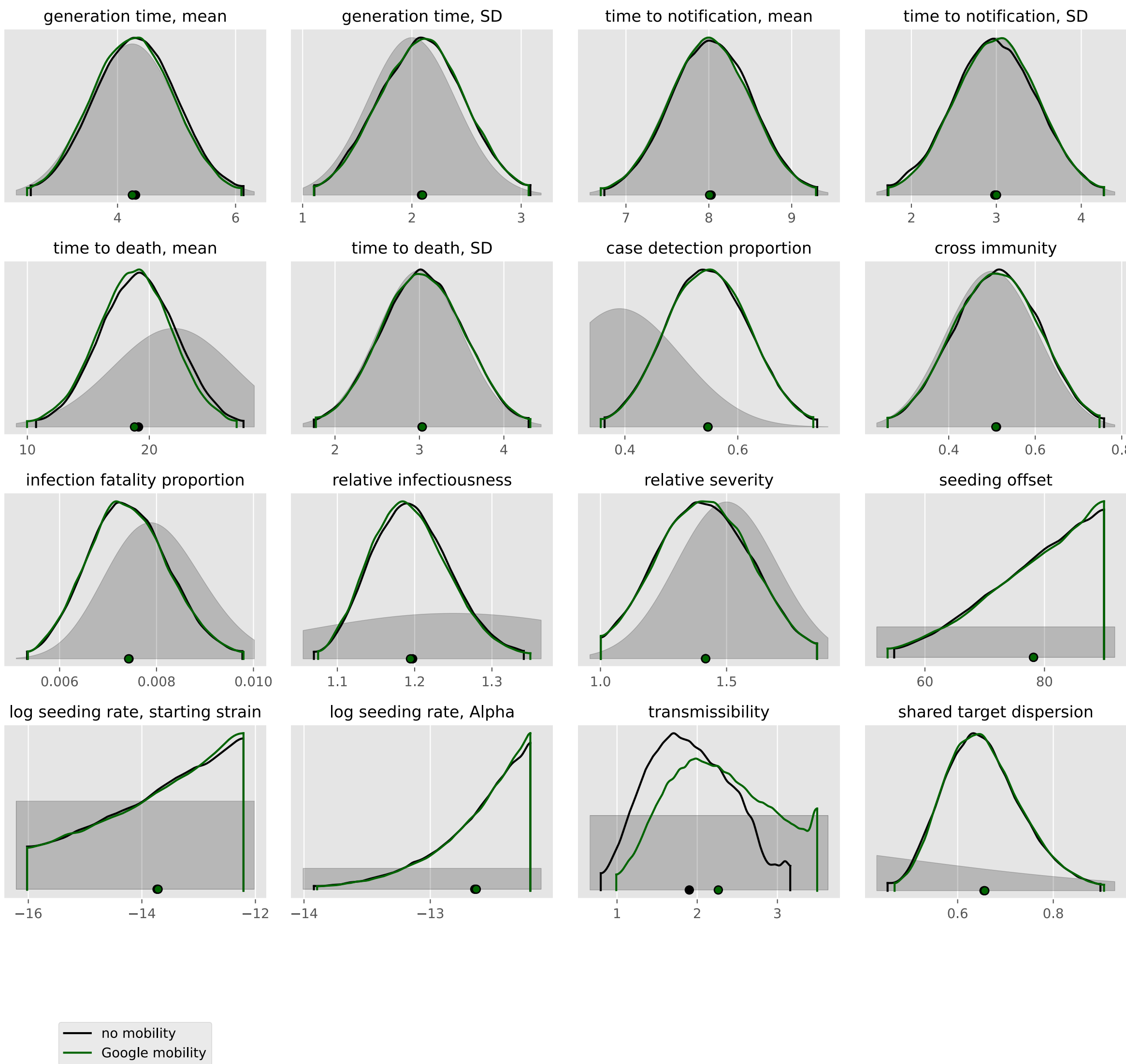

Bahamas

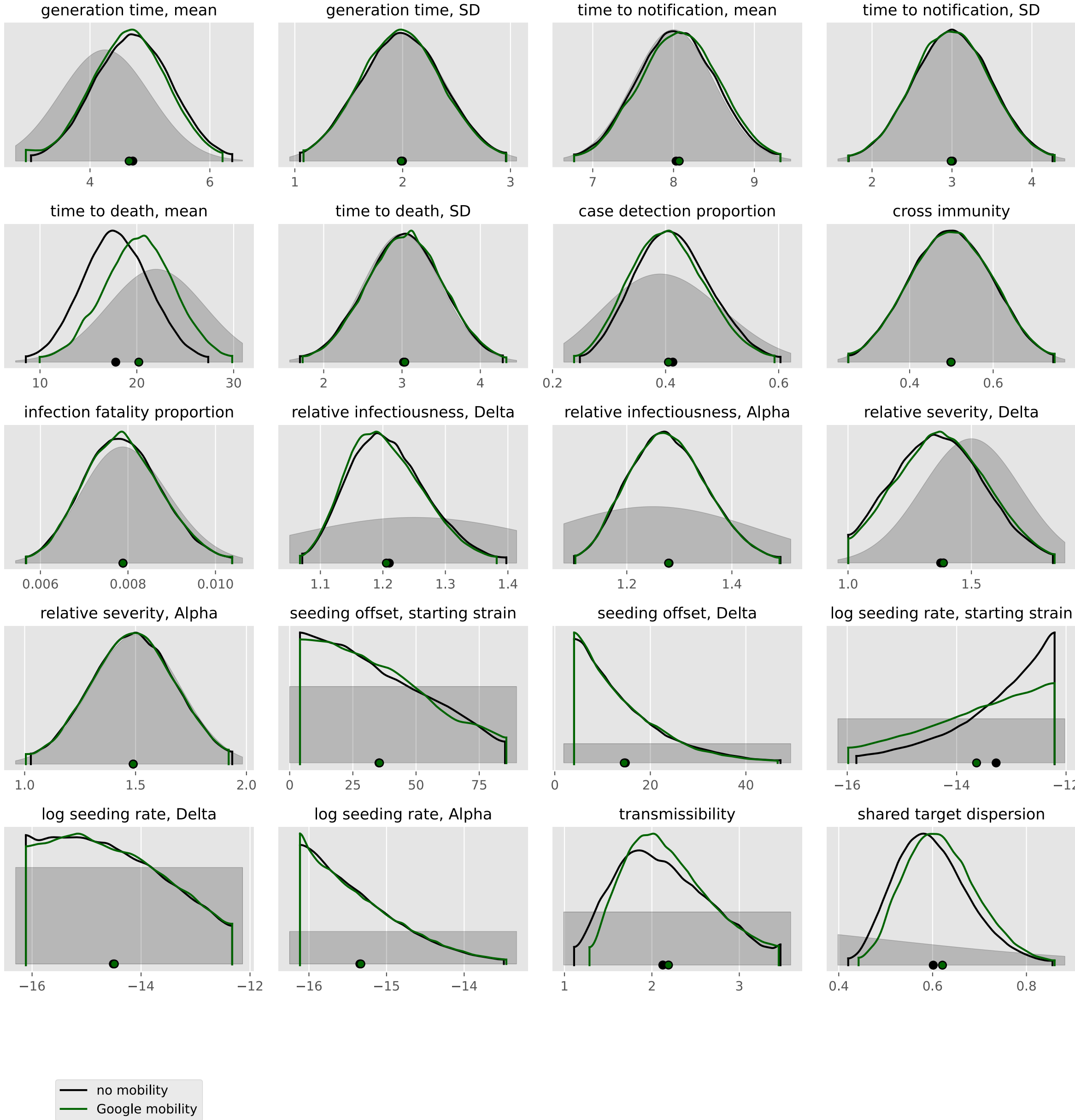

generation time, mean
generation time, SD
time to notification, mean
time to notification, SD
time to death, mean
time to death, SD
case detection proportion
cross immunity
infection fatality proportion
relative infectiousness, Delta
relative infectiousness, Alpha
relative severity, Delta
relative severity, Alpha
seeding offset, starting strain
seeding offset, Delta
log seeding rate, starting strain
log seeding rate, Delta
log seeding rate, Alpha
transmissibility
shared target dispersion
no mobility
Google mobility

Canada

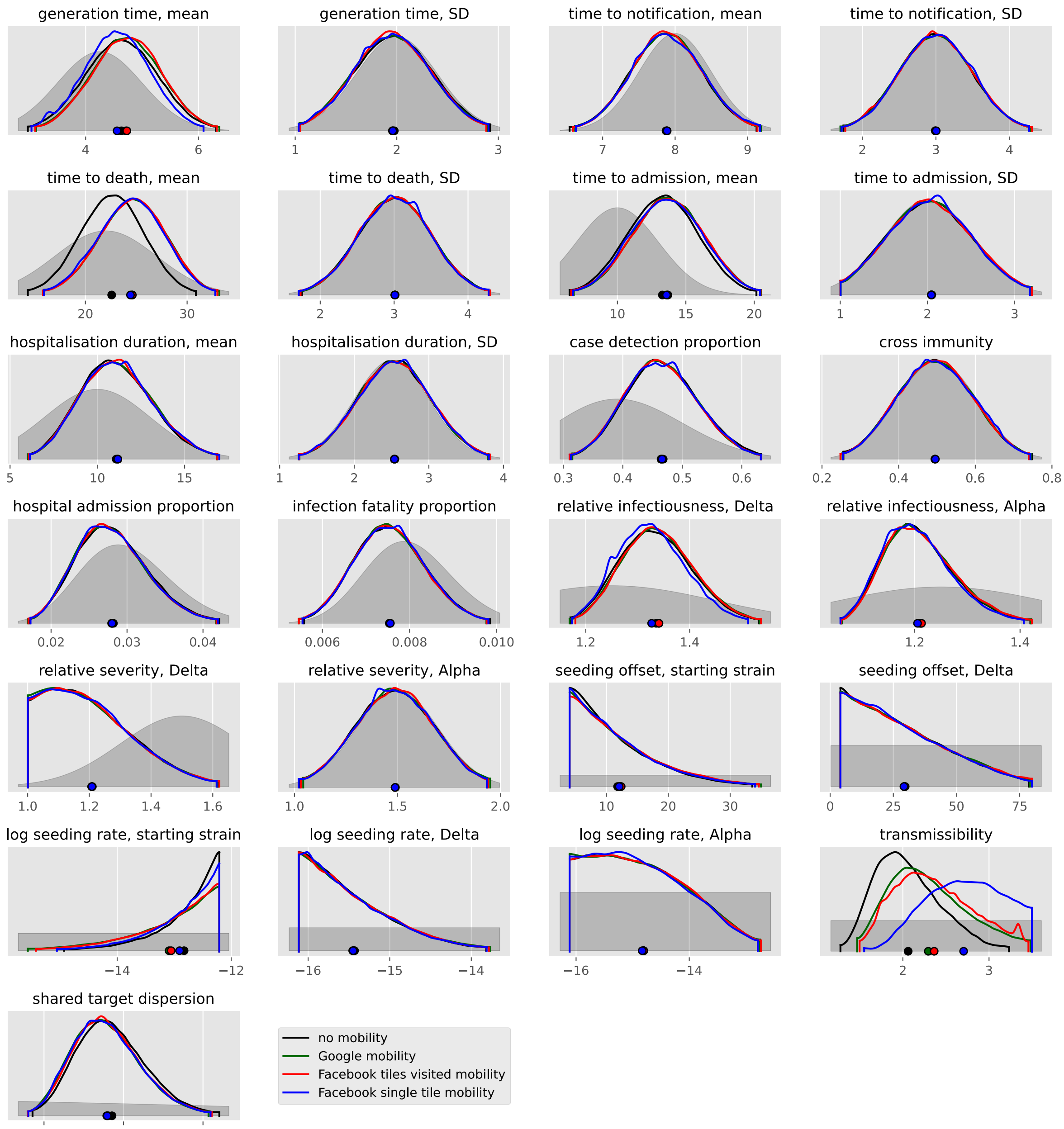

generation time, mean
generation time, SD
time to notification, mean
time to notification, SD
time to death, mean
time to death, SD
time to admission, mean
time to admission, SD
hospitalisation duration, mean
hospitalisation duration, SD
case detection proportion
cross immunity
hospital admission proportion
infection fatality proportion
relative infectiousness, Delta
relative infectiousness, Alpha
relative severity, Delta
relative severity, Alpha
seeding offset, starting strain
seeding offset, Delta
log seeding rate, starting strain
log seeding rate, Delta
log seeding rate, Alpha
transmissibility
shared target dispersion
no mobility
Google mobility
Facebook tiles visited mobility
Facebook single tile mobility

Costa Rica

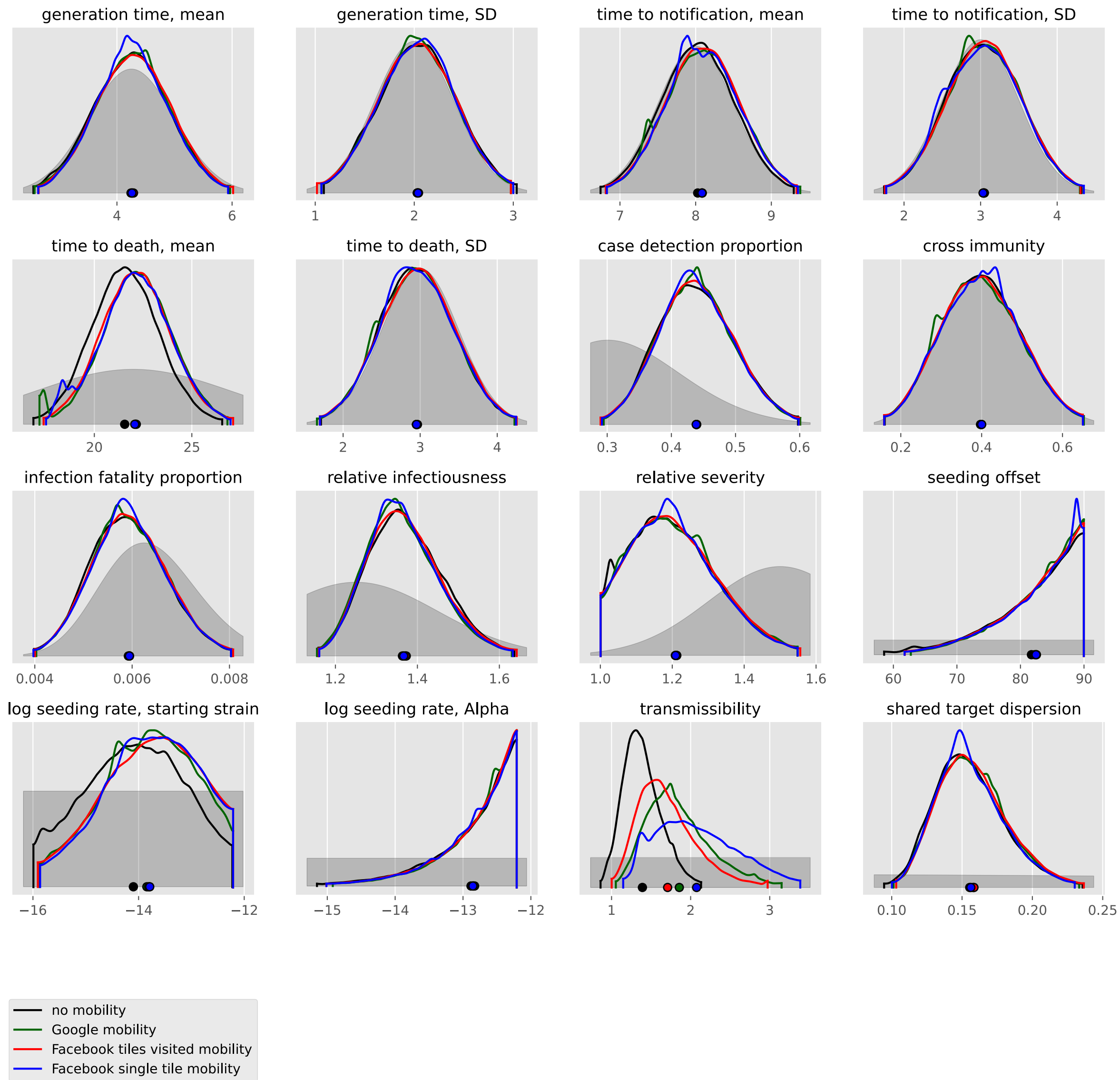
generation time, mean
generation time, SD
time to notification, mean
time to notification, SD
time to death, mean
time to death, SD
case detection proportion
cross immunity
infection fatality proportion
relative infectiousness
relative severity
seeding offset
log seeding rate, starting strain
log seeding rate, Alpha
transmissibility
shared target dispersion
no mobility
Google mobility
Facebook tiles visited mobility
Facebook single tile mobility

Dominican Republic

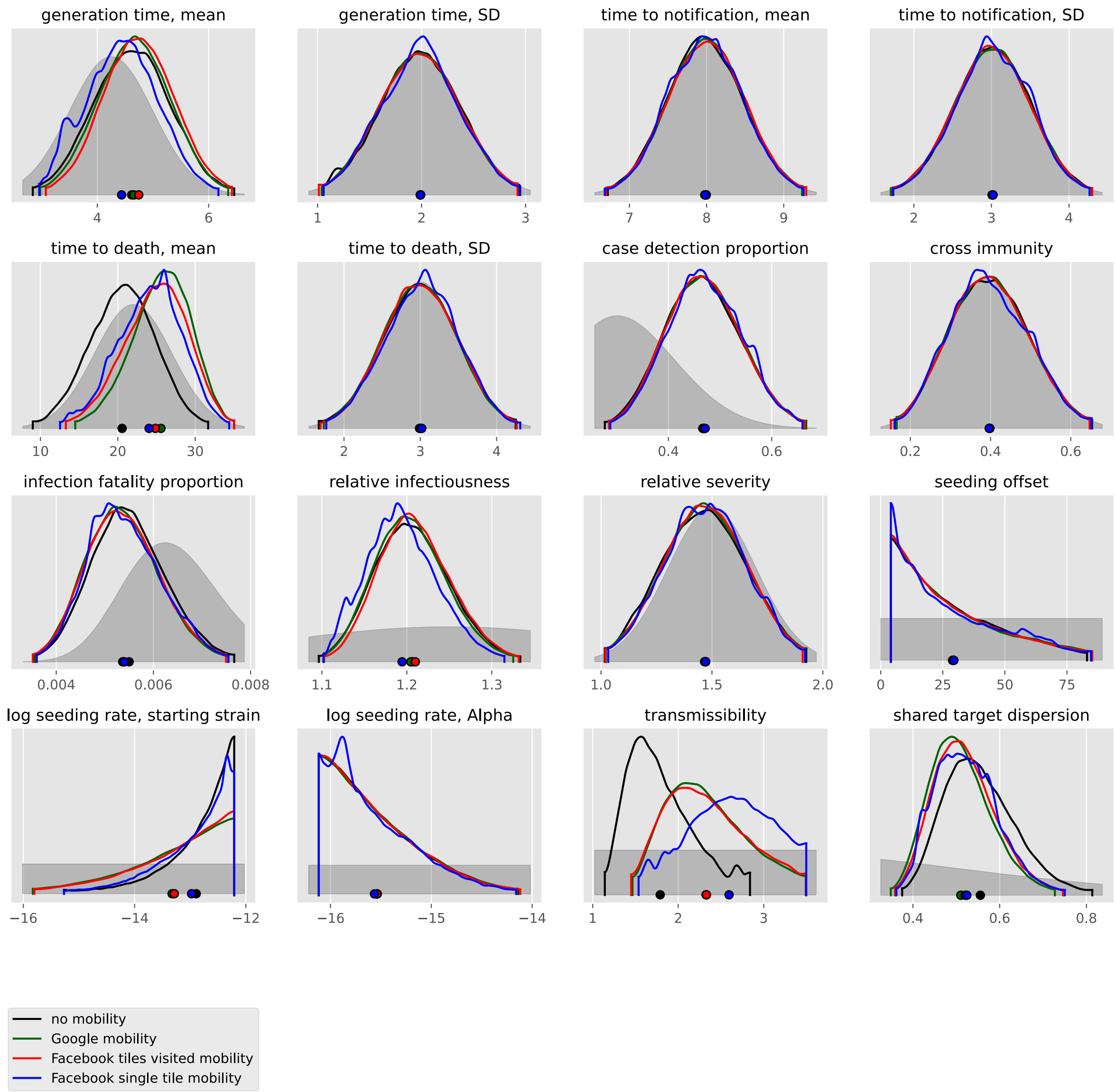

El Salvador

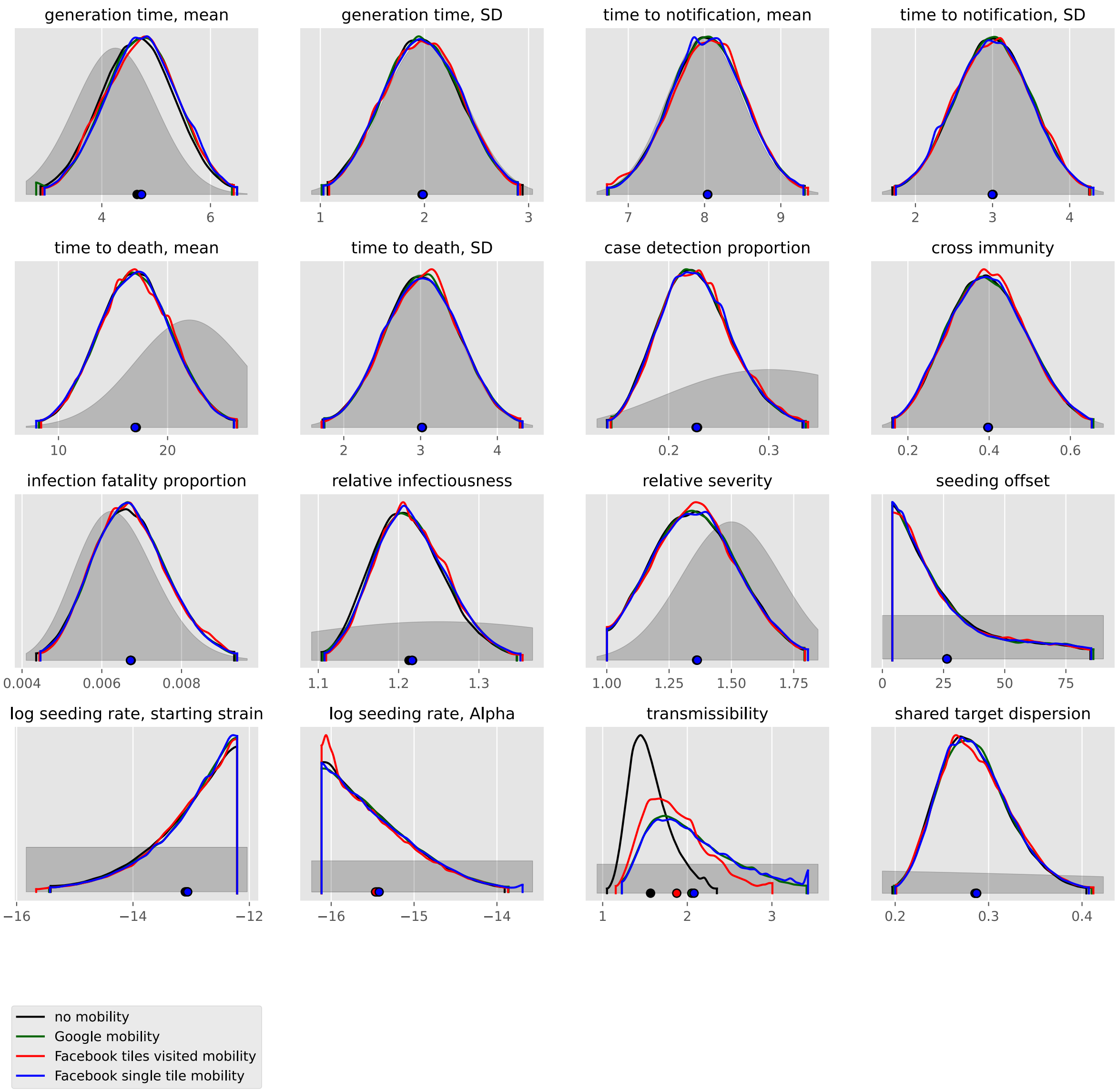

generation time, mean
generation time, SD
time to notification, mean
time to notification, SD
time to death, mean
time to death, SD
case detection proportion
cross immunity
infection fatality proportion
relative infectiousness
relative severity
seeding offset
log seeding rate, starting strain
log seeding rate, Alpha
transmissibility
shared target dispersion
no mobility
Google mobility
Facebook tiles visited mobility
Facebook single tile mobility

Haiti

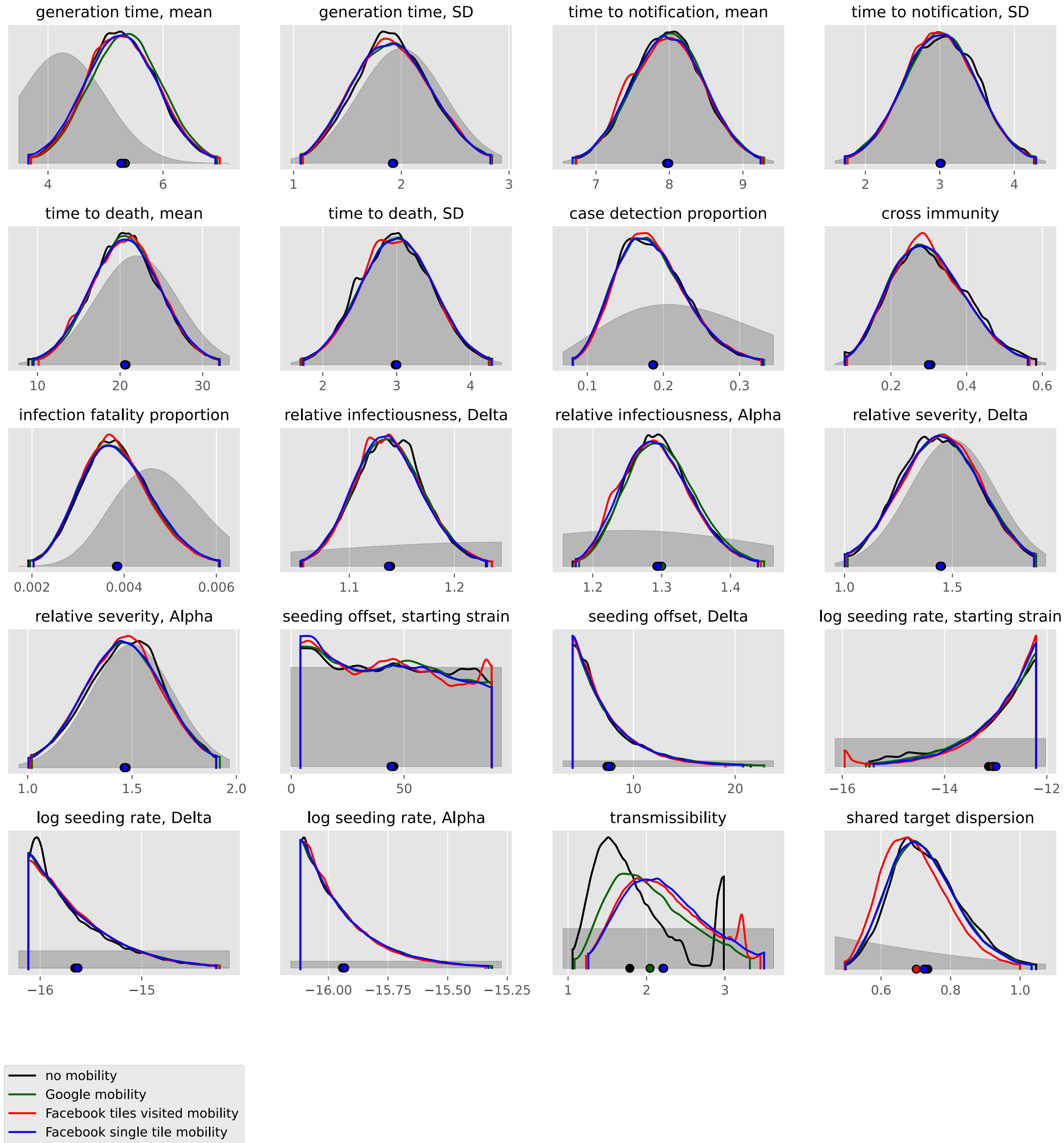

generation time, mean
generation time, SD
time to notification, mean
time to notification, SD
time to death, mean
time to death, SD
case detection proportion
cross immunity
infection fatality proportion
relative infectiousness, Delta
relative infectiousness, Alpha
relative severity, Delta
relative severity, Alpha
seeding offset, starting strain
seeding offset, Delta
log seeding rate, starting strain
log seeding rate, Delta
log seeding rate, Alpha
transmissibility
shared target dispersion
no mobility
Google mobility
Facebook tiles visited mobility
Facebook single tile mobility

Honduras

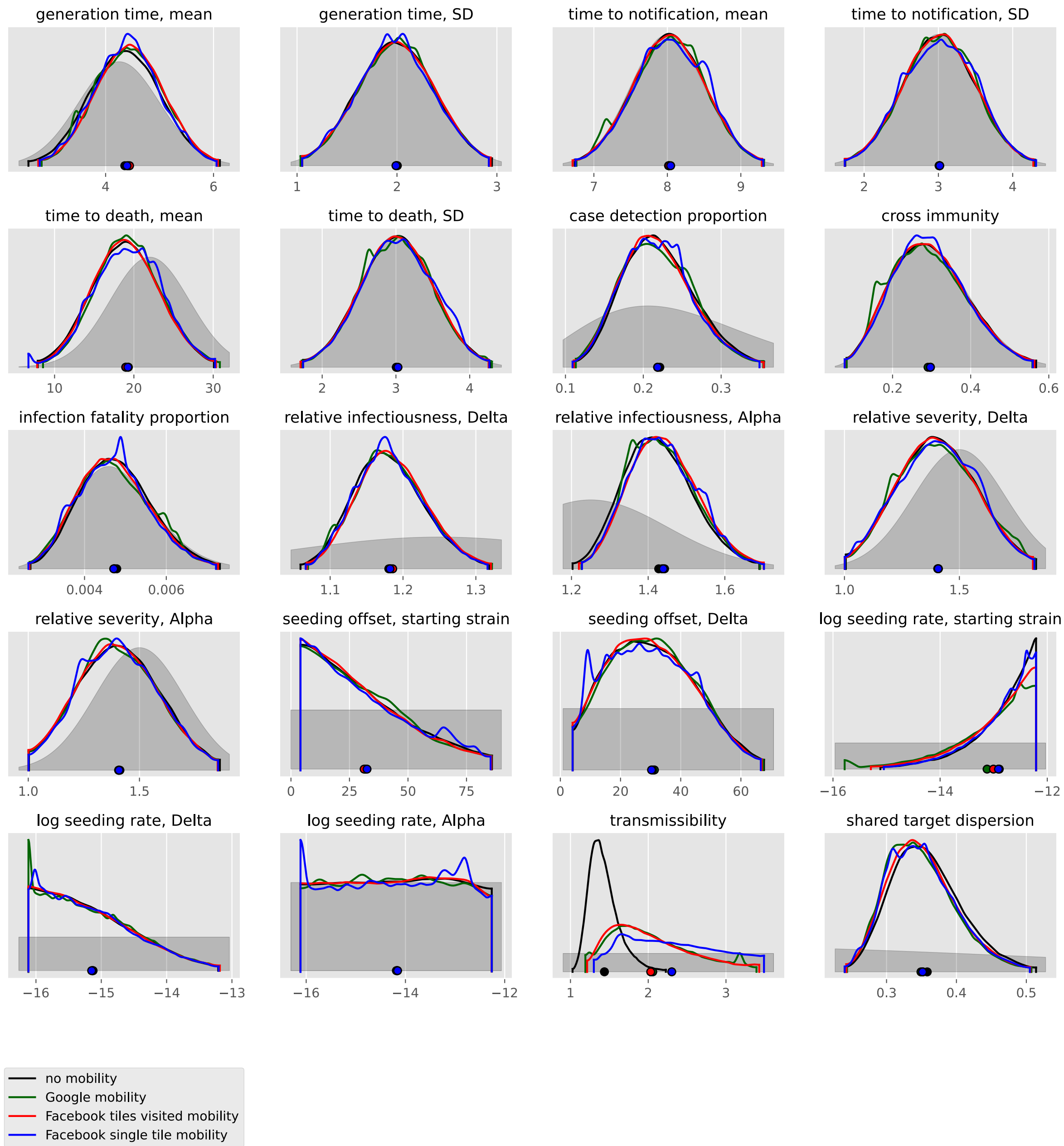
generation time, mean
generation time, SD
time to notification, mean
time to notification, SD
time to death, mean
time to death, SD
case detection proportion
cross immunity
infection fatality proportion
relative infectiousness, Delta
relative infectiousness, Alpha
relative severity, Delta
relative severity, Alpha
seeding offset, starting strain
seeding offset, Delta
log seeding rate, starting strain
log seeding rate, Delta
log seeding rate, Alpha
transmissibility
shared target dispersion
no mobility
Google mobility
Facebook tiles visited mobility
Facebook single tile mobility

Jamaica

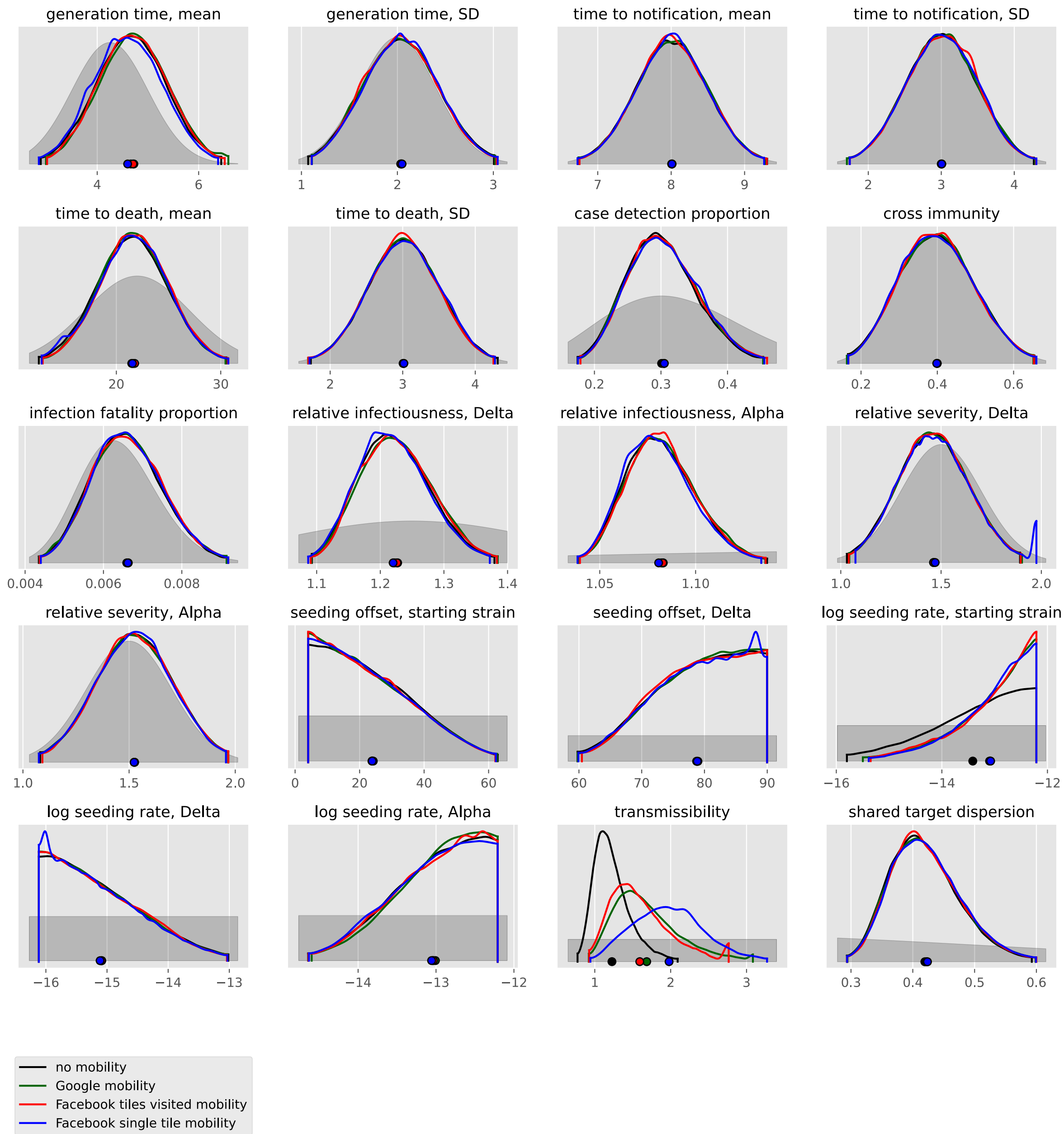

generation time, mean
generation time, SD
time to notification, mean
time to notification, SD
time to death, mean
time to death, SD
case detection proportion
cross immunity
infection fatality proportion
relative infectiousness, Delta
relative infectiousness, Alpha
relative severity, Delta
relative severity, Alpha
seeding offset, starting strain
seeding offset, Delta
log seeding rate, starting strain
log seeding rate, Delta
log seeding rate, Alpha
transmissibility
shared target dispersion
no mobility
Google mobility
Facebook tiles visited mobility
Facebook single tile mobility

Martinique

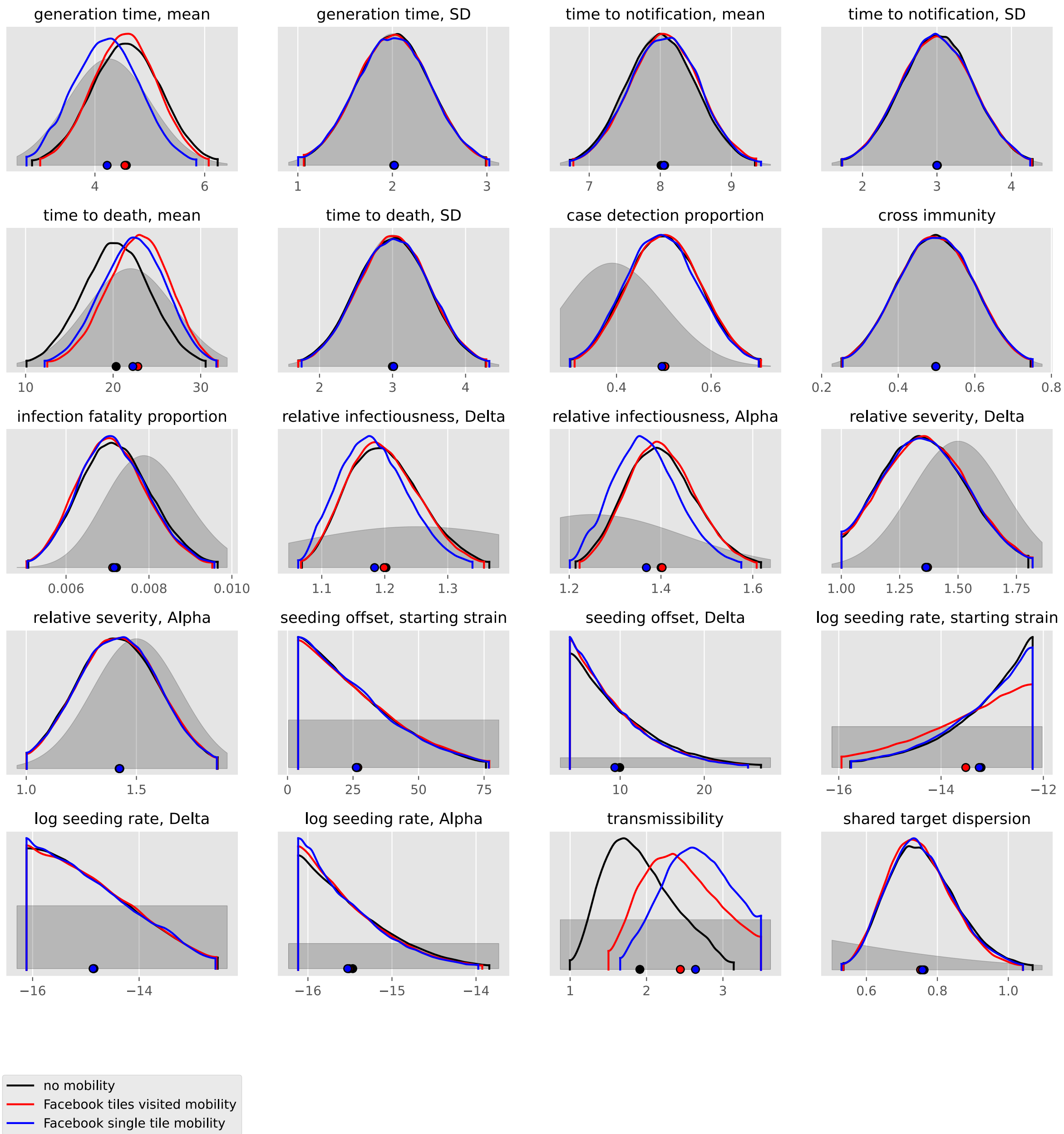

generation time, mean
4
6
generation time, SD
1
2
3
time to notification, mean
7
8
9
time to notification, SD
2
3
4
time to death, mean
10
20
30
time to death, SD
2
3
4
case detection proportion
0.4
0.6
cross immunity
0.2
0.4
0.6
0.8
infection fatality proportion
0.006
0.008
0.010
relative infectiousness, Delta
1.1
1.2
1.3
relative infectiousness, Alpha
1.2
1.4
1.6
relative severity, Delta
1.00
1.25
1.50
1.75
relative severity, Alpha
1.0
1.5
seeding offset, starting strain
0
25
50
75
seeding offset, Delta
10
20
log seeding rate, starting strain
−16
−14
−12
log seeding rate, Delta
−16
−14
log seeding rate, Alpha
−16
−15
−14
transmissibility
1
2
3
shared target dispersion
0.6
0.8
1.0
no mobility
Facebook tiles visited mobility
Facebook single tile mobility

Mexico

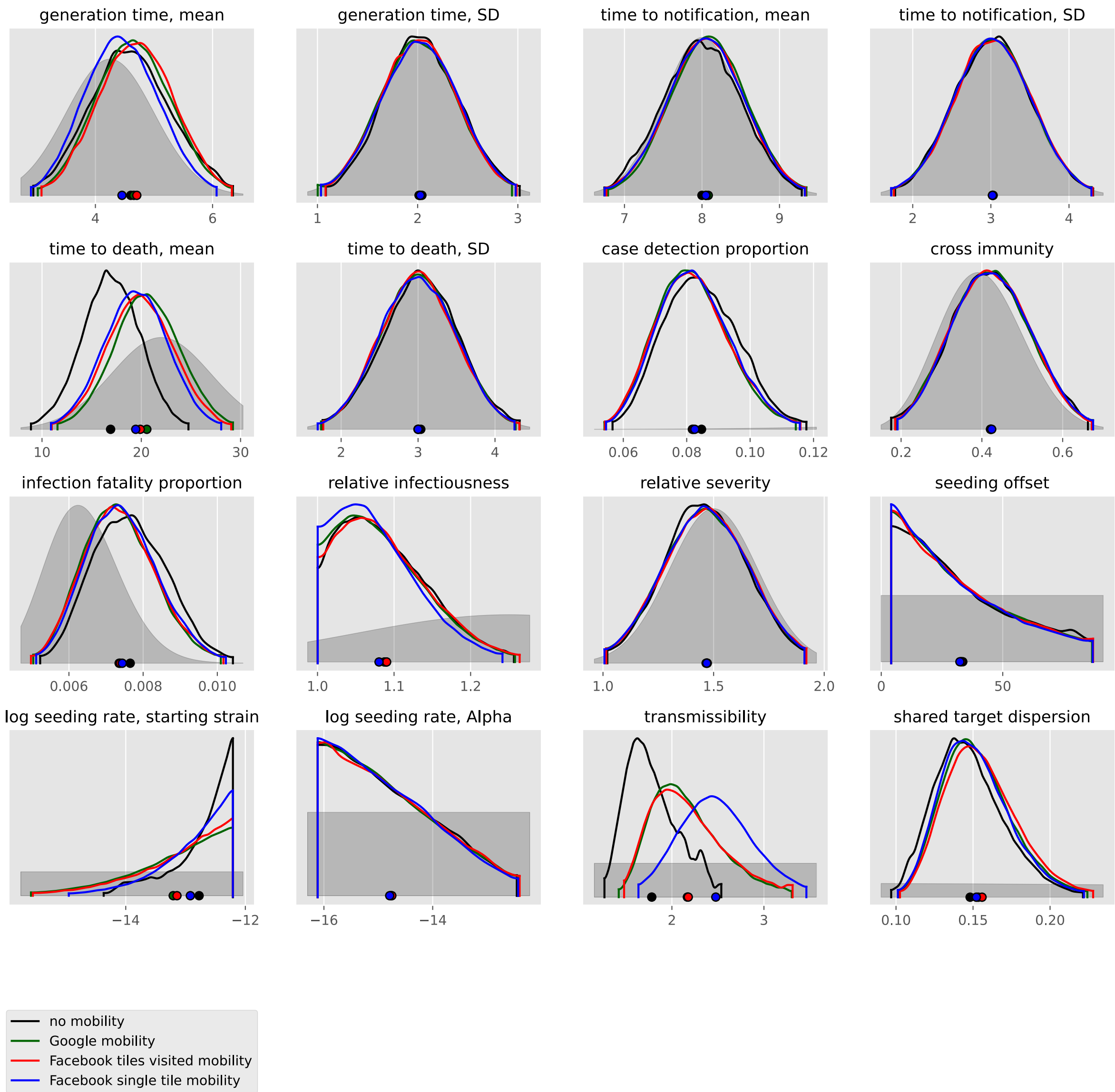

generation time, mean
4
6
generation time, SD
1
2
3
time to notification, mean
7
8
9
time to notification, SD
2
3
4
time to death, mean
10
20
30
time to death, SD
2
3
4
case detection proportion
0.06
0.08
0.10
0.12
cross immunity
0.2
0.4
0.6
infection fatality proportion
0.006
0.008
0.010
relative infectiousness
1.0
1.1
1.2
relative severity
1.0
1.5
2.0
seeding offset
0
50
log seeding rate, starting strain
−14
−12
log seeding rate, Alpha
−16
−14
transmissibility
2
3
shared target dispersion
0.10
0.15
0.20
no mobility
Google mobility
Facebook tiles visited mobility
Facebook single tile mobility

Panama

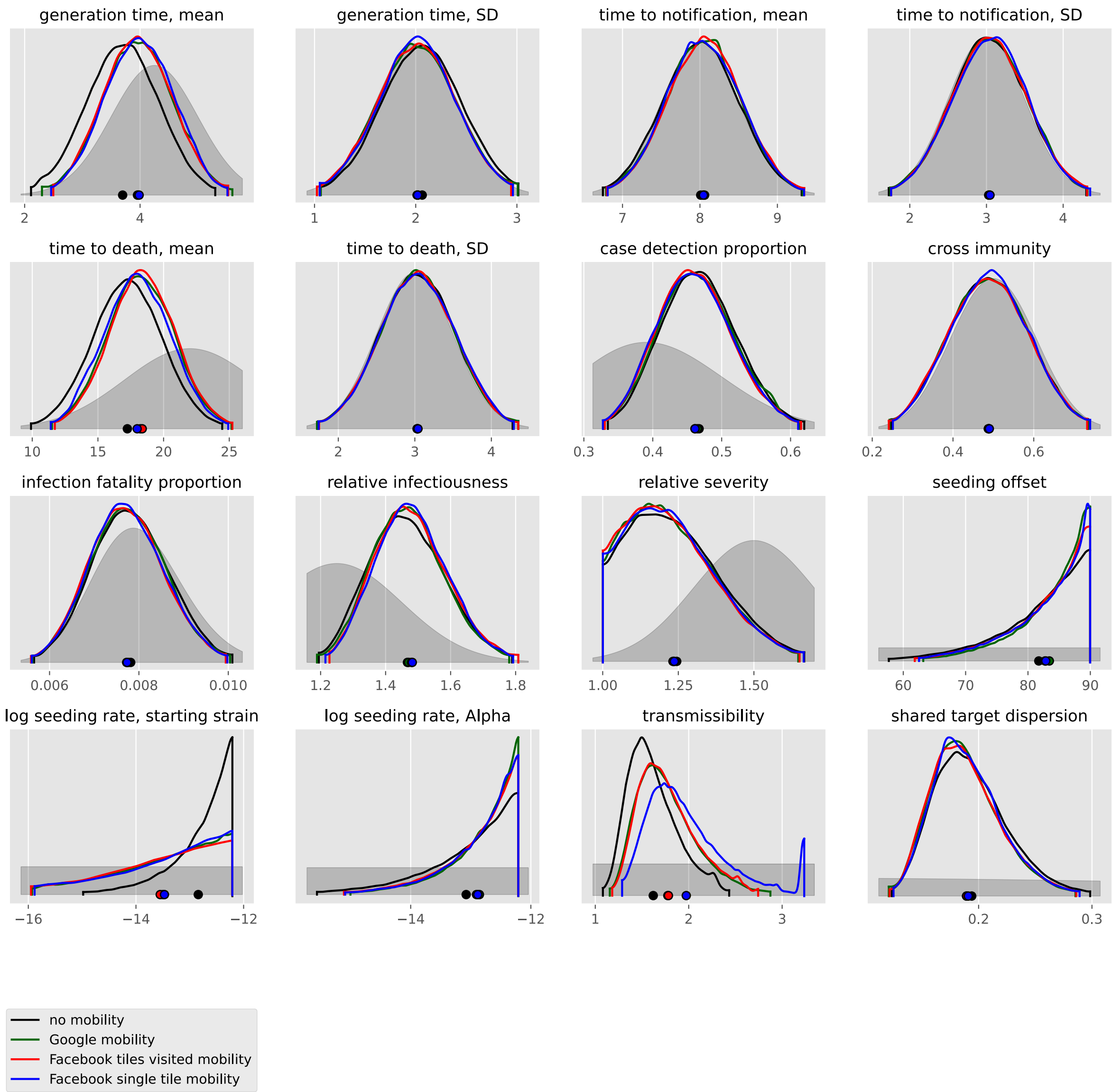

generation time, mean
generation time, SD
time to notification, mean
time to notification, SD
time to death, mean
time to death, SD
case detection proportion
cross immunity
infection fatality proportion
relative infectiousness
relative severity
seeding offset
log seeding rate, starting strain
log seeding rate, Alpha
transmissibility
shared target dispersion
no mobility
Google mobility
Facebook tiles visited mobility
Facebook single tile mobility

Puerto Rico

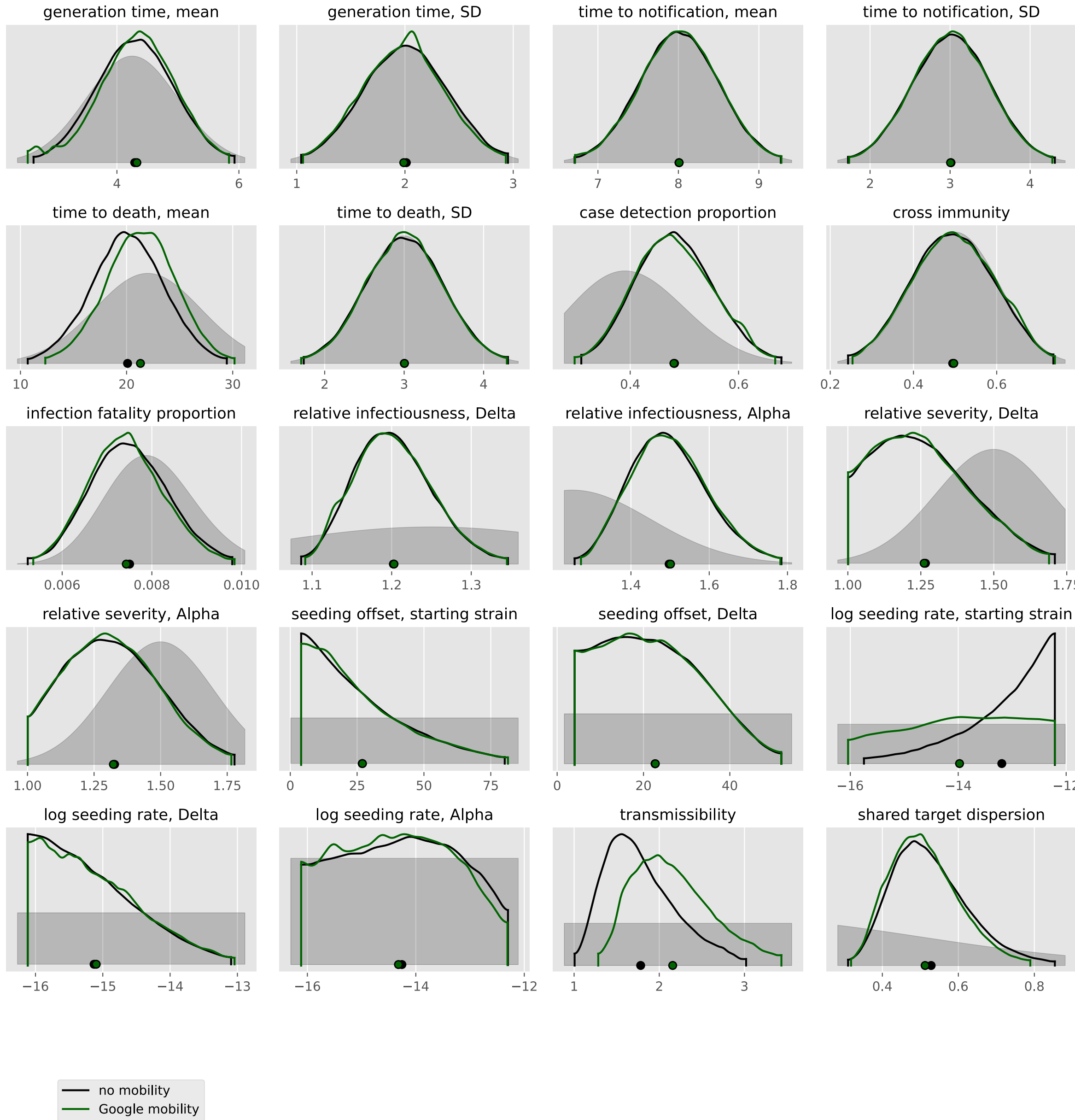
generation time, mean
generation time, SD
time to notification, mean
time to notification, SD
time to death, mean
time to death, SD
case detection proportion
cross immunity
infection fatality proportion
relative infectiousness, Delta
relative infectiousness, Alpha
relative severity, Delta
relative severity, Alpha
seeding offset, starting strain
seeding offset, Delta
log seeding rate, starting strain
log seeding rate, Delta
log seeding rate, Alpha
transmissibility
shared target dispersion
no mobility
Google mobility

United States

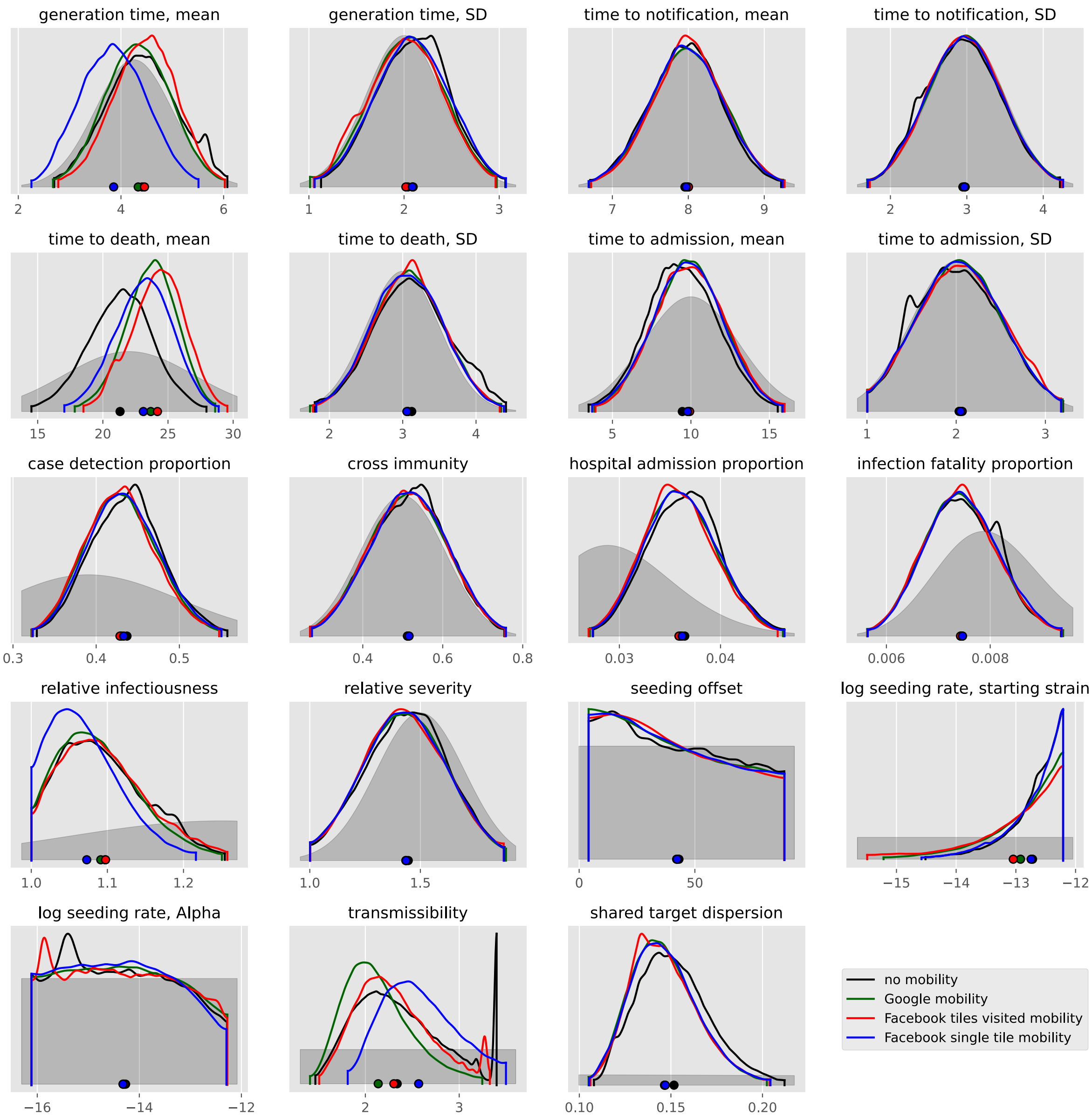

Oceania

Australia

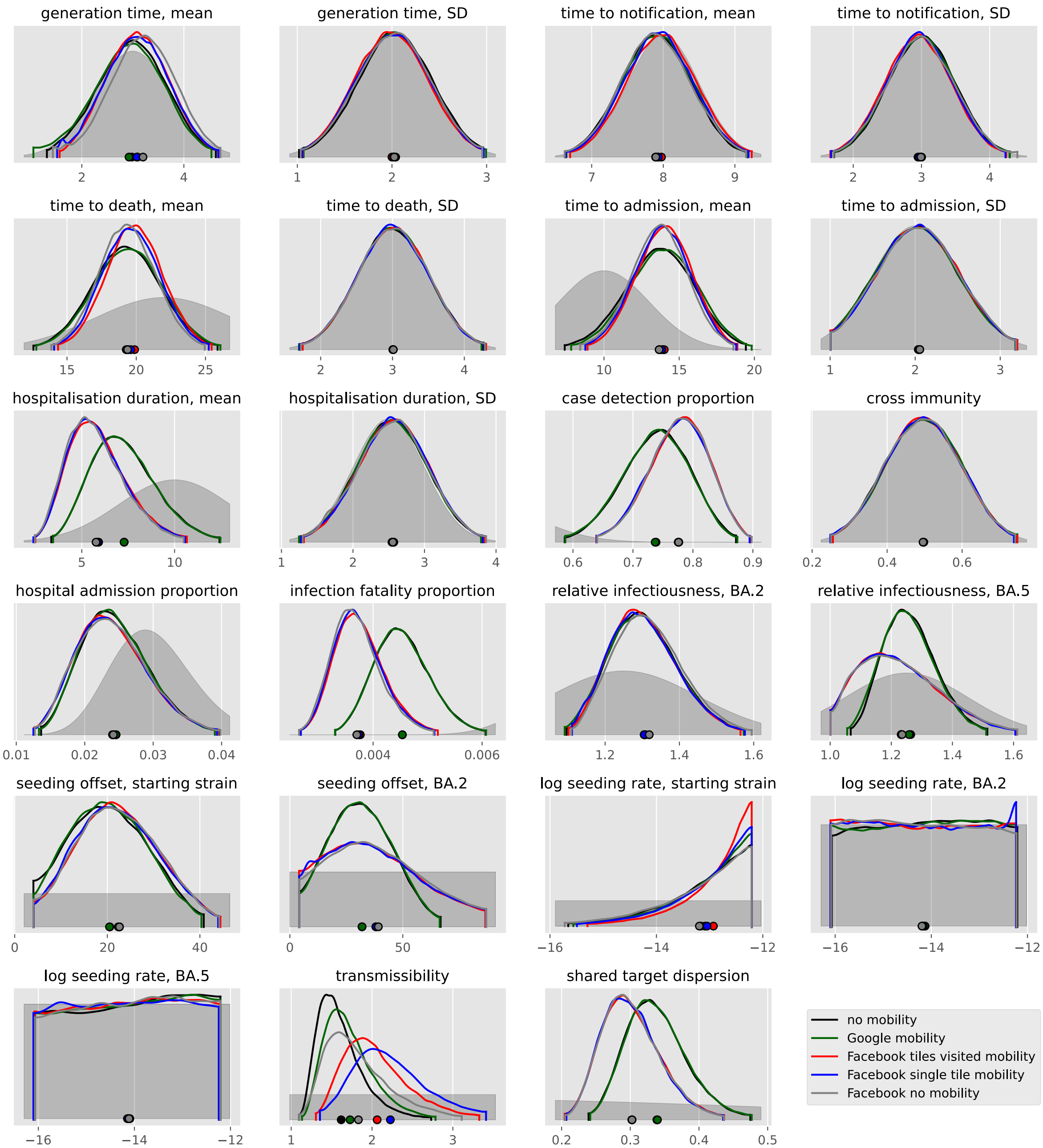

Fiji

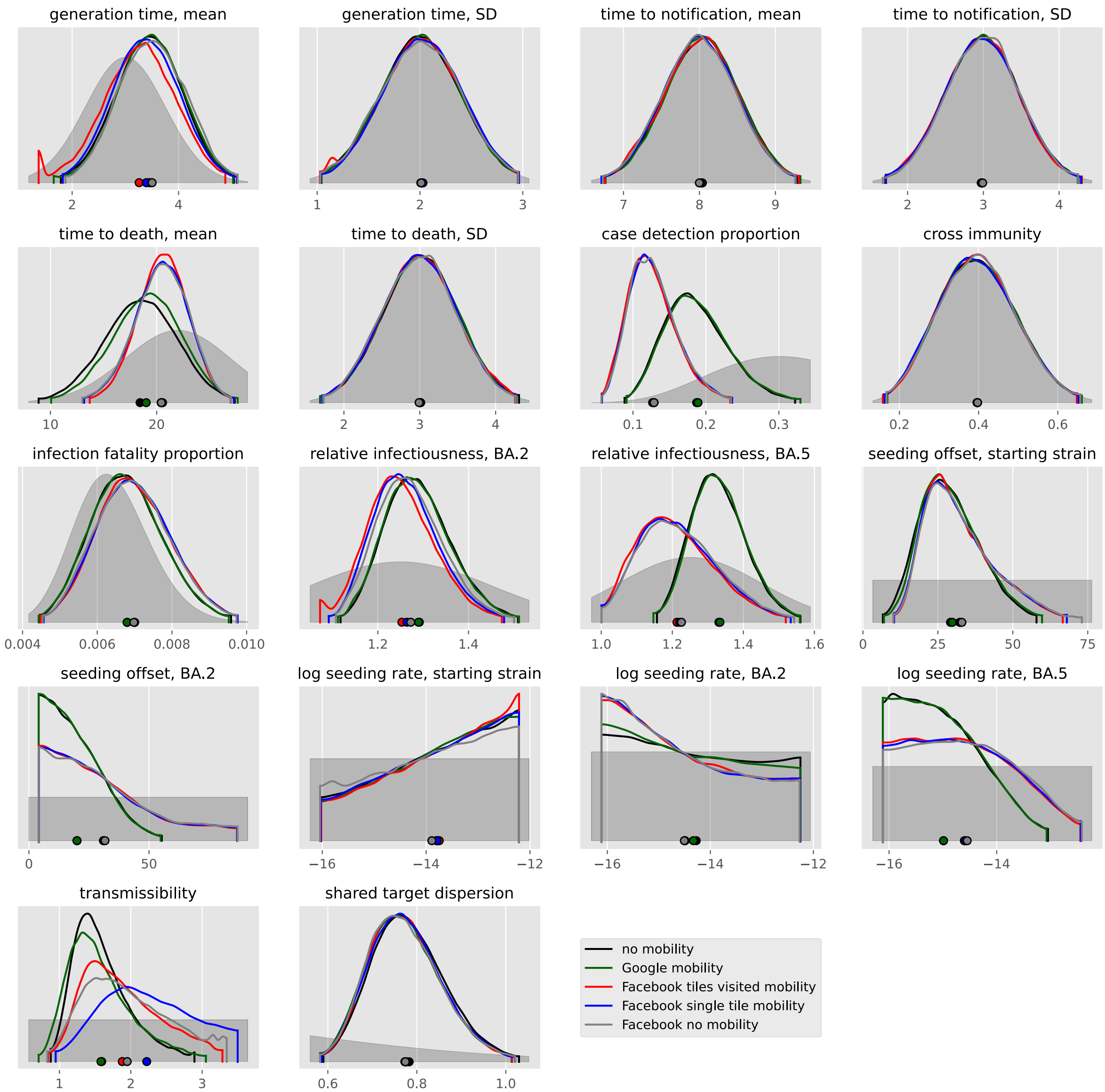

generation time, mean
generation time, SD
time to notification, mean
time to notification, SD
time to death, mean
time to death, SD
case detection proportion
cross immunity
infection fatality proportion
relative infectiousness, BA.2
relative infectiousness, BA.5
seeding offset, starting strain
seeding offset, BA.2
log seeding rate, starting strain
log seeding rate, BA.2
log seeding rate, BA.5
transmissibility
shared target dispersion
no mobility
Google mobility
Facebook tiles visited mobility
Facebook single tile mobility
Facebook no mobility

New Zealand

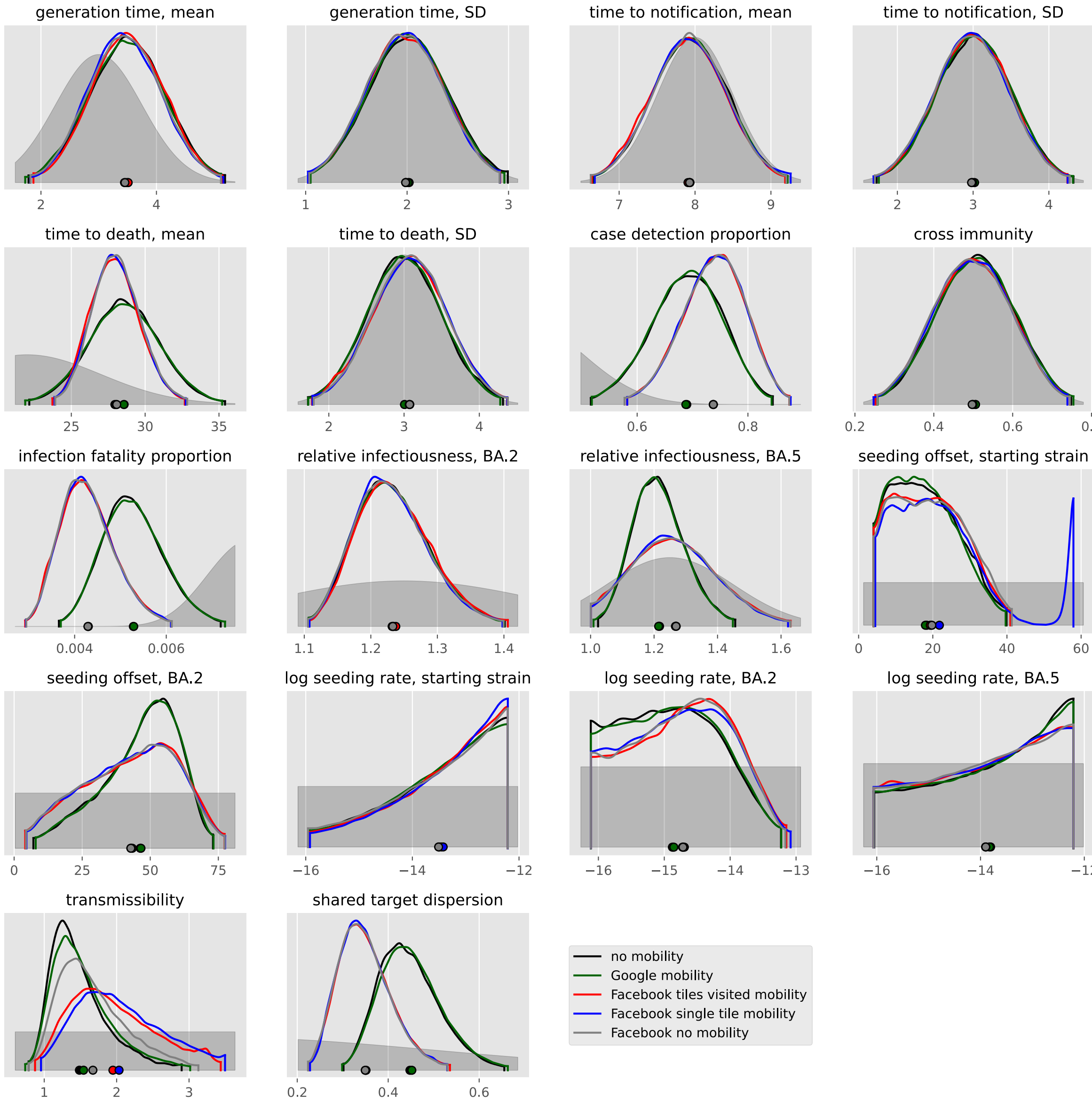
generation time, mean
generation time, SD
time to notification, mean
time to notification, SD
time to death, mean
time to death, SD
case detection proportion
cross immunity
infection fatality proportion
relative infectiousness, BA.2
relative infectiousness, BA.5
seeding offset, starting strain
seeding offset, BA.2
log seeding rate, starting strain
log seeding rate, BA.2
log seeding rate, BA.5
transmissibility
shared target dispersion
no mobility
Google mobility
Facebook tiles visited mobility
Facebook single tile mobility
Facebook no mobility

Singapore

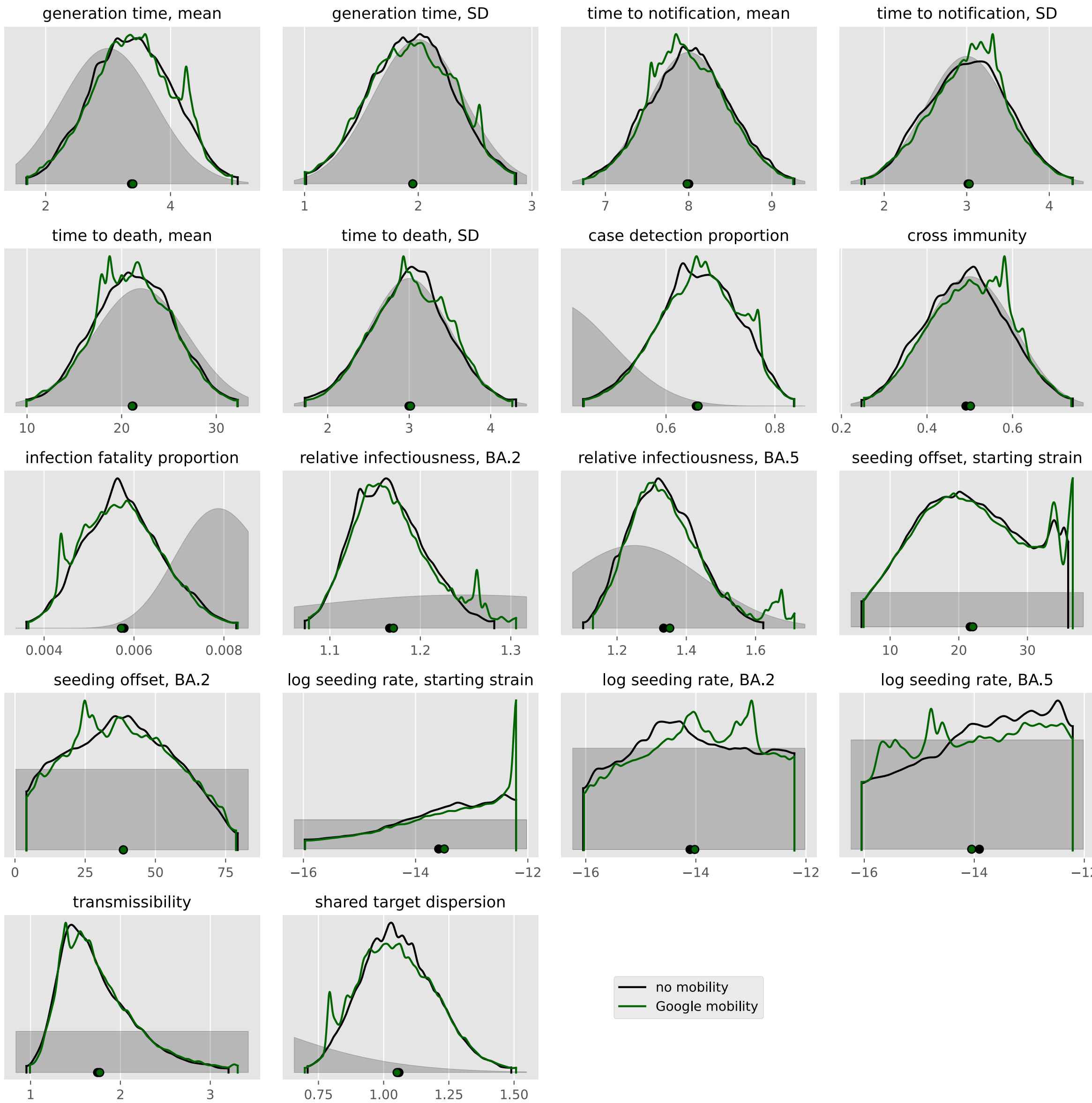

generation time, mean
2
4
generation time, SD
1
2
3
time to notification, mean
7
8
9
time to notification, SD
2
3
4
time to death, mean
10
20
30
time to death, SD
2
3
4
case detection proportion
0.6
0.8
cross immunity
0.2
0.4
0.6
infection fatality proportion
0.004
0.006
0.008
relative infectiousness, BA.2
1.1
1.2
1.3
relative infectiousness, BA.5
1.2
1.4
1.6
seeding offset, starting strain
10
20
30
seeding offset, BA.2
0
25
50
75
log seeding rate, starting strain
−16
−14
−12
log seeding rate, BA.2
−16
−14
−12
log seeding rate, BA.5
−16
−14
−12
transmissibility
1
2
3
shared target dispersion
0.75
1.00
1.25
1.50
no mobility
Google mobility

South America

Argentina

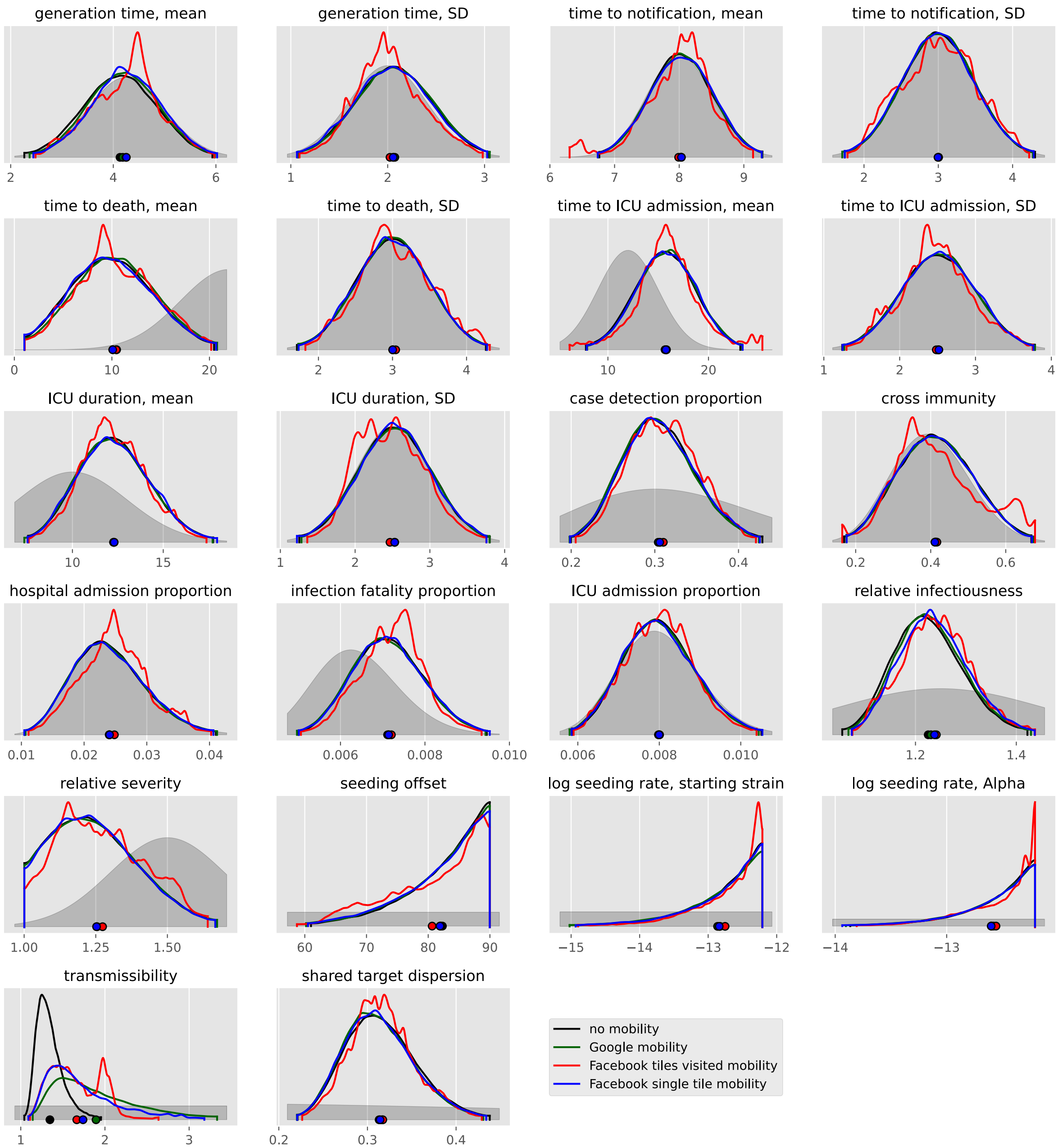

Brazil

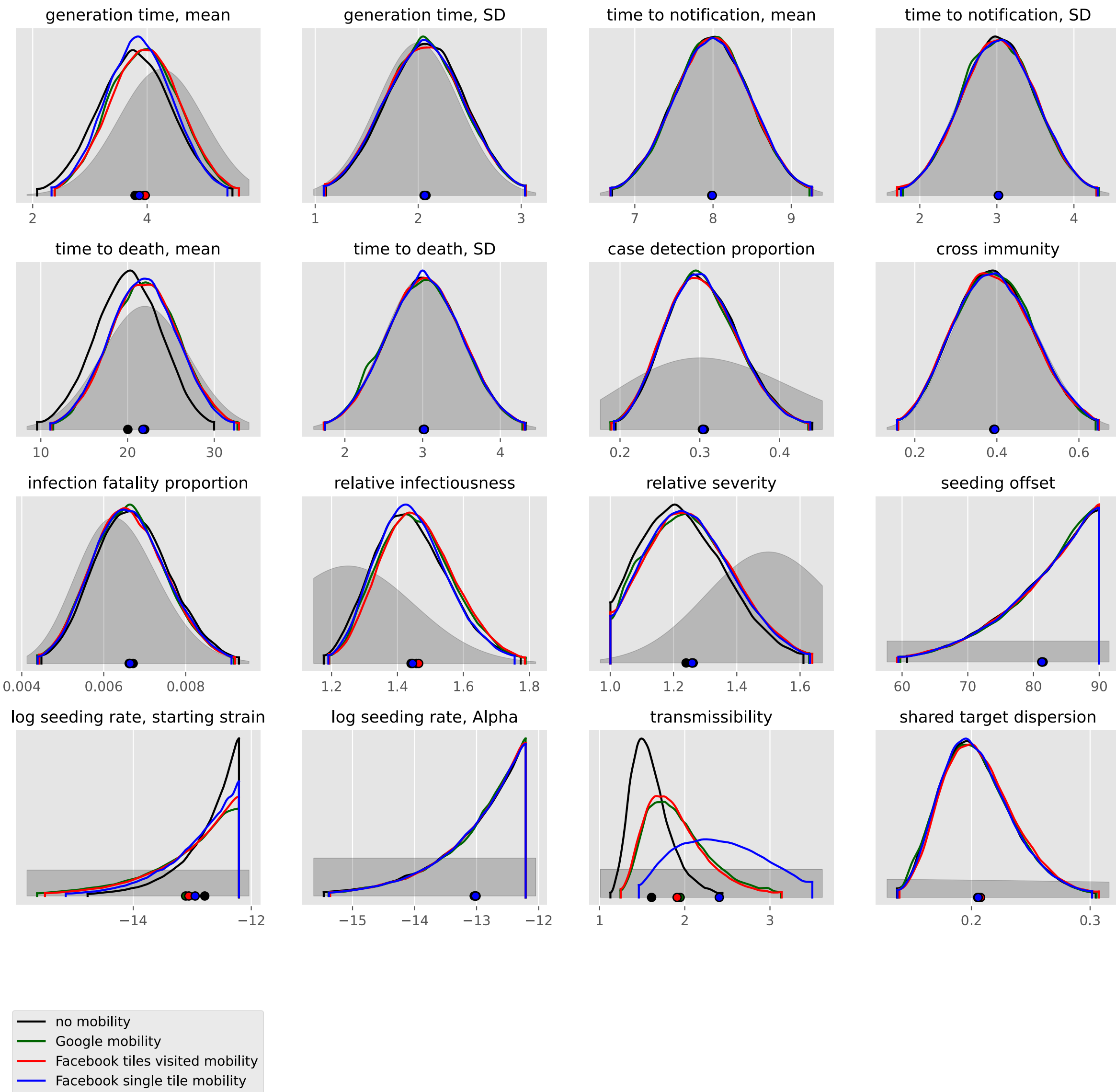

generation time, mean
2
4
generation time, SD
1
2
3
time to notification, mean
7
8
9
time to notification, SD
2
3
4
time to death, mean
10
20
30
time to death, SD
2
3
4
case detection proportion
0.2
0.3
0.4
cross immunity
0.2
0.4
0.6
infection fatality proportion
0.004
0.006
0.008
relative infectiousness
1.2
1.4
1.6
1.8
relative severity
1.0
1.2
1.4
1.6
seeding offset
60
70
80
90
log seeding rate, starting strain
−14
−12
log seeding rate, Alpha
−15
−14
−13
−12
transmissibility
1
2
3
shared target dispersion
0.2
0.3
no mobility
Google mobility
Facebook tiles visited mobility
Facebook single tile mobility

Chile

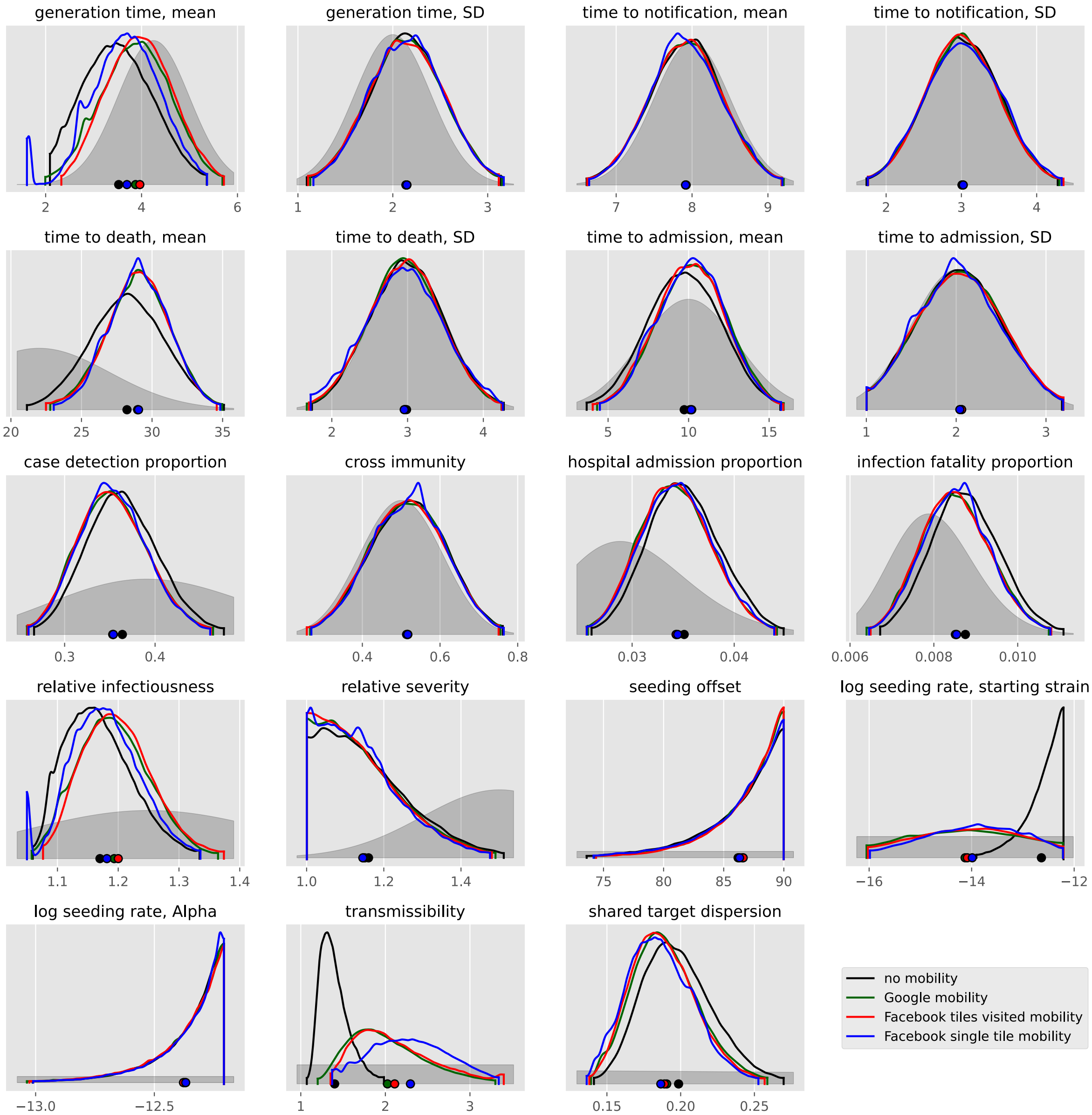
generation time, mean
generation time, SD
time to notification, mean
time to notification, SD
time to death, mean
time to death, SD
time to admission, mean
time to admission, SD
case detection proportion
cross immunity
hospital admission proportion
infection fatality proportion
relative infectiousness
relative severity
seeding offset
log seeding rate, starting strain
log seeding rate, Alpha
transmissibility
shared target dispersion
no mobility
Google mobility
Facebook tiles visited mobility
Facebook single tile mobility

Colombia

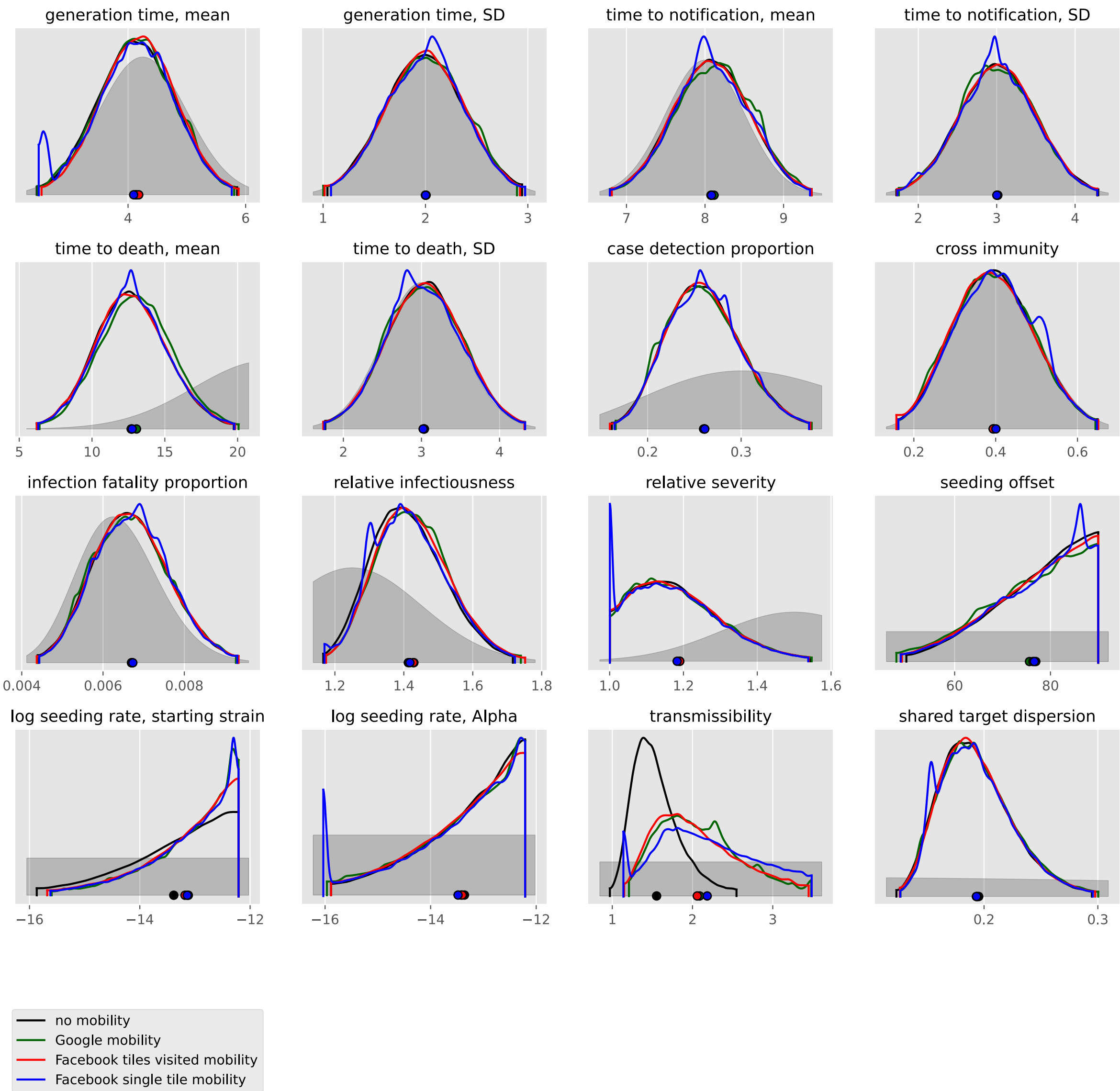

generation time, mean
generation time, SD
time to notification, mean
time to notification, SD
time to death, mean
time to death, SD
case detection proportion
cross immunity
infection fatality proportion
relative infectiousness
relative severity
seeding offset
log seeding rate, starting strain
log seeding rate, Alpha
transmissibility
shared target dispersion
no mobility
Google mobility
Facebook tiles visited mobility
Facebook single tile mobility

French Guiana

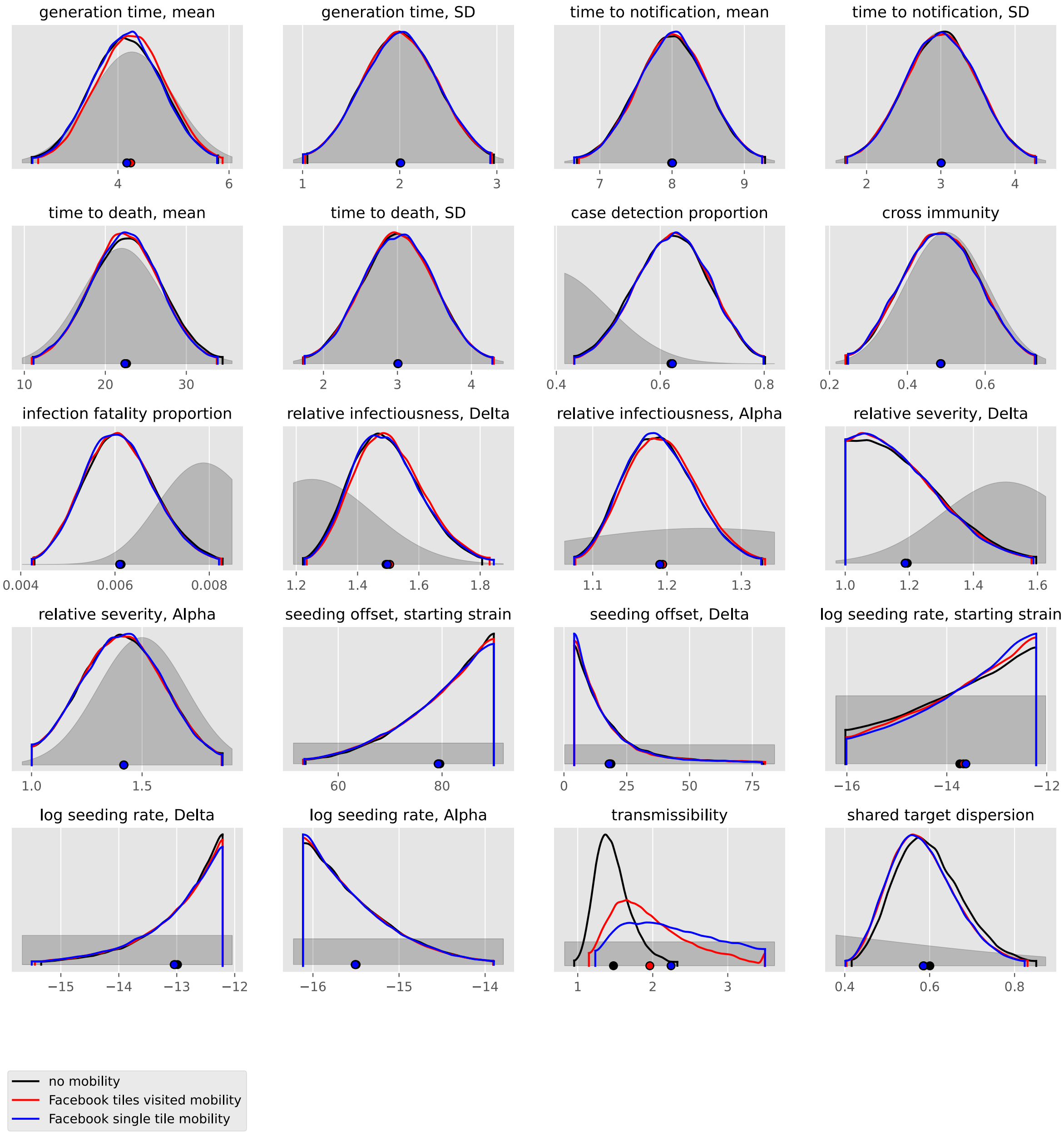

Guyana

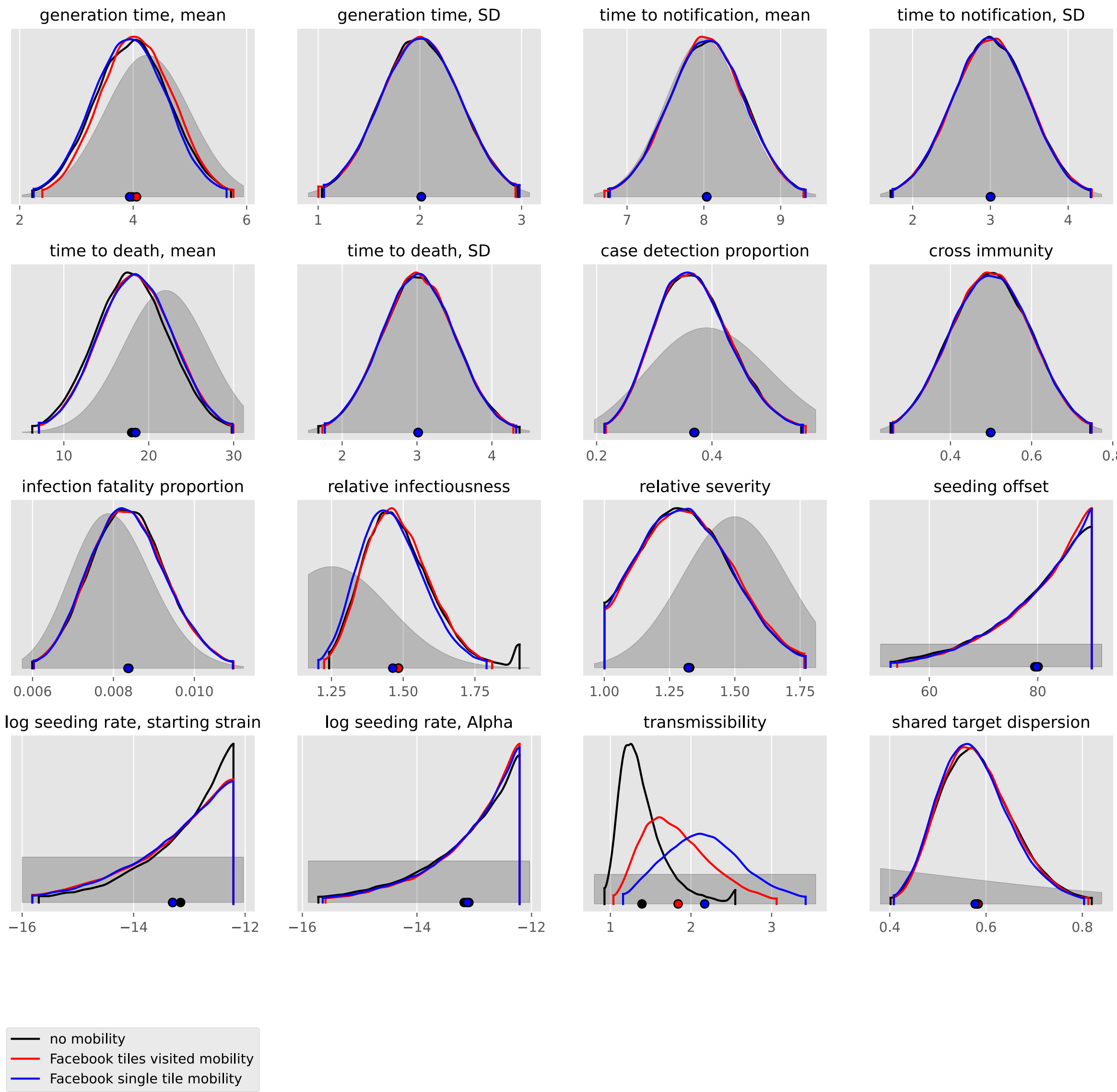

generation time, mean
generation time, SD
time to notification, mean
time to notification, SD
time to death, mean
time to death, SD
case detection proportion
cross immunity
infection fatality proportion
relative infectiousness
relative severity
seeding offset
log seeding rate, starting strain
log seeding rate, Alpha
transmissibility
shared target dispersion
no mobility
Facebook tiles visited mobility
Facebook single tile mobility

Paraguay

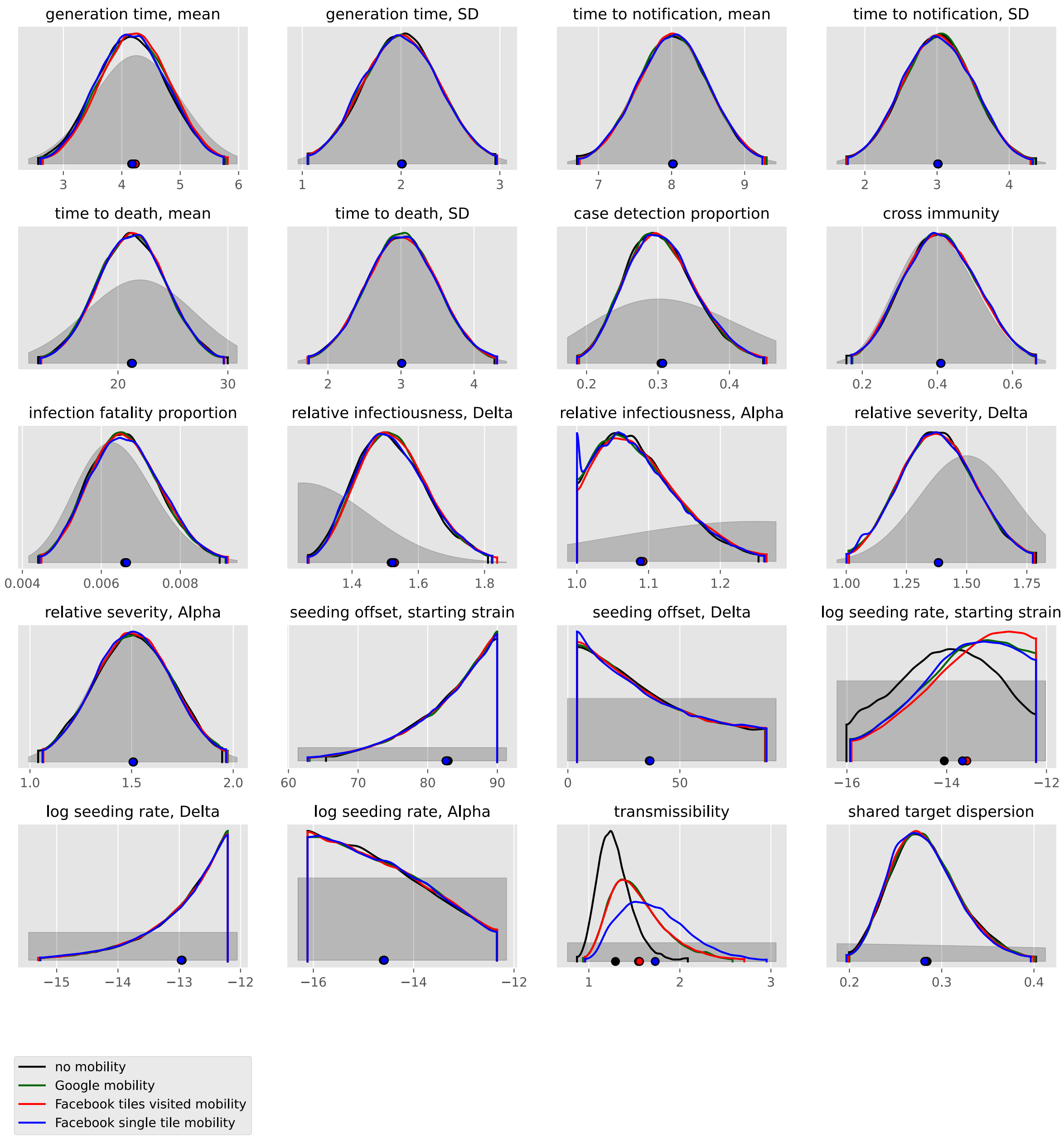

generation time, mean
generation time, SD
time to notification, mean
time to notification, SD
time to death, mean
time to death, SD
case detection proportion
cross immunity
infection fatality proportion
relative infectiousness, Delta
relative infectiousness, Alpha
relative severity, Delta
relative severity, Alpha
seeding offset, starting strain
seeding offset, Delta
log seeding rate, starting strain
log seeding rate, Delta
log seeding rate, Alpha
transmissibility
shared target dispersion
no mobility
Google mobility
Facebook tiles visited mobility
Facebook single tile mobility

Peru

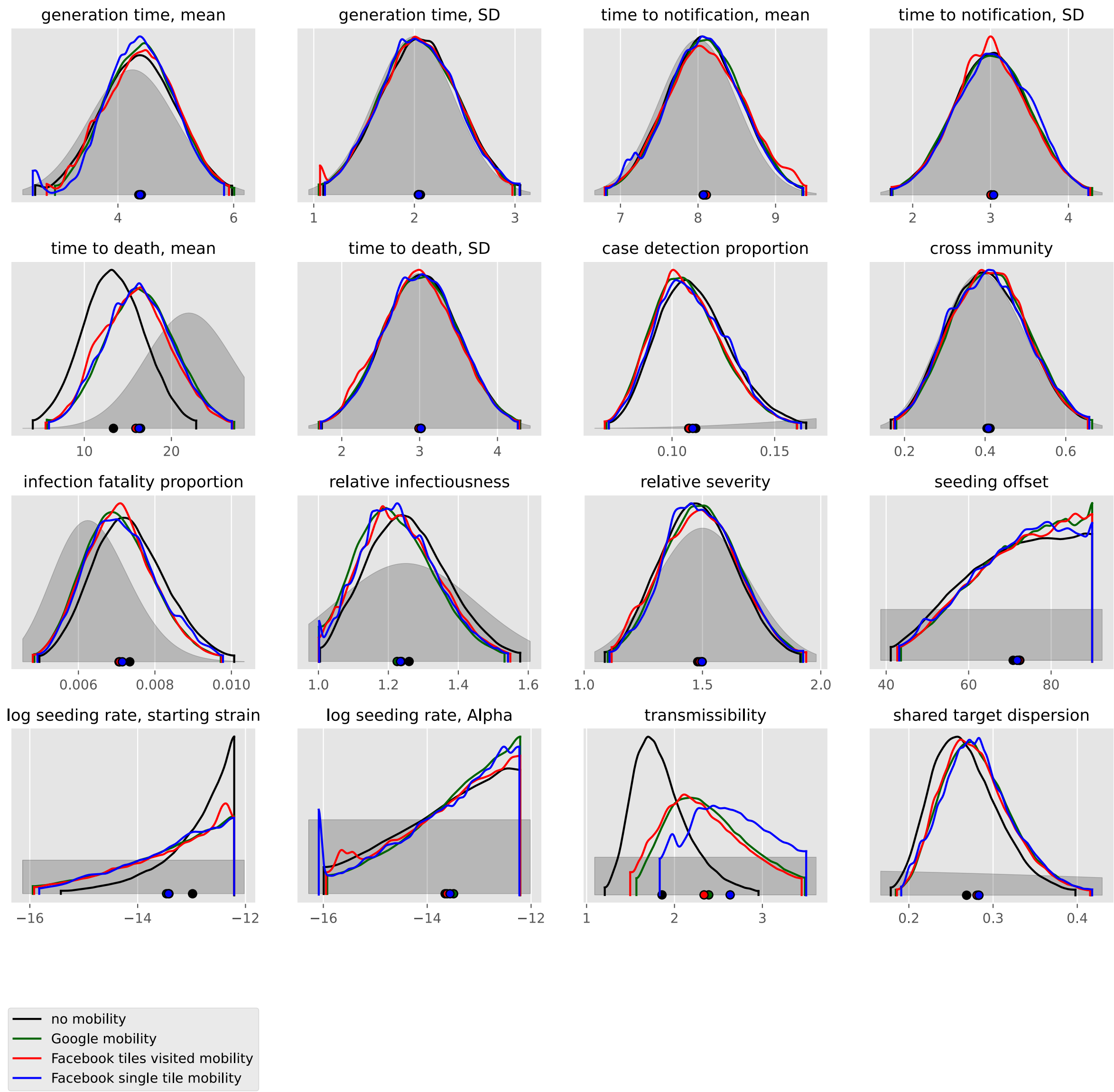
generation time, mean
generation time, SD
time to notification, mean
time to notification, SD
time to death, mean
time to death, SD
case detection proportion
cross immunity
infection fatality proportion
relative infectiousness
relative severity
seeding offset
log seeding rate, starting strain
log seeding rate, Alpha
transmissibility
shared target dispersion
no mobility
Google mobility
Facebook tiles visited mobility
Facebook single tile mobility

Suriname

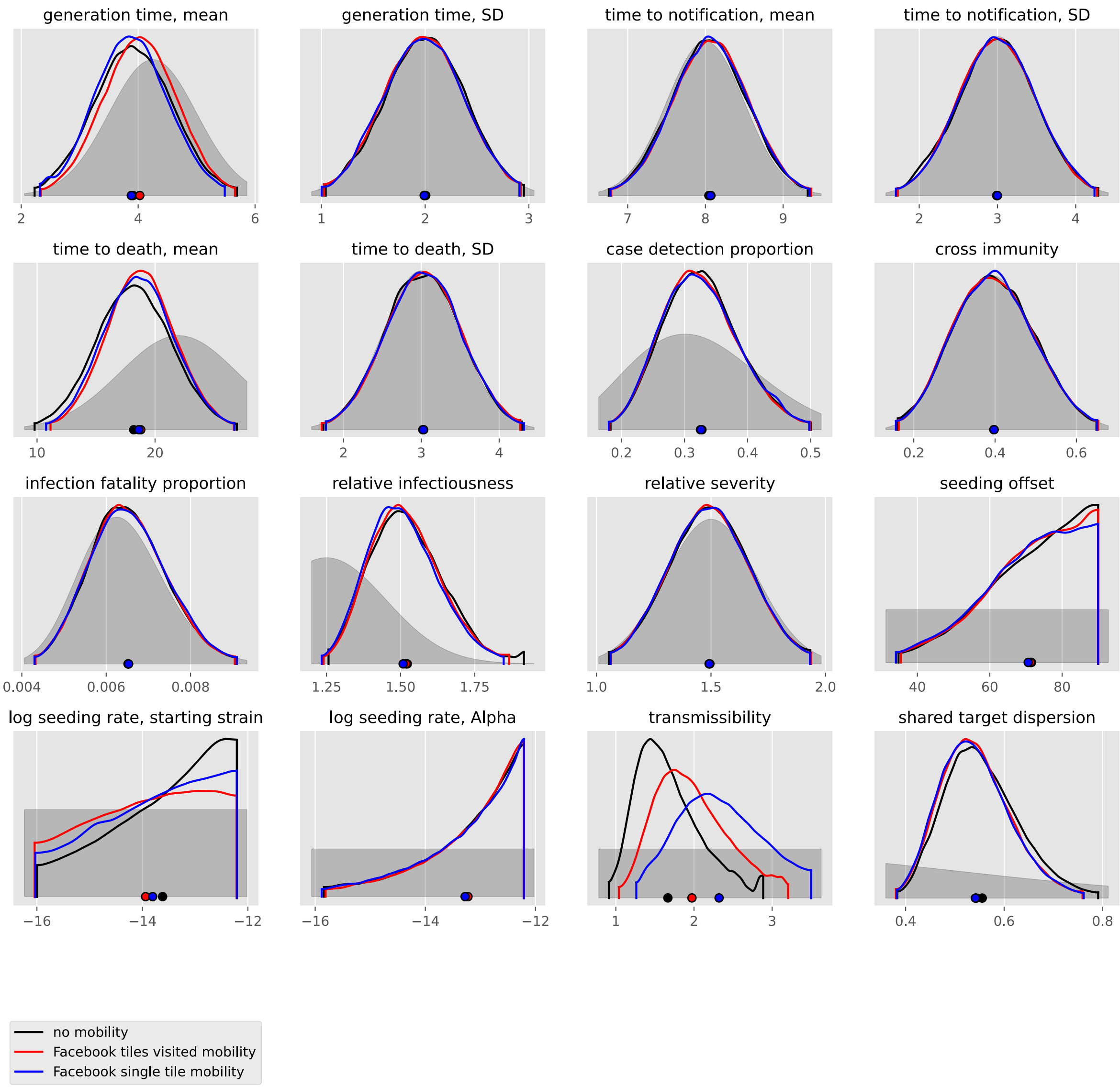

generation time, mean
generation time, SD
time to notification, mean
time to notification, SD
time to death, mean
time to death, SD
case detection proportion
cross immunity
infection fatality proportion
relative infectiousness
relative severity
seeding offset
log seeding rate, starting strain
log seeding rate, Alpha
transmissibility
shared target dispersion
no mobility
Facebook tiles visited mobility
Facebook single tile mobility

Uruguay

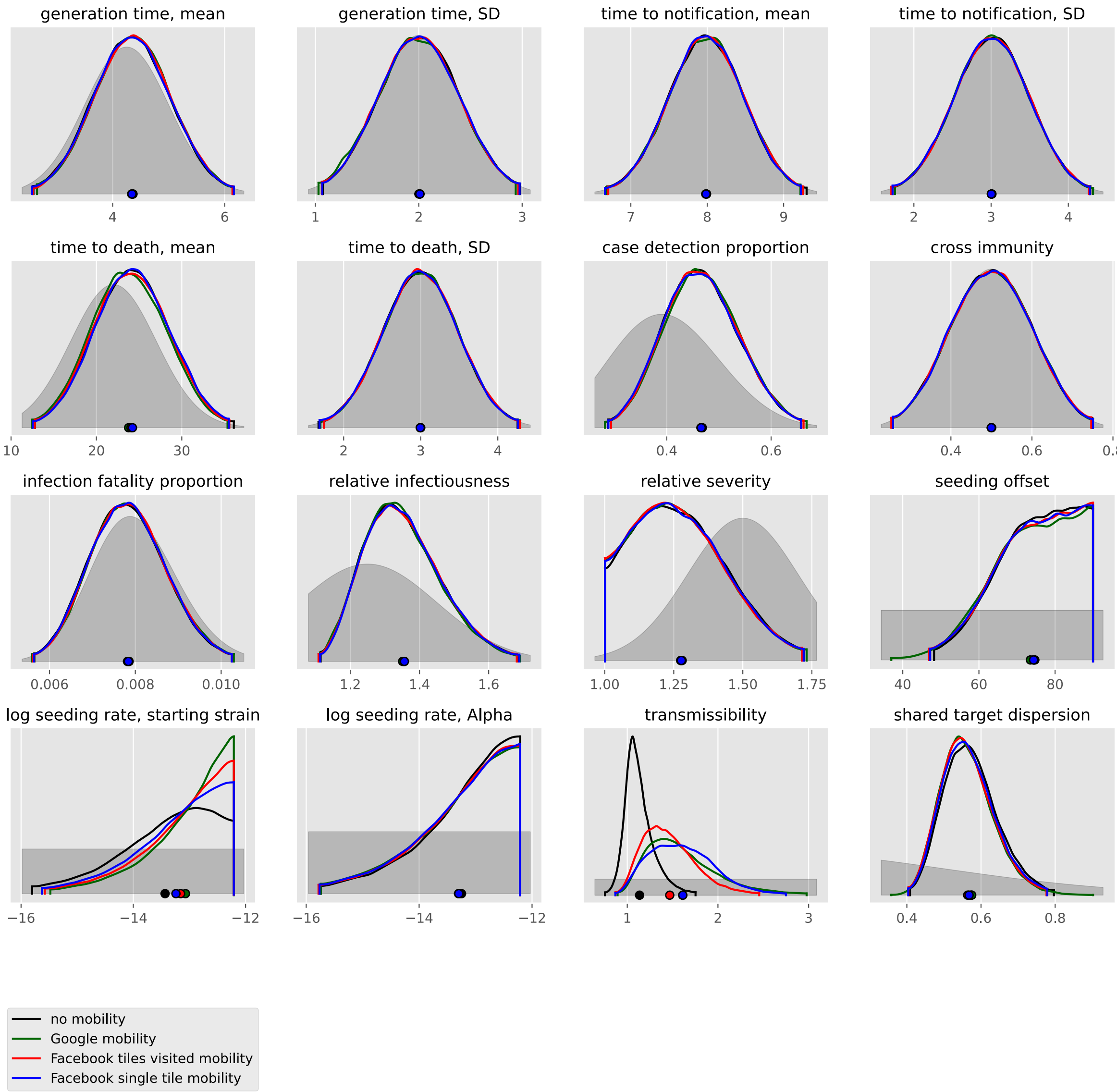
generation time, mean
generation time, SD
time to notification, mean
time to notification, SD
time to death, mean
time to death, SD
case detection proportion
cross immunity
infection fatality proportion
relative infectiousness
relative severity
seeding offset
log seeding rate, starting strain
log seeding rate, Alpha
transmissibility
shared target dispersion
no mobility
Google mobility
Facebook tiles visited mobility
Facebook single tile mobility

Venezuela, Bolivarian Republic of

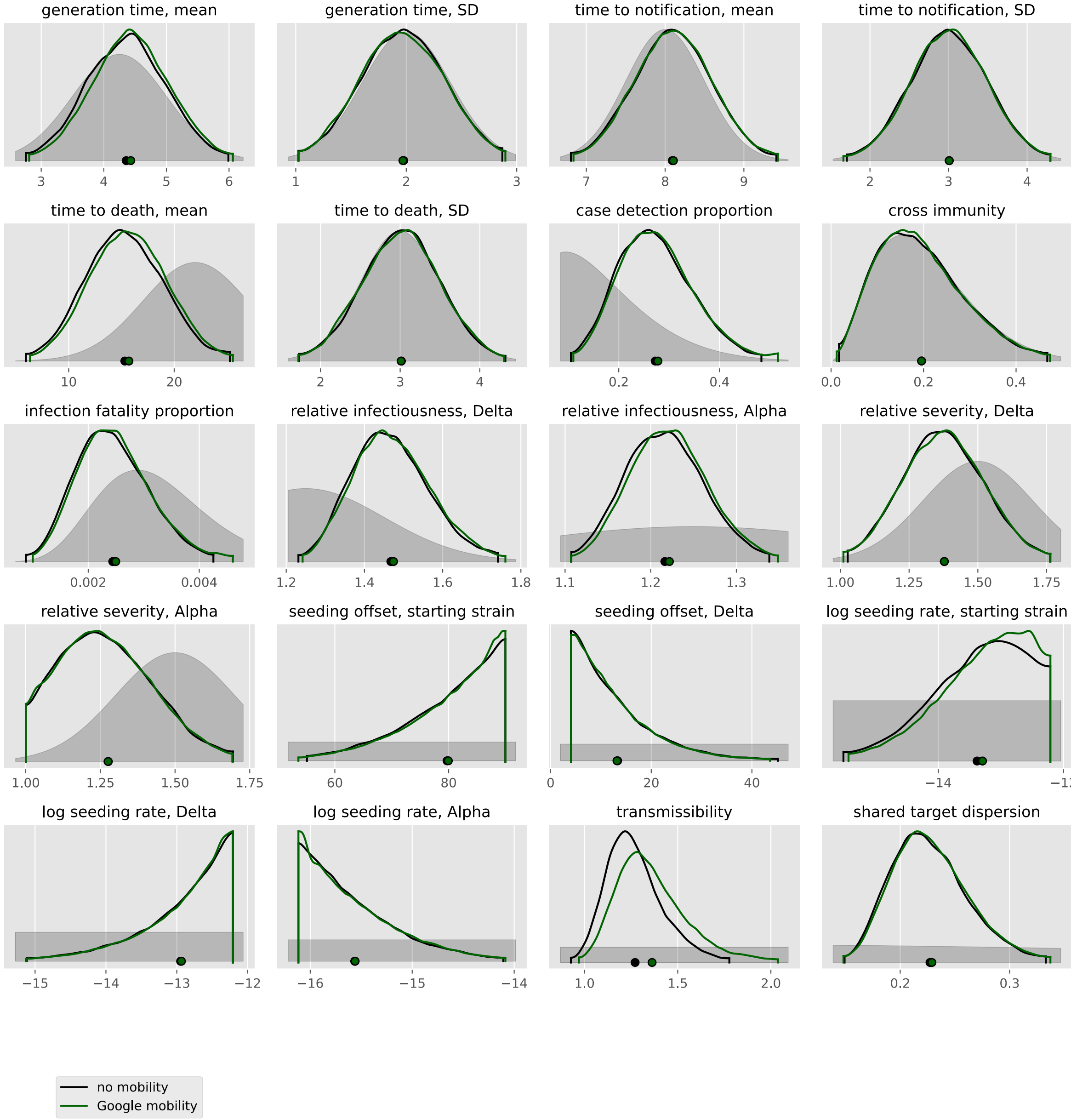

Prior posterior comparisons by continent and country, waning immunity included

This section presents comparisons of parameter prior distributions to their corresponding posterior distributions.

Africa

Algeria

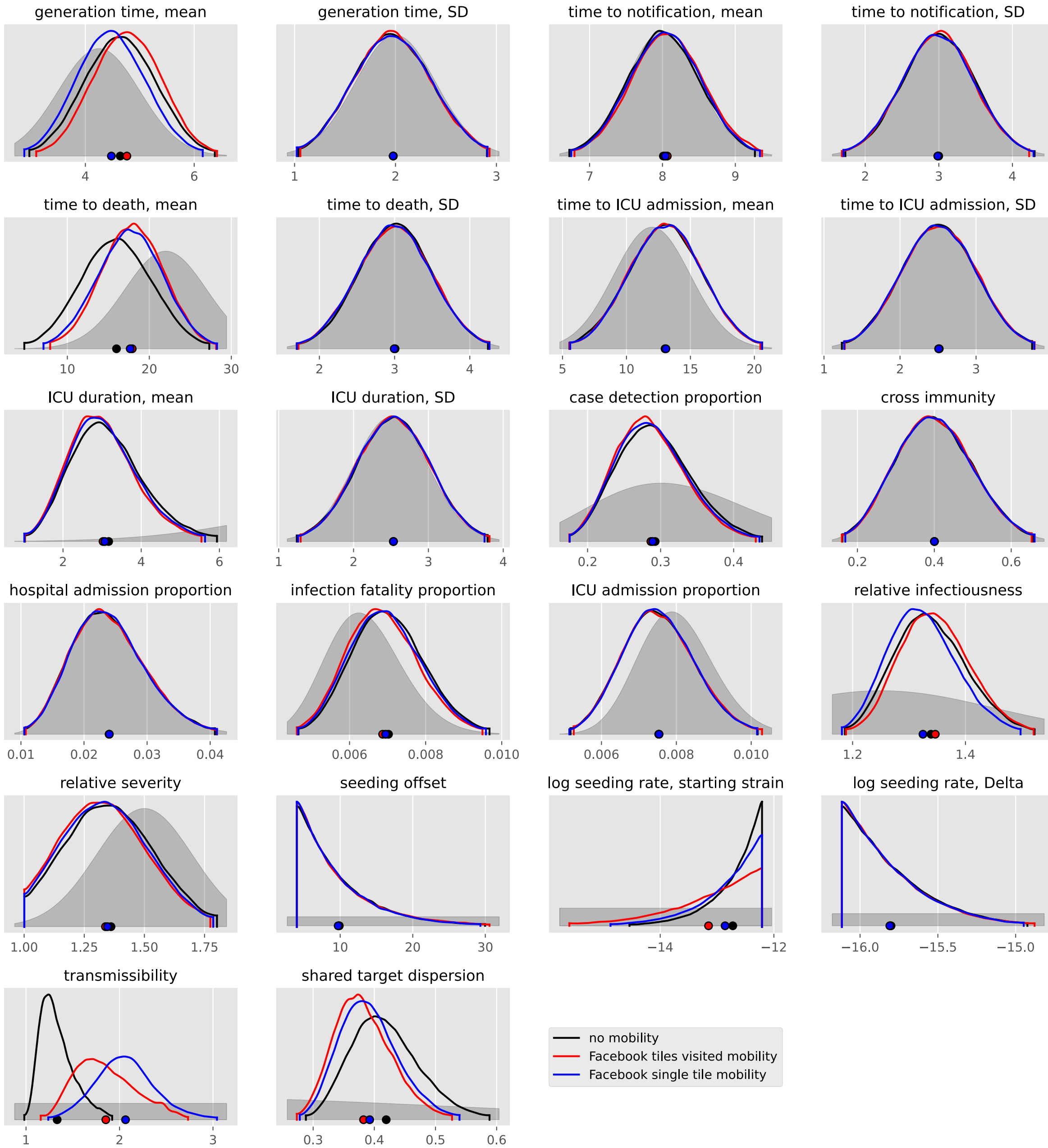

Angola

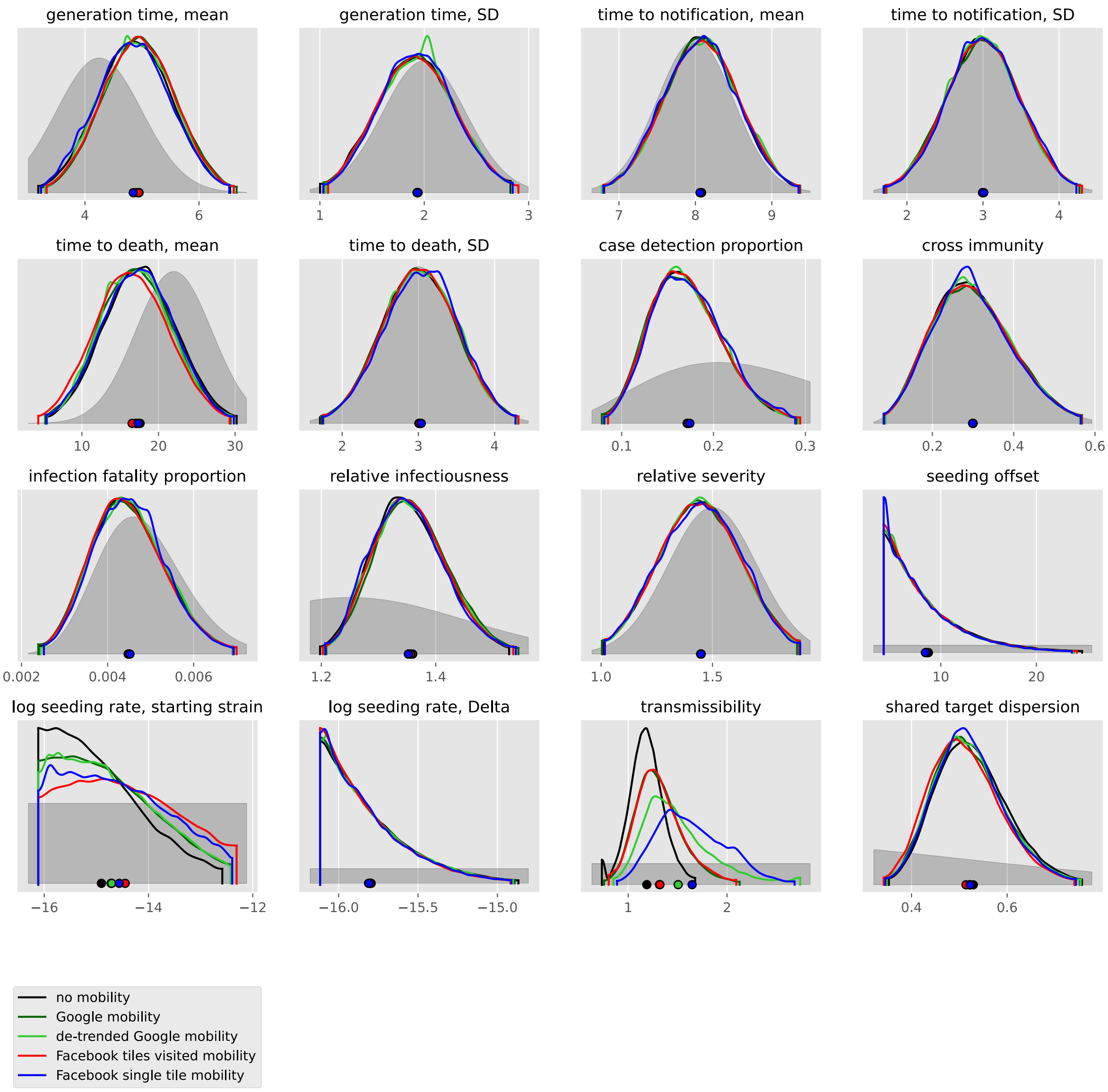

generation time, mean
generation time, SD
time to notification, mean
time to notification, SD
time to death, mean
time to death, SD
case detection proportion
cross immunity
infection fatality proportion
relative infectiousness
relative severity
seeding offset
log seeding rate, starting strain
log seeding rate, Delta
transmissibility
shared target dispersion
no mobility
Google mobility
de-trended Google mobility
Facebook tiles visited mobility
Facebook single tile mobility

Benin

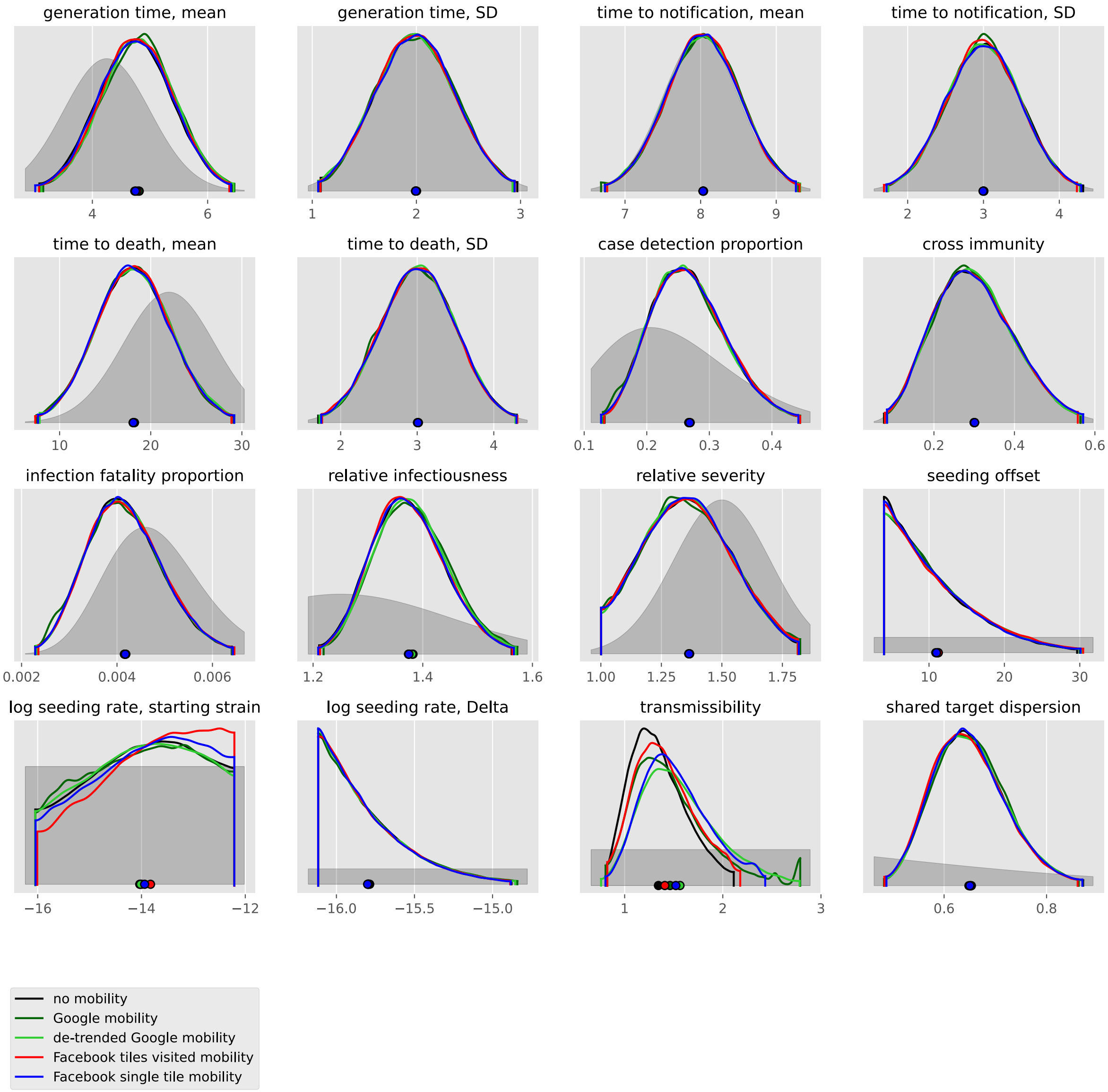

generation time, mean
generation time, SD
time to notification, mean
time to notification, SD
time to death, mean
time to death, SD
case detection proportion
cross immunity
infection fatality proportion
relative infectiousness
relative severity
seeding offset
log seeding rate, starting strain
log seeding rate, Delta
transmissibility
shared target dispersion
no mobility
Google mobility
de-trended Google mobility
Facebook tiles visited mobility
Facebook single tile mobility

Burkina Faso

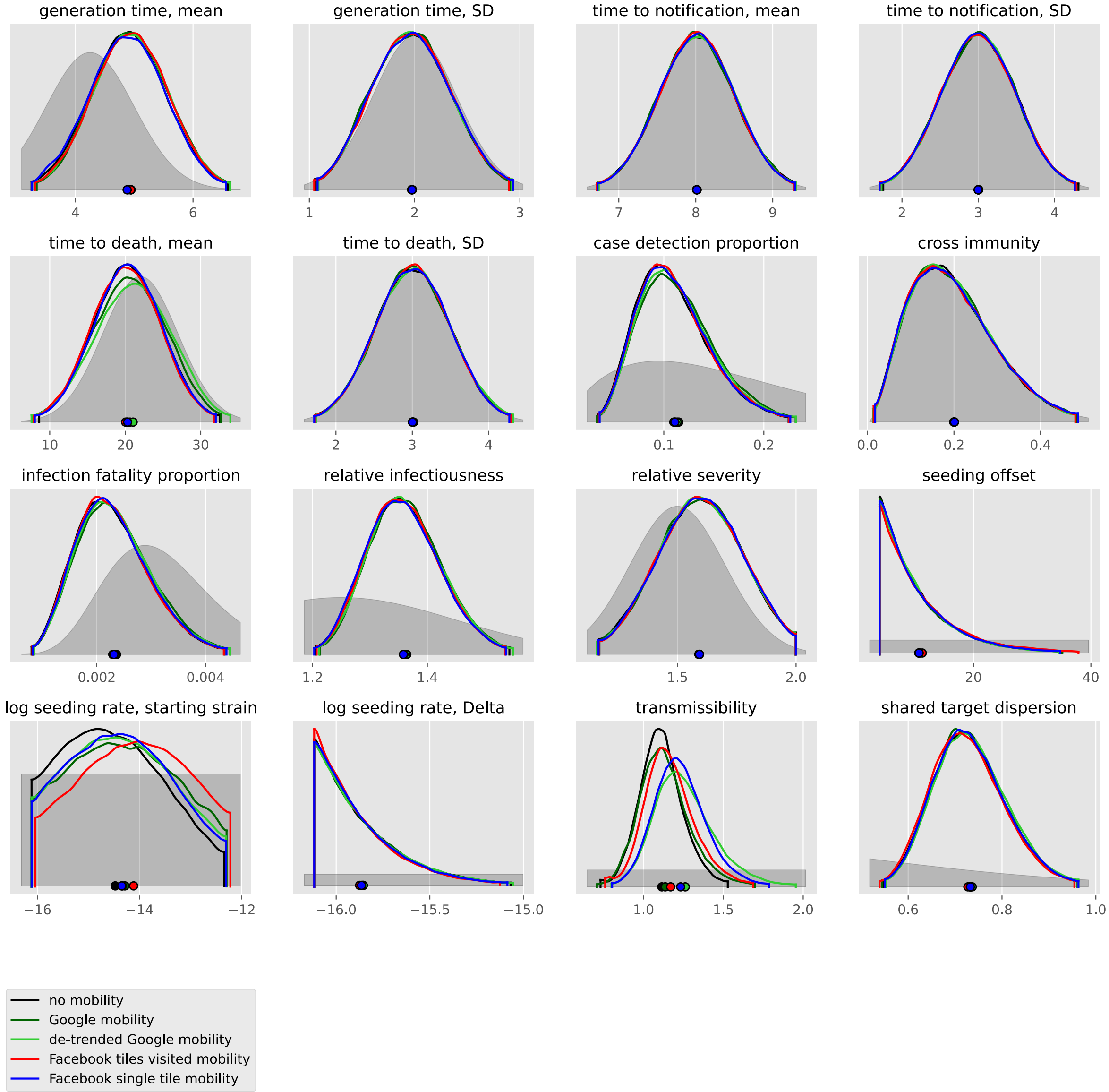

Cabo Verde

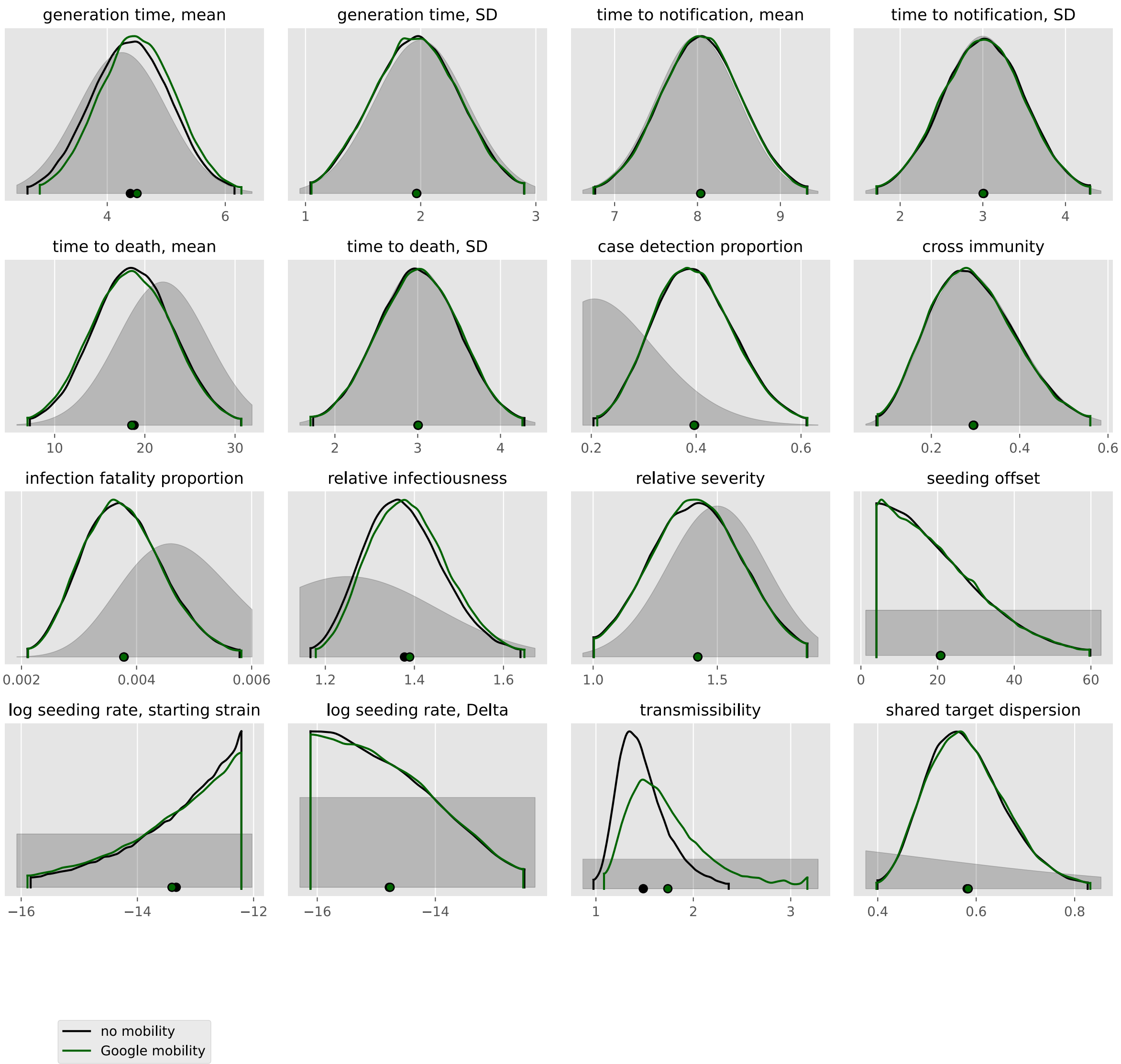

generation time, mean
generation time, SD
time to notification, mean
time to notification, SD
time to death, mean
time to death, SD
case detection proportion
cross immunity
infection fatality proportion
relative infectiousness
relative severity
seeding offset
log seeding rate, starting strain
log seeding rate, Delta
transmissibility
shared target dispersion
no mobility
Google mobility

Cameroon

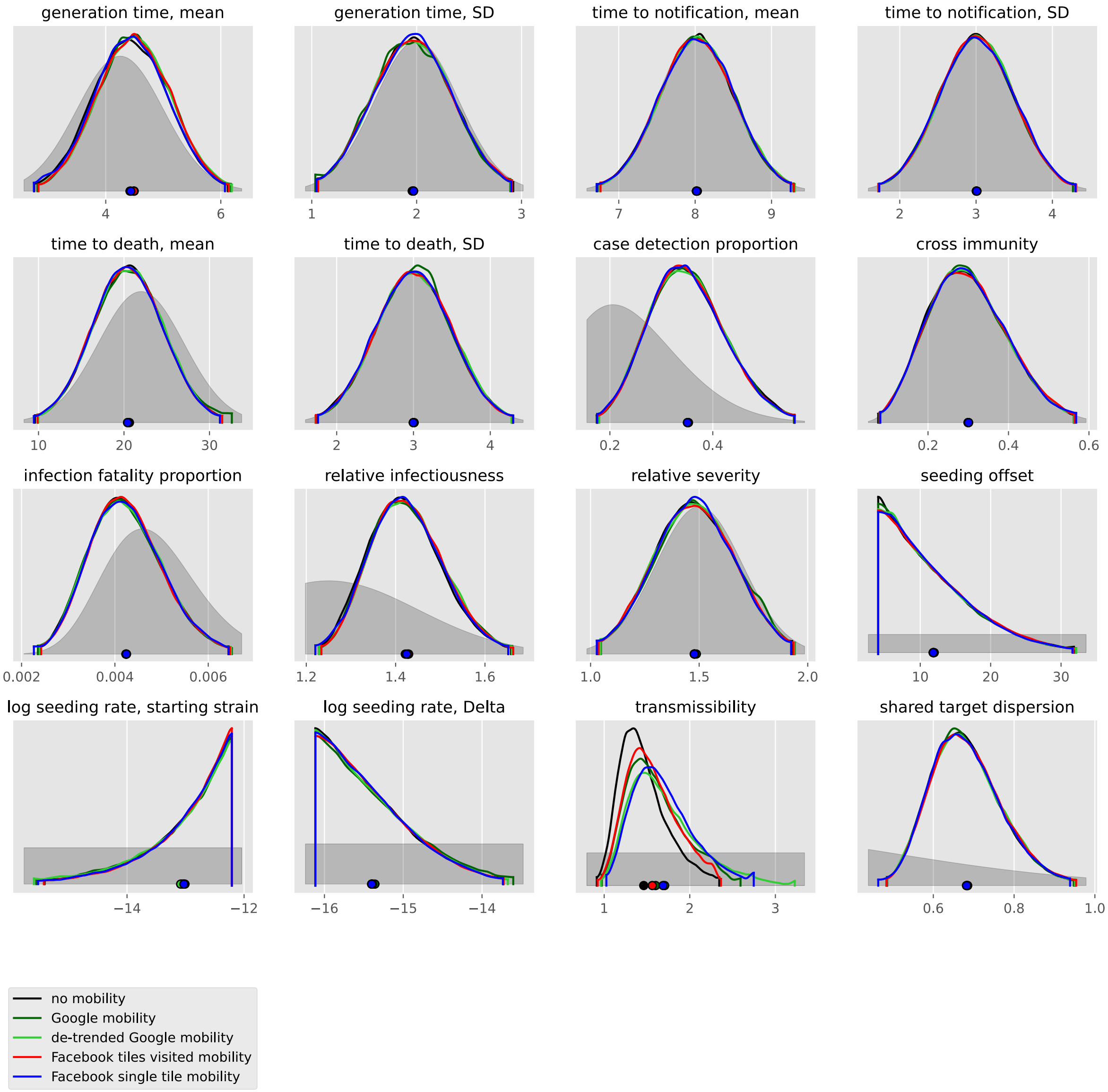

generation time, mean
4
6
generation time, SD
1
2
3
time to notification, mean
7
8
9
time to notification, SD
2
3
4
time to death, mean
10
20
30
time to death, SD
2
3
4
case detection proportion
0.2
0.4
cross immunity
0.2
0.4
0.6
infection fatality proportion
0.002
0.004
0.006
relative infectiousness
1.2
1.4
1.6
relative severity
1.0
1.5
2.0
seeding offset
10
20
30
log seeding rate, starting strain
−14
−12
log seeding rate, Delta
−16
−15
−14
transmissibility
1
2
3
shared target dispersion
0.6
0.8
1.0
no mobility
Google mobility
de-trended Google mobility
Facebook tiles visited mobility
Facebook single tile mobility

Congo

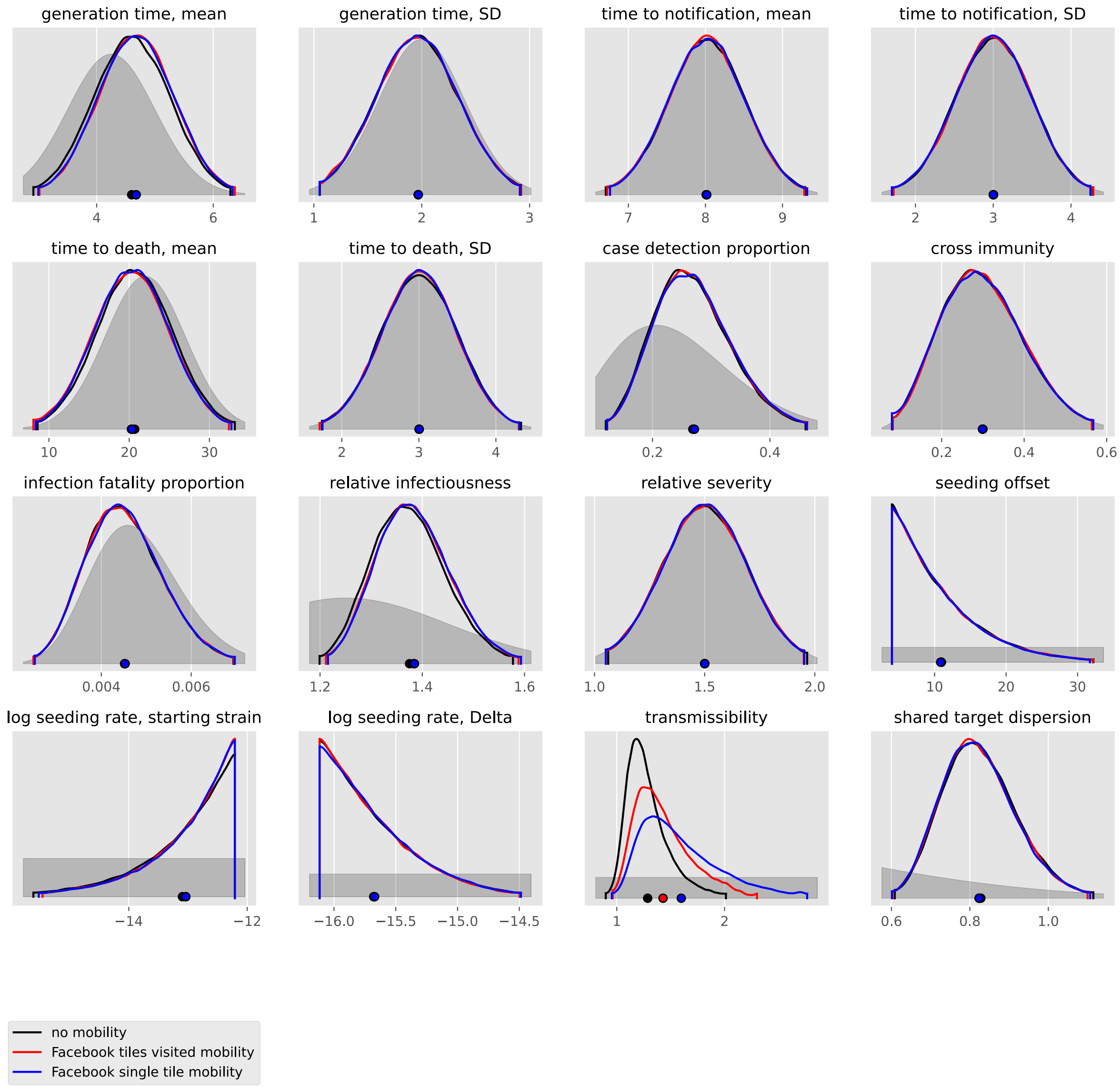

generation time, mean
generation time, SD
time to notification, mean
time to notification, SD
time to death, mean
time to death, SD
case detection proportion
cross immunity
infection fatality proportion
relative infectiousness
relative severity
seeding offset
log seeding rate, starting strain
log seeding rate, Delta
transmissibility
shared target dispersion
no mobility
Facebook tiles visited mobility
Facebook single tile mobility

Côte d'Ivoire

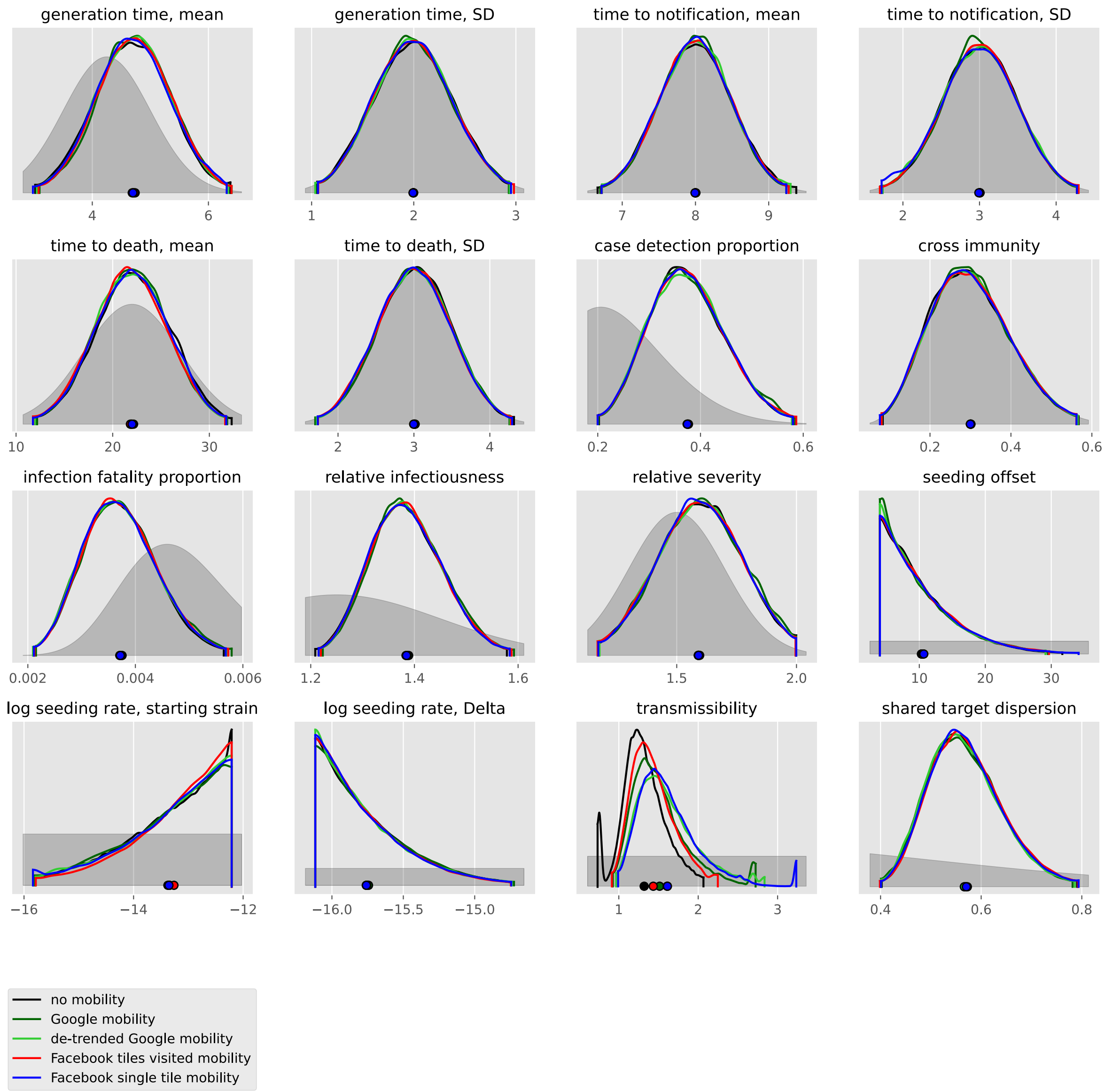

Egypt

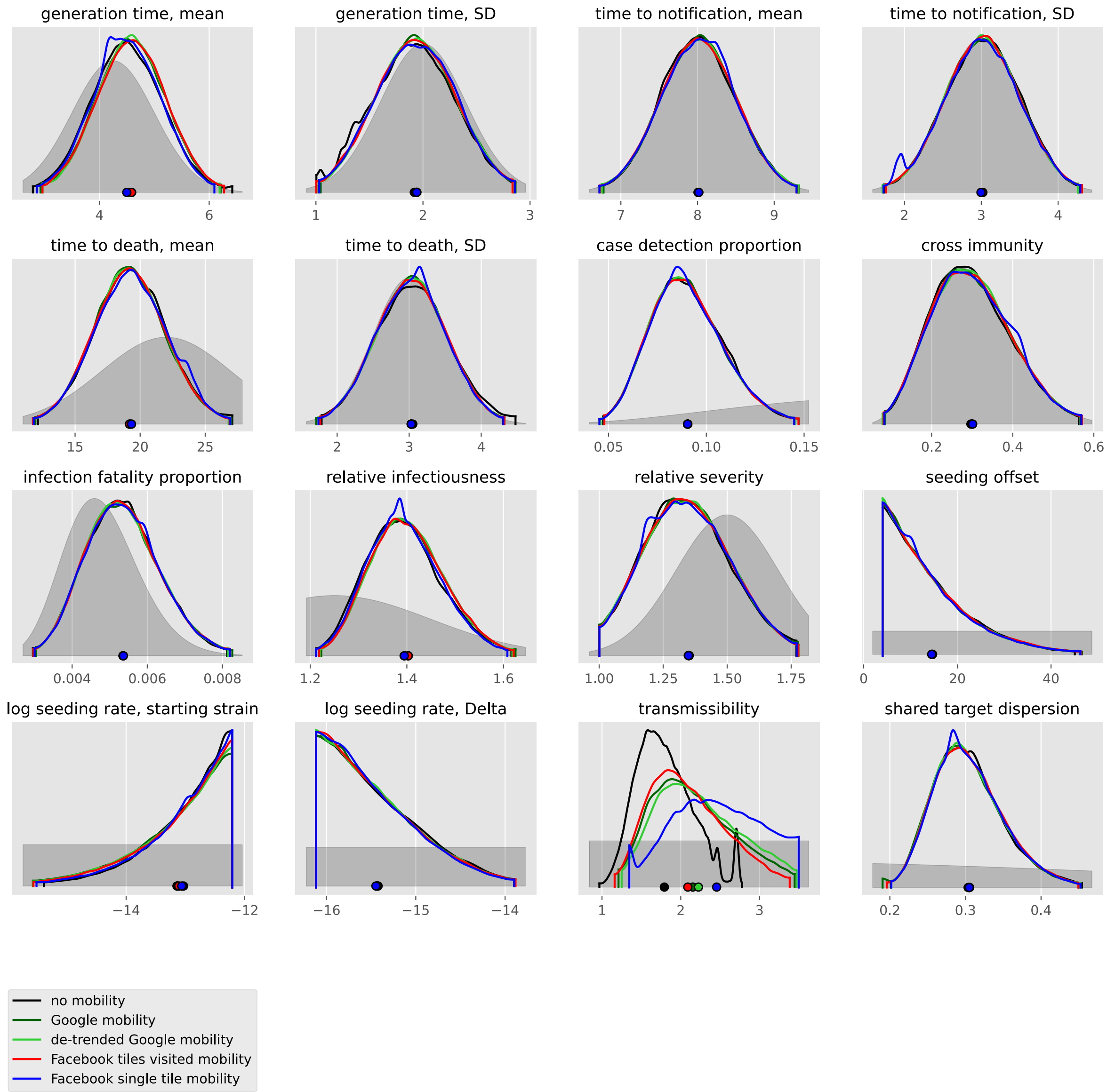
generation time, mean
generation time, SD
time to notification, mean
time to notification, SD
time to death, mean
time to death, SD
case detection proportion
cross immunity
infection fatality proportion
relative infectiousness
relative severity
seeding offset
log seeding rate, starting strain
log seeding rate, Delta
transmissibility
shared target dispersion
no mobility
Google mobility
de-trended Google mobility
Facebook tiles visited mobility
Facebook single tile mobility

Equatorial Guinea

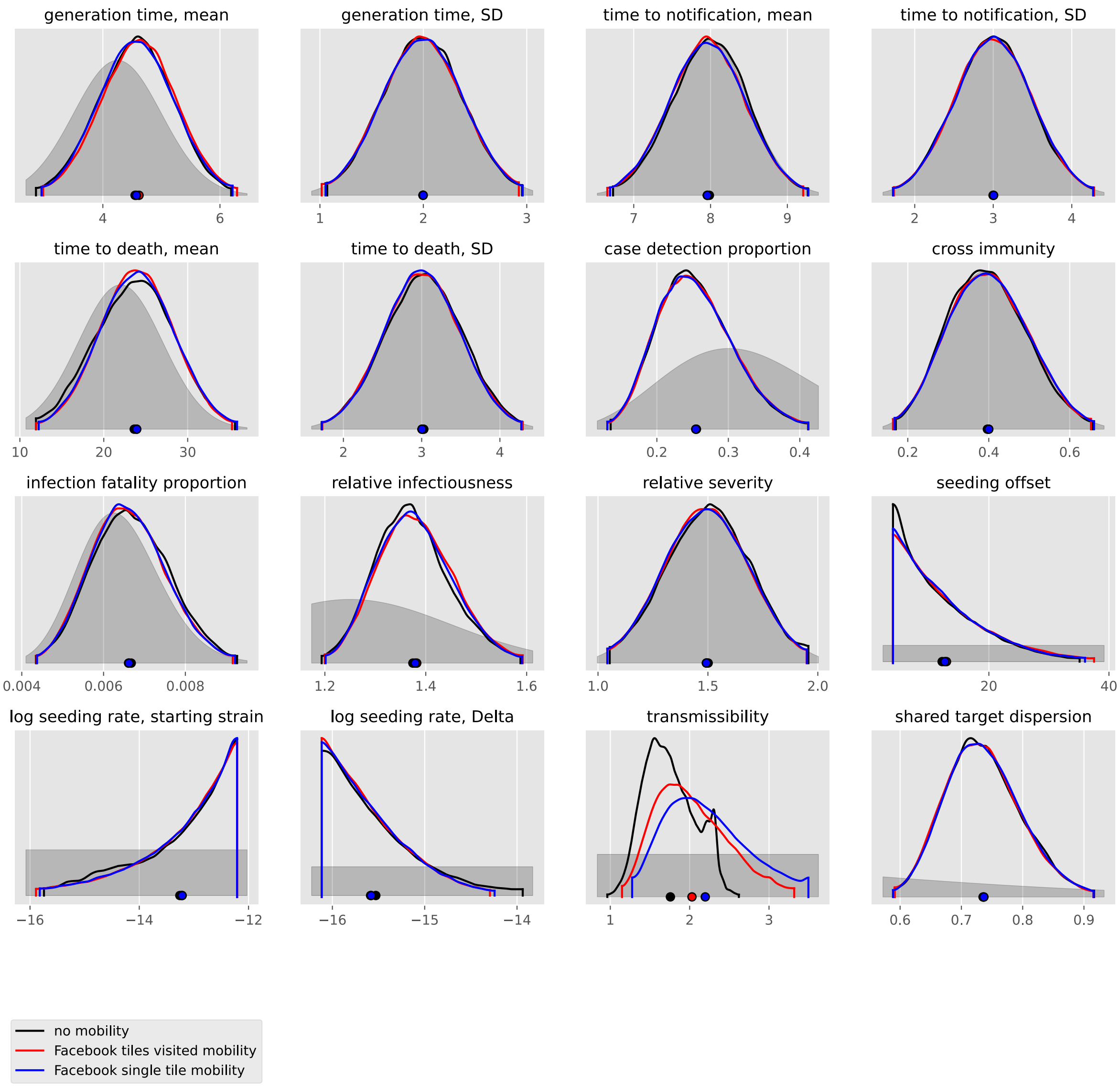

Ethiopia

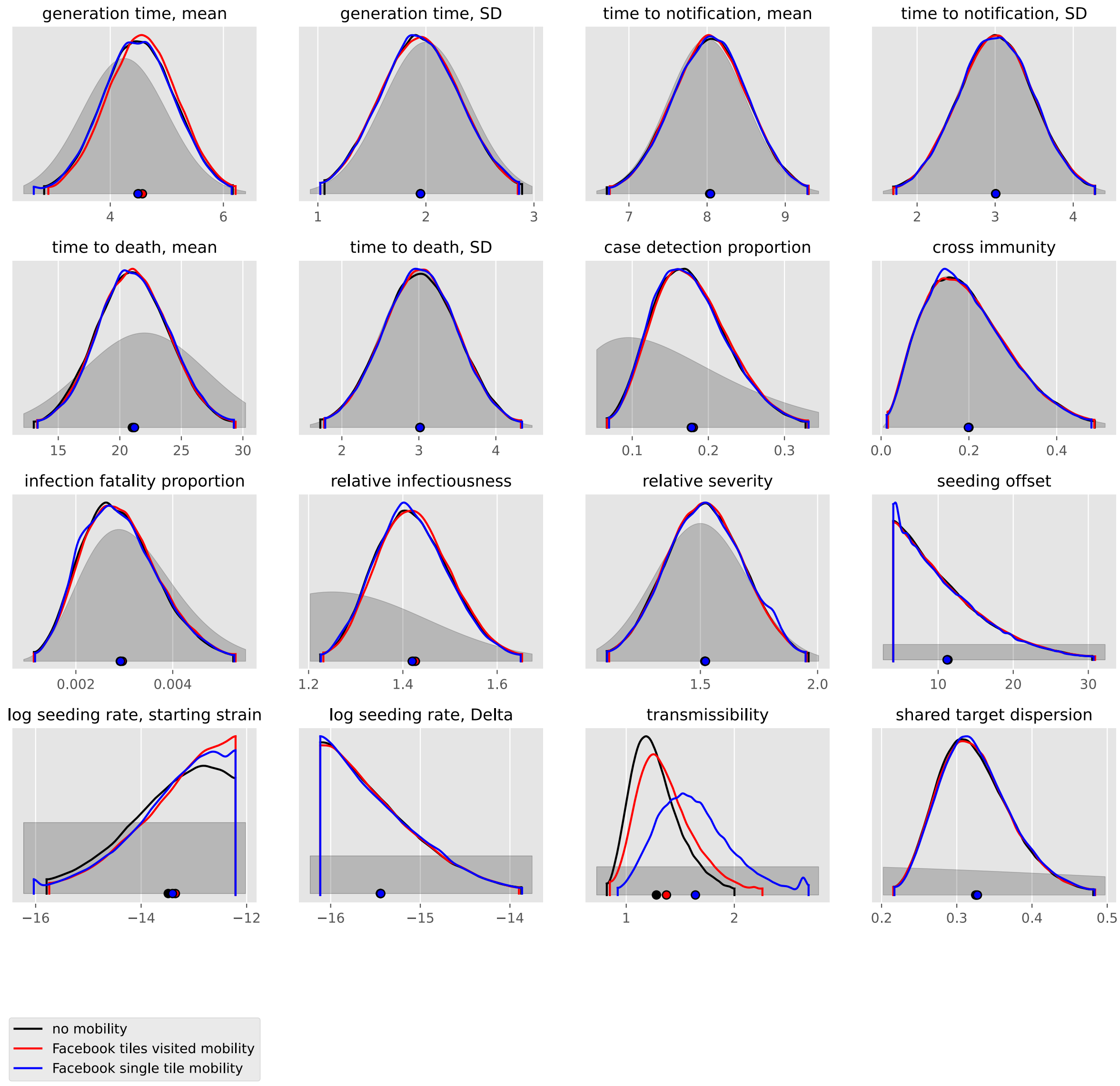
generation time, mean
generation time, SD
time to notification, mean
time to notification, SD
time to death, mean
time to death, SD
case detection proportion
cross immunity
infection fatality proportion
relative infectiousness
relative severity
seeding offset
log seeding rate, starting strain
log seeding rate, Delta
transmissibility
shared target dispersion
no mobility
Facebook tiles visited mobility
Facebook single tile mobility

Gabon

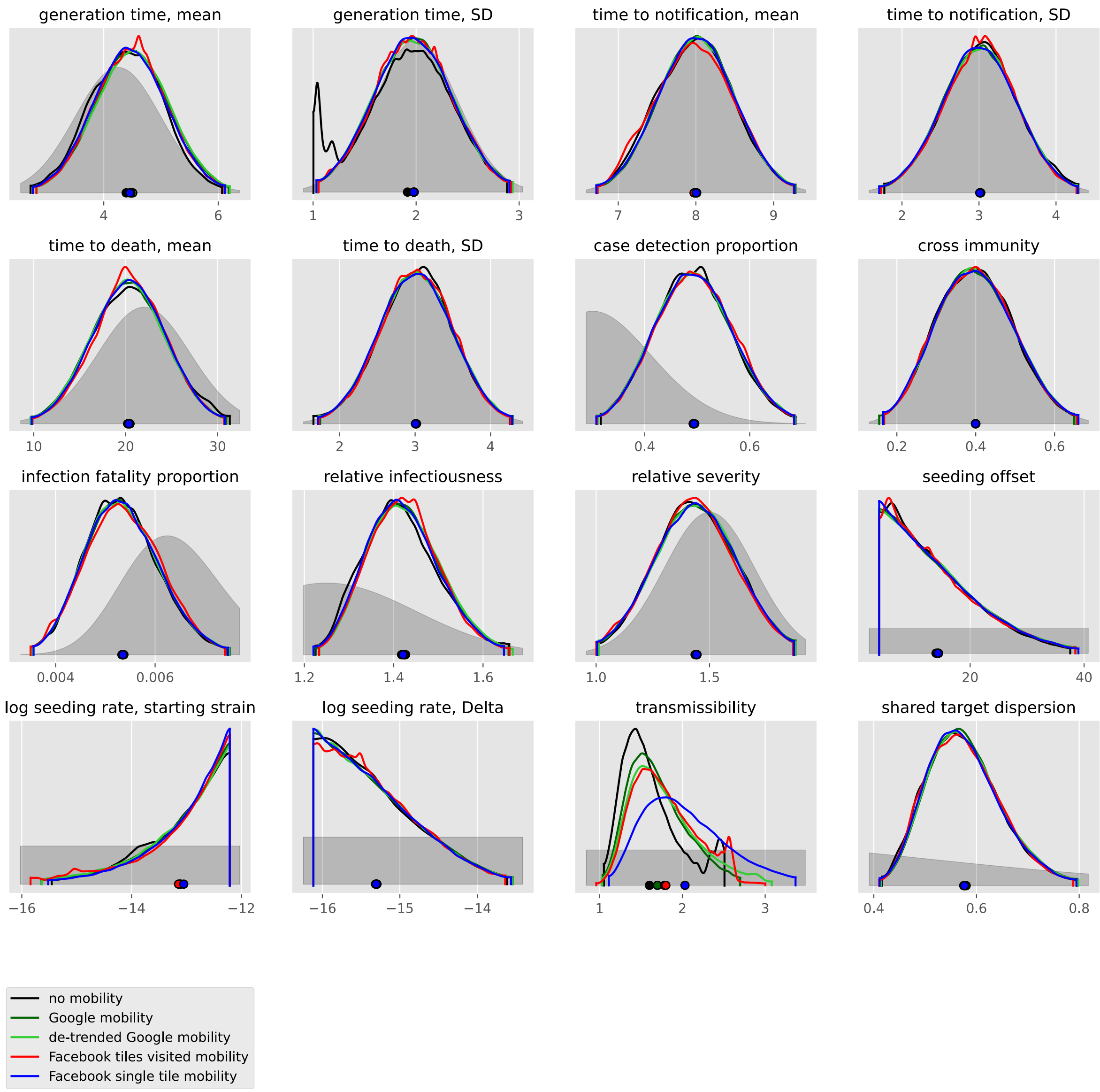

generation time, mean
4
6
generation time, SD
1
2
3
time to notification, mean
7
8
9
time to notification, SD
2
3
4
time to death, mean
10
20
30
time to death, SD
2
3
4
case detection proportion
0.4
0.6
cross immunity
0.2
0.4
0.6
infection fatality proportion
0.004
0.006
relative infectiousness
1.2
1.4
1.6
relative severity
1.0
1.5
seeding offset
20
40
log seeding rate, starting strain
−16
−14
−12
log seeding rate, Delta
−16
−15
−14
transmissibility
1
2
3
shared target dispersion
0.4
0.6
0.8
no mobility
Google mobility
de-trended Google mobility
Facebook tiles visited mobility
Facebook single tile mobility

Guinea

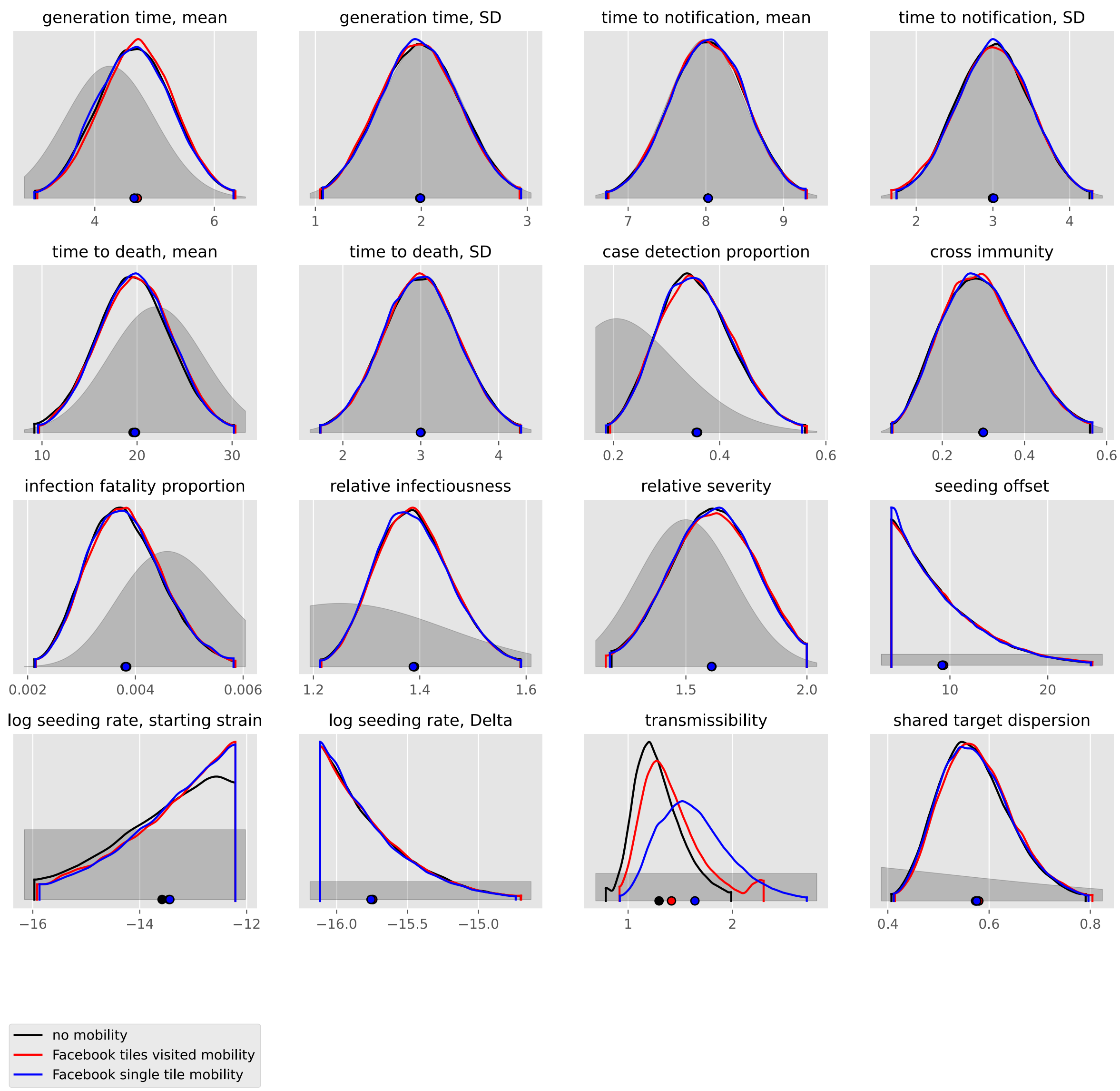

generation time, mean
4
6
generation time, SD
1
2
3
time to notification, mean
7
8
9
time to notification, SD
2
3
4
time to death, mean
10
20
30
time to death, SD
2
3
4
case detection proportion
0.2
0.4
0.6
cross immunity
0.2
0.4
0.6
infection fatality proportion
0.002
0.004
0.006
relative infectiousness
1.2
1.4
1.6
relative severity
1.5
2.0
seeding offset
10
20
log seeding rate, starting strain
−16
−14
−12
log seeding rate, Delta
−16.0
−15.5
−15.0
transmissibility
1
2
shared target dispersion
0.4
0.6
0.8
no mobility
Facebook tiles visited mobility
Facebook single tile mobility

Guinea-Bissau

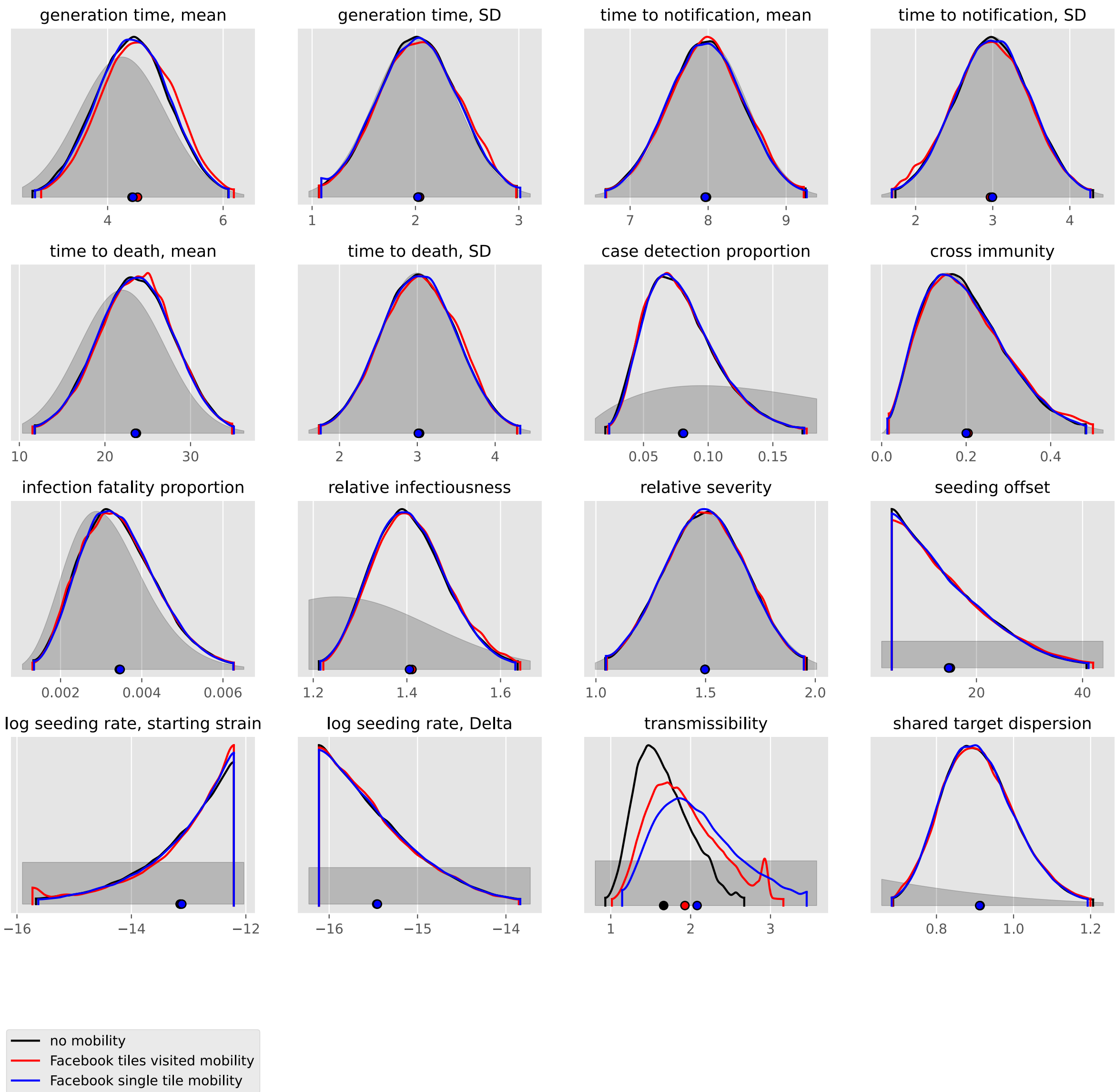

Kenya

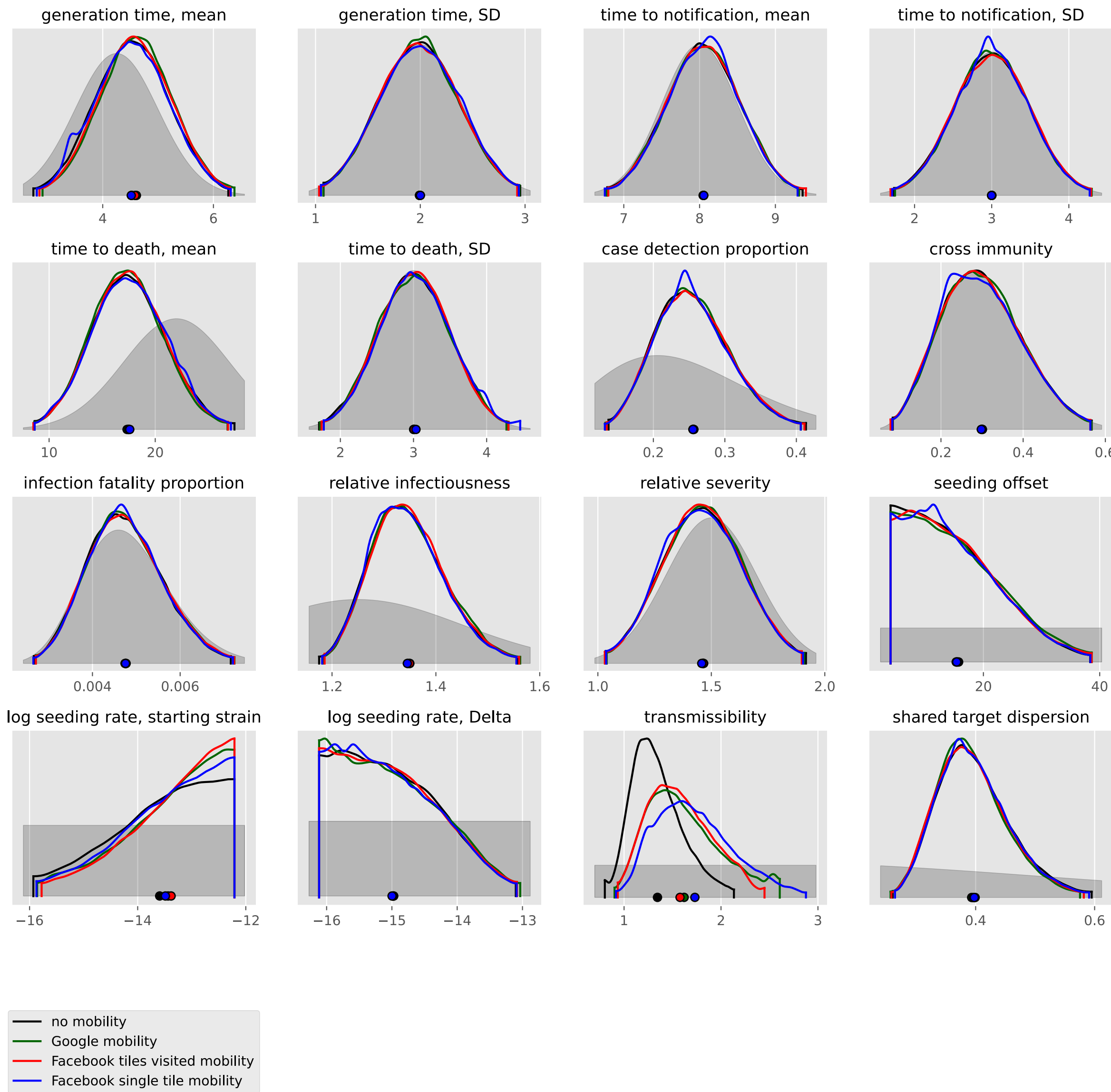

generation time, mean
4
6
generation time, SD
1
2
3
time to notification, mean
7
8
9
time to notification, SD
2
3
4
time to death, mean
10
20
time to death, SD
2
3
4
case detection proportion
0.2
0.3
0.4
cross immunity
0.2
0.4
0.6
infection fatality proportion
0.004
0.006
relative infectiousness
1.2
1.4
1.6
relative severity
1.0
1.5
2.0
seeding offset
20
40
log seeding rate, starting strain
−16
−14
−12
log seeding rate, Delta
−16
−15
−14
−13
transmissibility
1
2
3
shared target dispersion
0.4
0.6
no mobility
Google mobility
Facebook tiles visited mobility
Facebook single tile mobility

Lesotho

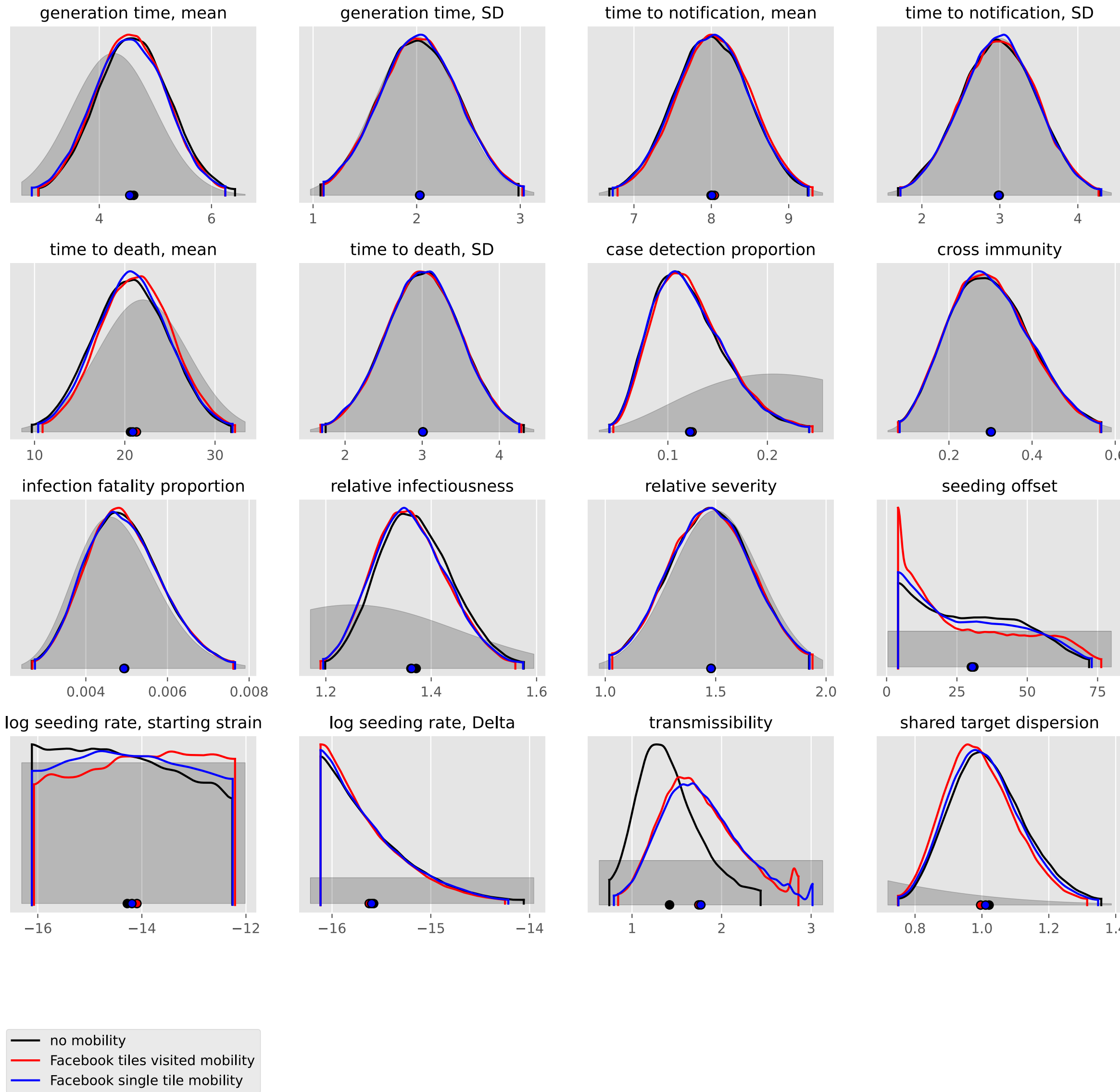
generation time, mean
generation time, SD
time to notification, mean
time to notification, SD
time to death, mean
time to death, SD
case detection proportion
cross immunity
infection fatality proportion
relative infectiousness
relative severity
seeding offset
log seeding rate, starting strain
log seeding rate, Delta
transmissibility
shared target dispersion
no mobility
Facebook tiles visited mobility
Facebook single tile mobility

Liberia

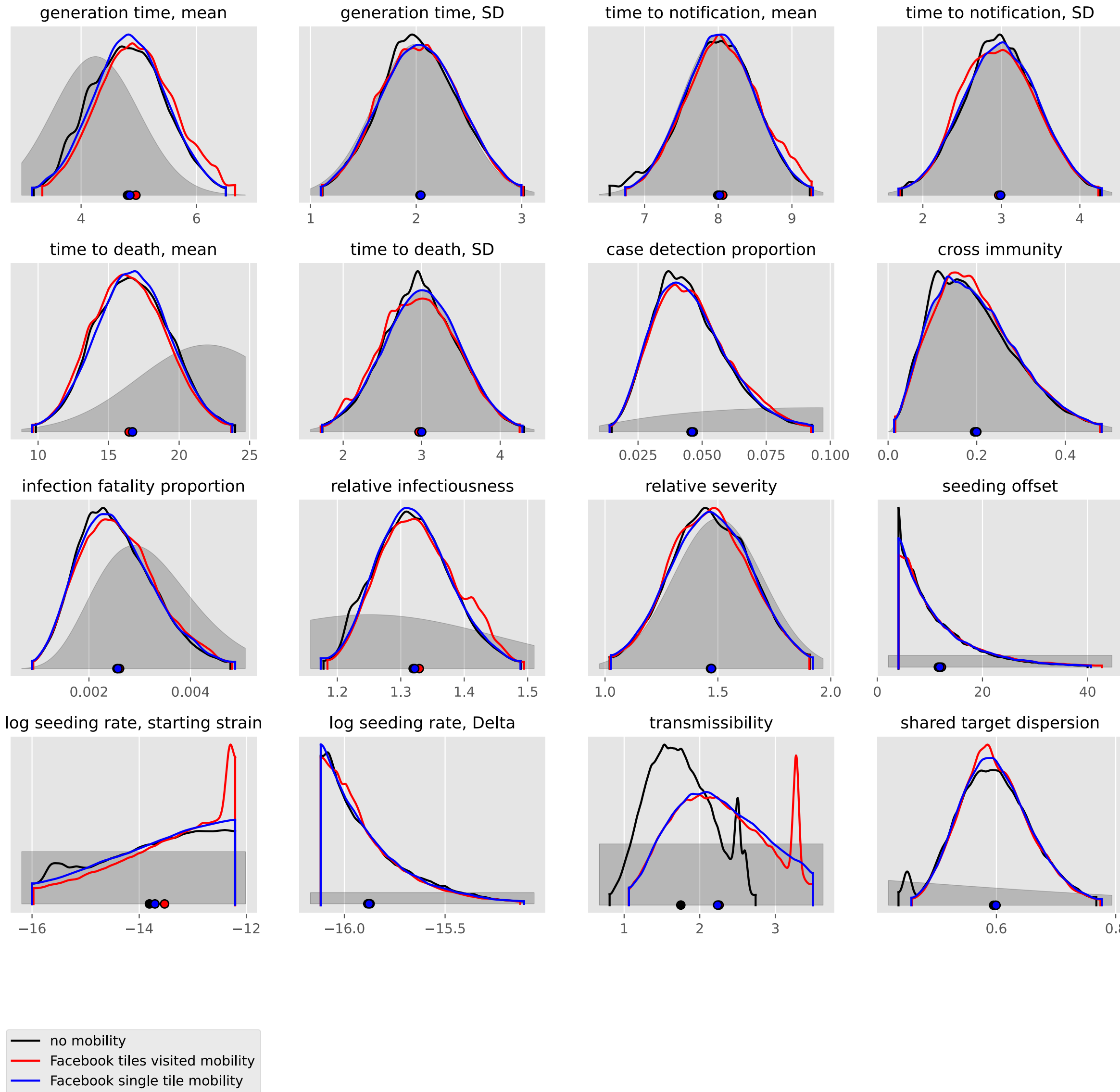

generation time, mean
4
6
generation time, SD
1
2
3
time to notification, mean
7
8
9
time to notification, SD
2
3
4
time to death, mean
10
15
20
25
time to death, SD
2
3
4
case detection proportion
0.025
0.050
0.075
0.100
cross immunity
0.0
0.2
0.4
infection fatality proportion
0.002
0.004
relative infectiousness
1.2
1.3
1.4
1.5
relative severity
1.0
1.5
2.0
seeding offset
0
20
40
log seeding rate, starting strain
−16
−14
−12
log seeding rate, Delta
−16.0
−15.5
transmissibility
1
2
3
shared target dispersion
0.6
0.8
no mobility
Facebook tiles visited mobility
Facebook single tile mobility

Libya

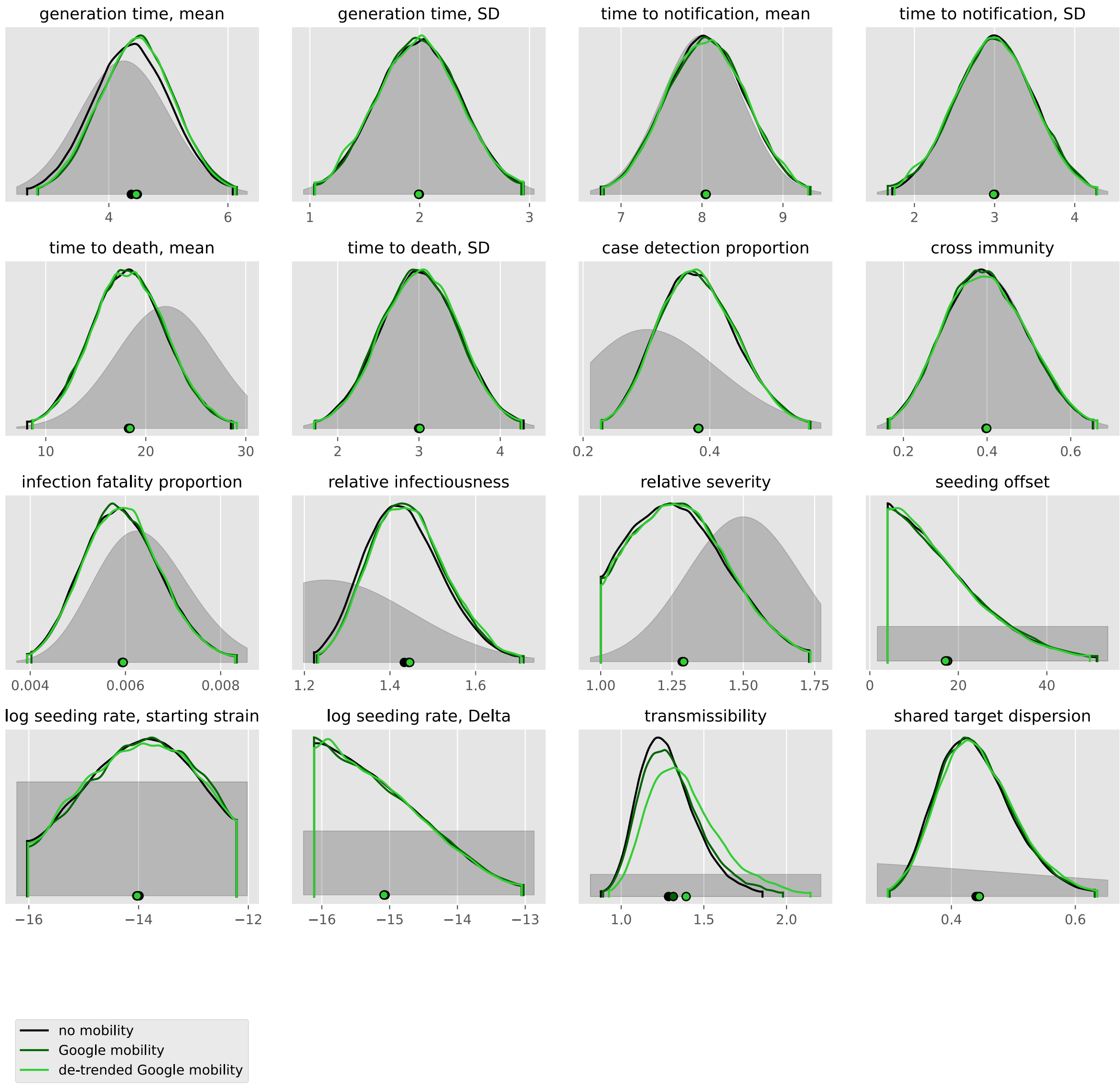

generation time, mean
4
6
generation time, SD
1
2
3
time to notification, mean
7
8
9
time to notification, SD
2
3
4
time to death, mean
10
20
30
time to death, SD
2
3
4
case detection proportion
0.2
0.4
cross immunity
0.2
0.4
0.6
infection fatality proportion
0.004
0.006
0.008
relative infectiousness
1.2
1.4
1.6
relative severity
1.00
1.25
1.50
1.75
seeding offset
0
20
40
log seeding rate, starting strain
−16
−14
−12
log seeding rate, Delta
−16
−15
−14
−13
transmissibility
1.0
1.5
2.0
shared target dispersion
0.4
0.6
no mobility
Google mobility
de-trended Google mobility

Madagascar

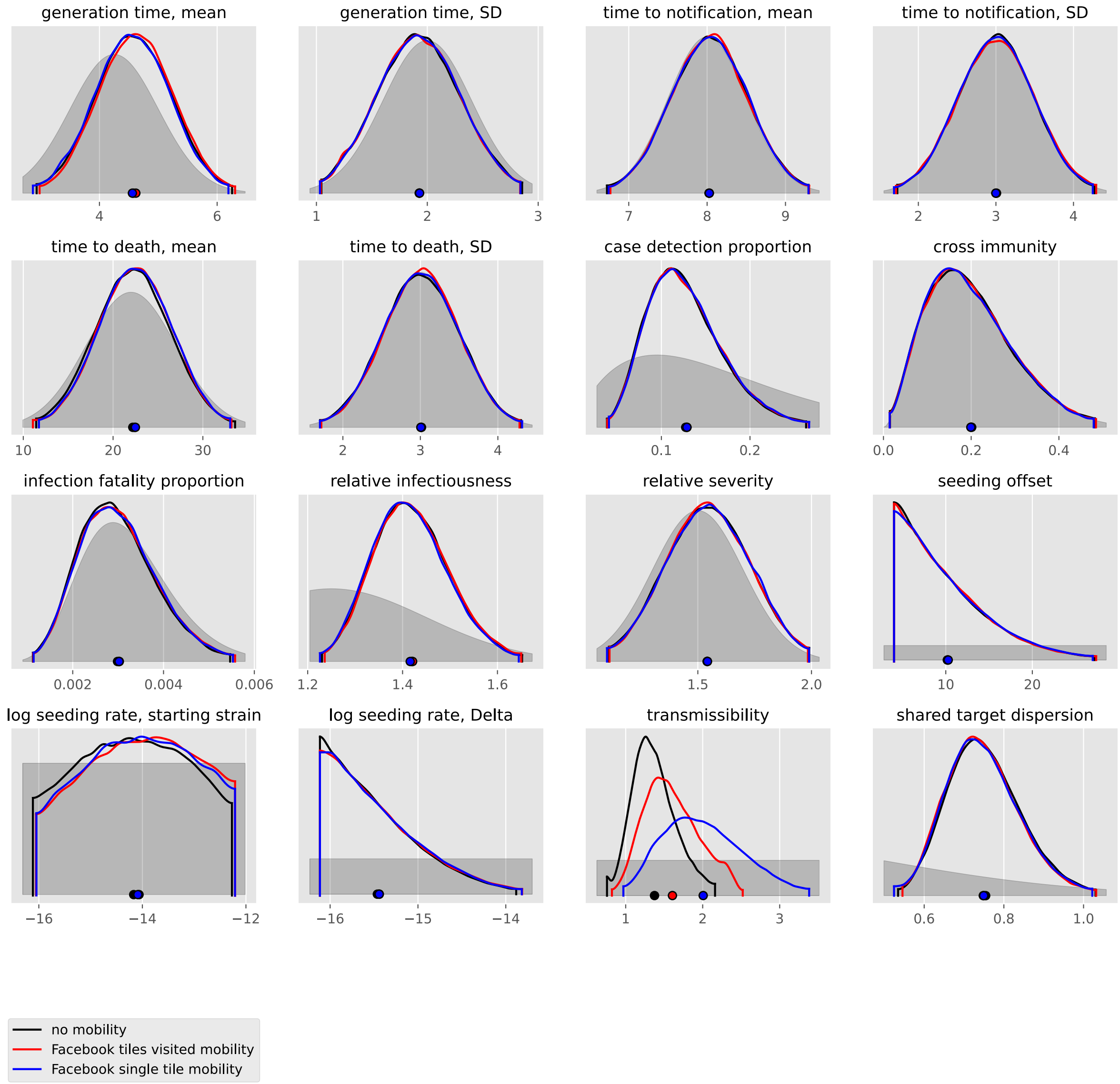
generation time, mean
generation time, SD
time to notification, mean
time to notification, SD
time to death, mean
time to death, SD
case detection proportion
cross immunity
infection fatality proportion
relative infectiousness
relative severity
seeding offset
log seeding rate, starting strain
log seeding rate, Delta
transmissibility
shared target dispersion
no mobility
Facebook tiles visited mobility
Facebook single tile mobility

Malawi

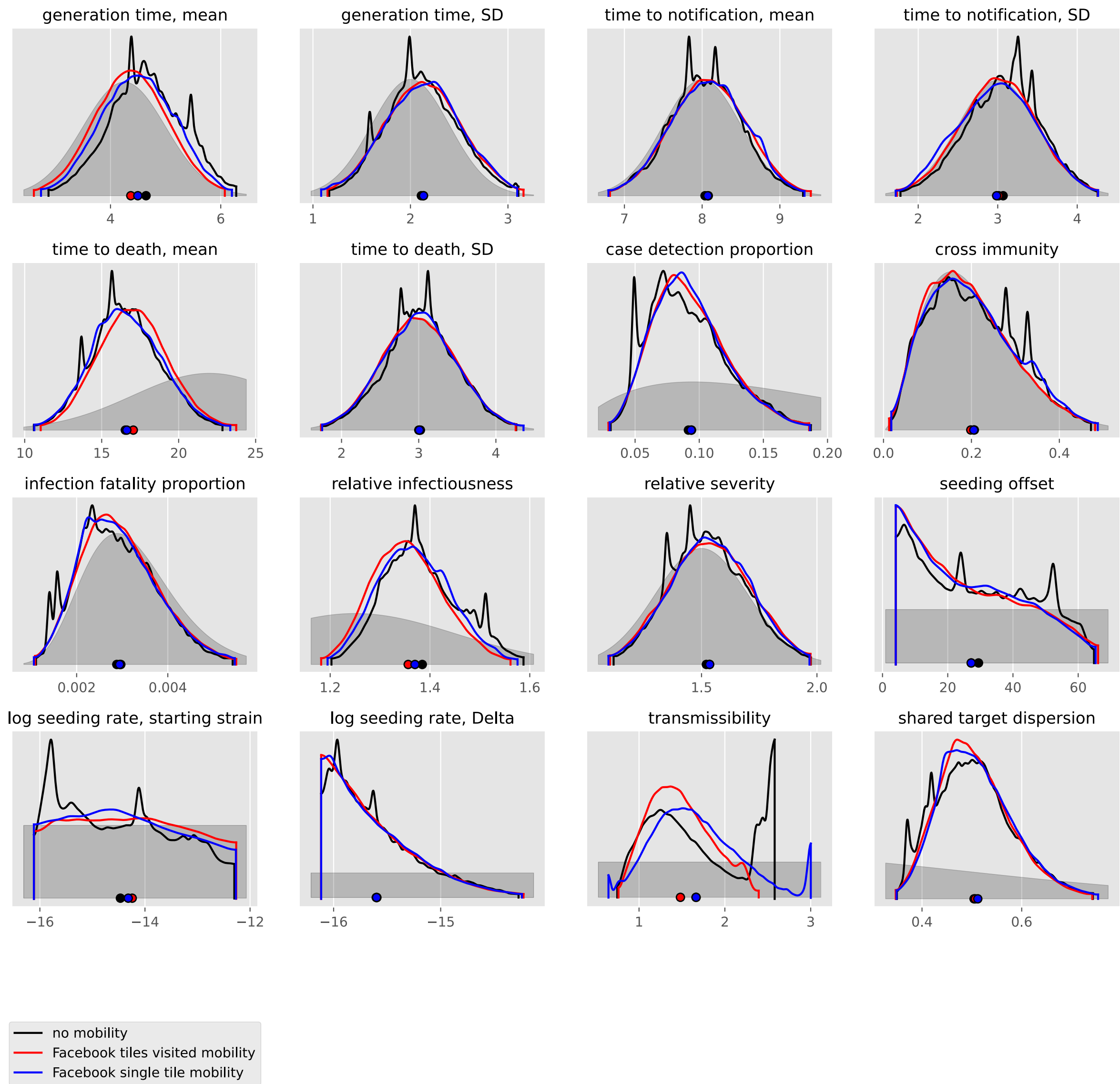
generation time, mean
4
6
generation time, SD
1
2
3
time to notification, mean
7
8
9
time to notification, SD
2
3
4
time to death, mean
10
15
20
25
time to death, SD
2
3
4
case detection proportion
0.05
0.10
0.15
0.20
cross immunity
0.0
0.2
0.4
infection fatality proportion
0.002
0.004
relative infectiousness
1.2
1.4
1.6
relative severity
1.5
2.0
seeding offset
0
20
40
60
log seeding rate, starting strain
−16
−14
−12
log seeding rate, Delta
−16
−15
transmissibility
1
2
3
shared target dispersion
0.4
0.6
no mobility
Facebook tiles visited mobility
Facebook single tile mobility

Mali

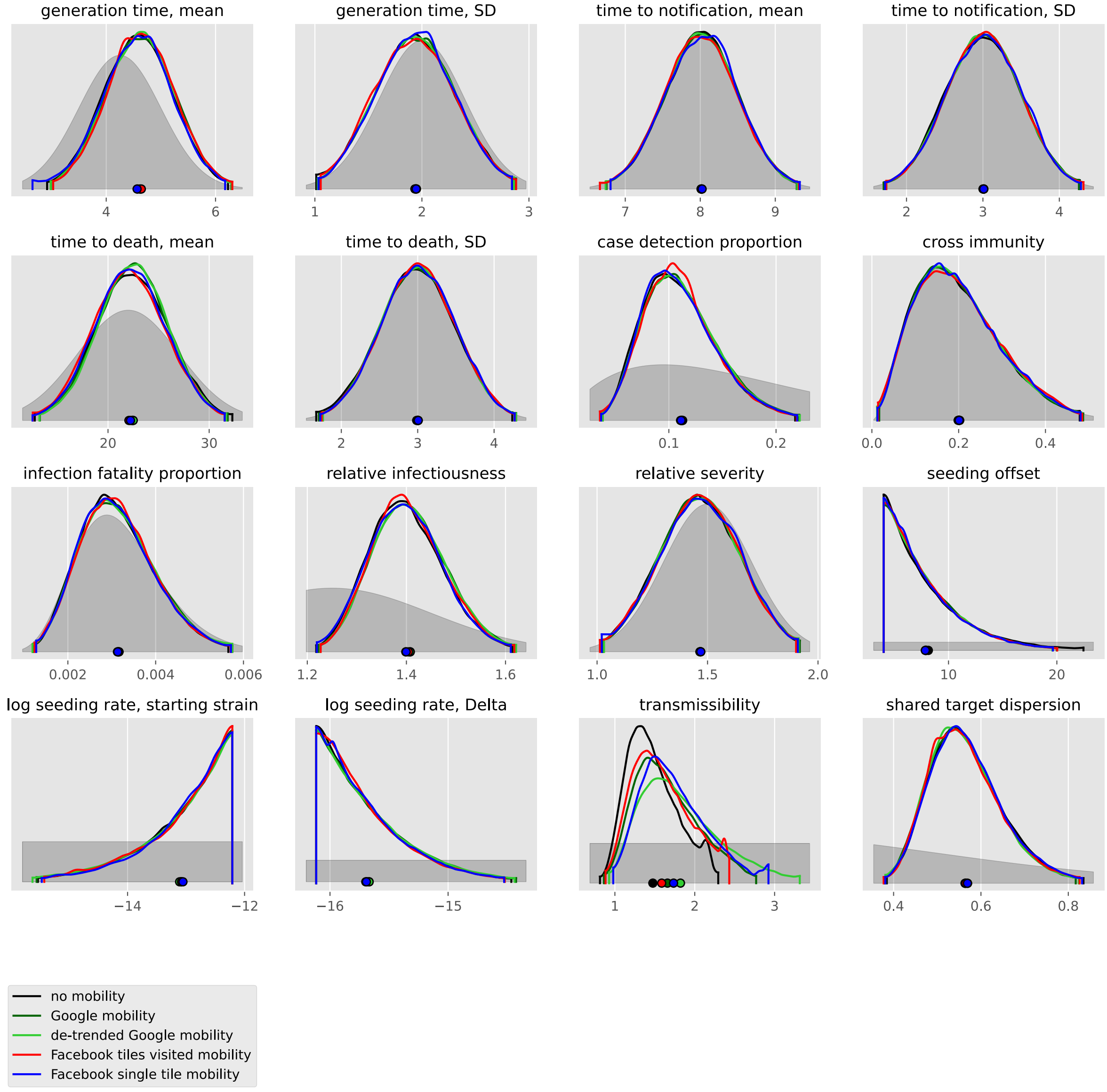
generation time, mean
generation time, SD
time to notification, mean
time to notification, SD
time to death, mean
time to death, SD
case detection proportion
cross immunity
infection fatality proportion
relative infectiousness
relative severity
seeding offset
log seeding rate, starting strain
log seeding rate, Delta
transmissibility
shared target dispersion
no mobility
Google mobility
de-trended Google mobility
Facebook tiles visited mobility
Facebook single tile mobility

Morocco

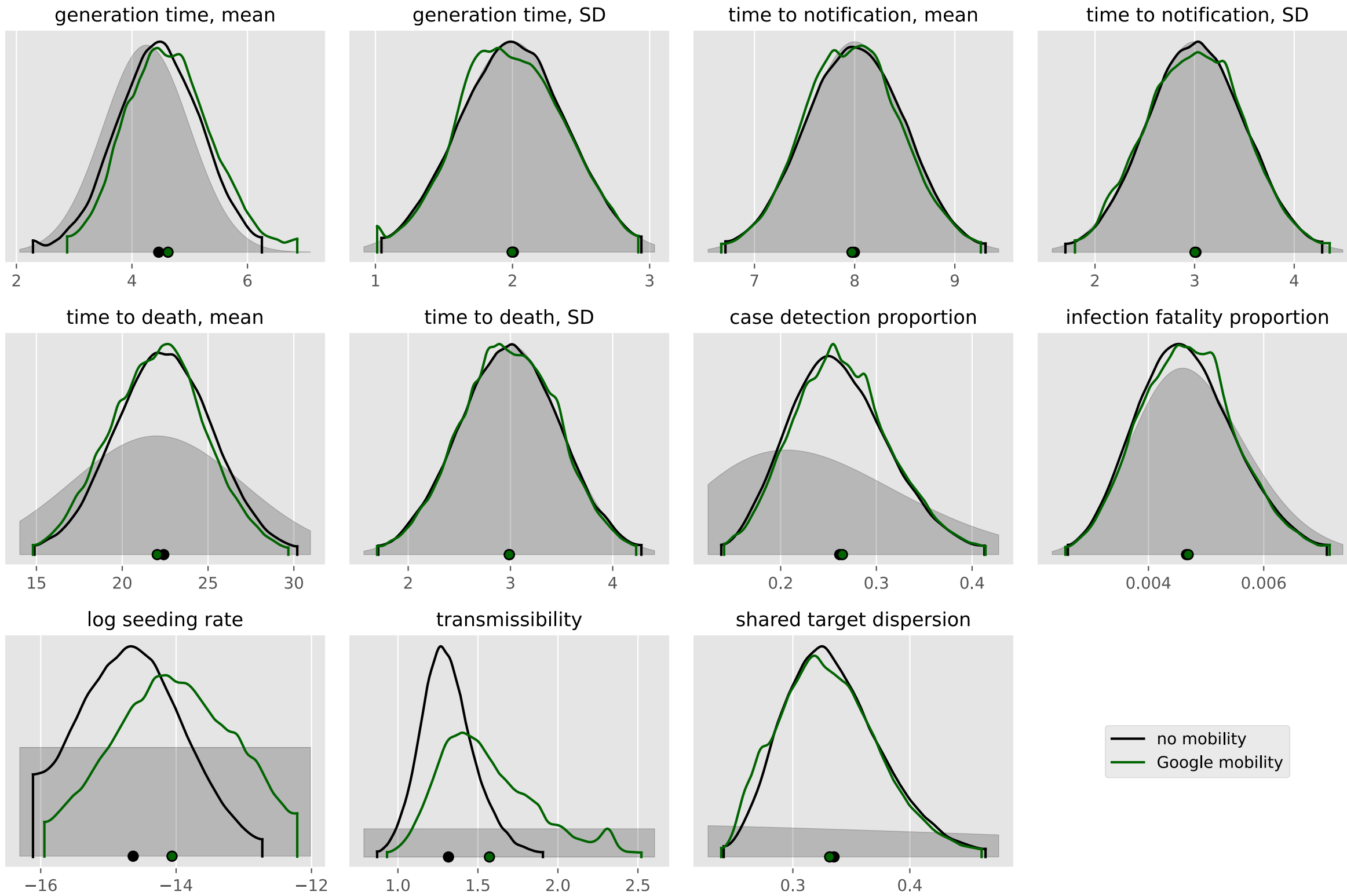

generation time, mean
2
4
6
generation time, SD
1
2
3
time to notification, mean
7
8
9
time to notification, SD
2
3
4
time to death, mean
15
20
25
30
time to death, SD
2
3
4
case detection proportion
0.2
0.3
0.4
infection fatality proportion
0.004
0.006
log seeding rate
−16
−14
−12
transmissibility
1.0
1.5
2.0
2.5
shared target dispersion
0.3
0.4
no mobility
Google mobility

Mozambique

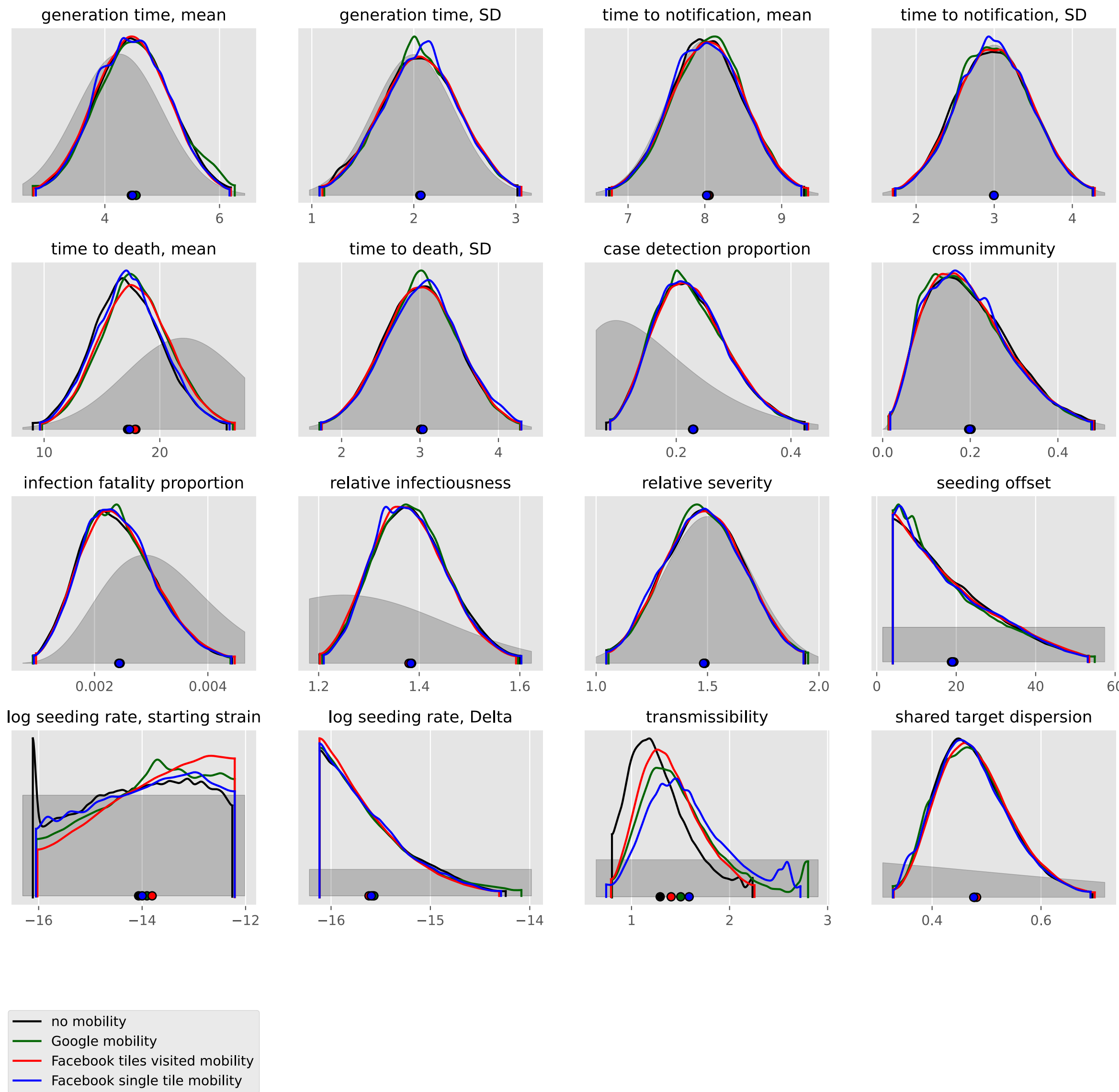

generation time, mean
generation time, SD
time to notification, mean
time to notification, SD
time to death, mean
time to death, SD
case detection proportion
cross immunity
infection fatality proportion
relative infectiousness
relative severity
seeding offset
log seeding rate, starting strain
log seeding rate, Delta
transmissibility
shared target dispersion
no mobility
Google mobility
Facebook tiles visited mobility
Facebook single tile mobility

Nigeria

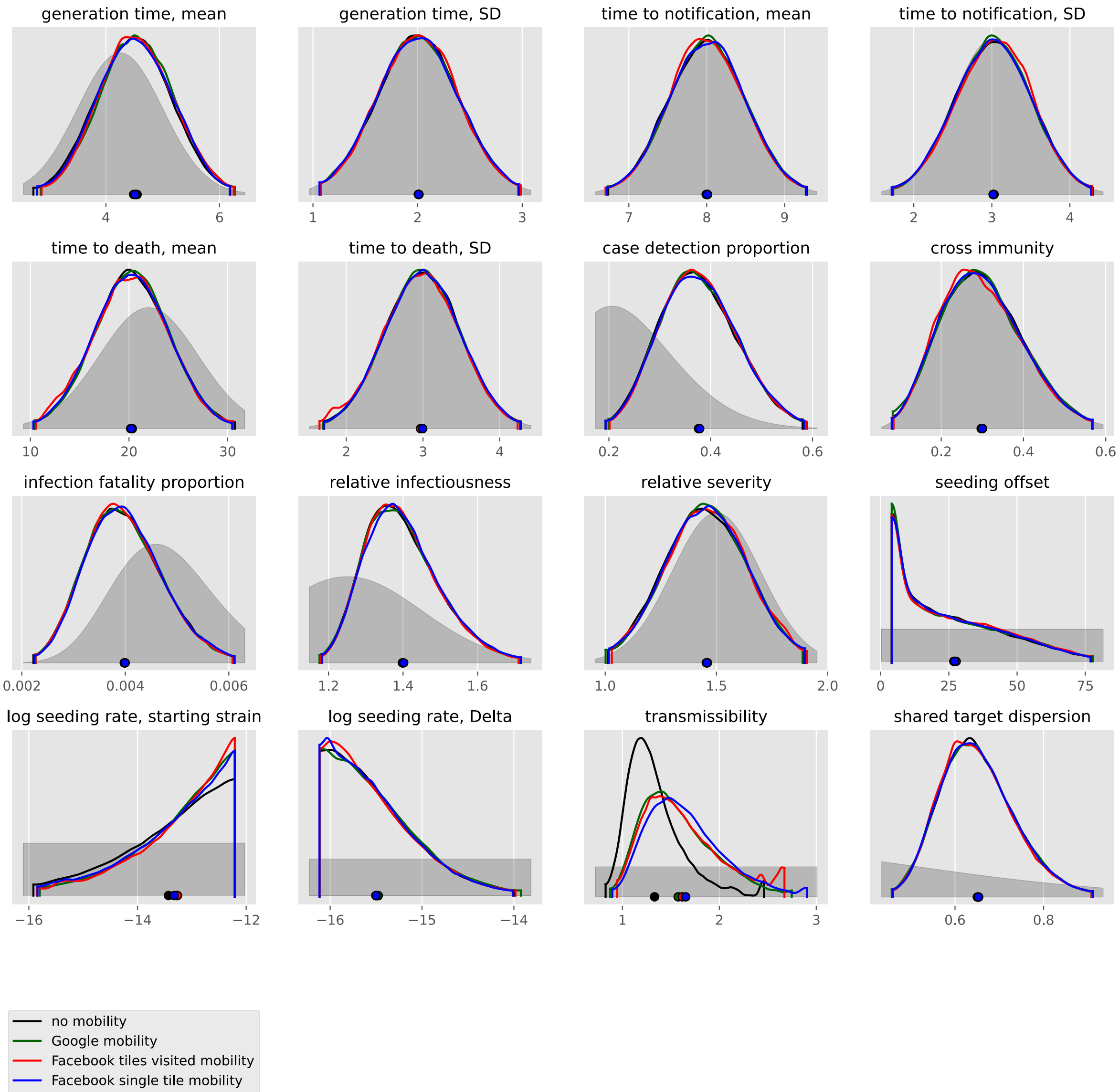
generation time, mean
4
6
generation time, SD
1
2
3
time to notification, mean
7
8
9
time to notification, SD
2
3
4
time to death, mean
10
20
30
time to death, SD
2
3
4
case detection proportion
0.2
0.4
0.6
cross immunity
0.2
0.4
0.6
infection fatality proportion
0.002
0.004
0.006
relative infectiousness
1.2
1.4
1.6
relative severity
1.0
1.5
2.0
seeding offset
0
25
50
75
log seeding rate, starting strain
−16
−14
−12
log seeding rate, Delta
−16
−15
−14
transmissibility
1
2
3
shared target dispersion
0.6
0.8
no mobility
Google mobility
Facebook tiles visited mobility
Facebook single tile mobility

Rwanda

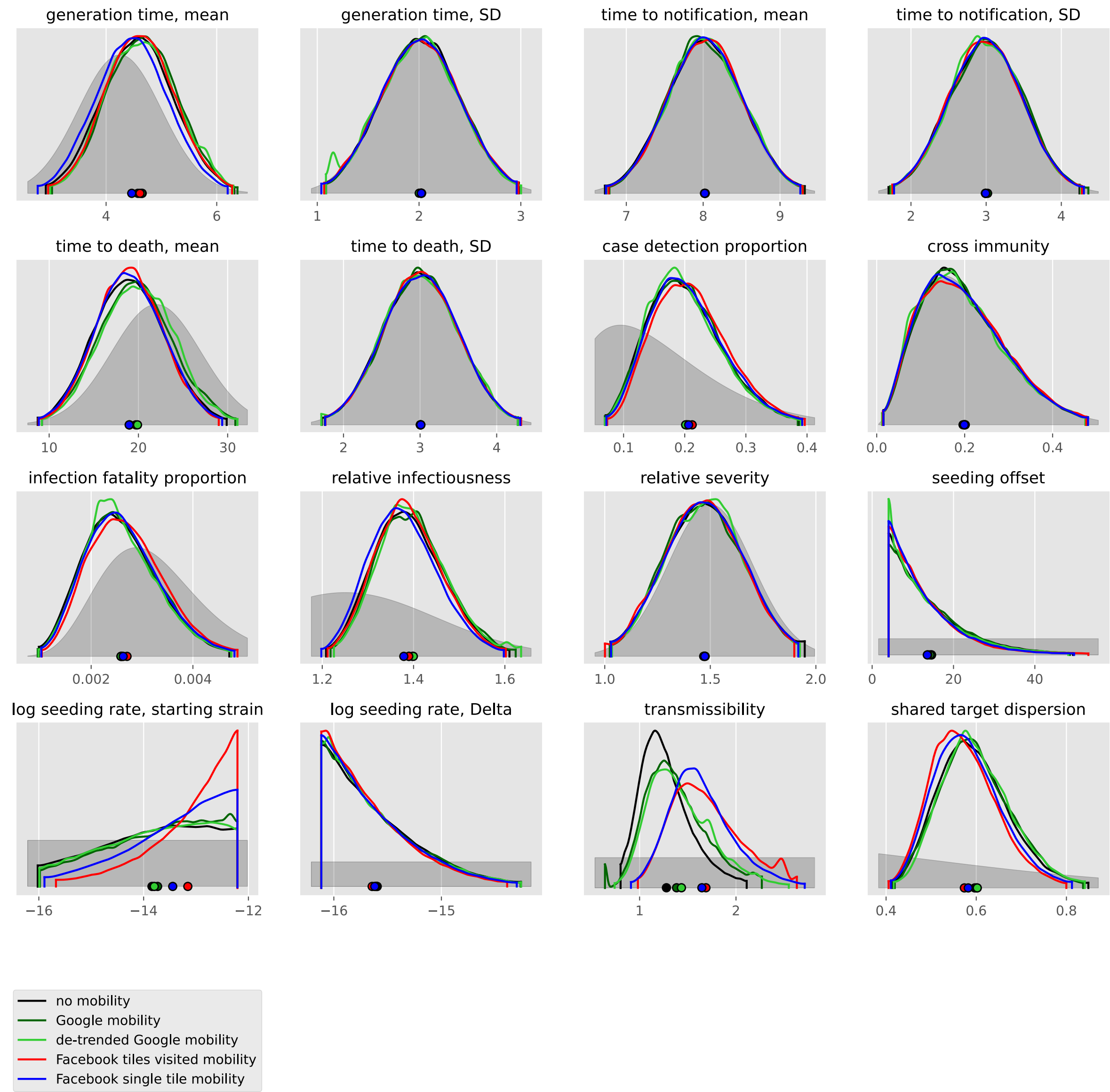
generation time, mean
generation time, SD
time to notification, mean
time to notification, SD
time to death, mean
time to death, SD
case detection proportion
cross immunity
infection fatality proportion
relative infectiousness
relative severity
seeding offset
log seeding rate, starting strain
log seeding rate, Delta
transmissibility
shared target dispersion
no mobility
Google mobility
de-trended Google mobility
Facebook tiles visited mobility
Facebook single tile mobility

Sao Tome and Principe

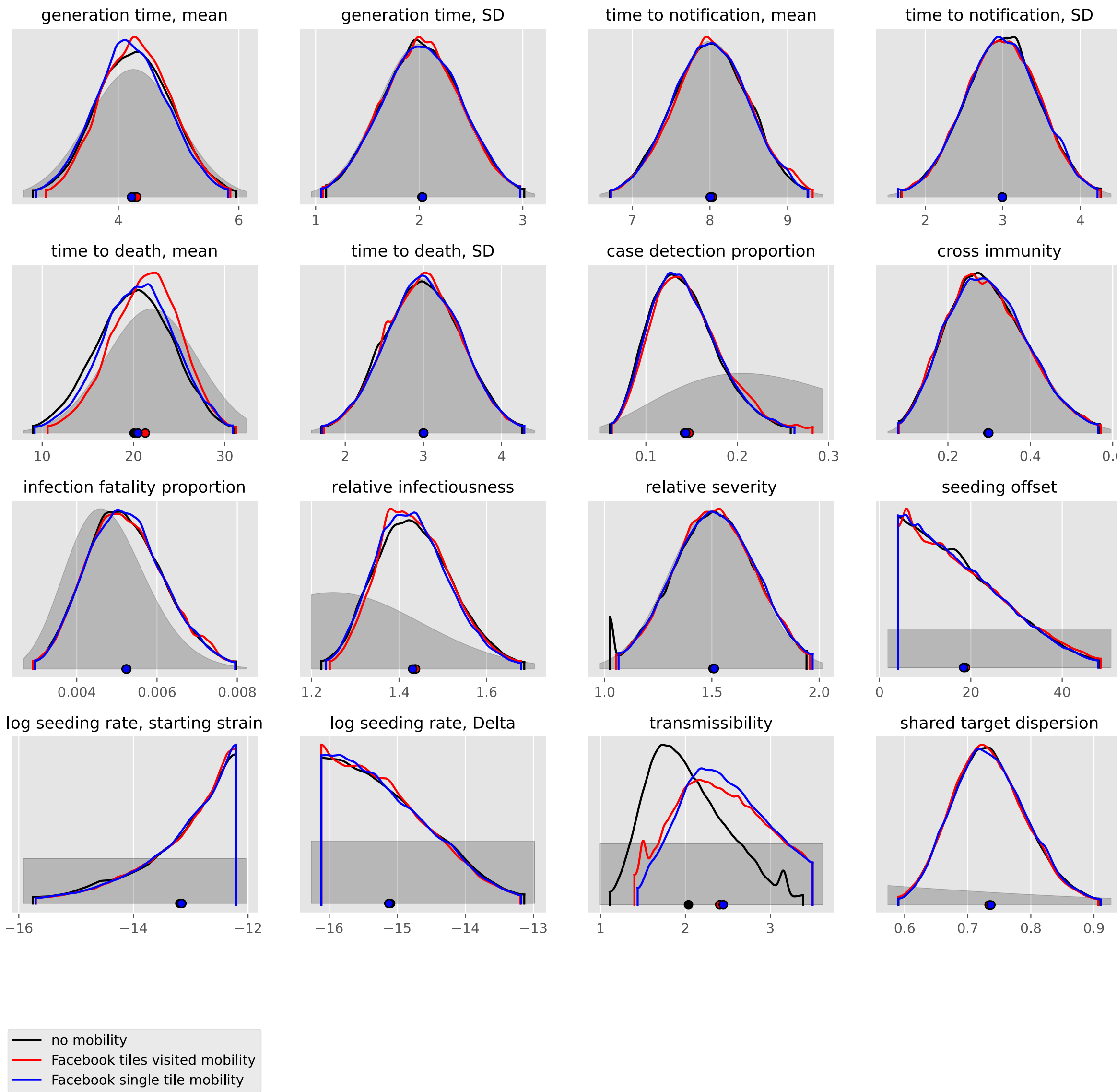

Senegal

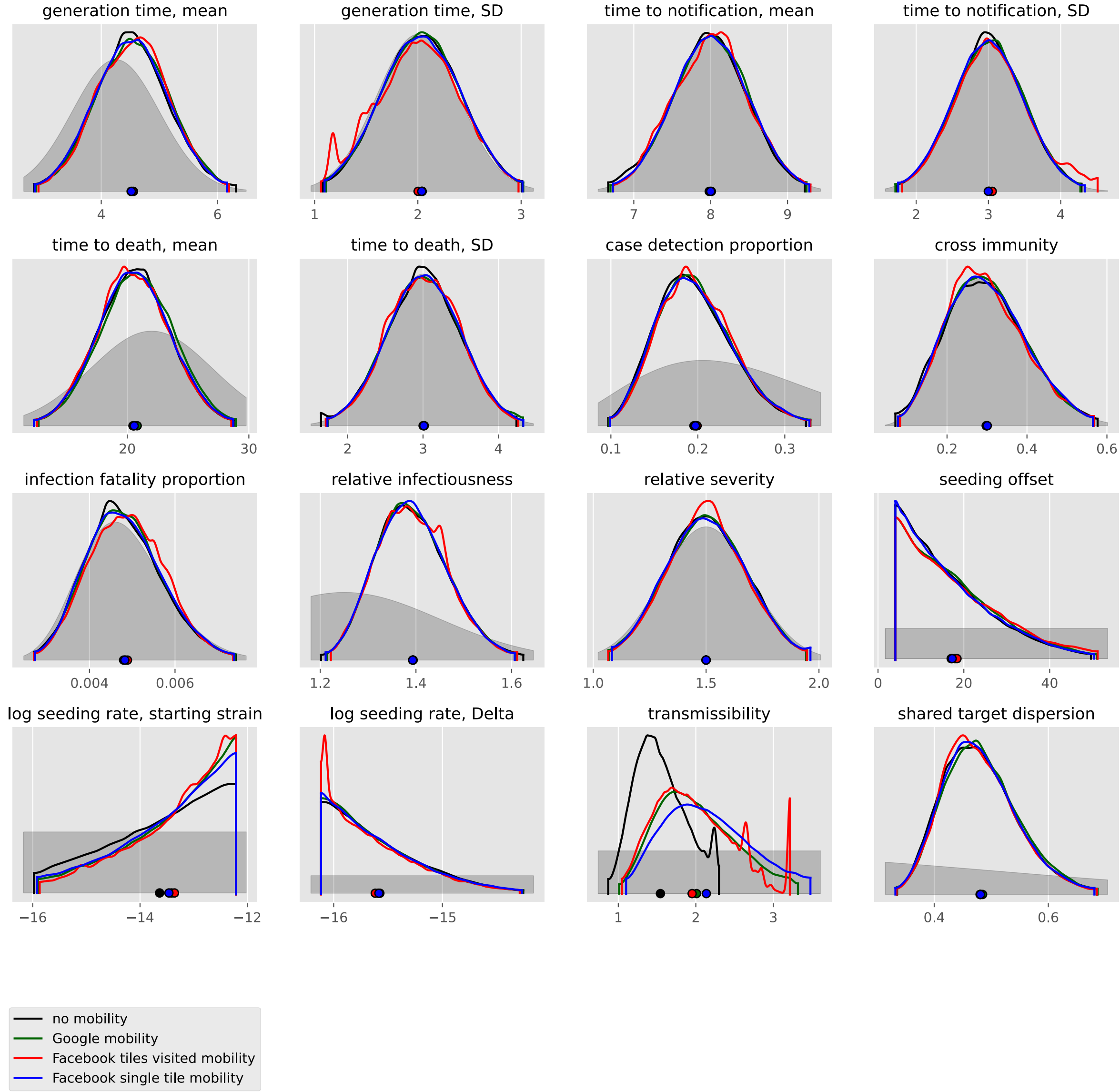

generation time, mean
generation time, SD
time to notification, mean
time to notification, SD
time to death, mean
time to death, SD
case detection proportion
cross immunity
infection fatality proportion
relative infectiousness
relative severity
seeding offset
log seeding rate, starting strain
log seeding rate, Delta
transmissibility
shared target dispersion
no mobility
Google mobility
Facebook tiles visited mobility
Facebook single tile mobility

South Africa

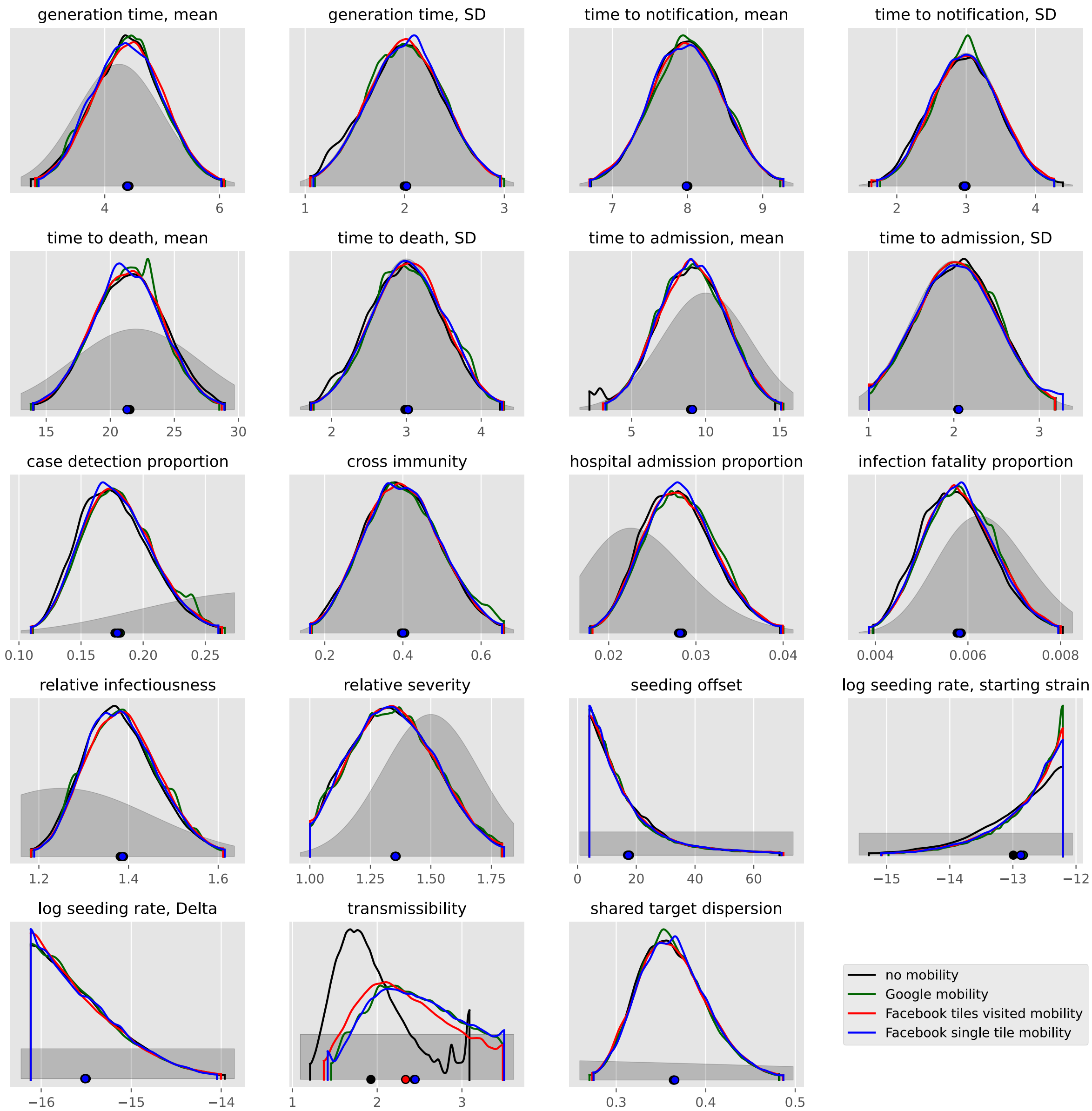

Togo

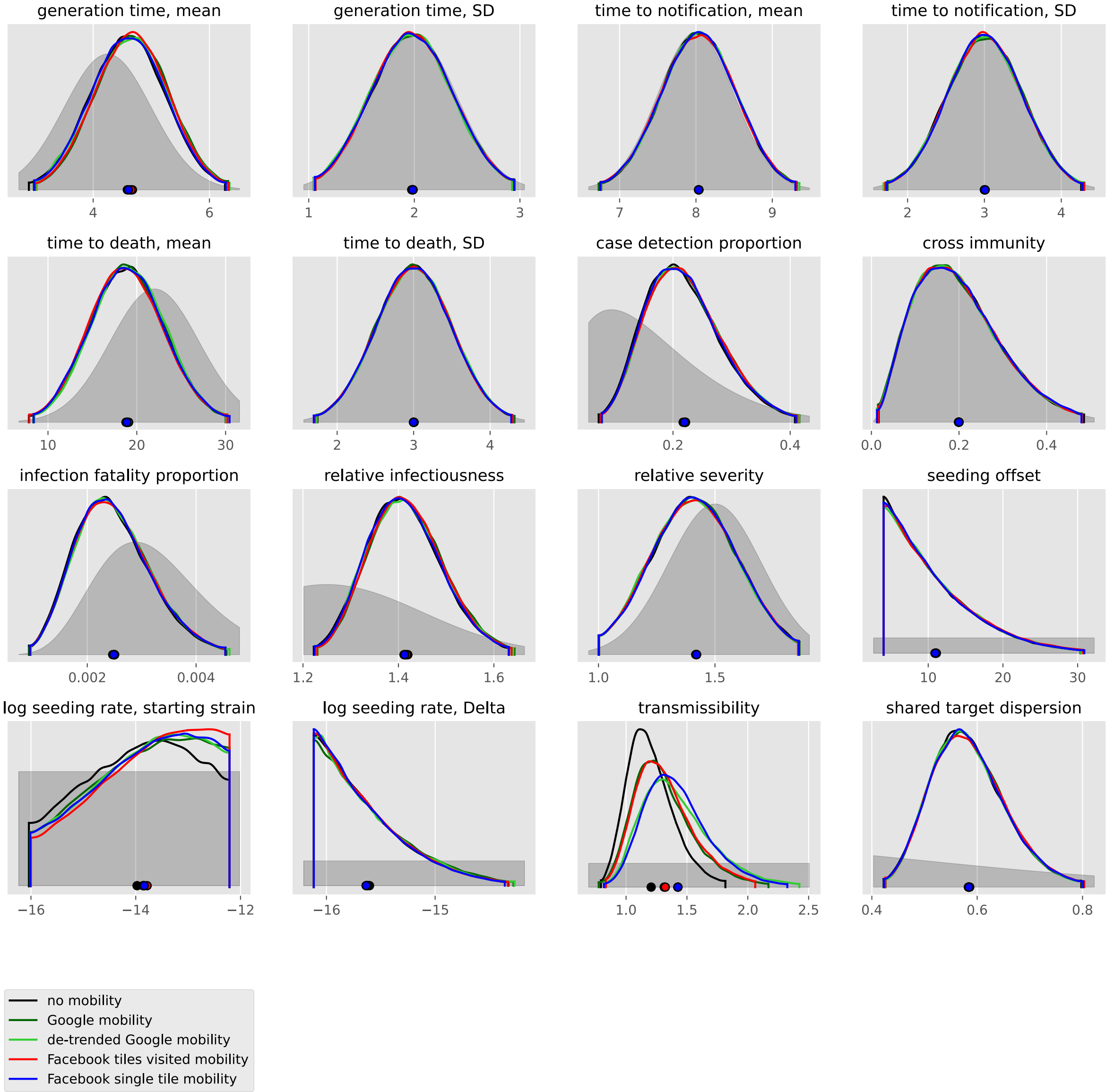
generation time, mean
4
6
generation time, SD
1
2
3
time to notification, mean
7
8
9
time to notification, SD
2
3
4
time to death, mean
10
20
30
time to death, SD
2
3
4
case detection proportion
0.2
0.4
cross immunity
0.0
0.2
0.4
infection fatality proportion
0.002
0.004
relative infectiousness
1.2
1.4
1.6
relative severity
1.0
1.5
seeding offset
10
20
30
log seeding rate, starting strain
−16
−14
−12
log seeding rate, Delta
−16
−15
transmissibility
1.0
1.5
2.0
2.5
shared target dispersion
0.4
0.6
0.8
no mobility
Google mobility
de-trended Google mobility
Facebook tiles visited mobility
Facebook single tile mobility

Tunisia

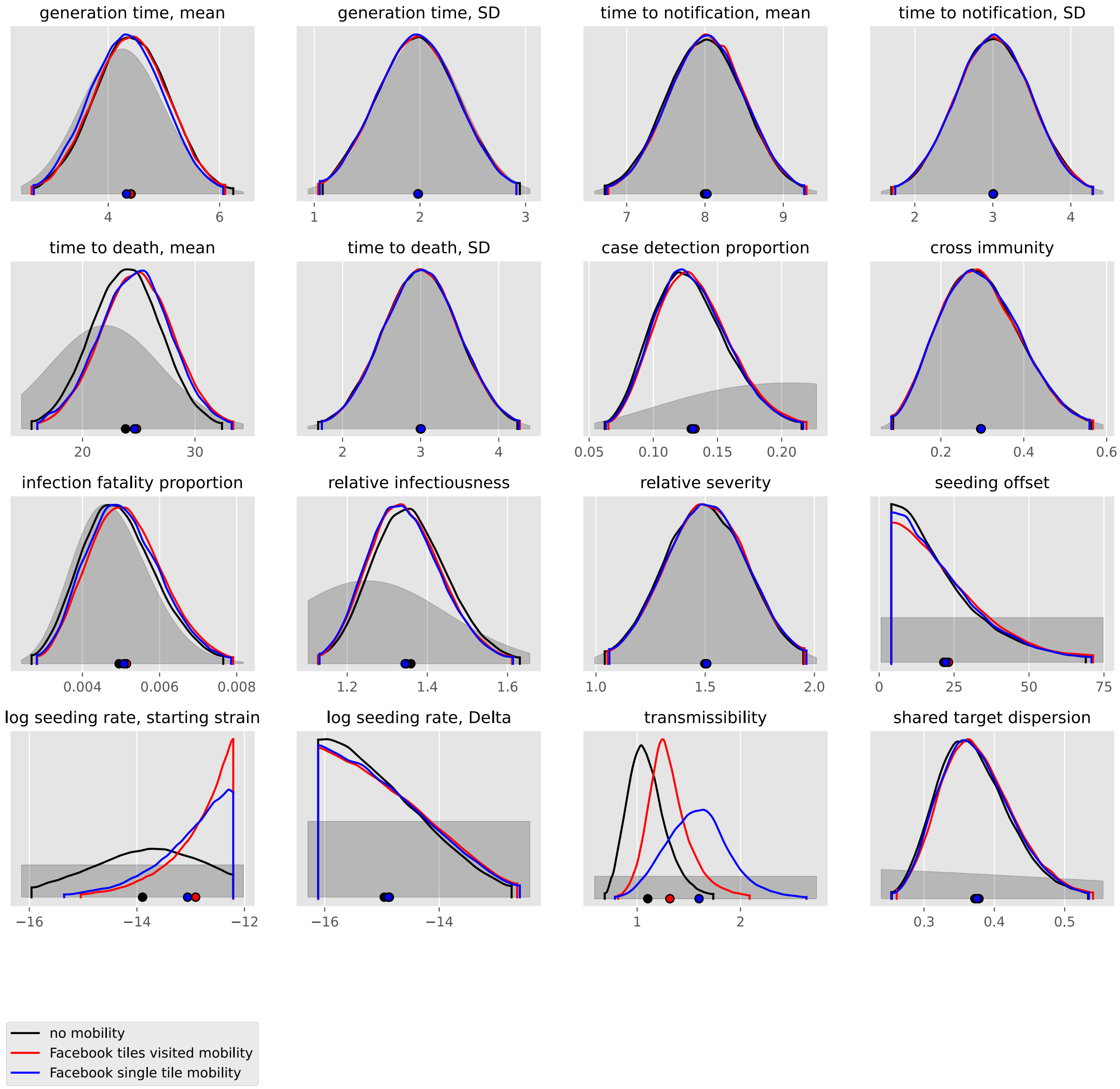
generation time, mean
4
6
generation time, SD
1
2
3
time to notification, mean
7
8
9
time to notification, SD
2
3
4
time to death, mean
20
30
time to death, SD
2
3
4
case detection proportion
0.05
0.10
0.15
0.20
cross immunity
0.2
0.4
0.6
infection fatality proportion
0.004
0.006
0.008
relative infectiousness
1.2
1.4
1.6
relative severity
1.0
1.5
2.0
seeding offset
0
25
50
75
log seeding rate, starting strain
−16
−14
−12
log seeding rate, Delta
−16
−14
transmissibility
1
2
shared target dispersion
0.3
0.4
0.5
no mobility
Facebook tiles visited mobility
Facebook single tile mobility

Zambia

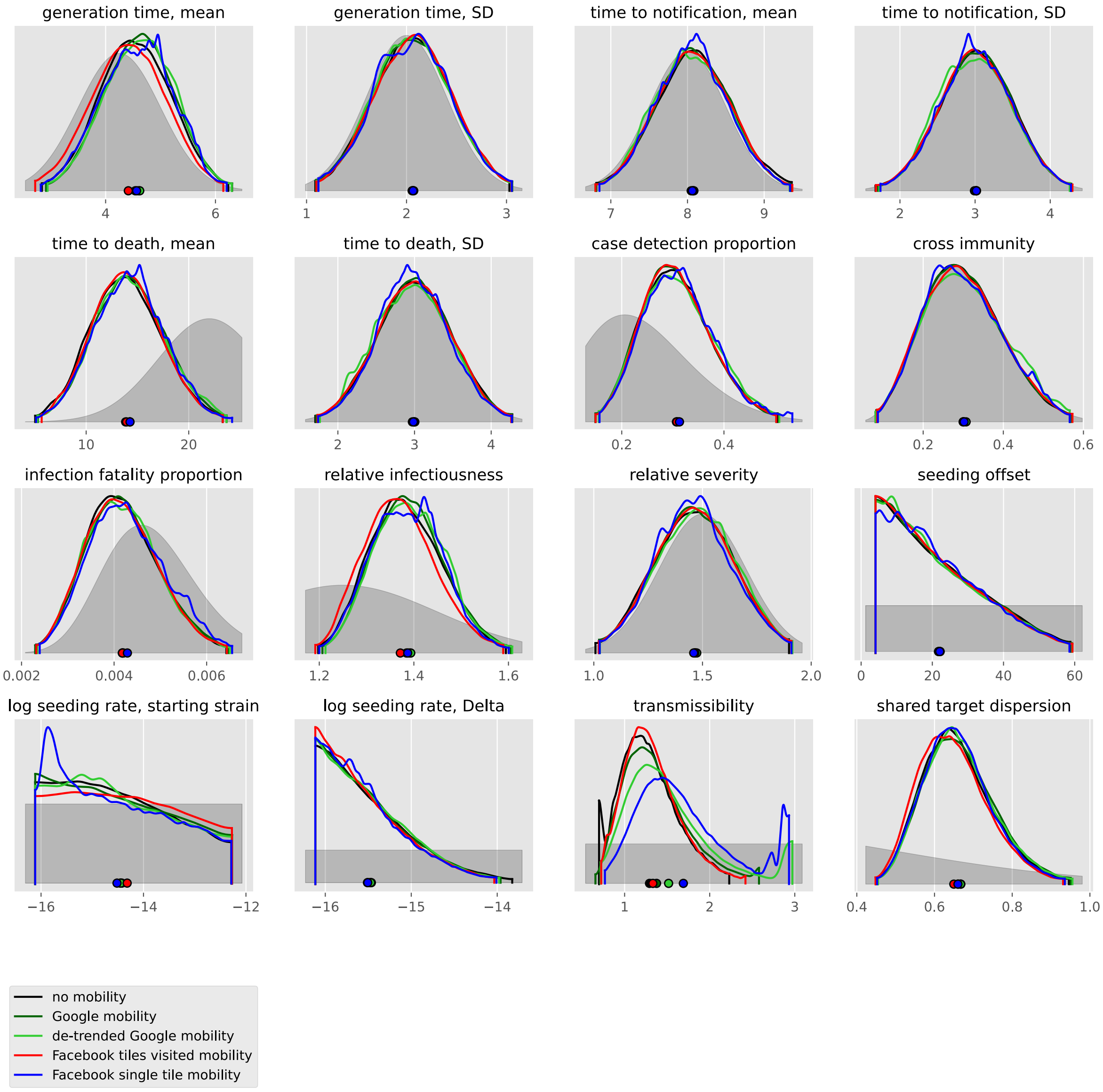

generation time, mean
generation time, SD
time to notification, mean
time to notification, SD
time to death, mean
time to death, SD
case detection proportion
cross immunity
infection fatality proportion
relative infectiousness
relative severity
seeding offset
log seeding rate, starting strain
log seeding rate, Delta
transmissibility
shared target dispersion
no mobility
Google mobility
de-trended Google mobility
Facebook tiles visited mobility
Facebook single tile mobility

Zimbabwe

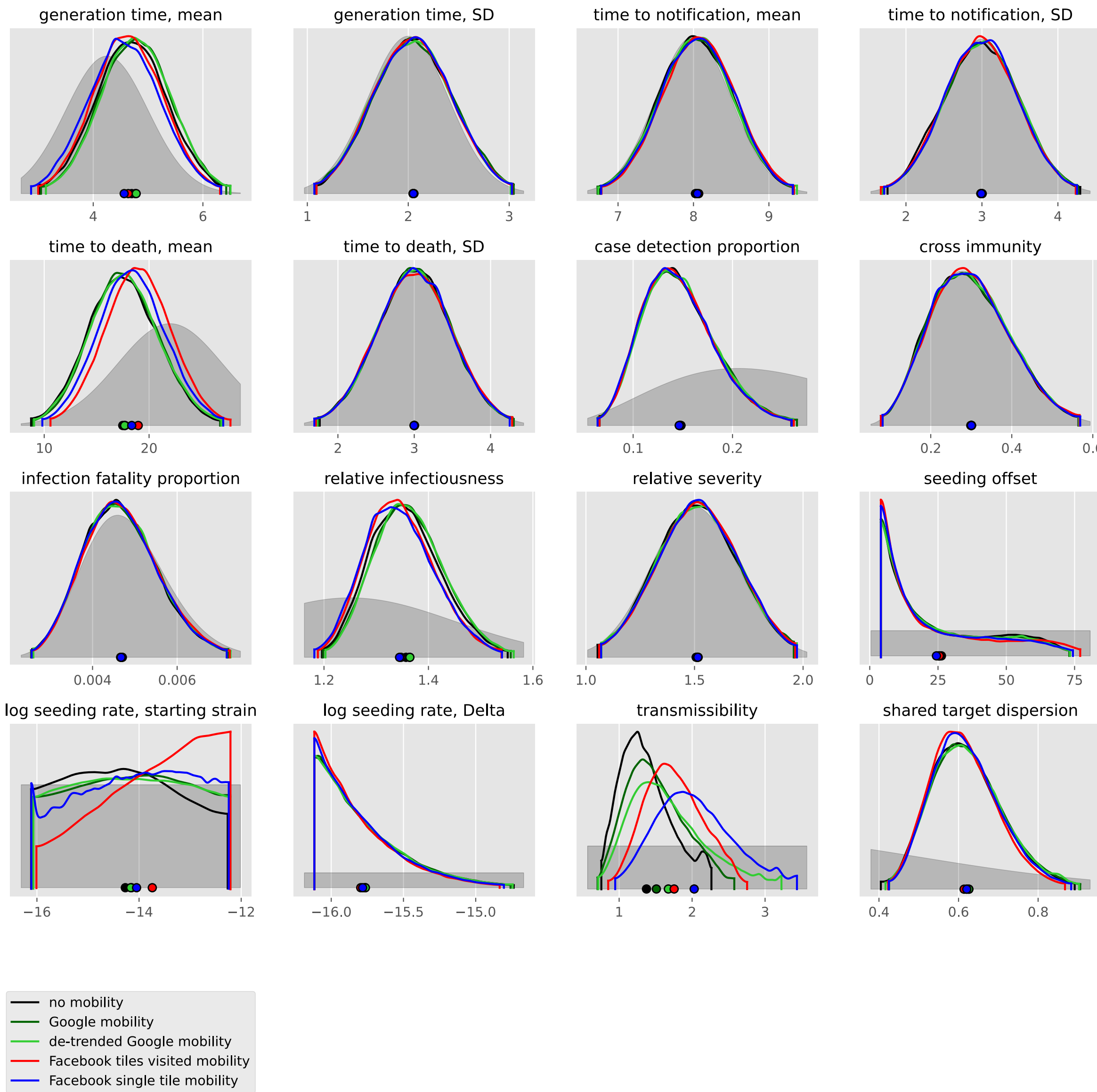

generation time, mean
4
6
generation time, SD
1
2
3
time to notification, mean
7
8
9
time to notification, SD
2
3
4
time to death, mean
10
20
time to death, SD
2
3
4
case detection proportion
0.1
0.2
cross immunity
0.2
0.4
0.6
infection fatality proportion
0.004
0.006
relative infectiousness
1.2
1.4
1.6
relative severity
1.0
1.5
2.0
seeding offset
0
25
50
75
log seeding rate, starting strain
−16
−14
−12
log seeding rate, Delta
−16.0
−15.5
−15.0
transmissibility
1
2
3
shared target dispersion
0.4
0.6
0.8
no mobility
Google mobility
de-trended Google mobility
Facebook tiles visited mobility
Facebook single tile mobility

Asia

Afghanistan

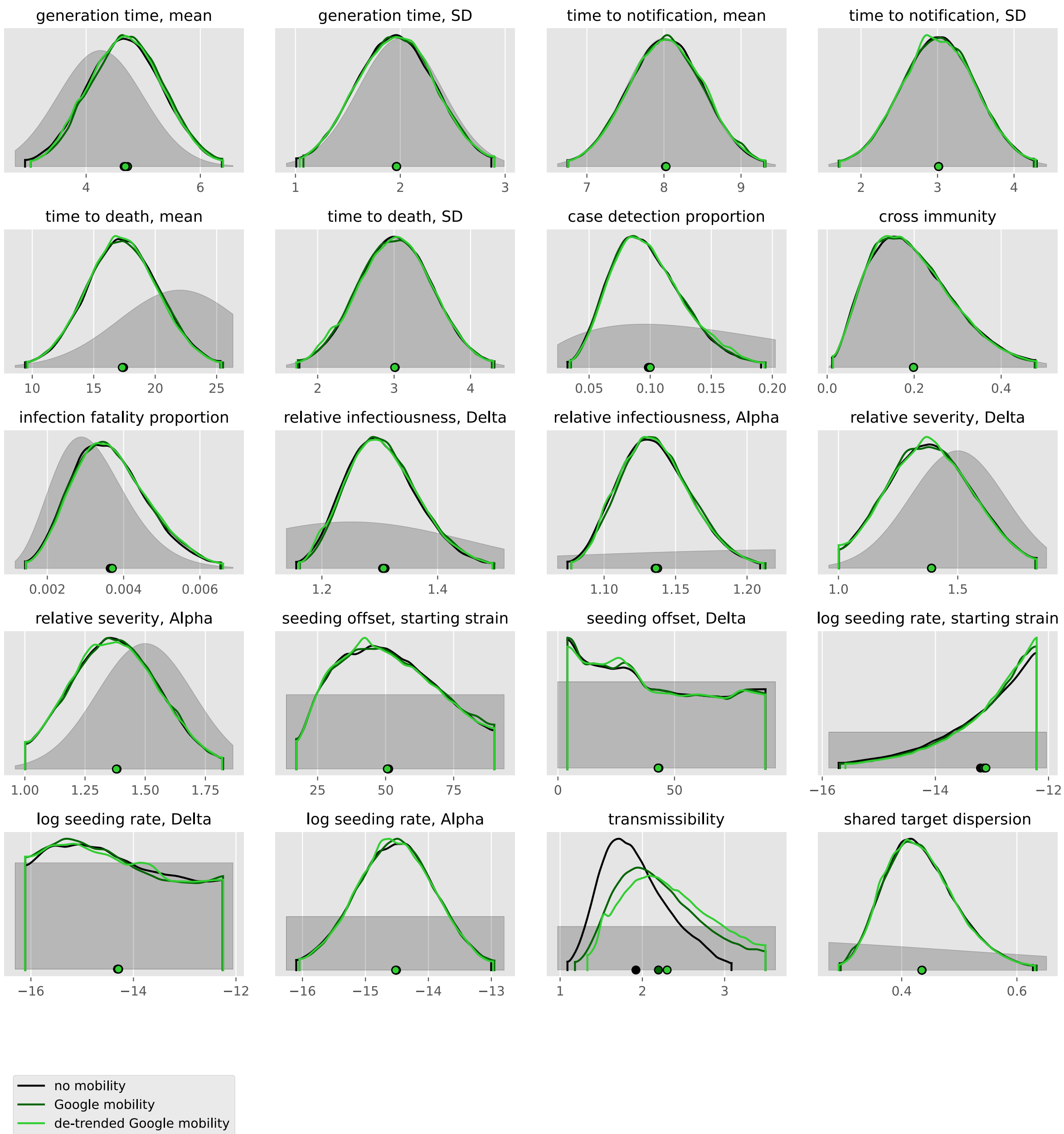

Bahrain

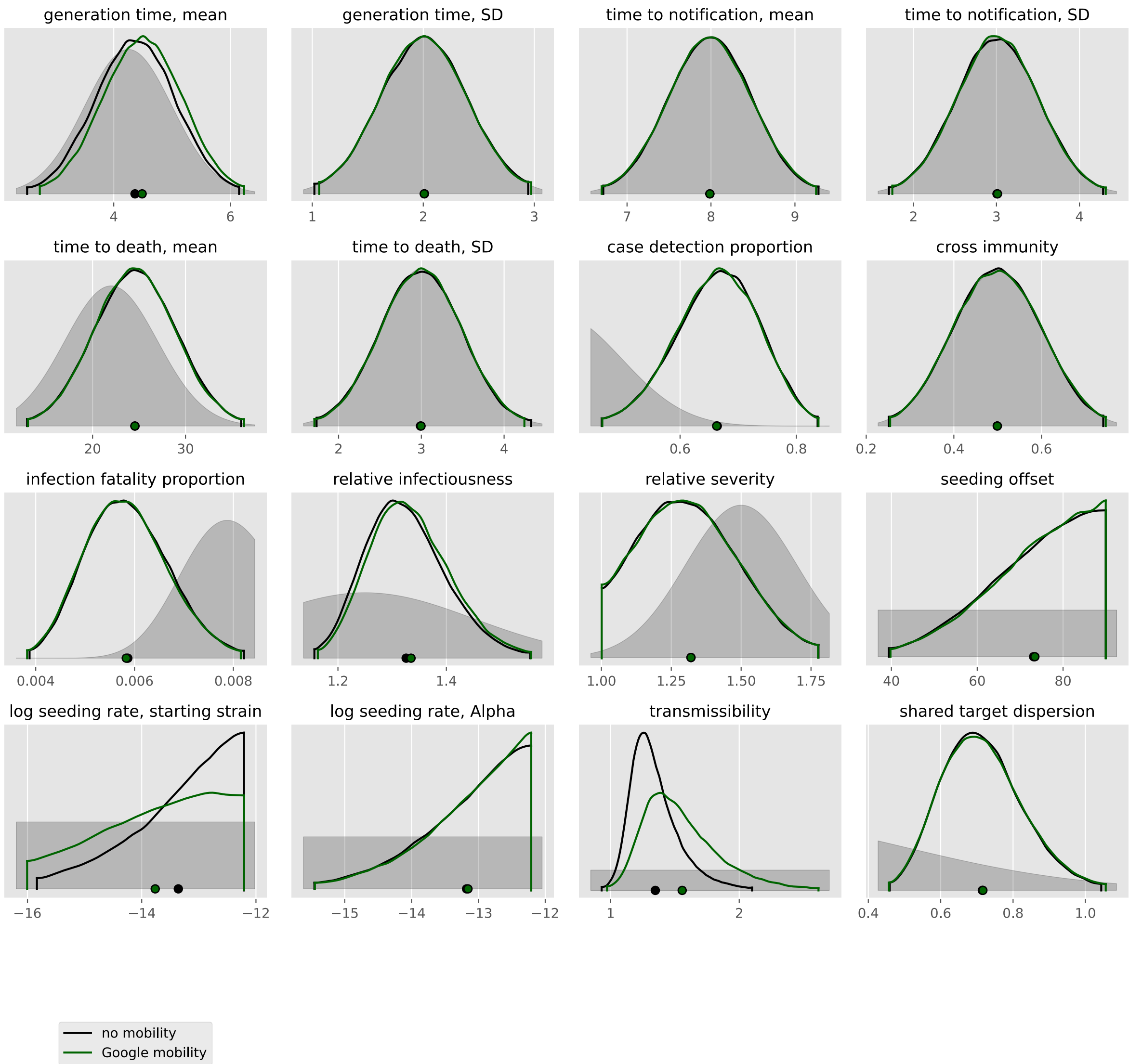
generation time, mean
4
6
generation time, SD
1
2
3
time to notification, mean
7
8
9
time to notification, SD
2
3
4
time to death, mean
20
30
time to death, SD
2
3
4
case detection proportion
0.6
0.8
cross immunity
0.2
0.4
0.6
infection fatality proportion
0.004
0.006
0.008
relative infectiousness
1.2
1.4
relative severity
1.00
1.25
1.50
1.75
seeding offset
40
60
80
log seeding rate, starting strain
−16
−14
−12
log seeding rate, Alpha
−15
−14
−13
−12
transmissibility
1
2
shared target dispersion
0.4
0.6
0.8
1.0
no mobility
Google mobility

Bangladesh

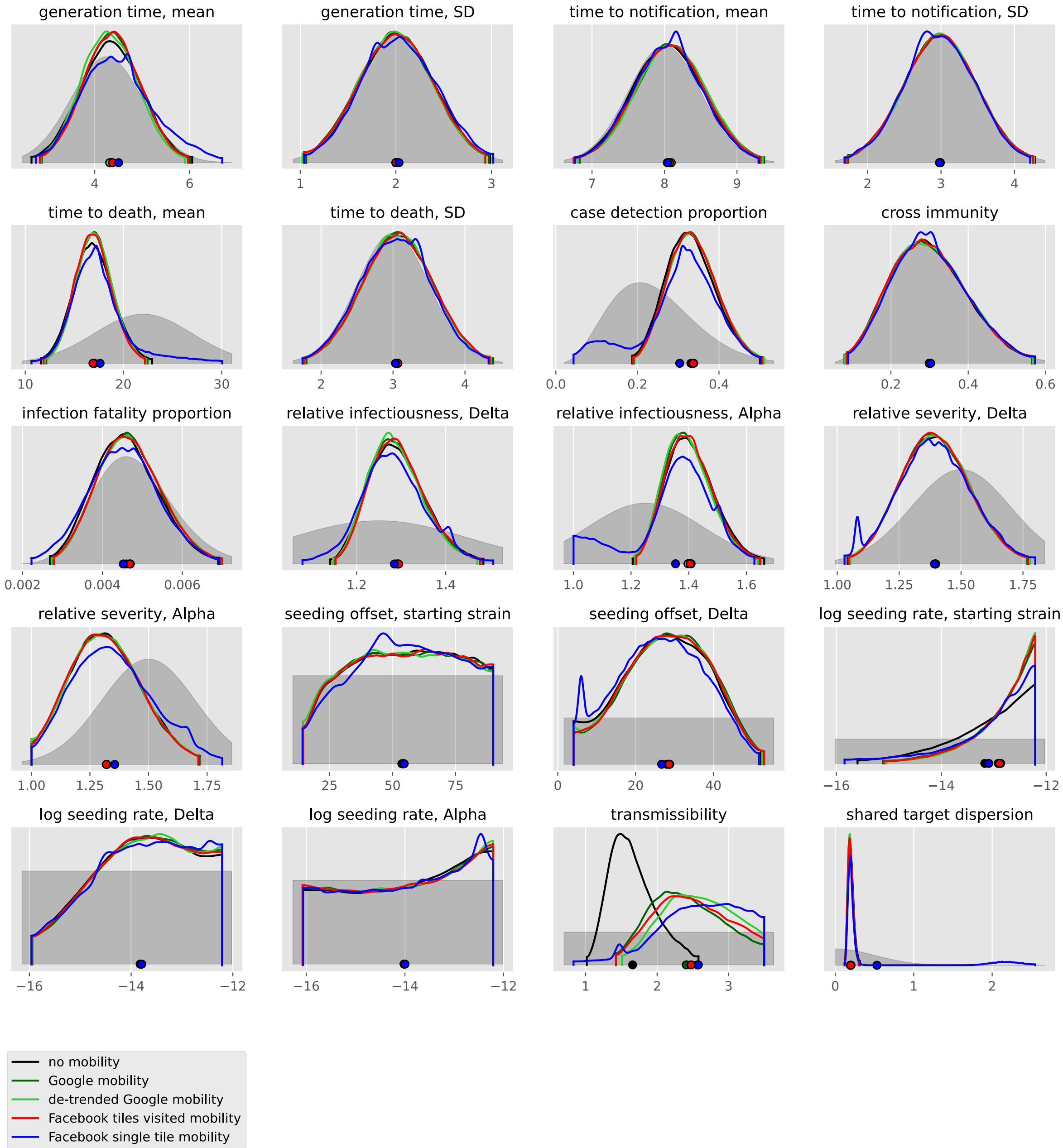

generation time, mean
generation time, SD
time to notification, mean
time to notification, SD
time to death, mean
time to death, SD
case detection proportion
cross immunity
infection fatality proportion
relative infectiousness, Delta
relative infectiousness, Alpha
relative severity, Delta
relative severity, Alpha
seeding offset, starting strain
seeding offset, Delta
log seeding rate, starting strain
log seeding rate, Delta
log seeding rate, Alpha
transmissibility
shared target dispersion
no mobility
Google mobility
de-trended Google mobility
Facebook tiles visited mobility
Facebook single tile mobility

Georgia

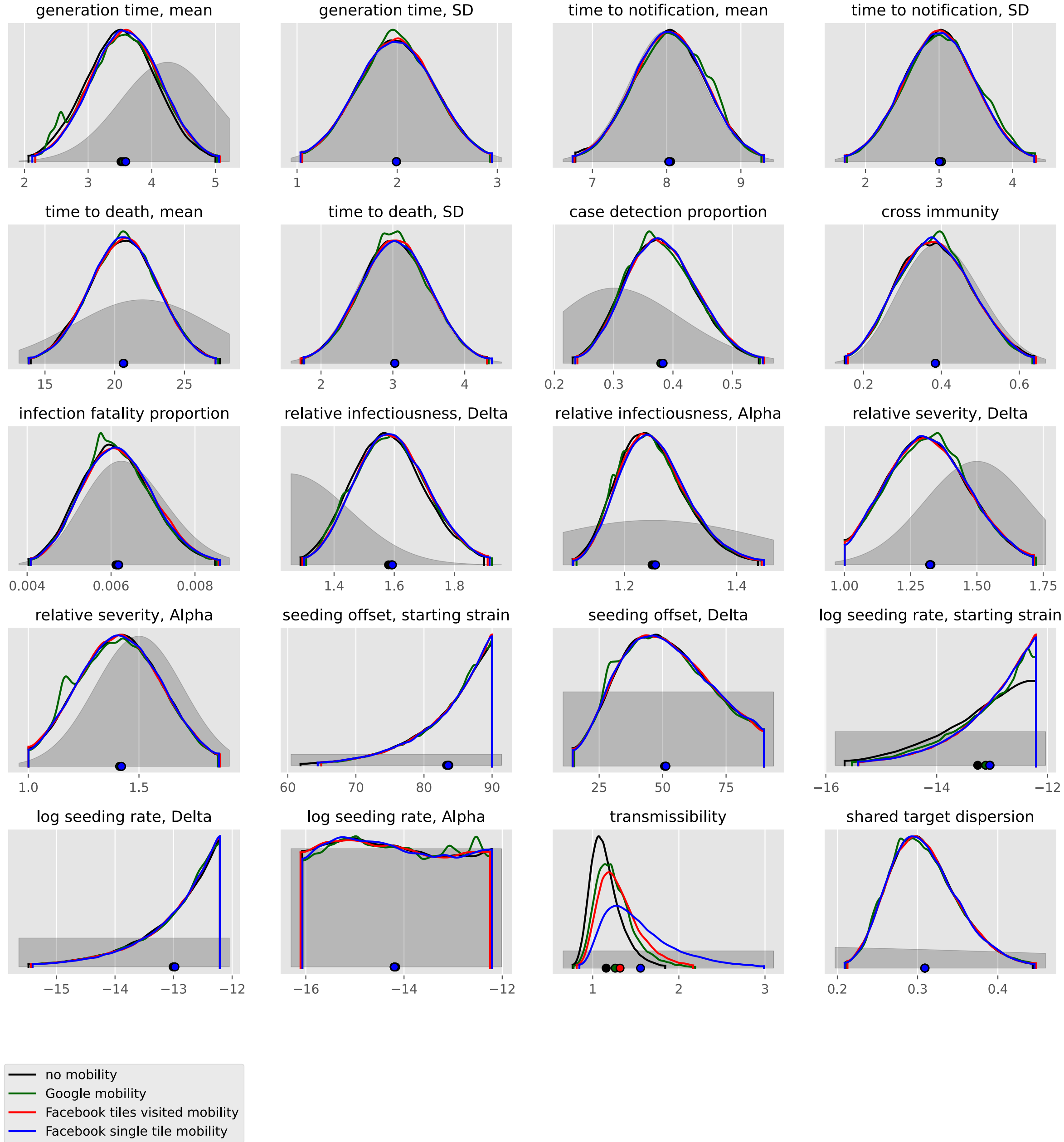

generation time, mean
generation time, SD
time to notification, mean
time to notification, SD
time to death, mean
time to death, SD
case detection proportion
cross immunity
infection fatality proportion
relative infectiousness, Delta
relative infectiousness, Alpha
relative severity, Delta
relative severity, Alpha
seeding offset, starting strain
seeding offset, Delta
log seeding rate, starting strain
log seeding rate, Delta
log seeding rate, Alpha
transmissibility
shared target dispersion
no mobility
Google mobility
Facebook tiles visited mobility
Facebook single tile mobility

India

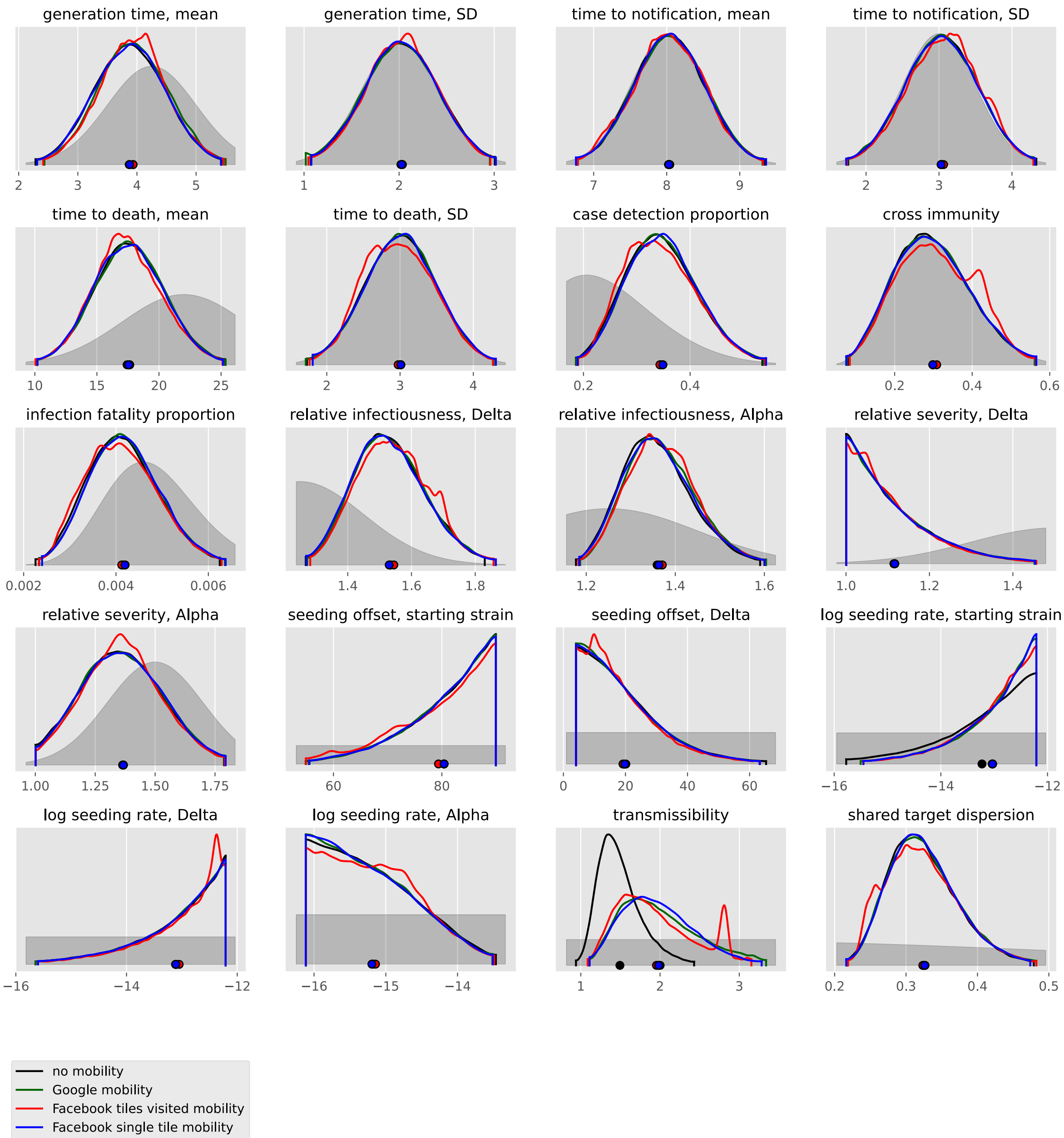
generation time, mean
generation time, SD
time to notification, mean
time to notification, SD
time to death, mean
time to death, SD
case detection proportion
cross immunity
infection fatality proportion
relative infectiousness, Delta
relative infectiousness, Alpha
relative severity, Delta
relative severity, Alpha
seeding offset, starting strain
seeding offset, Delta
log seeding rate, starting strain
log seeding rate, Delta
log seeding rate, Alpha
transmissibility
shared target dispersion
no mobility
Google mobility
Facebook tiles visited mobility
Facebook single tile mobility

Indonesia

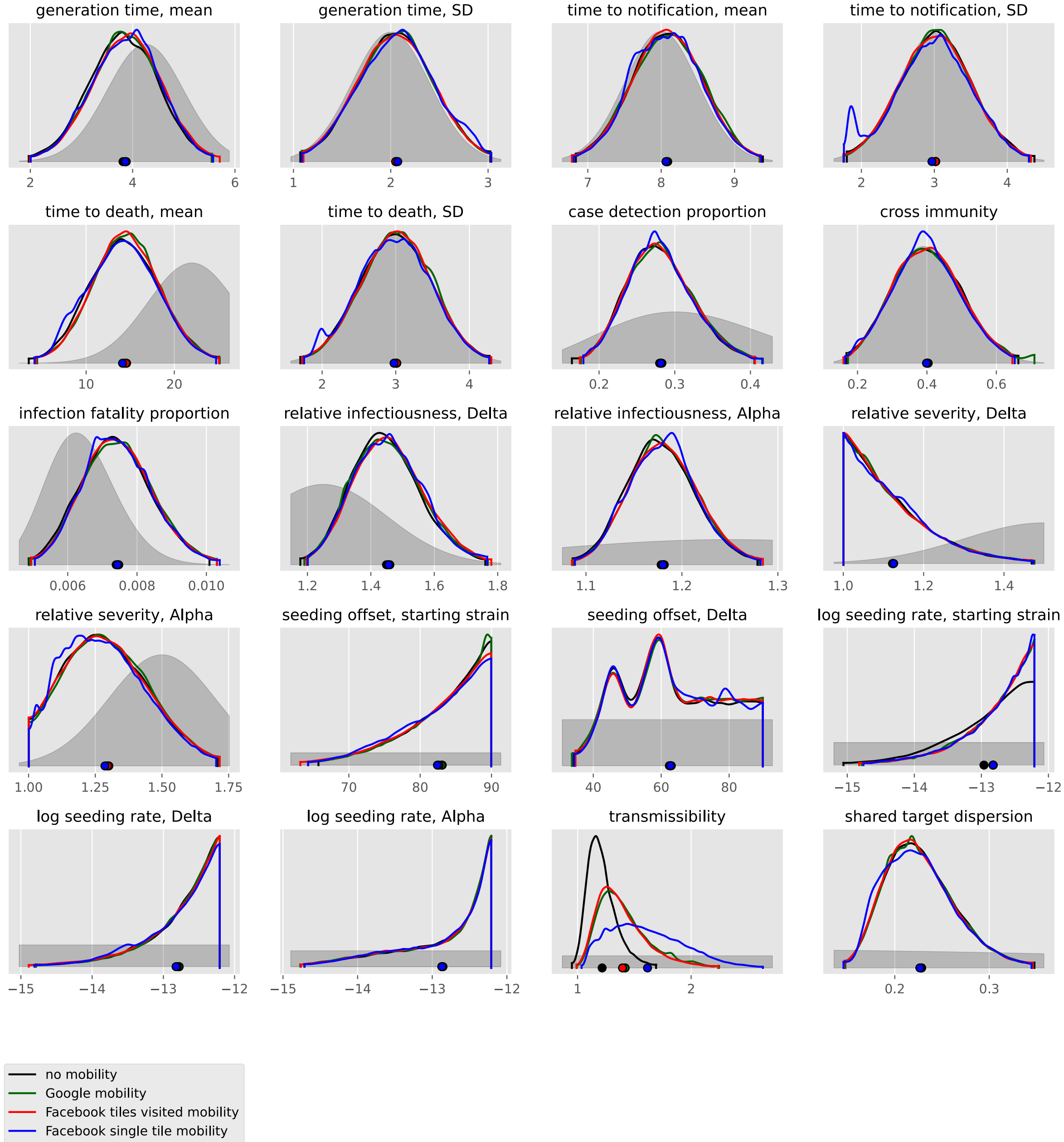

generation time, mean
generation time, SD
time to notification, mean
time to notification, SD
time to death, mean
time to death, SD
case detection proportion
cross immunity
infection fatality proportion
relative infectiousness, Delta
relative infectiousness, Alpha
relative severity, Delta
relative severity, Alpha
seeding offset, starting strain
seeding offset, Delta
log seeding rate, starting strain
log seeding rate, Delta
log seeding rate, Alpha
transmissibility
shared target dispersion
no mobility
Google mobility
Facebook tiles visited mobility
Facebook single tile mobility

Iraq

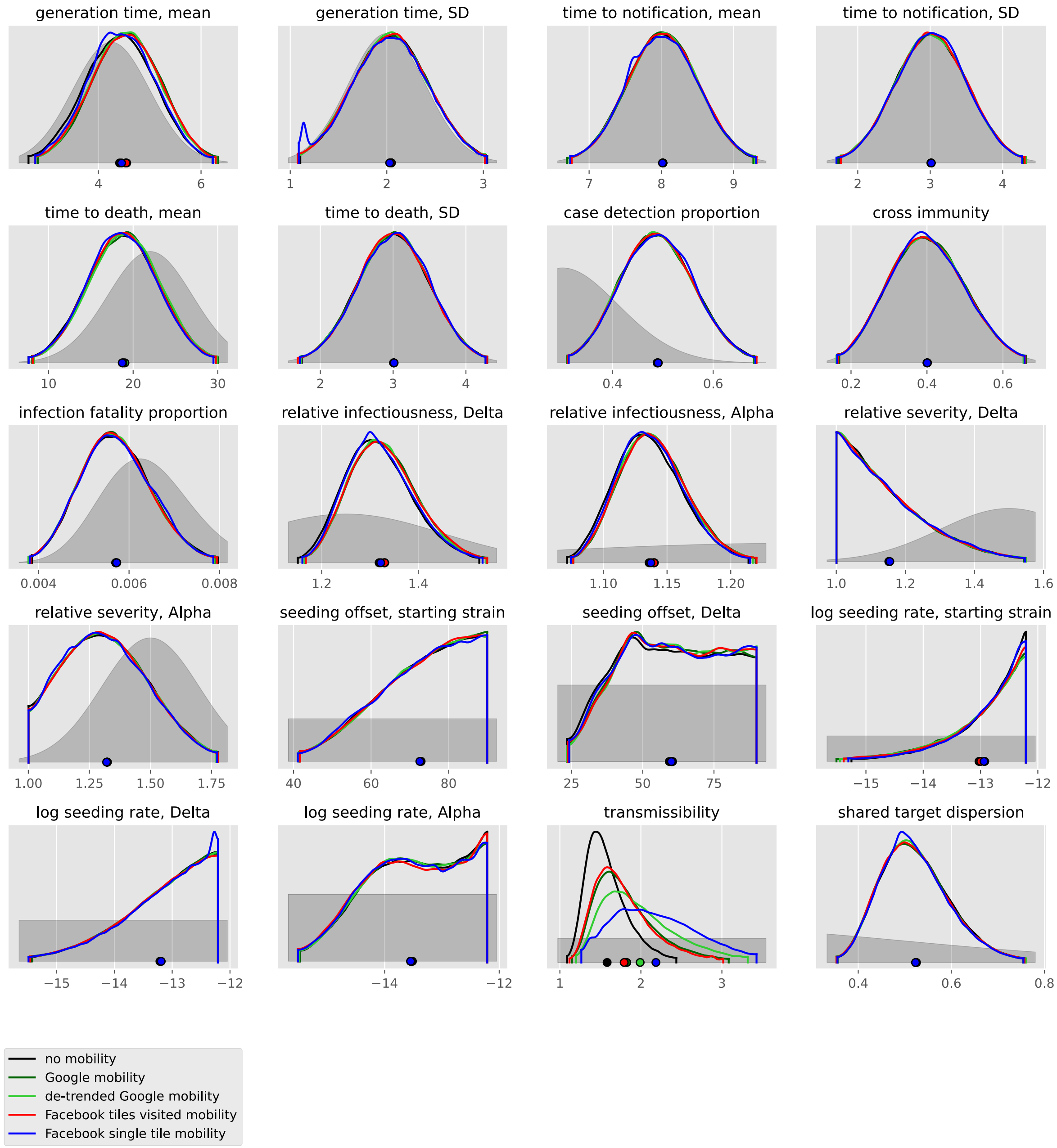
generation time, mean
generation time, SD
time to notification, mean
time to notification, SD
time to death, mean
time to death, SD
case detection proportion
cross immunity
infection fatality proportion
relative infectiousness, Delta
relative infectiousness, Alpha
relative severity, Delta
relative severity, Alpha
seeding offset, starting strain
seeding offset, Delta
log seeding rate, starting strain
log seeding rate, Delta
log seeding rate, Alpha
transmissibility
shared target dispersion
no mobility
Google mobility
de-trended Google mobility
Facebook tiles visited mobility
Facebook single tile mobility

Israel

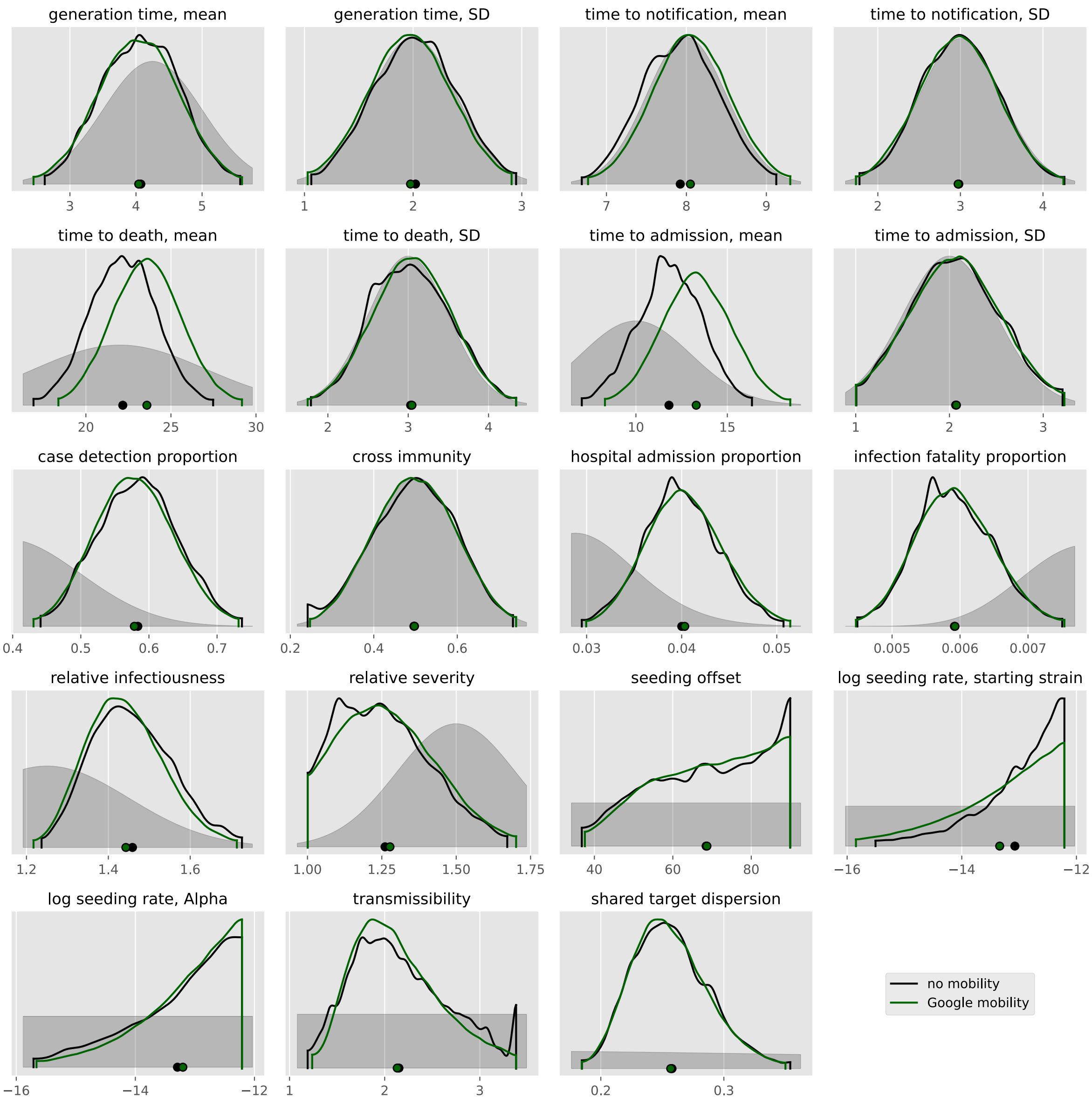

generation time, mean
generation time, SD
time to notification, mean
time to notification, SD
time to death, mean
time to death, SD
time to admission, mean
time to admission, SD
case detection proportion
cross immunity
hospital admission proportion
infection fatality proportion
relative infectiousness
relative severity
seeding offset
log seeding rate, starting strain
log seeding rate, Alpha
transmissibility
shared target dispersion
no mobility
Google mobility

Japan

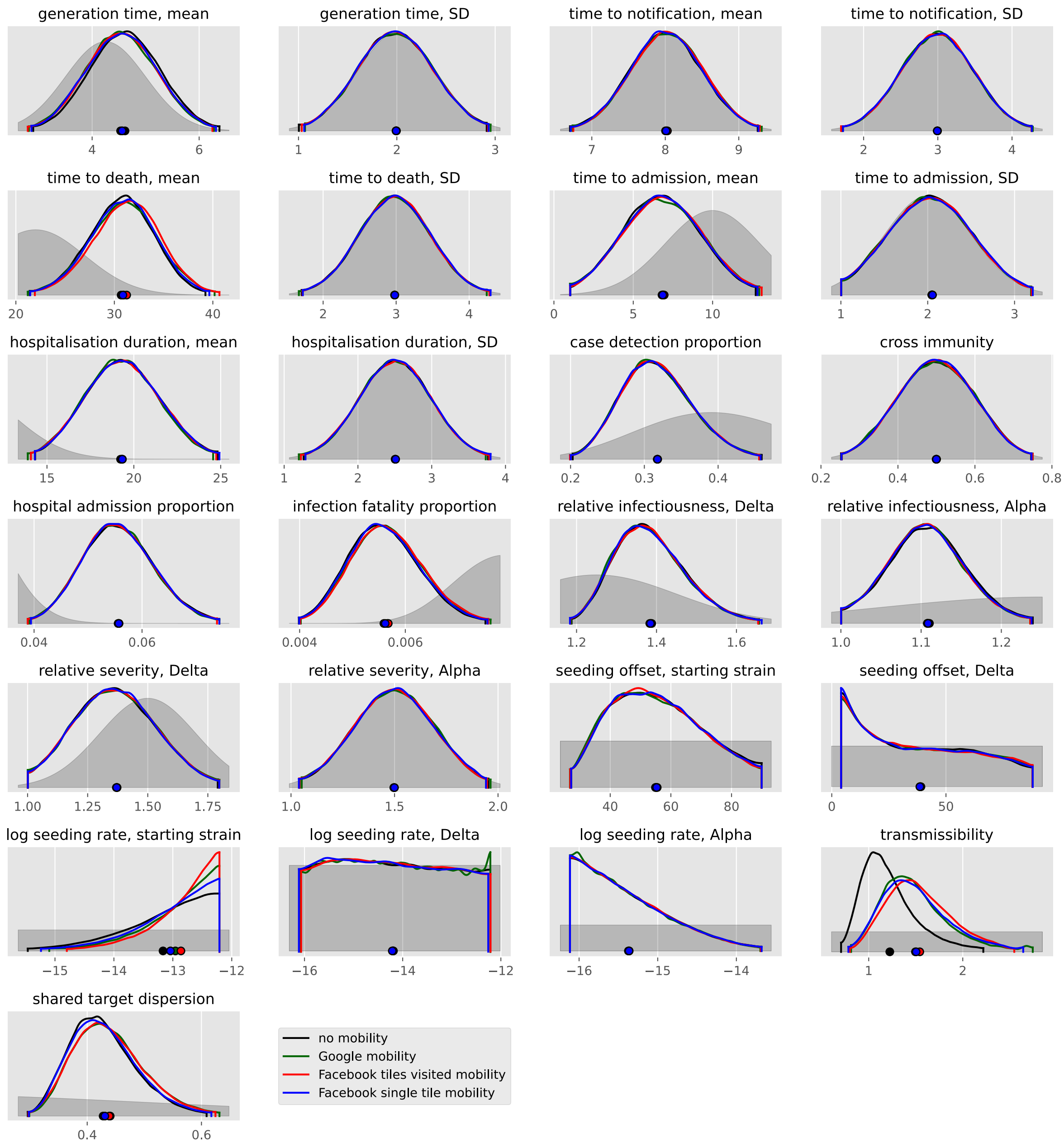
generation time, mean
generation time, SD
time to notification, mean
time to notification, SD
time to death, mean
time to death, SD
time to admission, mean
time to admission, SD
hospitalisation duration, mean
hospitalisation duration, SD
case detection proportion
cross immunity
hospital admission proportion
infection fatality proportion
relative infectiousness, Delta
relative infectiousness, Alpha
relative severity, Delta
relative severity, Alpha
seeding offset, starting strain
seeding offset, Delta
log seeding rate, starting strain
log seeding rate, Delta
log seeding rate, Alpha
transmissibility
shared target dispersion
no mobility
Google mobility
Facebook tiles visited mobility
Facebook single tile mobility

Jordan

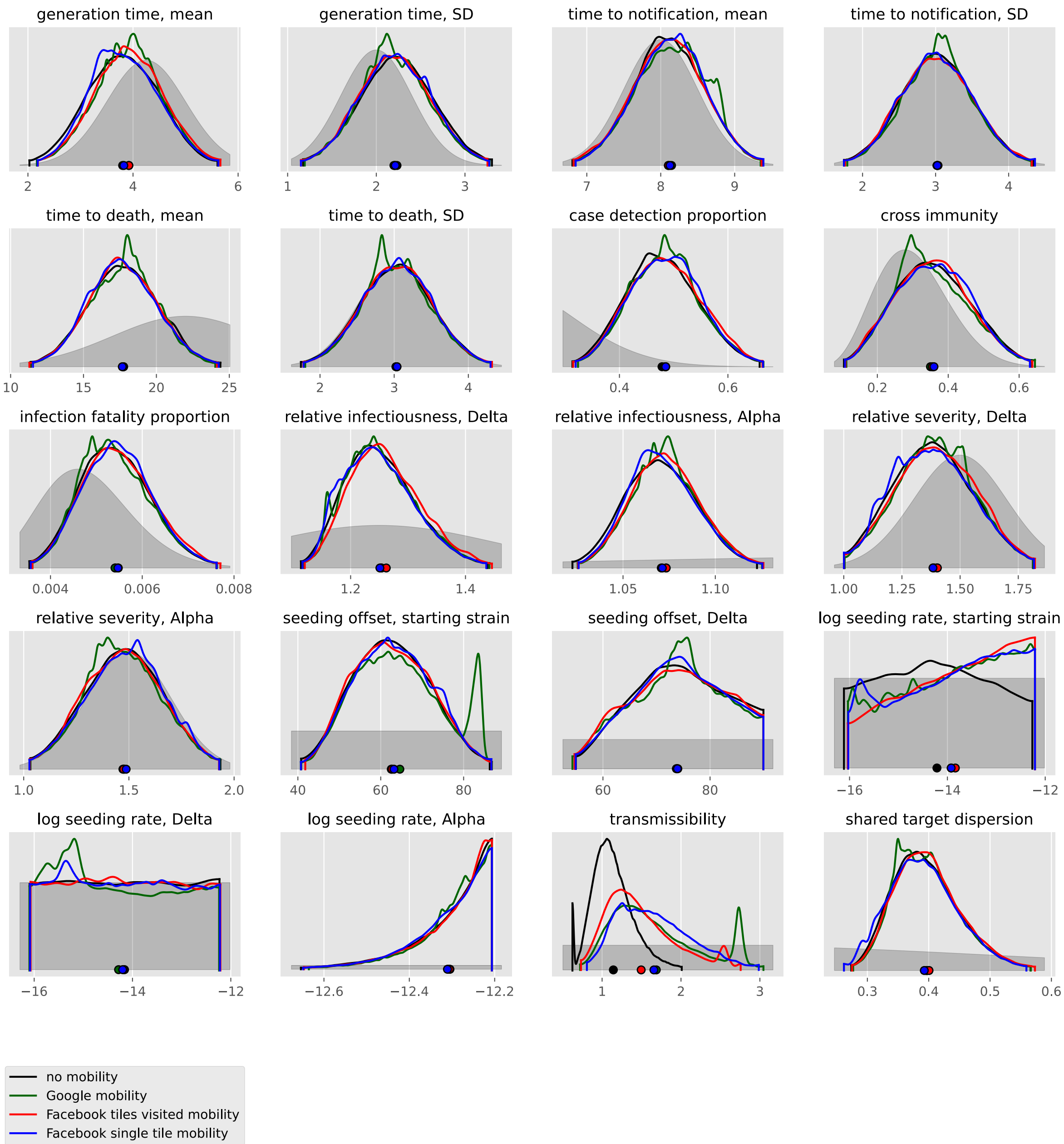

generation time, mean
generation time, SD
time to notification, mean
time to notification, SD
time to death, mean
time to death, SD
case detection proportion
cross immunity
infection fatality proportion
relative infectiousness, Delta
relative infectiousness, Alpha
relative severity, Delta
relative severity, Alpha
seeding offset, starting strain
seeding offset, Delta
log seeding rate, starting strain
log seeding rate, Delta
log seeding rate, Alpha
transmissibility
shared target dispersion
no mobility
Google mobility
Facebook tiles visited mobility
Facebook single tile mobility

Korea, Republic of

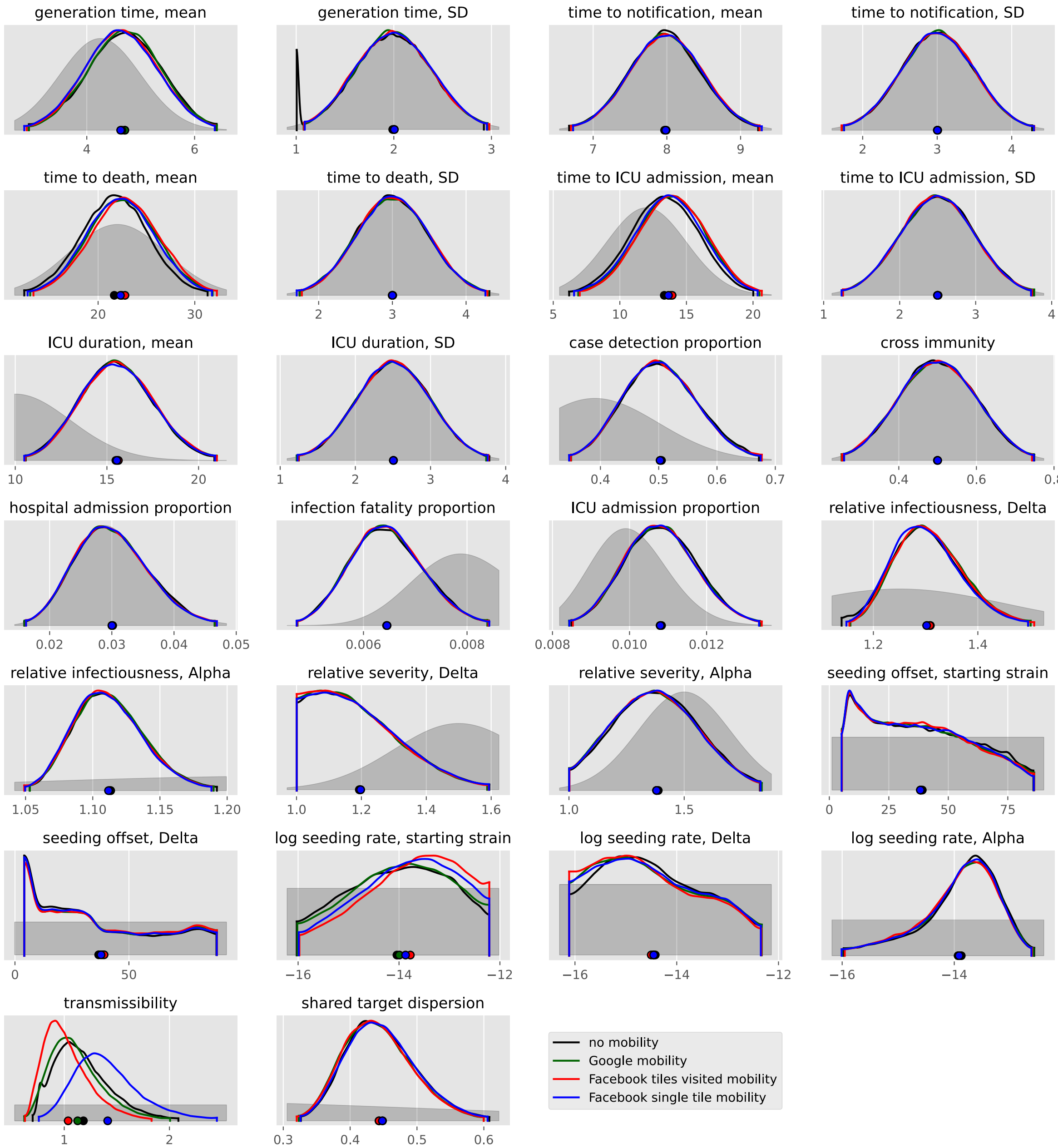

Kuwait

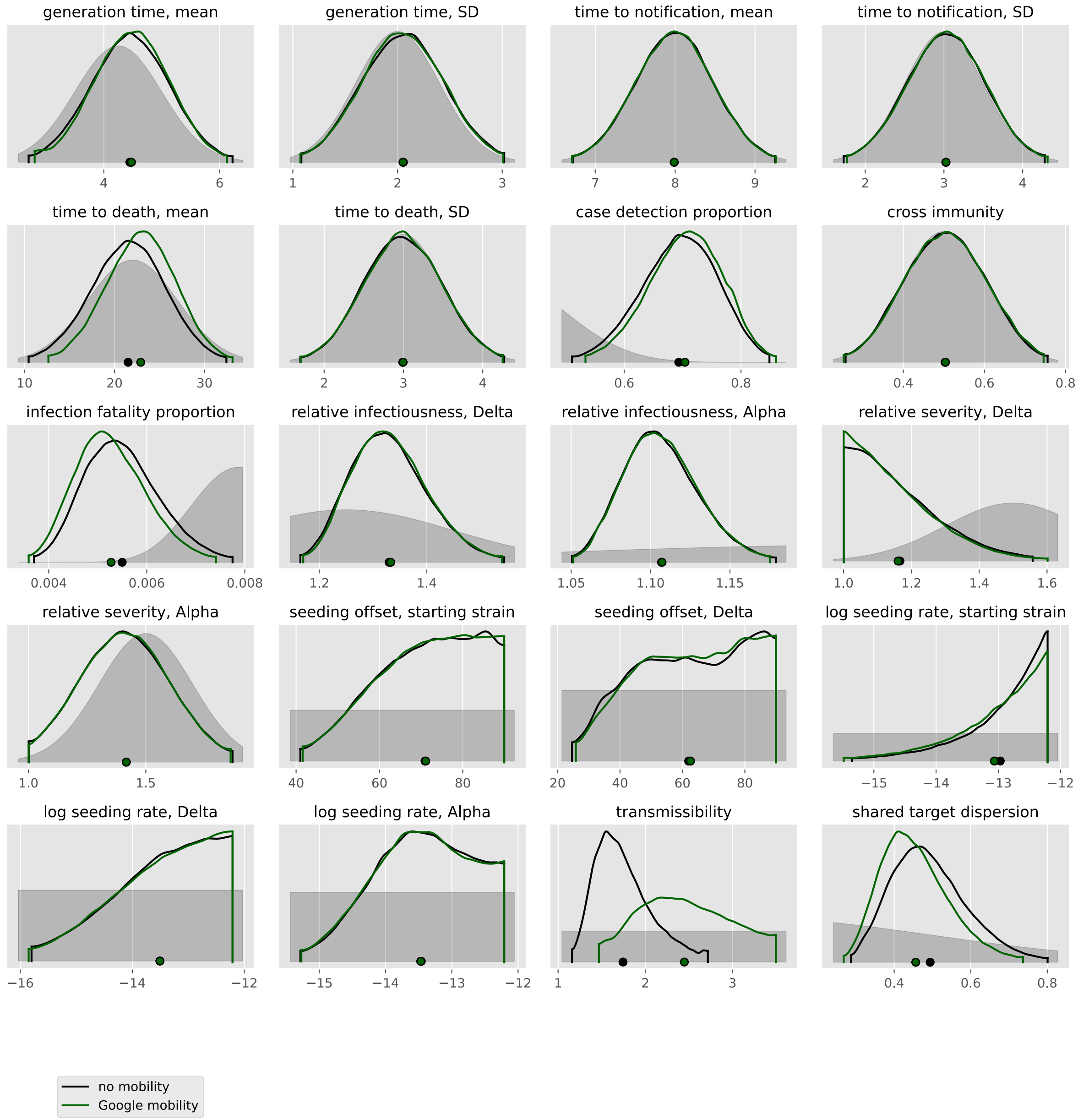

generation time, mean
4
6
generation time, SD
1
2
3
time to notification, mean
7
8
9
time to notification, SD
2
3
4
time to death, mean
10
20
30
time to death, SD
2
3
4
case detection proportion
0.6
0.8
cross immunity
0.4
0.6
0.8
infection fatality proportion
0.004
0.006
0.008
relative infectiousness, Delta
1.2
1.4
relative infectiousness, Alpha
1.05
1.10
1.15
relative severity, Delta
1.0
1.2
1.4
1.6
relative severity, Alpha
1.0
1.5
seeding offset, starting strain
40
60
80
seeding offset, Delta
20
40
60
80
log seeding rate, starting strain
−15
−14
−13
−12
log seeding rate, Delta
−16
−14
−12
log seeding rate, Alpha
−15
−14
−13
−12
transmissibility
1
2
3
shared target dispersion
0.4
0.6
0.8
no mobility
Google mobility

Lebanon

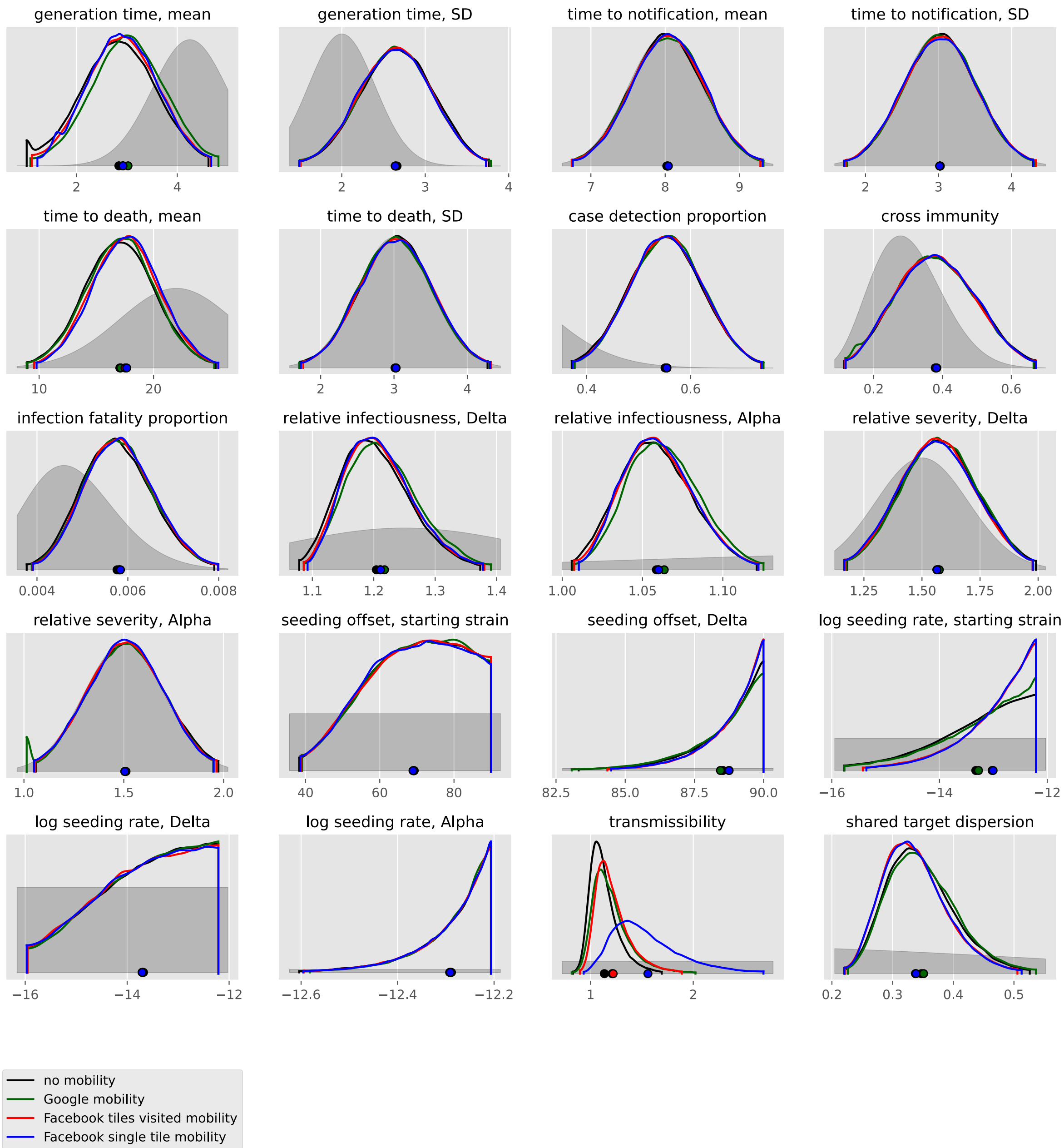

generation time, mean
generation time, SD
time to notification, mean
time to notification, SD
time to death, mean
time to death, SD
case detection proportion
cross immunity
infection fatality proportion
relative infectiousness, Delta
relative infectiousness, Alpha
relative severity, Delta
relative severity, Alpha
seeding offset, starting strain
seeding offset, Delta
log seeding rate, starting strain
log seeding rate, Delta
log seeding rate, Alpha
transmissibility
shared target dispersion
no mobility
Google mobility
Facebook tiles visited mobility
Facebook single tile mobility

Malaysia

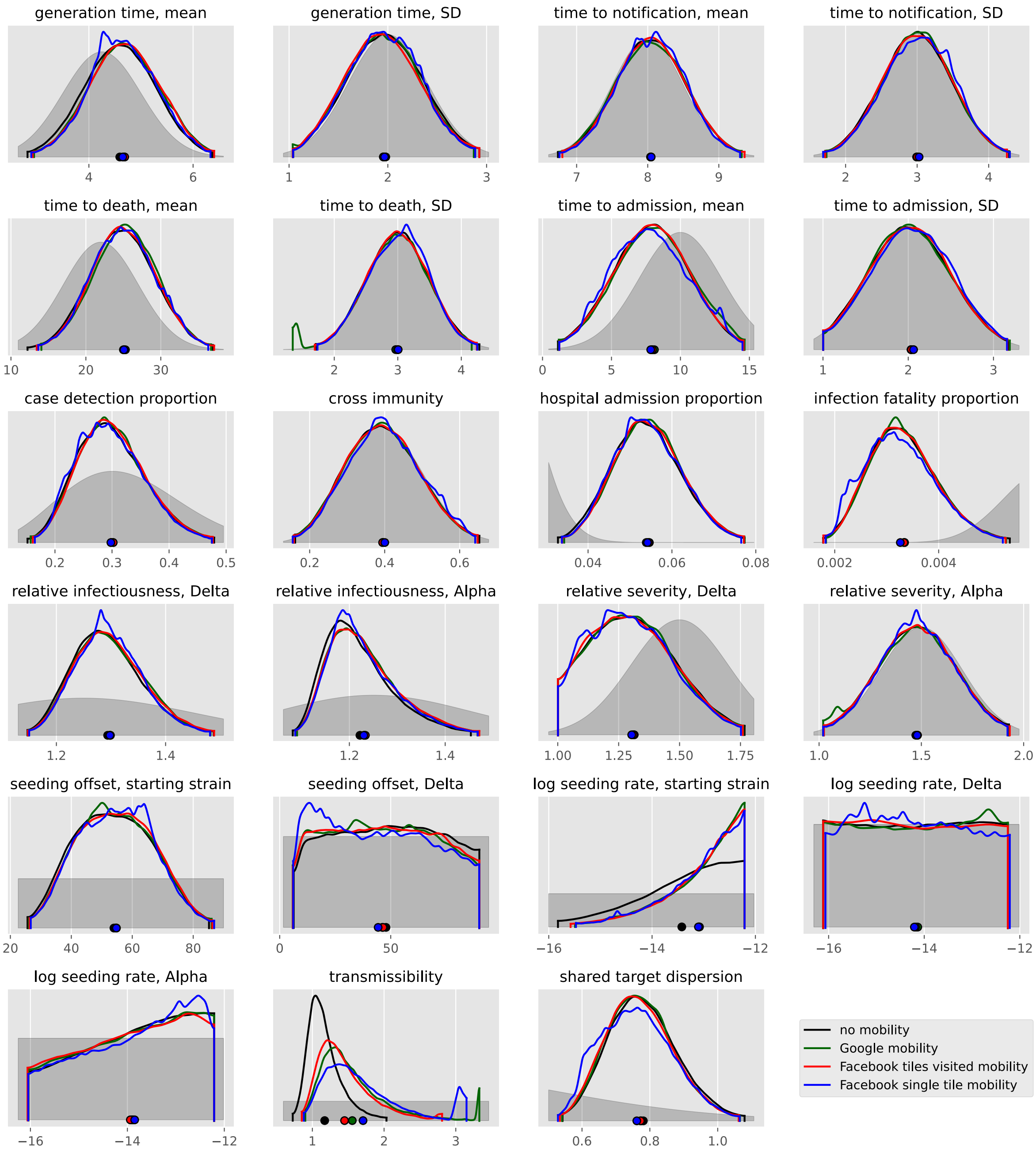

generation time, mean
4
6
generation time, SD
1
2
3
time to notification, mean
7
8
9
time to notification, SD
2
3
4
time to death, mean
10
20
30
time to death, SD
2
3
4
time to admission, mean
0
5
10
15
time to admission, SD
1
2
3
case detection proportion
0.2
0.3
0.4
0.5
cross immunity
0.2
0.4
0.6
hospital admission proportion
0.04
0.06
0.08
infection fatality proportion
0.002
0.004
relative infectiousness, Delta
1.2
1.4
relative infectiousness, Alpha
1.2
1.4
relative severity, Delta
1.00
1.25
1.50
1.75
relative severity, Alpha
1.0
1.5
2.0
seeding offset, starting strain
20
40
60
80
seeding offset, Delta
0
50
log seeding rate, starting strain
−16
−14
−12
log seeding rate, Delta
−16
−14
−12
log seeding rate, Alpha
−16
−14
−12
transmissibility
1
2
3
shared target dispersion
0.6
0.8
1.0
no mobility
Google mobility
Facebook tiles visited mobility
Facebook single tile mobility

Nepal

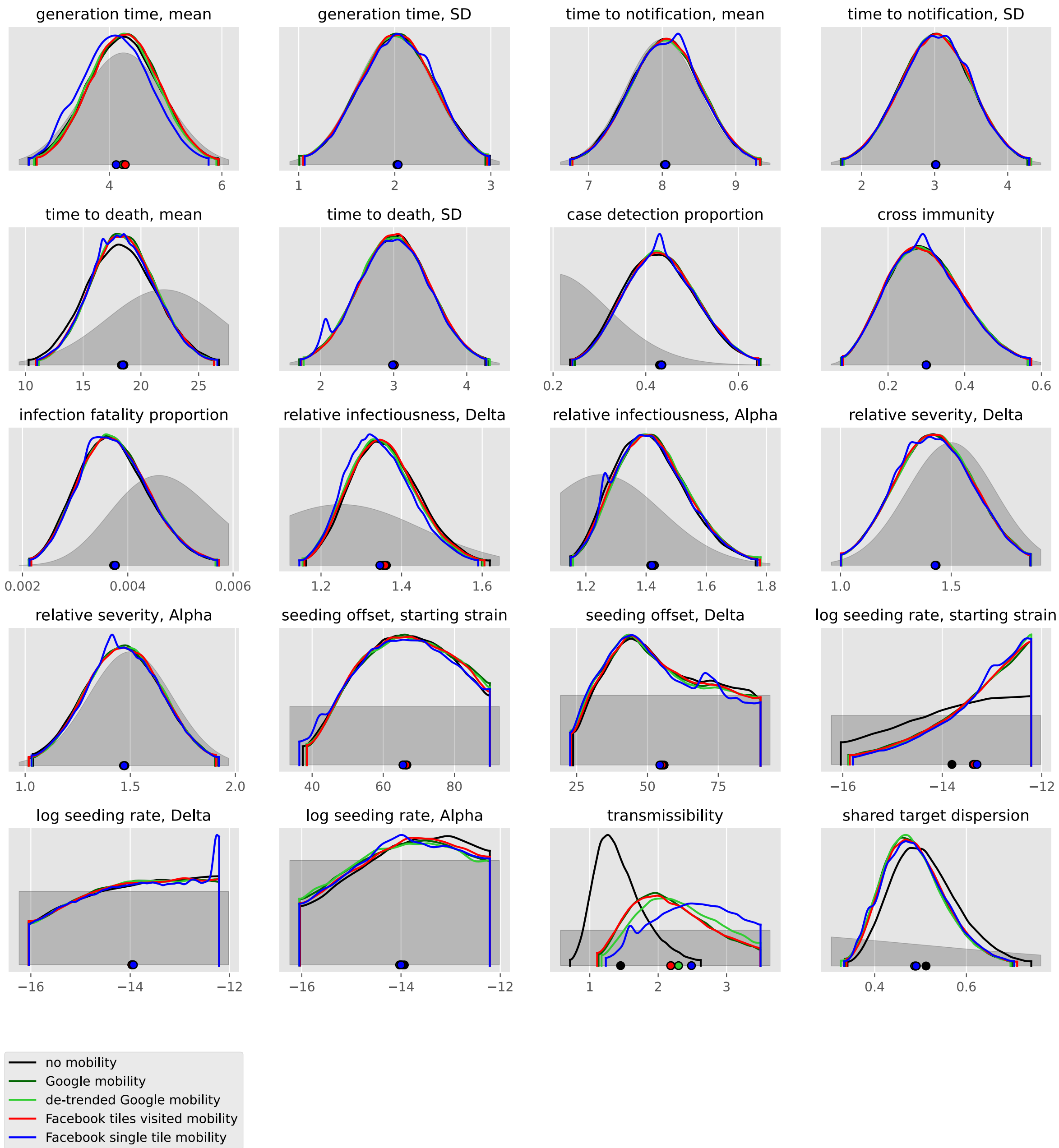

generation time, mean
generation time, SD
time to notification, mean
time to notification, SD
time to death, mean
time to death, SD
case detection proportion
cross immunity
infection fatality proportion
relative infectiousness, Delta
relative infectiousness, Alpha
relative severity, Delta
relative severity, Alpha
seeding offset, starting strain
seeding offset, Delta
log seeding rate, starting strain
log seeding rate, Delta
log seeding rate, Alpha
transmissibility
shared target dispersion
no mobility
Google mobility
de-trended Google mobility
Facebook tiles visited mobility
Facebook single tile mobility

Oman

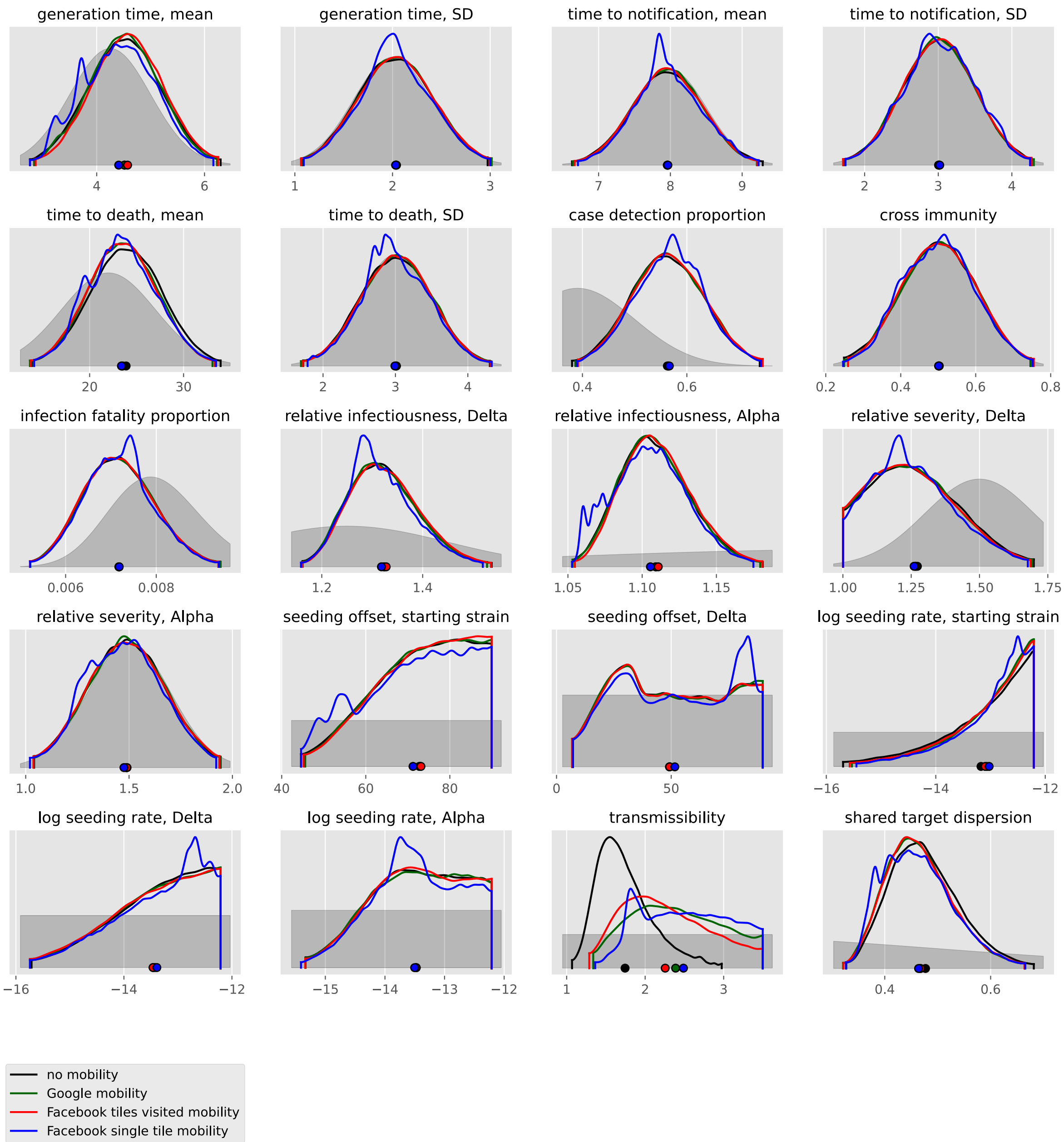

generation time, mean
generation time, SD
time to notification, mean
time to notification, SD
time to death, mean
time to death, SD
case detection proportion
cross immunity
infection fatality proportion
relative infectiousness, Delta
relative infectiousness, Alpha
relative severity, Delta
relative severity, Alpha
seeding offset, starting strain
seeding offset, Delta
log seeding rate, starting strain
log seeding rate, Delta
log seeding rate, Alpha
transmissibility
shared target dispersion
no mobility
Google mobility
Facebook tiles visited mobility
Facebook single tile mobility

Pakistan

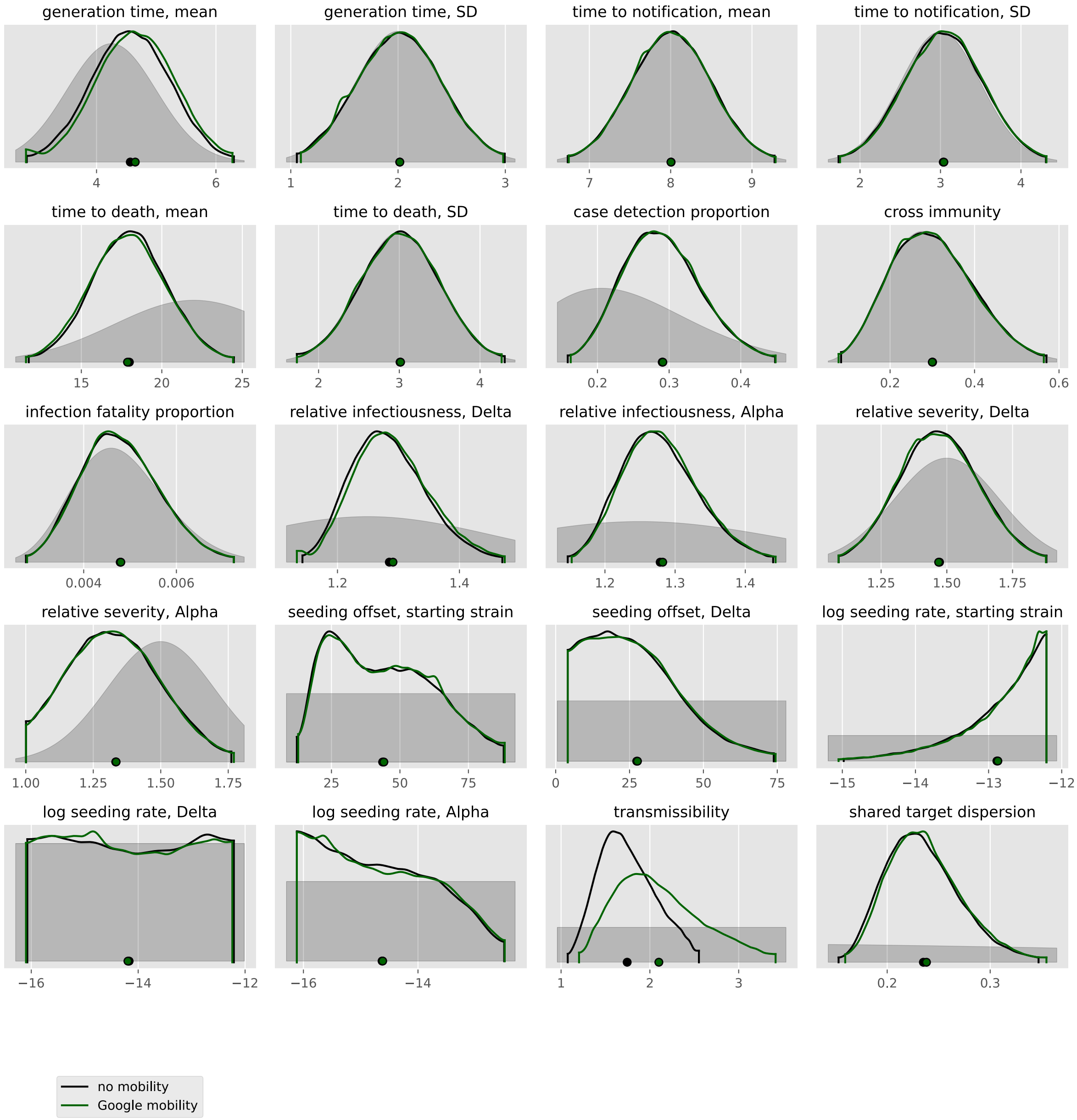

generation time, mean
generation time, SD
time to notification, mean
time to notification, SD
time to death, mean
time to death, SD
case detection proportion
cross immunity
infection fatality proportion
relative infectiousness, Delta
relative infectiousness, Alpha
relative severity, Delta
relative severity, Alpha
seeding offset, starting strain
seeding offset, Delta
log seeding rate, starting strain
log seeding rate, Delta
log seeding rate, Alpha
transmissibility
shared target dispersion
no mobility
Google mobility

Philippines

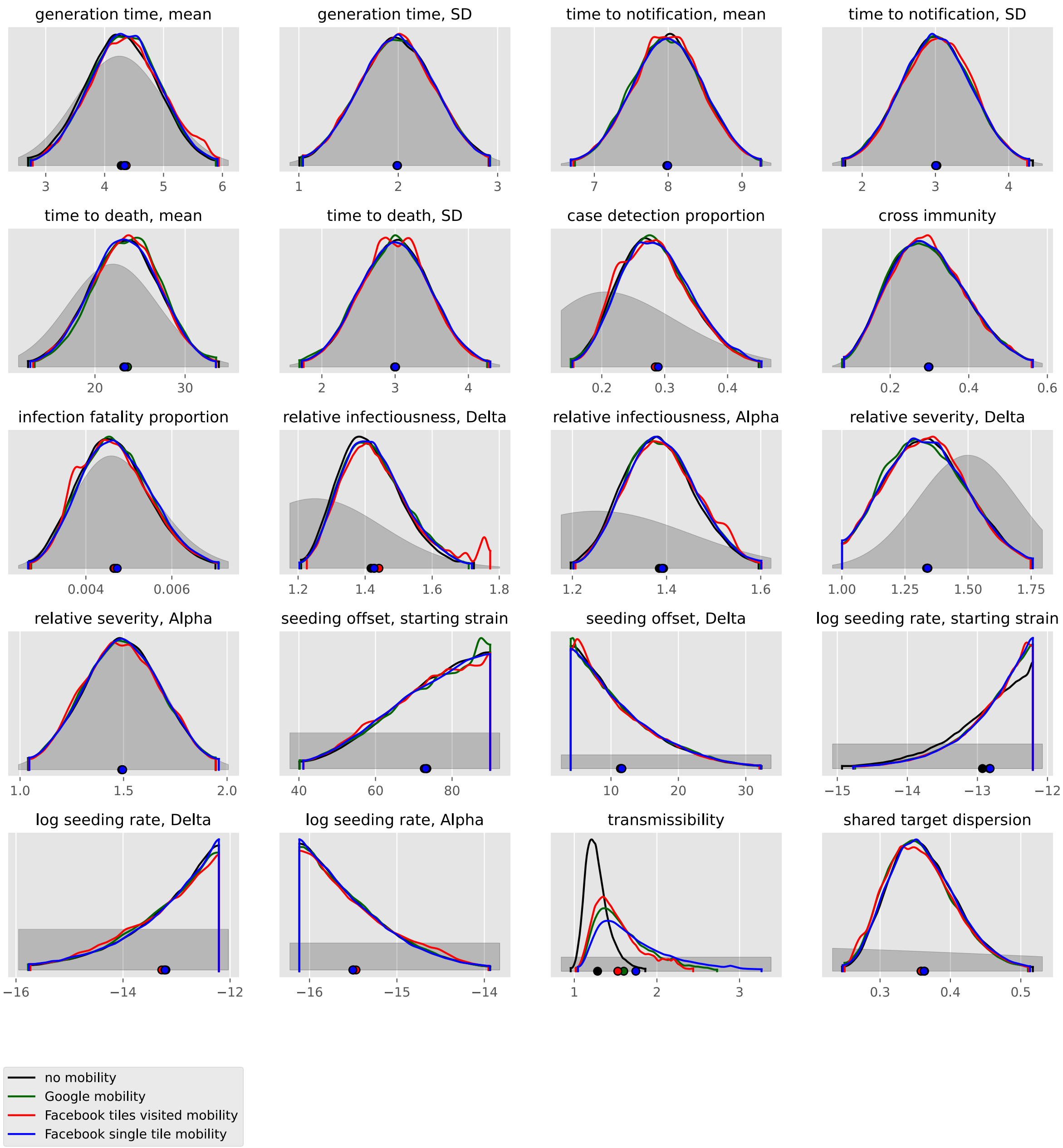

Qatar

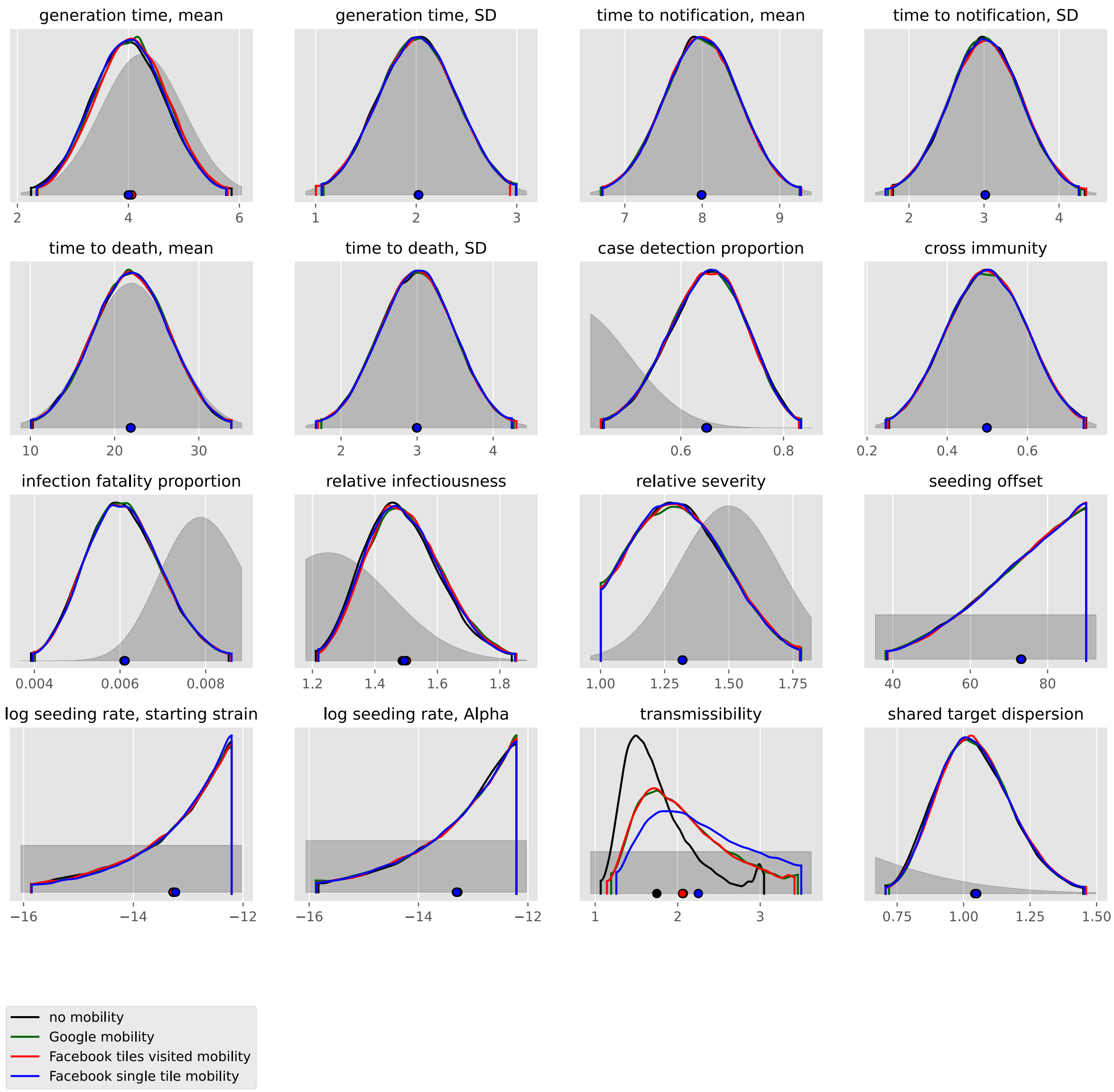

generation time, mean
generation time, SD
time to notification, mean
time to notification, SD
time to death, mean
time to death, SD
case detection proportion
cross immunity
infection fatality proportion
relative infectiousness
relative severity
seeding offset
log seeding rate, starting strain
log seeding rate, Alpha
transmissibility
shared target dispersion
no mobility
Google mobility
Facebook tiles visited mobility
Facebook single tile mobility

Saudi Arabia

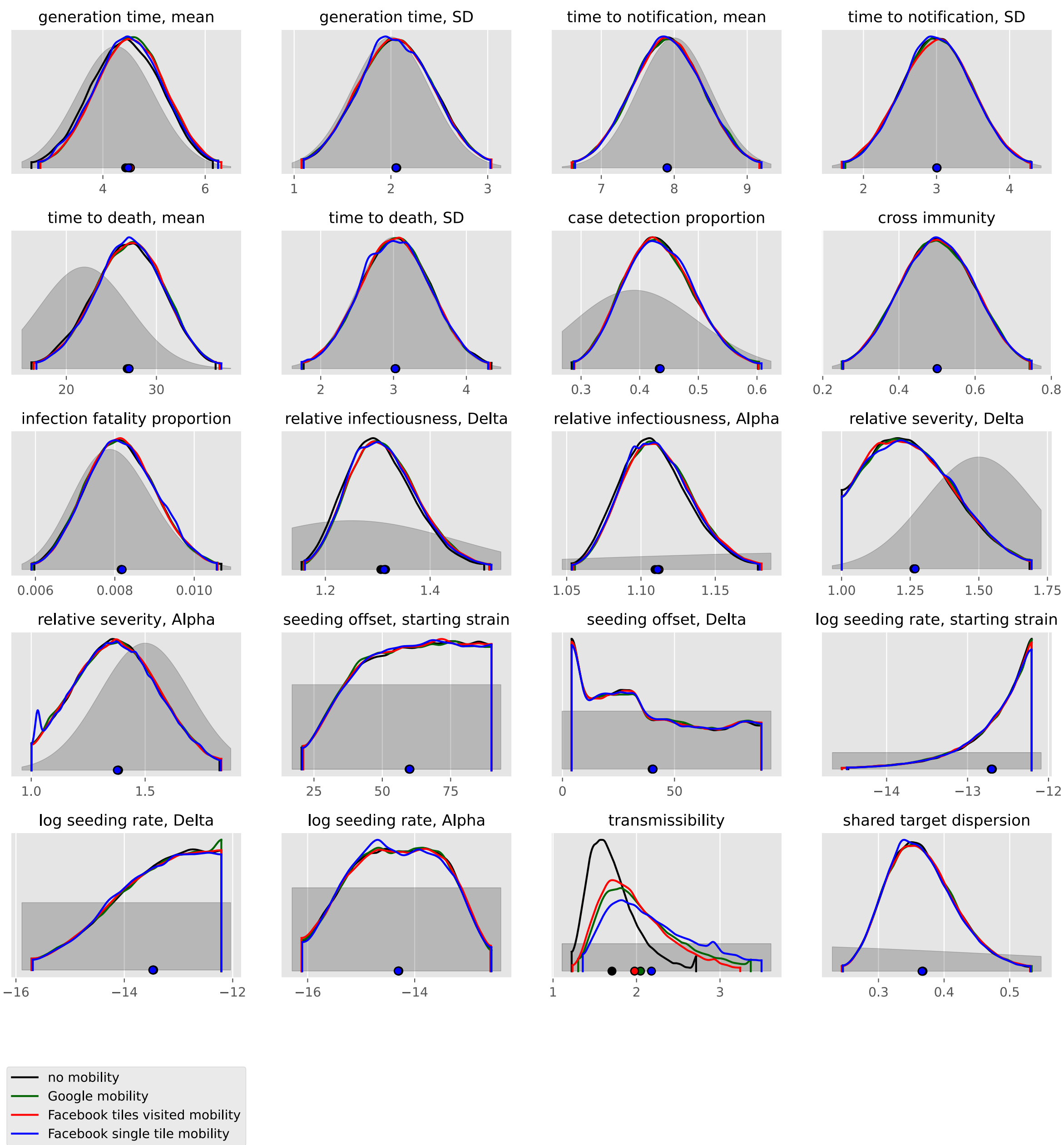

Sri Lanka

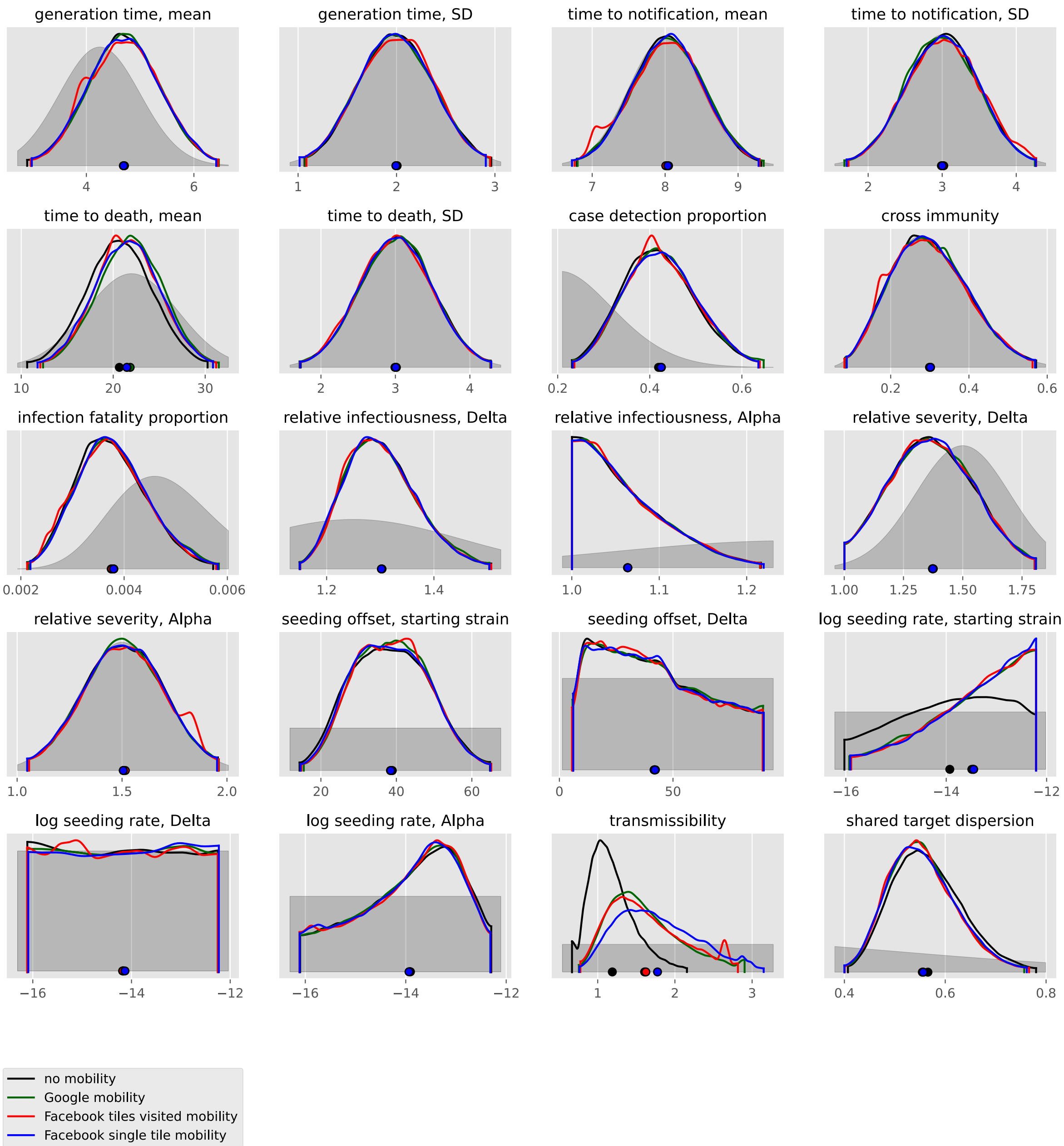

generation time, mean
4
6
generation time, SD
1
2
3
time to notification, mean
7
8
9
time to notification, SD
2
3
4
time to death, mean
10
20
30
time to death, SD
2
3
4
case detection proportion
0.2
0.4
0.6
cross immunity
0.2
0.4
0.6
infection fatality proportion
0.002
0.004
0.006
relative infectiousness, Delta
1.2
1.4
relative infectiousness, Alpha
1.0
1.1
1.2
relative severity, Delta
1.00
1.25
1.50
1.75
relative severity, Alpha
1.0
1.5
2.0
seeding offset, starting strain
20
40
60
seeding offset, Delta
0
50
log seeding rate, starting strain
−16
−14
−12
log seeding rate, Delta
−16
−14
−12
log seeding rate, Alpha
−16
−14
−12
transmissibility
1
2
3
shared target dispersion
0.4
0.6
0.8
no mobility
Google mobility
Facebook tiles visited mobility
Facebook single tile mobility

Türkiye

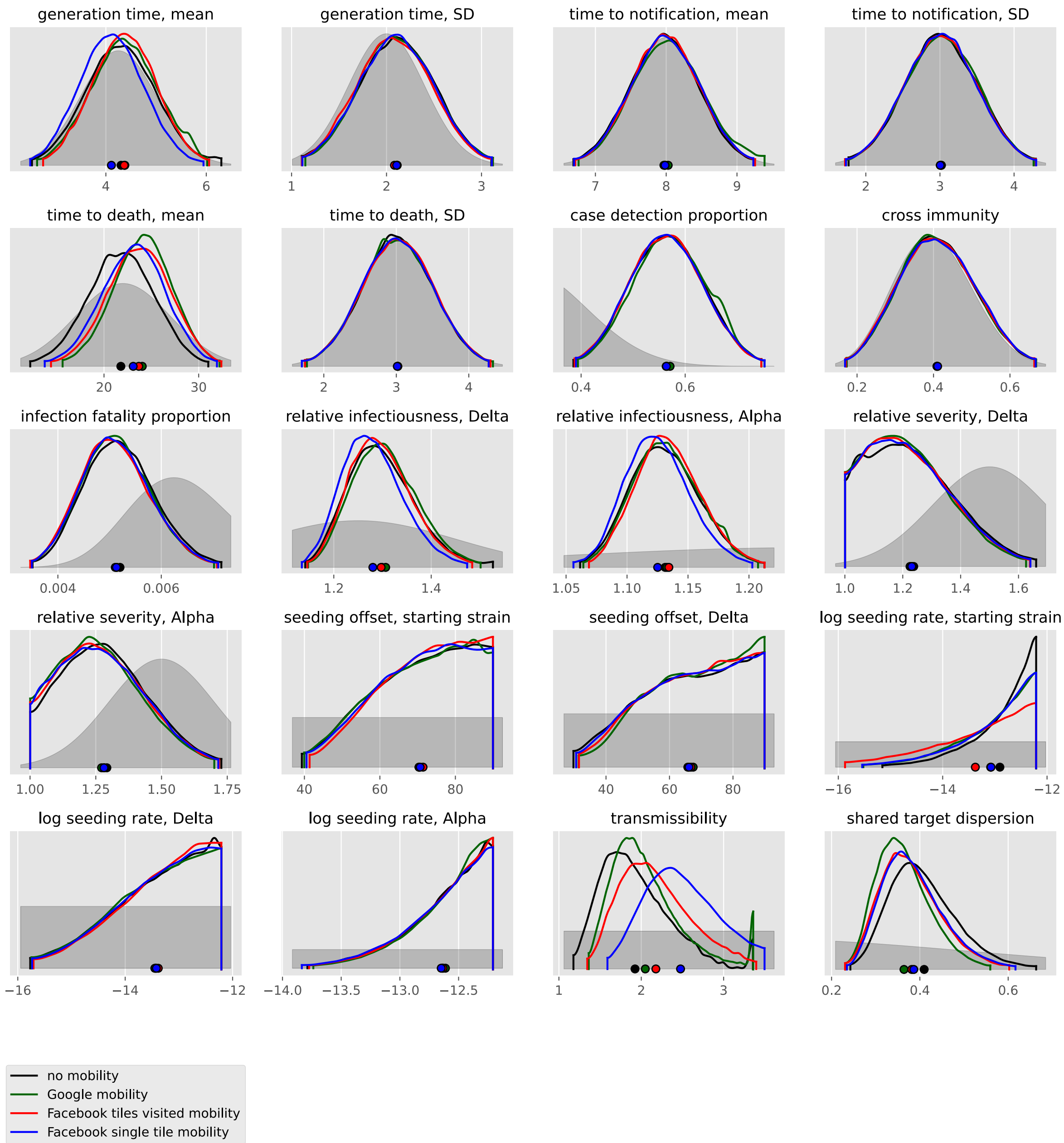
generation time, mean
generation time, SD
time to notification, mean
time to notification, SD
time to death, mean
time to death, SD
case detection proportion
cross immunity
infection fatality proportion
relative infectiousness, Delta
relative infectiousness, Alpha
relative severity, Delta
relative severity, Alpha
seeding offset, starting strain
seeding offset, Delta
log seeding rate, starting strain
log seeding rate, Delta
log seeding rate, Alpha
transmissibility
shared target dispersion
no mobility
Google mobility
Facebook tiles visited mobility
Facebook single tile mobility

United Arab Emirates

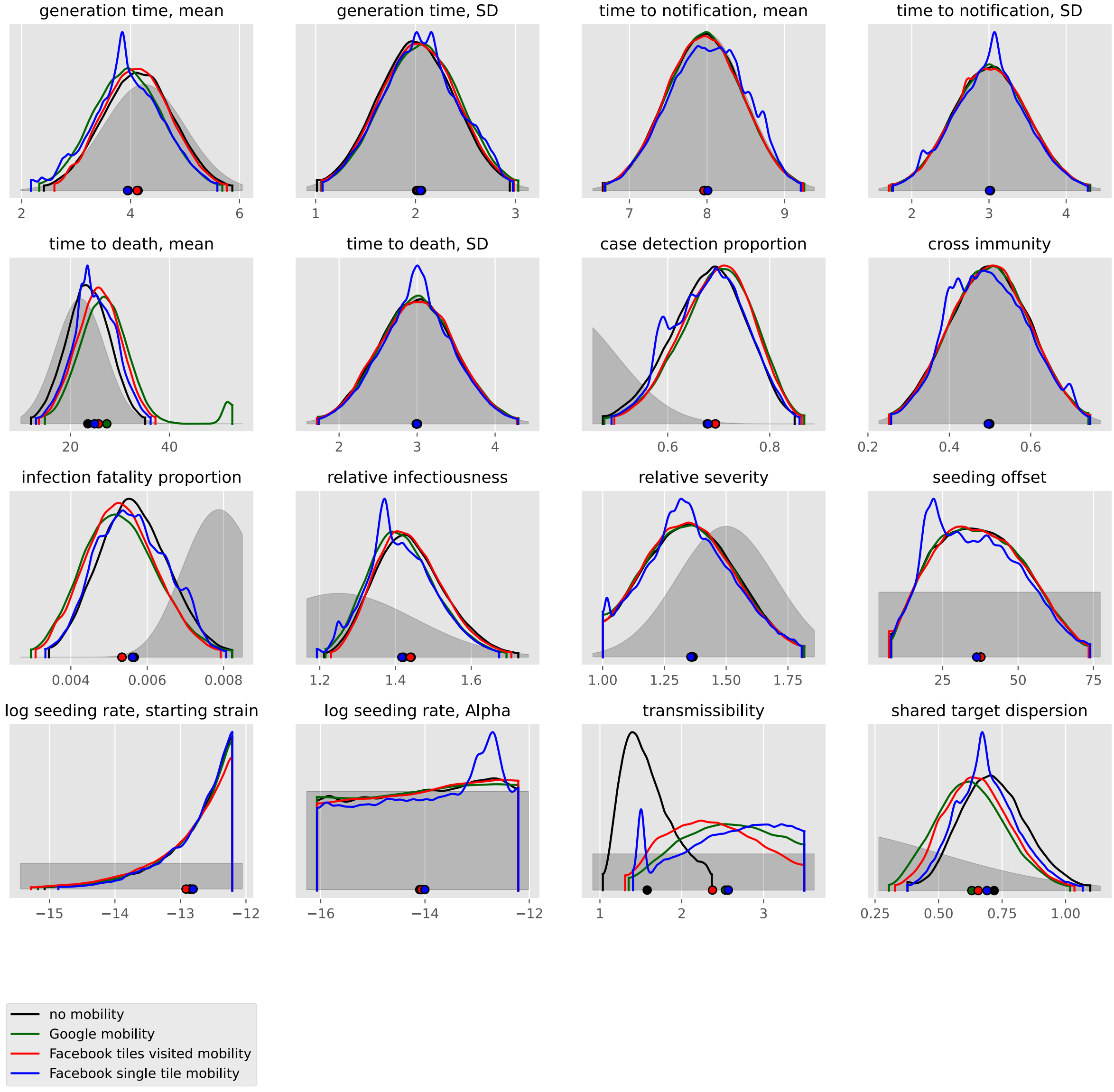

Yemen

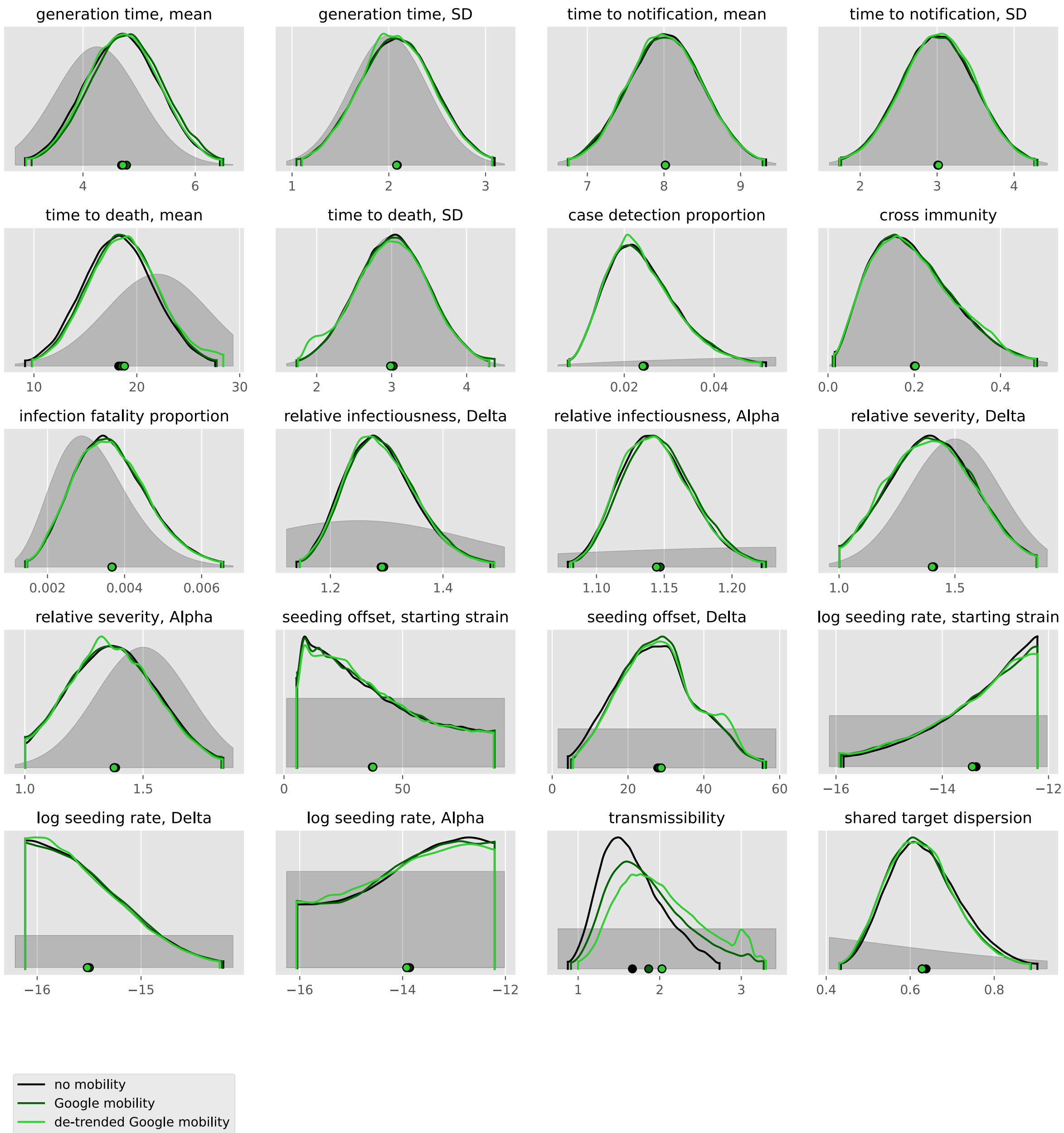

generation time, mean
4
6
generation time, SD
1
2
3
time to notification, mean
7
8
9
time to notification, SD
2
3
4
time to death, mean
10
20
30
time to death, SD
2
3
4
case detection proportion
0.02
0.04
cross immunity
0.0
0.2
0.4
infection fatality proportion
0.002
0.004
0.006
relative infectiousness, Delta
1.2
1.4
relative infectiousness, Alpha
1.10
1.15
1.20
relative severity, Delta
1.0
1.5
relative severity, Alpha
1.0
1.5
seeding offset, starting strain
0
50
seeding offset, Delta
0
20
40
60
log seeding rate, starting strain
−16
−14
−12
log seeding rate, Delta
−16
−15
log seeding rate, Alpha
−16
−14
−12
transmissibility
1
2
3
shared target dispersion
0.4
0.6
0.8
no mobility
Google mobility
de-trended Google mobility

Europe

Albania

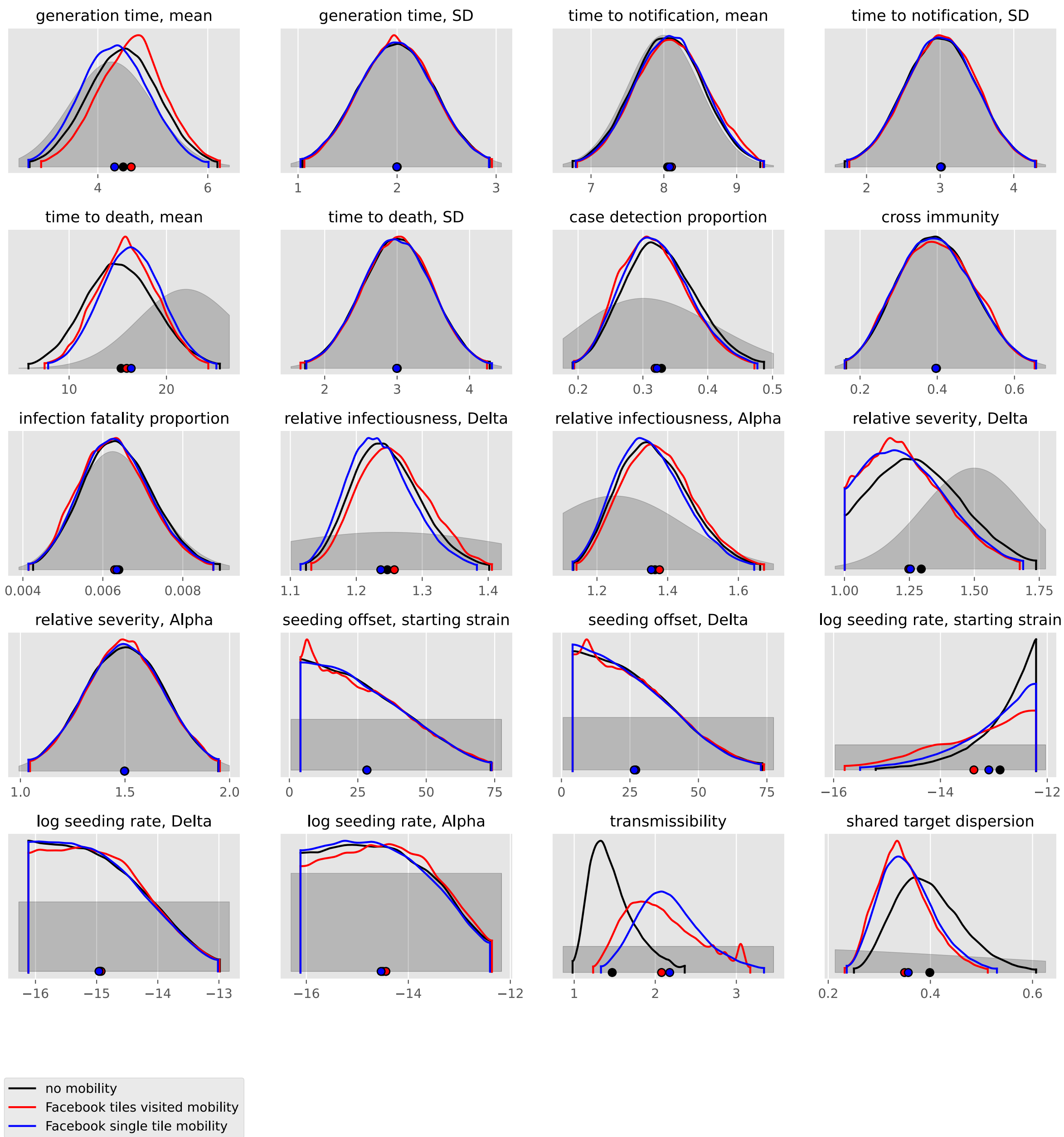

Austria

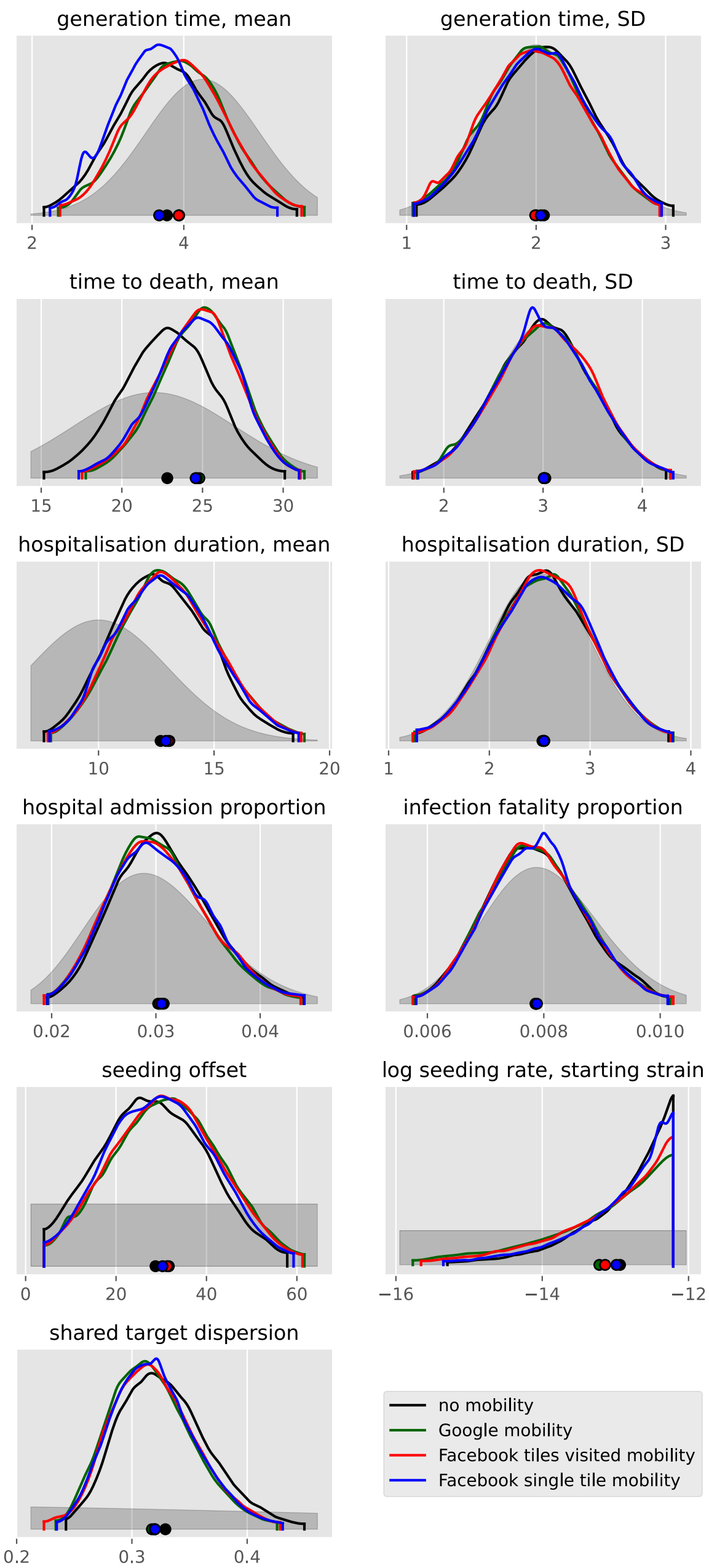
generation time, mean
2
4
generation time, SD
1
2
3
time to notification, mean
7
8
9
time to notification, SD
2
3
4
time to death, mean
15
20
25
30
time to death, SD
2
3
4
time to admission, mean
10
15
20
time to admission, SD
1
2
3
hospitalisation duration, mean
10
15
20
hospitalisation duration, SD
1
2
3
4
case detection proportion
0.3
0.4
0.5
0.6
cross immunity
0.2
0.4
0.6
hospital admission proportion
0.02
0.03
0.04
infection fatality proportion
0.006
0.008
0.010
relative infectiousness
1.2
1.4
1.6
1.8
relative severity
1.00
1.25
1.50
1.75
seeding offset
0
20
40
60
log seeding rate, starting strain
−16
−14
−12
log seeding rate, Alpha
−16
−14
−12
transmissibility
2
3
shared target dispersion
0.2
0.3
0.4
no mobility
Google mobility
Facebook tiles visited mobility
Facebook single tile mobility

Belarus

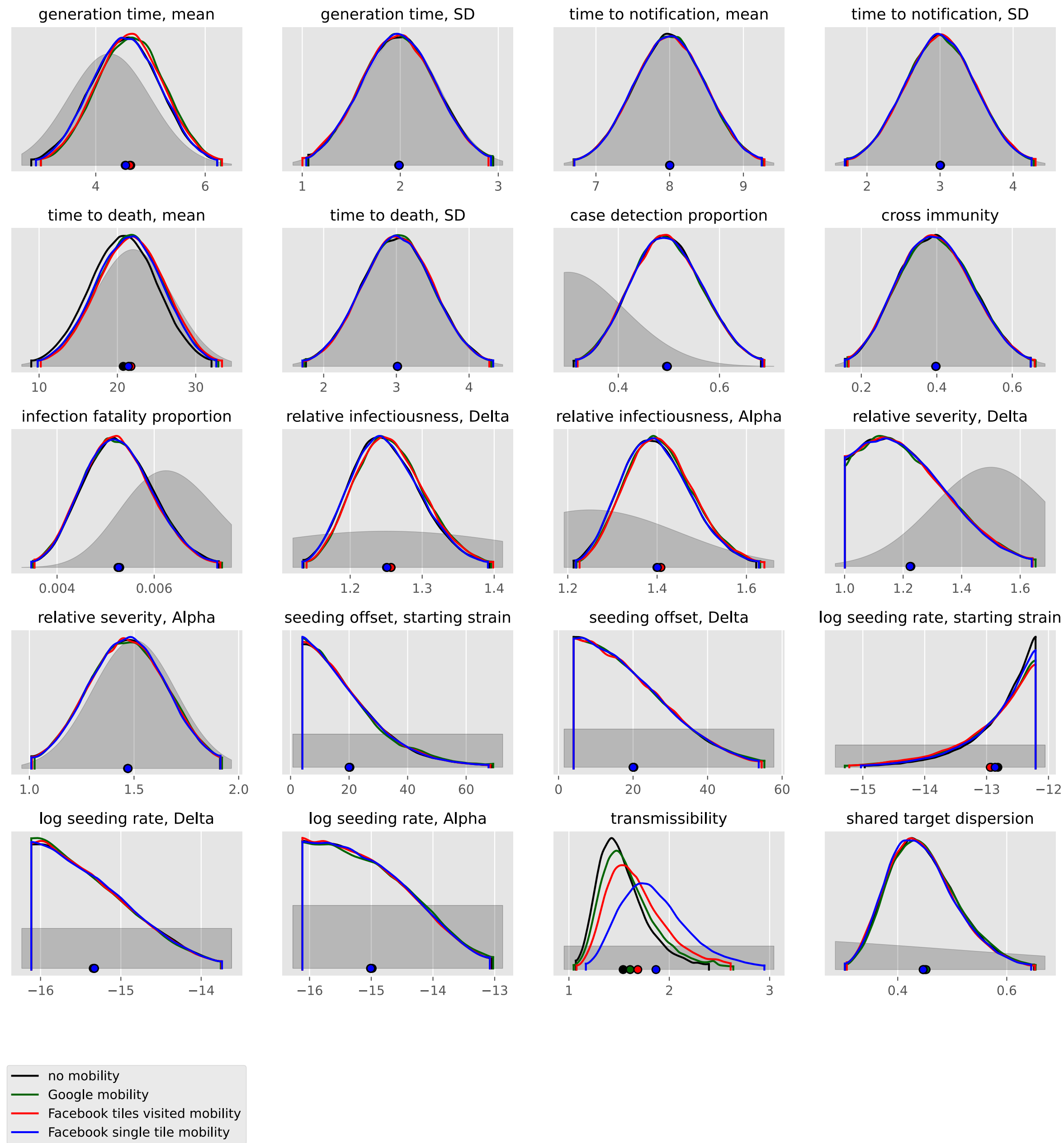
generation time, mean
generation time, SD
time to notification, mean
time to notification, SD
time to death, mean
time to death, SD
case detection proportion
cross immunity
infection fatality proportion
relative infectiousness, Delta
relative infectiousness, Alpha
relative severity, Delta
relative severity, Alpha
seeding offset, starting strain
seeding offset, Delta
log seeding rate, starting strain
log seeding rate, Delta
log seeding rate, Alpha
transmissibility
shared target dispersion
no mobility
Google mobility
Facebook tiles visited mobility
Facebook single tile mobility

Belgium

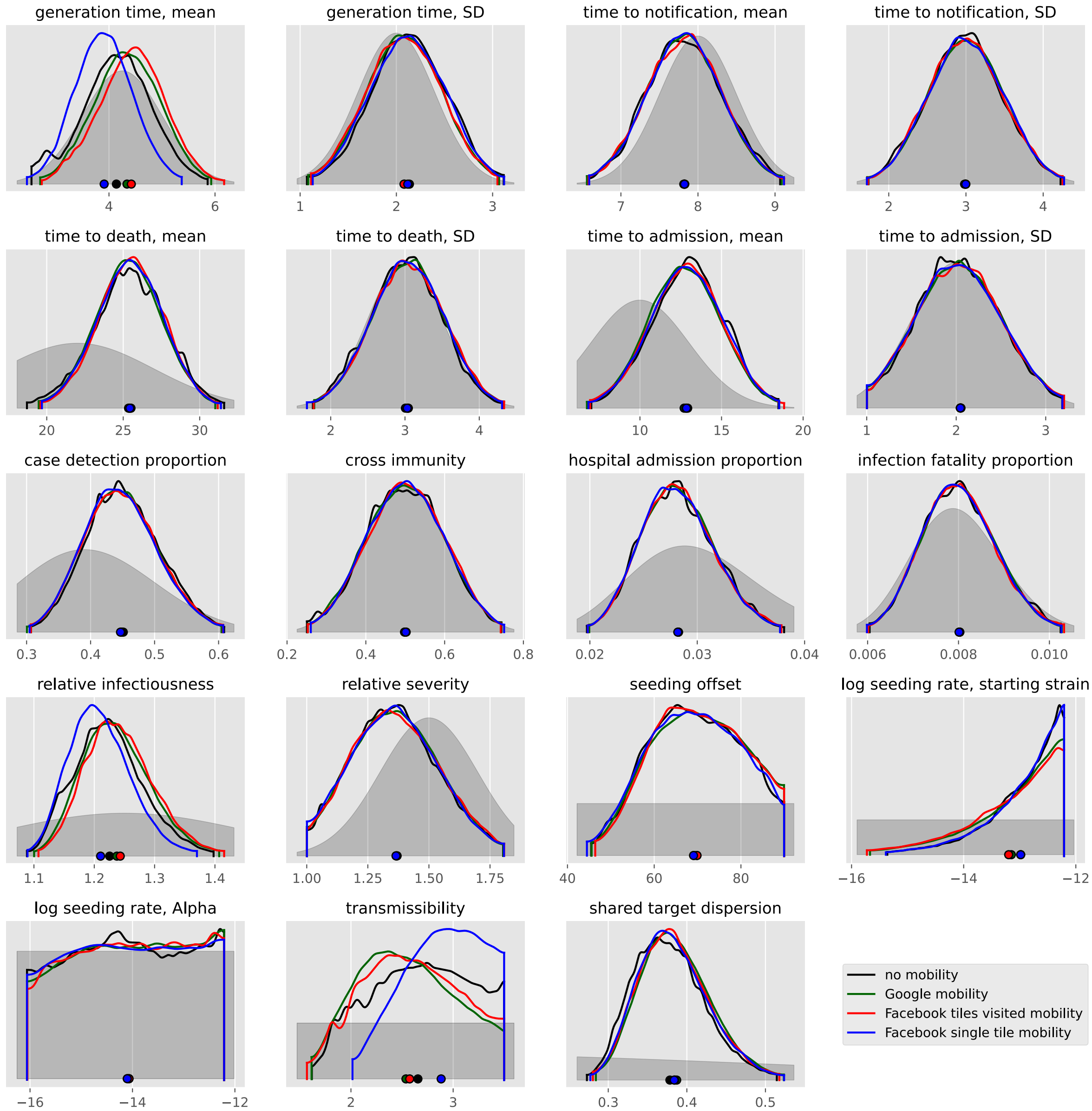
generation time, mean
generation time, SD
time to notification, mean
time to notification, SD
time to death, mean
time to death, SD
time to admission, mean
time to admission, SD
case detection proportion
cross immunity
hospital admission proportion
infection fatality proportion
relative infectiousness
relative severity
seeding offset
log seeding rate, starting strain
log seeding rate, Alpha
transmissibility
shared target dispersion
no mobility
Google mobility
Facebook tiles visited mobility
Facebook single tile mobility

Bosnia and Herzegovina

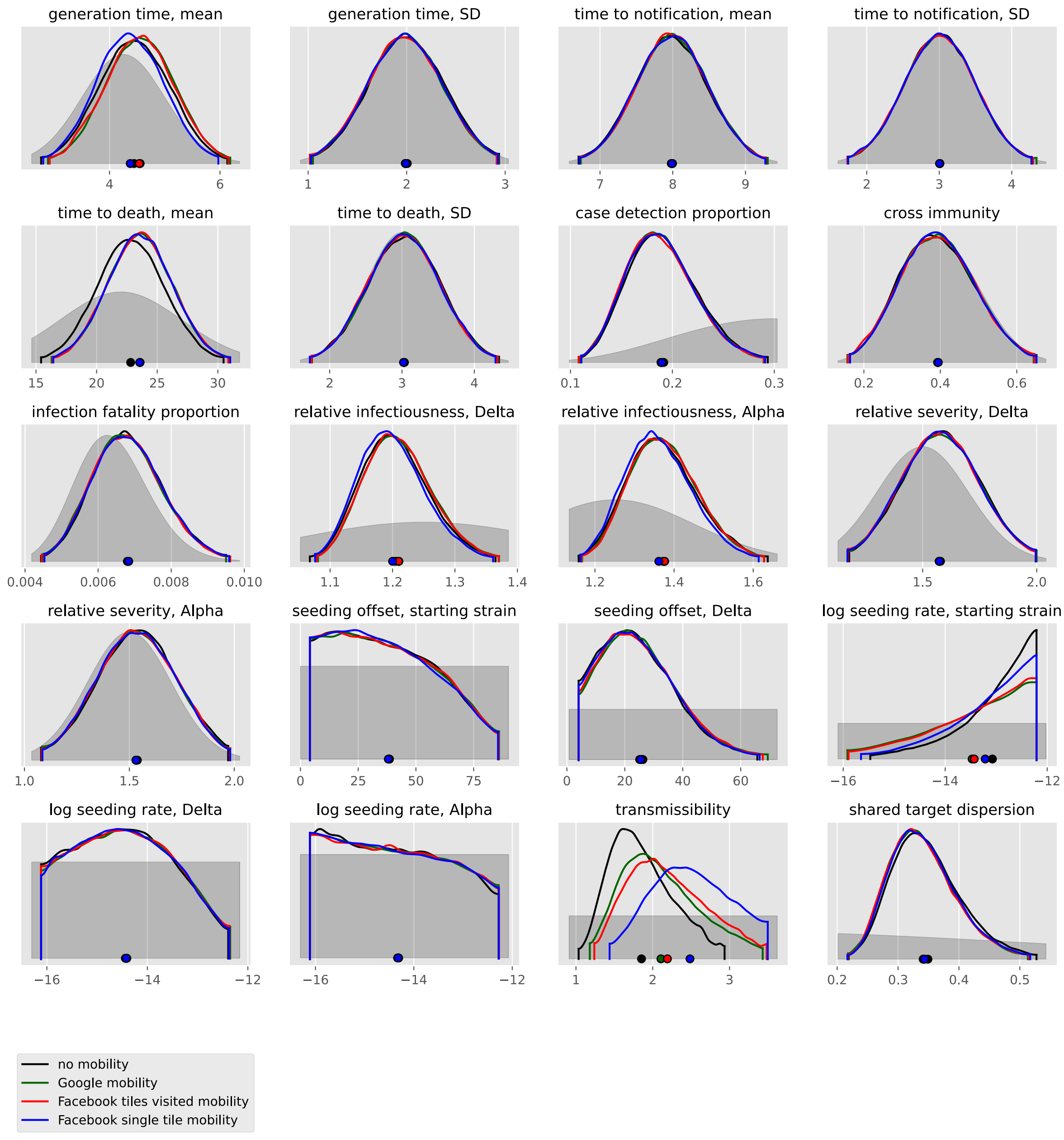

Bulgaria

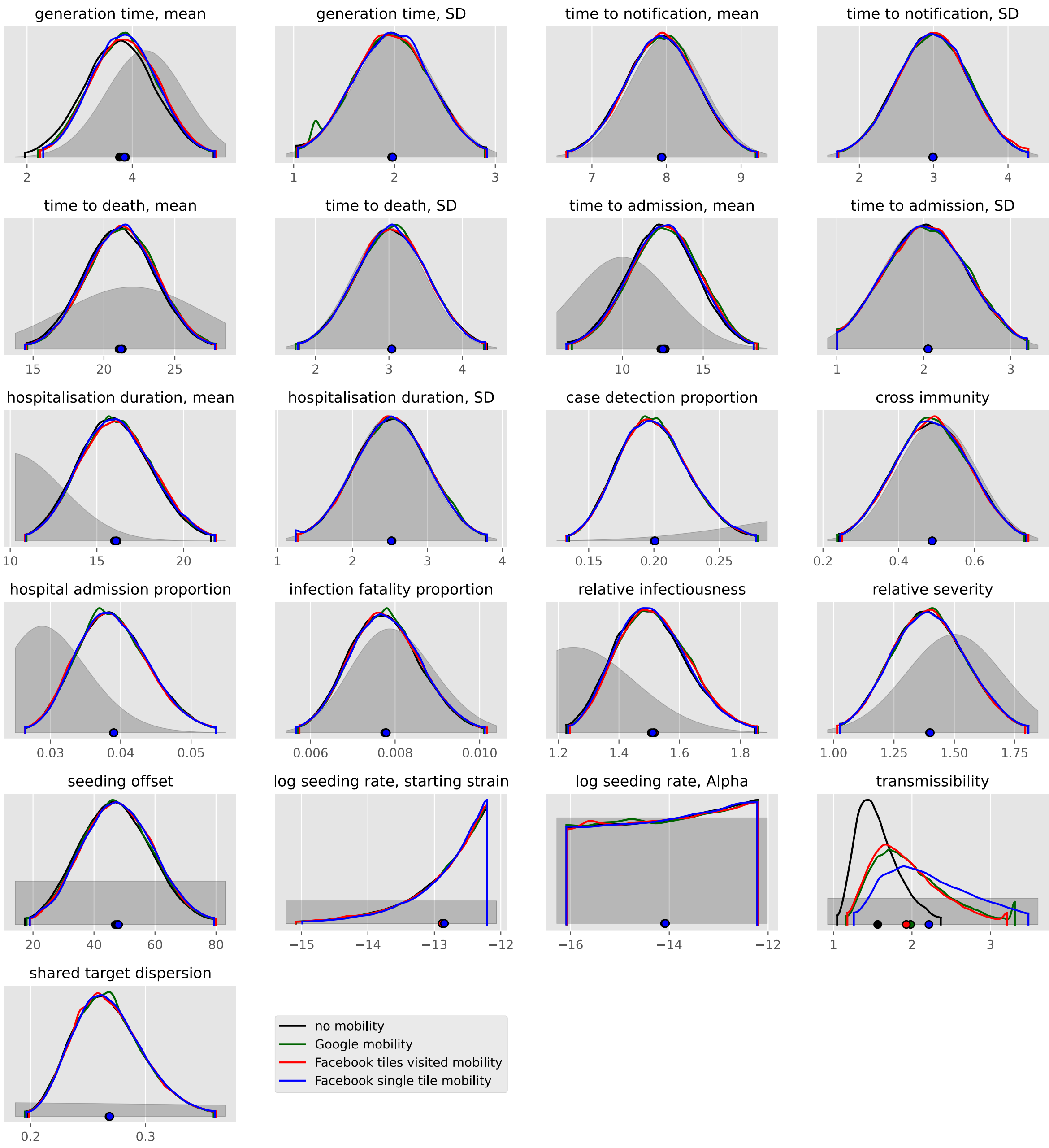

generation time, mean
2
4
generation time, SD
1
2
3
time to notification, mean
7
8
9
time to notification, SD
2
3
4
time to death, mean
15
20
25
time to death, SD
2
3
4
time to admission, mean
10
15
time to admission, SD
1
2
3
hospitalisation duration, mean
10
15
20
hospitalisation duration, SD
1
2
3
4
case detection proportion
0.15
0.20
0.25
cross immunity
0.2
0.4
0.6
hospital admission proportion
0.03
0.04
0.05
infection fatality proportion
0.006
0.008
0.010
relative infectiousness
1.2
1.4
1.6
1.8
relative severity
1.00
1.25
1.50
1.75
seeding offset
20
40
60
80
log seeding rate, starting strain
−15
−14
−13
−12
log seeding rate, Alpha
−16
−14
−12
transmissibility
1
2
3
shared target dispersion
0.2
0.3
no mobility
Google mobility
Facebook tiles visited mobility
Facebook single tile mobility

Croatia

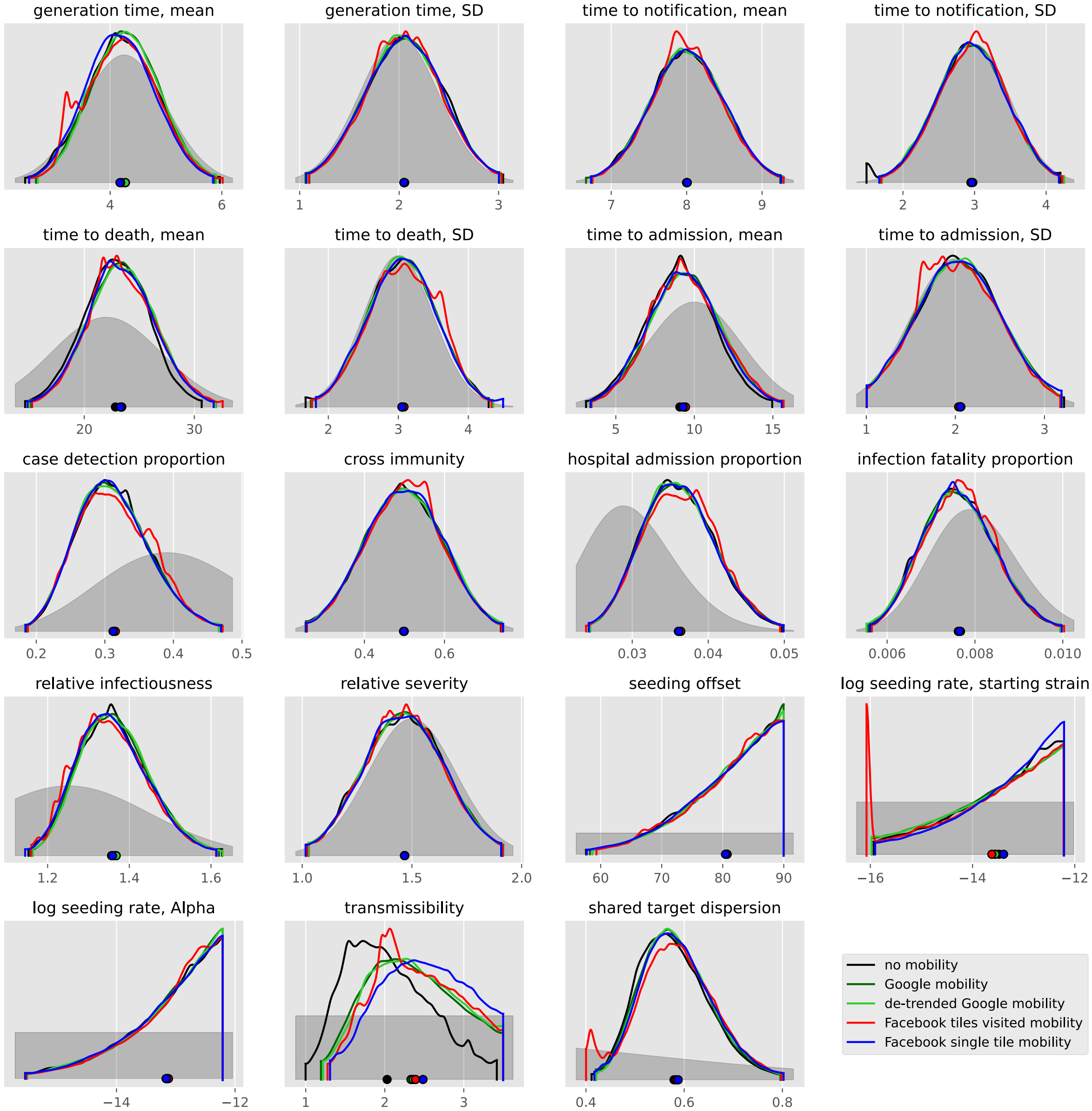

generation time, mean
generation time, SD
time to notification, mean
time to notification, SD
time to death, mean
time to death, SD
time to admission, mean
time to admission, SD
case detection proportion
cross immunity
hospital admission proportion
infection fatality proportion
relative infectiousness
relative severity
seeding offset
log seeding rate, starting strain
log seeding rate, Alpha
transmissibility
shared target dispersion
no mobility
Google mobility
de-trended Google mobility
Facebook tiles visited mobility
Facebook single tile mobility

Czechia

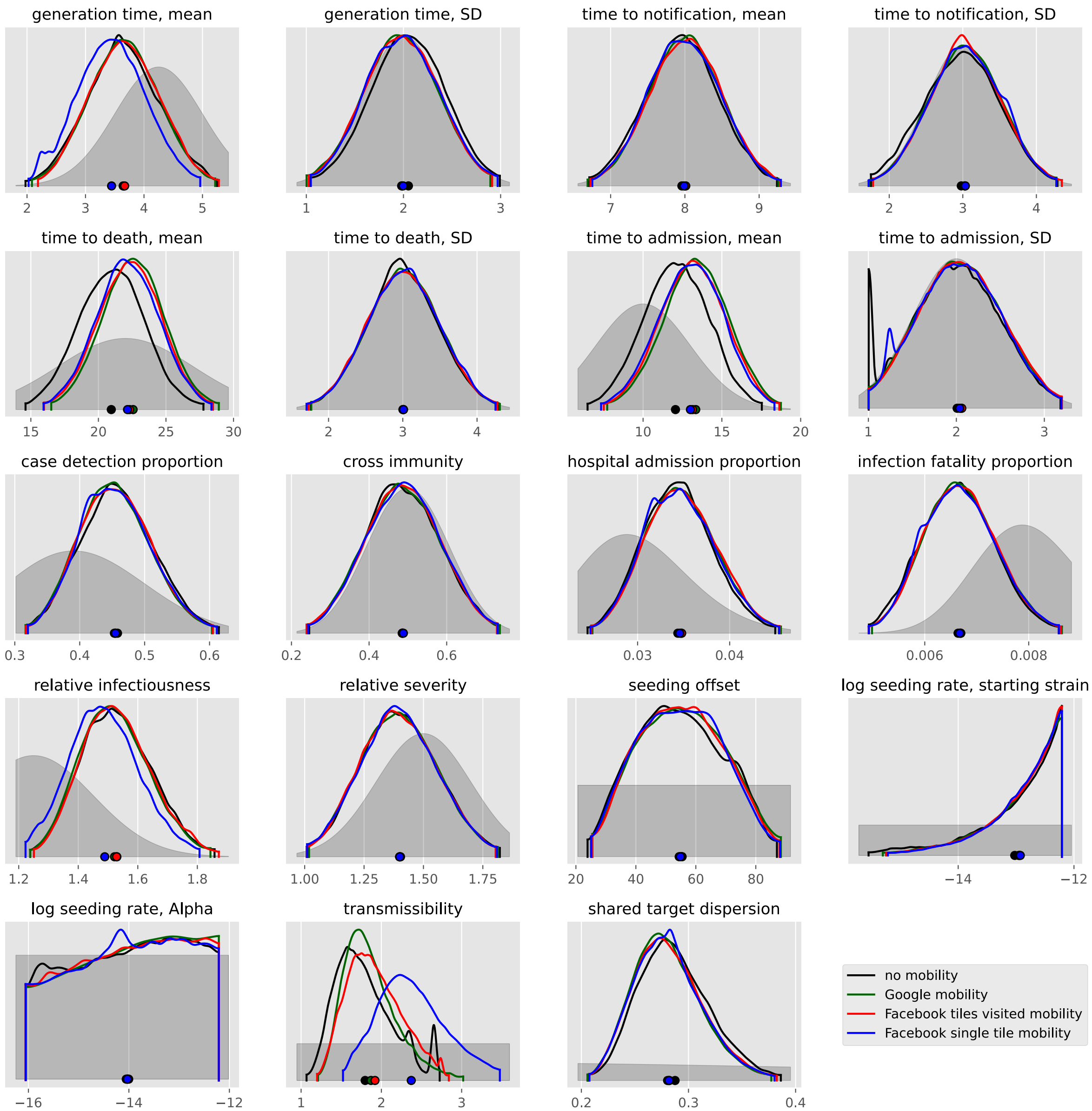
generation time, mean
generation time, SD
time to notification, mean
time to notification, SD
time to death, mean
time to death, SD
time to admission, mean
time to admission, SD
case detection proportion
cross immunity
hospital admission proportion
infection fatality proportion
relative infectiousness
relative severity
seeding offset
log seeding rate, starting strain
log seeding rate, Alpha
transmissibility
shared target dispersion
no mobility
Google mobility
Facebook tiles visited mobility
Facebook single tile mobility

Denmark

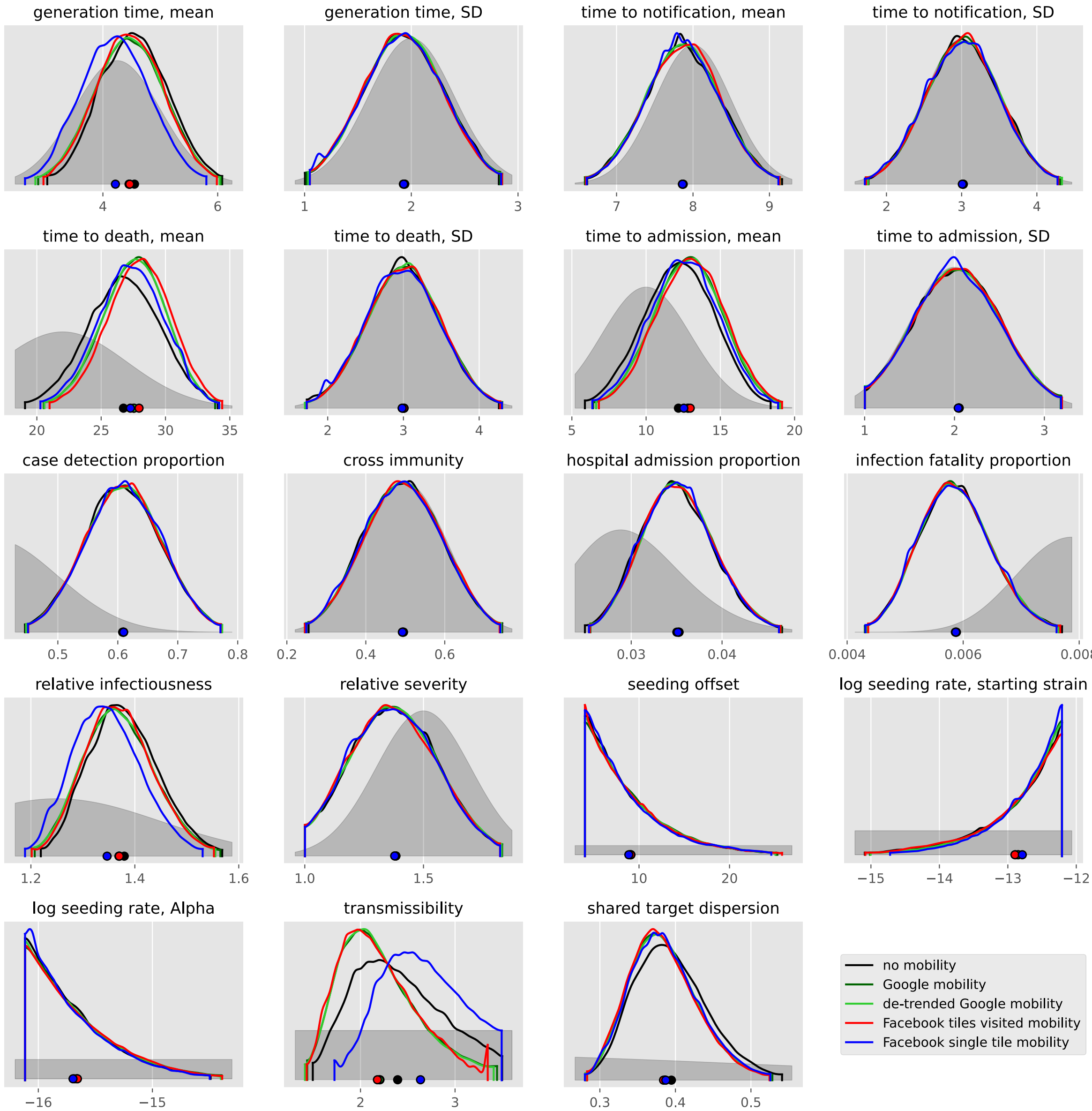
generation time, mean
generation time, SD
time to notification, mean
time to notification, SD
time to death, mean
time to death, SD
time to admission, mean
time to admission, SD
case detection proportion
cross immunity
hospital admission proportion
infection fatality proportion
relative infectiousness
relative severity
seeding offset
log seeding rate, starting strain
log seeding rate, Alpha
transmissibility
shared target dispersion
no mobility
Google mobility
de-trended Google mobility
Facebook tiles visited mobility
Facebook single tile mobility

Estonia

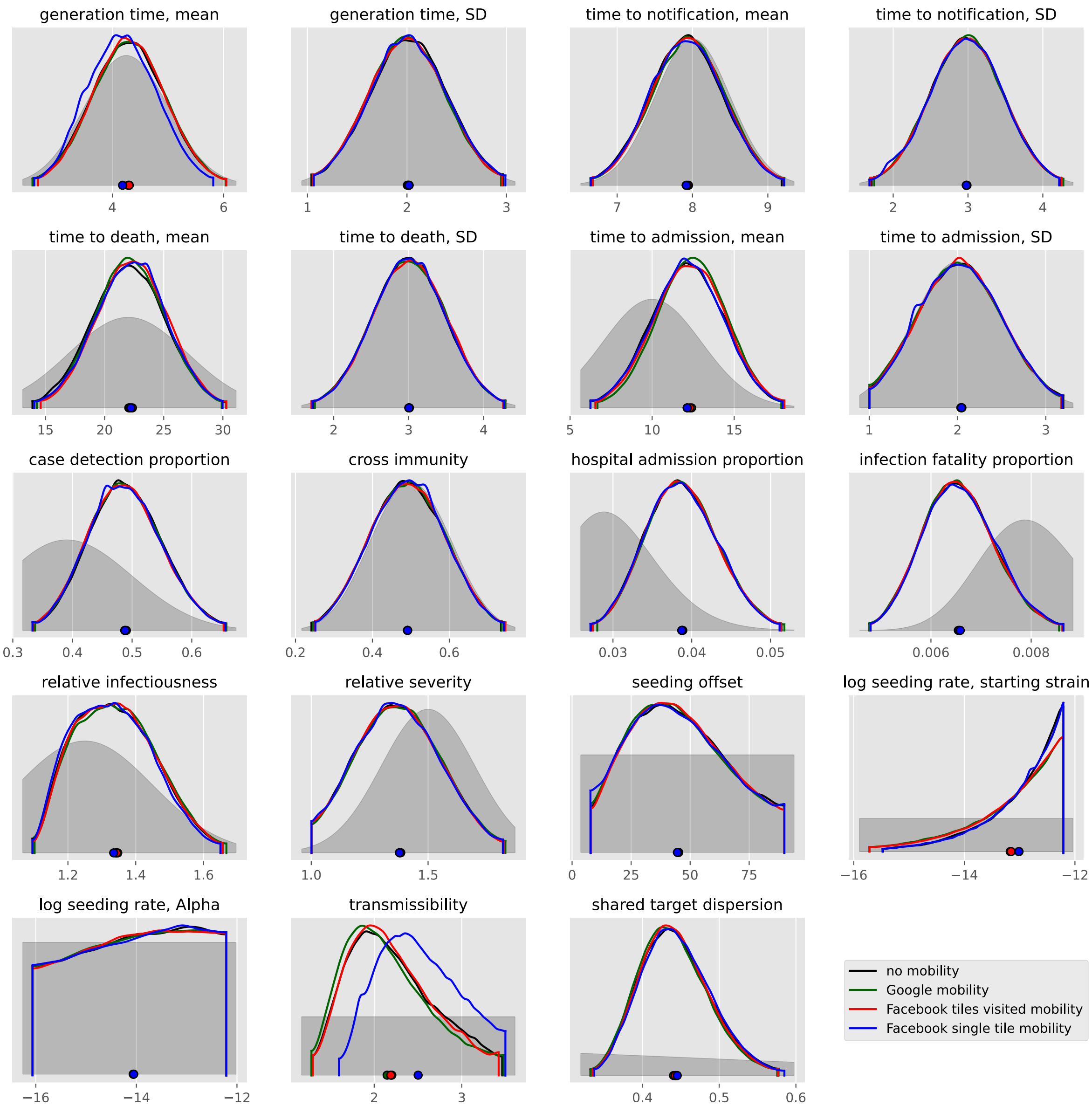

generation time, mean
generation time, SD
time to notification, mean
time to notification, SD
time to death, mean
time to death, SD
time to admission, mean
time to admission, SD
case detection proportion
cross immunity
hospital admission proportion
infection fatality proportion
relative infectiousness
relative severity
seeding offset
log seeding rate, starting strain
log seeding rate, Alpha
transmissibility
shared target dispersion
no mobility
Google mobility
Facebook tiles visited mobility
Facebook single tile mobility

Finland

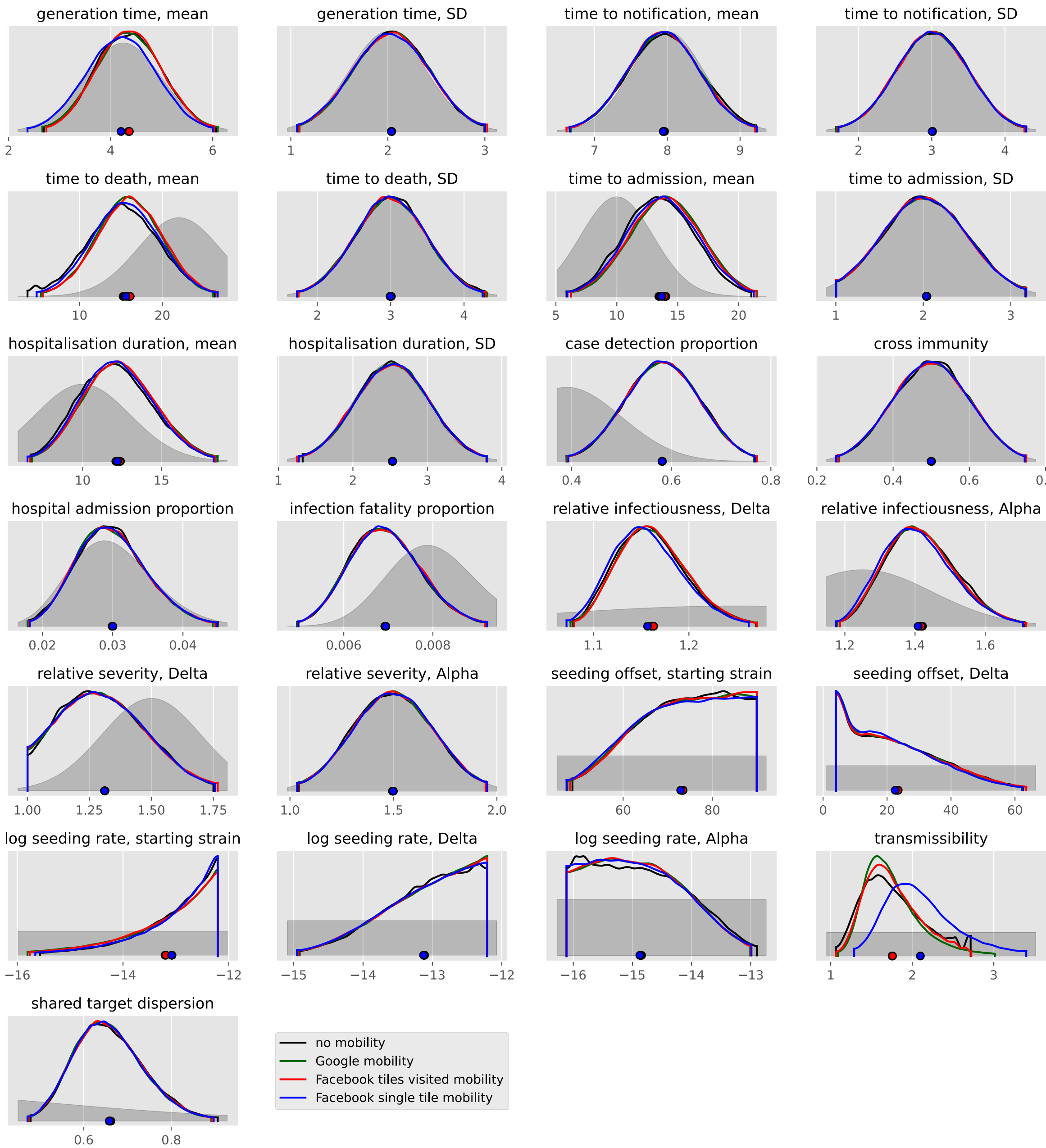
generation time, mean
generation time, SD
time to notification, mean
time to notification, SD
time to death, mean
time to death, SD
time to admission, mean
time to admission, SD
hospitalisation duration, mean
hospitalisation duration, SD
case detection proportion
cross immunity
hospital admission proportion
infection fatality proportion
relative infectiousness, Delta
relative infectiousness, Alpha
relative severity, Delta
relative severity, Alpha
seeding offset, starting strain
seeding offset, Delta
log seeding rate, starting strain
log seeding rate, Delta
log seeding rate, Alpha
transmissibility
shared target dispersion
no mobility
Google mobility
Facebook tiles visited mobility
Facebook single tile mobility

France

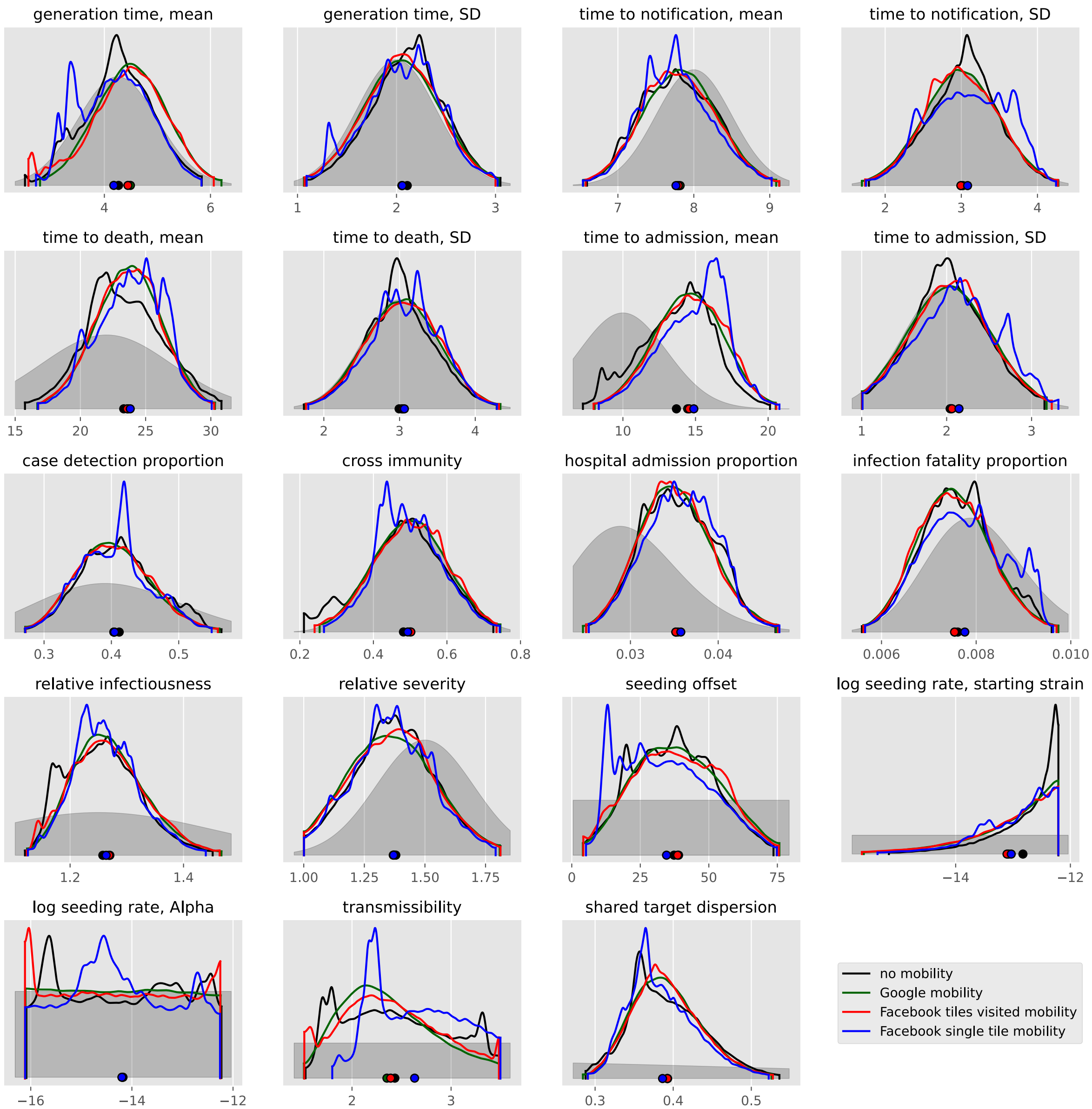

generation time, mean
generation time, SD
time to notification, mean
time to notification, SD
time to death, mean
time to death, SD
time to admission, mean
time to admission, SD
case detection proportion
cross immunity
hospital admission proportion
infection fatality proportion
relative infectiousness
relative severity
seeding offset
log seeding rate, starting strain
log seeding rate, Alpha
transmissibility
shared target dispersion
no mobility
Google mobility
Facebook tiles visited mobility
Facebook single tile mobility

Germany

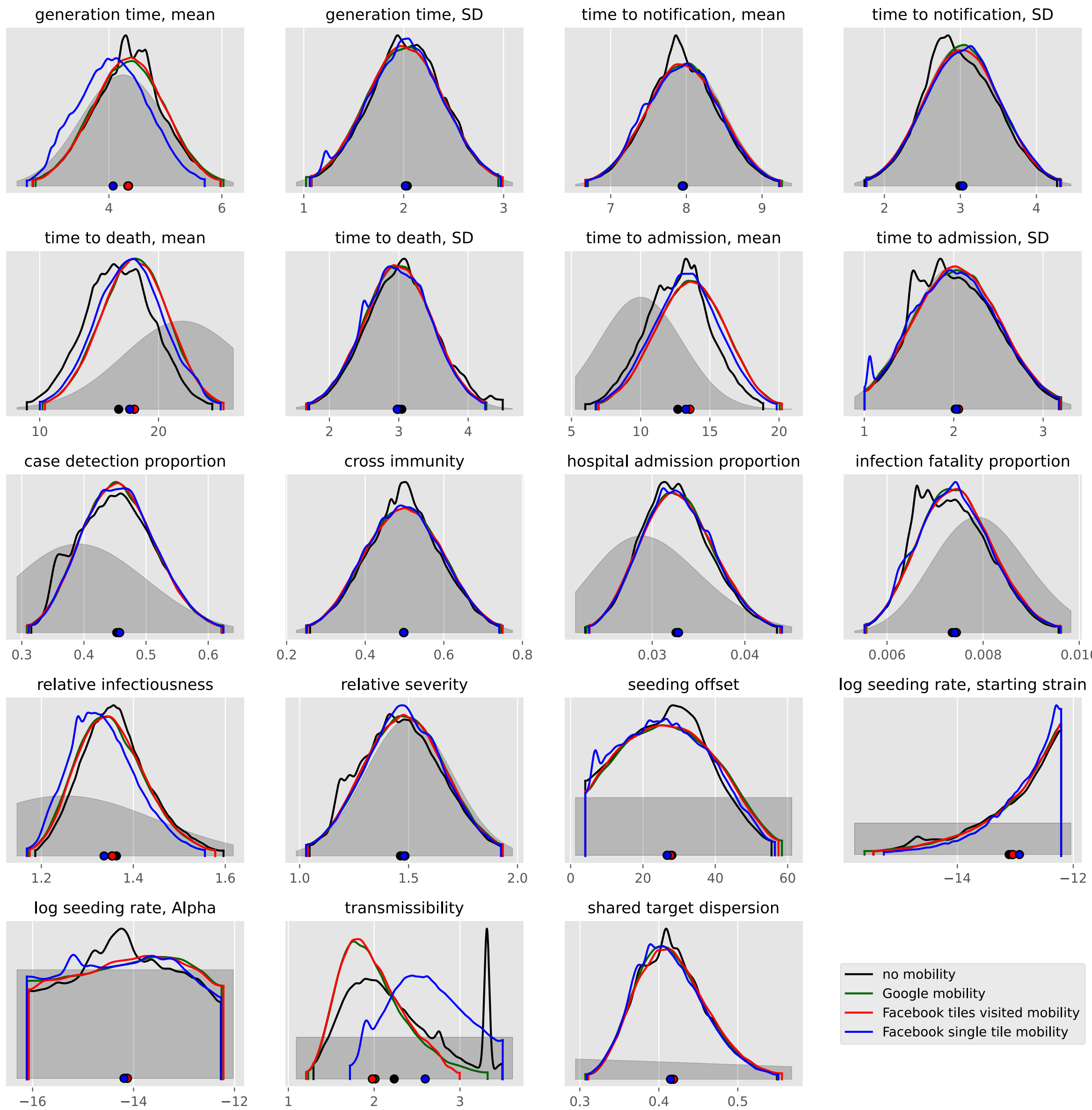
generation time, mean
generation time, SD
time to notification, mean
time to notification, SD
time to death, mean
time to death, SD
time to admission, mean
time to admission, SD
case detection proportion
cross immunity
hospital admission proportion
infection fatality proportion
relative infectiousness
relative severity
seeding offset
log seeding rate, starting strain
log seeding rate, Alpha
transmissibility
shared target dispersion
no mobility
Google mobility
Facebook tiles visited mobility
Facebook single tile mobility

Greece

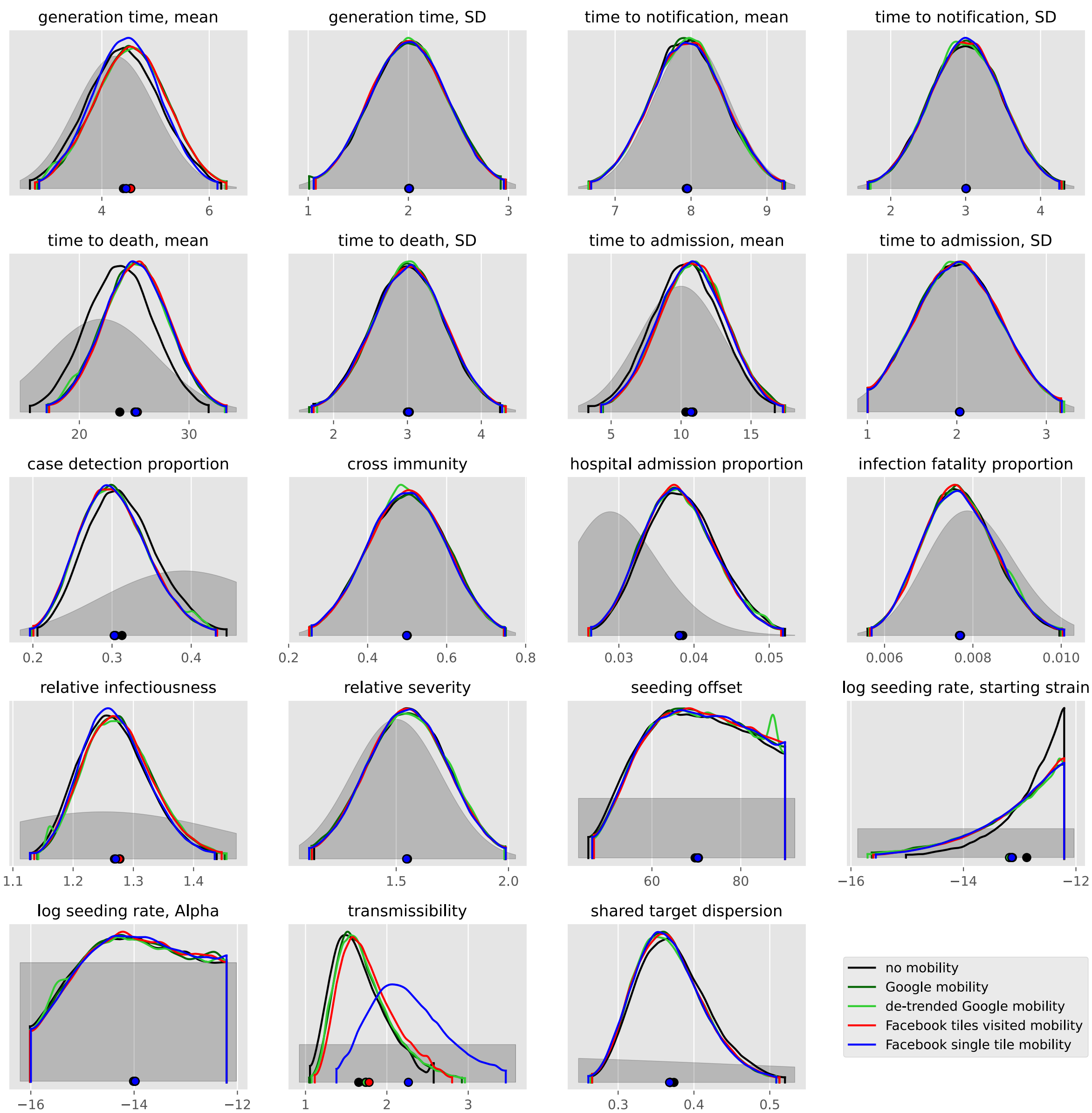

generation time, mean
generation time, SD
time to notification, mean
time to notification, SD
time to death, mean
time to death, SD
time to admission, mean
time to admission, SD
case detection proportion
cross immunity
hospital admission proportion
infection fatality proportion
relative infectiousness
relative severity
seeding offset
log seeding rate, starting strain
log seeding rate, Alpha
transmissibility
shared target dispersion
no mobility
Google mobility
de-trended Google mobility
Facebook tiles visited mobility
Facebook single tile mobility

Hungary

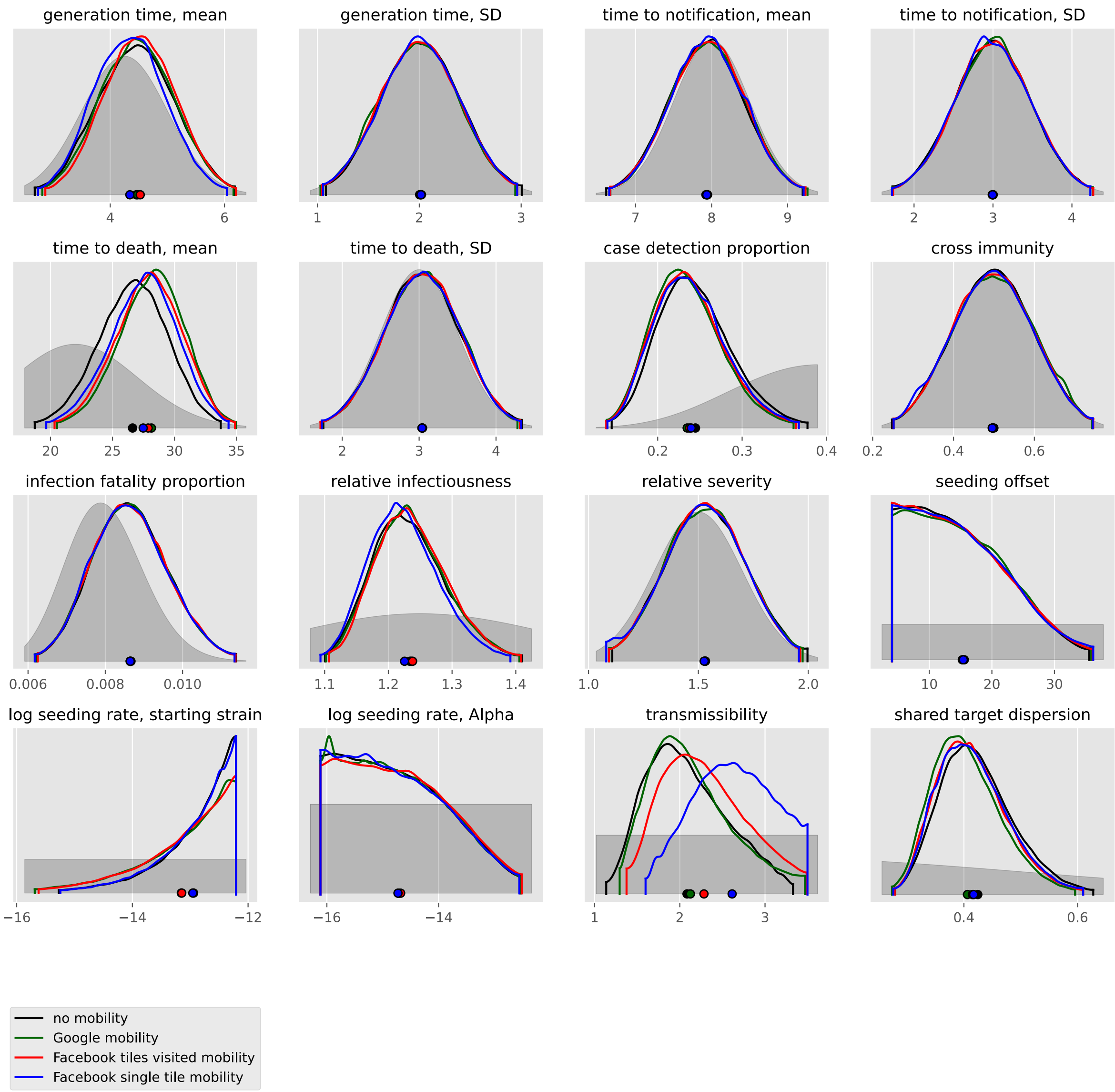

generation time, mean
generation time, SD
time to notification, mean
time to notification, SD
time to death, mean
time to death, SD
case detection proportion
cross immunity
infection fatality proportion
relative infectiousness
relative severity
seeding offset
log seeding rate, starting strain
log seeding rate, Alpha
transmissibility
shared target dispersion
no mobility
Google mobility
Facebook tiles visited mobility
Facebook single tile mobility

Ireland

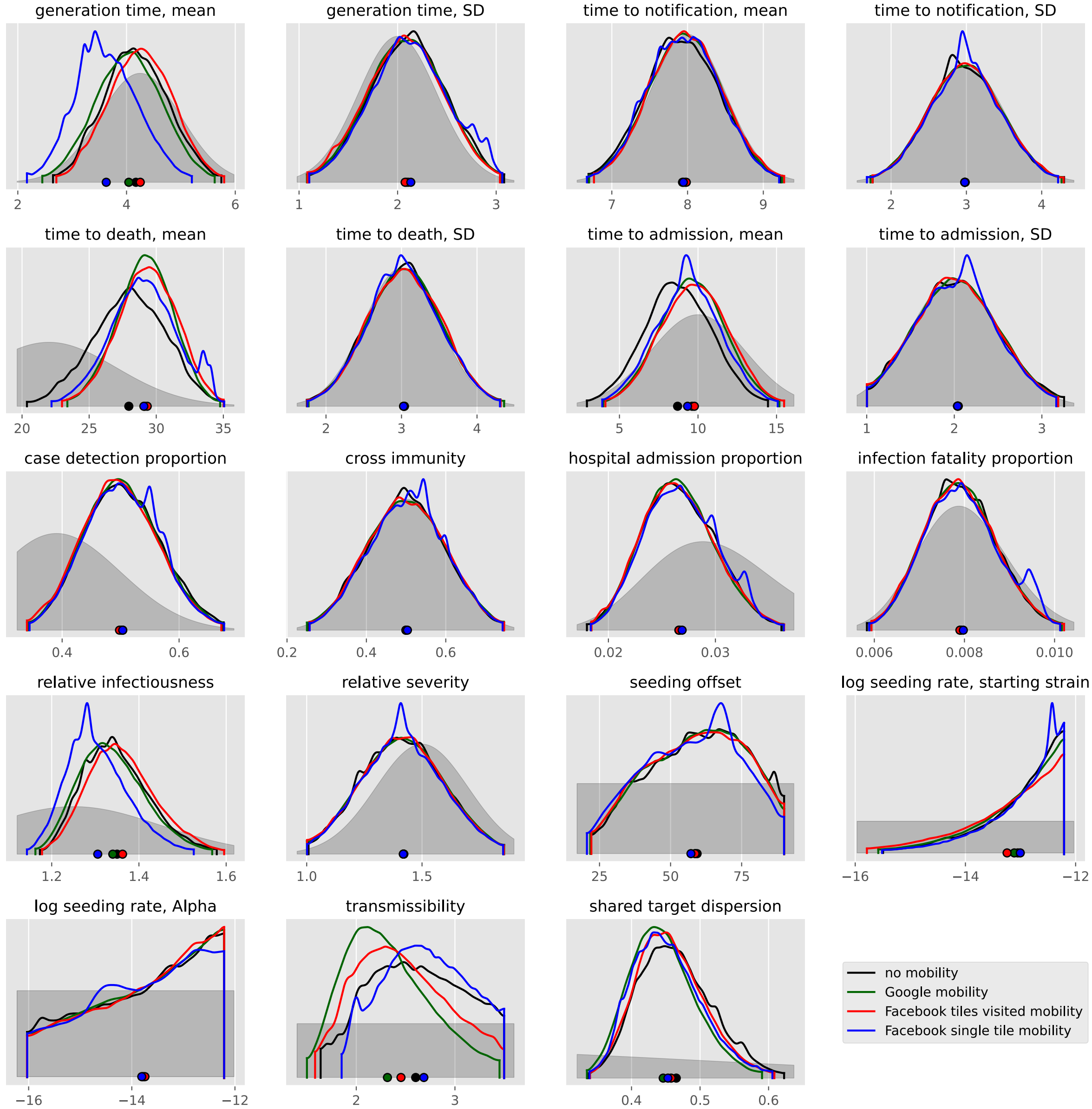

generation time, mean
generation time, SD
time to notification, mean
time to notification, SD
time to death, mean
time to death, SD
time to admission, mean
time to admission, SD
case detection proportion
cross immunity
hospital admission proportion
infection fatality proportion
relative infectiousness
relative severity
seeding offset
log seeding rate, starting strain
log seeding rate, Alpha
transmissibility
shared target dispersion
no mobility
Google mobility
Facebook tiles visited mobility
Facebook single tile mobility

Italy

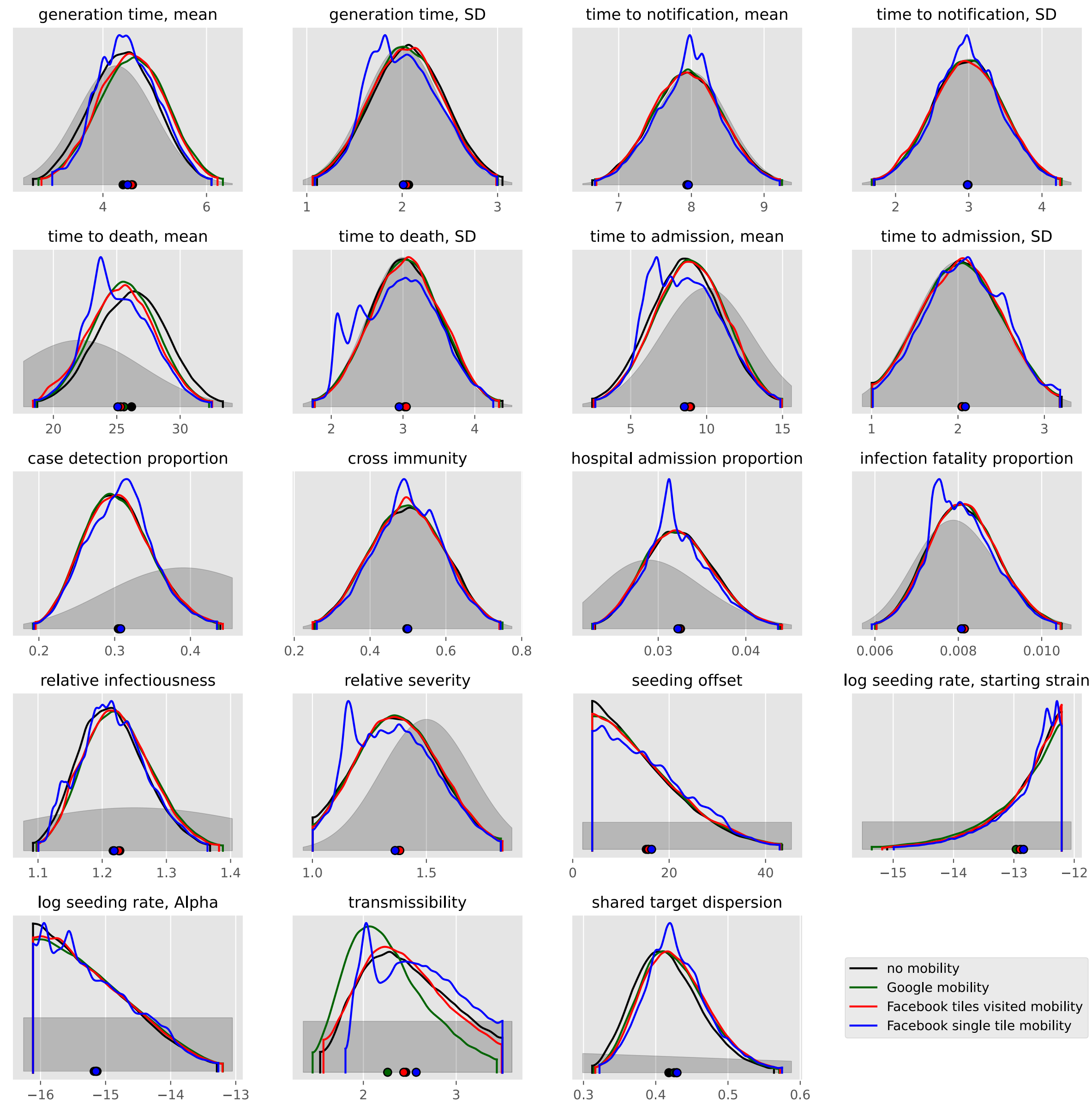
generation time, mean
generation time, SD
time to notification, mean
time to notification, SD
time to death, mean
time to death, SD
time to admission, mean
time to admission, SD
case detection proportion
cross immunity
hospital admission proportion
infection fatality proportion
relative infectiousness
relative severity
seeding offset
log seeding rate, starting strain
log seeding rate, Alpha
transmissibility
shared target dispersion
no mobility
Google mobility
Facebook tiles visited mobility
Facebook single tile mobility

Latvia

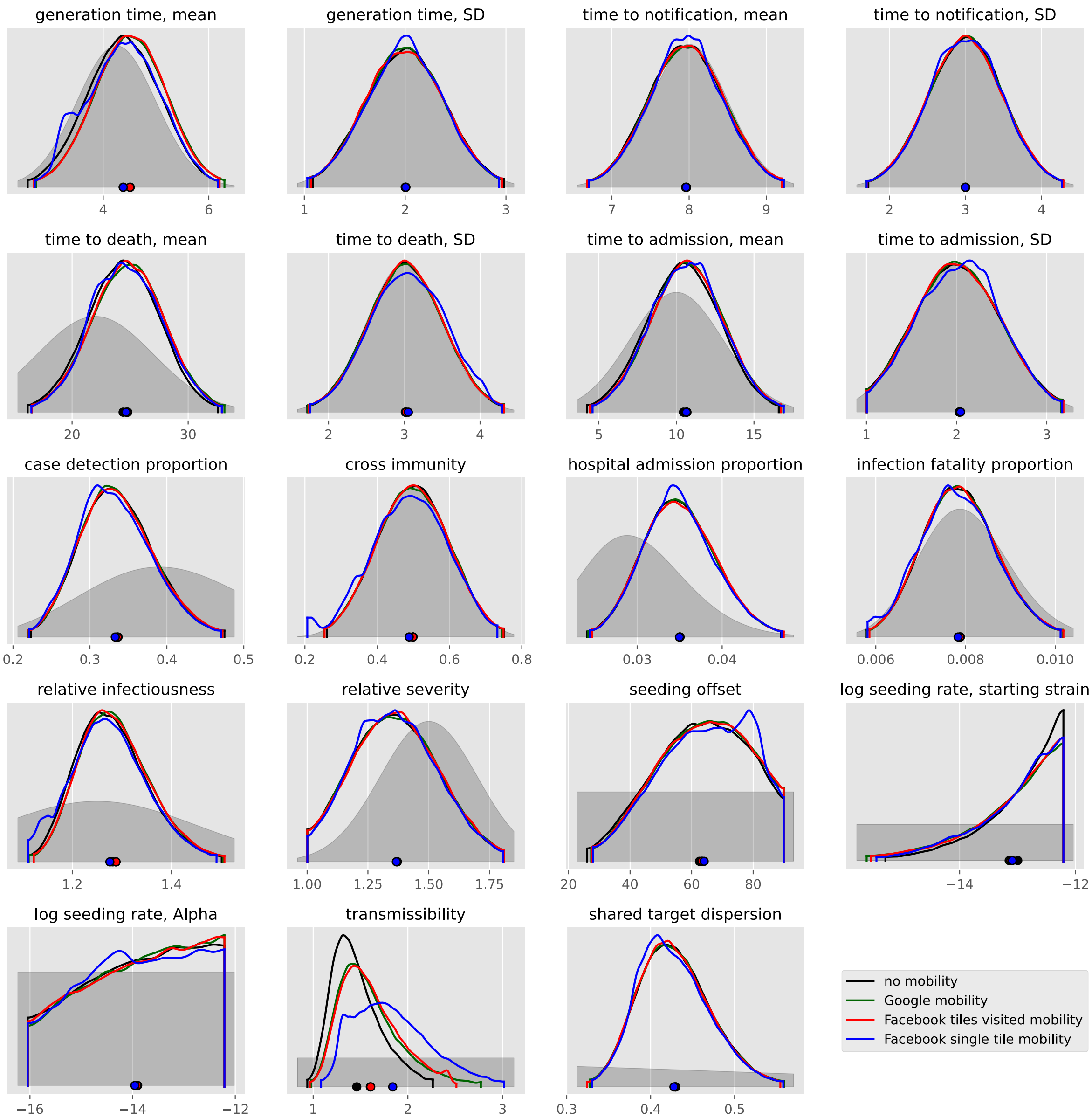

generation time, mean
generation time, SD
time to notification, mean
time to notification, SD
time to death, mean
time to death, SD
time to admission, mean
time to admission, SD
case detection proportion
cross immunity
hospital admission proportion
infection fatality proportion
relative infectiousness
relative severity
seeding offset
log seeding rate, starting strain
log seeding rate, Alpha
transmissibility
shared target dispersion
no mobility
Google mobility
Facebook tiles visited mobility
Facebook single tile mobility

Lithuania

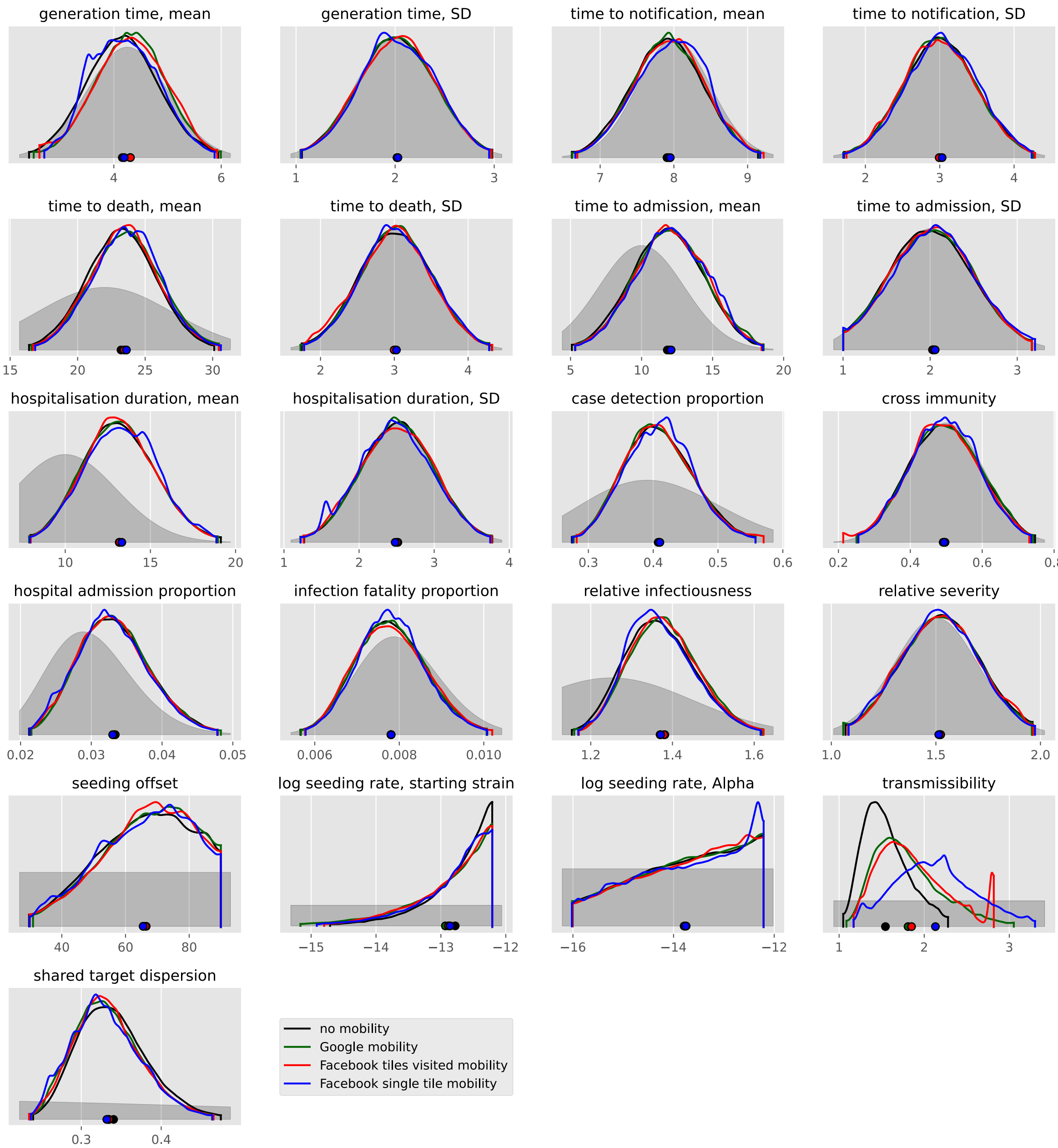
generation time, mean
4
6
generation time, SD
1
2
3
time to notification, mean
7
8
9
time to notification, SD
2
3
4
time to death, mean
15
20
25
30
time to death, SD
2
3
4
time to admission, mean
5
10
15
20
time to admission, SD
1
2
3
hospitalisation duration, mean
10
15
20
hospitalisation duration, SD
1
2
3
4
case detection proportion
0.3
0.4
0.5
0.6
cross immunity
0.2
0.4
0.6
0.8
hospital admission proportion
0.02
0.03
0.04
0.05
infection fatality proportion
0.006
0.008
0.010
relative infectiousness
1.2
1.4
1.6
relative severity
1.0
1.5
2.0
seeding offset
40
60
80
log seeding rate, starting strain
−15
−14
−13
−12
log seeding rate, Alpha
−16
−14
−12
transmissibility
1
2
3
shared target dispersion
0.3
0.4
no mobility
Google mobility
Facebook tiles visited mobility
Facebook single tile mobility

Luxembourg

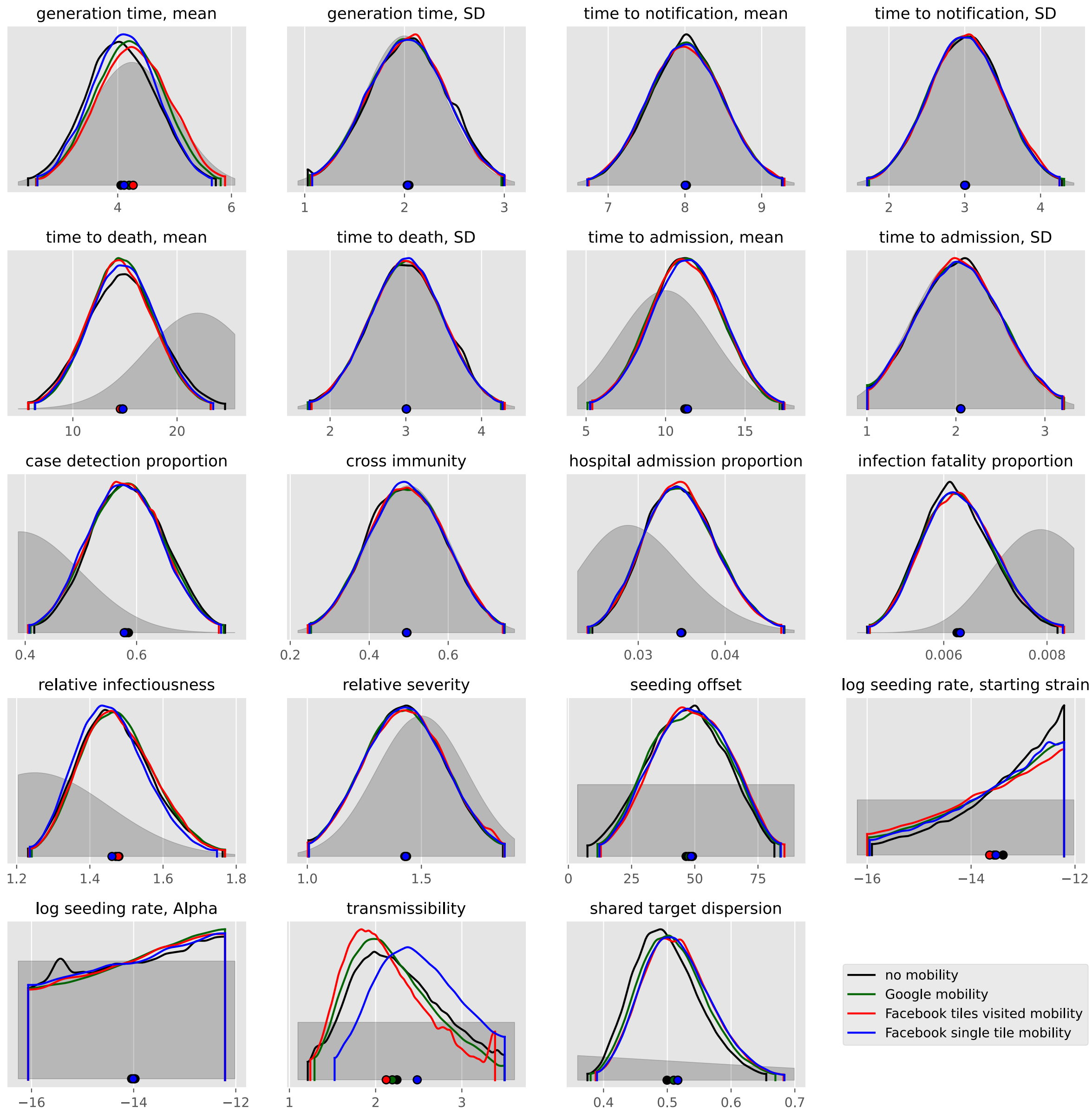

generation time, mean
4
6
generation time, SD
1
2
3
time to notification, mean
7
8
9
time to notification, SD
2
3
4
time to death, mean
10
20
time to death, SD
2
3
4
time to admission, mean
5
10
15
time to admission, SD
1
2
3
case detection proportion
0.4
0.6
cross immunity
0.2
0.4
0.6
hospital admission proportion
0.03
0.04
infection fatality proportion
0.006
0.008
relative infectiousness
1.2
1.4
1.6
1.8
relative severity
1.0
1.5
seeding offset
0
25
50
75
log seeding rate, starting strain
−16
−14
−12
log seeding rate, Alpha
−16
−14
−12
transmissibility
1
2
3
shared target dispersion
0.4
0.5
0.6
0.7
no mobility
Google mobility
Facebook tiles visited mobility
Facebook single tile mobility

Malta

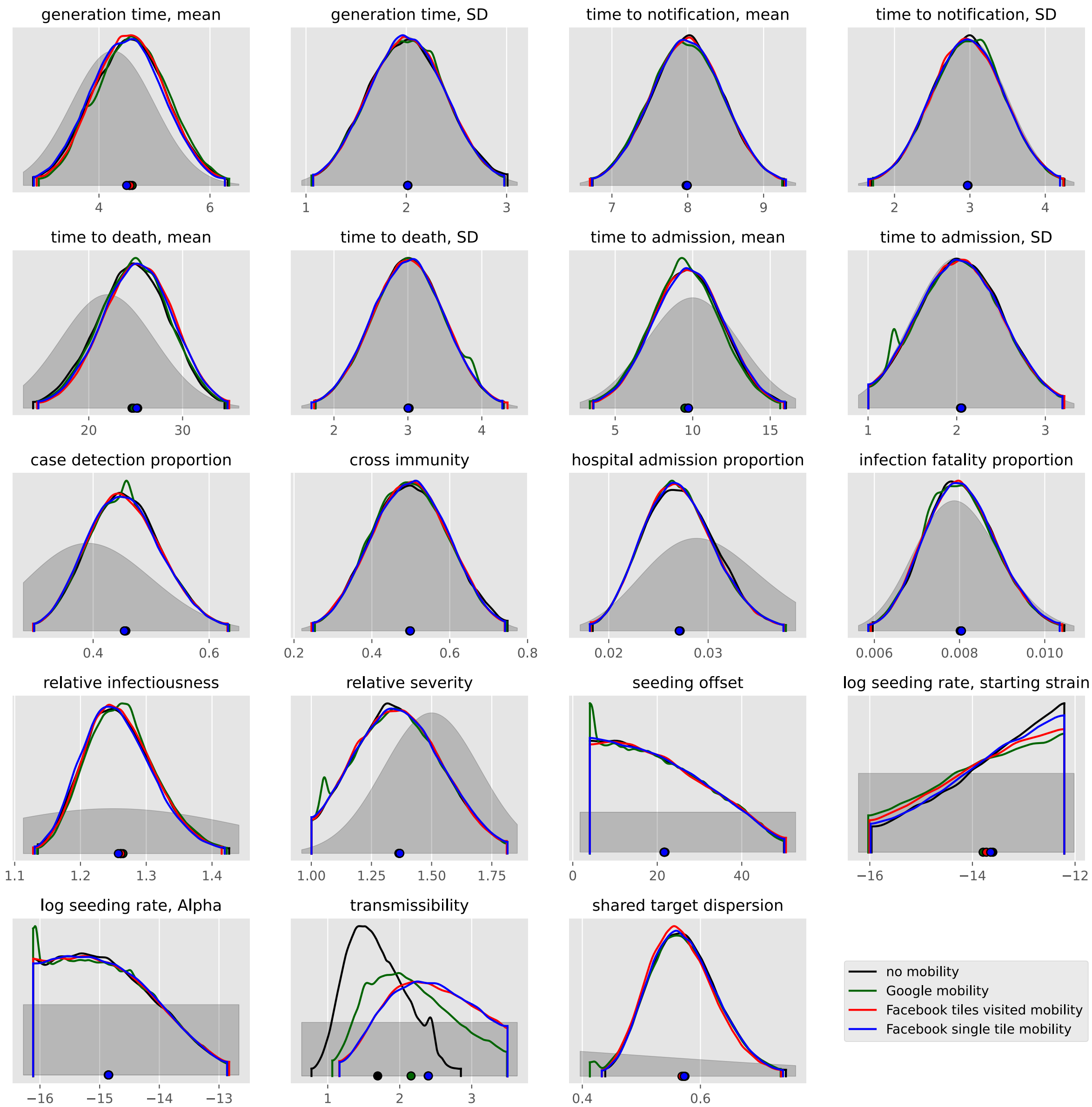

generation time, mean
generation time, SD
time to notification, mean
time to notification, SD
time to death, mean
time to death, SD
time to admission, mean
time to admission, SD
case detection proportion
cross immunity
hospital admission proportion
infection fatality proportion
relative infectiousness
relative severity
seeding offset
log seeding rate, starting strain
log seeding rate, Alpha
transmissibility
shared target dispersion
no mobility
Google mobility
Facebook tiles visited mobility
Facebook single tile mobility

Moldova, Republic of

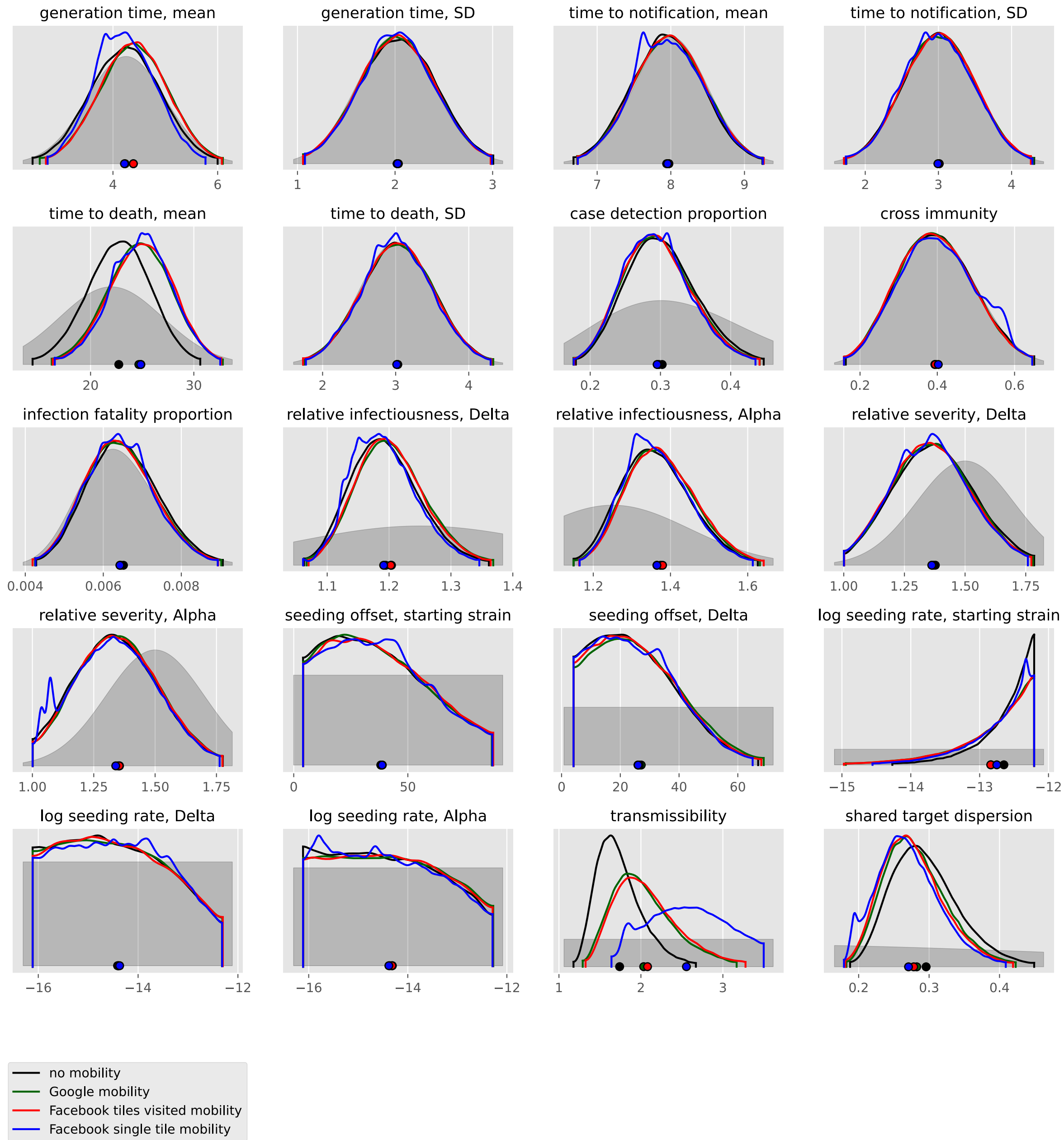

Netherlands

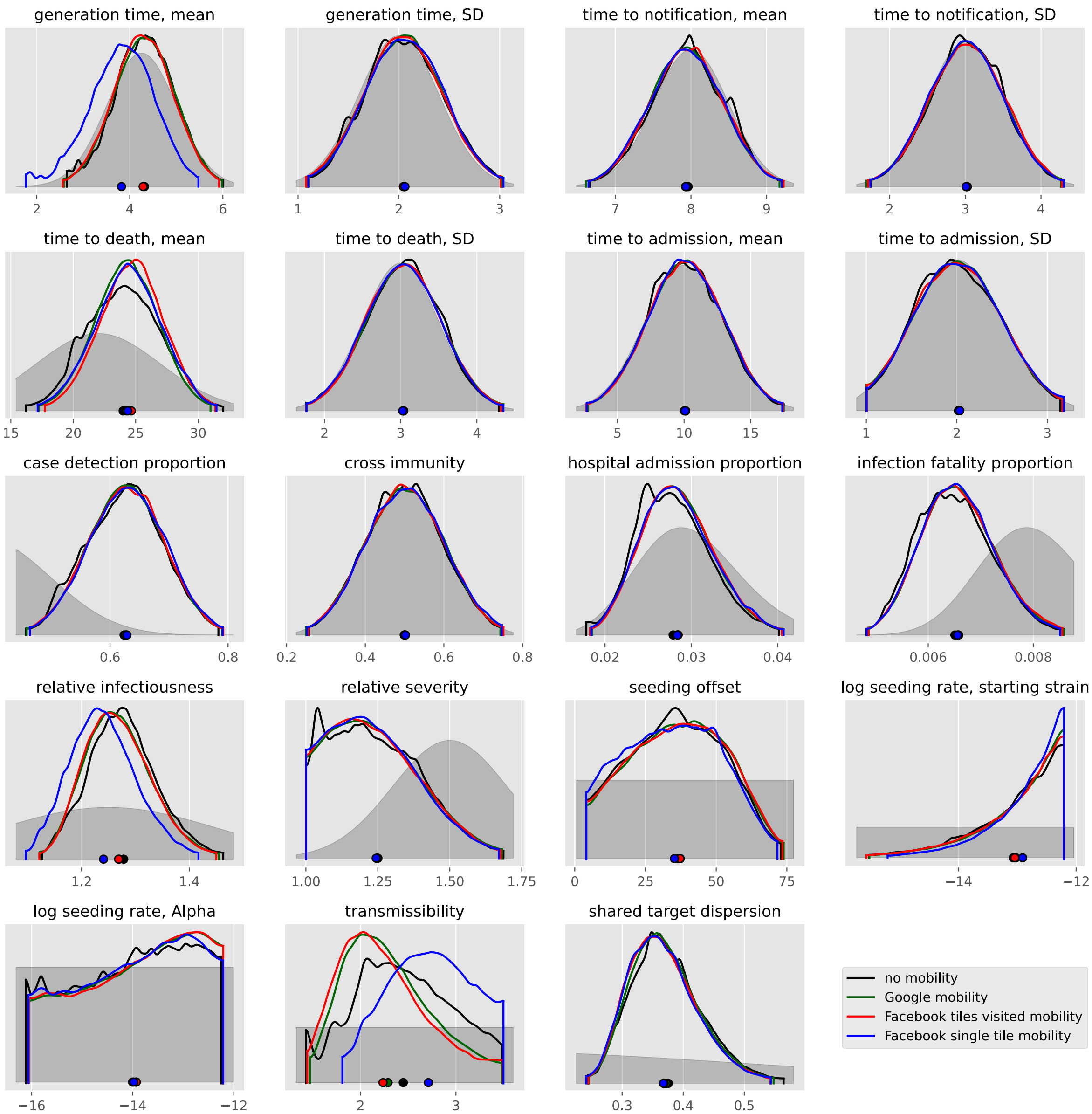

North Macedonia

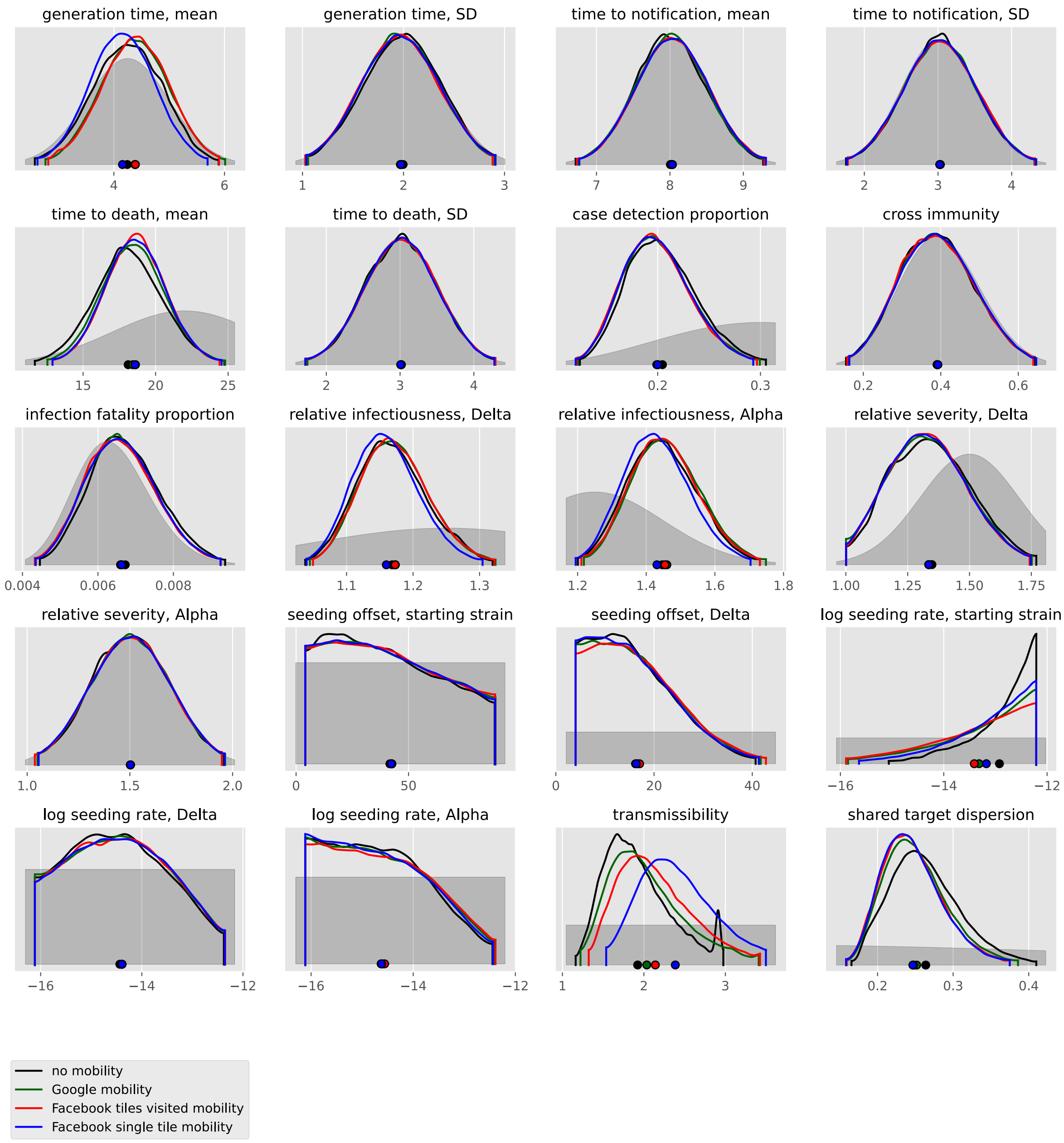

Norway

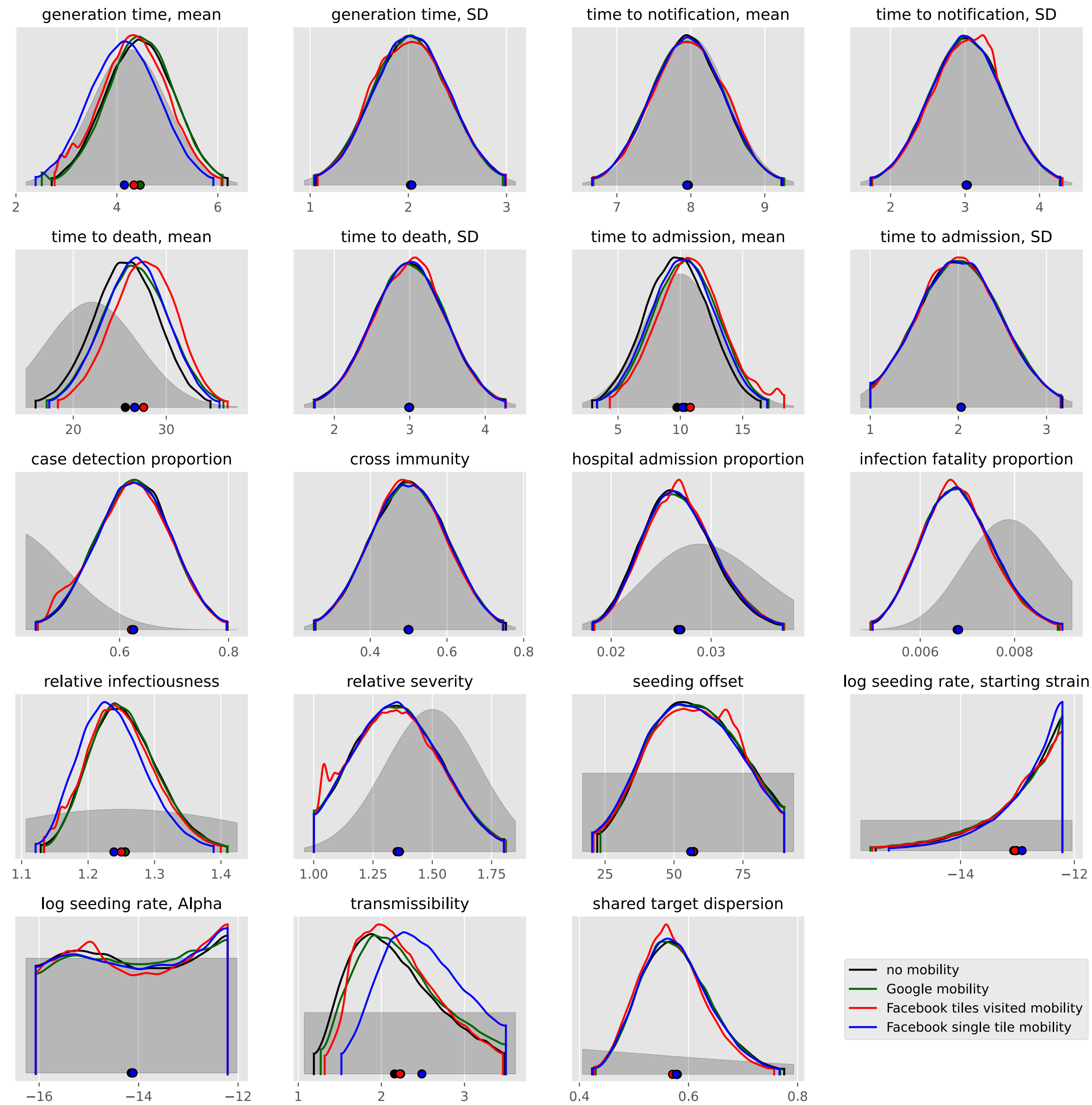
generation time, mean
generation time, SD
time to notification, mean
time to notification, SD
time to death, mean
time to death, SD
time to admission, mean
time to admission, SD
case detection proportion
cross immunity
hospital admission proportion
infection fatality proportion
relative infectiousness
relative severity
seeding offset
log seeding rate, starting strain
log seeding rate, Alpha
transmissibility
shared target dispersion
no mobility
Google mobility
Facebook tiles visited mobility
Facebook single tile mobility

Poland

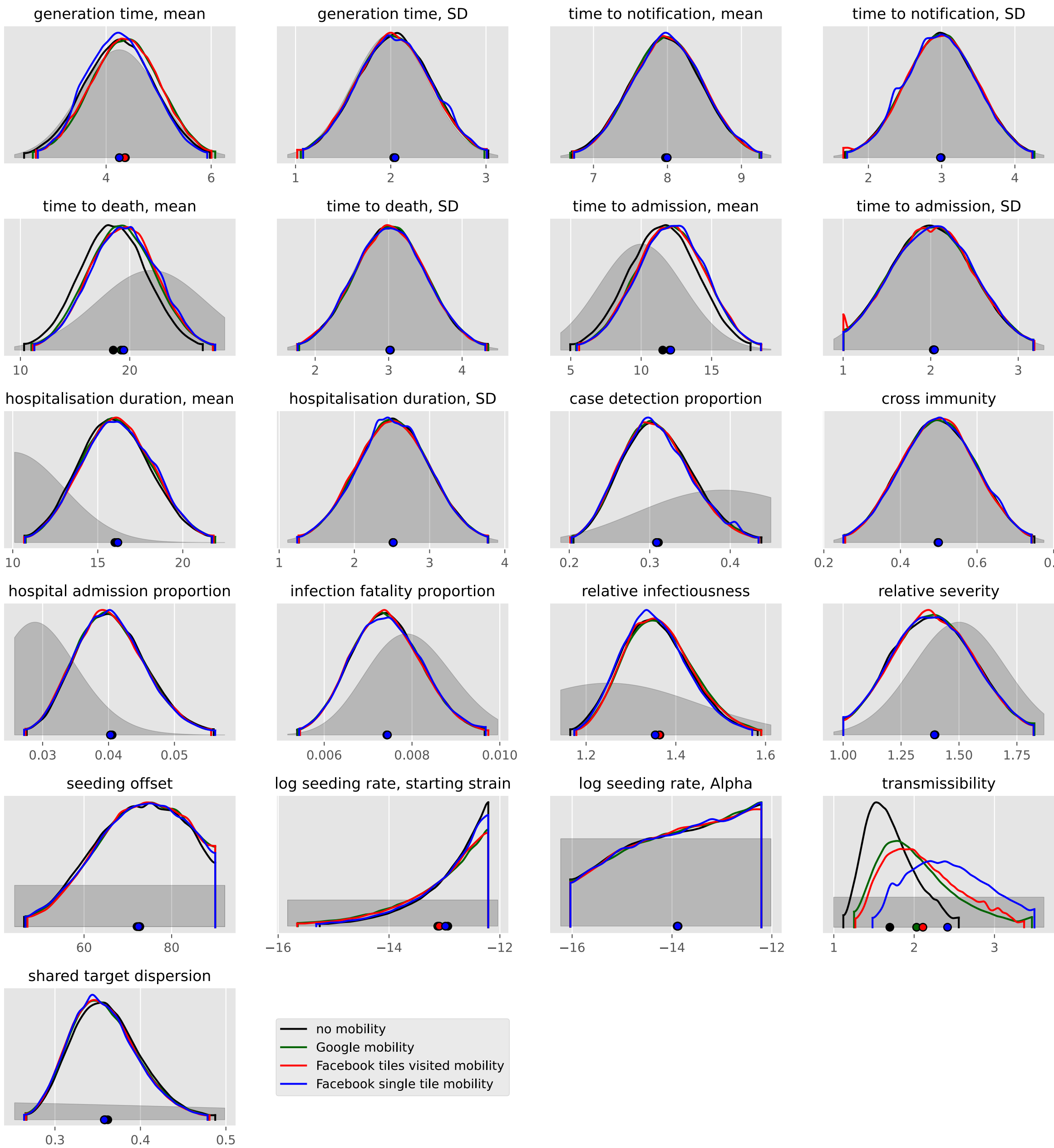
generation time, mean
generation time, SD
time to notification, mean
time to notification, SD
time to death, mean
time to death, SD
time to admission, mean
time to admission, SD
hospitalisation duration, mean
hospitalisation duration, SD
case detection proportion
cross immunity
hospital admission proportion
infection fatality proportion
relative infectiousness
relative severity
seeding offset
log seeding rate, starting strain
log seeding rate, Alpha
transmissibility
shared target dispersion
no mobility
Google mobility
Facebook tiles visited mobility
Facebook single tile mobility

Portugal

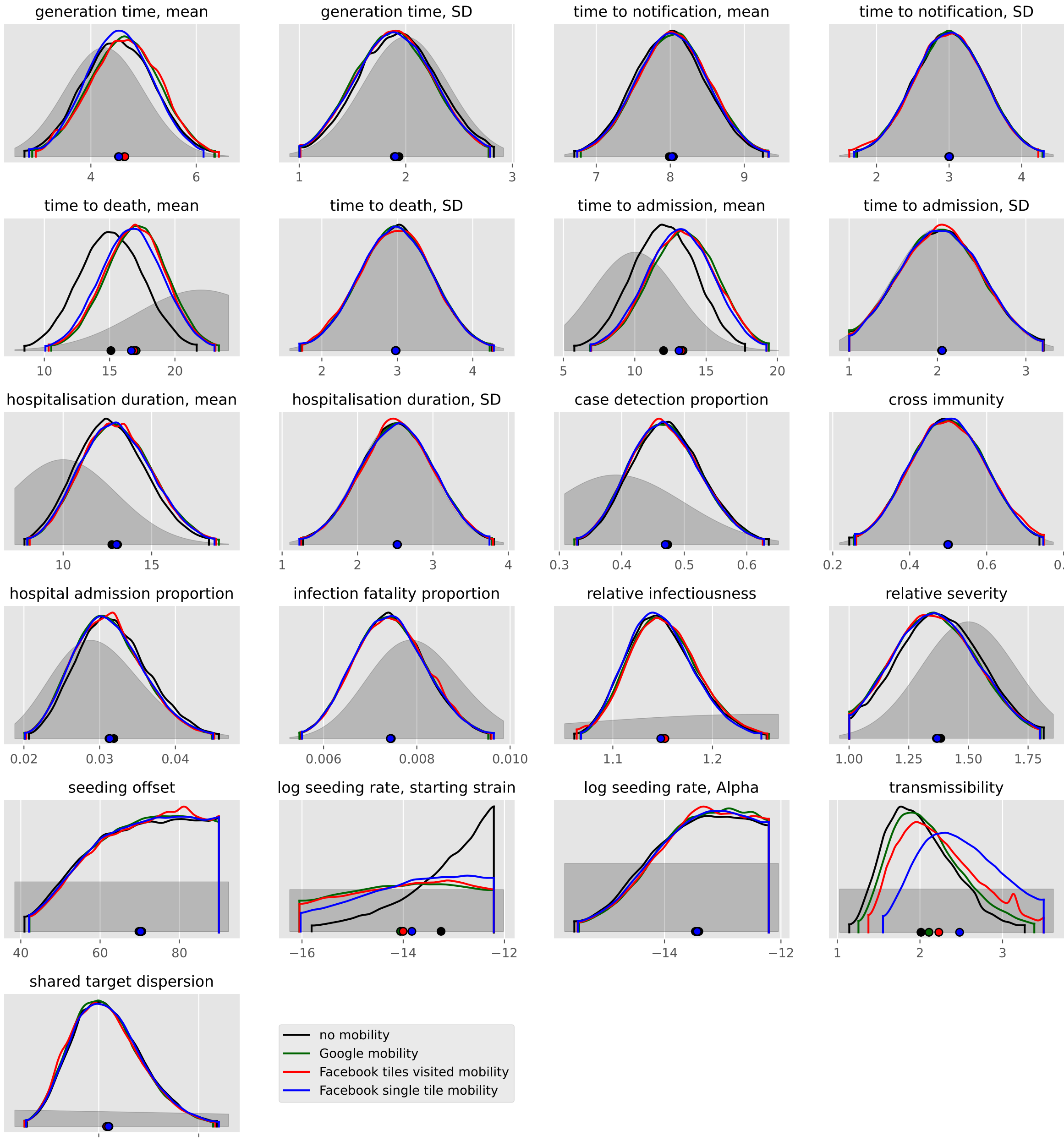

generation time, mean
4
6
generation time, SD
1
2
3
time to notification, mean
7
8
9
time to notification, SD
2
3
4
time to death, mean
10
15
20
time to death, SD
2
3
4
time to admission, mean
5
10
15
20
time to admission, SD
1
2
3
hospitalisation duration, mean
10
15
hospitalisation duration, SD
1
2
3
4
case detection proportion
0.3
0.4
0.5
0.6
cross immunity
0.2
0.4
0.6
0.8
hospital admission proportion
0.02
0.03
0.04
infection fatality proportion
0.006
0.008
0.010
relative infectiousness
1.1
1.2
relative severity
1.00
1.25
1.50
1.75
seeding offset
40
60
80
log seeding rate, starting strain
−16
−14
−12
log seeding rate, Alpha
−14
−12
transmissibility
1
2
3
shared target dispersion
0.3
0.4
no mobility
Google mobility
Facebook tiles visited mobility
Facebook single tile mobility

Romania

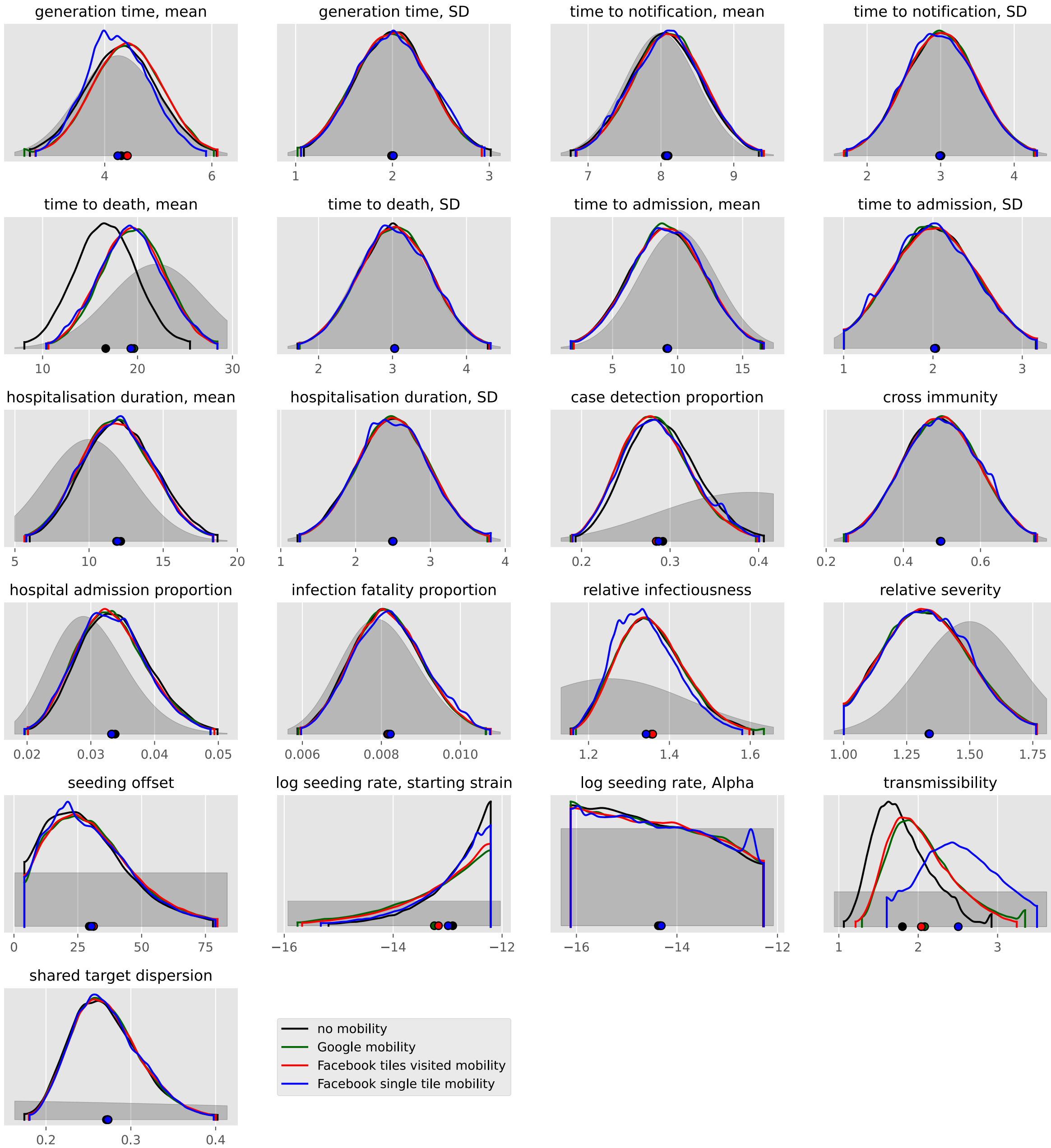

generation time, mean
generation time, SD
time to notification, mean
time to notification, SD
time to death, mean
time to death, SD
time to admission, mean
time to admission, SD
hospitalisation duration, mean
hospitalisation duration, SD
case detection proportion
cross immunity
hospital admission proportion
infection fatality proportion
relative infectiousness
relative severity
seeding offset
log seeding rate, starting strain
log seeding rate, Alpha
transmissibility
shared target dispersion
no mobility
Google mobility
Facebook tiles visited mobility
Facebook single tile mobility

Russian Federation

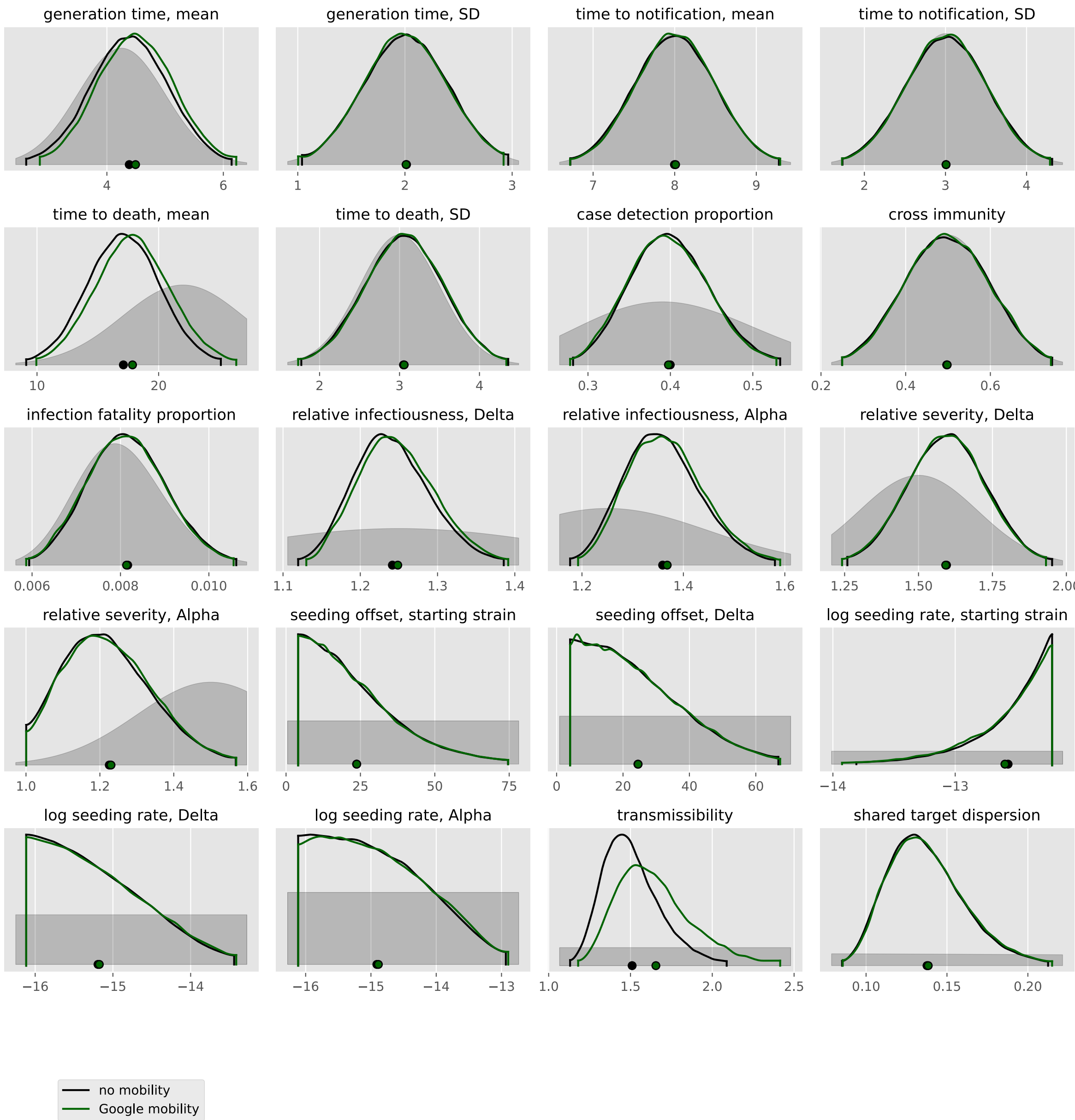

Serbia

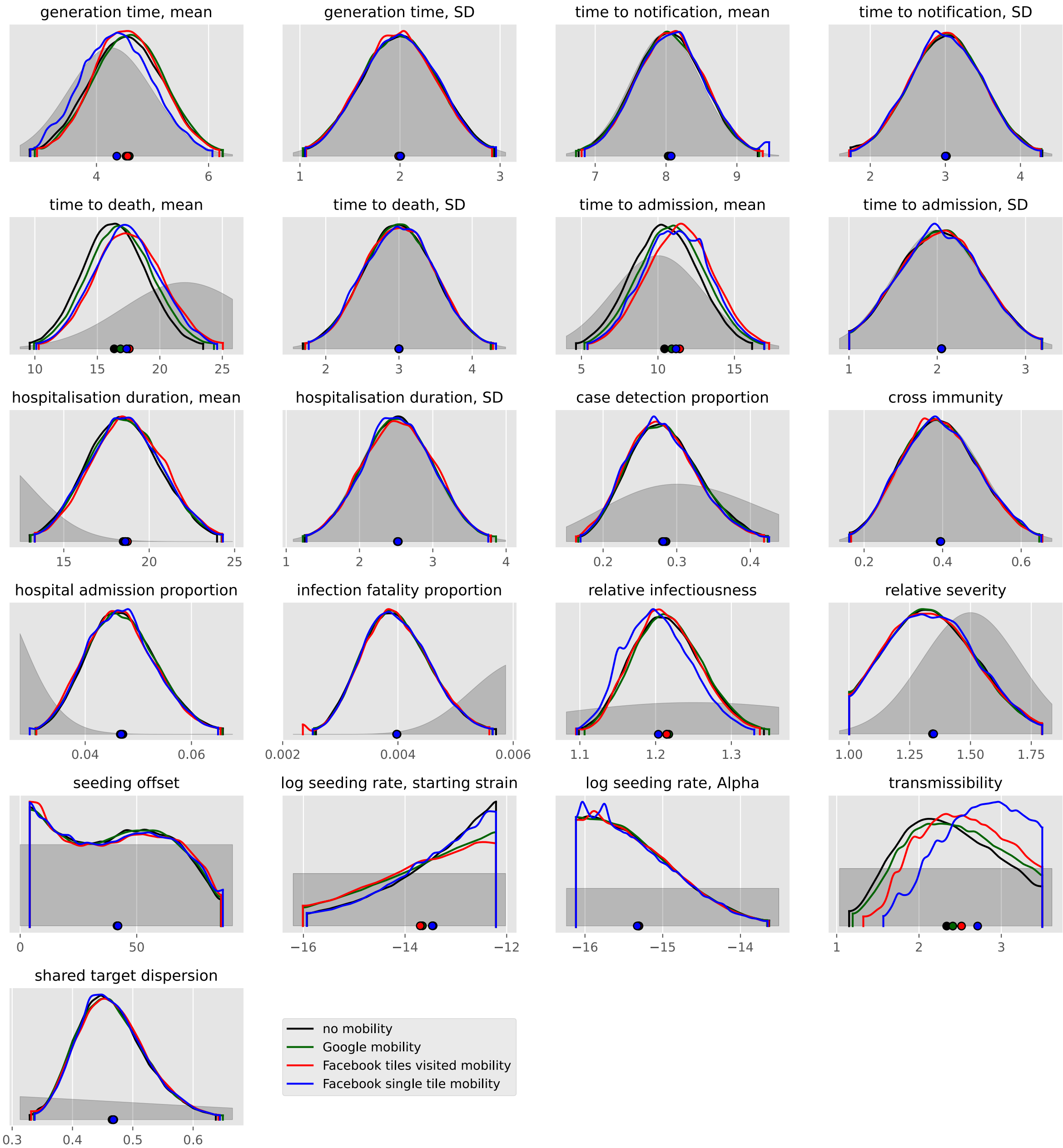
generation time, mean
generation time, SD
time to notification, mean
time to notification, SD
time to death, mean
time to death, SD
time to admission, mean
time to admission, SD
hospitalisation duration, mean
hospitalisation duration, SD
case detection proportion
cross immunity
hospital admission proportion
infection fatality proportion
relative infectiousness
relative severity
seeding offset
log seeding rate, starting strain
log seeding rate, Alpha
transmissibility
shared target dispersion
no mobility
Google mobility
Facebook tiles visited mobility
Facebook single tile mobility

Slovakia

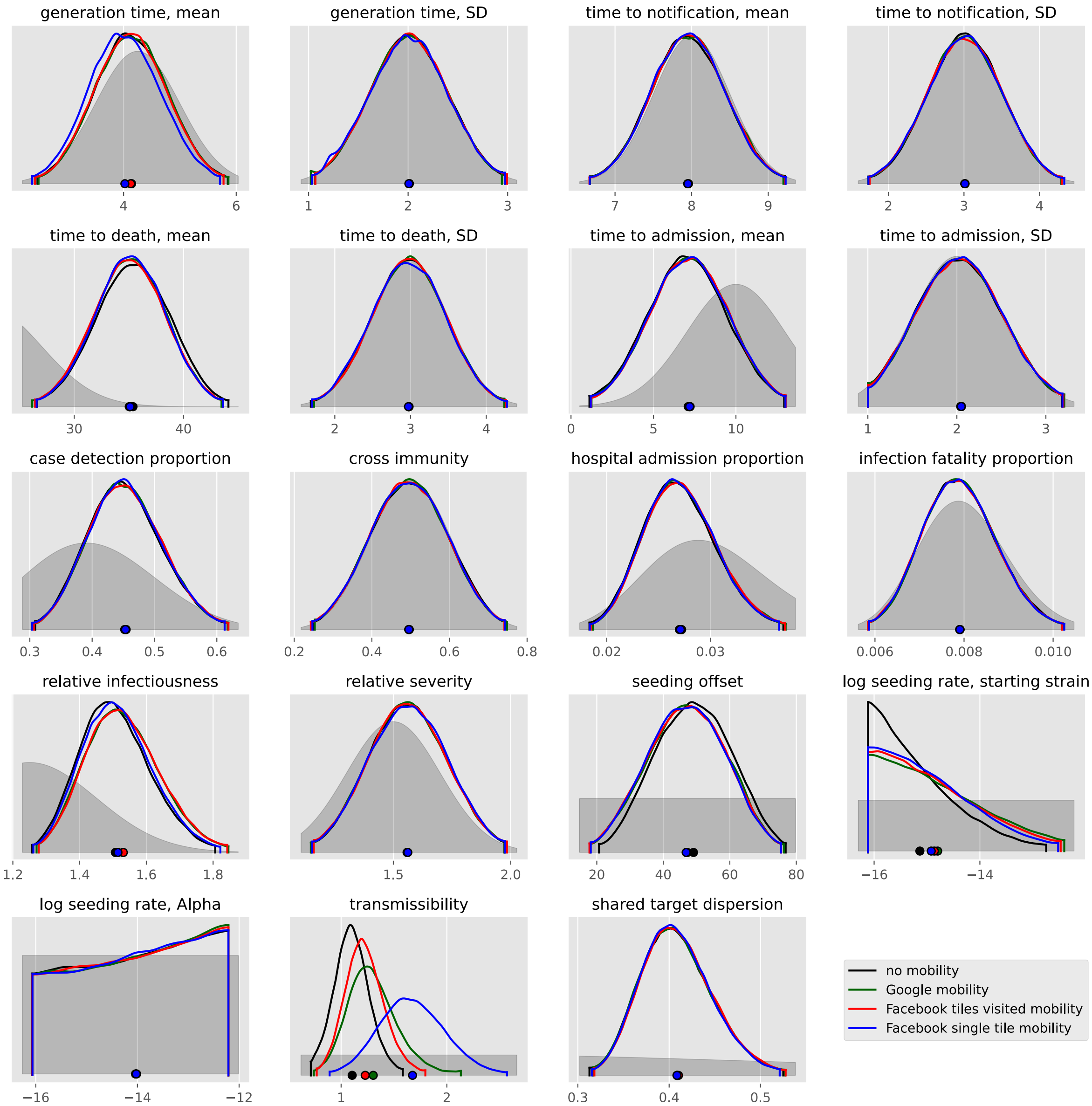

generation time, mean
generation time, SD
time to notification, mean
time to notification, SD
time to death, mean
time to death, SD
time to admission, mean
time to admission, SD
case detection proportion
cross immunity
hospital admission proportion
infection fatality proportion
relative infectiousness
relative severity
seeding offset
log seeding rate, starting strain
log seeding rate, Alpha
transmissibility
shared target dispersion
no mobility
Google mobility
Facebook tiles visited mobility
Facebook single tile mobility

Slovenia

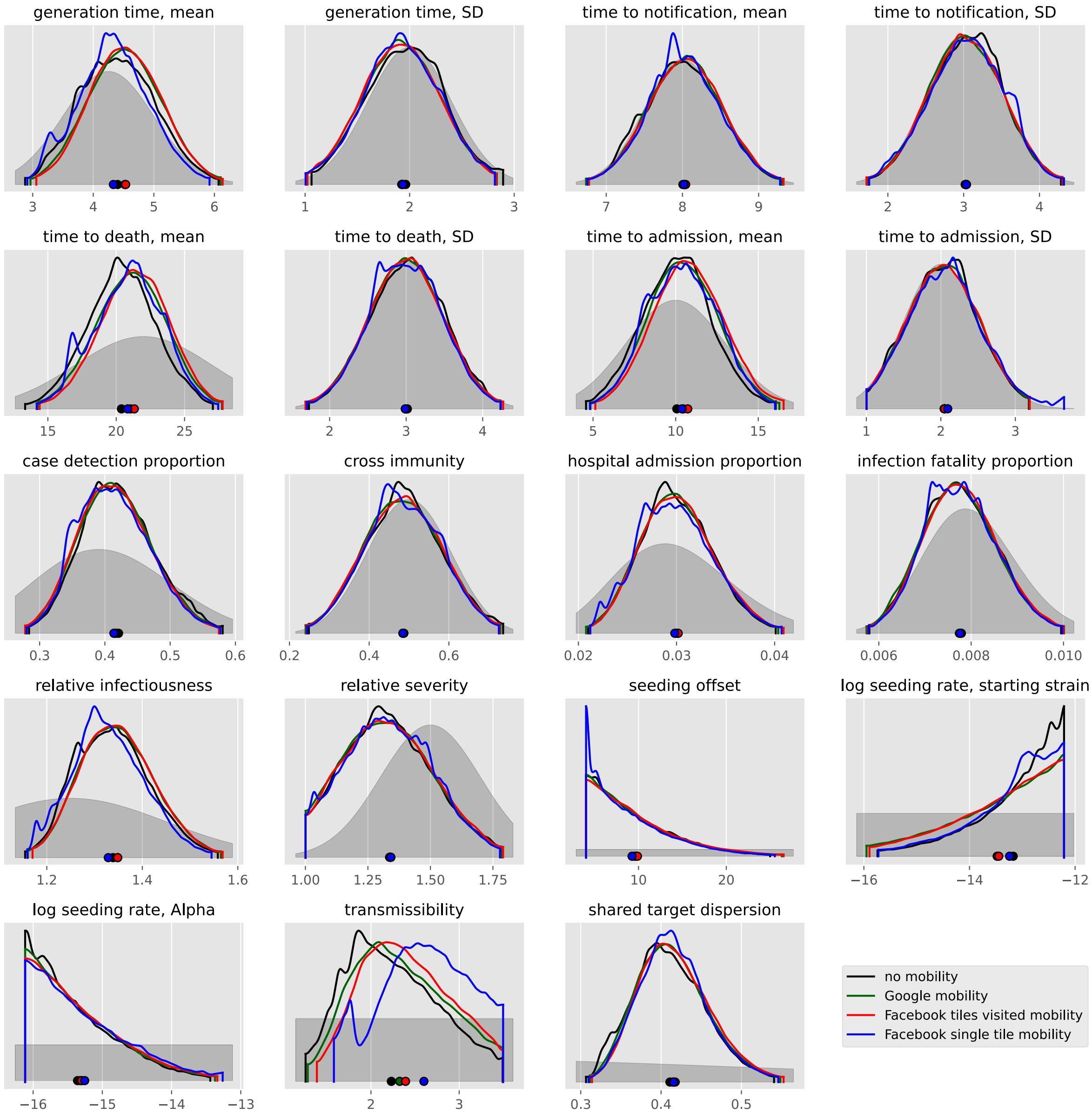
generation time, mean
3 4 5 6
generation time, SD
1 2 3
time to notification, mean
7 8 9
time to notification, SD
2 3 4
time to death, mean
15 20 25
time to death, SD
2 3 4
time to admission, mean
5 10 15
time to admission, SD
1 2 3
case detection proportion
0.3 0.4 0.5 0.6
cross immunity
0.2 0.4 0.6
hospital admission proportion
0.02 0.03 0.04
infection fatality proportion
0.006 0.008 0.010
relative infectiousness
1.2 1.4 1.6
relative severity
1.00 1.25 1.50 1.75
seeding offset
10 20
log seeding rate, starting strain
−16 −14 −12
log seeding rate, Alpha
−16 −15 −14 −13
transmissibility
2 3
shared target dispersion
0.3 0.4 0.5
no mobility
Google mobility
Facebook tiles visited mobility
Facebook single tile mobility

Spain

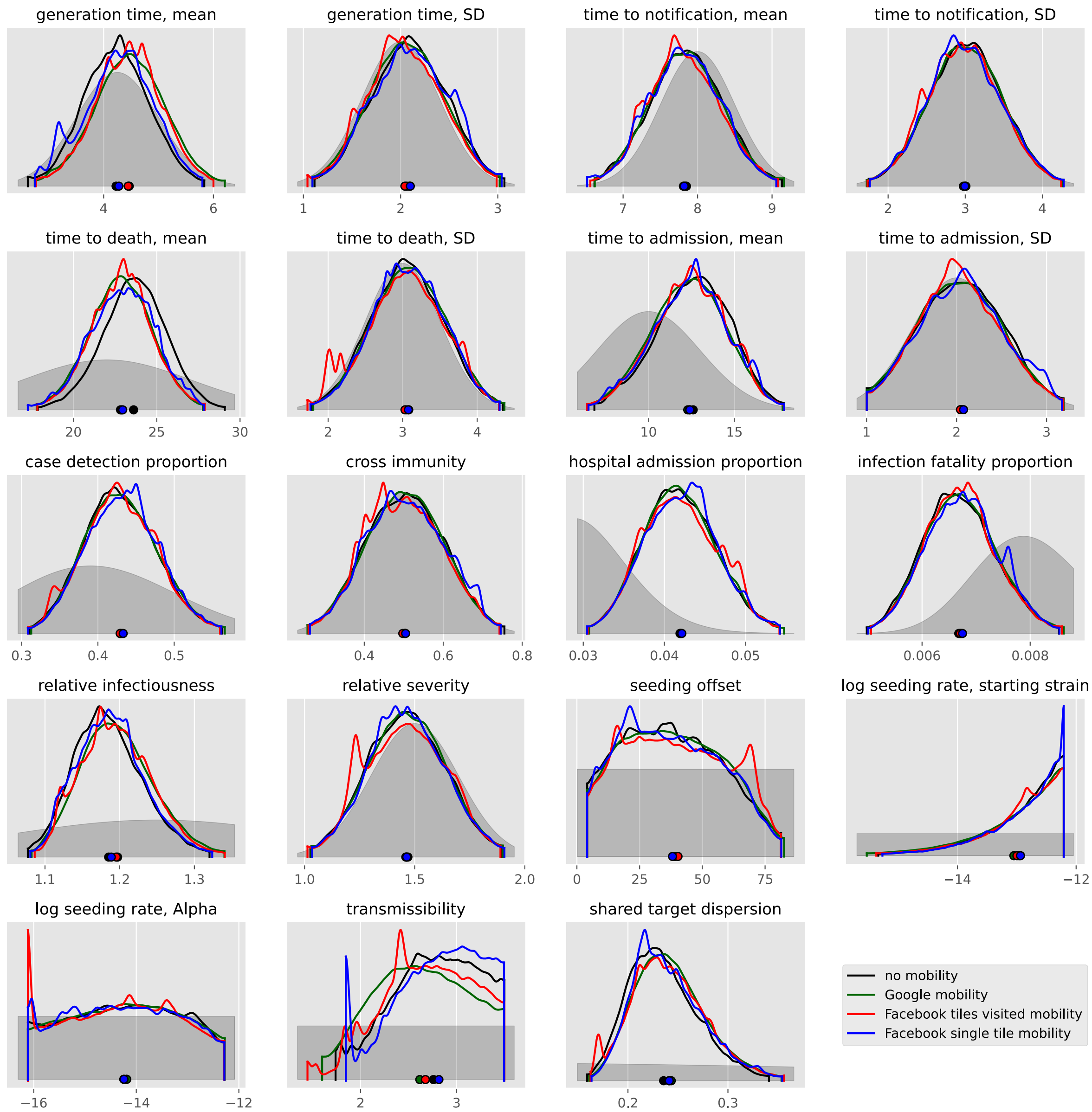

generation time, mean
generation time, SD
time to notification, mean
time to notification, SD
time to death, mean
time to death, SD
time to admission, mean
time to admission, SD
case detection proportion
cross immunity
hospital admission proportion
infection fatality proportion
relative infectiousness
relative severity
seeding offset
log seeding rate, starting strain
log seeding rate, Alpha
transmissibility
shared target dispersion
no mobility
Google mobility
Facebook tiles visited mobility
Facebook single tile mobility

Sweden

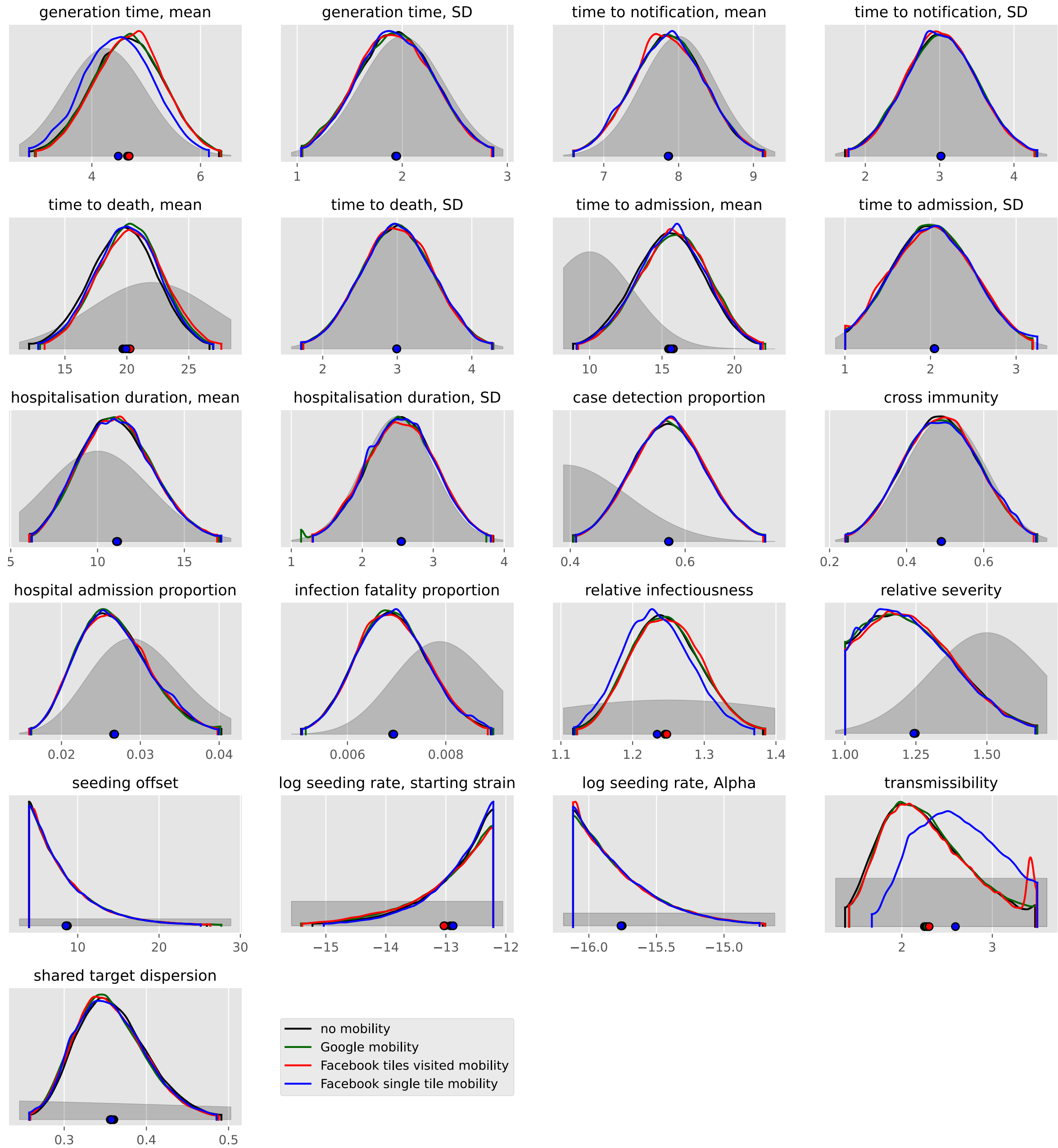
generation time, mean
generation time, SD
time to notification, mean
time to notification, SD
time to death, mean
time to death, SD
time to admission, mean
time to admission, SD
hospitalisation duration, mean
hospitalisation duration, SD
case detection proportion
cross immunity
hospital admission proportion
infection fatality proportion
relative infectiousness
relative severity
seeding offset
log seeding rate, starting strain
log seeding rate, Alpha
transmissibility
shared target dispersion
no mobility
Google mobility
Facebook tiles visited mobility
Facebook single tile mobility

Switzerland

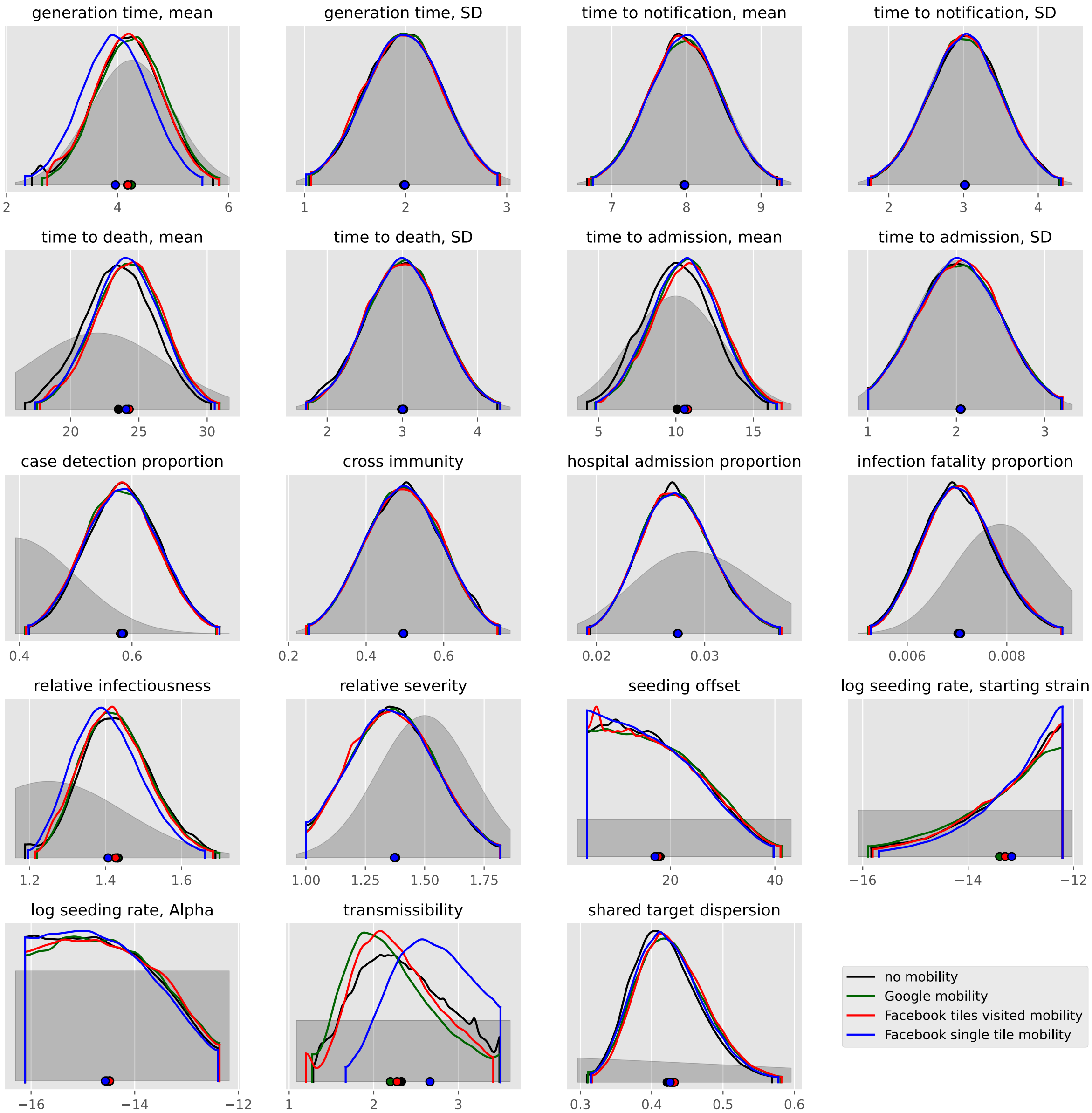

Ukraine

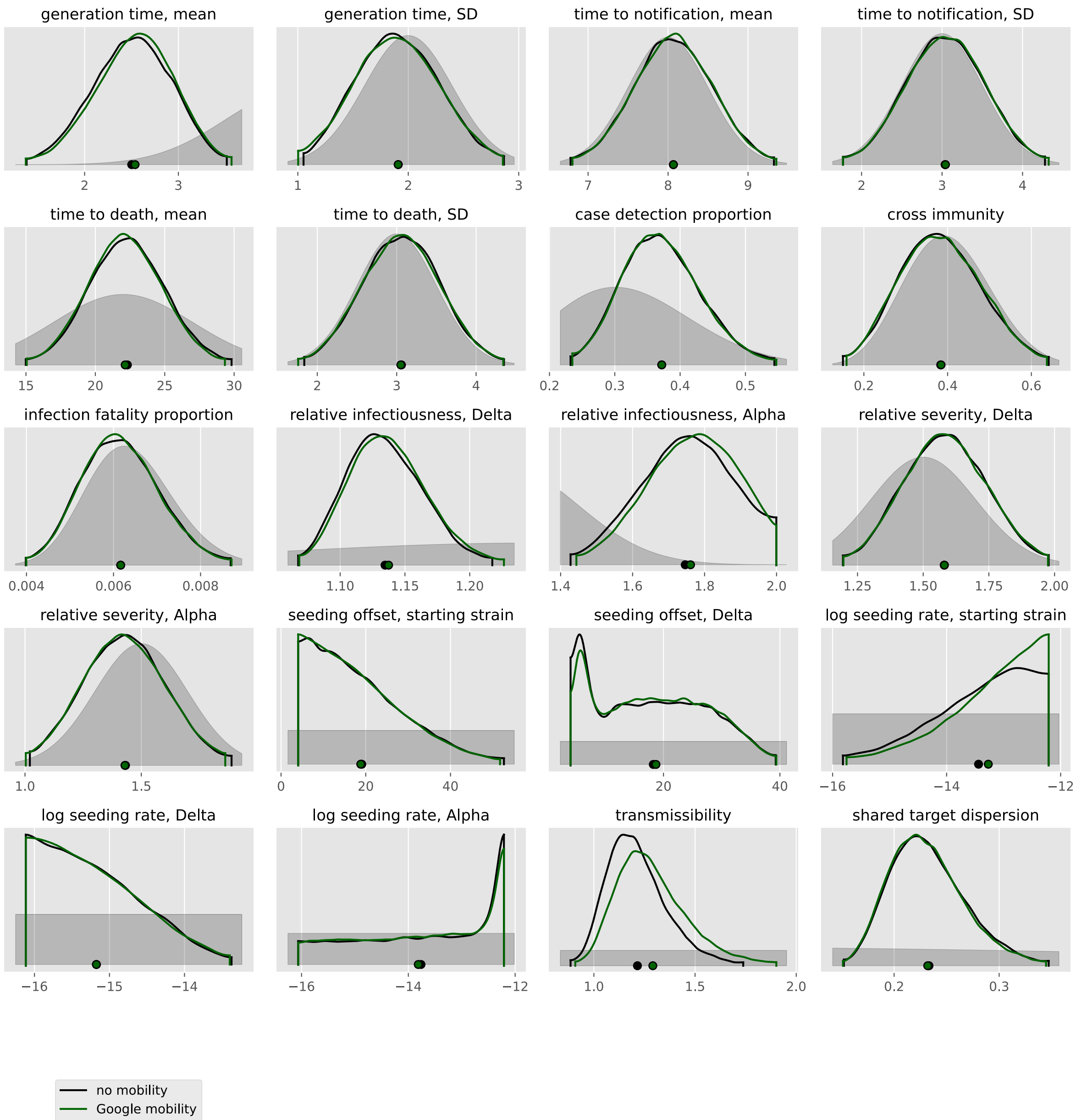

generation time, mean
generation time, SD
time to notification, mean
time to notification, SD
time to death, mean
time to death, SD
case detection proportion
cross immunity
infection fatality proportion
relative infectiousness, Delta
relative infectiousness, Alpha
relative severity, Delta
relative severity, Alpha
seeding offset, starting strain
seeding offset, Delta
log seeding rate, starting strain
log seeding rate, Delta
log seeding rate, Alpha
transmissibility
shared target dispersion
no mobility
Google mobility

United Kingdom

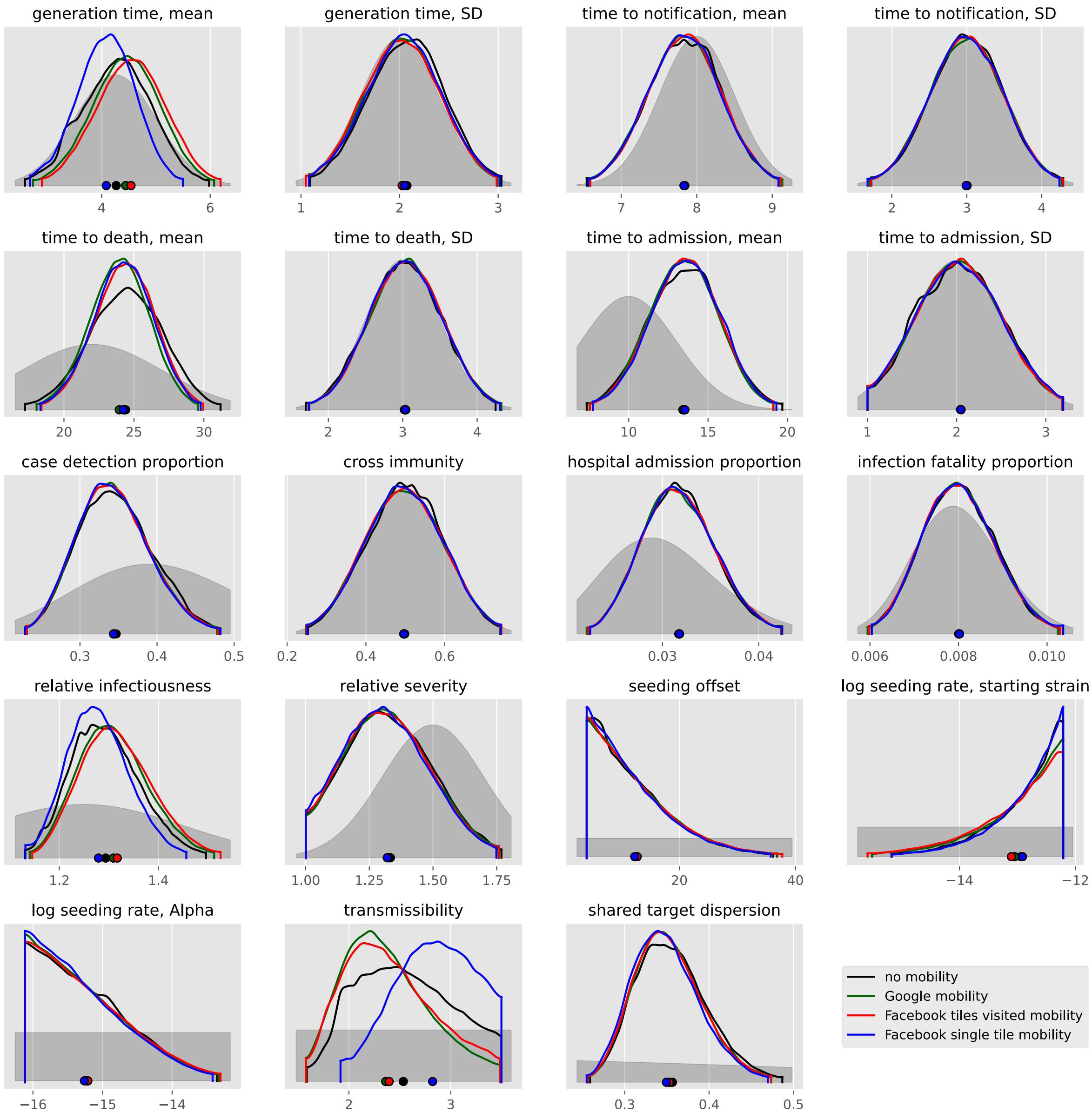

North America

Aruba

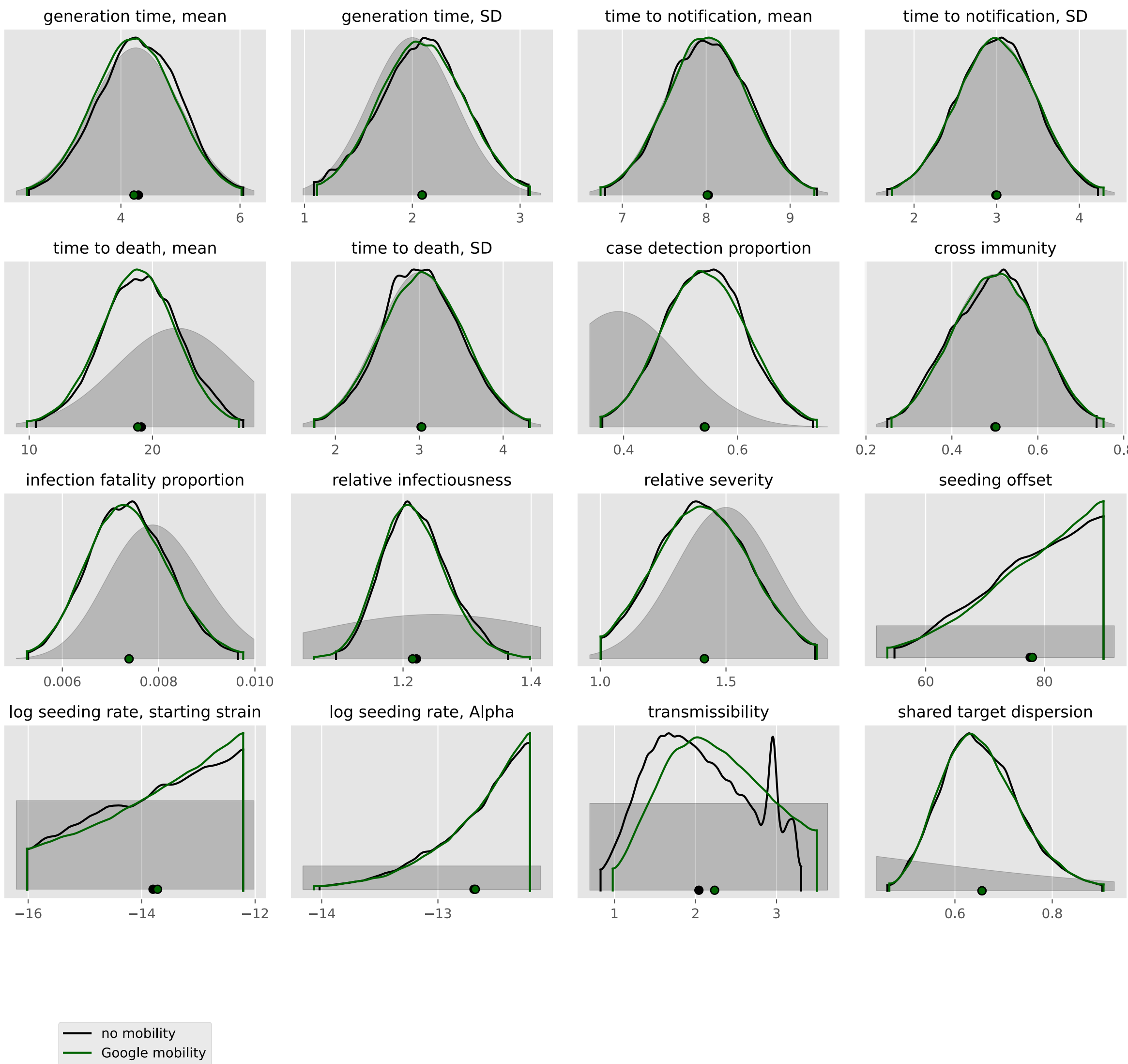

Bahamas

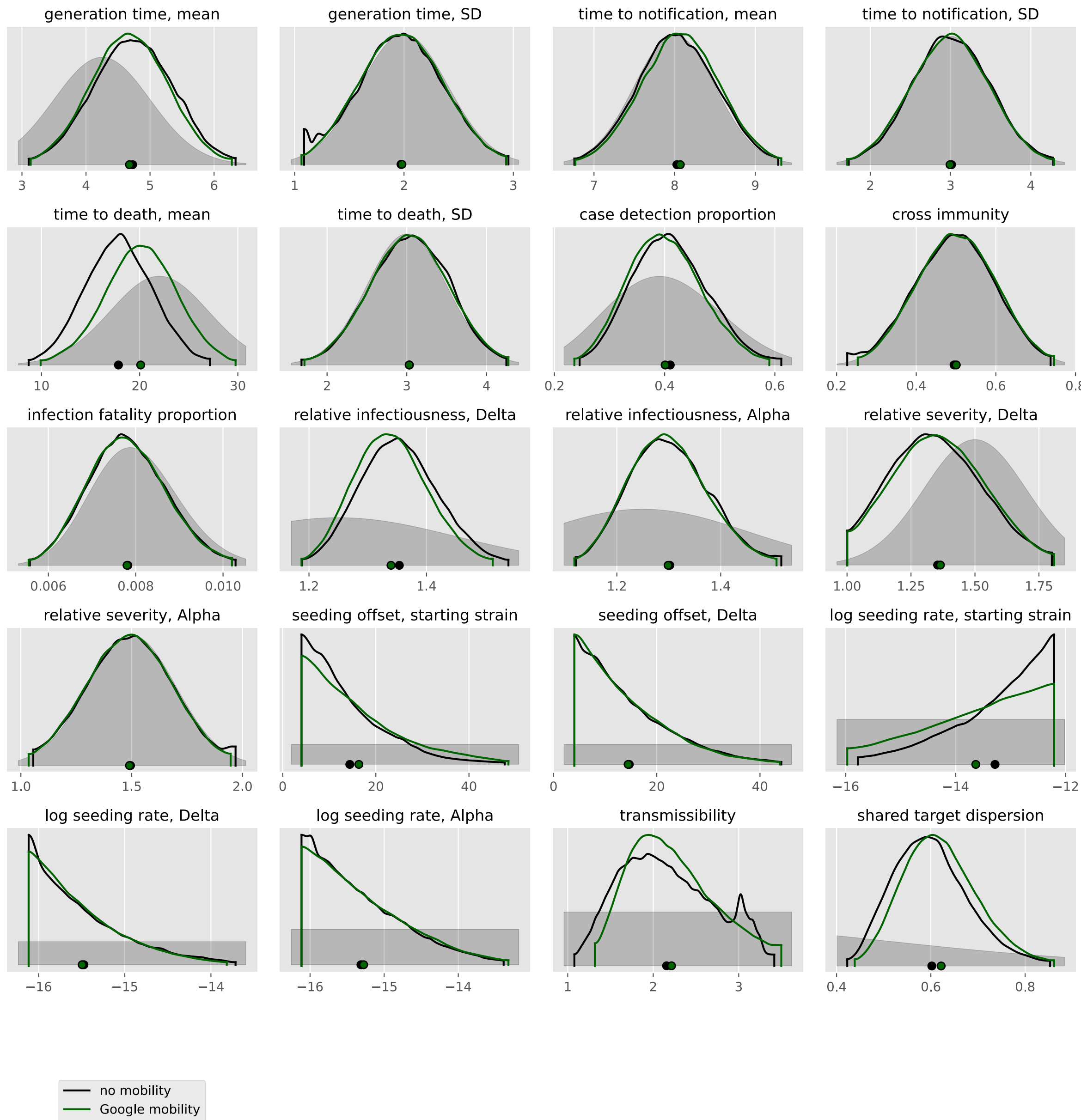

generation time, mean
generation time, SD
time to notification, mean
time to notification, SD
time to death, mean
time to death, SD
case detection proportion
cross immunity
infection fatality proportion
relative infectiousness, Delta
relative infectiousness, Alpha
relative severity, Delta
relative severity, Alpha
seeding offset, starting strain
seeding offset, Delta
log seeding rate, starting strain
log seeding rate, Delta
log seeding rate, Alpha
transmissibility
shared target dispersion
no mobility
Google mobility

Canada

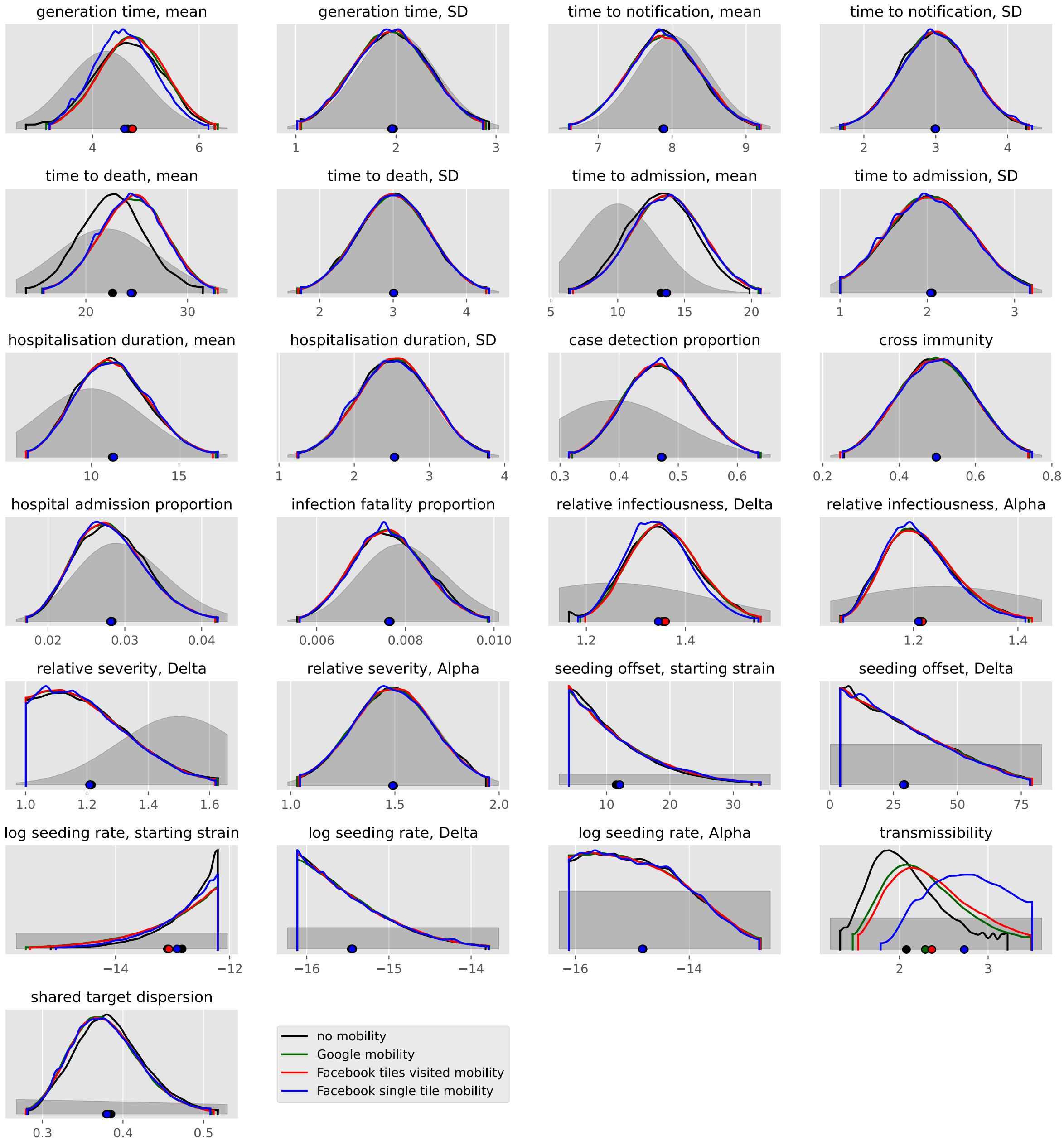
generation time, mean
generation time, SD
time to notification, mean
time to notification, SD
time to death, mean
time to death, SD
time to admission, mean
time to admission, SD
hospitalisation duration, mean
hospitalisation duration, SD
case detection proportion
cross immunity
hospital admission proportion
infection fatality proportion
relative infectiousness, Delta
relative infectiousness, Alpha
relative severity, Delta
relative severity, Alpha
seeding offset, starting strain
seeding offset, Delta
log seeding rate, starting strain
log seeding rate, Delta
log seeding rate, Alpha
transmissibility
shared target dispersion
no mobility
Google mobility
Facebook tiles visited mobility
Facebook single tile mobility

Costa Rica

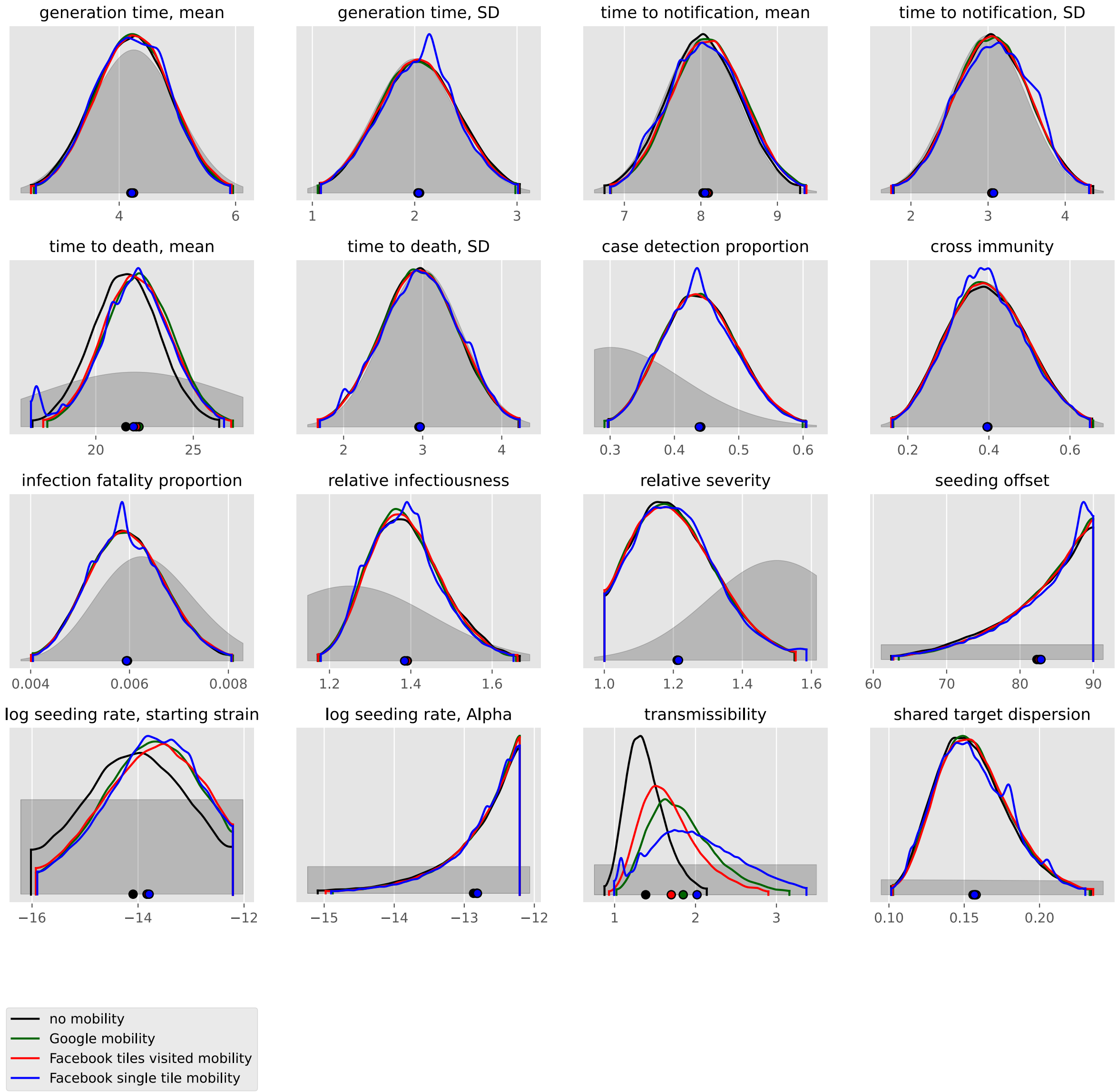

generation time, mean
generation time, SD
time to notification, mean
time to notification, SD
time to death, mean
time to death, SD
case detection proportion
cross immunity
infection fatality proportion
relative infectiousness
relative severity
seeding offset
log seeding rate, starting strain
log seeding rate, Alpha
transmissibility
shared target dispersion
no mobility
Google mobility
Facebook tiles visited mobility
Facebook single tile mobility

Dominican Republic

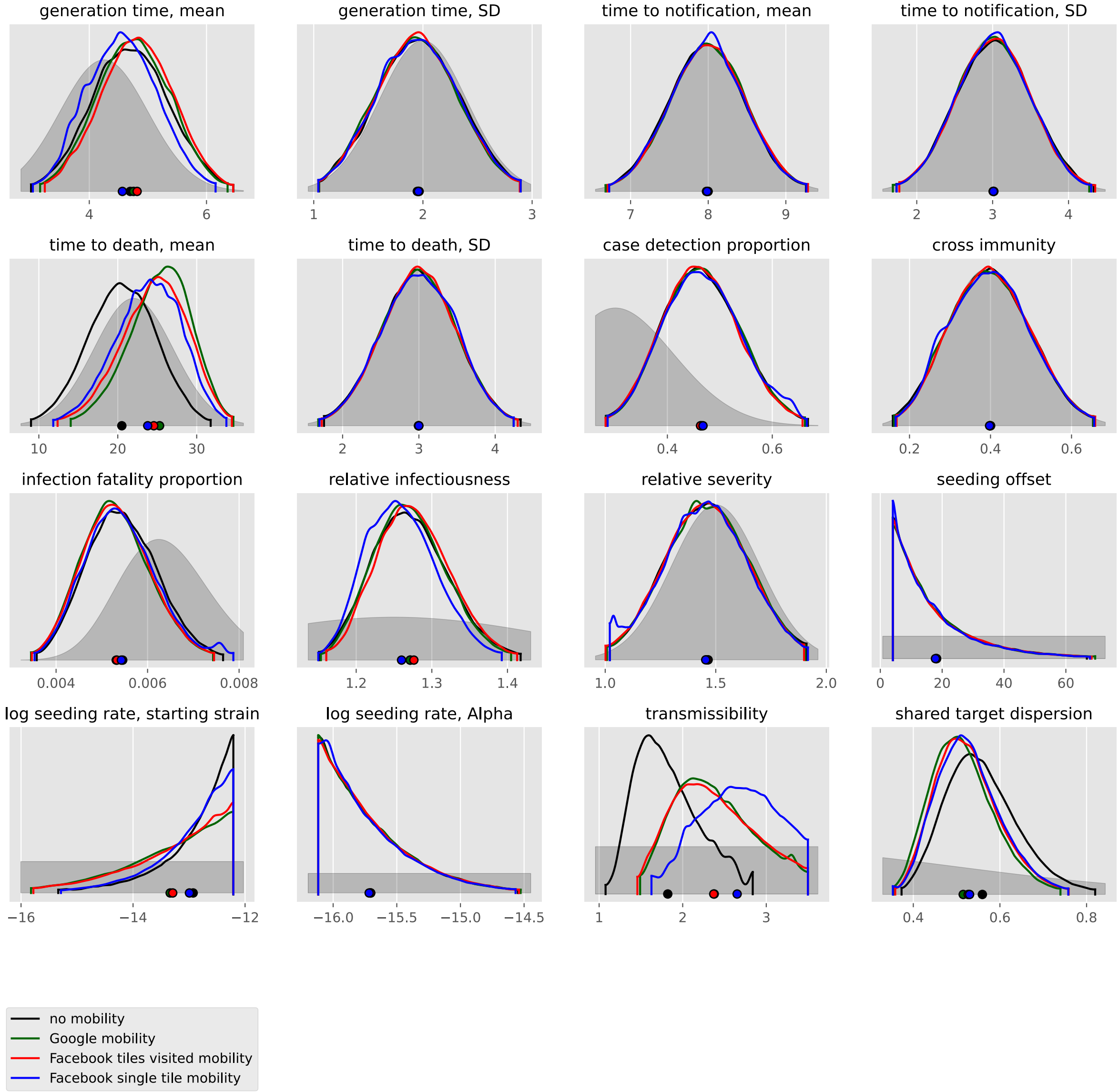

El Salvador

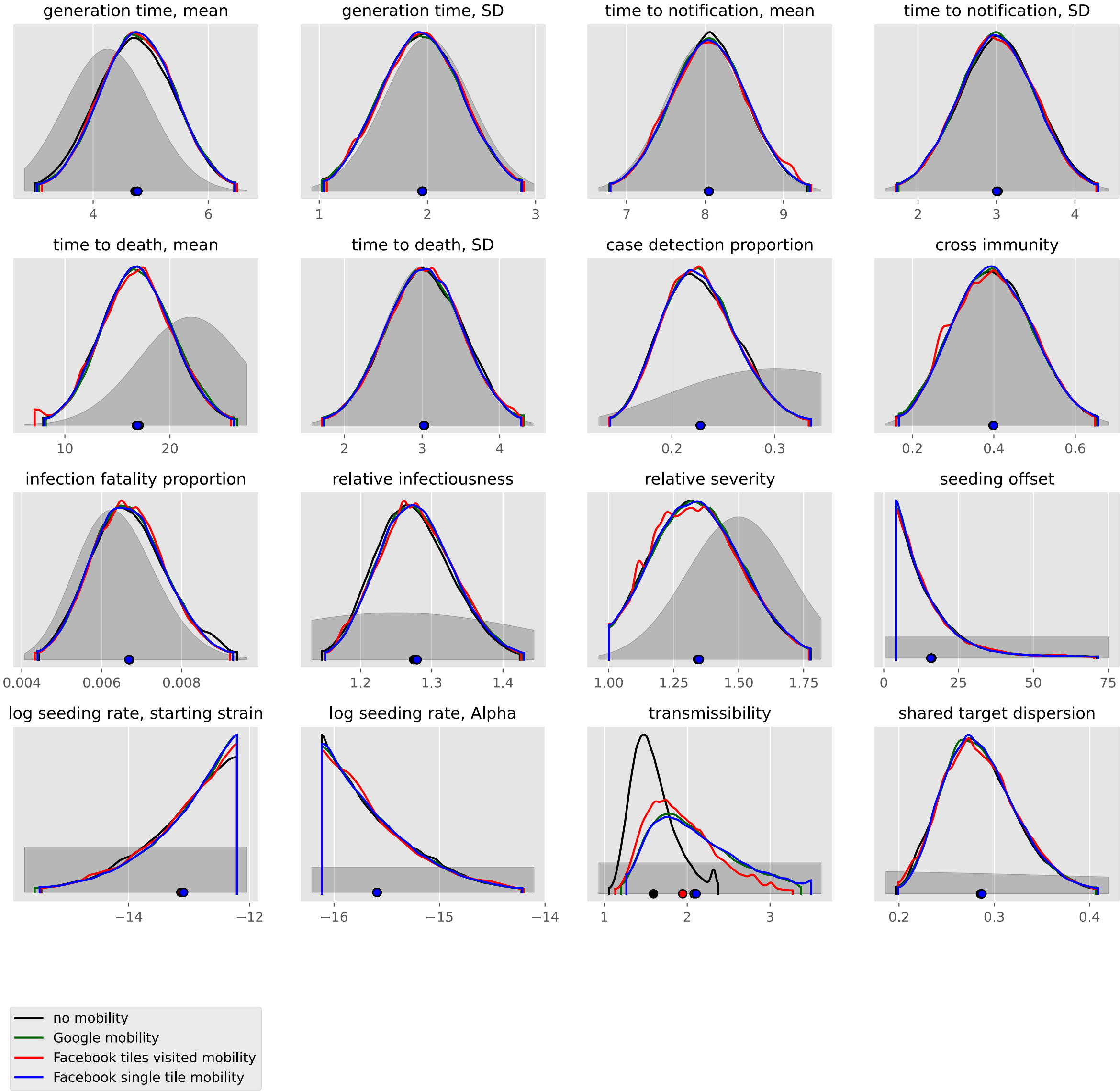
generation time, mean
generation time, SD
time to notification, mean
time to notification, SD
time to death, mean
time to death, SD
case detection proportion
cross immunity
infection fatality proportion
relative infectiousness
relative severity
seeding offset
log seeding rate, starting strain
log seeding rate, Alpha
transmissibility
shared target dispersion
no mobility
Google mobility
Facebook tiles visited mobility
Facebook single tile mobility

Haiti

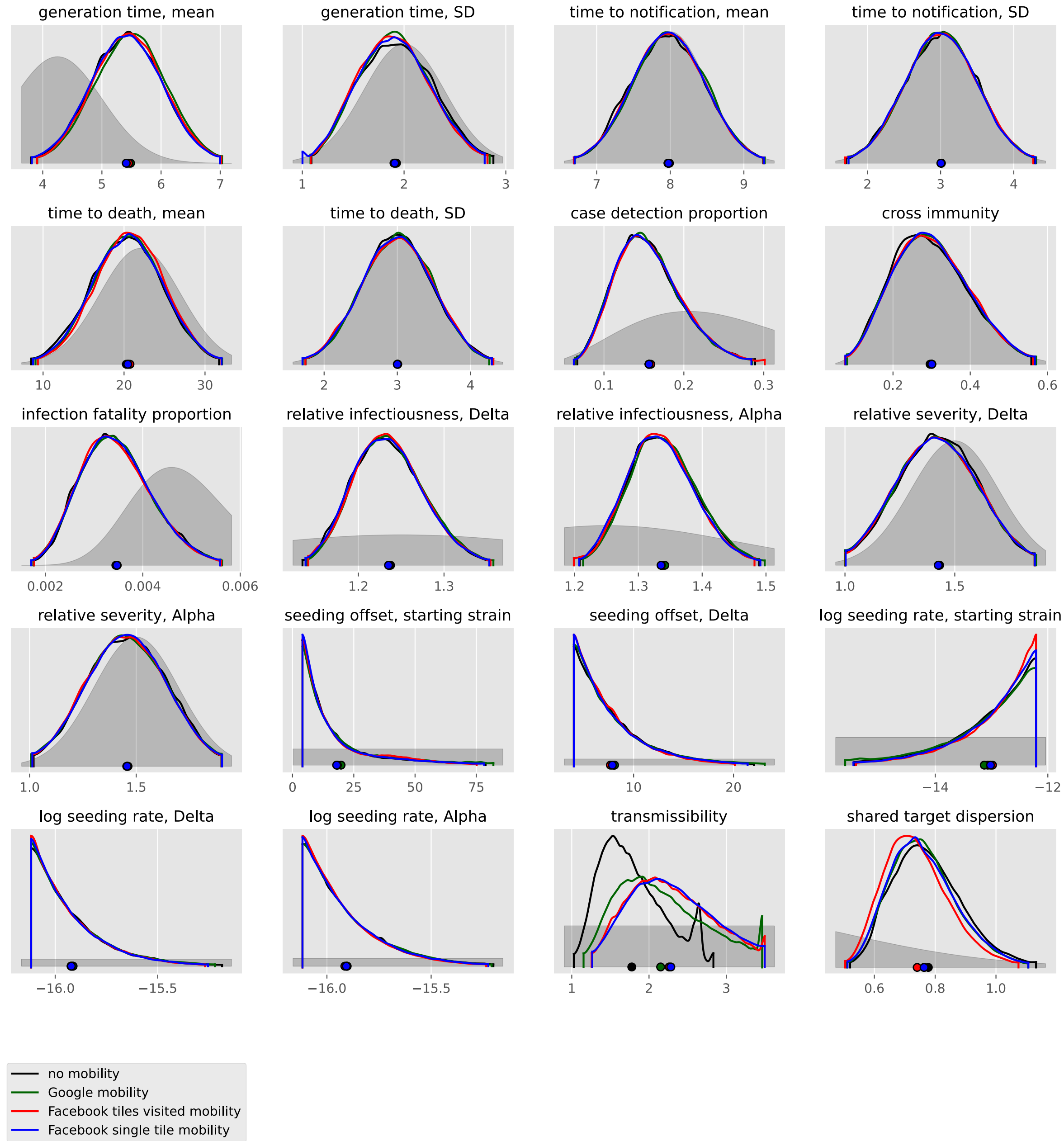
generation time, mean
generation time, SD
time to notification, mean
time to notification, SD
time to death, mean
time to death, SD
case detection proportion
cross immunity
infection fatality proportion
relative infectiousness, Delta
relative infectiousness, Alpha
relative severity, Delta
relative severity, Alpha
seeding offset, starting strain
seeding offset, Delta
log seeding rate, starting strain
log seeding rate, Delta
log seeding rate, Alpha
transmissibility
shared target dispersion
no mobility
Google mobility
Facebook tiles visited mobility
Facebook single tile mobility

Honduras

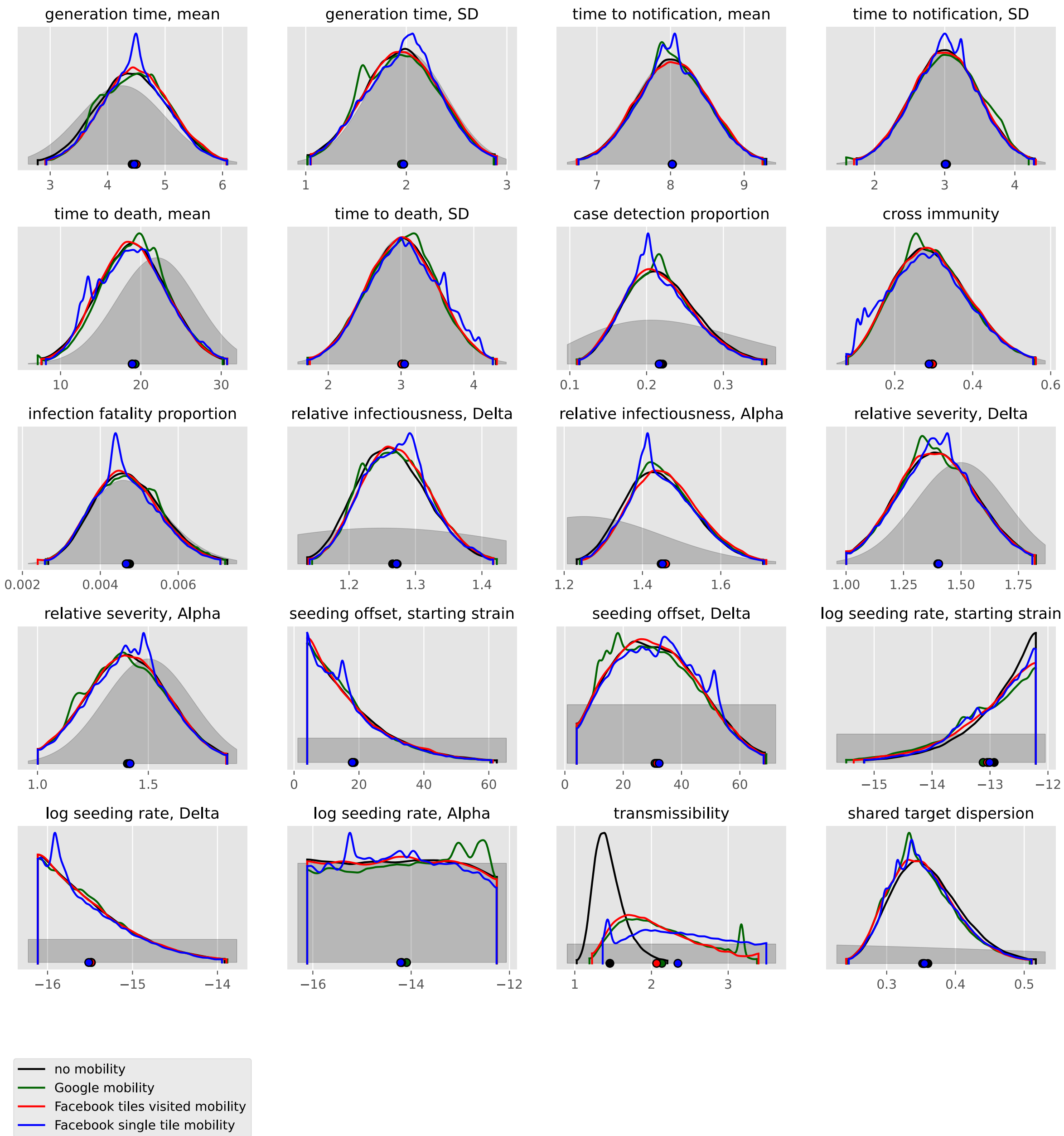
generation time, mean
generation time, SD
time to notification, mean
time to notification, SD
time to death, mean
time to death, SD
case detection proportion
cross immunity
infection fatality proportion
relative infectiousness, Delta
relative infectiousness, Alpha
relative severity, Delta
relative severity, Alpha
seeding offset, starting strain
seeding offset, Delta
log seeding rate, starting strain
log seeding rate, Delta
log seeding rate, Alpha
transmissibility
shared target dispersion
no mobility
Google mobility
Facebook tiles visited mobility
Facebook single tile mobility

Jamaica

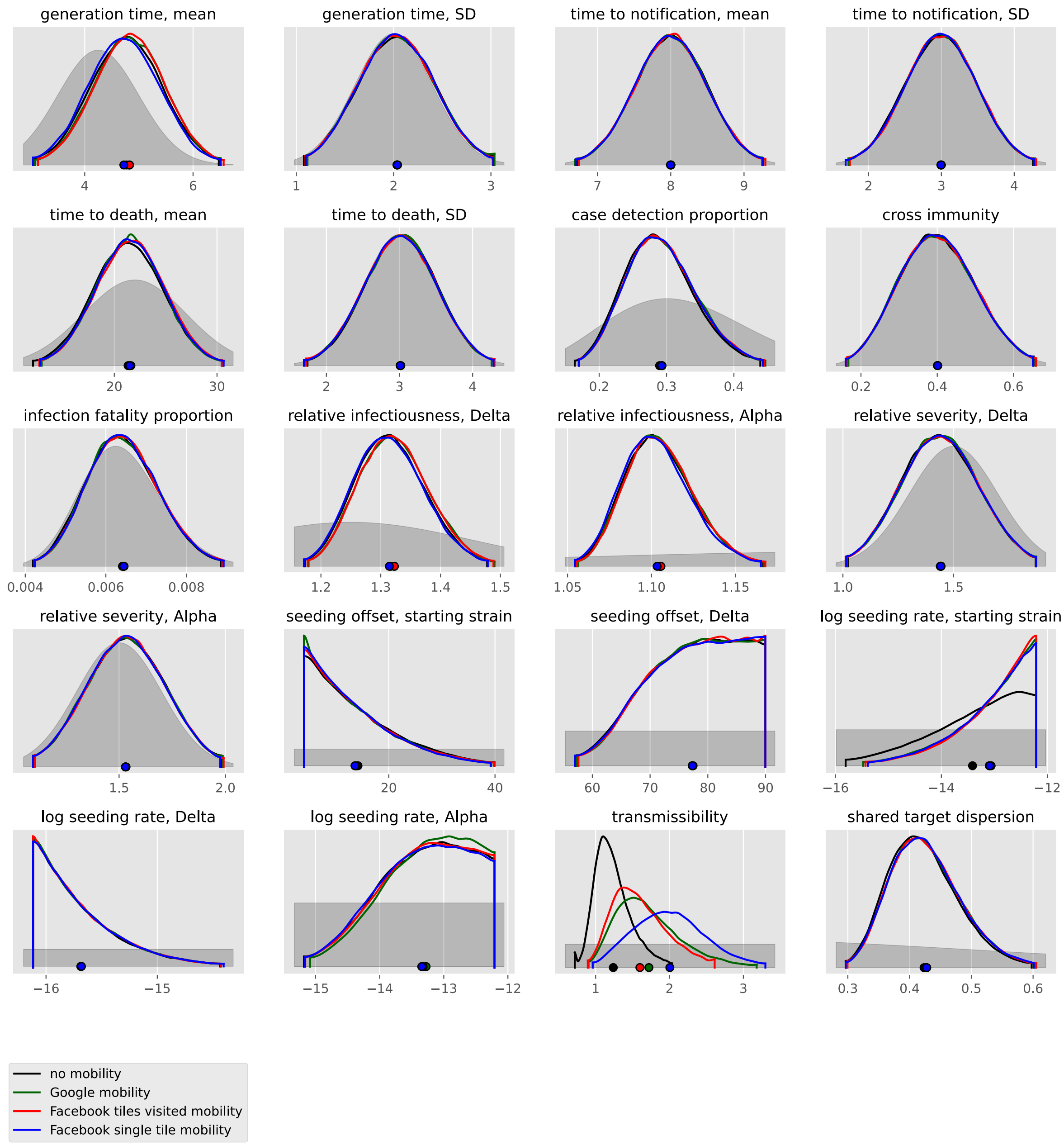
generation time, mean
4
6
generation time, SD
1
2
3
time to notification, mean
7
8
9
time to notification, SD
2
3
4
time to death, mean
20
30
time to death, SD
2
3
4
case detection proportion
0.2
0.3
0.4
cross immunity
0.2
0.4
0.6
infection fatality proportion
0.004
0.006
0.008
relative infectiousness, Delta
1.2
1.3
1.4
1.5
relative infectiousness, Alpha
1.05
1.10
1.15
relative severity, Delta
1.0
1.5
relative severity, Alpha
1.5
2.0
seeding offset, starting strain
20
40
seeding offset, Delta
60
70
80
90
log seeding rate, starting strain
−16
−14
−12
log seeding rate, Delta
−16
−15
log seeding rate, Alpha
−15
−14
−13
−12
transmissibility
1
2
3
shared target dispersion
0.3
0.4
0.5
0.6
no mobility
Google mobility
Facebook tiles visited mobility
Facebook single tile mobility

Martinique

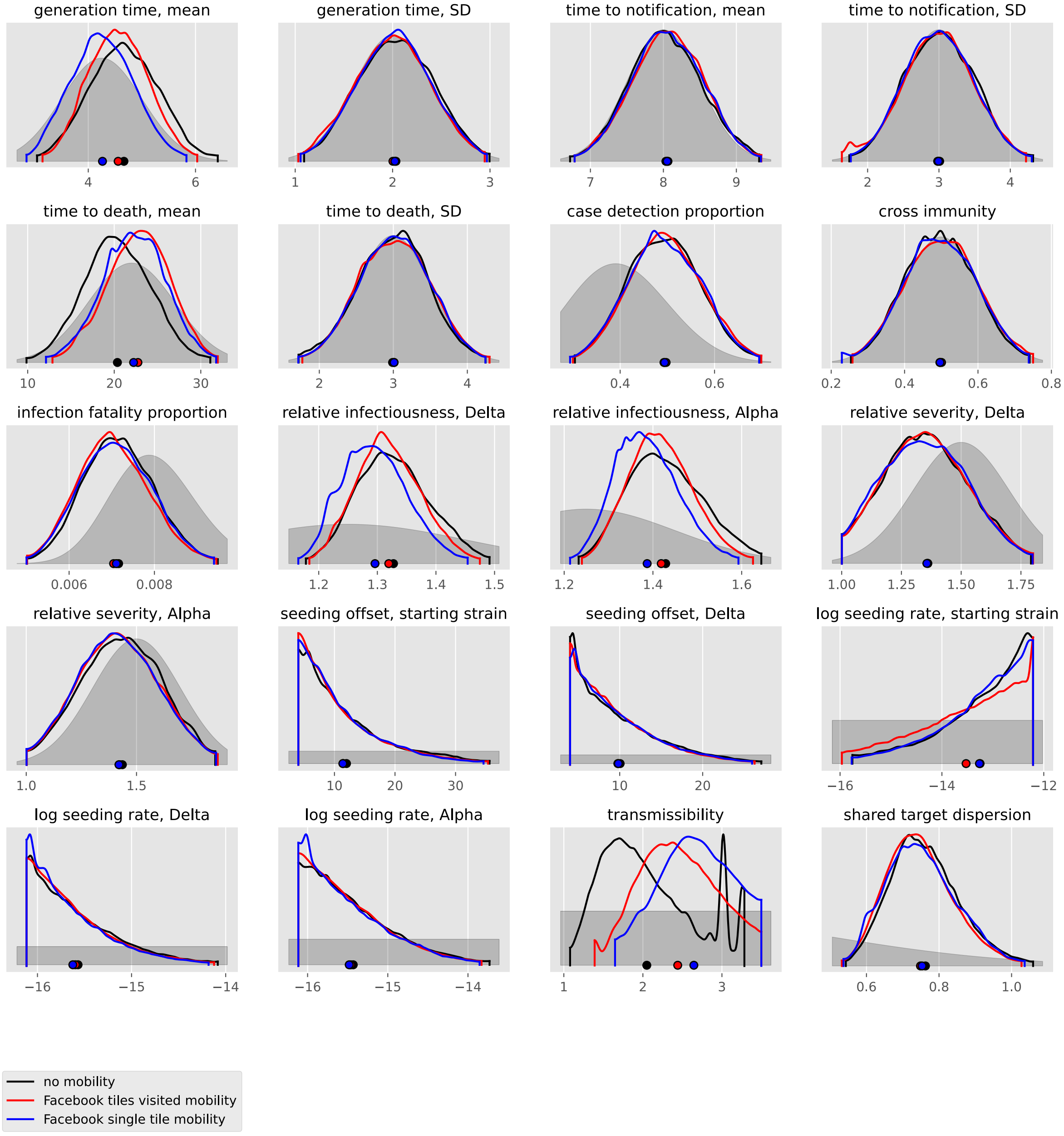

generation time, mean
generation time, SD
time to notification, mean
time to notification, SD
time to death, mean
time to death, SD
case detection proportion
cross immunity
infection fatality proportion
relative infectiousness, Delta
relative infectiousness, Alpha
relative severity, Delta
relative severity, Alpha
seeding offset, starting strain
seeding offset, Delta
log seeding rate, starting strain
log seeding rate, Delta
log seeding rate, Alpha
transmissibility
shared target dispersion
no mobility
Facebook tiles visited mobility
Facebook single tile mobility

Mexico

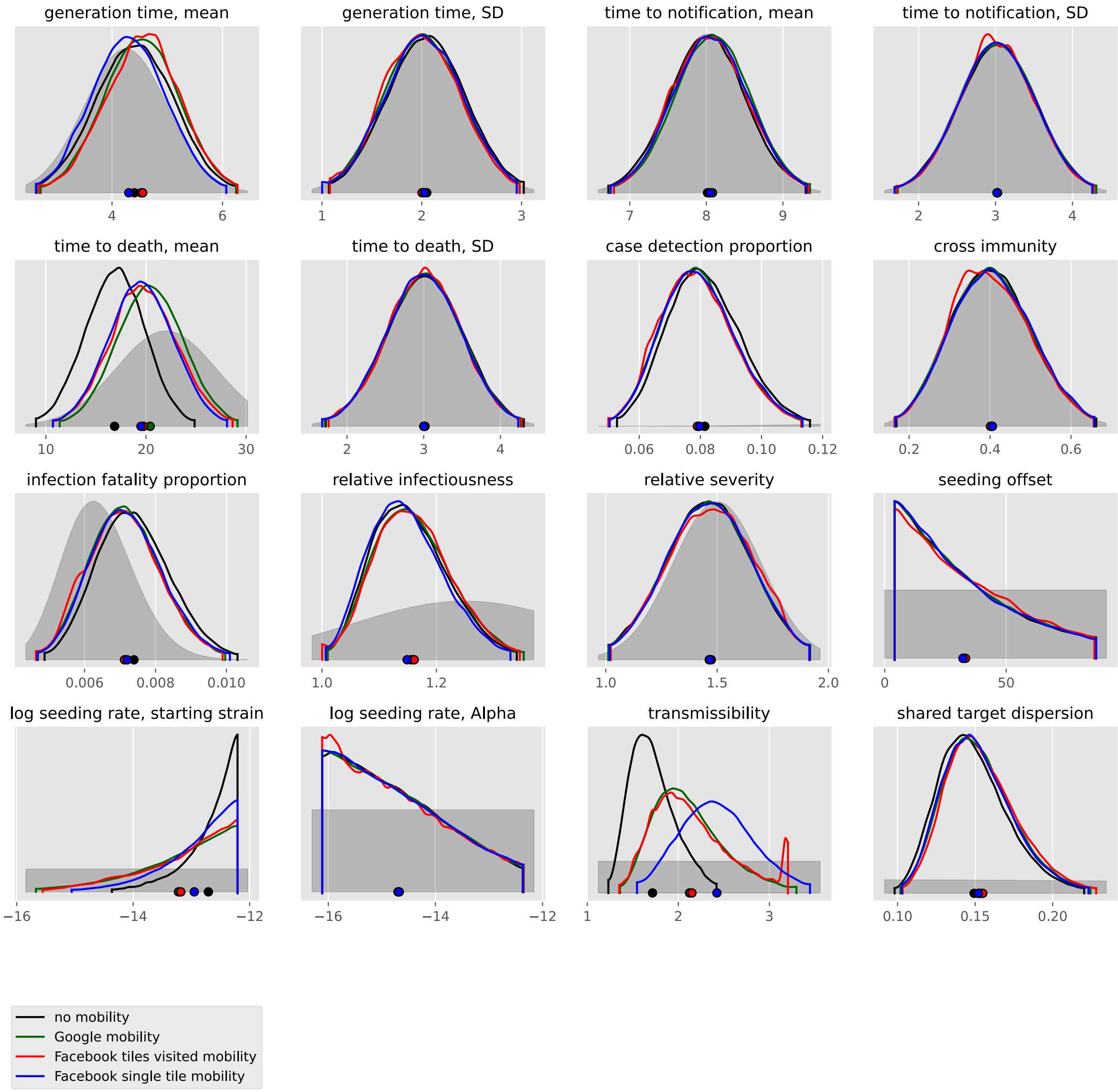

generation time, mean
4
6
generation time, SD
1
2
3
time to notification, mean
7
8
9
time to notification, SD
2
3
4
time to death, mean
10
20
30
time to death, SD
2
3
4
case detection proportion
0.06
0.08
0.10
0.12
cross immunity
0.2
0.4
0.6
infection fatality proportion
0.006
0.008
0.010
relative infectiousness
1.0
1.2
relative severity
1.0
1.5
2.0
seeding offset
0
50
log seeding rate, starting strain
−16
−14
−12
log seeding rate, Alpha
−16
−14
−12
transmissibility
1
2
3
shared target dispersion
0.10
0.15
0.20
no mobility
Google mobility
Facebook tiles visited mobility
Facebook single tile mobility

Panama

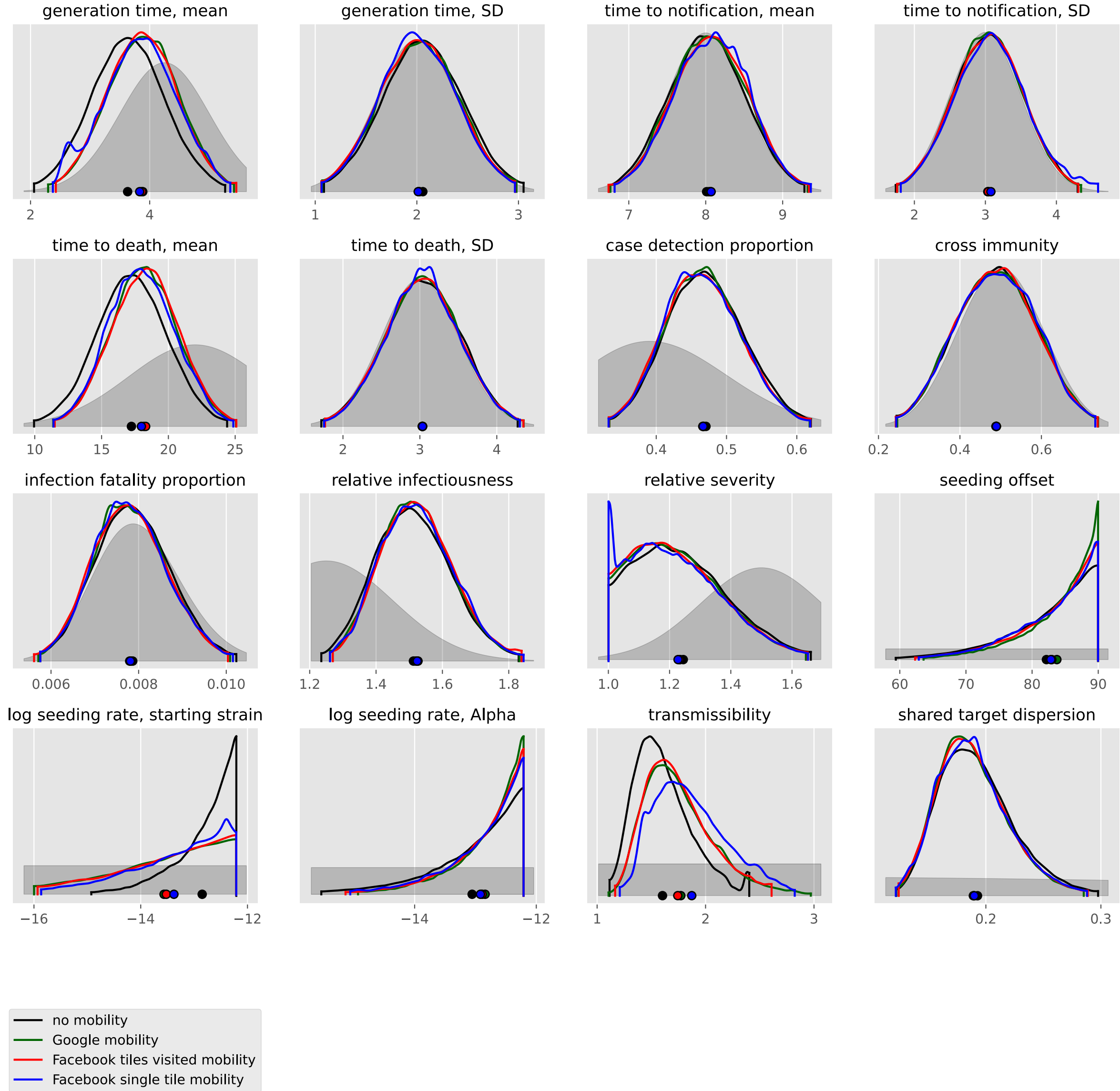

generation time, mean
generation time, SD
time to notification, mean
time to notification, SD
time to death, mean
time to death, SD
case detection proportion
cross immunity
infection fatality proportion
relative infectiousness
relative severity
seeding offset
log seeding rate, starting strain
log seeding rate, Alpha
transmissibility
shared target dispersion
no mobility
Google mobility
Facebook tiles visited mobility
Facebook single tile mobility

Puerto Rico

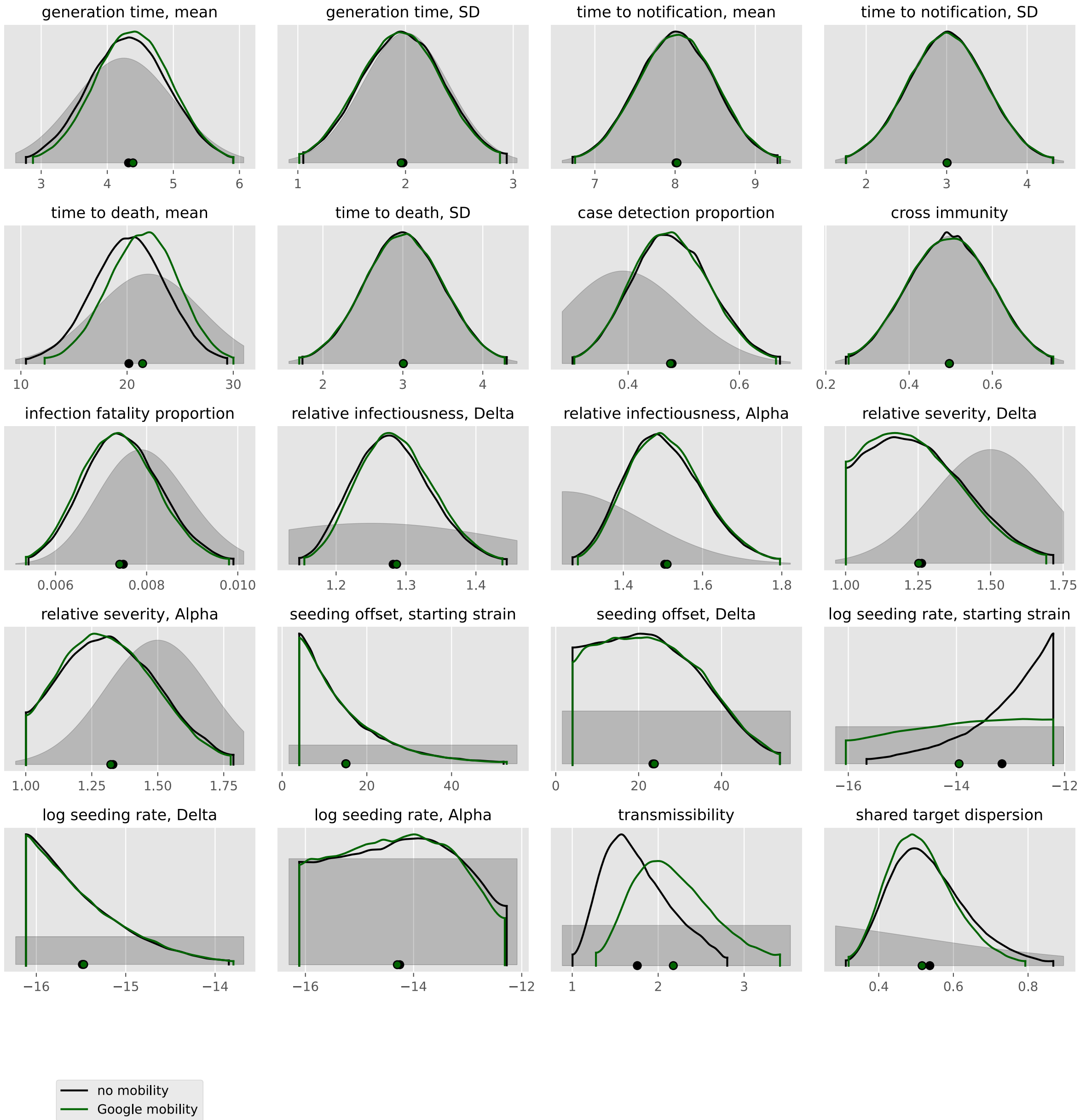

generation time, mean
generation time, SD
time to notification, mean
time to notification, SD
time to death, mean
time to death, SD
case detection proportion
cross immunity
infection fatality proportion
relative infectiousness, Delta
relative infectiousness, Alpha
relative severity, Delta
relative severity, Alpha
seeding offset, starting strain
seeding offset, Delta
log seeding rate, starting strain
log seeding rate, Delta
log seeding rate, Alpha
transmissibility
shared target dispersion
no mobility
Google mobility

United States

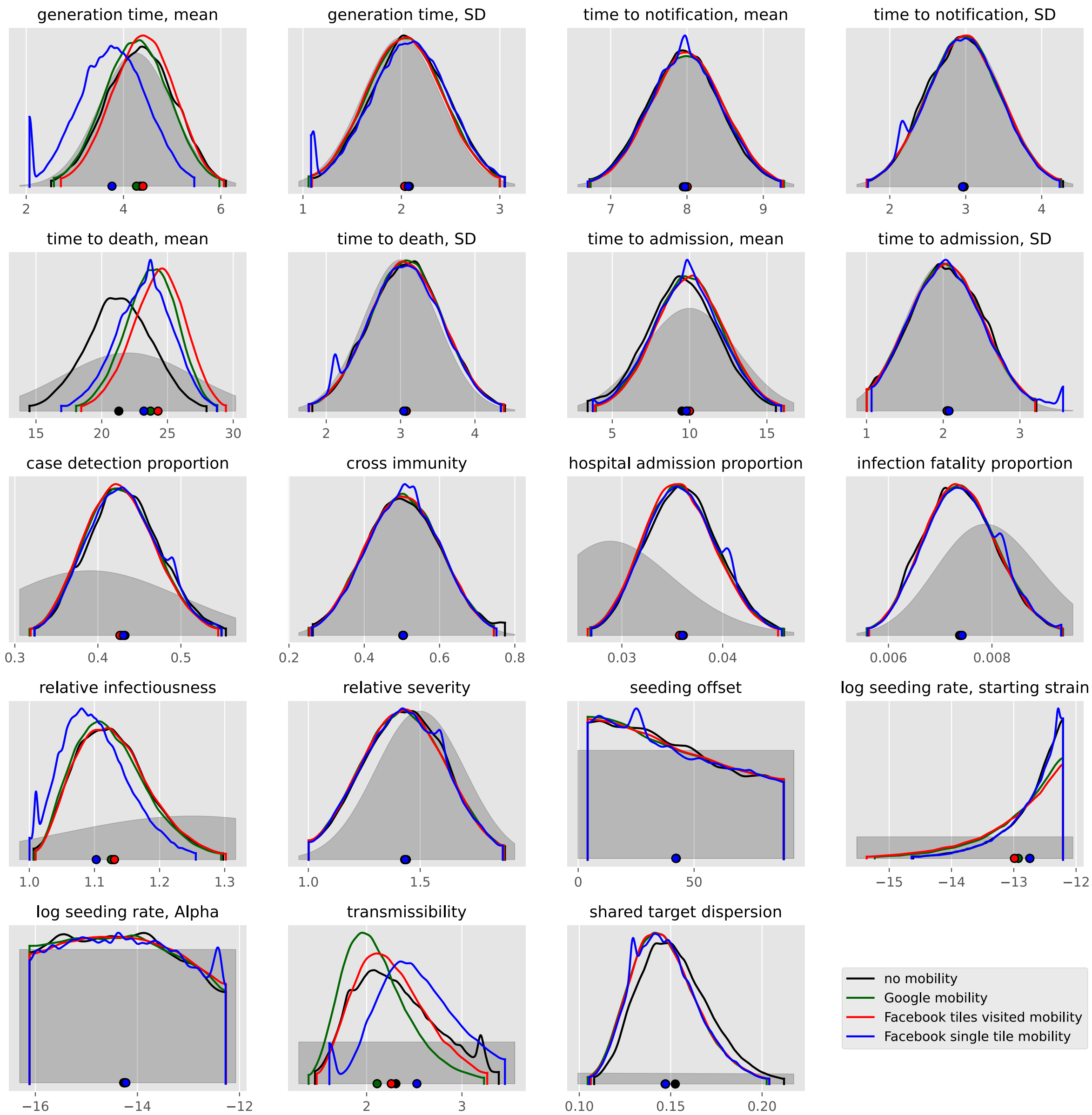

Oceania

Australia

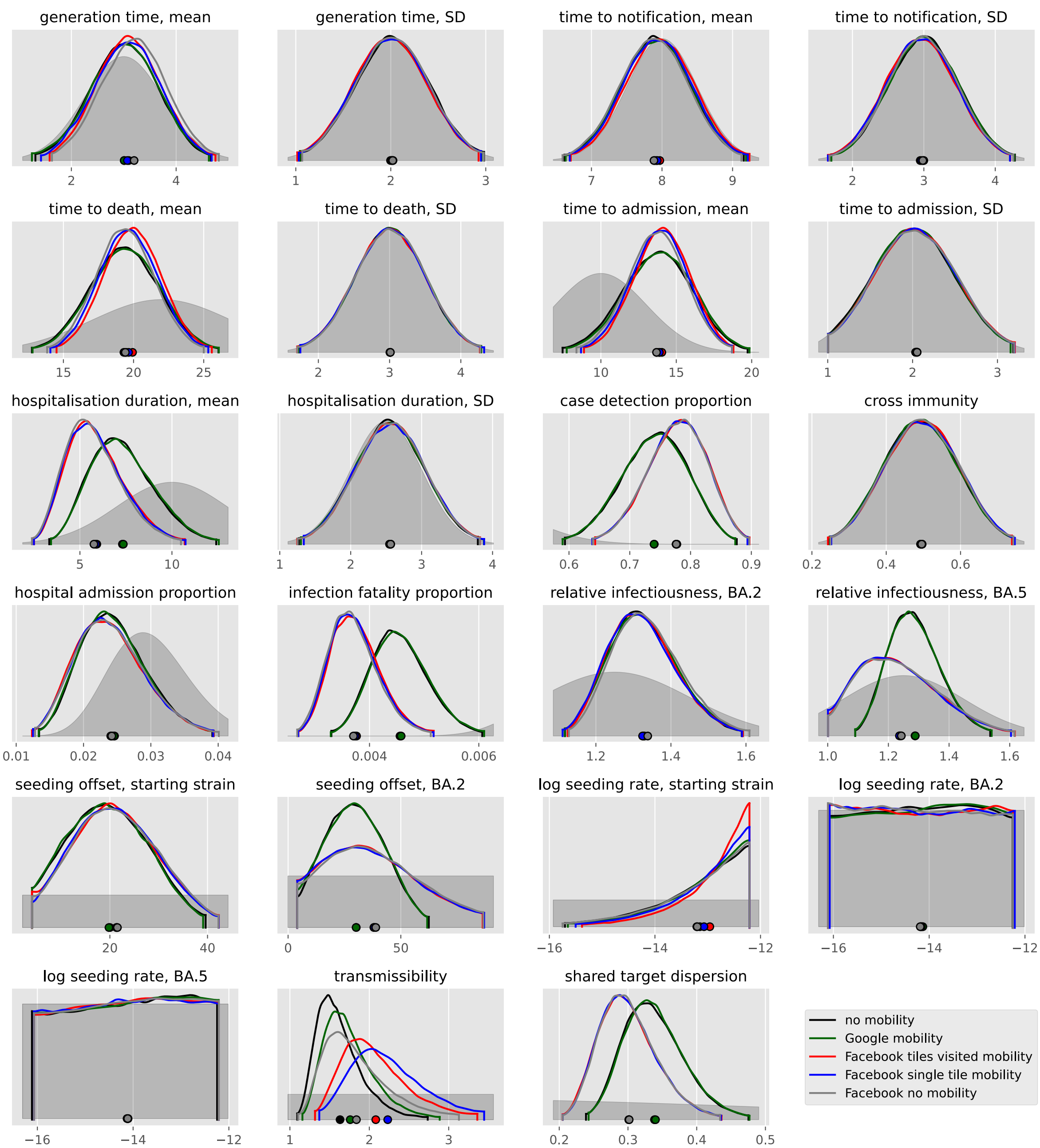

Fiji

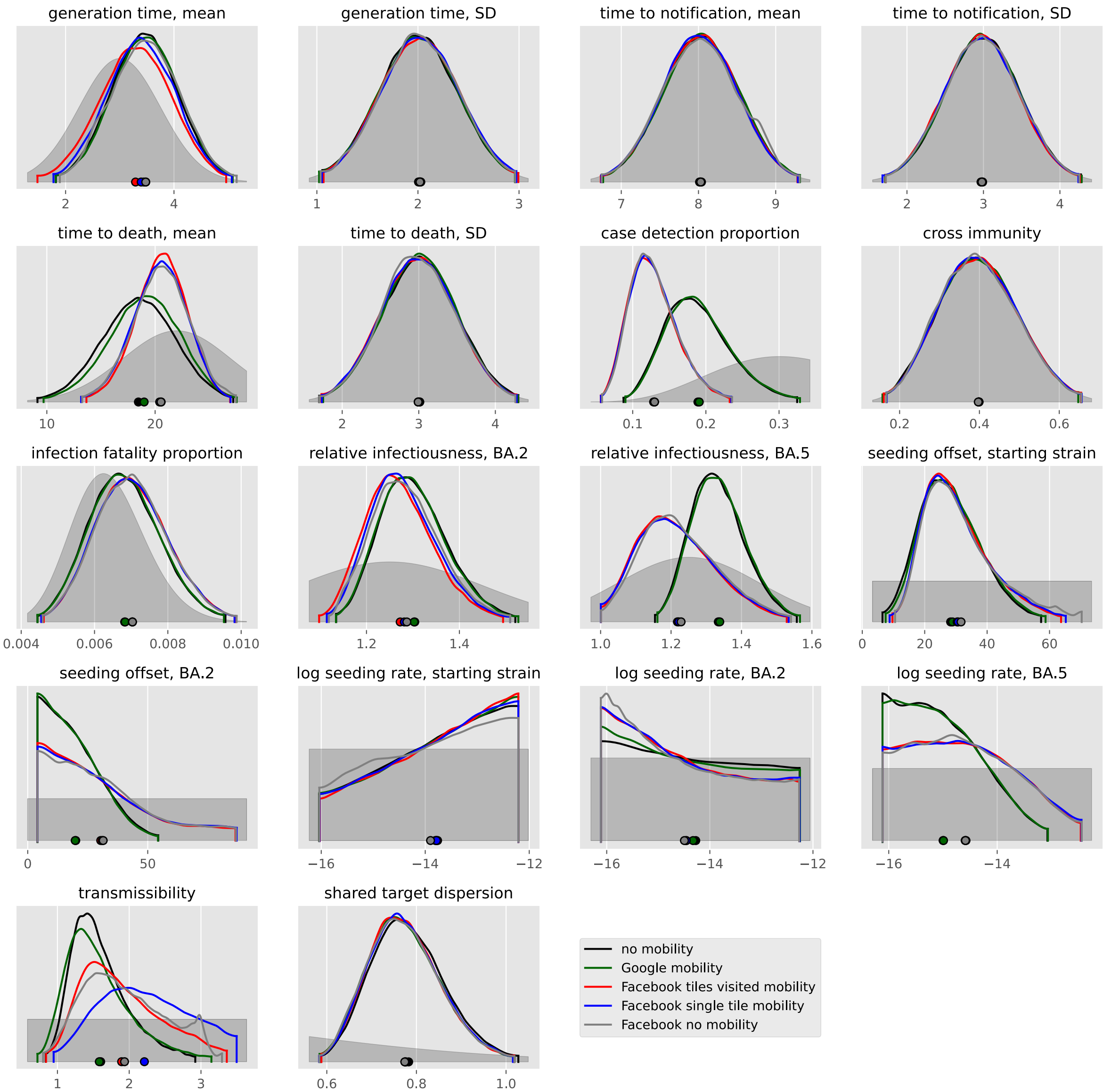

generation time, mean
generation time, SD
time to notification, mean
time to notification, SD
time to death, mean
time to death, SD
case detection proportion
cross immunity
infection fatality proportion
relative infectiousness, BA.2
relative infectiousness, BA.5
seeding offset, starting strain
seeding offset, BA.2
log seeding rate, starting strain
log seeding rate, BA.2
log seeding rate, BA.5
transmissibility
shared target dispersion
no mobility
Google mobility
Facebook tiles visited mobility
Facebook single tile mobility
Facebook no mobility

New Zealand

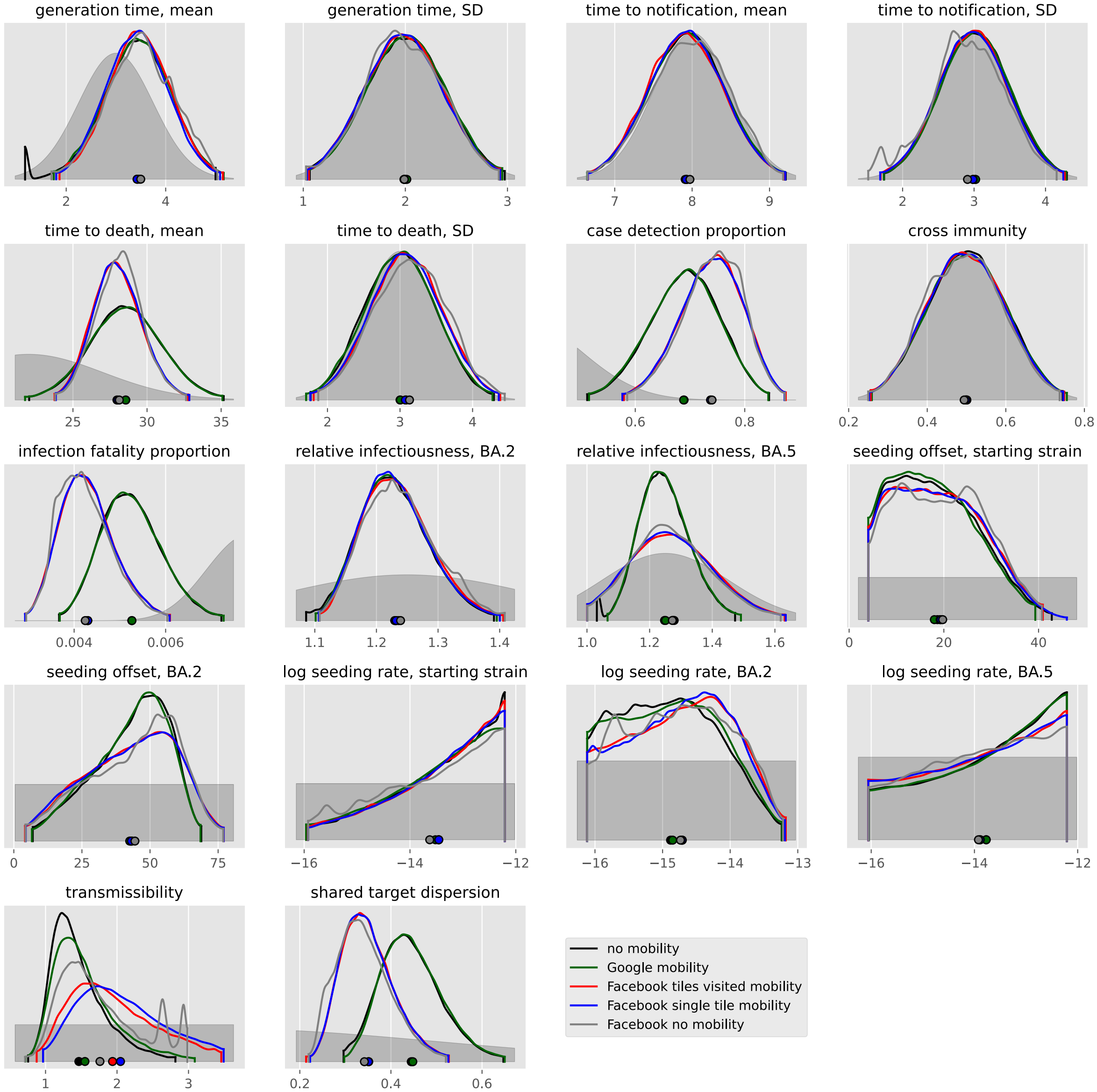

generation time, mean
generation time, SD
time to notification, mean
time to notification, SD
time to death, mean
time to death, SD
case detection proportion
cross immunity
infection fatality proportion
relative infectiousness, BA.2
relative infectiousness, BA.5
seeding offset, starting strain
seeding offset, BA.2
log seeding rate, starting strain
log seeding rate, BA.2
log seeding rate, BA.5
transmissibility
shared target dispersion
no mobility
Google mobility
Facebook tiles visited mobility
Facebook single tile mobility
Facebook no mobility

Singapore

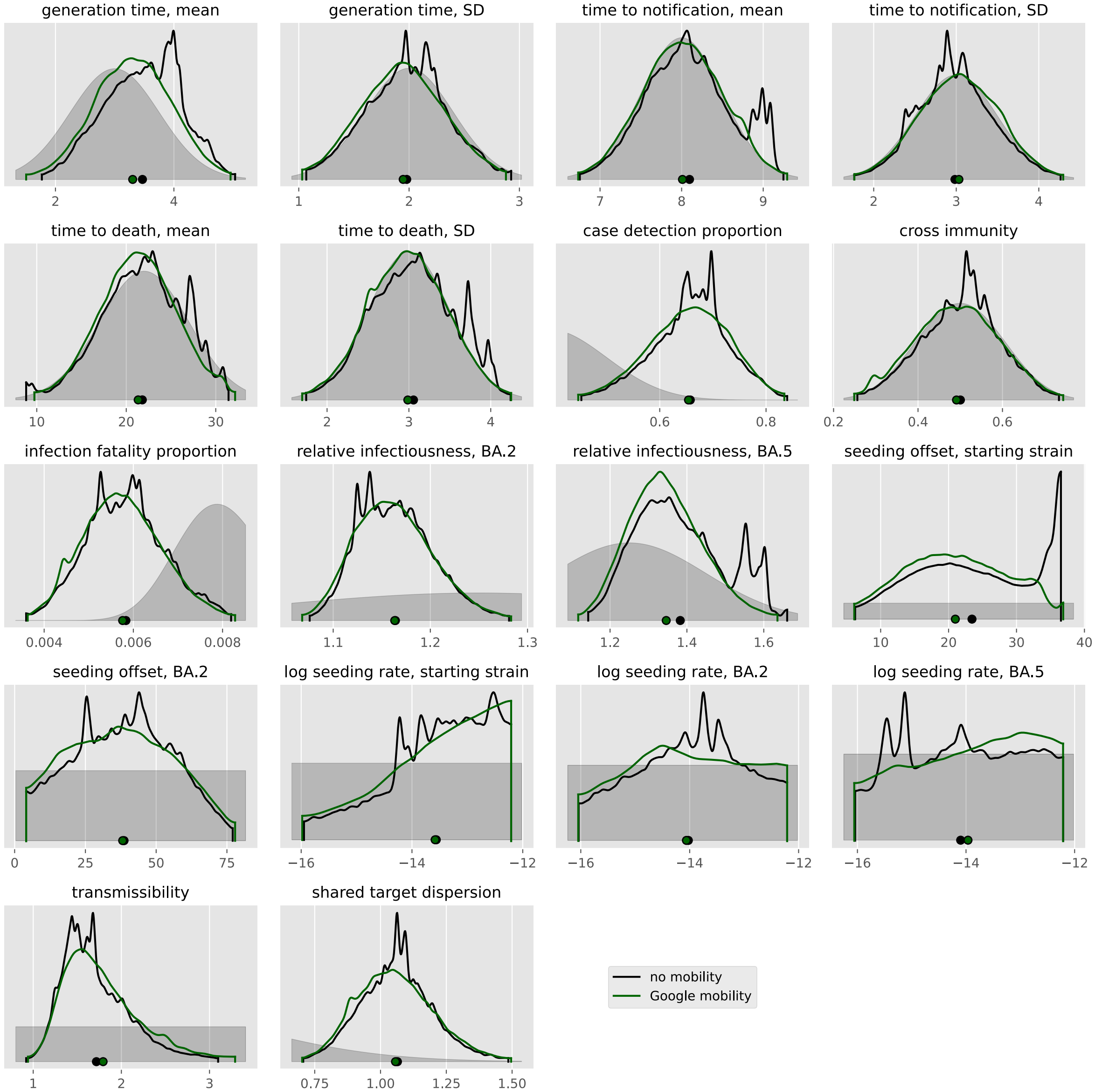

generation time, mean
2
4
generation time, SD
1
2
3
time to notification, mean
7
8
9
time to notification, SD
2
3
4
time to death, mean
10
20
30
time to death, SD
2
3
4
case detection proportion
0.6
0.8
cross immunity
0.2
0.4
0.6
infection fatality proportion
0.004
0.006
0.008
relative infectiousness, BA.2
1.1
1.2
1.3
relative infectiousness, BA.5
1.2
1.4
1.6
seeding offset, starting strain
10
20
30
40
seeding offset, BA.2
0
25
50
75
log seeding rate, starting strain
−16
−14
−12
log seeding rate, BA.2
−16
−14
−12
log seeding rate, BA.5
−16
−14
−12
transmissibility
1
2
3
shared target dispersion
0.75
1.00
1.25
1.50
no mobility
Google mobility

South America

Argentina

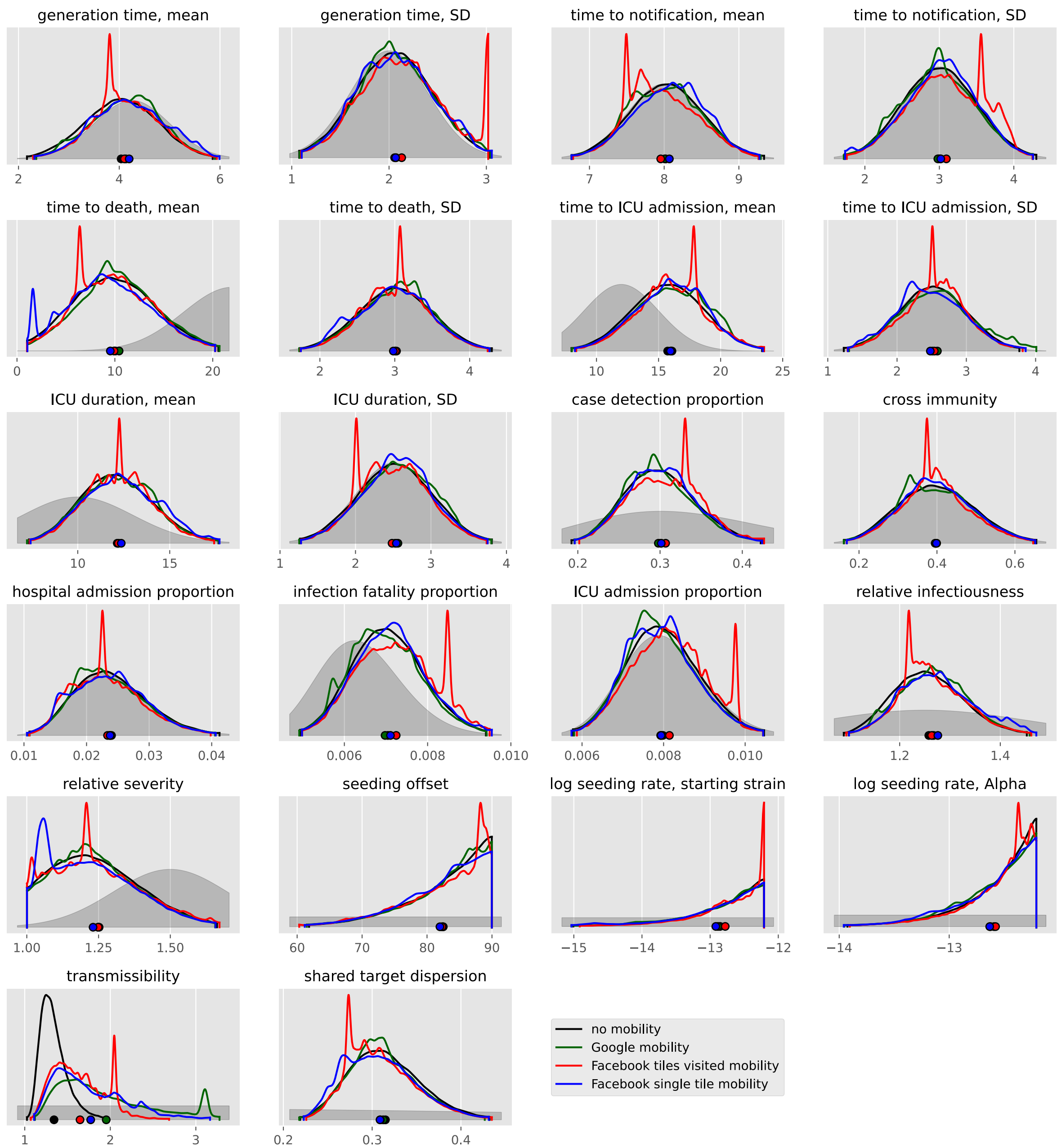

Brazil

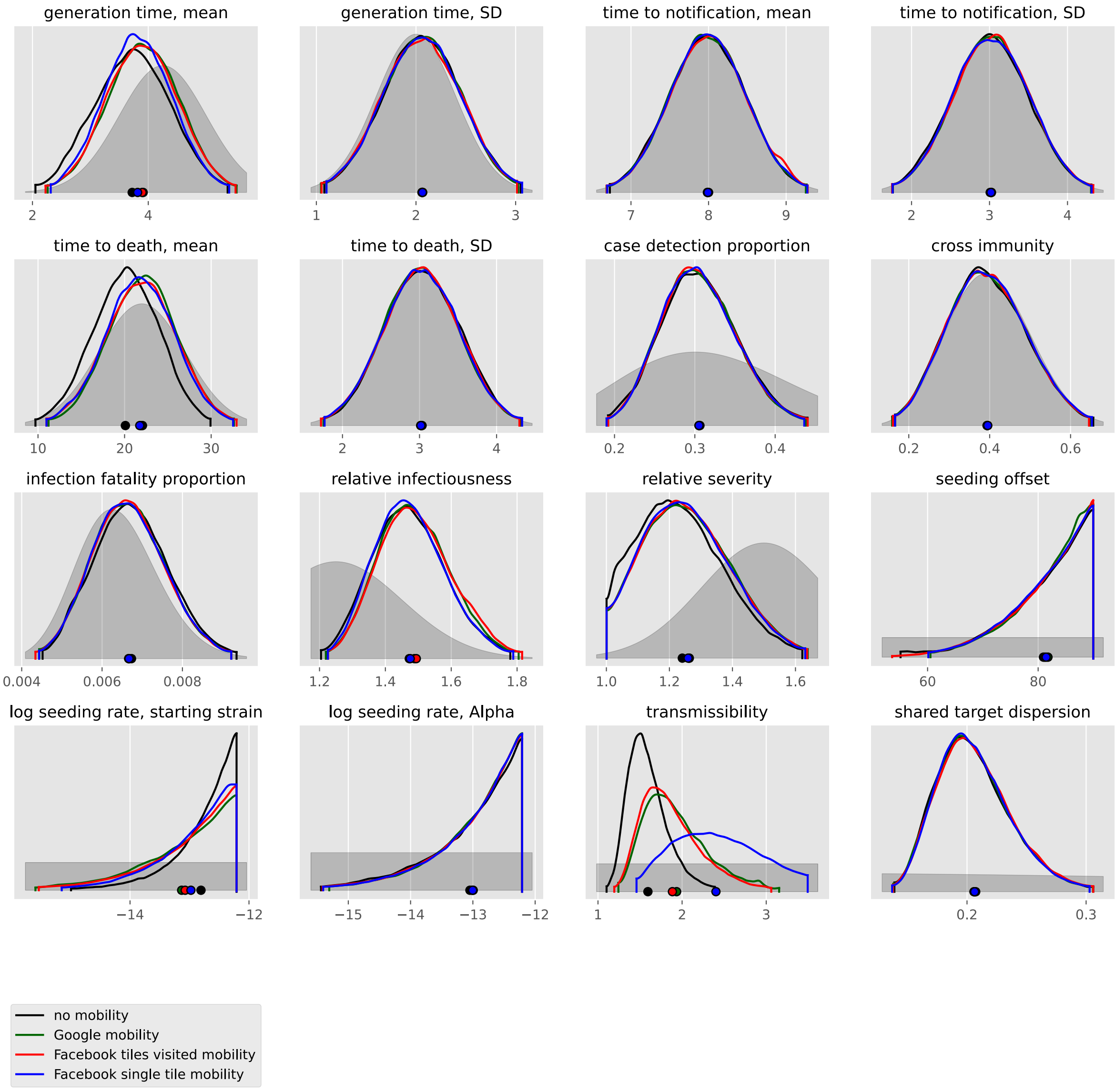

generation time, mean
generation time, SD
time to notification, mean
time to notification, SD
time to death, mean
time to death, SD
case detection proportion
cross immunity
infection fatality proportion
relative infectiousness
relative severity
seeding offset
log seeding rate, starting strain
log seeding rate, Alpha
transmissibility
shared target dispersion
no mobility
Google mobility
Facebook tiles visited mobility
Facebook single tile mobility

Chile

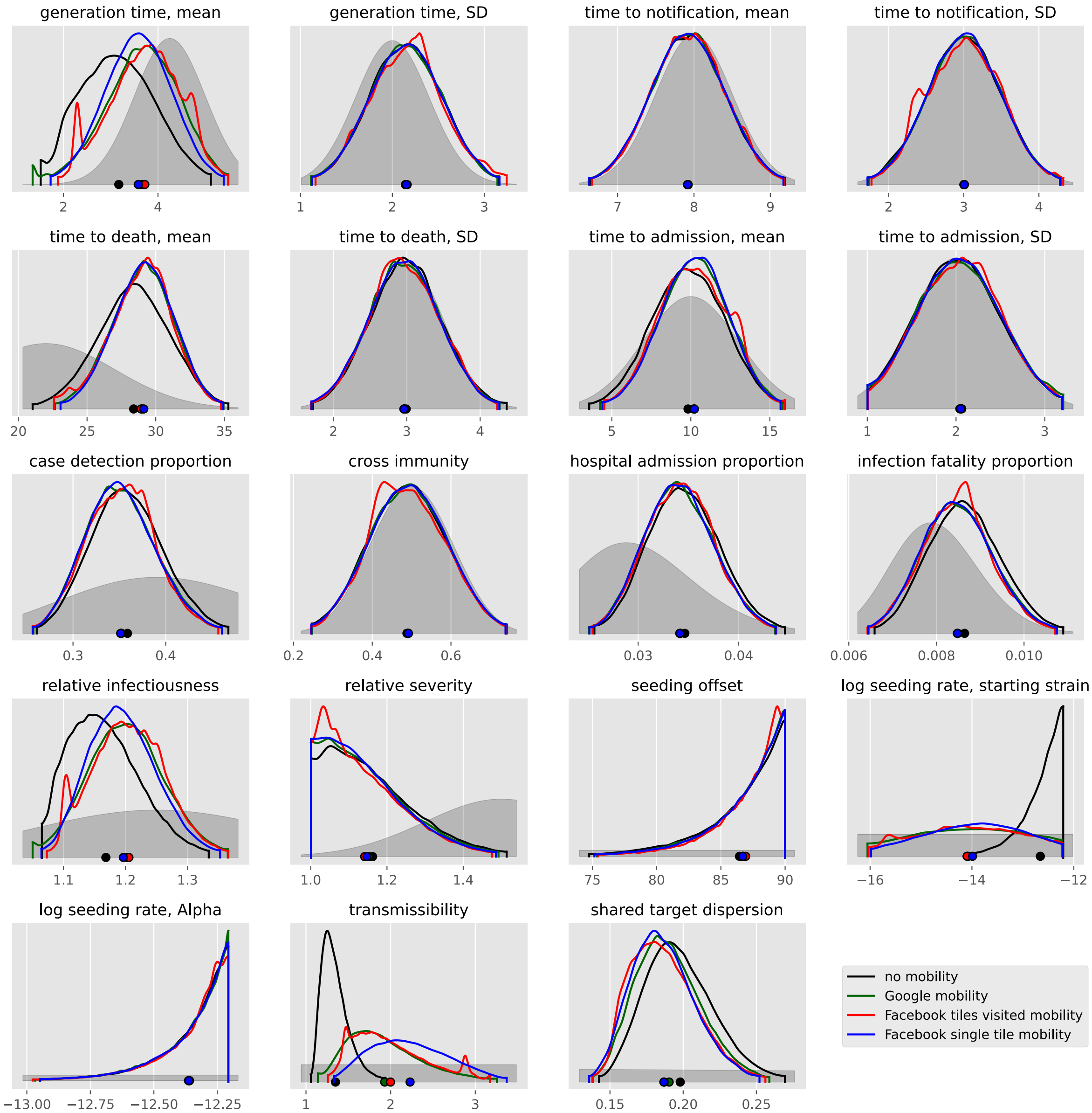
generation time, mean
generation time, SD
time to notification, mean
time to notification, SD
time to death, mean
time to death, SD
time to admission, mean
time to admission, SD
case detection proportion
cross immunity
hospital admission proportion
infection fatality proportion
relative infectiousness
relative severity
seeding offset
log seeding rate, starting strain
log seeding rate, Alpha
transmissibility
shared target dispersion
no mobility
Google mobility
Facebook tiles visited mobility
Facebook single tile mobility

Colombia

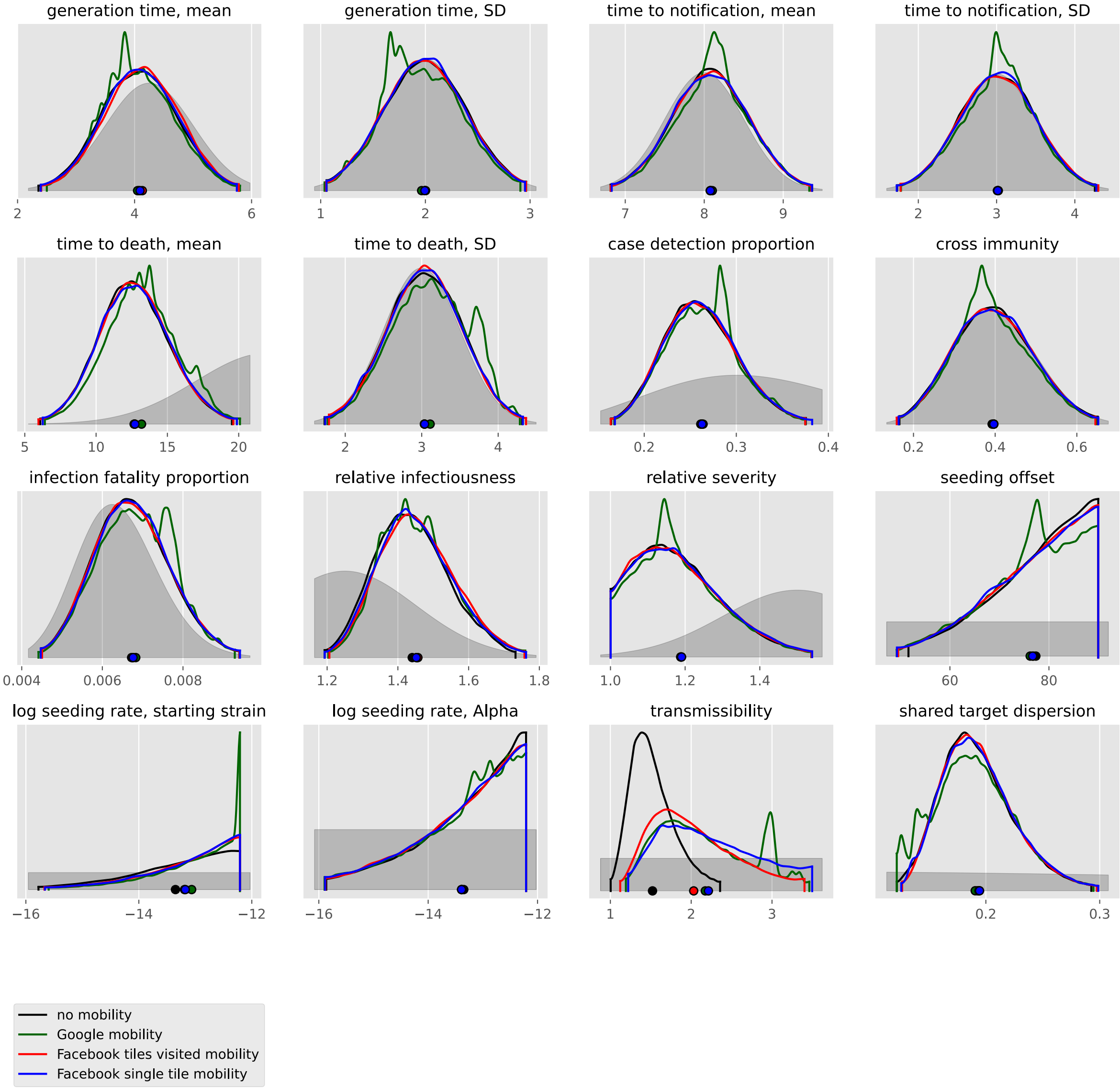

generation time, mean
generation time, SD
time to notification, mean
time to notification, SD
time to death, mean
time to death, SD
case detection proportion
cross immunity
infection fatality proportion
relative infectiousness
relative severity
seeding offset
log seeding rate, starting strain
log seeding rate, Alpha
transmissibility
shared target dispersion
no mobility
Google mobility
Facebook tiles visited mobility
Facebook single tile mobility

French Guiana

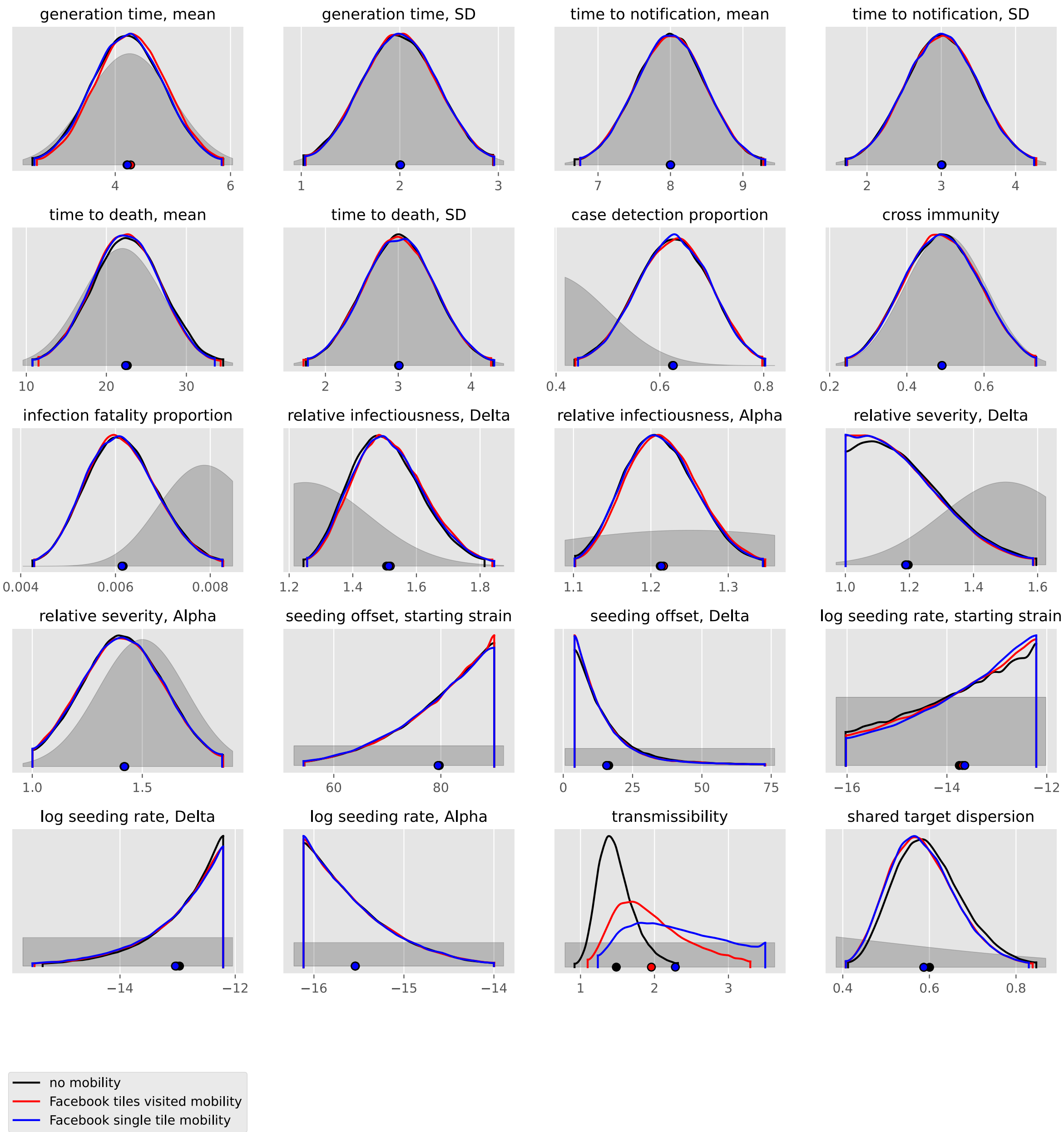

Guyana

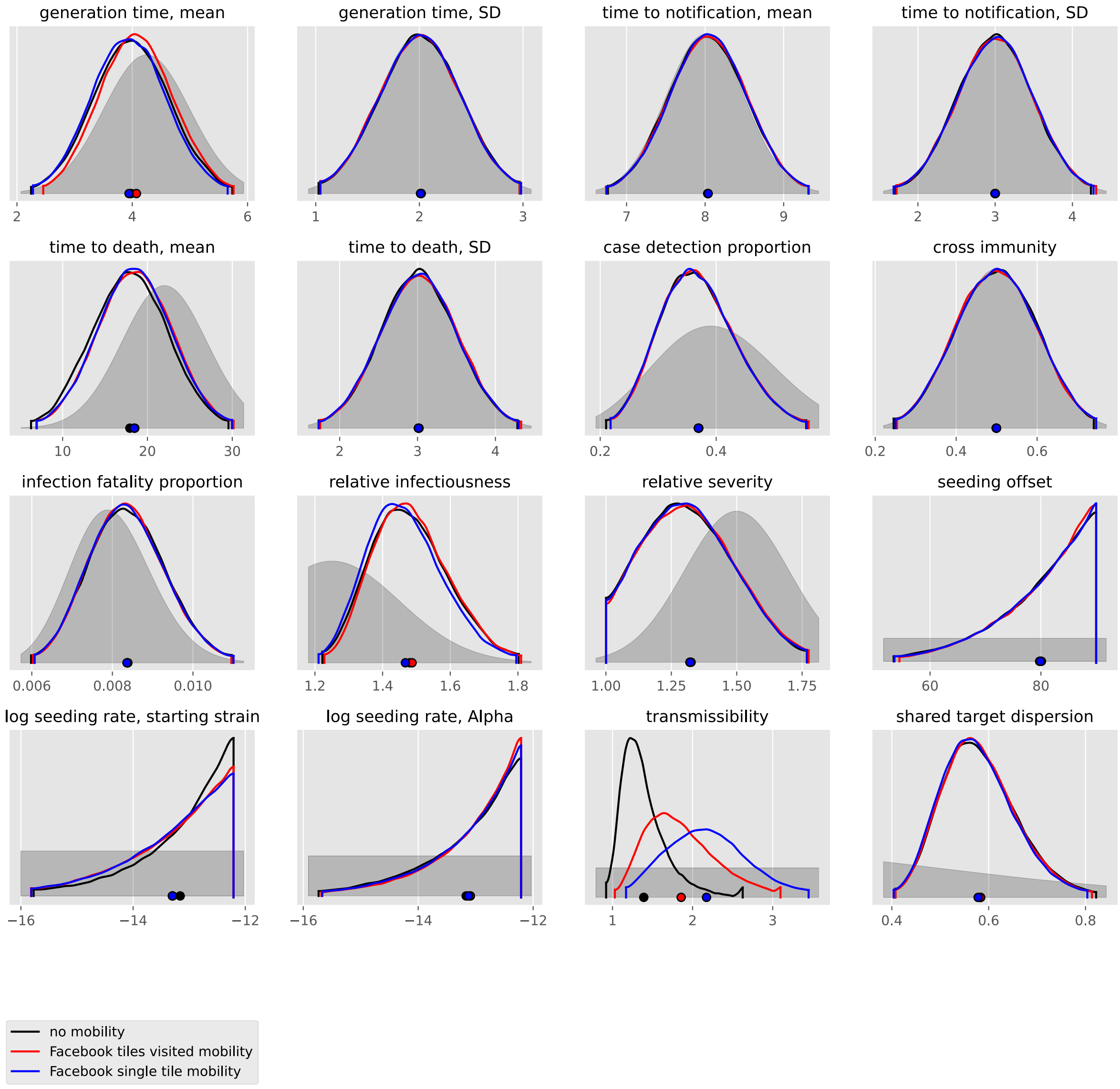

generation time, mean
generation time, SD
time to notification, mean
time to notification, SD
time to death, mean
time to death, SD
case detection proportion
cross immunity
infection fatality proportion
relative infectiousness
relative severity
seeding offset
log seeding rate, starting strain
log seeding rate, Alpha
transmissibility
shared target dispersion
no mobility
Facebook tiles visited mobility
Facebook single tile mobility

Paraguay

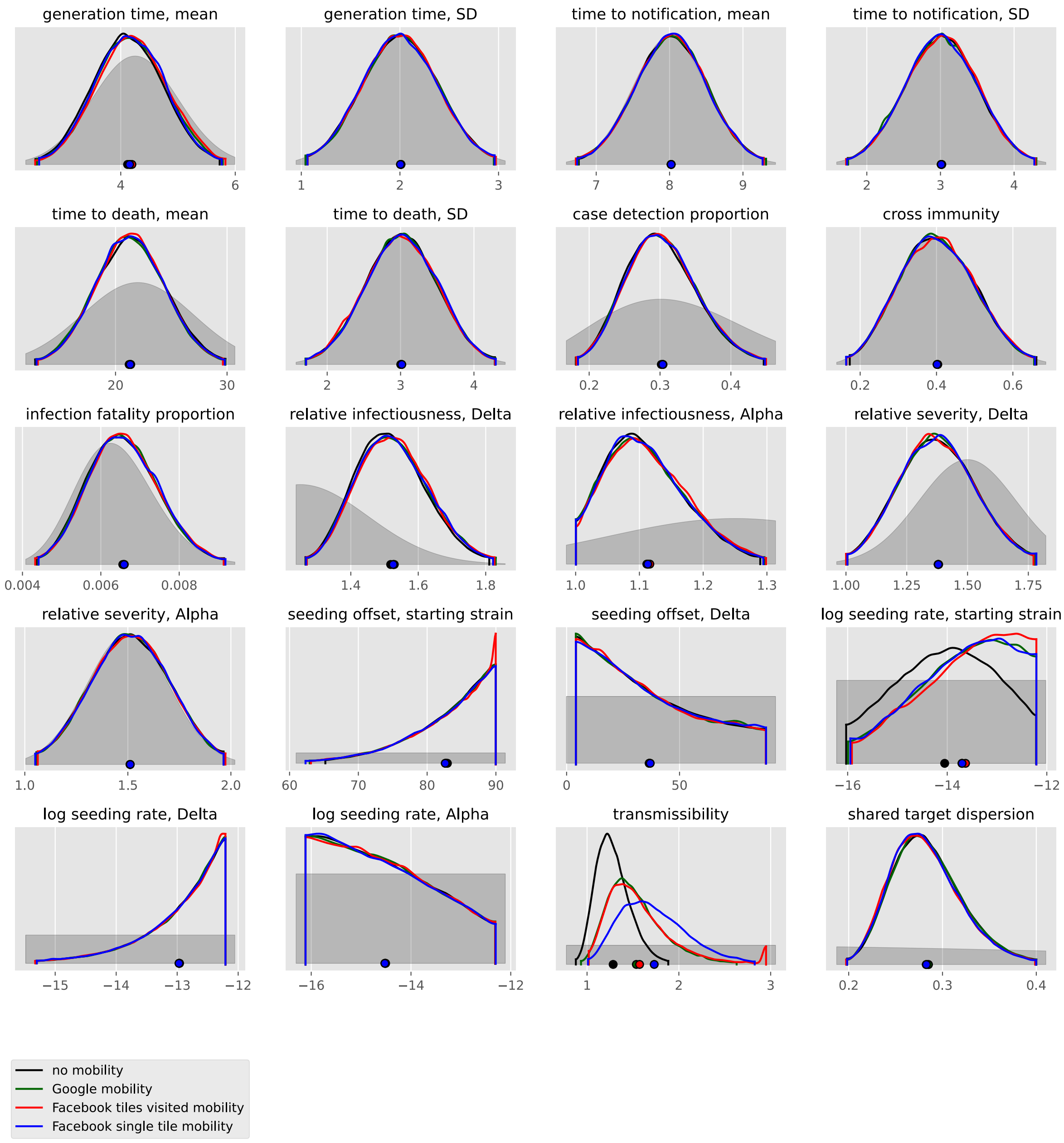

generation time, mean
generation time, SD
time to notification, mean
time to notification, SD
time to death, mean
time to death, SD
case detection proportion
cross immunity
infection fatality proportion
relative infectiousness, Delta
relative infectiousness, Alpha
relative severity, Delta
relative severity, Alpha
seeding offset, starting strain
seeding offset, Delta
log seeding rate, starting strain
log seeding rate, Delta
log seeding rate, Alpha
transmissibility
shared target dispersion
no mobility
Google mobility
Facebook tiles visited mobility
Facebook single tile mobility

Peru

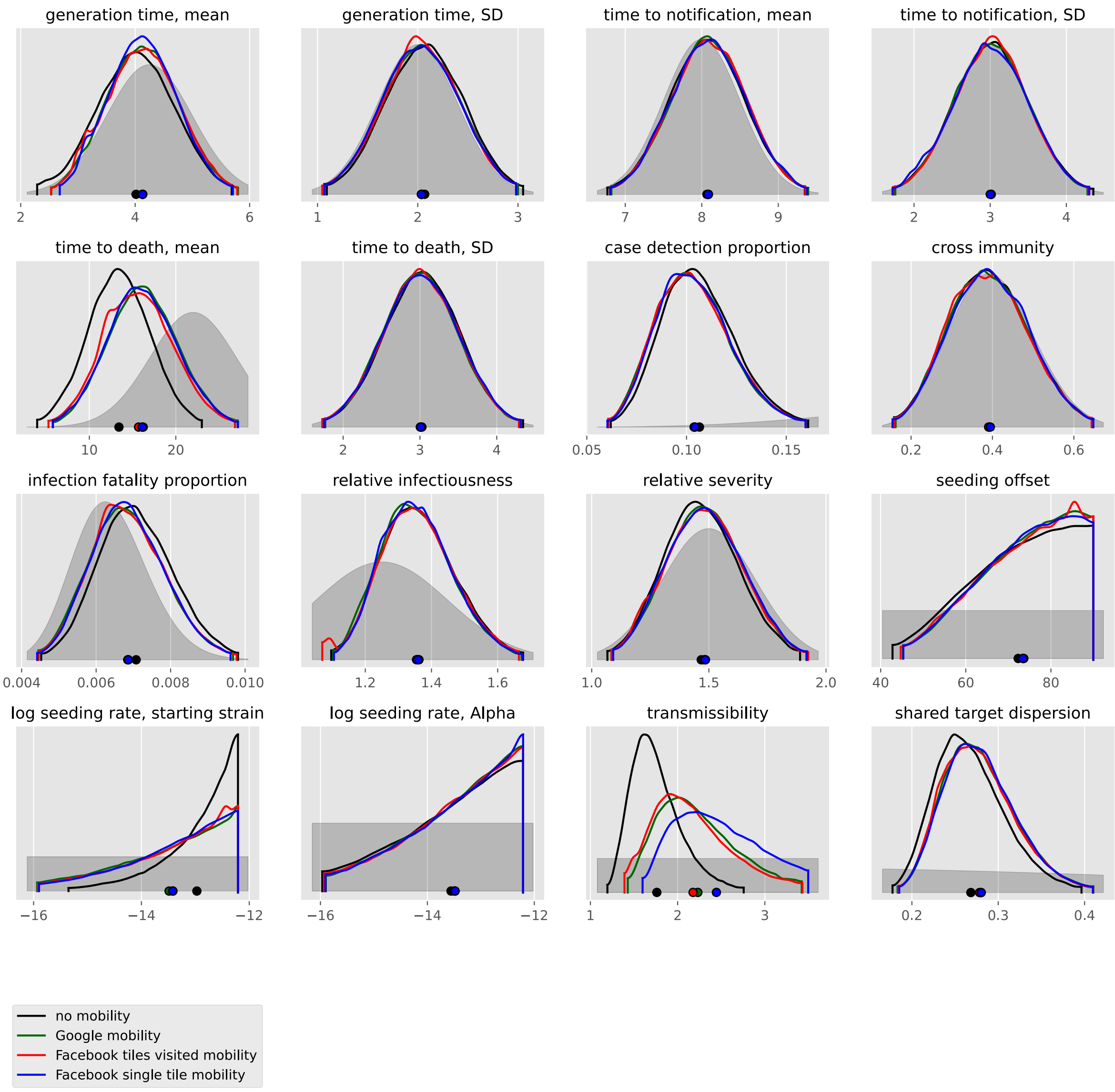
generation time, mean
generation time, SD
time to notification, mean
time to notification, SD
time to death, mean
time to death, SD
case detection proportion
cross immunity
infection fatality proportion
relative infectiousness
relative severity
seeding offset
log seeding rate, starting strain
log seeding rate, Alpha
transmissibility
shared target dispersion
no mobility
Google mobility
Facebook tiles visited mobility
Facebook single tile mobility

Suriname

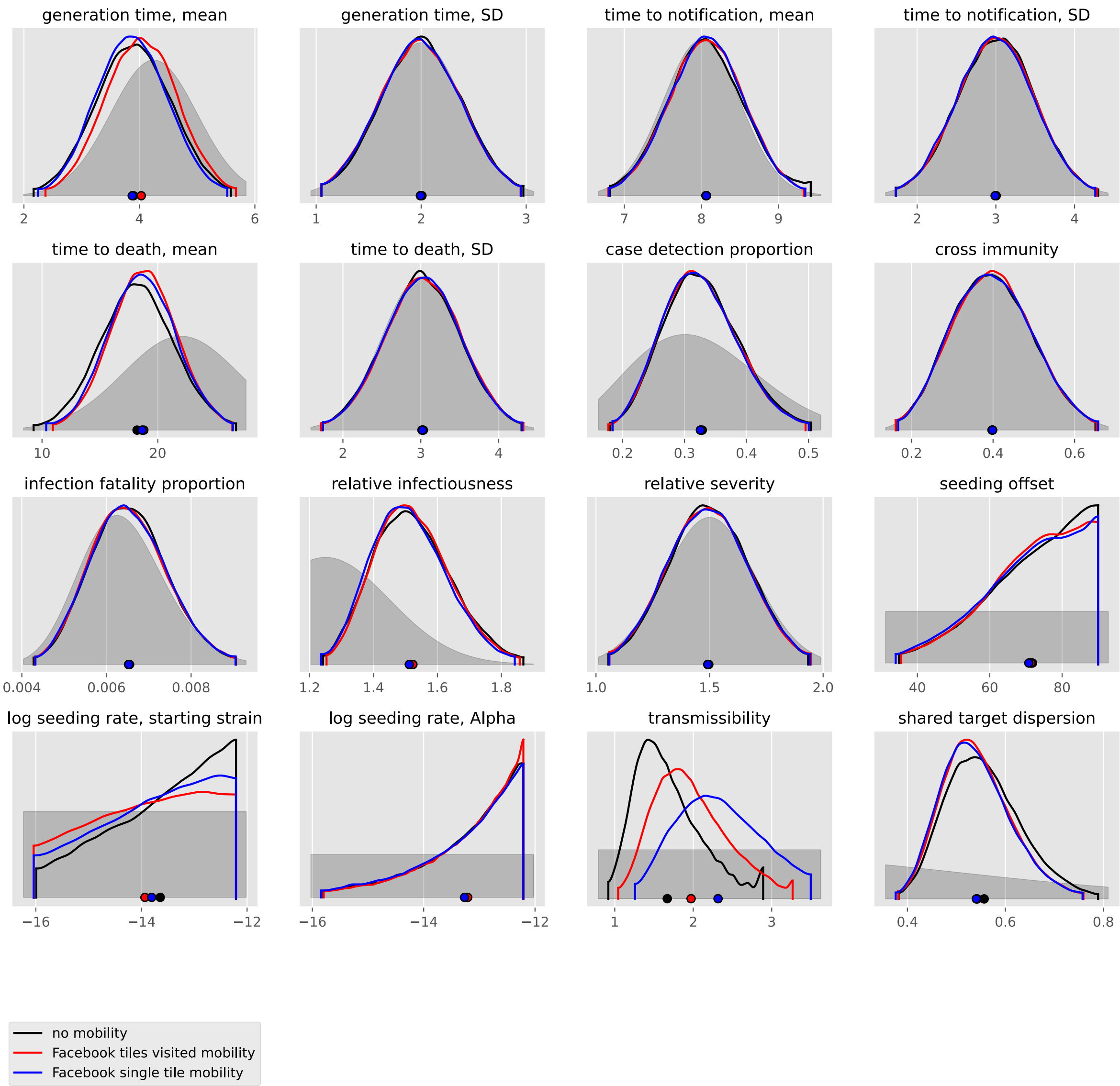

generation time, mean
2
4
6
generation time, SD
1
2
3
time to notification, mean
7
8
9
time to notification, SD
2
3
4
time to death, mean
10
20
time to death, SD
2
3
4
case detection proportion
0.2
0.3
0.4
0.5
cross immunity
0.2
0.4
0.6
infection fatality proportion
0.004
0.006
0.008
relative infectiousness
1.2
1.4
1.6
1.8
relative severity
1.0
1.5
2.0
seeding offset
40
60
80
log seeding rate, starting strain
−16
−14
−12
log seeding rate, Alpha
−16
−14
−12
transmissibility
1
2
3
shared target dispersion
0.4
0.6
0.8
no mobility
Facebook tiles visited mobility
Facebook single tile mobility

Uruguay

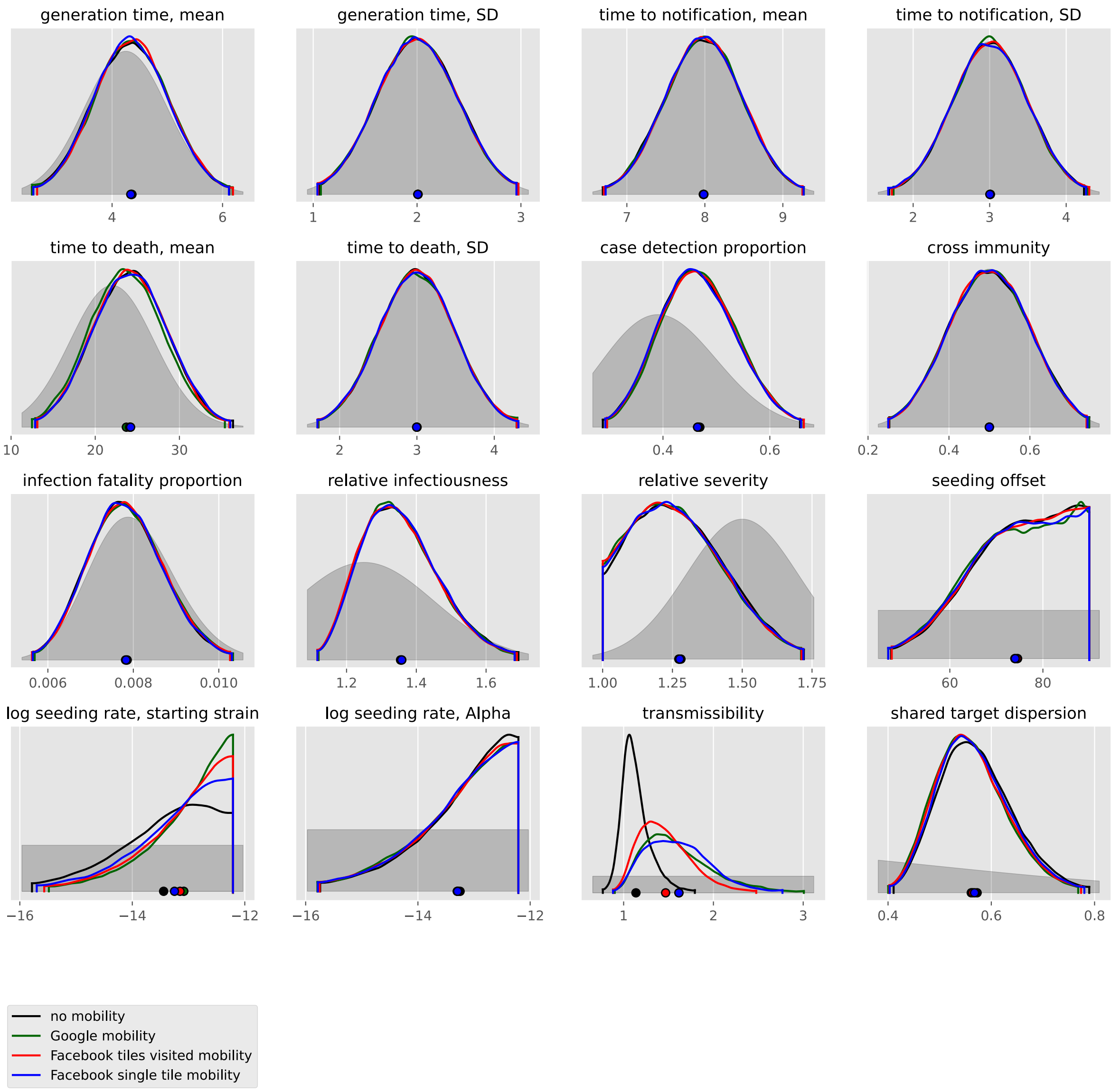

generation time, mean
generation time, SD
time to notification, mean
time to notification, SD
time to death, mean
time to death, SD
case detection proportion
cross immunity
infection fatality proportion
relative infectiousness
relative severity
seeding offset
log seeding rate, starting strain
log seeding rate, Alpha
transmissibility
shared target dispersion
no mobility
Google mobility
Facebook tiles visited mobility
Facebook single tile mobility

Venezuela, Bolivarian Republic of

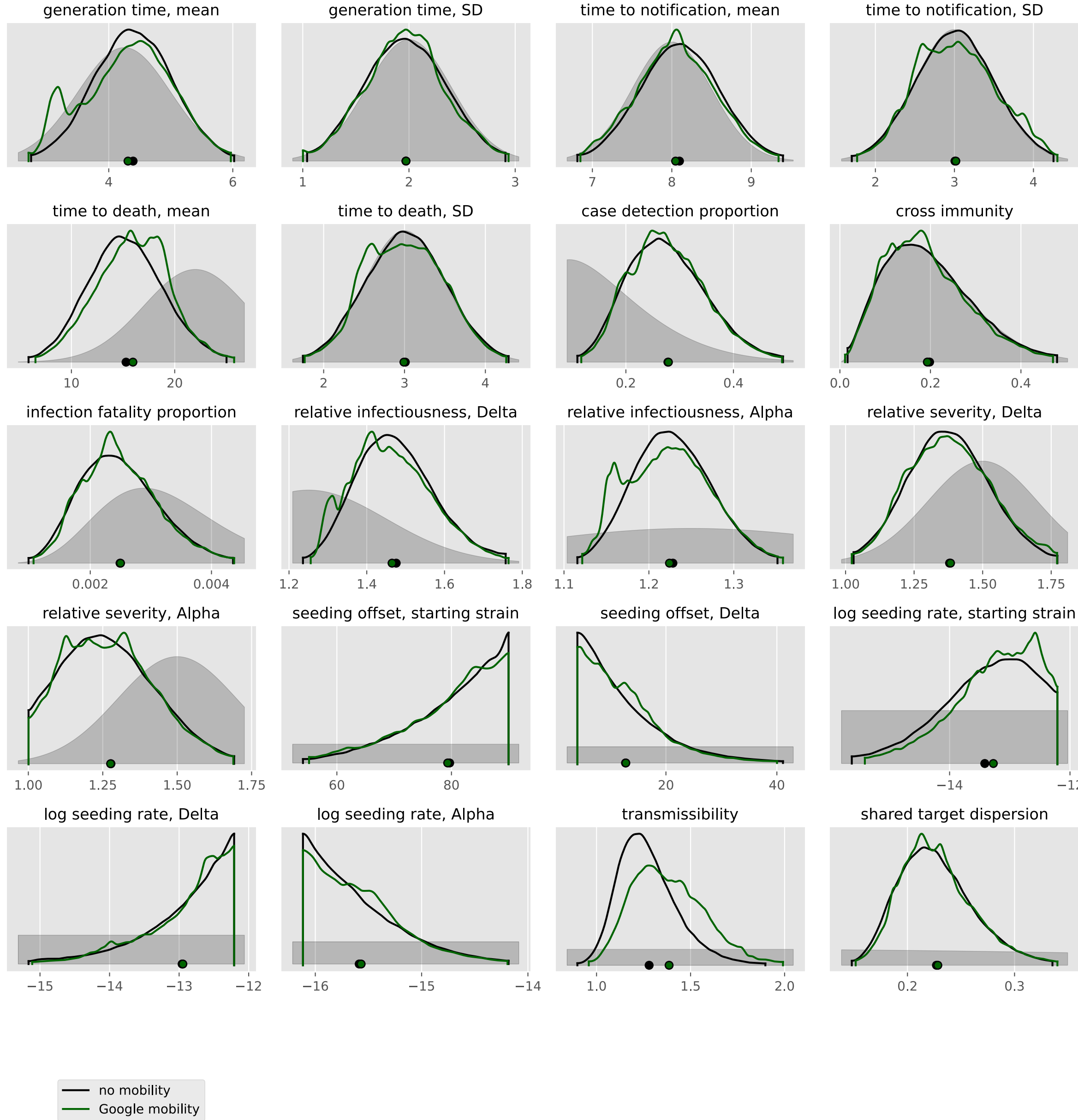

Comparison of the non-mechanistic scaling of transmission process with mobility domains

This section presents the residual transmission scaling (with uncertainty) implemented without scaling for mobility for each country. It is intended to provide a raw comparison between the residual non-mechanistic transmission that needed to be applied for each analysis in the absence of mobility scaling and the various mobility data fields available from both Google and Facebook. We also show comparisons against the composite Google mobility metric after weighting, with associated credible intervals. From this section onward, results are presented for the model configuration without waning immunity included.

Individual mobility metric comparisons (Google and Facebook)

Google retail and recreation mobility metric comparison

Mobility values presented as Google data divided by 100, plus one.

Africa

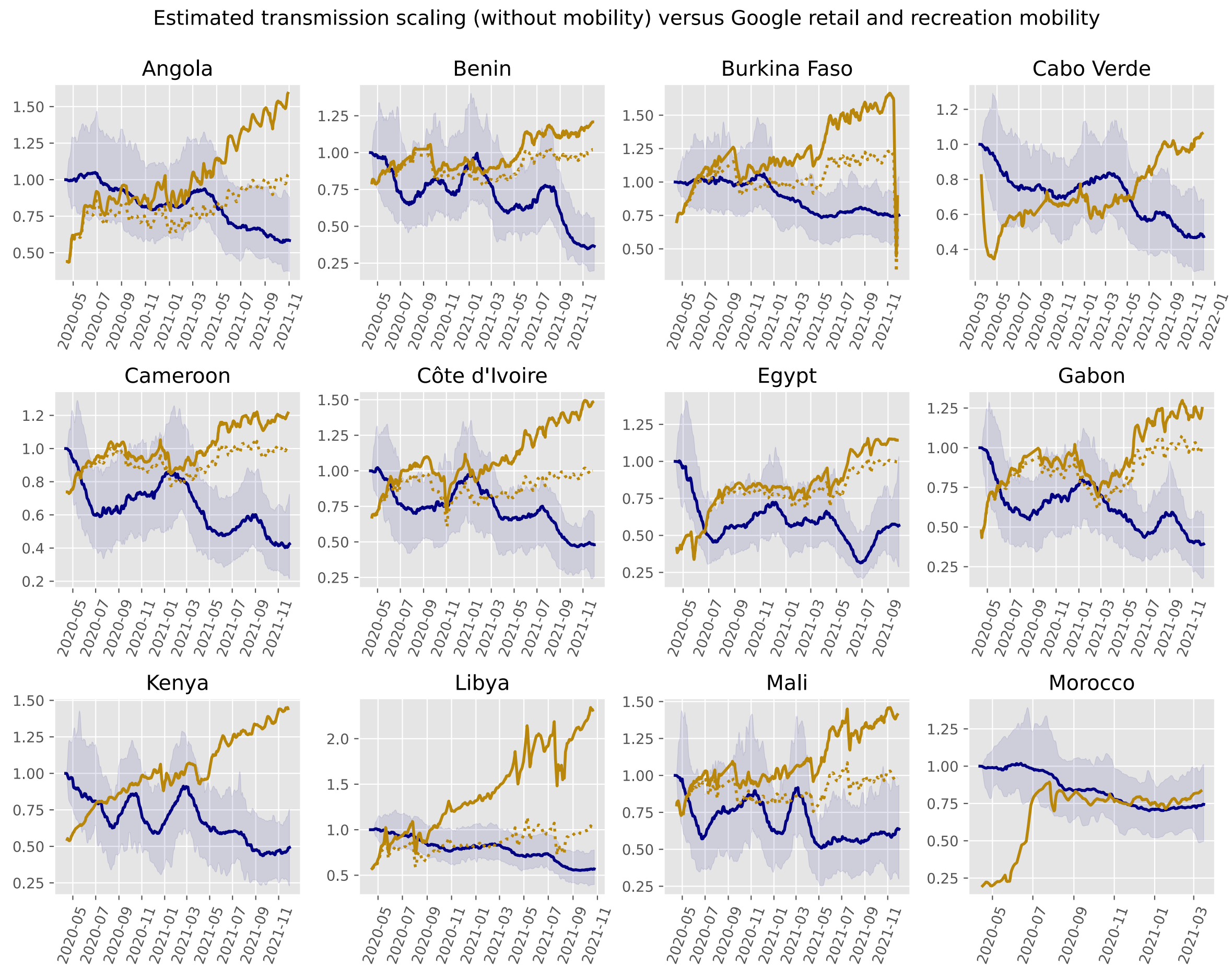

Estimated transmission scaling (without mobility) versus Google retail and recreation mobility

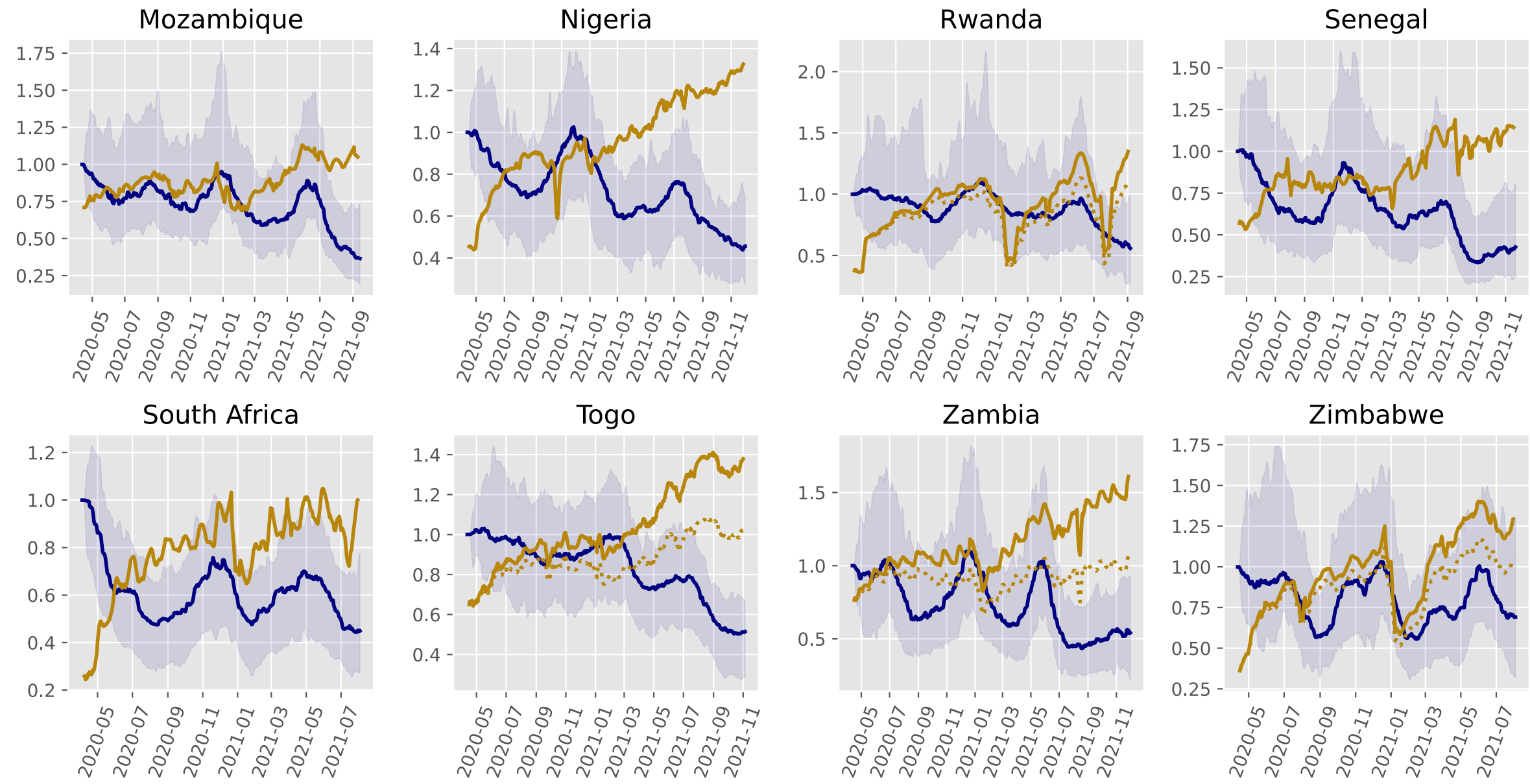

Asia

Estimated transmission scaling (without mobility) versus Google retail and recreation mobility

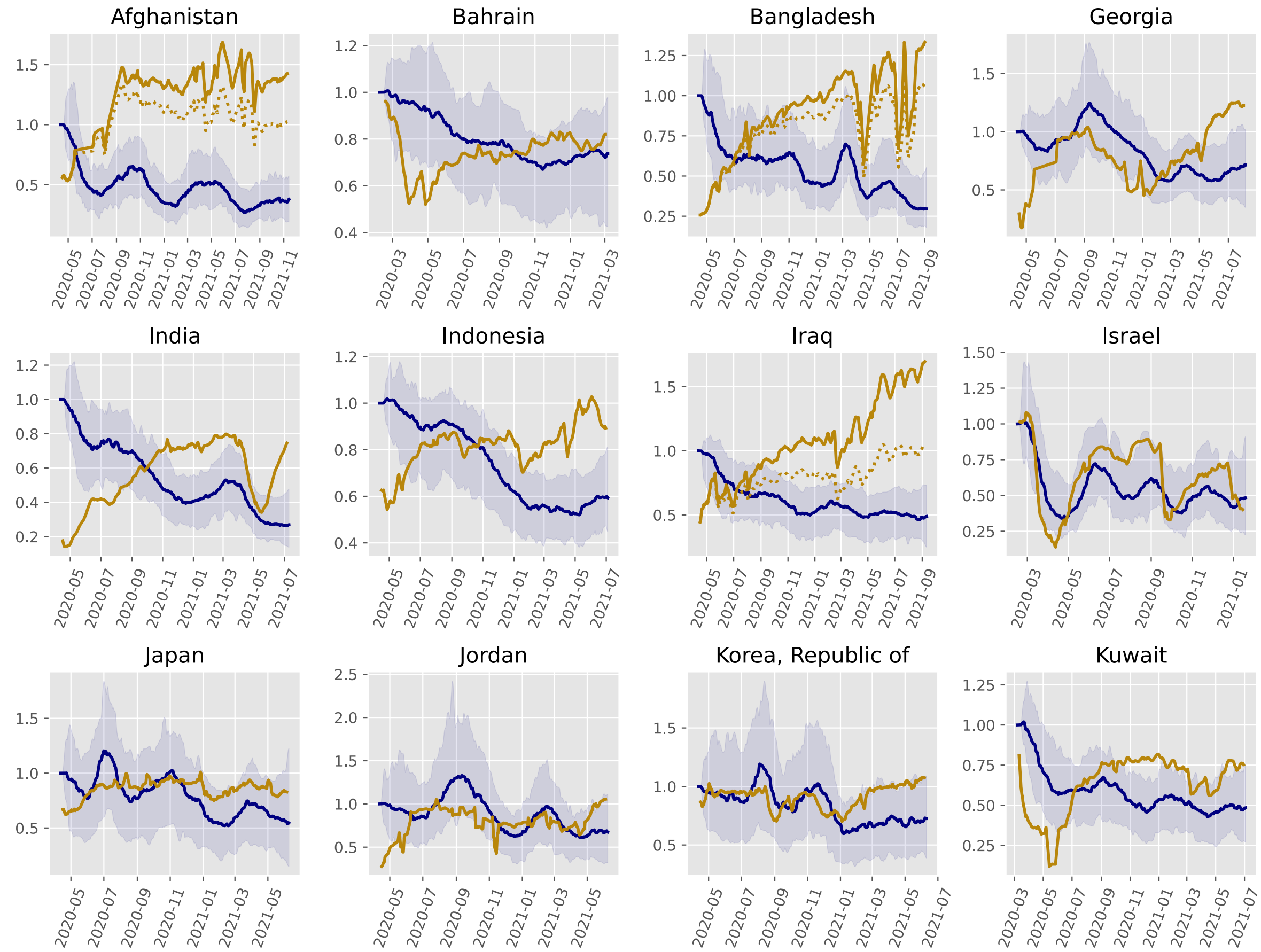

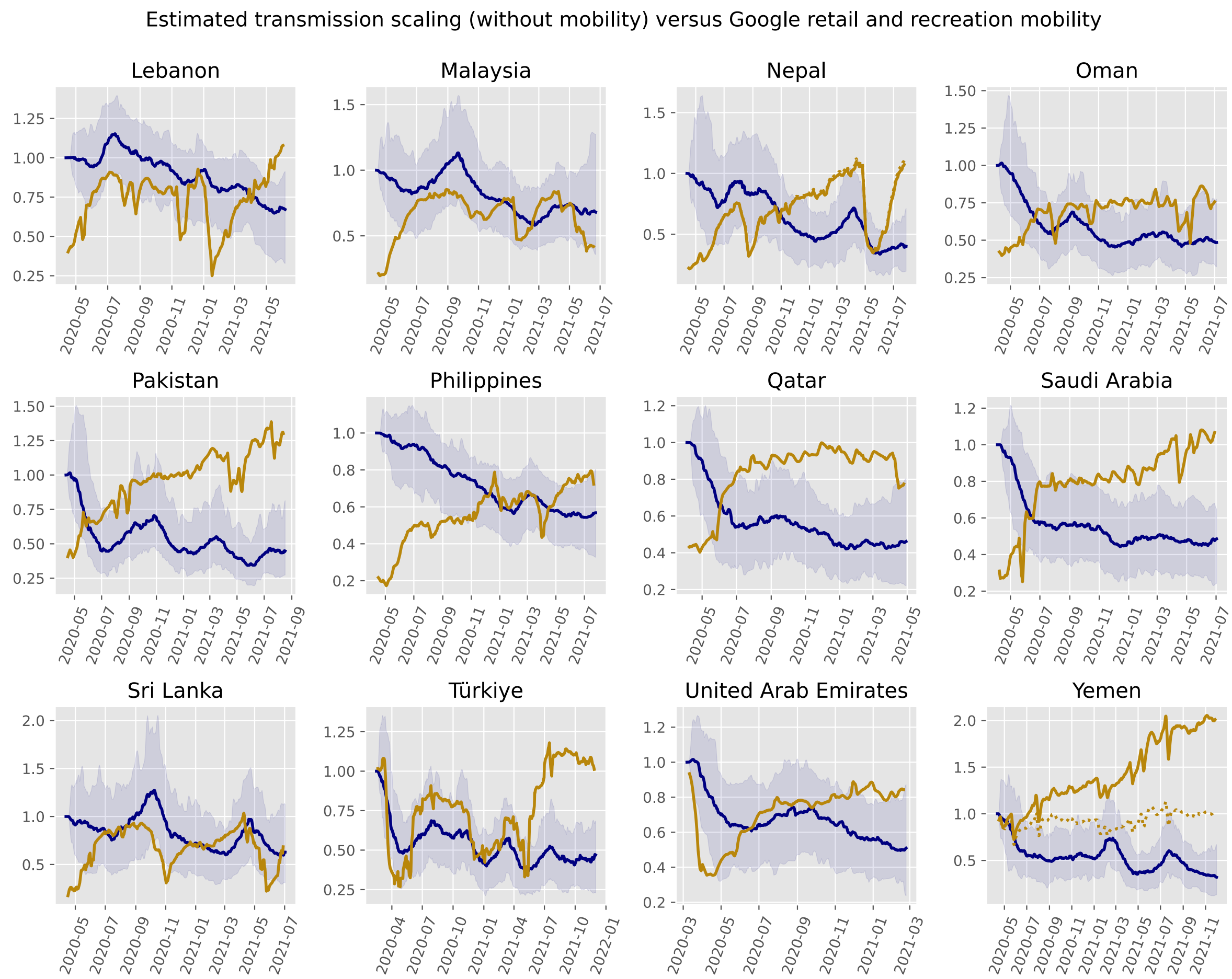
Estimated transmission scaling (without mobility) versus Google retail and recreation mobility
Lebanon
Malaysia
Nepal
Oman
Pakistan
Philippines
Qatar
Saudi Arabia
Sri Lanka
Türkiye
United Arab Emirates
Yemen

Europe

Estimated transmission scaling (without mobility) versus Google retail and recreation mobility

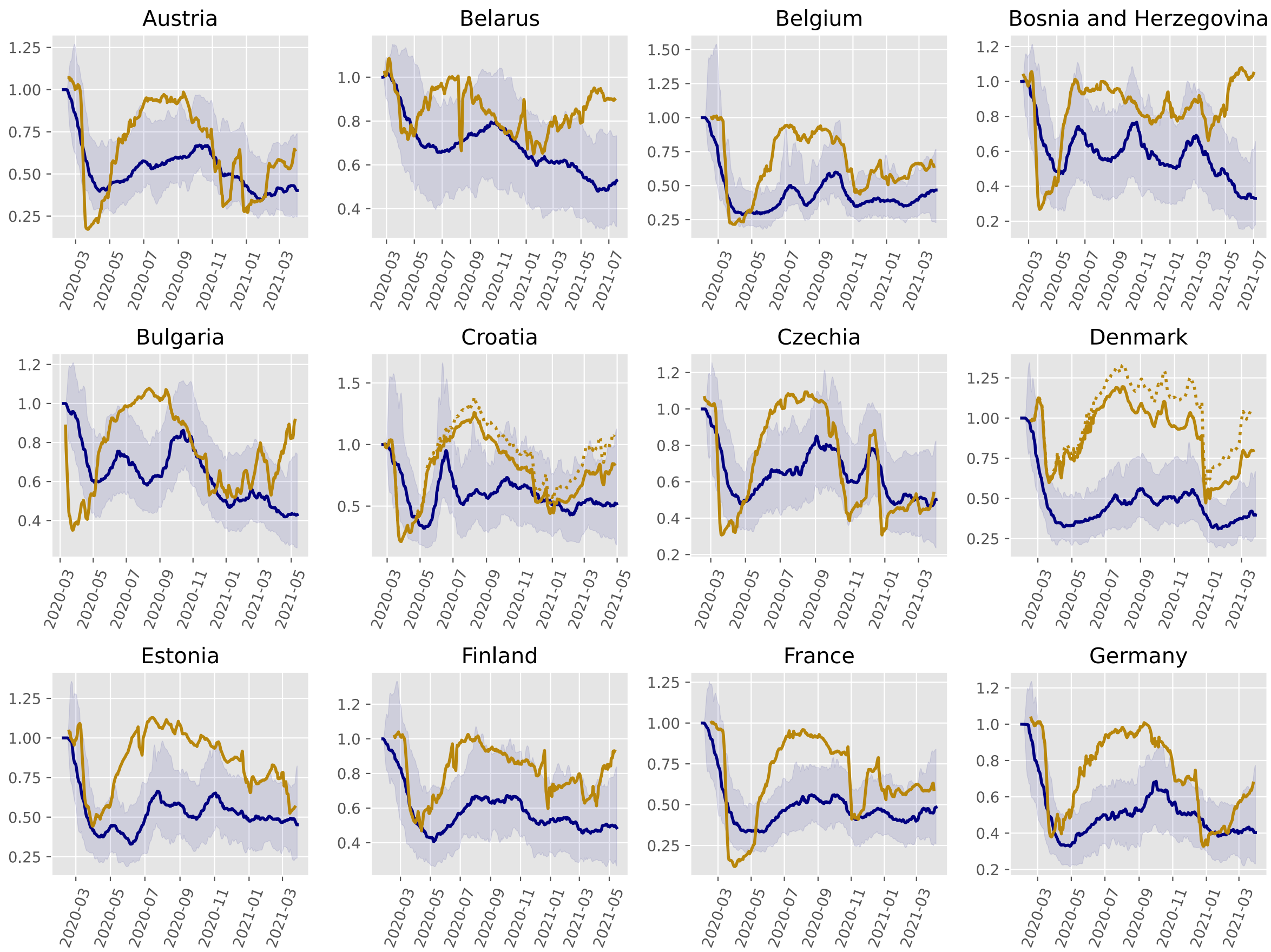

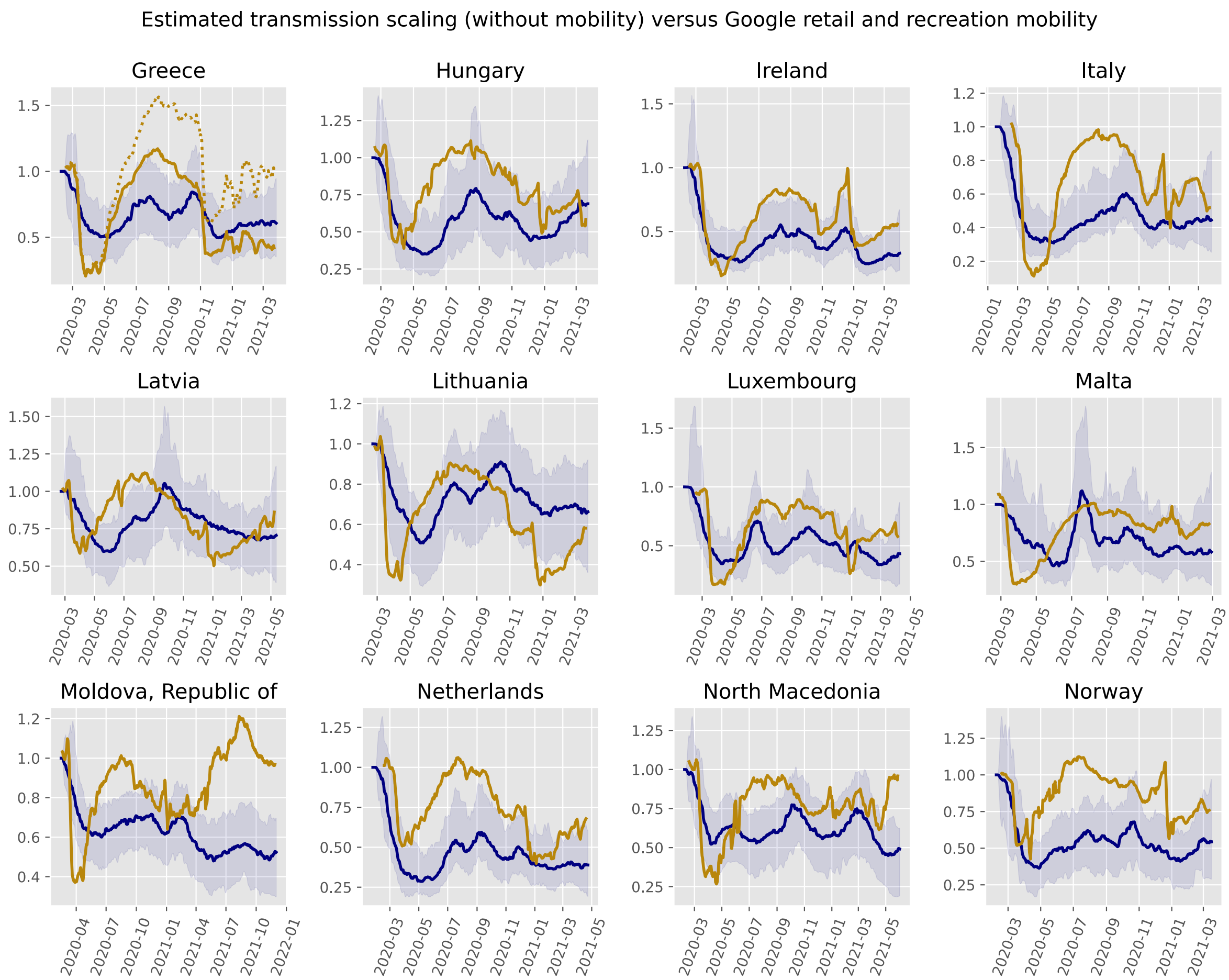
Estimated transmission scaling (without mobility) versus Google retail and recreation mobility
Greece
Hungary
Ireland
Italy
Latvia
Lithuania
Luxembourg
Malta
Moldova, Republic of
Netherlands
North Macedonia
Norway

Estimated transmission scaling (without mobility) versus Google retail and recreation mobility

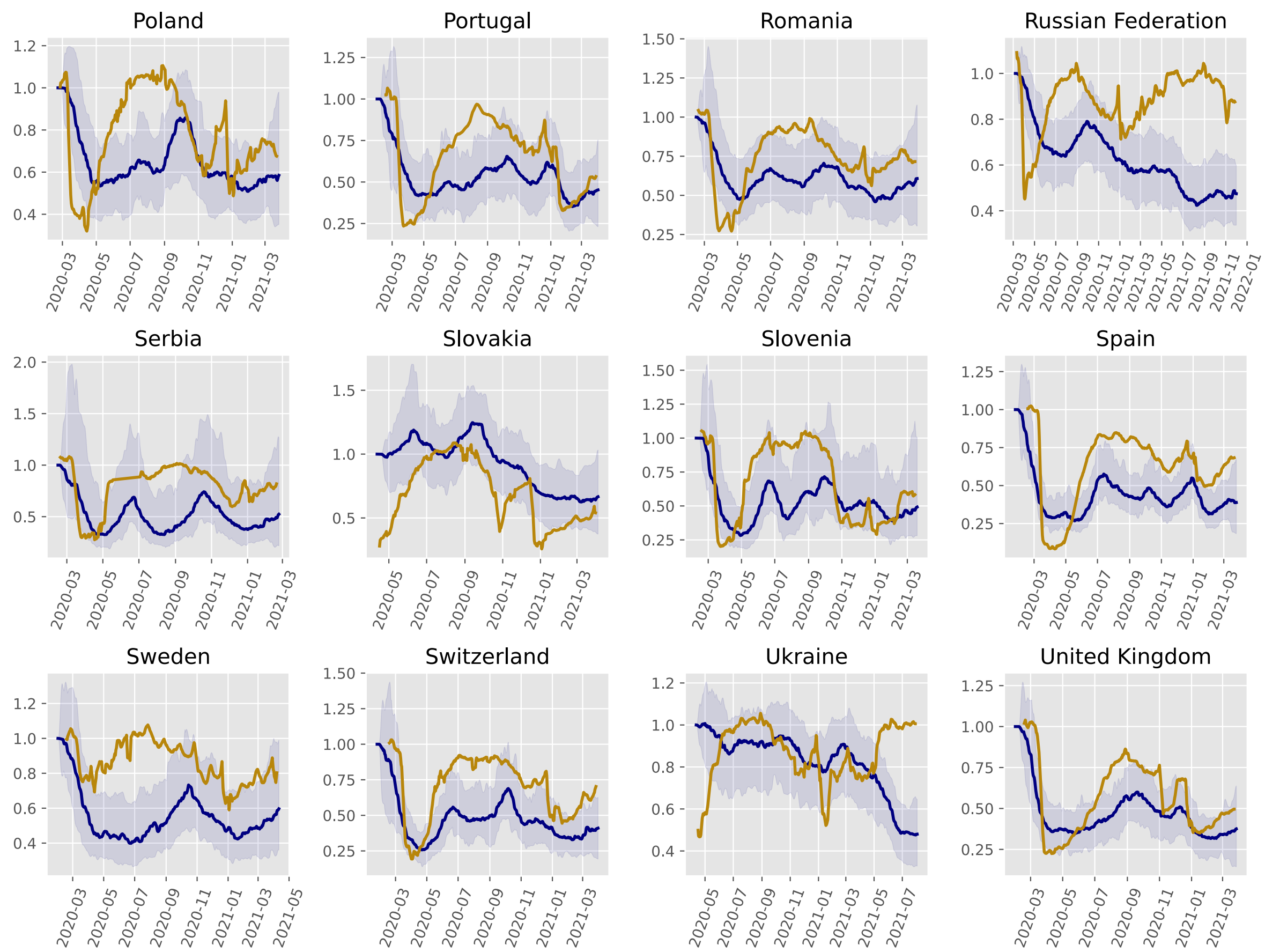

North America

Estimated transmission scaling (without mobility) versus Google retail and recreation mobility

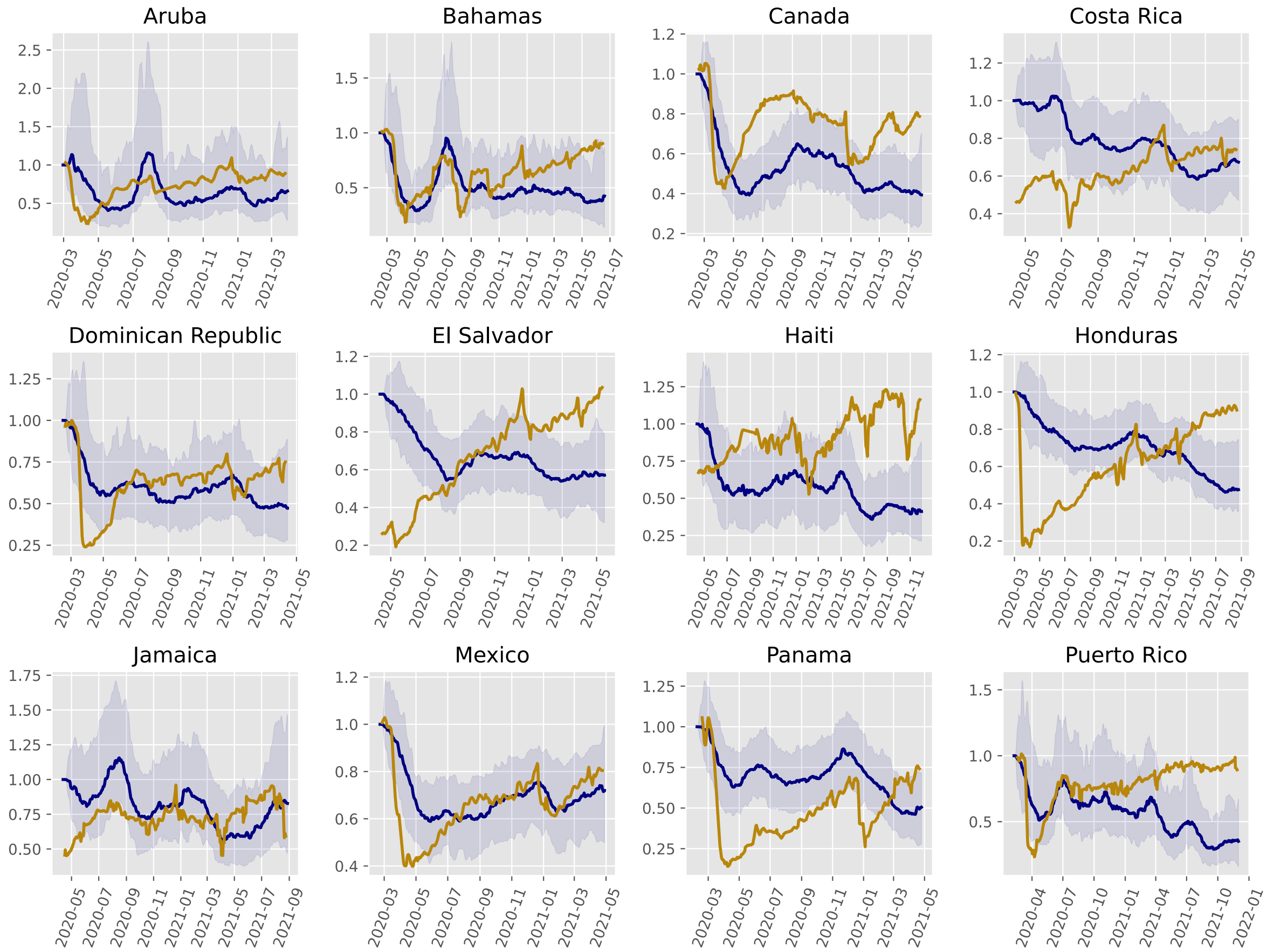

Estimated transmission scaling (without mobility) versus Google retail and recreation mobility

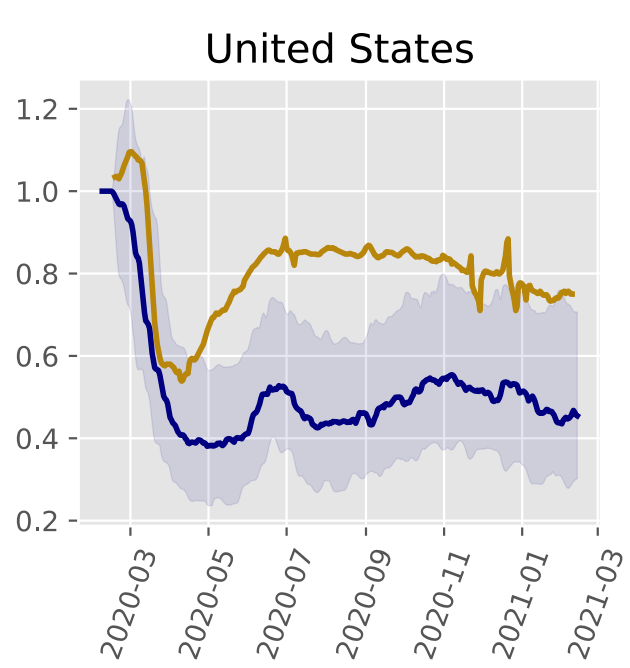

Oceania

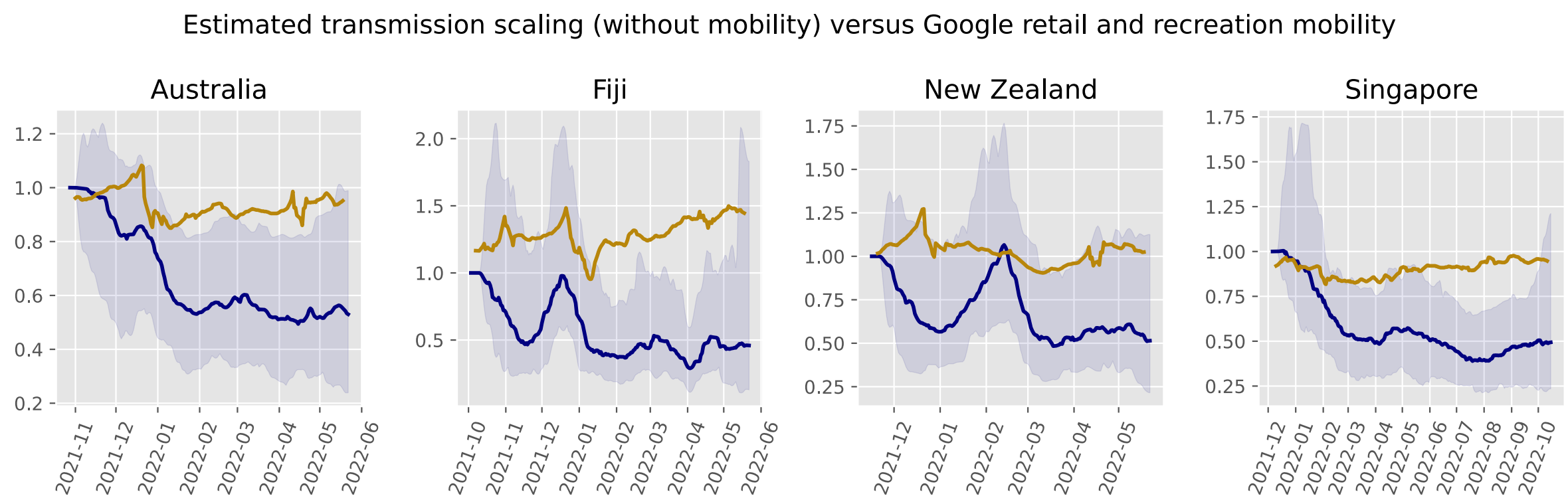

South America

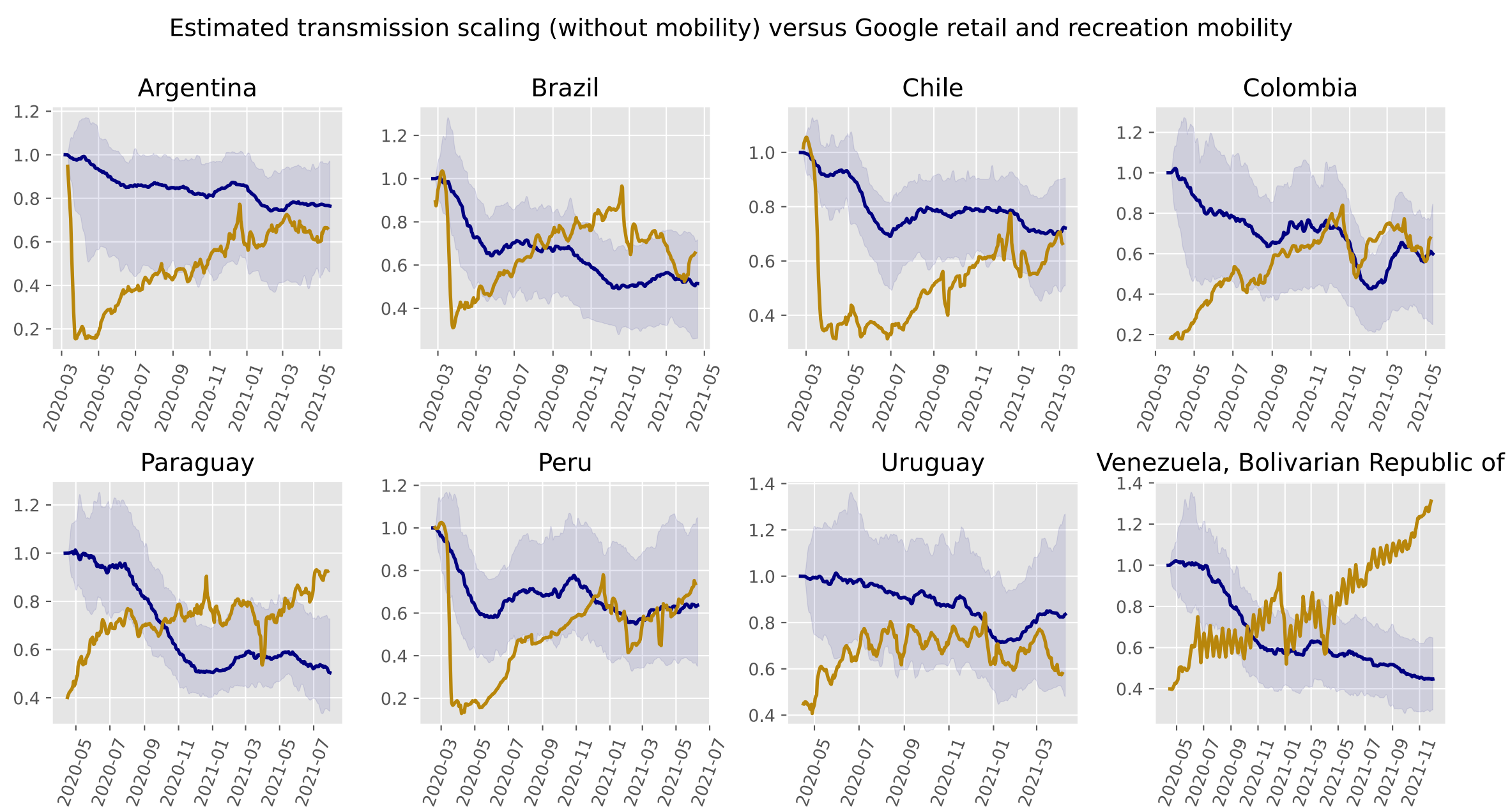

Google grocery and pharmacy mobility metric comparison

Mobility values presented as Google data divided by 100, plus one.

Africa

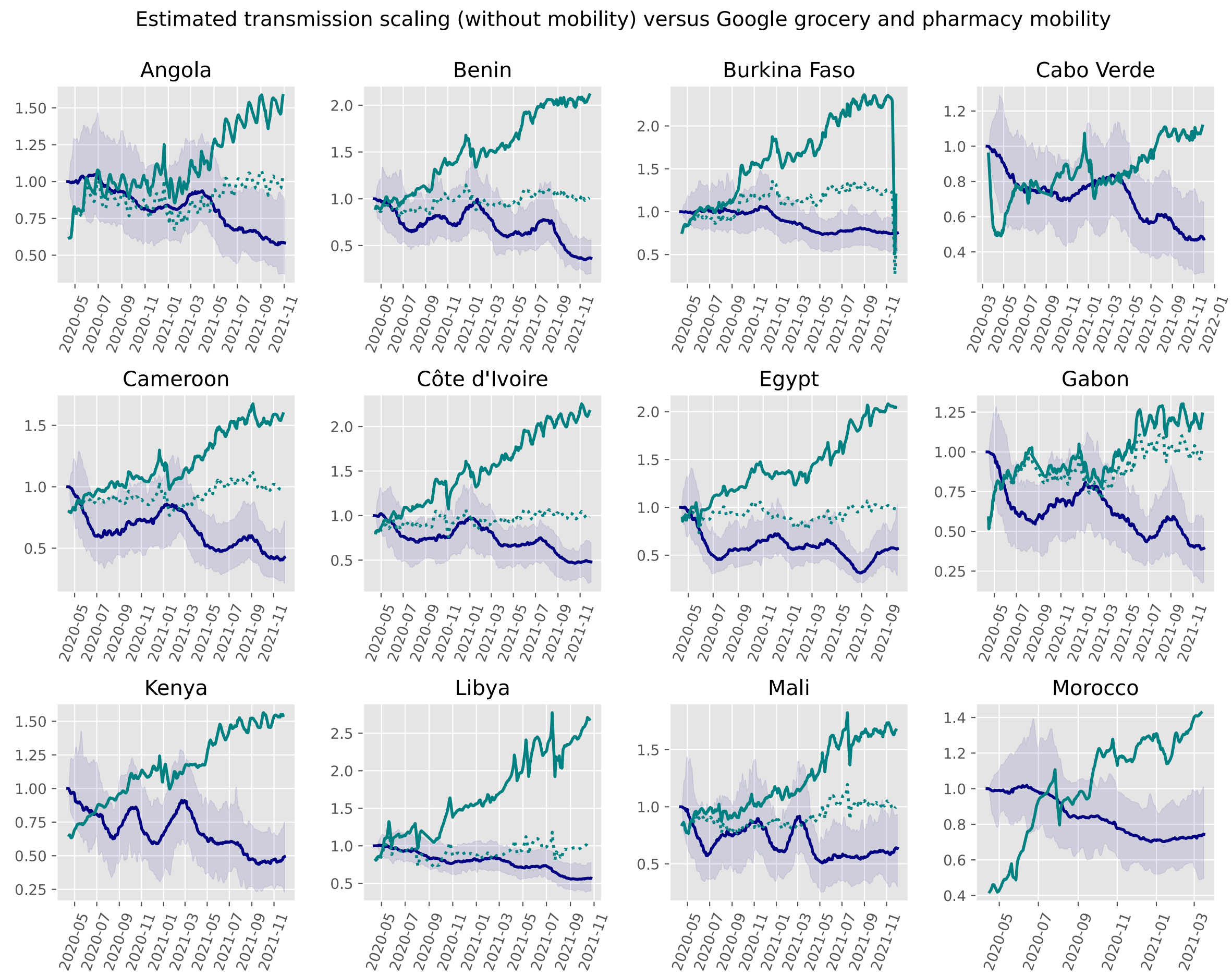

Estimated transmission scaling (without mobility) versus Google grocery and pharmacy mobility

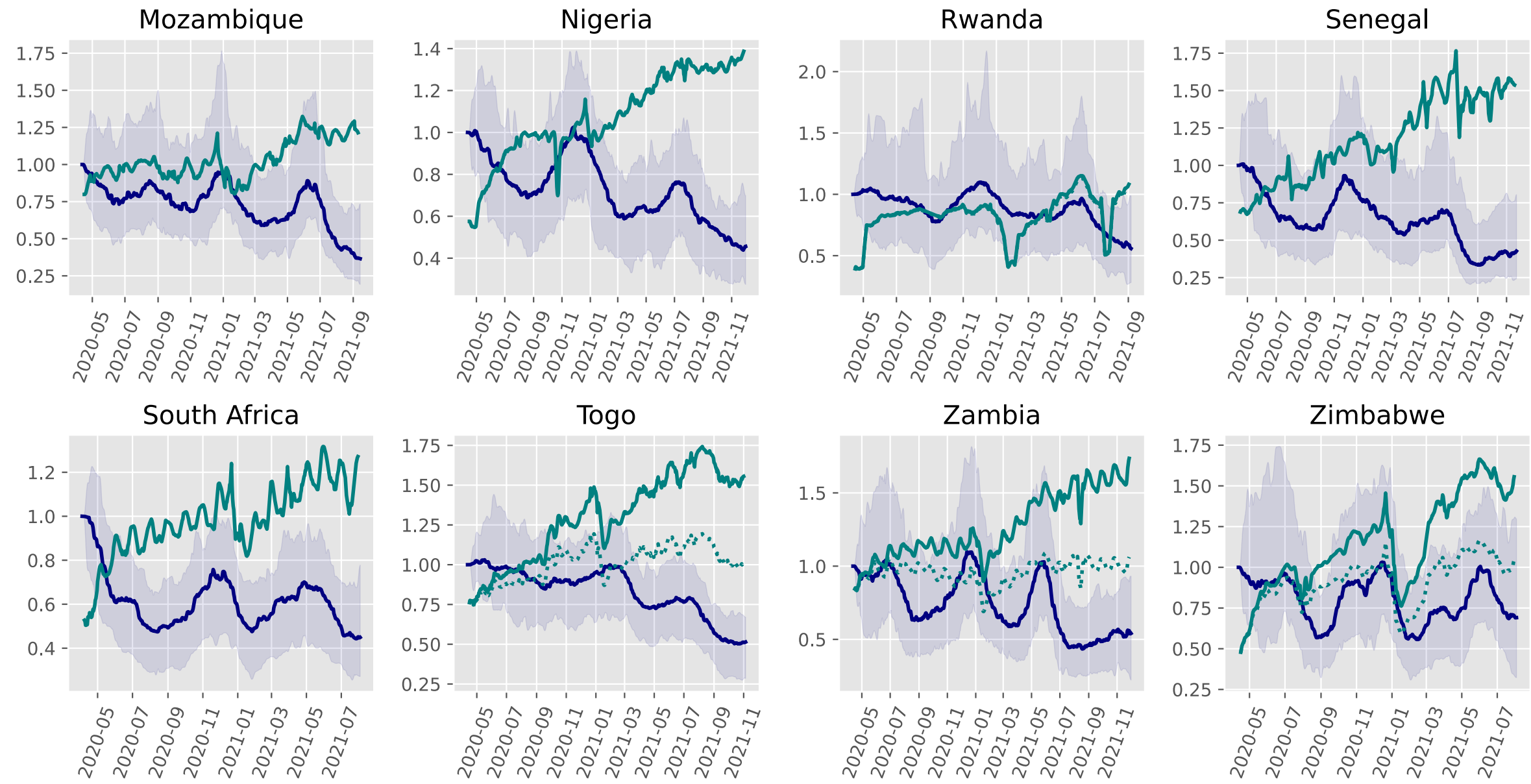

Asia

Estimated transmission scaling (without mobility) versus Google grocery and pharmacy mobility

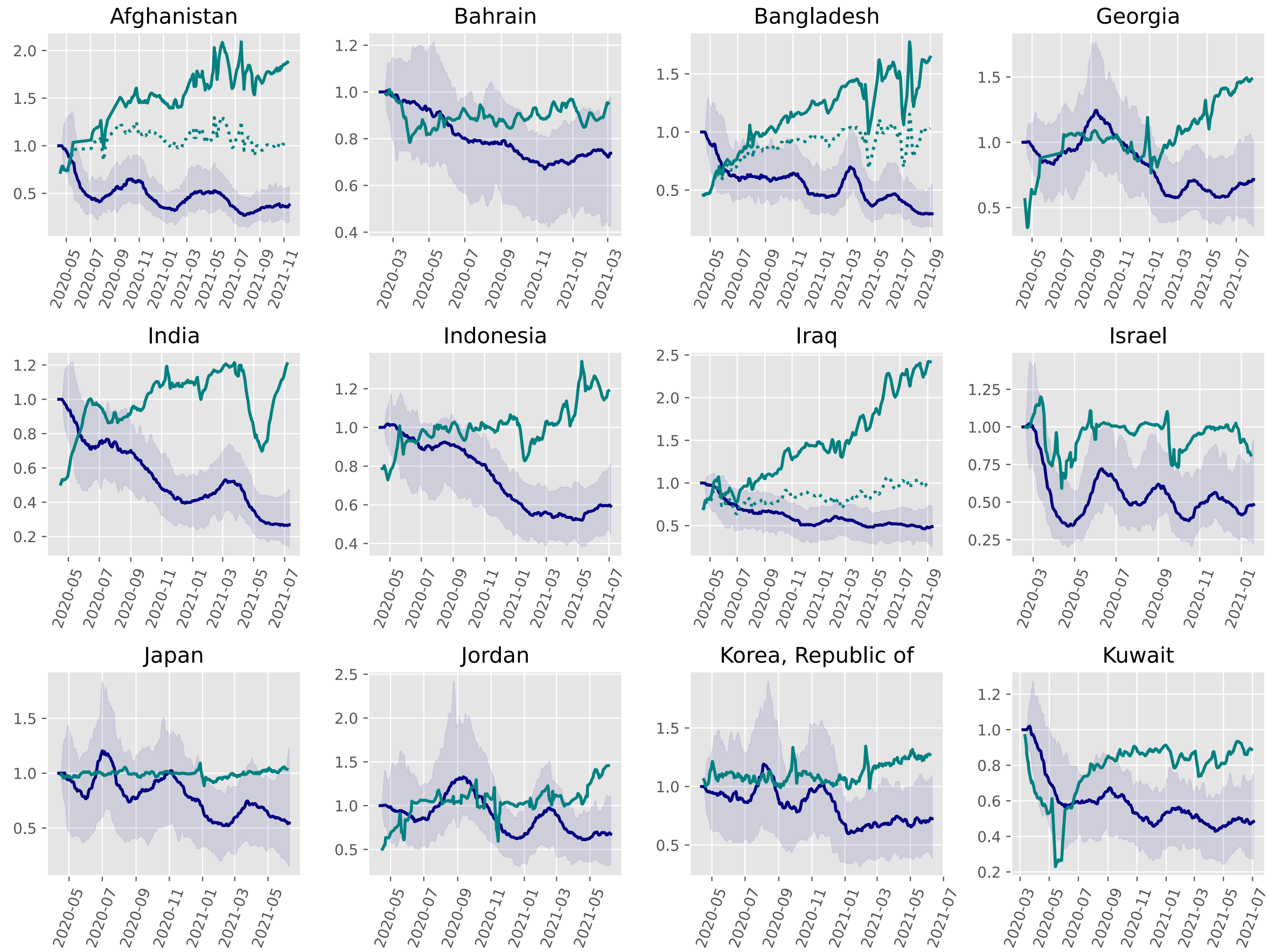

Estimated transmission scaling (without mobility) versus Google grocery and pharmacy mobility

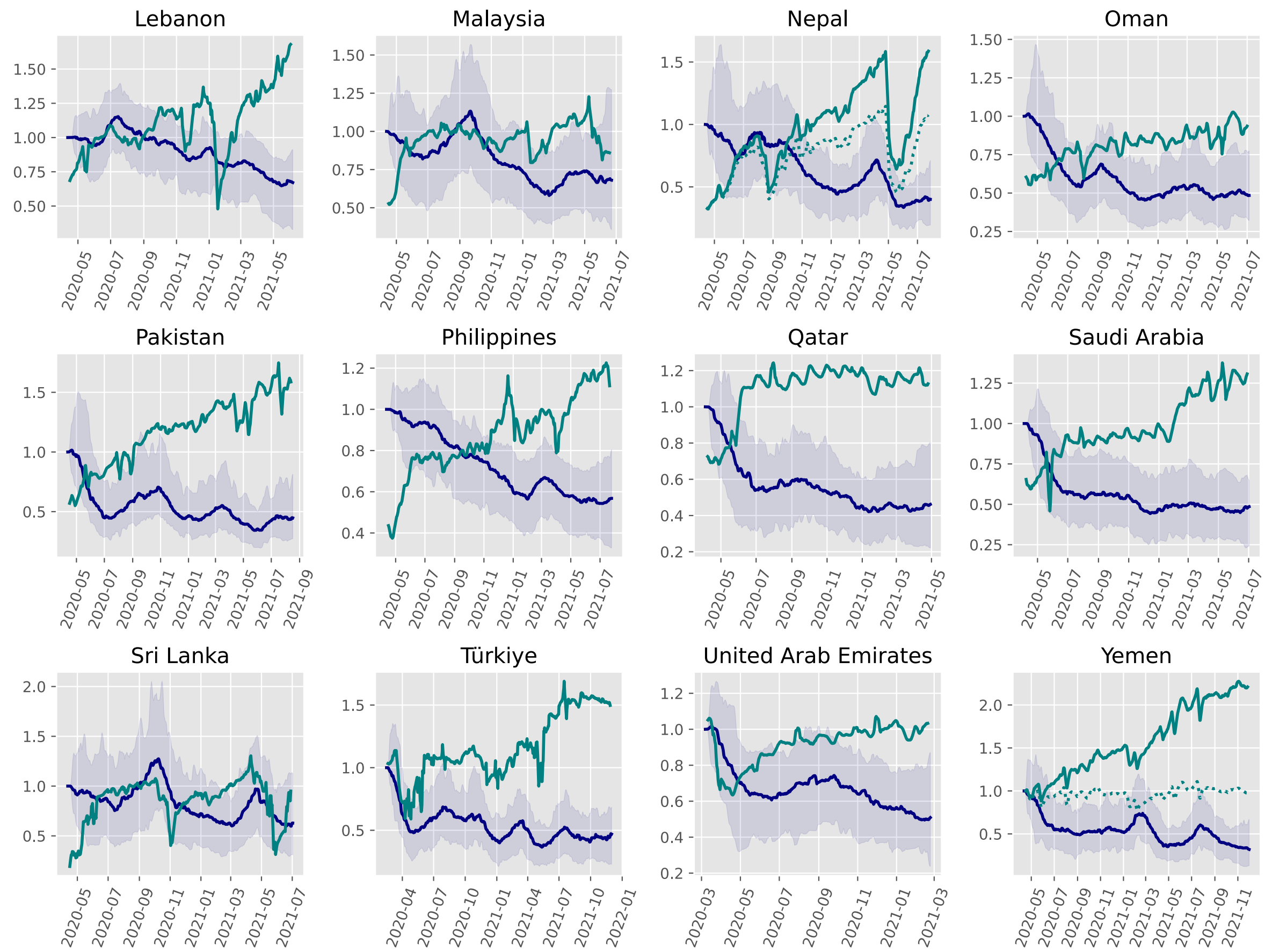

Europe

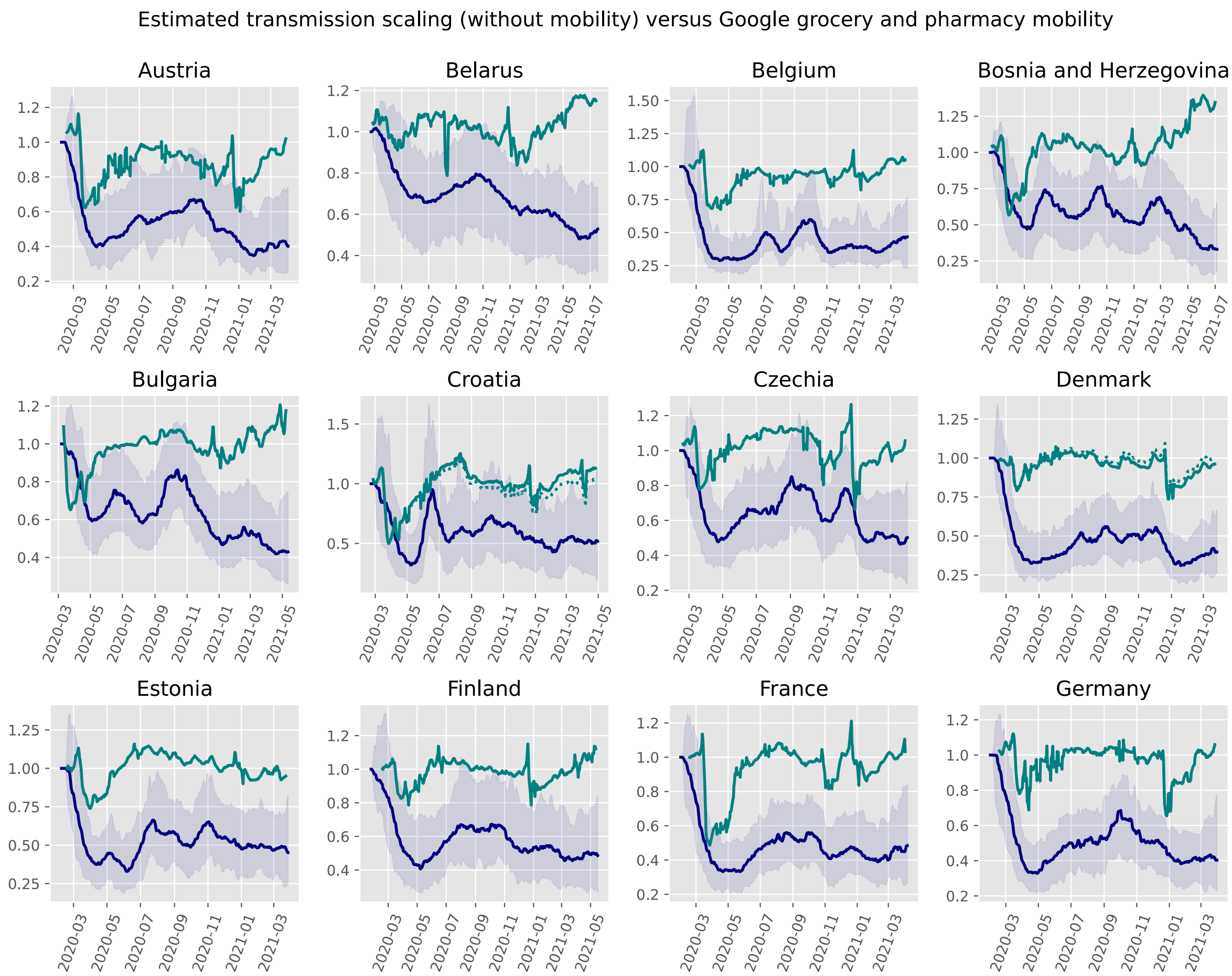
Estimated transmission scaling (without mobility) versus Google grocery and pharmacy mobility
Austria
Belarus
Belgium
Bosnia and Herzegovina
Bulgaria
Croatia
Czechia
Denmark
Estonia
Finland
France
Germany
2020-03
2020-05
2020-07
2020-09
2020-11
2021-01
2021-03
2021-05
2021-07

Estimated transmission scaling (without mobility) versus Google grocery and pharmacy mobility

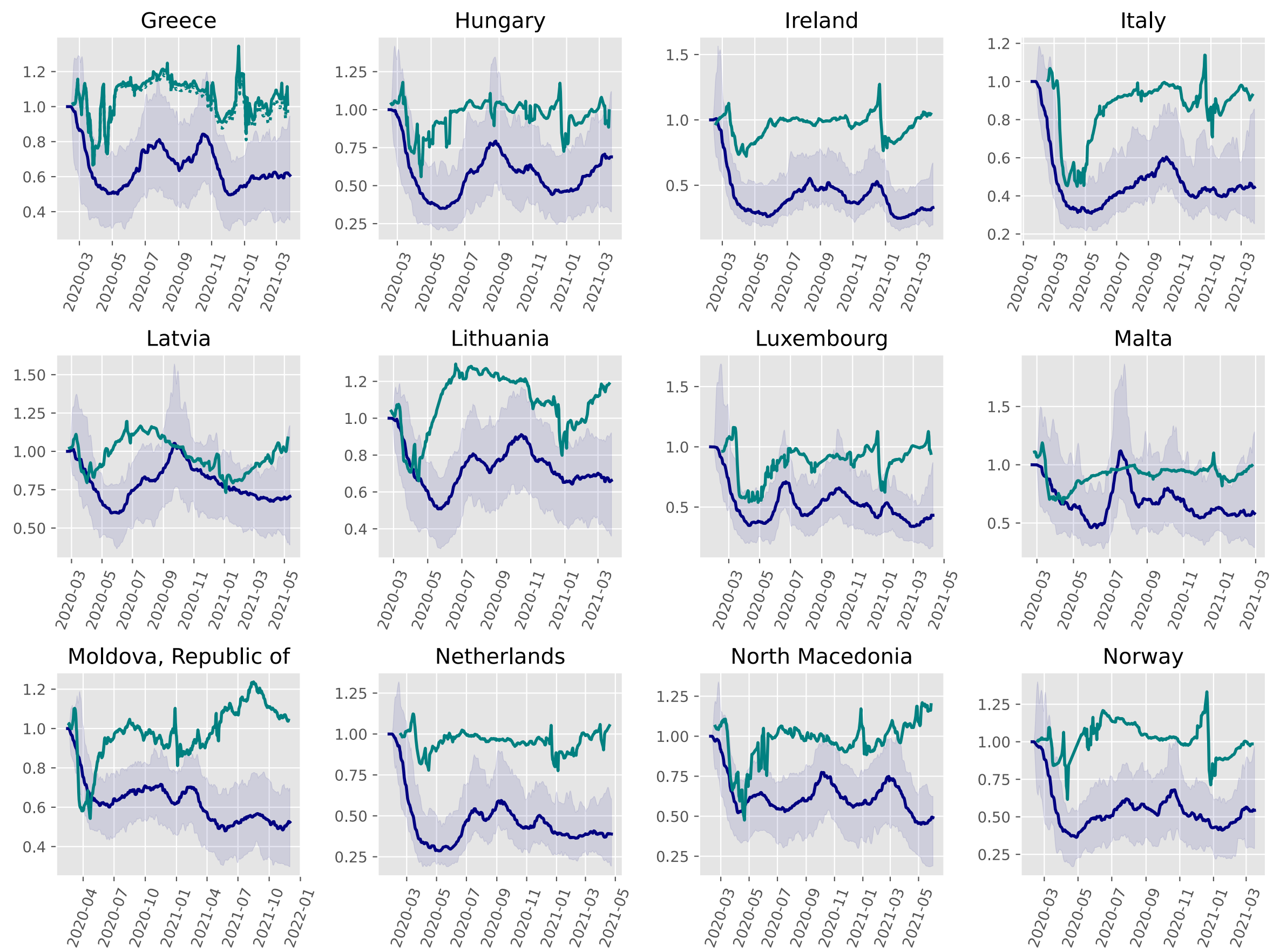

Estimated transmission scaling (without mobility) versus Google grocery and pharmacy mobility

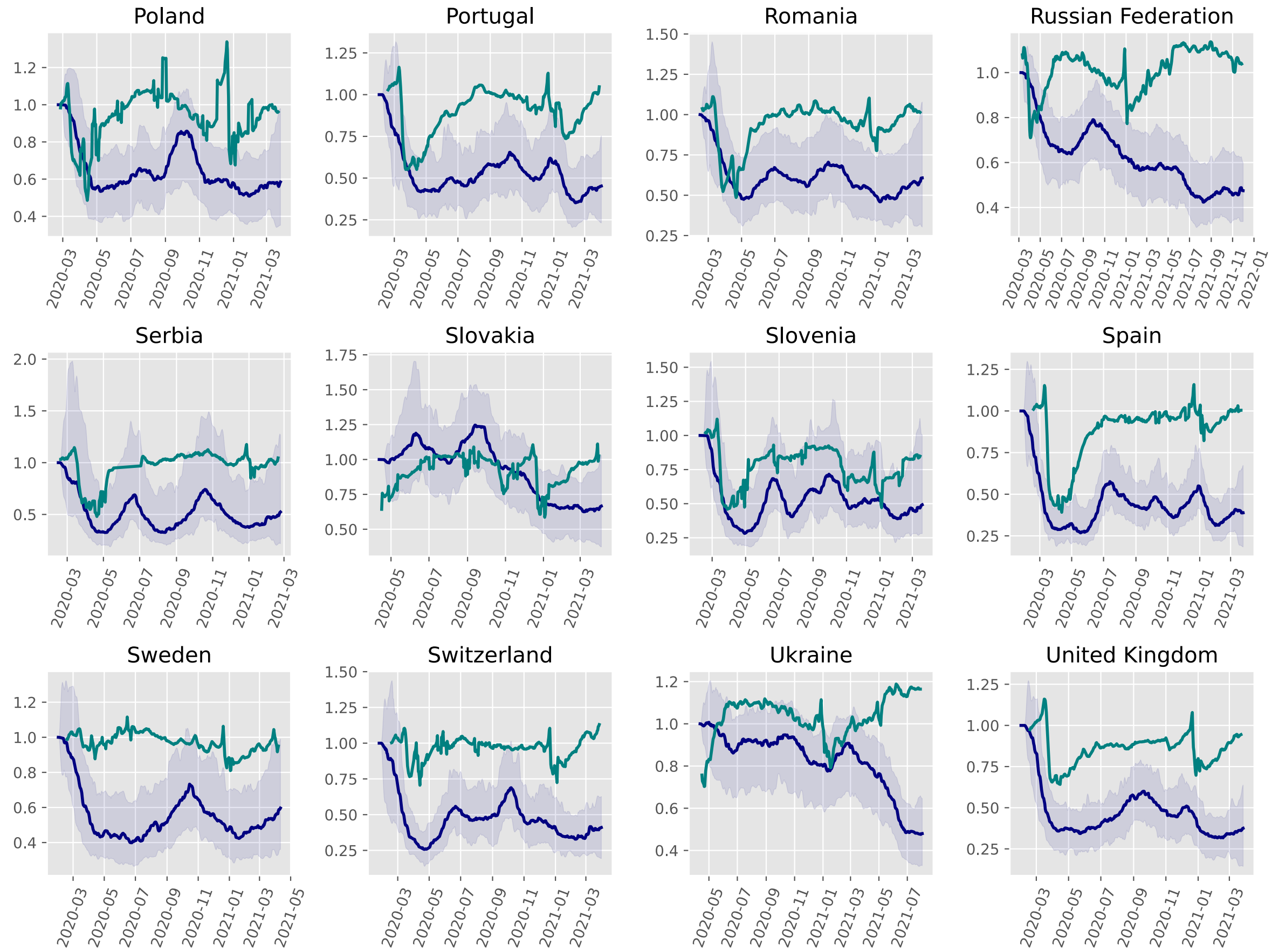

North America

Estimated transmission scaling (without mobility) versus Google grocery and pharmacy mobility

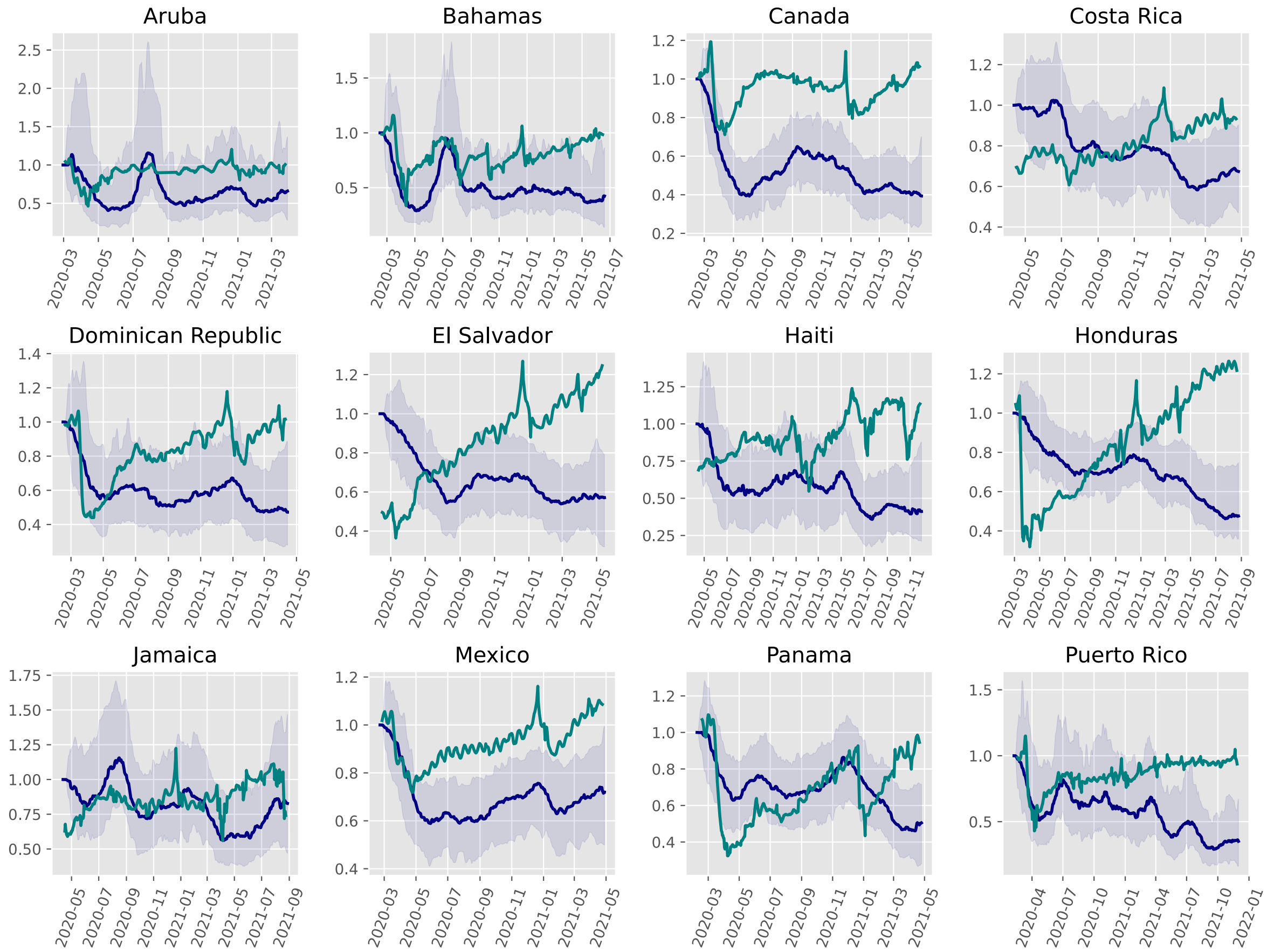

Estimated transmission scaling (without mobility) versus Google grocery and pharmacy mobility

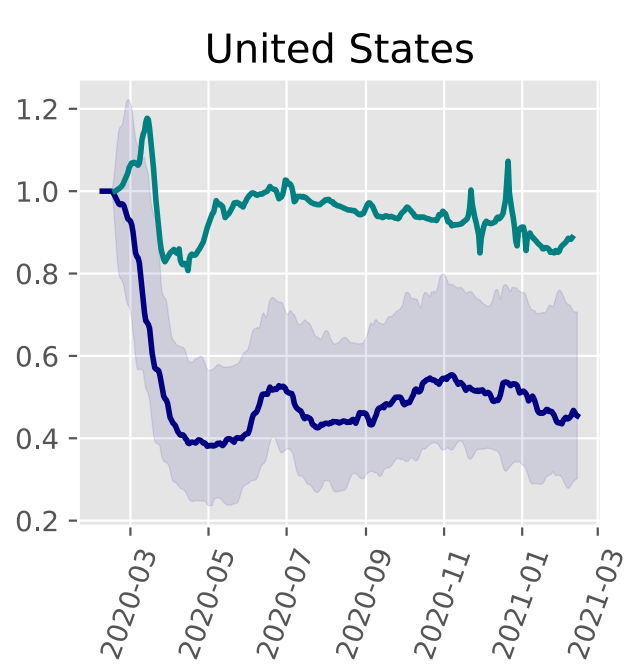

Oceania

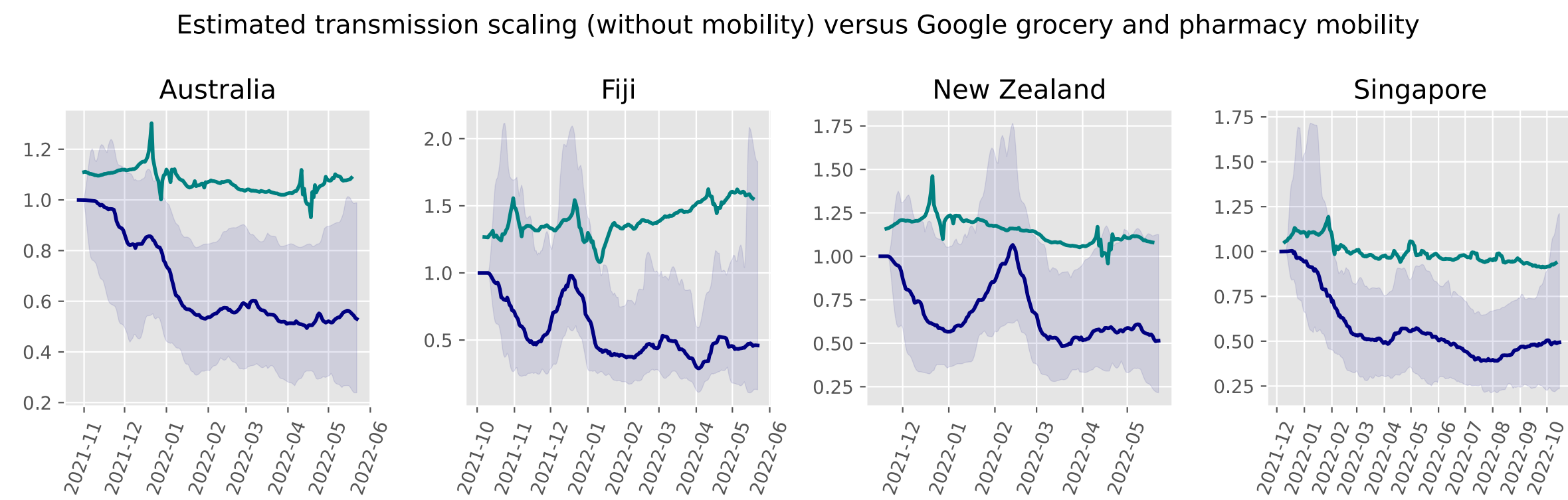

South America

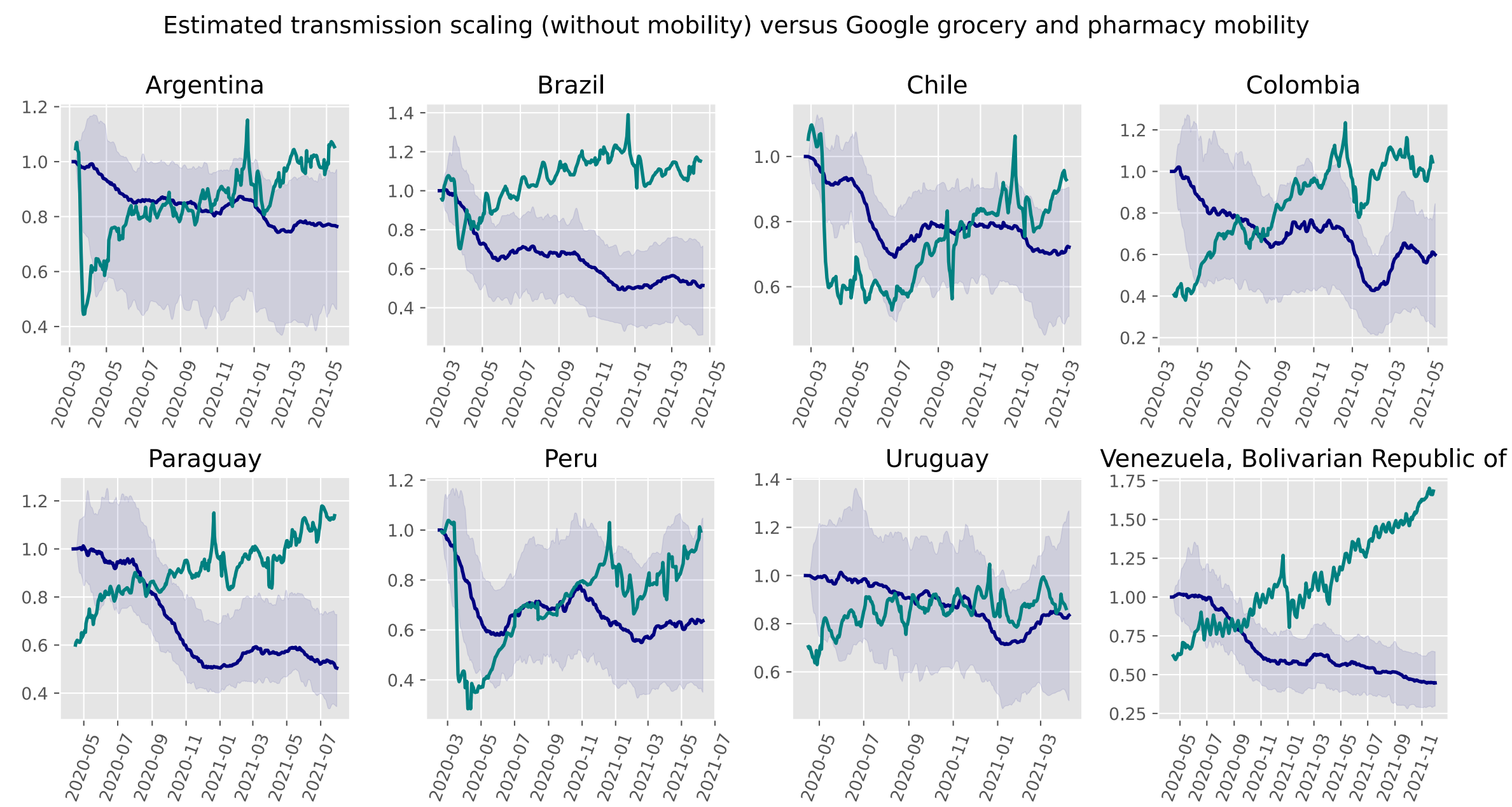

Google parks mobility metric comparison

Mobility values presented as Google data divided by 100, plus one.

Africa

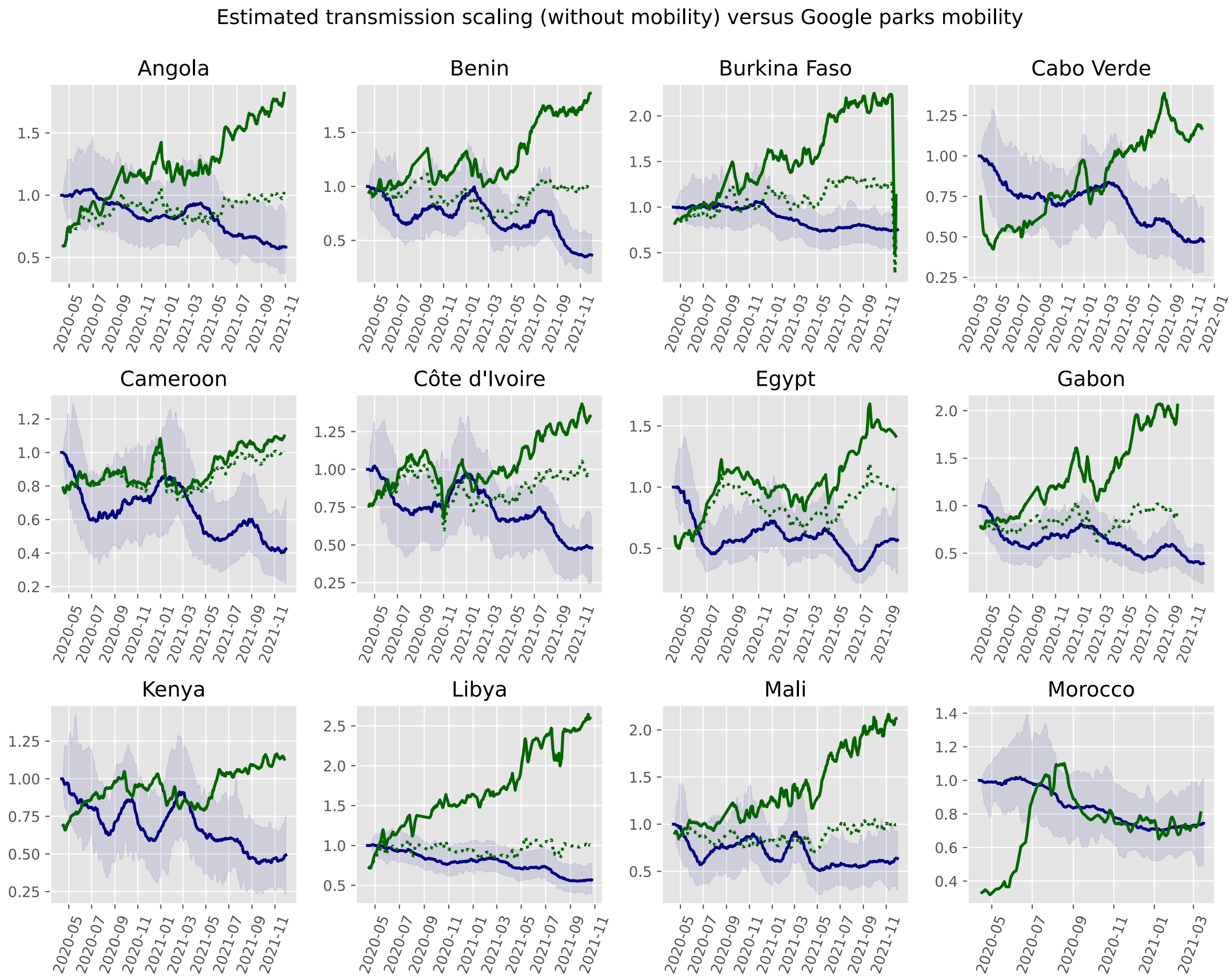

Estimated transmission scaling (without mobility) versus Google parks mobility

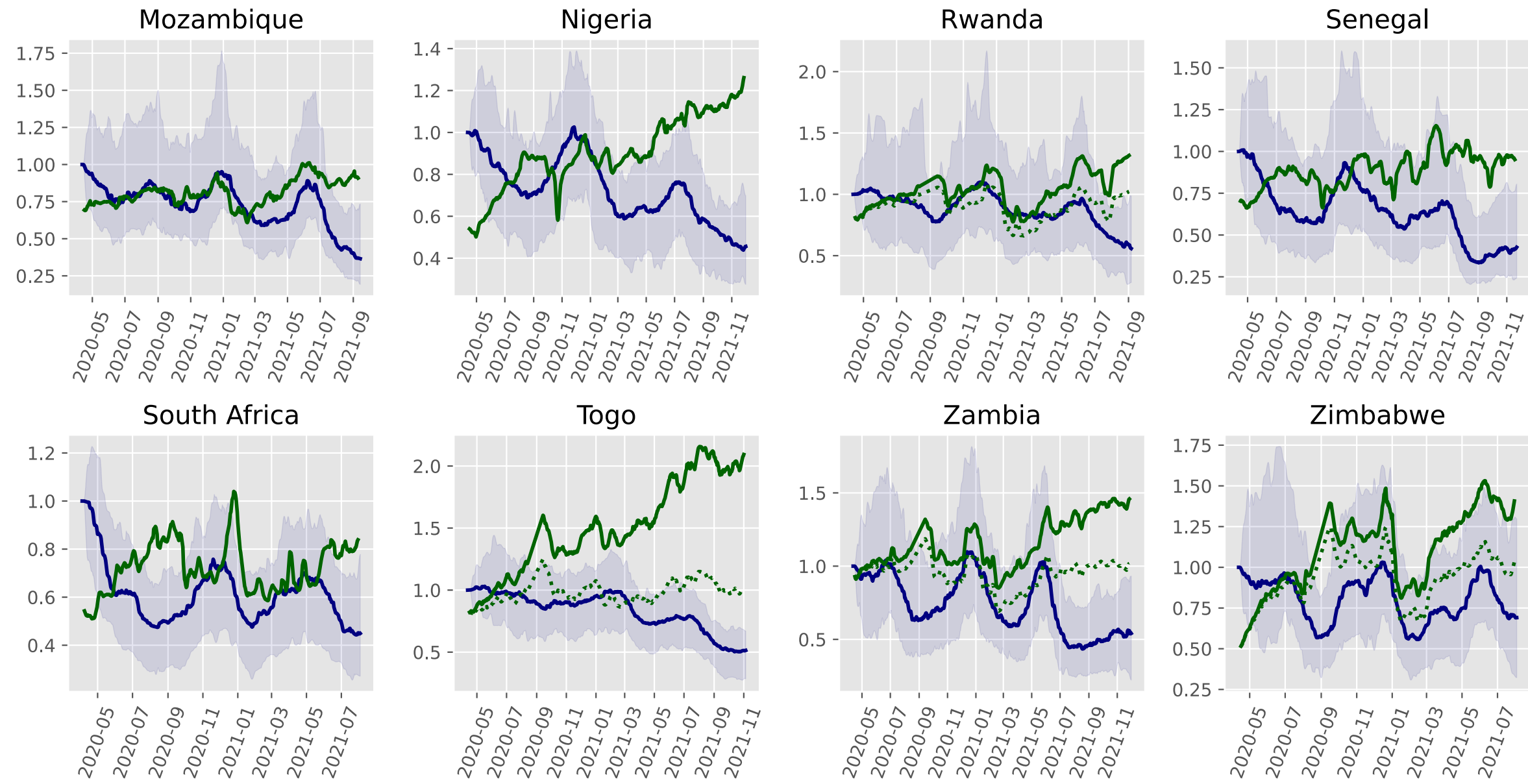

Asia

Estimated transmission scaling (without mobility) versus Google parks mobility

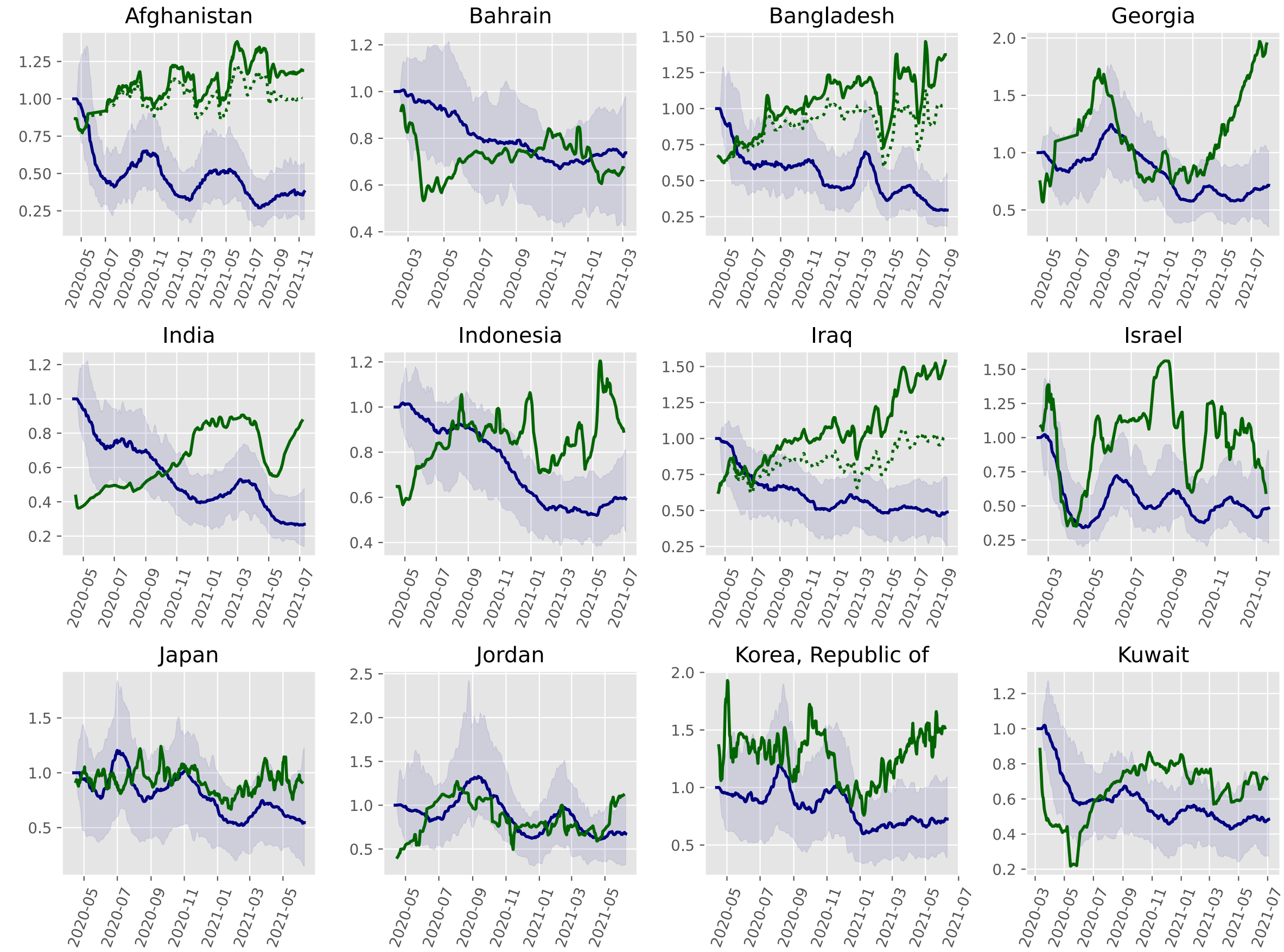

Estimated transmission scaling (without mobility) versus Google parks mobility

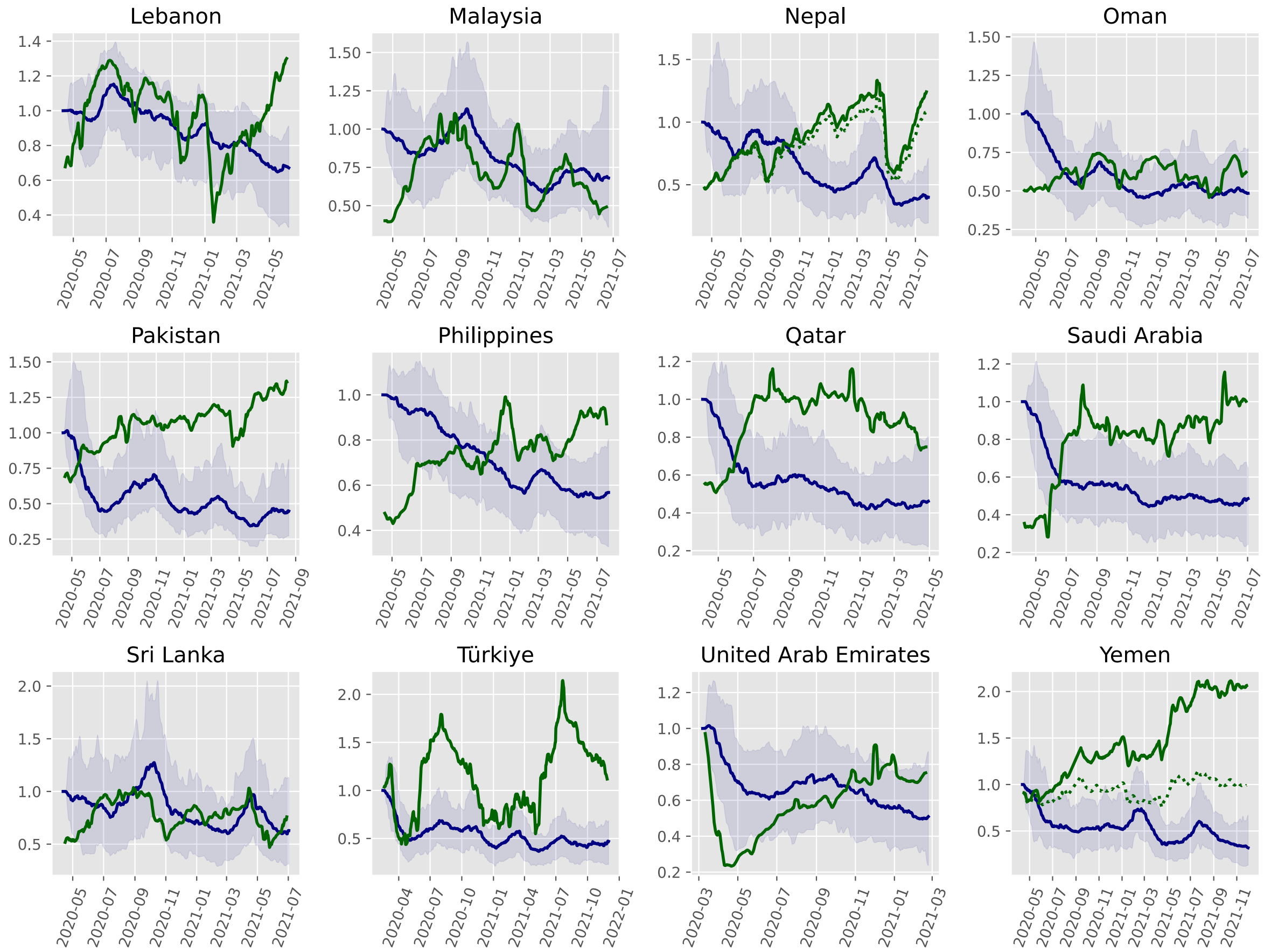

Europe

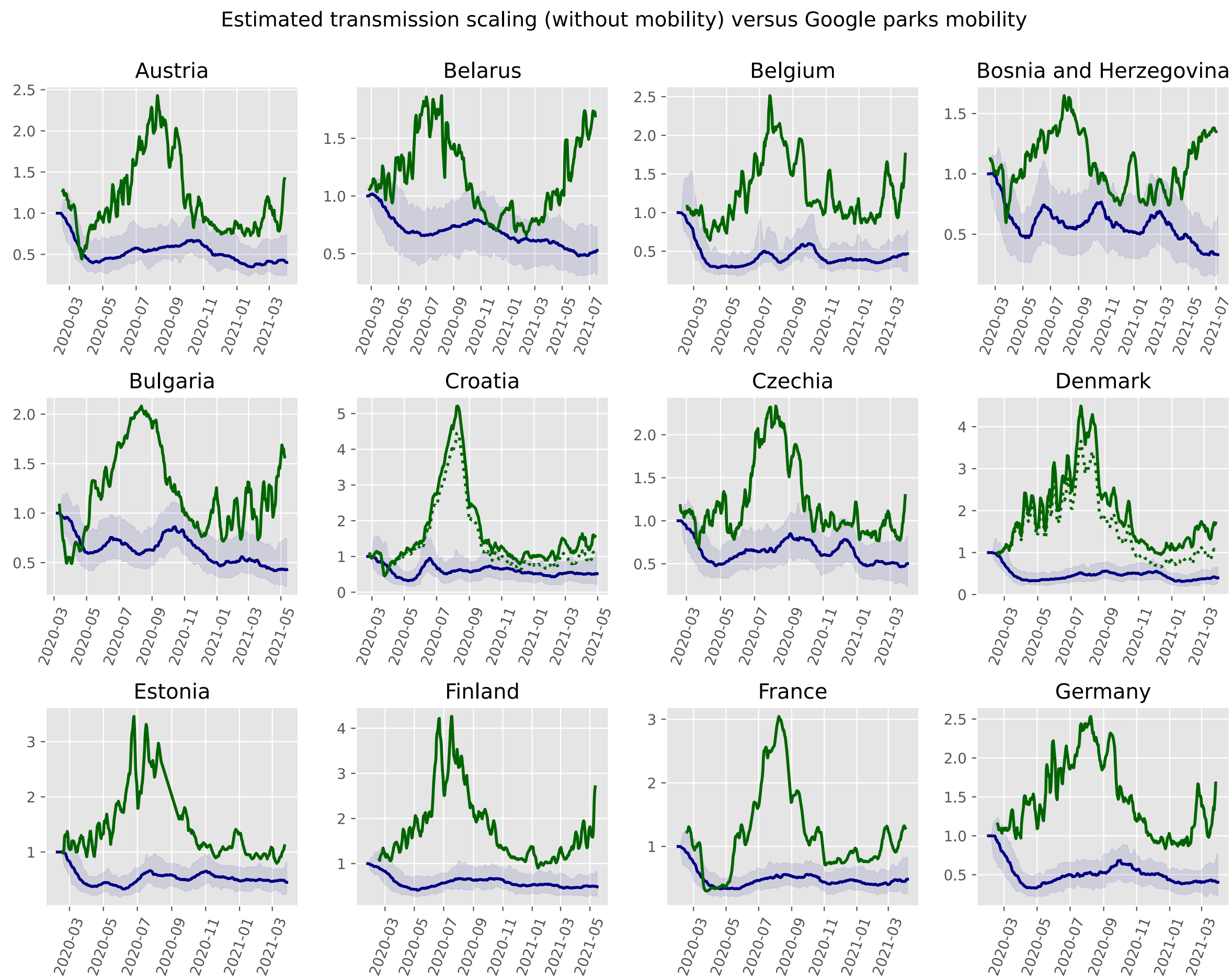

Estimated transmission scaling (without mobility) versus Google parks mobility
Austria
Belarus
Belgium
Bosnia and Herzegovina
Bulgaria
Croatia
Czechia
Denmark
Estonia
Finland
France
Germany

Estimated transmission scaling (without mobility) versus Google parks mobility

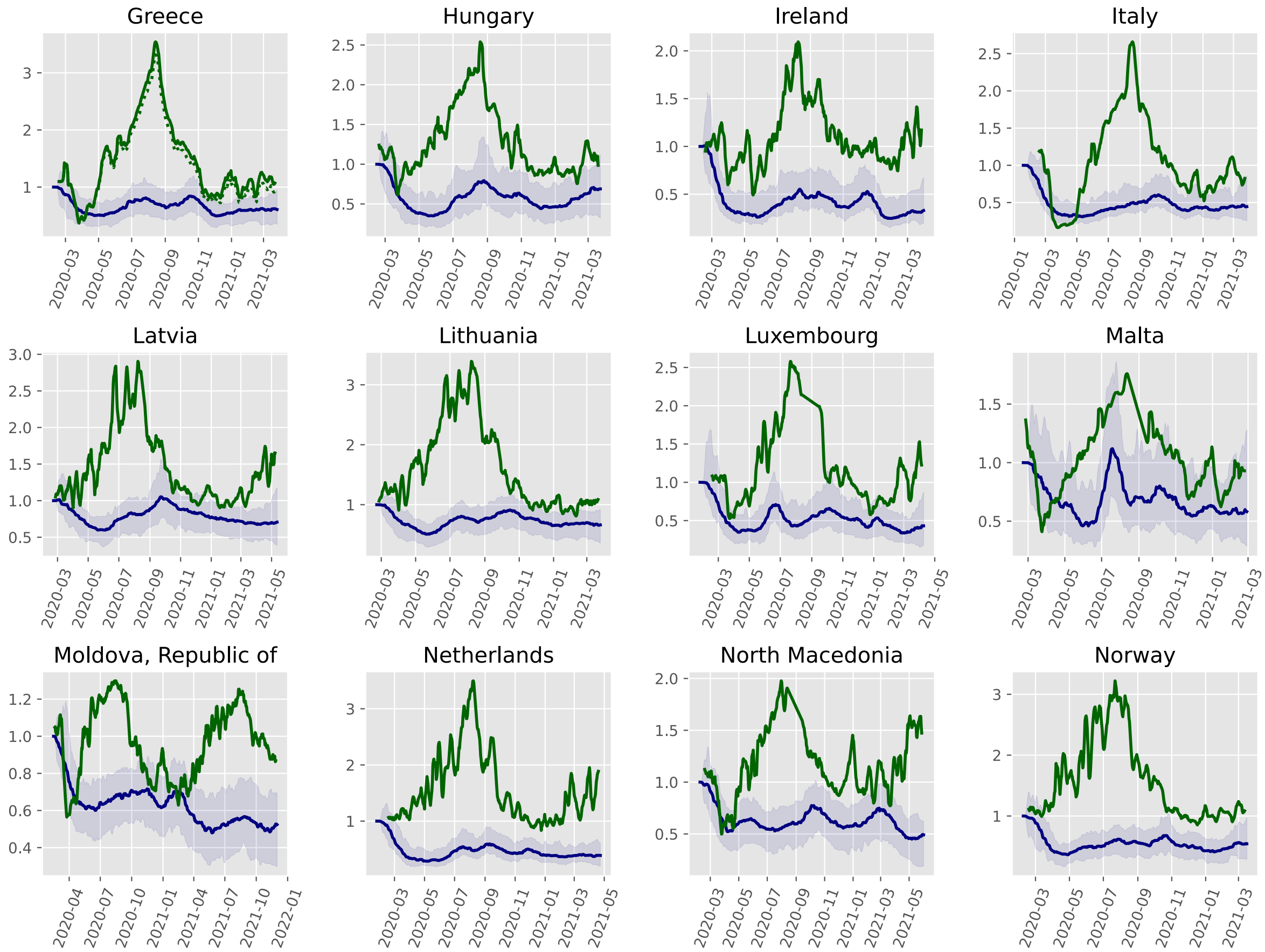

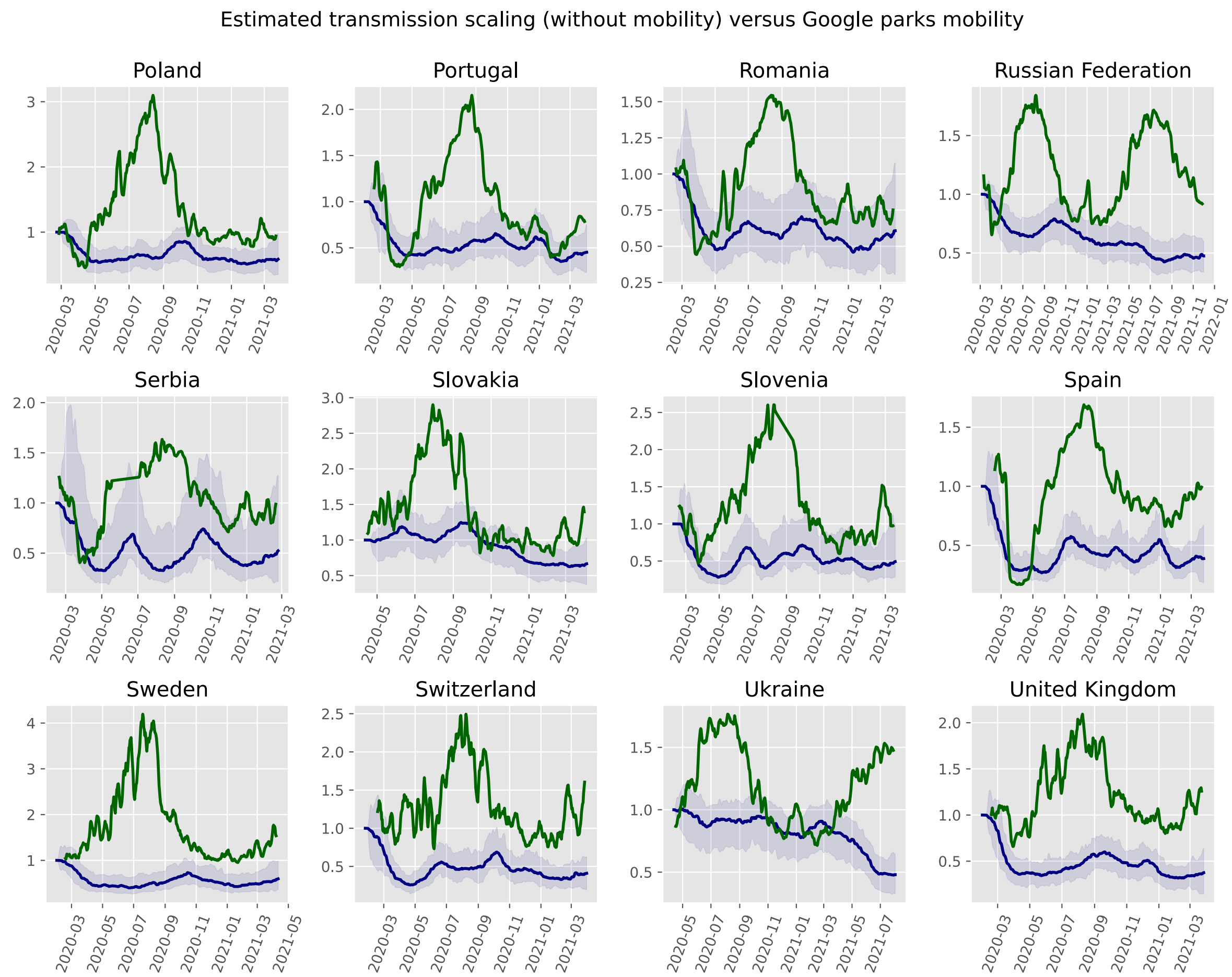

North America

Estimated transmission scaling (without mobility) versus Google parks mobility

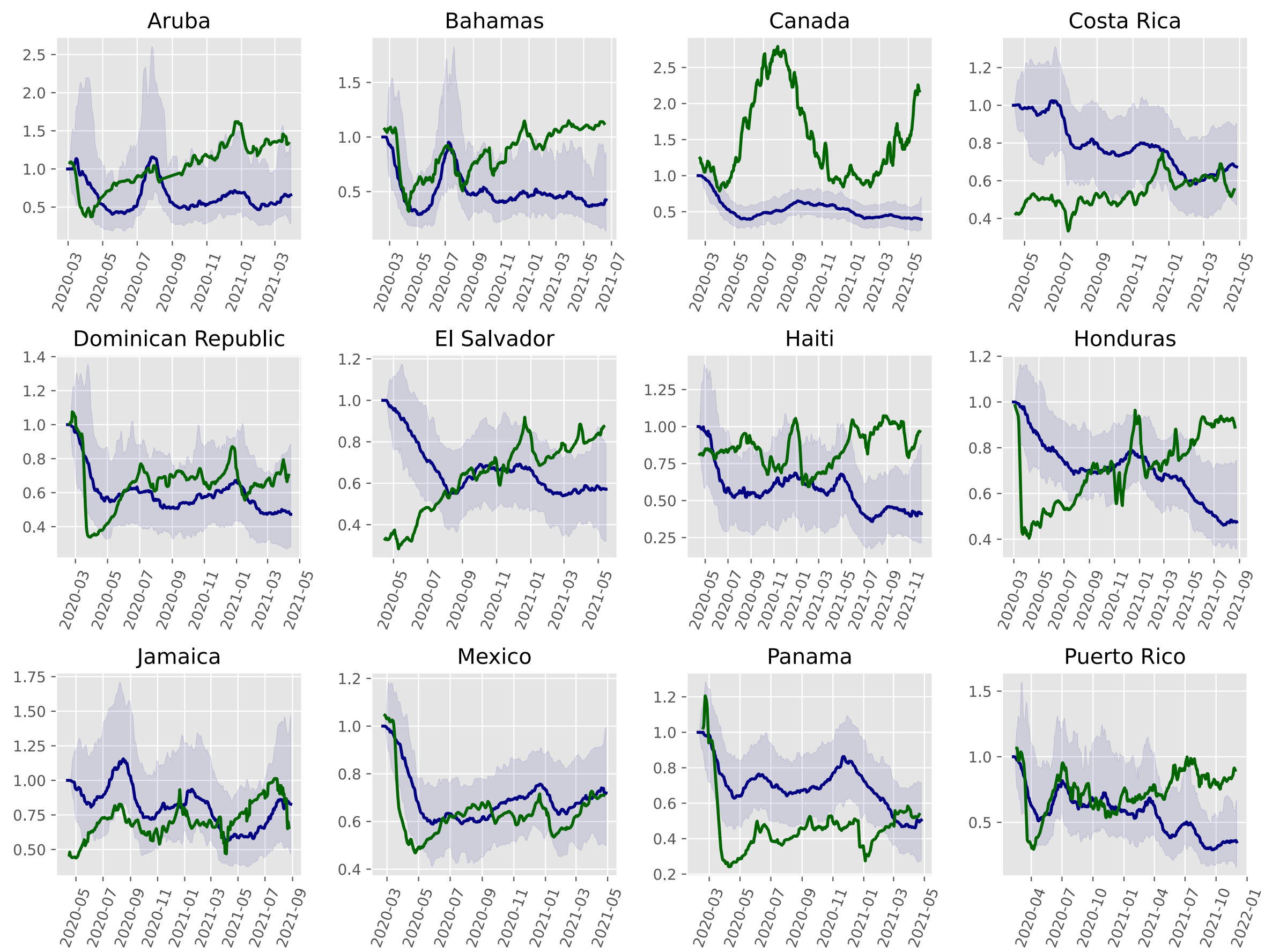

Estimated transmission scaling (without mobility) versus Google parks mobility

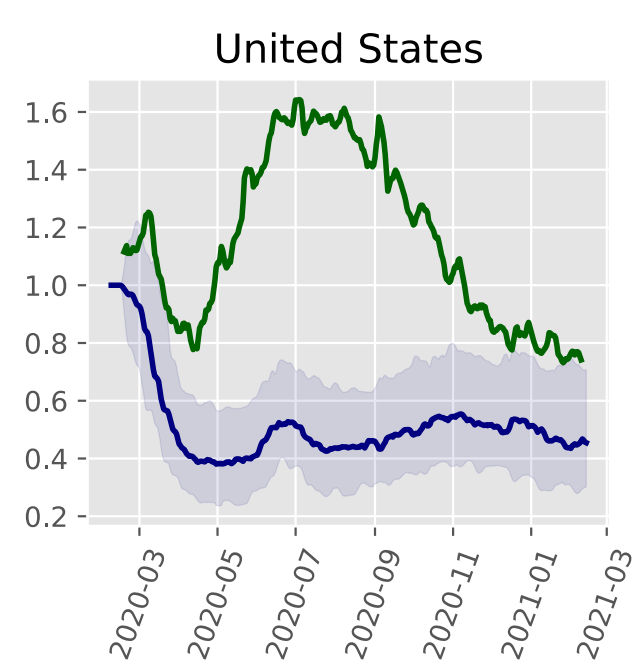

Oceania

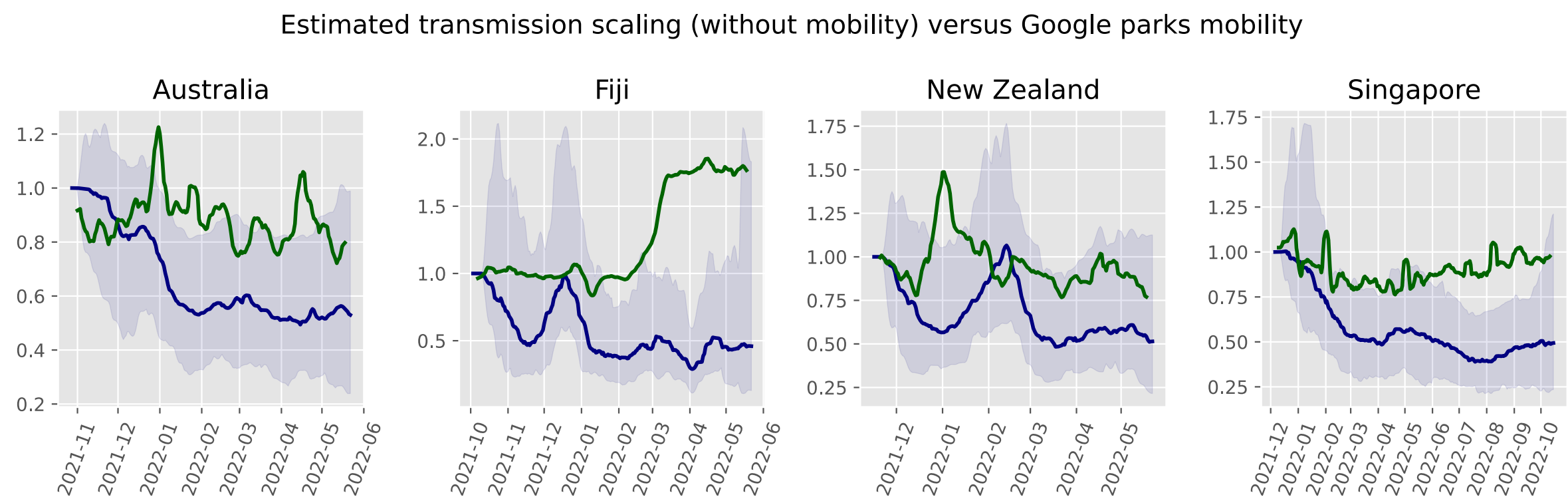

South America

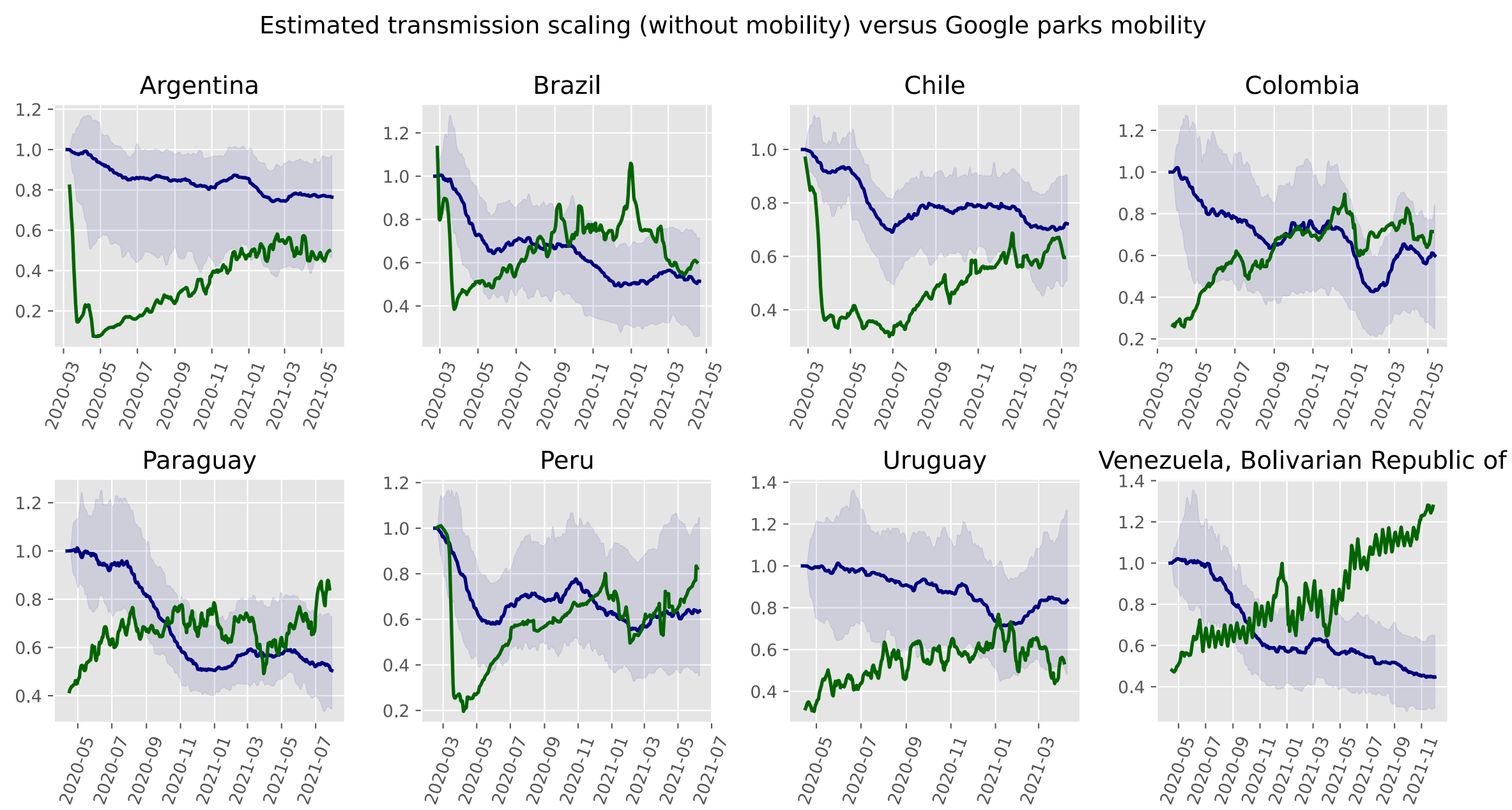

Google transit stations mobility metric comparison

Mobility values presented as Google data divided by 100, plus one.

Africa

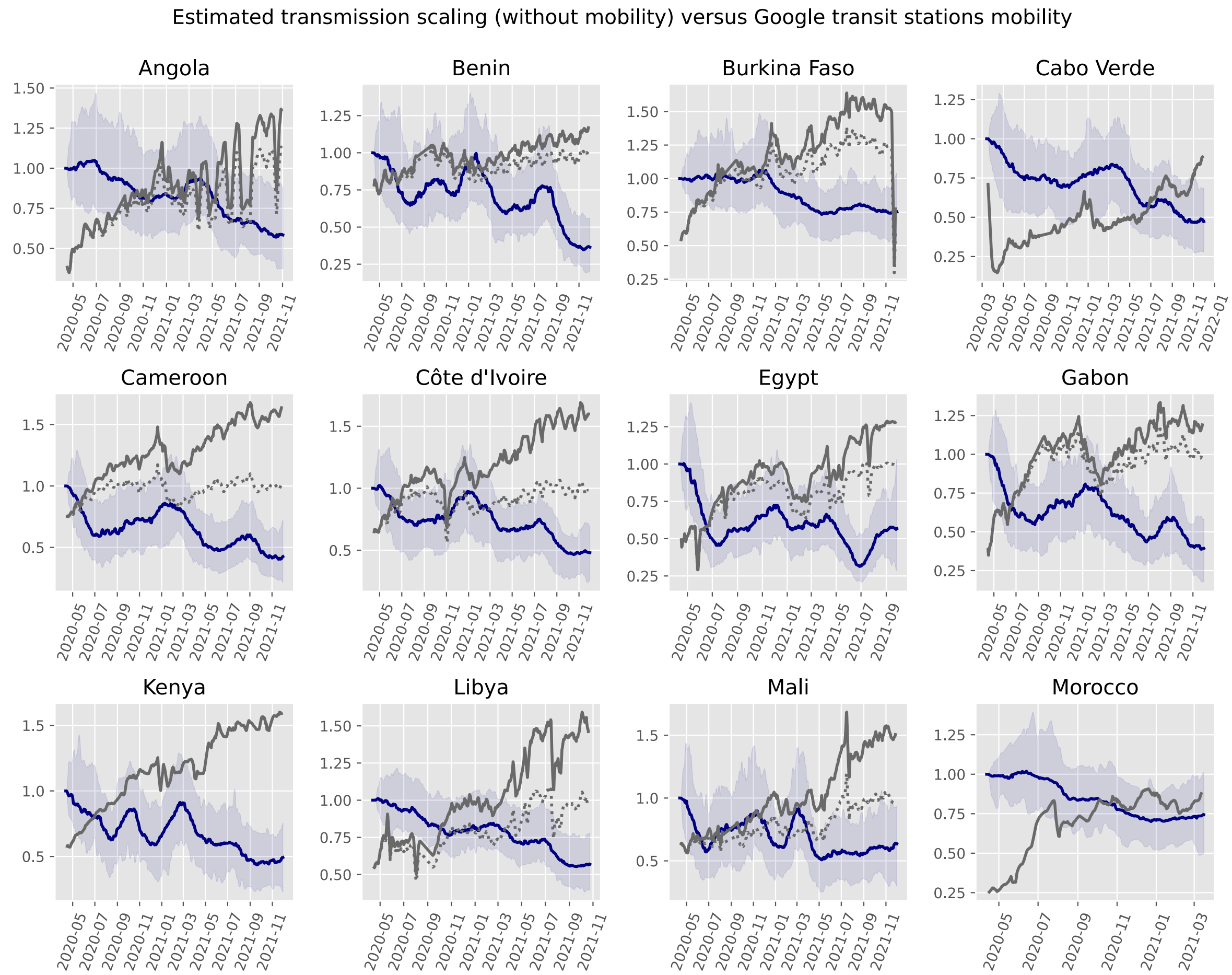

Estimated transmission scaling (without mobility) versus Google transit stations mobility

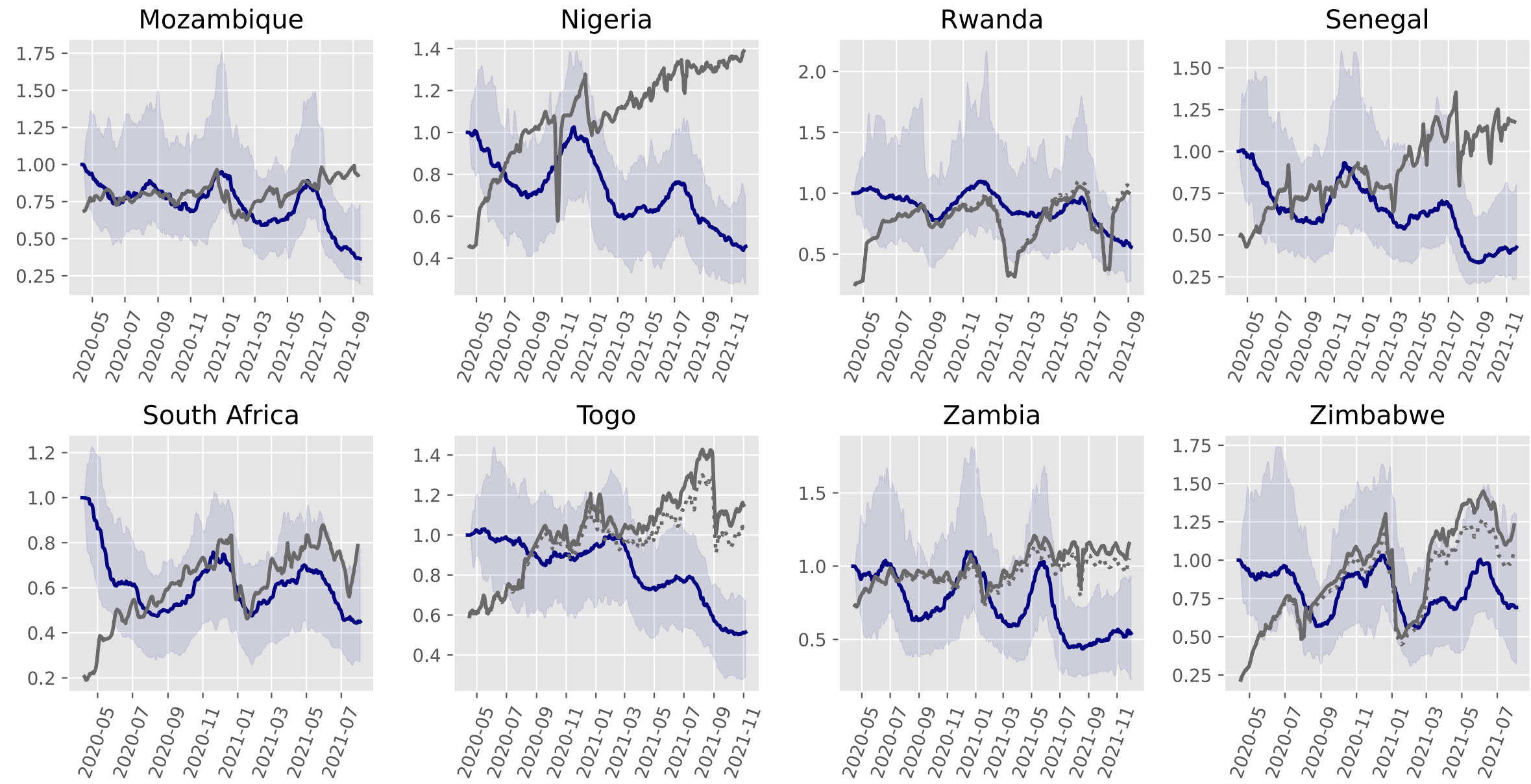

Asia

Estimated transmission scaling (without mobility) versus Google transit stations mobility

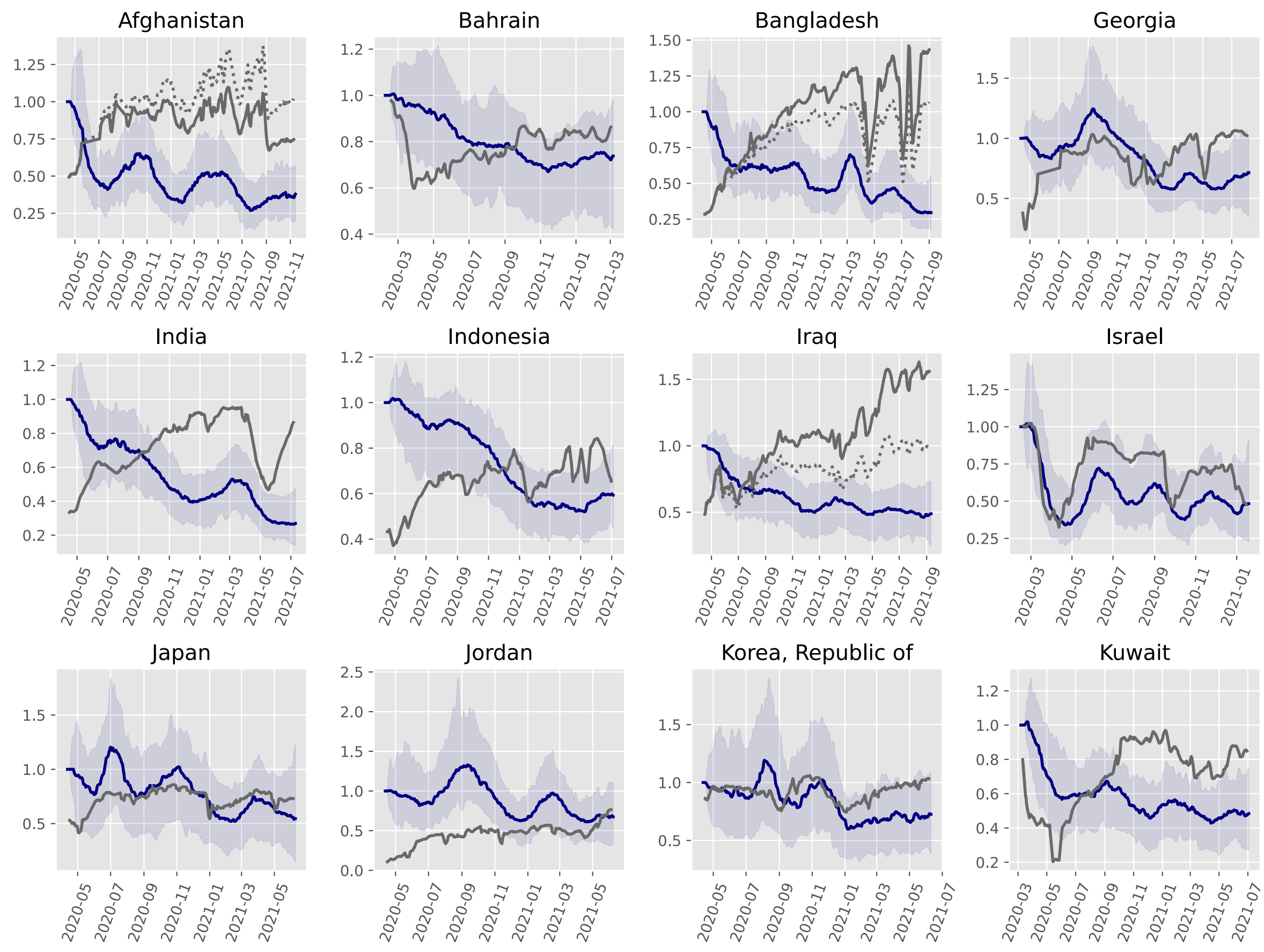

Estimated transmission scaling (without mobility) versus Google transit stations mobility

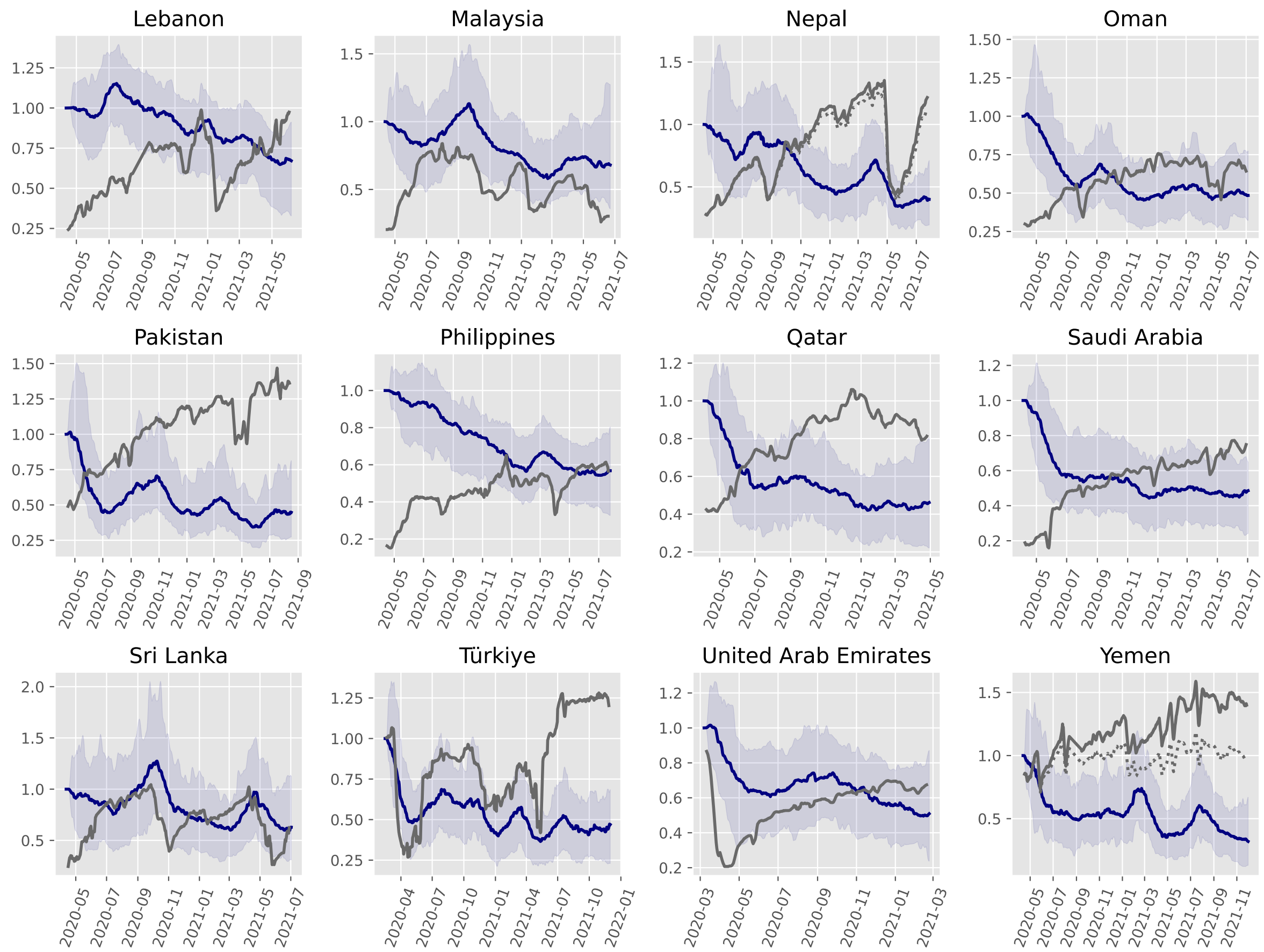

Europe

Estimated transmission scaling (without mobility) versus Google transit stations mobility

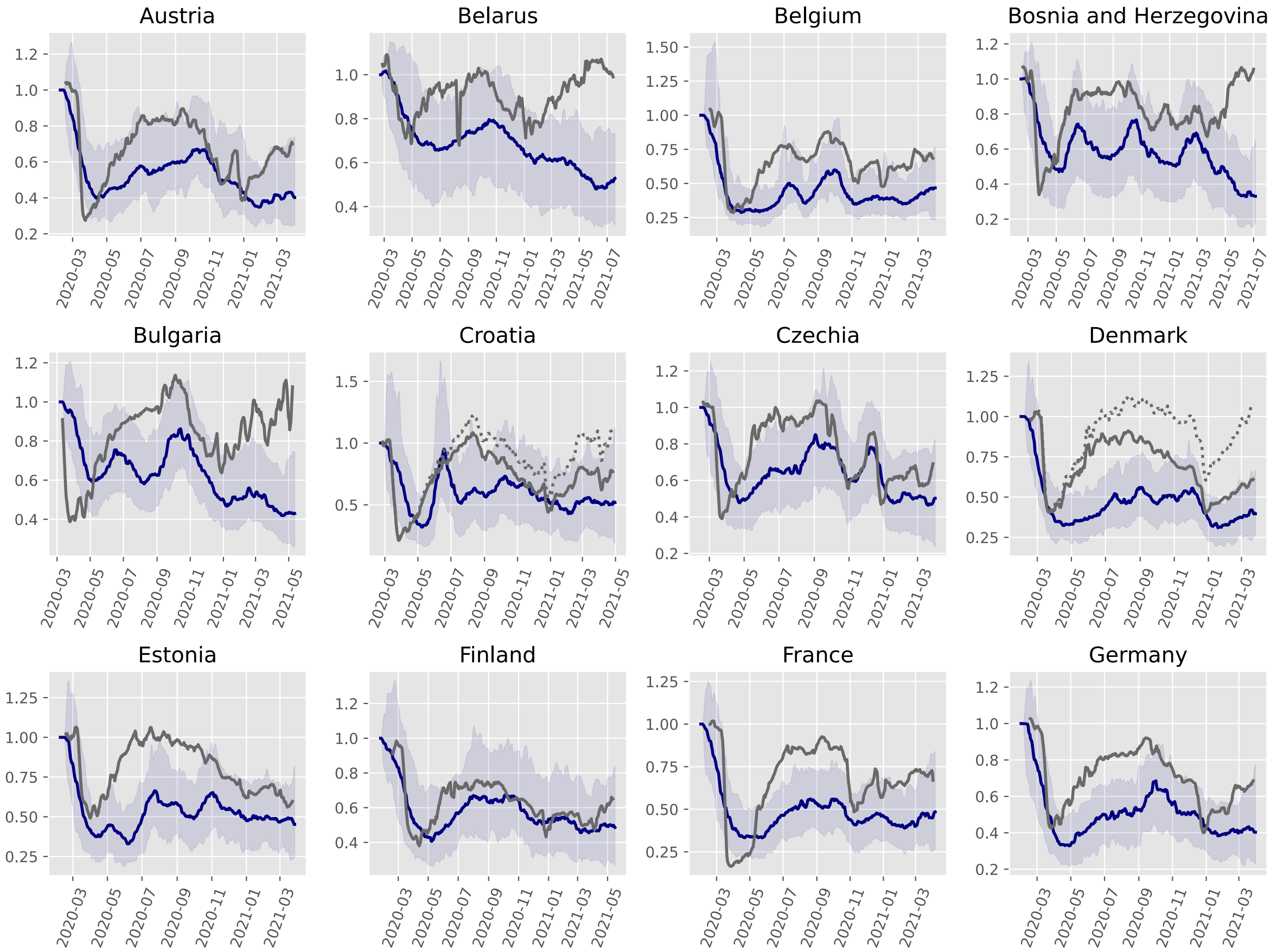

Estimated transmission scaling (without mobility) versus Google transit stations mobility

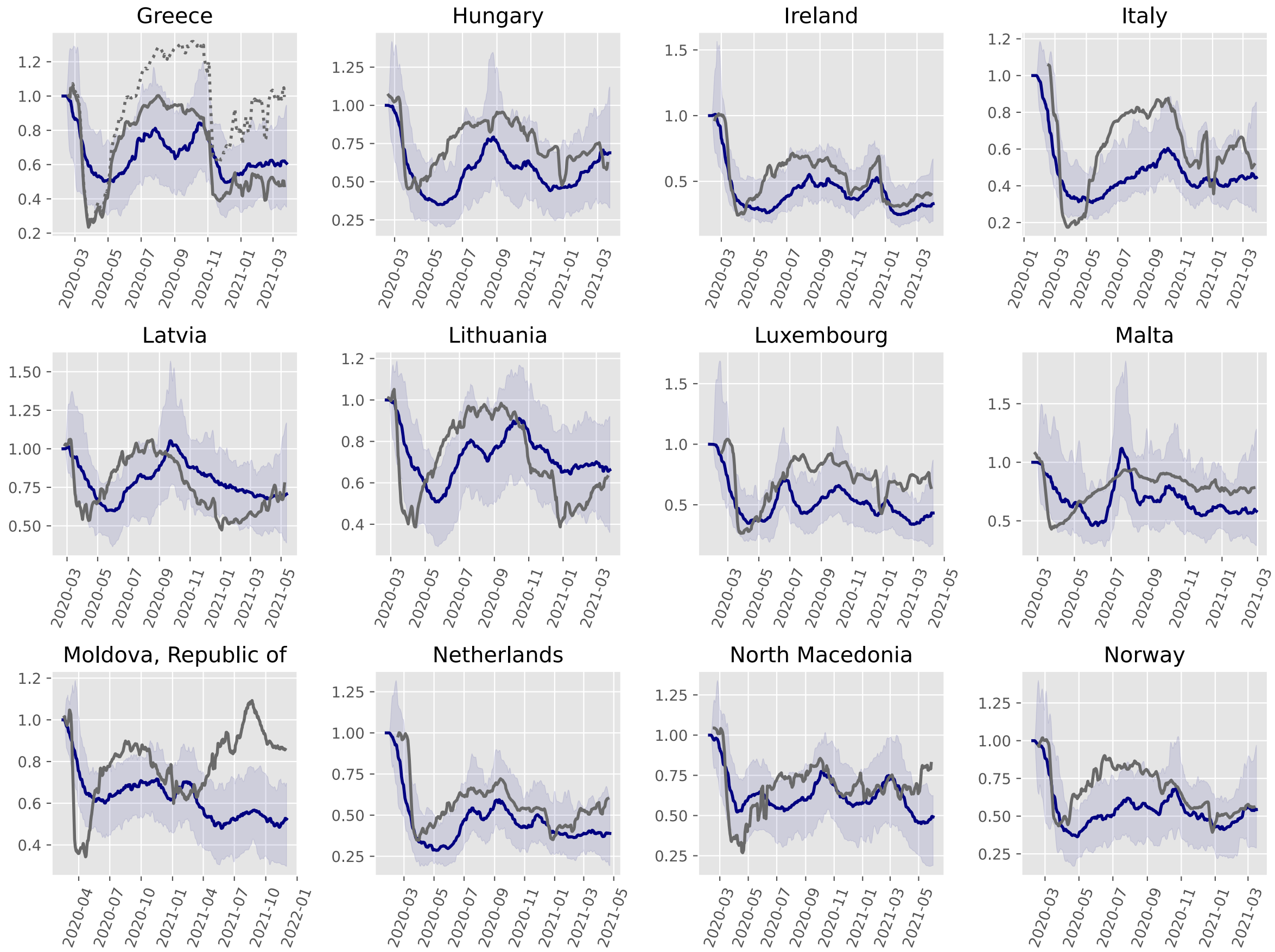

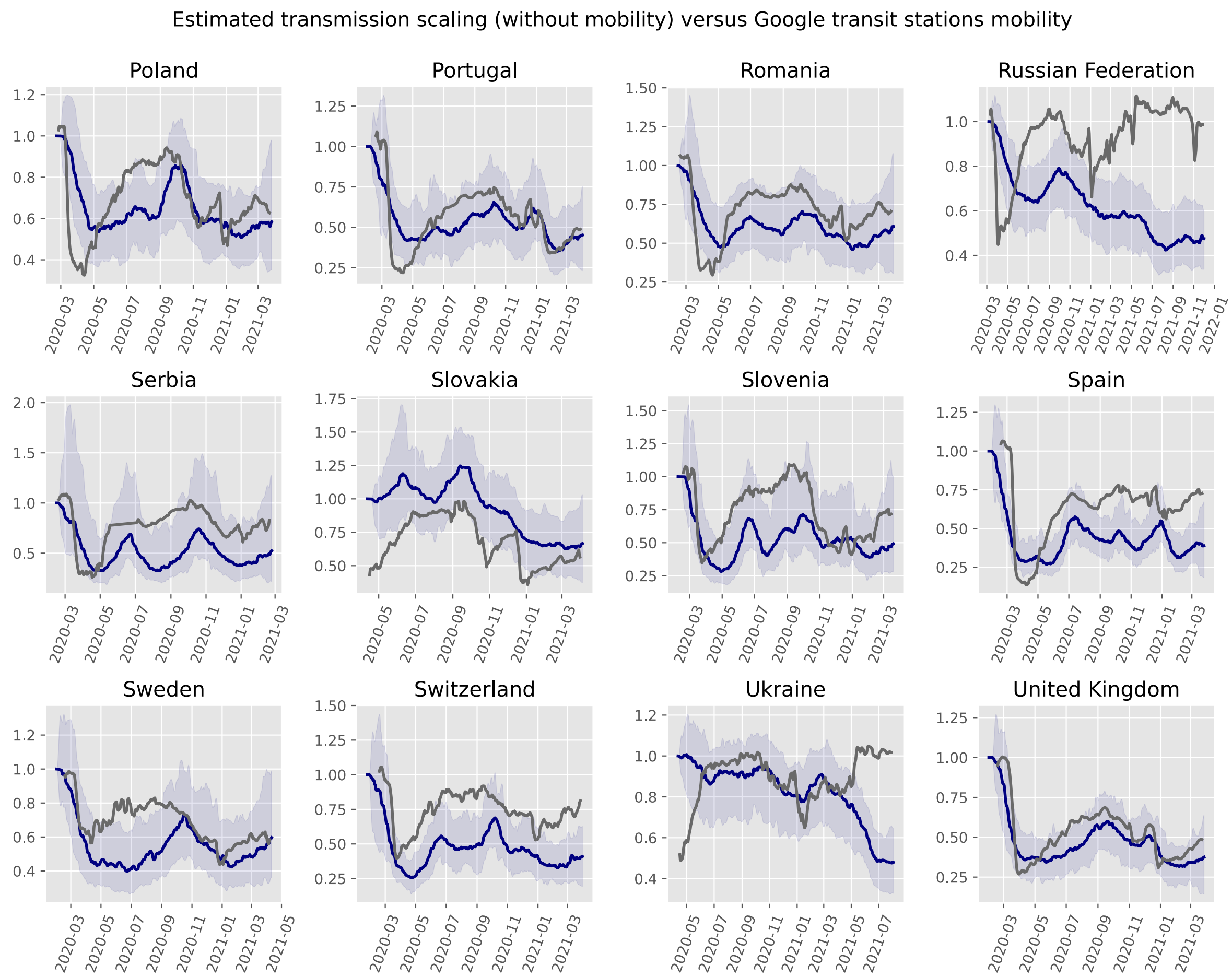

North America

Estimated transmission scaling (without mobility) versus Google transit stations mobility

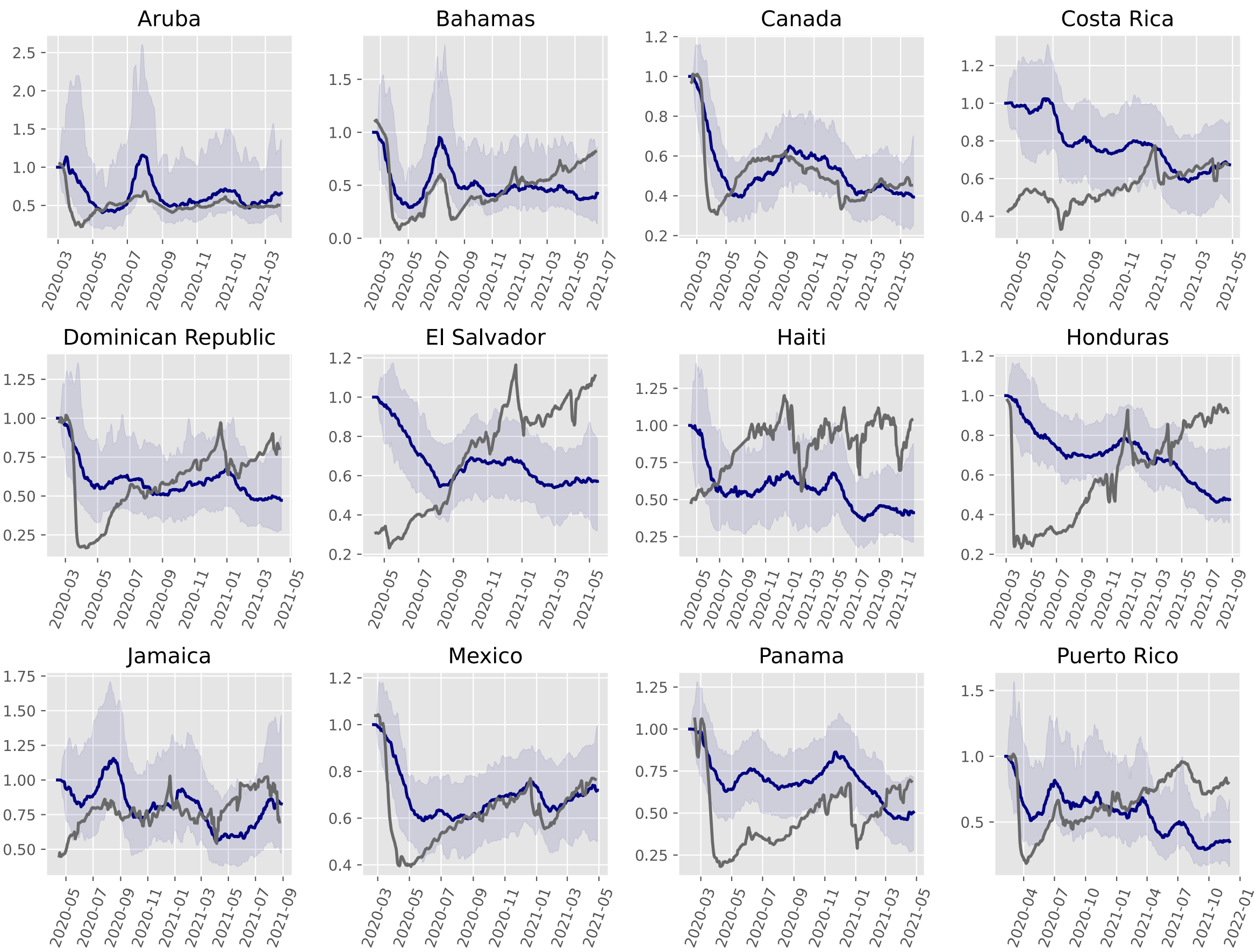

Estimated transmission scaling (without mobility) versus Google transit stations mobility

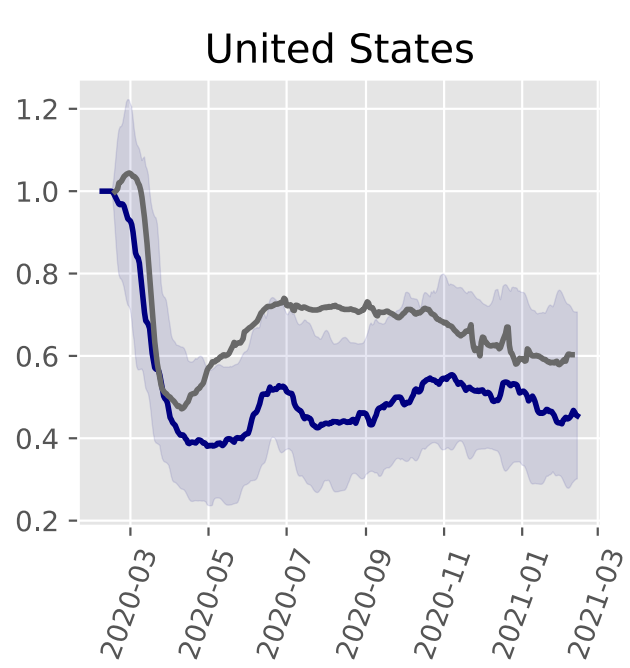

Oceania

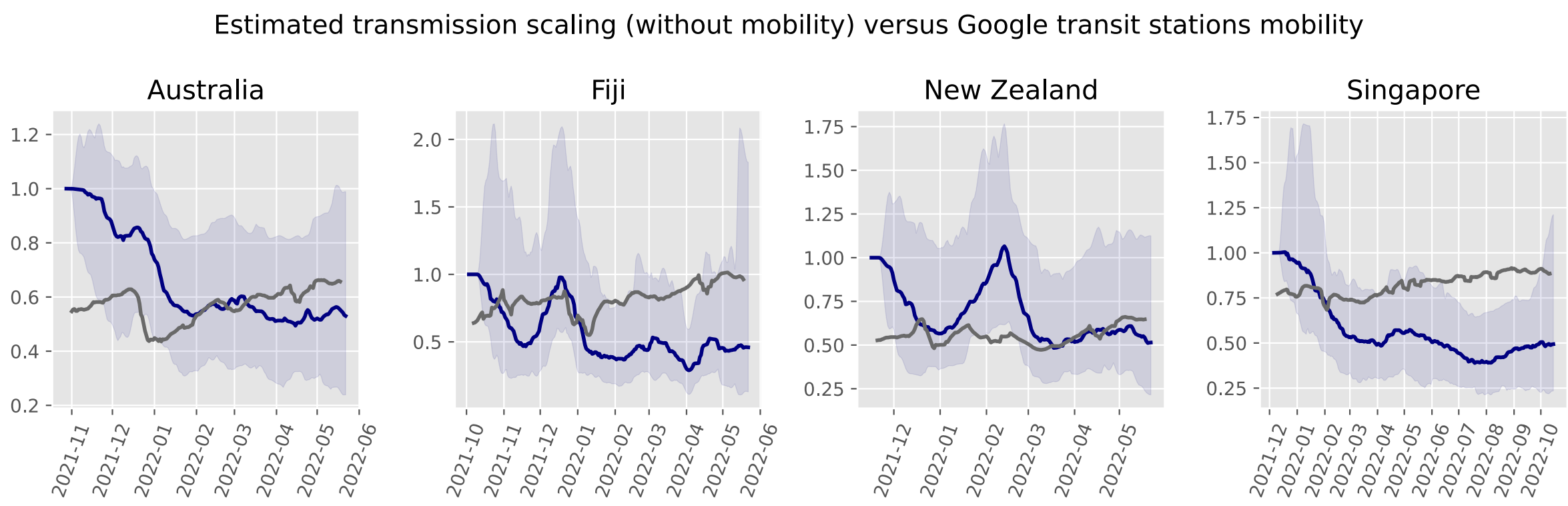

South America

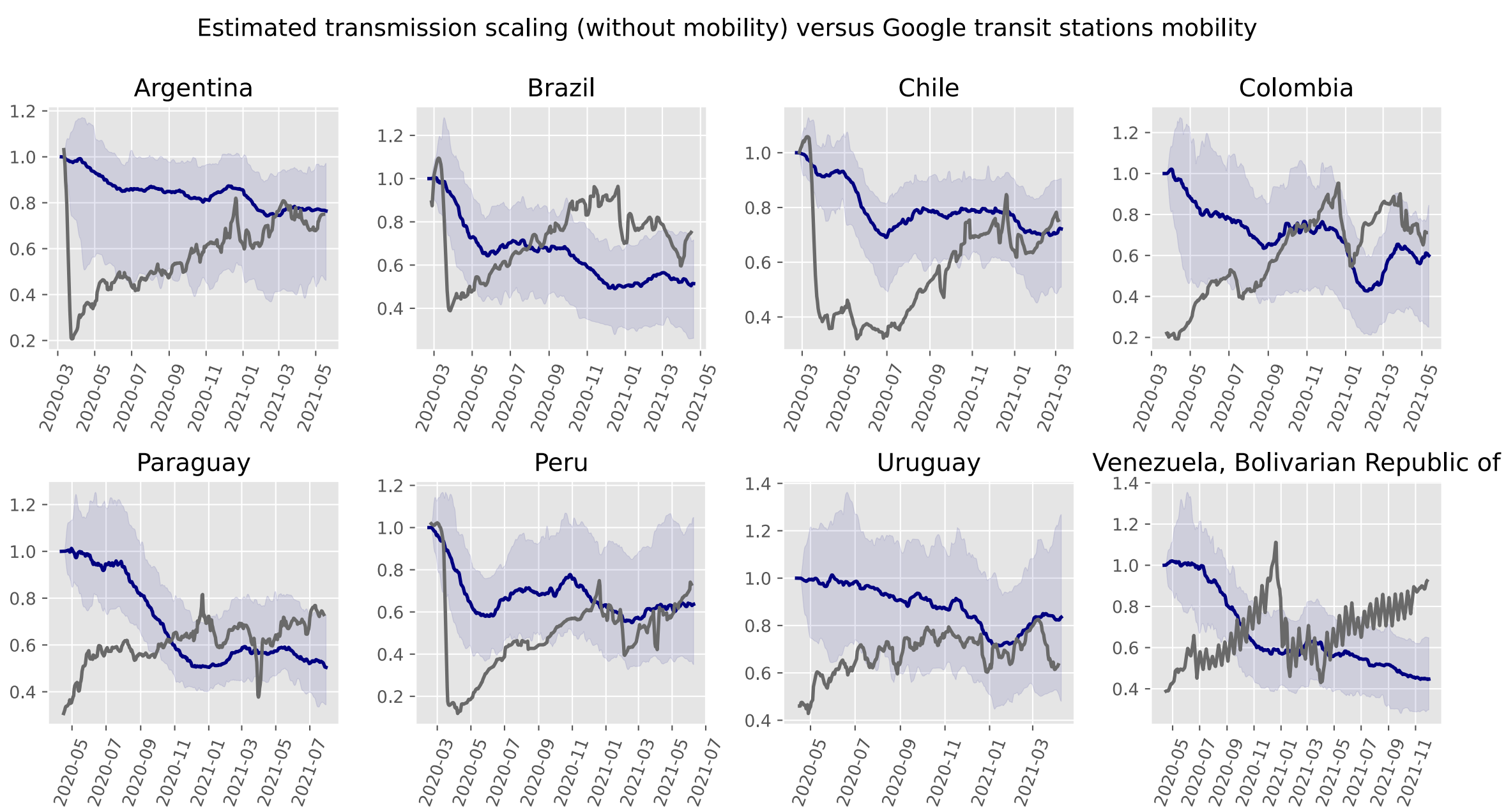

Google workplaces mobility metric comparison

Mobility values presented as Google data divided by 100, plus one.

Africa

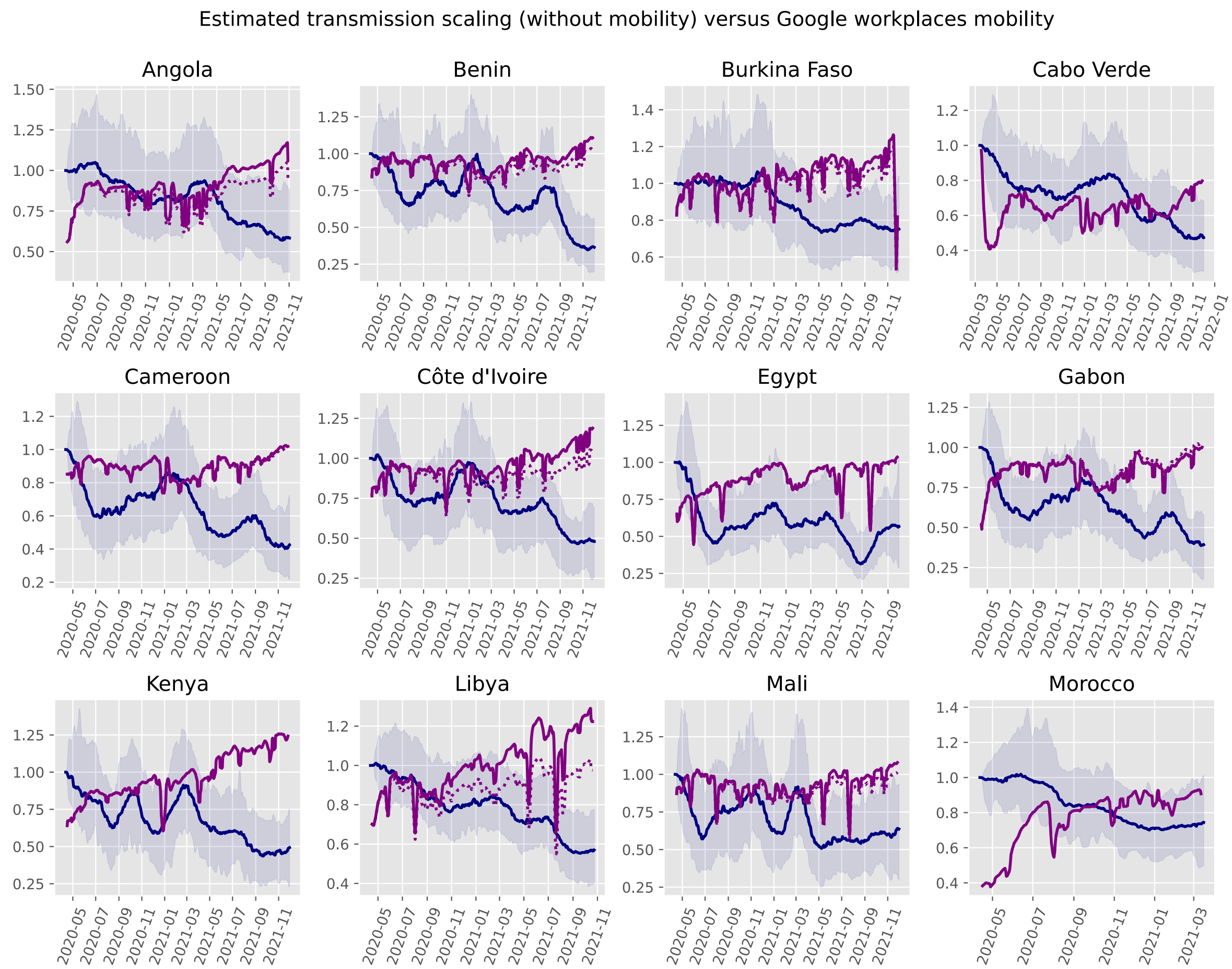

Estimated transmission scaling (without mobility) versus Google workplaces mobility

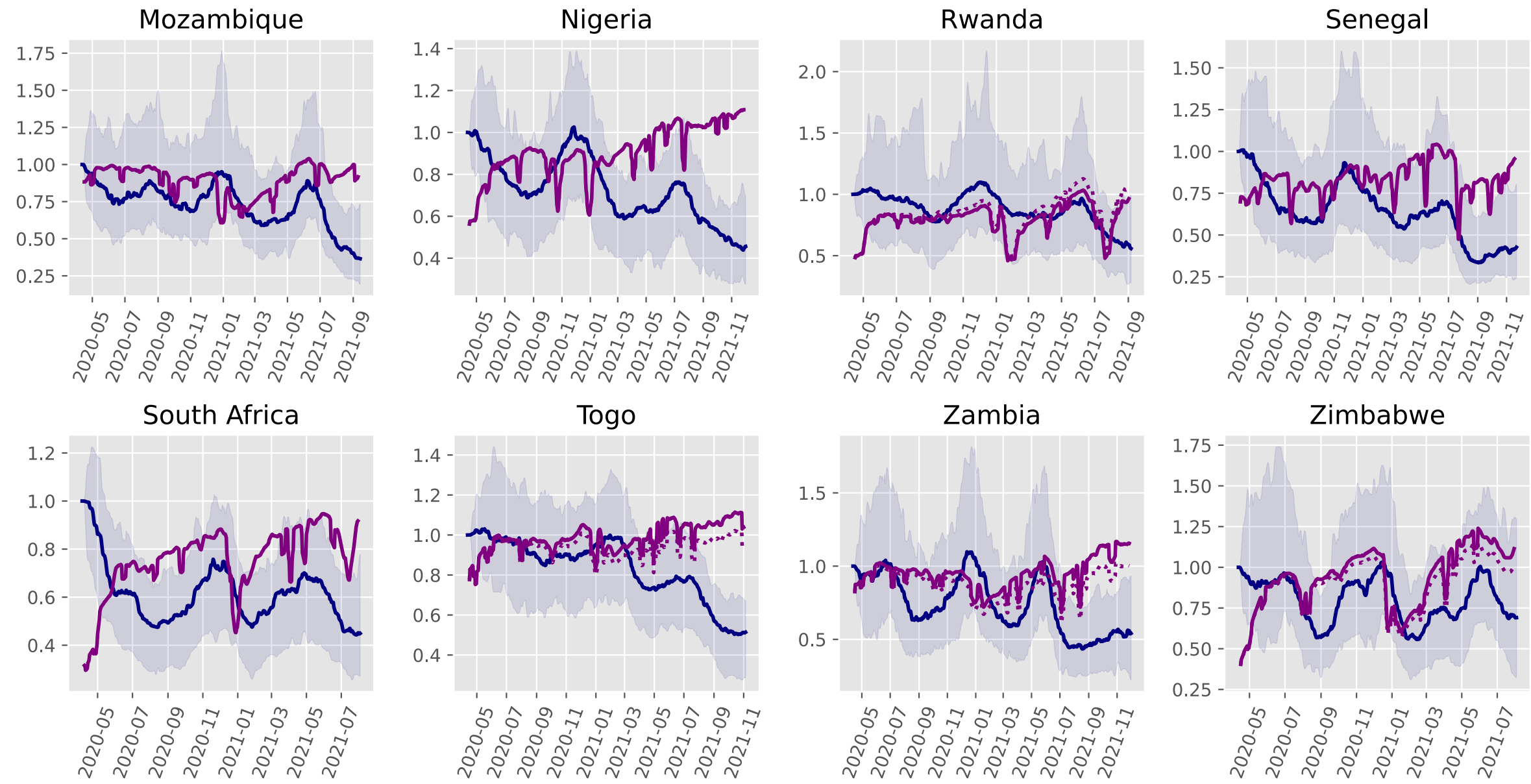

Asia

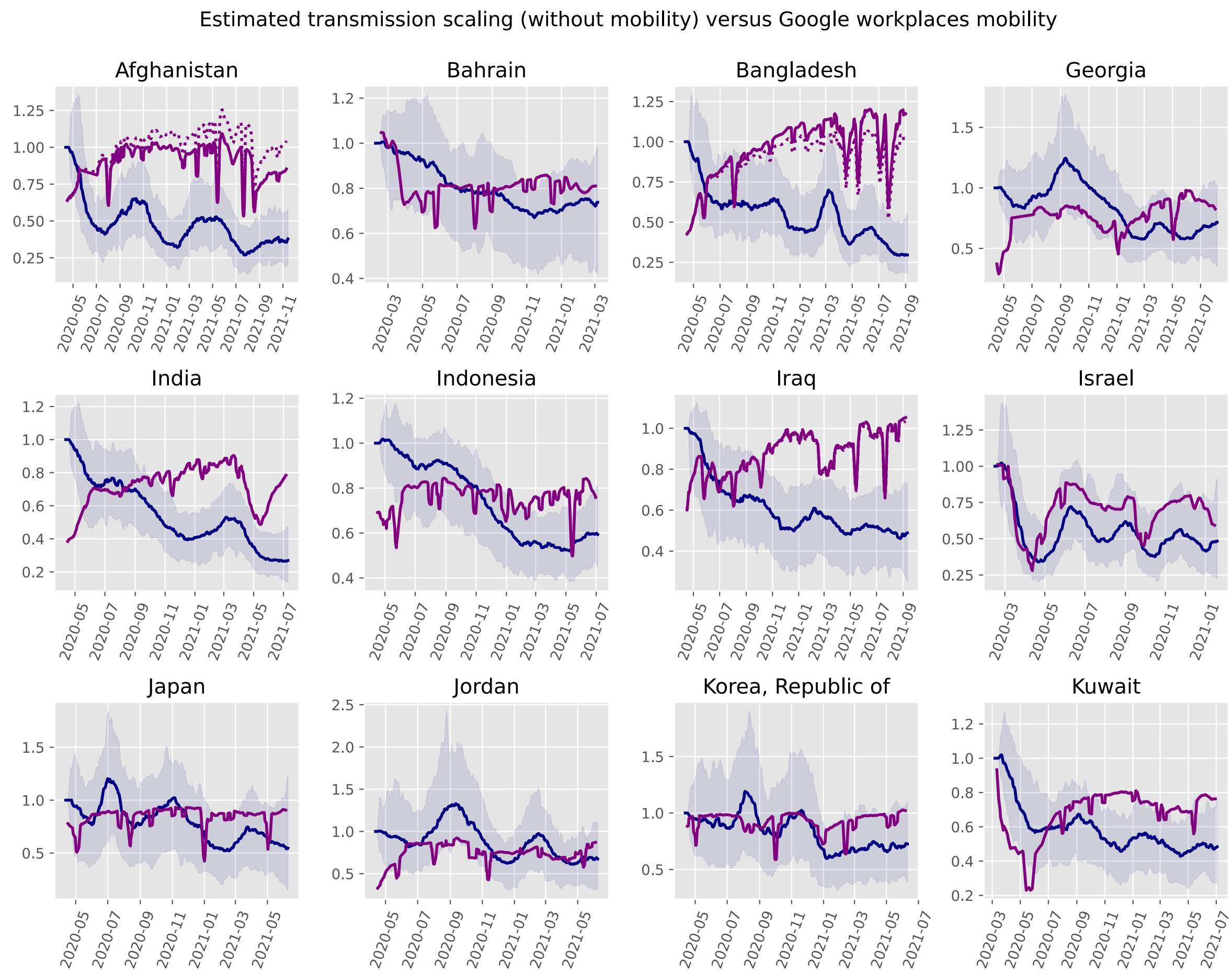
Estimated transmission scaling (without mobility) versus Google workplaces mobility
Afghanistan
Bahrain
Bangladesh
Georgia
India
Indonesia
Iraq
Israel
Japan
Jordan
Korea, Republic of
Kuwait

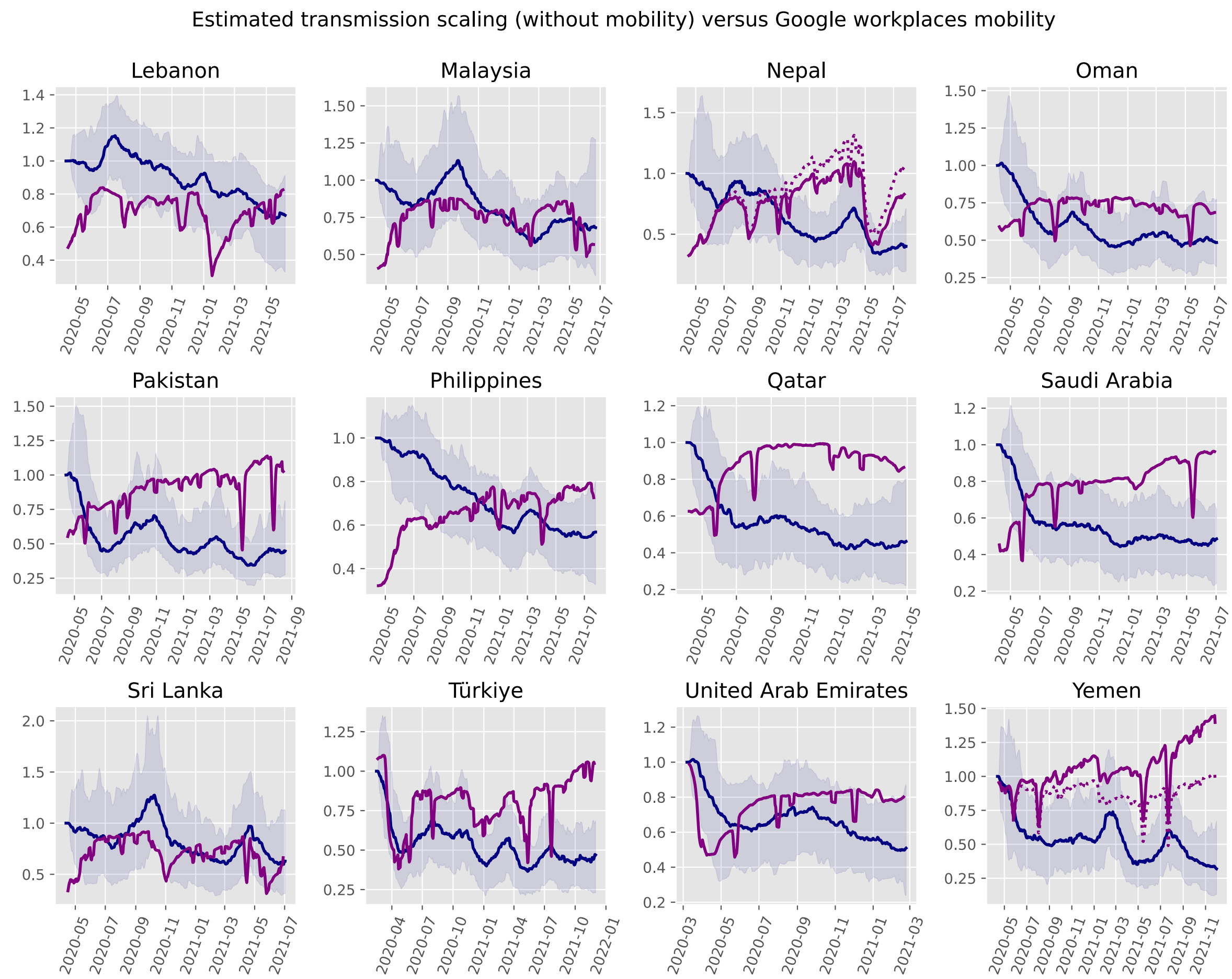
Estimated transmission scaling (without mobility) versus Google workplaces mobility
Lebanon
Malaysia
Nepal
Oman
Pakistan
Philippines
Qatar
Saudi Arabia
Sri Lanka
Türkiye
United Arab Emirates
Yemen

Europe

Estimated transmission scaling (without mobility) versus Google workplaces mobility

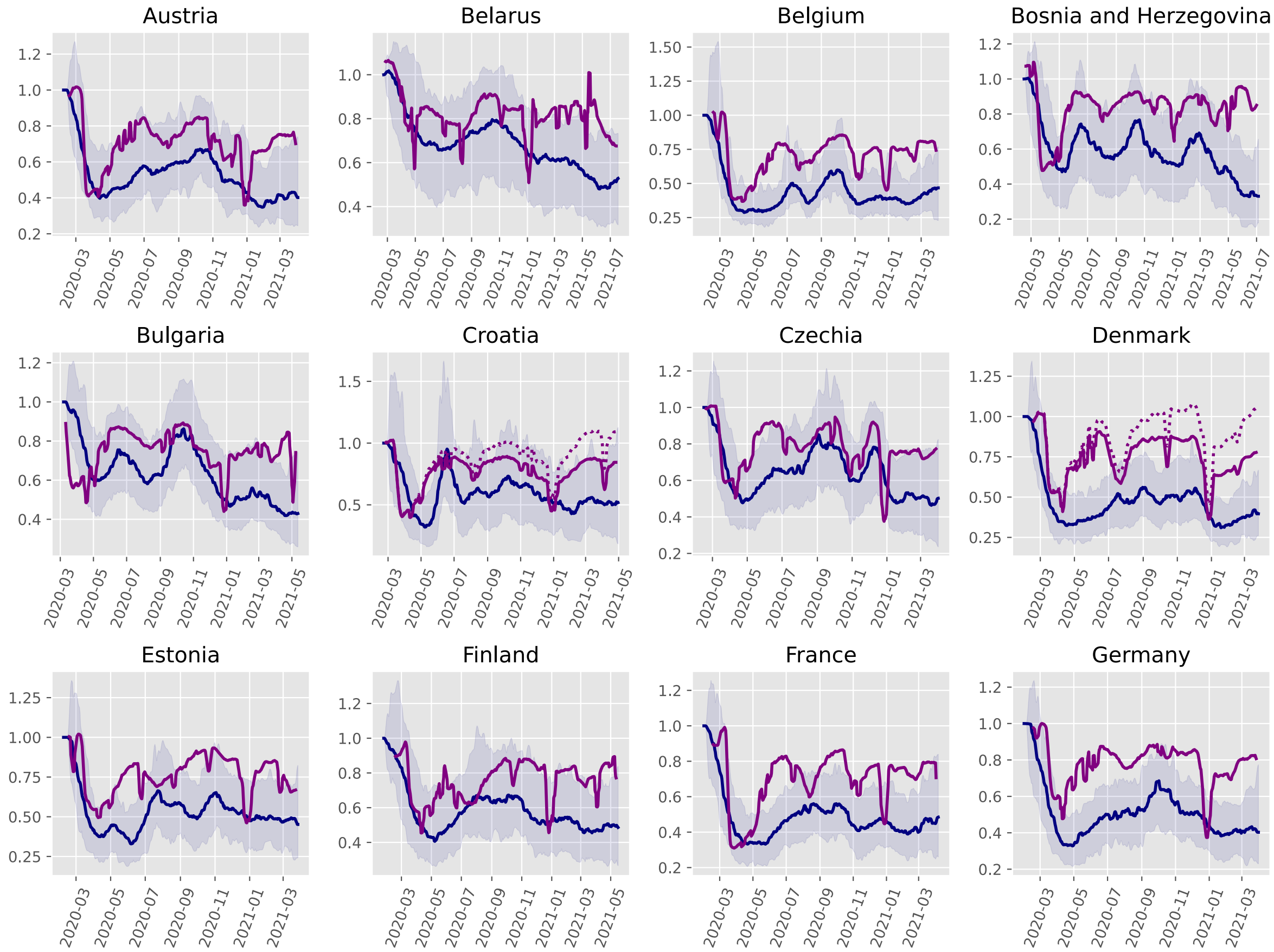

Estimated transmission scaling (without mobility) versus Google workplaces mobility

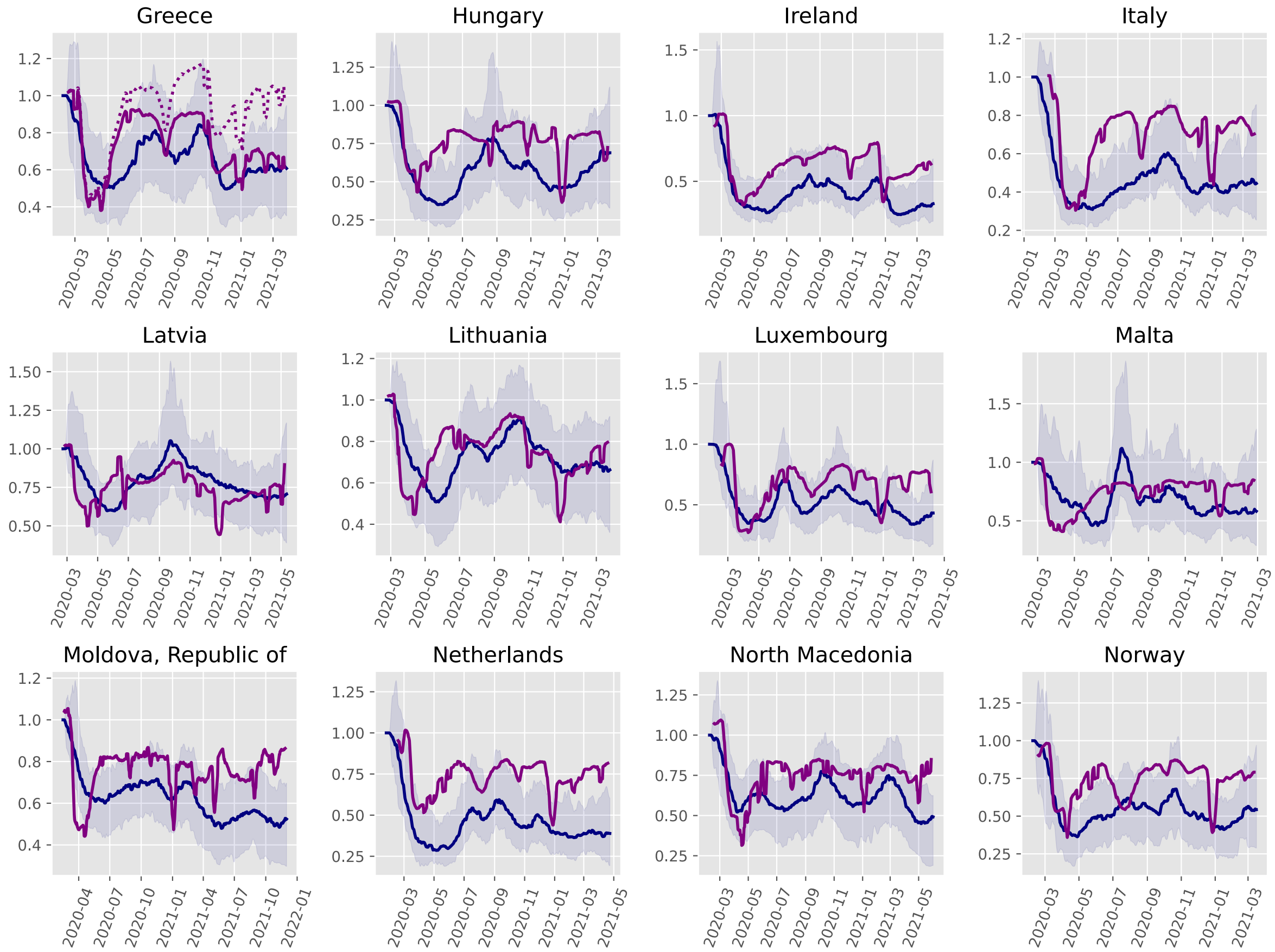

Estimated transmission scaling (without mobility) versus Google workplaces mobility

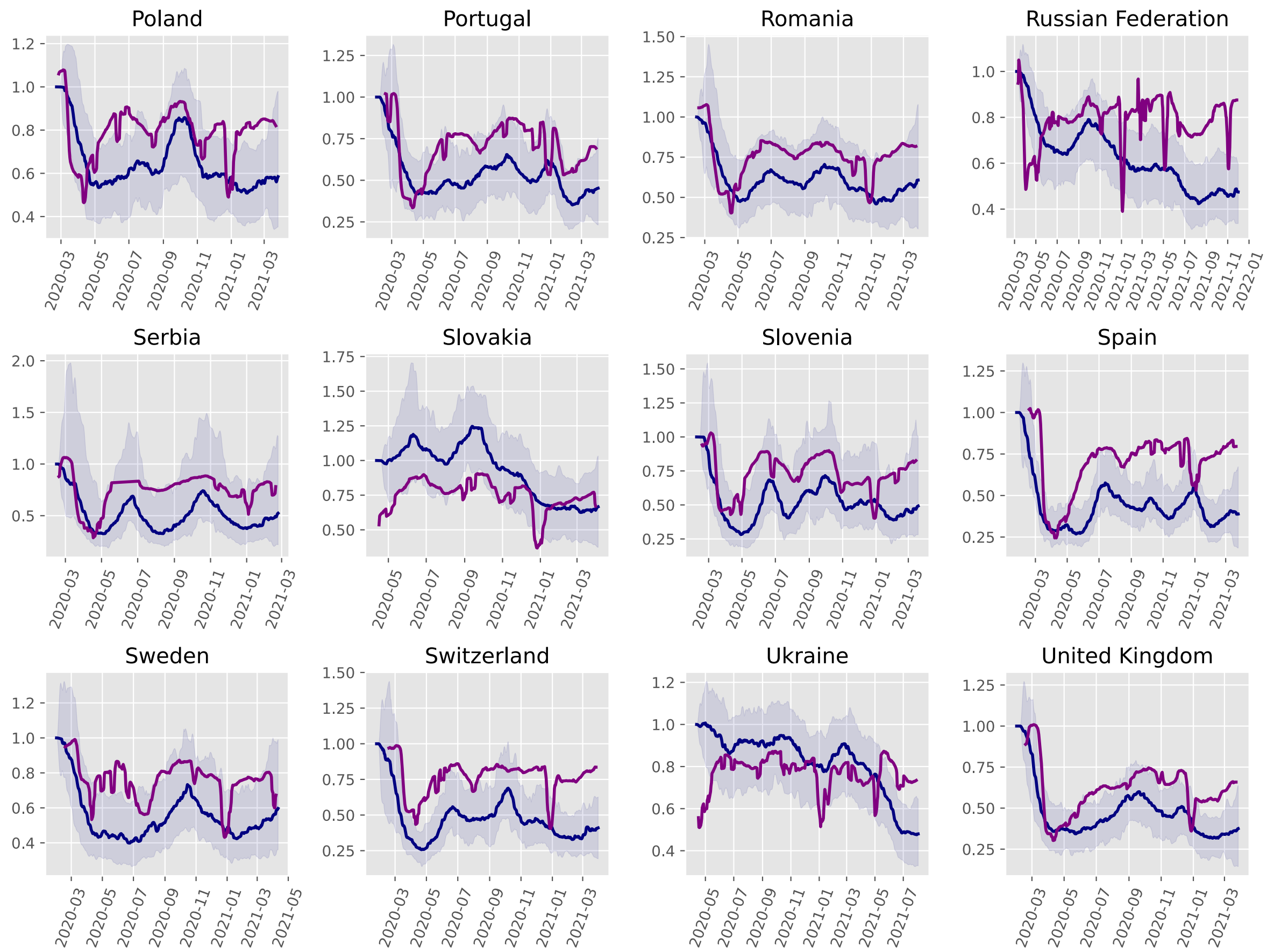

North America

Estimated transmission scaling (without mobility) versus Google workplaces mobility

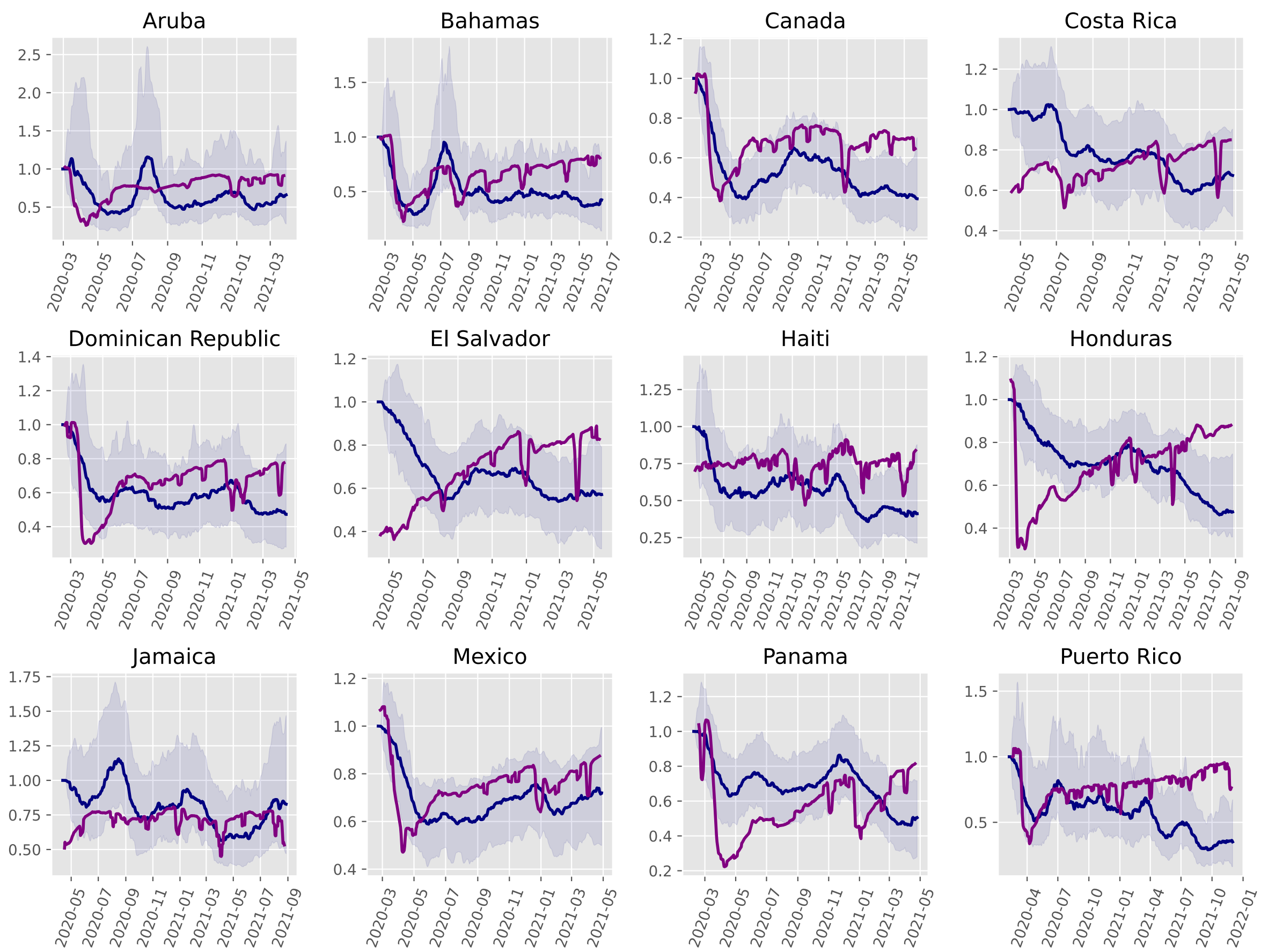

Estimated transmission scaling (without mobility) versus Google workplaces mobility

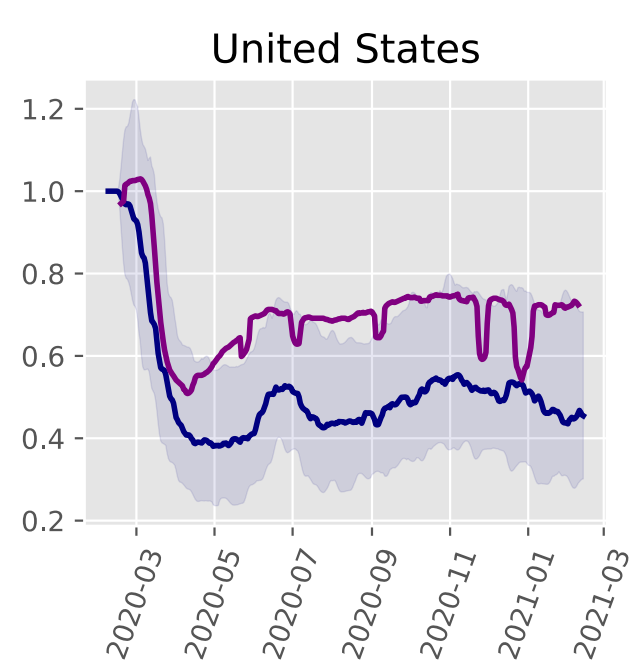

Oceania

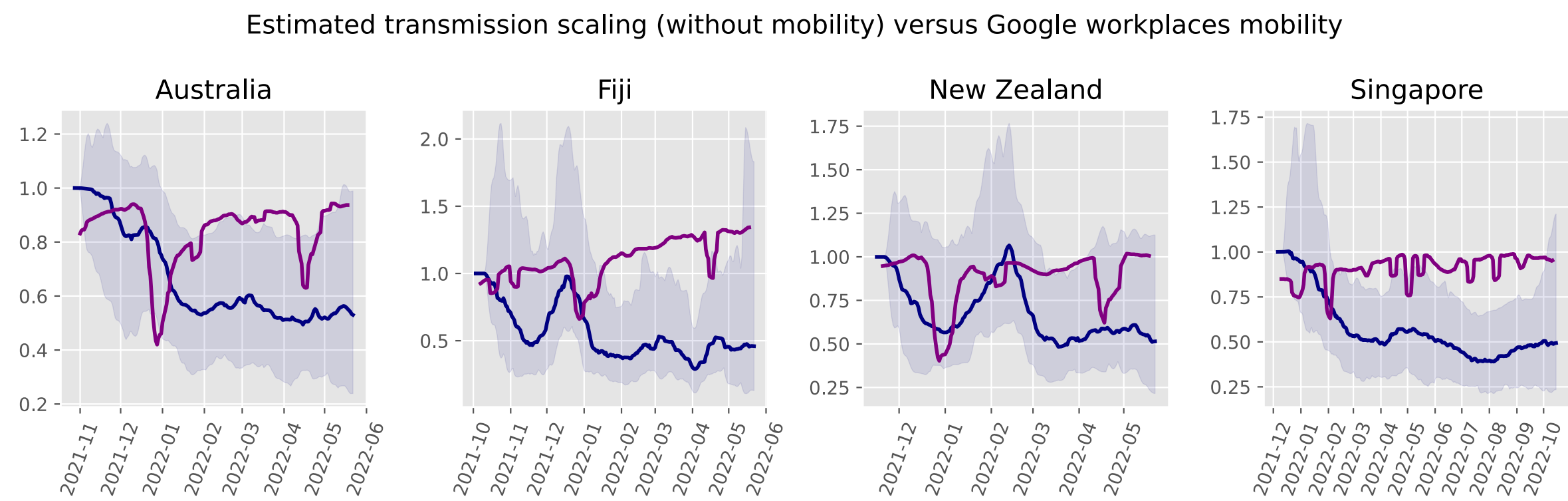

South America

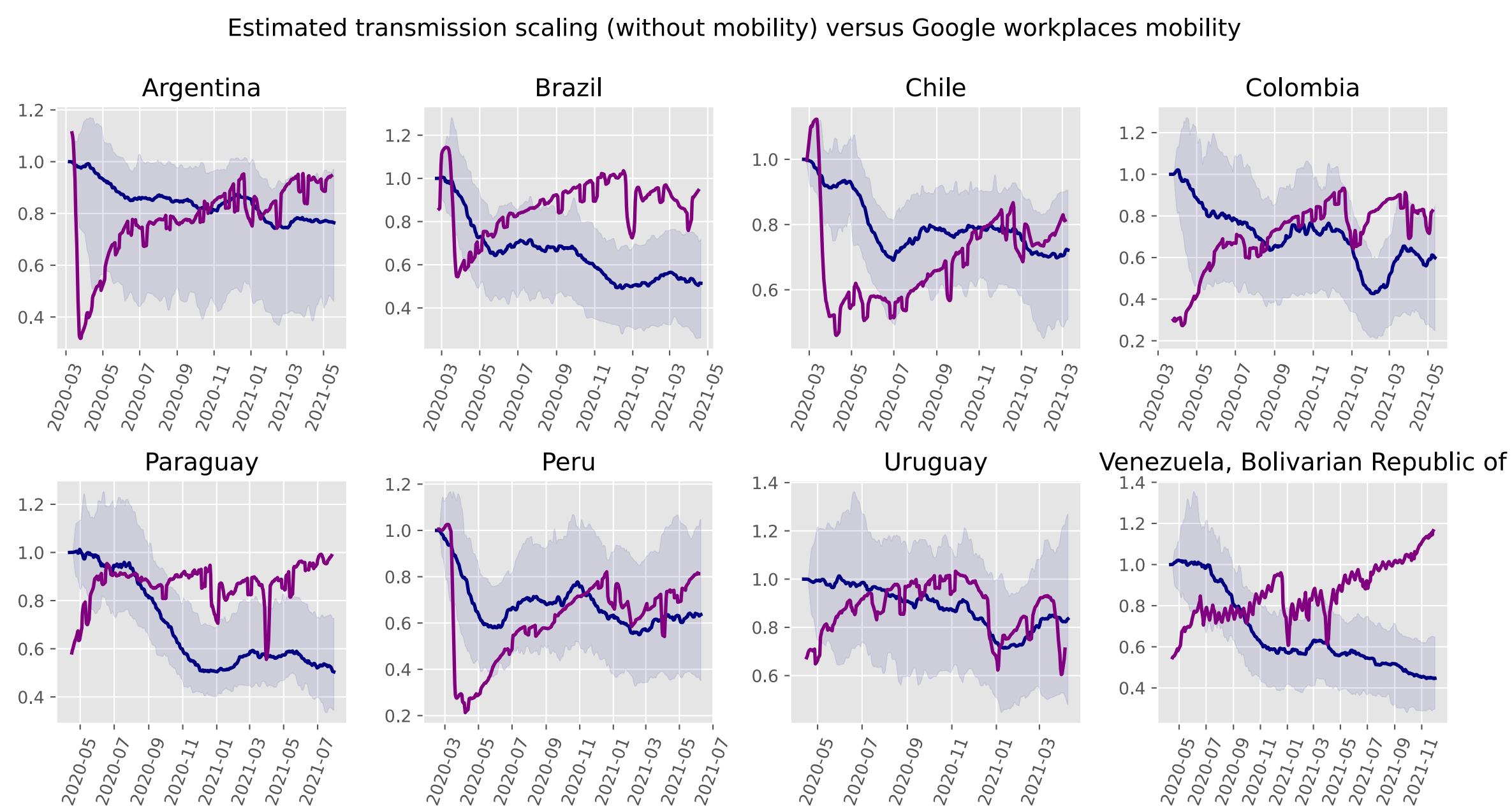

Google residential mobility metric comparison

Mobility values presented as Google data divided by 100, plus one.

Africa

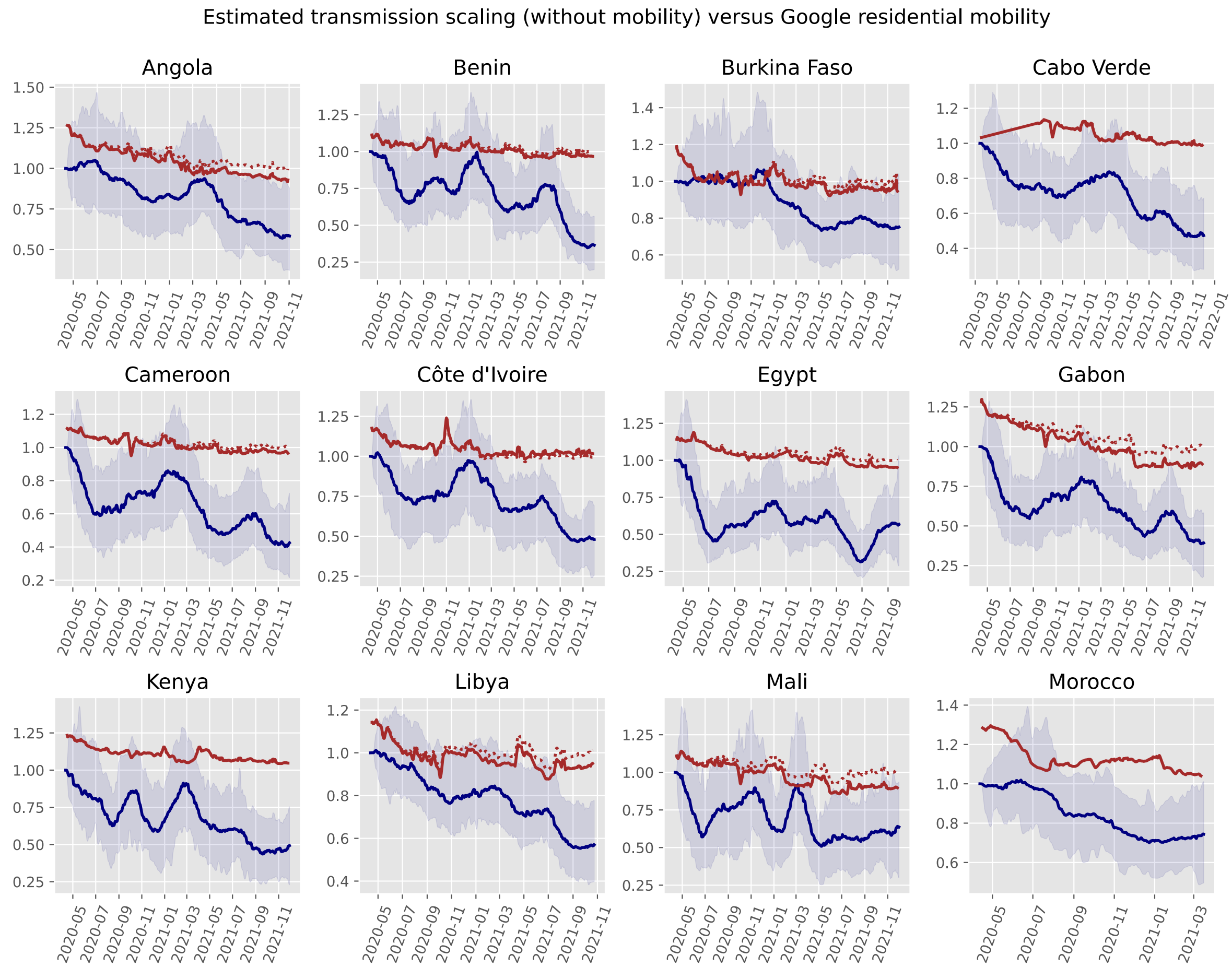

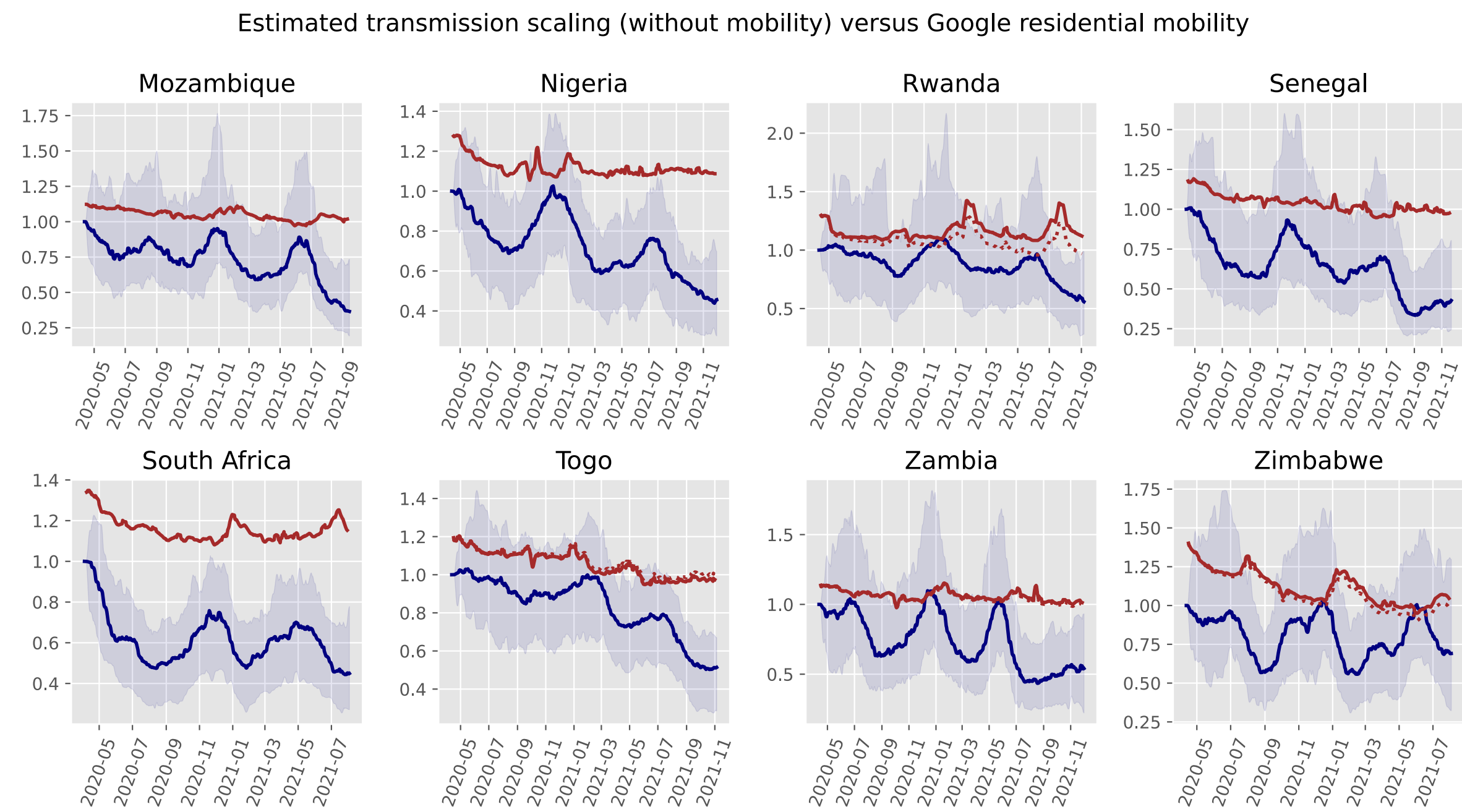
Estimated transmission scaling (without mobility) versus Google residential mobility
Mozambique
Nigeria
Rwanda
Senegal
South Africa
Togo
Zambia
Zimbabwe

Asia

Estimated transmission scaling (without mobility) versus Google residential mobility

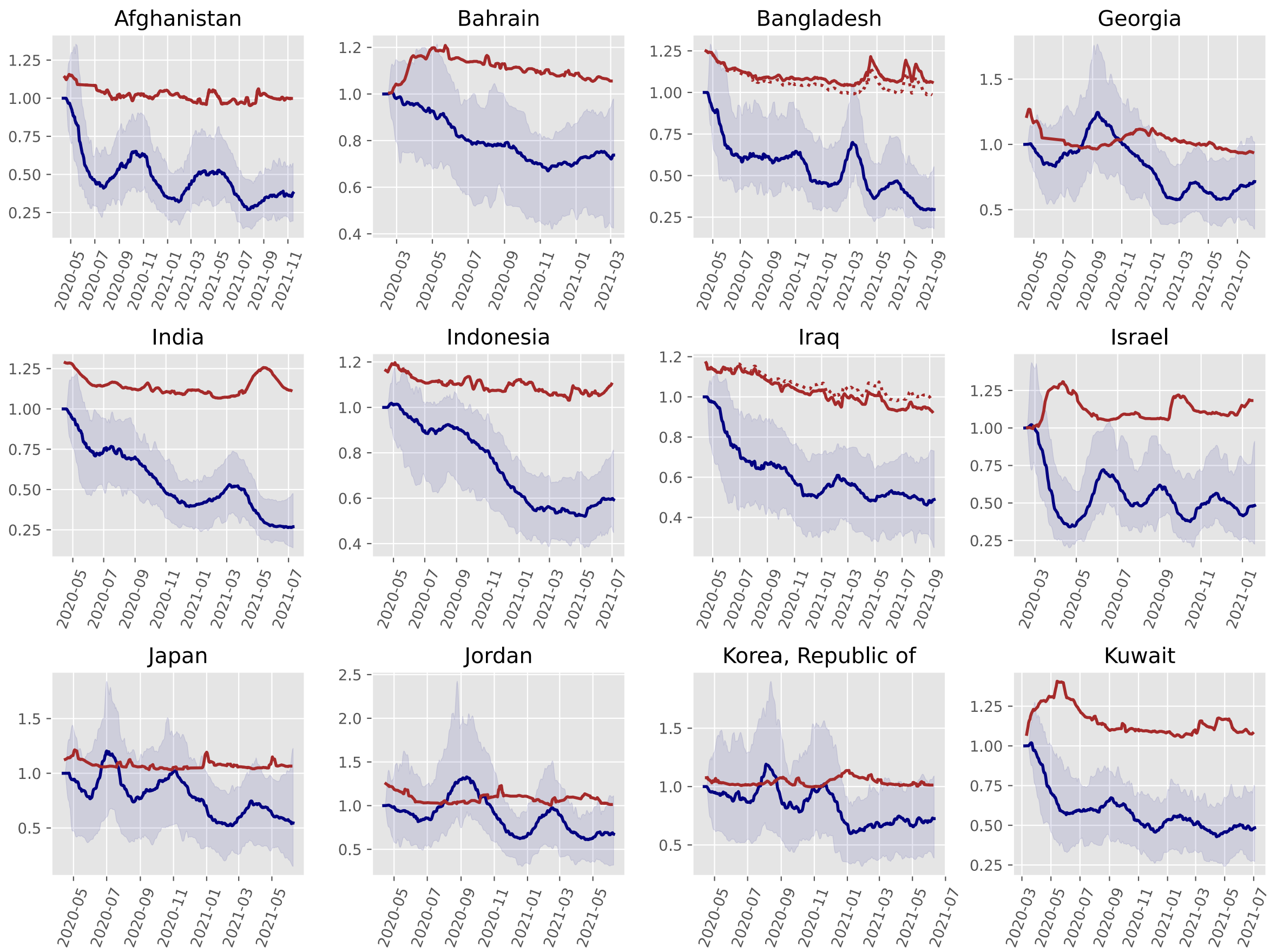

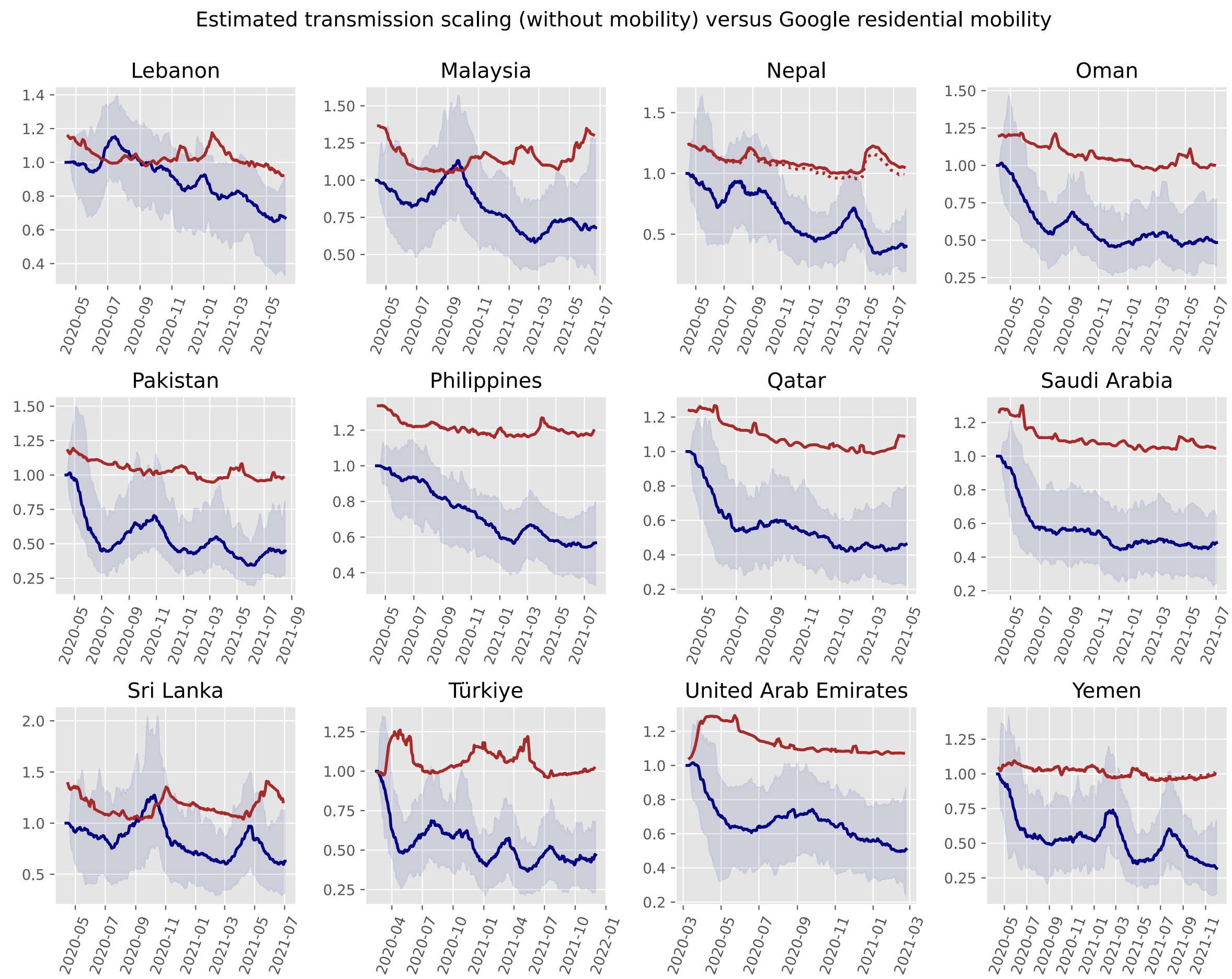
Estimated transmission scaling (without mobility) versus Google residential mobility
Lebanon
Malaysia
Nepal
Oman
Pakistan
Philippines
Qatar
Saudi Arabia
Sri Lanka
Türkiye
United Arab Emirates
Yemen

Europe

Estimated transmission scaling (without mobility) versus Google residential mobility

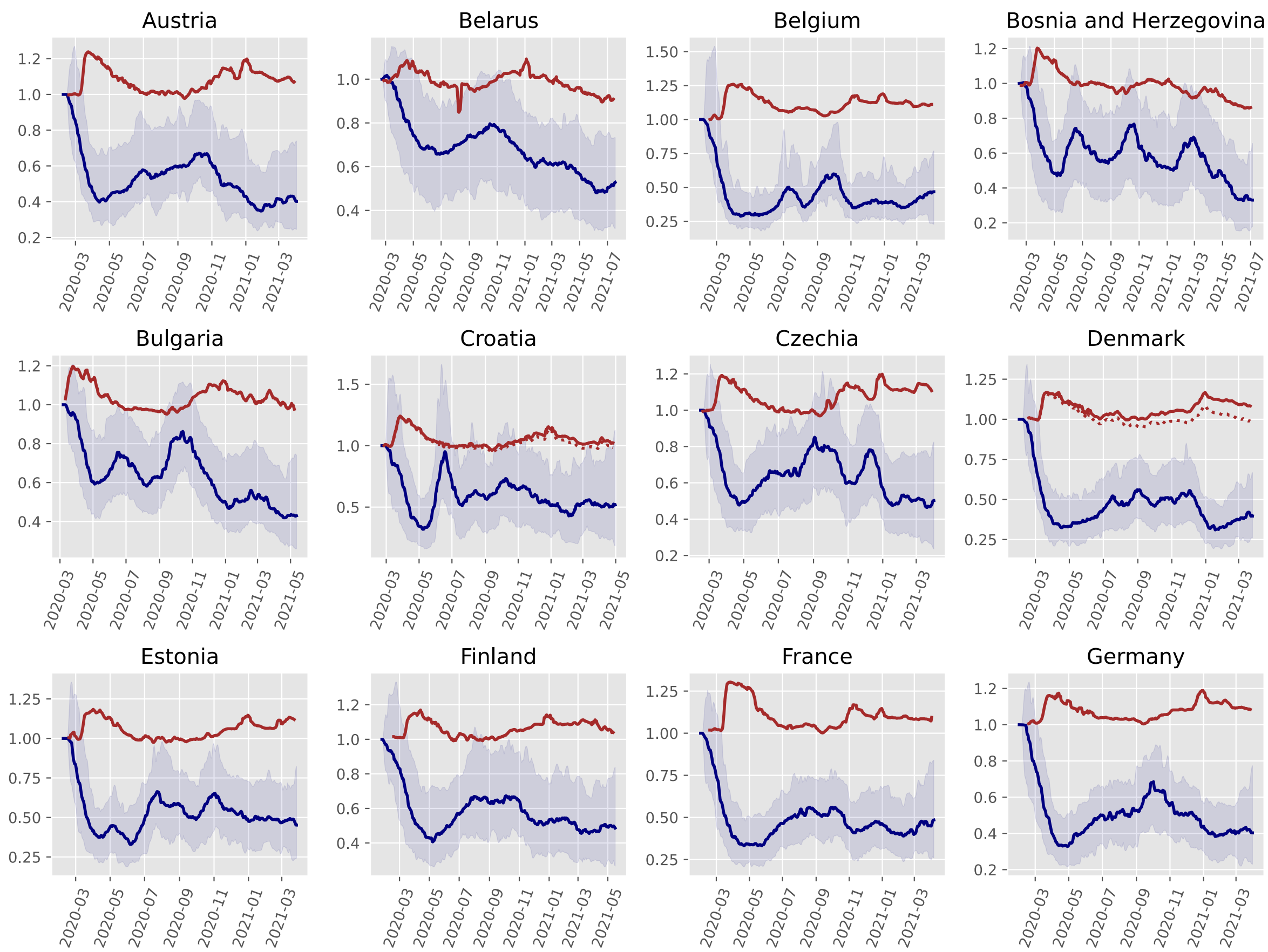

Estimated transmission scaling (without mobility) versus Google residential mobility

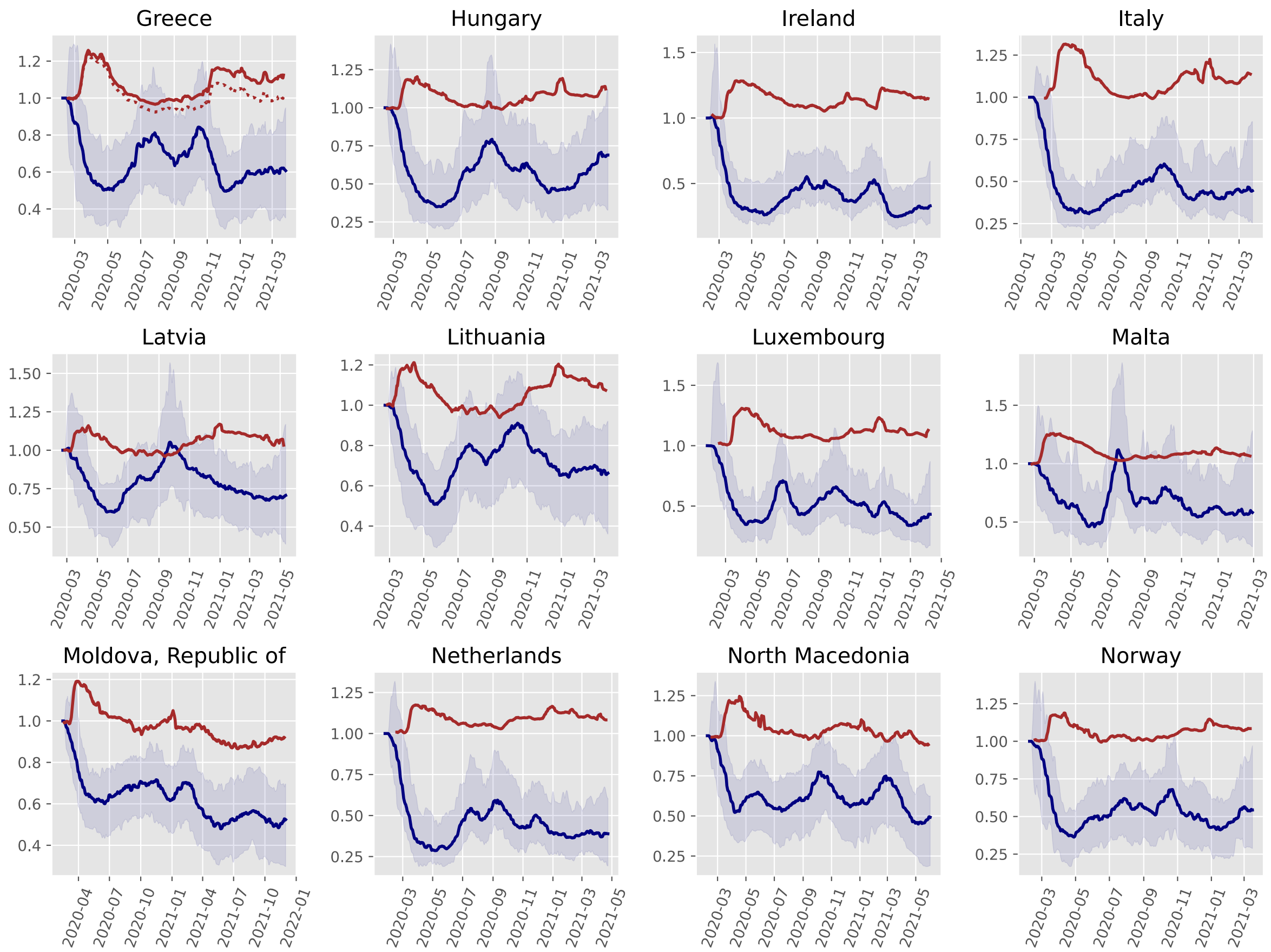

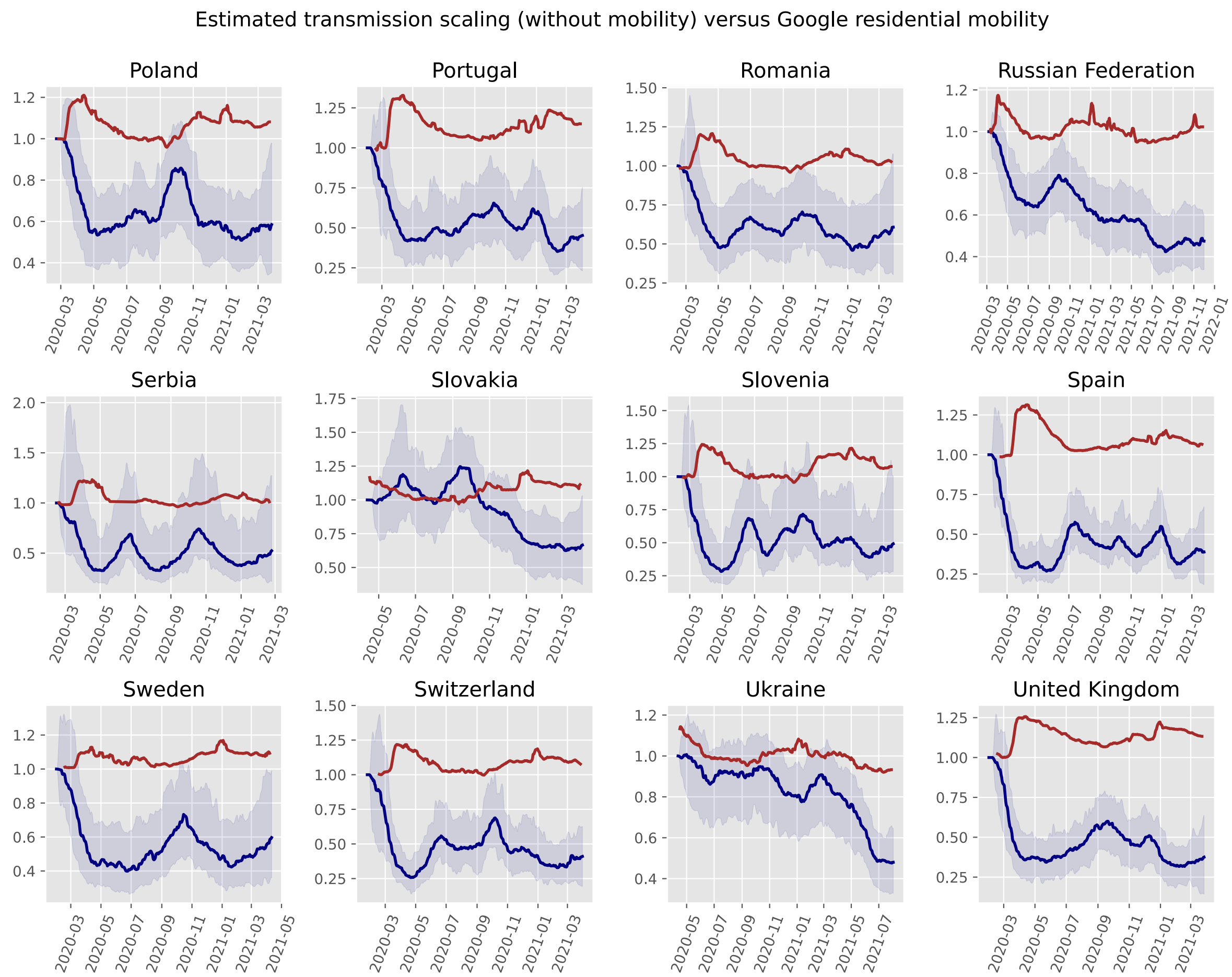

North America

Estimated transmission scaling (without mobility) versus Google residential mobility

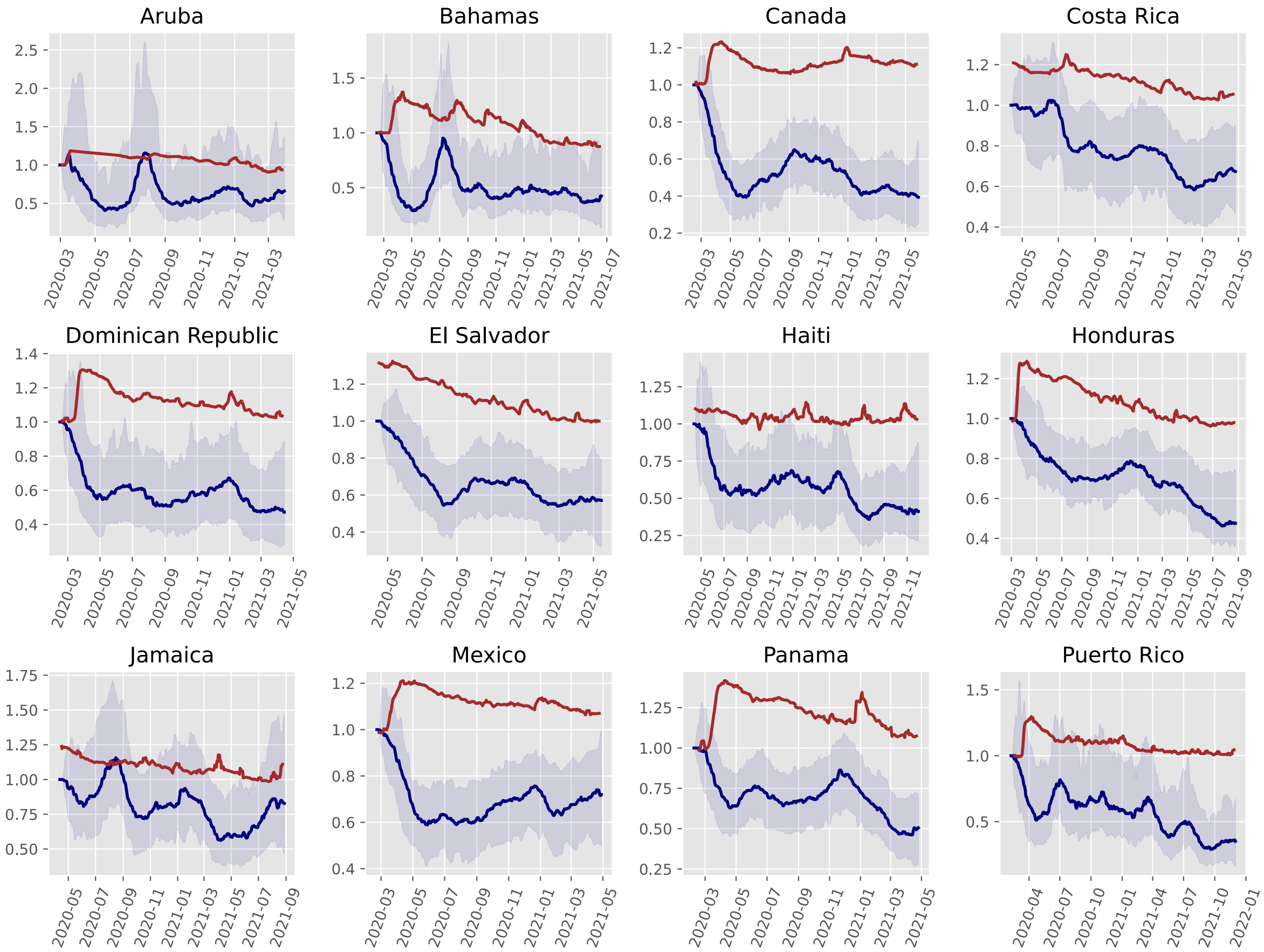

Estimated transmission scaling (without mobility) versus Google residential mobility

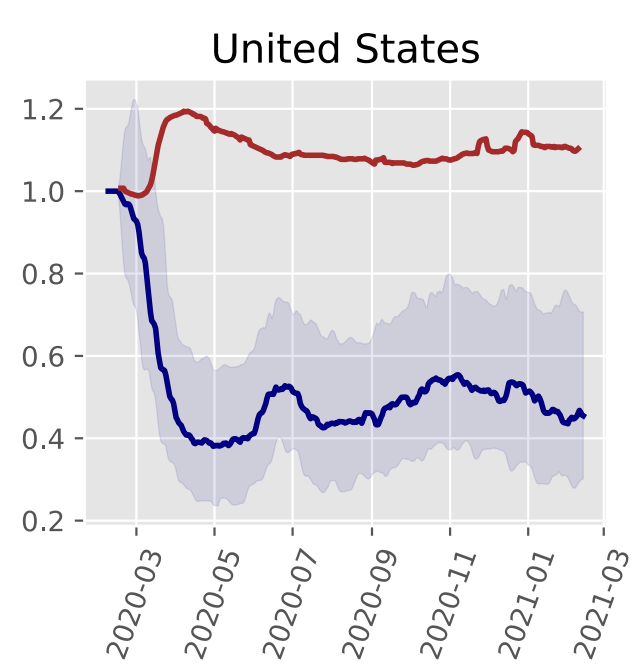

Oceania

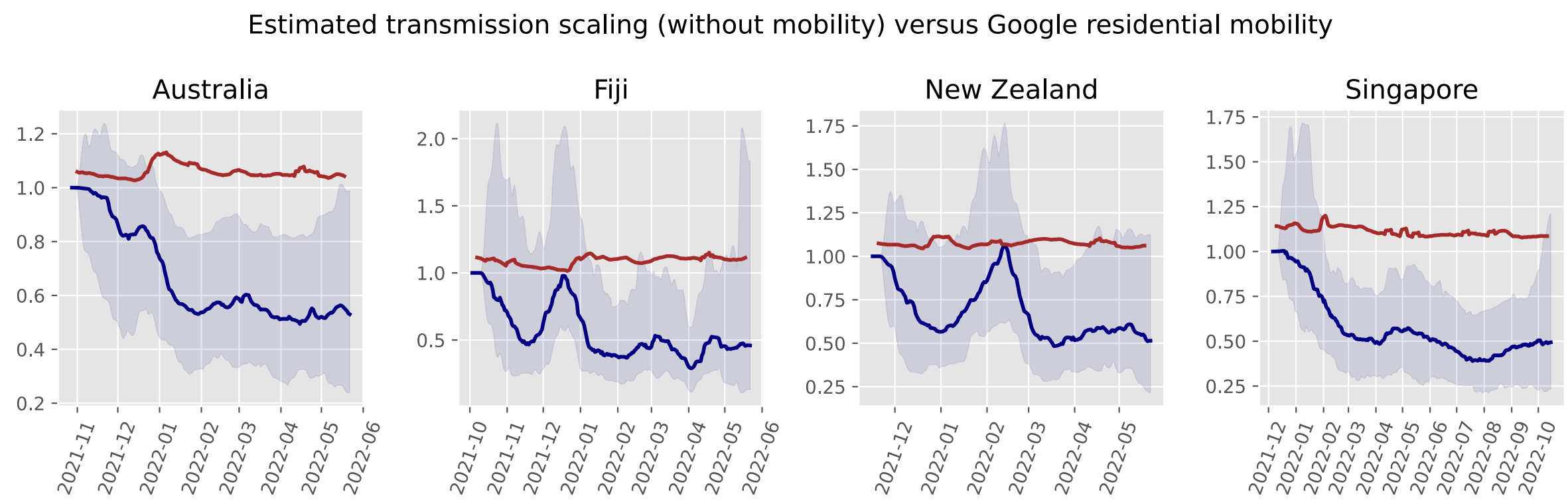

South America

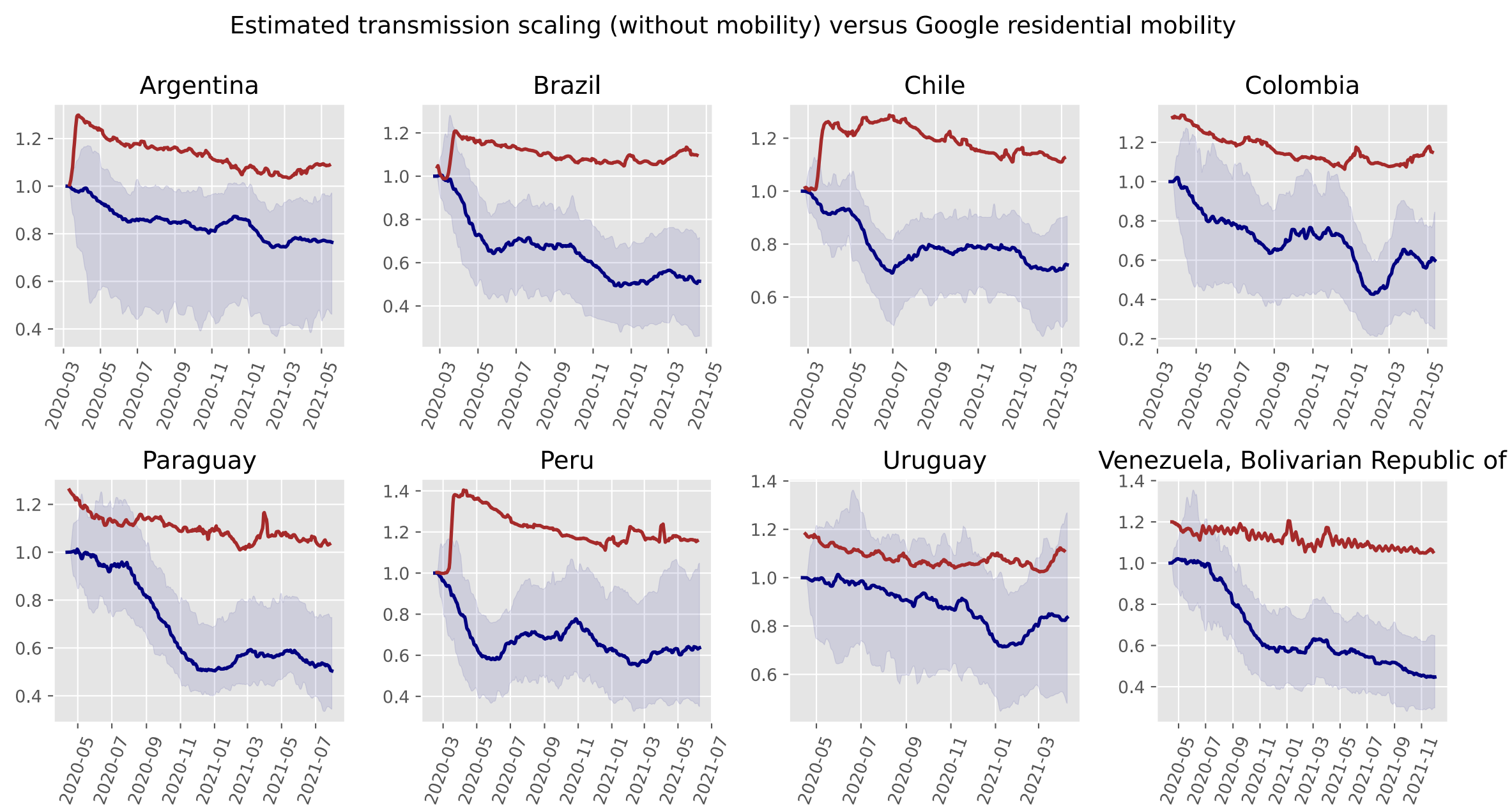

Facebook tiles visited mobility metric comparison

Mobility values presented as one plus Facebook data.

Africa

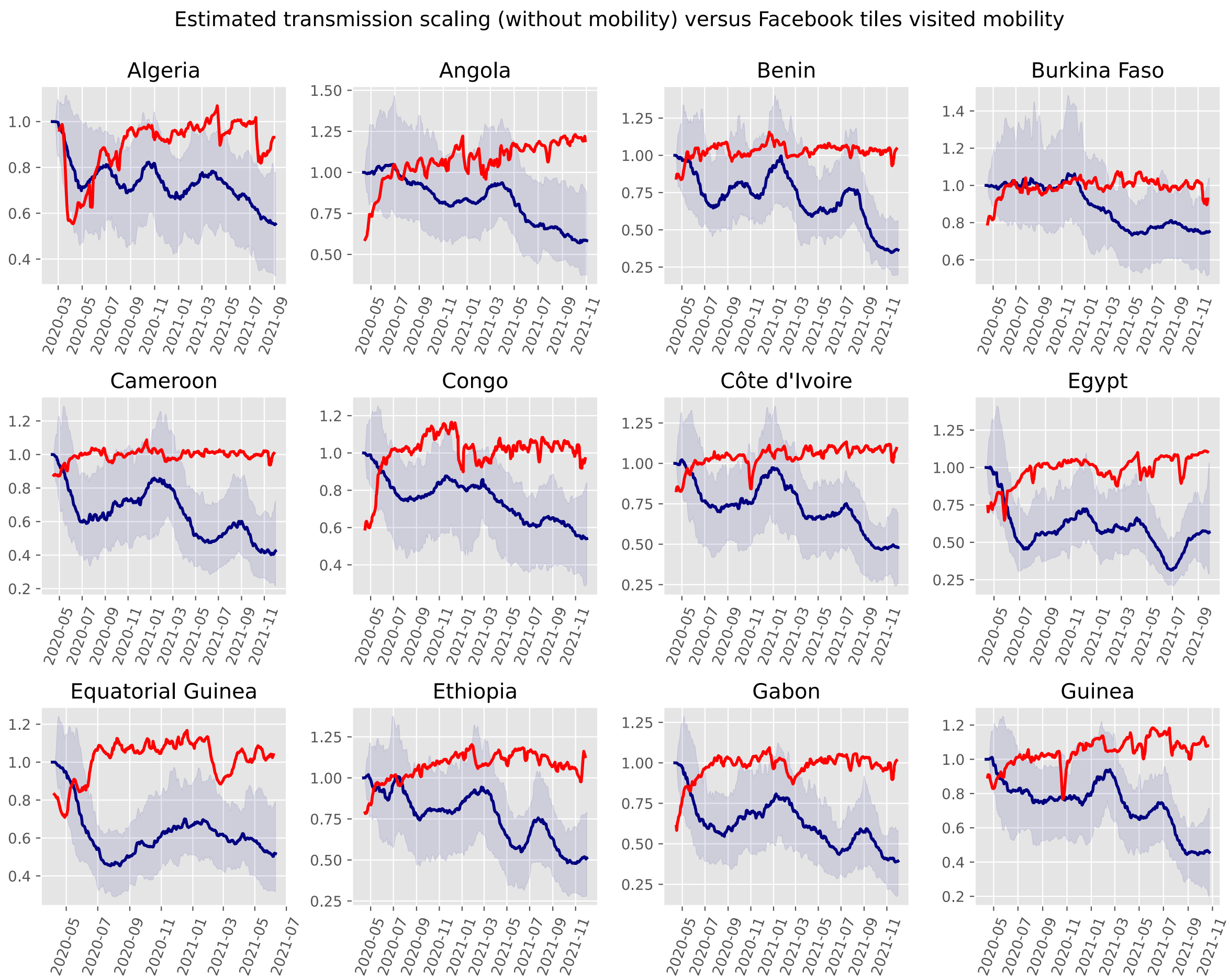

Estimated transmission scaling (without mobility) versus Facebook tiles visited mobility

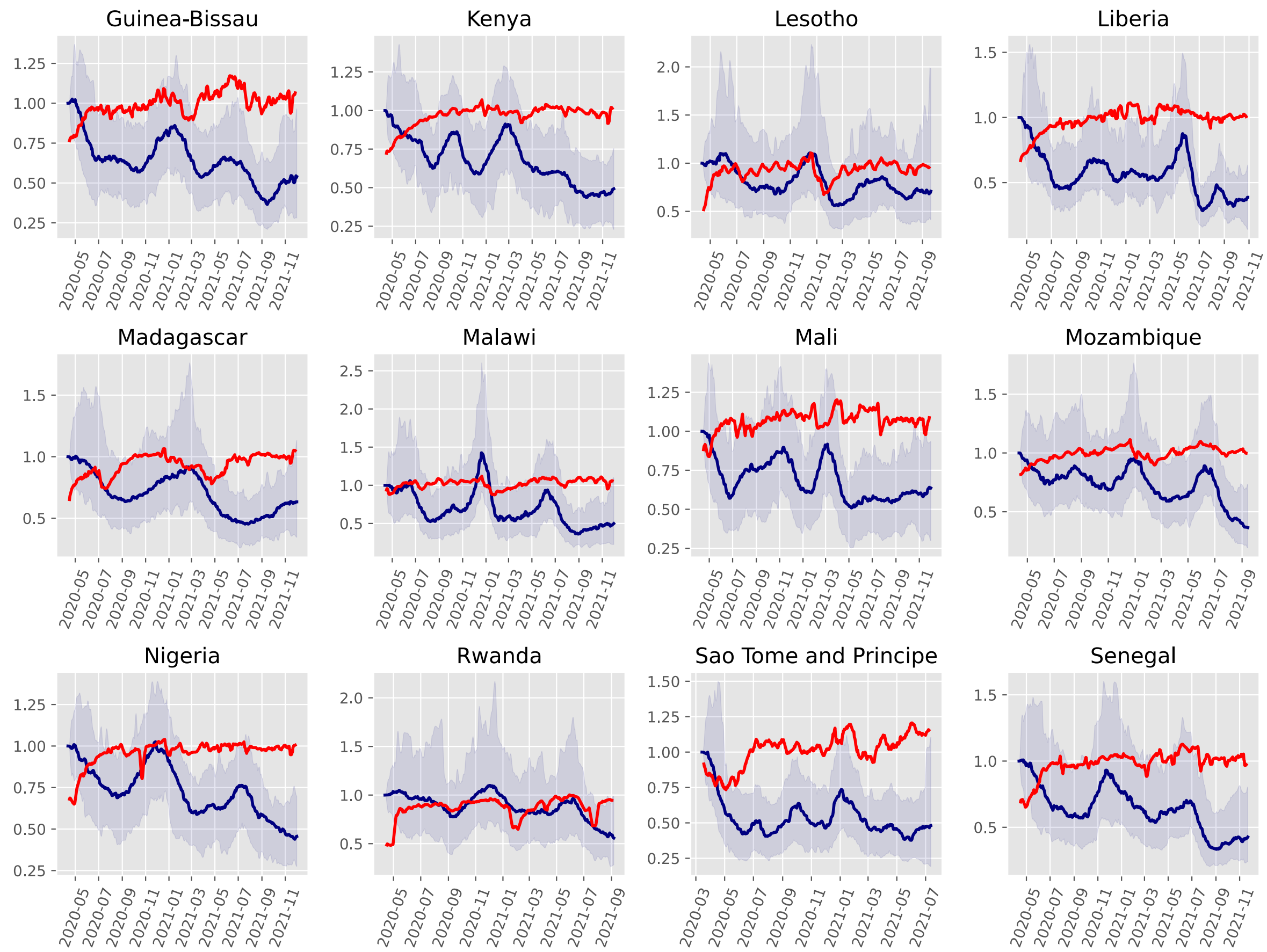

Estimated transmission scaling (without mobility) versus Facebook tiles visited mobility

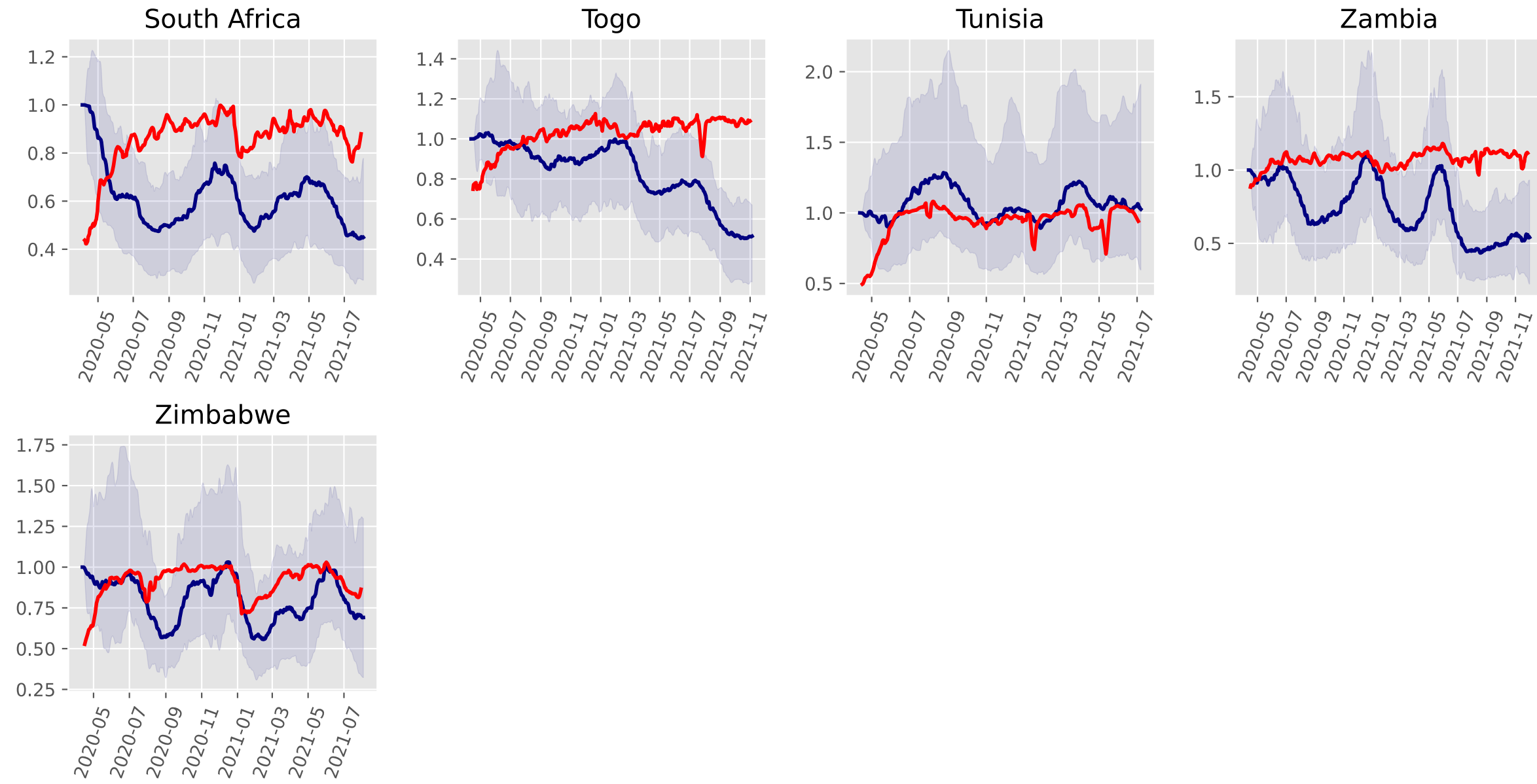

Asia

Estimated transmission scaling (without mobility) versus Facebook tiles visited mobility

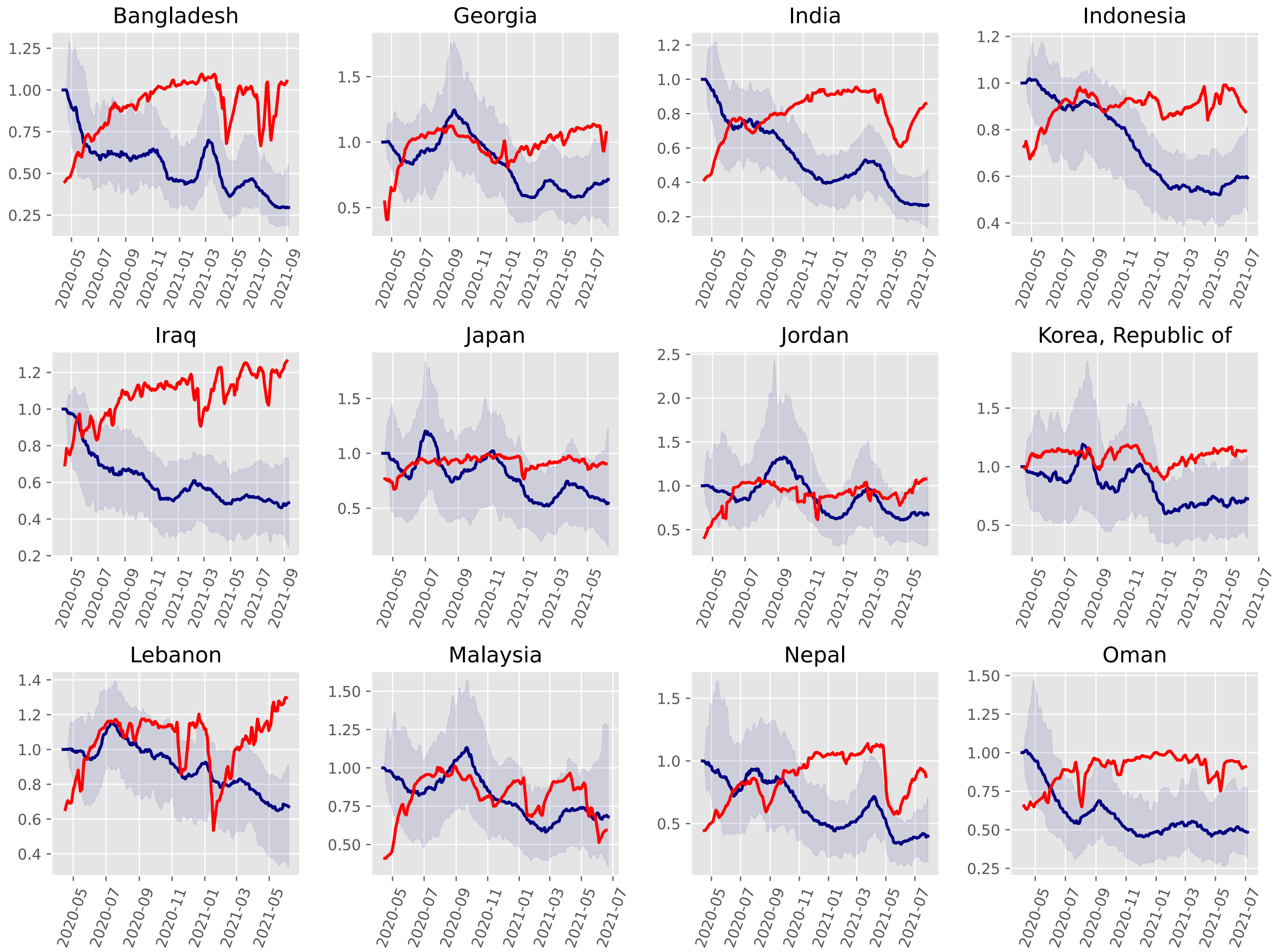

Estimated transmission scaling (without mobility) versus Facebook tiles visited mobility

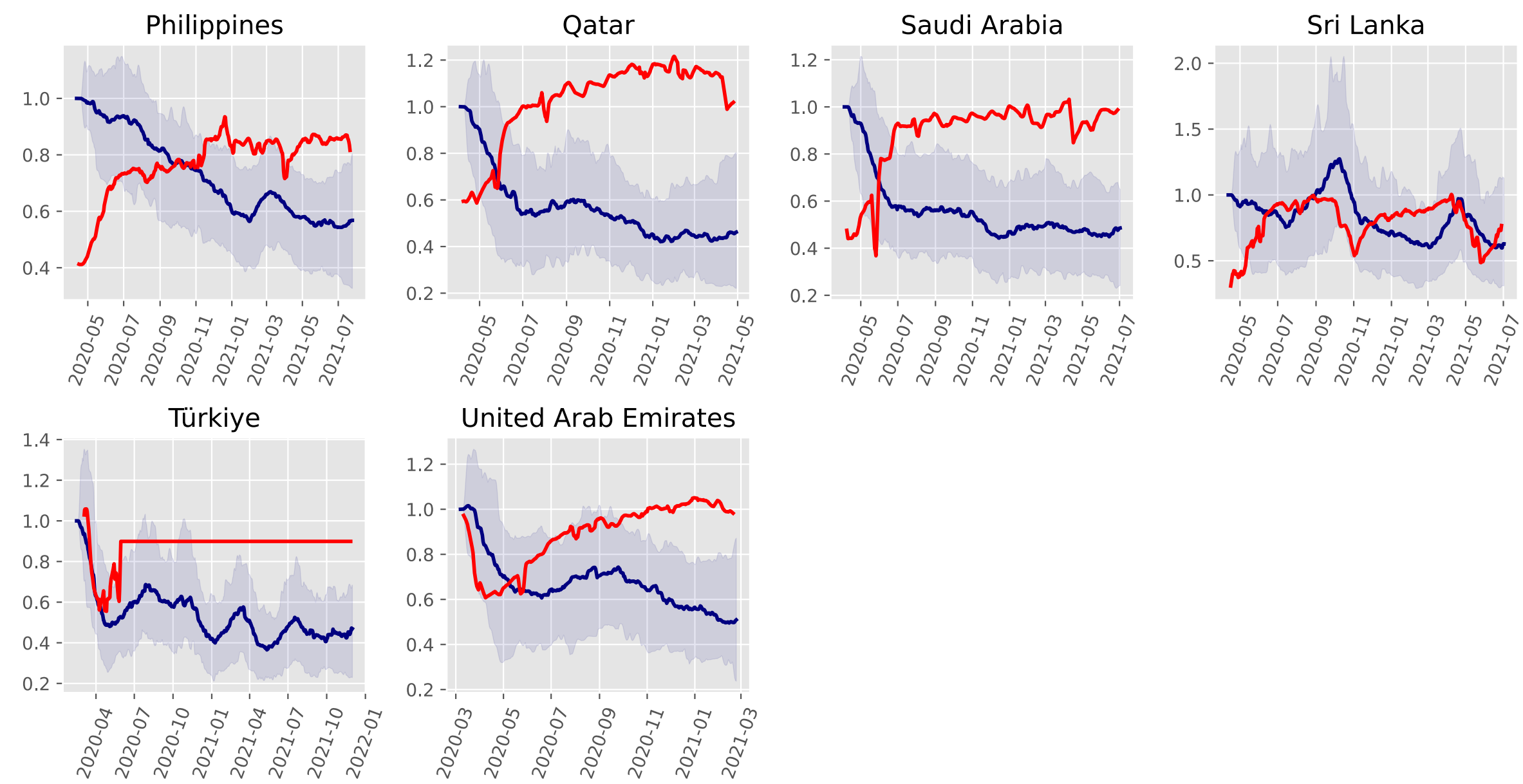

Europe

Estimated transmission scaling (without mobility) versus Facebook tiles visited mobility

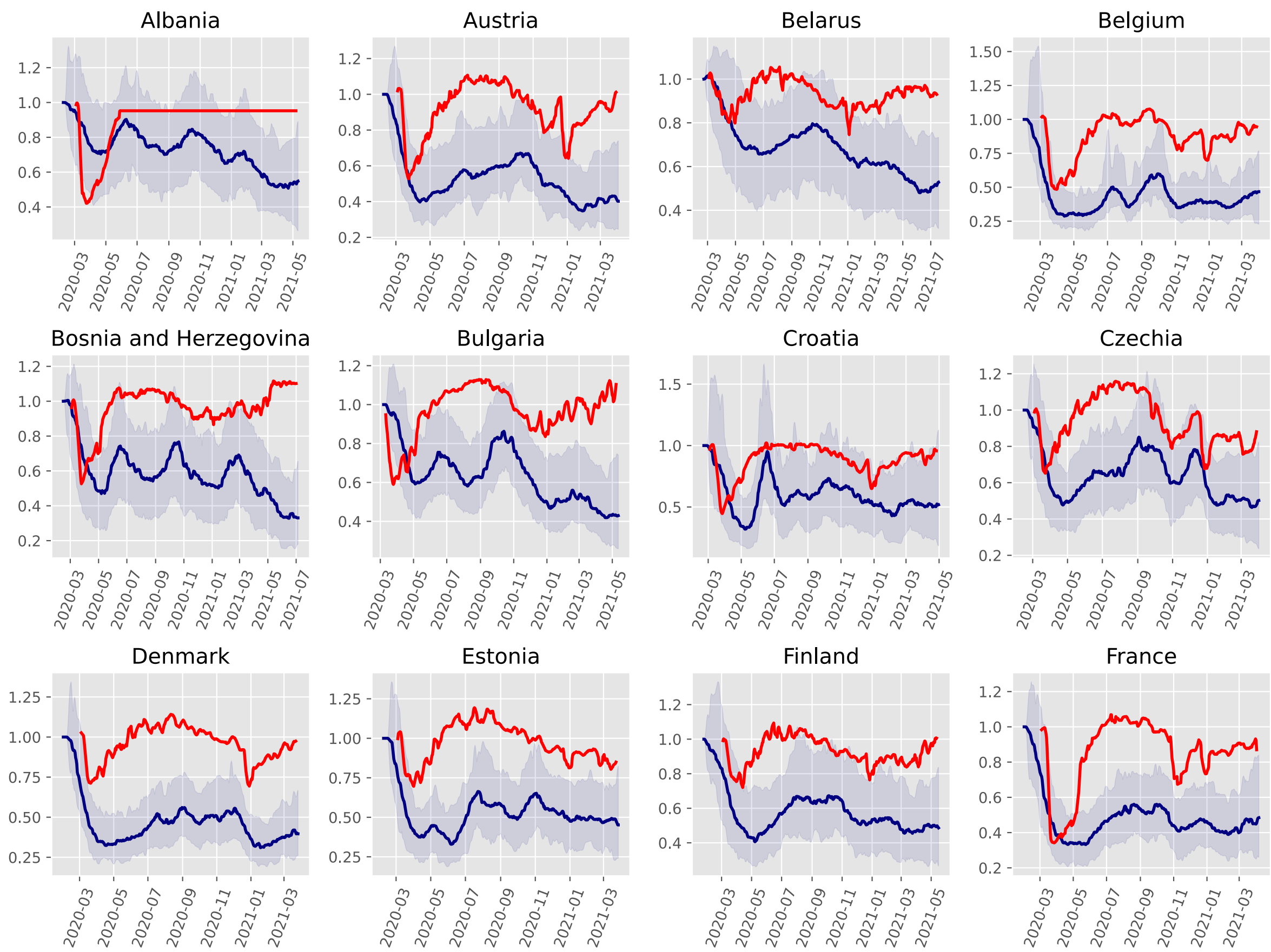

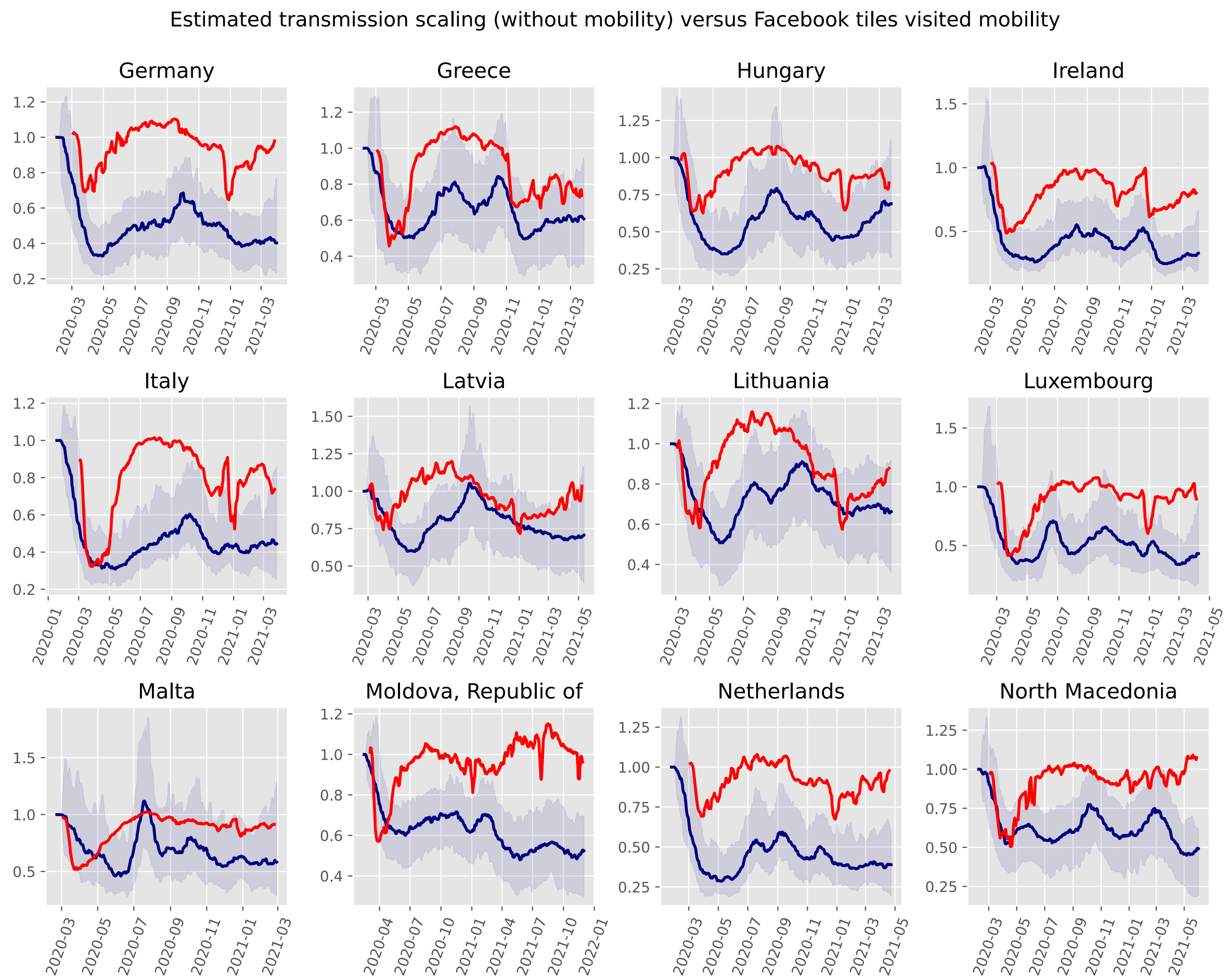
Estimated transmission scaling (without mobility) versus Facebook tiles visited mobility
Germany
Greece
Hungary
Ireland
Italy
Latvia
Lithuania
Luxembourg
Malta
Moldova, Republic of
Netherlands
North Macedonia

Estimated transmission scaling (without mobility) versus Facebook tiles visited mobility

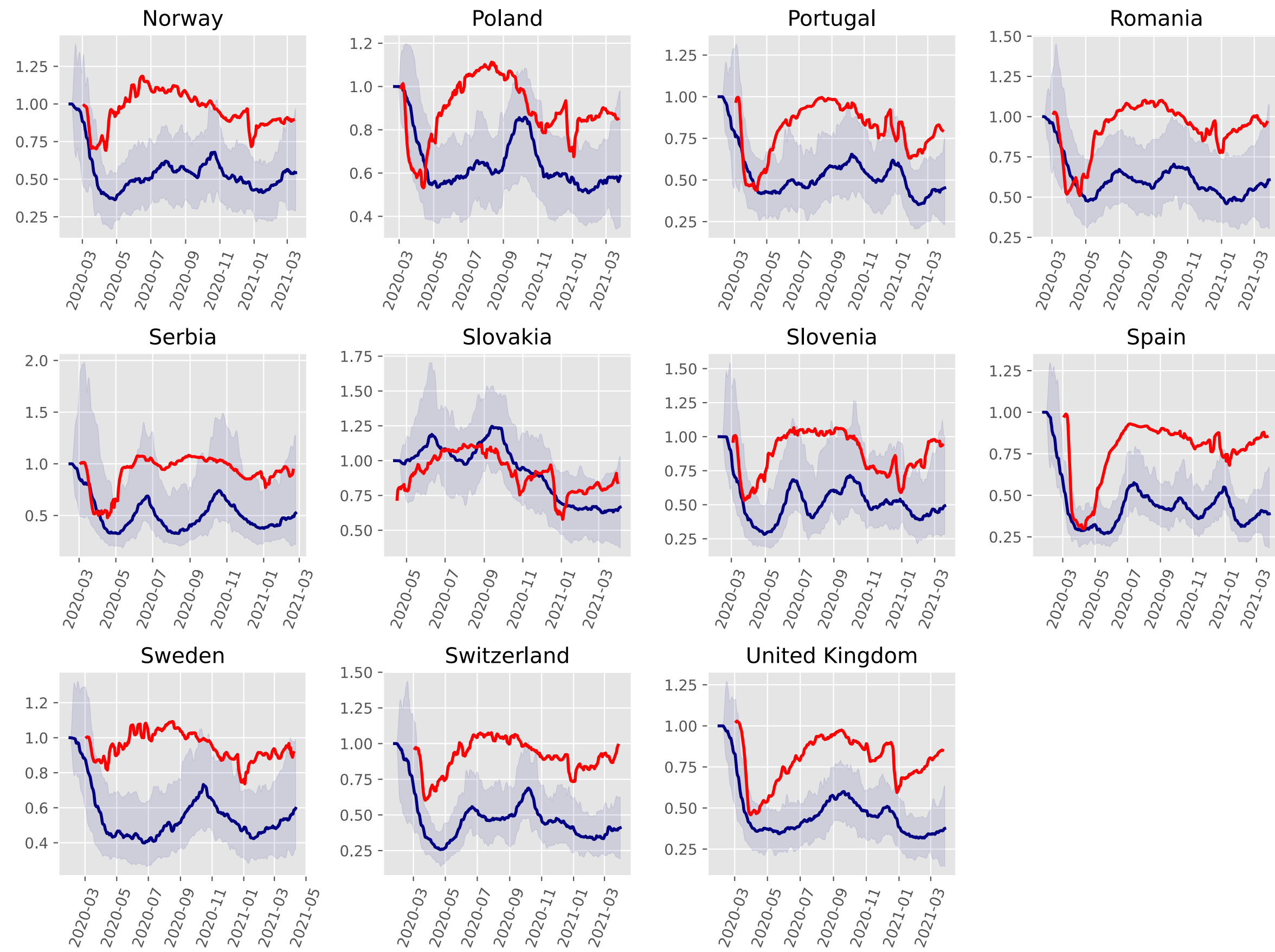

North America

Estimated transmission scaling (without mobility) versus Facebook tiles visited mobility

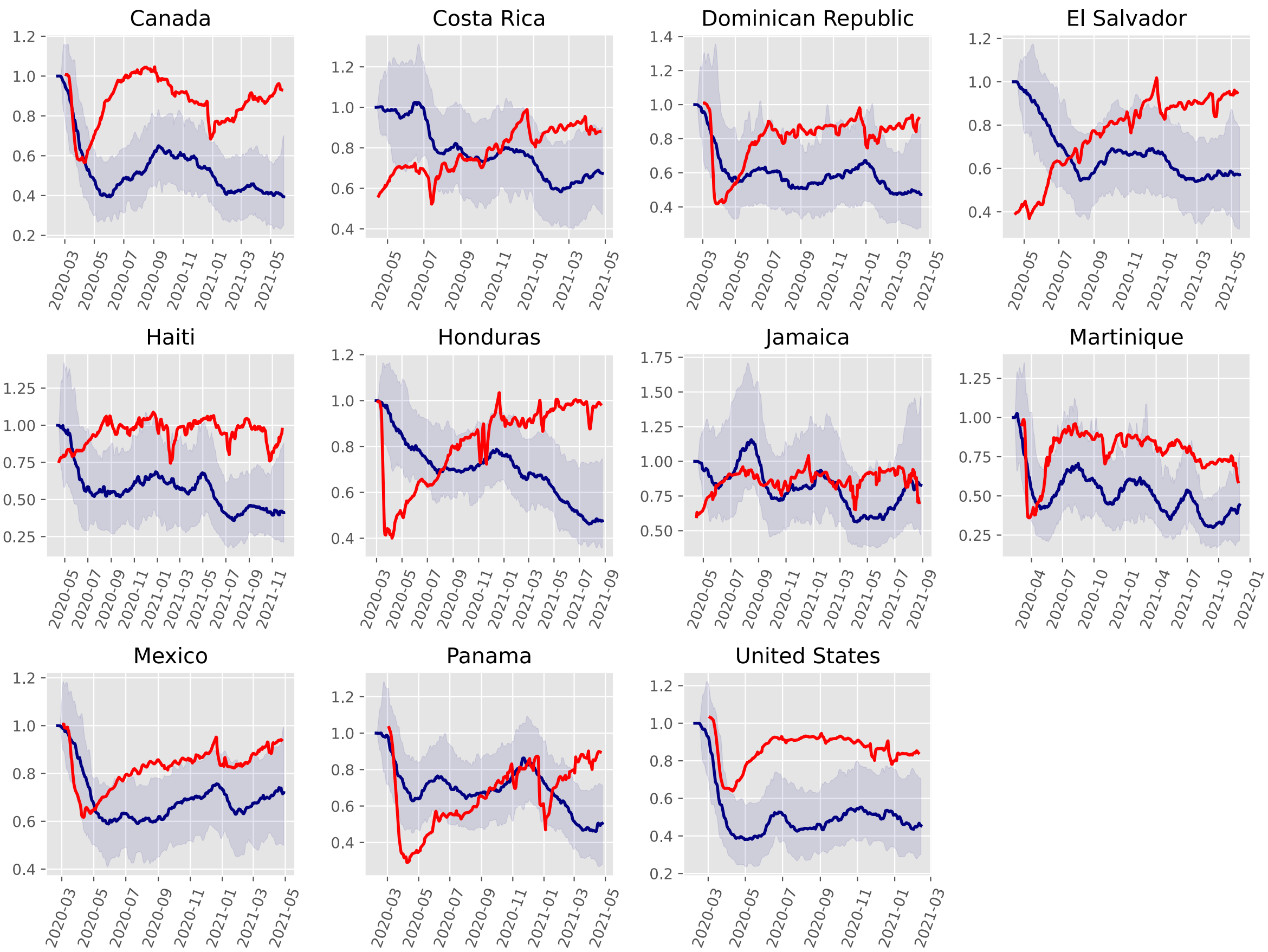

Oceania

Estimated transmission scaling (without mobility) versus Facebook tiles visited mobility

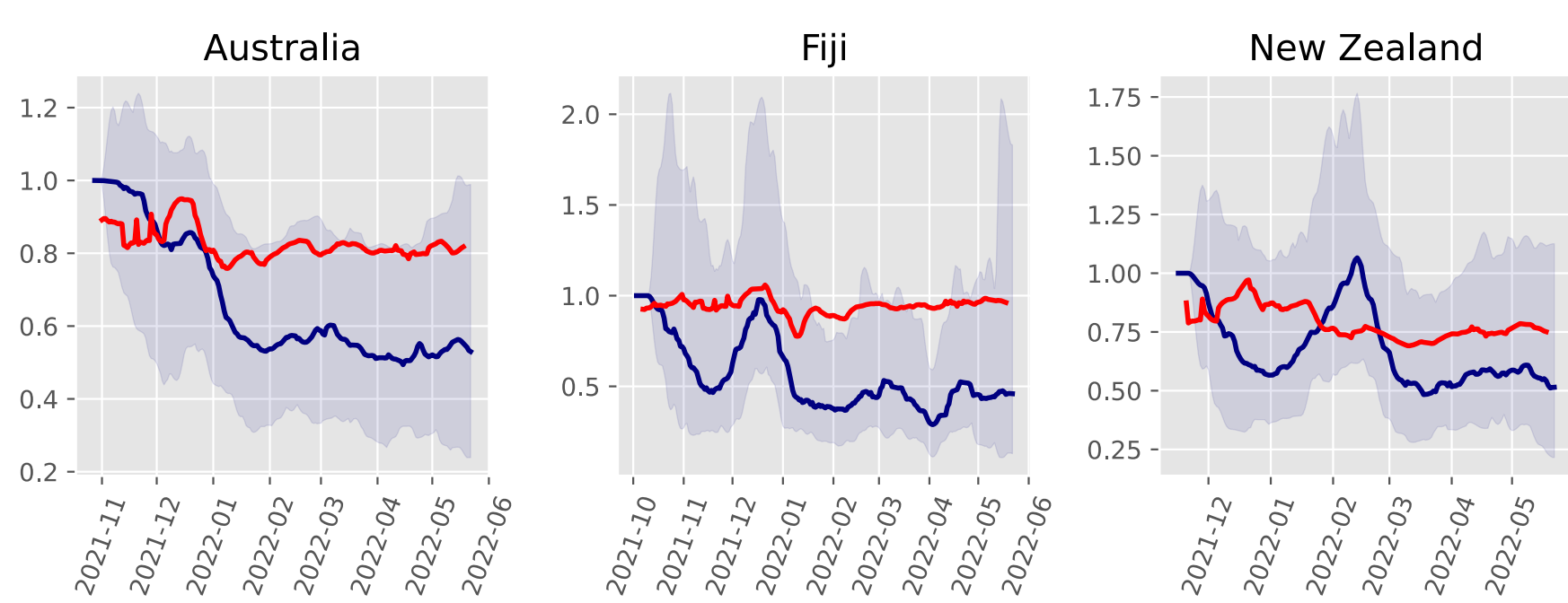

South America

Estimated transmission scaling (without mobility) versus Facebook tiles visited mobility

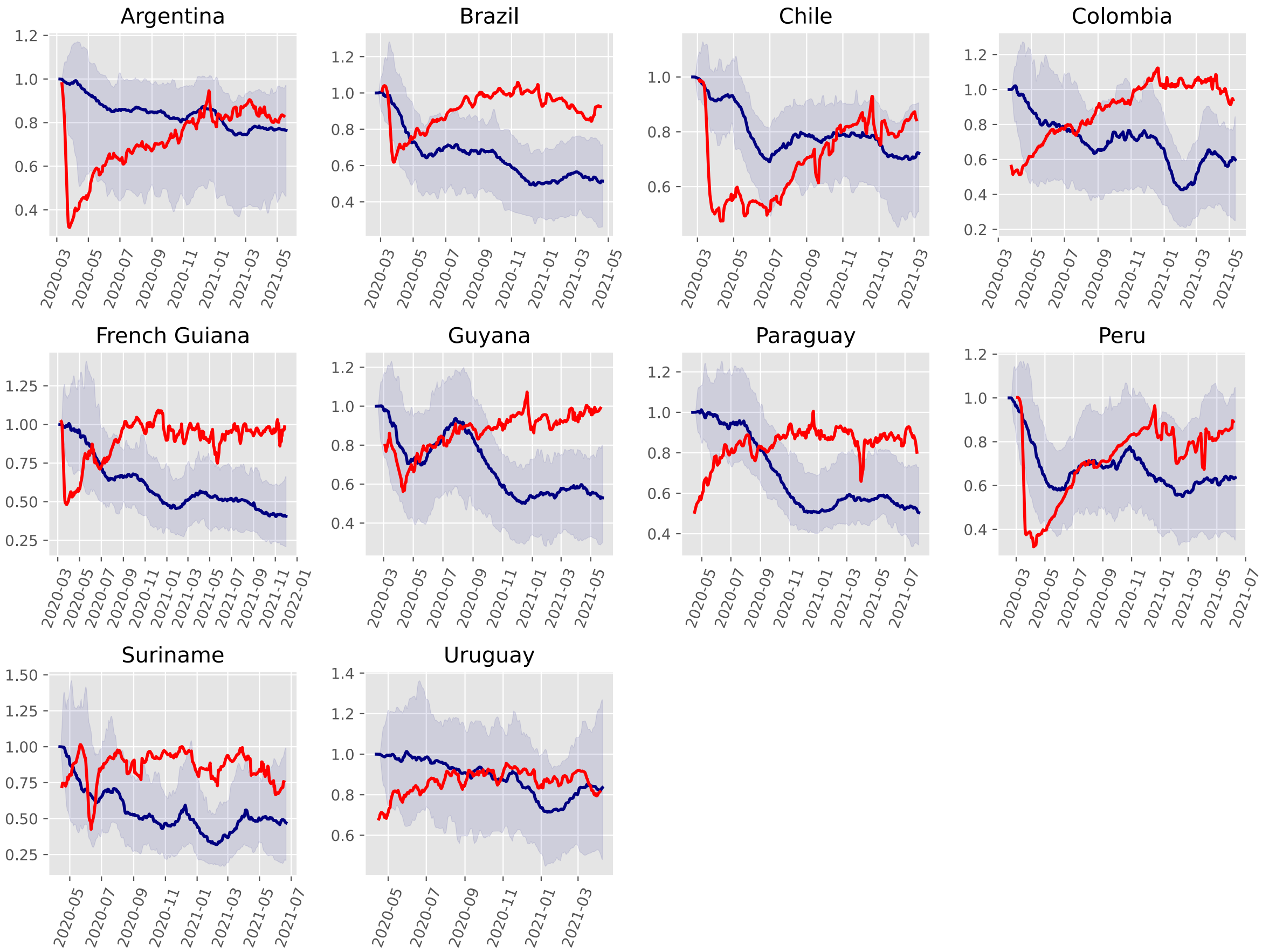

Facebook single tile mobility metric comparison

Mobility values presented as one minus Facebook data.

Africa

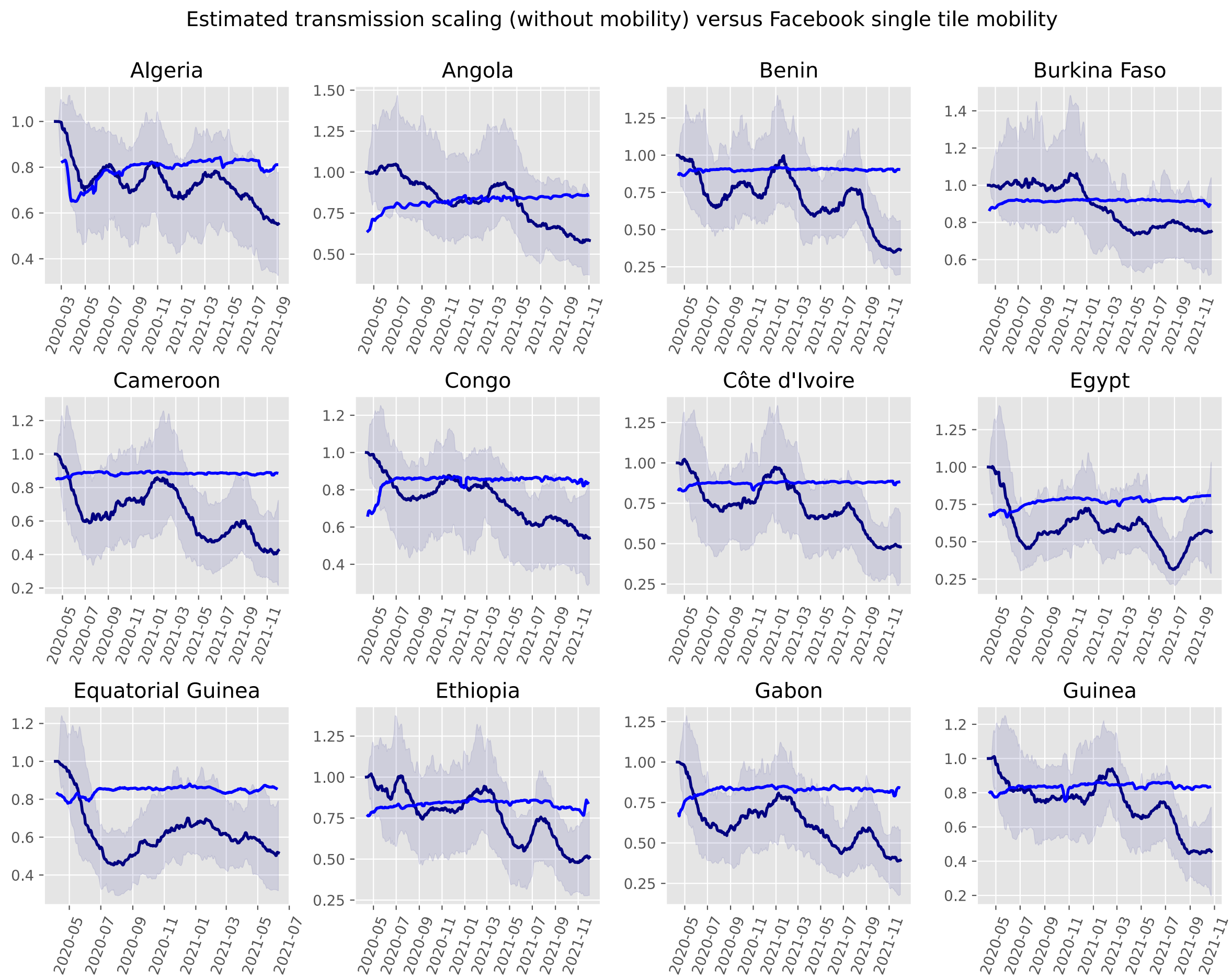

Estimated transmission scaling (without mobility) versus Facebook single tile mobility

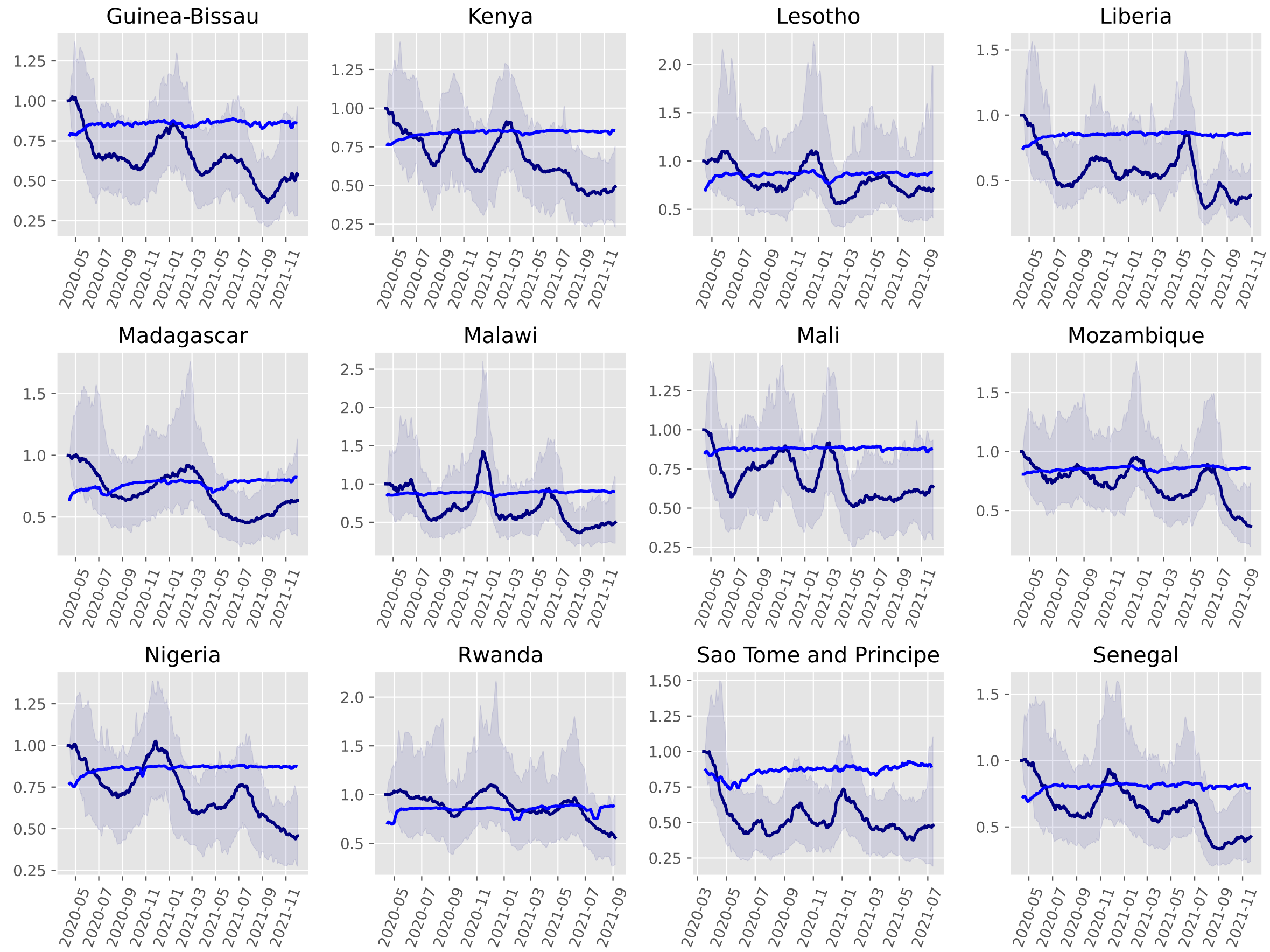

Estimated transmission scaling (without mobility) versus Facebook single tile mobility

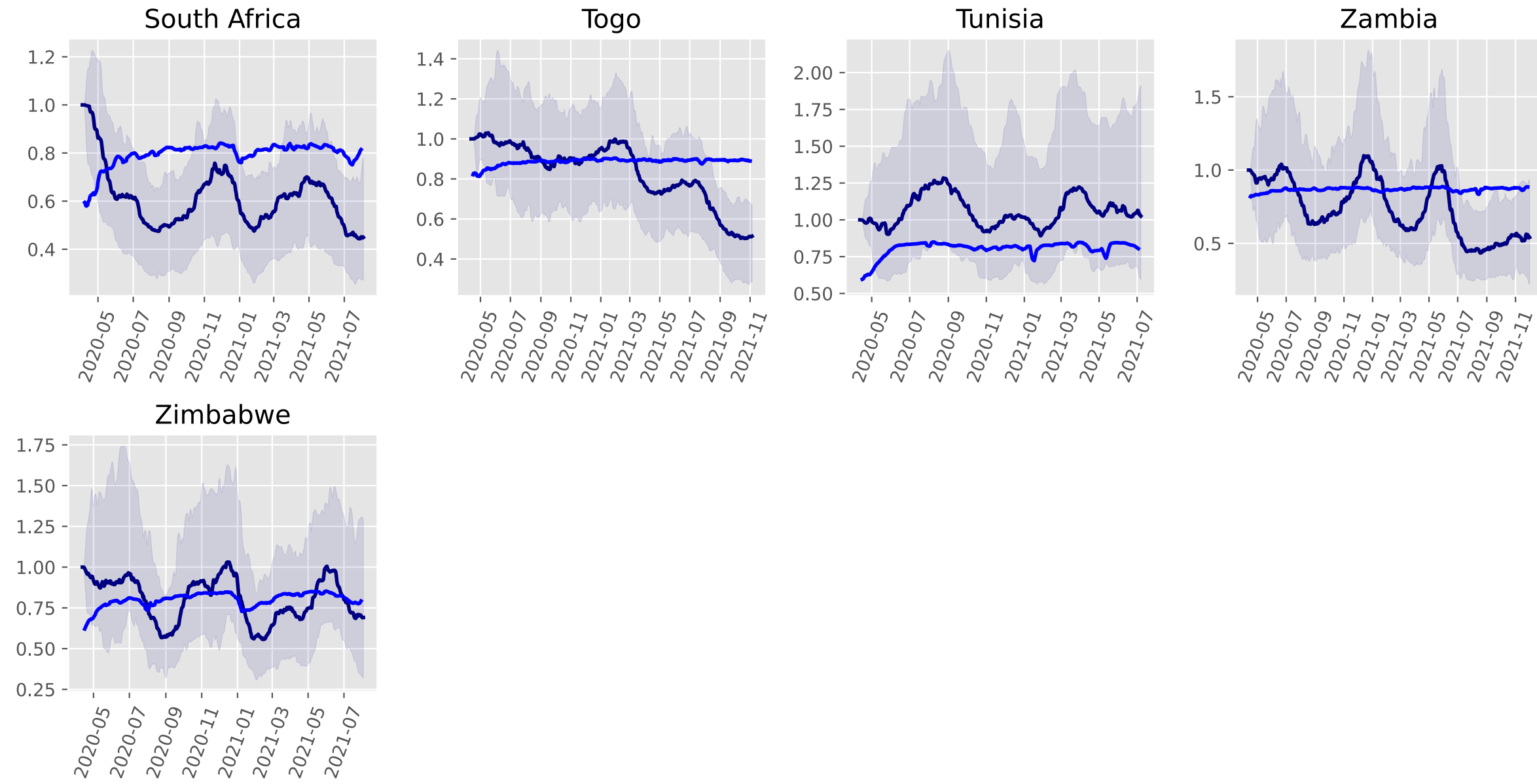

Asia

Estimated transmission scaling (without mobility) versus Facebook single tile mobility

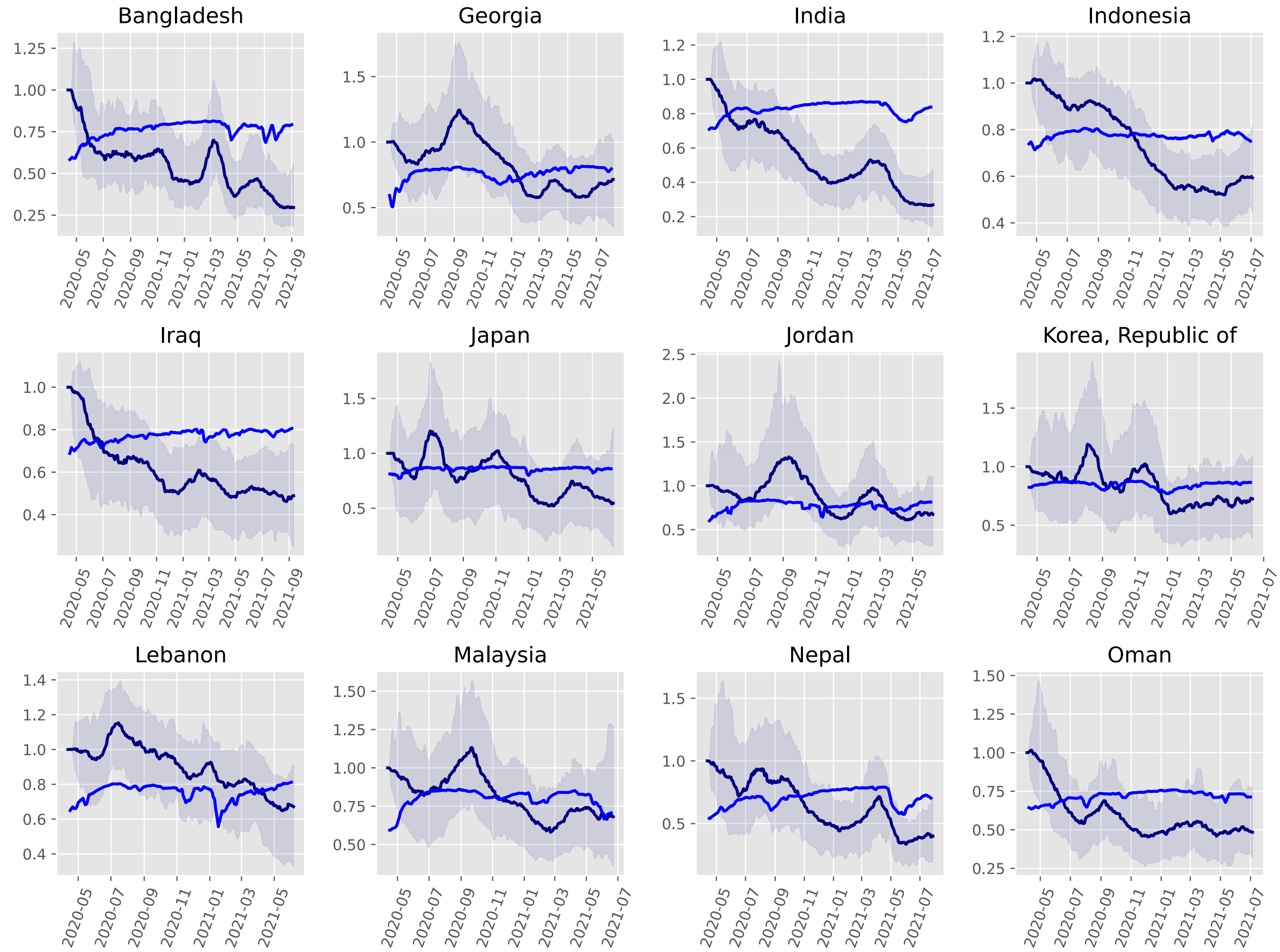

Estimated transmission scaling (without mobility) versus Facebook single tile mobility

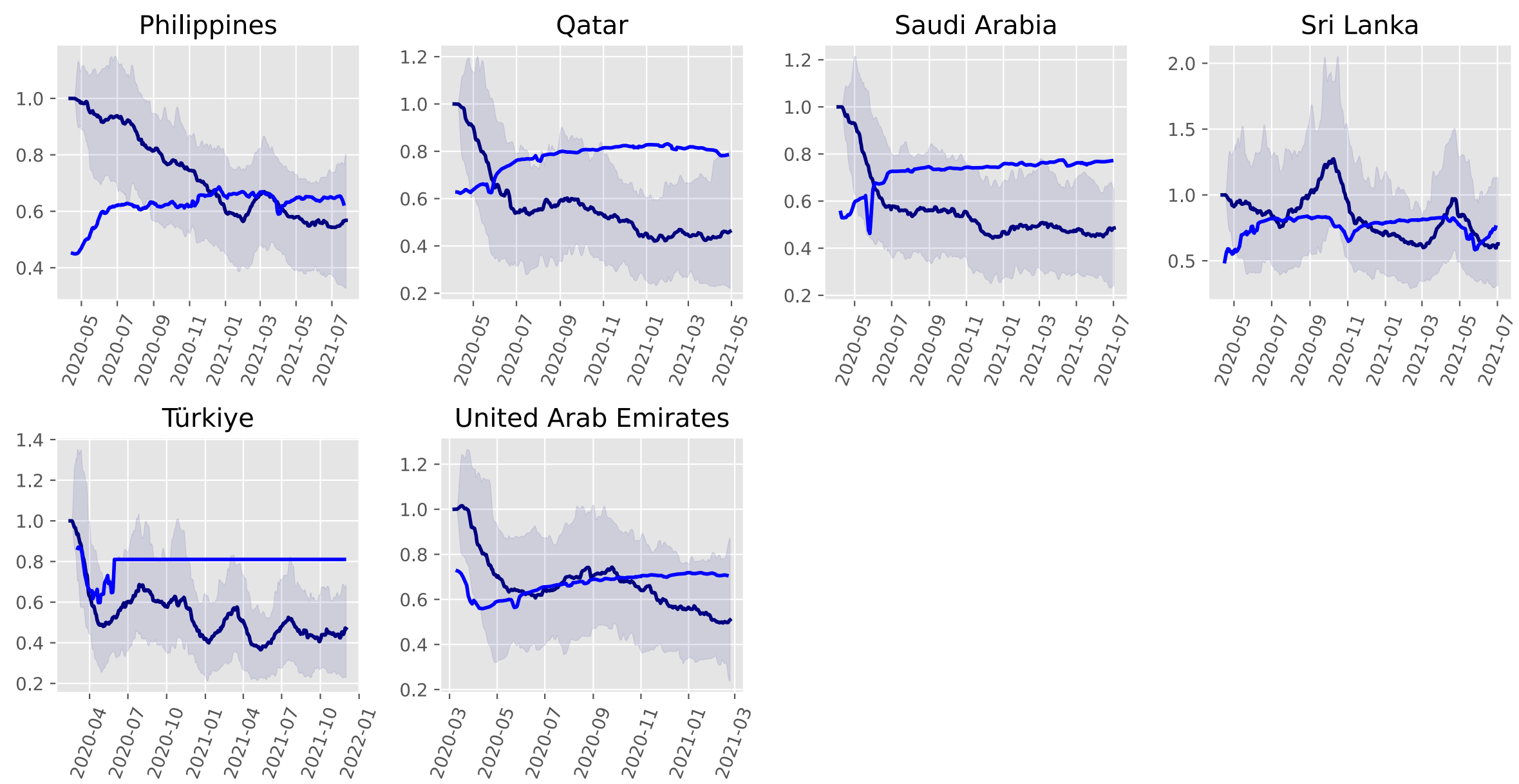

Europe

Estimated transmission scaling (without mobility) versus Facebook single tile mobility

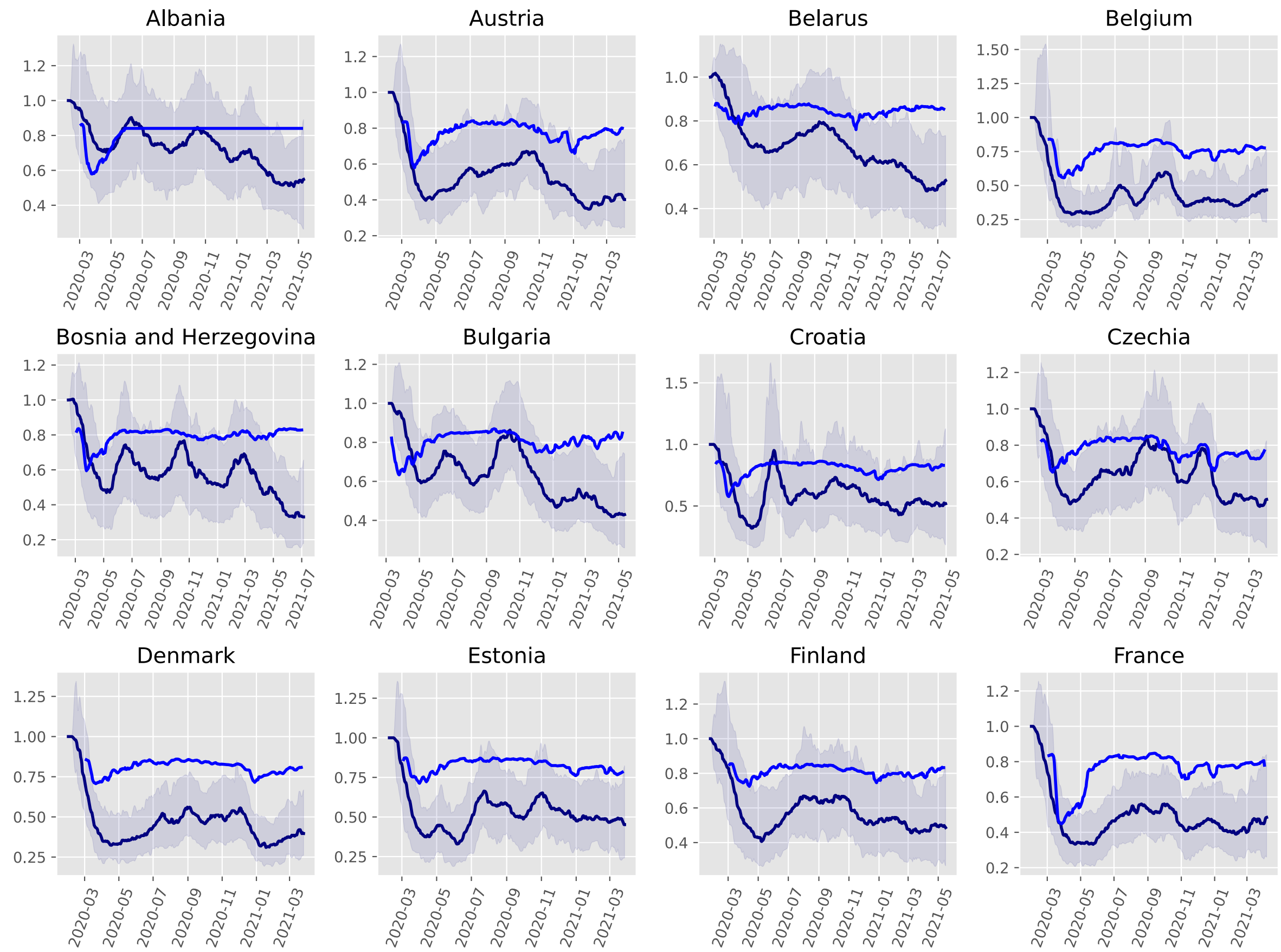

Estimated transmission scaling (without mobility) versus Facebook single tile mobility

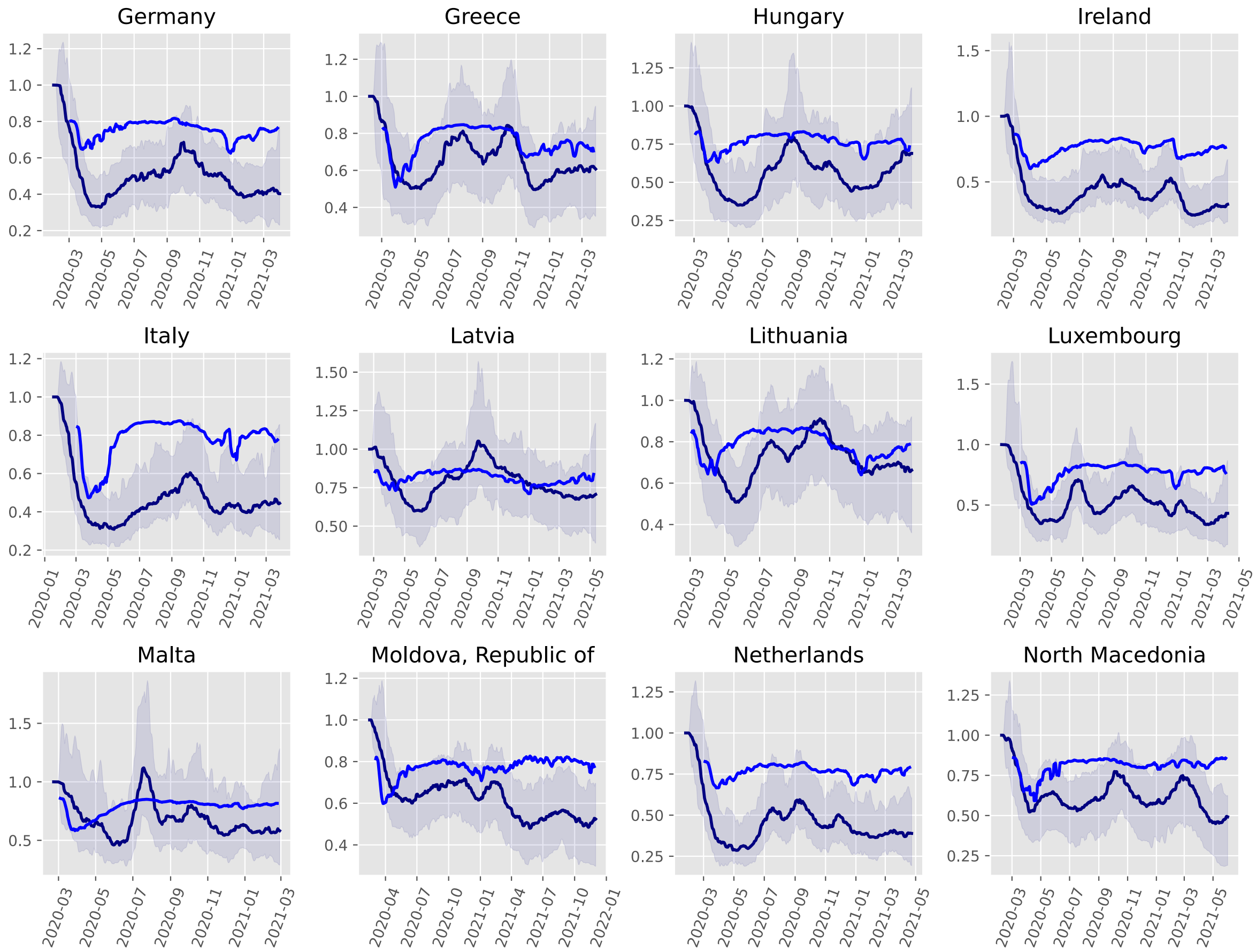

Estimated transmission scaling (without mobility) versus Facebook single tile mobility

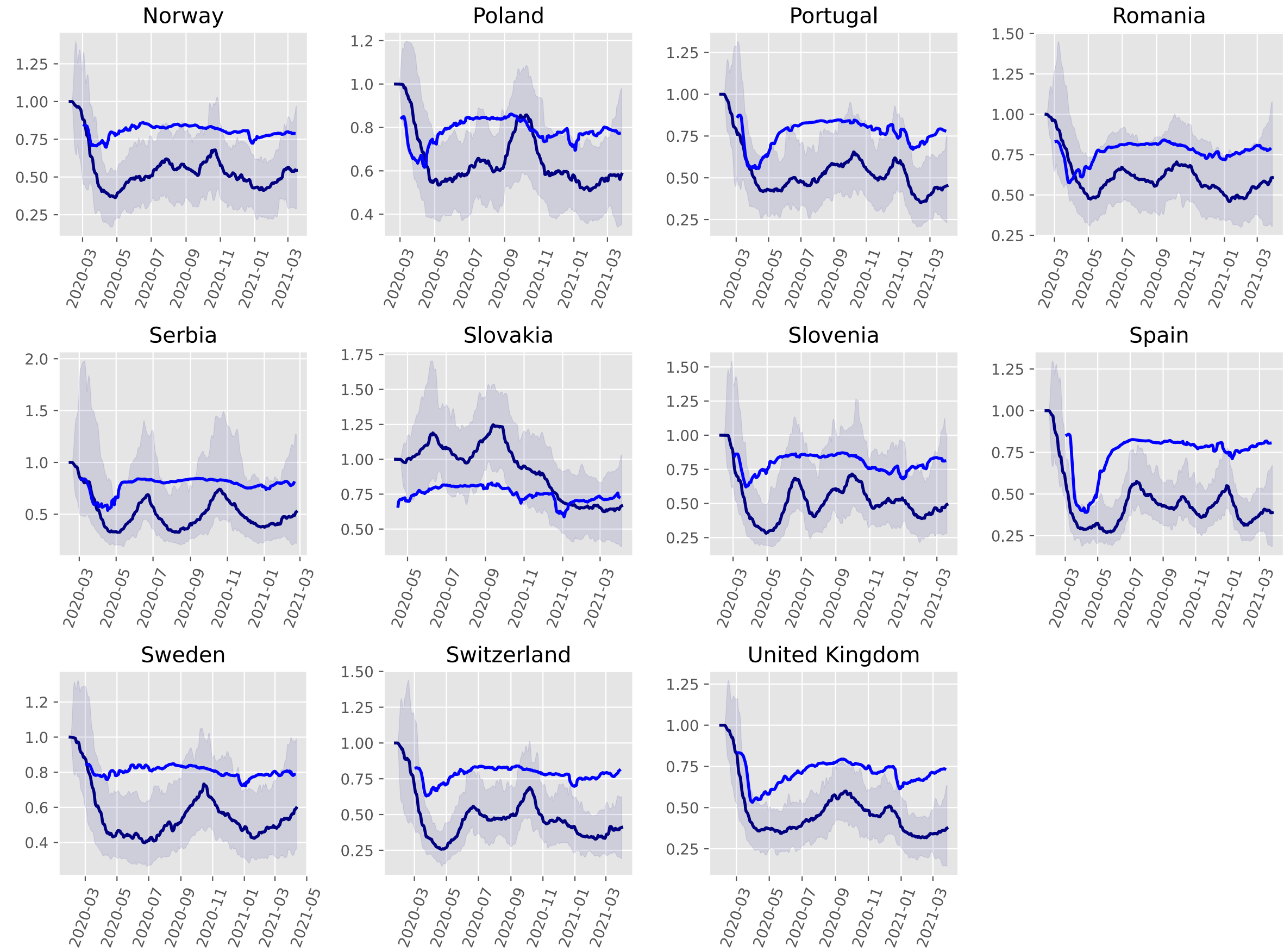

North America

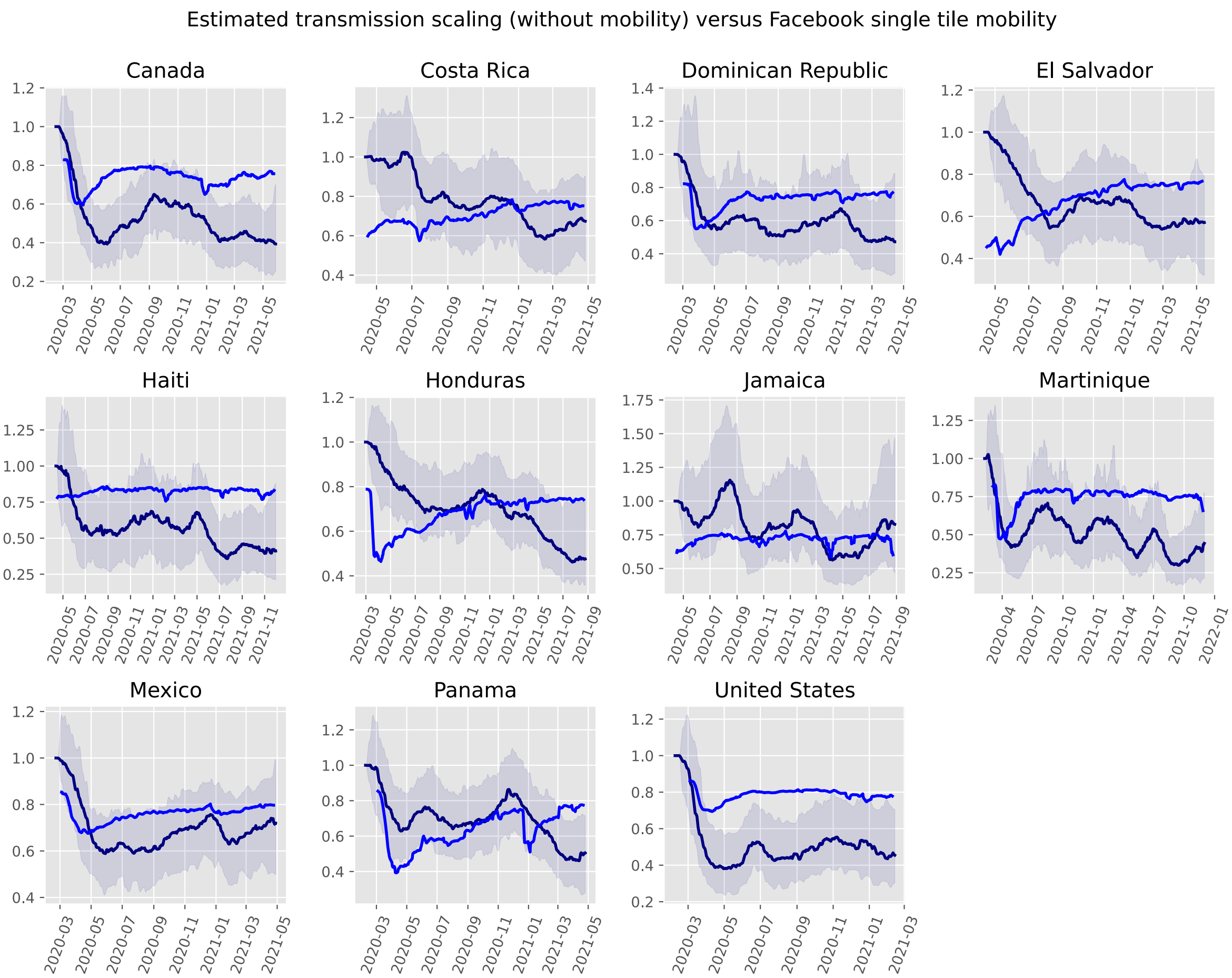

Oceania

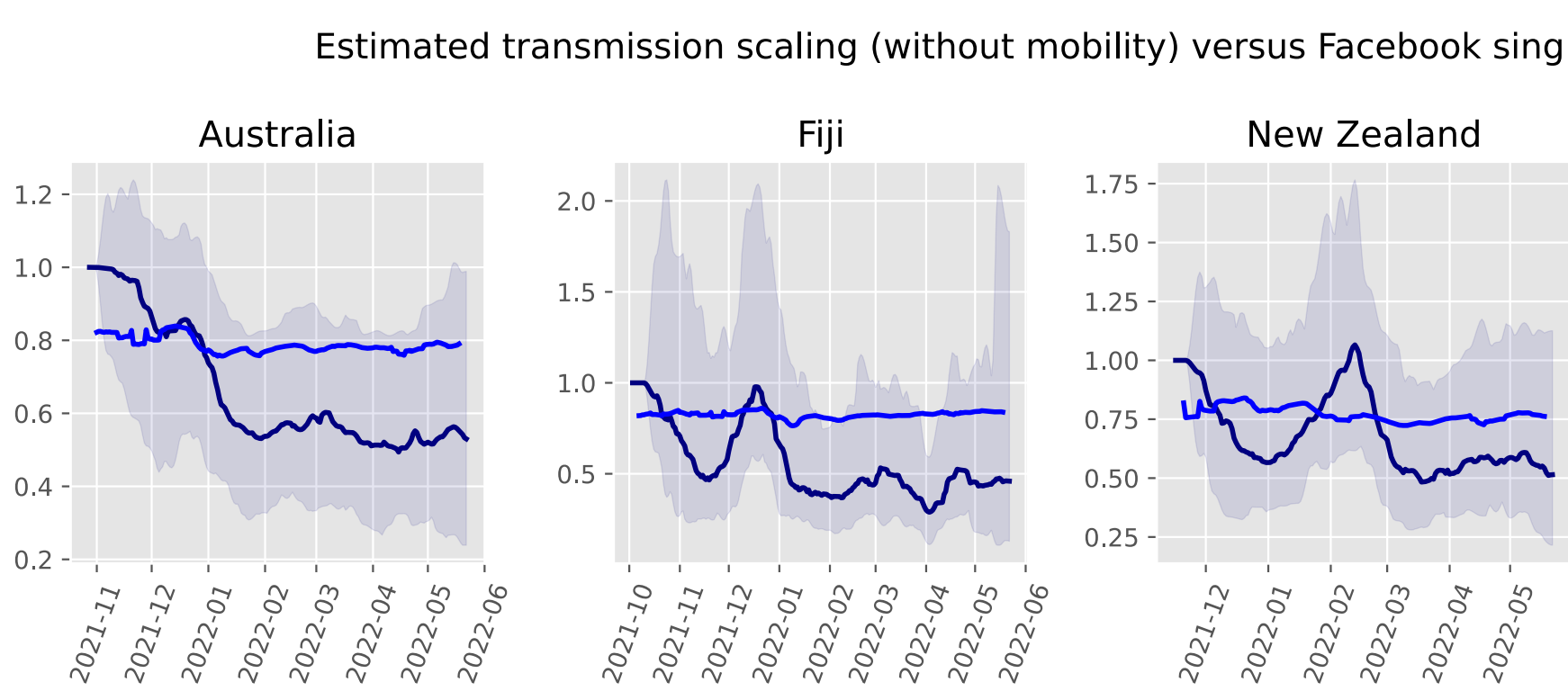

South America

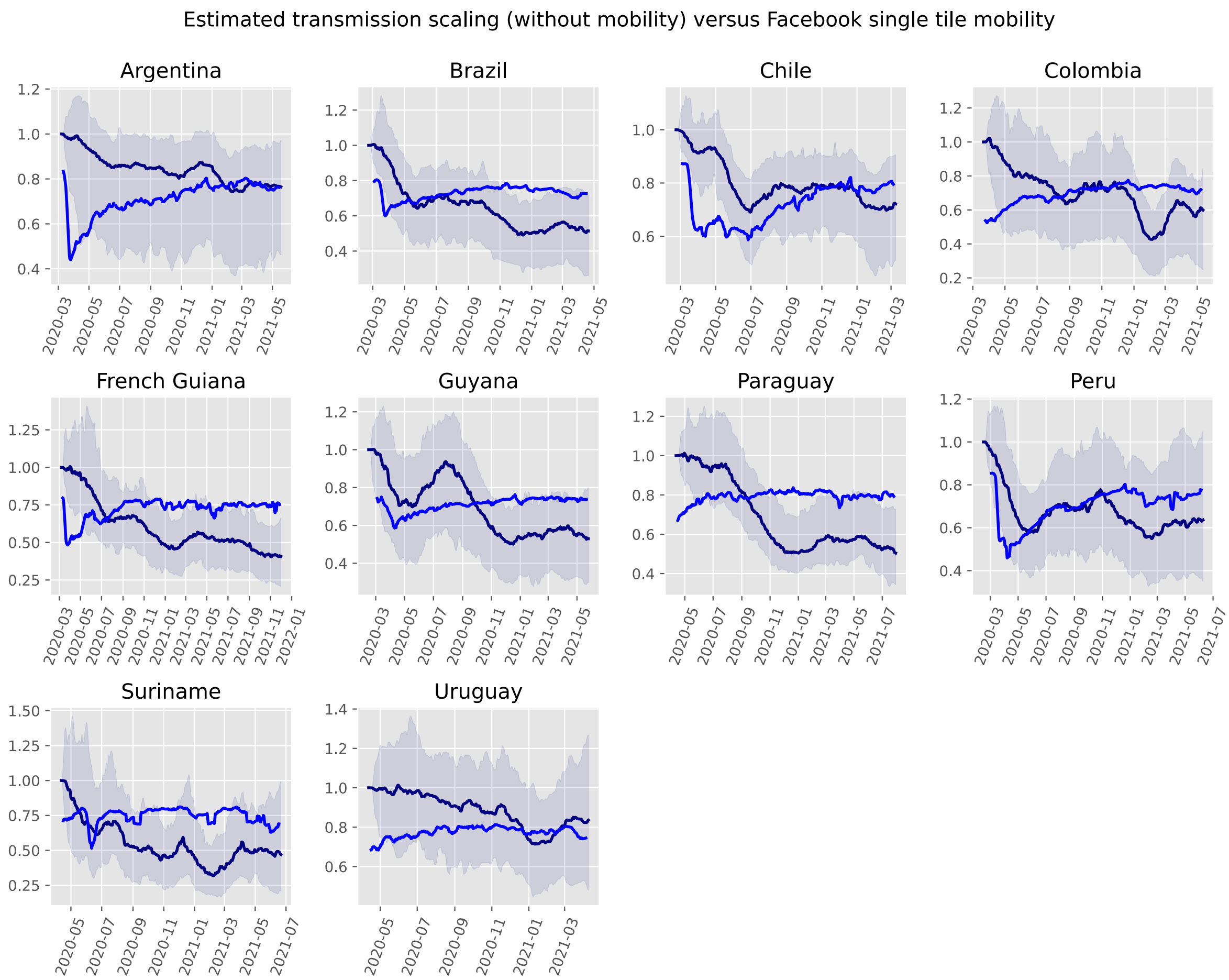
Estimated transmission scaling (without mobility) versus Facebook single tile mobility
Argentina
Brazil
Chile
Colombia
French Guiana
Guyana
Paraguay
Peru
Suriname
Uruguay

Weighted scaling process versus residual comparison

Africa

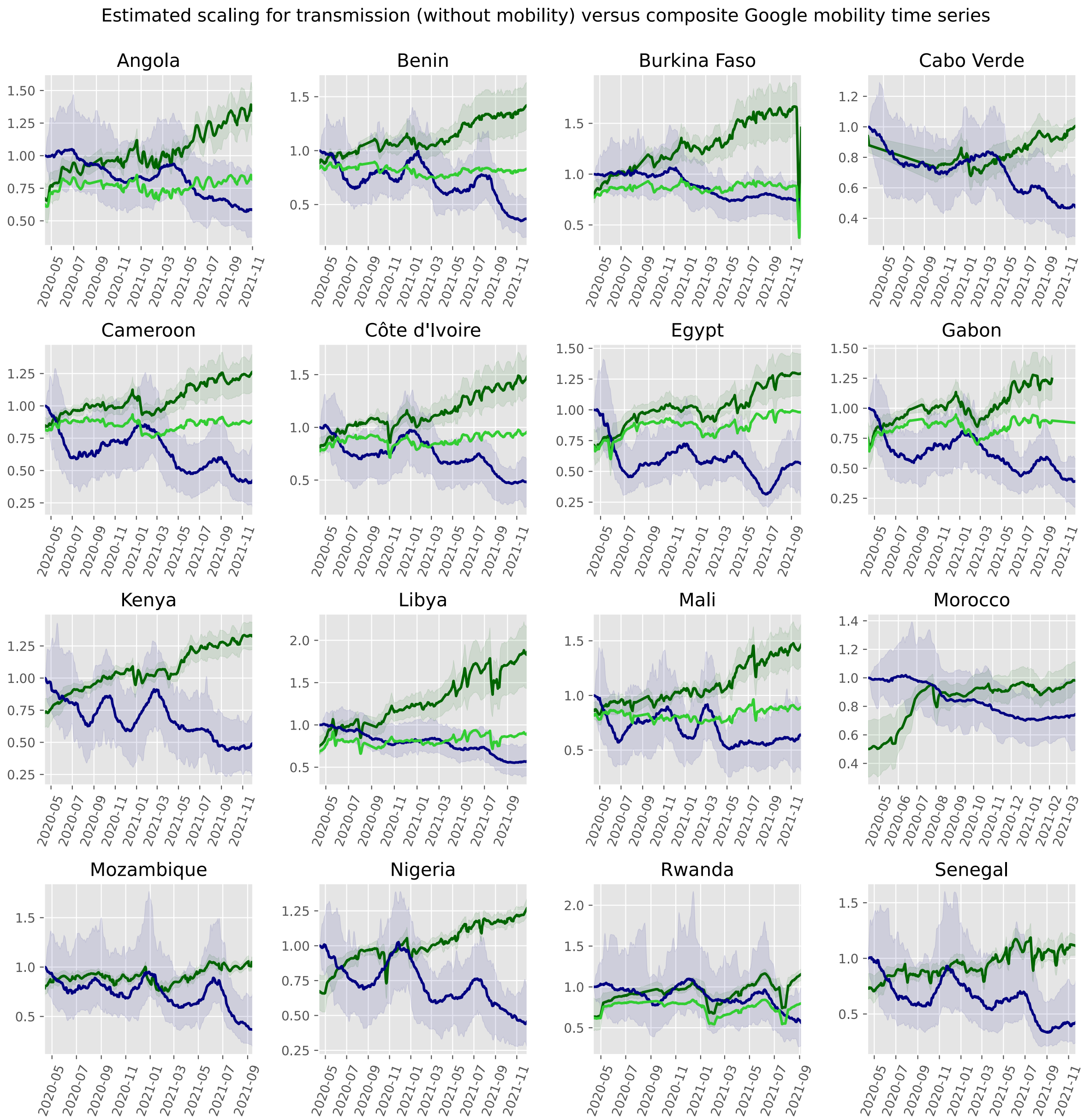

Estimated scaling for transmission (without mobility) versus composite Google mobility time series

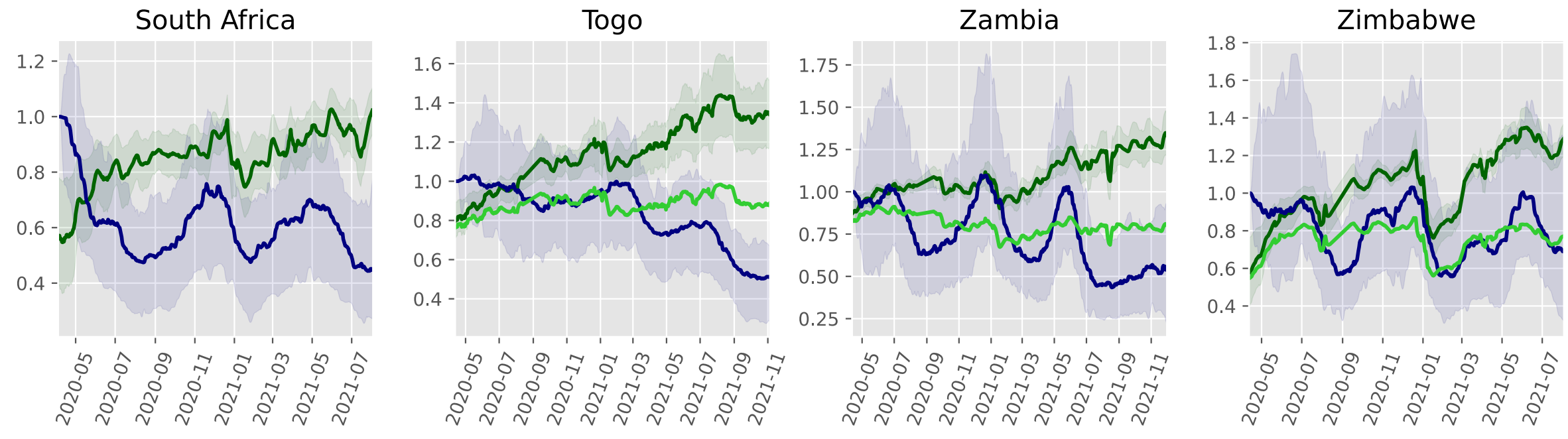

Asia

Estimated scaling for transmission (without mobility) versus composite Google mobility time series

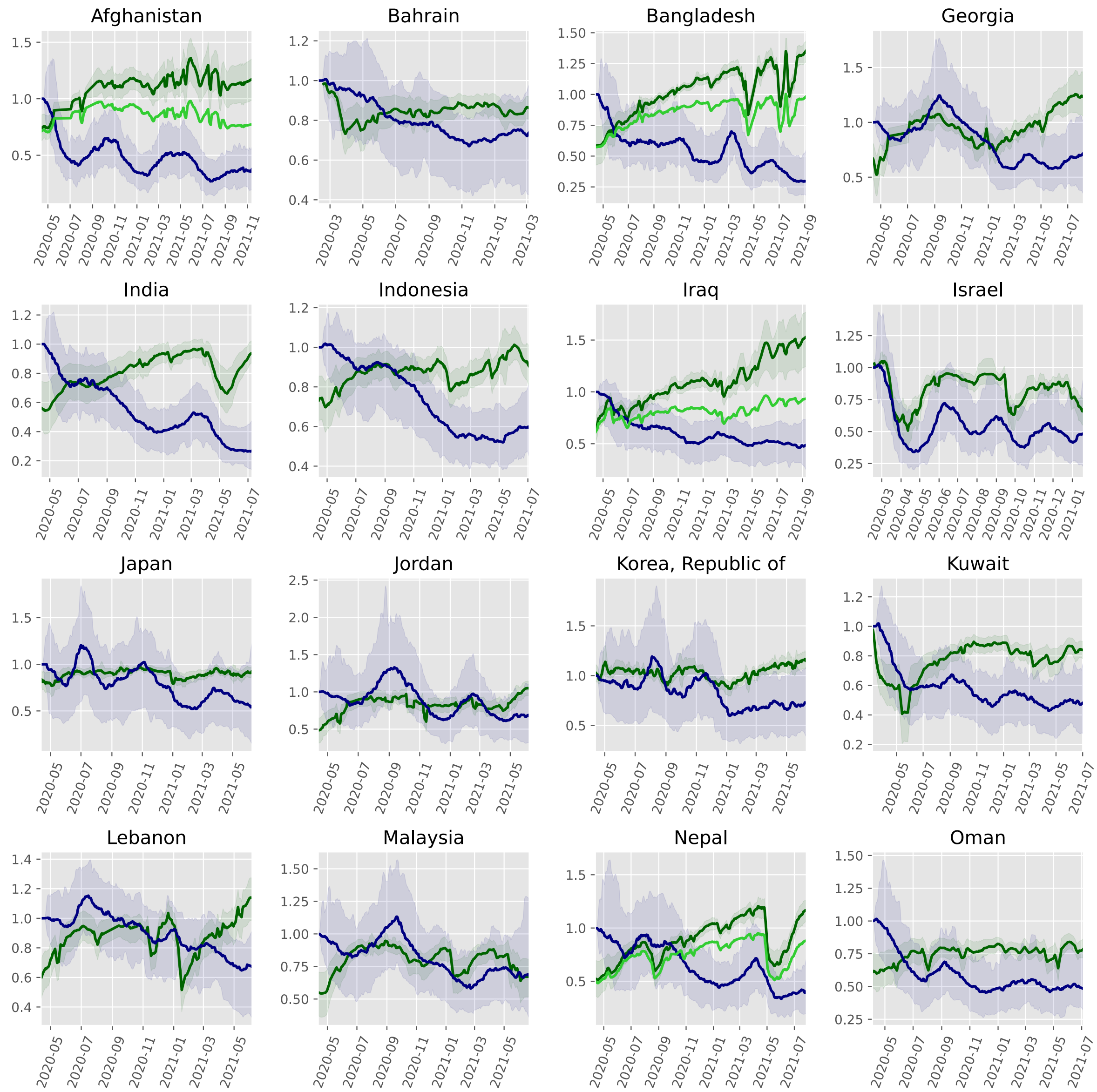

Estimated scaling for transmission (without mobility) versus composite Google mobility time series

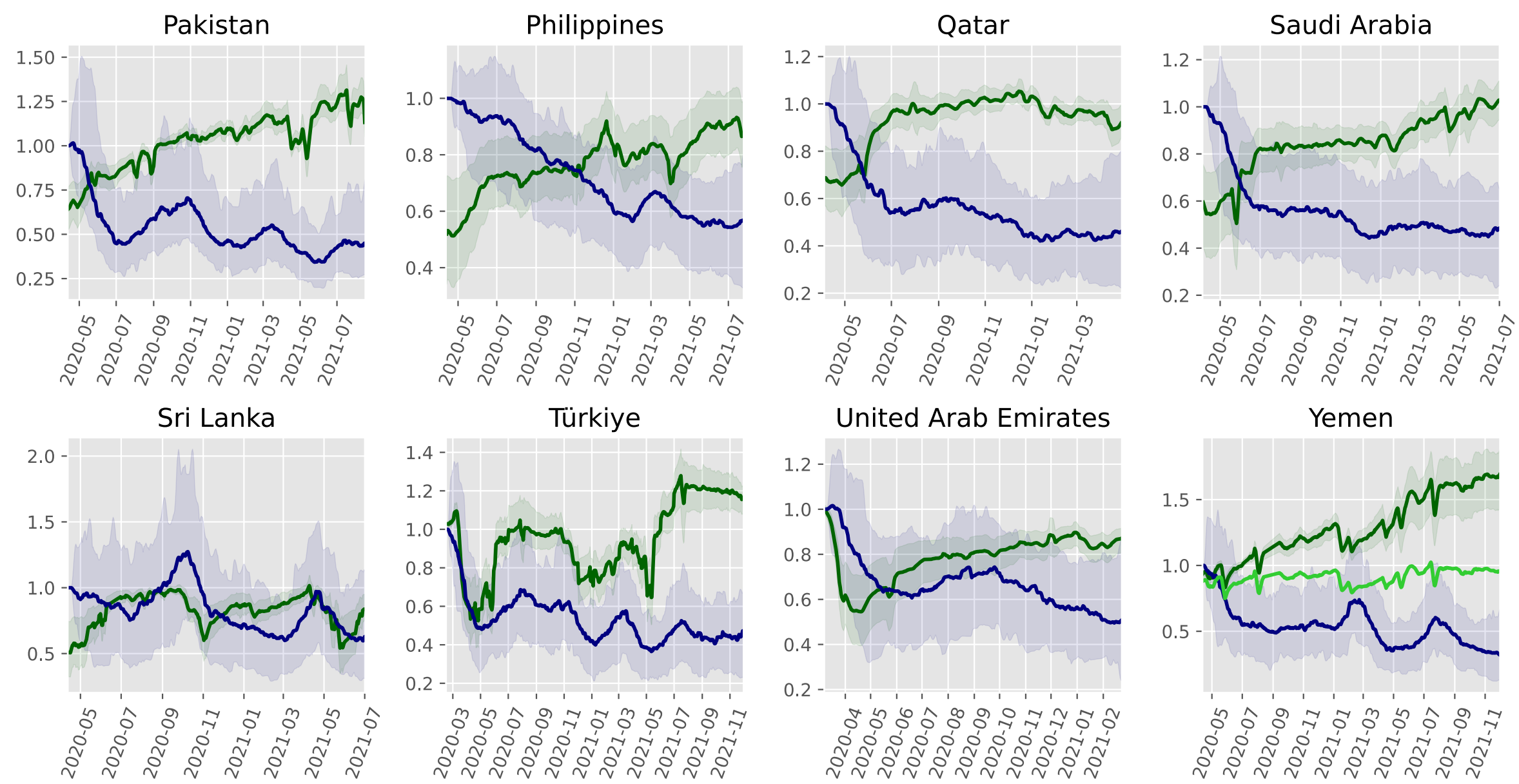

Europe

Estimated scaling for transmission (without mobility) versus composite Google mobility time series

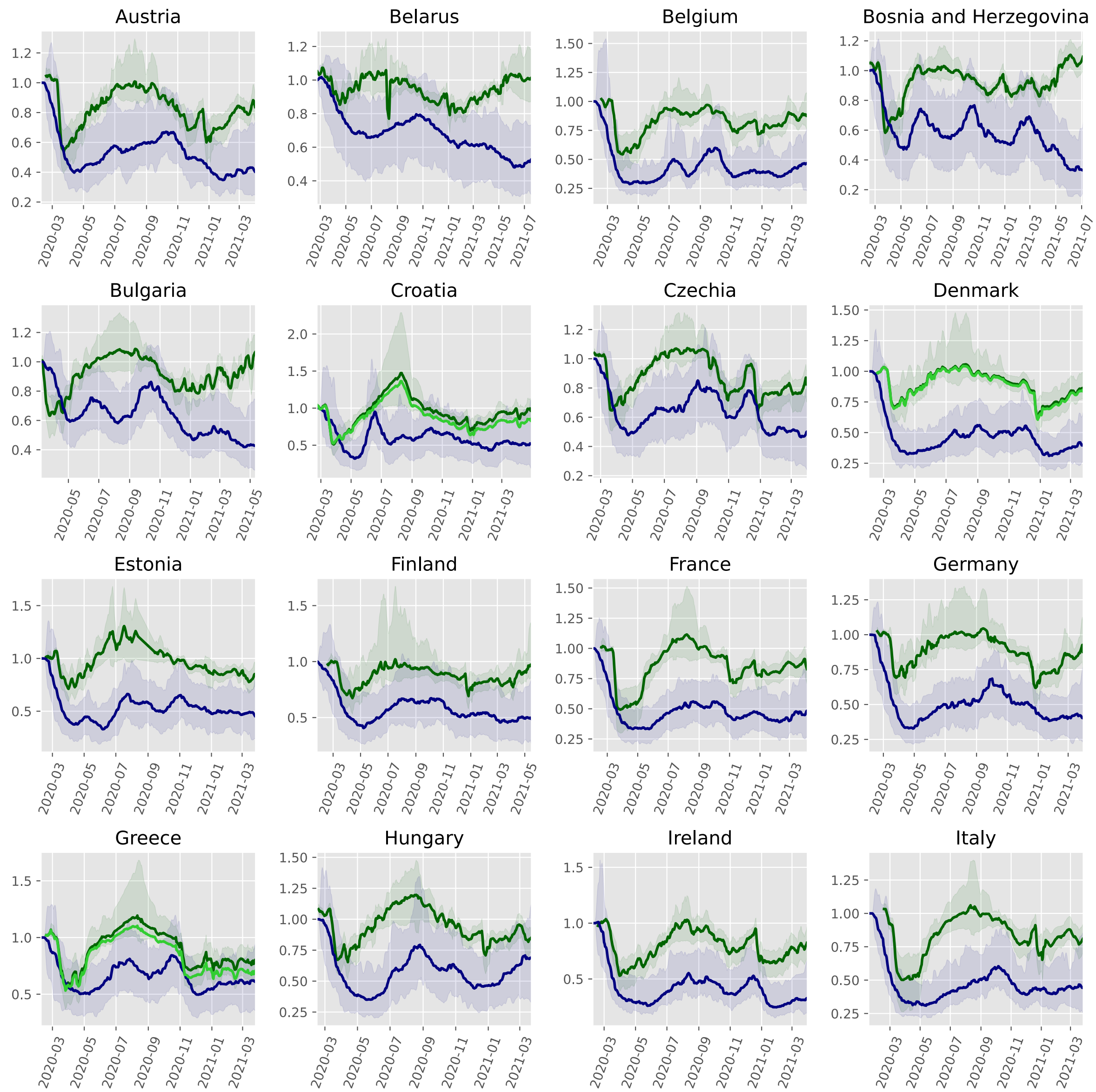

Estimated scaling for transmission (without mobility) versus composite Google mobility time series

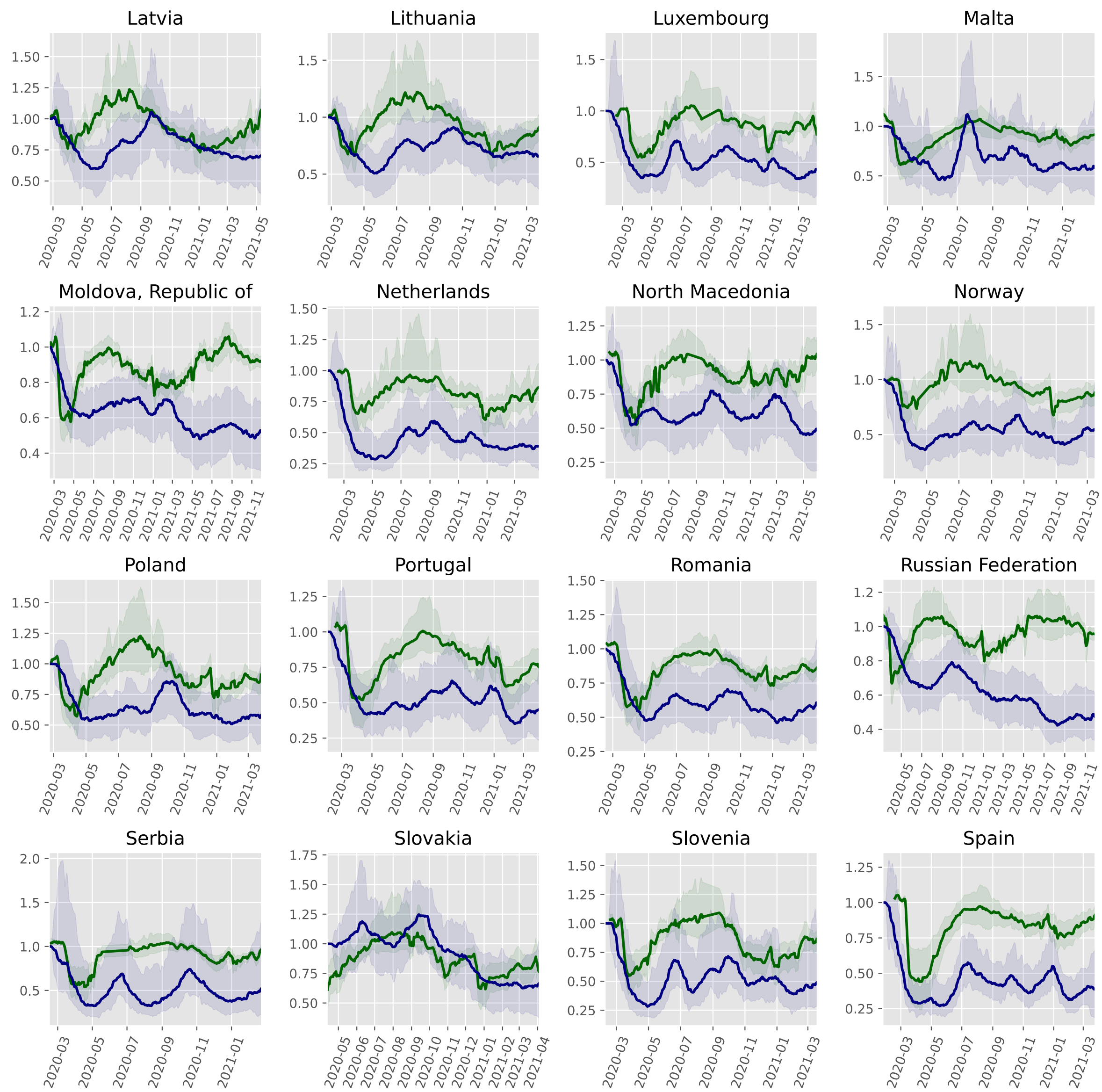

Estimated scaling for transmission (without mobility) versus composite Google mobility time series

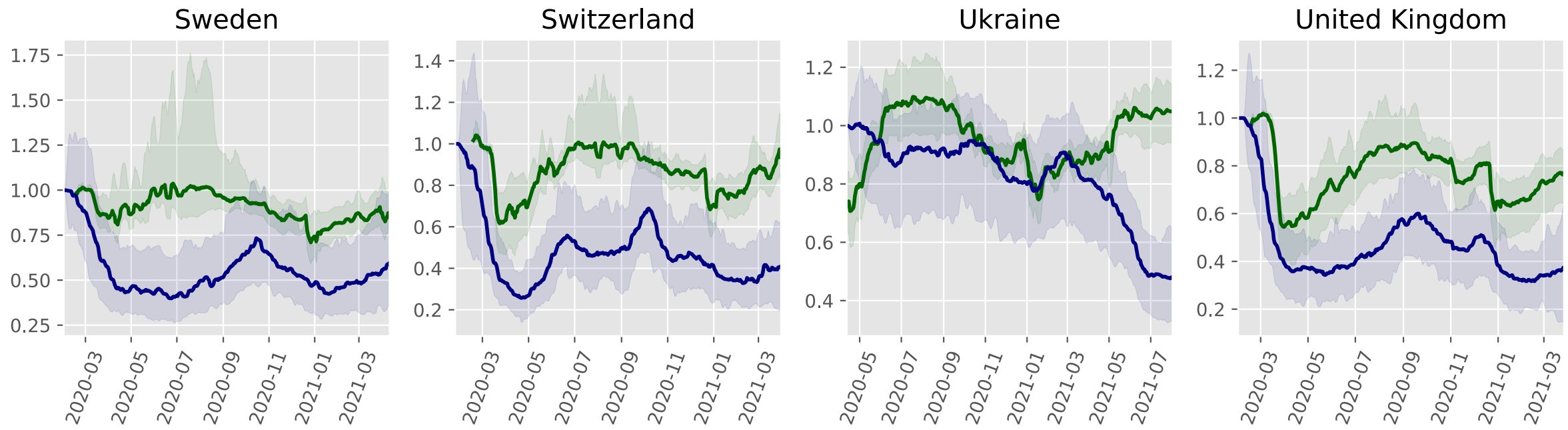

North America

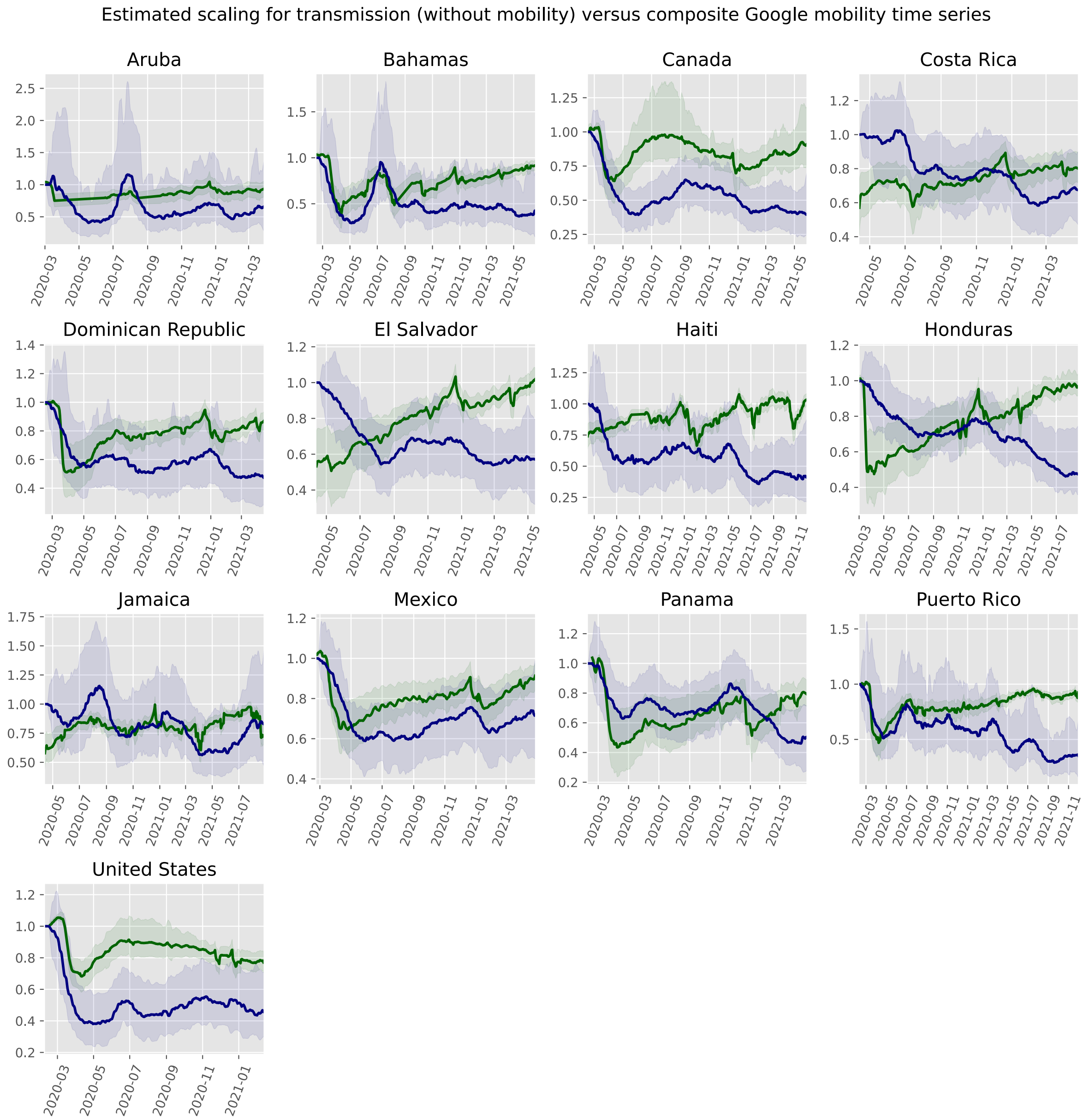

Oceania

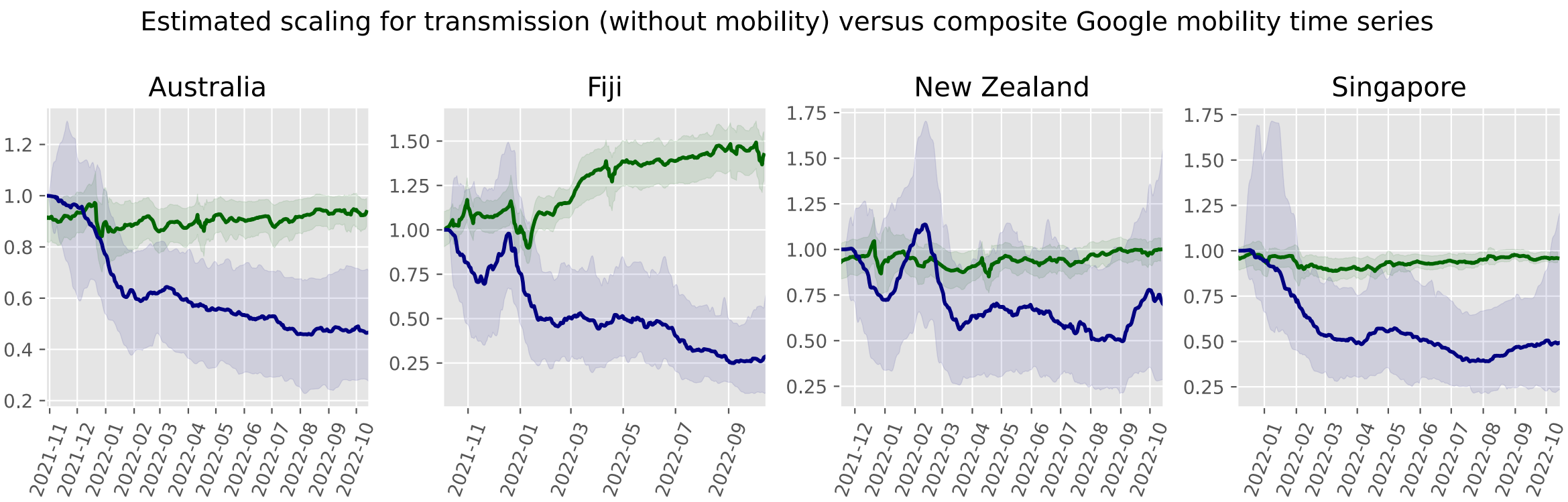

South America

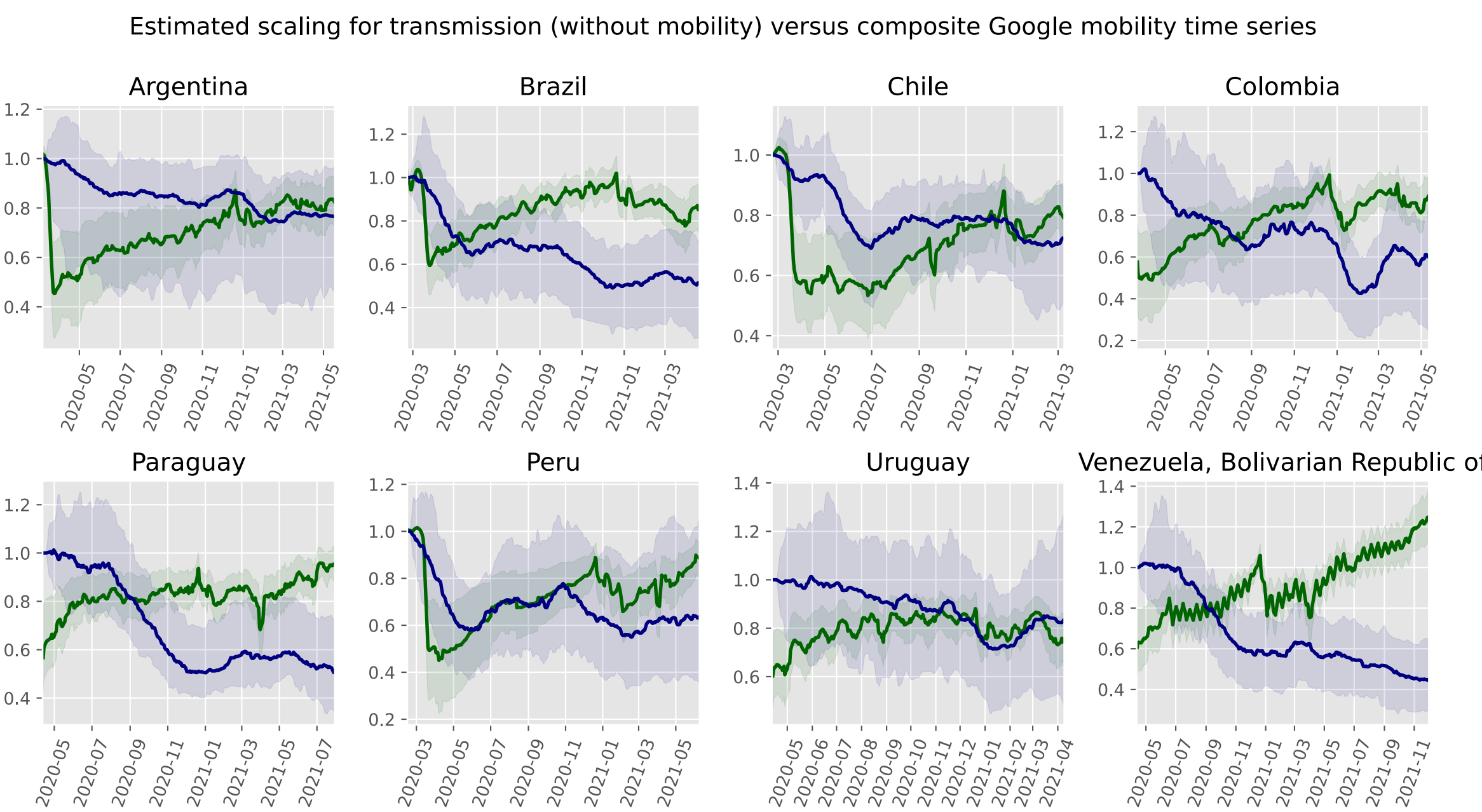

Comparisons of the non-mechanistic scaling of transmission process under each mobility configuration

This section presents comparisons of residual transmission scaling across each mobility analysis approach for each country, along with the 95% credible interval associated with each. We would expect that approaches to analysis that if applying mobility captured some part of the

variation in transmission that would otherwise need to be included through residual variation, then that mobility approach would be associated with less dramatic changes in the residual transmission scaling.

Results by continent

Africa

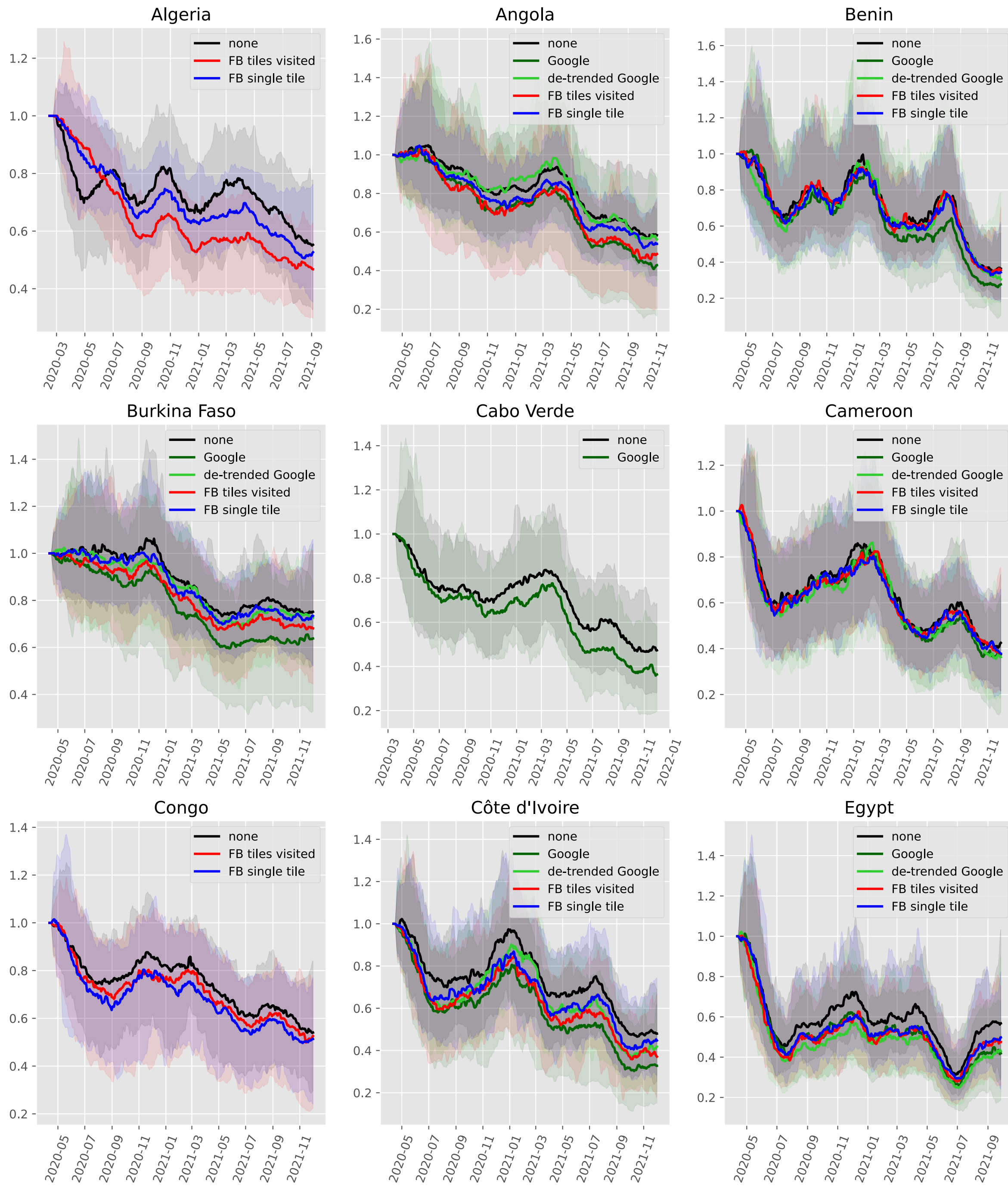

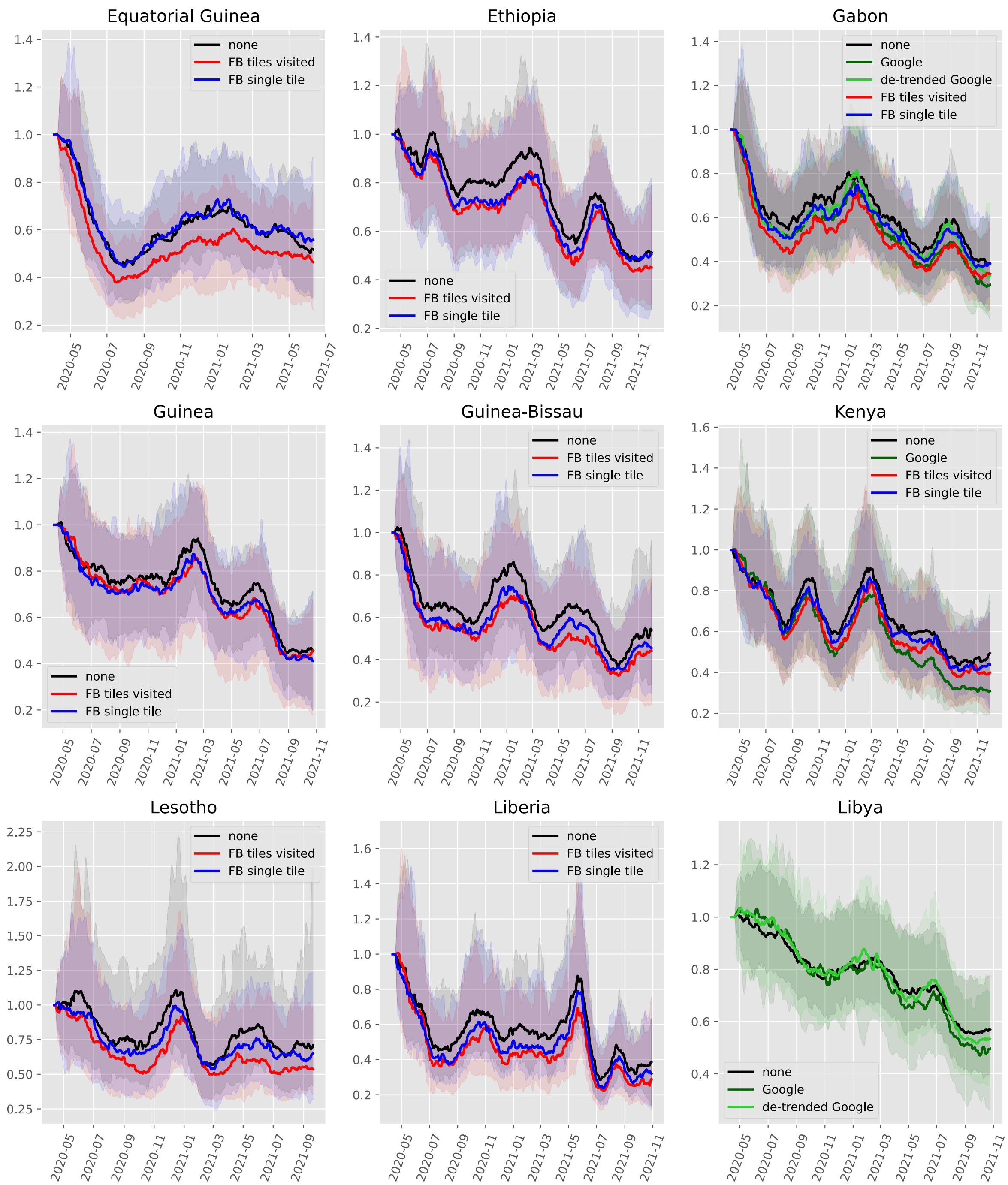

Equatorial Guinea
none
FB tiles visited
FB single tile
Ethiopia
none
FB tiles visited
FB single tile
Gabon
none
Google
de-trended Google
FB tiles visited
FB single tile
Guinea
none
FB tiles visited
FB single tile
Guinea-Bissau
none
FB tiles visited
FB single tile
Kenya
none
Google
FB tiles visited
FB single tile
Lesotho
none
FB tiles visited
FB single tile
Liberia
none
FB tiles visited
FB single tile
Libya
none
Google
de-trended Google

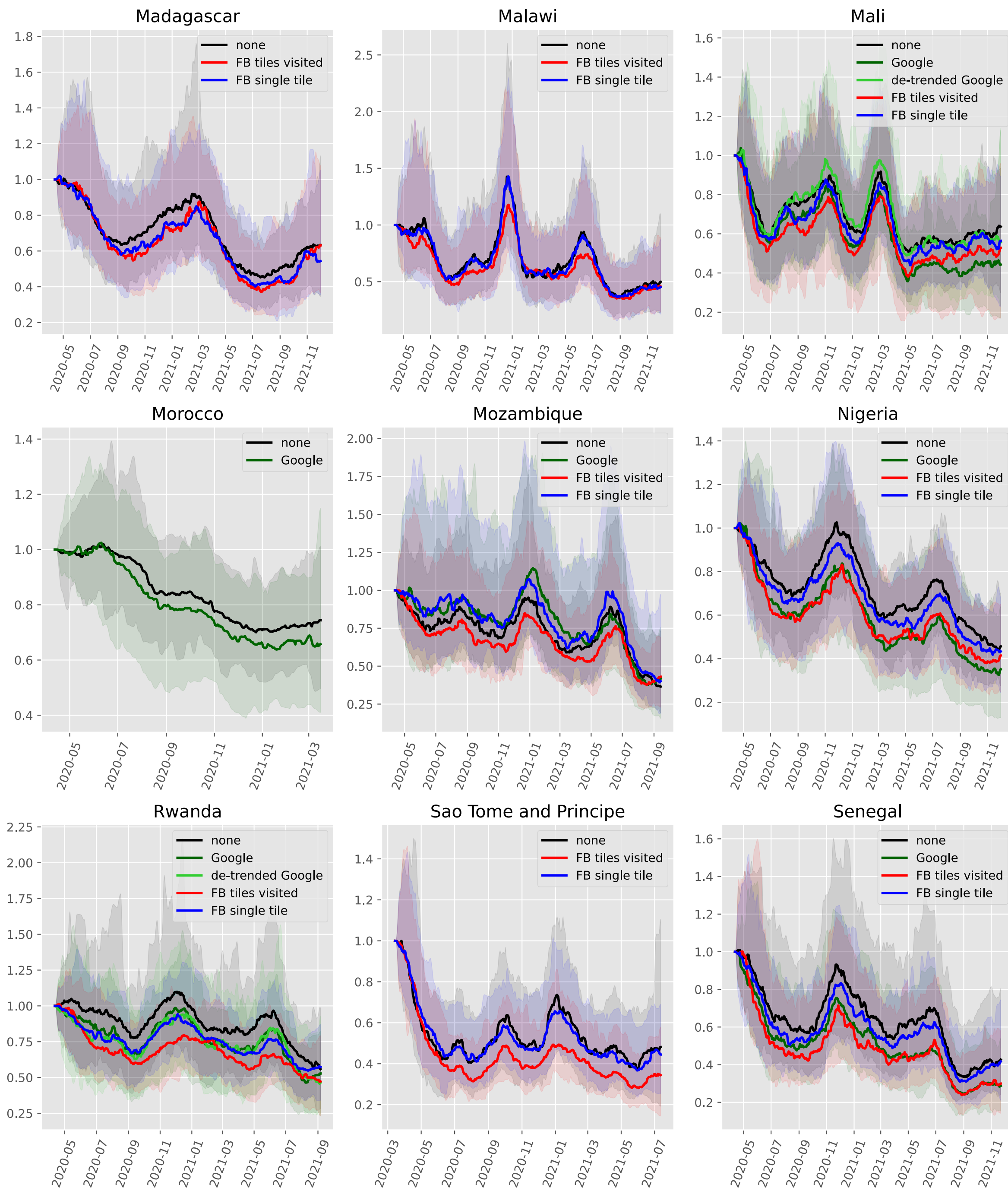
Madagascar
none
FB tiles visited
FB single tile
Malawi
none
FB tiles visited
FB single tile
Mali
none
Google
de-trended Google
FB tiles visited
FB single tile
Morocco
none
Google
Mozambique
none
Google
FB tiles visited
FB single tile
Nigeria
none
Google
FB tiles visited
FB single tile
Rwanda
none
Google
de-trended Google
FB tiles visited
FB single tile
Sao Tome and Principe
none
FB tiles visited
FB single tile
Senegal
none
Google
FB tiles visited
FB single tile

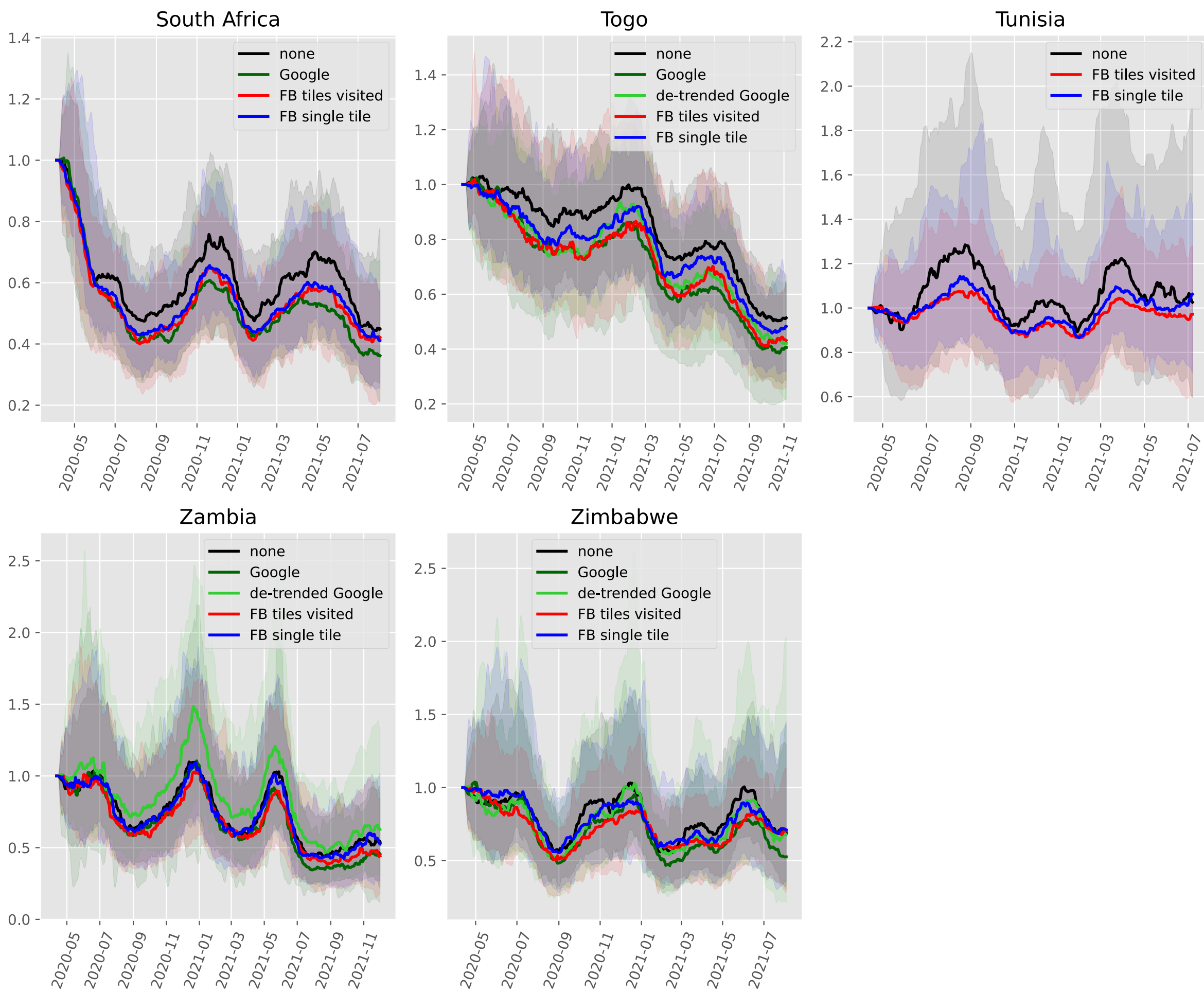
South Africa
none
Google
FB tiles visited
FB single tile
Togo
none
Google
de-trended Google
FB tiles visited
FB single tile
Tunisia
none
FB tiles visited
FB single tile
Zambia
none
Google
de-trended Google
FB tiles visited
FB single tile
Zimbabwe
none
Google
de-trended Google
FB tiles visited
FB single tile

Asia

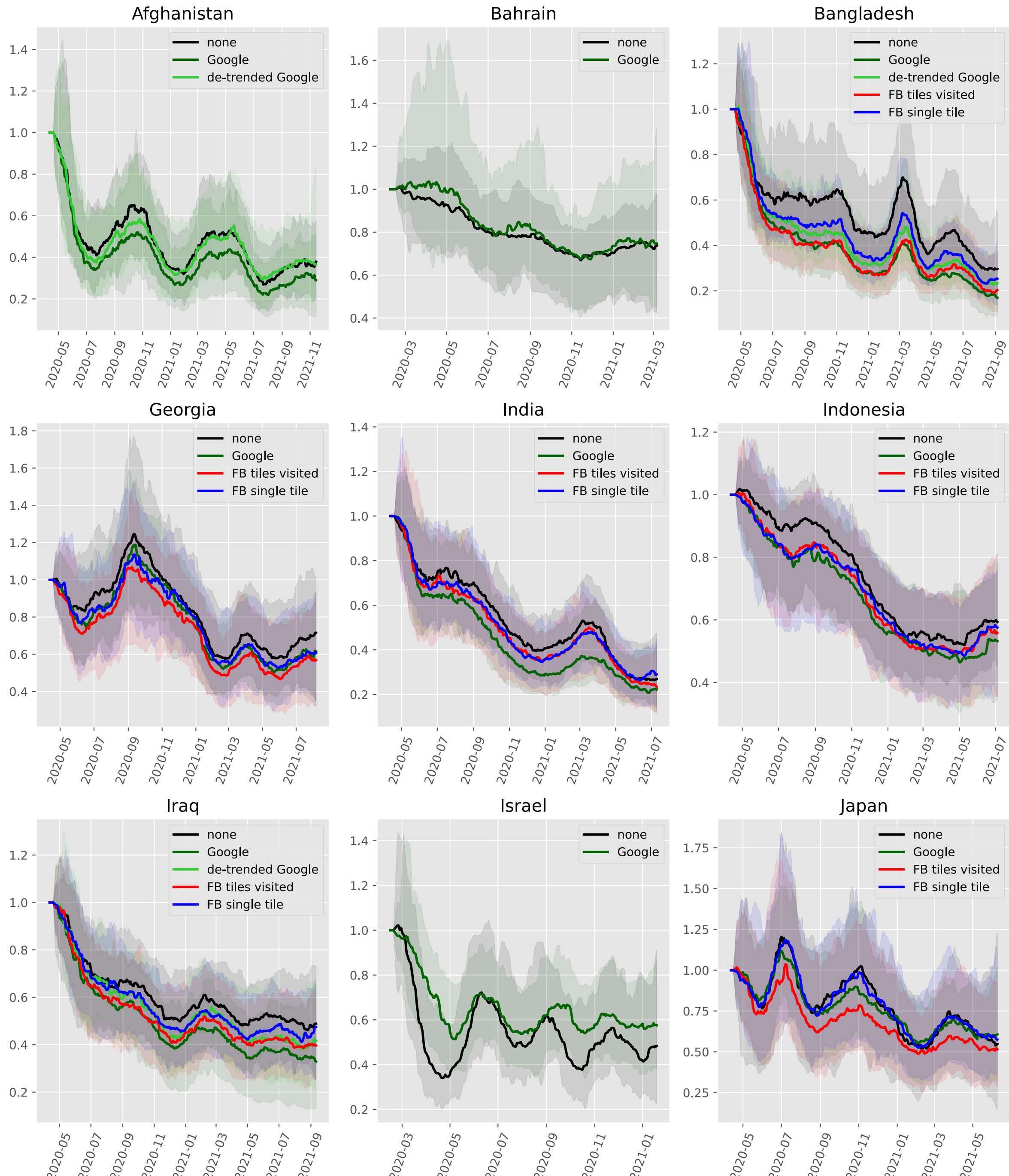
Afghanistan
none
Google
de-trended Google
0.2 0.4 0.6 0.8 1.0 1.2 1.4
2020-05 2020-07 2020-09 2020-11 2021-01 2021-03 2021-05 2021-07 2021-09 2021-11
Bahrain
none
Google
0.4 0.6 0.8 1.0 1.2 1.4 1.6
2020-03 2020-05 2020-07 2020-09 2020-11 2021-01 2021-03
Bangladesh
none
Google
de-trended Google
FB tiles visited
FB single tile
0.2 0.4 0.6 0.8 1.0 1.2
2020-05 2020-07 2020-09 2020-11 2021-01 2021-03 2021-05 2021-07 2021-09
Georgia
none
Google
FB tiles visited
FB single tile
0.4 0.6 0.8 1.0 1.2 1.4 1.6 1.8
2020-05 2020-07 2020-09 2020-11 2021-01 2021-03 2021-05 2021-07
India
none
Google
FB tiles visited
FB single tile
0.2 0.4 0.6 0.8 1.0 1.2 1.4
2020-05 2020-07 2020-09 2020-11 2021-01 2021-03 2021-05 2021-07
Indonesia
none
Google
FB tiles visited
FB single tile
0.4 0.6 0.8 1.0 1.2
2020-05 2020-07 2020-09 2020-11 2021-01 2021-03 2021-05 2021-07
Iraq
none
Google
de-trended Google
FB tiles visited
FB single tile
0.2 0.4 0.6 0.8 1.0 1.2
2020-05 2020-07 2020-09 2020-11 2021-01 2021-03 2021-05 2021-07 2021-09
Israel
none
Google
0.2 0.4 0.6 0.8 1.0 1.2 1.4
2020-03 2020-05 2020-07 2020-09 2020-11 2021-01
Japan
none
Google
FB tiles visited
FB single tile
0.25 0.50 0.75 1.00 1.25 1.50 1.75
2020-05 2020-07 2020-09 2020-11 2021-01 2021-03 2021-05

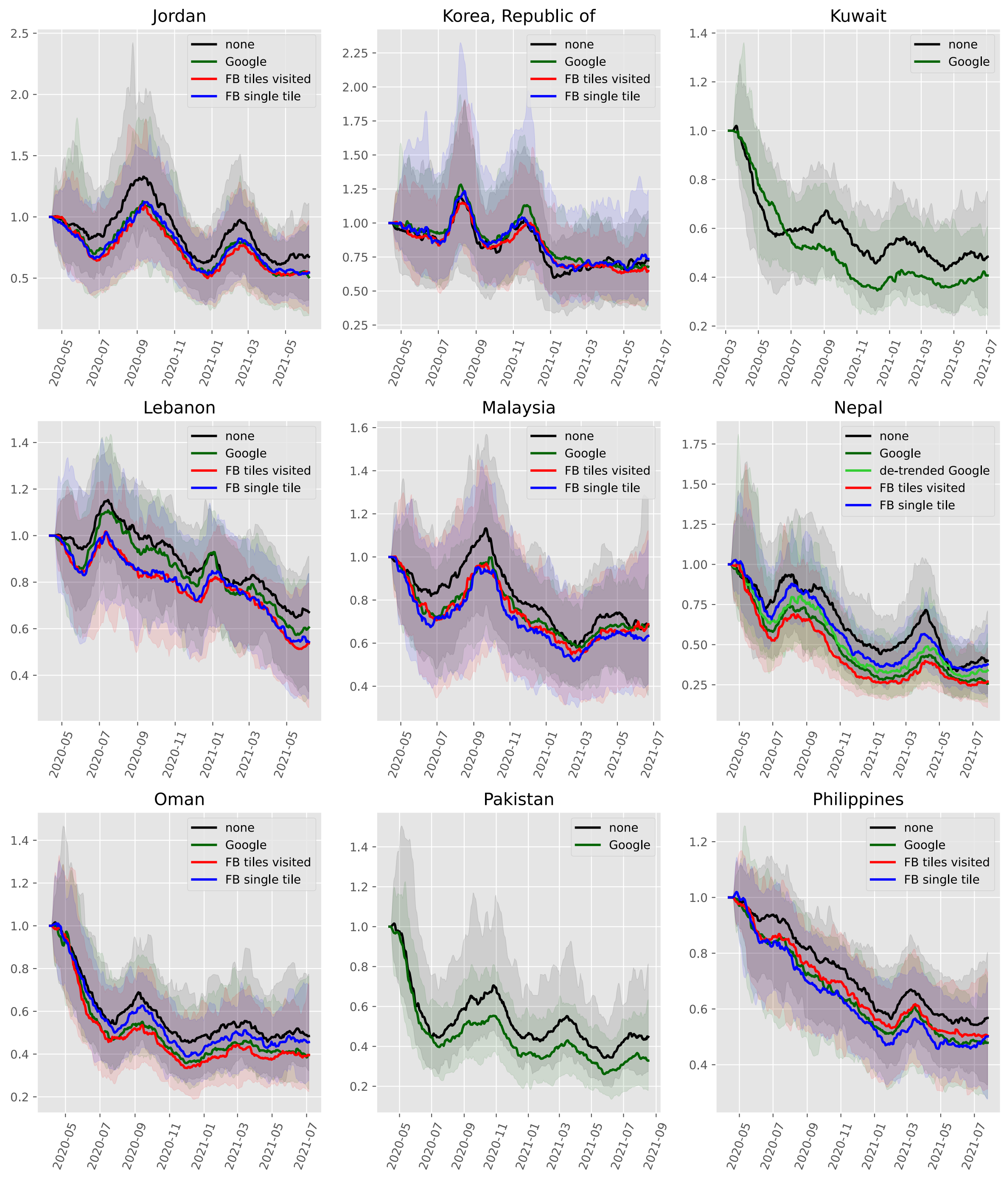
Jordan
none
Google
FB tiles visited
FB single tile
Korea, Republic of
none
Google
FB tiles visited
FB single tile
Kuwait
none
Google
Lebanon
none
Google
FB tiles visited
FB single tile
Malaysia
none
Google
FB tiles visited
FB single tile
Nepal
none
Google
de-trended Google
FB tiles visited
FB single tile
Oman
none
Google
FB tiles visited
FB single tile
Pakistan
none
Google
Philippines
none
Google
FB tiles visited
FB single tile

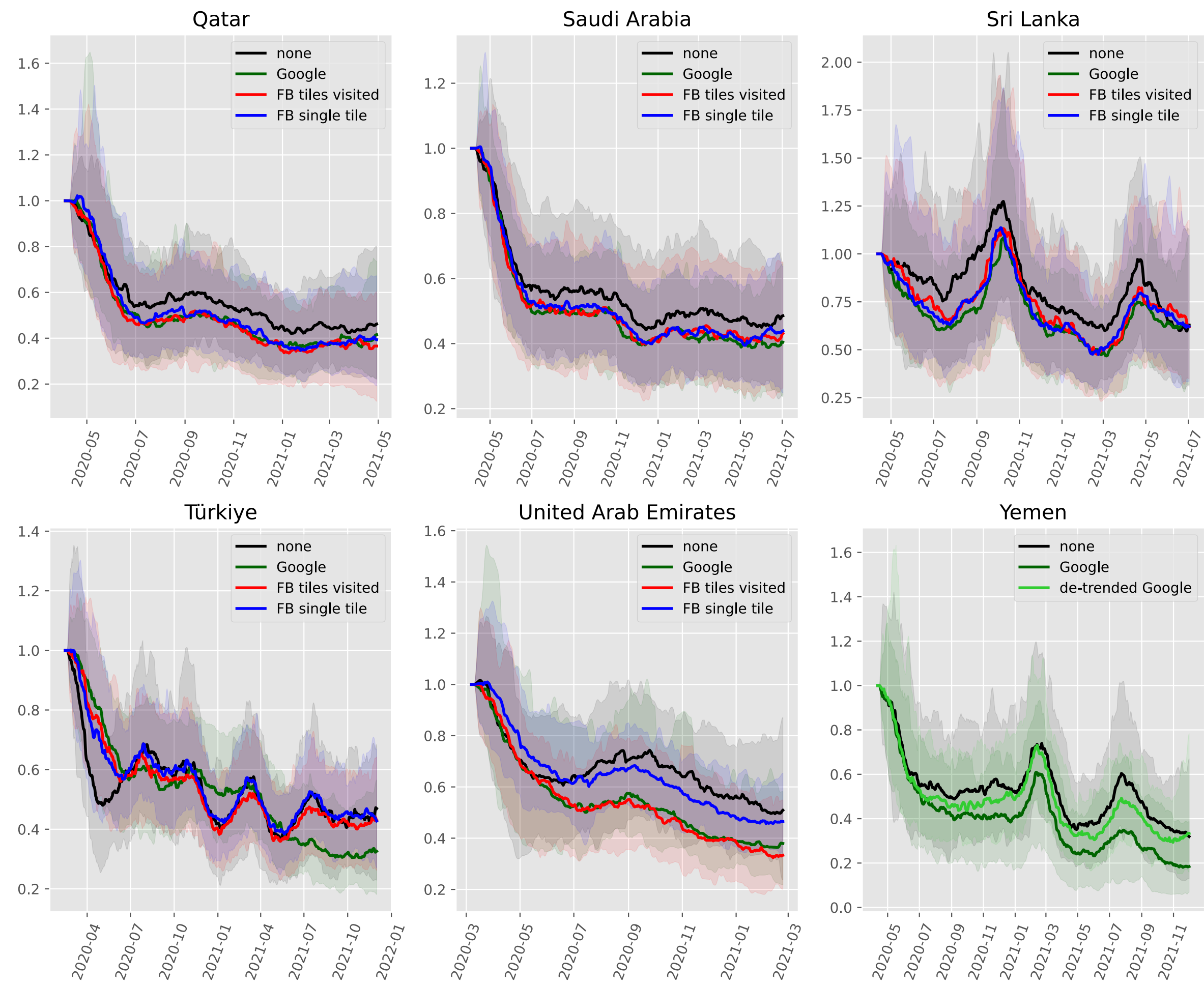
Qatar
Saudi Arabia
Sri Lanka
Türkiye
United Arab Emirates
Yemen
none
Google
FB tiles visited
FB single tile
de-trended Google

Europe

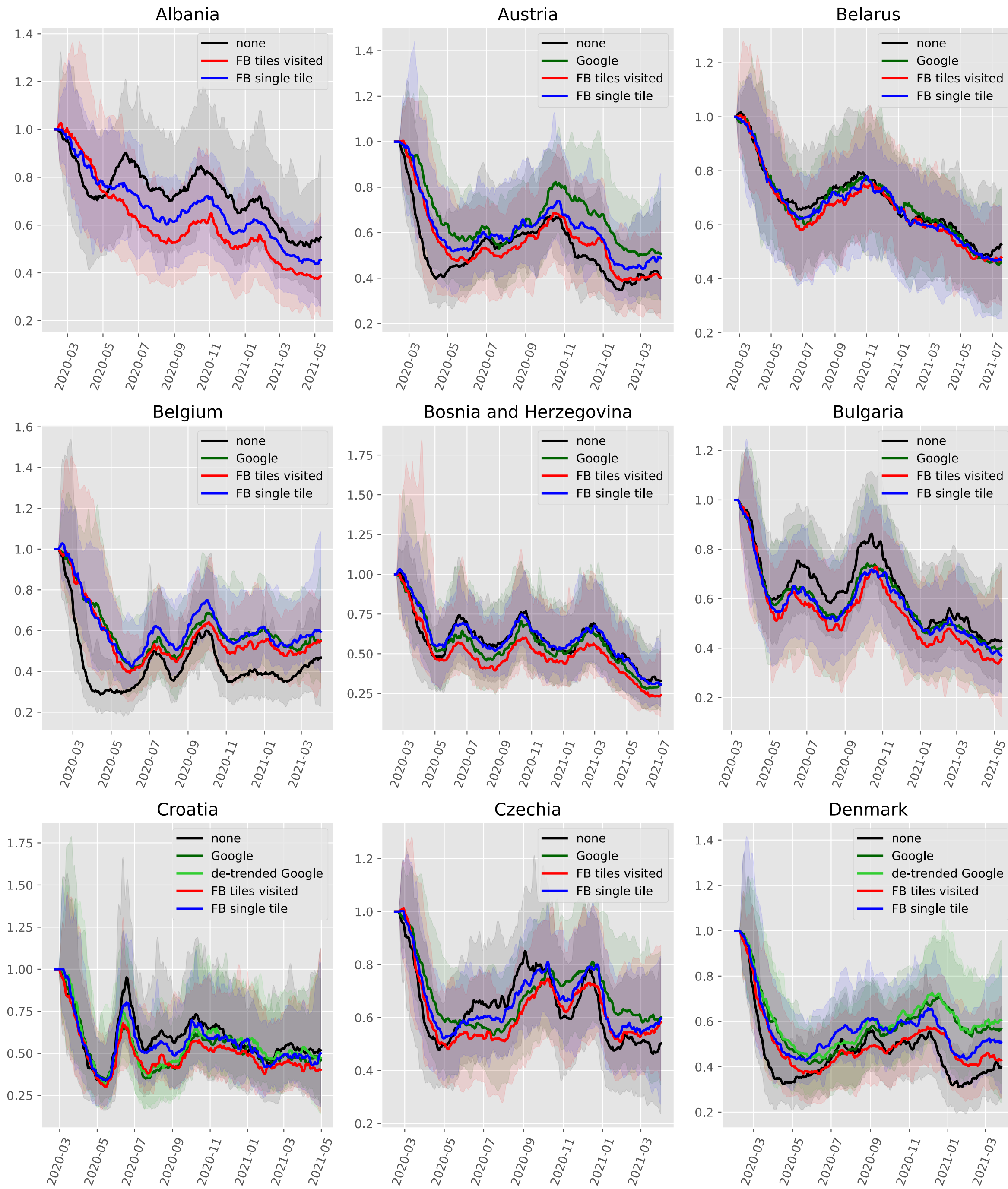

Albania
none
FB tiles visited
FB single tile
Austria
none
Google
FB tiles visited
FB single tile
Belarus
none
Google
FB tiles visited
FB single tile
Belgium
none
Google
FB tiles visited
FB single tile
Bosnia and Herzegovina
none
Google
FB tiles visited
FB single tile
Bulgaria
none
Google
FB tiles visited
FB single tile
Croatia
none
Google
de-trended Google
FB tiles visited
FB single tile
Czechia
none
Google
FB tiles visited
FB single tile
Denmark
none
Google
de-trended Google
FB tiles visited
FB single tile

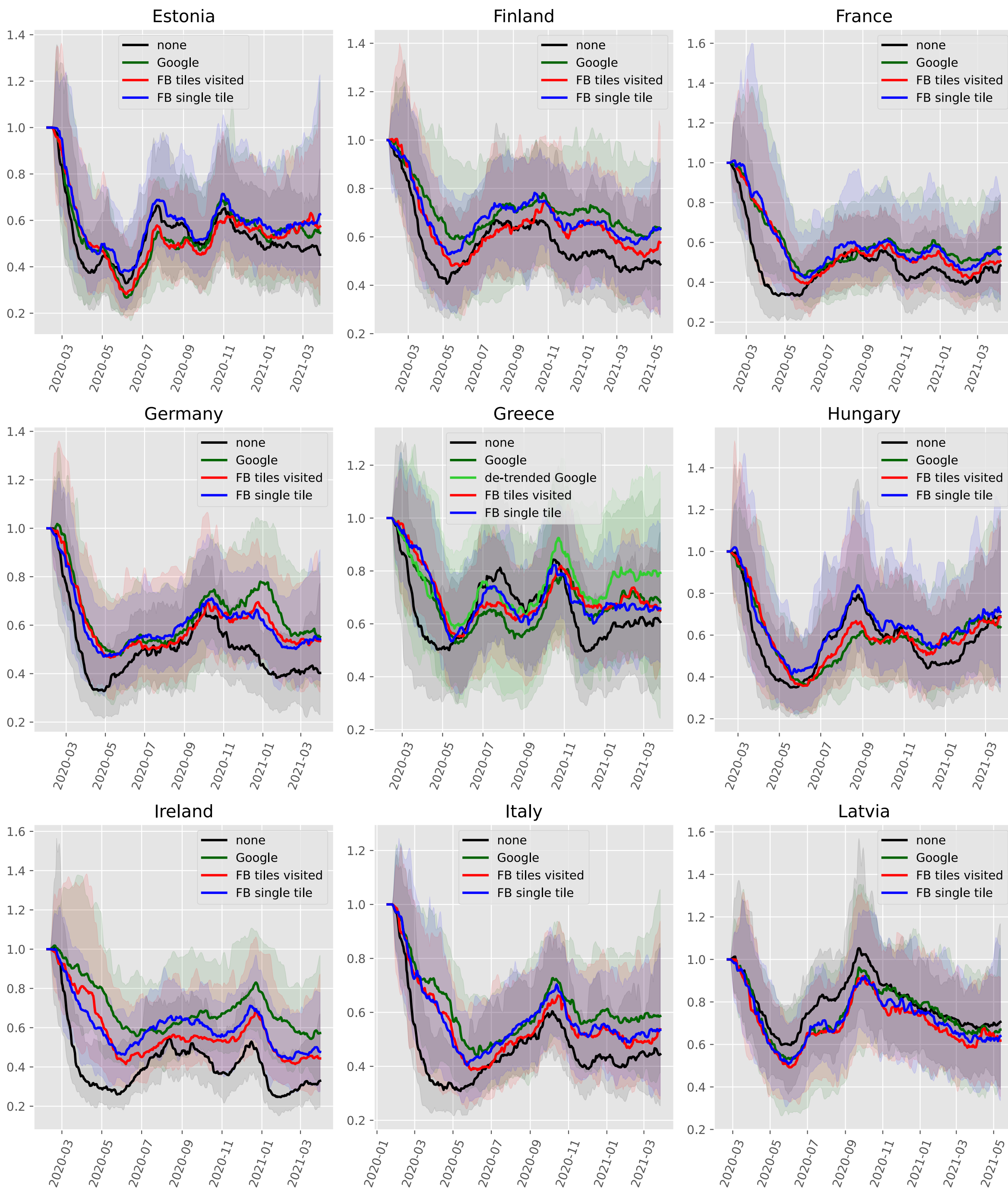
Estonia
none
Google
FB tiles visited
FB single tile
Finland
none
Google
FB tiles visited
FB single tile
France
none
Google
FB tiles visited
FB single tile
Germany
none
Google
FB tiles visited
FB single tile
Greece
none
Google
de-trended Google
FB tiles visited
FB single tile
Hungary
none
Google
FB tiles visited
FB single tile
Ireland
none
Google
FB tiles visited
FB single tile
Italy
none
Google
FB tiles visited
FB single tile
Latvia
none
Google
FB tiles visited
FB single tile

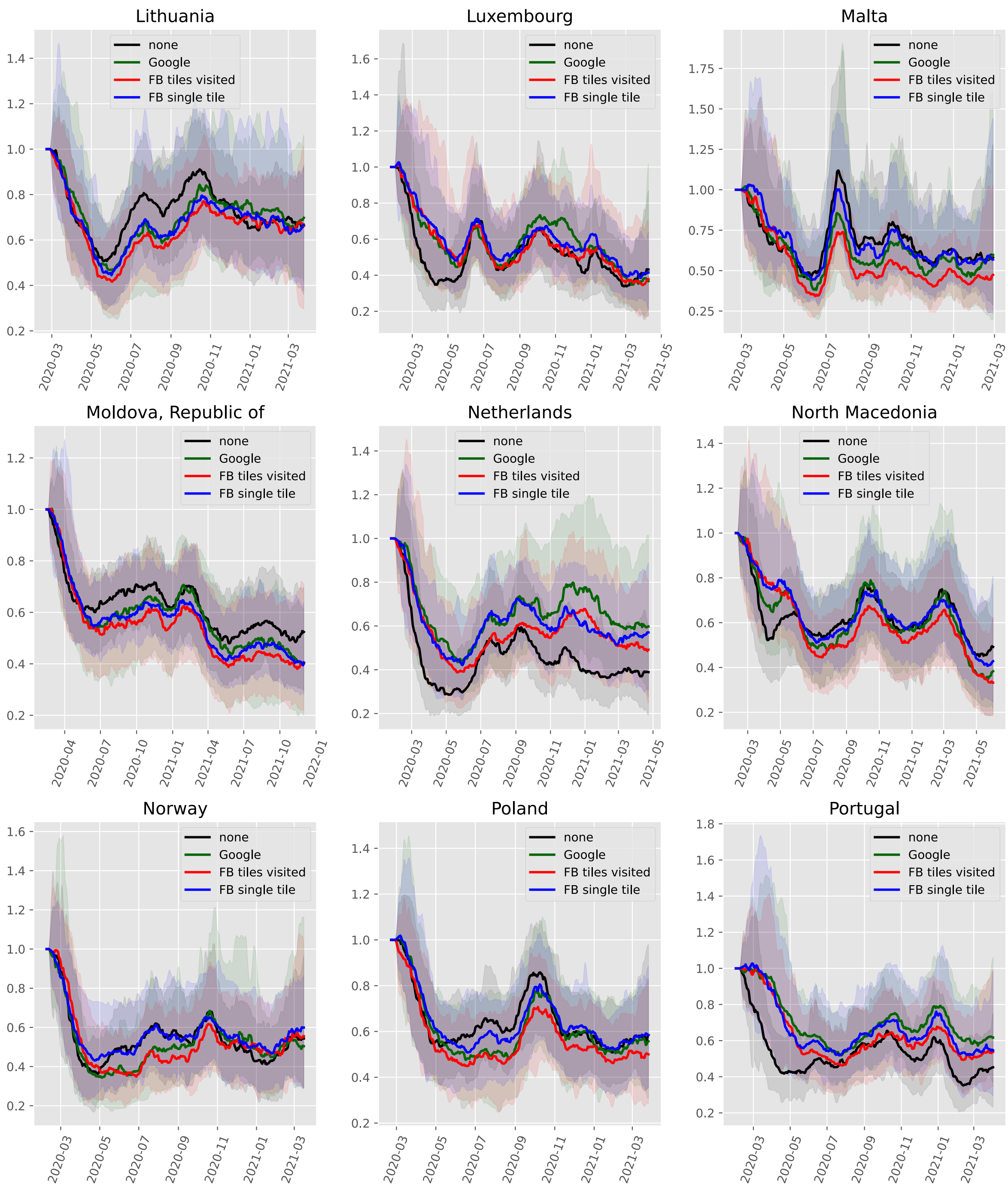
Lithuania
Luxembourg
Malta
Moldova, Republic of
Netherlands
North Macedonia
Norway
Poland
Portugal
none
Google
FB tiles visited
FB single tile

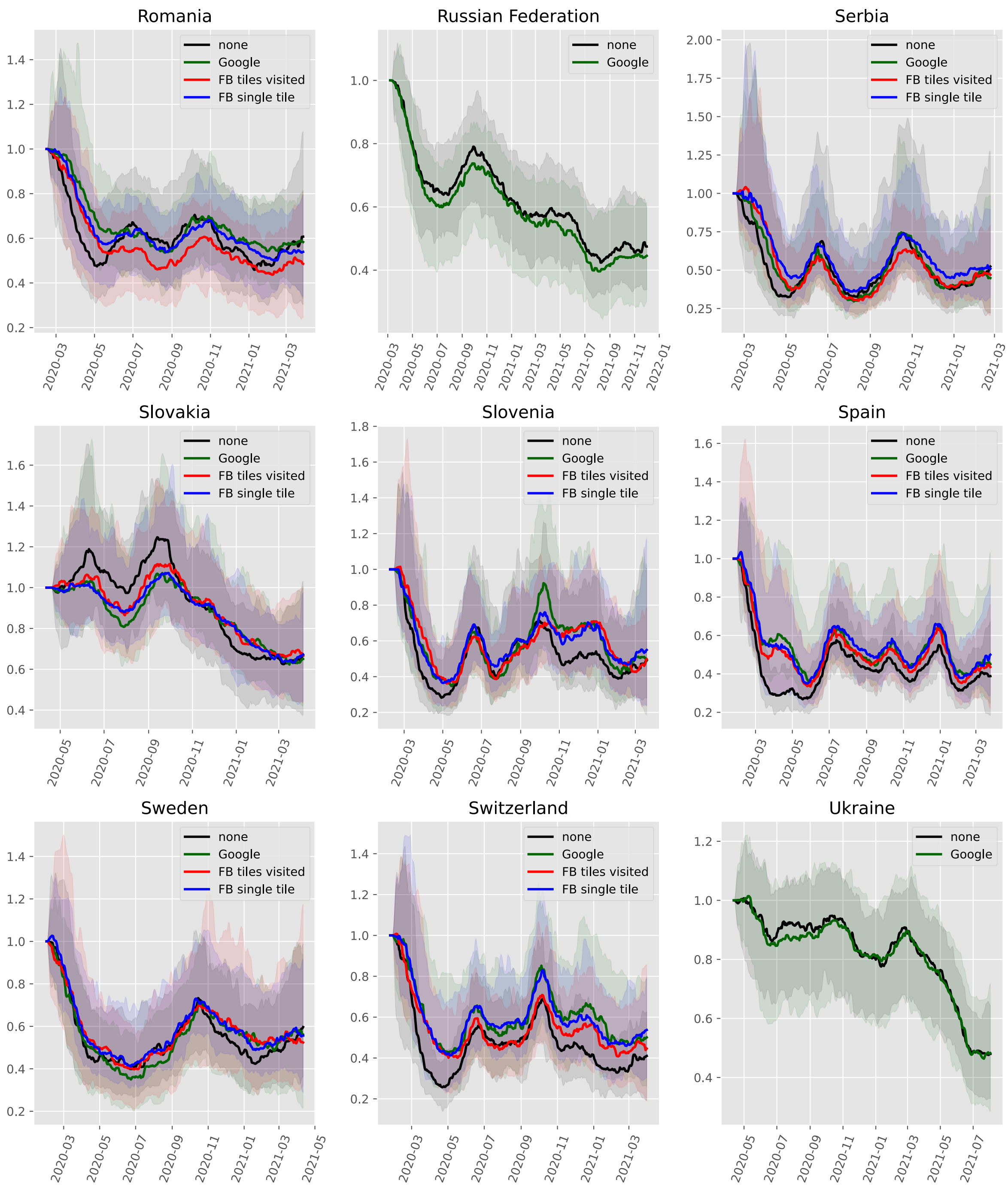
Romania
none
Google
FB tiles visited
FB single tile
Russian Federation
none
Google
Serbia
none
Google
FB tiles visited
FB single tile
Slovakia
none
Google
FB tiles visited
FB single tile
Slovenia
none
Google
FB tiles visited
FB single tile
Spain
none
Google
FB tiles visited
FB single tile
Sweden
none
Google
FB tiles visited
FB single tile
Switzerland
none
Google
FB tiles visited
FB single tile
Ukraine
none
Google

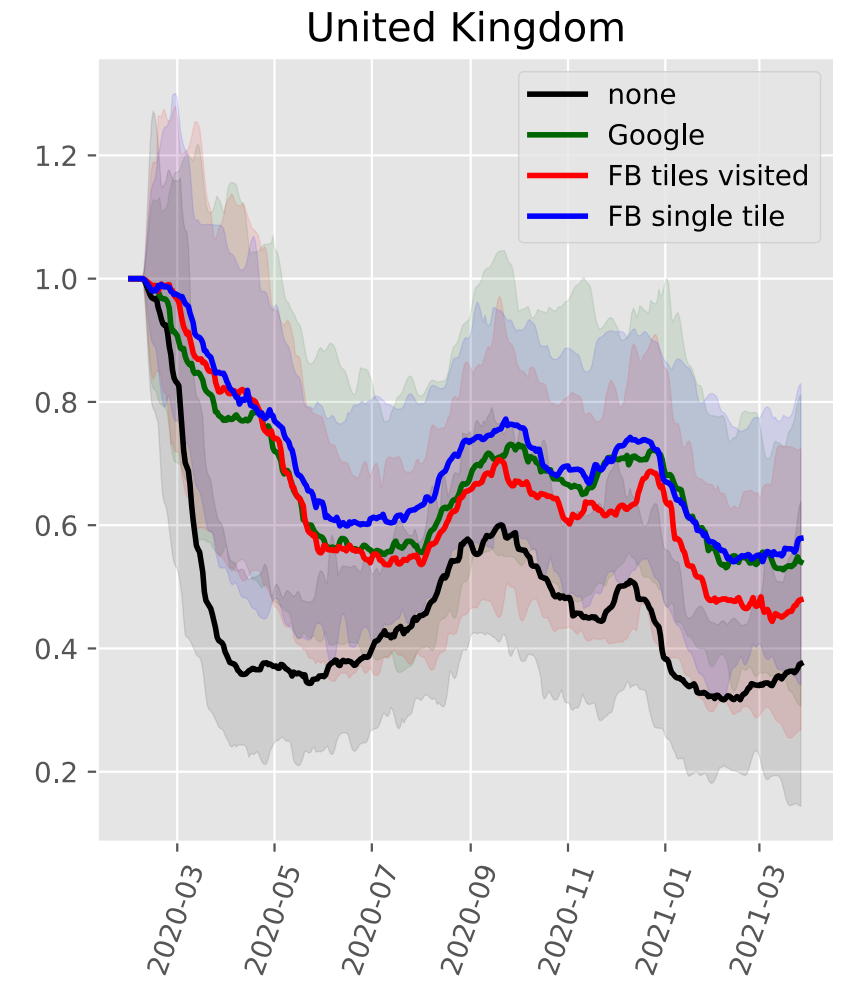
United Kingdom
none
Google
FB tiles visited
FB single tile
1.2
1.0
0.8
0.6
0.4
0.2
2020-03
2020-05
2020-07
2020-09
2020-11
2021-01
2021-03

North America

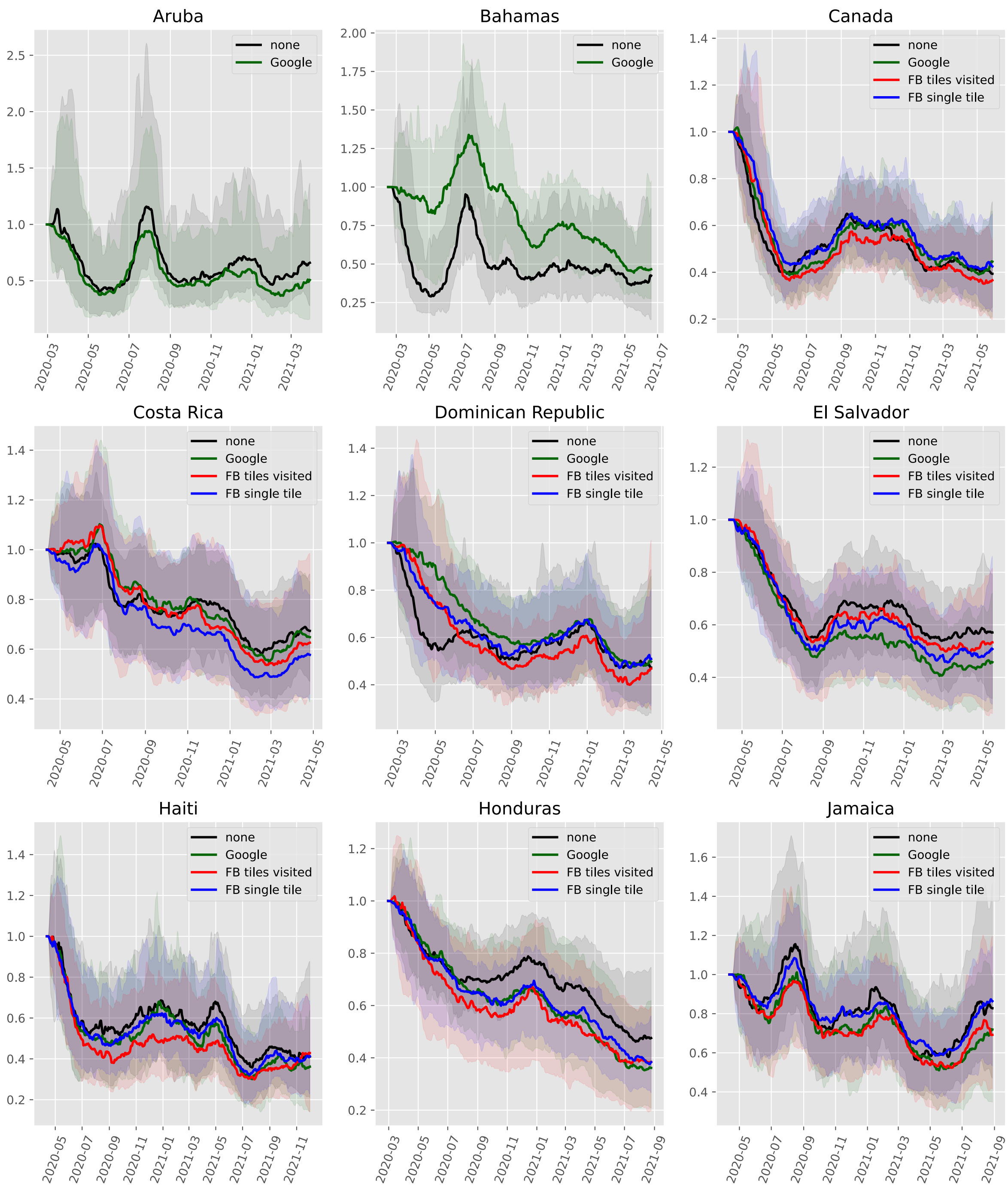

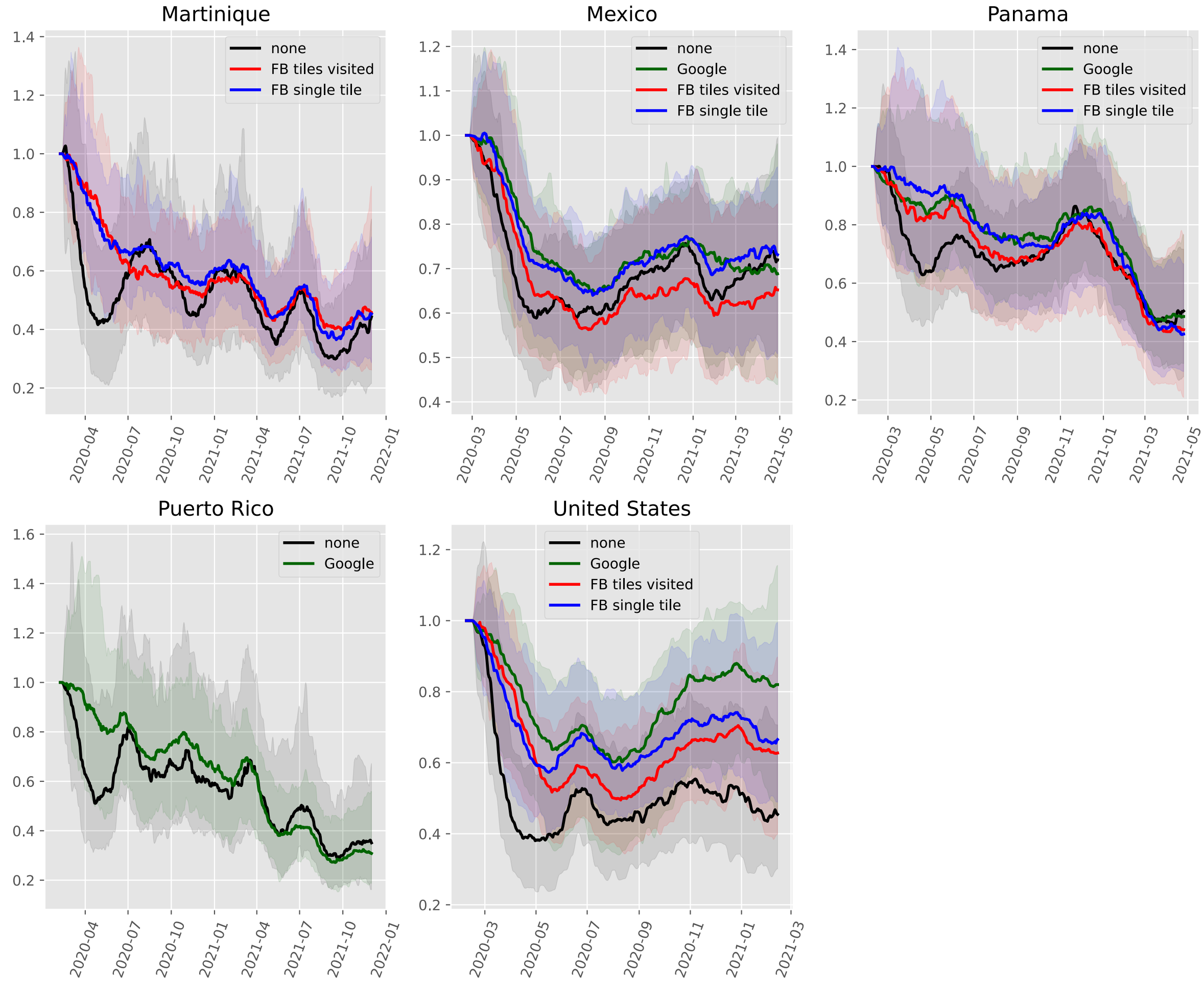
Martinique
none
FB tiles visited
FB single tile
1.4
1.2
1.0
0.8
0.6
0.4
0.2
2020-04
2020-07
2020-10
2021-01
2021-04
2021-07
2021-10
2022-01
Mexico
none
Google
FB tiles visited
FB single tile
1.2
1.1
1.0
0.9
0.8
0.7
0.6
0.5
0.4
2020-03
2020-05
2020-07
2020-09
2020-11
2021-01
2021-03
2021-05
Panama
none
Google
FB tiles visited
FB single tile
1.4
1.2
1.0
0.8
0.6
0.4
0.2
2020-03
2020-05
2020-07
2020-09
2020-11
2021-01
2021-03
2021-05
Puerto Rico
none
Google
1.6
1.4
1.2
1.0
0.8
0.6
0.4
0.2
2020-04
2020-07
2020-10
2021-01
2021-04
2021-07
2021-10
2022-01
United States
none
Google
FB tiles visited
FB single tile
1.2
1.0
0.8
0.6
0.4
0.2
2020-03
2020-05
2020-07
2020-09
2020-11
2021-01
2021-03

Oceania

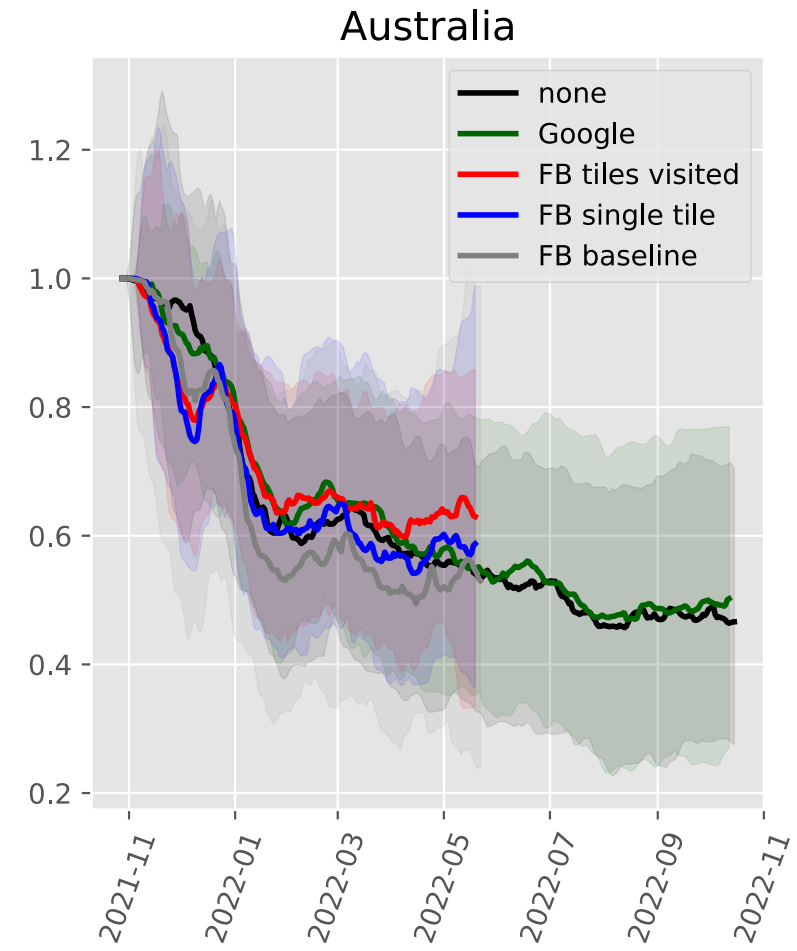
Australia
none
Google
FB tiles visited
FB single tile
FB baseline
0.2
0.4
0.6
0.8
1.0
1.2
2021-11
2022-01
2022-03
2022-05
2022-07
2022-09
2022-11

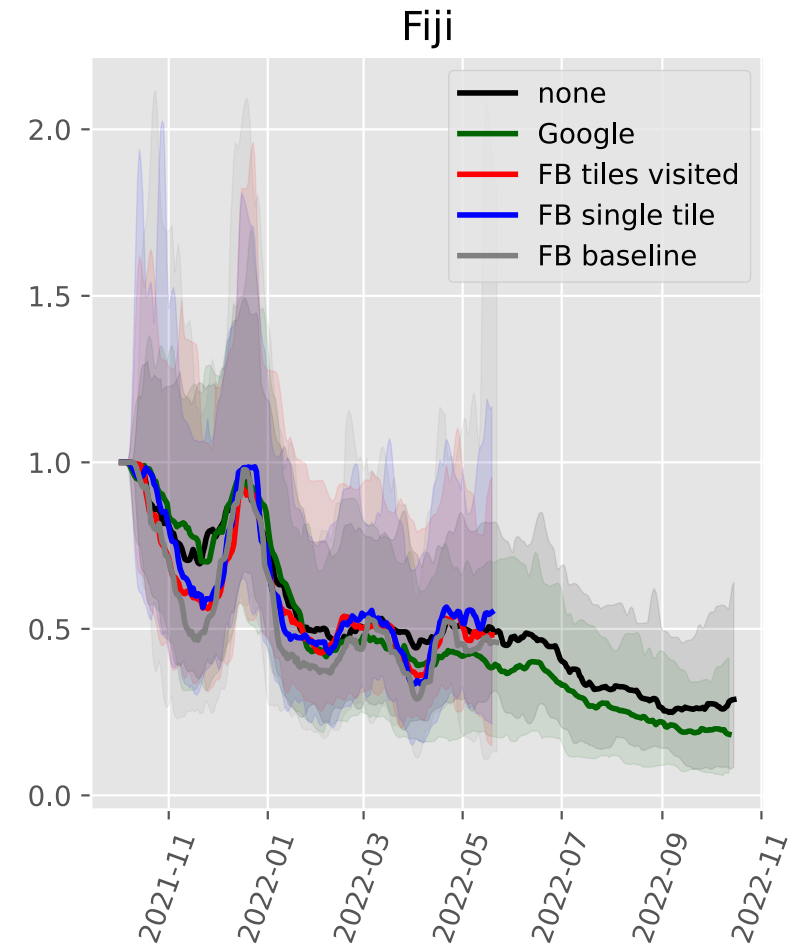
Fiji
none
Google
FB tiles visited
FB single tile
FB baseline
0.0
0.5
1.0
1.5
2.0
2021-11
2022-01
2022-03
2022-05
2022-07
2022-09
2022-11

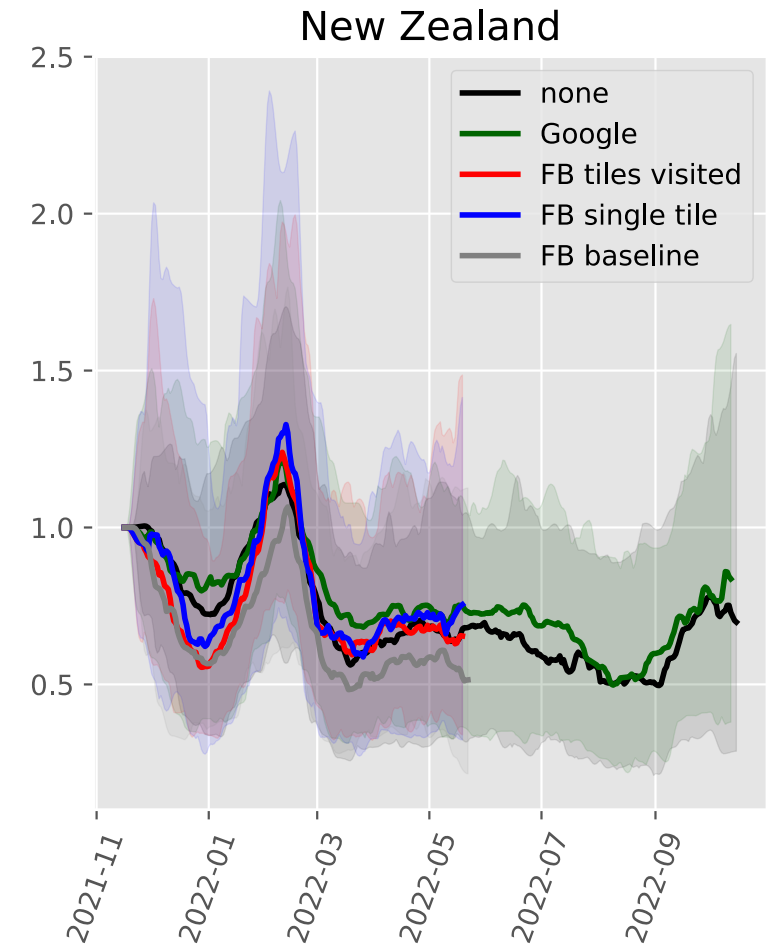
New Zealand
none
Google
FB tiles visited
FB single tile
FB baseline
0.5
1.0
1.5
2.0
2.5
2021-11
2022-01
2022-03
2022-05
2022-07
2022-09

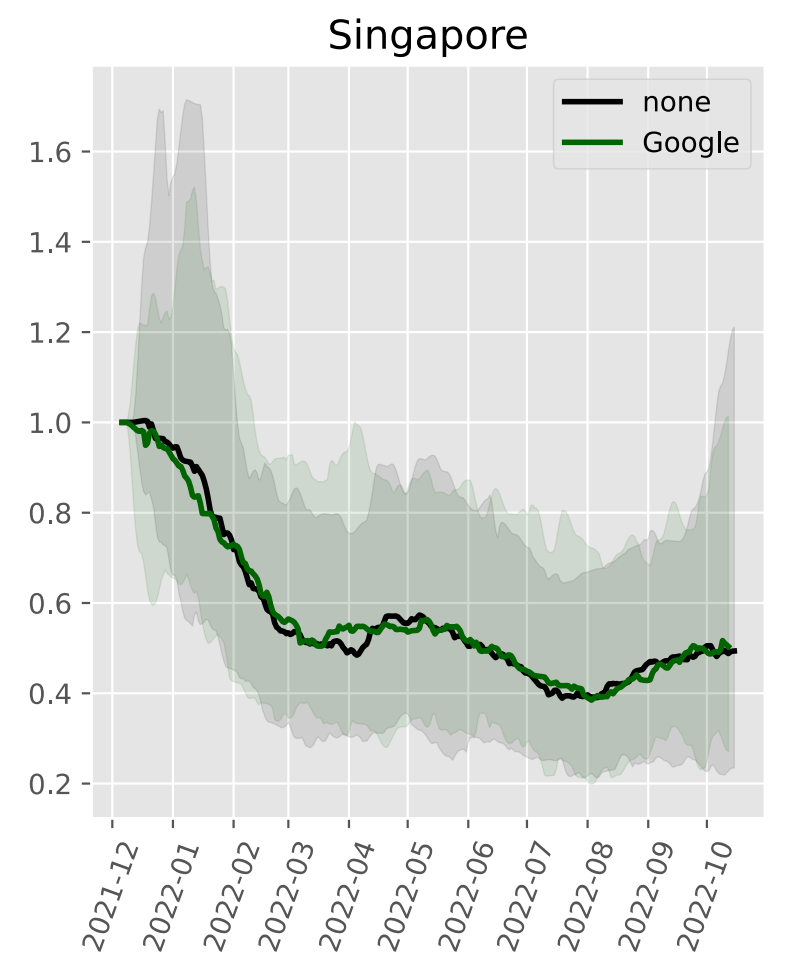
Singapore
none
Google
0.2
0.4
0.6
0.8
1.0
1.2
1.4
1.6
2021-12
2022-01
2022-02
2022-03
2022-04
2022-05
2022-06
2022-07
2022-08
2022-09
2022-10

South America

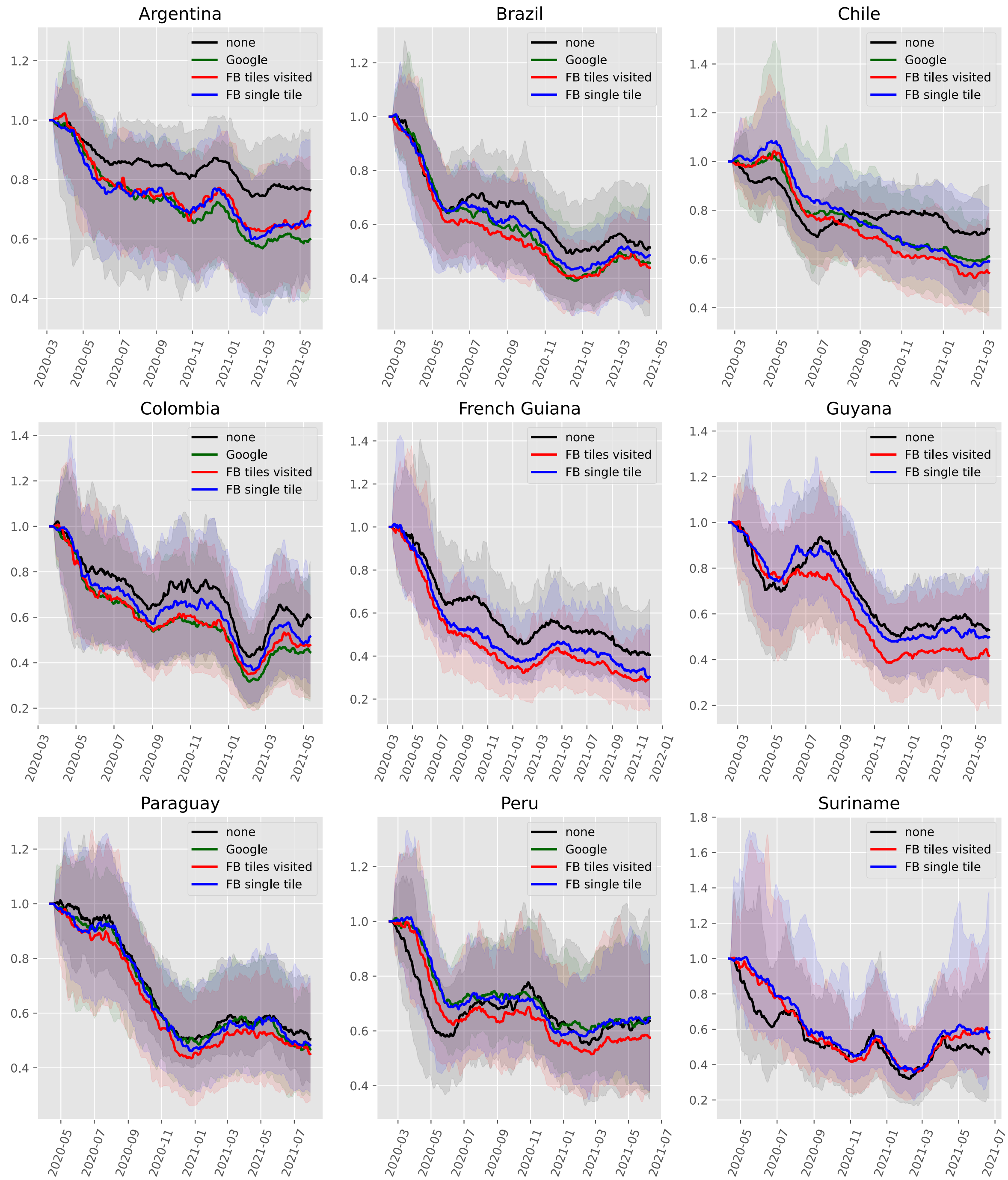

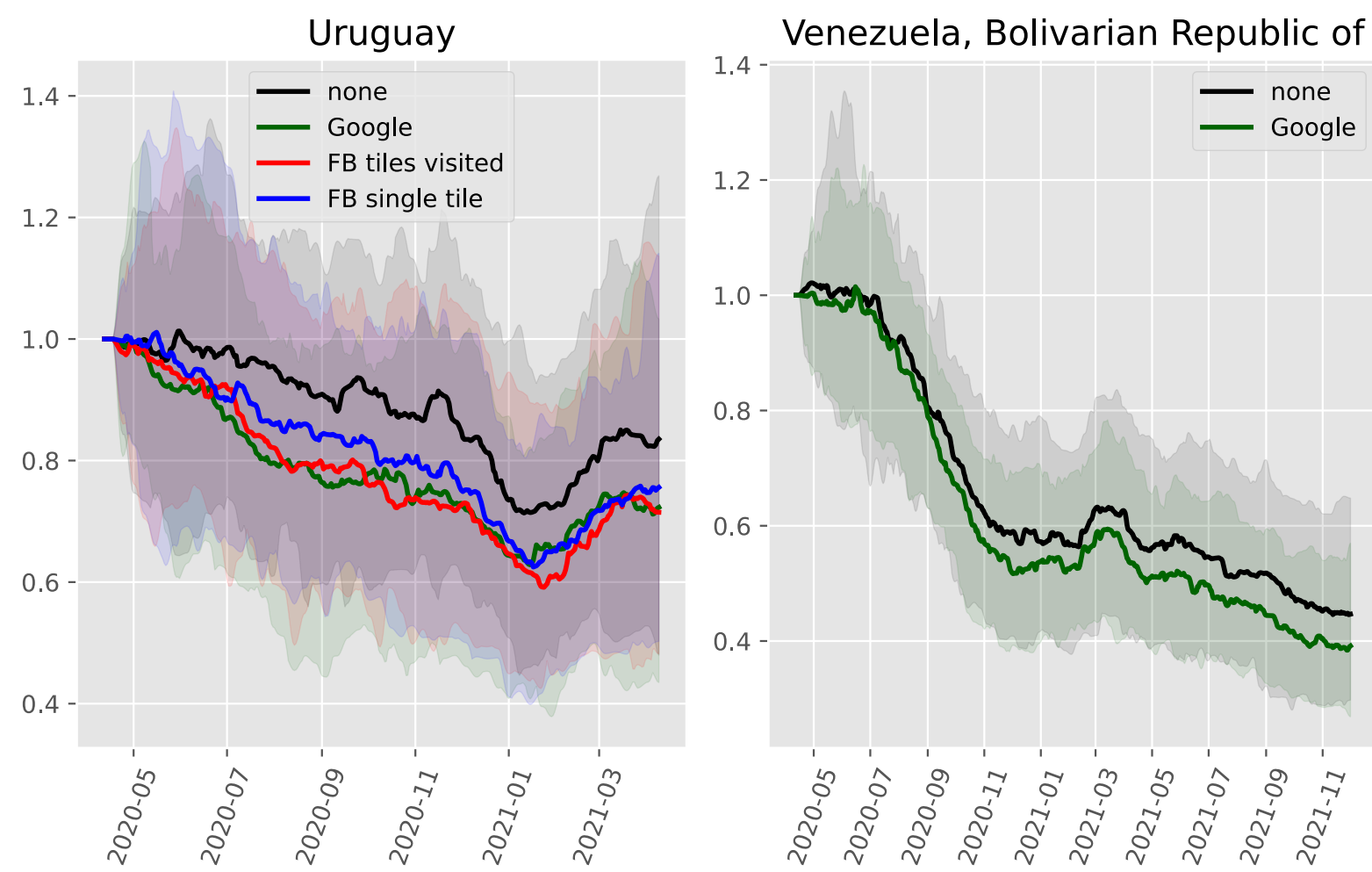
Uruguay
none
Google
FB tiles visited
FB single tile
1.4
1.2
1.0
0.8
0.6
0.4
2020-05
2020-07
2020-09
2020-11
2021-01
2021-03
Venezuela, Bolivarian Republic of
none
Google
1.4
1.2
1.0
0.8
0.6
0.4
2020-05
2020-07
2020-09
2020-11
2021-01
2021-03
2021-05
2021-07
2021-09
2021-11

Comparisons of the non-mechanistic transmission scaling dispersion parameter

This section presents results based on the posterior distribution of the residual transmission scaling dispersion parameter. This quantity governs the distribution in the change in residual scaling for transmission from one value in the process series to the subsequent update. As such, smaller values imply that smaller updates could still result in good calibrations. Lower values for this parameter can therefore be interpreted as less dramatic changes needing to be applied through the non-mechanistic component of the model. As such, we interpreted mobility analysis approaches for which this posterior distribution of this parameter was lower as being a more plausible representation of reality. This is intended as a more formal quantification of the differences in variation in the non-mechanistic transmission scaling presented in the previous section.

Dispersion parameter distributions by analysis and country

Africa

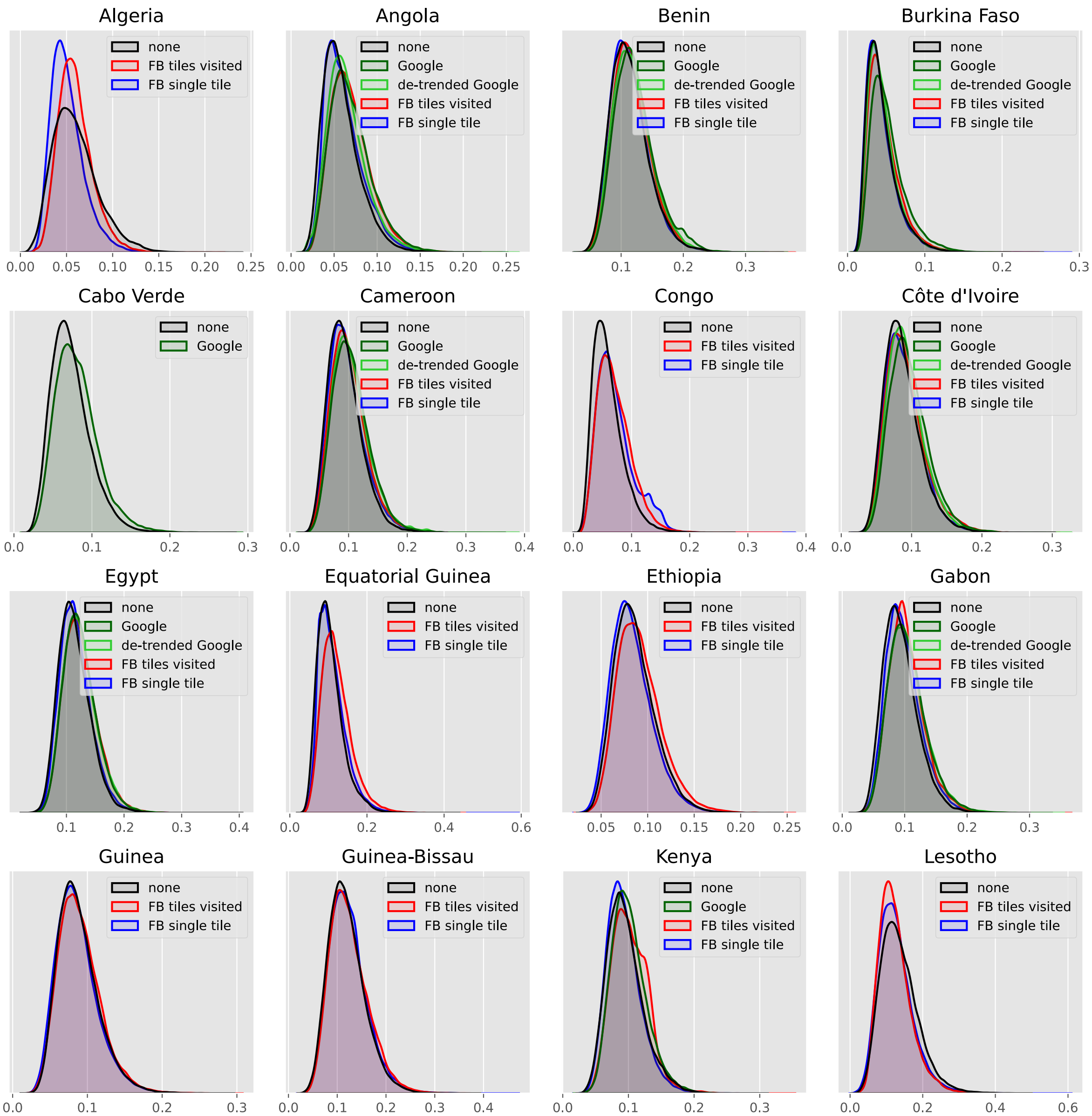

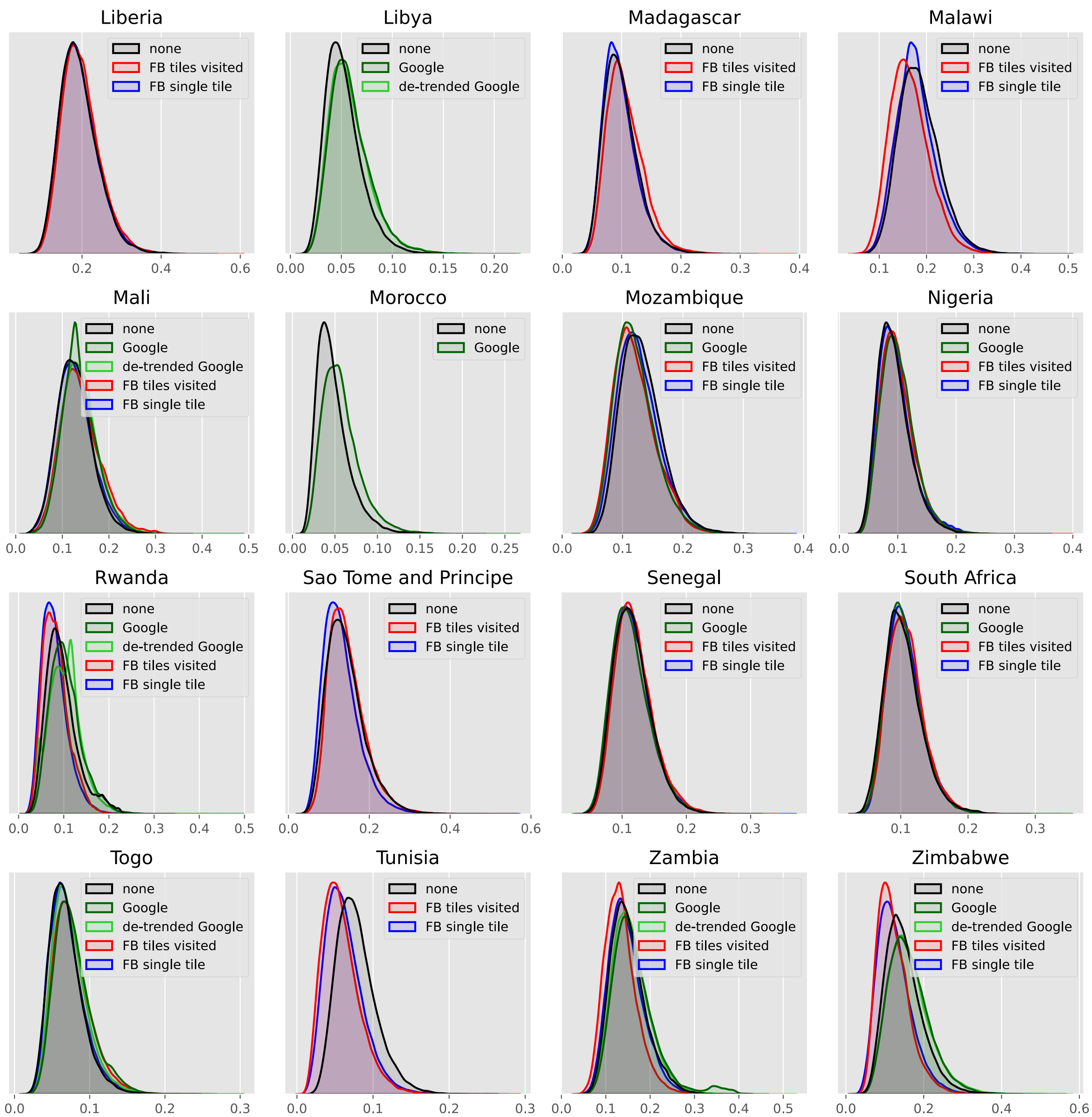
Liberia
none
FB tiles visited
FB single tile
0.2 0.4 0.6
Libya
none
Google
de-trended Google
0.00 0.05 0.10 0.15 0.20
Madagascar
none
FB tiles visited
FB single tile
0.0 0.1 0.2 0.3 0.4
Malawi
none
FB tiles visited
FB single tile
0.1 0.2 0.3 0.4 0.5
Mali
none
Google
de-trended Google
FB tiles visited
FB single tile
0.0 0.1 0.2 0.3 0.4 0.5
Morocco
none
Google
0.00 0.05 0.10 0.15 0.20 0.25
Mozambique
none
Google
FB tiles visited
FB single tile
0.0 0.1 0.2 0.3 0.4
Nigeria
none
Google
FB tiles visited
FB single tile
0.0 0.1 0.2 0.3 0.4
Rwanda
none
Google
de-trended Google
FB tiles visited
FB single tile
0.0 0.1 0.2 0.3 0.4 0.5
Sao Tome and Principe
none
FB tiles visited
FB single tile
0.0 0.2 0.4 0.6
Senegal
none
Google
FB tiles visited
FB single tile
0.1 0.2 0.3
South Africa
none
Google
FB tiles visited
FB single tile
0.1 0.2 0.3
Togo
none
Google
de-trended Google
FB tiles visited
FB single tile
0.0 0.1 0.2 0.3
Tunisia
none
FB tiles visited
FB single tile
0.0 0.1 0.2 0.3
Zambia
none
Google
de-trended Google
FB tiles visited
FB single tile
0.0 0.1 0.2 0.3 0.4 0.5
Zimbabwe
none
Google
de-trended Google
FB tiles visited
FB single tile
0.0 0.2 0.4 0.6

Asia

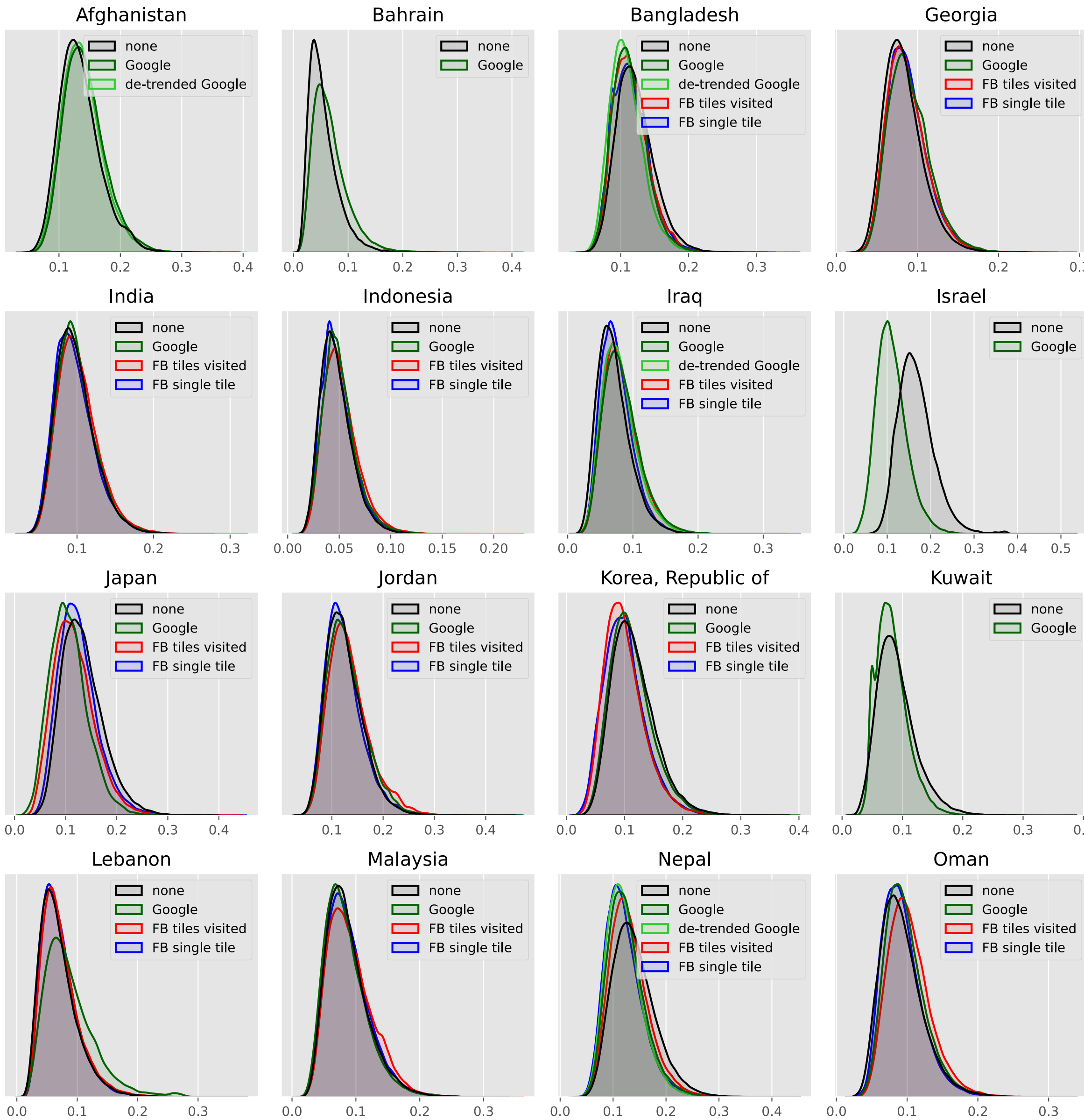

Afghanistan
none
Google
de-trended Google
0.1 0.2 0.3 0.4
Bahrain
none
Google
0.0 0.1 0.2 0.3 0.4
Bangladesh
none
Google
de-trended Google
FB tiles visited
FB single tile
0.1 0.2 0.3
Georgia
none
Google
FB tiles visited
FB single tile
0.0 0.1 0.2 0.3
India
none
Google
FB tiles visited
FB single tile
0.1 0.2 0.3
Indonesia
none
Google
FB tiles visited
FB single tile
0.00 0.05 0.10 0.15 0.20
Iraq
none
Google
de-trended Google
FB tiles visited
FB single tile
0.0 0.1 0.2 0.3
Israel
none
Google
0.0 0.1 0.2 0.3 0.4 0.5
Japan
none
Google
FB tiles visited
FB single tile
0.0 0.1 0.2 0.3 0.4
Jordan
none
Google
FB tiles visited
FB single tile
0.1 0.2 0.3 0.4
Korea, Republic of
none
Google
FB tiles visited
FB single tile
0.0 0.1 0.2 0.3 0.4
Kuwait
none
Google
0.0 0.1 0.2 0.3 0.4
Lebanon
none
Google
FB tiles visited
FB single tile
0.0 0.1 0.2 0.3
Malaysia
none
Google
FB tiles visited
FB single tile
0.0 0.1 0.2 0.3
Nepal
none
Google
de-trended Google
FB tiles visited
FB single tile
0.0 0.1 0.2 0.3 0.4
Oman
none
Google
FB tiles visited
FB single tile
0.0 0.1 0.2 0.3

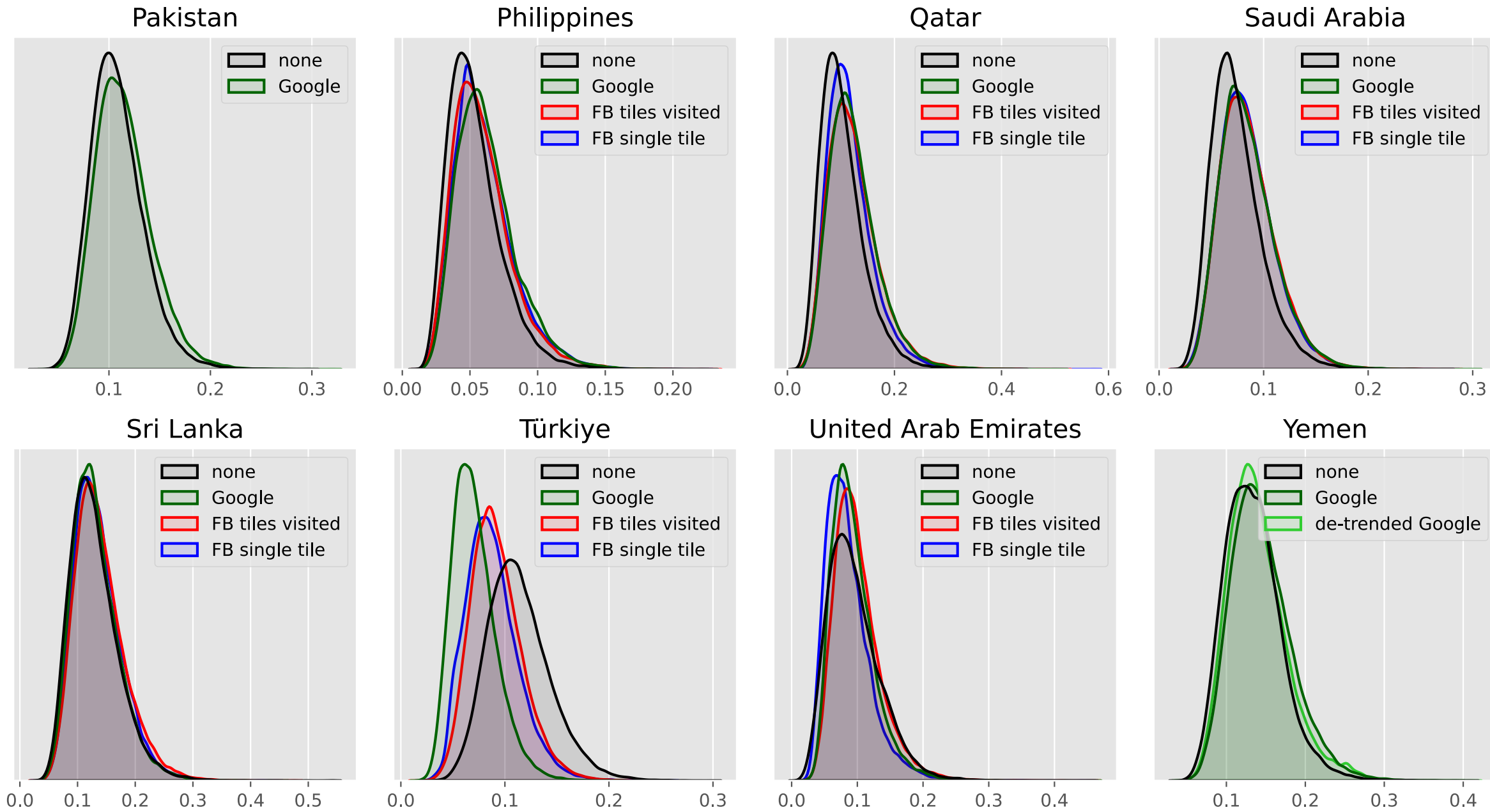
Pakistan
none
Google
0.1
0.2
0.3
Philippines
none
Google
FB tiles visited
FB single tile
0.00
0.05
0.10
0.15
0.20
Qatar
none
Google
FB tiles visited
FB single tile
0.0
0.2
0.4
0.6
Saudi Arabia
none
Google
FB tiles visited
FB single tile
0.0
0.1
0.2
0.3
Sri Lanka
none
Google
FB tiles visited
FB single tile
0.0
0.1
0.2
0.3
0.4
0.5
Türkiye
none
Google
FB tiles visited
FB single tile
0.0
0.1
0.2
0.3
United Arab Emirates
none
Google
FB tiles visited
FB single tile
0.0
0.1
0.2
0.3
0.4
Yemen
none
Google
de-trended Google
0.1
0.2
0.3
0.4

Europe

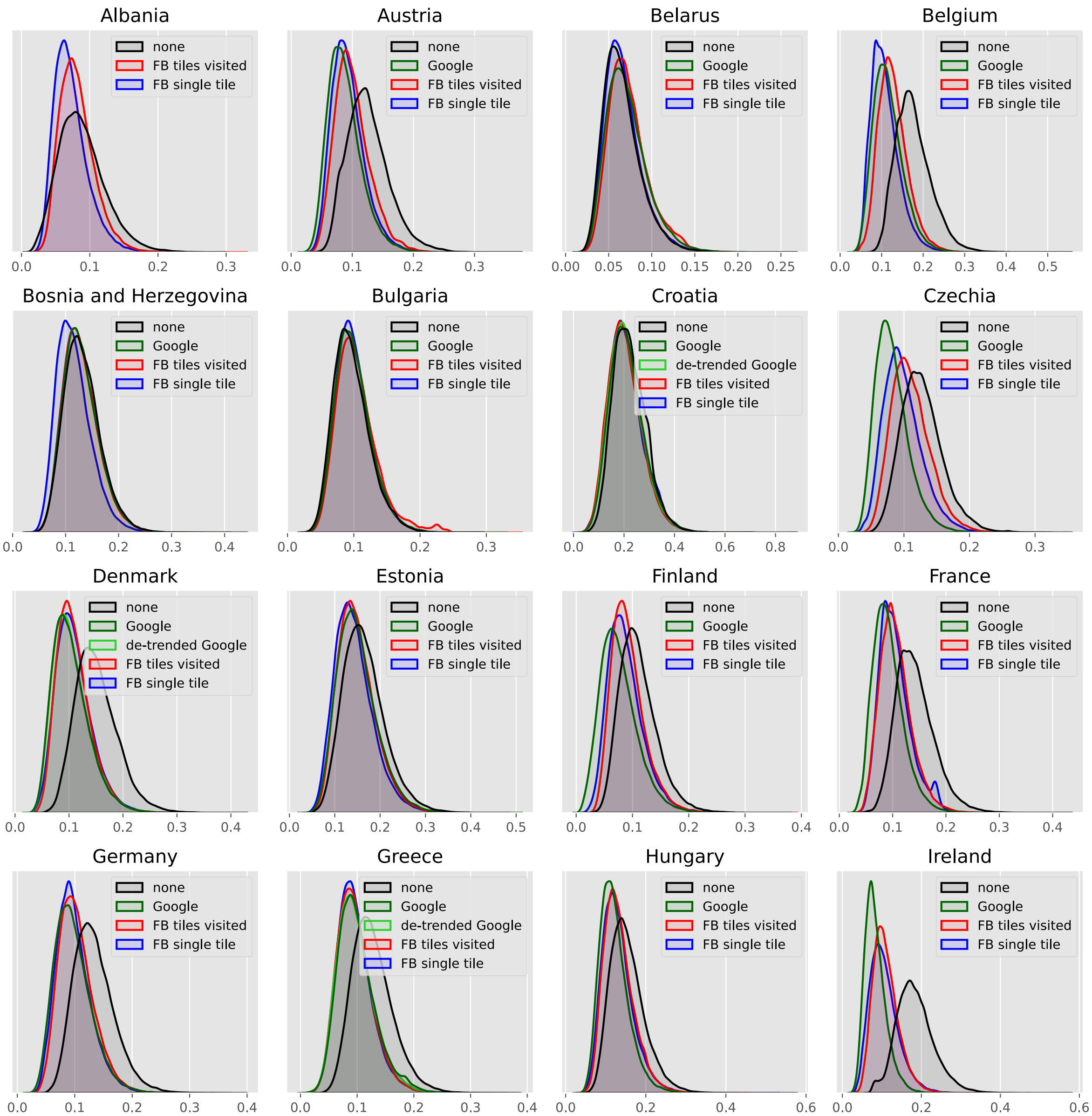
Albania
none
FB tiles visited
FB single tile
0.0 0.1 0.2 0.3
Austria
none
Google
FB tiles visited
FB single tile
0.0 0.1 0.2 0.3
Belarus
none
Google
FB tiles visited
FB single tile
0.00 0.05 0.10 0.15 0.20 0.25
Belgium
none
Google
FB tiles visited
FB single tile
0.0 0.1 0.2 0.3 0.4 0.5
Bosnia and Herzegovina
none
Google
FB tiles visited
FB single tile
0.0 0.1 0.2 0.3 0.4
Bulgaria
none
Google
FB tiles visited
FB single tile
0.0 0.1 0.2 0.3
Croatia
none
Google
de-trended Google
FB tiles visited
FB single tile
0.0 0.2 0.4 0.6 0.8
Czechia
none
Google
FB tiles visited
FB single tile
0.0 0.1 0.2 0.3
Denmark
none
Google
de-trended Google
FB tiles visited
FB single tile
0.0 0.1 0.2 0.3 0.4
Estonia
none
Google
FB tiles visited
FB single tile
0.0 0.1 0.2 0.3 0.4 0.5
Finland
none
Google
FB tiles visited
FB single tile
0.0 0.1 0.2 0.3 0.4
France
none
Google
FB tiles visited
FB single tile
0.0 0.1 0.2 0.3 0.4
Germany
none
Google
FB tiles visited
FB single tile
0.0 0.1 0.2 0.3 0.4
Greece
none
Google
de-trended Google
FB tiles visited
FB single tile
0.0 0.1 0.2 0.3 0.4
Hungary
none
Google
FB tiles visited
FB single tile
0.0 0.2 0.4 0.6
Ireland
none
Google
FB tiles visited
FB single tile
0.0 0.2 0.4 0.6

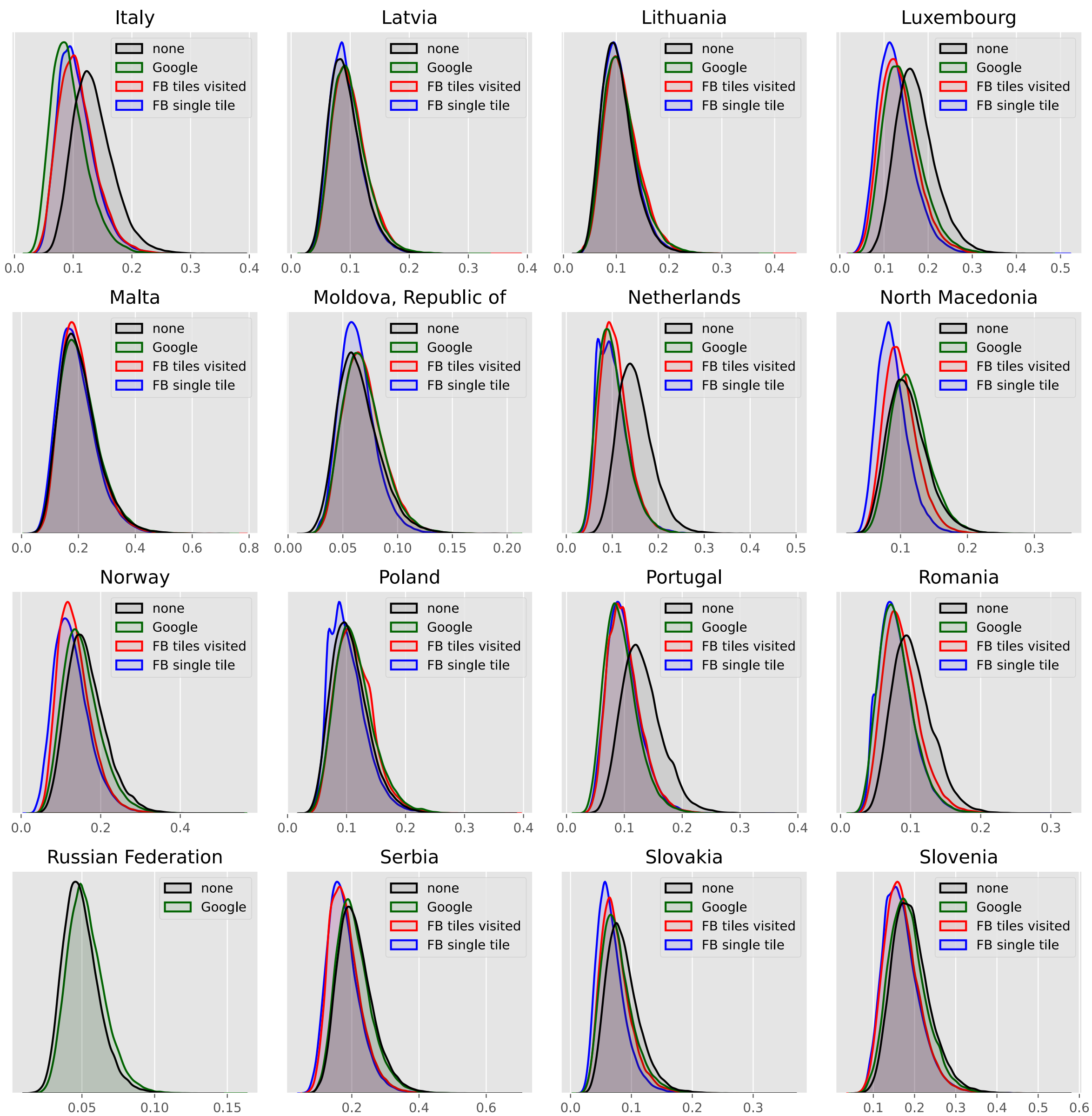
Italy
none
Google
FB tiles visited
FB single tile
0.0 0.1 0.2 0.3 0.4
Latvia
none
Google
FB tiles visited
FB single tile
0.0 0.1 0.2 0.3 0.4
Lithuania
none
Google
FB tiles visited
FB single tile
0.0 0.1 0.2 0.3 0.4
Luxembourg
none
Google
FB tiles visited
FB single tile
0.0 0.1 0.2 0.3 0.4 0.5
Malta
none
Google
FB tiles visited
FB single tile
0.0 0.2 0.4 0.6 0.8
Moldova, Republic of
none
Google
FB tiles visited
FB single tile
0.00 0.05 0.10 0.15 0.20
Netherlands
none
Google
FB tiles visited
FB single tile
0.0 0.1 0.2 0.3 0.4 0.5
North Macedonia
none
Google
FB tiles visited
FB single tile
0.1 0.2 0.3
Norway
none
Google
FB tiles visited
FB single tile
0.0 0.2 0.4
Poland
none
Google
FB tiles visited
FB single tile
0.0 0.1 0.2 0.3 0.4
Portugal
none
Google
FB tiles visited
FB single tile
0.0 0.1 0.2 0.3 0.4
Romania
none
Google
FB tiles visited
FB single tile
0.0 0.1 0.2 0.3
Russian Federation
none
Google
0.05 0.10 0.15
Serbia
none
Google
FB tiles visited
FB single tile
0.2 0.4 0.6
Slovakia
none
Google
FB tiles visited
FB single tile
0.0 0.1 0.2 0.3
Slovenia
none
Google
FB tiles visited
FB single tile
0.1 0.2 0.3 0.4 0.5 0.6

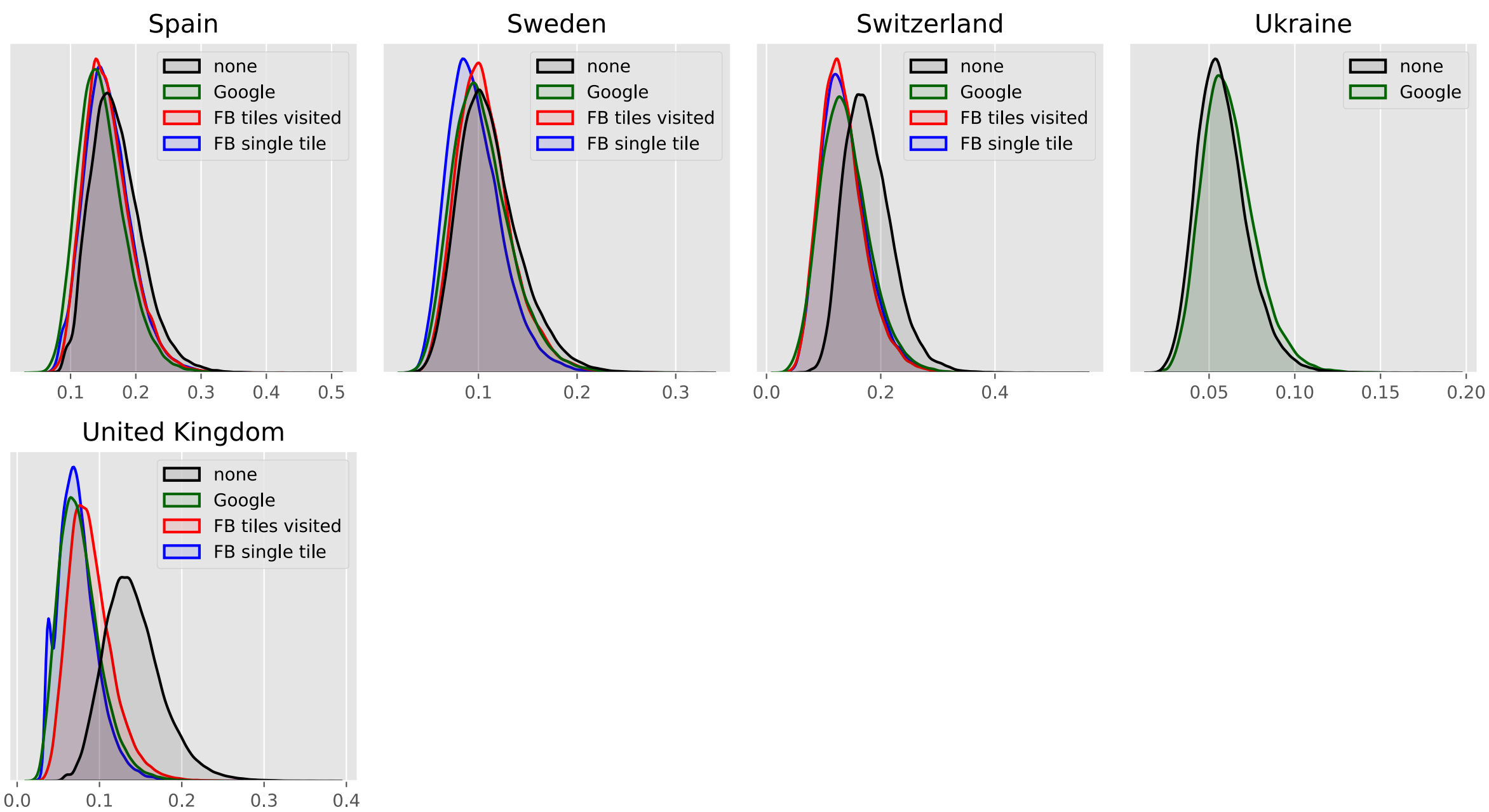
Spain
none
Google
FB tiles visited
FB single tile
0.1 0.2 0.3 0.4 0.5
Sweden
none
Google
FB tiles visited
FB single tile
0.1 0.2 0.3
Switzerland
none
Google
FB tiles visited
FB single tile
0.0 0.2 0.4
Ukraine
none
Google
0.05 0.10 0.15 0.20
United Kingdom
none
Google
FB tiles visited
FB single tile
0.0 0.1 0.2 0.3 0.4

North America

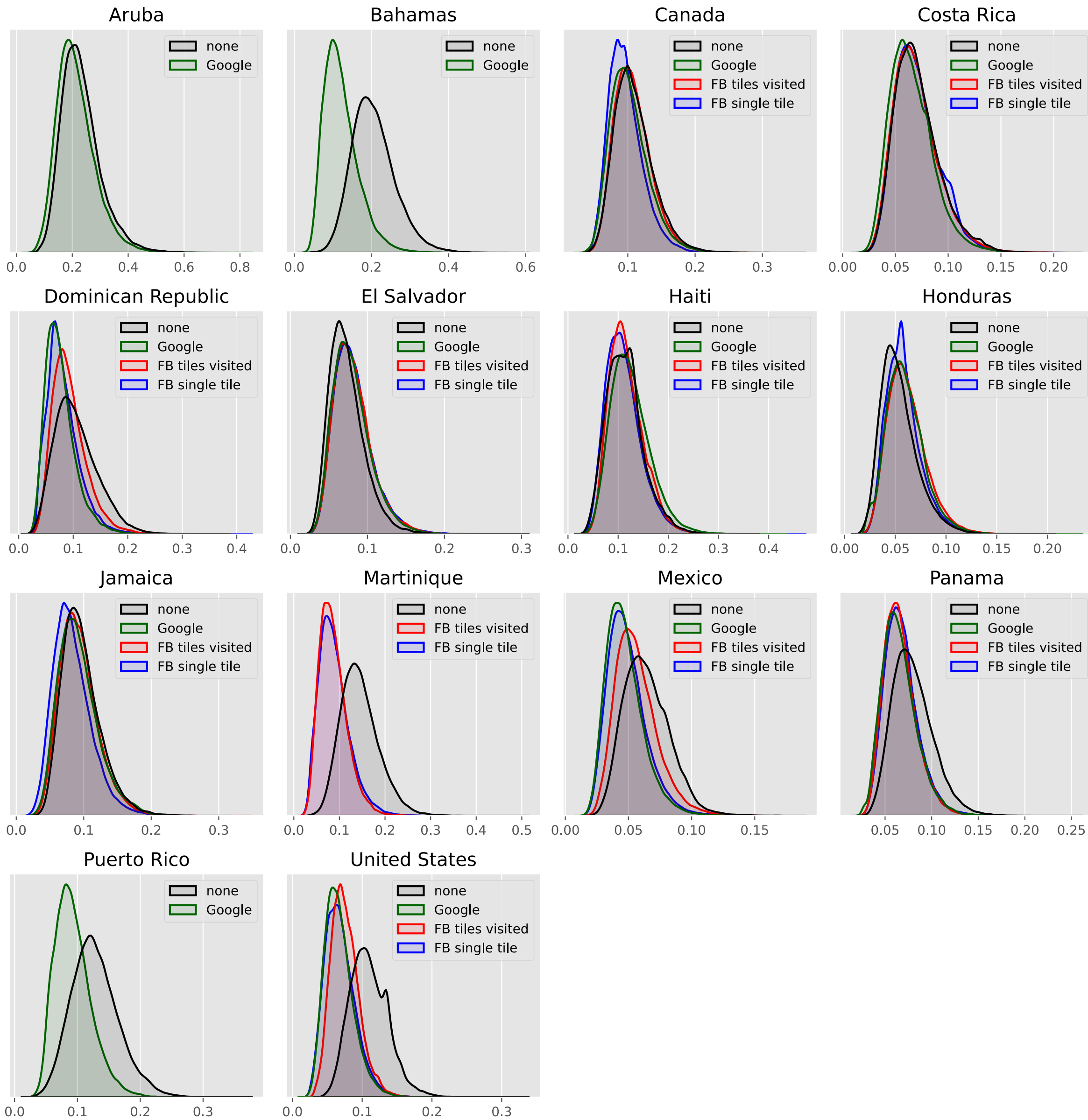

Oceania

Note that for the countries of Oceania, a different baseline analysis was used for to compare Google and Facebook analyses against. This was done because the conclusion of the Facebook data meant that the analysis period differed from the Google analysis.

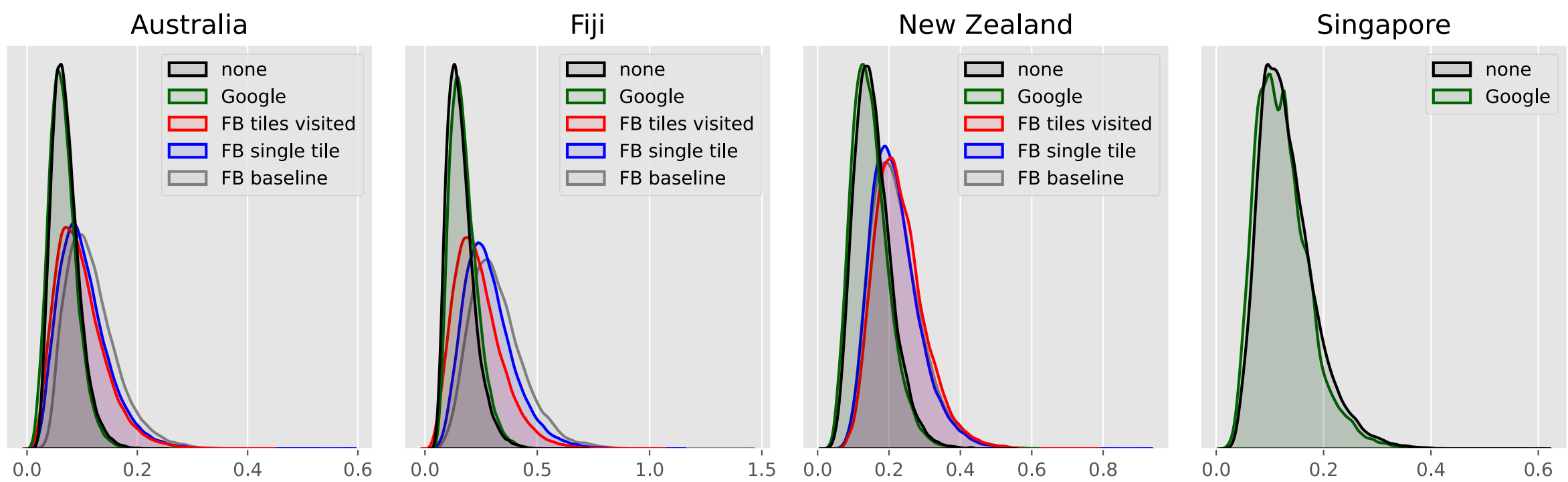

South America

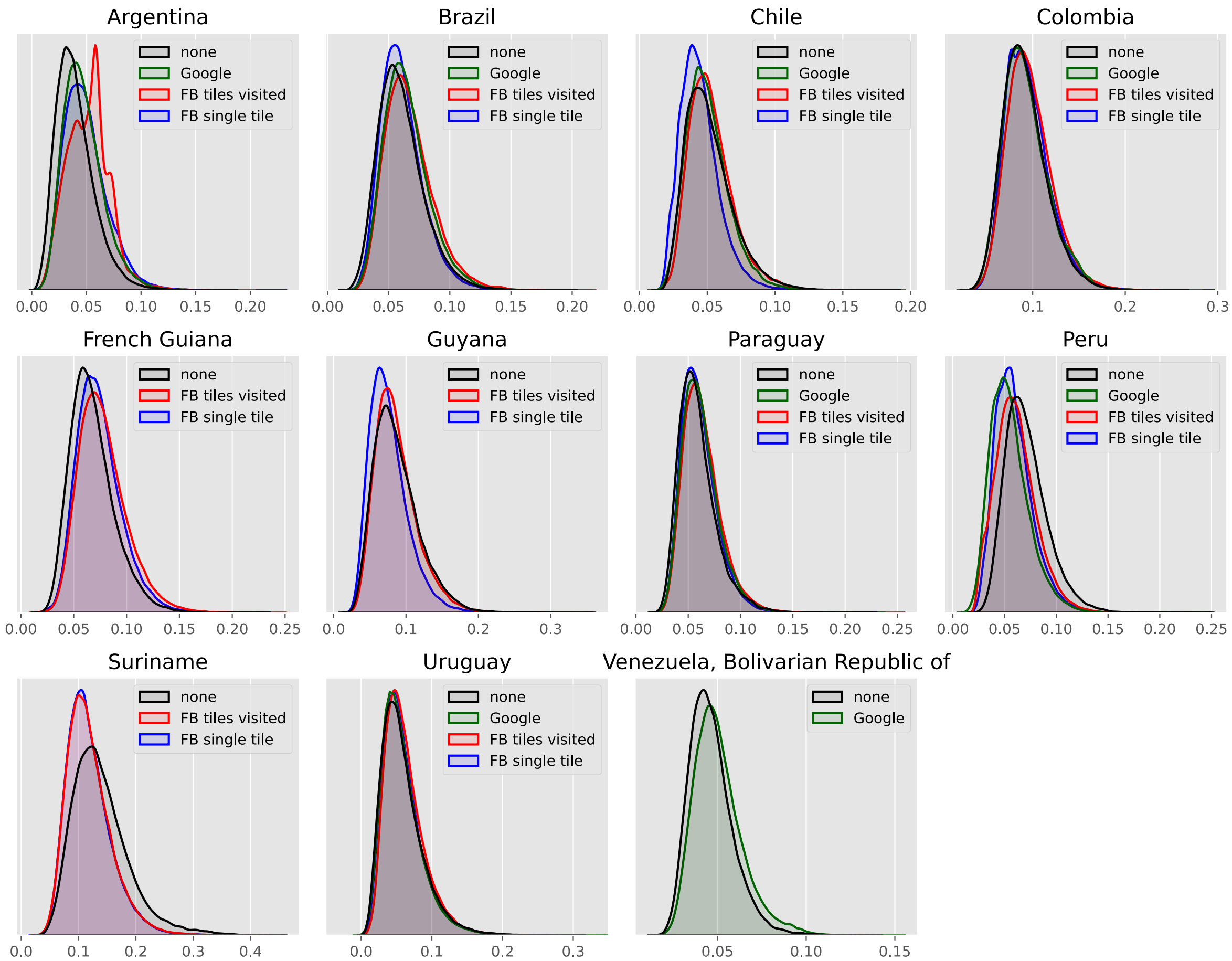

Mobility exponent parameter posteriors

This section presents estimates of the mobility exponent parameter posteriors by country and mobility analysis type. Values from zero to two were considered plausible (the prior for this parameter was uniform over domain $[0, 2]$). A value of zero represents no effect, a value of one represents a linear scaling in transmission with mobility changes, and a value of two represents a quadratic scaling in transmission with mobility changes (which could be epidemiologically considered as an effect on both infector and infectee). For all violin plots, the vertical axis represents the mobility exponent parameter distribution, while the darkness of the coloured shading represents the median of the relative values of the dispersion parameter posterior under the analysis with mobility included compared against the baseline simulation.

Mobility exponent parameter posterior distributions by country

Google mobility

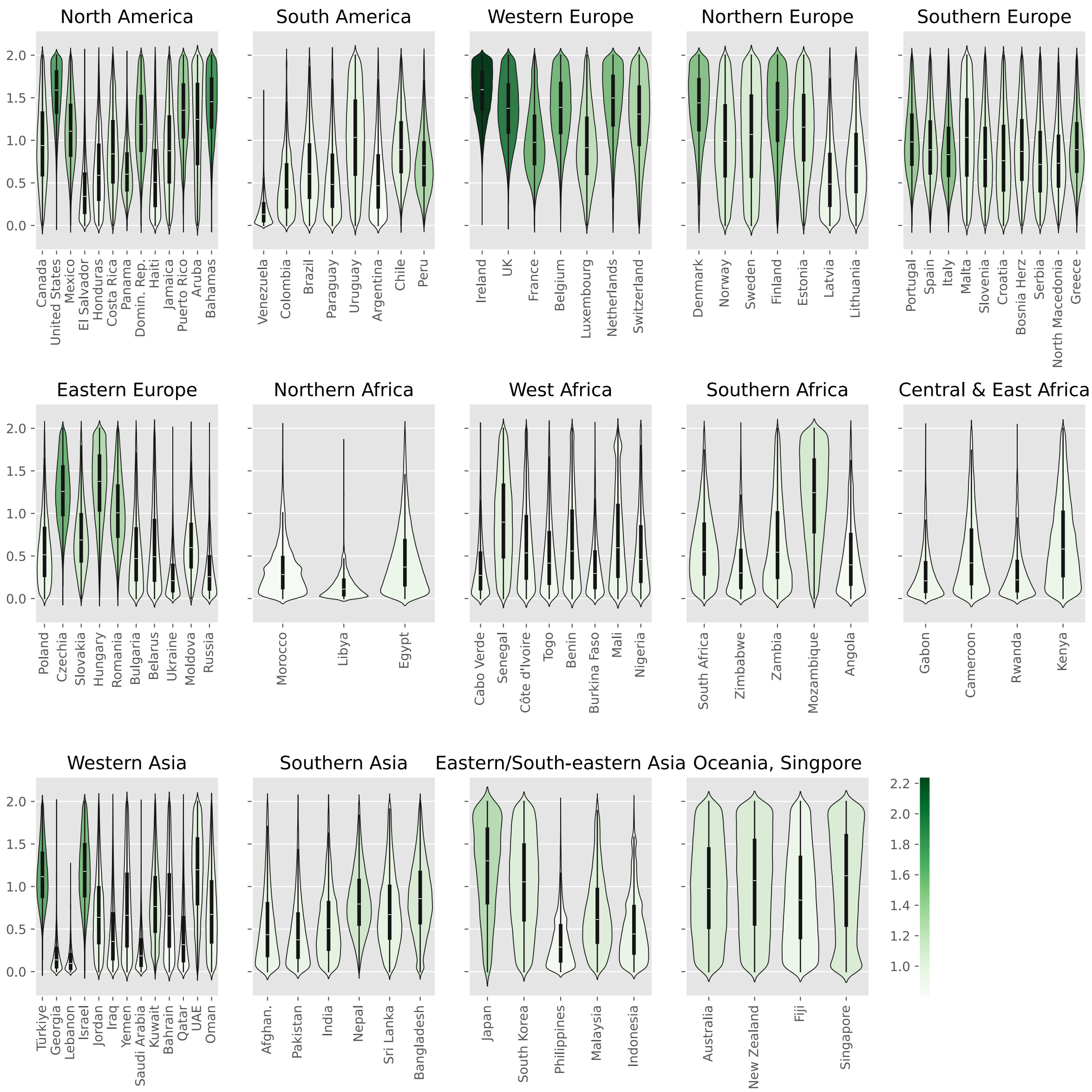

de-trended Google mobility

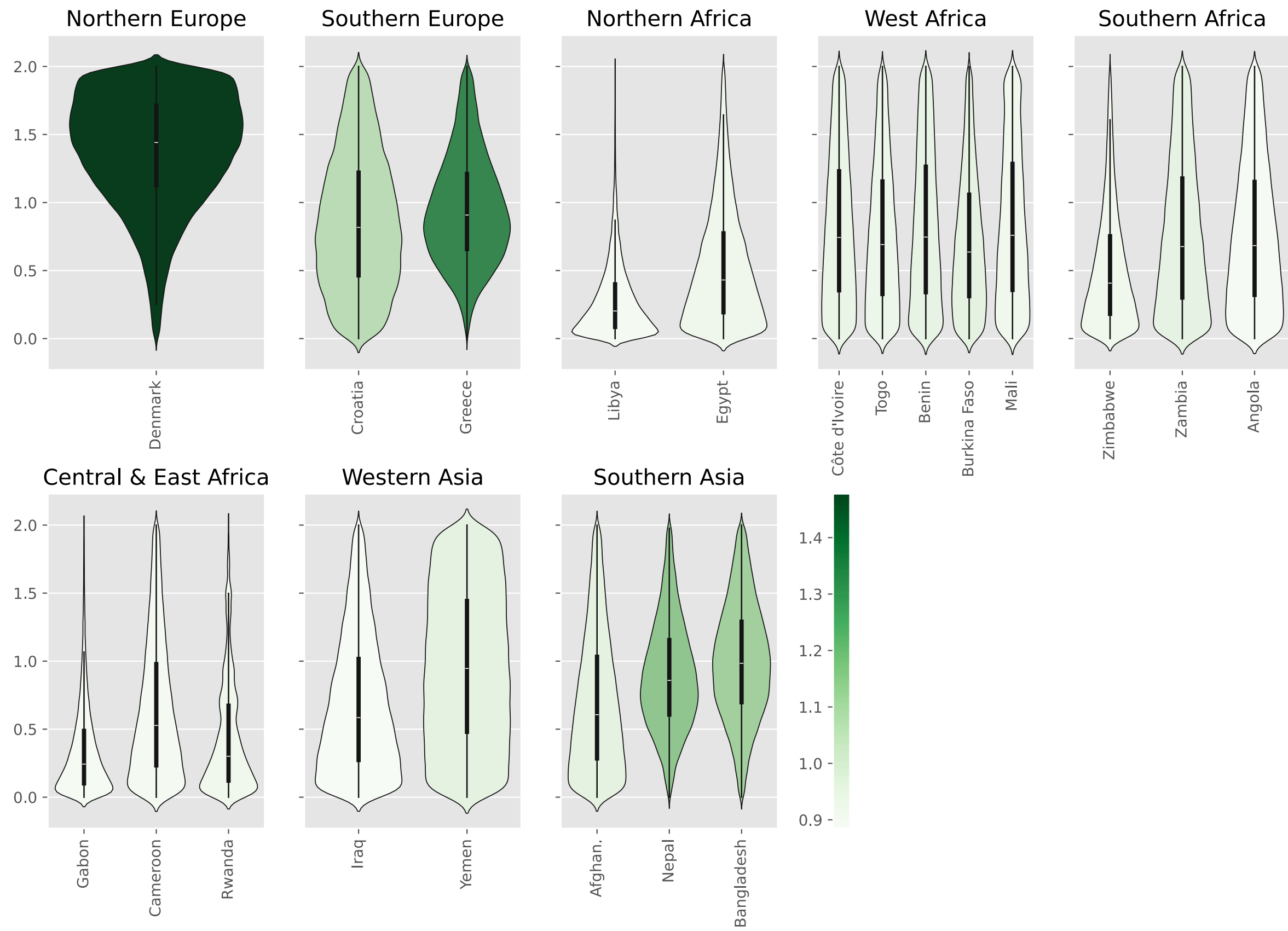

Facebook tiles visited mobility

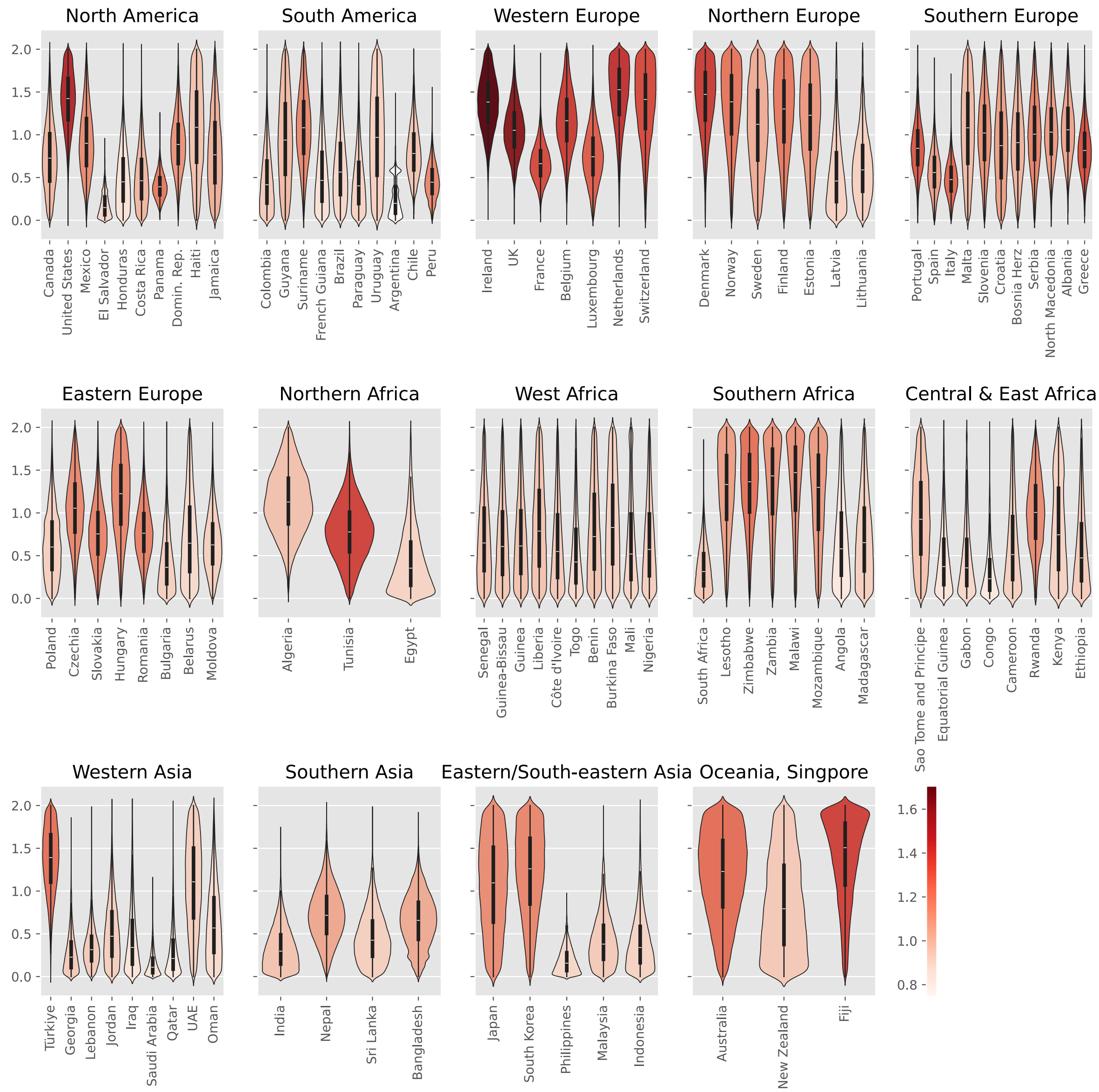

Facebook single tile mobility

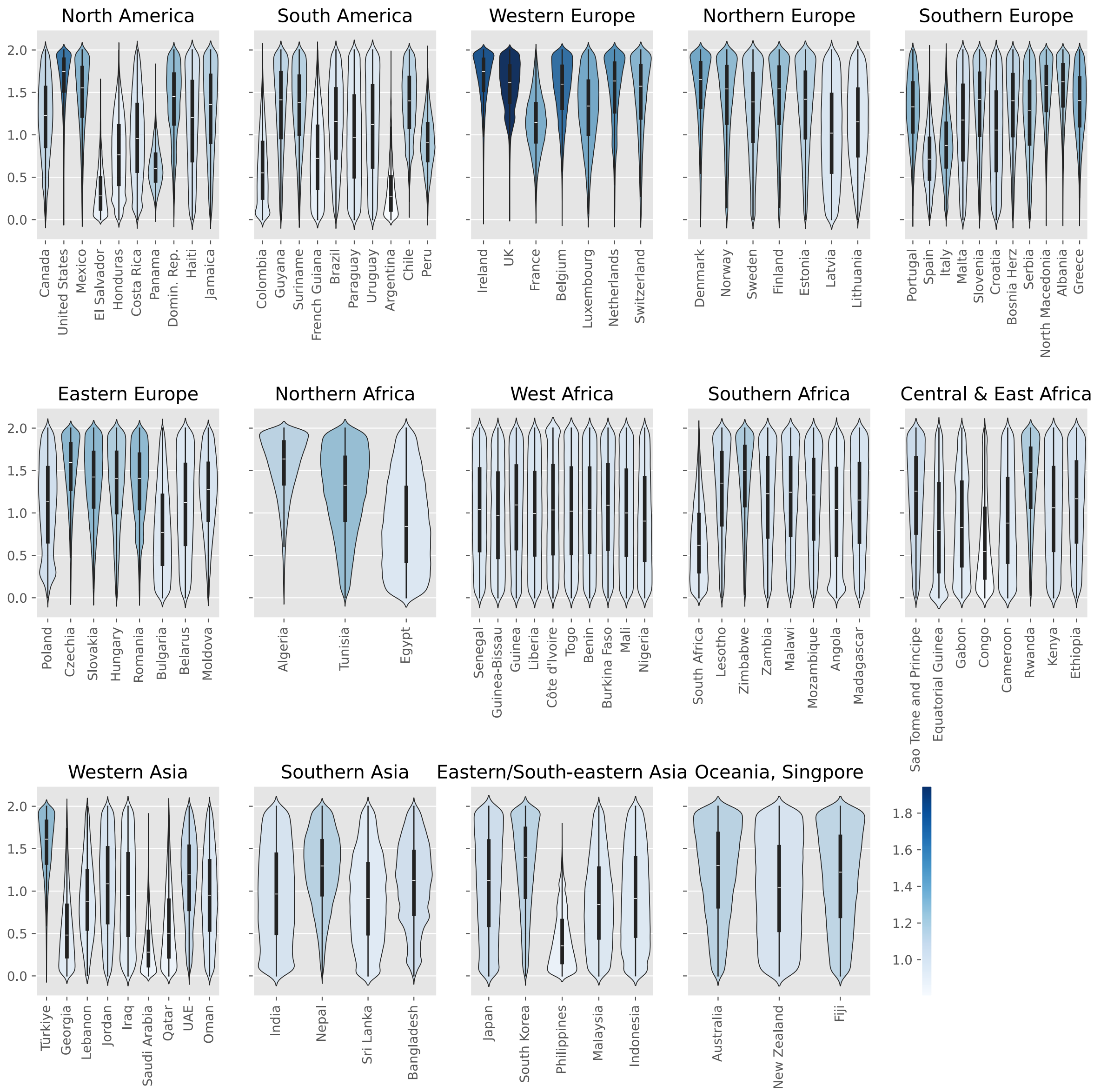

Mobility weight posteriors

This document presents results for the weights for each Google mobility location. Under the Google mobility analysis approach, multiple locations were available. To create an overall time series of Google mobility, we weighted each contribution according to a calibrated parameter

ranging between zero and one, which was then normalised to sum to one. The distribution of the parameter for each location is presented as follows.

Results by continent

Africa

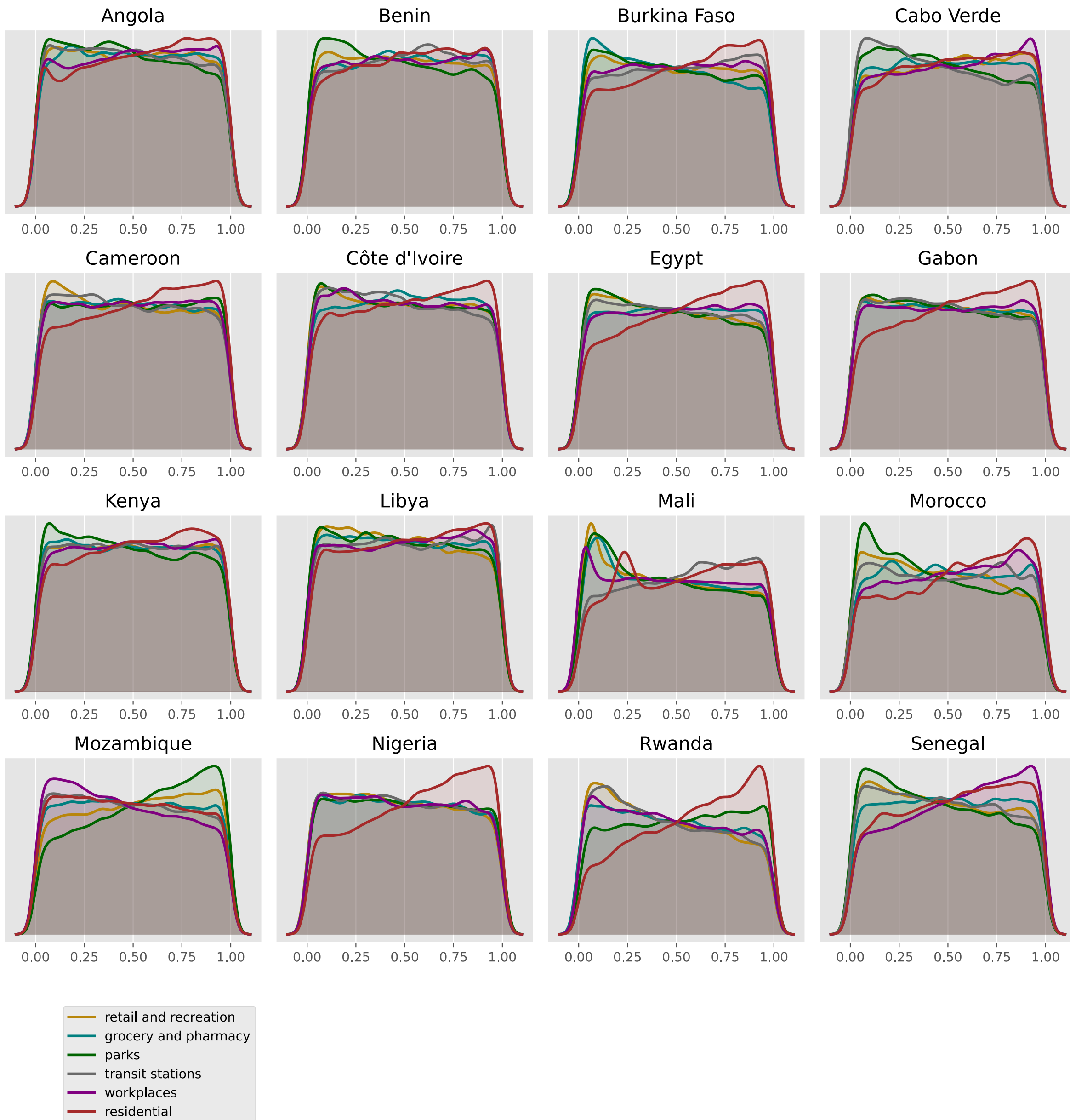

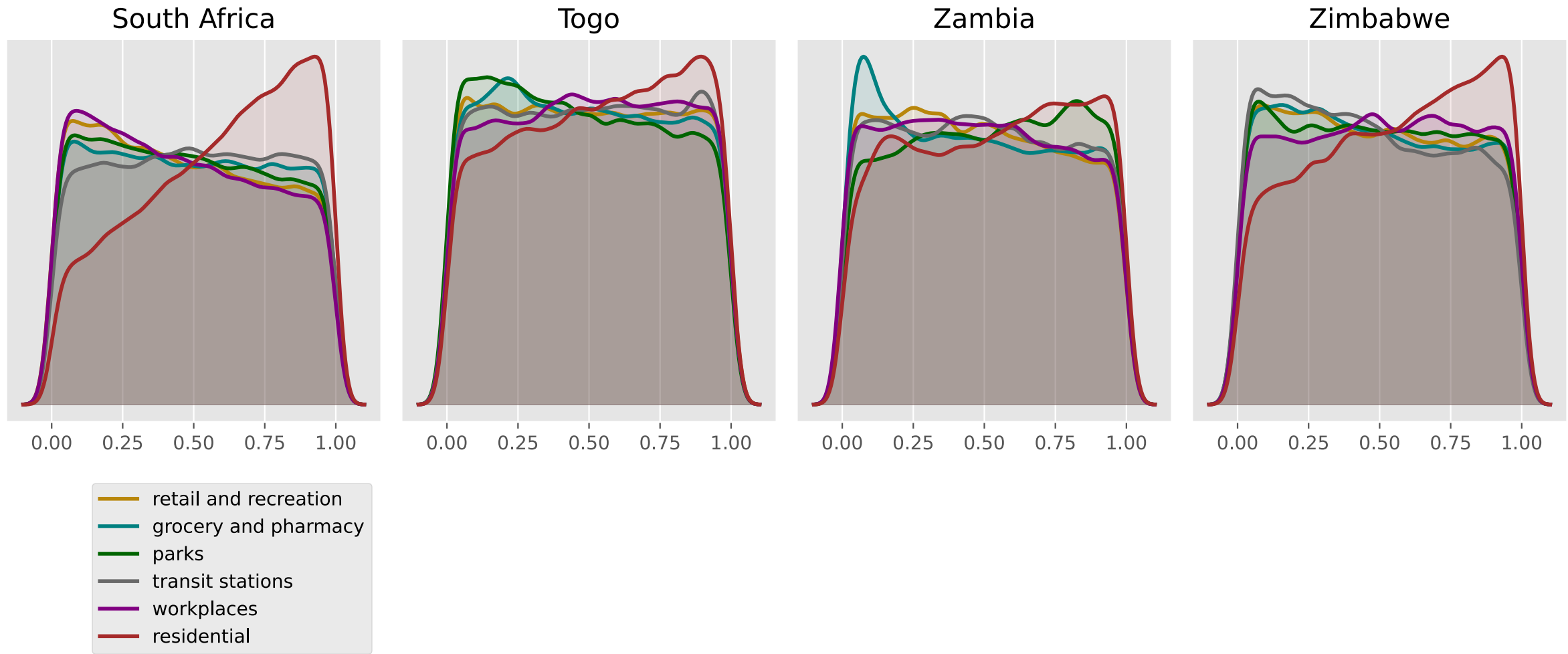
South Africa
Togo
Zambia
Zimbabwe
0.00
0.25
0.50
0.75
1.00
retail and recreation
grocery and pharmacy
parks
transit stations
workplaces
residential

Asia

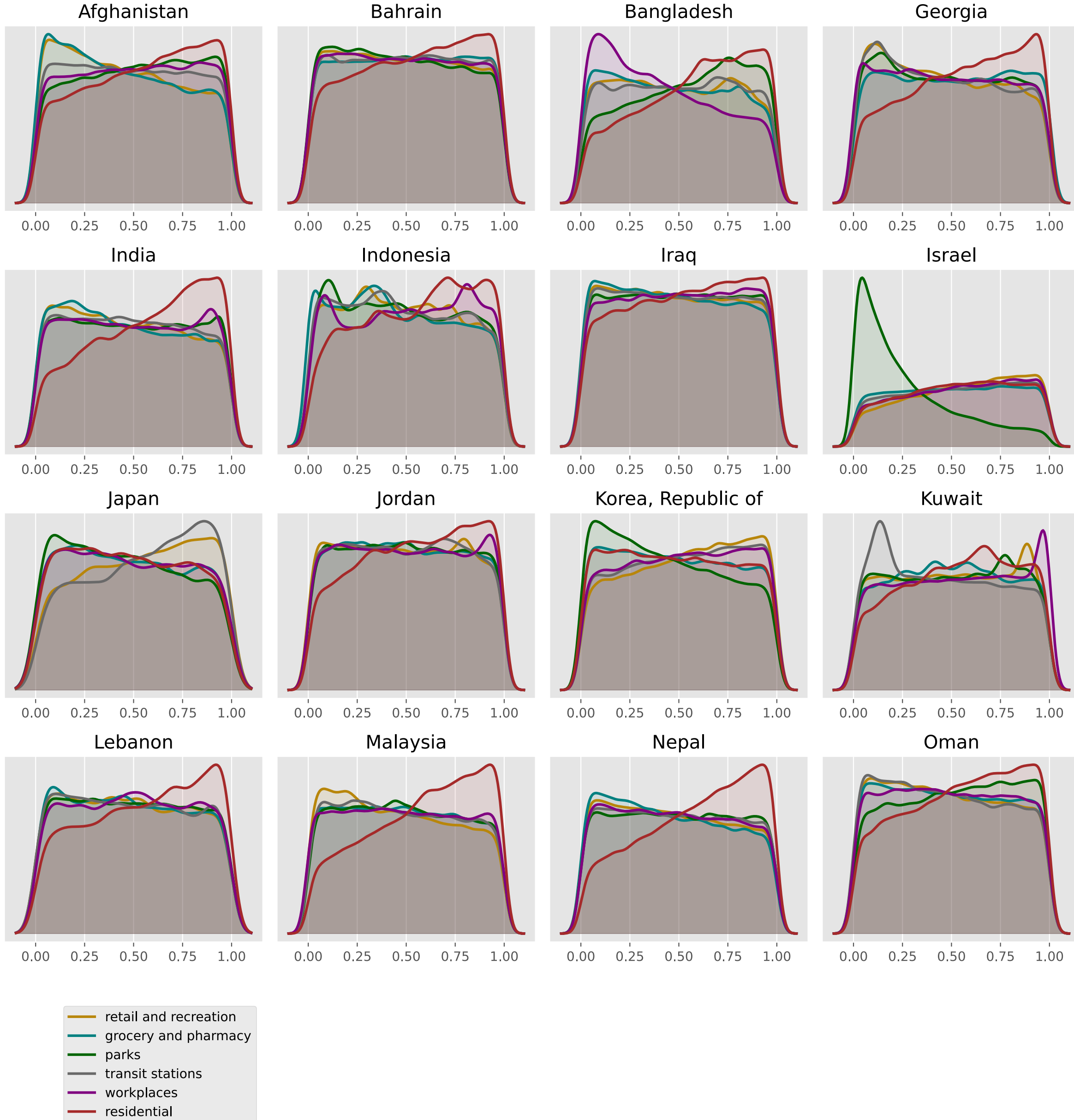
Afghanistan
Bahrain
Bangladesh
Georgia
India
Indonesia
Iraq
Israel
Japan
Jordan
Korea, Republic of
Kuwait
Lebanon
Malaysia
Nepal
Oman
0.00 0.25 0.50 0.75 1.00
retail and recreation
grocery and pharmacy
parks
transit stations
workplaces
residential

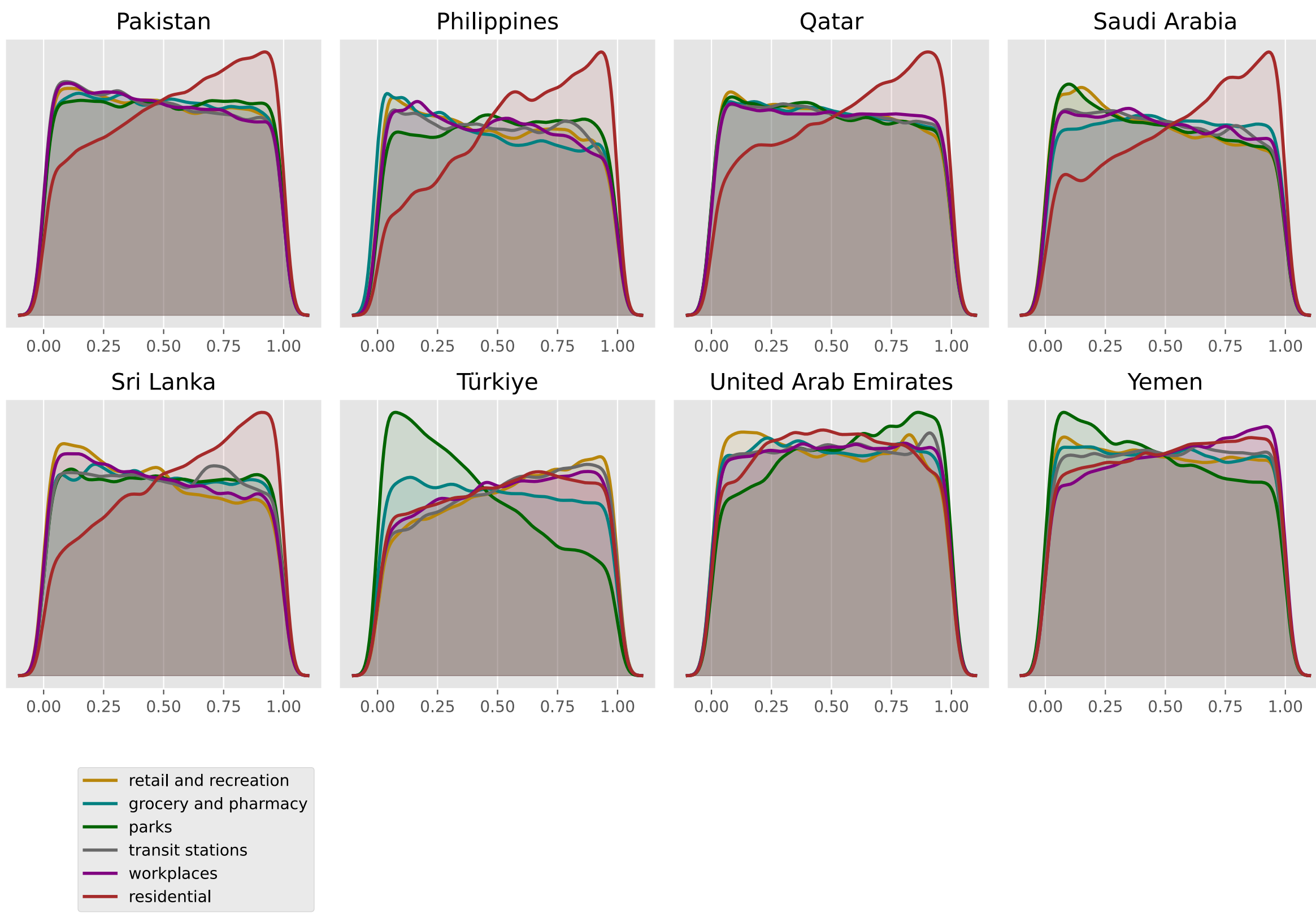
Pakistan
Philippines
Qatar
Saudi Arabia
Sri Lanka
Türkiye
United Arab Emirates
Yemen
0.00
0.25
0.50
0.75
1.00
retail and recreation
grocery and pharmacy
parks
transit stations
workplaces
residential

Europe

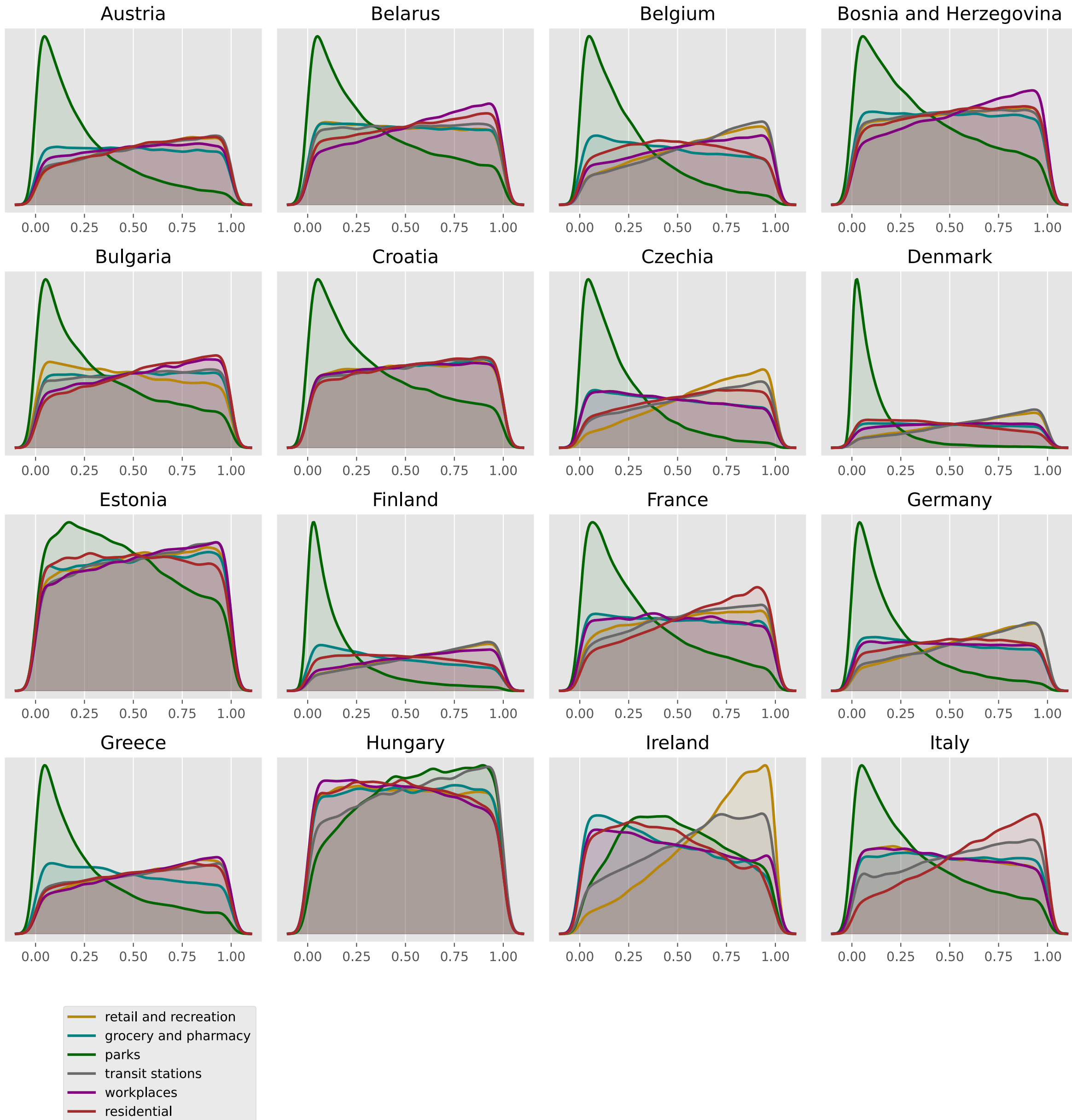
Austria
Belarus
Belgium
Bosnia and Herzegovina
Bulgaria
Croatia
Czechia
Denmark
Estonia
Finland
France
Germany
Greece
Hungary
Ireland
Italy
0.00 0.25 0.50 0.75 1.00
retail and recreation
grocery and pharmacy
parks
transit stations
workplaces
residential

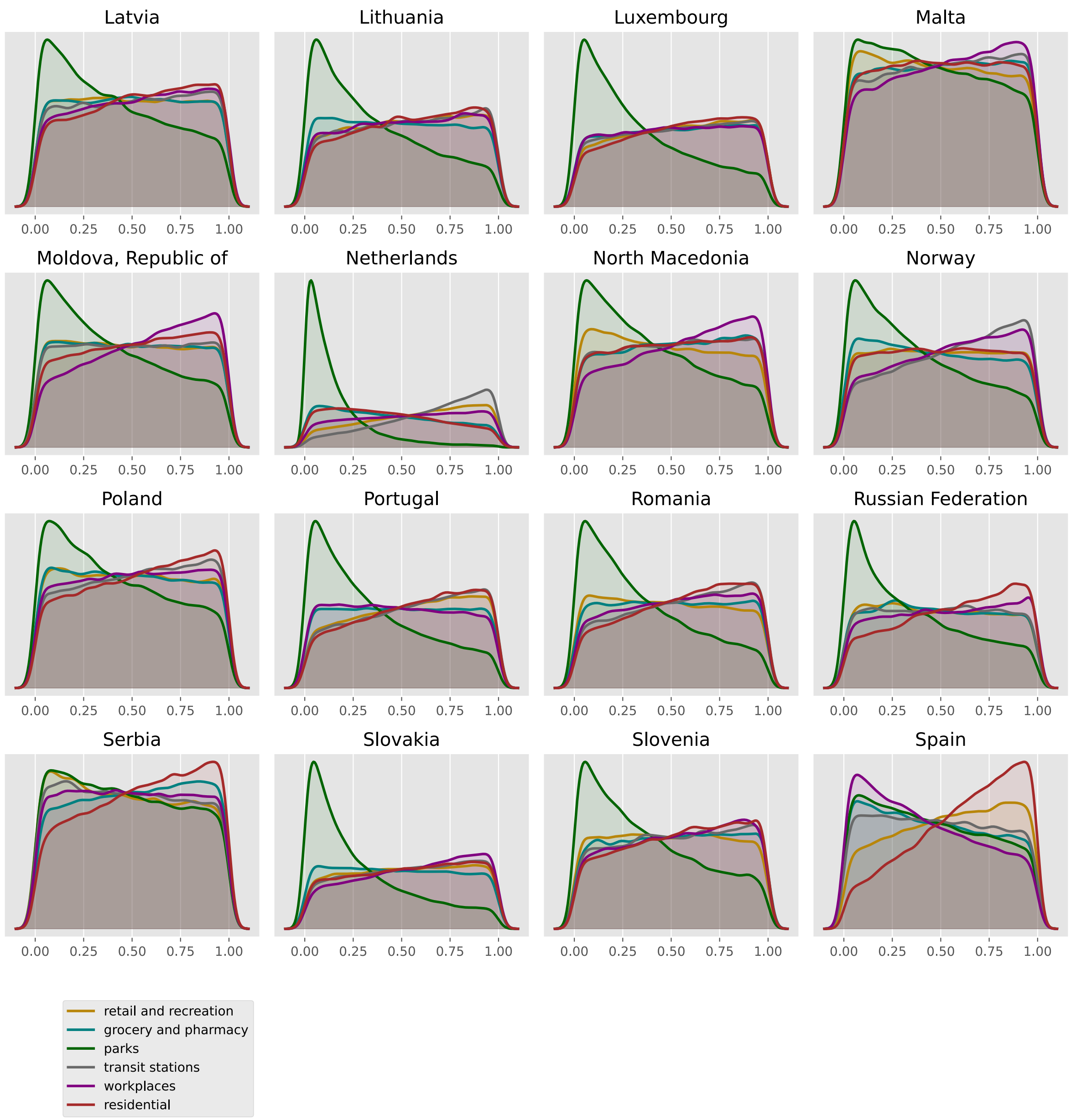
Latvia
Lithuania
Luxembourg
Malta
Moldova, Republic of
Netherlands
North Macedonia
Norway
Poland
Portugal
Romania
Russian Federation
Serbia
Slovakia
Slovenia
Spain
0.00
0.25
0.50
0.75
1.00
retail and recreation
grocery and pharmacy
parks
transit stations
workplaces
residential

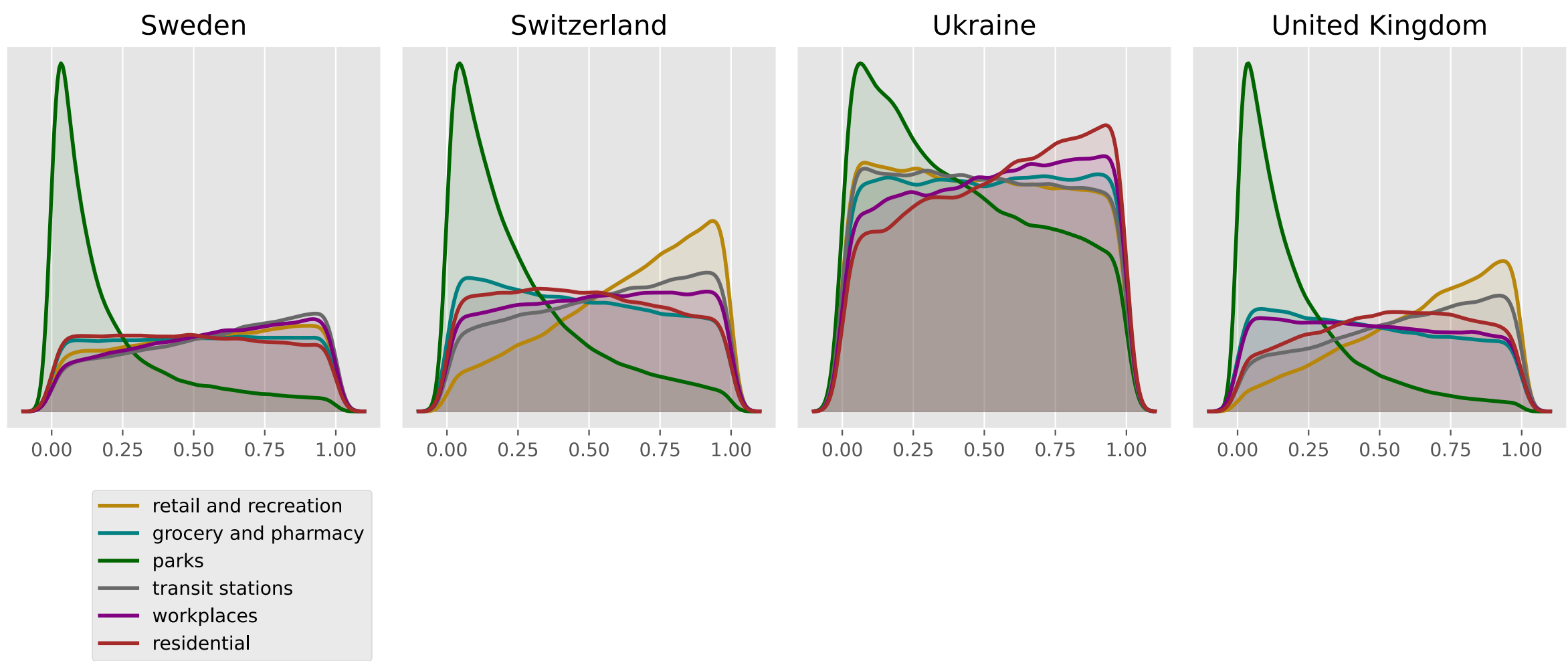
Sweden
Switzerland
Ukraine
United Kingdom
0.00
0.25
0.50
0.75
1.00
retail and recreation
grocery and pharmacy
parks
transit stations
workplaces
residential

North America

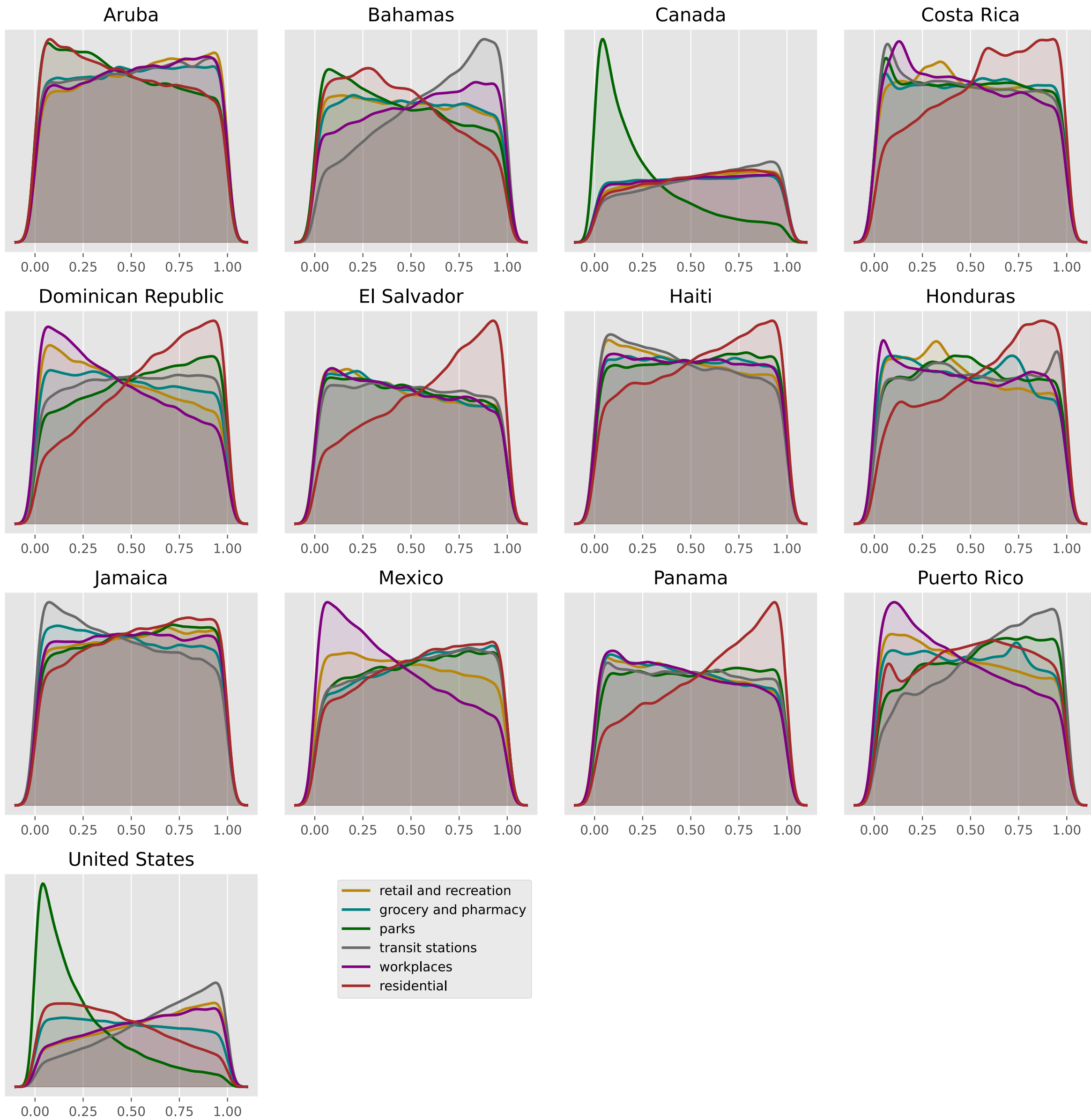

Oceania

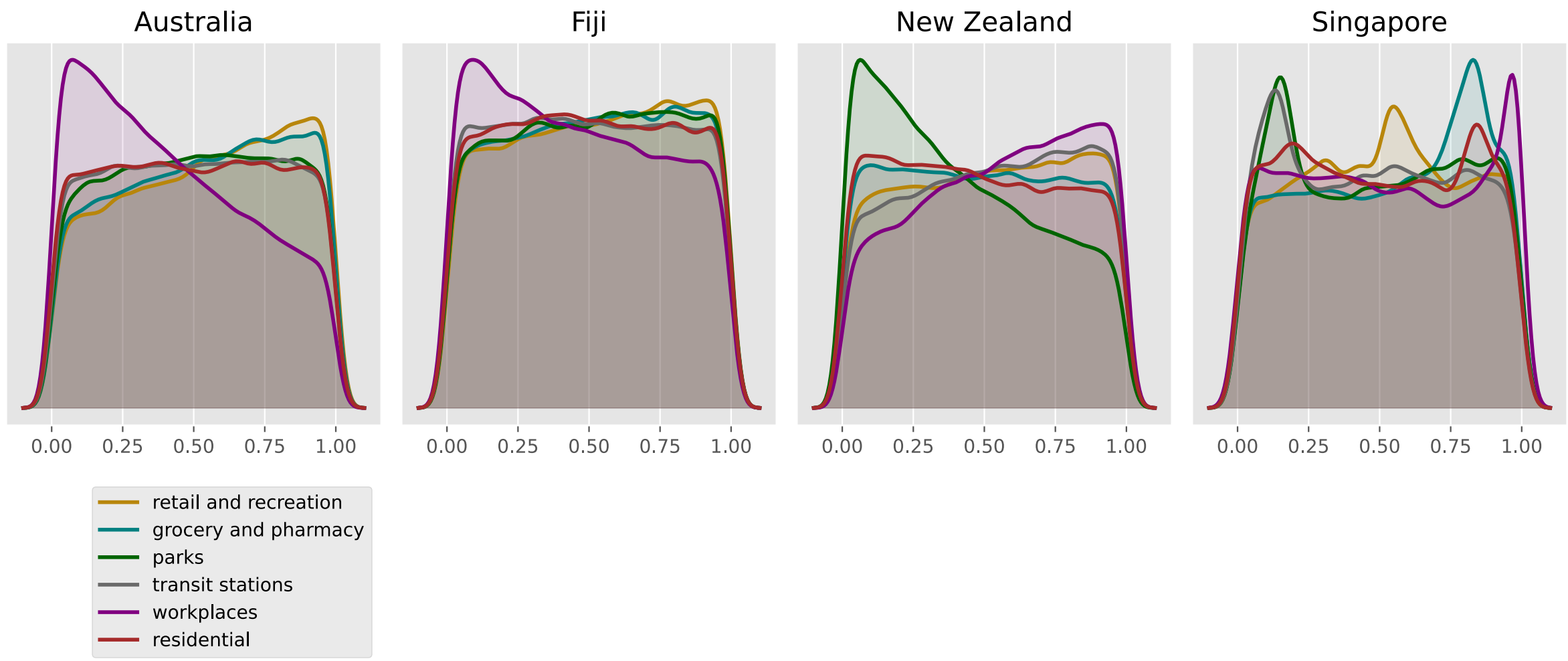
Australia
Fiji
New Zealand
Singapore
0.00
0.25
0.50
0.75
1.00
retail and recreation
grocery and pharmacy
parks
transit stations
workplaces
residential

South America

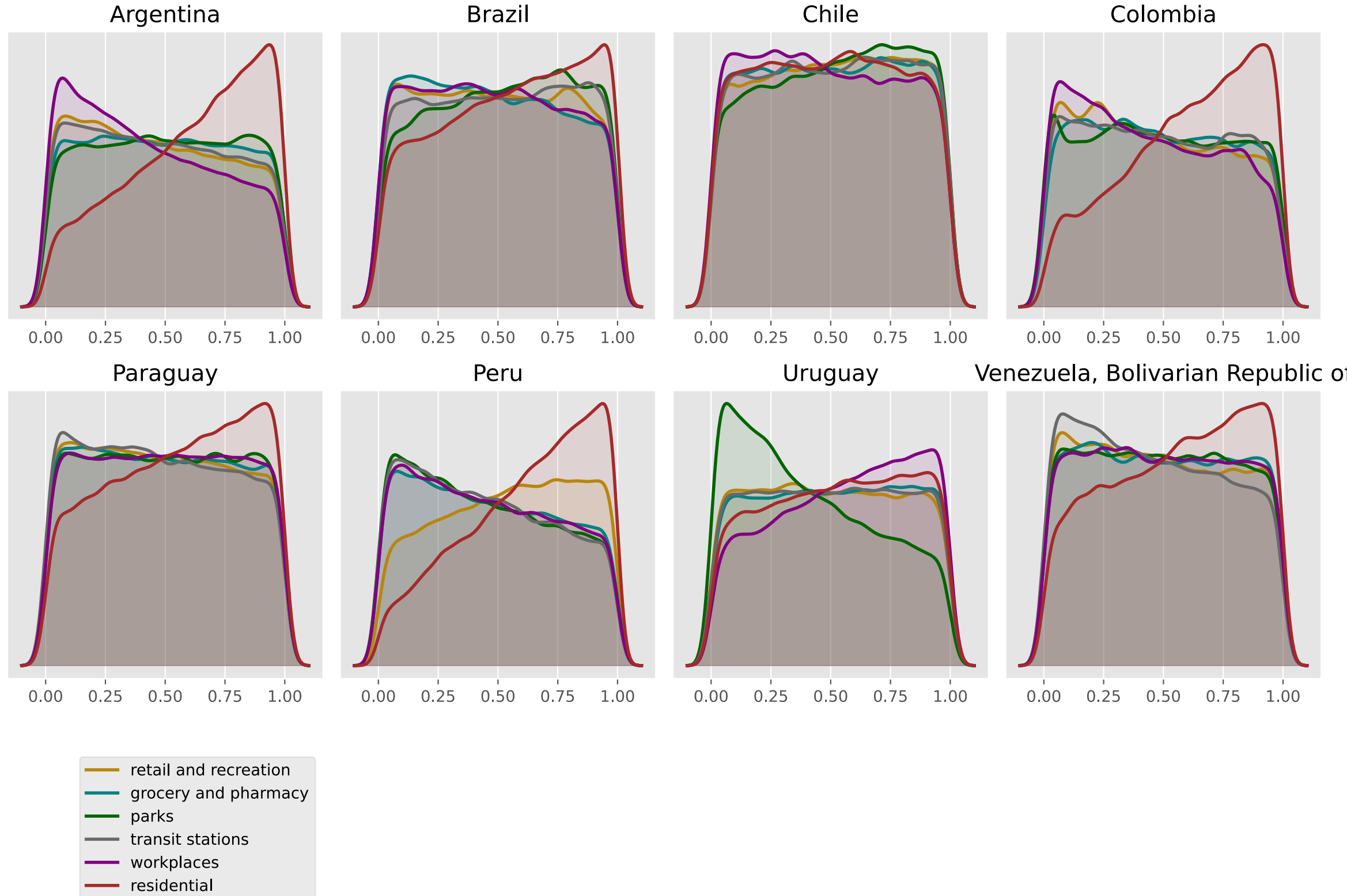

Effect of waning immunity

This section presents comparisons of key model parameters with and without the inclusion of waning immunity.

Example dispersion parameter posterior distributions

The following plots show the posterior distribution of the dispersion to the variable process with and without the incorporation of waning immunity into the model.

These are presented for a random sample of country-analysis combinations sampled from the 463 total analyses, with 12 random analyses were selected for display.

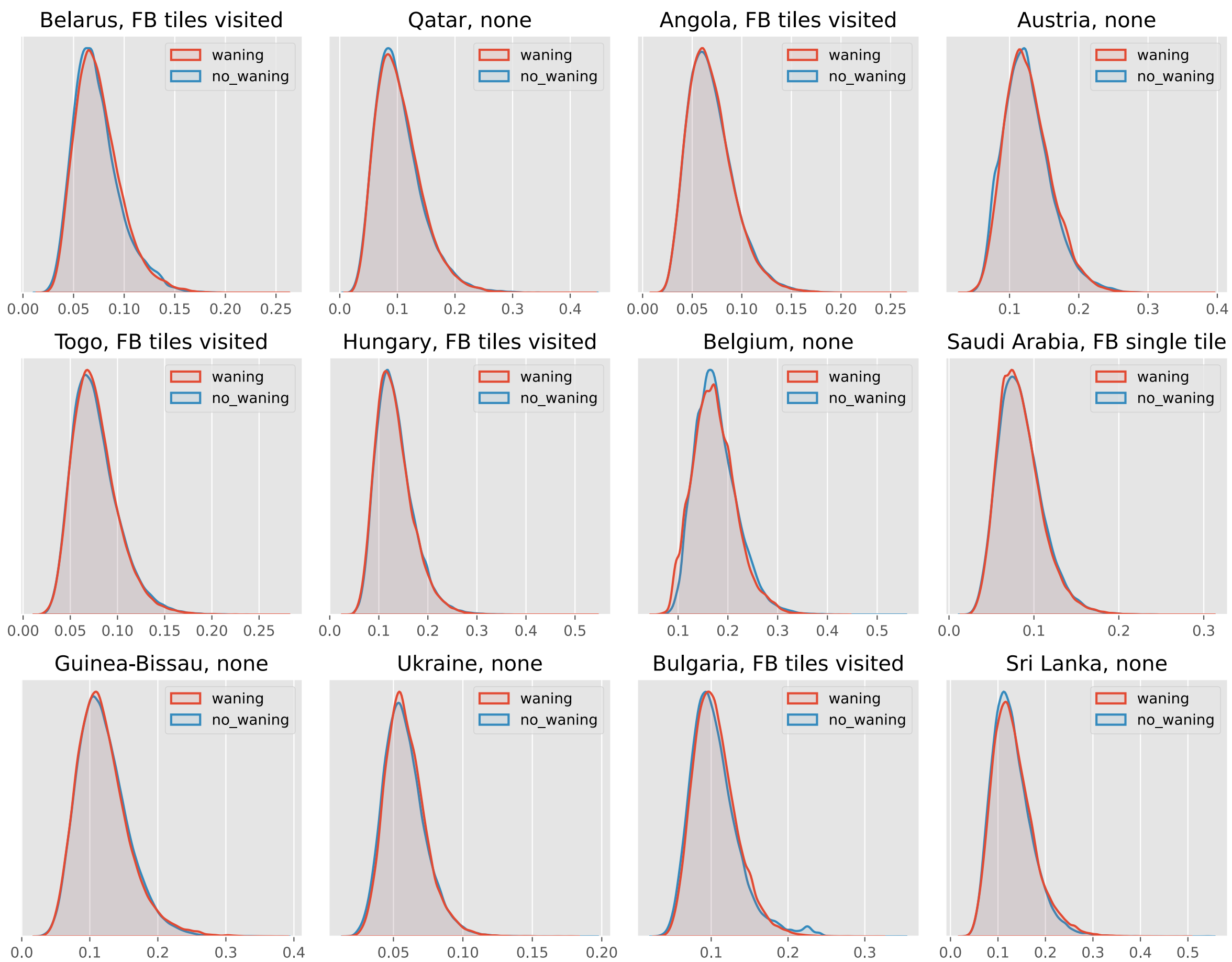

Quantile-quantile posterior comparisons

The following quantile-quantile graph shows the proportion of runs under the analysis that included waning immunity for which the variable process dispersion was greater than under the base case analysis, for the equivalent country-analysis type combination.

Across all country-analysis type combinations, 95.5 per cent of values fell between 0.4 and 0.6.

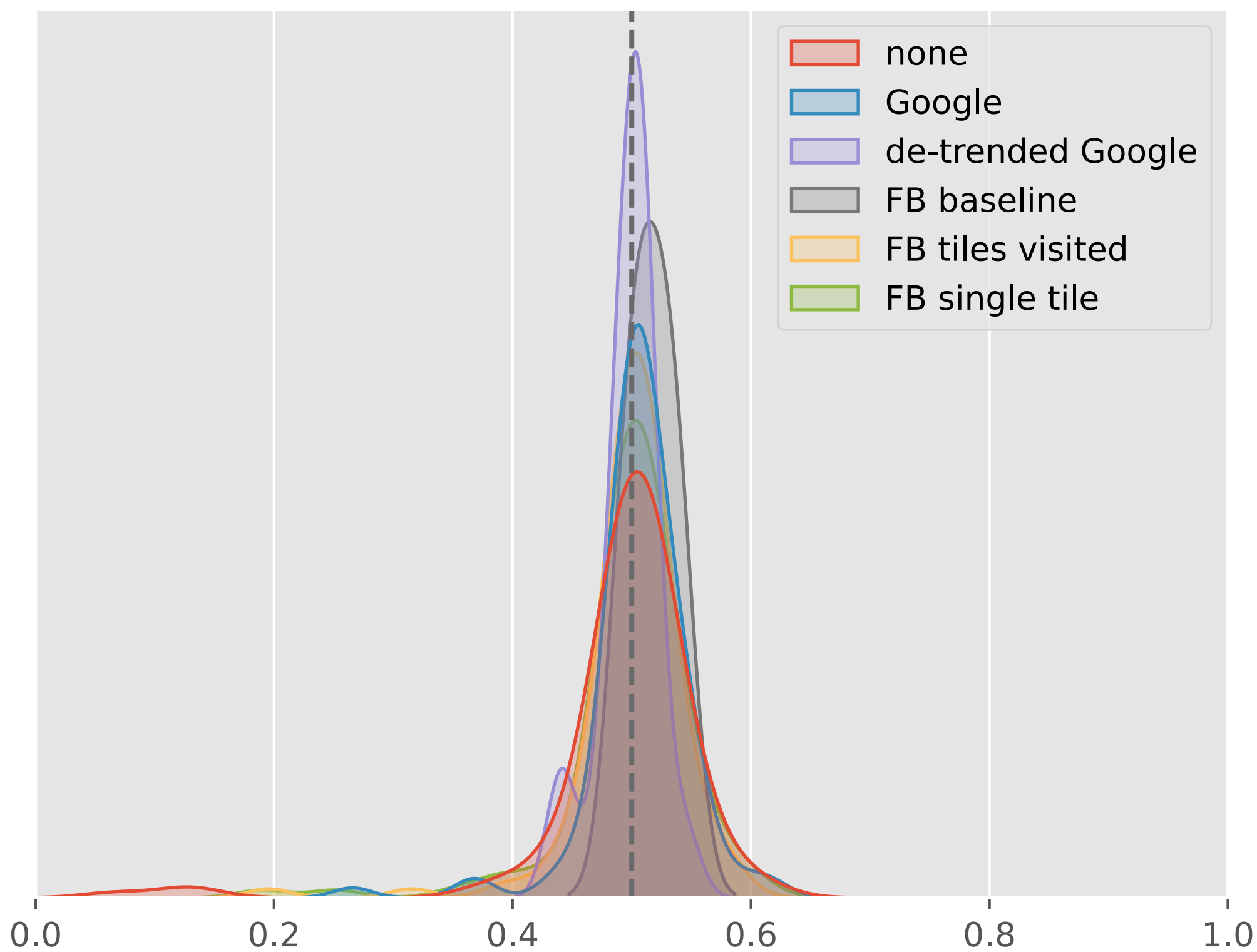

The individual proportions for each country-analysis combination are listed in the following table.

	none	Google	de-trended Google	FB baseline	FB tiles visited	FB single tile
Afghanistan	0.498	0.498	0.48	no analysis	no analysis	no analysis
Albania	0.52	no analysis	no analysis	no analysis	0.539	0.528
Algeria	0.508	no analysis	no analysis	no analysis	0.496	0.486
Angola	0.502	0.497	0.513	no analysis	0.498	0.518
Argentina	0.518	0.546	no analysis	no analysis	0.315	0.536
Aruba	0.515	0.506	no analysis	no analysis	no analysis	no analysis
Australia	0.577	0.54	no analysis	0.516	0.469	0.388
Austria	0.519	0.519	no analysis	no analysis	0.491	0.494
Bahamas	0.532	0.559	no analysis	no analysis	no analysis	no analysis
Bahrain	0.531	0.5	no analysis	no analysis	no analysis	no analysis
Bangladesh	0.507	0.496	0.442	no analysis	0.446	0.444
Belarus	0.545	0.538	no analysis	no analysis	0.535	0.528
Belgium	0.484	0.499	no analysis	no analysis	0.497	0.524
Benin	0.487	0.507	0.503	no analysis	0.509	0.499
Bosnia and Herzegovina	0.512	0.529	no analysis	no analysis	0.517	0.544
Brazil	0.502	0.502	no analysis	no analysis	0.491	0.496
Bulgaria	0.522	0.542	no analysis	no analysis	0.536	0.543
Burkina Faso	0.505	0.487	0.522	no analysis	0.519	0.515

	none	Google	de-trended Google	FB baseline	FB tiles visited	FB single tile
Cabo Verde	0.463	0.56	no analysis	no analysis	no analysis	no analysis
Cameroon	0.514	0.492	0.504	no analysis	0.52	0.514
Canada	0.524	0.513	no analysis	no analysis	0.51	0.515
Chile	0.384	0.484	no analysis	no analysis	0.472	0.522
Colombia	0.462	0.49	no analysis	no analysis	0.475	0.491
Congo	0.5	no analysis	no analysis	no analysis	0.485	0.5
Costa Rica	0.505	0.51	no analysis	no analysis	0.513	0.502
Croatia	0.476	0.496	0.489	no analysis	0.496	0.496
Czechia	0.497	0.443	no analysis	no analysis	0.443	0.474
Côte d'Ivoire	0.527	0.489	0.502	no analysis	0.5	0.492
Denmark	0.515	0.496	0.498	no analysis	0.521	0.519
Dominican Republic	0.555	0.592	no analysis	no analysis	0.555	0.579
Egypt	0.515	0.433	0.5	no analysis	0.48	0.507
El Salvador	0.591	0.572	no analysis	no analysis	0.574	0.562
Equatorial Guinea	0.46	no analysis	no analysis	no analysis	0.497	0.535
Estonia	0.505	0.498	no analysis	no analysis	0.488	0.507
Ethiopia	0.507	no analysis	no analysis	no analysis	0.51	0.526
Fiji	0.507	0.5	no analysis	0.497	0.514	0.502
Finland	0.507	0.498	no analysis	no analysis	0.497	0.508
France	0.455	0.533	no analysis	no analysis	0.485	0.252

	none	Google	de-trended Google	FB baseline	FB tiles visited	FB single tile
French Guiana	0.468	no analysis	no analysis	no analysis	0.538	0.542
Gabon	0.532	0.489	0.507	no analysis	0.521	0.527
Georgia	0.508	0.486	no analysis	no analysis	0.509	0.522
Germany	0.529	0.51	no analysis	no analysis	0.503	0.52
Greece	0.436	0.495	0.492	no analysis	0.498	0.495
Guinea	0.51	no analysis	no analysis	no analysis	0.5	0.48
Guinea-Bissau	0.493	no analysis	no analysis	no analysis	0.503	0.482
Guyana	0.492	no analysis	no analysis	no analysis	0.51	0.513
Haiti	0.549	0.606	no analysis	no analysis	0.576	0.586
Honduras	0.61	0.62	no analysis	no analysis	0.591	0.607
Hungary	0.482	0.471	no analysis	no analysis	0.486	0.484
India	0.513	0.51	no analysis	no analysis	0.52	0.19
Indonesia	0.128	0.371	no analysis	no analysis	0.196	0.556
Iraq	0.511	0.515	0.516	no analysis	0.527	0.507
Ireland	0.446	0.453	no analysis	no analysis	0.533	0.51
Israel	0.067	0.486	no analysis	no analysis	no analysis	no analysis
Italy	0.494	0.522	no analysis	no analysis	0.52	0.424
Jamaica	0.548	0.531	no analysis	no analysis	0.528	0.547
Japan	0.485	0.528	no analysis	no analysis	0.478	0.5
Jordan	0.422	0.47	no analysis	no analysis	0.409	0.411

	none	Google	de-trended Google	FB baseline	FB tiles visited	FB single tile
Kenya	0.486	0.509	no analysis	no analysis	0.448	0.506
Korea, Republic of	0.48	0.501	no analysis	no analysis	0.501	0.492
Kuwait	0.491	0.54	no analysis	no analysis	no analysis	no analysis
Latvia	0.51	0.522	no analysis	no analysis	0.53	0.513
Lebanon	0.411	0.365	no analysis	no analysis	0.39	0.388
Lesotho	0.491	no analysis	no analysis	no analysis	0.499	0.508
Liberia	0.483	no analysis	no analysis	no analysis	0.525	0.499
Libya	0.544	0.539	0.545	no analysis	no analysis	no analysis
Lithuania	0.493	0.489	no analysis	no analysis	0.495	0.462
Luxembourg	0.498	0.504	no analysis	no analysis	0.443	0.488
Madagascar	0.495	no analysis	no analysis	no analysis	0.505	0.503
Malawi	0.605	no analysis	no analysis	no analysis	0.504	0.517
Malaysia	0.491	0.528	no analysis	no analysis	0.487	0.539
Mali	0.49	0.519	0.483	no analysis	0.489	0.498
Malta	0.502	0.496	no analysis	no analysis	0.498	0.496
Martinique	0.547	no analysis	no analysis	no analysis	0.561	0.574
Mexico	0.419	0.522	no analysis	no analysis	0.497	0.482
Moldova, Republic of	0.575	0.556	no analysis	no analysis	0.571	0.556
Morocco	0.521	0.511	no analysis	no analysis	no analysis	no analysis
Mozambique	0.475	0.52	no analysis	no analysis	0.498	0.494

	none	Google	de-trended Google	FB baseline	FB tiles visited	FB single tile
Nepal	0.501	0.504	0.507	no analysis	0.489	0.493
Netherlands	0.496	0.511	no analysis	no analysis	0.514	0.522
New Zealand	0.487	0.521	no analysis	0.537	0.483	0.492
Nigeria	0.504	0.5	no analysis	no analysis	0.526	0.517
North Macedonia	0.532	0.515	no analysis	no analysis	0.541	0.547
Norway	0.517	0.516	no analysis	no analysis	0.499	0.485
Oman	0.496	0.518	no analysis	no analysis	0.491	0.354
Pakistan	0.511	0.504	no analysis	no analysis	no analysis	no analysis
Panama	0.491	0.523	no analysis	no analysis	0.458	0.494
Paraguay	0.529	0.536	no analysis	no analysis	0.525	0.54
Peru	0.374	0.537	no analysis	no analysis	0.473	0.486
Philippines	0.495	0.497	no analysis	no analysis	0.539	0.511
Poland	0.499	0.503	no analysis	no analysis	0.477	0.523
Portugal	0.538	0.523	no analysis	no analysis	0.538	0.536
Puerto Rico	0.477	0.522	no analysis	no analysis	no analysis	no analysis
Qatar	0.514	0.478	no analysis	no analysis	0.505	0.495
Romania	0.503	0.503	no analysis	no analysis	0.506	0.539
Russian Federation	0.553	0.552	no analysis	no analysis	no analysis	no analysis
Rwanda	0.49	0.499	0.441	no analysis	0.478	0.5
Sao Tome and Principe	0.494	no analysis	no analysis	no analysis	0.494	0.493

	none	Google	de-trended Google	FB baseline	FB tiles visited	FB single tile
Saudi Arabia	0.506	0.506	no analysis	no analysis	0.493	0.485
Senegal	0.486	0.493	no analysis	no analysis	0.521	0.485
Serbia	0.502	0.506	no analysis	no analysis	0.519	0.45
Singapore	0.551	0.466	no analysis	no analysis	no analysis	no analysis
Slovakia	0.524	0.546	no analysis	no analysis	0.531	0.542
Slovenia	0.525	0.52	no analysis	no analysis	0.521	0.484
South Africa	0.506	0.481	no analysis	no analysis	0.478	0.47
Spain	0.552	0.491	no analysis	no analysis	0.504	0.469
Sri Lanka	0.527	0.5	no analysis	no analysis	0.502	0.484
Suriname	0.497	no analysis	no analysis	no analysis	0.512	0.513
Sweden	0.543	0.537	no analysis	no analysis	0.538	0.464
Switzerland	0.473	0.513	no analysis	no analysis	0.514	0.5
Togo	0.521	0.509	0.512	no analysis	0.505	0.513
Tunisia	0.46	no analysis	no analysis	no analysis	0.451	0.46
Türkiye	0.507	0.557	no analysis	no analysis	0.517	0.524
Ukraine	0.131	0.266	no analysis	no analysis	no analysis	no analysis
United Arab Emirates	0.48	0.489	no analysis	no analysis	0.517	0.48
United Kingdom	0.474	0.556	no analysis	no analysis	0.565	0.593
United States	0.482	0.475	no analysis	no analysis	0.486	0.478
Uruguay	0.52	0.501	no analysis	no analysis	0.483	0.503

	none	Google	de-trended Google	FB baseline	FB tiles visited	FB single tile
Venezuela, Bolivarian Republic of	0.535	0.535	no analysis	no analysis	no analysis	no analysis
Yemen	0.469	0.512	0.503	no analysis	no analysis	no analysis
Zambia	0.518	0.476	0.485	no analysis	0.504	0.501
Zimbabwe	0.51	0.489	0.51	no analysis	0.501	0.495

Identifiability analysis

The following analyses are intended to show the model's ability to recover key known parameters through its calibration algorithm.

Analysis steps

The steps of this analysis are as follows:

1. One country was selected from the countries included in the main analysis other than those from Oceania (without replacement on iterations)
2. One mobility approach was selected from the types of mobility analysis that were run for that country
3. One accepted parameter set was randomly selected from that country-mobility type calibration
4. Only the targets for deaths and cases were identified from this analysis
5. A calibration was run for that country-mobility type combination, with all parameters fixed at the values selected in Step 3 - except for the variable process and the mobility exponent mapping parameter (if mobility was included), which were calibrated
6. The results were plotted as below The upper panels of figures below compare the calibration fit to data for cases and deaths. The lower-left panel compares the target values for the variable process from the base case analysis (red circles) against the variable process estimates from this re-calibration analysis (black lines). The lower-right panel compares the target value for the mobility exponent mapping parameter (vertical blue bar) against the estimated posterior density for this re-calibration analysis (red area).

Analysis results

Equatorial Guinea, Facebook tiles visited mobility

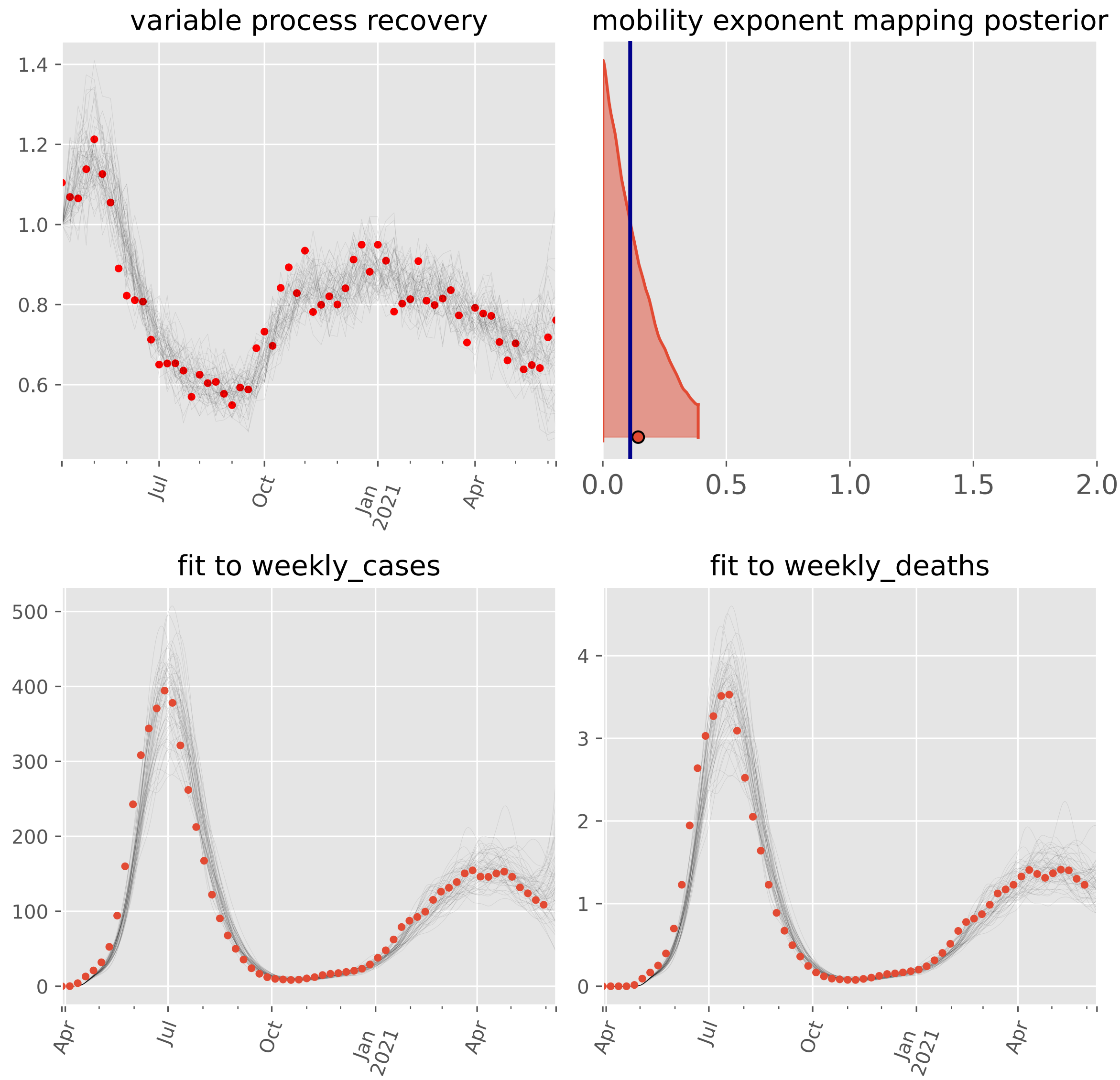

Angola, Facebook single tile mobility

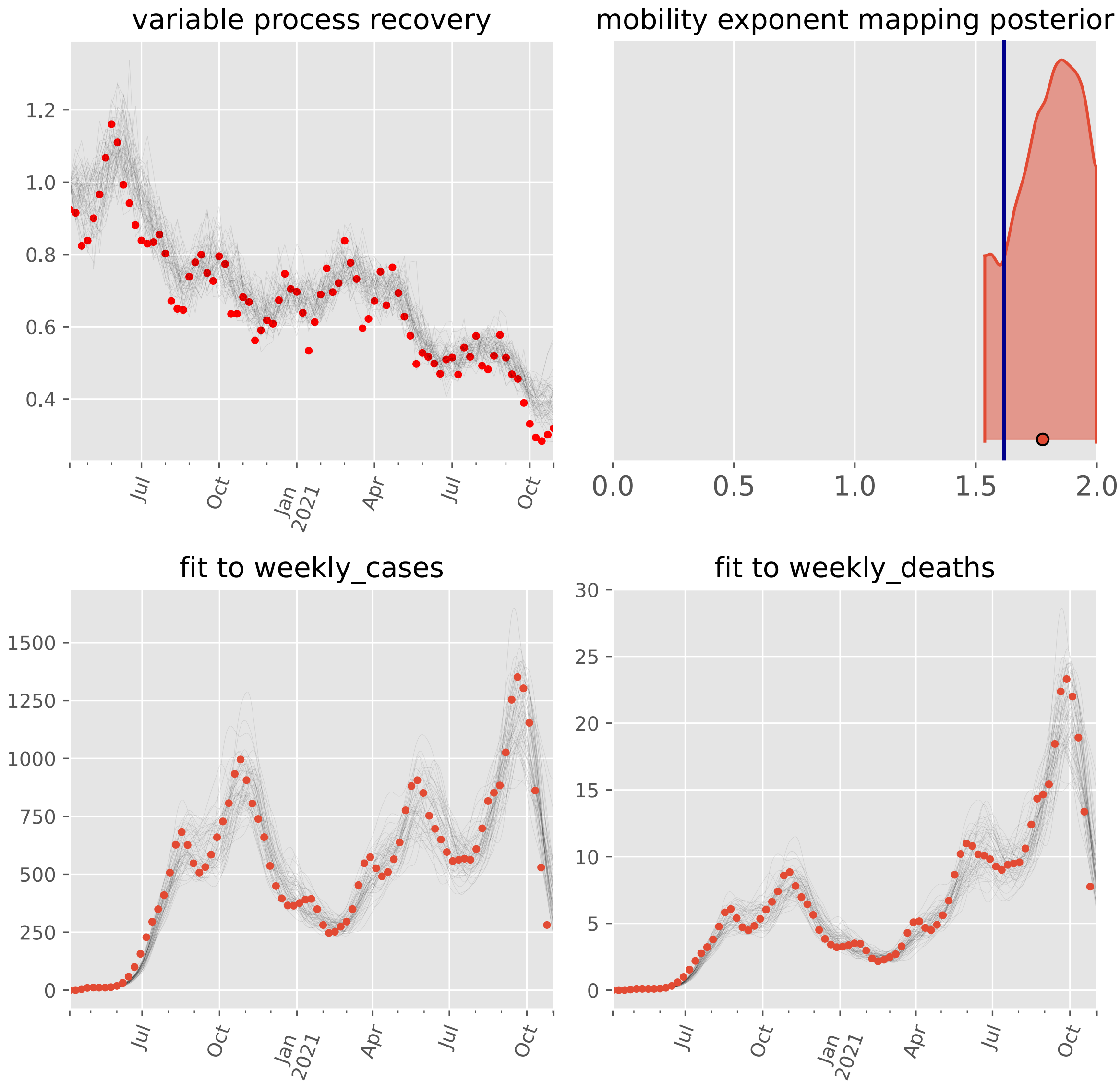

United Kingdom, no mobility

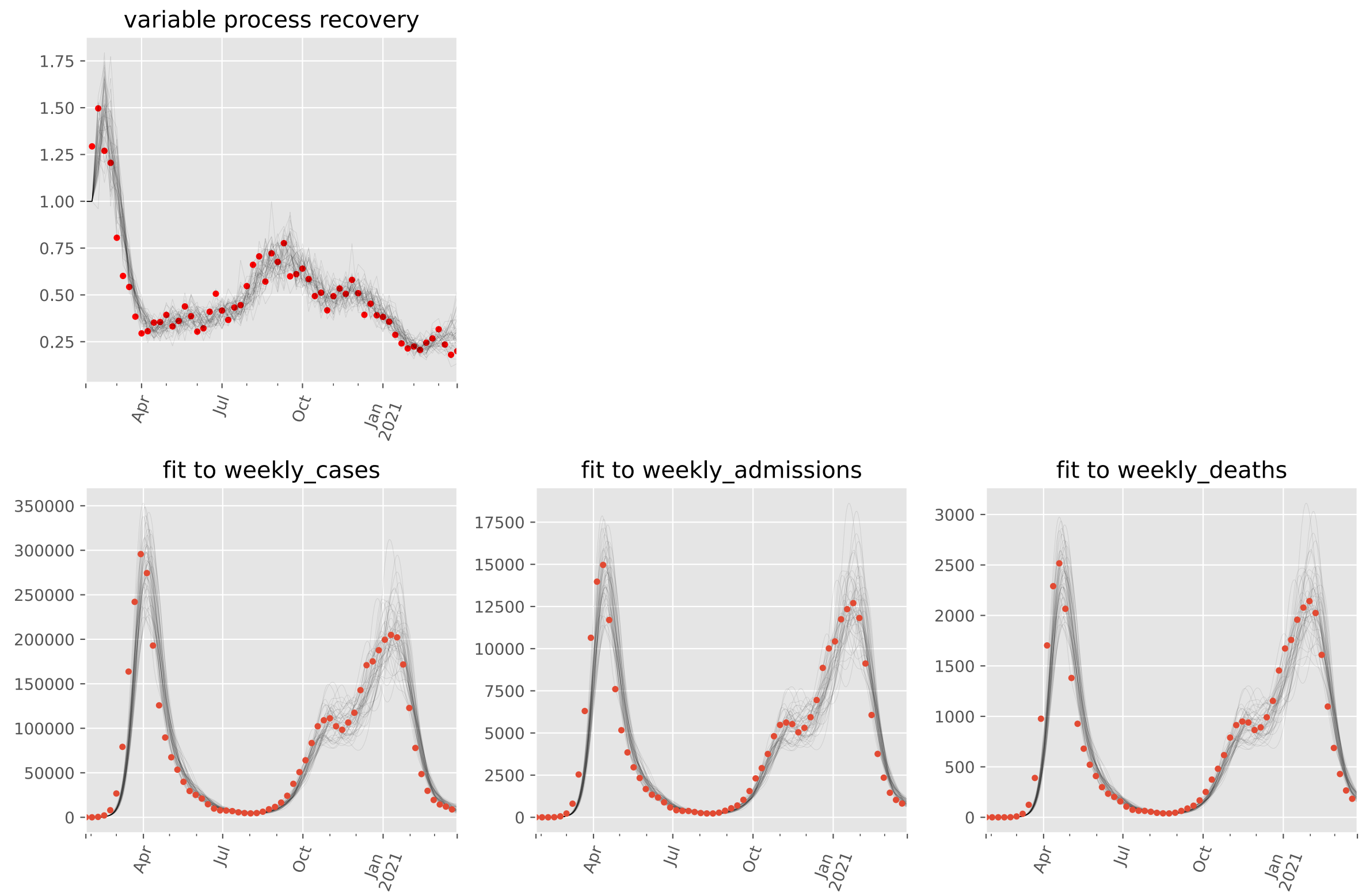

India, Facebook tiles visited mobility

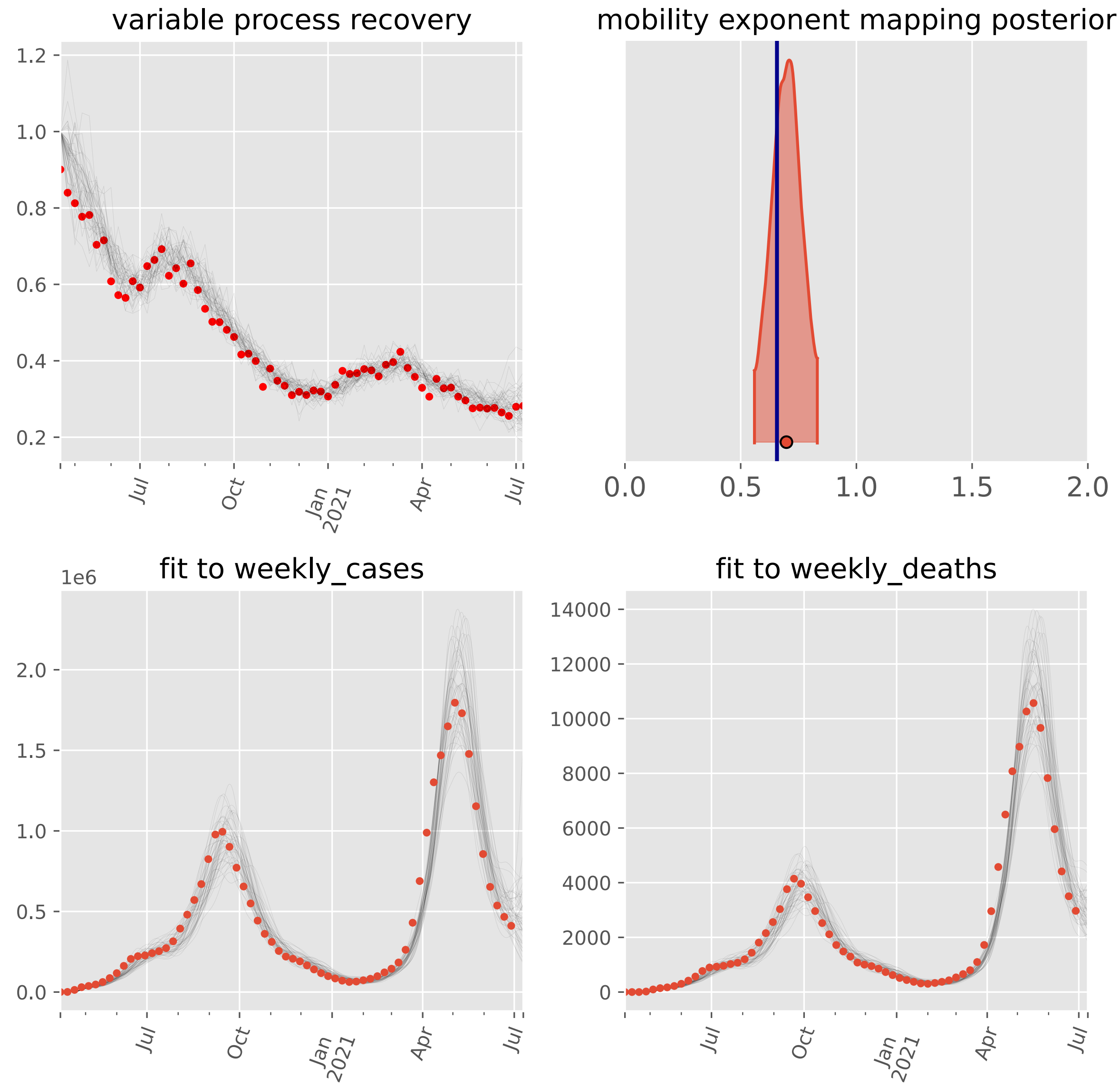

Portugal, no mobility

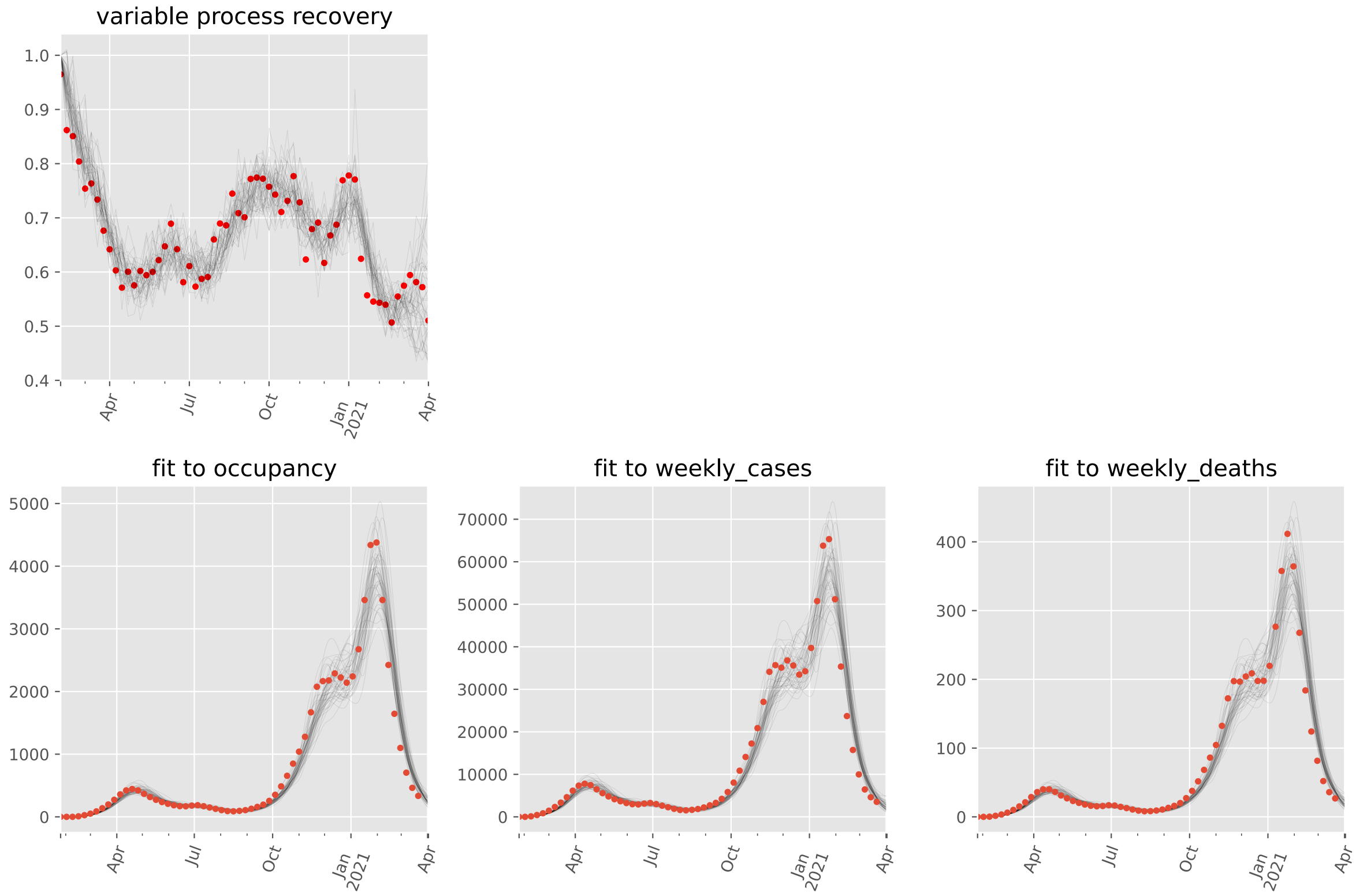

Israel, Google mobility

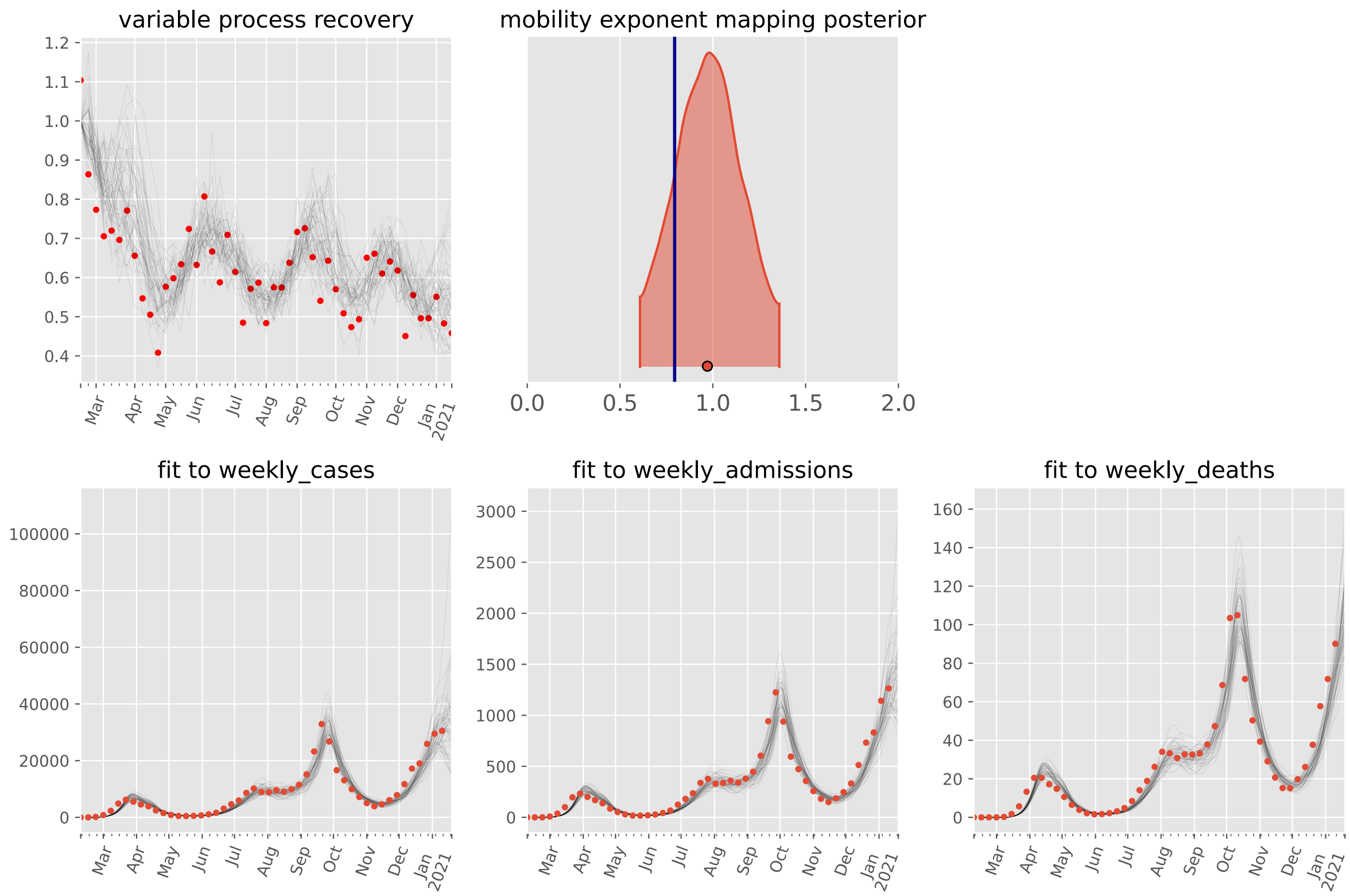

Qatar, Google mobility

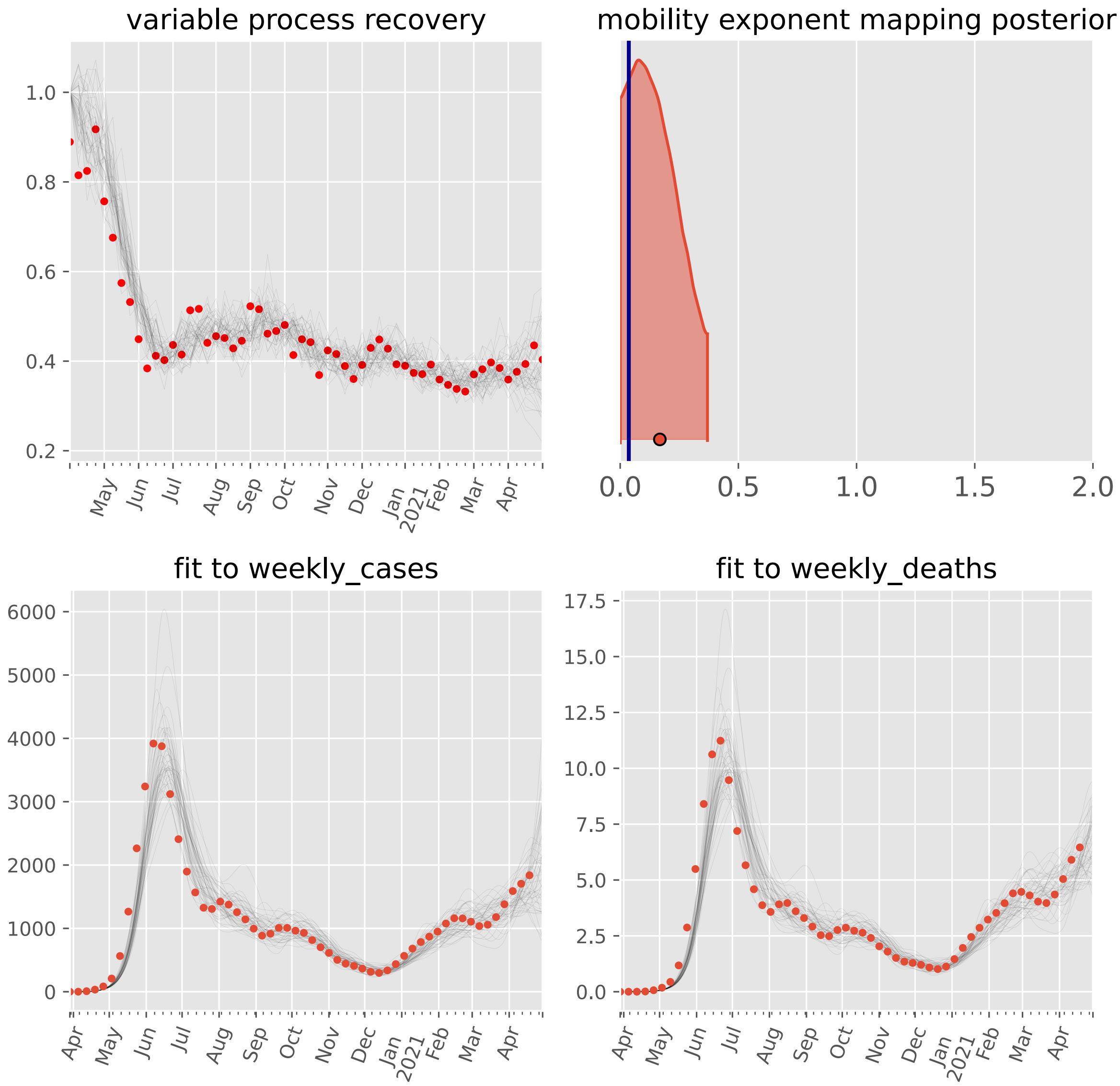

Germany, Facebook single tile mobility

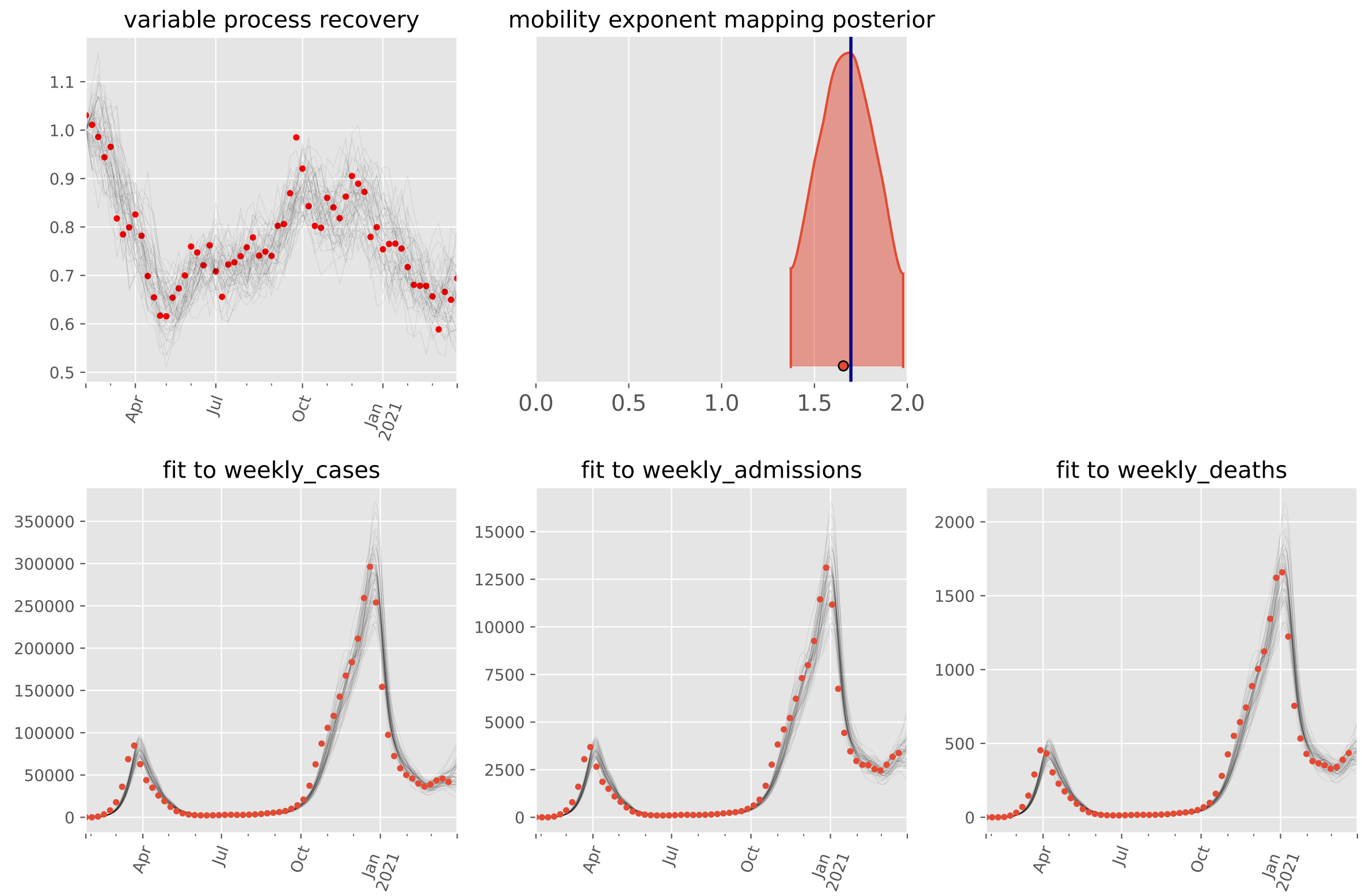

Puerto Rico, no mobility

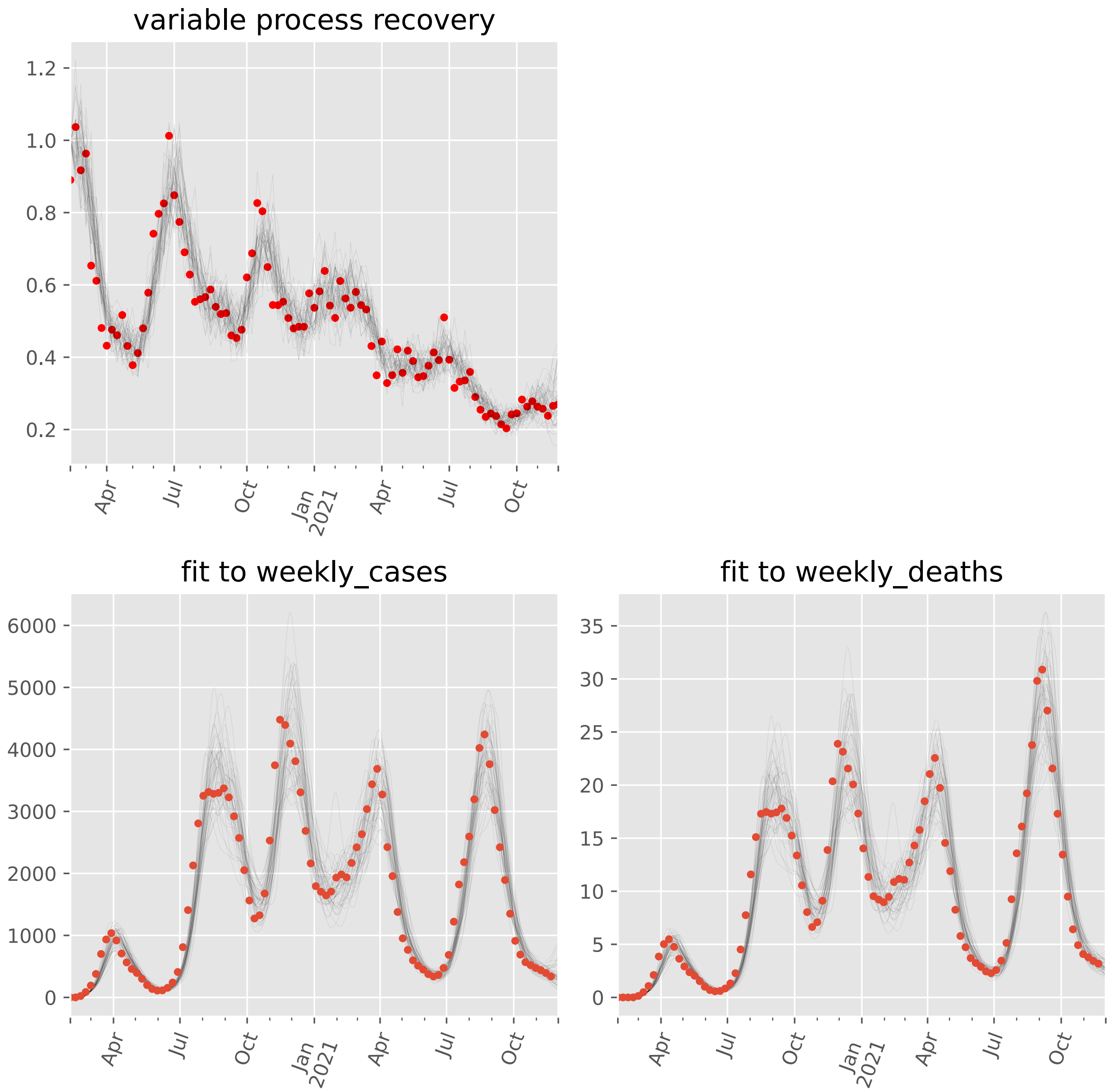

Ireland, Google mobility

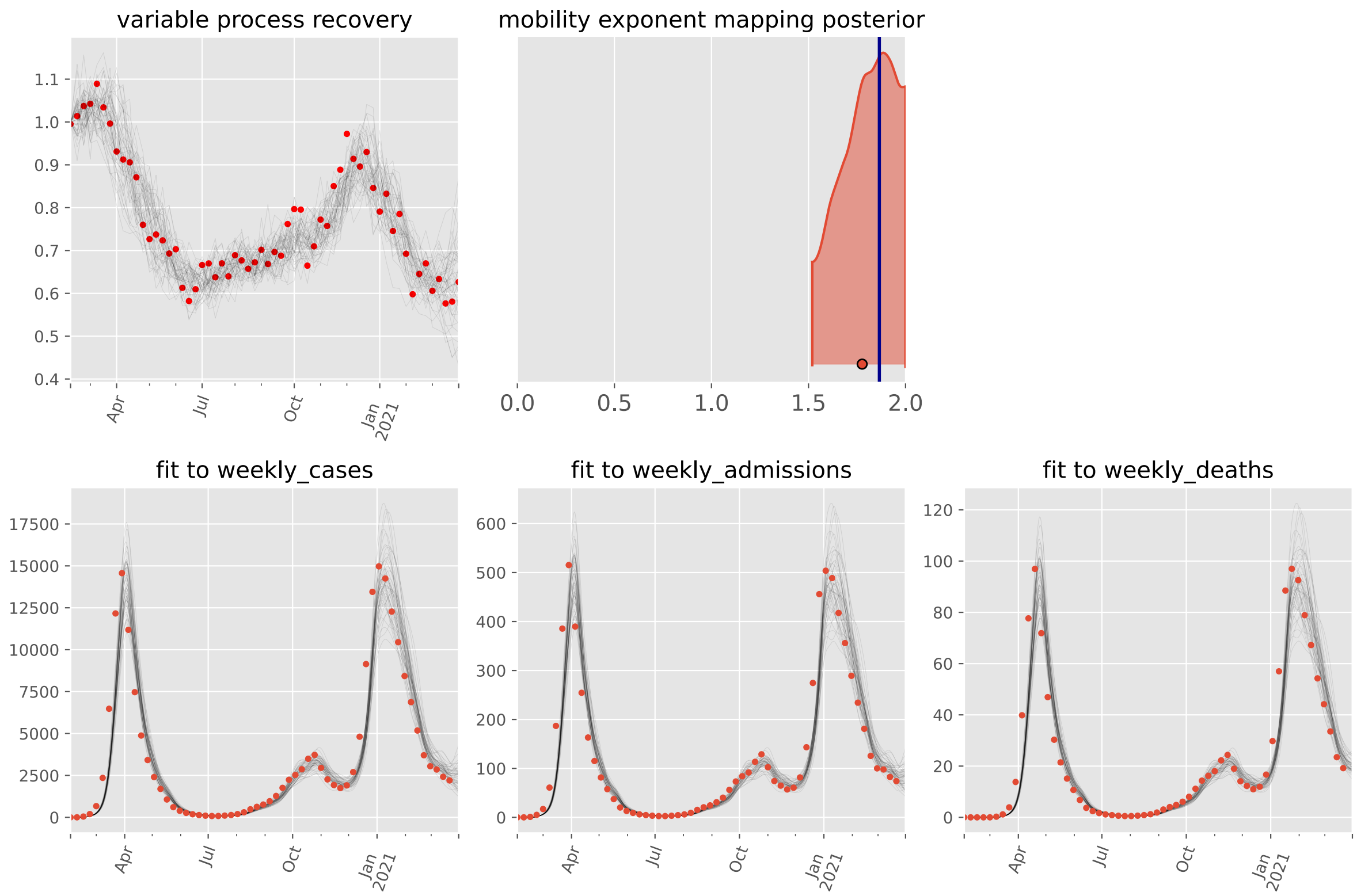

Gabon, Facebook single tile mobility

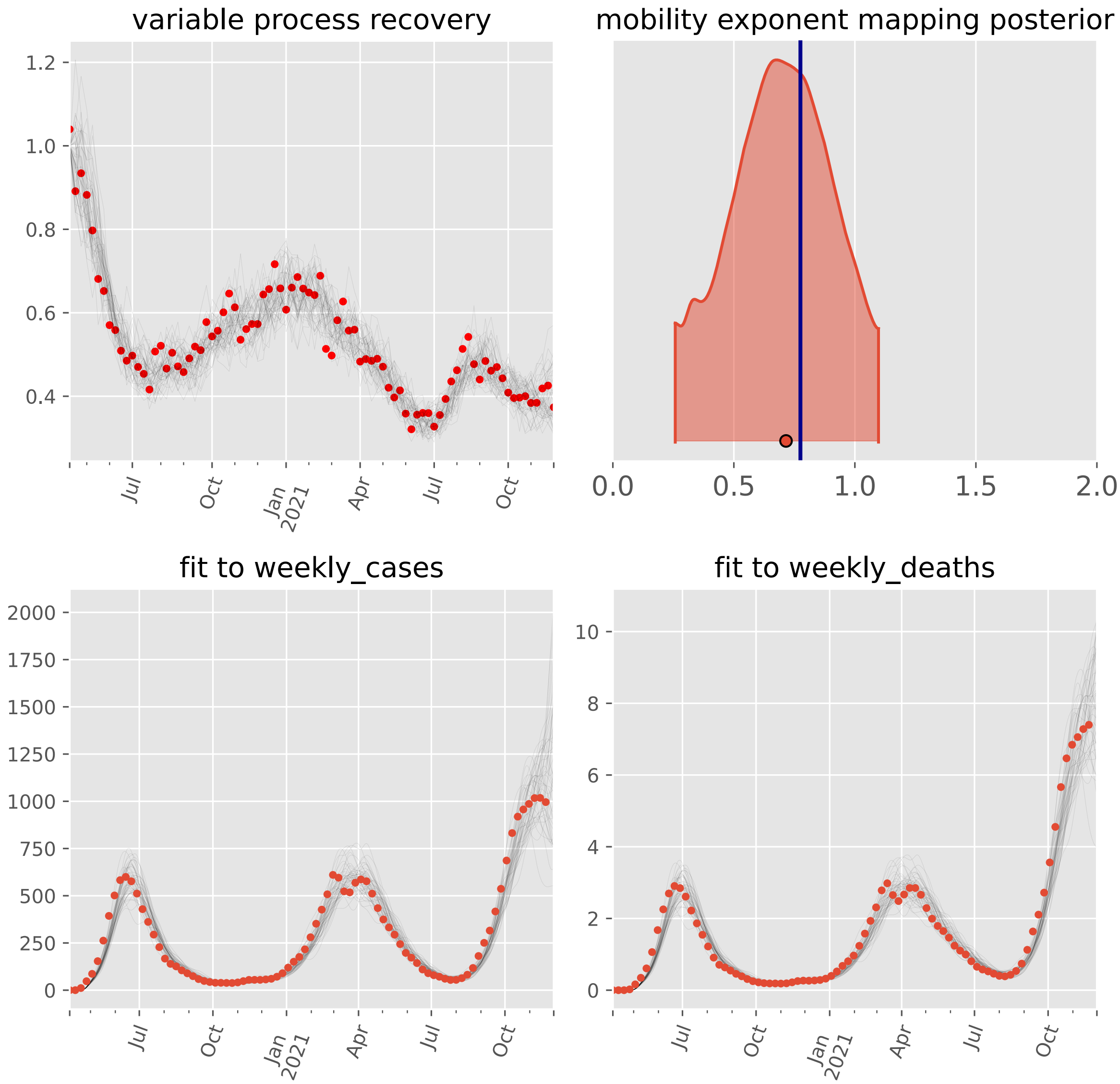

Czechia, Facebook tiles visited mobility

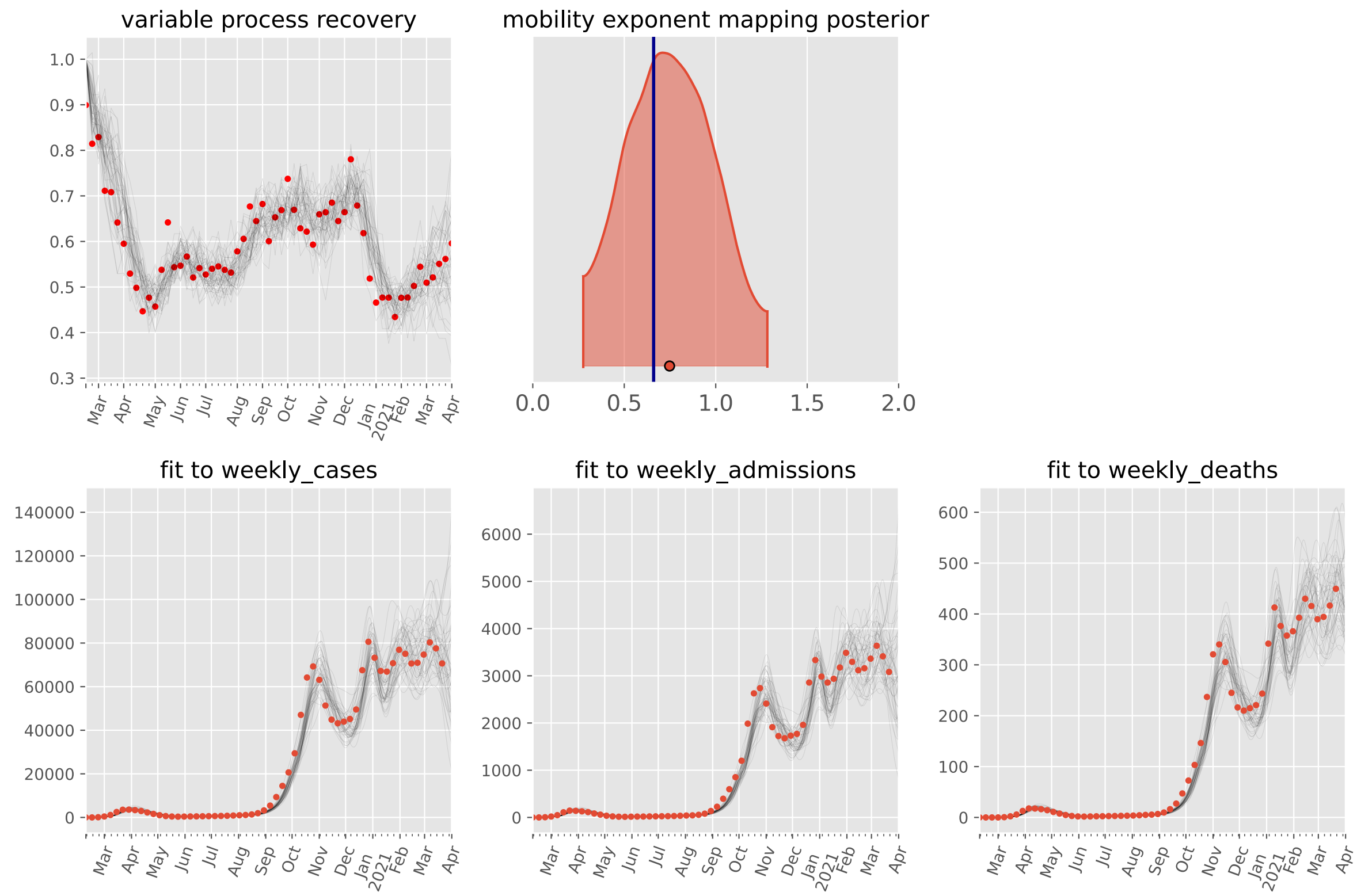

United Arab Emirates, Facebook single tile mobility

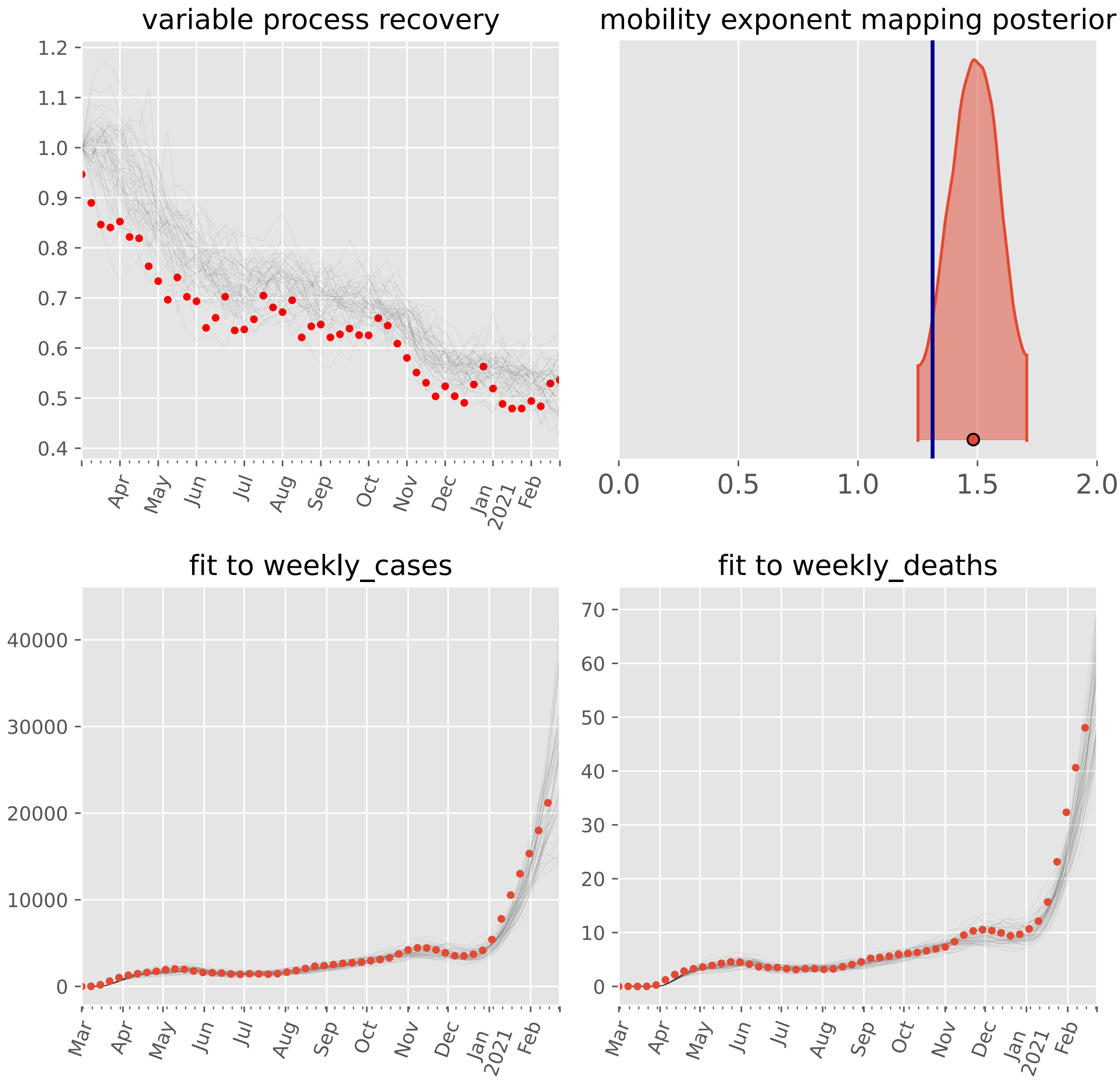

Sri Lanka, Google mobility

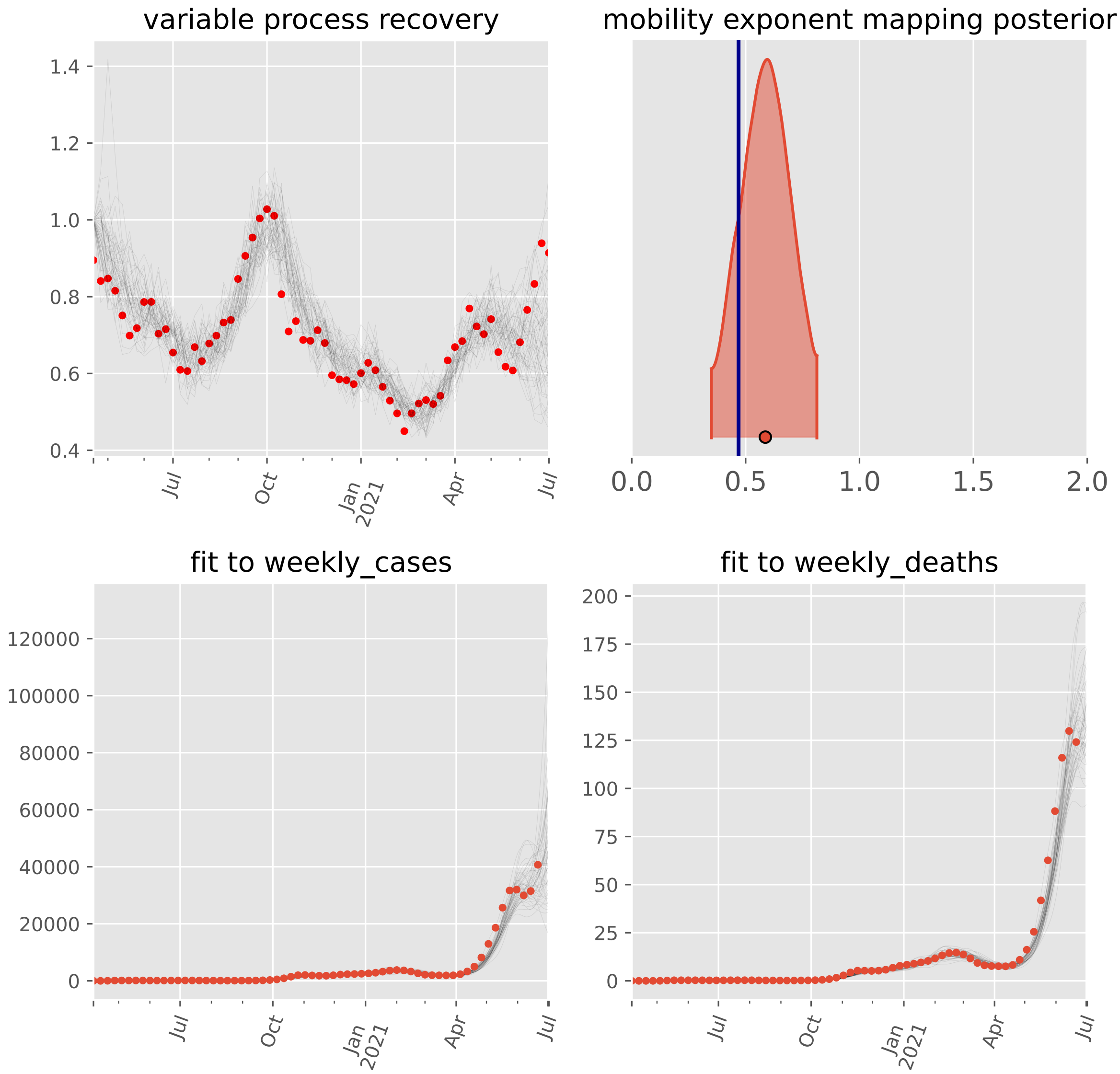

Korea, Republic of, Facebook single tile mobility

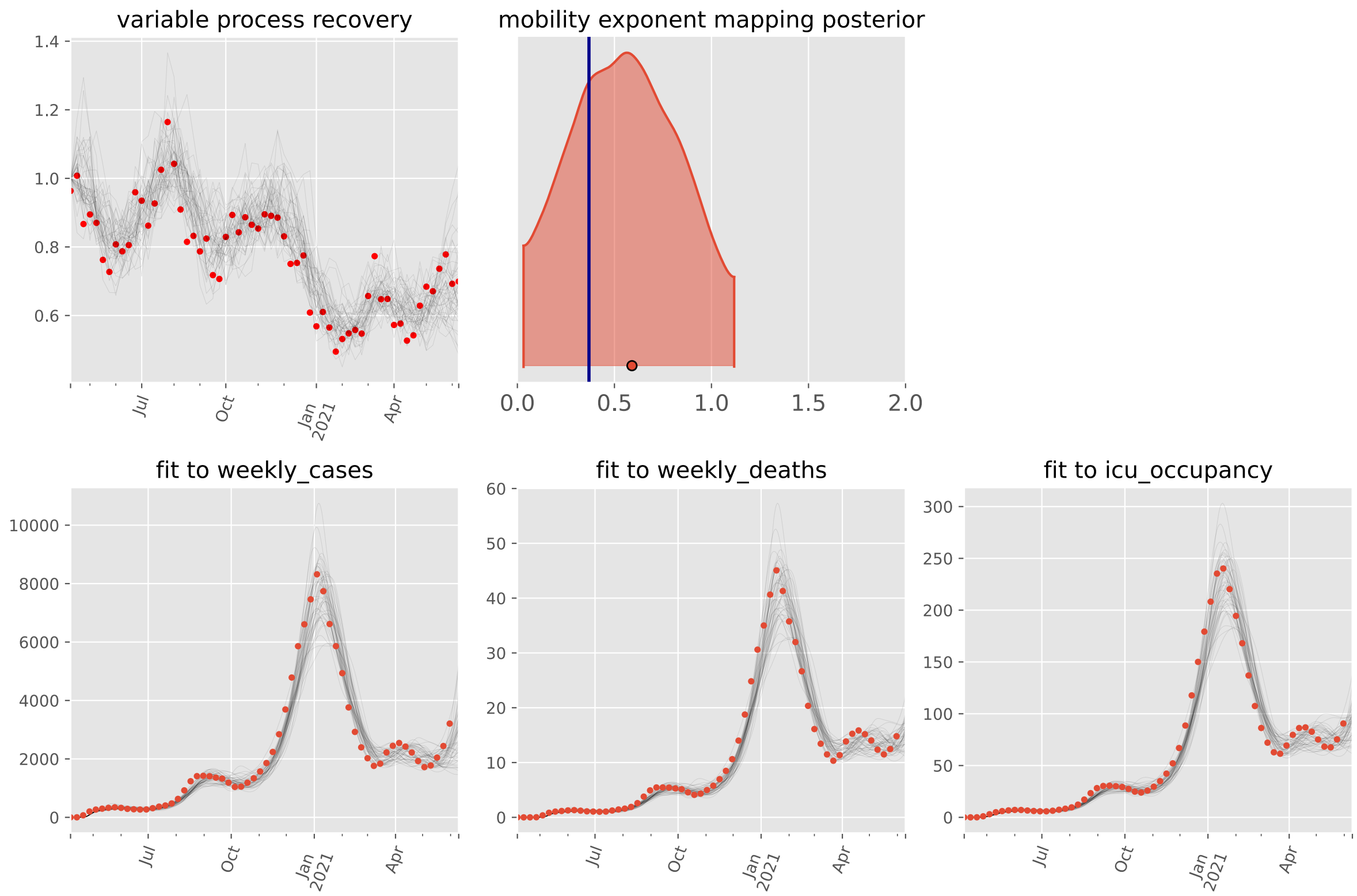

Commits used for analyses

For reproducibility, the following table gives the (short) commit SHA for each analysis.

	no mobility	Google mobility	de-trended Google mobility	Facebook no mobility	Facebook tiles visited mobility	Facebook single tile mobility
Afghanistan	31b7d78	31b7d78	31b7d78	no analysis	no analysis	no analysis
Albania	31b7d78	no analysis	no analysis	no analysis	31b7d78	31b7d78
Algeria	31b7d78	no analysis	no analysis	no analysis	31b7d78	31b7d78
Angola	31b7d78	31b7d78	31b7d78	no analysis	31b7d78	31b7d78
Argentina	31b7d78	31b7d78	no analysis	no analysis	31b7d78	31b7d78
Aruba	31b7d78	31b7d78	no analysis	no analysis	no analysis	no analysis
Australia	31b7d78	31b7d78	no analysis	31b7d78	31b7d78	31b7d78
Austria	31b7d78	31b7d78	no analysis	no analysis	31b7d78	31b7d78
Bahamas	31b7d78	31b7d78	no analysis	no analysis	no analysis	no analysis
Bahrain	31b7d78	31b7d78	no analysis	no analysis	no analysis	no analysis
Bangladesh	31b7d78	31b7d78	17a83c4	no analysis	17a83c4	17a83c4
Belarus	31b7d78	31b7d78	no analysis	no analysis	31b7d78	31b7d78
Belgium	31b7d78	31b7d78	no analysis	no analysis	31b7d78	31b7d78
Benin	31b7d78	31b7d78	31b7d78	no analysis	31b7d78	31b7d78
Bosnia and Herzegovina	31b7d78	31b7d78	no analysis	no analysis	31b7d78	31b7d78
Brazil	31b7d78	31b7d78	no analysis	no analysis	31b7d78	31b7d78
Bulgaria	31b7d78	31b7d78	no analysis	no analysis	31b7d78	31b7d78
Burkina Faso	31b7d78	31b7d78	31b7d78	no analysis	31b7d78	31b7d78

	no mobility	Google mobility	de-trended Google mobility	Facebook no mobility	Facebook tiles visited mobility	Facebook single tile mobility
Cabo Verde	31b7d78	31b7d78	no analysis	no analysis	no analysis	no analysis
Cameroon	31b7d78	31b7d78	31b7d78	no analysis	31b7d78	31b7d78
Canada	31b7d78	31b7d78	no analysis	no analysis	31b7d78	17a83c4
Chile	31b7d78	31b7d78	no analysis	no analysis	4f68504	31b7d78
Colombia	31b7d78	31b7d78	no analysis	no analysis	31b7d78	31b7d78
Congo	31b7d78	no analysis	no analysis	no analysis	31b7d78	31b7d78
Costa Rica	31b7d78	31b7d78	no analysis	no analysis	31b7d78	31b7d78
Croatia	31b7d78	31b7d78	31b7d78	no analysis	31b7d78	31b7d78
Czechia	31b7d78	31b7d78	no analysis	no analysis	31b7d78	31b7d78
Côte d'Ivoire	31b7d78	31b7d78	31b7d78	no analysis	31b7d78	31b7d78
Denmark	31b7d78	31b7d78	31b7d78	no analysis	31b7d78	17a83c4
Dominican Republic	31b7d78	31b7d78	no analysis	no analysis	31b7d78	31b7d78
Egypt	31b7d78	31b7d78	eca5e47	no analysis	31b7d78	17a83c4
El Salvador	31b7d78	31b7d78	no analysis	no analysis	31b7d78	31b7d78
Equatorial Guinea	31b7d78	no analysis	no analysis	no analysis	31b7d78	31b7d78
Estonia	31b7d78	31b7d78	no analysis	no analysis	31b7d78	17a83c4
Ethiopia	31b7d78	no analysis	no analysis	no analysis	31b7d78	31b7d78
Fiji	31b7d78	31b7d78	no analysis	31b7d78	31b7d78	31b7d78
Finland	31b7d78	31b7d78	no analysis	no analysis	31b7d78	31b7d78
France	31b7d78	31b7d78	no analysis	no analysis	31b7d78	31b7d78

	no mobility	Google mobility	de-trended Google mobility	Facebook no mobility	Facebook tiles visited mobility	Facebook single tile mobility
French Guiana	31b7d78	no analysis	no analysis	no analysis	31b7d78	31b7d78
Gabon	31b7d78	31b7d78	31b7d78	no analysis	31b7d78	31b7d78
Georgia	31b7d78	31b7d78	no analysis	no analysis	31b7d78	31b7d78
Germany	31b7d78	31b7d78	no analysis	no analysis	31b7d78	31b7d78
Greece	31b7d78	31b7d78	31b7d78	no analysis	31b7d78	31b7d78
Guinea	31b7d78	no analysis	no analysis	no analysis	31b7d78	31b7d78
Guinea-Bissau	31b7d78	no analysis	no analysis	no analysis	31b7d78	31b7d78
Guyana	31b7d78	no analysis	no analysis	no analysis	31b7d78	31b7d78
Haiti	31b7d78	31b7d78	no analysis	no analysis	31b7d78	31b7d78
Honduras	31b7d78	31b7d78	no analysis	no analysis	31b7d78	31b7d78
Hungary	31b7d78	31b7d78	no analysis	no analysis	31b7d78	31b7d78
India	31b7d78	31b7d78	no analysis	no analysis	31b7d78	31b7d78
Indonesia	31b7d78	31b7d78	no analysis	no analysis	31b7d78	31b7d78
Iraq	31b7d78	31b7d78	31b7d78	no analysis	31b7d78	31b7d78
Ireland	31b7d78	31b7d78	no analysis	no analysis	31b7d78	31b7d78
Israel	31b7d78	31b7d78	no analysis	no analysis	no analysis	no analysis
Italy	31b7d78	31b7d78	no analysis	no analysis	31b7d78	31b7d78
Jamaica	31b7d78	31b7d78	no analysis	no analysis	31b7d78	31b7d78
Japan	31b7d78	17a83c4	no analysis	no analysis	17a83c4	8dc6095

	no mobility	Google mobility	de-trended Google mobility	Facebook no mobility	Facebook tiles visited mobility	Facebook single tile mobility
Jordan	31b7d78	31b7d78	no analysis	no analysis	31b7d78	31b7d78
Kenya	31b7d78	31b7d78	no analysis	no analysis	31b7d78	31b7d78
Korea, Republic of	31b7d78	31b7d78	no analysis	no analysis	31b7d78	31b7d78
Kuwait	31b7d78	31b7d78	no analysis	no analysis	no analysis	no analysis
Latvia	31b7d78	31b7d78	no analysis	no analysis	31b7d78	31b7d78
Lebanon	31b7d78	31b7d78	no analysis	no analysis	31b7d78	31b7d78
Lesotho	31b7d78	no analysis	no analysis	no analysis	31b7d78	31b7d78
Liberia	31b7d78	no analysis	no analysis	no analysis	31b7d78	31b7d78
Libya	31b7d78	31b7d78	31b7d78	no analysis	no analysis	no analysis
Lithuania	31b7d78	31b7d78	no analysis	no analysis	31b7d78	31b7d78
Luxembourg	31b7d78	31b7d78	no analysis	no analysis	31b7d78	31b7d78
Madagascar	31b7d78	no analysis	no analysis	no analysis	31b7d78	31b7d78
Malawi	31b7d78	no analysis	no analysis	no analysis	31b7d78	31b7d78
Malaysia	31b7d78	31b7d78	no analysis	no analysis	31b7d78	31b7d78
Mali	31b7d78	31b7d78	31b7d78	no analysis	31b7d78	31b7d78
Malta	31b7d78	31b7d78	no analysis	no analysis	31b7d78	31b7d78
Martinique	31b7d78	no analysis	no analysis	no analysis	31b7d78	31b7d78
Mexico	31b7d78	31b7d78	no analysis	no analysis	31b7d78	31b7d78
Moldova, Republic of	31b7d78	31b7d78	no analysis	no analysis	31b7d78	31b7d78

	no mobility	Google mobility	de-trended Google mobility	Facebook no mobility	Facebook tiles visited mobility	Facebook single tile mobility
Morocco	31b7d78	31b7d78	no analysis	no analysis	no analysis	no analysis
Mozambique	31b7d78	31b7d78	no analysis	no analysis	31b7d78	31b7d78
Nepal	31b7d78	31b7d78	31b7d78	no analysis	31b7d78	31b7d78
Netherlands	31b7d78	31b7d78	no analysis	no analysis	31b7d78	31b7d78
New Zealand	31b7d78	31b7d78	no analysis	31b7d78	31b7d78	31b7d78
Nigeria	31b7d78	31b7d78	no analysis	no analysis	31b7d78	31b7d78
North Macedonia	31b7d78	31b7d78	no analysis	no analysis	31b7d78	31b7d78
Norway	31b7d78	31b7d78	no analysis	no analysis	31b7d78	31b7d78
Oman	31b7d78	31b7d78	no analysis	no analysis	31b7d78	31b7d78
Pakistan	31b7d78	31b7d78	no analysis	no analysis	no analysis	no analysis
Panama	31b7d78	31b7d78	no analysis	no analysis	31b7d78	31b7d78
Paraguay	31b7d78	31b7d78	no analysis	no analysis	31b7d78	31b7d78
Peru	31b7d78	31b7d78	no analysis	no analysis	31b7d78	31b7d78
Philippines	31b7d78	31b7d78	no analysis	no analysis	31b7d78	31b7d78
Poland	31b7d78	31b7d78	no analysis	no analysis	31b7d78	31b7d78
Portugal	31b7d78	31b7d78	no analysis	no analysis	31b7d78	31b7d78
Puerto Rico	31b7d78	31b7d78	no analysis	no analysis	no analysis	no analysis
Qatar	31b7d78	31b7d78	no analysis	no analysis	31b7d78	31b7d78
Romania	31b7d78	31b7d78	no analysis	no analysis	31b7d78	31b7d78
Russian Federation	31b7d78	31b7d78	no analysis	no analysis	no analysis	no analysis

	no mobility	Google mobility	de-trended Google mobility	Facebook no mobility	Facebook tiles visited mobility	Facebook single tile mobility
Rwanda	31b7d78	31b7d78	31b7d78	no analysis	31b7d78	31b7d78
Sao Tome and Principe	31b7d78	no analysis	no analysis	no analysis	31b7d78	31b7d78
Saudi Arabia	31b7d78	31b7d78	no analysis	no analysis	31b7d78	31b7d78
Senegal	31b7d78	31b7d78	no analysis	no analysis	31b7d78	31b7d78
Serbia	31b7d78	31b7d78	no analysis	no analysis	31b7d78	31b7d78
Singapore	31b7d78	31b7d78	no analysis	no analysis	no analysis	no analysis
Slovakia	31b7d78	31b7d78	no analysis	no analysis	31b7d78	31b7d78
Slovenia	31b7d78	31b7d78	no analysis	no analysis	31b7d78	31b7d78
South Africa	31b7d78	31b7d78	no analysis	no analysis	31b7d78	31b7d78
Spain	31b7d78	31b7d78	no analysis	no analysis	31b7d78	4f68504
Sri Lanka	31b7d78	31b7d78	no analysis	no analysis	31b7d78	31b7d78
Suriname	31b7d78	no analysis	no analysis	no analysis	31b7d78	31b7d78
Sweden	31b7d78	31b7d78	no analysis	no analysis	31b7d78	31b7d78
Switzerland	31b7d78	31b7d78	no analysis	no analysis	31b7d78	31b7d78
Togo	31b7d78	31b7d78	31b7d78	no analysis	31b7d78	31b7d78
Tunisia	31b7d78	no analysis	no analysis	no analysis	31b7d78	31b7d78
Türkiye	31b7d78	31b7d78	no analysis	no analysis	31b7d78	31b7d78
Ukraine	31b7d78	31b7d78	no analysis	no analysis	no analysis	no analysis
United Arab Emirates	31b7d78	31b7d78	no analysis	no analysis	31b7d78	31b7d78

	no mobility	Google mobility	de-trended Google mobility	Facebook no mobility	Facebook tiles visited mobility	Facebook single tile mobility
United Kingdom	31b7d78	31b7d78	no analysis	no analysis	31b7d78	31b7d78
United States	31b7d78	31b7d78	no analysis	no analysis	31b7d78	31b7d78
Uruguay	31b7d78	31b7d78	no analysis	no analysis	31b7d78	31b7d78
Venezuela, Bolivarian Republic of	31b7d78	31b7d78	no analysis	no analysis	no analysis	no analysis
Yemen	31b7d78	31b7d78	31b7d78	no analysis	no analysis	no analysis
Zambia	31b7d78	31b7d78	31b7d78	no analysis	31b7d78	31b7d78
Zimbabwe	31b7d78	31b7d78	31b7d78	no analysis	31b7d78	31b7d78

References

1 An der Heiden M, Buchholz U. Serial interval in households infected with SARS-CoV-2 variant b.1.1.529 (omicron) is even shorter compared to delta. *Epidemiol Infect* 2022; **150**: e165.

2 Chen D, Lau Y-C, Xu X-K, *et al.* Inferring time-varying generation time, serial interval, and incubation period distributions for COVID-19. *Nature Communications* 2022; **13**: 7727.

3 Ganyani T, Kremer C, Chen D, *et al.* Estimating the generation interval for coronavirus disease (COVID-19) based on symptom onset data, march 2020. *Euro Surveill* 2020; **25**. DOI:10.2807/1560-7917.Es.2020.25.17.2000257.

4 Del Águila-Mejía J, Wallmann R, Calvo-Montes J, Rodríguez-Lozano J, Valle-Madrazo T, Aginagalde-Llorente A. Secondary attack rate, transmission and incubation periods, and serial interval of SARS-CoV-2 omicron variant, spain. *Emerg Infect Dis* 2022; **28**: 1224–8.

5 He X, Lau EHY, Wu P, *et al.* Temporal dynamics in viral shedding and transmissibility of COVID-19. *Nat Med* 2020; **26**: 672–5.

6 Xu X, Wu Y, Kummer AG, *et al.* Assessing changes in incubation period, serial interval, and generation time of SARS-CoV-2 variants of concern: A systematic review and meta-analysis. *BMC Med* 2023; **21**: 374.

7 Madewell ZJ, Yang Y, Longini Jr I. M., Halloran ME, Vespignani A, Dean NE. Rapid review and meta-analysis of serial intervals for SARS-CoV-2 delta and omicron variants. *BMC Infect Dis* 2023; **23**: 429.

8 Xin H, Wang Z, Feng S, *et al.* Transmission dynamics of SARS-CoV-2 omicron variant infections in hangzhou, zhejiang, china, january-february 2022. *Int J Infect Dis* 2023; **126**: 132–5.

9 Khalili M, Karamouzian M, Nasiri N, Javadi S, Mirzazadeh A, Sharifi H. Epidemiological characteristics of COVID-19: A systematic review and meta-analysis. *Epidemiol Infect* 2020; **148**: e130.

10 Li Q, Guan X, Wu P, *et al.* Early transmission dynamics in wuhan, china, of novel coronavirus-infected pneumonia. *N Engl J Med* 2020; **382**: 1199–207.

11 Golding N, Price DJ, Ryan G, McVernon J, McCaw JM, Shearer FM. A modelling approach to estimate the transmissibility of SARS-CoV-2 during periods of high, low, and zero case incidence. *Elife* 2023; **12**. DOI:10.7554/eLife.78089.

12 Tenforde MW, Kim SS, Lindsell CJ, *et al.* Symptom duration and risk factors for delayed return to usual health among outpatients with COVID-19 in a multistate health care systems network - united states, march-june 2020. *MMWR Morb Mortal Wkly Rep* 2020; **69**: 993–8.

13 Harris JE. Timely epidemic monitoring in the presence of reporting delays: Anticipating the COVID-19 surge in new york city, september 2020. *BMC Public Health* 2022; **22**: 871.

14 Organization WH. Report of the WHO-china joint mission on coronavirus disease 2019 (COVID-19). World Health Organization, 2020.

15 Trauer JM, Hughes AE, Shipman DS, *et al.* A data science pipeline applied to australia's 2022 COVID-19 omicron waves. *Infect Dis Model* 2025; **10**: 99–109.

16 Lee HR, Choe YJ, Jang EJ, *et al.* Time from exposure to diagnosis among quarantined close contacts of SARS-CoV-2 omicron variant index case-patients, south korea. *Emerg Infect Dis* 2022; **28**: 901–3.

17 Lobay R, Srivastava A, Tibshirani RJ, McDonald DJ. Incident COVID-19 infections before omicron in the u.s. *Epidemics* 2025; **52**: 100838.

18 Verity R, Okell LC, Dorigatti I, *et al.* Estimates of the severity of coronavirus disease 2019: A model-based analysis. *Lancet Infect Dis* 2020; **20**: 669–77.

19 Zhang B, Zhou X, Qiu Y, *et al.* Clinical characteristics of 82 cases of death from COVID-19. *PLoS One* 2020; **15**: e0235458.

20 Chen T, Wu D, Chen H, *et al.* Clinical characteristics of 113 deceased patients with coronavirus disease 2019: Retrospective study. *BMJ* 2020; **368**: m1091.

21 Sousa GJB, Garces TS, Cestari VRF, Florêncio RS, Moreira TMM, Pereira MLD. Mortality and survival of COVID-19. *Epidemiol Infect* 2020; **148**: e123.

22 Marschner IC. Estimating age-specific COVID-19 fatality risk and time to death by comparing population diagnosis and death patterns: Australian data. *BMC Med Res Methodol* 2021; **21**: 126.

23 Huang C, Wang Y, Li X, *et al.* Clinical features of patients infected with 2019 novel coronavirus in wuhan, china. *Lancet* 2020; **395**: 497–506.

24 Gentilotti E, Savoldi A, Compri M, *et al.* Assessment of COVID-19 progression on day 5 from symptoms onset. *BMC Infect Dis* 2021; **21**: 883.

25 Faes C, Abrams S, Van Beckhoven D, Meyfroidt G, Vlieghe E, Hens N. Time between symptom onset, hospitalisation and recovery or death: Statistical analysis of belgian COVID-19 patients. *Int J Environ Res Public Health* 2020; **17**. DOI:10.3390/ijerph17207560.

26 Group ICC, Baillie JK, Baruch J, *et al.* ISARIC COVID-19 clinical data report: Final report january 2020 – january 2023. *medRxiv* 2023; : 2020.07.17.20155218.

27 Finelli L, Gupta V, Petigara T, Yu K, Bauer KA, Puzniak LA. Mortality among US patients hospitalized with SARS-CoV-2 infection in 2020. *JAMA Netw Open* 2021; **4**: e216556.

28 Rees EM, Nightingale ES, Jafari Y, *et al.* COVID-19 length of hospital stay: A systematic review and data synthesis. *BMC Med* 2020; **18**: 270.

29 Byrne AW, McEvoy D, Collins AB, *et al.* Inferred duration of infectious period of SARS-CoV-2: Rapid scoping review and analysis of available evidence for asymptomatic and symptomatic COVID-19 cases. *BMJ Open* 2020; **10**: e039856.

30 Alimohamadi Y, Yekta EM, Sepandi M, Sharafoddin M, Arshadi M, Hesari E. Hospital length of stay for COVID-19 patients: A systematic review and meta-analysis. *Multidiscip Respir Med* 2022; **17**: 856.

31 Yang X, Yu Y, Xu J, *et al.* Clinical course and outcomes of critically ill patients with SARS-CoV-2 pneumonia in wuhan, china: A single-centered, retrospective, observational study. *Lancet Respir Med* 2020; **8**: 475–81.

32 Zhou F, Yu T, Du R, *et al.* Clinical course and risk factors for mortality of adult inpatients with COVID-19 in wuhan, china: A retrospective cohort study. *Lancet* 2020; **395**: 1054–62.

33 Souza WM de, Buss LF, Candido DDS, *et al.* Epidemiological and clinical characteristics of the COVID-19 epidemic in brazil. *Nat Hum Behav* 2020; **4**: 856–65.

34 Pullano G, Di Domenico L, Sabbatini CE, *et al.* Underdetection of cases of COVID-19 in france threatens epidemic control. *Nature* 2021; **590**: 134–9.

35 Russell TW, Golding N, Hellewell J, *et al.* Reconstructing the early global dynamics of under-ascertained COVID-19 cases and infections. *BMC Med* 2020; **18**: 332.

36 Security Agency U health. Estimating the proportion of COVID-19 cases detected by testing during the pandemic: September 2020 to march 2022. +, 2024 https://assets.publishing.service.gov.uk/media/65fc251965ca2f001b7da75d/estimating-the-proportion-of-COVID-19-cases-detected-by-testing-during-the-pandemic.pdf.

37 Impouma B, Mboussou F, Shahpar C, *et al.* Estimating the SARS-CoV2 infections detection rate and cumulative incidence in the world health organization african region 10 months into the pandemic. *Epidemiol Infect* 2021; **149**: e264.

38 Reynolds SL, Kaufman HW, Meyer WA, *et al.* Duration of protection against SARS-CoV-2 reinfection and associated risk of reinfection assessed with real-world data. *medRxiv* 2022. DOI:10.1101/2022.02.25.22271515.

39 Past SARS-CoV-2 infection protection against re-infection: A systematic review and meta-analysis. *Lancet* 2023; **401**: 833–42.

40 Hu WH, Cai HL, Yan HC, *et al.* Protective effectiveness of previous infection against subsequent SARS-cov-2 infection: Systematic review and meta-analysis. *Front Public Health* 2024; **12**: 1353415.

41 Bobrovitz N, Ware H, Ma X, *et al.* Protective effectiveness of previous SARS-CoV-2 infection and hybrid immunity against the omicron variant and severe disease: A systematic review and meta-regression. *Lancet Infect Dis* 2023; **23**: 556–67.

42 Ward T, Fyles M, Glaser A, Paton RS, Ferguson W, Overton CE. The real-time infection hospitalisation and fatality risk across the COVID-19 pandemic in england. *Nat Commun* 2024; **15**: 4633.

43 Menachemi N, Dixon BE, Wools-Kaloustian KK, Yiannoutsos CT, Halverson PK. How many SARS-CoV-2-infected people require hospitalization? Using random sample testing to better inform preparedness efforts. *J Public Health Manag Pract* 2021; **27**: 246–50.

44 Deng Y, Kim Y, Bratcher A, *et al.* Ratio of infections to COVID-19 cases and hospitalizations in the united states based on SARS-CoV-2 seroprevalence data, september 2021-february 2022. *Open Forum Infect Dis* 2025; **12**: ofae719.

45 O'Driscoll M, Ribeiro Dos Santos G, Wang L, *et al.* Age-specific mortality and immunity patterns of SARS-CoV-2. *Nature* 2021; **590**: 140–5.

46 Brazeau NF, Verity R, Jenks S, *et al.* Estimating the COVID-19 infection fatality ratio accounting for seroreversion using statistical modelling. *Commun Med (Lond)* 2022; **2**: 54.

47 Sauré D, Neira I, Goic M, *et al.* Infection with SARS-CoV-2 variant gamma (p.1) in chile increased ICU admission risk three to five-fold. *PLoS One* 2023; **18**: e0283085.

48 Abate SM, Ahmed Ali S, Mantfardo B, Basu B. Rate of intensive care unit admission and outcomes among patients with coronavirus: A systematic review and meta-analysis. *PLoS One* 2020; **15**: e0235653.

49 Polack FP, Thomas SJ, Kitchin N, *et al.* Safety and efficacy of the BNT162b2 mRNA covid-19 vaccine. *N Engl J Med* 2020; **383**: 2603–15.

50 Liu Q, Qin C, Liu M, Liu J. Effectiveness and safety of SARS-CoV-2 vaccine in real-world studies: A systematic review and meta-analysis. *Infect Dis Poverty* 2021; **10**: 132.

51 Grint DJ, Wing K, Houlihan C, *et al.* Severity of severe acute respiratory system coronavirus 2 (SARS-CoV-2) alpha variant (b.1.1.7) in england. *Clin Infect Dis* 2022; **75**: e1120–7.

52 Pascall DJ, Vink E, Blacow R, *et al.* The SARS-CoV-2 alpha variant was associated with increased clinical severity of COVID-19 in scotland: A genomics-based retrospective cohort analysis. *PLoS One* 2023; **18**: e0284187.

53 Twohig KA, Nyberg T, Zaidi A, *et al.* Hospital admission and emergency care attendance risk for SARS-CoV-2 delta (b.1.617.2) compared with alpha (b.1.1.7) variants of concern: A cohort study. *Lancet Infect Dis* 2022; **22**: 35–42.

54 Veneti L, Valcarcel Salamanca B, Seppälä E, *et al.* No difference in risk of hospitalization between reported cases of the SARS-CoV-2 delta variant and alpha variant in norway. *Int J Infect Dis* 2022; **115**: 178–84.
