# Supplementary material for: Evaluating the effects of population mobility on transmission during the COVID-19 pandemic
Source: Nat Commun. 2026 Oct 14;17:11634. doi: 10.1038/s41467-026-78247-9 (PMC13755426; doi:10.1038/s41467-026-78247-9)
Supplement: Supplementary file 2 [file 41467_2026_78247_MOESM2_ESM.pdf]

nature portfolio

Corresponding author(s): James M Trauer, NCOMMS-25-90404-Z

Last updated by author(s): Jul 13, 2026

Reporting Summary

Nature Portfolio wishes to improve the reproducibility of the work that we publish. This form provides structure for consistency and transparency in reporting. For further information on Nature Portfolio policies, see our Editorial Policies and the Editorial Policy Checklist.

Statistics

For all statistical analyses, confirm that the following items are present in the figure legend, table legend, main text, or Methods section.

n/a	Confirmed	
[ ]	[x]	The exact sample size (*n*) for each experimental group/condition, given as a discrete number and unit of measurement
[x]	[ ]	A statement on whether measurements were taken from distinct samples or whether the same sample was measured repeatedly
[x]	[ ]	The statistical test(s) used AND whether they are one- or two-sided<br>*Only common tests should be described solely by name; describe more complex techniques in the Methods section.*
[x]	[ ]	A description of all covariates tested
[x]	[ ]	A description of any assumptions or corrections, such as tests of normality and adjustment for multiple comparisons
[ ]	[x]	A full description of the statistical parameters including central tendency (e.g. means) or other basic estimates (e.g. regression coefficient) AND variation (e.g. standard deviation) or associated estimates of uncertainty (e.g. confidence intervals)
[x]	[ ]	For null hypothesis testing, the test statistic (e.g. *F*, *t*, *r*) with confidence intervals, effect sizes, degrees of freedom and *P* value noted<br>*Give P values as exact values whenever suitable.*
[ ]	[x]	For Bayesian analysis, information on the choice of priors and Markov chain Monte Carlo settings
[x]	[ ]	For hierarchical and complex designs, identification of the appropriate level for tests and full reporting of outcomes
[x]	[ ]	Estimates of effect sizes (e.g. Cohen's *d*, Pearson's *r*), indicating how they were calculated

Our web collection on statistics for biologists contains articles on many of the points above.

Software and code

Policy information about availability of computer code

Data collection	All input notebooks used for data collection and initial processing are available at https://github.com/monash-emu/renewal/tree/submission_2/notebooks/inputs. All data were extracted using the "read_csv" from pandas version 2.2.3. Country identification processes used pycountry version 24.6.1.
Data analysis	We chose pixi for environment and project management to ensure model outputs are reproducible across users and architectures. The lock file specifying the version used for each analysis is available at https://github.com/monash-emu/renewal/blob/submission_2/pixi.lock and the commit ID used for each analysis is specified in tables at the end of each supplementary outputs file.

For manuscripts utilizing custom algorithms or software that are central to the research but not yet described in published literature, software must be made available to editors and reviewers. We strongly encourage code deposition in a community repository (e.g. GitHub). See the Nature Portfolio guidelines for submitting code & software for further information.

Data

Policy information about availability of data

All manuscripts must include a data availability statement. This statement should provide the following information, where applicable:

- Accession codes, unique identifiers, or web links for publicly available datasets
- A description of any restrictions on data availability
- For clinical datasets or third party data, please ensure that the statement adheres to our policy

The URLs to extract the data used in our analyses are specified in out inputs folder at https://github.com/monash-emu/renewal/tree/submission_2/notebooks/

inputs and in our Supplementary Methods file. Population data was obtained from https://population.un.org/wpp/assets/Excel%20Files/1_Indicator%20(Standard)/EXCEL_FILES/2_Population/WPP2024_POP_F02_1_POPULATION_5-YEAR_AGE_GROUPS_BOTH_SEXES.xlsx, hospitalisation data from https://raw.githubusercontent.com/owid/covid-19-data/refs/heads/master/public/data/hospitalizations/covid-hospitalizations.csv, seroprevalence data from https://github.com/serotracker/sars-cov-2-data/raw/refs/heads/main/serotracker_dataset.csv, variant data from https://github.com/hodcroftlab/covariants/raw/refs/heads/master/cluster_tables/, Google mobility data from https://www.gstatic.com/covid19/mobility/Region_Mobility_Report_CSVs.zip and Facebook mobility data from : https://data.humdata.org/dataset/.

Research involving human participants, their data, or biological material

Policy information about studies with human participants or human data. See also policy information about sex, gender (identity/presentation), and sexual orientation and race, ethnicity and racism.

Reporting on sex and gender	N/A
Reporting on race, ethnicity, or other socially relevant groupings	N/A
Population characteristics	N/A
Recruitment	N/A
Ethics oversight	N/A

Note that full information on the approval of the study protocol must also be provided in the manuscript.

Field-specific reporting

Please select the one below that is the best fit for your research. If you are not sure, read the appropriate sections before making your selection.

[x] Life sciences [ ] Behavioural & social sciences [ ] Ecological, evolutionary & environmental sciences

For a reference copy of the document with all sections, see nature.com/documents/nr-reporting-summary-flat.pdf

Life sciences study design

All studies must disclose on these points even when the disclosure is negative.

Sample size	No formal sample size calculation was performed. The study included all countries for which sufficient publicly available epidemiological and mobility data were available to support the analysis, resulting in the inclusion of 122 countries.
Data exclusions	We only included countries for which multiple data streams were available to estimate variations in transmission rates over time. Selection of countries for inclusion/exclusion is detailed under "Selection of countries for inclusion" and Table 1 of the Supplementary Methods. Data sources used in model calibration are detailed under "Calibration targets" of the Supplementary Methods. In general, all available and relevant data streams were included as calibration targets, except that case notifications prior to 1st July 2020 were not incorporated and a maximum of one hospitalisation-related target was included for each country.
Replication	We chose pixi for environment and project management to ensure model outputs are reproducible across users and architectures. The commit ID (SHA) for each analysis presented is provided in tables at the end of each supplemental output file.
Randomization	Randomisation was not relevant to this analysis because it did not involve experimental group allocation.
Blinding	Blinding was not relevant to this analysis because it did not involve experimental group allocation.

Reporting for specific materials, systems and methods

We require information from authors about some types of materials, experimental systems and methods used in many studies. Here, indicate whether each material, system or method listed is relevant to your study. If you are not sure if a list item applies to your research, read the appropriate section before selecting a response.

Materials & experimental systems

n/a	Involved in the study
☒	☐ Antibodies
☒	☐ Eukaryotic cell lines
☒	☐ Palaeontology and archaeology
☒	☐ Animals and other organisms
☒	☐ Clinical data
☒	☐ Dual use research of concern
☒	☐ Plants

Methods

n/a	Involved in the study
☒	☐ ChIP-seq
☒	☐ Flow cytometry
☒	☐ MRI-based neuroimaging

Plants

Seed stocks

Report on the source of all seed stocks or other plant material used. If applicable, state the seed stock centre and catalogue number. If plant specimens were collected from the field, describe the collection location, date and sampling procedures.

Novel plant genotypes

Describe the methods by which all novel plant genotypes were produced. This includes those generated by transgenic approaches, gene editing, chemical/radiation-based mutagenesis and hybridization. For transgenic lines, describe the transformation method, the number of independent lines analyzed and the generation upon which experiments were performed. For gene-edited lines, describe the editor used, the endogenous sequence targeted for editing, the targeting guide RNA sequence (if applicable) and how the editor was applied.

Authentication

Describe any authentication procedures for each seed stock used or novel genotype generated. Describe any experiments used to assess the effect of a mutation and, where applicable, how potential secondary effects (e.g. second site T-DNA insertions, mosiacism, off-target gene editing) were examined.
