# Supplementary material for: Evaluating the effects of population mobility on transmission during the COVID-19 pandemic
Source: Nat Commun. 2026 Oct 14;17:11634. doi: 10.1038/s41467-026-78247-9 (PMC13755426; doi:10.1038/s41467-026-78247-9)
Supplement: Supplementary file 3 [file 41467_2026_78247_MOESM3_ESM.pdf]

nature portfolio

Peer Review File

Evaluating the effects of population mobility on transmission during the COVID-19 pandemic

Corresponding Author: Dr James Trauer

This file contains all reviewer reports in order by version, followed by all author rebuttals in order by version.

Attachments originally included by the reviewers as part of their assessment can be found at the end of this file.

A version of this paper was originally rejected for publication by Nature Communications, however that decision was reconsidered after appeal by the authors.

Version 0:

Reviewer comments:

Reviewer #1

(Remarks to the Author)
Please see the attached pdf for my full comments. In here, I am just copying my summary.

This manuscript presents a large-scale, multi-country assessment (122 nations) of the relationship between digital mobility metrics (Google and Facebook) and SARS-CoV-2 transmission dynamics. The authors employ a renewal model framework augmented by a flexible "residual transmission process" (Wiener process) to quantify the extent to which mobility data reduces unexplained variance in transmission rates (Rt).
The scale of the analysis and the harmonized computational framework are impressive. The attempt to validate mobility data not merely by correlation, but by its ability to reduce the dispersion of a residual process, represents a methodologically creative contribution. The use of a modern probabilistic programming stack (JAX/NumPyro) and the commitment to reproducibility are commendable.
However, as a submission in 2025, the manuscript faces a fundamental crisis of relevance and epidemiological specification. The core research question—"Did mobility restrictions reduce transmission in 2020?"—has been extensively settled in the literature. For this work to provide value five years post-emergence, it must offer novel insights into surveillance infrastructure or long-term transmission dynamics.
Unfortunately, the current model omits critical biological realities—specifically waning immunity, variant specific cross-immunity, and variant-specific severity (IFR)—that are non-negotiable for a retrospective analysis of this duration. These omissions introduce significant bias, potentially confounding the very mobility effects the authors seek to measure. Furthermore, the null findings in Low-and-Middle-Income-Countries (LMICs) may appear to be driven by data quality artifacts that are insufficiently interrogated.
Unless the manuscript is radically reframed to focus on the limitations of digital surveillance and the model is structurally corrected to account for reinfection, cross-immunity and severity changes, it does not meet the criteria for publication in current landscape.

(Remarks on code availability)
Code is well documented, and I was able to run the scripts for single countries for corss-checking. Hwever, I did find it a bit diffcult to adapt code with new data streams or tweaking model to account for few chnages I wanted to check.

Reviewer #2

(Remarks to the Author)

In general I felt this was an interesting paper that likely merits publication in Nature Communications, but requires a fair bit more detail in the main text in order to be publishable, particularly with regard to the Methods.

Line 42: “considering changes to policy rather than mobility itself” needs to be clarified a bit--there were policies that changed policies but governments didn’t have a way other than policy change to modify mobility.

At the end of the Introduction, I think a more specific statement of how you tested for the effect of mobility would be helpful. It’s well-described in the Abstract, basically looking for that level of detail.

Figure 1: I can infer on the left that the red dots are the actual data but that should be made explicit.

Lines 63-70. Simply stating that the calibration fits are ‘excellent’ is not very convincing. It would be more convincing to take each statement, “we achieved excellent calibration fits”, “close replicate the profile of daily new deaths…” and either make those topic sentences of independent paragraphs, or simply use the first paragraph of the results to summarize how the model was analyzed and the kinds of results created without stating the quality of the model/results.

For lines 95 - 118, I think some reorganization is needed with Figure 2. It is not very easy to determine by eye the correlation between a mobility metric and the residual transmission estimate, where I think the goal of the figure is to show the correlation of the two in different countries, for different sectors.

In general Lines 95 - 138 is a lot of information to parse in text. I would suggest either 1) trying to identify key themes and centering your paragraphs around each. You have partly done this already by aggregating by location type, but I guess I am wondering if there are patterns that occur by region e.g. African countries in the dataset vs Europe in terms of relationship between mobility and scaling transmission, and whether that would help readability. Another option 2) would be to create a table showing your correspondences to more easily show which ones were positive/negative (this could either be done by changing Figure 2 itself, which may be preferable considering I am not sure Figure 2 shows what the text implies very clearly), and option 3) would be to remove some of the information and move it to the supplementary info (e.g. for the parks/transit station metrics which did not have significant impact for most countries)

Lines 139 - 159: I am a little unclear on how comparing the weights would differ from looking at each sector’s correspondence with the transmission scaling. That is, wouldn’t the types of places that have the highest weights in the transmission scaling by definition have the strongest correspondence? I recognize these are different analyses but I wonder if they are different ways of assessing what ends up being very similar in the end.

Figure 4 is an interesting figure and I feel shows the main point of the paper better than preceding ones. I would consider moving this section/figure earlier.

Figure 5 seems important but it isn’t related to relevance for public health/analyses of these data. What would be a potential takehome here?

In the discussion, lines 232- 268, you should cite figures to help the reader see the evidence provided for various inferences.

One critical limitation/potential reason for some of these results is because of uptake of Google/Facebook services and the overall quality of the data. Figure 4 (and the end of the discussion) shows that the data work best in high income countries, which is noted in text, but it’s not noted how this could be due to the number of people using these services. Same with figure 5--the richest countries have larger fitted exponents, which might have something to do with the relationship between mobility and transmission. It’s noted in the discussion that this could be due to an effect for both infector and infectee, but I also wonder if the larger sample for these countries plays a role--a 10% reduction in mobility may mean more of a behavioral change in the Netherlands than in Senegal because it represents change in a larger fraction of the overall population.

I was a little confused by the representation of residual transmission scaling in the top right of Figure 2. Based on the summary/text preceding the residual transmission scaling text in the results, my first assumption was that residual transmission scaling referred to the residual transmission not accounted for after the mechanistic features and mobility, in which case, lower is better. But in the top right of Figure 2, adding in the mobility data increased the scaling, which implies that “residual transmission scaling” shows the amount of transmission scaling that was captured with/without mobility. If it’s the latter, I think that is reasonable, but it is easy to confuse with the bottom right of Figure 2, where the models with mobility data had lower residual transmission dispersion (Which is what I expected to see). The term ‘residual’ is what confuses me here… Perhaps something like non-mechanistic would be clearer.

Methods: I would add some of the text from the first part of the methods supplement to the methods in the main text. Text such as “we explicitly represented one or both of the alpha/delta strains” is too vague to really be useful in the main text but it’s improved if you mention that you have 1) cross-strain immunity parameters and 2) strain-specific transmission modfiiers. In general, I think it would strengthen the paper greatly to change the Methods section to be an abridged version of the Methods from the Supplementary Information (including some of the equations). As-is, the methods section is not terribly useful to read until you read the SI. I think the detail is generally present in the SI, but more of it needs to be present in the main text.

(Remarks on code availability)

Version 1:

Reviewer comments:

Reviewer #1

(Remarks to the Author)
Please look at attached file for my full review.

(Remarks on code availability)
As stated last time I did review code and code quality is good and I assume it will be a good source to the community.

Reviewer #2

(Remarks to the Author)
In general, the authors have done a good job addressing my comments, and have improved the description of the science and overall readability. I only really have one comment at this stage.

Specifically, the Methods is much clearer than before (and in general what was done is pretty clear after reading the Methods supplement), but some of the equations/outputs section from the Methods supplement should be in the main text. The current text is not incredibly relevant without some basis in equations--to make space for this I think details on scaling mobility data e.g. the paragraph starting on line 429 and the details 457-on are much less important for the main text and could be moved to the Supplement.

(Remarks on code availability)

Open Access This Peer Review File is licensed under a Creative Commons Attribution 4.0 International License, which permits use, sharing, adaptation, distribution and reproduction in any medium or format, as long as you give appropriate credit to the original author(s) and the source, provide a link to the Creative Commons license, and indicate if changes were made.
In cases where reviewers are anonymous, credit should be given to 'Anonymous Referee' and the source.
The images or other third party material in this Peer Review File are included in the article's Creative Commons license, unless indicated otherwise in a credit line to the material. If material is not included in the article's Creative Commons license and your intended use is not permitted by statutory regulation or exceeds the permitted use, you will need to obtain permission directly from the copyright holder.
To view a copy of this license, visit https://creativecommons.org/licenses/by/4.0/

Response to reviewers

We thank both reviewers for their detailed, constructive, and thoughtful comments. We have carefully considered each point raised and have revised the analysis and manuscript accordingly. In most cases, we have implemented the suggested changes directly; in others, we have expanded or clarified the modelling framework or manuscript text in ways that address the underlying concerns.

Summary of response

In response to the reviewer comments, we have made the following substantive modifications to the methods underpinning all analyses:

- Strain-specific severity. We have extended our model structure and parameterisation to incorporate this epidemiological consideration.
- Variant proportion calibration. We no longer recursively pool the estimates of variant proportions in generating our calibration targets.

In addition, we have incorporated two further analyses:

- Waning immunity sensitivity analysis. We evaluate the impact of incorporating host-related waning of immunity into our model and show that our core inferential results are robust to its inclusion.
- Identifiability analysis. We assess the identifiability of key model components, particularly the non-mechanistic transmission process and the mobility-mapping parameter, demonstrating that these quantities are recoverable and not confounded.

Finally, we have made substantial revisions to the manuscript text, including expanded methodological descriptions, revised framing in the Introduction and Discussion, and clarifications to improve readability in the Results.

Our point-by-point response to each comment follows.

Reviewer 1 response

1. Summary

This manuscript … current landscape

We thank the reviewer for their careful reading of the manuscript and for their generous appraisal of the scale, computational framework and methods of the study.

We further thank the reviewer for recognising the methodological novelty of our approach, which we have now highlighted explicitly in our revised submission. As the reviewer notes, the use of a flexible residual transmission process to assess the extent to which candidate covariates reduce unexplained temporal variation represents a general inferential technique that extends beyond the specific

application to SARS-CoV-2 and mobility data. We have now revised the manuscript to highlight that our novel approach has general applicability for inference using time series data applied to mechanistic models.

With regards to relevance, we believe our paper reports several findings that are both substantially novel and of clear scientific significance. While prior studies (including those cited in our Discussion) have identified associations between mobility and SARS-CoV-2 transmission in specific countries or time periods, we are not aware of previous work that applies a harmonised inference framework to evaluate these effects across settings that span the large majority of the world's population. This global scope allows us to move beyond a binary assessment of whether mobility restrictions did or did not work and instead to identify substantial heterogeneity in the strength – and, in many settings, the apparent absence – of a detectable association. We further demonstrate that this variation aligns with systematic differences in epidemiological context, data fidelity and surveillance capacity – yielding new insights into where digital mobility data can meaningfully support transmission inference. We therefore view the identification and interrogation of this heterogeneity as a novel contribution, with important implications for the interpretation and limitations of digital mobility data as a global surveillance tool.

In relation to biological realism, we agree that including additional epidemiologically relevant processes in our analysis can improve model specification and address these individually in our point-by-point responses below. Our original modelling choices reflected a deliberate trade-off between realism and parsimony, with the objective of mechanistically capturing short-term fluctuations in transmission that could plausibly confound or mask mobility effects in our modelling framework. For this reason, we did not incorporate waning immunity to previously infecting strains or variant-specific severity following infection in our original submission, but have now addressed these issues as described below.

2. Major Critiques

2.1 Novelty and Timeliness, Settled Science

As noted above (Reviewer 1, Summary, paragraph 3), we agree that an association between certain classes of mobility and transmission during the early phases of the pandemic is established in some contexts. The particular contribution of our manuscript is to provide a contextualised, quantitative assessment of their magnitude, consistency and detectability across diverse epidemiological and surveillance environments worldwide. By applying a harmonised inference framework, we were also able to compare the relative importance of different community settings and demonstrate their heterogeneous effects on transmission. While the qualitative pattern – stronger associations for indoor settings and weaker or absent associations for outdoor settings – is consistent with existing evidence, this concordance also provides an internal check on the behaviour of the modelling framework, supporting the validity of our residual-process approach. Our analysis

therefore extends prior work by identifying where mobility signals are informative, where they are not and how this depends on context – with relevance for future epidemiological investigations and prior specification for mechanistic models.

2.1 The Missed Opportunity: The true novelty … global health surveillance.

This comment is addressed in our response to comment 2.4, Reviewer 1 below.

2.1 Requirement: The paper must pivot. … social contact structure?

We thank the reviewer for this thoughtful and constructive comment. We agree that associations between mobility reductions and decreases in transmission were demonstrated early in the pandemic in several settings. However, we emphasise that no previous study has evaluated these relationships across such a large and diverse set of countries using a harmonised mechanistic framework and a consistent inferential metric. This global and methodologically coherent perspective is essential not only for revealing heterogeneity in settings that have been under-studied, but also for providing a quantitatively comparable assessment of mobility-transmission relationships in countries that have previously been analysed in isolation. Even in settings where mobility restrictions were known to influence transmission, our framework provides a consistent, cross-country quantification of how strongly mobility data helps explain short-term transmission dynamics relative to other sources of temporal variation.

We fully agree with the reviewer that a particularly consequential contribution of our work is our demonstration of the systematic absence of a usable mobility signal in many lower-income settings, particularly in West Africa and parts of Eastern Europe. This constitutes a clear illustration of the digital divide in global health surveillance, and we appreciate the reviewer's insight in highlighting this aspect of the results.

In response, we have revised the framing of the manuscript to emphasise its methodological novelty, its global comparative contribution and its role as a critical evaluation of when and where digital mobility proxies function as meaningful behavioural indicators. We have also expanded the Discussion to consider factors that may underpin the decoupling of mobility and transmission in some settings, including smartphone and app penetration, and differences in social contact patterns.

2.2 Epidemiological Misspecification

We agree that assuming permanent infection-blocking immunity is not biologically realistic over multi-year time horizons and that waning of protection against infection is an important feature of SARS-CoV-2 epidemiology. As formulated, the model in our original submission does incorporate some aspects of loss of immunity through variant-specific, directional cross-immunity, capturing what can be considered pathogen-driven waning associated with antigenic divergence. Individuals previously infected with a given variant retain partial susceptibility to later, antigenically distinct variants, governed by a calibrated immunity parameter. Complete cross-protection is only assumed to apply under the rare situation of a person previously infected with a

later variant (e.g. Delta) being exposed to an earlier one (e.g. Alpha). As such, susceptibility in the model is graded rather than binary.

Our original modelling choices reflect our deliberate goals of balancing parsimony and complexity described above, with a focus on including processes expected to generate short-term fluctuations in transmission. Explicit host-related waning of immunity over calendar time was not included in the base case analyses because we did not expect it to generate short-timescale variability in population-level susceptibility of sufficient magnitude to markedly influence our primary inferential target: the posterior distribution of the residual transmission dispersion.

To directly address the reviewer's concern, we have now repeated our analyses for all countries as a sensitivity analysis in which the model is extended to explicitly include waning immunity. Specifically, at each time step, a proportion of each immune class ($p \neq \{0\}^V$) is transitioned back to the fully susceptible state ($p = \{0\}^V$). The mean waning duration is assigned a truncated normal prior (lower bound 30 days, mean 180 days, standard deviation 30 days, no upper truncation). Under this extension, the posterior estimates of the residual transmission process and its dispersion are essentially unchanged relative to the base-case model, indicating that our conclusions regarding the informativeness of mobility data are robust to the inclusion of host-related waning over realistic timescales. We include a comparison of our analyses with waning immunity incorporated as a supplementary document.

In summary, while we agree that explicit waning is an important biological process over longer time horizons, the model already captures loss of protection associated with variant turnover, and incorporating additional host-related waning does not materially alter the central inferences of the study. We have revised the Methods and Discussion to clarify these issues.

2.3 Confounding Severity: The "Constant IFR" Fallacy

We agree with the reviewer that assuming a constant infection fatality rate across variants could be problematic, because variant-specific changes in clinical severity can distort inference during periods of new variant emergence when models are calibrated to deaths and other severe outcomes.

In response, we have extended the model (under both base-case and sensitivity configurations) to include explicit variant-specific severity parameters, with each defined relative to the preceding circulating variant. These parameters modify the risk of severe outcomes given infection, and are applied equivalently to the risk of both death and hospital admission (by extension modifying hospital occupancy and the ICU indicators equivalently).

We implemented variant-specific severity adjustments for Alpha and Delta in all analyses spanning those periods. For post-Omicron analyses, we did not further stratify severity across Omicron sublineages (BA.1, BA.2, BA.5), reflecting the absence of evidence for a marked difference in the severity for each of these

subvariants. The posterior distributions for each of the severity parameters are presented in Supplemental File 3 and our additional methods have been incorporated into the manuscript.

Incorporating variant-specific severity parameters eliminates the confounding pathway identified by the reviewer, whereby changes in clinical severity would otherwise be absorbed by our non-mechanistic transmission process. In our revised models, variation in deaths and other severe outcomes driven by changing virulence is now correctly attributed to severity, preserving the interpretability of the non-mechanistic process dispersion parameter as a metric of unexplained transmission dynamics. Although this led to some changes in the posterior distributions of our epidemiological input parameters, the posterior distributions of the non-mechanistic process dispersion and the mobility exponent parameters did not change appreciably, such that our main findings remain unchanged.

2.4 Data Artifacts in LMICs: The "Null Result" … technological penetration signal.

We agree with the reviewer that the strong secular increases over time observed in some Google mobility domains for some countries appear to be artefactual and so unlikely to reflect behavioural changes.

In response, we have run additional analyses for several countries in which an explicit detrending step is run before the mobility data are incorporated. Specifically, for any country for which the unweighted average of the Google mobility metrics more than doubled over the reporting period, we detrended each location-specific mobility series individually before applying our original weighting and aggregation procedure. This approach is described in our revised Methods.

This comment also prompted us to examine these trends in more detail. We found that the strong upward drifts typically occurred in only a subset of Google's location categories, with residential mobility often remaining stable while commercial categories (e.g. grocery and pharmacy or retail and recreation) showed pronounced increases. This pattern is consistent with artefacts that arose from changes in data capture or location categorisation within specific Google mobility domains. This underpinned our decision to detrend each location-specific metric separately, rather than adjusting only the aggregated series.

The results of the detrended analyses are included throughout our supplementary materials. Overall, the detrending step had little impact on our substantive findings, including the conclusions regarding the limited informativeness of mobility data in several LMIC settings. However, it sheds additional light on the utility of data from LMICs and substantially increases our confidence that these conclusions are not driven by data-capture artifacts.

2.5 Statistical Validation: "Fit" vs. "Prediction": The authors … model without it?

We thank the reviewer for raising this important point. We strongly agree with the principle that improved in-sample fit is insufficient to establish that a covariate

provides additional explanatory value, particularly if a flexible latent process is also included. However, we wish to clarify that our primary metric of the strength of evidence for an effect of mobility – the posterior distribution of the non-mechanistic transmission process dispersion – is not intended as a generic goodness-of-fit statistic. Rather, it quantifies the extent to which a candidate covariate can explain short-term temporal variation in transmission that would otherwise be absorbed within the regularised stochastic process. The near-identical target dispersion parameters for all mobility configurations demonstrate that goodness-of-fit is equivalent for all model types, such that comparison based on the residual transmission process dispersion is preferred. This distinction is central to our methods and we have revised the manuscript to make this clearer.

In our framework, the residual transmission component is not an unconstrained function that can freely substitute for the mobility covariate. Rather, it is a Wiener latent process with a prior structure that favours lesser degrees of short-term variation. The mobility term and the residual process therefore compete under regularisation: if mobility explains systematic temporal variation in transmission, the posterior favours a smaller dispersion because less latent variation is required to account for the observed dynamics and vice versa. Importantly, this comparison is made under the same residual process prior structure across all models. Our analytic approach therefore provides a comparison of how much latent variability remains necessary once mobility is included.

Despite these qualifications, we firmly agree with the reviewer's general concern that collinearity between the mobility covariate and the latent residual process could compromise identifiability and lead to misleading attribution. In response, we have added an explicit identifiability assessment designed to determine whether the model can distinguish the mobility mapping from the residual transmission updates. Specifically, we draw parameter sets from fitted base-case posterior samples, fix all other parameters at their sampled values, and then recalibrate allowing the model to recover the non-mechanistic transmission process updates vector, its dispersion parameter and the exponential mobility mapping parameter. These experiments show excellent recovery of the residual-process updates and the mobility mapping parameter, indicating that the mobility effect is not merely substituting for the latent process and that the two components are distinguishable under our framework. We report these analyses and associated diagnostics in the revised supplementary material and refer to them in the revised Methods/Results as an explicit statistical validation of the decomposition.

With regards to the reviewer's specific request for out-of-sample forecasting in this context, we believe that the additional analyses we have included are a more direct response to the general underlying concern. Moreover, forecasting transmission using a model that conditions on mobility requires either an external forecast of future mobility (which is unavailable in practice), or an additional model for mobility dynamics. Either approach would introduce an additional modelling problem –

forecasting human mobility under shifting policies and behavioural responses – which is outside the scope of the present study. More importantly, our objective is not real-time forecasting performance but the retrospective quantification of the informational value of mobility data for explaining short-term variation in transmission.

In summary, we agree that it is essential to demonstrate that mobility is not simply absorbing variance by virtue of added flexibility. We have addressed this by clarifying that the residual-process dispersion metric measures the reduction in required latent variability under a regularisation structure and have added identifiability analyses showing that the mobility mapping and residual transmission updates are recoverable.

3 Specific Technical Corrections

3.1 Seropositivity Definition Error: The Supplementary Methods … a logic error.

This error was in code documentation and has now been corrected. The validity of the original code can be demonstrated by reference to the following link, which shows the approach to seropositivity calculation in the originally submitted analyses: https://github.com/monash-emu/renewal/blob/2826a3ccd832c7ace778db4c093f1eeb3adb56b0/code/emu_renewal/renew.py#L581

3.2 Cross-Immunity Simplification: The use of … than vice versa).

The symbol $x_{p,v}$ may take three possible values, rather than being a single scalar, as was indicated by the subscripts and explained in our Supplemental Methods. These subscript markers indicate the variant combinations to which the individual was previously exposed and the currently infecting variants respectively. For the example provided of a later variant escaping immunity from an earlier one (although none of the included models include both Omicron and Delta variants), $x_{p,v}$xpv is the complement of the immune escape parameter for exposure to the later variant following exposure to the earlier one, but zero for exposure to the earlier variant following exposure to the later one. A fuller discussion of our approach to implementing immunity in our model is presented above in response to Comment 2.2, Reviewer 1.

3.3 Variant Pooling: The method … of variant replacement.

We acknowledge this comment, which is a reasonable and valid concern. In response, we have removed the recursive pooling approach to calculating variant prevalence in our revised analyses. Briefly to note the original reason for including this was that all our simulations result in monotonous increasing proportions of each new variant, and this approach provided slightly more robust calibrations in earlier iterations of our analyses. However, the current approach appears robust to this consideration and so this algorithm is no longer necessary. The irregular increases in

the variant proportions can now be seen in Supplemental Appendix 2, which displays the calibration targets.

4. Conclusion: This manuscript … multi-year analysis.

We address the general concerns of the reviewer in our response to 1. Summary above.

Reviewer 2 response

In general I felt this was an interesting paper that likely merits publication in Nature Communications, but requires a fair bit more detail in the main text in order to be publishable, particularly with regard to the Methods.

We thank the reviewer for the positive comments and have addressed the issues with methodological description, which we detail in response to the individual comments below.

Line 42: "considering changes to policy rather than mobility itself" needs to be clarified a bit--there were policies that changed policies but governments didn't have a way other than policy change to modify mobility.

We agree with the reviewer that this sentence was ambiguous and have now revised it for clarity. Our intent was to distinguish studies that used policy indicators (e.g. mandates, recommendations, stringency indices) as proxies for behavioural change from those that used empiric mobility data. We have now revised this sentence to:

"Analyses from several settings have shown that behavioural interventions can influence transmission, although many relied on timelines of changes in government mandates and recommendations rather than empirical measures of mobility, and were often limited to specific settings or countries."

At the end of the Introduction, I think a more specific statement of how you tested for the effect of mobility would be helpful. It's well-described in the Abstract, basically looking for that level of detail.

We thank the reviewer for this helpful suggestion. We have revised the end of the Introduction to provide a clearer description of how we assess the influence of mobility, consistent with the level of detail in the Abstract. The revised text now reads:

"In addition to publicly accessible mobility and surveillance data, the extensive epidemiological evidence now available, including the recent completion of comprehensive systematic reviews of key aspects of COVID-19 epidemiology, strengthens our understanding of the pandemic. To estimate the influence of mobility time series on transmission, we propose an approach that quantifies the extent to which short-term temporal variation in transmission must be absorbed by a latent process with and without mobility included under a renewal-model framework. We implemented this by estimating the reduction in the variability of a regularised non-mechanistic transmission component when mobility covariates were introduced. We

integrated this technique with epidemiological data sources and tools for high-performance numerical computing, and used this platform to estimate country-level effects of changes in mobility on SARS-CoV-2 transmission in 122 countries."

Figure 1: I can infer on the left that the red dots are the actual data but that should be made explicit.

We agree with this comment and have adjusted the figure caption to address it.

Lines 63-70. Simply stating that the calibration fits are 'excellent' is not very convincing. It would be more convincing to take each statement, "we achieved excellent calibration fits", "close replicate the profile of daily new deaths…" and either make those topic sentences of independent paragraphs, or simply use the first paragraph of the results to summarize how the model was analyzed and the kinds of results created without stating the quality of the model/results.

We agree with this comment and have revised this paragraph substantially, including to remove the overly evaluative statement that calibration fits were excellent. The revised paragraph is:

"Using a renewal model incorporating susceptible depletion, variant-specific transmissibility and incomplete immunity to emerging variants, we closely replicated epidemiological targets for each country analysed (Figure 1 of Supplemental File 1 shows country inclusion, Supplemental File 2 shows all calibration fits, Figure 1 shows results for France as an example country). These targets included time series profiles for daily new deaths, case notifications and up to one hospitalisation indicator, and the proportional prevalence of key variants of concern. Where seroprevalence targets could be sourced, these were included to give a broad guide to epidemic size and the effect of susceptible depletion, and were matched in many cases, including most high-income settings. We conducted analyses with and without adjustment of transmission according to changes in mobility, and focused on epidemiological contexts with low natural immunity and for which changes in vaccine-induced immunity were likely to be negligible – either because the analysis period preceded reaching 5% vaccination coverage (118 countries) or because coverage had recently reached 90% of the final coverage achieved (Singapore and countries of Oceania)."

For lines 95 - 118, I think some reorganization is needed with Figure 2. It is not very easy to determine by eye the correlation between a mobility metric and the residual transmission estimate, where I think the goal of the figure is to show the correlation of the two in different countries, for different sectors.

The reviewer is correct in her/his interpretation of this figure. We have revised this figure for clarity, moving the mobility considered to the top of each column of panels, rather than repeating this text above each panel. We have also slightly widened the horizontal gap between each column of panels. In combination with our revision of the text that matches to this figure, we believe that this part of our results is now easier to understand.

In general Lines 95 - 138 is a lot of information to parse in text. I would suggest either 1) trying to identify key themes and centering your paragraphs around each. You have partly done this already by aggregating by location type, but I guess I am wondering if there are patterns that occur by region e.g. African countries in the dataset vs Europe in terms of relationship between mobility and scaling transmission, and whether that would help readability. Another option 2) would be to create a table showing your correspondences to more easily show which ones were positive/negative (this could either be done by changing Figure 2 itself, which may be preferable considering I am not sure Figure 2 shows what the text implies very clearly), and option 3) would be to remove some of the information and move it to the supplementary info (e.g. for the parks/transit station metrics which did not have significant impact for most countries)

We have adapted both the figure (see preceding comment) and the text (see following comment) in response to this reasonable suggestion.

Lines 139 - 159: I am a little unclear on how comparing the weights would differ from looking at each sector's correspondence with the transmission scaling. That is, wouldn't the types of places that have the highest weights in the transmission scaling by definition have the strongest correspondence? I recognize these are different analyses but I wonder if they are different ways of assessing what ends up being very similar in the end.

We thank the reviewer for these very helpful suggestions. In response, we have reorganised this section to improve clarity and flow. Specifically, we have rewritten the text to present the exploratory findings around key correspondence patterns, rather than listing individual country-metric pairs. Related to these points, we have also revised Figure 2, as explained in more detail below.

The revised text is structured into the following six thematic paragraphs:

1. Overview, explanation and rationale
2. Settings with strong correspondence
3. Settings with weak or inconsistent correspondence
4. Additional domain-specific considerations
5. Patterns in the weighted Google function
6. Comparison of Google and Facebook mobility metrics

These changes substantially improve the organisation of the section and make the exploratory role of Figure 2 clearer in context, while preserving the breadth of examples.

Figure 4 is an interesting figure and I feel shows the main point of the paper better than preceding ones. I would consider moving this section/figure earlier.

This is a good point and we have reordered the figures in response to this suggestion. What was previously Figure 3 (the Google weight posteriors) has been moved to the end of the results and is now Figure 5. This promotes the figures that

provide more general results from their previous positions as Figures 4 and 5, to now being Figures 3 and 4. Figures 1 and 2 remain at the beginning of the Results to preserve the logical progression from illustrating the core components of the analysis and raw comparisons to the formal inferential results.

Figure 5 seems important but it isn't related to relevance for public health/analyses of these data. What would be a potential takehome here?

We thank the reviewer for highlighting the need to clarify the purpose and interpretation of this figure. The mobility-exponent parameter represents the functional scale required to map each mobility metric onto transmission in the context of our semi-mechanistic model. In practice, it quantifies the degree of transformation needed for mobility to optimally explain short-term variation in transmission.

The public health relevance of the figure is that settings with strong evidence for a mobility effect (high dispersion ratios) consistently show mobility-exponent values around or moderately above one. This indicates that in these typically high-income settings the mobility metric requires a proportional or super-proportional scaling to optimally reduce the residual temporal variation. A proportional mapping would represent a direct relationship between changes in mobility and the instantaneous reproduction number.

In contrast, settings with weak or no evidence of an effect (dispersion ratios near or below one) often have mobility-exponent values below one, implying that the raw mobility series in these locations does not provide a behavioural signal that aligns naturally with transmission dynamics and must be compressed or attenuated to fit the model. This pattern is seen predominantly in lower-income settings, which may reflect different behavioural structures or deficiencies in the mobility data or the data used for calibration.

The take-home message is that where mobility is informative, it behaves in a stable and interpretable manner, requiring predictable levels of transformation. By contrast, in settings where mobility contributes little explanatory value, the required mapping becomes unstable or implausibly attenuated, consistent with mobility data being a poor or indirect proxy for behavioural change in those contexts. This is now explained more intuitively in our Results.

In the discussion, lines 232- 268, you should cite figures to help the reader see the evidence provided for various inferences.

We agree. References to specific figures have been added to the Discussion as requested.

One critical limitation/potential reason for some of these results is because of uptake of Google/Facebook services and the overall quality of the data. Figure 4 (and the end of the discussion) shows that the data work best in high income countries, which is noted in text, but it's not noted how this could be due to the number of people using these services. Same with figure 5--the richest countries have larger fitted exponents, which might have something to do with the relationship between mobility and transmission. It's noted in the discussion that this could be due to an effect for both infector and infectee, but I also wonder if the larger sample for these countries plays a role--a 10% reduction in mobility may mean more of a behavioral change in the Netherlands than in Senegal because it represents change in a larger fraction of the overall population.

We thank the reviewer for raising this point. We agree that the quality and stability of mobility datasets vary substantially across settings, and that differences in factors such as smartphone and app penetration and user representativeness are likely contributors to the stronger correspondence observed in high-income countries. The mobility metrics we use are normalised relative measures, so differences in the absolute number of contributing devices do not necessarily introduce bias. However, in settings where the contributing user base is small, socioeconomically skewed, or evolving over time – as may well be the case in some LMICs – the resulting mobility series may reflect systematic sampling variation rather than population-level behavioural change.

In light of this comment and the requests from Reviewer 1, we have added text to the Discussion to clarify this distinction and to note that both behavioural heterogeneity and data quality issues likely contributed to the observed patterns of mobility informativeness across income settings.

I was a little confused by the representation of residual transmission scaling in the top right of Figure 2. Based on the summary/text preceding the residual transmission scaling text in the results, my first assumption was that residual transmission scaling referred to the residual transmission not accounted for after the mechanistic features and mobility, in which case, lower is better. But in the top right of Figure 2, adding in the mobility data increased the scaling, which implies that "residual transmission scaling" shows the amount of transmission scaling that was captured with/without mobility. If it's the latter, I think that is reasonable, but it is easy to confuse with the bottom right of Figure 2, where the models with mobility data had lower residual transmission dispersion (Which is what I expected to see). The term 'residual' is what confuses me here… Perhaps something like non-mechanistic would be clearer.

We thank the reviewer for raising this point and believe the reviewer is referring to Figure 1. The top-right panel of Figure 1 is intended to illustrate the temporal smoothness and magnitude of the non-mechanistic transmission component under models with and without mobility. The quantity plotted reflects the scaling value of the non-mechanistic process over time, rather than its variation. (For this reason, the absolute vertical values are not meaningful and we have intentionally omitted the

y-axis tick labels.) The key visual signal in this panel is the degree of fluctuation rather than the height of the curve. When mobility is included, the non-mechanistic process typically shows smaller and less abrupt temporal changes, even if its absolute level appears higher in places. Although this figure shows only one example country, this is the concept that underpins much of our analysis and interpretation of the supplemental materials that provide comprehensive results for all the countries analysed.

We agree that terminology played a role in this confusion. In response, we have replaced the term “residual transmission” with “non-mechanistic transmission” throughout the manuscript, as the latter more clearly describes its purpose: capturing short-term variation not explained by the mechanistic components or mobility covariates. We have also updated figure captions and substantially revised the Methods and Results to make this interpretation of our results clearer.

Methods: I would add some of the text from the first part of the methods supplement to the methods in the main text. Text such as “we explicitly represented one or both of the alpha/delta strains” is too vague to really be useful in the main text but it’s improved if you mention that you have 1) cross-strain immunity parameters and 2) strain-specific transmission modfiiers. In general, I think it would strengthen the paper greatly to change the Methods section to be an abridged version of the Methods from the Supplementary Information (including some of the equations). As-is, the methods section is not terribly useful to read until you read the SI. I think the detail is generally present in the SI, but more of it needs to be present in the main text.

We thank the reviewer for this valuable suggestion and agree that a more detailed description of the model in the main text strengthens the manuscript. While journal format requirements preclude inclusion of the full technical specification in the main text, we have expanded the Methods section to provide a clearer and more self-contained description of the core modelling components. In particular, we now provide additional detail on the structure of the non-mechanistic transmission process, the rationale for its use, and how it integrates with the renewal framework, as well as a more explicit summary of the variant representation and immunity structure.

The full mathematical and algorithmic details remain in our supplementary materials – which was generated directly from the code used in our analysis – but the revised Methods now present a fuller conceptual overview that allows readers to understand the modelling framework without necessarily needing to read the full Supplementary Methods document.

Response to reviewers

We thank both reviewers for their further comments.

Reviewer 1

We have not received the attached file referred to.

We thank the reviewer for her/his comments on the code.

Reviewer 2

In general, the authors have done a good job addressing my comments, and have improved the description of the science and overall readability. I only really have one comment at this stage.

Specifically, the Methods is much clearer than before (and in general what was done is pretty clear after reading the Methods supplement), but some of the equations/outputs section from the Methods supplement should be in the main text. The current text is not incredibly relevant without some basis in equations--to make space for this I think details on scaling mobility data e.g. the paragraph starting on line 429 and the details 457-on are much less important for the main text and could be moved to the Supplement.

In response to this comment, the first two equations from the Supplementary Material specifying the model implementation have been added to the main manuscript. These equations provide the central intuition underlying the renewal process and the implementation of mobility effects, allowing readers to understand the modelling approach without reference to the Supplementary Material.

Peer Review Report

Review for *Evaluating the effects of population mobility on the COVID-19 pandemic*

December 17, 2025

Recommendation: Major Revision / Referral to Specialized Methods Journal

1 Summary

This manuscript presents a large-scale, multi-country assessment (122 nations) of the relationship between digital mobility metrics (Google and Facebook) and SARS-CoV-2 transmission dynamics. The authors employ a renewal model framework augmented by a flexible "residual transmission process" (Wiener process) to quantify the extent to which mobility data reduces unexplained variance in transmission rates (R_t).

The scale of the analysis and the harmonized computational framework are impressive. The attempt to validate mobility data not merely by correlation, but by its ability to reduce the dispersion of a residual process, represents a methodologically creative contribution. The use of a modern probabilistic programming stack (JAX/NumPyro) and the commitment to reproducibility are commendable.

However, as a submission in 2025, the manuscript faces a fundamental crisis of relevance and epidemiological specification. The core research question— *"Did mobility restrictions reduce transmission in 2020?"*—has been extensively settled in the literature. For this work to provide value five years post-emergence, it must offer novel insights into **surveillance infrastructure** or **long-term transmission dynamics**.

Unfortunately, the current model omits critical biological realities—specifically **waning immunity**, **variant specific cross-immunity**, and **variant-specific severity (IFR)**—that are non-negotiable for a retrospective analysis of this duration. These omissions introduce significant bias, potentially confounding the very mobility effects the authors seek to measure. Furthermore, the null findings in Low-and-Middle-Income-Countries (LMICs) may appear to be driven by data quality artifacts that are insufficiently interrogated.

Unless the manuscript is radically reframed to focus on the **limitations of digital surveillance** and **the model is structurally corrected to account for reinfection, cross-immunity and severity changes**, it does not meet the criteria for publication in current landscape.

2 Major Critiques

2.1 Novelty and Timeliness:

The manuscript asks a question that was urgent in 2020 but is largely historical in 2025.

- **Settled Science:** The link between mobility reduction and R_t reduction during the immunologically naïve phase of the pandemic is well-established. Confirming that indoor settings (retail/transit) correlate with spread while outdoor settings (parks) do not is essentially a validation of aerosol transmission physics, which is already consensus.

- **The Missed Opportunity:** The true novelty of this paper lies in its **negative findings**. The fact that digital mobility proxies failed to explain transmission in West Africa and parts of Eastern Europe is a profound insight into the "Digital Divide" in global health surveillance.

- **Requirement:** The paper must pivot. Instead of a confirmatory analysis of mobility efficacy, it should be framed as a critical audit of **digital surveillance limitations**. It should rigorously analyze *why* the signal decoupled in LMICs. Is it a failure of the proxy (smartphone penetration) or a difference in social contact structure?

2.2 Epidemiological Misspecification:

The most critical technical flaw is the model's treatment of immunity. The manuscript states: *"persons who had previously been infected were assumed to derive complete immunity to the infecting strain and to variant strains that had emerged prior to the infecting strain"*.

- **The Flaw:** In a multi-year analysis (2020–2022), assuming permanent immunity is statistically and biologically invalid. SARS-CoV-2 immunity wanes significantly, particularly against infection. By assuming permanent immunity, the model artificially depletes the Susceptible pool (S_t). When late-stage waves (Delta/Omicron) occur, the model forces the "Residual Transmission" process (W_t) or mobility parameters to account for infections that are actually driven by **reinfection** of the previously "immune."

- **The Consequence:** This creates a structural bias. If mobility is low but cases rise (due to waning immunity/reinfection), the model may incorrectly conclude that mobility is non-predictive.

- **Requirement:** The authors must introduce a waning immunity mechanism (e.g., transition from Recovered to Susceptible) and variant specific cross-immunity . Without this, the S_t dynamics for any period beyond $\sim$12 months are incorrect.

2.3 Confounding Severity: The "Constant IFR" Fallacy

The model calibrates to **deaths** but explicitly excludes *"differing risk of severe outcomes given infection by infecting variant"*.

- **The Flaw:** The Infection Fatality Rate (IFR) varied dramatically between variants. Alpha was $\sim$55% more severe than Wild-type; Delta was more severe than Alpha; Omicron was significantly less severe.

- **The Consequence:** By using a fixed IFR, the model misinterprets death data. During the **Delta** wave, high deaths will be interpreted by the model as "massive transmission," potentially overestimating the attack rate. Conversely, during **Omicron**, low deaths relative to cases will be misinterpreted. This forces the residual process (W_t) to absorb the severity error, invalidating the paper's core evidence metric (reduction in residual dispersion).

2.4 Data Artifacts in LMICs

The "Null Result" in LMICS countries can be a data artifact. The authors note "strong secular trends" (continuous increases) in Google mobility data for nations like Nigeria.

- **The Critique:** These trends likely reflect increased smartphone adoption or Google's data capture improvements, not behavioral changes. Interpreting a lack of correlation between a **trending** variable (mobility artifacts) and a **cyclical** variable (epidemic waves) as "mobility doesn't drive transmission" is ecologically fallacious.

- **Requirement:** The authors must attempt to **de-trend** the African mobility data to isolate the behavioral signal from the technological penetration signal.

2.5 Statistical Validation: "Fit" vs. "Prediction"

The authors use the "reduction in residual dispersion" as their primary evidence metric. While elegant, this effective measures **goodness-of-fit**.

- **The Critique:** The residual process (W_t) and the mobility scalar (M_t) are partially collinear—they can substitute for each other. A model with mobility might have lower residual dispersion simply because it has more degrees of freedom, not because it has better predictive validity.

- **Requirement:** To prove utility in 2025, the model must demonstrate **out-of-sample forecasting skill**. Does adding mobility data allow the model to predict the *next* wave better than a model without it?

3 Specific Technical Corrections

1. **Seropositivity Definition Error:** The Supplementary Methods state: *"Seropositivity was calculated as the proportion of the population remaining in the entirely infection-naïve immunity sub-population"*.

   - This defines **seronegativity**, not seropositivity. If this logic is hard-coded (e.g., calibrating "60% seroprevalence" to "60% naïve"), the model is inverted and effectively invalid. The authors must clarify if this is a text typo or a logic error.

2. **Cross-Immunity Simplification:** The use of a single scalar for cross-immunity $(1-x_{p,v})$ averages out the directed nature of immune escape (e.g., Omicron escapes Delta immunity more than vice versa).

3. **Variant Pooling:** The method of "recursively pooling" variant proportions to ensure they are strictly increasing is epidemiologically unsound. Variant prevalence can fluctuate; forcing monotonicity distorts the timing of variant replacement.

4 Conclusion

This manuscript is a technically sophisticated answer to a question that is no longer being asked. Its utility for future preparedness is compromised by the omission of the very dynamics that define modern respiratory pandemics: **waning immunity, reinfection, and variable virulence.**

If the authors can (1) implement waning immunity, variant-specific cross-immunity and variant-specific IFR, and (2) reframe the paper as a critique of surveillance data limitations in the LMICs, it may be suitable for publication as a retrospective lesson. Otherwise, it is fundamentally misspecified for a multi-year analysis.

Reviewer report : *Evaluating the effects of population mobility on transmission during the COVID-19 pandemic*

Second round review

Recommendation: Major revision.

Summary

The authors apply a renewal-model framework with a regularised non-mechanistic (Wiener) scaling process to assess how Google and Facebook mobility metrics align with SARS-CoV-2 transmission dynamics across 122 countries. The central inferential device, comparing the posterior dispersion of the non-mechanistic process with and without mobility, is methodologically creative, and the harmonised cross-country scope, computational stack (JAX/NumPyro), and reproducibility commitment are real strengths. The revised manuscript directly addresses several of the prior round's most substantive concerns, particularly the inclusion of variant-specific severity in the base case, secular detrending of Google data in LMICs, removal of the recursive variant-pooling step, and reframing toward the digital-divide narrative.

However, several issues remain that, in aggregate, prevent acceptance in the current form. The dispersion-reduction metric is the paper's primary inferential target but has not been validated against simulated ground truth. The mobility series is applied as an instantaneous scaling on transmission, ignoring the generation interval and reporting-delay structure of SARS-CoV-2. Mobility is treated as a stand-alone covariate without controlling for concurrent NPIs and testing-policy changes, yet the Discussion sometimes uses mechanistic language. Several technical issues (mathematical validity of exponentiating raw mobility series, the MCMC pairing logic for the dispersion ratio, and joint identifiability of the location weights) need direct attention.

Revisions handled well

- **Variant-specific severity now in the base-case model**, not as a sensitivity. This was the strongest prior-round concern and the authors addressed it head-on. The fact that mobility/dispersion posteriors did not shift materially after this change is reassuring, conditional on the recovered severity values being epidemiologically plausible (see Major Concern 7).
- **Detrending of secular Google trends** in LMIC countries; the diagnostic observation that drift was domain-specific (commercial categories, not residential) is a nice argument that it reflects a data-capture artefact rather than behaviour.
- **Reframing toward the digital-divide narrative**, the Discussion now appropriately foregrounds heterogeneity and LMIC data quality.

- **Recursive variant pooling removed**, clean fix.
- **Terminology shift from "residual" to "non-mechanistic"**, small but genuine clarification, addressing R2's confusion.

Major concerns

1. The dispersion-reduction metric has not been validated against ground truth.

The paper's core inferential claim rests on the reduction in the posterior of the Wiener-process dispersion parameter when mobility is included. The authors' identifiability analysis (synthetic data drawn from the fitted posteriors with most parameters fixed) is a useful internal-consistency check but does not test whether the metric correctly recovers a known mobility–transmission relationship under a realistic calibration pipeline. A controlled simulation study is needed: generate synthetic epidemic trajectories under (a) a known nonzero mobility effect, (b) a null scenario where mobility has no effect, and (c) a scenario where the latent process drives all temporal variation. Apply the full calibration pipeline (not the constrained refit) and demonstrate that the dispersion ratio reliably distinguishes these cases. Without this, the metric's interpretation as "strength of evidence" cannot be supported.

2. Instantaneous mobility scaling ignores epidemiological lags.

The methods state that transmission at time t is scaled by the mobility metric at time t. This is biologically inconsistent: changes in population mobility require at least one generation interval, plus incubation and reporting delays, before they manifest in observed cases or deaths. Forcing instantaneous coupling either (a) requires the non-mechanistic process to absorb the temporal mismatch, artefactually inflating the without-mobility dispersion and producing a spurious dispersion ratio, or (b) biases the mobility exponent as the algorithm compensates for the missing lag. Either a fixed lag (informed by the generation interval) or convolution of the mobility series with the generation-time distribution before exponentiation is needed. A sensitivity analysis with 7-, 14-, and 21-day lags would establish whether the dispersion reduction holds under biologically plausible delays.

3. Confounding by concurrent NPIs and testing-policy changes.

The authors explicitly note that variation in testing rates, contact tracing, and clinical care improvements were not modelled. Yet government mandates simultaneously restricted movement and altered contact patterns through channels other than mobility (mask mandates, capacity limits, ventilation guidance). The reduction in the non-mechanistic dispersion attributed to mobility likely captures a composite of these effects. Either incorporate a policy-stringency index (e.g. Oxford OxCGRT) as an additional covariate in the scaling process, or reframe results as associative rather than effects of mobility per se. A comparison of dispersion reductions when adding mobility versus adding NPI indicators on a representative subset of countries would clarify the unique contribution of each.

4. Overinterpretation of the mobility exponent as a mechanistic parameter.

The Discussion interprets mobility-exponent values greater than one as reflecting a quadratic effect where both infectors and infectees experience reduced contact opportunities. The exponent is a statistical scaling parameter fitted to absorb residual variation; it is not a mechanistic contact-rate multiplier. Values > 1 could equally arise from unmodelled venue-specific transmission heterogeneity, seasonal forcing, or from low-variance mobility series (as the authors themselves note for Facebook within-tile metrics). Restrict the discussion to the exponent's role as an empirical transformation that optimises model fit. A profile-likelihood or prior-sensitivity check showing how tightly the data constrain m relative to the prior would strengthen the argument substantially.

5. Joint identifiability of Google location weights and dispersion is untested.

The identifiability analysis fixes all parameters except the non-mechanistic updates and the mobility-mapping exponent. This does not test the joint identifiability of the six Google location weights and the dispersion parameter. With the flexibility of the Wiener process, the model can plausibly trade off location weights against the non-mechanistic scaling to achieve similar likelihoods, particularly under the non-negativity constraint on weights. The location-specific conclusions (e.g. the consistent down-weighting of "parks") cannot be trusted without evidence of structural identifiability. Expand the synthetic-data experiment to jointly sample and refit the location weights, dispersion parameter, and mobility exponent, with posterior correlation matrices reported to surface trade-offs.

6. Unquantified sampling bias in mobility data.

The regional pattern (strong signal in high-income settings, weak in low-income) is attributed partly to data limitations but lacks quantitative support. Google and Facebook indices oversample smartphone users, urban residents, and higher-income demographics, directly correlated with the GDP stratification in Figure 4. Without measurement, the model cannot distinguish a genuinely weaker epidemiological relationship from a signal masked by unrepresentative data. Either include smartphone penetration rates or urbanisation indices as country-level covariates in a meta-analysis of dispersion ratios, or restrict the primary analysis to countries above a defined representativeness threshold and move the rest to sensitivity analysis.

7. Robustness checks need quantitative substantiation.

(a) Waning-immunity prior is too long-tailed. The sensitivity uses a prior centred at 180 days, which is at the longer end of the empirical evidence base. A shorter-waning prior (mean 90–120 days) would be a stronger check, since the current sensitivity essentially asks "if waning is slow, does it matter?".

(b) "Essentially unchanged" needs a number. Provide the maximum absolute difference in posterior means, the overlap of 95% credible intervals, or a Wasserstein/KL distance between the dispersion-parameter posteriors under base case and sensitivity. The current phrasing is unverifiable.

(c) Variant-severity recovery is hidden in the supplement. The response to reviewers states that severity-parameter posteriors are in Supplemental File 3 but does not report them. The recovered values for Alpha and Delta should be quoted in the main text and compared against the literature (Alpha

≈ 1.4–1.7× wild-type hazard for hospitalisation; Delta ≈ 1.3–1.6× over Alpha). If the priors dominated the posteriors, the variant-severity correction may not be doing the inferential work the authors claim.

8. Multiple comparisons across 122 countries.

The headline result that 47 of 122 countries are best fit by the no-mobility configuration risks overinterpretation without a noise-floor argument. Under a null model where mobility carries no information, what fraction of countries would be expected to favour the no-mobility configuration by chance, given posterior overlap and finite-sample variability? A short simulation or analytic argument addressing the family-wise picture would strengthen Figure 3.

Specific technical issues

S1. Main-text scaling equation does not match the supplement.

The main-text Methods give the Facebook scaling as f_t^m, where f_t is the relative-change mobility metric. The Facebook tiles-visited metric is reported as a relative change from baseline and frequently takes negative values during lockdowns; raising a negative base to a continuous real-valued exponent is undefined in the reals. The supplement clarifies the actual preprocessing, "one plus this mobility metric" for tiles visited, "one minus this metric" for the within-tile ratio, and "the raw value interpreted as a percentage plus one" for Google, so the implementation is valid, but the main-text equation is misleading and should explicitly show the $(1+f_t)^m$ form (and the analogous Google form) to match the supplement.

S2. Non-standard MCMC pairing for the dispersion ratio.

Constructing a ratio distribution by randomly pairing accepted iterations from two independent posteriors (one with mobility, one without) is unconventional and can produce unstable summaries when posteriors are skewed. The standard Bayesian quantity for this question is $P(\sigma_{no-mobility} > \sigma_{mobility})$, which can be computed directly from the marginal posteriors without the pairing step. Report this probability alongside or in place of the median ratio.

S3. Daily-vs-weekly temporal resolution mismatch.

The renewal process operates at daily resolution but the non-mechanistic scaling updates occur weekly. The piecewise-cosine interpolation is described, but the daily mapping should be made fully unambiguous (specifically, whether the daily scaling value is the cosine evaluation at that day or held piecewise constant between knots). Reproducibility requires this be explicit.

S4. Contradictory description of posteriors.

The text describes Facebook within-tile posteriors as "uninformative" while simultaneously stating that values "approaching two" are suggested for the United States, Türkiye, and several Western European countries. A genuinely uninformative posterior cannot suggest specific values. Either rephrase as "broad posteriors with modes near two" if they are informative, or remove the directional claim if they are flat.

S5. Mexico misclassified as high-income.

In the Results, Mexico is grouped with Belgium and the United Kingdom under "high-income settings". The World Bank classifies Mexico as upper-middle-income. Given that the income-stratification argument is central to the Discussion, this miscategorisation should be corrected ("high- and upper-middle-income settings" or a separate sub-grouping).

S6. Seropositivity definition error in supplement.

The authors note this was a documentation error rather than a code error and provide a code reference. The corrected definition should be reproduced verbatim in the supplement to leave no residual ambiguity.

Recommendation

Major revision. The methodological contribution is genuine and broadly applicable beyond COVID-19/mobility, and the revised manuscript meaningfully addresses the prior round’s most pressing concerns. However, the issues above, particularly the validation of the dispersion-reduction metric, the missing lag structure, and the conflation of mobility with concurrent policy effects, are substantive enough that they should be addressed before acceptance. None requires re-running the entire 122-country analysis from scratch; most can be addressed via targeted simulation studies, sensitivity analyses on a representative subset, and clarifications to the framing.
